# Supplementary material for: Sustainable purification-free synthesis of N–H ketimines by solid acid catalysis
Source: Nat Commun. 2025 Dec 10;16:10904. doi: 10.1038/s41467-025-66949-5 (PMC12695866; doi:10.1038/s41467-025-66949-5)
Supplement: Supplementary file 1 — Supplementary Information [file 41467_2025_66949_MOESM1_ESM.pdf]

Supplementary Information

Sustainable purification-free synthesis of *N*–H ketimines by solid acid catalysis

Shintaro Shibata[1]*, Makoto Onaka[2]*

[1]Research Foundation ITSUU Laboratory; Kanagawa, 213–0012, Japan

[2]Faculty of Life Sciences, Tokyo University of Agriculture; Tokyo 156–8502, Japan

Corresponding authors: sshibata@itsuu.or.jp, conaka@g.ecc.u-tokyo.ac.jp

Table of Contents

I **Instrumentation and materials**...3

II **Experimental procedures**

1. Synthesis of ketones, preparation of solid acid catalysts, and data of steroids...5

2. Preparation and characterization of *meso*-Al_2O_3/SO_4^{2-} and *meso*-Al_2O_3 catalysts

2.1 Experimental procedures...16

2.2 Nitrogen adsorption/desorption measurements and elemental analysis...17

2.3 IR-ATR, XRD, XPS and SEM-EDS measurements...20

3. Synthesis of *N*–H ketimine and *N*–H ketimine hydrochloride

3.1 Optimization of the reaction conditions (Fig. 2a, 2b)...29

3.2 Investigation on the effects of base additions (Fig. 2c)...29

3.3 Reactivity of HMDS for methanolysis...31

3.4 General procedures for the substrate scopes (Fig. 3a, 3b)...31

3.5 Synthesis of *N*–H ketimine on a large scale (Fig. 4a)...33

3.6 Air exposure test for catalyst usability (Fig. 4b)...33

3.7 Facile dehydrochlorination of *N*–H ketimine hydrochlorides via salt metathesis (Fig. 4c) 33

3.8 Hydrolysis instability of *N*–H ketimine during silica gel column chromatography...34

4. Synthesis of *N*–R ketimines (Fig. 4d)...36

5. Synthesis of α-aminonitriles

5.1 General procedures for the substrate scopes (Fig. 5a, 5c)...37

5.2 Hydrolysis instability of α-aminonitrile during silica gel column chromatography...38

6. Synthesis of hydantoin compounds

6.1 Optimization of the reaction conditions...39

6.2 General procedures for substrate scopes (Fig. 5b, 5c)...39

6.3 Comparison with the classical Bucherer–Bergs reactions...40

III **Quantum chemical calculation methods and results**

7. Thermodynamic evaluations on the condensation of a ketone with ammonia or an amine to form a ketimine...43

8. Exploration into reaction pathways (Fig. 2c)...45

IV **Compound data**

9. Reaction conditions and assignments

9.1 *N*–H Ketimine and *N*–H ketimine hydrochloride...49

9.2 *N*–R Ketimine...79

9.3 α-Aminonitrile...83

9.4 Hydantoin compound...89

10. Spectral data for NMR, FT-IR, and HRMS

10.1 *N*–H Ketimine and *N*–H ketimine hydrochloride...100

10.2 *N*–R Ketimine...243

10.3 α-Aminonitrile...259

10.4 Hydantoin compound...281

V **Energetic and coordinate data from quantum chemical calculations**...325

VI **References**...414

I Instrumentation and materials

Analysis devices

The solution NMR spectra were recorded using a JEOL ECZ 400S NMR spectrometer at 400 MHz for ^{1}H, 100 MHz for ^{13}C NMR, and 376 MHz for ^{19}F NMR. The chemical shift values for ^{1}H were referenced to $(CH_3)_4Si$ (δ = 0 in $CDCl_3$) or residual solvents (δ = 2.50 in DMSO-d_6 and δ = 1.94 ppm in CD_3CN), and those for ^{13}C were referenced to the residual solvent (δ = 77.0 ppm in $CDCl_3$, δ = 39.5 ppm, DMSO-d_6, and δ = 1.32 ppm in CD_3CN). The chemical shift values for ^{19}F were referenced to trifluoroacetic acid (δ = −78.5 ppm as an external standard). Chemical shifts are reported in δ ppm. Multiplicities are indicated as: br (broad), s (singlet), d (doublet), t (triplet), q (quartet), quin (quintet), sext (sextet), sep (septet) or m (multiplet). The coupling constants (*J*) are given in Hz. Isomers, if present, were identified using the 2D NMR study (NOESY and ROESY). IR spectroscopy was performed on a JASCO FT/IR-4600 with ATR (attenuated total reflection) system of "ATR PRO ONE" using a ZnSe plate. The ZnSe plate could be noisy at <550 cm^{-1}, hence the measurements were taken in the range of 4000–600 cm^{-1}). The high-resolution mass spectroscopy (HRMS) was performed on a Thermo Fisher Scientific Exactive Plus (ESI-quadrupole-Orbitrap MS). Nitrogen adsorption measurements were carried out on a BELSORP MAX X for *meso*-Al_2O_3/SO_4^{2-} and *meso*-Al_2O_3/SO_4^{2-} in sol, and on a BELSORP MAX G for *meso*-Al_2O_3. The sulfur content was measured by the combustion-infrared absorption method with a HORIBA EMIA-Expert carbon/sulfur analyzer. X-ray diffraction (XRD) measurements were performed on a SmartLab X-ray diffractometer. X-ray photoelectron spectroscopy (XPS) was analyzed with a Quantera SXM spectrometer. Surface morphology and particle sizes were examined using a JSM-IT800SHL scanning electron microscope (SEM), and elemental composition and distribution were analyzed with a Gather-X Dry SDD Windowless detector coupled to the SEM. All quantum chemistry calculations were performed using Gaussian 16, Revision C.01 software[1].

Solid acid catalysts

The powder forms of MS3A (Sigma-Aldrich®), MS5A (Sigma-Aldrich®), MS5A (Sigma-Aldrich®), Na-X (UNION SHOWA K.K., Na-2.0X, Si/Al = 1.0), Na-Y (TOSOH Co., Ltd., HSZ-320NAA, Si/Al = 2.75, specific surface area = 740 m^2 g^{-1}), H-Y (TOSOH Co., Ltd., HSZ-390HUA, Si/Al = 120, specific surface area = 630 m^2 g^{-1}; TOSOH Co., Ltd., HSZ-371HUA, Si/Al = 14.5,specific surface area = 550 m^2 g^{-1}; TOSOH Co., Ltd., HSZ-320HOA, Si/Al = 2.75, specific surface area = 570 m^2 g^{-1}), H-USY (UNION SHOWA K.K., Si/Al = 10), H-Beta (TOSOH Co., Ltd., HSZ-980HOA, Si/Al = 250, specific surface area = 500 m^2 g^{-1}; Süd-Chemie Catalysts Japan Inc., JRC-Z-HB150, Si/Al = 75, specific surface area = >500 m^2 g^{-1}; Süd-Chemie Catalysts Japan Inc., JRC-Z-HB25, Si/Al = 12.5, specific surface area = >400 m^2 g^{-1}), H-Mor (TOSOH Co., Ltd., HSZ-690HOA, Si/Al = 120, specific surface area = 450 m^2 g^{-1}; TOSOH Co., Ltd., HSZ-640HOA, Si/Al = 9, specific surface area = 380 m^2 g^{-1}), H-ZSM-5 (Süd-Chemie Catalysts Japan Inc., JRC-Z5-90H, Si/Al = 45, specific surface area = >300 m^2 g^{-1}), SiO_2-Al_2O_3 (JGC Catalysts and Chemicals Ltd., JRC-SAH-1, Si/Al = 1.8, specific surface area = 510 m^2 g^{-1}; JGC Catalysts and Chemicals Ltd., JRC-SAL-2, Si/Al = 5, specific surface area = 560 m^2 g^{-1}), acidic SiO_2 (FUJIFILM Wako Pure Chemical Corporation, Wakogel® C-200, pH = 5.5–7.0), neutral SiO_2 (KANTO CHEMICAL CO., INC., Silicagel 60N, pH = 7.0 ± 0.5), Q-15 (Fuji Silysia Chemical Ltd., CARiACT Q-15, pH = 7.6, specific surface area = 215 m^2 g^{-1}), Q-3 (Fuji Silysia Chemical Ltd., CARiACT Q-3, pH = 4.9, specific surface area = 680 m^2 g^{-1}), α-Al_2O_3 (FUJIFILM Wako

Pure Chemical Corporation, α-alumina, 0.5 μm for fine polishing, pH = ca.9), γ-Al_2O_3 (JGC Catalysts and Chemicals Ltd., JRC-ALO-2, SO_4^{2-}: 1.72%, pore volume: 0.72 cm^3 g^{-1}, specific surface area: 285 m^2 g^{-1}), γ-Al_2O_3 (JGC JAPAN CORPORATION, JRC-ALO-3, Na_2O: 0.3%, pore volume: 0.51 cm^3/g, specific surface area: 123 m^2 g^{-1}), γ-Al_2O_3 (JGC JAPAN CORPORATION, JRC-ALO-4, high purity, pore volume: 0.66 cm^3 g^{-1}, specific surface area: 117 m^2 g^{-1}), acidic γ-Al_2O_3 (Sigma-Aldrich®, Aluminum oxide activated, acidic, Brockmann I, pH = 4.5 ± 0.5), neutral γ-Al_2O_3 (Sigma-Aldrich®, Aluminum oxide activated, neutral, Brockmann I, pH = 7.0 ± 0.5), basic γ-Al_2O_3 (Sigma-Aldrich®, Aluminum oxide activated, basic, Brockmann I, pH = 9.5 ± 0.5), Mont K10 (Sigma-Aldrich®, Montmorillonite K10), Na-Mont (KUNIMINE INDUSTRIES Co., Ltd., Kunipia-F, Na: 2.69; Al: 11.8; Fe: 1.46; Mg: 1.97%, cation-exchange capacity = 1.19 meq g^{-1}, specific surface area = 26 m^2 g^{-1}), $Al_2(SO_4)_3$ [Thermo Fisher Scientific Inc., anhydrous, 99.99% (metals basis)], $Al(OH)_3$ (Tokyo Chemical Industry Co., Ltd.), NH_4Cl (FUJIFILM Wako Pure Chemical Corporation), and $(NH_4)_2SO_4$ (FUJIFILM Wako Pure Chemical Corporation) were purchased. All crystal structures of seven types of aluminas (α-Al_2O_3, γ-Al_2O_3 (SO_4^{2-}:1.72%), γ-Al_2O_3 (Na_2O:0.3%), γ-Al_2O_3 (high purity), acidic γ-Al_2O_3 (Aldrich), neutral γ-Al_2O_3 (Aldrich), basic γ-Al_2O_3 (Aldrich)) were confirmed on direct inquiry from the manufacturers. The bead forms of polymer-*p*-$C_6H_4SO_3H$ (Sigma-Aldrich®, Inc., *p*-toluenesulfonic acid, polymer-bound, macroporous, 30–60 mesh, extent of labeling: 2.0–3.0 mmol g^{-1} loading) and polymer-$C_6H_4NMe_2$ (Sigma-Aldrich®, Diaion® WA30 free base, water content: 50.3%, total exchange capacity: 1.6 meq mL^{-1}) were purchased. Ag-Y catalyst from the same batch reported in our previous study (Ref. 2) was used in this work. M-Mont was prepared from Na-Mont based on the literature (H-Mont[3], Al-Mont [from $Al(NO_3)_3$][4], Al-Mont [from $AlCl_3$][5], Ti-Mont[6], Fe-Mont[7], Cu-Mont[8], Sn-Mont[9]) using ultrapure water (Milli-Q system, Millipore). Mesoporous alumina (*meso*-Al_2O_3) was prepared according to Ref. 10. Mesoporous alumina containing sulfate ions (*meso*-Al_2O_3/SO_4^{2-}) was prepared following the procedure described in Ref. 10, with detailed experimental procedures shown in Section 2.1.

Reagents

Among the ketones used in the experiments, **1a**, **1b**, **1e**–**1l**, **1p**–**1s**, **1u**–**1ae**, **1ag**–**1bo,** and **1bp** were purchased, while **1c**, **1d**, **1m**–**1o**, **1t**, and **1af** were synthesized. As for steroids **1bq** and **1br**, synthesized by Dr. Suzuki in the course of her previous research[11,12], were donated by Research Foundation ITSUU Laboratory (spectral data for **1bq** and **1br** are shown in Section 1). Other reagents were purchased from FUJIFILM Wako Pure Chemical Corporation, KANTO CHEMICAL CO., INC., Tokyo Chemical Industry Co., Ltd., and Sigma-Aldrich®.

II Experimental procedures

1. Synthesis of ketones, preparation of solid acid catalysts, and data of steroids

Synthesis of ketones **1**

Ketones **1c**, **1d**, **1m**, **1n**, and **1af** were synthesized in our previous study (Ref. 13) and the same samples were used in this work. The spectral data for steroids **1bq** and **1br** are shown below.

General procedures for the synthesis of diaryl ketones (1c, 1d, 1af).

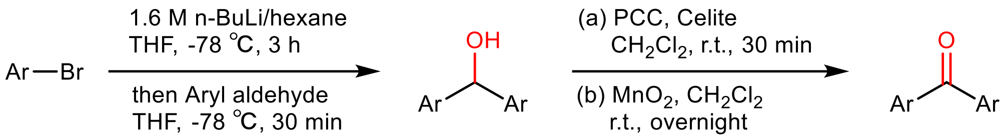

A 1.6 M solution of *n*-butyllithium (*n*-BuLi) in hexane (50 mmol, 31 mL) was slowly added to a solution of the aryl bromide (55 mmol) in dry tetrahydrofuran (THF, 50 mL) at −78 °C under a nitrogen atmosphere. The reaction mixture was stirred at −78 °C for 3 h, and a solution of the aryl aldehyde (50 mmol) in THF (15 mL) was then added dropwise. After stirring for 30 min at −78 °C, the reaction was quenched with 1 M HCl (aq) or water at room temperature. The aqueous layer was extracted with CH_2Cl_2. The combined organic extracts were dried over anhydrous Na_2SO_4, filtered, and concentrated under reduced pressure. The crude product was purified by column chromatography on silica gel to afford the corresponding diarylmethanol.

Pyridinium chlorochromate (PCC, 56 mmol) and Celite, or MnO_2 (600 mmol), were added to a solution of the diarylmethanol (40 mmol) in CH_2Cl_2 (130 mL). The mixture was stirred at room temperature for 30 min or overnight. The insoluble material was removed by filtration through a pad of Celite, and the filtrate was concentrated under reduced pressure. The crude product was purified by column chromatography on silica gel to afford the corresponding diaryl ketone.

(4-*t*-Butylphenyl)phenylmethanol

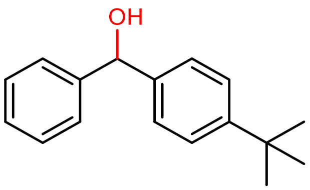

Purification by column chromatography on silica gel using ethyl acetate (EtOAc)/hexane (1:5) as the eluent afforded (4-*t*-butylphenyl)phenylmethanol as a white solid (93% yield).

^{1}H NMR (400 MHz, $CDCl_3$): δ = 7.43–7.27 (m, 9H), 5.83 (s, 1H), 1.99 (br, 1H), 1.30 (s, 9H).

^{13}C NMR (100 MHz, $CDCl_3$): δ = 150.50, 143.82, 140.85, 128.42, 127.44, 126.43, 126.26, 125.42, 76.05, 34.47, 31.30.

Bis(4-*t*-butylphenyl)methanol

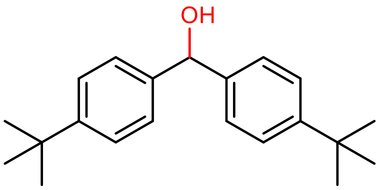

Purification by column chromatography on silica gel using EtOAc/hexane (1:5) as the eluent afforded bis(4-*tert*-butylphenyl)methanol as a white solid (91% yield).

^{1}H NMR (400 MHz, $CDCl_3$): δ = 8.29–8.39 (m, 8H), 5.81 (s, 1H), 1.30 (s, 18H).

^{13}C NMR (100 MHz, $CDCl_3$): δ = 150.33, 140.95, 126.20, 125.35, 75.87, 34.46, 31.31.

(2-Furyl)phenylmethanol

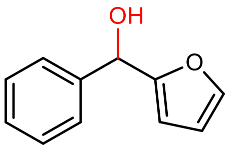

Purification by column chromatography on silica gel using EtOAc/hexane (1:5) as the eluent afforded (2-furyl)phenylmethanol as a colorless liquid (95% yield).

^{1}H NMR (400 MHz, $CDCl_3$): δ = 7.44 (d, 2H, J_{HH} = 7.7 Hz), 7.41–7.29 (m, 4H), 6.33–6.30 (m, 1H), 6.13–6.11 (m, 1H), 5.83 (br, 1H), 2.40 (br, 1H).

^{13}C NMR (100 MHz, $CDCl_3$): δ = 155.80, 142.23, 140.70, 128.18, 127.72, 126.44, 110.01, 107.18, 69.67.

(4-*t*-Butylphenyl)phenylmethanone (1c)

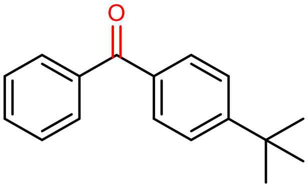

1c

Oxidation with PCC was performed. Purification by column chromatography on silica gel using EtOAc/hexane (1:10) as the eluent afforded (4-*t*-butylphenyl)phenylmethanone **1c** as a colorless liquid (>99% yield).

^{1}H NMR (400 MHz, $CDCl_3$): δ = 7.81 (d, 2H, J_{HH} = 7.4 Hz), 7.77 (d, 2H, J_{HH} = 8.5 Hz), 7.58 (t, 1H, J_{HH} = 7.4 Hz), 7.50 (d, 2H, J_{HH} = 8.5 Hz), 7.48 (t, 2H, J_{HH} = 7.4 Hz), 1.37 (s, 9H).

^{13}C NMR (100 MHz, $CDCl_3$): δ = 196.49, 156.17, 137.89, 134.77, 132.17, 130.13, 129.97, 128.19, 125.23, 35.09, 31.12.

Bis(4-*t*-butylphenyl)methanone (1d)

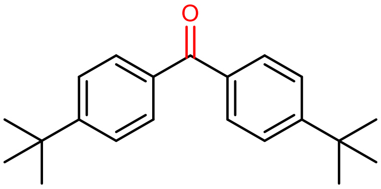

1d

Oxidation with PCC was performed. Purification by column chromatography on silica gel using EtOAc/hexane (1:10) as the eluent afforded bis(4-*t*-butylphenyl)methanone **1d** as a colorless liquid (95% yield).

^{1}H NMR (400 MHz, $CDCl_3$): δ = 7.76 (d, 4H, J_{HH} = 8.3 Hz), 7.49 (d, 4H, J_{HH} = 8.3 Hz), 1.37 (s, 18H).

^{13}C NMR (100 MHz, $CDCl_3$): δ = 196.18, 155.86, 135.09, 130.23, 125.14, 35.05, 31.13.

(2-Furyl)phenylmethanone (1af)

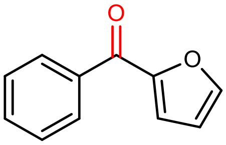

1af

Oxidation with MnO_2 was performed. Purification by column chromatography on silica gel using EtOAc/hexane (1:9) as the eluent afforded (2-furyl)phenylmethanone **1af** as a colorless liquid (88% yield).

^{1}H NMR (400 MHz, $CDCl_3$) δ = 7.97 (d, 2H, J_{HH} = 7.9 Hz), 7.72–7.70 (m, 1H), 7.60 (t, 1H, J_{HH} = 7.9 Hz), 7.50 (d, 2H, J_{HH} = 7.9 Hz), 7.26–7.23 (m, 1H), 6.61–6.58 (m, 1H).

^{13}C NMR (100 MHz, $CDCl_3$) δ = 182.39, 152.02, 147.03, 137.06, 132.44, 129.09, 128.26, 120.53, 112.10.

Synthesis of methyl 4-benzoylbenzoate (1m)

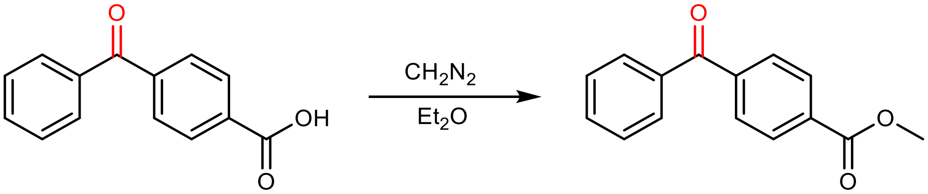

1l **1m**

After addition of potassium hydroxide (3.5 g, 62 mmol), ethanol (5 mL), and water (4 mL) to a Mini Diazald® apparatus (Aldrich), the reaction vessel was heated to 65 °C. *N*-methyl-*N*-nitroso-*p*-toluenesulfonamide (5.3 g, 25 mmol) was dissolved in dry diethyl ether (Et_2O, 25 mL) and added dropwise. The yellow distillate of diazomethane was passed into a solution of 4-benzoylbenzoic acid (2.86 g, 12.6 mmol) in diethyl ether (40 mL). After stirring for 10 min, 1 M acetic acid in

Et_2O was added until the solution became colorless. The organic layer was dried over anhydrous Na_2SO_4, filtered, and concentrated under reduced pressure. The crude product was purified by recrystallization from CH_2Cl_2/hexane to afford methyl 4-benzoylbenzoate **1m** as a white solid (2.07 g, 8.62 mmol, 78% yield).

^1H NMR (400 MHz, $CDCl_3$): δ = 8.15 (d, 2H, J_{HH} = 8.3 Hz), 7.84 (d, 2H, J_{HH} = 8.3 Hz), 7.81 (d, 2H, J_{HH} = 7.6 Hz), 7.61 (t, 1H, J_{HH} = 7.6 Hz), 7.50 (t, 2H, J_{HH} = 7.6 Hz), 3.96 (s, 3H).

^{13}C NMR (100 MHz, $CDCl_3$): δ = 195.98, 166.28, 141.30, 136.94, 133.19, 132.92, 130.07, 129.74, 129.48, 128.43, 52.43.

Synthesis of *t*-butyl 4-benzoylbenzoate (1n)

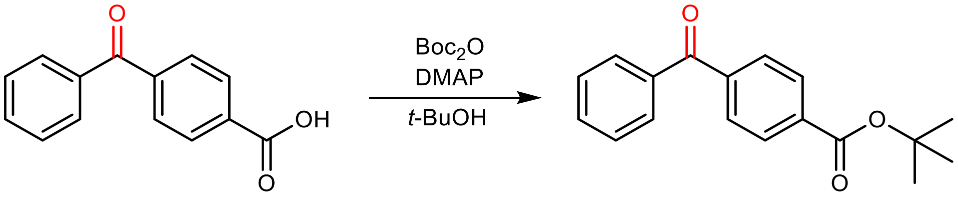

1l **1n**

Di-*t*-butyl dicarbonate (Boc_2O, 969 mg, 4.44 mmol) was slowly added to a mixture of 4-benzoylbenzoic acid (990 mg, 4.38 mmol) and 4-dimethylaminopyridine (DMAP, 543 mg, 4.45 mmol) in *t*-butanol (*t*-BuOH, 20 mL) at 0 °C under a nitrogen atmosphere. The reaction mixture was stirred at room temperature for 18 h. The resulting solids were removed by suction filtration, and the filtrate was concentrated under reduced pressure. The crude product was purified by column chromatography on silica gel using EtOAc/hexane (1:20) as the eluent to afford *t*-butyl 4-benzoylbenzoate **1n** as a white solid (869 mg, 3.22 mmol, 74% yield).

^1H NMR (400 MHz, $CDCl_3$) δ = 8.09 (d, 2H, J_{HH} = 8.1 Hz), 7.84–7.77 (m, 4H), 7.61 (t, 1H, J_{HH} = 7.8 Hz), 7.49 (t, 2H, J_{HH} = 7.8 Hz), 1.62 (s, 9H).

^{13}C NMR (100 MHz, $CDCl_3$) δ = 196.16, 164.90, 140.79, 137.02, 135.10, 132.84, 130.08, 129.64, 129.27, 128.39, 81.71, 28.11.

Synthesis of 4-benzoyl-*N*-butylbenzamide (1o)

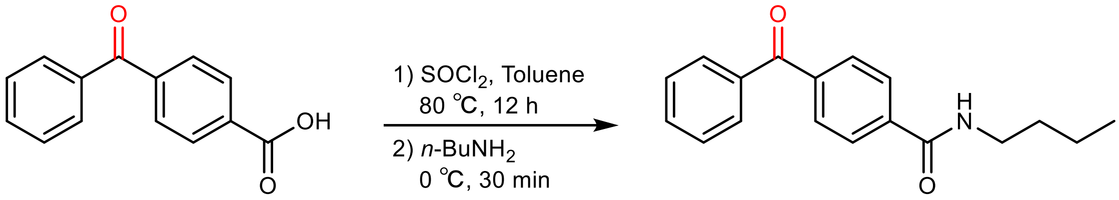

1l **1o**

A solution of 4-benzoylbenzoic acid **1l** (2.25 g, 9.95 mmol) and thionyl chloride (10.0 mL, 13.8 mmol) in dry toluene (10 mL) was stirred at 80 °C for 12 h, and then residual thionyl chloride was removed under reduced pressure. 4-Benzoylbenzoic acid chloride was obtained by distillation using a glass tube oven. The solution of 4-benzoylbenzoic acid chloride was slowly added to a *n*-butylamine (*n*-$BuNH_2$, 2.4 mL, 24 mmol) in CH_2Cl_2 (20 mL) at 0 °C. After stirred for 30 min, the

mixture was quenched with 1 M HCl (aq) at 0 °C until it became acidic. The aqueous layer was extracted with CH_2Cl_2. The combined organic layer was dried over anhydrous sodium sulfate, and concentrated. The crude product was purified by silica gel column chromatography using an eluting solvent of EtOAc/hexane (1/2) to afford 4-benzoyl-*N*-butylbenzamide **1o** as a white solid (2.51 g, 8.92 mmol, 90% yield).

^{1}H NMR (400 MHz, $CDCl_3$): δ = 7.90–7.57 (m, 5H), 7.61 (t, 1H, J_{HH} = 7.5 Hz), 7.49 (t, 2H, J_{HH} = 7.5 Hz), 6.37 (br, 1H), 3.49 (q, 2H, J_{HH} = 7.5 Hz), 1.63 (quin, 2H, J_{HH} = 7.5 Hz) 1.43 (sext, 2H, J_{HH} = 7.5 Hz), 0.97 (t, 3H, J_{HH} = 7.5 Hz) ppm.

^{13}C NMR (100 MHz, $CDCl_3$): δ = 195.98, 166.65, 139.82, 138.14, 137.01, 132.82, 130.04, 130.01, 128.39, 126.84, 39.95, 31.63, 20.13, 13.74 ppm.

Synthesis of (3-((*tert*-Butyldimethylsilyl)oxy)phenyl)(phenyl)methanone (1t)

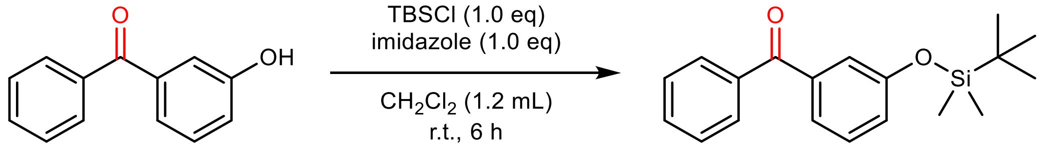

1s **1t**

t-Butylchlorodimethylsilane (TBSCl, 2.28 g, 15.1 mmol) was slowly added to a mixture of (3-hydroxyphenyl)(phenyl)methanone **1s** (2.97 g, 15.0 mmol) and imidazole (1.05 g, 15.4 mmol) in dry CH_2Cl_2 (50 mL) at 0 °C under a nitrogen atmosphere. After stirring at room temperature for 6 h, the solids in the suspension were removed by suction filtration and the solution was concentrated. The crude product was purified with silica gel column chromatography using an eluting solvent of EtOAc/hexane (1/20) to afford ketone **1t** as a colorless liquid (3.23 g, 10.4 mmol, 69% yield).

^{1}H NMR (400 MHz, $CDCl_3$): δ = 7.80 (d, 2H, J_{HH} = 7.5 Hz), 7.59 (t, 1H, J_{HH} = 7.5 Hz), 7.48 (t, 2H, J_{HH} = 7.5 Hz), 7.40–7.29 (m, 2H), 7.08–7.04 (m, 1H), 0.99 (s, 9H) 0.21 (s, 6H) ppm.

^{13}C NMR (100 MHz, $CDCl_3$): δ = 196.39, 155.62, 139.00, 137.59, 132.39, 130.03, 129.27, 128.21, 124.27, 123.24, 121.25, 25.62, 18.18, −4.44 ppm.

<u>Preparation of solid acid catalysts</u>

Ion-exchange procedure for Ag-Y

The same batch of the Ag-Y catalyst, which had been prepared from Na-Y by a conventional ion-exchange method and reported in our previous study (Ref. 2), was used in this work.

Powdered Na-Y zeolite (10.0 g, Si/Al = 2.75, HSZ-320NAA, Tosoh Corporation; specific surface area, 740 m^2 g^{-1}; pore volume, 0.34 cm^3 g^{-1}) was added to a solution of silver nitrate (15.3 g, 90.1 mmol) in distilled water (200 mL), and the mixture was stirred at 50 °C for 18 h under a dark nitrogen atmosphere. After suction filtration, the zeolite was washed with distilled water (100 mL). The washed solid was again added to a solution of silver nitrate (7.70 g, 45.3 mmol) in distilled water (100 mL), and the mixture was stirred at 50 °C for 48 h under a dark nitrogen atmosphere, followed by suction filtration and washing with distilled water (100 mL). The dispersion of the solid in distilled water was repeatedly filtered and washed until no silver chloride

formed upon addition of aqueous sodium chloride to the filtrate. The resulting zeolite was dried overnight at 80 °C in air and ground in a mortar. The sample was then gradually heated to 400 °C over 2 h in air and maintained at 400 °C for an additional 2 h in a muffle furnace, affording Ag-Y (10.2 g; specific surface area, 650 $m^2 g^{-1}$; pore volume, 0.33 $cm^3 g^{-1}$). The Ag-Y zeolite exhibited >99% ion exchange of Ag^+, as determined by inductively coupled plasma atomic emission spectroscopy (ICP–AES).

Ion-exchange procedure for H-Mont

H-Mont was prepared based on the reported procedure (Ref. 3). Na-Mont (3.0 g, Kunipia-F, Na: 2.69; Al: 11.8; Fe: 1.46; Mg: 1.97%, cation-exchange capacity = 1.19 meq g^{-1}) was suspended in an aqueous solution of HCl (0.36 mol L^{-1}, 200 mL, prepared using ultrapure water [Milli-Q system, Millipore]) and the mixture was heated with stirring at 90 °C for 24 h. After the reaction, the clay was separated by filtration and thoroughly washed with ultrapure water until the filtrate became nearly neutral (pH = 7) and no chloride ions were detected upon addition of aqueous $AgNO_3$. The obtained solid was dried in air at 110 °C for 12 h, then ground to a fine powder using an agate mortar and pestle to afford H-Mont.

Ion-exchange procedures for Al-Mont [from $Al(NO_3)_3$] and Sn-Mont

Al-Mont [from $Al(NO_3)_3$][4] and Sn-Mont[9] were prepared according to the reported procedures. Na-Mont (8.0 g, Kunipia-F; Na, 2.69%; Al, 11.8%; Fe, 1.46%; Mg, 1.97%; cation-exchange capacity = 1.19 meq g^{-1}) was treated with an aqueous solution of a metal salt (0.30 M, 80 mL) at room temperature for 2 h, where the metal salt was $Al(NO_3)_3{\cdot}9H_2O$ (Ref. 4) or $SnCl_4{\cdot}5H_2O$ (Ref. 9). After suction filtration, the ion-exchange procedure was repeated once. The resulting solid was separated by filtration and washed successively twice with ultrapure water (80 mL), six times with a 1:1 (v/v) mixture of ultrapure water (40 mL) and methanol (40 mL), and finally once with absolute methanol (80 mL). The obtained solid was dried under reduced pressure (0.5 Torr) at room temperature for 12 h, and then ground in air to a fine powder using an agate mortar and pestle to afford Al-Mont [from $Al(NO_3)_3$] or Sn-Mont.

Ion-exchange procedures for Al-Mont [from $AlCl_3$] and M-Mont (M = Ti, Fe, Cu)

Al-Mont [from $AlCl_3$][5], Ti-Mont[6], Fe-Mont[7], and Cu-Mont[8] were prepared according to the reported procedures. Na-Mont (4.2 g, Kunipia-F; Na, 2.69%; Al, 11.8%; Fe, 1.46%; Mg, 1.97%; cation-exchange capacity = 1.19 meq g^{-1}) was suspended in an aqueous solution of a metal salt (3.3×10^{-2} mol L^{-1}, 200 mL) and stirred at 50 °C for 24 h, where the metal salt was $AlCl_3{\cdot}6H_2O$ (Ref. 5), $TiCl_4$ (Ref. 6), $Fe(NO_3)_3{\cdot}9H_2O$ (Ref. 7), or $Cu(NO_3)_2{\cdot}3H_2O$ (Ref. 8). The resulting slurry was filtered, and the solid was washed with ultrapure water (200 mL). The washed solid was then dispersed in 200 mL of ultrapure water, followed by suction filtration and further washing with an additional 200 mL of ultrapure water. These dispersion and washing procedures were repeated once. The obtained sample was dried in air at 110 °C and subsequently ground to a fine powder using an agate mortar and pestle to afford Al-Mont [from $AlCl_3$] and M-Mont (M = Ti, Fe, Cu).

Data of steroid **1bq**

(8*R*,9*S*,13*S*,14*S*)-3-(Benzyloxy)-13-methyl-4-nitro-6,7,8,9,11,12,13,14,15,16-decahydro-17*H*-cyclopenta[*a*]phenanthren-17-one (1bq)

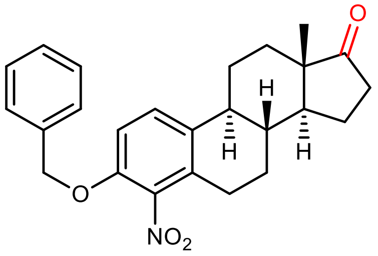

1bq

1bq was a white solid. See Section I (Instrumentation and materials) for the source of **1bq**.

^{1}H NMR (400 MHz, $CDCl_3$): δ = 7.40–7.25 (m, 6H), 6.86 (d, 1H, *J* = 8.8 Hz), 5.14 (s, 2H), 2.90–2.74 (m, 2H), 2.57–1.88 (m, 7H), 1.72-1.31 (m, 6H), 0.90 (s, 3H) ppm.

^{13}C NMR (100 MHz, $CDCl_3$): δ = 220.28, 147.45, 142.09, 135.80, 133.49, 128.89, 128.60, 128.10, 127.49, 126.97, 111.50, 70.82, 50.09, 47.74, 43.78, 37.41, 35.73, 31.37, 25.84, 25.36, 23.89, 21.44, 13.73 ppm.

ESI-quadrupole-Orbitrap MS (*m/z*): $[M+H]^+$ calcd for $C_{25}H_{28}NO_4^+$, 406.2013; found 406.2007.

<u>Data of steroid **1br**</u>

(8*R*,9*S*,13*S*,14*S*)-2-Bromo-3-hydroxy-13-methyl-4-nitro-6,7,8,9,11,12,13,14,15,16-decahydro-17*H*-cyclopenta[*a*]phenanthren-17-one (1br)

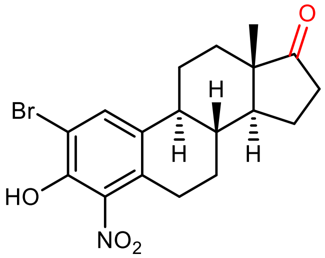

1br

1br was a white solid. See Section I (Instrumentation and materials) for the source of **1br**.

^{1}H NMR (400 MHz, $CDCl_3$): δ = 11.15 (br, 1H), 8.05 (s, 1H), 3.20–2.97 (m, 1H), 3.20–2.97 (m, 1H), 2.95–2.71 (m, 1H), 2.64–1.88 (m, 7H), 1.81–1.23 (m, 6H), 0.92 (s,3H) ppm.

^{13}C NMR (100 MHz, $CDCl_3$): δ = 219.89, 150.03, 148.01, 133.87, 131.85, 120.25, 115.58, 50.18, 47.63, 43.69, 37.03, 35.73, 31.92, 31.17, 26.15, 25.88, 21.45, 13.66 ppm.

ESI-quadrupole-Orbitrap MS (*m/z*): $[M-H]^-$ calcd for $C_{18}H_{19}BrNO_4^-$, 392.0503; found 392.0487.

<u>^{1}H NMR (400 MHz, DMSO-*d*$_6$) and ^{13}C NMR (100 MHz, DMSO-*d*$_6$) spectra for steroid **1bq**</u>

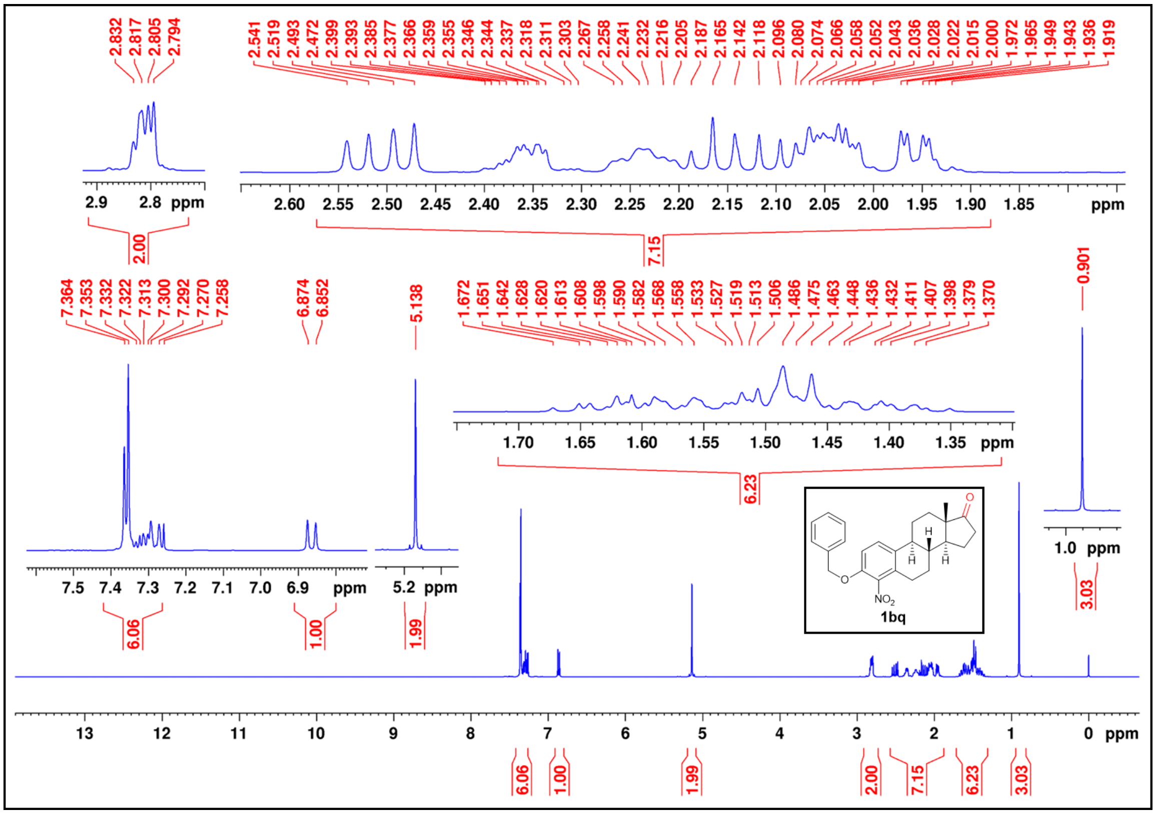

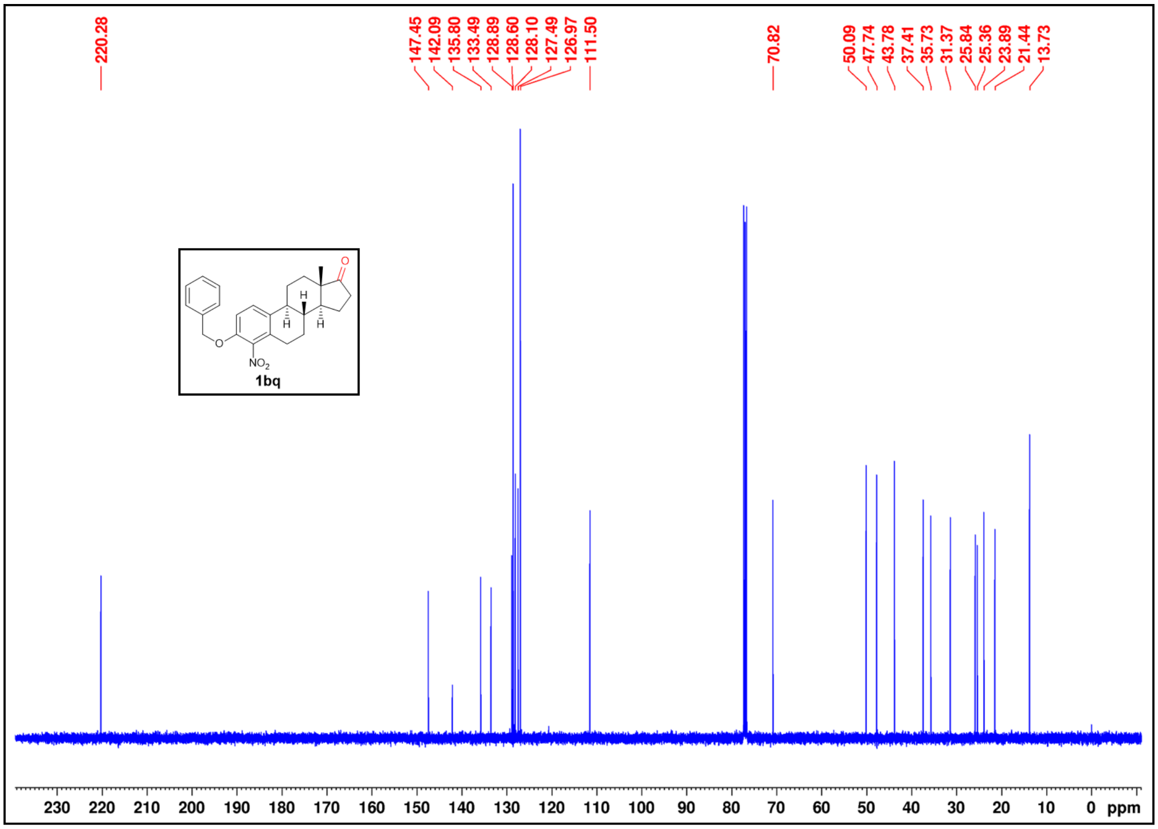

<u>FT-IR (ATR, neat) and HRMS (ESI-positive) spectra for **1bq**</u>

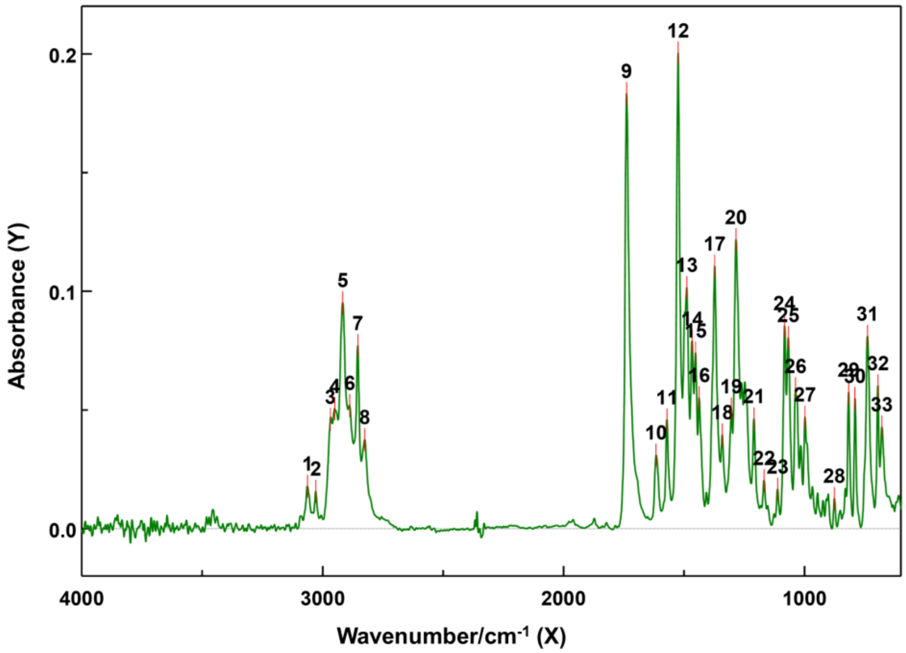

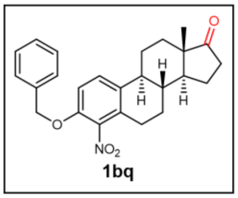

No.	X	Y	No.	X	Y	No.	X	Y
1	3063.4	0.018	12	1524.5	0.2	23	1112	0.017
2	3028.7	0.016	13	1488.8	0.101	24	1083	0.085
3	2967.9	0.046	14	1466.6	0.079	25	1067	0.08
4	2949.6	0.05	15	1452.1	0.074	26	1038	0.059
5	2916.8	0.095	16	1437.7	0.055	27	998	0.047
6	2887.9	0.052	17	1373.1	0.11	28	876.5	0.013
7	2854.1	0.077	18	1341.2	0.039	29	816.7	0.057
8	2825.2	0.037	19	1303.6	0.05	30	790.7	0.055
9	1738.5	0.183	20	1284.4	0.122	31	738.6	0.081
10	1616.1	0.031	21	1210.1	0.046	32	695.2	0.06
11	1570.7	0.046	22	1167.7	0.02	33	678.8	0.043

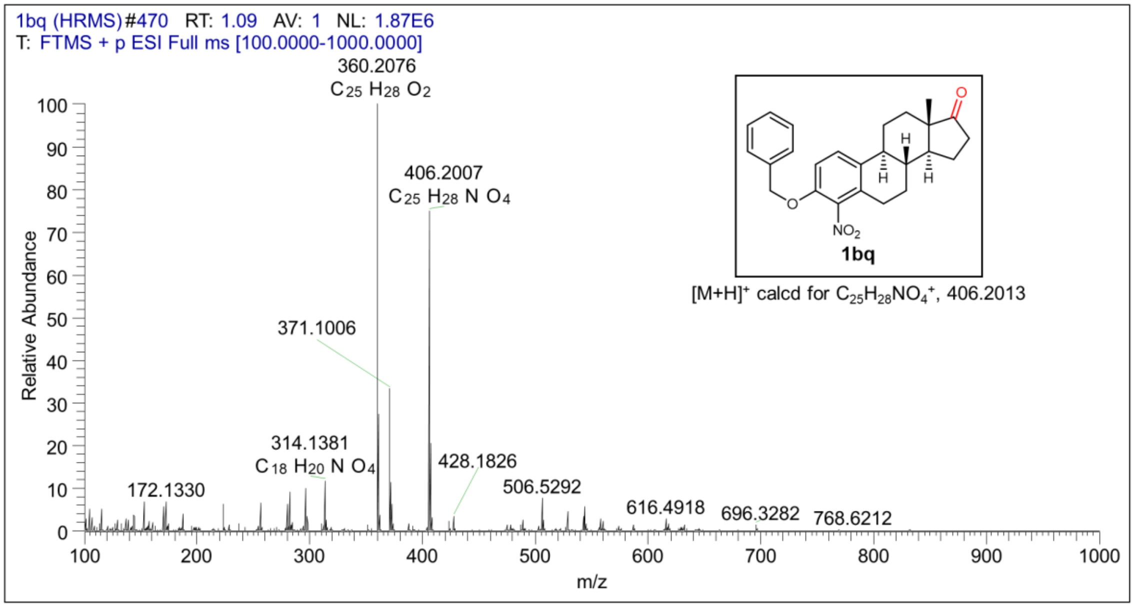

<u>^{1}H NMR (400 MHz, DMSO-d_6) and ^{13}C NMR (100 MHz, DMSO-d_6) spectra for steroid **1br**</u>

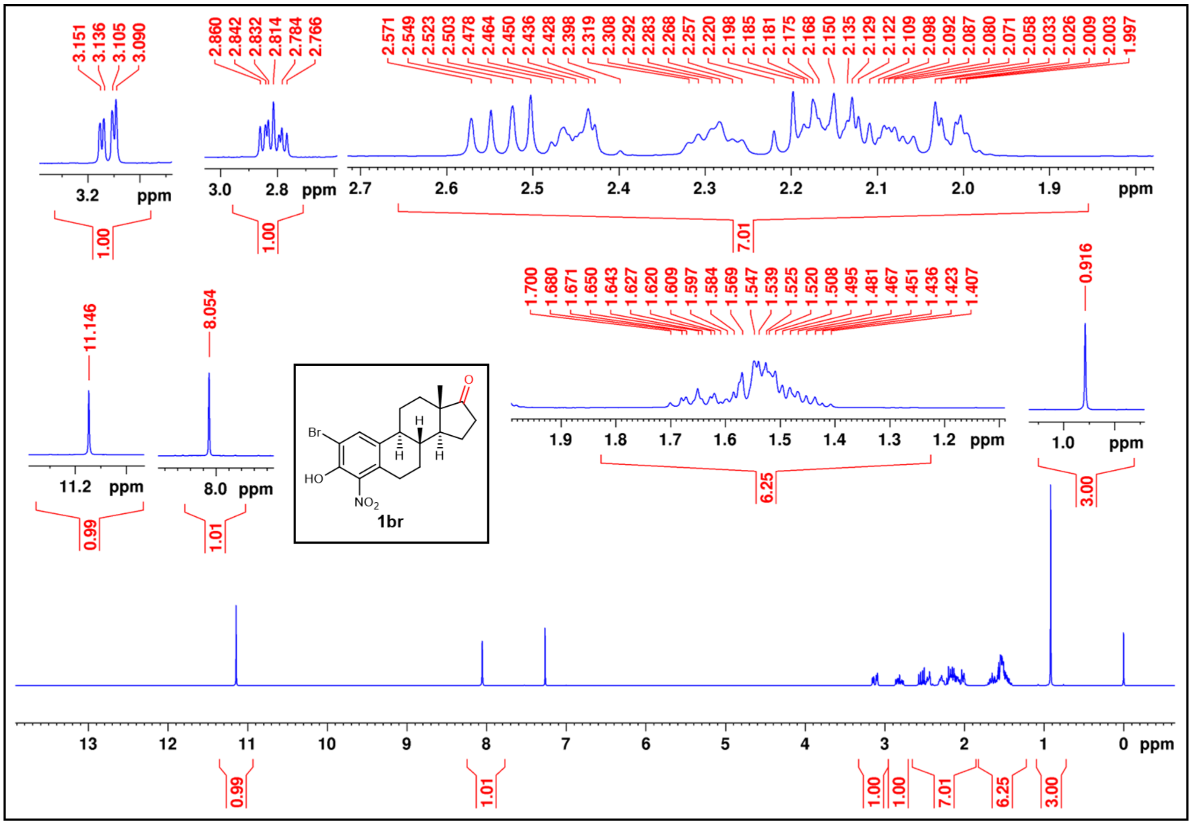

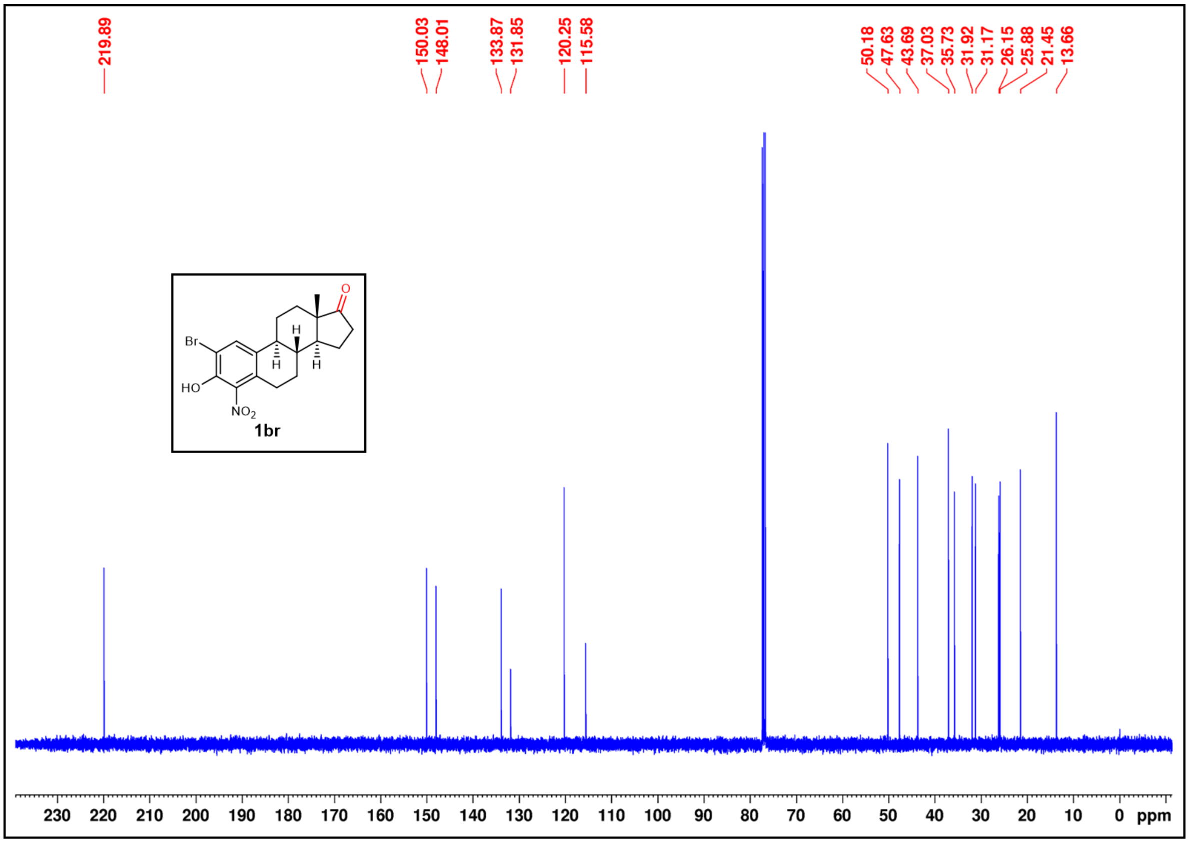

FT-IR (ATR, neat) and HRMS (ESI-negative) spectra for **1br**

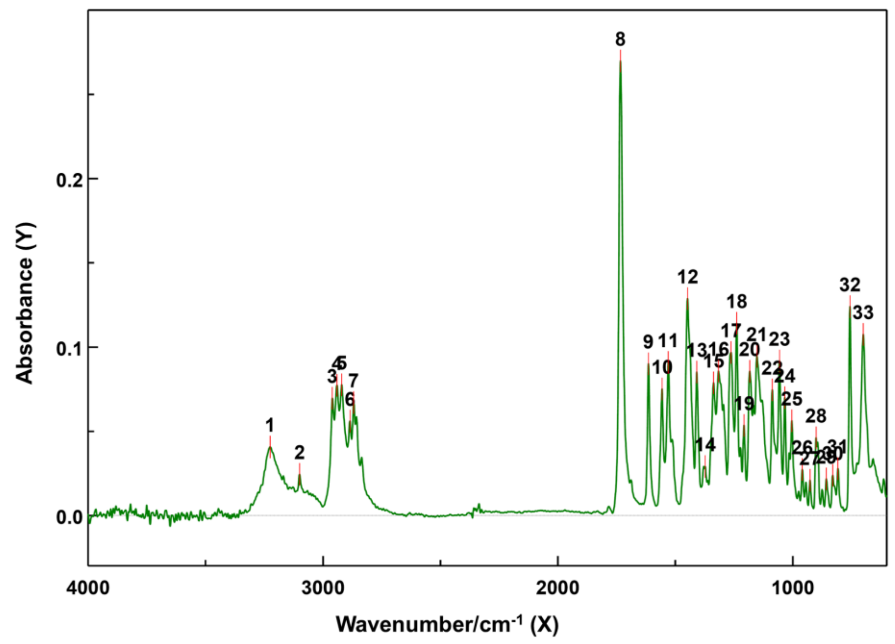

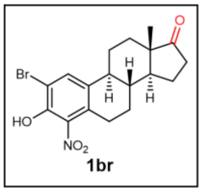

No.	X	Y	No.	X	Y	No.	X	Y
1	3225.4	0.041	12	1448.3	0.129	23	1057	0.092
2	3100	0.024	13	1408.7	0.085	24	1034	0.07
3	2960.2	0.069	14	1373.1	0.029	25	1004	0.056
4	2940.9	0.077	15	1337.4	0.079	26	959.4	0.027
5	2920.7	0.077	16	1316.2	0.086	27	926.6	0.021
6	2885	0.056	17	1263.1	0.097	28	900.6	0.046
7	2869.6	0.067	18	1239	0.114	29	857.2	0.022
8	1733.7	0.27	19	1207.2	0.053	30	830.2	0.024
9	1614.1	0.09	20	1183.1	0.085	31	808	0.028
10	1556.3	0.075	21	1152.3	0.094	32	756.9	0.124
11	1530.2	0.091	22	1087.7	0.074	33	700	0.107

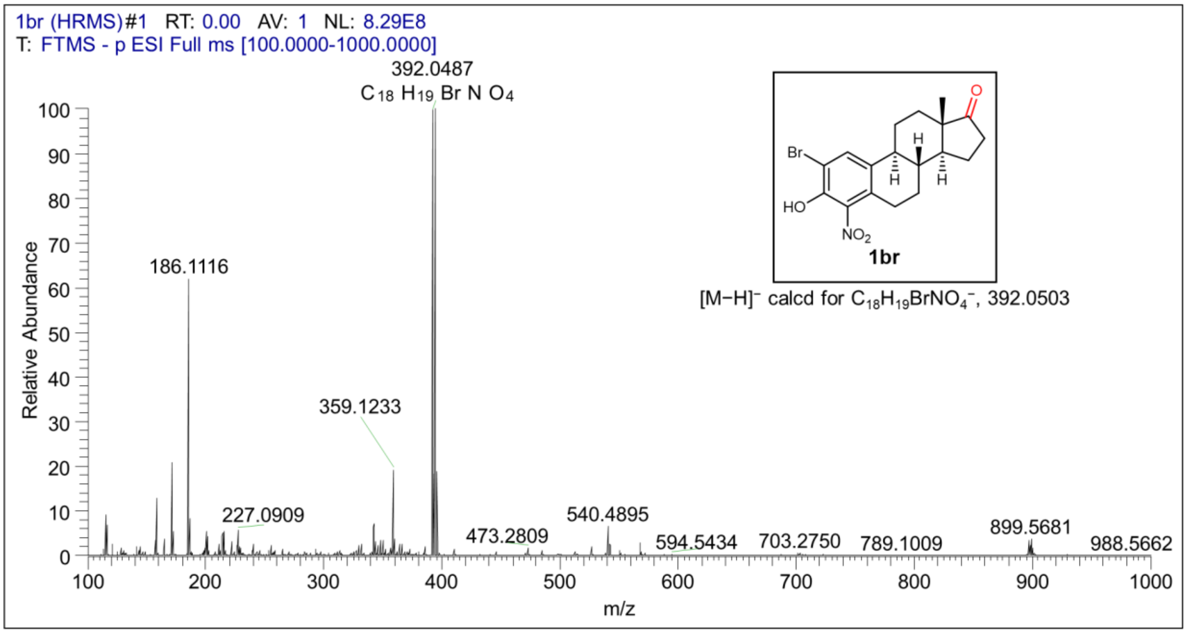

2. Preparation and characterization of *meso*-Al_2O_3/SO_4^{2-} and *meso*-Al_2O_3

2.1 Experimental procedures

Mesoporous alumina containing sulfate ions (*meso*-Al_2O_3/SO_4^{2-}**)** was synthesized according to Ref. 10. A mixture of Al(O-*sec*-Bu)$_3$ (19.6 g, 79.6 mmol), $Al_2(SO_4)_3$ (1.7 g, 5.0 mmol), 1-propanol (120 g, 2.00 mol), and deionized water (5.19 g, 288 mmol) was placed in a 200 mL flask and stirred vigorously for 1 h at room temperature (RT). Then lauric acid (5.40 g, 27.0 mmol) and 1-propanol (17.9 g, 298 mmol) were added and thoroughly stirred for 24 h at RT (Fig.S1A). The resulting white suspension was transferred to a 500 mL autoclave polypropylene-made bottle (heat resistance temperature: 130 °C), the bottom of which was immersed in an oil bath and heated at 110 °C for 48 h without stirring (Fig. S1B). The gel and supernatant were then separated in the bottle. From the gel part the ***meso*-Al_2O_3/SO_4^{2-}** and from the supernatant solution the ***meso*-Al_2O_3/SO_4^{2-} in sol** were both prepared as follows.

***Meso*-Al_2O_3/SO_4^{2-}**: After gently washing the surface of the gel with a small amount of ethanol, the gel was dried overnight in a dryer at 110 °C (Figure S1C). The dried solid was crushed in a mortar and was set in a glass-made calcination tube. The heat-resistant tube was gradually heated up to 600 °C in an electric heater for 2 h under a flow of N_2 (Fig. S1D). The resulting black powder was then transferred to a ceramic calcination dish, gradually heated to 600 °C over 2 h in the air in a muffle furnace, and kept at 600 °C for an additional 5 h (Fig. S1E). The resulting white powder (Fig. S1F) was ground in a mortar and sieved to a size of less than 200 μm. The ***meso*-Al_2O_3/SO_4^{2-}** was obtained as a white powder (a yield of 2.05 g, SO_4^{2-} content: 4.0 wt% determined by the combustion-infrared absorption method, pore volume: 0.46 cm^3/g based on the BJH method, and specific surface area: 362 m^2/g based on the BET method).

***Meso*-Al_2O_3/SO_4^{2-} in sol**: After concentrating the separated supernatant, the solid was treated in the same manner as the ***meso*-Al_2O_3/SO_4^{2-}**. The ***meso*-Al_2O_3/SO_4^{2-} in sol** was obtained as a white powder (a yield of 3.24 g, SO_4^{2-} content: 0.03 wt% determined by the combustion-infrared absorption method, pore volume: 0.43 cm^3/g based on the BJH method, specific surface area: 329 m^2/g based on the BET method).

***Meso*-Al_2O_3** was prepared in the same manner as ***meso*-Al_2O_3/SO_4^{2-}**, except that $Al_2(SO_4)_3$ was not added.

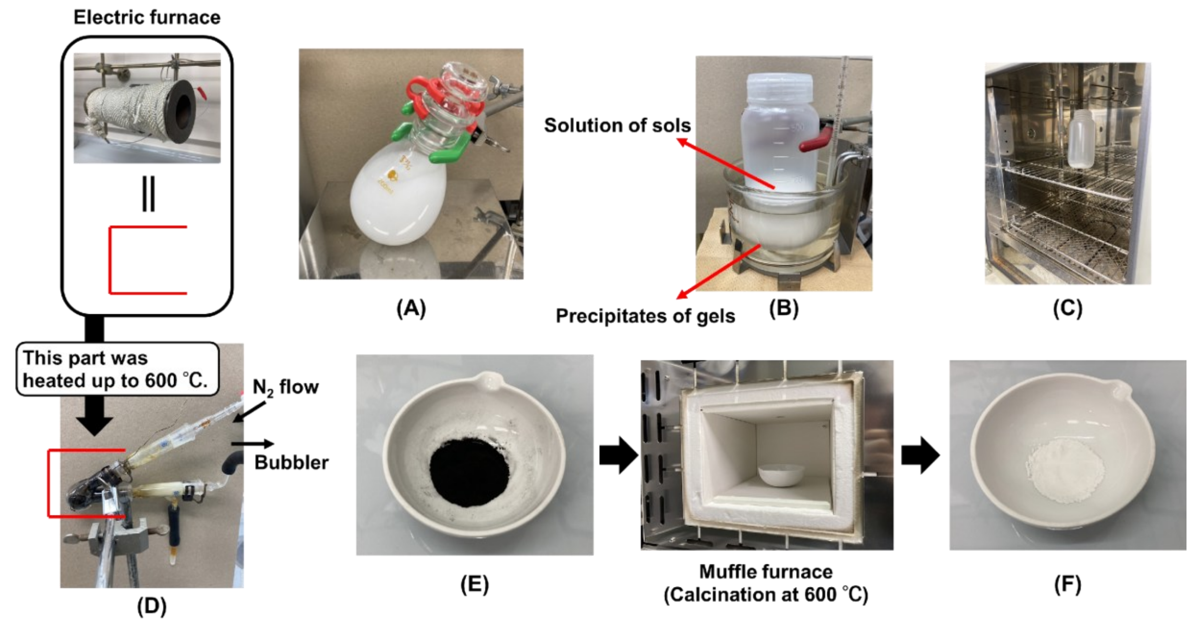

Fig. S1 Supplemental figures for the *meso*-Al_2O_3/SO_4^{2-} preparations. (**A**) Formation of the sol by agitation. (**B**) Formation of the gel by standing at 110 °C. (**C**) Drying in an electric furnace. (**D**) The heating tube after being heated up to 600 °C under a flow of N_2. (**E**) The black solids before calcinated in the air at 600 °C for 5 h. (**F**) The white solids, ***meso*-Al_2O_3/SO_4^{2-}**, after calcinated in the air at 600 °C for 5 h.

2.2 Nitrogen adsorption/desorption measurements and elemental analysis

For the analysis of ***meso*-Al_2O_3/SO_4^{2-}**, ***meso*-Al_2O_3/SO_4^{2-} in sol**, and ***meso*-Al_2O_3**, Fig. S2 and Fig. S3 show the adsorption/desorption isotherms and pore-size distributions, while Table S1 summarizes the results of nitrogen adsorption measurements and the elemental analysis of sulfur content. The values such as the pore volume and specific surface area were obtained by the BET, BJH, and *t*-plot methods. The pore-size distribution obtained by the BJH method is shown in Fig. S3.

Table S1. Analysis of the prepared mesoporous alumina catalysts

Method	Properties	***meso*-Al_2O_3/SO_4^{2-}**	***meso*-Al_2O_3/SO_4^{2-} in sol**	***meso*-Al_2O_3**
BET	Specific surface area	362 m^2/g	329 m^2/g	317 m^2/g
Adsorption side, BJH	Pore volume	0.456 cm^3/g	0.433 cm^3/g	0.451 cm^3/g
	Average pore diameter	4.3 nm	4.0 nm	5.5 nm
	Pore surface area	452 m^2/g	400 m^2/g	375 m^2/g
Desorption side, BJH	Pore volume	0.463 cm^3/g	0.441 cm^3/g	0.458 cm^3/g
	Average pore diameter	4.1 nm	3.9 nm	4.8 nm
	Pore surface area	479 m^2/g	433 m^2/g	399 m^2/g
t-plot	Total surface area	325 m^2/g	322 m^2/g	310 m^2/g
	Pore surface area	313 m^2/g	320 m^2/g	312 m^2/g
	Pore volume	0.395 cm^3/g	0.410 cm^3/g	0.440 cm^3/g
Combustion-infrared absorption	Sulfur content	4.0 wt%	0.03 wt%	–

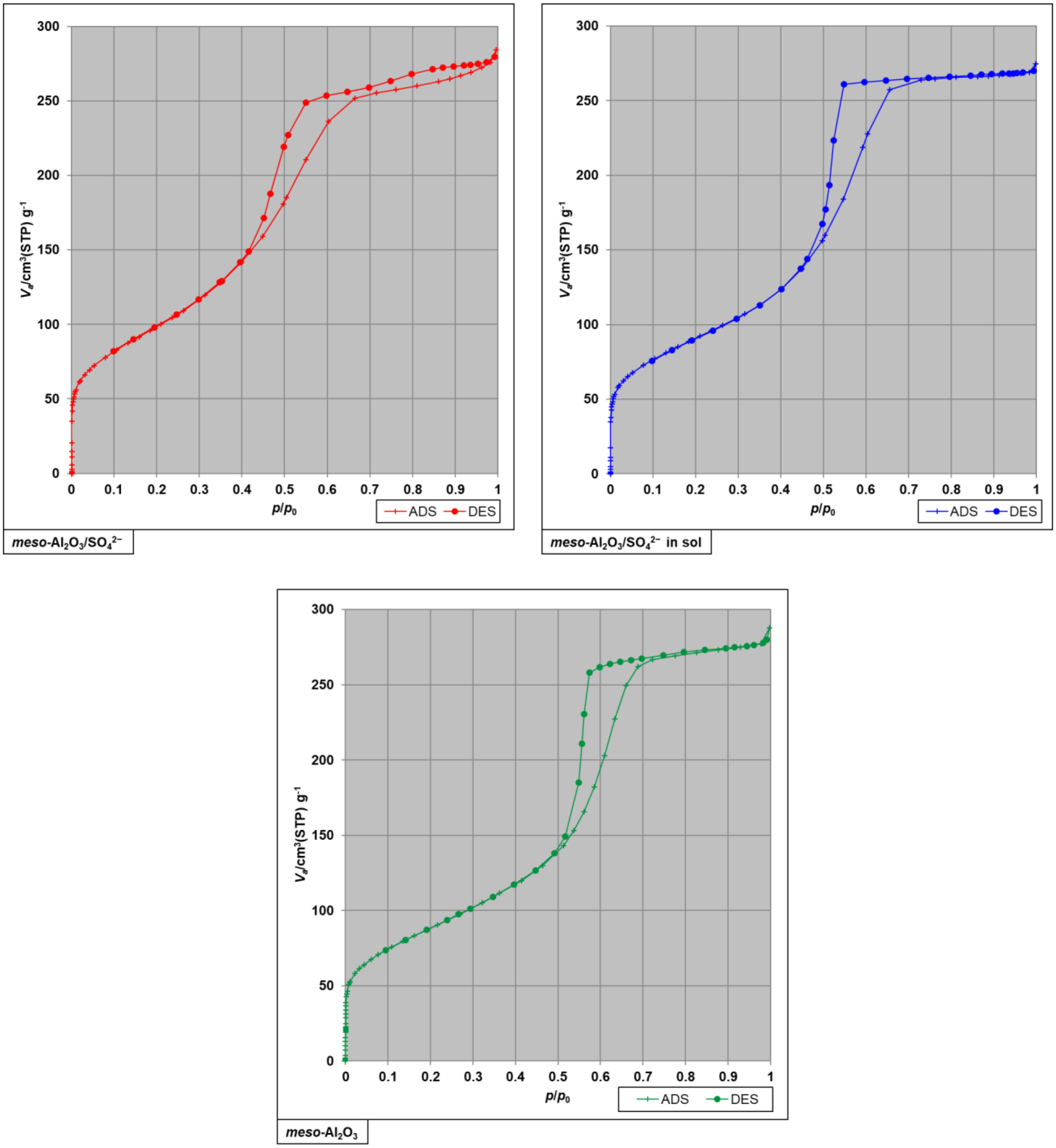

Fig. S2 Adsorption/desorption isotherms of ***meso*-Al_2O_3/SO_4^{2-}** (red lines), ***meso*-Al_2O_3/SO_4^{2-} in sol** (blue lines), and ***meso*-Al_2O_3** (green lines).

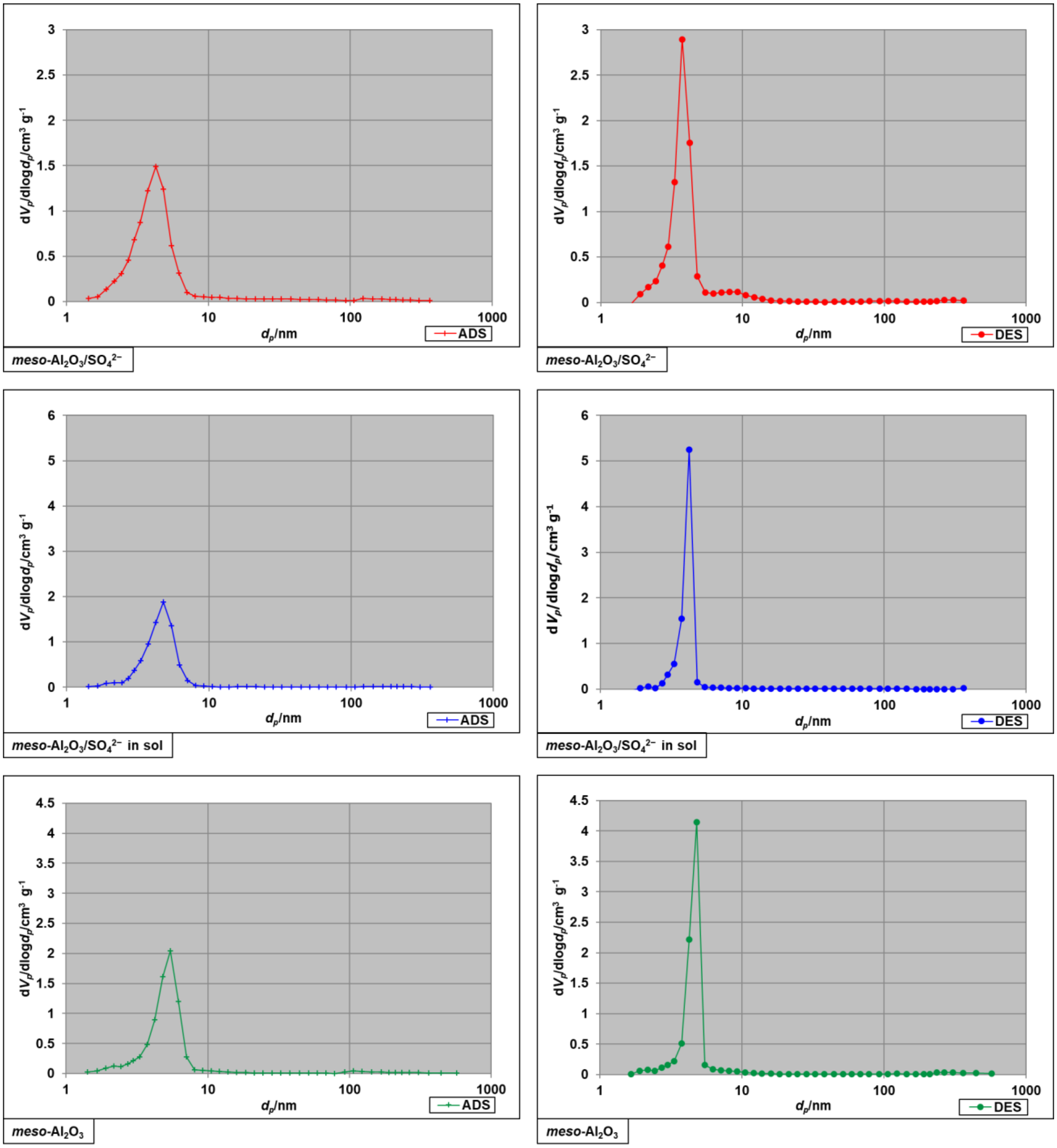

Fig. S3 BJH pore size distributions of ***meso*-Al_2O_3/SO_4^{2-}** (red lines), ***meso*-Al_2O_3/SO_4^{2-} in sol** (blue lines), and ***meso*-Al_2O_3** (green lines).

2.3 IR-ATR, XRD, XPS and SEM-EDS measurements

As described in Section 2.2, elemental analysis detected sulfur in the ***meso*-Al_2O_3/SO_4^{2-}** catalyst. To investigate the chemical form of sulfur in ***meso*-Al_2O_3/SO_4^{2-}**, solid samples were characterized by infrared spectroscopy with attenuated total reflectance (IR-ATR), X-ray diffraction (XRD), X-ray photoelectron spectroscopy (XPS), and scanning electron microscopy with energy-dispersive X-ray spectroscopy (SEM-EDS). For comparison, anhydrous **$Al_2(SO_4)_3$** was also measured by IR-ATR and XRD. According to the thermogravimetric analysis reported in Ref. 14, the **$Al_2(SO_4)_3$** used in the preparation of catalyst ***meso*-Al_2O_3/SO_4^{2-}** remains stable as a solid when calcined at 600 °C. To match the calcination conditions used in the catalyst preparation, the anhydrous **$Al_2(SO_4)_3$** was also calcined at 600 °C. All solid materials were dried at 400 °C under <0.5 torr for 2 h prior to each measurement. In the case of ***meso*-Al_2O_3 + $Al_2(SO_4)_3$** used for IR-ATR, **$Al_2(SO_4)_3$** was added to ***meso*-Al_2O_3** to match the sulfur content of ***meso*-Al_2O_3/SO_4^{2-}**, and the mixture was then ground using a mortar and pestle and dehydrated (400 °C, 2 h, <0.5 Torr).

IR-ATR analysis

The IR-ATR spectra of ***meso*-Al_2O_3** (brown line) and ***meso*-Al_2O_3** physically mixed with **$Al_2(SO_4)_3$** (***meso*-Al_2O_3 + $Al_2(SO_4)_3$**, orange line), shown in Figure S4(a), both exhibited a broad absorption band below 950 cm^{-1}, attributable to the Al–O stretching vibrations. In contrast, only ***meso*-Al_2O_3/SO_4^{2-}** (blue line) displayed a weak and broad absorption around 1200 cm^{-1}. The spectrum of **$Al_2(SO_4)_3$** (green line) shows characteristic absorptions near 1200 cm^{-1} and 650 cm^{-1}, corresponding to the stretching and bending vibrations of the sulfate ion, respectively. Therefore, the weak and broad absorption observed around 1200 cm^{-1} in ***meso*-Al_2O_3/SO_4^{2-}** can be attributed to sulfate bonding. As shown in the expanded spectra of the 1500–800 cm^{-1} region in Figure S4(b), the peak top of ***meso*-Al_2O_3/SO_4^{2-}** appears at 1179.3 cm^{-1}, slightly shifted to higher wavenumber compared with the corresponding peak of **$Al_2(SO_4)_3$** (1169.6 cm^{-1}). Furthermore, other characteristic stretching vibration peaks of **$Al_2(SO_4)_3$** were not observed in ***meso*-Al_2O_3/SO_4^{2-}**. These results indicate that, based on the IR-ATR measurements, ***meso*-Al_2O_3/SO_4^{2-}** cannot be regarded as a simple physical mixture of ***meso*-Al_2O_3** and **$Al_2(SO_4)_3$**.

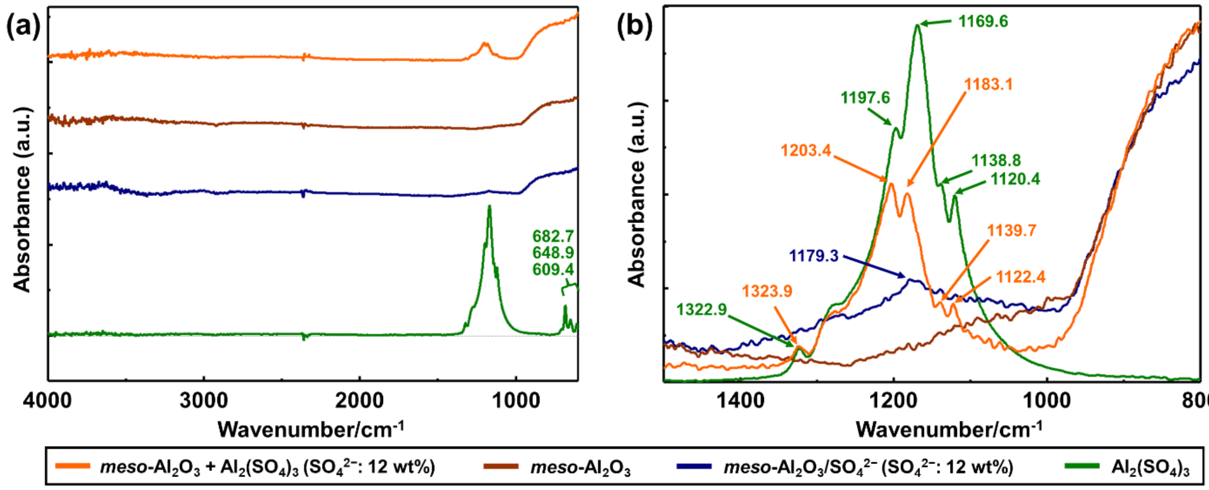

Fig. S4 IR-ATR spectra of ***meso*-Al_2O_3 + $Al_2(SO_4)_3$** (orange line), ***meso*-Al_2O_3** (brown line), ***meso*-Al_2O_3/SO_4^{2-}** (blue line), and **$Al_2(SO_4)_3$** (green line): (a) full range spectrum; (b) 1500–800 cm^{-1} region with the expanded vertical axes only for ***meso*-Al_2O_3** and ***meso*-Al_2O_3/SO_4^{2-}**.

XRD analysis

XRD measurements were carried out on a SmartLab diffractometer using Cu Kα radiation at 40 kV and 30 mA. Data were collected in $2\theta/\theta$ mode over a 2θ range of 8–90° with a step size of 0.02° and a scan speed of 5°/min. The incident slit was 1/3°, and both receiving slits were set to 1.0 mm.

Fig. S5 shows the powder XRD patterns of $\mathbf{Al_2(SO_4)_3}$, ***meso*-$\mathbf{Al_2O_3/SO_4^{2-}}$**, and ***meso*-$\mathbf{Al_2O_3}$** samples. The spectrum of ***meso*-$\mathbf{Al_2O_3}$** (brown line) exhibits broad diffraction peaks characteristic of amorphous materials. In contrast, $\mathbf{Al_2(SO_4)_3}$ (green line) displays sharp diffraction peaks arising from its crystalline structure. The ***meso*-$\mathbf{Al_2O_3/SO_4^{2-}}$** sample (blue) shows a smooth baseline similar to that of ***meso*-$\mathbf{Al_2O_3}$** and ***meso*-$\mathbf{Al_2O_3/SO_4^{2-}}$** prepared in Ref. 10, but a small peak at 25.44° is observed, indicating the slight presence of residual $\mathbf{Al_2(SO_4)_3}$. In Ref. 10, ***meso*-$\mathbf{Al_2O_3/SO_4^{2-}}$** contained 5.15 wt% sulfate ions (1.72 wt% sulfur), and no peak at 25.44° was observed, indicating that all sulfate ions were fully incorporated into the alumina framework. In the present study, ***meso*-$\mathbf{Al_2O_3/SO_4^{2-}}$** contains 12.0 wt% sulfate ions (4.0 wt% sulfur), which is 2.3 times higher than that in Ref. 10. These results suggest that because more sulfur was introduced than in previous studies, some of it was not incorporated into the alumina framework and instead exists as $\mathbf{Al_2(SO_4)_3}$.

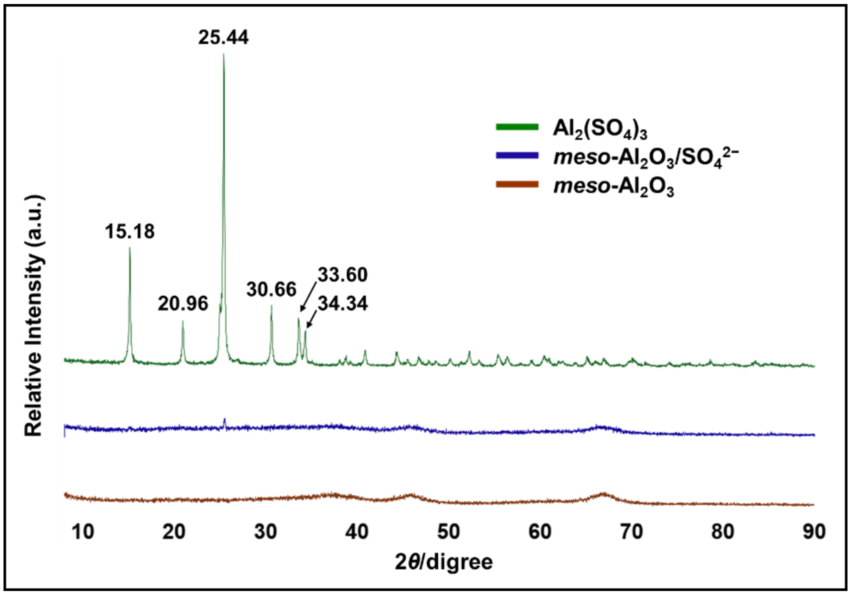

Fig. S5 XRD spectra of $\mathbf{Al_2(SO_4)_3}$ (green line), ***meso*-$\mathbf{Al_2O_3/SO_4^{2-}}$** (blue line), and ***meso*-$\mathbf{Al_2O_3}$** (brown line).

XPS analysis

X-ray photoelectron spectroscopy (XPS) measurements were conducted on powder samples mounted on conductive tapes and introduced into a Quantera SXM spectrometer. A monochromatic Al Kα X-ray source (25.0 W, 100 μm beam diameter) was employed. Wide-scan spectra were acquired with a pass energy of 280.00 eV and a step size of 1.00 eV, while narrow-scan spectra were obtained with a pass energy of 69.00 eV and a step size of 0.125 eV. Charge neutralization was achieved using both Ar^+ ions and electrons.

For the ***meso*-Al_2O_3/SO_4^{2-}** (blue line) and ***meso*-Al_2O_3** (red line), Fig. S6 shows the full XPS spectra. Fig. S7 presents the C 1s peak used as the reference (calibrated to 248.8 eV), together with the measured and normalized spectra. Fig. S8 shows the enlarged peak of S 2p, Fig. S9 displays the enlarged peaks of Al 2s, Al 2p, and O 1s, and Fig. S10 presents the normalized enlarged spectra of Al 2s, Al 2p, and O 1s. The reference peak positions of $Al_2(SO_4)_3$ were taken from the XPS database[15]. Since the database values were calibrated with the C 1s peak at 285.0 eV, the values shown in the spectra were corrected by subtracting 0.2 eV, so that 284.8 eV was used as the reference.

In the S spectrum shown in Fig. 8, only ***meso*-Al_2O_3/SO_4^{2-}** exhibited a trace sulfur peak. As shown in Figs. 9 and 10, the Al 2s, Al 2p, and O 1s peaks of ***meso*-Al_2O_3/SO_4^{2-}** did not display any additional features such as shoulders. A comparison of the Al 2s, Al 2p, and O 1s peaks between ***meso*-Al_2O_3/SO_4^{2-}** and ***meso*-Al_2O_3** revealed that those of ***meso*-Al_2O_3/SO_4^{2-}** were slightly shifted toward the higher binding energy side, closer to the reference peaks of **$Al_2(SO_4)_3$**. However, the shift was only about 0.5 eV, and the possibility that it arose from charging effects of the insulating alumina cannot be ruled out. Therefore, these results suggest that no significant spectral differences due to the incorporation of sulfate species were observed.

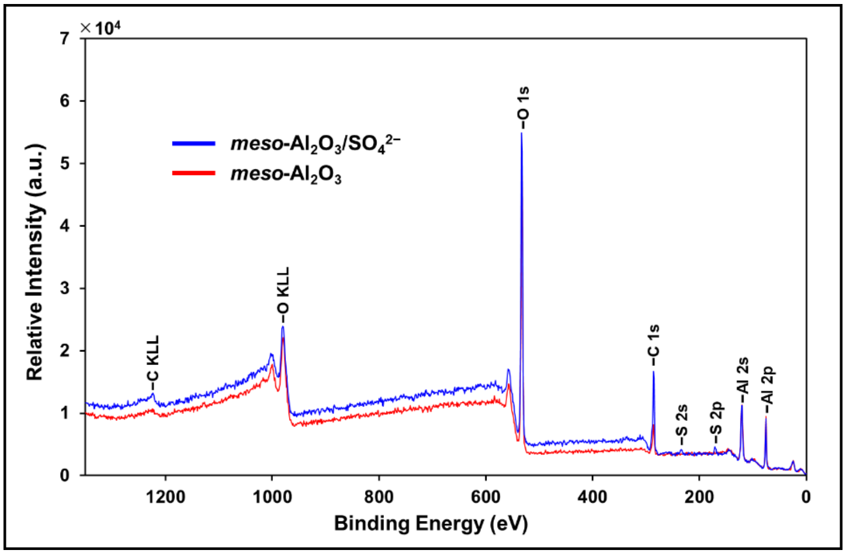

Fig. S6 XPS survey spectra of ***meso*-Al_2O_3/SO_4^{2-}** (blue line) and ***meso*-Al_2O_3** (red line).

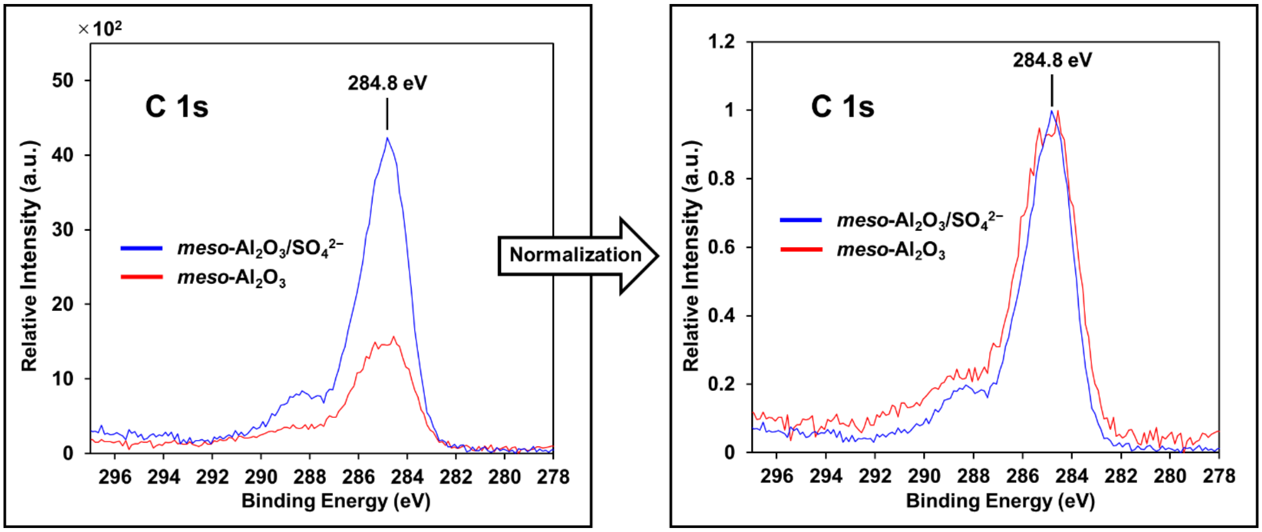

Fig. S7 XPS spectra of C 1s for ***meso*-Al$_2$O$_3$/SO$_4^{2-}$** (blue line) and ***meso*-Al$_2$O$_3$** (red line) used as references, calibrated with the C 1s peak at 248.8 eV, and their normalized spectra.

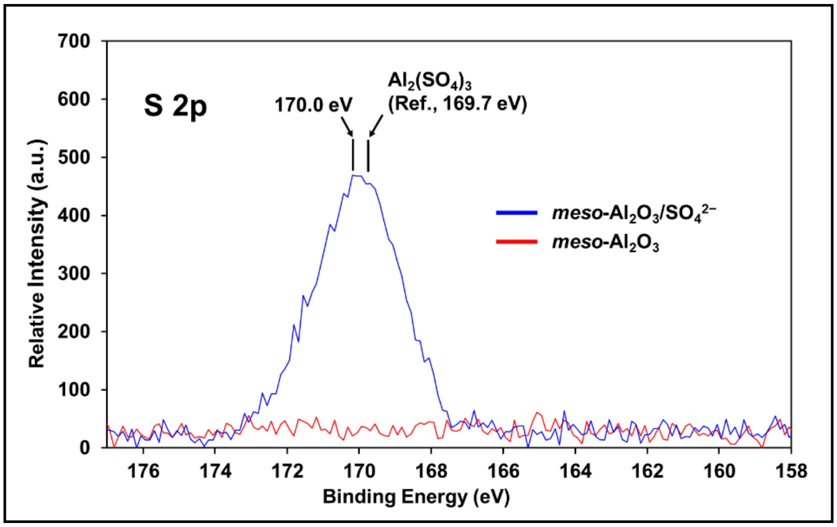

Fig. S8. XPS spectra of S 2p for ***meso*-Al$_2$O$_3$/SO$_4^{2-}$** (blue line) and ***meso*-Al$_2$O$_3$** (red line)

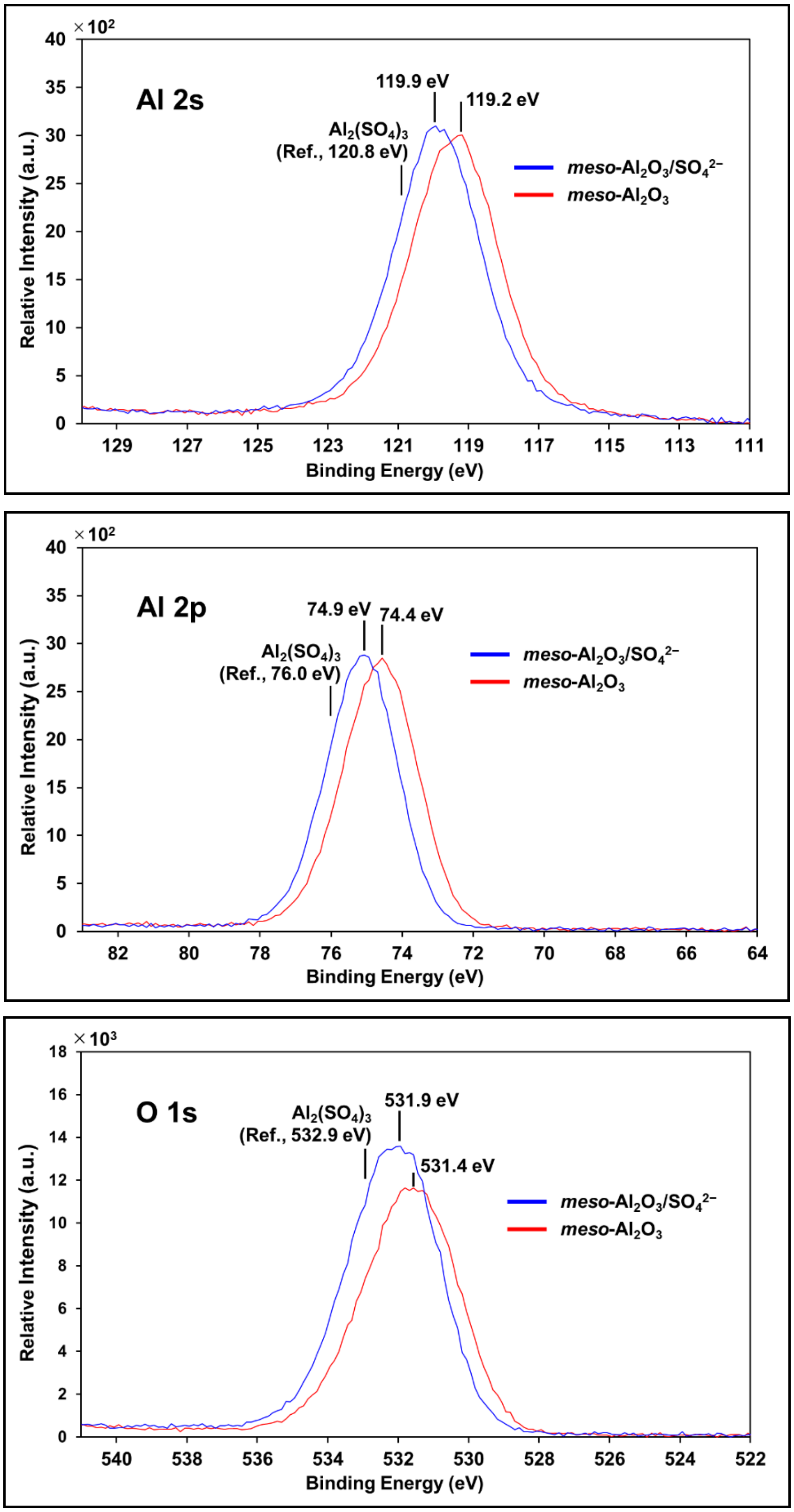

Fig. S9. XPS spectra of Al 2s, Al 2p, and O1s for ***meso*-Al_2O_3/SO_4^{2-}** (blue line) and ***meso*-Al_2O_3** (red line).

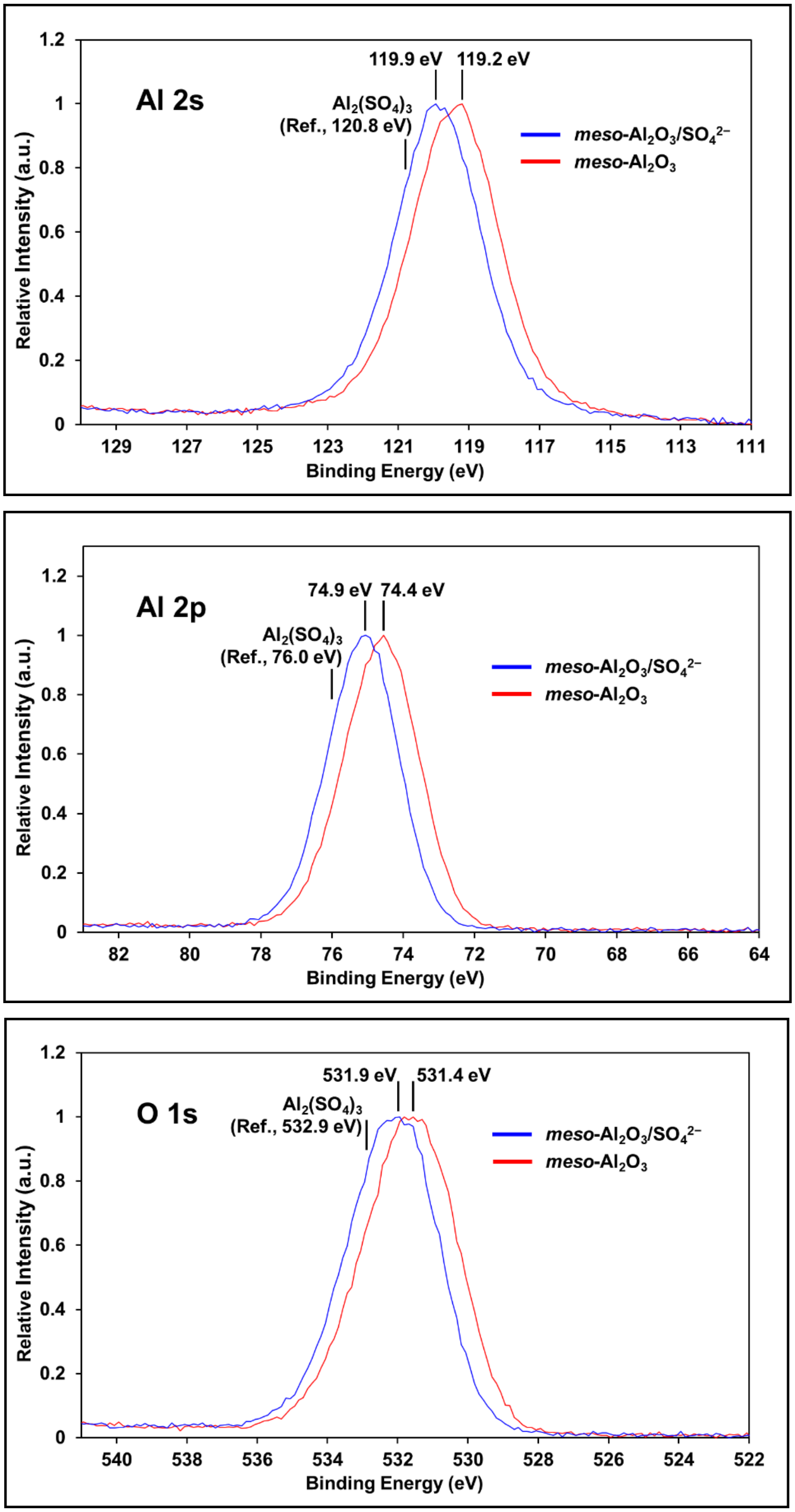

Fig. S10. Normalized XPS spectra of Al 2s, Al 2p, and O1s for ***meso*-Al_2O_3/SO_4^{2-}** (blue line) and ***meso*-Al_2O_3** (red line).

SEM-EDS analysis

The surface morphology and particle sizes of powdery ***meso*-Al$_2$O$_3$/SO$_4^{2-}$** were examined using SEM. The samples were dispersed and coated with an osmium (Os) layer (ca. 1 nm thickness). Observations were carried out with a JSM-IT800SHL SEM, acquiring secondary electron imaging (SEI) and backscattered electron imaging (BEI) images at an accelerating voltage of 1.0 kV with a sample bias of 4.0 kV, over a magnification range of ×3,000–300,000 (Fig. S11 and S12). Elemental analysis by EDS was performed using two JEOL Gather-X Dry SDD Windowless detectors at an accelerating voltage of 1.5 kV, a beam current of 900 pA, and a magnification of ×30,000 (Fig. S13). A small carbon (C) signal was also detected, likely due to the measurement process or surface contamination, and does not indicate intrinsic carbon in the material. The analysis showed that sulfur (S), like oxygen (O) and aluminum (Al), was uniformly distributed throughout the solid, with no aggregation observed.

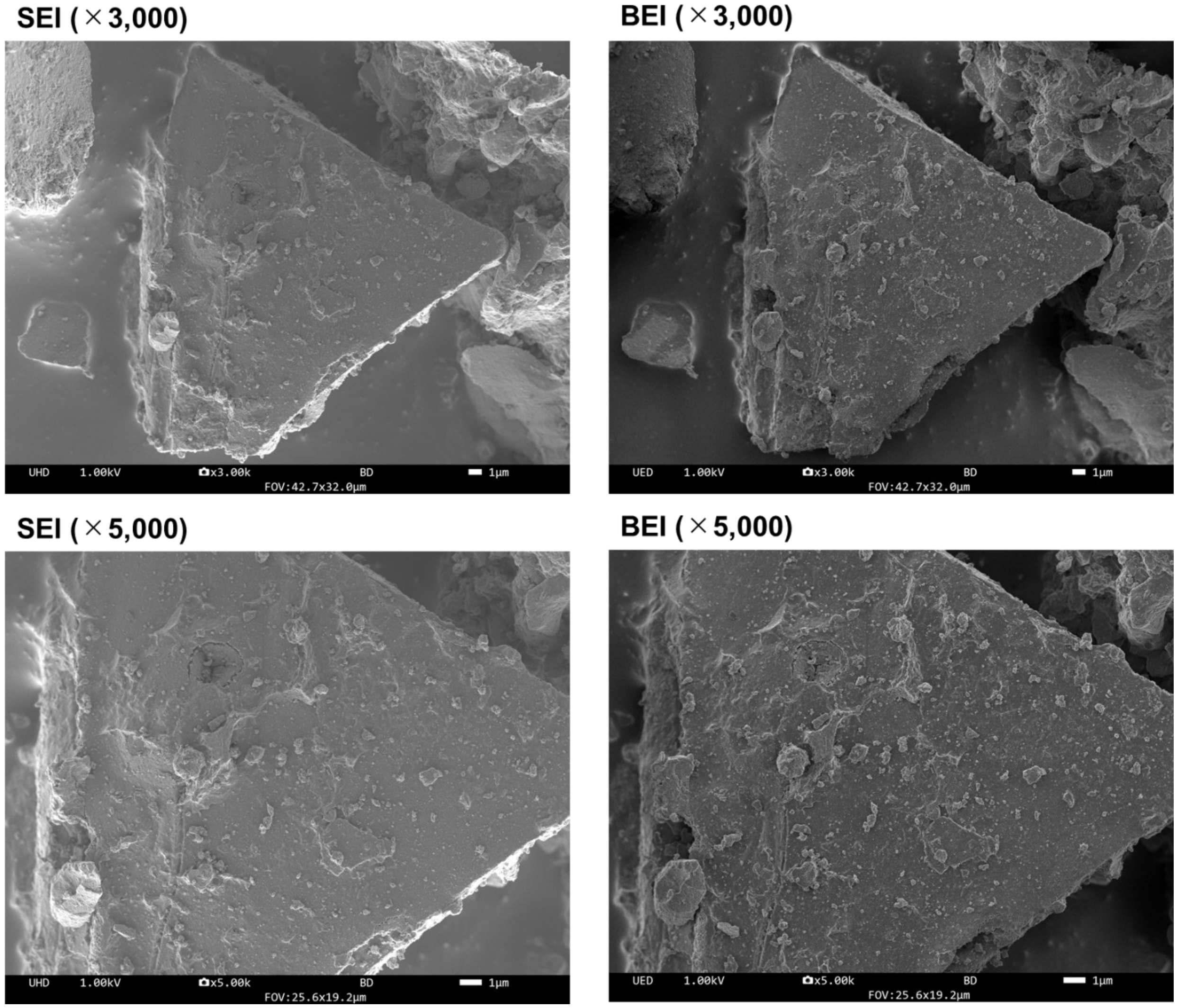

Fig. S11 Low-magnification SEI (secondary electron image) and BEI (backscattered electron image) images of ***meso*-Al$_2$O$_3$/SO$_4^{2-}$**.

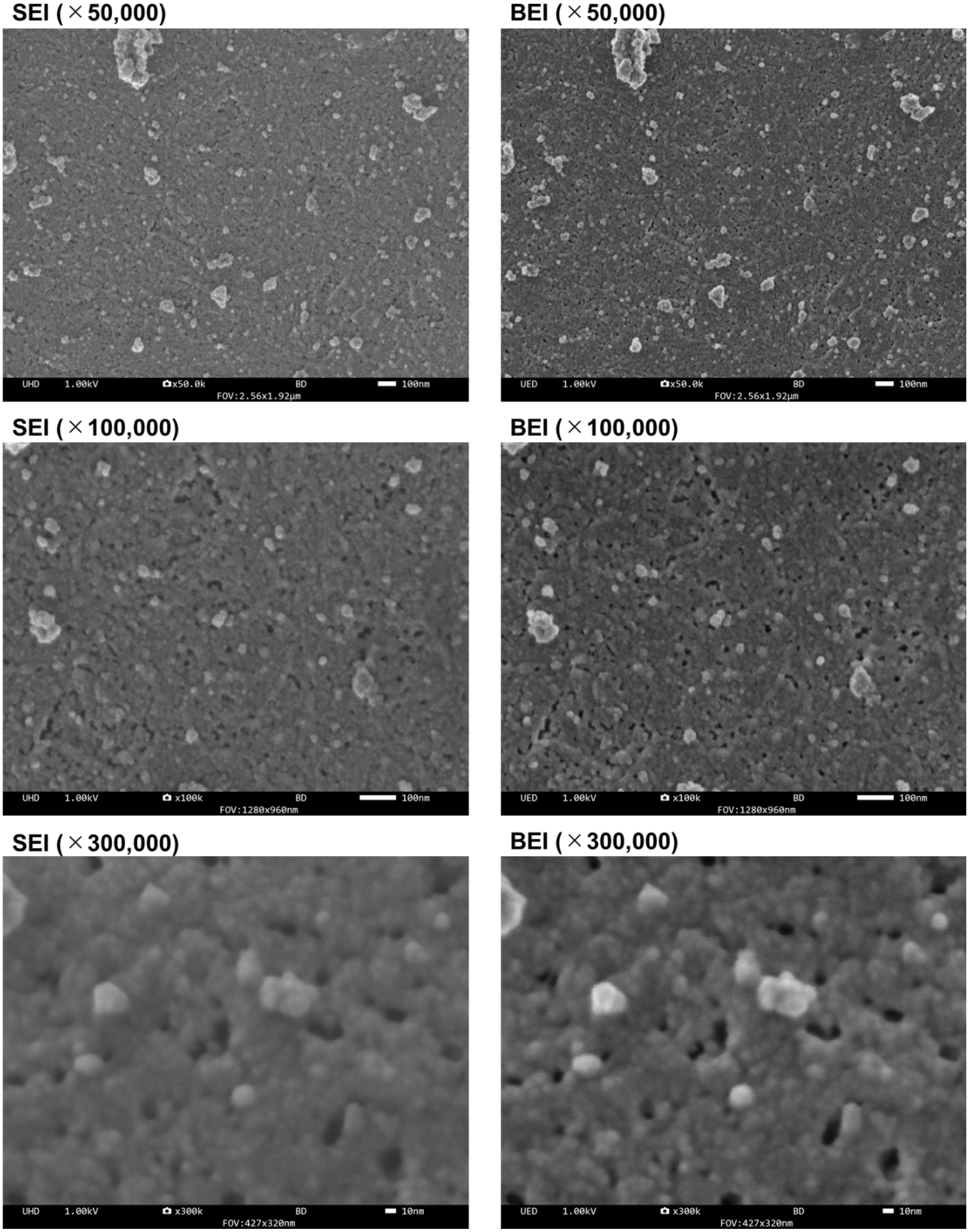

Fig. S12 High-magnification SEI (secondary electron image) and BEI (backscattered electron image) images of ***meso*-Al_2O_3/SO_4^{2-}**.

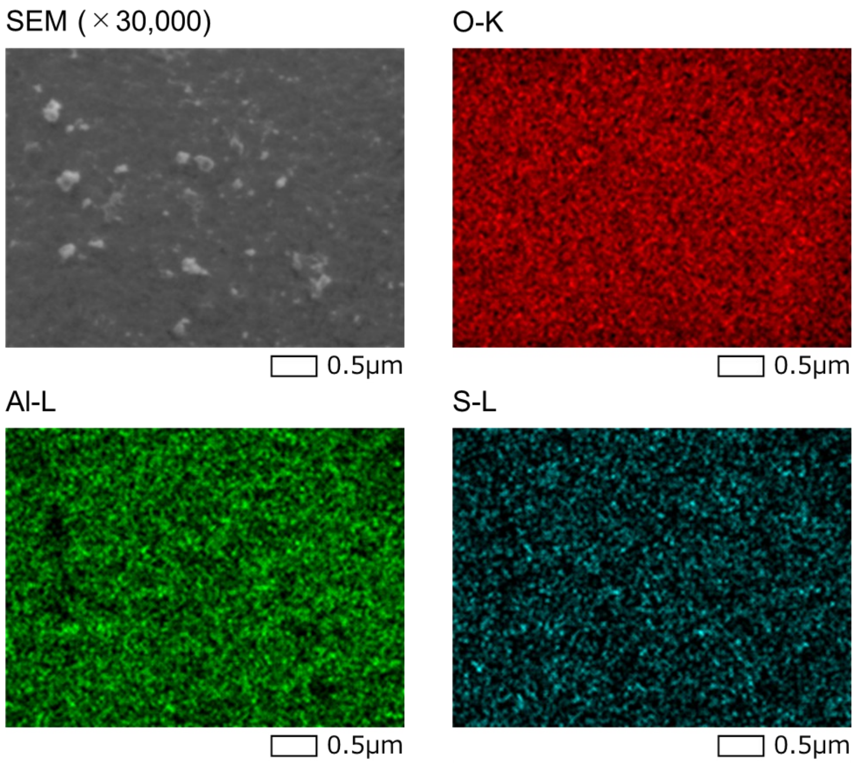

Element	Line	Mass%	Atom%
C	K	7.46±0.01	12.05±0.01
O	K	44.71±0.04	54.25±0.05
Al	L	41.54±0.02	29.89±0.02
S	L	6.30±0.01	3.81±0.01
Total		100.00	100.00
			Fitting coefficient 0.0071

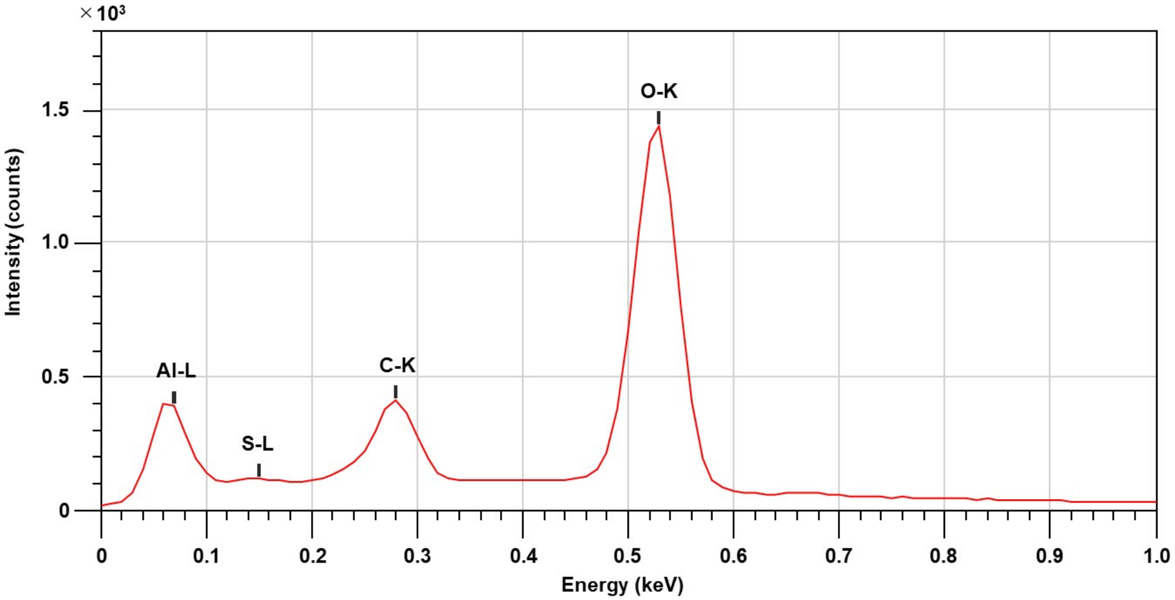

Fig. S13 EDS (energy dispersive X-ray spectroscopy) maps and semi-quantitative elemental analysis of the surface at ×30,000 magnification.

3. Synthesis of *N*–H ketimine and *N*–H ketimine hydrochloride

3.1 Optimization of reaction conditions (Fig. 2a, 2b)

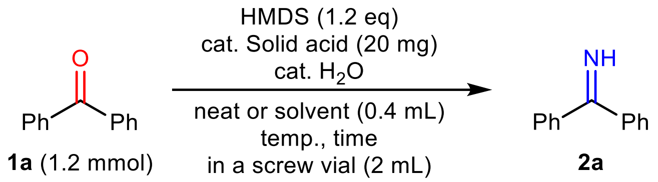

A solid acid was placed in a 10 mL flask, activated under vacuum (<0.5 torr) at 400 °C [$Al_2(SO_4)_3$ and Montmorillonite catalysts such as K10 and M-Mont were activated at 120 °C/<0.5 torr] for 2 h, and then cooled to RT. The activated solid acid (20 mg) was placed in a 2 mL screw vial (PTFE/nitrile inner lid). $Al(OH)_3$, NH_4Cl, $(NH_4)_2SO_4$, polymer-*p*-$C_6H_4SO_3H$ and polymer-$C_6H_4NMe_2$ were used without activation. Benzophenone **1a** (220 mg, 1.20 mmol), water (0–0.36 mmol, 0–15 mol%), and HMDS (232 mg, 1.44 mmol) were added to the vial in that order. If a solvent was necessary, 0.4 mL of solvent was added. After the vial was filled with argon gas and tightly sealed, the mixture was stirred at a specified temperature for a certain period. After the reaction, the reaction solution was filtered by gradual pressurization using a disposable plastic syringe (12 mL, material: injection cylinder/polypropylene, plunger and seal/polyethylene) with a membrane filter (pore size: 0.45 µm, ADVANTEC DISMIC 13HP045AN) installed at the outlet, and the remaining solids were washed with CH_2Cl_2. The filtrate was condensed, a proper amount of 1,4-dioxane or mesitylene was added as the internal standard, and the yield of *N*–H ketimine **2a** was determined by ^1H NMR in $CDCl_3$.

3.2 Investigation on the effects of base additions (Fig. 2c)

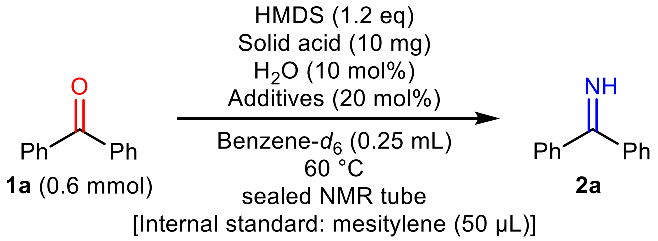

Meso-Al_2O_3/SO_4^{2-} or acidic γ-Al_2O_3 was placed in a 10 mL flask, activated at 400 °C under vacuum (<0.5 Torr) for 2 h, and then cooled to RT. The activated *meso*-Al_2O_3/SO_4^{2-} or acidic γ-Al_2O_3 (10 mg) was placed in an NMR tube. Then, benzophenone **1a** (110 mg, 0.60 mmol), water (1.1 mg, 10 mol%), mesitylene (as the internal standard, 50 µL), base additives (10–20 mol%), HMDS (116 mg, 0.72 mmol), and benzene-d_6 (0.25 mL) were rapidly added to the NMR tube in that order. The NMR tube was sealed with a burner (Fig. S14) and rotated at 10 rpm in an oil bath at 60 °C to start the reaction (a glass tube oven device was used for the rotation, as shown in Fig. S15). The change in the amount of each substance was observed by ^1H NMR in benzene-d_6. Fig. S16 also shows the result without the base added.

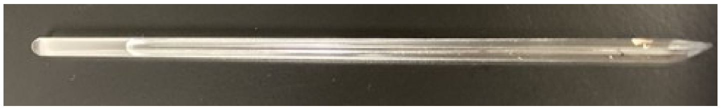

Fig. S14 A sealed NMR tube containing the reaction mixture

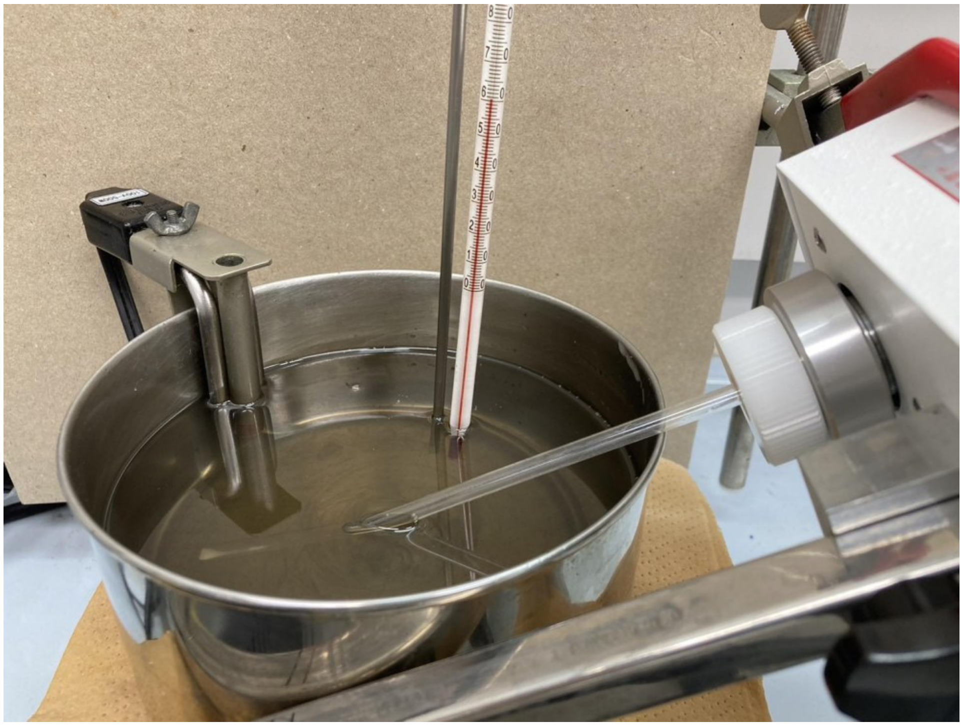

Fig. S15 An experimental setup for investigating the effects of base addition on the formation of *N*–H ketimine **2a**

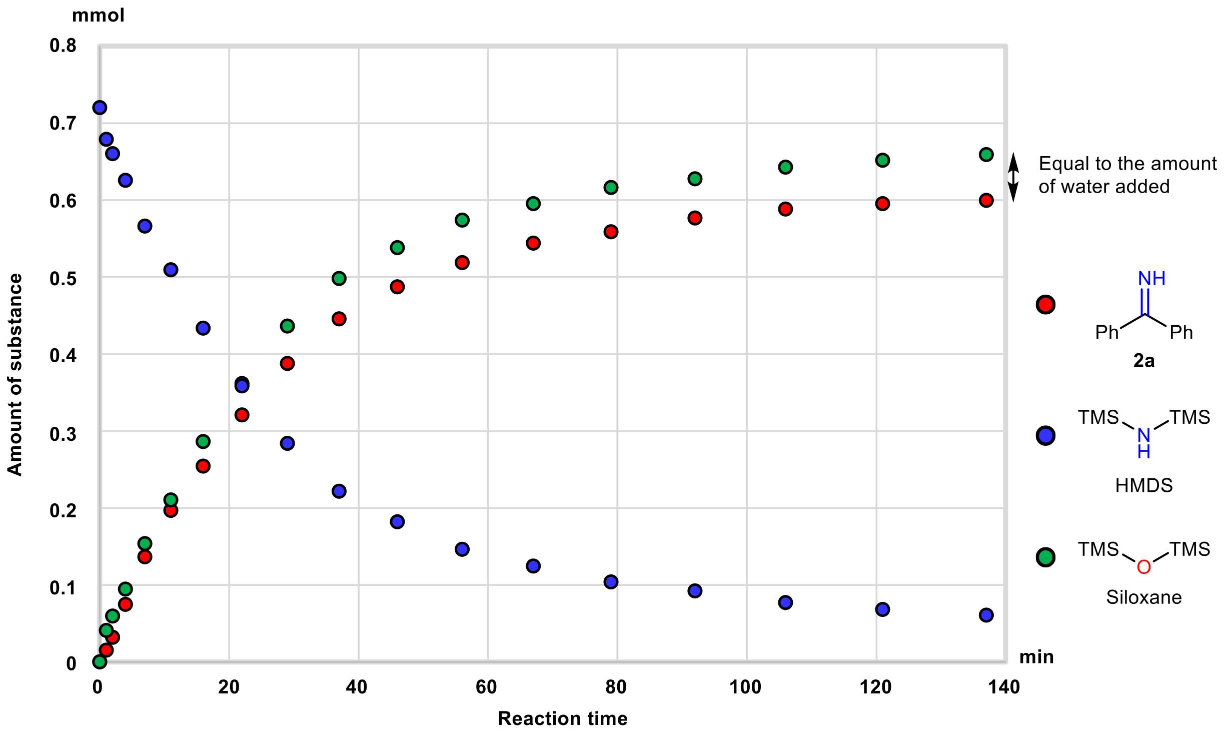

Fig. S16 Amounts of *N*–H ketimine **2a** and siloxane formed over time under the conditions without base addition

3.3 Reactivity of HMDS for methanolysis

To facilitate the isolation of *N*–H ketimines as their hydrochloride salts, we investigated the conversion of residual HMDS into ammonia after the reaction. This allows for complete removal of HMDS by simple gas bubbling. Accordingly, the reaction between excess MeOH and HMDS was examined in the presence of *meso*-Al_2O_3/SO_4^{2-}. A mixture of *meso*-Al_2O_3/SO_4^{2-} catalyst, HMDS, MeOH, benzene-d₆, and mesitylene (internal standard) was placed in a sealed tube (Fig. S14). The tube was stirred at room temperature for the prescribed time to ensure a homogeneous mixture. Changes over time were monitored by ^{1}H NMR spectroscopy (Table S2). As shown in Table S2, vigorous stirring at room temperature for only 10 min led to the decomposition of more than 95% of HMDS. These results indicate that HMDS decomposes very rapidly under acidic catalysis. Based on this finding, the synthesis of *N*–H ketimine hydrochlorides (Section 3.4) was performed at 40 °C for 10 min, followed by 10 min of gas bubbling at room temperature to remove the generated ammonia.

Table S2. Time course of the decomposition of HMDS by excess methanol in the presence of catalyst *meso*-Al_2O_3/SO_4^{2-}

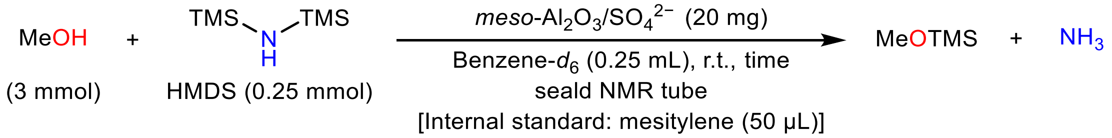

Time/min	Conversion of HMDS/%
5	90
10	95
15	97
20	>99

3.4 General procedure for substrate scopes (Fig. 3a, 3b)

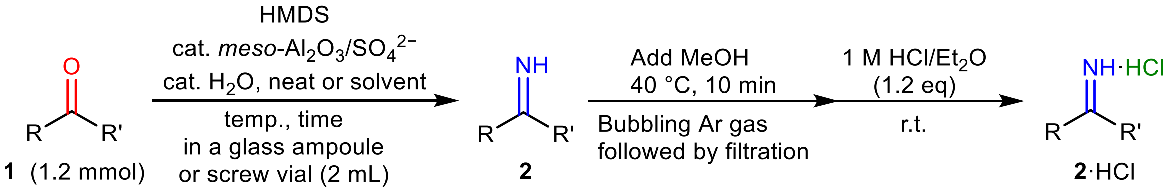

Dry *meso*-Al_2O_3/SO_4^{2-} (20–100 mg) and water (0.12–1.2 mmol, 10–100 mol%) were placed in a 2 mL screw vial (PTFE/nitrile inner lid) or a 2 mL glass ampoule. When a solvent was needed, it was better to use an ampoule. Then, ketone **1** (1.20 mmol) and HMDS (1.44–3.36 mmol) were added to the vessel. When a solvent was necessary, 1.2 mL of the solvent was added. After the vessel was filled with argon gas by blowing it in, it was tightly sealed, as shown in Fig. S17. When using a screw vial, employing a container that does not leak NH_3 gas generated is desirable. The mixture was stirred at a specified temperature for a certain period of time in Fig. S18 (a). Be aware of the ammonia smell when the screw vial is opened. Then, the workup was performed in the following two ways:

(i) **Isolation of *N*–H ketimine**: Dry MeOH (0.1 g, 3 mmol) was added to the reaction mixture, which was then stirred at 40 °C for 10 min. If any substrate does not have hydroxy groups, this operation can be omitted, but it is necessary to perform it in order to completely decompose the remaining HMDS. The resulting mixture was then filtered by gradual pressurization using a disposable plastic syringe (12 mL, material: injection cylinder/polypropylene, plunger and seal/polyethylene) with a membrane filter (pore size: 0.45 μm, ADVANTEC DISMIC 13HP045AN) installed at the outlet, and the remaining solids were washed with CH_2Cl_2 in Fig S18 (b), and the filtrate was condensed. If necessary, freeze-drying with benzene, *t*-BuOH, or 1,4-dioxane was performed. The *N*–H ketimine was isolated by removing the low-boiling-point compounds.

(ii) **Isolation as an *N*–H ketimine hydrochloride salt**: Dry MeOH (0.1 g, 3 mmol) was added to the reaction mixture, which was then stirred at 40 °C for 10 min, and bubbled with argon gas for 10 min (flow rate: 10–20 mL/min). In a glove bag filled with argon gas, the mixture was filtered by gradual pressurization using a disposable plastic syringe (6 mL, material: injection cylinder/polypropylene, plunger and seal/ polyethylene) with a membrane filter (pore size: 0.45 μm, ADVANTEC DISMIC 13HP045AN) at the outlet, and the remaining solids were washed with $CDCl_3$ (3 mL). Subsequently, 1,4-dioxane or mesitylene was added to the filtrate as the internal standard, and the yield of *N*–H ketimine **2** was determined by ^{1}H NMR in $CDCl_3$. Dry Et_2O (5 mL) and 1 M HCl/Et_2O (1.2 eq) were then added to the filtrate and the produced precipitate was filtered off and washed with dry Et_2O. If the solid did not precipitate well, a small amount of the solvent was removed under reduced pressure, and 1–3 mL of ether was added. The mixture was cooled to 4 °C to obtain precipitate. The precipitate was dried to afford an *N*–H ketimine hydrochloride salt **2**·HCl. Throughout the entire procedure, care was taken to minimize exposure to air.

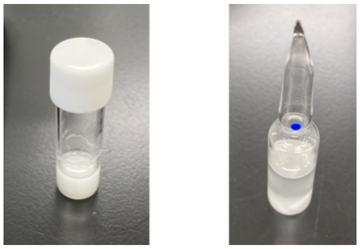

Fig. S17 A screw vial or a glass ampoule containing the reaction mixture

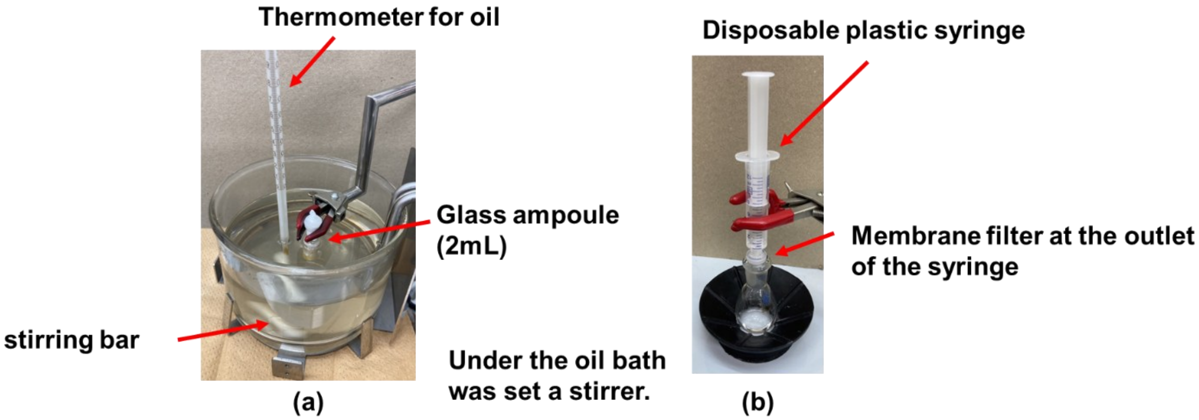

Fig. S18 (a) How to place a reaction vessel in an oil bath on a stirrer. (b) How to set up a syringe and a membrane filter for the filtration system after the reaction.

3.5 Synthesis of *N*–H ketimine on a large scale (Fig. 4a)

A solid catalyst of dry acidic γ-Al_2O_3 (9 g) and water (0.8 g, 44 mmol, 8 mol%) were placed in a 1 L screw-cap autoclave bottle (material: polypropylene). Then, ketone **1a** (100 g, 546 mmol) and HMDS (106 g, 656 mmol) were added to the vessel. After the bottle was filled with argon gas and tightly sealed, the mixture was stirred at 60 °C for 24 h. Afterward, dry MeOH (44 g, 1.37 mol) was added, and the mixture was stirred at 40 °C for 30 min. The mixture was then suction filtered on a glass filter, followed by washing the solid with CH_2Cl_2 (pore size: 0.45 µm). The combined filtrate was passed through a 24 mL disposable plastic syringe (material: injection cylinder/polypropylene, plunger and seal/polyethylene) installing a membrane filter (pore size: 0.45 µm, ADVANTEC DISMIC 13HP045AN) installed at the outlet. A colorless liquid of *N*–H ketimine **2a** was isolated (99 g, 543 mmol, yield >99%).

Following a large-scale reaction of **1a** (100 g), the recovered catalyst was calcined at 600 °C in the air and reused under identical conditions, showing no loss of catalytic activity. γ-Al_2O_3 is thermally stable at approximately 600 °C, and its simple regeneration by calcination in the air represents a significant advantage for large-scale synthesis.

Synthesis of **2i**, **2q**, **2z**·HCl, **2ag**, **2bb**, **2bd**·HCl, **2bf**, and **2bg**·HCl was performed on an approximately 10-fold scale using a 20 mL ampoule and a commercial acidic γ-Al_2O_3 catalyst, following the procedure described in Section 3.3. The reaction times for **2i** and **2q** were extended from 24 to 60 hours, and that for **2z**·HCl was extended from 24 to 48 hours. Additionally, the amounts of HMDS and water were changed from 14.4 to 18.0 mmol and from 10 to 20 mol%, respectively, for **2bf** and **2bg**·HCl.

3.6 Air exposure test for catalyst usability (Fig. 4b)

meso-Al_2O_3/SO_4^{2-}or acidic γ-Al_2O_3 was placed in a 10 mL flask, activated at 400 °C under vacuum (<0.5 Torr) for 2 h, and then cooled to RT. The solid acid was transferred to a petri dish, exposed to the air, and let it stay at RT for at least 3 months. The amount of water adsorbed on the solid acid was measured by an increase in weight after exposure. The reaction was conducted with air-exposed solid acid (20 mg), following the protocol in Section 3.1 without water addition.

3.7 Facile dehydrochlorination of *N*–H ketimine hydrochlorides via salt metathesis (Fig. 4c)

N–H ketimine hydrochloride **2z**·HCl, as synthesized in Section 3.4, was used. The other hydrochloride salts—**2a**·HCl, **2i**·HCl, **2q**·HCl, **2ag**·HCl, and **2bf**·HCl—were prepared from the corresponding isolated *N*–H ketimines using 1 M HCl, following a procedure similar to that in Section 3.3 (see Section 9.1 for the reaction scheme). Following dehydrochlorination, the *N*–H ketimine was purified using two different methods, depending on the solubility of the two by-product salts, Et_3N·HCl and NH_4Cl, in the solvent. Prior to use, hexane and CH_2Cl_2 were dehydrated with activated Molecular Sieves 3A.

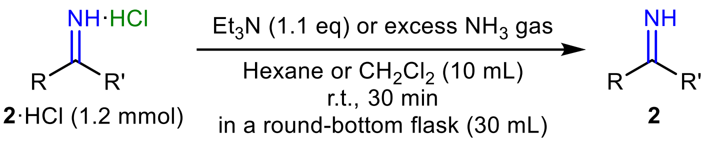

N–H ketimine hydrochloride (1.2 mmol) was suspended in 10 mL of dry hexane or CH_2Cl_2 in a round-bottom flask and treated with either E_3N (1.32 mmol) or excess NH_3 gas. Completely dissolving the *N*–H ketimine hydrochloride was not required. The resulting mixture was stirred at 20–25 °C for 30 min. Then, the workup was performed in the following two ways:

Filtration method: The suspension was filtered, and the flask wall and solids were washed with dry hexane or CH_2Cl_2. The combined filtrate was concentrated under reduced pressure to isolate the free *N*–H ketimine.

Dry short column method: To the reaction mixture, 10 mL of dry hexane was added to induce precipitation. The resulting suspension was passed directly through a dry, neutral silica gel column (Fuji Silysia Chemical Ltd., CHROMATOREX Q-PACK SI30 SIZE20; column length: 7 cm; column diameter: 2 cm; pH = 7.0; 11 g of silica gel activated at 120 °C for 1 h under <0.5 torr) using an eluent mixture of hexane/CH_2Cl_2/Et_3N (65:30:5, v/v/v) and a medium-pressure column separation system of Yamazen EPCLC W-Prep 2XY. The eluate was concentrated to isolate the free *N*–H ketimine. For an explanation of the necessity of drying silica gel, see Section 3.7 below.

3.8 Hydrolysis instability of *N*–H ketimine during silica gel column chromatography

In order to recognize the instability of *N*–H ketimines, we examined how *N*–H diarylketimines **2a**, **2i**, and **2q** (1.0 mmol) were sensitive to hydrolysis on silica gels using flash column chromatography (Table S3). When dried silica gel was used, it was removed from a commercially packed column, activated at high temperature, and repacked into the column for use. The results in Table S3 show that most of *N*–H ketimine **2** was significantly hydrolyzed to ketones **1** after passing through the three columns with an eluent that did not contain Et_3N base. As a result, obtaining high-purity *N*–H ketimines requires tedious pretreatment procedures, such as adding Et_3N to the eluent and drying the silica gel under harsh conditions. These experiments demonstrate that *N*–H ketimines are highly unstable, sensitive to hydrolysis, and very difficult to purify by column chromatography.

Types of silica gel and experimental conditions: Three types of commercially packed silica gels were selected: (1) Neutral silica (Fuji Silysia Chemical Ltd., CHROMATOREX Q-PACK SI30 SIZE20, column length: 7 cm, column diameter: 2 cm, pH = 7.0) After drying at 120 °C/0.5 torr for 1 h, 12 g of the original silica gel used was found to include 4 mmol/g of water, (2) NH_2-modified silica (Fuji Silysia Chemical Ltd., CHROMATOREX Q-PACK NH60 SIZE20, column length: 7 cm, column diameter: 2 cm, pH = 9.5, use of 15 g), and (3) octadecylsilyl (ODS)-modified silica (YAMAZEN CORPORATION, UNIVERSAL Premium ODS-SM, UW219, column length: 7.5 cm, column diameter: 1.5 cm, use of 7 g). A medium-pressure column separation system of Yamazen EPCLC W-Prep 2XY was applied with 8 mL/min of an eluent flow rate. Hexane, EtOAc, and CH_2Cl_2 were used as solvents after drying over molecular sieves 3A (activated at 400 °C for 2 h under 0.5 torr).

Table S3. Sensitivity tests of *N*–H ketimines to hydrolysis on various types of silica gel columns using medium-pressure liquid chromatography

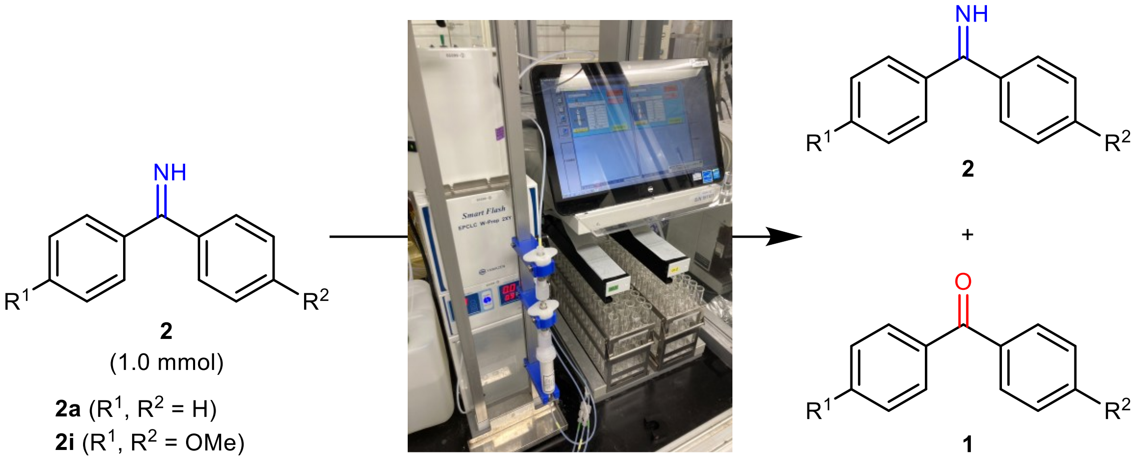

2a (R^1, R^2 = H)
2i (R^1, R^2 = OMe)
2q (R^1= H, R^2 =NO_2)

Ketimine	Silica gel	Eluent composition (Et_3N:Hexane:EtOAc:CH_2Cl_2)	Intact recovery of **2**/%*	Hydrolyzed ketone **1**/%*
**2a**	Neutral silica** (12 g, pH = 7.0)	0:90:10:0	25	74
		10:90:0:0	92	7
			94†	5†
	Dried neutral silica‡ (11 g, pH = 7.0)	10:90:0:0	>99	n.d.
	Dried neutral silica§ (11 g, pH = 7.0)	10:90:0:0	>99	n.d.
	Dried neutral silica¶ (11 g, pH = 7.0)	10:90:0:0	>99	n.d.
	NH_2-modified silica (15 g, pH = 9.5)	10:85:5:0	55	4
	ODS-modified silica (7 g)	MeOH only	96	3
**2i**	Neutral silica** (12 g, pH = 7.0)	0:85:5:10	n.d.	>99
		10:80:0:10	91	8
	Dried neutral silica‡ (11 g, pH = 7.0)	10:80:0:10	>99	n.d.
	Dried neutral silica¶ (11 g, pH = 7.0)	10:80:0:10	>99	n.d.
**2q**	Neutral silica** (12 g, pH = 7.0)	0:85:15:0	39	60
		10:80:10:0	95	4
	Dried neutral silica‡ (11 g, pH = 7.0)	10:80:10:0	>99	n.d.
	Dried neutral silica¶ (11 g, pH = 7.0)	10:80:10:0	>99	n.d.

*NMR yields using 1,4-dioxane as the internal standard. **The water content adsorbed on the silica gel was 4 mmol/g before drying them at 120 °C/0.5 torr for 1 h. † Ketimine **2a** of 2.0 mmol was used. ‡ Dry eluent and the silica gel dried at 400 °C/<0.5 torr for 2 h were used. § Dry eluent and the silica gel dried at 200 °C/<0.5 torr for 1 h were used. ¶ Dry eluent and the silica gel dried at 120 °C/<0.5 torr for 1 h were used.

4. Synthesis of *N*–R ketimines (Fig. 4c)

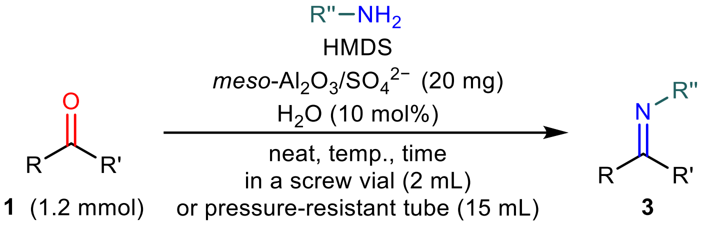

Reaction protocol for 3a, 3b, 3e, and 3f: Dried *meso*-Al_2O_3/SO_4^{2-} (20 mg) and water (0.12 mmol, 10 mol%) were placed in a 2 mL screw vial (Fig. S17, PTFE/nitrile inner lid). Then, ketone **1** (1.20 mmol), amine (1.32 mmol) and HMDS (1.44 mmol) were added to the vial. After the vial was filled with argon gas by blowing it in, it was tightly sealed. The mixture was stirred at 40 °C for 24 h. The internal pressure of the reaction vial was slowly released, followed by the standard workup protocol described below.

Reaction protocol for 3c, 3d, 3g, and 3h: Dried *meso*-Al_2O_3/SO_4^{2-} (20 mg) and water (0.12 mmol, 10 mol%) were placed in a 15 mL pressure-resistant tube (Fig. S19, ACE GLASS). Ketone **1** (1.20 mmol), amine (1.32 mmol), and HMDS (1.80 mmol) were added to the tube. After the vial was filled with argon gas by blowing it in, it was tightly sealed. The mixture was stirred at a specified temperature (**3c**: 100 °C, **3d**: 40 °C, **3g**: 80 °C, **3h**: 140 °C) for 24 h. Then, amine (0.48 mmol) was added, and the reaction mixture was heated at the same temperature for 24 h, followed by the standard workup protocol described below.

Workup protocol: Dry MeOH (0.1 g, 3 mmol) was added to the reaction mixture, which was stirred at 40 °C for 10 min. The mixture was then filtered by gradual pressurization using a disposable plastic syringe (12 mL, material: injection cylinder/polypropylene and plunger, seal/polyethylene) with a membrane filter (pore size: 0.45 μm, ADVANTEC DISMIC 13HP045AN) installed at the outlet, and the remaining solids were washed with CH_2Cl_2. The filtrate was then condensed. If necessary, freeze-drying with benzene was performed. *N*–R ketimines **3** were isolated by removing the low-boiling-point compounds.

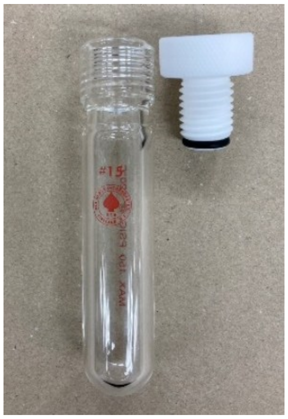

Fig. S19 A pressure-resistant tube (15 mL, ACE GLASS)

5. Synthesis of α-aminonitriles

5.1 General procedure for substrate scopes (Fig. 5a, 5c)

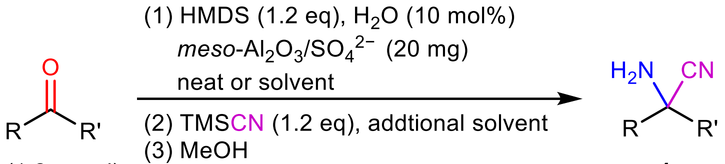

1 (1.2 mmol) **4**

Dried *meso*-Al_2O_3/SO_4^{2-} (20 mg) and water (0.12 mmol, 10 mol%) were placed in a 2 mL screw vial (PTFE/nitrile inner lid). Then, ketone **1** (1.20 mmol) and HMDS (1.44–2.88 mmol) were added to the vial. If a solvent was necessary, 1.2 mL of the solvent was added. After the vial was filled with argon gas by blowing it in, it was tightly sealed. The mixture was stirred at a specified temperature for a certain period. TMSCN (1.44–2.88 mmol) and, if necessary, a solvent was added, and the mixture was stirred under the prescribed conditions. Then, MeOH (0.5 or 1 mL) was also added, and the mixture was stirred under the prescribed conditions. The mixture was filtered by gradual pressurization using a disposable plastic syringe (6 mL, material: injection cylinder/polypropylene, plunger and seal/polyethylene) with a membrane filter (pore size: 0.45 µm, ADVANTEC DISMIC 13HP045AN) installed at the outlet, and the remaining solids were washed with CH_2Cl_2. The filtrate was condensed. If necessary, freeze-drying with benzene was performed. α-aminonitrile **4** was isolated by removing the low boiling-point compounds.

5.2 Hydrolysis instability of α-aminonitrile during silica gel column chromatography

As in Section 3.7, hydrolysis tests were also conducted for α-aminonitrile. We examined how much of aliphatic α-aminonitrile **4h** could survive after passing through the three original and thermally-treated silica columns in the same manner as described in Section 3.7 (Table S4).

Table S4. Hydrolysis studies of α-aminonitrile **4h** with medium-pressure liquid chromatography

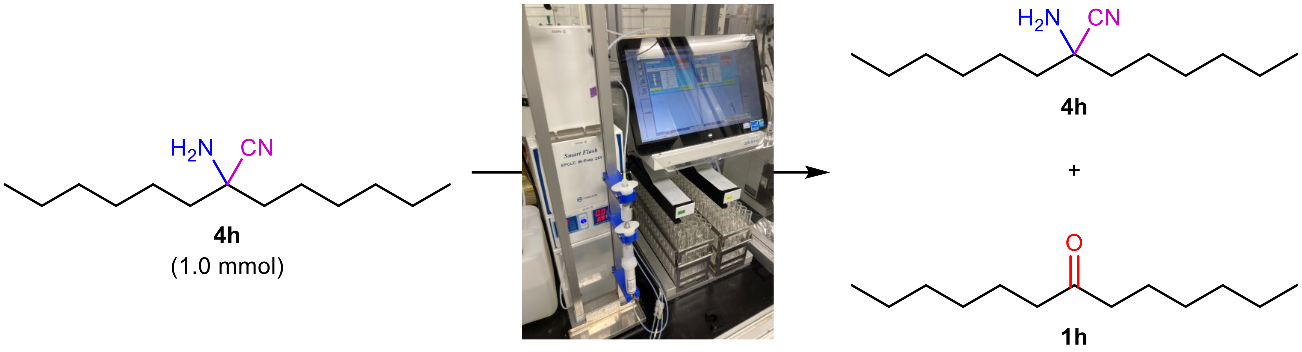

Silica type	Eluent solvents (Et_3N:Hexane:EtOAc)	Intact recovery of **4h**/%*	Hydrolysis to Ketone **1h**/%*
Neutral silica** (12 g, pH = 7.0)	0:80:20	70	29
	10:75:15	90	9
Dried neutral silica† (11 g, pH = 7.0)	10:75:15	>99	n.d.
NH_2-modified silica (15 g, pH = 9.5)	10:75:15	85	14
ODS-modified silica (7 g)	MeOH only	86	13

*NMR yields using 1,4-dioxane as the internal standard. **The water content adsorbed on the silica gel was 0.4 mmol/g before drying them at 200 °C/0.5 torr for 1 h. †Dry eluent and the silica gel dried at 400 °C/<0.5 torr for 2 h were used.

It was demonstrated that aliphatic α-aminonitrile was also unstable on silica gel columns, but that the combined use of a basic eluent and dried silica gel enabled the purification of aliphatic α-aminonitrile to be purified by column chromatography, yielding pure product.

6. Synthesis of hydantoin compounds

6.1 Optimization of the reaction conditions

An optimal amount of $(NH_4)_2CO_3$ and proper reaction temperatures needed for the cyclization of α-aminonitrile were investigated using benzophenone **1a**. The experiment was performed according to the procedure in Section 6, as shown in Table S5. Although 1.2 equiv. of $(NH_4)_2CO_3$ are sufficient, more than 2 equiv. were used to explore the substrate scope in order to ensure the reaction proceeded reliably.

Table S5. Optimization of reaction conditions for hydantoin **5a** synthesis

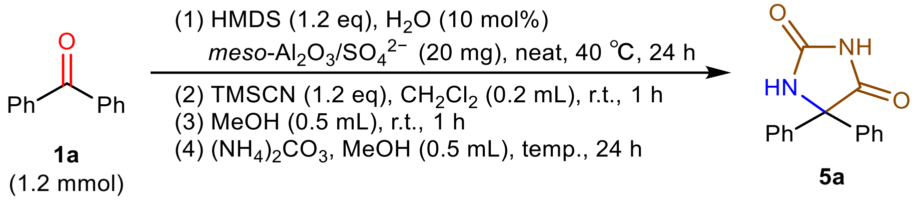

Entry	$(NH_4)_2CO_3$/eq	Temp./°C	Yield of **5a**/%
1	3	70	>99
2	3	60	>99
3	3	40	>99
4	3	r.t.	>99
5	2	r.t.	>99
6	1.2	r.t.	>99

6.2 General procedures for substrate scopes (Fig. 5b, 5c)

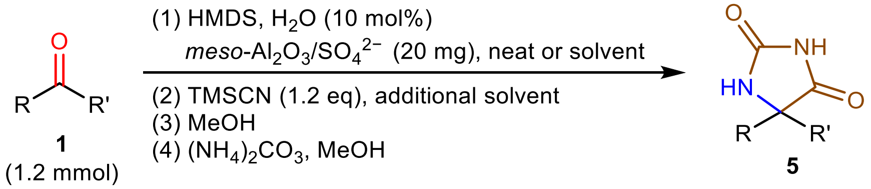

Dried *meso*-Al_2O_3/SO_4^{2-} (20 mg) and water (0.12 mmol, 10 mol%) were placed in a 2 mL screw vial (PTFE/nitrile inner lid). Then, ketone **1** (1.20 mmol) and HMDS (1.44–2.88 mmol) were added to the vial. If a solvent was necessary, 1.2 mL of solvent was added. After the vial was filled with argon gas by blowing it in, it was tightly sealed. The mixture was stirred at a specified temperature for a certain period of time. TMSCN (1.44–2.88 mmol) and, if necessary, CH_2Cl_2 (0.2 mL) were added, and the mixture was stirred under the prescribed conditions. Then, MeOH (0.5–3 mL) was also added, and the mixture was stirred under the prescribed conditions. At this point, the solution was transferred to a 10 mL screw vial (PTFE/nitrile inner lid), depending on the amount of MeOH used. $(NH_4)_2CO_3$ (1.8–7.2 mmol) and MeOH (0.5–3 mL) were added, and the mixture was stirred vigorously at a specified temperature for a certain period. During the reactions at over 40°C, ammonium carbonate may adhere to the top of the vial, but this is not a problem (Fig. S20). The mixture was filtered by gradual pressurization using a disposable plastic syringe (6 mL, material: injection cylinder/polypropylene, plunger and seal/ polyethylene) with a membrane filter (pore size: 0.45 µm, ADVANTEC DISMIC 13HP045AN) installed at the outlet, and the remaining

solids were washed with (CH_2Cl_2:MeOH = 1:1, DMF, or DMSO). The filtrate was then condensed. If necessary, freeze-drying with benzene or reprecipitation with water added was conducted. Hydantoin compounds **5** were isolated by removing the low-boiling-point compounds.

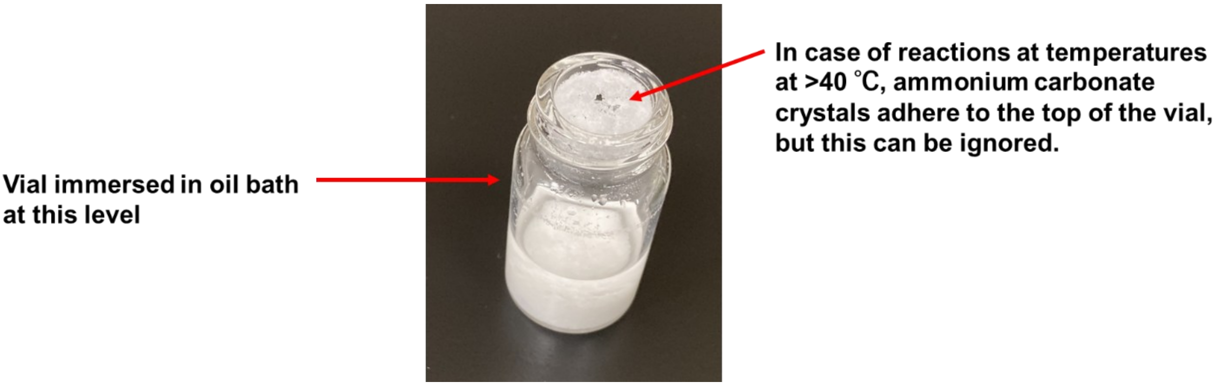

Fig. S20 The vial was opened after the reaction at 40 °C or higher.

6.3 Comparison with the classical Bucherer–Bergs reactions

Control experiments were performed to compare our study with the conventional Bucherer–Bergs reaction[16] for several substrates, where the molar ratios of the reagents used were the same as those in our study.

Experimental procedure: Ketones **1** (1.20 mmol), NaCN (1.44 mmol), $(NH_4)_2CO_3$ (2.4 mmol), and 1.2 mL of H_2O/EtOH (1/1) were added to a 2 mL screw vial. After the vial was filled with argon gas by blowing it in, it was tightly sealed. The mixture was stirred at 80 °C for 24 h. After cooled to RT, the reaction solution was transferred to a 100 mL flask with MeOH, and mixed with silica gel (12 g, YAMAZEN CORPORATION, INJECT COLUMN, W827), and the mixture was completely concentrated. Then, the crude mixture adsorbed on the silica gel was packed in the inject column, and purified using medium-pressure column chromatography using a gradient eluent system (hexane/EtOAc = 9/1 to pure EtOAc, then EtOAc/MeOH = 9/1) and a column of packed separation silica gel (35 g, Fuji Silysia Chemical Ltd., CHROMATOREX Q-PACK SI30 SIZE60, pH = 9.5) to afford hydantoins **5** in Table S6. Hydantoin **5r**, which has a steroid skeleton, has low solubility in organic solvents other than highly polar solvents such as DMSO and cannot be sufficiently eluted by column chromatography. Therefore, the NMR yield was determined for the mixture obtained by reprecipitation with water.

As shown in Table S6, except for simple hydantoins **5k** and **5m**, most hydantoins **5** were only synthesized in low yields. In particular, as shown in Fig S21 (b), which is a ^{1}H NMR spectrum of crude **5f** prepared by the Bucherer–Bergs reaction, the product **5f** was found to contain a large amount of inseparable byproducts. On the other hand, our catalytic reaction does not require separation by column chromatography and can synthesize various pure hydantoins, including **5f**, in nearly quantitative yields at low temperatures. This simple method is expected to replace the classical Bucherer-Bergs reaction.

Table S6. The classical Bucherer–Bergs reactions with several ketones[a]

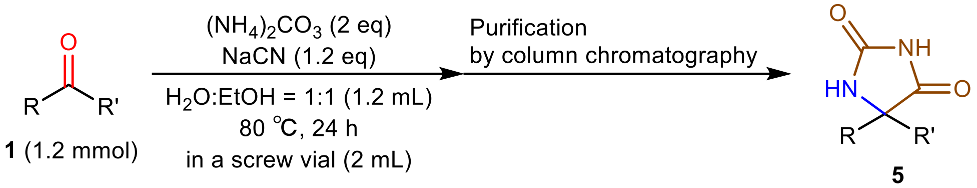

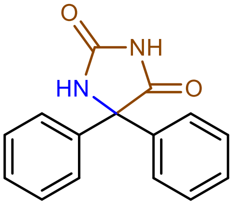

5a

58% (Ketone **1a**: 37%)[b]
[40 °C: n.d. (Ketone **1a**: >99%)[b]]

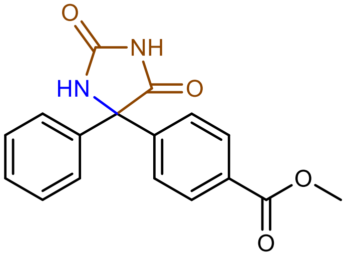

5b

18%[c] (Ketone: 2%[d])[b]

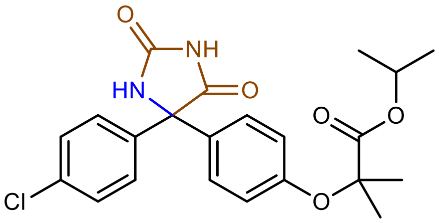

5c

14% (Ketone **1k**: 85%)[b]

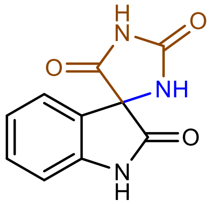

5f

Complex mixturee

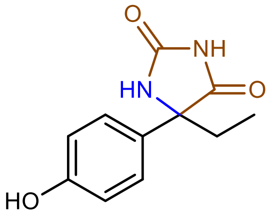

5g

72% (Ketone **1ar**: 3%)[b]

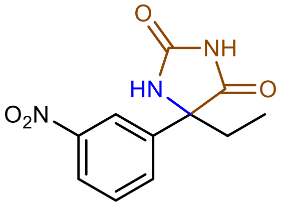

5h

87% (Ketone **1at**: trace)[b]

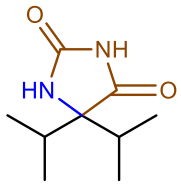

5k

98%
(40 °C: 30%)

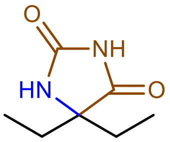

5m

92%
(r.t.: 91%)

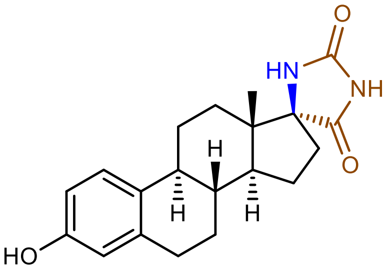

5r

52%[f], d.r. = 78:22 (Ketone **1bp**: 44%)[f]
70%[f,g], d.r. = 85:15 (Ketone **1bp**: 21%)[f]

[a] Isolated yield. [b] Recovery of ketone **1**. [c] Containing a byproduct (14%) of an ethyl ester changing from the product **5b**. [d] Most of the recovery were ketones of ethyl esters. [e] A large number of inseparable byproducts were formed. [f] ^{1}H NMR yields calculated from the reprecipitated mixture. [g] $(NH_4)_2CO_3$ (4 eq) was used.

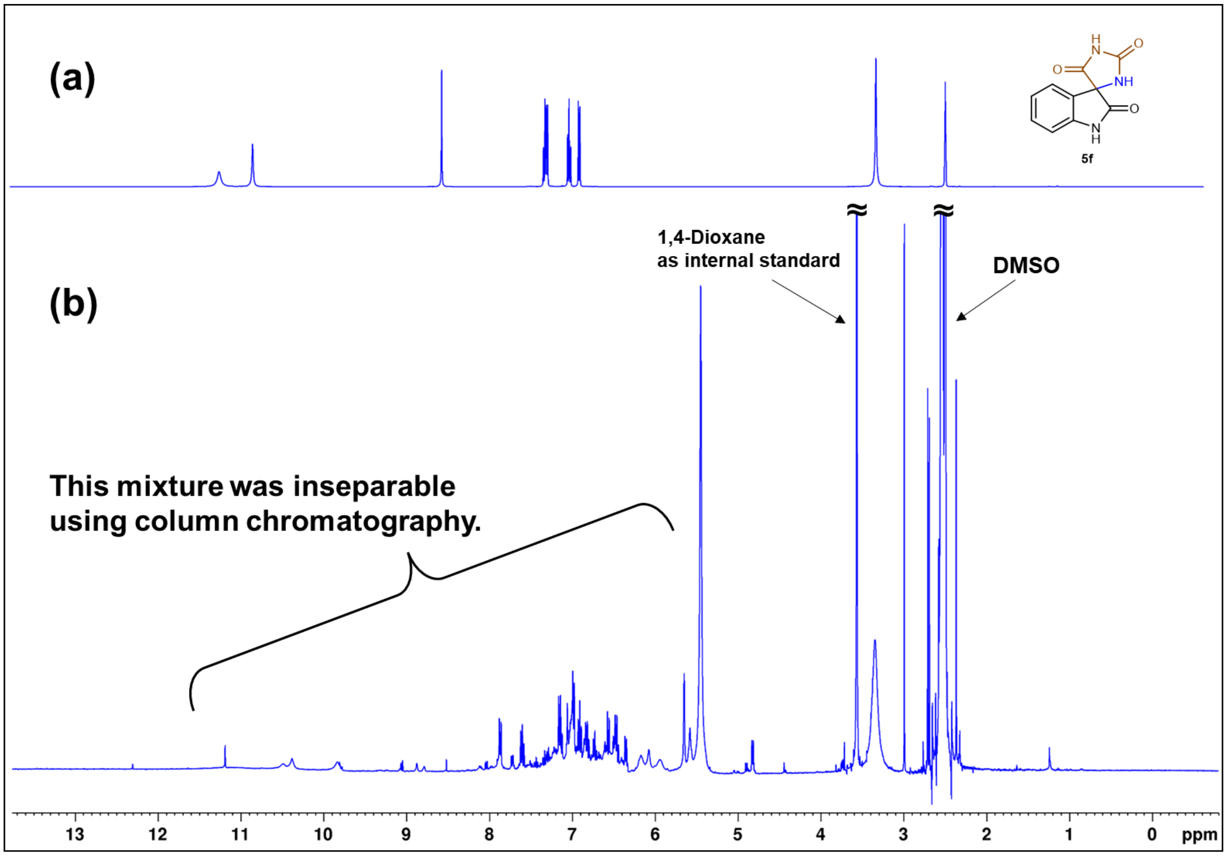

Fig. S21 Definite differences in the ^{1}H NMR spectra (400 MHz in DMSO-d_6) of hydantoin **5f**. (**a**) The isolated product **5f** after filtering and concentrating the reaction solution using our solid acid method (**b**) Crude product after filtering with DMSO using the classical Bucherer–Bergs reaction.

III Quantum chemical calculation methods and results

Quantum chemical calculations were performed at RT using Gaussian 16[1]. The calculation method used was APFD[17] which takes dispersion forces into account and is more reliable than B3LYP. The coordinate data calculating the γ-Al_2O_3 cluster are provided in Section V.

7. Thermodynamic evaluations on the condensation of a ketone with ammonia or an amine to form a ketimine

N–R ketimines are often used as key intermediates in the synthesis of nitrogen-containing compounds. By contrast, there are few examples of taking advantage of *N*–H ketimines, as discussed in the main manuscript. This is because of the thermodynamic stability/instability problems of the ketimines. We calculated the ΔG for several condensation reactions of typical ketones with ammonia/amine to form the corresponding ketimines (Fig. S22). These calculations were performed at the APFD/6-311+G(2d,p) level. The results clearly show that *N*–R ketimines are much more stable than *N*–H ketimines in all cases. On the other hand, when the water generated from these condensations reacts with HMDS to form siloxanes, the formation of *N*–H ketimines becomes more thermodynamically favorable (Fig. S22). Therefore, we conclude that the formation of stable siloxane bonds makes the condensation reaction irreversible, which leads to the formation of labile *N*–H ketimines.

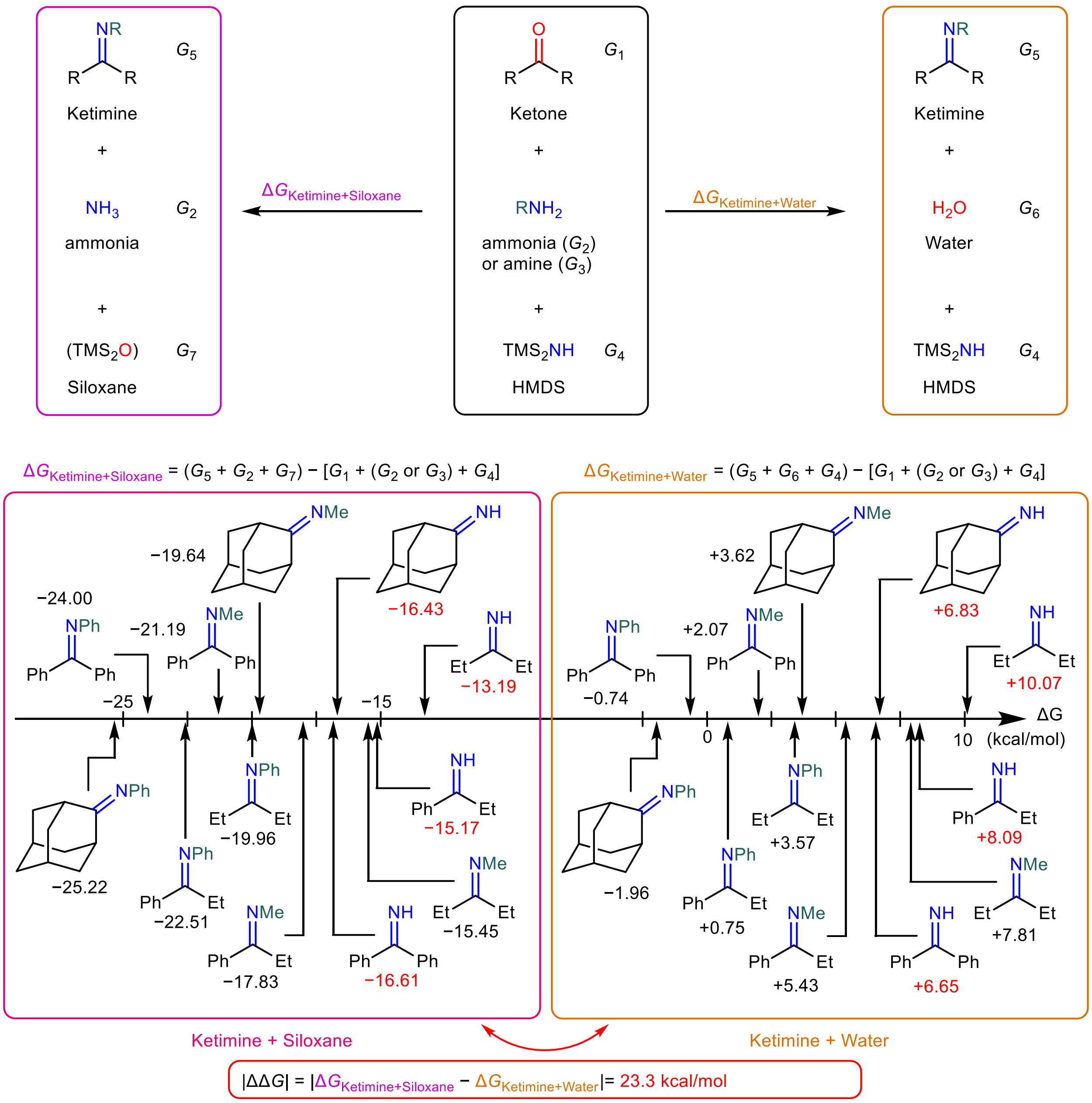

Calculation method : APFD/6-311+G(2d,p)

Fig. S22 Calculated results for condensation reactions using ammonia or amines to produce water or siloxane

8. Exploration into reaction pathways (Fig. 2c)

The following three nitrogen nucleophiles can participate in the formation of *N*–H ketimine: (i) NH_3, (ii) $TMSNH_2$ or (iii) HMDS, which first reacts with the ketone activated on alumina. The experimental result in Fig. 2b of the main manuscript indicated that no reaction occurred unless a small amount of H_2O was added to the reaction mixture. This suggests that HMDS does not directly react with the ketone.

Then, we compared the differences in activation energy for the reactions of the ketone with NH_3 or $TMSNH_2$. As mentioned, the active Lewis acid site on γ-Al_2O_3 is conventionally considered to be a three-coordinated Al atom.[18-20] Similarly, the active site on *meso*-Al_2O_3/SO_4^{2-}, which shows the highest catalytic activity among solid acids, is presumed to be a three-coordinated Al atom, based on the results of the base addition experiments in Section 3.2. Initially, we created a cluster ($[AlO_3]_{clus}$) that mimics the surface structure of γ-Al_2O_3, with reference to Refs. 18, 19, and 20. The $[AlO_3]_{clus}$ was then optimized at the APFD/6-31+G(d,p) level. In the subsequent calculations, the atoms other than the three-coordinated Al and the three O atoms bonded to the $[AlO_3]_{clus}$ around the reaction site were fixed to simulate the rigid framework structure of γ-Al_2O_3. Transition states (TS) in the addition process of NH_3 or $TMSNH_2$ were calculated at the APFD/6-31G(d,p) level by placing a NH_3 or $TMSNH_2$ close to the ketone coordinated to the three-coordinated Al atom. Single-point energies and frequencies were calculated at the APFD/6-311+G(2d,p) and APFD/6-31G(d,p) levels, respectively. After confirming the presence of one imaginary vibration in the TS structure, the IRC calculations were perfomed, followed by the structural optimization of the starting and ending points to obtain the energies for the reactant and product structures. The desorption process of H_2O from *N*–H ketimine or *N*–TMS ketimine adsorbed on the $[AlO_3]_{clus}$ was also calculated in the same way (Fig. S23). Fig. S23 also shows the calculated results without the use of $[AlO_3]_{clus}$. Additionally, a figure of the atomic configuration resulting from the calculation using $[AlO_3]_{clus}$ is shown in Fig. S24.

Fig. S23 shows that the reactions without a catalyst are uphill reactions, where the products (Ph_2C=NH or Ph_2C=NTMS, along with H_2O) are more unstable, as indicated by their higher ΔG, than the reactants (Ph_2C=O, and NH_3 or $TMSNH_2$). On the other hand, when $[AlO_3]_{clus}$ was applied, the reaction turns into a downhill one. It is thought that this is due to the fact that the adsorption energy of imines is higher than that of ketones. In fact, when the adsorption energies of the reactants and products on the cluster were calculated, it was found that the adsorption energies of the imines (Ph_2C=NH and Ph_2C=NTMS) was the highest (Table S7). Therefore, the bonds between $[AlO_3]_{clus}$ and the imine compounds were stronger than the bond between $[AlO_3]_{clus}$ and the ketone, which is one of the factors driving the reaction forward. Furthermore, in pathway (ii) with $[AlO_3]_{clus}$, the overall energy is higher than in pathway (i) due to the steric hindrance from the bulky TMS groups (compare the activation energy, $\Delta\Delta G_{\mathrm{TS\ III}} - \Delta\Delta G_{\mathrm{TS\ I}}$ = 2.5 kcal/mol, with that of $\Delta\Delta G_{\mathrm{TS\ IV}} - \Delta\Delta G_{\mathrm{TS\ II}}$ = 7.5 kcal/mol).

Based on these calculation results and the experimental data presented in Sections 3.1 and 3.2 (see also Fig. 2 in the main manuscript), we can conclude that the ketone primarily reacts with NH_3, rather than with $TMSNH_2$ (Fig. S25).

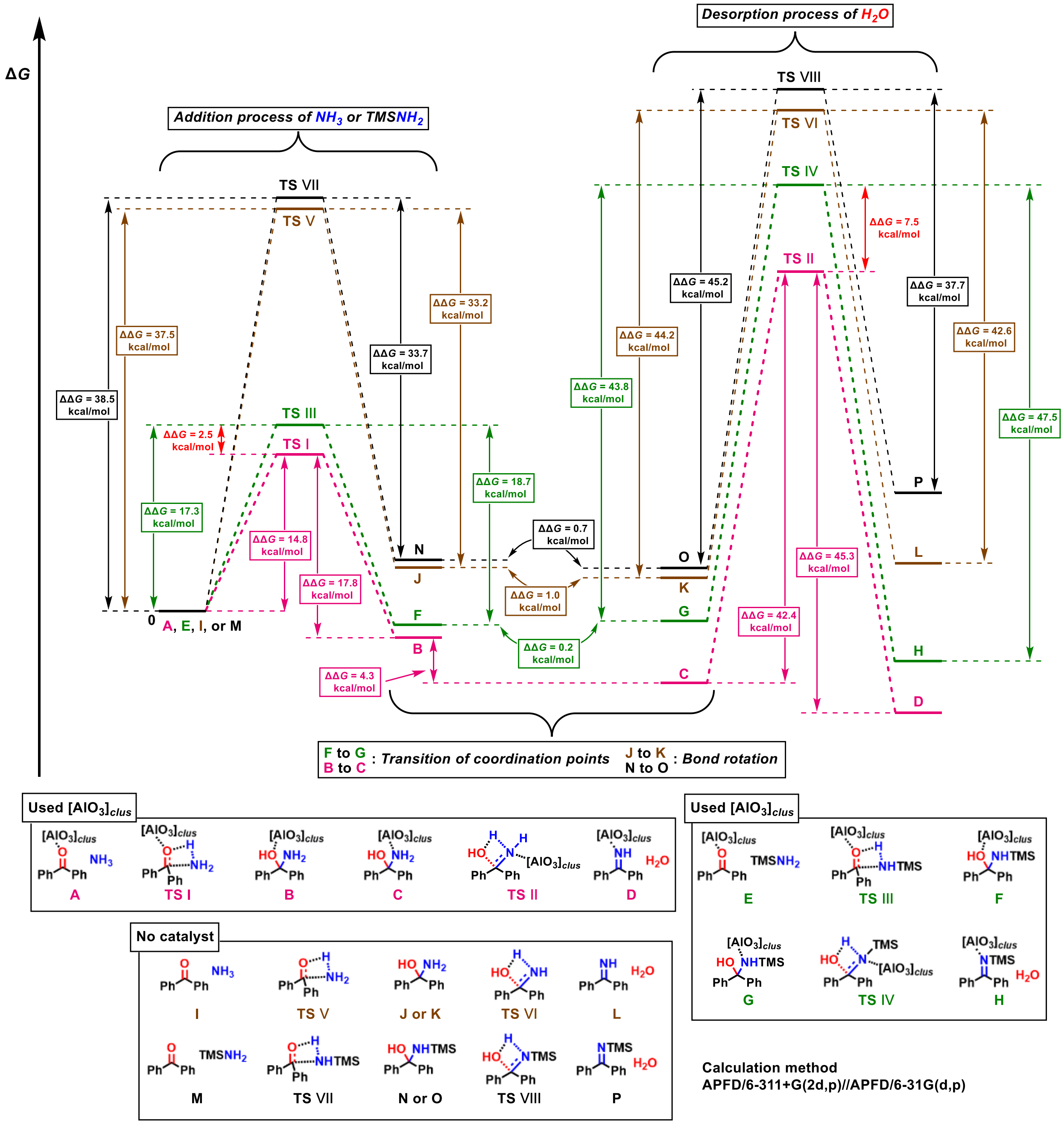

Fig. S23 Δ*G* change diagrams for the calculations and each structure in the reaction process

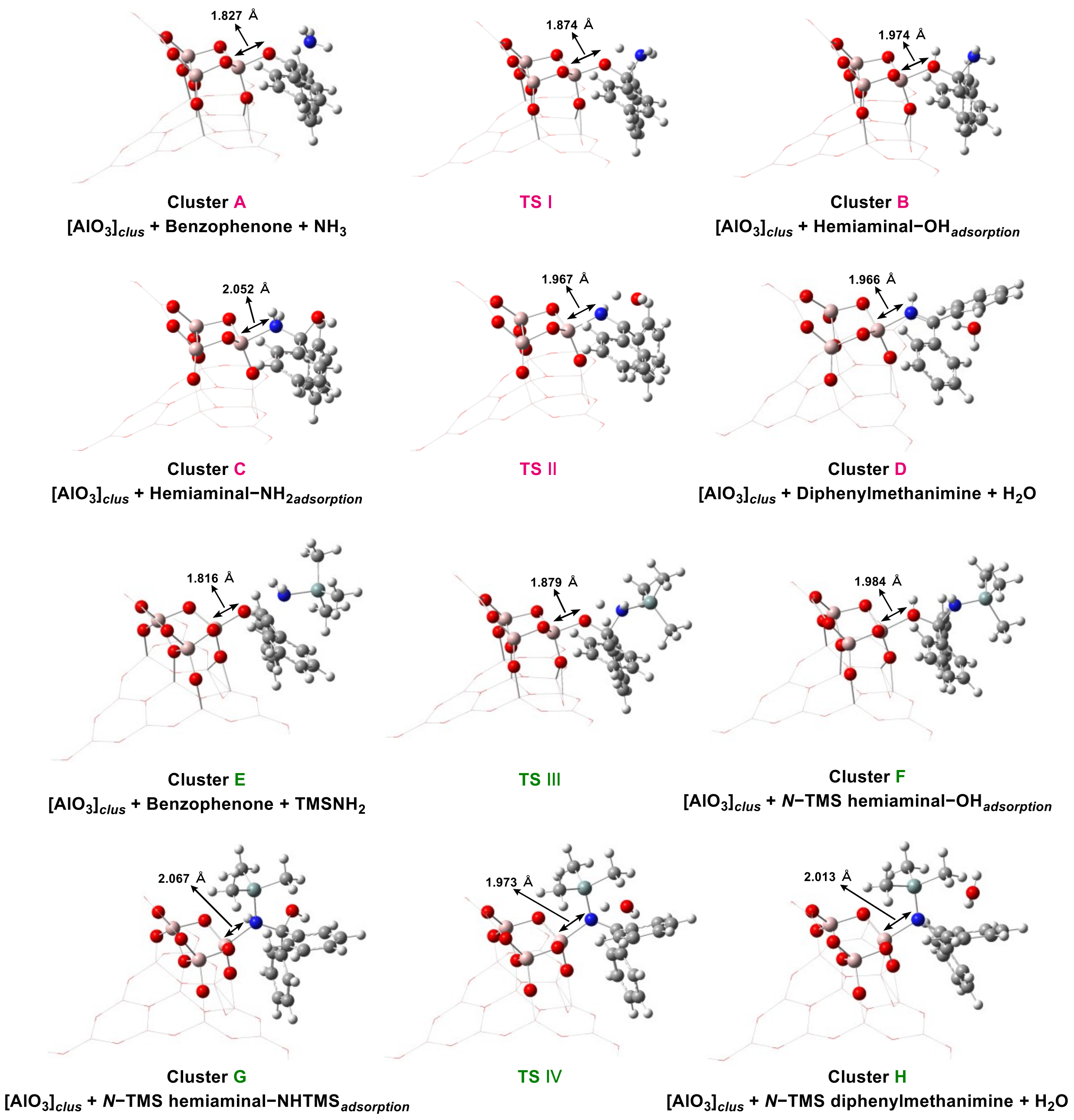

Fig. S24 Structures of each calculated cluster in Fig. S23

Table S7. Calculation results of adsorption energy.

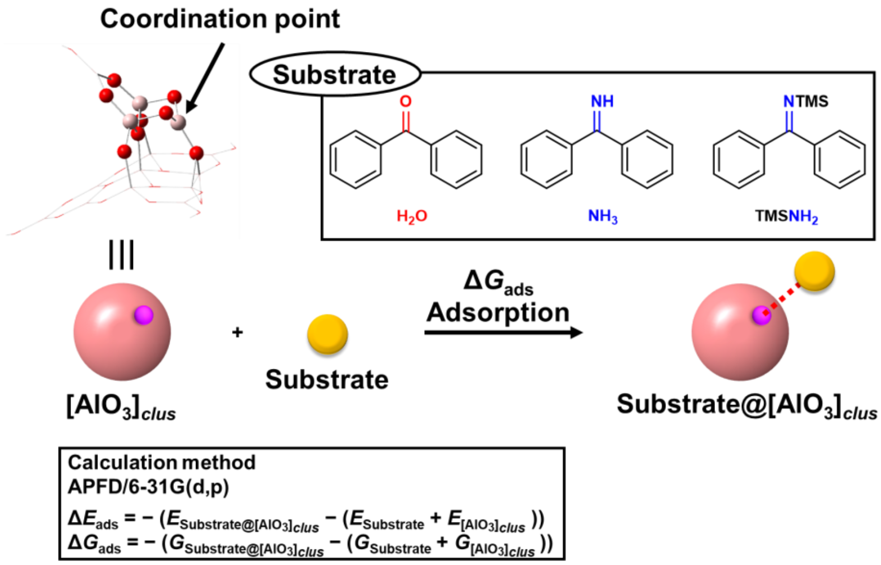

Calculation method	APFD/6-311+G(2d,p)// APFD/6-31G(d,p)		APFD/6-31+G(2d,p)// APFD/6-31G(d,p)		APFD/6-31G(d,p)		
Substrate	ΔE_{ads} (kcal/mol)	ΔG_{ads} (kcal/mol)	ΔE_{ads} (kcal/mol)	ΔG_{ads} (kcal/mol)	ΔE_{ads} (kcal/mol)	ΔG_{ads} (kcal/mol)	Coordination bond distance (Å)
$Ph_2C=O$	38.38	23.25	36.82	21.69	41.96	26.83	1.902
$Ph_2C=NH$	47.84	34.01	46.71	32.88	52.00	38.17	1.980
$Ph_2C=NTMS$	46.21	30.30	45.13	29.23	50.74	34.84	2.018
H_2O	26.88	15.15	25.65	13.91	33.45	21.71	1.971
NH_3	36.43	25.76	35.55	24.87	41.76	31.73	2.016
$TMSNH_2$	–*	–*	40.92	25.77	46.88	31.08	2.992

* Using the input settings of SCF=DIIS and SCF=QC, the energy did not converge, and as a result, this value could not be determined.

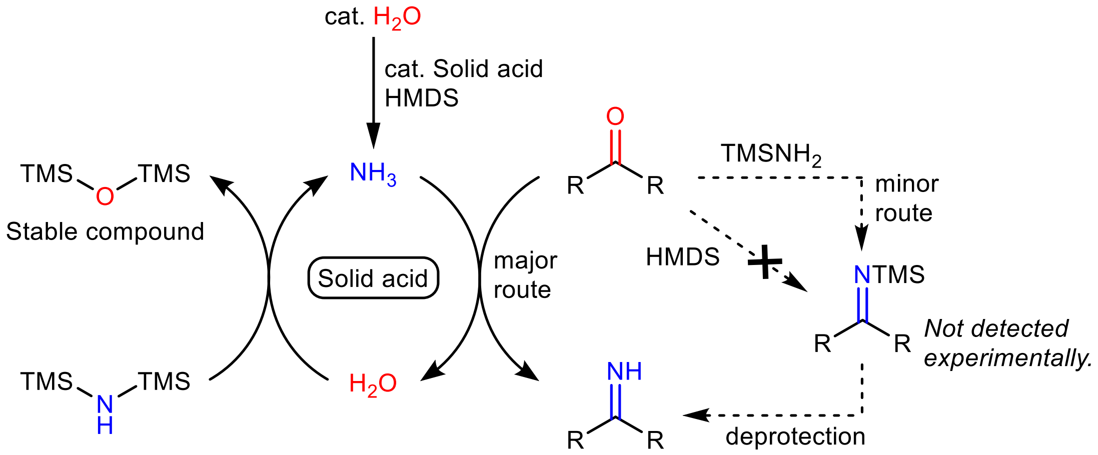

Fig. S25 Proposed reaction mechanism for the synthesis of *N*–H ketimine

IV Compound data

9. Reaction conditions and product assignments

9.1 *N*–H Ketimine and *N*–H ketimine hydrochloride

The solid samples of *N*–H ketimines and their hydrochloride salts are thermally highly unstable. Even when gently heated under conventional Schlenk conditions, complete prevention of hydrolysis to the corresponding ketones is difficult. Decomposition often occurs before melting, and discoloration is frequently observed. Therefore, accurate melting point could not be measured. It is considered that extremely strict anhydrous and oxygen-free conditions would be required for such measurements.

Asymmetric *N*–H ketimines are known to exsist as both *E* and *Z* isomers in solution and to undergo their mutual exchanges.[21] The isomer ratios may or may not be averaged, depending on factors such as molecular structure, solvent, and concentration in solution. When both *E* and *Z* isomers were observed in ^{1}H NMR spectra, and their configurations could not be assigned using NOESY and ROESY spectra, the assignments were made based on quantum chemical calculations using Gaussian 16. Specifically, the isomer assignments were determined by considering the difference in Gibbs free energy (ΔG) between the two isomers, as well as the up/down trends in the chemical shift values (δ) for the imino protons of both isomers, which were obtained through GIAO calculations for NMR. For stable structures containing rotational isomers of aromatic rings, the contribution of each rotational isomer was calculated from its ΔG and δ values, using the equilibrium constant formula ($K = \exp(-\Delta G/RT)$). Structure optimization, frequency, and GIAO calculations were performed at the APFD/6-311+G(2d,p) level using the SMD method to account for solvent effects in $CDCl_3$ or DMSO. The resulting calculated values and coordinate data are presented in Section V.

Diphenylmethanimine (2a)

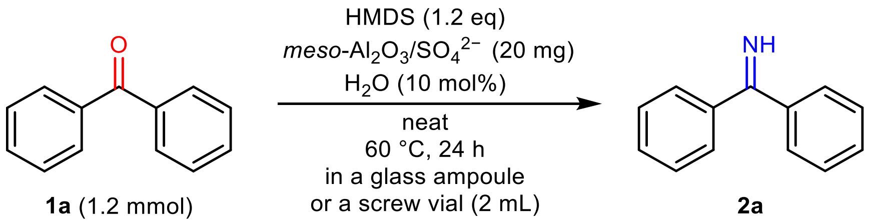

Isolated as a colorless liquid (yield >99%).

^{1}H NMR (400 MHz, DMSO-d_6): δ = 10.53 (br, 1H), 7.67–7.59 (m, 2H), 7.58–7.49 (m, 8H,) ppm.

^{13}C NMR (100 MHz, DMSO-d_6): δ = 175.51, 139.44, 138.42, 130.30, 129.70, 128.62, 128.43, 128.17, 127.49 ppm.

ESI-quadrupole-Orbitrap MS (*m/z*): [M+H]$^+$ calcd for $C_{13}H_{12}N^+$, 182.0964; found 182.0963.

Diphenylmethanimine hydrochloride (2a·HCl)

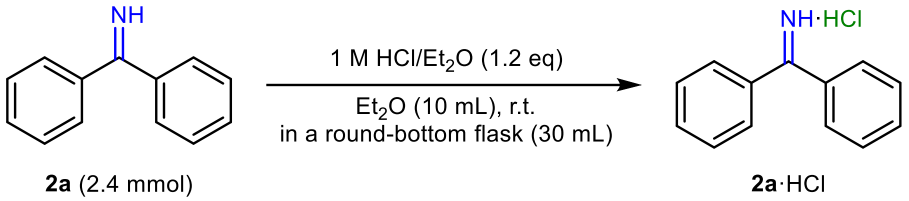

Isolated as a white solid (yield 96%).

^{1}H NMR (400 MHz, DMSO-d_6): δ = 13.03 (br, 2H), 7.86 (t, 2H, J_{HH} = 7.5 Hz), 7.81 (d, 4H, J_{HH} = 7.5 Hz), 7.68 (t, 4H, J_{HH} = 7.5 Hz) ppm.

^{13}C NMR (100 MHz, DMSO-d_6): δ = 181.79, 135.19, 131.74, 130.80, 129.06 ppm.

ESI-quadrupole-Orbitrap MS (*m/z*): $[M-Cl]^+$ calcd for $C_{13}H_{12}N^+$, 182.0964; found 182.0962.

Di-*p*-tolylmethanimine (2b)

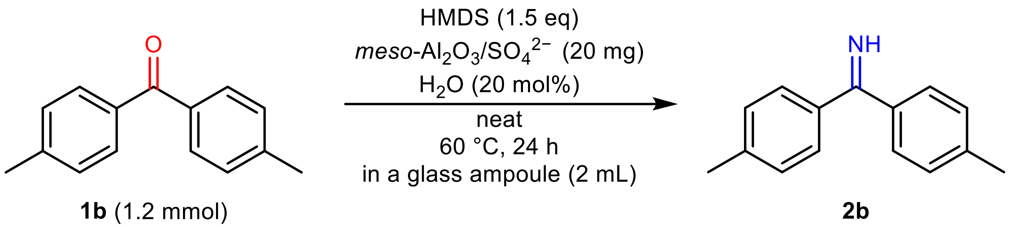

Isolated as a white solid (yield >99%).

^{1}H NMR (400 MHz, DMSO-d_6): δ = 10.27 (br, 1H), 7.51 (d, 2H, J_{HH} = 8.1 Hz), 7.32–7.20 (m, 6H), 2.36 (s, 6H) ppm.

^{13}C NMR (100 MHz, DMSO-d_6): δ = 175.29, 139.92, 139.31, 136.87, 135.83, 128.90, 128.67 (2C), 127.47, 20.88, 20.80 ppm.

ESI-quadrupole-Orbitrap MS (*m/z*): $[M+H]^+$ calcd for $C_{15}H_{16}N^+$, 210.1277; found 210.1275.

(4-(*tert*-Butyl)phenyl)(phenyl)methanimine (2c)

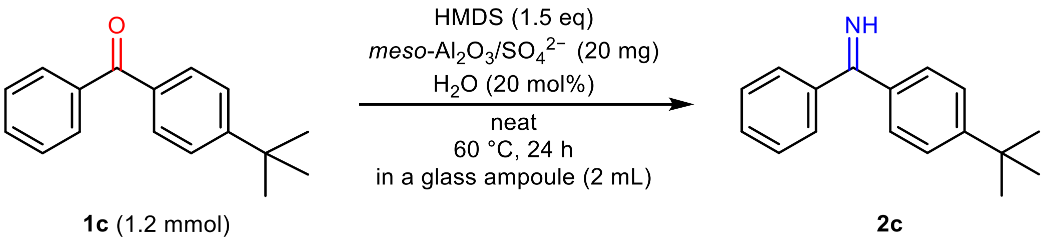

Isolated as a colorless liquid (yield >99%).

^{1}H NMR (400 MHz, DMSO-d_6): δ = 10.46 (br, 0.52H, *Z*), 10.37 (br, 0.48H, *E*), 7.75–7.27 (m, 9H, *E*+*Z*), 1.31 (s, 9H, *E*+*Z*) ppm. Isomer assignment was determined by ROESY measurements.

^{13}C NMR (100 MHz, $CDCl_3$ *E*+*Z* isomers): δ =175.35, 175.29, 153.00, 152.34, 139.68, 138.54, 136.59, 135.61, 130.16, 129.56, 128.65, 128.46, 128.36, 128.08, 127.43, 127.33, 125.17, 124.90, 34.46, 34.45, 30.92 (2C) ppm.

ESI-quadrupole-Orbitrap MS (*m/z*): $[M+H]^+$ calcd for $C_{17}H_{20}N^+$, 238.1590; found 238.1589.

Bis(4-(*tert*-butyl)phenyl)methanimine (2d)

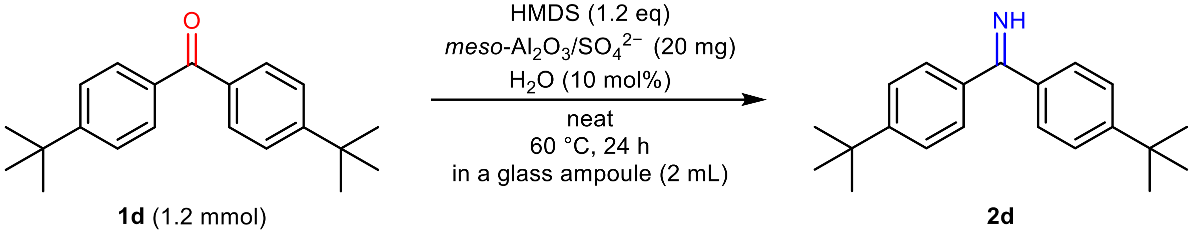

Isolated as a white solid (yield >99%).

^1H NMR (400 MHz, DMSO-*d*6): δ = 9.59 (br, 1H), 7.56 (d, 2H, J_{HH} = 8.1 Hz), 7.47 (m, 4H), 7.34 (d, 2H, J_{HH} = 8.1 Hz) 1.31 (s, 18H) ppm.

^{13}C NMR (100 MHz, DMSO-*d*6): δ = 175.1, 152.9, 152.2, 136.8, 135.77, 128.50, 127.32, 125.13, 124.88, 34.46 (2C), 30.95 (2C) ppm.

ESI-quadrupole-Orbitrap MS (*m/z*): $[M+H]^+$ calcd for $C_{21}H_{28}N^+$, 294.2216; found 294.2214.

Bis(4-fluorophenyl)methanimine (2e)

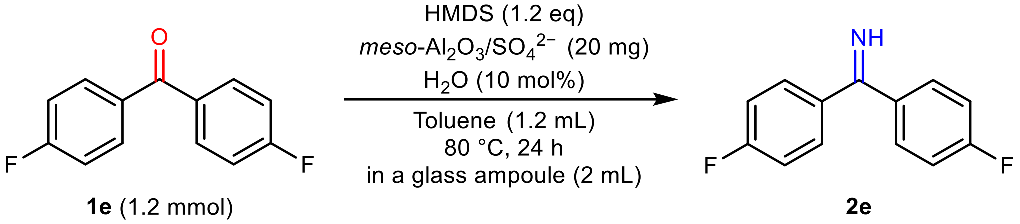

Isolated as a colorless liquid (yield >99%).

^1H NMR (400 MHz, DMSO-*d*6): δ = 10.58 (br, 1H, C=N*H*), 7.71–7.63 (m, 2H), 7.52–7.44 (m, 2H) 7.35–7.21 (m, 4H) ppm.

^{13}C NMR (100 MHz, DMSO-*d*6): δ = 173.2, 163.5 (d, J_{CF} = 248 Hz), 162.8 (d, J_{CF} = 248 Hz) 135.7 (d, J_{CF} = 3 Hz), 134.9 (d, J_{CF} = 3 Hz), 131.0 (d, J_{CF} = 9 Hz), 130.0 (d, J_{CF} = 9 Hz), 115.4 (d, J_{CF} = 22 Hz), 115.4 (d, J_{CF} = 22 Hz), 115.1 (d, J_{CF} = 22 Hz) ppm.

^{19}F NMR (376 MHz, DMSO-*d*6): δ = −114.2 to −114.2 (m, 1F), −114.7 to -114.6 (m, 1F) ppm.

ESI-quadrupole-Orbitrap MS (*m/z*): $[M+H]^+$ calcd for $C_{13}H_{10}F_2N^+$, 218.0776; found 218.0776.

Bis(4-chlorophenyl)methanimine (2f)

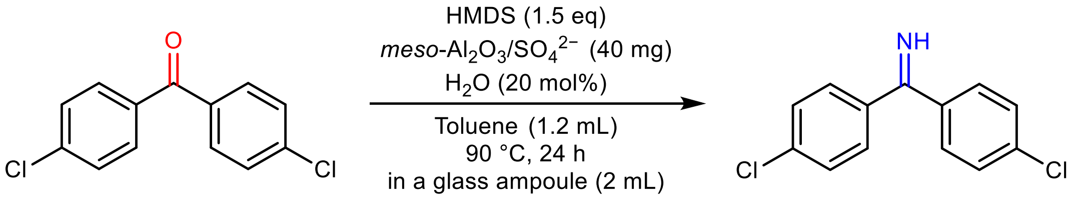

1f (1.2 mmol) **2f**

Isolated as a white solid (yield >99%).

^{1}H NMR (400 MHz, DMSO-d_6): δ = 10.77 (br, 1H), 7.62 (d, 2H, J_{HH} = 8.3 Hz), 7.53 (d, 2H, J_{HH} = 8.3 Hz), 7.50 (d, 2H, J_{HH} = 8.3 Hz), 7.42 (d, 2H, J_{HH} = 8.3 Hz) ppm.

^{13}C NMR (100 MHz, DMSO-d_6): δ = 173.20, 137.58, 136.94, 135.30, 134.59, 130.37, 129.46, 128.51, 128.34 ppm.

ESI-quadrupole-Orbitrap MS (*m/z*): $[M+H]^+$ calcd for $C_{13}H_{10}Cl_2N^+$, 250.0185; found 250.0182.

(4-Bromophenyl)(phenyl)methanimine (2g)

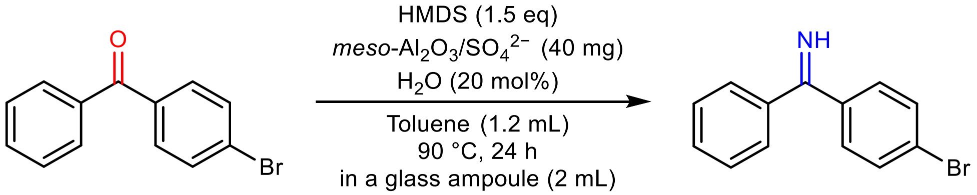

1g (1.2 mmol) **2g**

Isolated as a colorless liquid (yield >99%).

^{1}H NMR (400 MHz, DMSO-d_6, *E*+*Z* isomers): δ = 10.66 (br, 1H), 7.72–7.30 (m, 9H) ppm.

^{13}C NMR (100 MHz, DMSO-d_6, *E*+*Z* isomers): δ = 174.54, 174.36, 138.88, 138.43, 138.05, 137.59, 131.37, 131.21, 130.65, 130.44, 129.86, 129.67, 128.56, 128.49, 128.23, 127.48, 124.03, 123.17 ppm.

ESI-quadrupole-Orbitrap MS (*m/z*): $[M+H]^+$ calcd for $C_{13}H_{11}BrN^+$, 260.0096; found 260.0067.

4-(Imino(phenyl)methyl)phenol (2h)

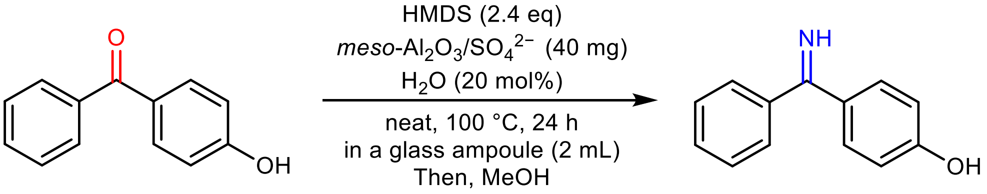

1h (1.2 mmol) **2h**

Isolated as a light yellow solid (yield >99%).

^{1}H NMR (400 MHz, DMSO-d_6): δ = 9.98 (br, 2H), 7.72–7.24 (m, 7H), 6.80 (d, 2H, J_{HH} = 8.6 Hz) ppm.

^{13}C NMR (100 MHz, DMSO-d_6): δ = 175.00, 159.69, 139.76, 130.07, 129.63, 129.14, 128.22, 128.00, 115.07 ppm.

ESI-quadrupole-Orbitrap MS (*m/z*): [M+H]$^+$ calcd for $C_{13}H_{12}NO^+$, 198.0913; found 198.0911.

Bis(4-Methoxyphenyl)methanimine (2i)

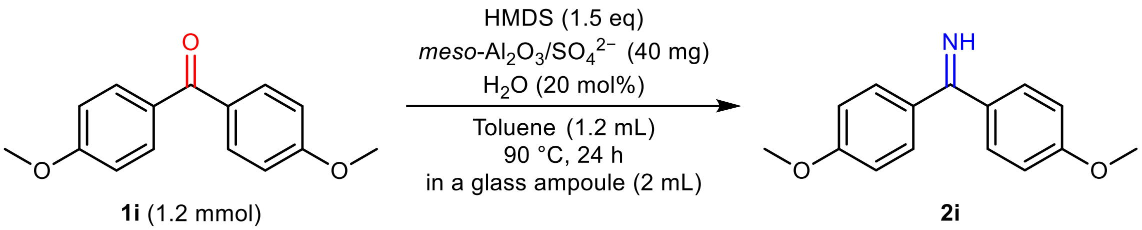

Isolated as a white solid (yield >99%).

^{1}H NMR (400 MHz, DMSO-d_6): δ = 9.35 (br, 1H), 7.51 (br, 4H), 6.94–6.87 (m, 4H), 3.84 (s, 6H) ppm.

^{13}C NMR (100 MHz, DMSO-d_6): δ = 174.30, 160.86, 160.31, 132.16, 131.15, 130.37, 129.18, 113.63, 113.45, 55.23 (2C) ppm.

ESI-quadrupole-Orbitrap MS (*m/z*): [M+H]$^+$ calcd for $C_{15}H_{16}NO_2^+$, 242.1176; found 242.1176.

Bis(4-Methoxyphenyl)methanimine hydrochloride (2i·HCl)

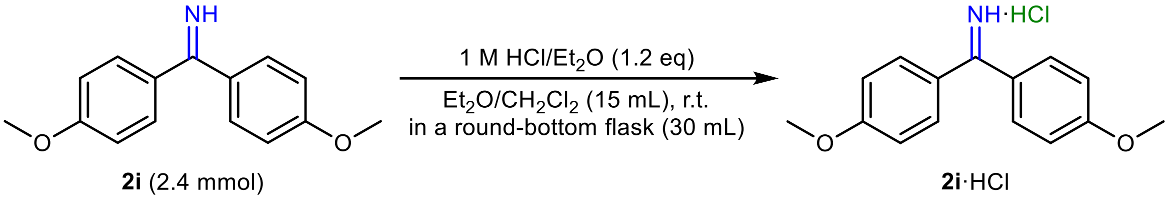

Isolated as a white solid (yield 96%).

^{1}H NMR (400 MHz, DMSO-d_6): δ = 12.27 (br, 1H), 12.21 (br, 1H) 7.78 (d, 4H, J_{HH} = 8.8 Hz), 7.21 (dd, 4H, J_{HH} = 8.8, 1.6 Hz), 3.91 (d, 6H, J_{HH} = 1.6 Hz) ppm.

^{13}C NMR (100 MHz, DMSO-d_6): δ = 178.70, 164.89, 134.41, 122.82, 114.64, 56.01 ppm.

ESI-quadrupole-Orbitrap MS (*m/z*): [M−Cl]$^+$ calcd for $C_{15}H_{16}NO_2^+$, 242.1176; found 242.1169.

(4-(Allyloxy)phenyl)(phenyl)methanimine (2j)

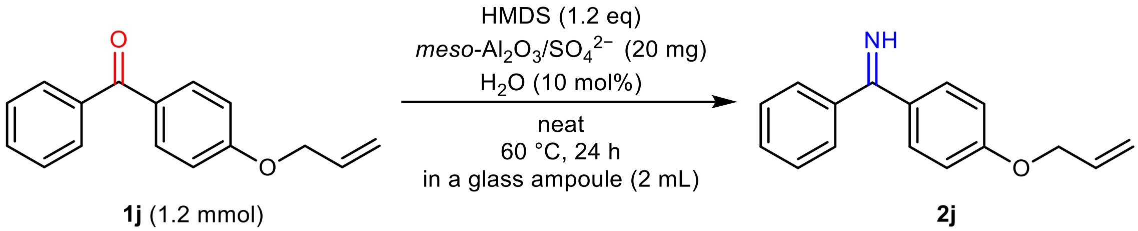

Isolated as a colorless liquid (yield >99%).

^{1}H NMR (400 MHz, DMSO-d_6): δ = 10.39 (br, 0.44H, *Z*), 10.16 (br, 0.56H, *E*), 7.64–7.55 (m, 2H, *E*+*Z*), 7.53–7.33 (m, 5H *E*+*Z*), 7.06–6.97 (m, 2H, *E*+*Z*), 6.12–5.99 (m, 1H, *E*+*Z*), 5.40 (d, 1H, J_{HH} = 17.2 Hz, *E*+*Z*), 5.28 (d, 1H, J_{HH} = 10.5 Hz, *E*+*Z*), 4.62 (s, 2H, *E*+*Z*) ppm. NOE correlations between the *E* and *Z* isomer did not appear with NOESY and ROESY measurements. There was little difference in the calculated Gibbs energy between the isomers ($|\Delta G_E - \Delta G_Z|$ = <0.1 kcal/mol), so the isomer assignment was determined by the tendency of chemical shifts of the imino protons (δ_E = 9.37 ppm, δ_Z = 9.64 ppm) [Gibbs energy and NMR (GIAO) calculations were performed at the level of APFD/6-311+G(2d,p) in DMSO (SMD method)].

^{13}C NMR (100 MHz, DMSO-d_6, *E*+*Z* isomers): δ = 174.84 (2C), 159.97, 159.38, 140.01, 138.91, 133.39 (2C), 131.62, 130.81, 130.26, 130.03, 129.47, 129.25, 128.70, 128.38, 128.06, 127.38, 117.61 (2C), 114.39, 114.18, 68.25, 68.23 ppm.

ESI-quadrupole-Orbitrap MS (*m/z*): [M+H]$^+$ calcd for $C_{16}H_{16}NO^+$, 238.1226; found 238.1221.

Isopropyl 2-(4-((4-chlorophenyl)(imino)methyl)phenoxy)-2-methylpropanoate (2k)

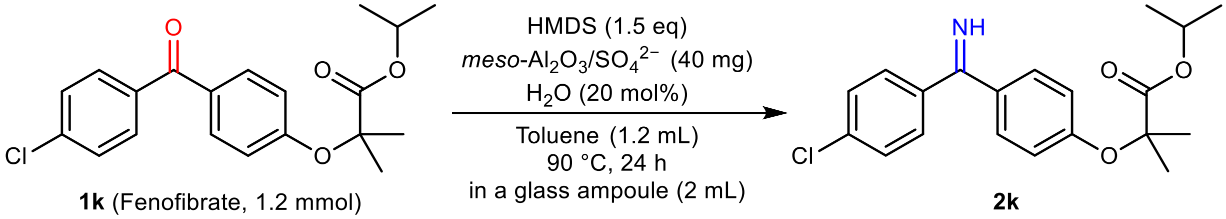

Isolated as a light yellow liquid (yield >99%).

^{1}H NMR (400 MHz, DMSO-d_6): δ = 10.48 (br, 0.5H, *E*), 10.23 (br, 0.5H, *Z*), 7.57 (d, 1H, J_{HH} = 8.5 Hz, *Z*), 7.51 (d, 1H, J_{HH} = 8.8 Hz, *E*), 7.49–7.42 (m, 2H, *E*+*Z*) 7.36 (d, 1H, J_{HH} = 8.5 Hz, *Z*), 7.32 (d, 1H, J_{HH} = 8.8 Hz, *E*), 6.83–6.74 (m, 2H, *E*+*Z*), 5.00–4.86 (m, 1H, *E*+*Z*), 1.52 (s, 6 H, *E*+*Z*), 1.16–1.07 (m, 6H, *E*+*Z*) ppm. Isomer assignment was determined by NOESY and COSY measurements.

^{13}C NMR (100 MHz, DMSO-d_6, *E*+*Z* isomers): δ = 173.61, 173.51, 172.33, 172.32, 157.16, 156.56, 138.48, 137.58, 134.92, 134.20, 131.88, 131.19, 130.44, 129.90, 129.32, 128.96, 128.40, 128.18, 117.60, 117.32, 78.79, 78.73, 68.70, 68.68, 24.98 (2C), 21.19 (2C) ppm.

ESI-quadrupole-Orbitrap MS (*m/z*): [M+H]$^+$ calcd for $C_{20}H_{23}ClNO_3^+$, 360.1361; found, 360.1360.

4-(Imino(phenyl)methyl)benzoic acid (2l)

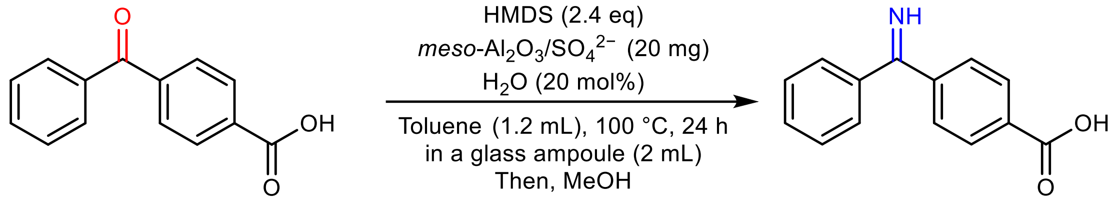

1l (1.2 mmol) **2l**

Isolated as a white solid (yield >99%).

^{1}H NMR (400 MHz, DMSO-d_6): δ = 11.91 (br, 2H), 8.01 (d, 2H, J_{HH} = 8.2 Hz), 7.62 (d, 2H, J_{HH} = 8.2 Hz), 7.56–7.42 (m, 5H) ppm.

^{13}C NMR (100 MHz, DMSO-d_6): δ = 174.94, 166.99, 142.69, 138.46, 132.34, 130.25, 128.43, 128.30, 128.04 ppm.

ESI-quadrupole-Orbitrap MS (*m/z*): [M+H]$^+$ calcd for $C_{14}H_{12}NO_2^+$, 226.0863; found 226.0859.

Methyl 4-(imino(phenyl)methyl)benzoate (2m)

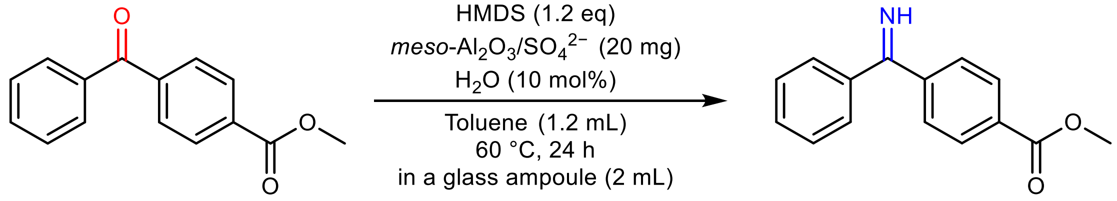

1m (1.2 mmol) **2m**

Isolated as a white solid (yield >99%).

^{1}H NMR (400 MHz, DMSO-d_6): δ = 10.89 (br, 0.55H, *E*), 10.75 (br, 0.45H, *Z*), 8.05–7.99 (m, 2H, *E*+*Z*), 7.75 (d, 1.1H, J_{HH} = 8.3 Hz, *E*), 7.62 (d, 0.9H, 8.3 Hz, *Z*), 7.56–7.39 (m, 5H, , *E*+*Z*), 3.88 (s, 1.35H, *Z*), 3.87 (s, 1.65H, *E*) ppm. NOE correlations between *E* and *Z* isomer did not appear with NOESY and ROESY measurements. There was little difference in the Gibbs energy between the isomers ($|\Delta G_E - \Delta G_Z|$ = <0.1 kcal/mol), so the isomer assignment was determined by the tendency of chemical shifts of the imino protons (δ_E = 10.09 ppm, δ_Z = 9.92 ppm) [Gibbs energy and NMR (GIAO) calculations were performed at the level of APFD/6-311+G(2d,p) in DMSO (SMD method)].

^{13}C NMR (100 MHz, DMSO-d_6, *E*+*Z* isomers): δ = 174.74, 174.61, 165.83, 165.71, 143.63, 142.75, 138.70, 137.87, 130.91, 130.55, 130.36, 129.94, 129.24, 129.03, 128.91, 128.51, 128.48, 128.26, 127.88, 127.49, 52.77, 52.22 ppm.

ESI-quadrupole-Orbitrap MS (*m/z*): [M+H]$^+$ calcd for $C_{15}H_{14}NO_2^+$, 240.1019; found 240.1015.

tert-Butyl 4-(imino(phenyl)methyl)benzoate (2n)

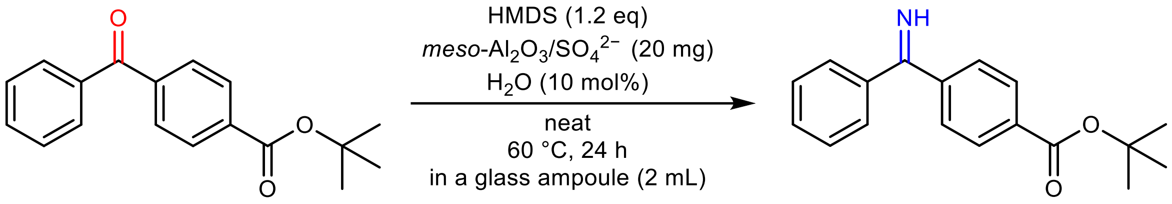

1n (1.2 mmol) **2n**

Isolated as a white solid (yield >99%).

^{1}H NMR (400 MHz, DMSO-d_6): δ = 10.84 (s, 0.53H, *E*), 10.68 (s, 0.47H, *E*), 7.96 (m, 2H, *E*+*Z*), 7.71 (d, 1.06H, J_{HH} = 8.4 Hz, *E*), 7.62, (d, 0.94H, J_{HH} = 8.4 Hz, *Z*), 7.53-7.38 (m, 5H, *E*+*Z*), 1.55 (s, 9H, *E*+*Z*) ppm. NOE correlations between *E* and *Z* isomer did not appear with NOESY and ROESY measurements. The isomer assignment was determined by the calculating tendency of $\Delta G_E - \Delta G_Z = -0.30$ kcal/mol and chemical shifts of the imino protons (δ_E = 10.05 ppm, δ_Z = 9.90 ppm) [Gibbs energy and NMR (GIAO) calculations were performed at the level of APFD/6-311+G(2d,p) in DMSO (SMD method)].

^{13}C NMR (100 MHz, DMSO-d_6, *E*+*Z* isomers): δ = 174.80, 174.77, 164.50, 164.36, 143.32, 142.41, 138.75, 137.87, 132.63, 132.09, 130.52, 129.93, 129.07, 128.83, 128.77, 128.51, 128.46, 128.21, 128.67, 127.49, 80.94, 80.89, 27.67 (2C) ppm.

ESI-quadrupole-Orbitrap MS (*m/z*): [M+H]$^+$ calcd for $C_{18}H_{20}NO_2^+$, 282.1489; found 282.1488.

***N*-Butyl-4-(imino(phenyl)methyl)benzamide (2o)**

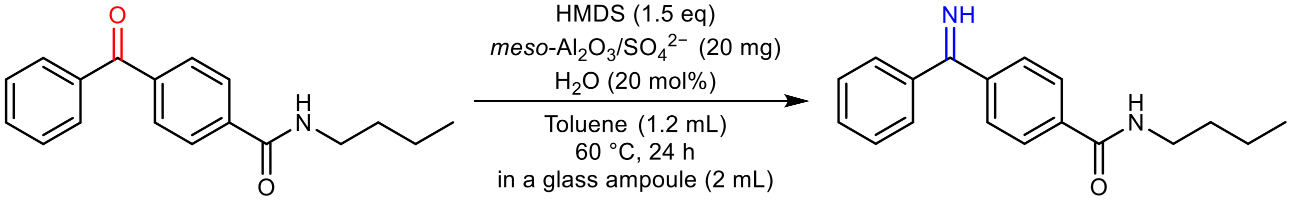

1o (1.2 mmol) **2o**

Isolated as a colorless liquid (yield >99%).

^{1}H NMR (400 MHz, $CDCl_3$): δ = 9.78 (br, 1H), 7.80 (d, 2H, J_{HH} = 8.2 Hz), 7.63 (d, 2H, J_{HH} = 8.2 Hz), 7.55-7.39 (m, 5H), 6.21 (br, 1H), 3.48 (q, 2H, J_{HH} = 7.3 Hz), 1.62 (quin, 2H, J_{HH} = 7.3 Hz), 1.43 (sext, 2H, J_{HH} = 7.3 Hz), 0.97 (t, 3H, J_{HH} = 7.3 Hz) ppm.

^{13}C NMR (100 MHz, $CDCl_3$): δ = 177.51, 166.83, 141.75, 138.84, 136.40, 130.50, 128.63, 128.43, 128.13, 126.88, 39.85, 31.65, 20.11, 13.72 ppm.

ESI-quadrupole-Orbitrap MS (*m/z*): [M+H]$^+$ calcd for $C_{18}H_{21}N_2O^+$, 281.1648; found 281.1646.

4-(Imino(phenyl)methyl)-*N*,*N*-dimethylaniline (2p)

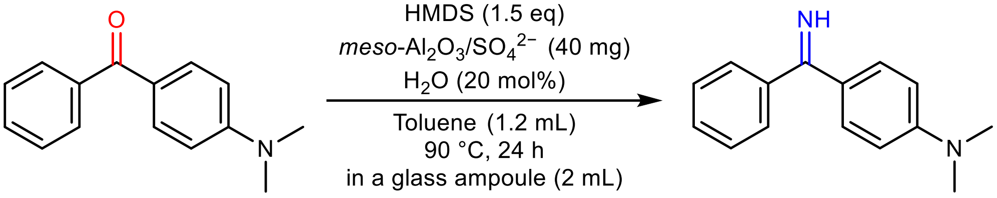

1p (1.2 mmol) **2p**

Isolated as a yellow solid (yield >99%).

^{1}H NMR (400 MHz, $CDCl_3$): δ = 9.27 (br, 1H), 7.52 (br, 4H), 7.46–7.30 (m, 3H), 6.66 (d, 2H, J_{HH} = 8.6 Hz), 2.99 (s, 6H) ppm.

^{13}C NMR (100 MHz, $CDCl_3$): δ = 177.91, 151.97, 140.90, 130.15, 129.71, 128.47, 128.29, 126.37, 111.28, 40.27 ppm.

ESI-quadrupole-Orbitrap MS (*m/z*): $[M+H]^+$ calcd for $C_{15}H_{17}N_2^+$, 225.1386; found 225.1384.

(4-Nitrophenyl)(phenyl)methanimine (2q)

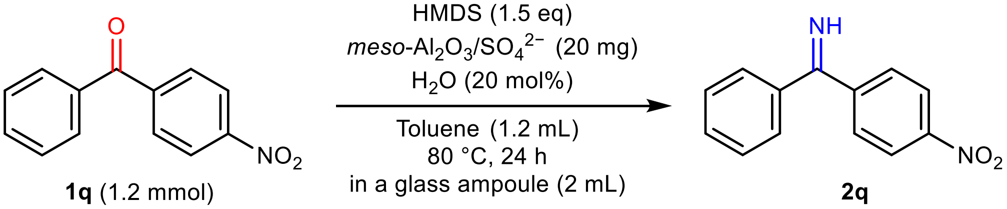

1q (1.2 mmol) **2q**

Isolated as a white solid (yield >99%).

^{1}H NMR (400 MHz, DMSO-d_6): δ = 11.1 (br, 0.6H, *E*), 10.9 (br, 0.4H, *Z*), 8.31–8.25 (m, 2H, *E*+*Z*), 7.86 (d, 1.2H, J_{HH} = 8.8 Hz, *E*), 7.75–7.24 (m, 5.8H, *E*+*Z*) ppm. Isomer assignment was determined by ROESY and COSY measurements.

^{13}C NMR (100 MHz, DMSO-d_6, *E*+*Z* isomers): δ = 173.97, 173.71, 148.39, 147.83, 145.34, 144.52, 138.13, 137.49, 130.77, 130.18, 129.92, 128.95, 128.59, 128.44, 128.59, 128.44, 128.36, 127.53, 123.58, 123.38 ppm.

ESI-quadrupole-Orbitrap MS (*m/z*): $[M+H]^+$ calcd for $C_{13}H_{11}N_2O_2^+$, 227.0815; found 227.0813.

(4-Nitrophenyl)(phenyl)methanimine hydrochloride (2q·HCl)

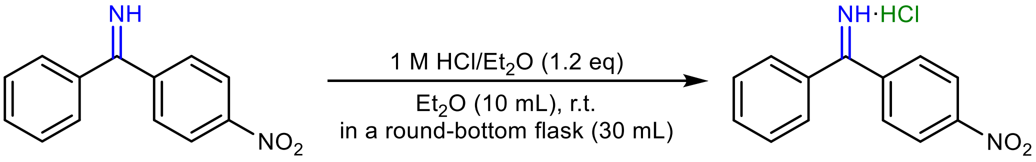

2q (2.4 mmol) **2q**·HCl

Isolated as a white solid (yield 95%).

^{1}H NMR (400 MHz, DMSO-d_6): δ = 13.33 (br, 2H), 8.46 (d, 2H, J_{HH} = 8.8 Hz), 8.02 (d, 2H, J_{HH} = 8.8 Hz), 7.88 (t, 1H, J_{HH} = 7.4 Hz), 7.84 (d, 2H, J_{HH} = 7.4 Hz), 7.69 (t, 2H, J_{HH} = 7.4 Hz) ppm.

^{13}C NMR (100 MHz, DMSO-d_6): δ = 180.16, 150.51, 136.93, 135.69, 132.81, 131.86. 130.57, 129.19, 123.63 ppm.

ESI-quadrupole-Orbitrap MS (*m/z*): [M−Cl]$^+$ calcd for $C_{13}H_{11}N_2O_2^+$, 227.0815; found 227.0811.

2-(imino(phenyl)methyl)phenol hydrochloride salt (**2r**·HCl)

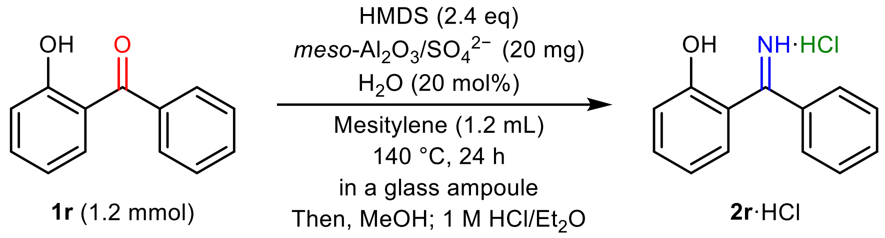

2s was obtained in 85% NMR yield and **2s**·HCl was isolated as a white solid (yield 82%).

^{1}H NMR (400 MHz, DMSO-d_6): δ = 12.4 (br, 3H), 7.83–7.73 (m, 3H), 7.68-7.54 (m, 3H), 7.31 (t, 2H, J_{HH} = 9.4 Hz), 6.98 (t, 1H, J_{HH} = 7.5 Hz) ppm.

^{13}C NMR (100 MHz, DMSO-d_6): δ = 180.03, 158.88, 136.09, 134.60, 132.91, 131.63, 130.65, 129.02, 119.07, 117.36, 117.20 ppm.

ESI-quadrupole-Orbitrap MS (*m/z*): [M−Cl]$^+$ calcd for $C_{13}H_{12}NO^+$, 198.0913; found 198.0913.

3-(Imino(phenyl)methyl)phenol (**2s**)

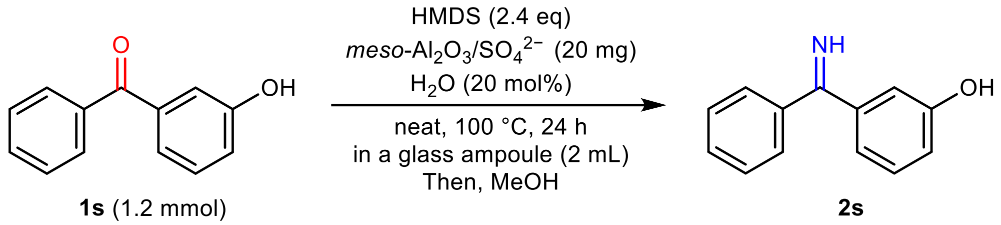

Isolated as a white solid (yield >99%).

^{1}H NMR (400 MHz, DMSO-d_6): δ = 10.38 (br, 0.46H, *E*), 10.35 (br, 0.54H, *Z*), 7.72–6.60 (m, 9H, *E*+*Z*) ppm. NOE correlations between *E* and *Z* isomer did not appear with NOESY and ROESY measurements. There was little difference of calculating results in Gibbs energy between the isomers ($|\Delta G_E - \Delta G_Z|$ = <0.1 kcal/mol), so the isomer assignment was determined by the tendency of chemical shifts of the imino protons (δ_E = 9.78 ppm, δ_Z = 9.74 ppm) [Each of ΔG_E, δ_E and ΔG_Z, δ_Z incorporates the contributions of the two most stable rotational isomers, and Gibbs energy and

NMR (GIAO) calculations were performed at the level of APFD/6-311+G(2d,p) in DMSO (SMD method)].

^{13}C NMR (100 MHz, DMSO-d_6, *E*+*Z* isomers): δ = 175.69 (2C), 157.49, 157.25, 140.89, 139.77, 139.73, 138.50, 130.25, 129.59 (2C), 129.14, 128.66, 128.36, 128.12, 127.47, 119.56, 117.96, 117.43, 116.79, 115.43, 114.39 ppm.

ESI-quadrupole-Orbitrap MS (*m/z*): $[M+H]^+$ calcd for $C_{13}H_{12}NO^+$, 198.0913; found 198.0911.

(3-((*tert*-Butyldimethylsilyl)oxy)phenyl)(phenyl)methanimine (2t)

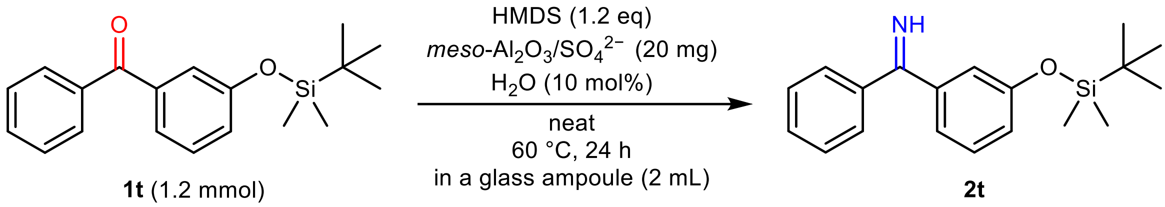

1t (1.2 mmol) **2t**

Isolated as a colorless liquid (yield >99%).

^{1}H NMR (400 MHz, DMSO-d_6): δ = 10.53 (br, 0.5H, *E*), 10.53 (br, 0.5H, *Z*), 7.64–7.58 (m, 1H, *E*+*Z*), 7.54–7.23 (m, 5H, *E*+*Z*), 7.20 (d, 0.5H, J_{HH} = 7.7 Hz, *E*), 7.09 (t, 0.5H, J_{HH} = 2.0 Hz, *E*), 7.05–6.94 (m, 1.5H, *E*+*Z*), 6.81 (t, 0.5H, J_{HH} = 2.0 Hz, *Z*), 0.93 (s, 9H, *E*+*Z*), 0.18 and 0.16 (s, 6H, *E*+*Z*) ppm. NOE correlations between *E* and *Z* isomer did not appear with NOESY and ROESY measurements. There was little difference of calculating results in Gibbs energy and chemical shifts between the isomers ($|\Delta G_E - \Delta G_Z|$ = <0.1 kcal/mol, δ_E and δ_Z = 9.75 ppm) [Each of ΔG_E, δ_E and ΔG_Z, δ_Z incorporates the contributions of the two most stable rotational isomers, and Gibbs energy and NMR (GIAO) calculations were performed at the level of APFD/6-311+G(2d,p) in DMSO (SMD method)]. Therefore, the assignment referred to a similar structure of **2s**.

^{13}C NMR (100 MHz, DMSO-d_6, *E*+*Z* isomers): δ = 175.09, 174.95, 154.91, 154.83, 140.95, 139.39, 138.35, 130.32, 129.80, 129.69, 129.39, 128.57, 128.36, 128.11, 127.44, 122.00, 121.82, 121.16, 120.72, 119.77, 118.91, 25.48 (2C), 17.89, 17.88, −4.60, −4.62 ppm.

ESI-quadrupole-Orbitrap MS (*m/z*): $[M+H]^+$ calcd for $C_{19}H_{26}NOSi^+$, 312.1778; found 312.1772.

Bis(3-(trifluoromethyl)phenyl)methanimine (2u)

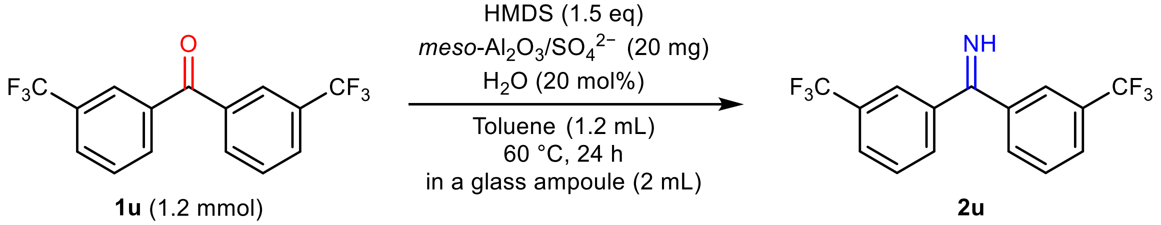

1u (1.2 mmol) **2u**

Isolated as a colorless liquid (yield >99%).

^1H NMR (400 MHz, DMSO-d_6): δ = 11.23 (br, 2H), 8.00 (br, 1H), 7.93-7.86 (m, 2H), 7.84–7.79 (m, 2H), 7.75–7.67 (m, 3H) ppm.

^{13}C NMR (100 MHz, DMSO-d_6): δ = 172.59, 139.25, 138.97, 132.62 (q, J_{CF} = 1 Hz), 131.59 (q, J_{CF} = 10 Hz), 129.66, 129.58, 129.45 (q, J_{CF} = 32 Hz), 129.29 (q, J_{CF} = 32 Hz), 127.05 (q, J_{CF} = 4 Hz), 126.55 (q, J_{CF} = 4 Hz), 124.58 (q, J_{CF} = 4 Hz), 124.30 (q, J_{CF} = 4 Hz), 123.96 (q, J_{CF} = 272 Hz), 123.89 (q, J_{CF} = 272 Hz) ppm.

^{19}F NMR (376 MHz, DMSO-d_6): δ = −64.5 (d, 3F, J_{CF} = 4.4 Hz), −64.6 (d, 3F, J_{CF} = 4.4 Hz) ppm

ESI-quadrupole-Orbitrap MS (*m/z*): $[M+H]^+$ calcd for $C_{15}H_{10}F_6N^+$, 318.0712; found 318.0706.

9*H*-Fluoren-9-imine (2v)

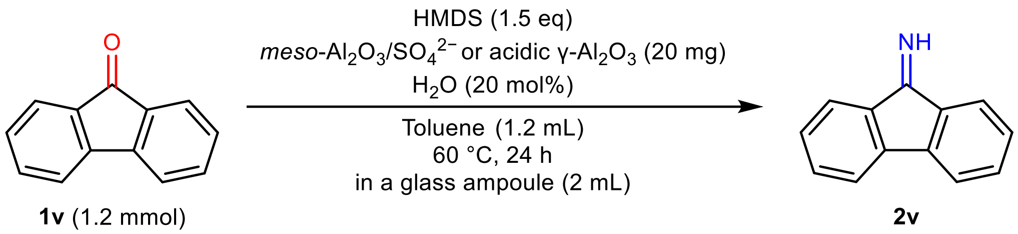

Isolated as a light yellow solid (yield >99%).

^1H NMR (400 MHz, $CDCl_3$): δ = 10.27 (br, 1H), 7.95 (d, 1H, J_{HH} = 7.5 Hz), 7.63-7.22 (m, 7H) ppm.
^{13}C NMR (100 MHz, $CDCl_3$): δ = 173.08, 142.56, 141.76, 137.08, 133.00, 132.16, 132.09,128.36, 127.83, 123.09, 121.27, 120.13, 119.91 ppm.

ESI-quadrupole-Orbitrap MS (*m/z*): $[M+H]^+$ calcd for $C_{13}H_{10}N^+$, 180.0808; found 180.0807.

10,10-Dimethylanthracen-9(10*H*)-imine (2w)

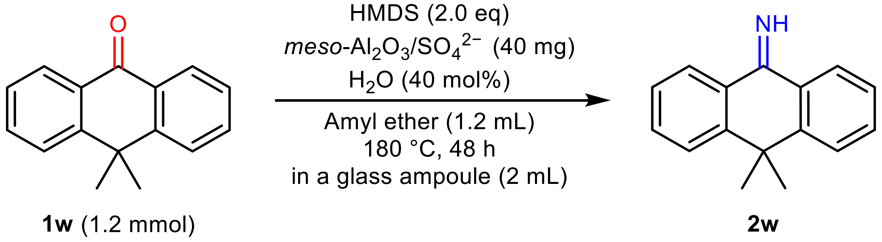

Isolated as a light brown solid (yield >99%).

^1H NMR (400 MHz, DMSO-d_6): δ = 10.86 (br, 1H), 8.36 (dd, 1H, J_{HH} = 8.0, 1.3 Hz), 8.11 (dd, 1H, J_{HH} = 8.0, 1.3 Hz), 7.80 (d, 1H, J_{HH} = 8.0), 7.75 (d, 1H, J_{HH} = 8.0), 7.59−7.51 (m, 2H), 7.46−7.33 (m, 2H), 1.63 (s, 6.ppH) ppm.

^{13}C NMR (100 MHz, DMSO-d_6): δ = 163.45, 146.50, 145.93, 131.45, 130.80, 130.79, 129.12, 126.67, 126.58, 126.24, 125.90, 125.40, 124.48, 37.78, 32.48 ppm.

ESI-quadrupole-Orbitrap MS (*m/z*): $[M+H]^+$ calcd for $C_{16}H_{16}N^+$, 222.1277; found 222.1276.

10,11-Dihydro-5*H*-dibenzo[*a*,*d*][7]annulen-5-imine (2x)

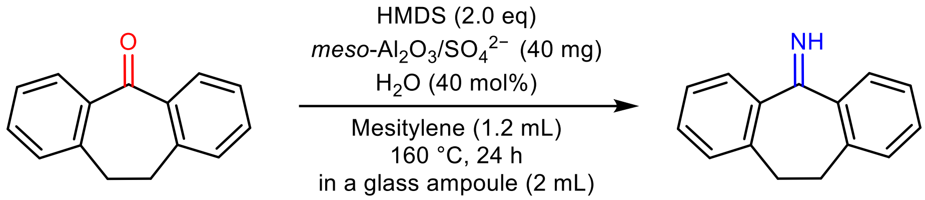

1x (1.2 mmol) **2x**

Isolated as a light yellow liquid (yield >99%).

^{1}H NMR (400 MHz, CDCl$_3$): δ = 10.27 (br, 1H), 7.95 (d, 1H, J_{HH} = 7.5 Hz), 7.63−7.22 (m, 7H) ppm.

^{13}C NMR (100 MHz, CDCl$_3$): δ = 173.08, 142.56, 141.76, 137.08, 133.00, 132.16, 132.09,128.36, 127.83, 123.09, 121.27, 120.13, 119.91 ppm.

ESI-quadrupole-Orbitrap MS (*m/z*): [M+H]$^+$ calcd for $C_{15}H_{14}N^+$, 208.1121; found 208.1119.

5*H*-Dibenzo[*a*,*d*][7]annulen-5-imine (2y)

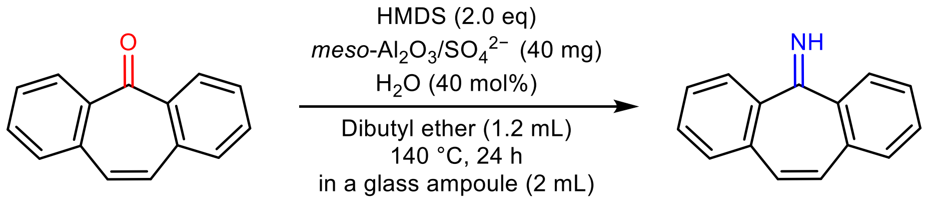

1y (1.2 mmol) **2y**

Isolated as a white solid (yield >99%).

^{1}H NMR (400 MHz, CDCl$_3$): δ = 9.73 (br, 1H), 7.88 (br, 1H), 7.52 (br, 1H), 7.45 (br, 4H), 7.41-7.32 (m, 2H), 6.92 (d, 2H, J_{HH} = 10.9 Hz) ppm.

^{13}C NMR (100 MHz, CDCl$_3$): δ = 178.47, 140.37, 139.02, 133.74, 132.64, 131.45, 130.21, 129.38 (2C), 129.18, 129.00 (3C), 128.04, 126.65 ppm.

ESI-quadrupole-Orbitrap MS (*m/z*): [M+H]$^+$ calcd for $C_{15}H_{12}N^+$, 206.0964; found 206.0967.

9*H*-Xanthen-9-imine (2z)

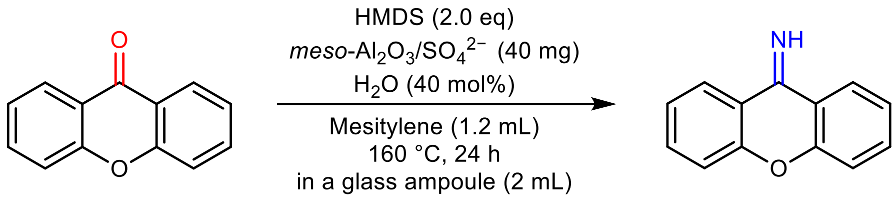

1z (1.2 mmol) **2z**

Isolated as a white solid (yield >99%).

^{1}H NMR (400 MHz, $CDCl_3$): δ = 9.49 (br, 1H), 8.16 (br, 2H), 7.60−7.52 (m, 2H), 7.38−7.23 (m, 4H) ppm.

^{13}C NMR (100 MHz, $CDCl_3$): δ = 158.97, 152.80, 132.48, 124.50, 123.60, 119.71, 117.84 ppm.

ESI-quadrupole-Orbitrap MS (*m/z*): $[M+H]^+$ calcd for $C_{13}H_{10}NO^+$, 196.0757; found 196.0757.

9*H*-Xanthen-9-imine hydrochloride (**2z**·HCl)

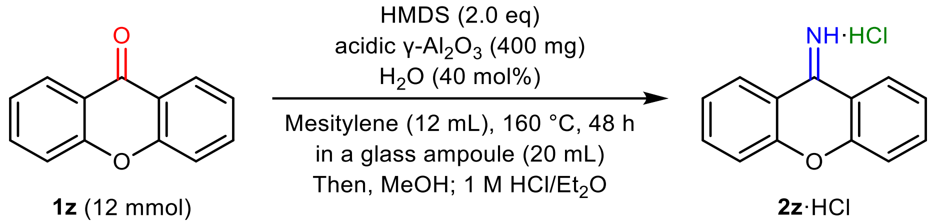

Isolated as a white solid (yield 88%).

^{1}H NMR (400 MHz, DMSO-d_6): δ = 12.02 (br, 2H), 8.95 (d, 2H, J_{HH} = 8.3 Hz), 8.12 (t, 2H, J_{HH} = 8.3 Hz), 7.88 (d, 2H, J_{HH} = 8.3 Hz), 7.70 (t, 2H, J_{HH} = 8.3 Hz) ppm.

^{13}C NMR (100 MHz, DMSO-d_6): δ = 160.53, 154.10, 138.28, 126.39, 125.68, 118.82, 112.98 ppm.

ESI-quadrupole-Orbitrap MS (*m/z*): $[M-Cl]^+$ calcd for $C_{13}H_{10}NO^+$, 196.0757; found 196.0761.

9*H*-Thioxanthen-9-imine (**2aa**)

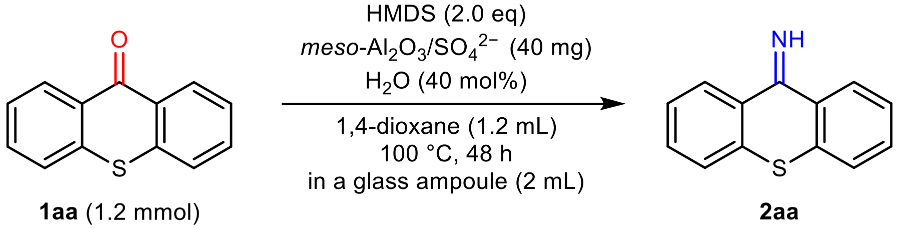

Isolated as a light yellow solid (yield 81%).

^{1}H NMR (400 MHz, DMSO-d_6): δ = 10.89 (br, 1H), 8.45 (d, 1H, J_{HH} = 8.0 Hz), 8.21 (d, 1H, J_{HH} = 8.0 Hz), 7.65−7.37 (m, 6H) ppm.

^{13}C NMR (100 MHz, DMSO-d_6): δ = 162.09, 132.18, 131.65, 130.52, 130.48, 130.42, 128.56, 127.72, 126.95, 126.8, 126.47, 126.39, 125.60 ppm.

ESI-quadrupole-Orbitrap MS (*m/z*): $[M+H]^+$ calcd for $C_{13}H_{10}NS^+$, 212.0528; found 212.0526.

Phenyl(pyridin-2-yl)methanimine (2ab)

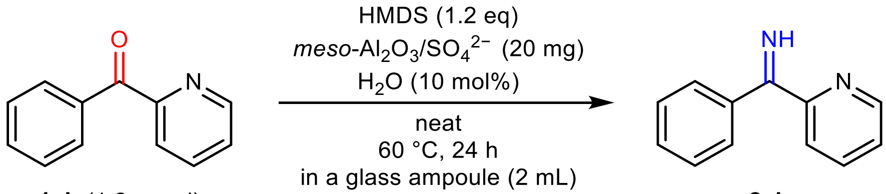

1ab (1.2 mmol) **2ab**

Isolated as a colorless liquid (yield >99%).

^{1}H NMR (400 MHz, DMSO-d_6): δ = 11.09 (br, 0.68H, *Z*), 11.03 (br, 0.32H, *E*), 8.73 (d, 0.68H, J_{HH} = 4.7 Hz, *Z*), 8.57 (d, 0.32H, J_{HH} = 4.7 Hz, *E*), 8.08 (d, 0.32H, J_{HH} = 7.9 Hz, *E*), 7.99−7.88 (m, 1H, *E*+*Z*) 7.68−7.38 (m, 6.68H *E*+*Z*) ppm. NOE correlations between *E* and *Z* isomers did not appear with NOESY and ROESY measurements. The isomer assignment was determined by a tendency of the calculated values of $\Delta G_E - \Delta G_Z$ = 0.57 kcal/mol and chemical shifts of the imino protons (δ_E = 10.25 ppm, δ_Z = 10.88 ppm) [Each of ΔG_E, δ_E and ΔG_Z, δ_Z incorporates the contributions of the two most stable rotational isomers, and Gibbs energy and NMR (GIAO) calculations were performed at the level of APFD/6-311+G(2d,p) in DMSO (SMD method)].

^{13}C NMR (100 MHz, DMSO-d_6, *E*+*Z* isomer): δ = 174.81, 173.72, 156.66, 153.32, 149.60, 148.23, 138.55, 137.48, 137.44, 136.91, 130.14, 129.50, 128.63, 128.19, 127.97, 127.91, 124.97, 124.70, 123.02, 122.89 ppm.

ESI-quadrupole-Orbitrap MS (*m/z*): $[M+H]^+$ calcd for $C_{12}H_{11}N_2^+$, 183.0917; found, 183.0917.

Phenyl(pyridin-3-yl)methanimine (2ac)

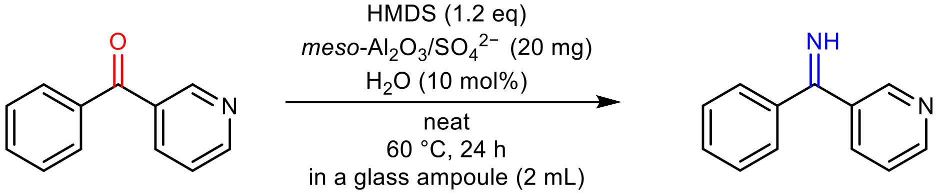

1ac (1.2 mmol) **2ac**

Isolated as a colorless liquid (yield >99%).

^{1}H NMR (400 MHz, $CDCl_3$): δ = 9.93 (br, 0.64H, *E*), 9.89 (br, 0.36H, *Z*), 8.90 (br, 0.64H, *E*), 8.76-8.64 (m, 1.36H, *E*+*Z*), 8.10 (d, 0.64H, J_{HH} = 7.8 Hz, *E*), 7.73 (br, 1.08H, *Z*), 7.58−7.32 (m, 5.28H, *E*+*Z*) ppm. NOE correlations between *E* and *Z* isomers did not appear with NOESY and ROESY measurements. The isomer assignment was determined by a tendency of the caluculated values of $\Delta G_E - \Delta G_Z$ = −0.12 kcal/mol and chemical shifts of the imino protons (δ_E = 9.93 ppm, δ_Z = 9.85 ppm) [Each of ΔG_E, δ_E and ΔG_Z, δ_Z incorporates the contributions of the two most stable rotational isomers, and Gibbs energy and NMR (GIAO) calculations were performed at the level of APFD/6-311+G(2d,p) in $CDCl_3$ (SMD method)].

^{13}C NMR (100 MHz, $CDCl_3$): δ = 176.00, 175.51, 151.37, 150.86, 150.39, 148.40, 139.10, 137.34, 136.28, 135.98, 134.71, 133.84, 131.08, 130.39, 128.94, 128.71, 128.34, 127.31, 123.15, 123.03 ppm.

ESI-quadrupole-Orbitrap MS (*m/z*): $[M+H]^+$ calcd for $C_{12}H_{11}N_2^+$, 183.0917; found, 183.0916.

Phenyl(pyridin-4-yl)methanimine (2ad)

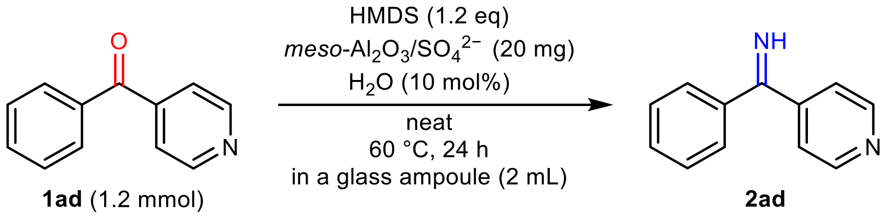

Isolated as a white solid (yield >99%).

^{1}H NMR (400 MHz, $CDCl_3$): δ = 10.12 (br, 0.58H, *E*), 9.87 (br, 0.42H, *Z*), 8.79−8.64 (m, 2H, *E*+*Z*), 7.76−7.68 (m, 0.84H, *Z*), 7.63−7.56 (m, 1.16H, *E*), 7.56−7.35 (m, 4.16H, *E*+*Z*), 7.34−7.27 (m, 0.84H, *Z*) ppm. NOE correlations between *E* and *Z* isomer did not appear with NOESY and ROESY measurements. There was little difference in Gibbs energy between the isomers ($|\Delta G_E - \Delta G_Z| = 0.1$ kcal/mol), so the isomer assignment was determined by a tendency of chemical shifts of the imino protons (δ_E = 10.21 ppm, δ_Z = 9.91 ppm). [Gibbs energy and NMR (GIAO) calculations were performed at the level of APFD/6-311+G(2d,p) in $CDCl_3$ (SMD method)].

^{13}C NMR (100 MHz, $CDCl_3$, *E*+*Z* isomers): δ = 176.46, 176.06, 150.33, 150.08, 147.41, 145.39, 138.53, 136.66, 131.25, 130.52, 128.87, 128.74, 128.38, 127.31, 123.06, 121.57 ppm.

ESI-quadrupole-Orbitrap MS (*m/z*): $[M+H]^+$ calcd for $C_{12}H_{11}N_2^+$, 183.0917; found, 183.0916.

Phenyl(1*H*-pyrrol-2-yl)methanimine (2ae)

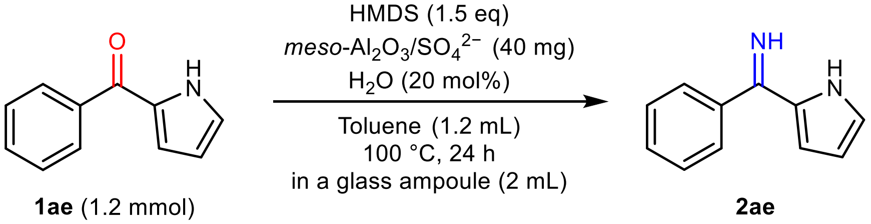

Isolated as a light brown solid (yield >88%).

^{1}H NMR (400 MHz, $CDCl_3$): δ = 9.82 (br, 1H), 8.82 (br, 1H), 7.57 (m, 2H), 7.53−7.40 (m, 3H), 7.03 (m, 1H), 6.27 (t, 1H, J_{HH} = 3.0 Hz) ppm.

^{13}C NMR (100 MHz, $CDCl_3$): δ = 169.68, 140.13, 130.39, 129.86, 128.40, 127.32, 122.87, 116.37, 109.74 ppm.

ESI-quadrupole-Orbitrap MS (*m/z*): $[M+H]^+$ calcd for $C_{11}H_{11}N_2^+$, 171.0917; found 171.0915.

Furan-2-yl(phenyl)methanimine (2af)

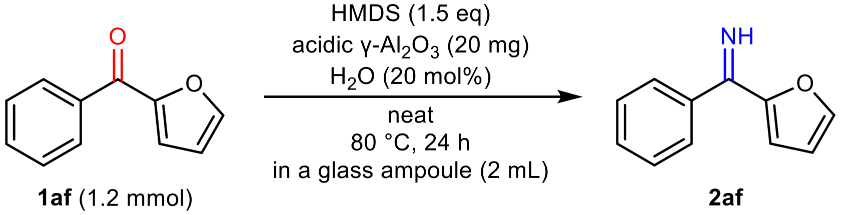

Isolated as a reddish brown liquid (yield >99%).

^{1}H NMR (400 MHz, DMSO-d_6): δ = 10.41 (br, 0.69H, *Z*), 10.30 (br, 0.31H, *E*), 7.95 (d, 0.69H, J_{HH} = 1.7 Hz, *Z*), 7.89 (d, 0.31H, J_{HH} = 1.7 Hz, *E*), 7.73 (m, 1.38H, *Z*), 7.58−7.44 (m, 3.62H, *E*+*Z*), 6.81 (d, 0.69H, J_{HH} = 3.4 Hz, *Z*), 6.81 (d, 0.69H, J_{HH} = 3.4 Hz, *Z*), 6.78 (d, 0.31H, J_{HH} = 3.4 Hz, *E*), 6.68 (dd, 0.69H, J_{HH} = 3.4, 1.7 Hz, *Z*), 6.63 (dd, 0.31H, J_{HH} = 3.4, 1.7 Hz, *E*) ppm. NOE correlations between *E* and *Z* isomers did not appear with NOESY and ROESY measurements. The isomer assignment was determined by a tendency of the caluculated values of $\Delta G_E - \Delta G_Z$ = 0.47 kcal/mol and chemical shifts of the imino protons (δ_E = 9.26 ppm, δ_Z = 9.77 ppm) [Each of ΔG_E, δ_E and ΔG_Z, δ_Z incorporates the contributions of the two most stable rotational isomers, and Gibbs energy and NMR (GIAO) calculations were performed at the level of APFD/6-311+G(2d,p) in DMSO (SMD method)].

^{13}C NMR (100 MHz, DMSO-d_6, *E*+*Z* isomers): δ = 165.45, 163.83, 152.21, 149.49, 145.99, 145.59, 138.61, 137.41, 130.32, 129.49, 128.42, 128.26, 128.22, 127.22, 115.32, 113.91, 112.33, 111.89 ppm.

ESI-quadrupole-Orbitrap MS (*m/z*): $[M+H]^+$ calcd for $C_{11}H_{10}NO^+$, 172.0757; found 172.0755.

Phenyl(thiophen-2-yl)methanimine (2ag)

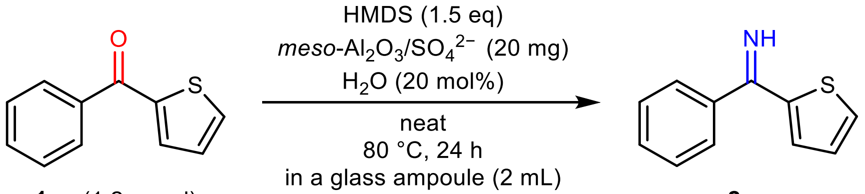

1ag (1.2 mmol) **2ag**

Isolated as a light yellow liquid (yield >99%).

^{1}H NMR (400 MHz, DMSO-d_6): δ = 10.51 (br, 0.16H, *Z*), 10.20 (br, 0.84H, *E*), 7.79 (d, 0.16H, J_{HH} = 4.6 Hz, *Z*), 7.74 (d, 0.84H, J_{HH} = 4.6 Hz, *E*), 7.69 (d, 0.32H, J_{HH} = 7.5 Hz, *Z*), 7.57−7.43 (m, 4.84H, *E*+*Z*), 7.22−7.08 (m, 1.84H, *E*+*Z*) ppm. NOE correlations between *E* and *Z* isomers did not appear with NOESY and ROESY measurements. The isomer assignment was determined by a tendency of the calculated values of $\Delta G_E - \Delta G_Z = -1.23$ kcal/mol and chemical shifts of the imino protons (δ_E = 8.92 ppm, δ_Z = 9.43 ppm) [Each of ΔG_E, δ_E and ΔG_Z, δ_Z incorporates the contributions of the two most stable rotational isomers, and Gibbs energy and NMR (GIAO) calculations were performed at the level of APFD/6-311+G(2d,p) in DMSO (SMD method)].

^{13}C NMR (100 MHz, DMSO-d_6, *E*+*Z* isomers): δ = 169.65, 167.72, 144.22, 141.61, 139.11, 139.08, 131.39, 130.61, 130.30, 129.86, 129.83, 129.58, 128.50, 128.40, 128.14, 128.02, 127.82, 127.15 ppm.

ESI-quadrupole-Orbitrap MS (*m/z*): $[M+H]^+$ calcd for $C_{11}H_{10}NS^+$, 188.0528; found 188.0528.

Phenyl(thiophen-2-yl)methanimine hydrochloride (**2ag**·HCl)

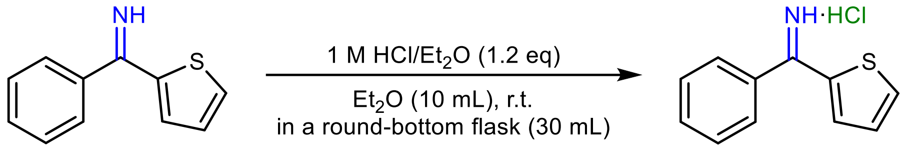

2ag (2.4 mmol) **2ag**·HCl

Isolated as a white solid (yield 94%).

^{1}H NMR (400 MHz, DMSO-d_6): δ = 12.64 (br, 2H), 8.64−8.40 (m, 2H), 8.92−7.77 (m, 3H), 7.74−7.60 (m, 2H), 7.58−7.48 (m, 1H)

^{13}C NMR (100 MHz, DMSO-d_6): δ = 172.35, 142.52, 140.06, 134.27, 133.35, 131.73, 130.51, 130.40, 128.91 ppm.

ESI-quadrupole-Orbitrap MS (*m/z*): [M−Cl]$^+$ calcd for $C_{11}H_{10}NS^+$, 188.0528; found 188.0525.

3-Iminoindolin-2-one (**2ah**)

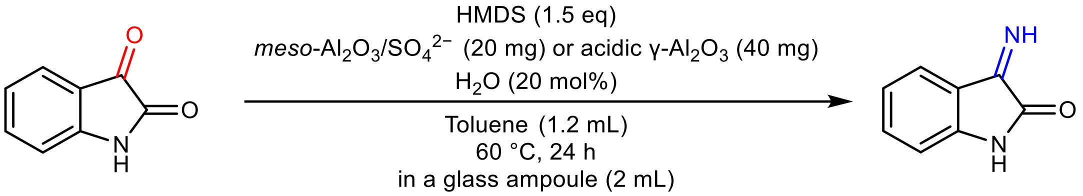

1ah (Isatin, 1.2 mmol) **2ah**

Isolated as a brick red solid (yield >99%).

^{1}H NMR (400 MHz, DMSO-d_6): δ = 12.32 (br, 0.2H, *E*), 11.24 (br, 0.8H, *Z*) 10.92 (br, 0.8H, *Z*), 10.80 (br, 0.2H, *E*), 7.71 (d, 0.2H, J_{HH} = 7.6 Hz, *E*), 7.63 (d, 0.2H, J_{HH} = 7.6 Hz, *Z*), 7.45 (t, 0.8H, J_{HH} = 7.6 Hz, *Z*), 7.39 (t, 0.2H, J_{HH} = 7.6 Hz, *E*), 7.06 (t, 0.8H, J_{HH} = 7.6 Hz, *Z*), 7.02 (t, 0.2H, J_{HH} = 7.6 Hz, *E*), 7.63 (d, 0.2H, J_{HH} = 7.6 Hz, *Z*), 6.91 (d, 0.8H, J_{HH} = 7.6 Hz, *Z*), 6.85 (d, 0.2H, J_{HH} = 7.6 Hz, *E*) ppm. NOE correlations between *E* and *Z* isomers did not appear with NOESY and ROESY measurements. The isomer assignment was determined by a tendency of the calculated values of $\Delta G_E - \Delta G_Z$ = 1.16 kcal/mol and chemical shifts of the imino protons (δ_E = 11.23 ppm, δ_Z = 10.84 ppm) [Gibbs energy and NMR (GIAO) calculations were performed at the level of APFD/6-311+G(2d,p) in DMSO (SMD method)].

^{13}C NMR (100 MHz, DMSO-d_6, *E*+*Z* isomers): δ = 164.97, 163.79, 163.52, 159.32, 146.42, 144.88, 134.33, 133.86, 123.52, 123.08, 122.53, 121.95, 120.40, 117.92, 111.28, 110.85 ppm.

ESI-quadrupole-Orbitrap MS (*m/z*): [M+H]$^+$ calcd for $C_8H_7N_2O^+$, 147.0553; found 147.0554.

4-(Imino(phenyl)methyl)ferrocene (2ai)

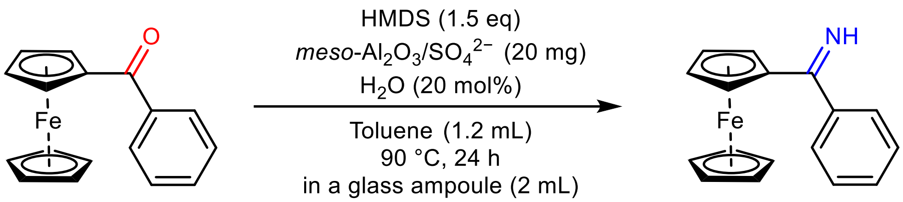

1ai (1.2 mmol) **2ai**

Isolated as a reddish brown liquid (yield >99%).

^{1}H NMR (400 MHz, $CDCl_3$): δ = 9.38 (br, 1H), 7.64 (br, 2H), 7.48−7.35 (m, 3H), 4.61 (br, 2H), 4.40 (t, 2H, J_{HH} = 1.8 Hz), 4.17 (br, 5H) ppm.

^{13}C NMR (100 MHz, $CDCl_3$): δ = 179.05, 140.69, 129.43, 128.09, 127.12, 80.82, 70.51, 69.75, 69.45 ppm.

ESI-quadrupole-Orbitrap MS (*m/z*): $[M+H]^+$ calcd for $C_{17}H_{16}FeN^+$, 290.0627; found 290.0621.

Phenanthrene-9,10-diimine (2aj)

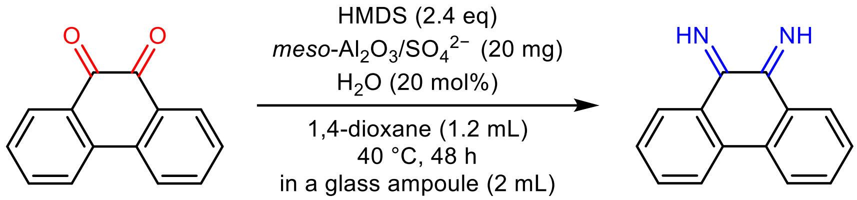

1aj (1.2 mmol) **2aj**

Isolated as a light yellow solid (yield 98%).

^{1}H NMR (400 MHz, $CDCl_3$): δ = 11.65 (br, 0.89H, *EZ*), 10.76 (br, 0.89H, *EZ*), 10.52 (br, 0.11H, *ZZ*), 8.51 (dd, 0.89H, J_{HH} = 7.9, 1.4 Hz, *EZ*), 8.35 (dd, 0.22H, J_{HH} = 7.9, 1.2 Hz, *ZZ*), 8.08−7.93 (m, 2H, *EZ*+*ZZ*), 7.74 (dd, 0.89H, J_{HH} = 1.4 Hz, *EZ*), 7.62-7.51 (m, 2H, *EZ*+*ZZ*), 7.48-7.35 (m, 2H, *EZ*+*ZZ*) ppm. NOE correlations between isomers did not appear with NOESY and ROESY measurements, so the isomers were assigned by quantum chemical calculations. Calculating the Gibbs energy with respect to the *EZ* isomers, we obtained the values of $\Delta G_{EZ} - \Delta G_{ZZ} = -0.83$ kcal/mol, $\Delta G_{EZ} - \Delta G_{EE} = -4.14$ kcal/mol, indicating that *EZ* is the most stable isomer and *EE* is the most unstable isomer. In addition, calculation results for chemical shifts of the imino protons were δ_{EE} = 10.77 ppm, δ_{EZ} = 11.48 and 10.89 ppm, and δ_{ZZ} = 10.52 ppm [Gibbs energy and NMR (GIAO) calculation: APFD/6-311+G(2d,p) in $CHCl_3$ (SMD method)]. The instability of the *EE* isomer is thought to be due to electron repulsions between the lone pairs on the nitrogen atoms.

^{13}C NMR (100 MHz, $CDCl_3$, *EZ*+*ZZ* isomers): δ = 166.06, 165.51, 163.01, 133.80, 133.37, 132.84, 132.62, 132.49, 132.24, 131.21, 131.08, 129.03, 128.64, 128.47, 128.30, 127.28, 127.03, 125,41, 124.26, 123.41, 123.28 ppm.

ESI-quadrupole-Orbitrap MS (*m/z*): $[M+H]^+$ calcd for $C_{14}H_{11}N_2^+$, 207.0917; found 219.0915.

1,4-Phenylenebis(phenylmethanimine) (2ak)

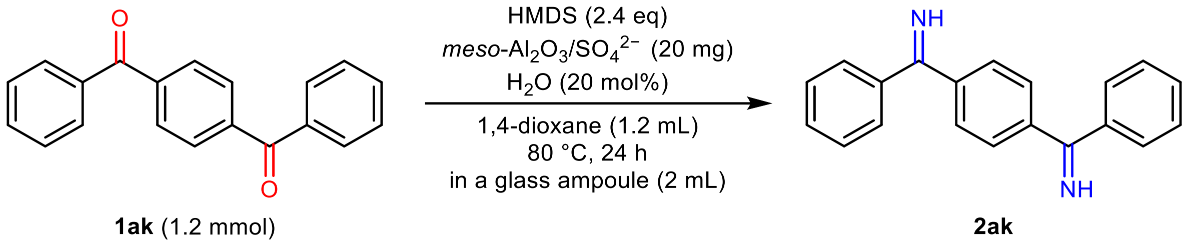

Isolated as a white solid (yield 98%).

^{1}H NMR (400 MHz, $CDCl_3$): δ = 9.87 (br, 2H), 7.78 (br, 4H), 7.60−7.28 (m, 10H) ppm.

^{13}C NMR (100 MHz, $CDCl_3$): δ = 177.55, 141.03, 139.02, 130.37, 128.37 (2C), 128.16 ppm.

ESI-quadrupole-Orbitrap MS (*m/z*): $[M+H]^+$ calcd for $C_{20}H_{17}N_2^+$, 285.1386; found 285.1382.

1-Phenylpropan-1-imine hydrochloride (**2al**·HCl)

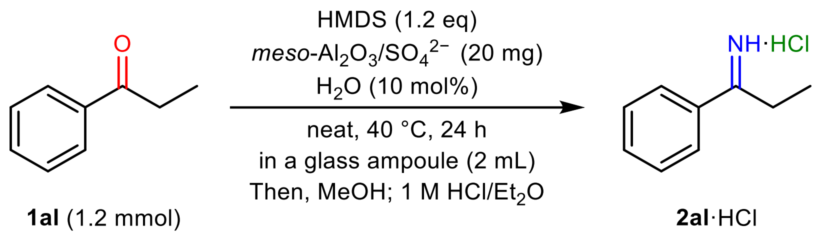

2al was obtained in 96% NMR yield and **2al**·HCl was isolated as a white solid (yield 83%).

^{1}H NMR (400 MHz, DMSO-d_6): δ = 12.90 (br, 1H), 12.81 (br, 1H), 8.19-8.08 (m, 2H), 7.85−7.78 (m, 1H), 7.70−7.62 (m, 2H), 7.32−7.21 (m, 2H), 1.22 (td, 3H, J_{HH} = 7.5, 2.0 Hz) ppm.

^{13}C NMR (100 MHz, DMSO-d_6): δ = 189.39, 135.46, 129.57, 129.55, 129.33, 27.83, 11.22 ppm.

ESI-quadrupole-Orbitrap MS (*m/z*): $[M-Cl]^+$ calcd for $C_9H_{12}N^+$, 134.0964; found 134.0964.

1-(*p*-Tolyl)propan-1-imine hydrochloride (**2am**·HCl)

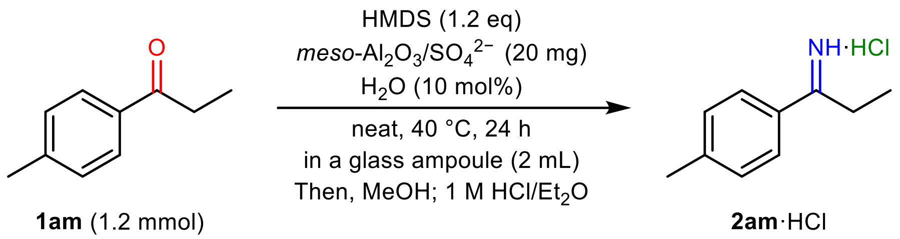

2am was obtained in 93% NMR yield and **2am**·HCl was isolated as a light yellow solid (yield 83%).

^{1}H NMR (400 MHz, DMSO-d_6): δ = 12.47 (br, 2H), 8.03 (d, 2H, J_{HH} = 8.1 Hz), 7.49 (d, 2H, J_{HH} = 8.1 Hz), 3.21 (q, 2H, J_{HH} = 7.5 Hz), 2.44 (s, 3H), 1.22 (t, 3H, J_{HH} = 7.5 Hz) ppm.

^{13}C NMR (100 MHz, DMSO-d_6): δ = 188.55, 146.89, 129.97, 129.78, 126.56, 27.56, 21.29, 12.00 ppm.

ESI-quadrupole-Orbitrap MS (*m/z*): $[M-Cl]^+$ calcd for $C_{10}H_{14}N^+$, 148.1121; found 148.1119.

1-(3-(Trifluoromethyl)phenyl)propan-1-imine hydrochloride (2an·HCl)

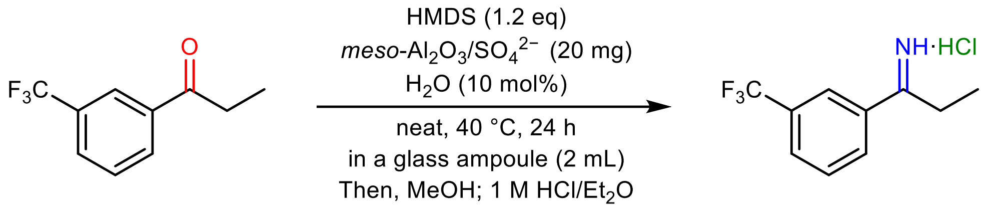

1an (1.2 mmol) **2an**·HCl

2an was obtained in 91% NMR yield and **2an**·HCl was isolated as a white solid (yield 89%).

^{1}H NMR (400 MHz, DMSO-d_6): δ = 13.03 (br, 2H), 8.49−8.40 (m, 1H), 8.36 (t, 1H, J_{HH} = 7.8 Hz), 8.16 (d, 1H, J_{HH} = 7.8 Hz), 7.90 (t, 1H, J_{HH} = 7.8 Hz), 3.36−3.23 (m, 2H), 1.25−1.16 (m, 3H) ppm.

^{13}C NMR (100 MHz, DMSO-d_6): δ = 188.87, 133.62, 131.26 (q, J_{CF} = 4 Hz), 131.00, 130.42, 129.83(q, J_{CF} = 33 Hz), 126.2 (q, J_{CF} = 4 Hz), 123.52 (q, J_{HH} = 273 Hz), 28.35, 11.11 ppm.

^{19}F NMR (376 MHz, DMSO-d_6): δ = −64.5 (d, 3F, J_{HF} = 57.8 Hz) ppm

ESI-quadrupole-Orbitrap MS (*m/z*): $[M-Cl]^+$ calcd for $C_{10}H_{11}F_3N^+$, 202.0838; found 202.0834.

1-(4-Fluorophenyl)propan-1-imine hydrochloride (2ao·HCl)

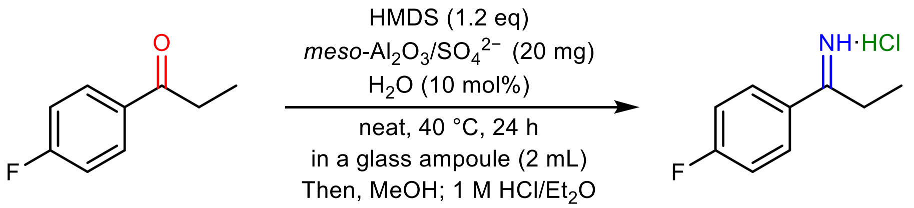

1ao (1.2 mmol) **2ao**·HCl

2ao was obtained in 95% NMR yield and **2ao**·HCl was isolated as a white solid (yield 83%).

^{1}H NMR (400 MHz, DMSO-d_6): δ = 12.72 (br, 2H), 8.30−8.17 (m, 2H), 7.54 (dd, 2H, J_{HH} = 8.8 Hz, J_{HF} = 8.8 Hz), 3.30−3.18 (m, 2H), 1.21 (t, 3H, J_{HH} = 7.5 Hz) ppm.

^{13}C NMR (100 MHz, DMSO-d_6): δ = 187.97, 166.3 (d, J_{CF} = 256 Hz), 133.03 (d, J_{CF} = 10 Hz), 126.18 (d, J_{CF} = 3 Hz), 116.63 (d, J_{CF} = 22 Hz), 27.97, 11.65 ppm.

^{19}F NMR (376 MHz, DMSO-d_6): δ = −105.3 to −105.2) (m, 1F) ppm

ESI-quadrupole-Orbitrap MS (*m/z*): $[M-Cl]^+$ calcd for $C_9H_{11}FN^+$, 152.0870; found 152.0869.

1-(4-Chlorophenyl)propan-1-imine hydrochloride (**2ap**·HCl)

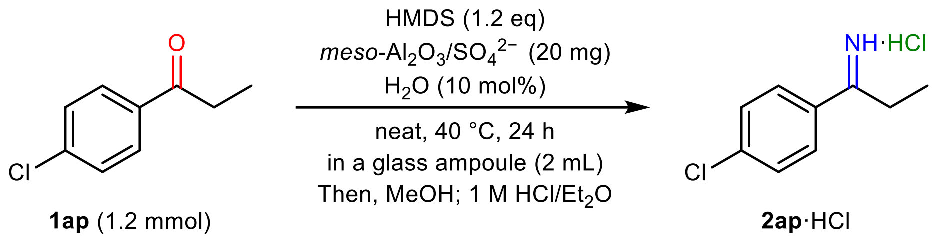

2ap was obtained in 85% NMR yield and **2ap**·HCl was isolated as a white solid (yield 83%).

^{1}H NMR (400 MHz, DMSO-d_6): δ = 12.72 (br, 2H), 8.30−8.17 (m, 2H), 7.54 (dd, 2H, J_{HH} = 8.8 Hz, J_{HF} = 8.8 Hz), 3.30−3.18 (m, 2H), 1.21 (t, 3H, J_{HH} = 7.5 Hz) ppm.

^{13}C NMR (100 MHz, DMSO-d_6): δ = 187.97, 166.3 (d, J_{CF} = 256 Hz), 133.03 (d, J_{CF} = 10 Hz), 126.18 (d, J_{CF} = 3 Hz), 116.63 (d, J_{CF} = 22 Hz), 27.97, 11.65 ppm.

ESI-quadrupole-Orbitrap MS (*m/z*): [M−Cl]$^+$ calcd for $C_9H_{11}ClN^+$, 168.0575; found 168.0576.

1-(4-Bromophenyl)propan-1-imine hydrochloride (**2aq**·HCl)

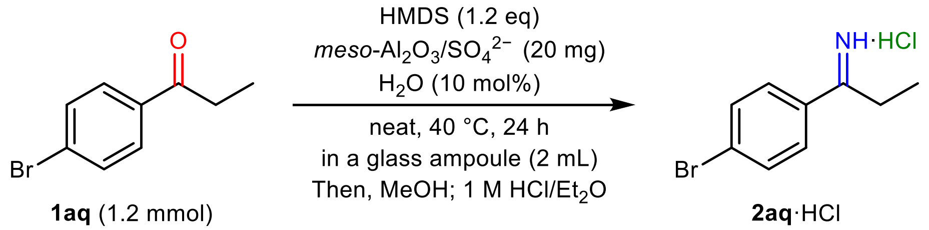

2aq was obtained in 87% NMR yield and **2aq**·HCl was isolated as a white solid (yield 82%).

^{1}H NMR (400 MHz, DMSO-d_6): δ = 12.88 (br, 2H), 8.08−8.00 (m, 2H), 7.94−7.86 (m, 2H), 3.23−3.16 (m, 2H), 1.20 (t, 3H, J_{HH} = 7.5 Hz) ppm.

^{13}C NMR (100 MHz, DMSO-d_6): δ = 188.64, 132.37, 131.50, 129.74, 128.82, 27.97, 11.48 ppm.

ESI-quadrupole-Orbitrap MS (*m/z*): [M−Cl]$^+$ calcd for $C_9H_{11}BrN^+$, 212.0069; found 212.0068.

4-(1-Iminopropyl)phenol hydrochloride (**2ar**·HCl)

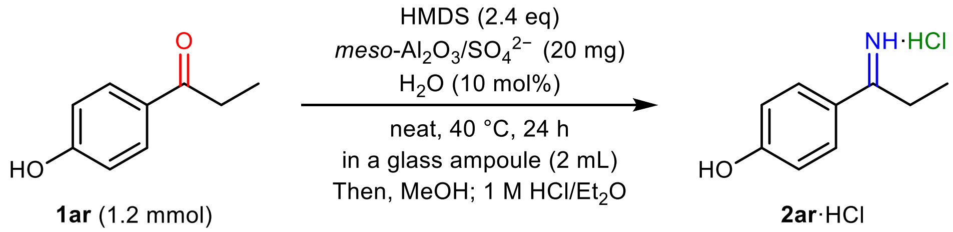

2ar was obtained in 96% NMR yield and **2ar**·HCl was isolated as a white solid (yield 76%).

^{1}H NMR (400 MHz, DMSO-d_6): δ = 11.93 (br, 3H), 8.11 (d, 2H, J_{HH} = 8.9 Hz), 7.05 (d, 2H, J_{HH} = 8.9 Hz), 3.13 (q, 2H, J_{HH} = 7.5 Hz), 1.23 (t, 3H, J_{HH} = 7.5 Hz) ppm.

^{13}C NMR (100 MHz, DMSO-d_6): δ =186.13, 165.26, 132.89, 119.06, 119.40, 27.07, 12.63 ppm.

ESI-quadrupole-Orbitrap MS (*m/z*): [M−Cl]$^+$ calcd for $C_9H_{12}NO^+$, 150.0913; found 150.0913.

1-(4-Methoxyphenyl)propan-1-imine hydrochloride (**2as**·HCl)

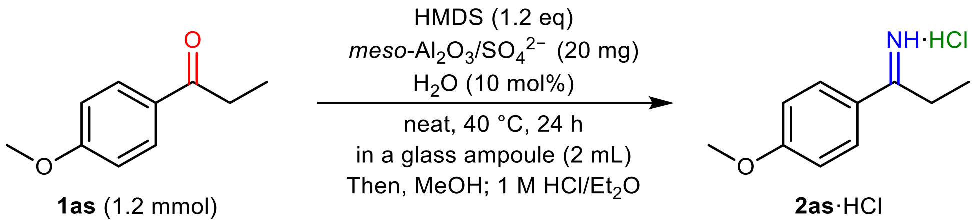

1as (1.2 mmol) **2as**·HCl

2as was obtained in 96% NMR yield and **2as**·HCl was isolated as a white solid (yield 86%).

^{1}H NMR (400 MHz, DMSO-d_6): δ = 12.40 (br, 2H), 8.22 (d, 2H, J_{HH} = 9.0 Hz), 7.19 (d, 2H, J_{HH} = 9.0 Hz), 3.19 (q, 2H, J_{HH} = 7.5 Hz), 2.23 (t, 3H, J_{HH} = 7.5 Hz) ppm.

^{13}C NMR (100 MHz, DMSO-d_6): δ =188.55, 146.89, 129.97, 129.78, 126.56, 27.56, 21.29, 12.00 ppm.

ESI-quadrupole-Orbitrap MS (*m/z*): [M−Cl]$^+$ calcd for $C_{10}H_{14}NO^+$, 164.1070; found 164.1069.

1-(3-Nitrophenyl)propan-1-imine hydrochloride (**2at**·HCl)

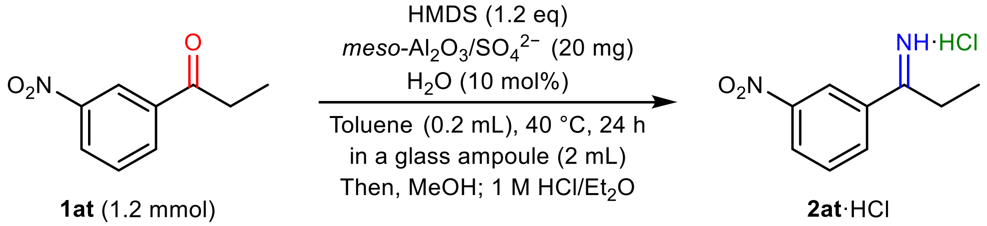

1at (1.2 mmol) **2at**·HCl

2at was obtained in 98% NMR yield and **2at**·HCl was isolated as a light yellow solid (yield 97%).

^{1}H NMR (400 MHz, DMSO-d_6): δ = 13.17 (br, 2H), 8.98−8.82 (m, 1H), 8.65−8.56 (m, 1H), 8.55−8.41 (m, 1H), 8.04−7.91 (m, 1H), 3.44−3.26 (m, 2H), 1.29−1.17 (m, 3H) ppm.

^{13}C NMR (100 MHz, DMSO-d_6): δ = 188.54, 147.89, 135.69, 131.49, 130.89, 128.93, 124.28, 28.56, 11.02 ppm.

ESI-quadrupole-Orbitrap MS (*m/z*): [M−Cl]$^+$ calcd for $C_9H_{11}N_2O_2^+$, 179.0815; found 179.0813.

1-Phenylbutan-1-imine hydrochloride (**2au**·HCl)

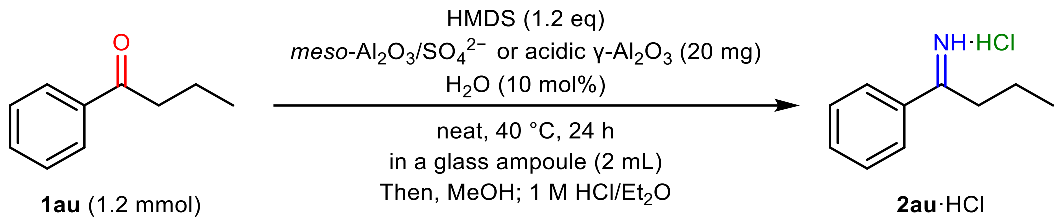

2au was obtained in 96% NMR yield (93% with γ-Al_2O_3), and **2au**·HCl was isolated as a white solid (yield 75%).

^{1}H NMR (400 MHz, DMSO-*d*$_6$): δ = 12.73 (br, 1H), 12.64 (br, 1H), 8.11 (m, 2H), 7.82 (m, 1H), 7.67 (m, 2H), 3.25−3.16 (m, 2H), 1.69−1.56 (m, 2H), 0.96−0.89 (m, 3H) ppm.

^{13}C NMR (100 MHz, DMSO-*d*$_6$): δ = 188.93, 136.14, 130.20, 130.15, 129.95, 36.25, 21.43, 13.74 ppm.

ESI-quadrupole-Orbitrap MS (*m/z*): $[M−Cl]^+$ calcd for $C_{10}H_{14}N^+$, 148.1121; found 148.1120.

Cyclopropyl(phenyl)methanimine hydrochloride (**2av**·HCl)

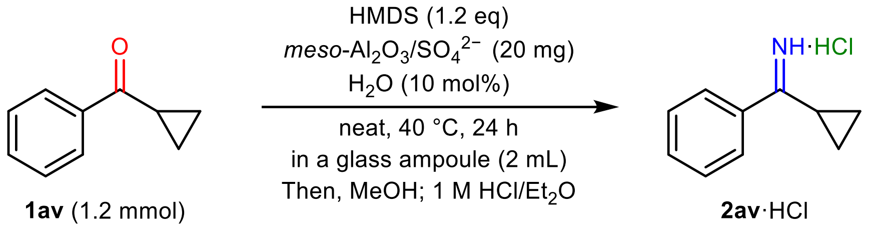

2av was obtained in 96% NMR yield and **2av**·HCl was isolated as a white solid (yield 75%).

^{1}H NMR (400 MHz, DMSO-*d*$_6$): δ = 12.01 (br, 2H), 11.96 (br, 2H), 8.07−8.01 (m, 2H), 7.80 (td, 1H, J_{HH} = 7.4, 1.4 Hz), 7.66 (td, 2H, J_{HH} = 7.4, 1.4 Hz), 2.68 (m, 1H), 1.67−1.50 (m, 4H) ppm.

^{13}C NMR (100 MHz, DMSO-*d*$_6$): δ = 189.88, 134.62, 131.91, 129.08, 129.00, 17.20, 14.81 ppm.

ESI-quadrupole-Orbitrap MS (*m/z*): $[M−Cl]^+$ calcd for $C_{10}H_{12}N^+$, 146.0964; found 146.0962.

2-Methyl-1-phenylpropan-1-imine hydrochloride (**2aw**·HCl)

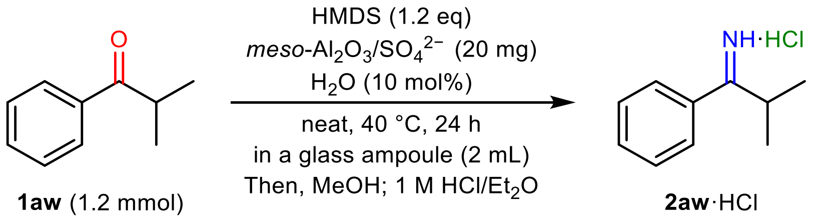

2aw was obtained in 87% NMR yield and **2aw**·HCl was isolated as a white solid (yield 80%).

^{1}H NMR (400 MHz, DMSO-d_6): δ = 12.78 (br, 2H), 8.09 (d, 2H, J_{HH} = 7.5 Hz), 7.80 (t, 1H, J_{HH} = 7.5 Hz), 7.64 (t, 2H, J_{HH} = 7.5 Hz), 3.72 (sep, 1H, J_{HH} = 6.9 Hz), 1.32 (d, 6H, J_{HH} = 6.9 Hz) ppm.

^{13}C NMR (100 MHz, DMSO-d_6): δ = 193.40, 137.8, 130.3, 128.5, 127.0, 40.0, 19.3, 13.7 ppm.

ESI-quadrupole-Orbitrap MS (*m/z*): [M−Cl]$^+$ calcd for $C_{10}H_{14}N^+$, 148.1121; found 148.1123.

Cyclohexyl(phenyl)methanimine hydrochloride (**2ax**·HCl)

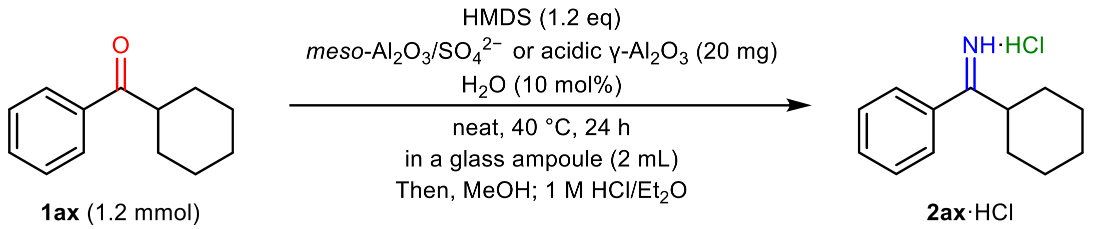

2ax was obtained in >99% NMR yield (>99% with γ-Al_2O_3), and **2ax**·HCl was isolated as a white solid (yield 91%).

^{1}H NMR (400 MHz, DMSO-d_6): δ = 12.69 (br, 2H), 8.08 (d, 2H, J_{HH} = 7.7 Hz), 7.80 (t, 1H, J_{HH} = 7.7 Hz), 7.64 (t, 2H, J_{HH} = 7.7 Hz), 3.38 (t, 1H, J_{HH} = 11.5 Hz), 1.94−1.14 (m, 10H) ppm.

^{13}C NMR (100 MHz, DMSO-d_6): δ = 192.19, 135.06, 130.52, 129.31, 129.23, 43.22, 29.83, 25.06, 24.89 ppm.

ESI-quadrupole-Orbitrap MS (*m/z*): [M−Cl]$^+$ calcd for $C_{13}H_{18}N^+$, 188.1434; found 188.1431.

2-Imino-1,2-diphenylethan-1-ol hydrochloride (**2ay**·HCl)

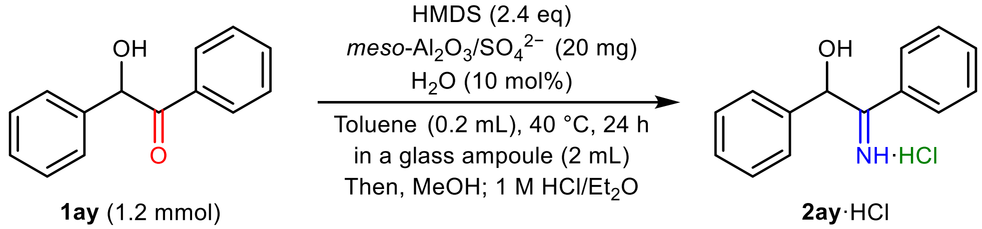

2ay was obtained in 74% NMR yield and **2ay**·HCl was isolated as a white solid (yield 72%).

^{1}H NMR (400 MHz, DMSO-d_6): δ = 9.11 (br, 3H), 8.06 (d, 2H, J_{HH} = 7.3 Hz), 7.65−7.53 (m, 3H), 7.52−7.45 (m, 2H), 7.43−7.31 (m, 3H), 6.38 (s, 1H) ppm.

^{13}C NMR (100 MHz, DMSO-d_6): δ = 193.36, 134.36, 133.14, 132.60, 129.50, 129.20, 129.07, 129.01, 128.94, 57.91 ppm.

ESI-quadrupole-Orbitrap MS (*m/z*): [M−Cl]$^+$ calcd for $C_{14}H_{14}NO^+$, 212.1070; found 212.1068.

2-Methyl-1-(4-(methylthio)phenyl)-2-morpholinopropan-1-imine (**2az**)

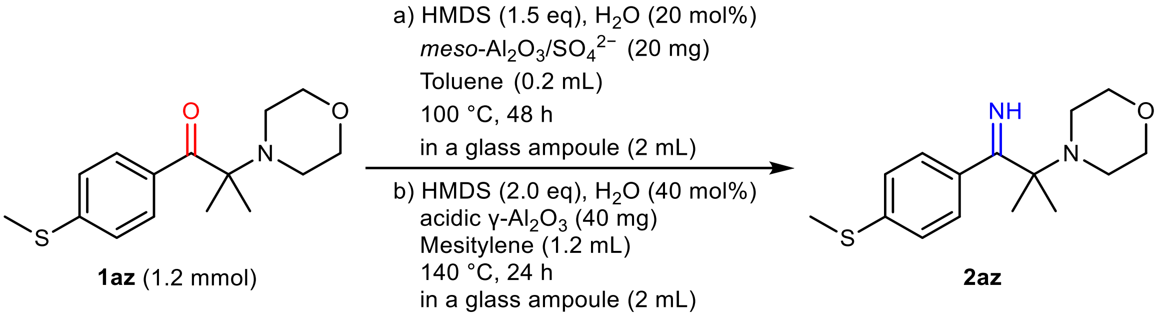

Isolated as a light yellow liquid (*meso*–Al_2O_3/SO_4^{2-}: >99% yield; γ-Al_2O_3: 98% yield).

^{1}H NMR (400 MHz, DMSO-d_6): δ = 10.25 (br, 1H), 7.81 (br, 2H), 7.23 (d, 2H, J_{HH} = 8.0 Hz), 3.59 (br, 4H), 2.48 (s, 3H), 2.47 (br, 4H), 1.15 (s, 6H) ppm.

^{13}C NMR (100 MHz, DMSO-d_6): δ = 180.60, 139.21, 136.10, 128.38, 124.65, 66.70, 63.72, 46.21, 20.83, 14.28 ppm.

ESI-quadrupole-Orbitrap MS (*m/z*): $[M+H]^+$ calcd for $C_{15}H_{21}NO_2S^+$, 279.1526; found 279.1518.

2,2-Dimethyl-1-phenylpropan-1-imine hydrochloride (**2ba**·HCl)

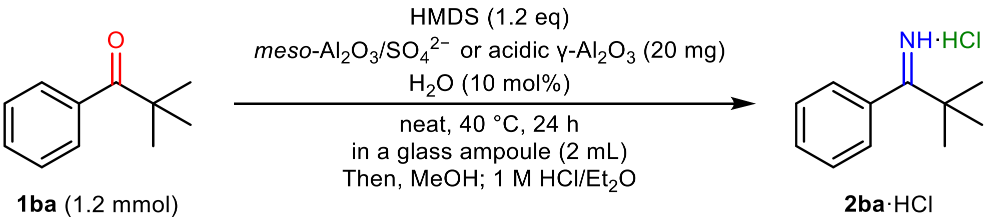

2ba was obtained in 94% NMR yield (95% with γ-Al_2O_3), and **2ba**·HCl was isolated as a white solid (yield 89%).

^{1}H NMR (400 MHz, DMSO-d_6): δ = 12.95 (br, 2H), 7.70−7.51 (m, 5H), 1.36 (s, 9H) ppm.

^{13}C NMR (100 MHz, DMSO-d_6): δ = 199.25, 132.17, 131.59, 128.39, 127.10, 40.46, 27.56 ppm.

ESI-quadrupole-Orbitrap MS (*m/z*): $[M-Cl]^+$ calcd for $C_{11}H_{16}N^+$, 162.1277; found 162.1278.

2,2-Dimethyl-3,4-dihydronaphthalen-1(2*H*)-imine (**2bb**)

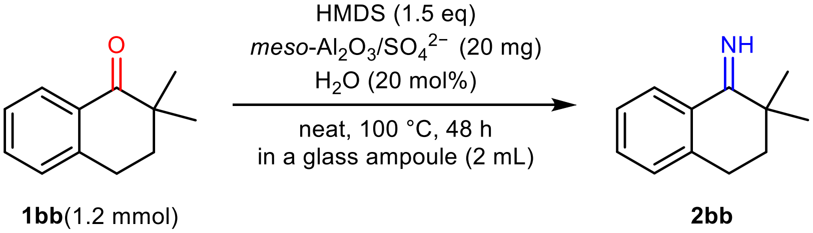

Isolated as a light yellow liquid (yield 97%).

^{1}H NMR (400 MHz, $CDCl_3$): δ = 9.41 (br, 1H), 8.10 (br, 1H), 7.33 (t, 1H, J_{HH} = 7.4 Hz), 7.25 (t, 1H, J_{HH} = 7.4 Hz), 7.15 (d, 1H, J_{HH} = 7.4 Hz), 2.91 (t, 2H, J_{HH} = 6.4 Hz), 1.83 (t, 2H, J_{HH} = 6.4 Hz), 1.21 (s, 6H) ppm.

^{13}C NMR (100 MHz, $CDCl_3$): δ =193.36, 134.36, 133.14, 132.60, 129.50, 129.20, 129.07, 129.01, 128.94, 57.91 ppm.

ESI-quadrupole-Orbitrap MS (*m/z*): $[M+H]^+$ calcd for $C_{12}H_{16}N^+$, 174.1277; found 174.1275.

2,2,4,4-Tetramethylpentan-3-imine hydrochloride (**2bc**·HCl)

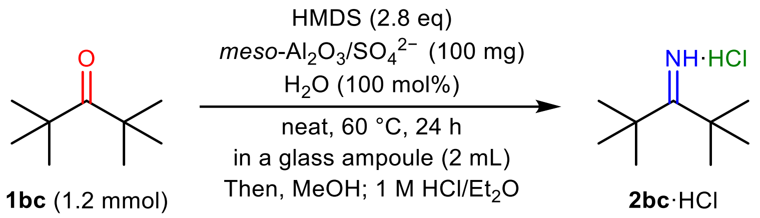

2bc was obtained in 85% NMR yield and **2bc**·HCl was isolated as a white solid (yield 82%).

^{1}H NMR (400 MHz, DMSO-d_6): δ = 11.88 (br, 2H), 1.41 (s, 9H) ppm.

^{13}C NMR (100 MHz, DMSO-d_6): δ = 209.14, 42.08, 28.66 ppm

ESI-quadrupole-Orbitrap MS (*m/z*): $[M-Cl]^+$ calcd for $C_9H_{20}N^+$, 142.1590; found 142.1589.

(1*S*,4*S*)-1,3,3-Trimethylbicyclo[2.2.1]heptan-2-imine hydrochloride (**2bd**·HCl)

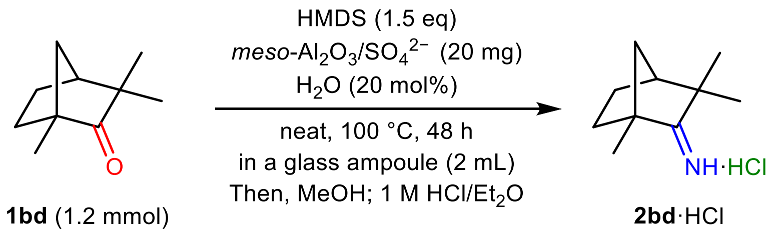

2bc was obtained in 96% NMR yield and **2bc**·HCl was isolated as a white solid (yield 87%).

^{1}H NMR (400 MHz, DMSO-d_6): δ = 12.39 (br, 2H), 2.11 (br, 1H), 1.96 (d, 1H, J_{HH} = 10.6 Hz), 1.90−1.77 (m, 1H), 1.75−1.58 (m, 3H), 1.49−1.39 (m, 1H), 1.35 (s, 3H), 1.29−1.25 (m, 6H) ppm.

^{13}C NMR (100 MHz, DMSO-d_6): δ = 211.42, 53.23, 46.42, 45.78, 42.51, 32.75, 23.97, 23.82, 22.04, 15.15 ppm.

ESI-quadrupole-Orbitrap MS (*m/z*): $[M-Cl]^+$ calcd for $C_{10}H_{18}N^+$, 152.1434; found 152.1432.

(1*S*,4*S*)-1,7,7-Trimethylbicyclo[2.2.1]heptan-2-imine hydrochloride (**2be**·HCl)

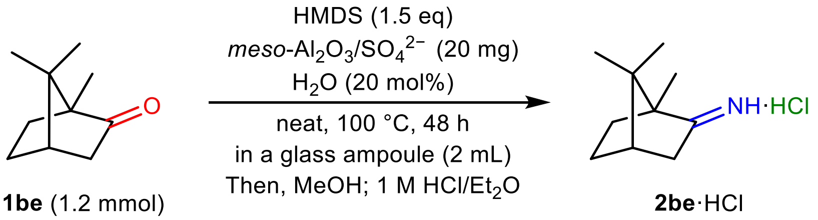

2be was obtained in 96% NMR yield and **2be**·HCl was isolated as a white solid (yield 87%).

^{1}H NMR (400 MHz, DMSO-*d*$_6$): δ = 12.57 (br, 2H), 3.00−3.83 (m, 1H), 2.44 (br, 1H), 2.16−2.06 (m, 1H), 1.98−1.78 (m, 2H), 1.48−1.30 (m, 2H), 1.11 (s, 3H), 0.94 (s, 3H), 0.79 (s, 3H) ppm.

^{13}C NMR (100 MHz, DMSO-*d*$_6$): δ = 206.33, 56.98, 49.30, 42.80, 37.91, 31.34, 25.46, 19.12, 18.10, 9.79 ppm.

ESI-quadrupole-Orbitrap MS (*m/z*): $[M-Cl]^+$ calcd for $C_{10}H_{18}N^+$, 152.1434; found 152.1437.

Dicyclohexylmethanimine (**2bf**)

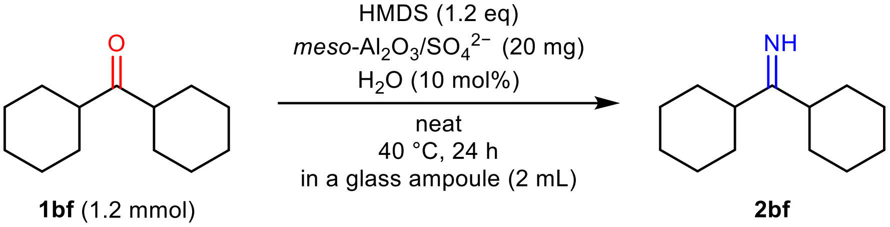

Isolated as a light yellow liquid (yield >99%).

^{1}H NMR (400 MHz, $CDCl_3$): δ = 8.93 (br, 1H), 2.23−2.09 (m, 2H), 1.88−1.57 (m, 10H), 1.38−1.12 (m, 10H) ppm.

^{13}C NMR (100 MHz, $CDCl_3$): δ = 192.19, 46.46, 30.60, 26.26, 26.02 ppm.

ESI-quadrupole-Orbitrap MS (*m/z*): $[M+H]^+$ calcd for $C_{13}H_{24}N^+$, 194.1903; found 194.1902.

Dicyclohexylmethanimine hydrochloride (**2bf**·HCl)

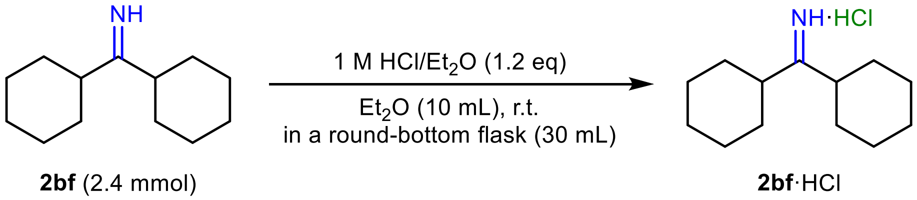

Isolated as a white solid (yield 94%).

^{1}H NMR (400 MHz, $CDCl_3$): δ = 12.50 (br, 1H), 2.86−2.68 (m, 2H), 1.89−1.41 (m, 14H), 1.40−1.12 (m, 6H) ppm.

^{13}C NMR (100 MHz, $CDCl_3$): δ = 204.76, 44.32, 28.66, 24.80, 24.68 ppm.

ESI-quadrupole-Orbitrap MS (*m/z*): $[M-Cl]^+$ calcd for $C_{13}H_{24}N^+$, 194.1903; found 194.1900.

Adamantan-2-imine hydrochloride (**2bg**·HCl)

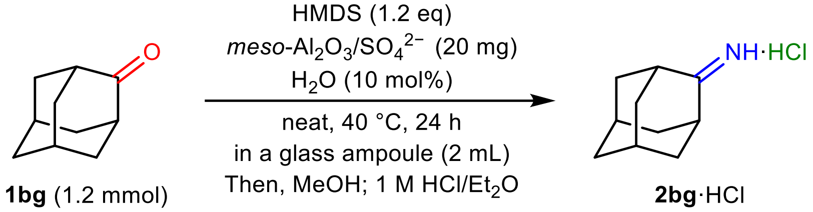

2bg was obtained in >99% NMR yield and **2bg**·HCl was isolated as a white solid (yield 89%).

^{1}H NMR (400 MHz, DMSO-d_6): δ = 12.53 (br, 2H), 3.10 (br, 2H), 2.21−2.11 (m, 4H), 2.04−1.92 (m, 6H), 1.88 (br, 2H) ppm.

^{13}C NMR (100 MHz, DMSO-d_6): δ = 204.89, 38.78, 38.42, 34.94, 26.27 ppm.

ESI-quadrupole-Orbitrap MS (*m/z*): $[M-Cl]^+$ calcd for $C_{10}H_{16}N^+$, 150.1277; found 150.1277.

Dicyclopropylmethanimine hydrochloride (**2bh**·HCl)

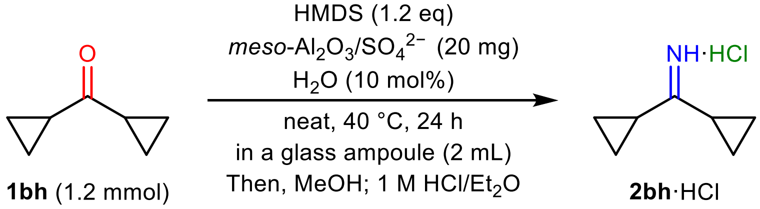

2bh was obtained in 93% NMR yield and **2bh**·HCl was isolated as a white solid (yield 89%).

^{1}H NMR (400 MHz, DMSO-d_6): δ = 11.28 (br, 2H), 2.00−1.90 (m, 2H), 1.44−1.32 (m, 10H), 1.38−1.12 (m, 8H) ppm.

^{13}C NMR (100 MHz, DMSO-d_6): δ = 200.67, 15.65, 13.07 ppm.

ESI-quadrupole-Orbitrap MS (*m/z*): $[M-Cl]^+$ calcd for $C_7H_{12}N^+$, 110.0964; found 110.0967.

2,4-Dimethylpentan-3-imine hydrochloride (**2bi**·HCl)

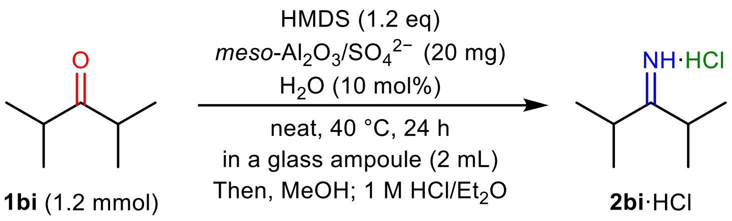

2bi was obtained in >96% NMR yield and **2bi**·HCl was isolated as a white solid (yield 94%).

^{1}H NMR (400 MHz, DMSO-d_6): δ = 12.41 (br, 2H), 3.12−2.98 (m, 2H), 1.26−1.18 (m, 12H) ppm.

^{13}C NMR (100 MHz, DMSO-d_6): δ = 208.36, 34.56, 19.73 ppm.

ESI-quadrupole-Orbitrap MS (*m/z*): [M−Cl]$^+$ calcd for $C_7H_{16}N^+$, 114.1277; found 114.1279.

3,3-Dimethylbutan-2-imine hydrochloride (**2bj**·HCl)

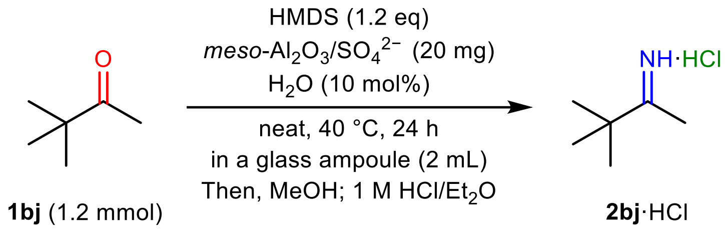

2bj was obtained in >86% NMR yield and **2bj**·HCl was isolated as a white solid (yield 92%).

^{1}H NMR (400 MHz, CD_3CN): δ = 13.63 (br, 1H), 11.79 (br, 1H), 2.55 (s, 3H), 1.32 (s,9H) ppm.

^{13}C NMR (100 MHz, CD_3CN): δ = 204.36, 41.41, 27.26, 20.78 ppm.

ESI-quadrupole-Orbitrap MS (*m/z*): [M−Cl]$^+$ calcd for $C_6H_{14}N^+$, 100.1121; found 100.1124.

9.2 *N*–R Ketimine

1,1-Diphenyl-*N*-propylmethanimine (3a)

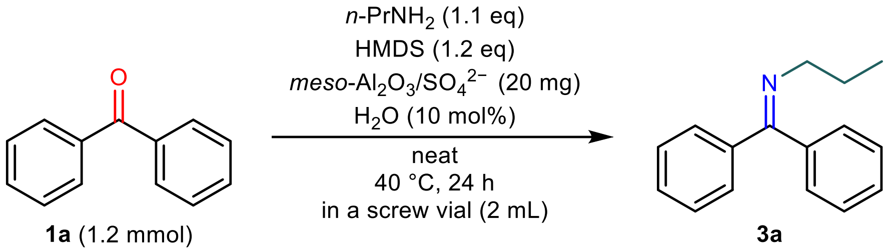

Isolated as a colorless liquid (yield >99%).

^{1}H NMR (400 MHz, $CDCl_3$): δ = 7.63−7.56 (m, 2H), 7.49−7.28 (m, 6H), 7.18−7.13 (m, 2H), 7.34 (t, 2H, J_{HH} = 7.2 Hz), 1.79 (sext, 2H, J_{HH} = 7.2 Hz), 0.91 (t, 3H, J_{HH} = 7.2 Hz) ppm.

^{13}C NMR (100 MHz, $CDCl_3$): δ = 167.73, 140.07, 137.11, 129.69, 128.37, 128.26, 128.16, 127.99, 127.85, 55.65, 24.46, 12.04 ppm.

ESI-quadrupole-Orbitrap MS (*m/z*): $[M+H]^+$ calcd for $C_{16}H_{18}N^+$, 224.1434; found 224.1428.

N-benzyl-1,1-diphenylmethanimine (3b)

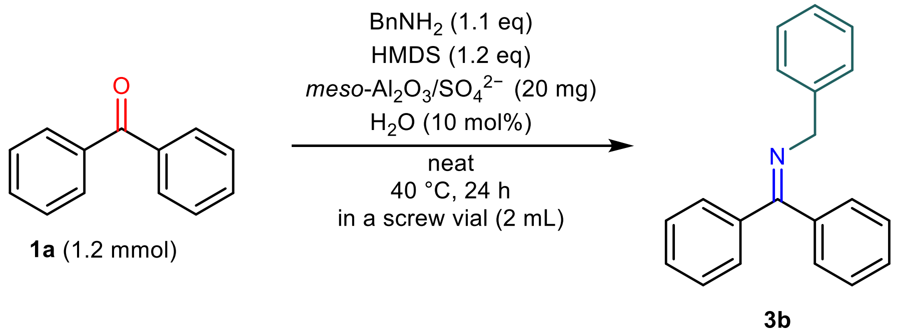

Isolated as a white solid (yield >99%).

^{1}H NMR (400 MHz, $CDCl_3$): δ = 7.72−7.66 (m, 2H), 7.50−7.23 (m, 10H), 7.26−7.16 (m, 3H), 4.61 (s, 2H) ppm.

^{13}C NMR (100 MHz, $CDCl_3$): δ = 168.77, 140.65, 139.76, 136.69, 130.02, 128.55, 128.53, 128.48, 128.29, 128.03, 127.76, 127.61, 126.48, 57.39 ppm.

ESI-quadrupole-Orbitrap MS (*m/z*): $[M+H]^+$ calcd for $C_{20}H_{18}N^+$, 272.1434; found 272.1429.

N,1,1-triphenylmethanimine (3c)

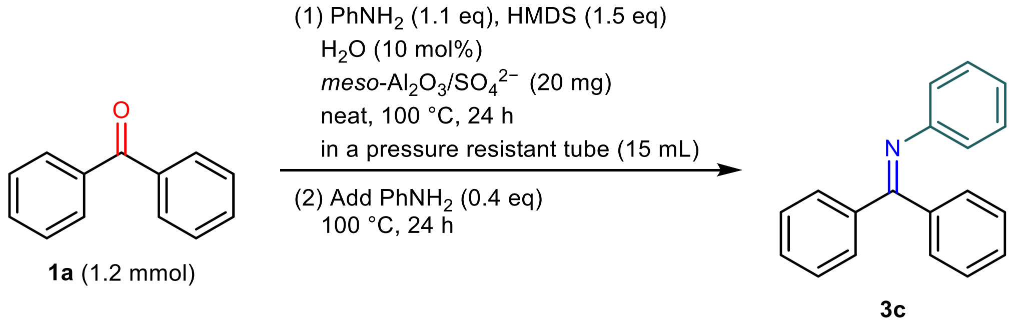

Isolated as a light yellow solid (yield >92%).

^{1}H NMR (400 MHz, $CDCl_3$): δ = 7.77−7.72 (m, 2H), 7.50−7.43 (m, 1H), 7.43−7.36 (m, 2H), 7.29−7.21 (m, 3H), 7.17−7.08 (m, 4H), 6.91 (t, 1H, J_{HH} = 7.4 Hz), 6.72 (d, 2H, J_{HH} = 7.4 Hz) ppm.

^{13}C NMR (100 MHz, $CDCl_3$): δ = 168.22, 151.25, 139.69, 136.23, 130.69, 129.51, 129.31, 128.53, 128.44, 128.18, 127.87, 123.11, 120.92 ppm.

ESI-quadrupole-Orbitrap MS (*m/z*): $[M+H]^+$ calcd for $C_{29}H_{16}N^+$, 258.1277; found 258.1273.

1-Phenyl-*N*-propyl-1-(pyridin-2-yl)methanimine (3d)

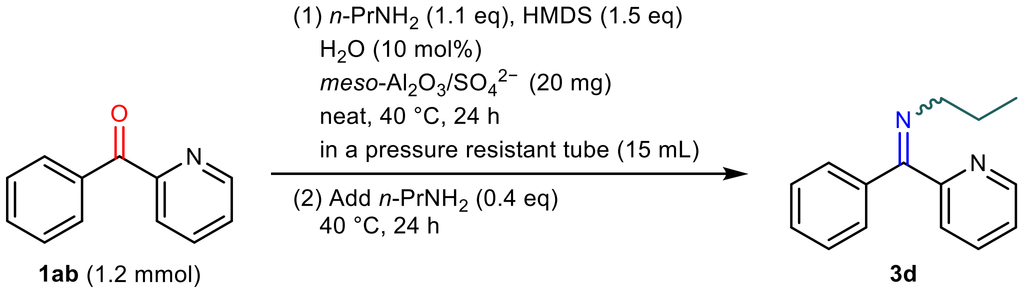

Isolated as a colorless liquid (yield 99%, *E*:*Z* = 1:1).

^{1}H NMR (400 MHz, $CDCl_3$): δ = 8.77−8.73 (m, 0.52H, *Z*), 8.61−8.57 (m, 0.48H, *E*), 7.94 (d, 0.48H, J_{HH} = 7.8 Hz, *E*), 7.80 (tt, 0.52H, J_{HH} = 7.8, 1.7 Hz, *Z*), 7.71 (tt, 0.48H, J_{HH} = 7.8, 1.7 Hz, *E*), 7.58−7.29 (m, 4.52H, *E*+*Z*), 7.26−7.17 (m, 2H, *E*+*Z*), 3.42 (t, 0.96H, J_{HH} = 7.1 Hz *E*), 3.33 (t, 1.04H, J_{HH} = 7.1 Hz, *Z*), 1.80−1.67 (m, 2H, *E*+*Z*), 0.95−0.89 (m, 3H, *E*+*Z*) ppm. The assignment was determined based on the ^{1}H NMR trends of *N*−H ketimine **2ab**, which has a similar structure.

^{13}C NMR (100 MHz, $CDCl_3$, *E*+*Z* isomers): δ = 167.96, 166.02, 157.57, 156.05, 149.97, 149.09, 138.95, 136.39, 136.18, 136.15, 129.84, 128.29, 128.27, 128.09, 128.05, 127.92, 123.78, 123.30, 122.98, 122.62, 55.91, 55.44, 24.34, 24.33, 12.07, 12.02 ppm.

ESI-quadrupole-Orbitrap MS (*m/z*): $[M+H]^+$ calcd for $C_{15}H_{17}N_2^+$, 225.1386; found 225.1382.

N-Benzyladamantan-2-imine (3e)

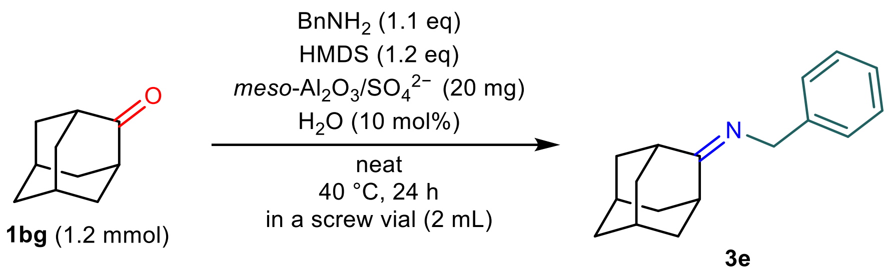

Isolated as a colorless liquid (yield >99%).

^{1}H NMR (400 MHz, $CDCl_3$): δ = 7.72–7.66 (m, 2H), 7.50–7.23 (m, 10H), 7.26–7.16 (m, 3H), 4.61 (s, 2H) ppm.

^{13}C NMR (100 MHz, $CDCl_3$): δ = 180.59, 140.75, 128.34, 127.72, 126.38, 53.41, 43.87, 39.18, 38.25, 36.50, 33.25, 27.78 ppm.

ESI-quadrupole-Orbitrap MS (*m/z*): $[M+H]^+$ calcd for $C_{17}H_{22}N^+$, 240.1747; found 240.1741.

1,1-Dicyclohexyl-N-propylmethanimine (3f)

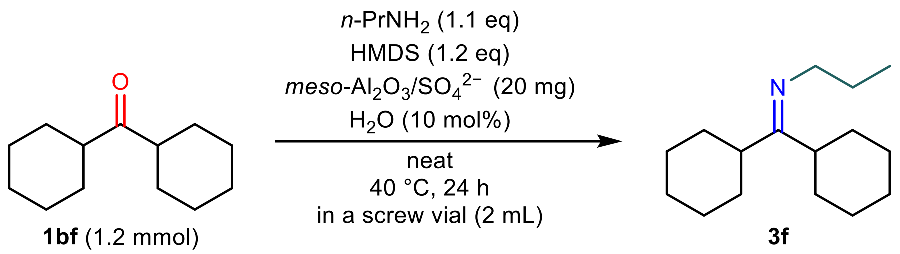

Isolated as a light yellow liquid (yield >99%).

^{1}H NMR (400 MHz, $CDCl_3$): δ = 3.34 (t, 2H, J_{HH} = 7.2 Hz), 2.69 (tt, 1H, J_{HH} = 11.5, 3.5 Hz), 2.29 (tt, 1H, J_{HH} = 11.5, 3.5 Hz), 1.86–1.13 (m, 22H), 0.90 (t, 3H, J_{HH} = 7.2 Hz) ppm.

^{13}C NMR (100 MHz, $CDCl_3$): δ = 179.53, 51.47, 42.57, 40.61, 32.25, 29.29, 26.69, 26.13. 26.12, 26.02, 24.57, 12.00 ppm.

ESI-quadrupole-Orbitrap MS (*m/z*): $[M+H]^+$ calcd for $C_{16}H_{30}N^+$, 236.2373; found 236.2367.

N-Benzyl-1,1-dicyclohexylmethanimine (3g)

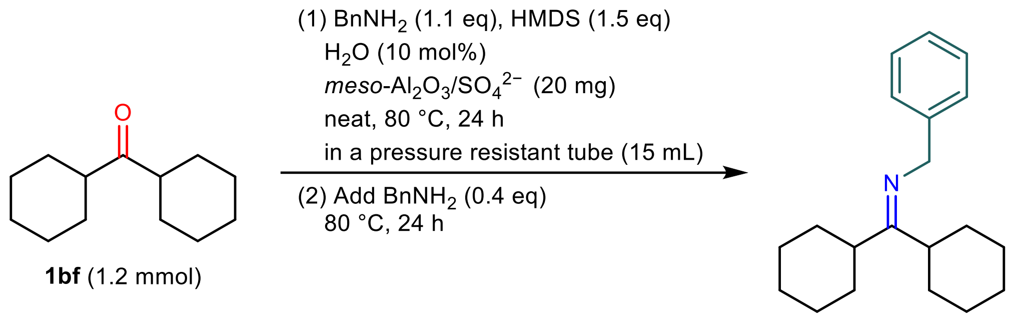

3g

Isolated as a white solid (yield >99%).

^{1}H NMR (400 MHz, $CDCl_3$): δ = 7.33−7.24 (m, 4H), 7.22−7.16 (m, 1H), 4.66 (s, 2H), 2.74 (tt, 1H, J_{HH} = 11.7, 3.5 Hz), 2.39 (tt, 1H, J_{HH} = 11.5, 3.3 Hz), 1.83−1.61 (m, 8H), 1.61−1.36 (m, 6H), 1.36−1.16 (m, 6H) ppm.

^{13}C NMR (100 MHz, $CDCl_3$): δ = 181.30, 141.26, 128.14, 127.15, 126.11, 52.79, 42.81, 41.09, 32.22, 29.02, 26.57, 25.95 ppm.

ESI-quadrupole-Orbitrap MS (*m/z*): $[M+H]^+$ calcd for $C_{20}H_{30}N^+$, 284.2373; found 284.2366.

1,1-Dicyclohexyl-*N*-phenylmethanimine (3h)

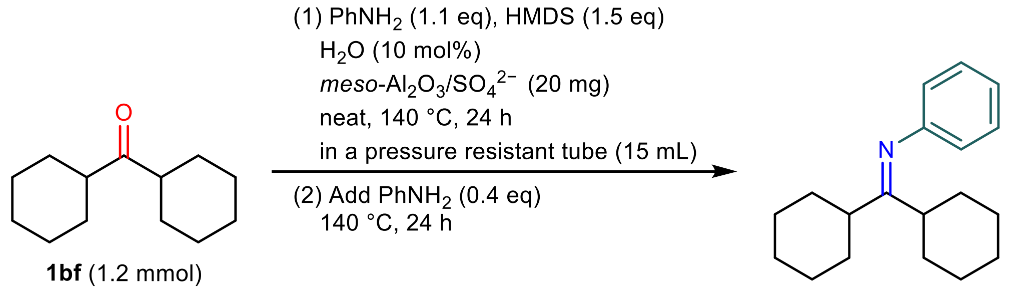

3h

Isolated as a white solid (yield 86%).

^{1}H NMR (400 MHz, $CDCl_3$): δ = 7.25 (t, 2H, J_{HH} = 7.6 Hz), 6.98 (t, 1H, J_{HH} = 7.6 Hz), 6.60 (d, 2H, J_{HH} = 7.6 Hz), 2.45–2.38 (m, 2H), 1.86–0.99 (m, 20H) ppm.

^{13}C NMR (100 MHz, $CDCl_3$): δ = 181.83, 151.52, 128.72, 122.24, 119.06, 43.27, 41.93. 32.24, 29.43, 26.46, 25.90, 25.76, 25.34 ppm.

ESI-quadrupole-Orbitrap MS (*m/z*): $[M+H]^+$ calcd for $C_{19}H_{28}N^+$, 270.2216; found 270.2210.

9.3 α-Aminonitrile

2-Amino-2,2-diphenylacetonitrile (4a)

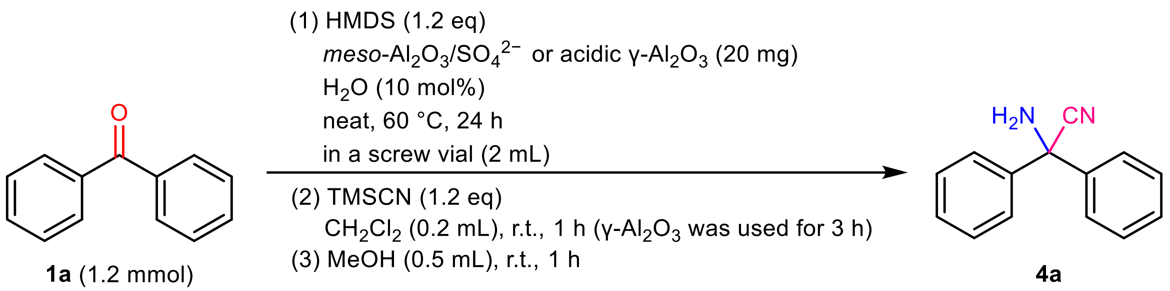

1a (1.2 mmol) **4a**

Isolated as a white solid (yield >99%).

^{1}H NMR (400 MHz, $CDCl_3$): δ = 7.67−7.61 (m, 4H), 7.41−7.28 (m, 6H), 2.40 (br, 2H) ppm.

^{13}C NMR (100 MHz, $CDCl_3$): δ = 141.12, 128.80, 128.55, 125.73, 123.42, 60.71 ppm.

ESI-quadrupole-Orbitrap MS (*m/z*): $[M{-}NH_2]^+$ calcd for $C_{14}H_{10}N^+$, 192.0808; found 192.0804, $[M{-}CN]^+$ calcd for $C_{13}H_{12}N^+$, 182.0964; found 182.0963.

2-(4-(Allyloxy)phenyl)-2-amino-2-phenylacetonitrile (4b)

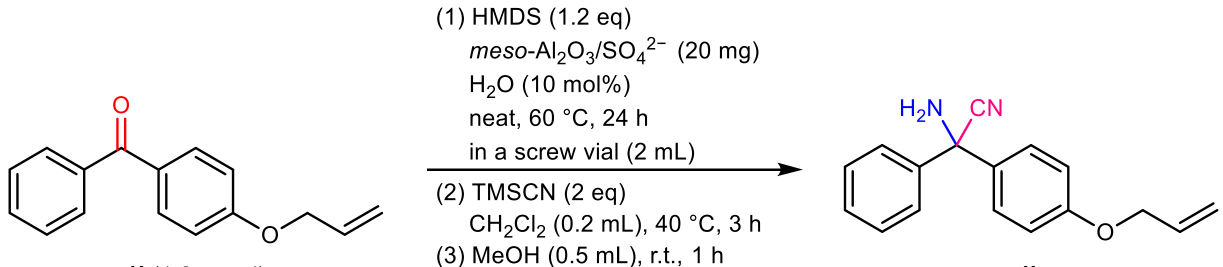

1j (1.2 mmol) **4b**

Isolated as a white solid (yield >99%)

^{1}H NMR (400 MHz, $CDCl_3$): δ = 7.65−7.59 (m, 2H), 7.55−7.50 (m, 2H), 7.40−7.27 (m, 3H), 6.91−6.86 (m, 2H), 6.08−5.96 (m, 1H), 5.39 (dq, 1H, J_{HH} = 17.3, 1.4 Hz), 5.28 (dq, 1H, J_{HH} = 10.5, 1.4 Hz), 4.52 (dt, 2H, J_{HH} = 5.3, 1.4 Hz), 2.36 (br, 2H) ppm.

^{13}C NMR (100 MHz, $CDCl_3$): δ =158.67, 141.39, 133.43, 132.92, 128.80, 128.49, 127.06, 125.70, 123.59, 117.85, 114.87, 68.83, 60.25 ppm.

ESI-quadrupole-Orbitrap MS (*m/z*): $[M{-}NH_2]^+$ calcd for $C_{17}H_{14}NO^+$, 248.1070; found 248.1072, $[M{-}CN]^+$ calcd for $C_{16}H_{16}NO^+$, 238.1226; found 238.1229.

2-Amino-2-(4-bromophenyl)butanenitrile (4c)

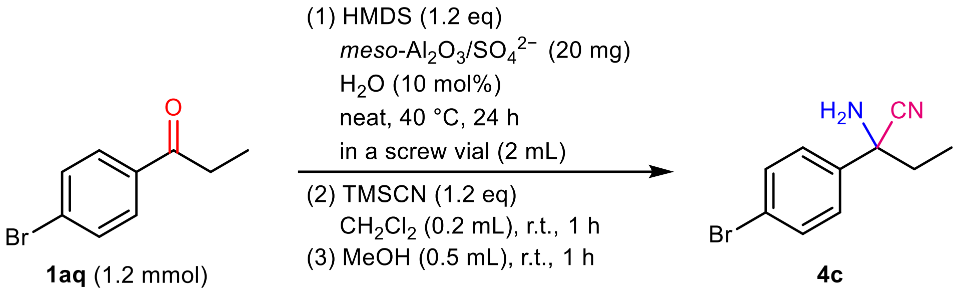

Isolated as a light yellow solid (yield >94%).

^{1}H NMR (400 MHz, $CDCl_3$): δ = 7.56 (m, 4H), 2.08 (br, 2H), 2.01−1.84 (m, 2H), 0.97 (t, 3H, J_{HH} = 7.5 Hz) ppm.

^{13}C NMR (100 MHz, $CDCl_3$): δ = 138.97, 131.78, 127.42, 122.81, 122.68, 58.36, 37.17, 8.93 ppm.

ESI-quadrupole-Orbitrap MS (*m/z*): $[M−NH_2]^+$ calcd for $C_{10}H_9BrN^+$, 221.9913; found 221.9913, $[M−CN]^+$ calcd for $C_9H_{11}BrN^+$, 212.0069; found 212.0070.

2-Amino-2-phenylpentanenitrile (4d)

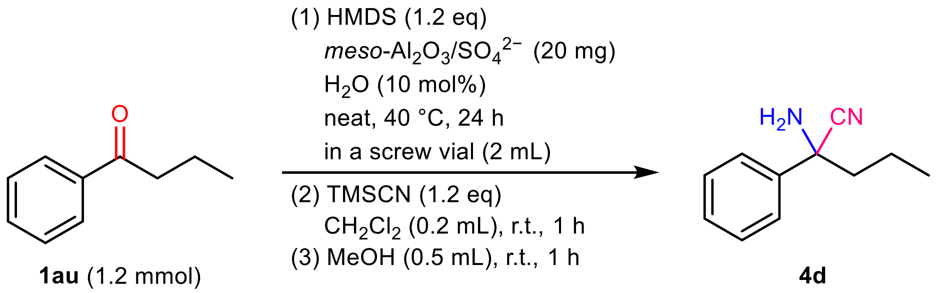

Isolated as a light yellow liquid (yield >99%).

^{1}H NMR (400 MHz, $CDCl_3$): δ = 7.63 (d, 2H, J_{HH} = 7.7 Hz), 7.44−7.32 (m, 3H), 2.09 (br, 2H), 1.97−1.81 (m, 2H), 1.54−1.25 (m, 2H), 0.91 (t, 3H, J_{HH} = 7.7 Hz) ppm.

^{13}C NMR (100 MHz, $CDCl_3$): δ = 140.20, 128.65, 128.54, 125.47, 123.45, 58.04, 46.17, 18.06, 13.67 ppm.

ESI-quadrupole-Orbitrap MS (*m/z*): $[M−NH_2]^+$ calcd for $C_{11}H_{12}N^+$, 158.0964; found 158.0959, $[M−CN]^+$ calcd for $C_{10}H_{14}N^+$, 148.1121; found 148.1115.

2-Amino-3-methyl-2-(4-(methylthio)phenyl)-3-morpholinobutanenitrile (4e)

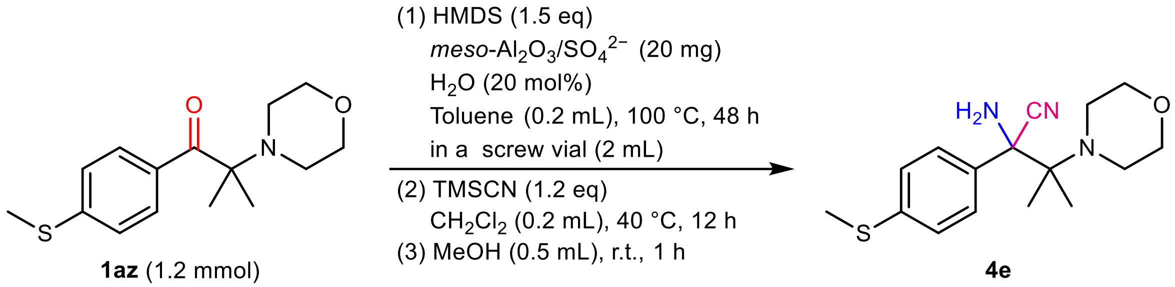

Isolated as a light yellow solid (yield >99%).

^{1}H NMR (400 MHz, CDCl$_3$): δ = 7.61 (d, 2H, J_{HH} = 8.5 Hz), 7.23 (d, 2H, J_{HH} = 8.5 Hz), 3.72 (t, 4H, J_{HH} = 4.6 Hz), 2.91−2.68 (m, 4H), 2.49 (s, 3H), 2.41 (br, 2H), 1.19 (s, 3H), 1.02 (s, 3H) ppm.

^{13}C NMR (100 MHz, CDCl$_3$): δ = 139.35, 134.60, 128.24, 125.33, 125.04, 68.14, 65.70, 49.11, 20.22, 19.77, 15.40 ppm.

ESI-quadrupole-Orbitrap MS (*m/z*): [M+H]$^+$ calcd for $C_{16}H_{24}N_3OS^+$, 306.1635; found 306.1627, [M−CN]$^+$ calcd for $C_{15}H_{23}N_2OS^+$, 279.1526; found 279.1520

2-Amino-2,2-dicyclohexylacetonitrile (4f)

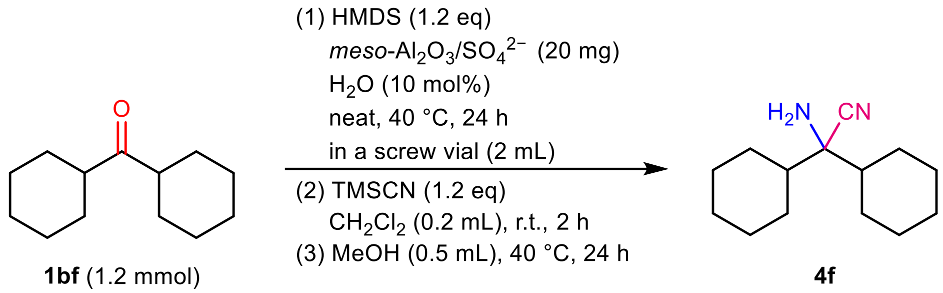

Isolated as a light yellow solid (yield 94%).

^{1}H NMR (400 MHz, CDCl$_3$): δ = 1.96−1.53 (m, 8H), 1.53−1.05 (m, 6H) ppm.

^{13}C NMR (100 MHz, CDCl$_3$): δ = 123.15, 61.26, 42.03, 27.83, 26.25, 26.07, 26.04, 25.94 ppm.

ESI-quadrupole-Orbitrap MS (*m/z*): [M+H]$^+$ calcd for $C_{14}H_{25}N_2^+$, 221.2012; found 221.2006, [M−CN]$^+$ calcd for $C_{13}H_{24}N^+$, 194.1903; found 194.1899.

2-Aminoadamantane-2-carbonitrile (4g)

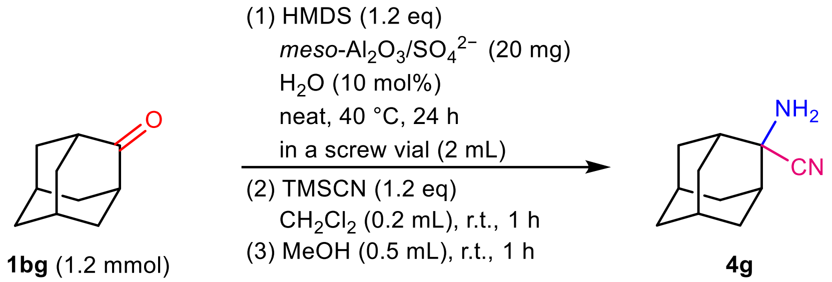

Isolated as a white solid (yield >99%).

^{1}H NMR (400 MHz, $CDCl_3$): δ = 2.33−2.04 (m, 4H), 2.04−1.48 (m, 12H) ppm.

^{13}C NMR (100 MHz, $CDCl_3$): δ = 125.06, 56.35, 37.46, 36.52, 34.91, 30.24, 26.74, 26.39 ppm.

ESI-quadrupole-Orbitrap MS (*m/z*): $[M+H]^+$ calcd for $C_{11}H_{17}N_2^+$, 177.1386; found 177.1383, $[M-CN]^+$ calcd for $C_{10}H_{16}N^+$, 150.1277; found 150.1276.

2-Amino-2-hexyloctanenitrile (4h)

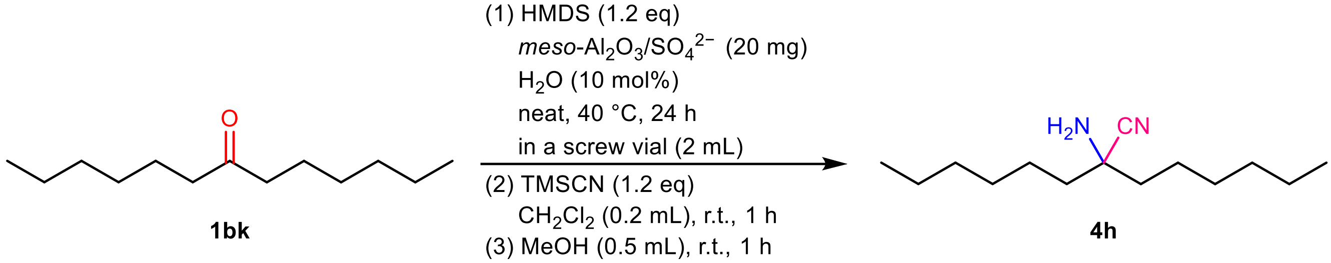

Isolated as a colorless liquid (yield 90%).

^{1}H NMR (400 MHz, $CDCl_3$): δ = 1.84−1.41 (m, 10H), 1.41-1.22 (m, 12H), 0.89 (t, 6H, J_{HH} = 6.8 Hz) ppm.

^{13}C NMR (100 MHz, $CDCl_3$): δ = 123.98, 53.85, 40.24, 31.54, 29.15, 24.12, 22.49, 13.99 ppm.

ESI-quadrupole-Orbitrap MS (*m/z*): $[M+H]^+$ calcd for $C_{14}H_{29}N_2^+$, 225.2325; found 225.2323 $[M-CN]^+$ calcd for $C_{13}H_{28}N^+$, 198.2216; found 198.2216.

2,2'-(1,4-Phenylene)bis(2-amino-2-phenylacetonitrile) (4i)

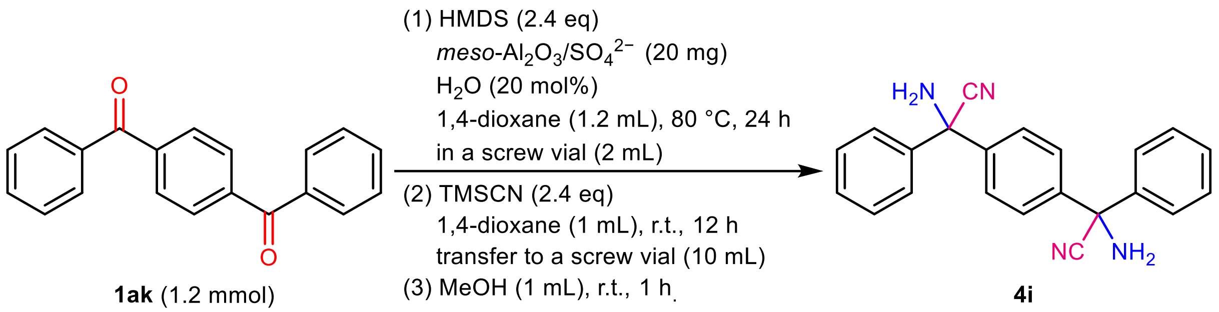

Isolated as a white solid (yield >99%).

^{1}H NMR (400 MHz, $CDCl_3$): δ = 7.68–7.54 (m, 8H), 7.45–7.28 (m, 6H), 2.37 (br, 4H) ppm.

^{13}C NMR (100 MHz, $CDCl_3$): δ = 141.66, 140.65, 128.95, 128.79, 126.33, 125.66, 123.11, 60.46 ppm.

ESI-quadrupole-Orbitrap MS (*m/z*): $[M{-}CN]^+$ calcd for $C_{21}H_{18}N_3^+$, 312.1495; found 312.1491, $[M{-}C_2HN_2]^+$ calcd for $C_{20}H_{17}N^+$, 285.1386; found 285.1384.

2-(Benzylamino)-2,2-diphenylacetonitrile (4j)

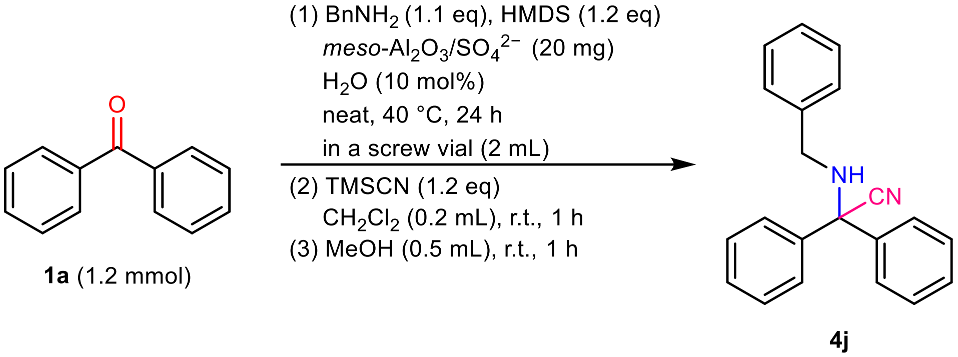

Isolated as a white solid (yield >99%: contains 5% of **4a** as a byproduct).

^{1}H NMR (400 MHz, $CDCl_3$): δ = 7.71 (d, 4H, J_{HH} = 7.7 Hz), 7.46–7.25 (m, 11H), 3.86 (d, 2H, J_{HH} = 7.7 Hz), 2.14 (t, 1H, J_{HH} = 7.7 Hz) ppm.

^{13}C NMR (100 MHz, $CDCl_3$): δ = 140.00, 138.84, 128.95, 128.62, 128.55, 128.27, 127.45, 125.98, 120.30, 68.27, 49.57 ppm.

ESI-quadrupole-Orbitrap MS (*m/z*): $[M{+}H]^+$ calcd for $C_{21}H_{19}N_2^+$, 299.1543; found 299.1538, $[M{-}CN]^+$ calcd for $C_{20}H_{18}N^+$, 272.1434; found 272.1428.

(3*S*,5*S*,8*R*,9*S*,10*S*,13*S*,14*S*,17*S*)-17-amino-10,13-dimethyl-3-((trimethylsilyl)oxy)hexadecahydro-1*H*-cyclopenta[*a*]phenanthrene-17-carbonitrile (**4k**)

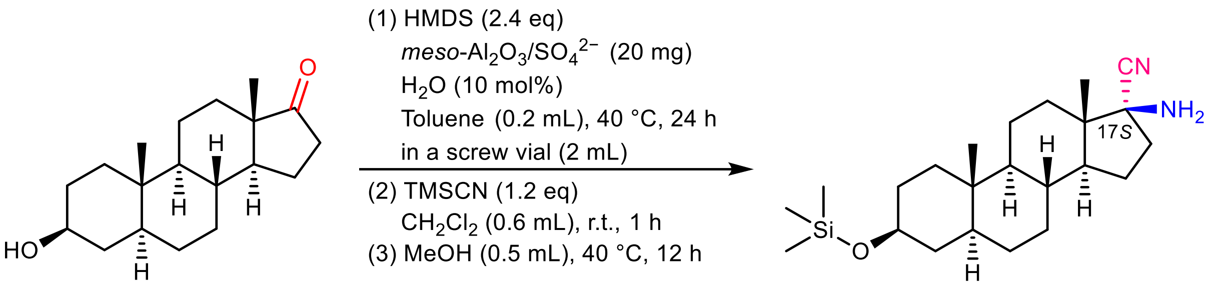

1bl
(Epiandrosterone, 1.2 mmol)

4k

Isolated as a white solid (yield >99%, containing ca. 2% of a compound with a TMS group deprotected. With reference to the configuration of hydantoin compound **5q**, aminonitrile **4k** was determined to have the 17*S* configuration).

^{1}H NMR (400 MHz, $CDCl_3$): δ = 3.61−3.49 (m, 1H), 2.45−2.32 (m, 1H), 1.87−0.62 (m, 29H), 0.11 (s, 9H) ppm.

^{13}C NMR (100 MHz, $CDCl_3$, 17*S* isomer): δ = 124.67, 71.66, 63.38, 53.61, 51.79, 46.84, 44.90, 38.44, 37.45, 37.11, 36.03, 35.46, 33.37, 31.73, 31.66, 28.44, 23.46, 20.77, 12.55, 12.23, 0.23 ppm.

ESI-quadrupole-Orbitrap MS (*m/z*): $[M+H]^+$ calcd for $C_{23}H_{41}N_2OSi^+$, 389.2983; found 389.2979, $[M−CN]^+$ calcd for $C_{22}H_{40}NOSi^+$, 362.2874; found 362.2871.

9.4 Hydantoin compound

5,5-Diphenylimidazolidine-2,4-dione (5a)

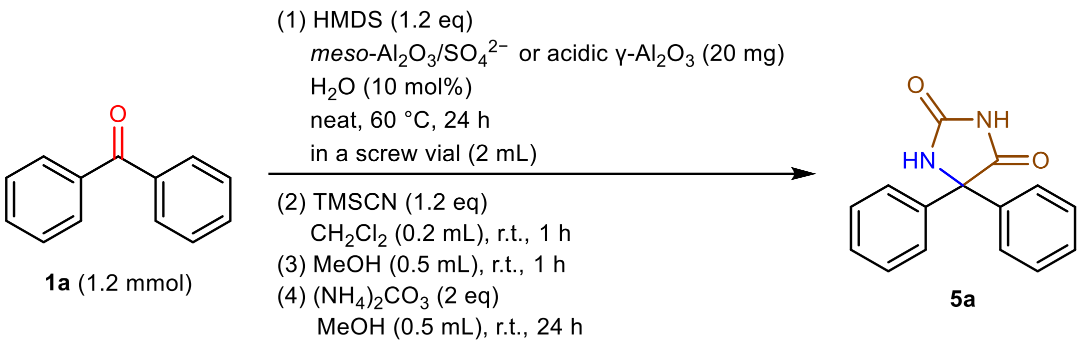

Isolated as a white solid (yield >99%).

^{1}H NMR (400 MHz, DMSO-d_6): δ = 11.09 (br, 1H), 9.30 (br, 1H), 7.45−7.30 (m, 10H) ppm.

^{13}C NMR (100 MHz, DMSO-d_6): δ = 174.81, 155.98, 139.91, 128.50, 128.01, 126.57, 70.20 ppm.

ESI-quadrupole-Orbitrap MS (*m/z*): $[M+H]^+$ calcd for $C_{15}H_{13}N_2O_2^+$, 253.0972; found 253.0966

Methyl 4-(2,5-dioxo-4-phenylimidazolidin-4-yl)benzoate (5b)

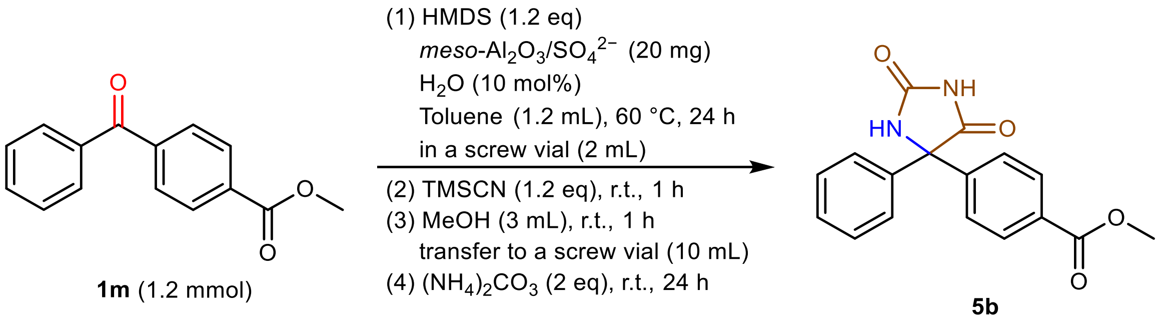

Isolated as a white solid (yield >99%).

^{1}H NMR (400 MHz, DMSO-d_6): δ = 11.19 (br, 1H), 9.40 (br, 1H), 8.00 (d, 2H, J_{HH} = 8.4 Hz), 7.53 (d, 2H, J_{HH} = 8.4 Hz), 8.49−8.29 (m, 5H), 3.85 (s, 3H) ppm.

^{13}C NMR (100 MHz, DMSO-d_6): δ = 174.43, 165.83, 156.06, 144.79, 139.52, 129.42, 129.30, 128.70, 128.27, 127.07, 126.54, 70.18, 52.24 ppm.

ESI-quadrupole-Orbitrap MS (*m/z*): $[M+H]^+$ calcd for $C_{17}H_{15}N_2O_4^+$, 311.1026; found 311.1024.

Isopropyl 2-(4-(4-(4-chlorophenyl)-2,5-dioxoimidazolidin-4-yl)phenoxy)-2-methylpropanoate (5c)

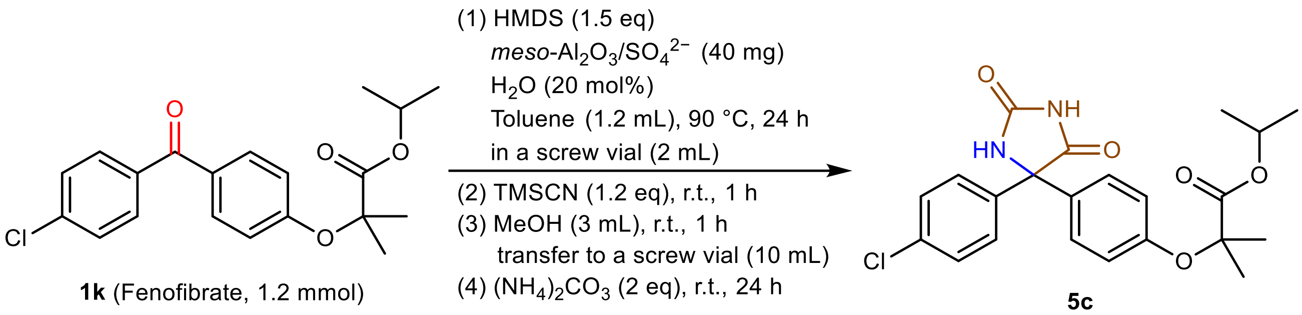

Isolated as a white solid (yield >99%).

^{1}H NMR (400 MHz, DMSO-d_6): δ = 11.11 (br, 1H), 9.26 (br, 1H), 7.47 (d, 2H, J_{HH} = 8.7 Hz), 7.37 (d, 2H, J_{HH} = 8.7 Hz), 7.19 (d, 2H, J_{HH} = 8.9 Hz), 6.79 (d, 2H, J_{HH} = 8.9 Hz), 4.94 (sep, 1H, J_{HH} = 6.3 Hz), 1.51 (s, 6H), 1.14 (d, 2H, J_{HH} = 6.3 Hz) ppm.

^{13}C NMR (100 MHz, DMSO-d_6): δ = 174.72, 172.36, 155.85, 154.95, 138.80, 132.85, 132.73, 128.48, 128.46, 127.60, 118.12, 78.69, 69.29, 68.62, 25.02, 25.01, 21.21 ppm.

ESI-quadrupole-Orbitrap MS (*m/z*): $[M+H]^+$ calcd for $C_{22}H_{24}ClN_2O_5^+$, 431.1368; found 431.1369.

5-Phenyl-5-(pyridin-4-yl)imidazolidine-2,4-dione (5d)

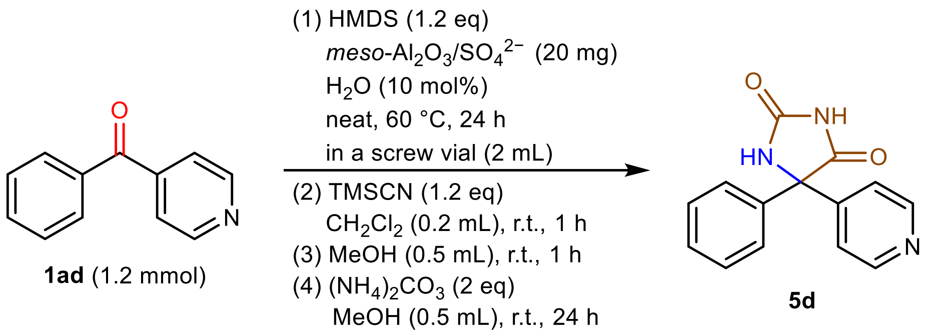

Isolated as a white solid (yield >99%).

^{1}H NMR (400 MHz, DMSO-d_6): δ = 11.27 (br, 1H), 9.46 (br, 1H), 8.61 (d, 2H, J_{HH} = 6.0 Hz), 7.48−7.28 (m, 7H) ppm.

^{13}C NMR (100 MHz, DMSO-d_6): δ = 173.79, 155.94, 150.02, 148.04, 138.82, 128.78, 128.40, 126.38, 121.39, 69.42 ppm.

ESI-quadrupole-Orbitrap MS (*m/z*): $[M+H]^+$ calcd for $C_{14}H_{12}N_3O_2^+$, 254.0924; found 254.0923.

5-Phenyl-5-(thiophen-2-yl)imidazolidine-2,4-dione (5e)

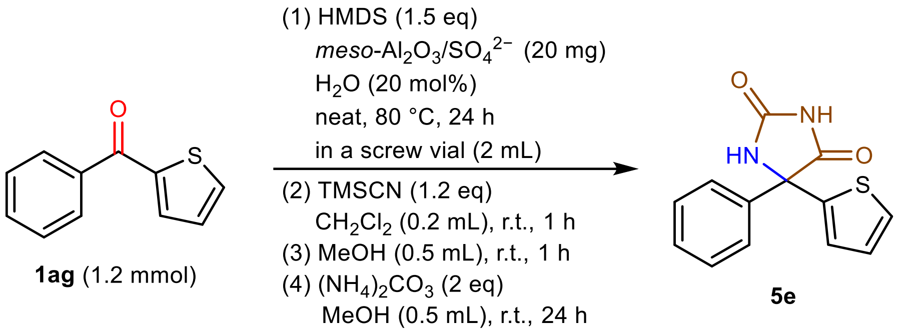

Isolated as a white solid (yield >99%).

^{1}H NMR (400 MHz, DMSO-d_6): δ = 11.17 (br, 1H), 9.49 (br, 1H), 7.60−7.32 (m, 6H), 7.09-6.96 (m, 2H) ppm.

^{13}C NMR (100 MHz, DMSO-d_6): δ = 174.10, 155.76, 143.38, 139.26, 128.51, 128.41, 126.98, 126.75, 126.26, 126.05 67.83 ppm.

ESI-quadrupole-Orbitrap MS (*m/z*): $[M+H]^+$ calcd for $C_{13}H_{11}N_2O_2S^+$, 259.0536; found 259.0537.

Spiro[imidazolidine-4,3'-indoline]-2,2',5-trione (5f)

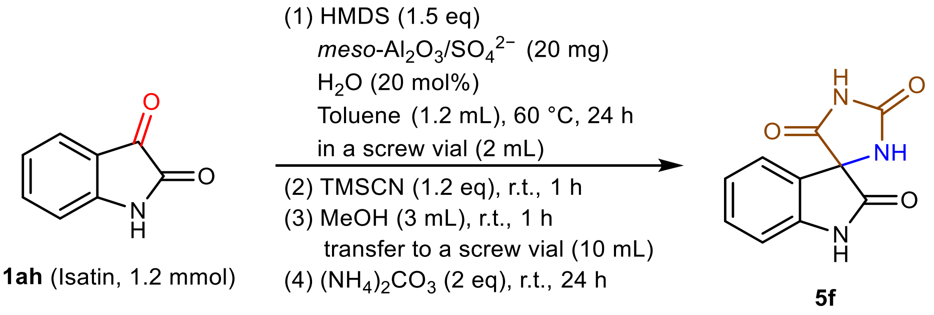

Isolated as a light pink solid (yield 96%).

^{1}H NMR (400 MHz, DMSO-d_6): δ = 11.27 (br, 1H), 10.86 (br, 1H), 8.58 (br, 1H), 7.36−7.28 (m, 2H), 7.07−7.01 (m, 1H), 6.92 (d, 1H, J_{HH} = 7.7 Hz) ppm.

^{13}C NMR (100 MHz, DMSO-d_6): δ = 172.45, 170.78, 157.70, 142.97, 130.59, 125.46, 124.31, 122.58, 110.45, 69.54 ppm.

ESI-quadrupole-Orbitrap MS (*m/z*): $[M+H]^+$ calcd for $C_{11}H_{13}N_2O_3^+$, 221.0921; found 221.0920.

5-Ethyl-5-(4-hydroxyphenyl)imidazolidine-2,4-dione (5g)

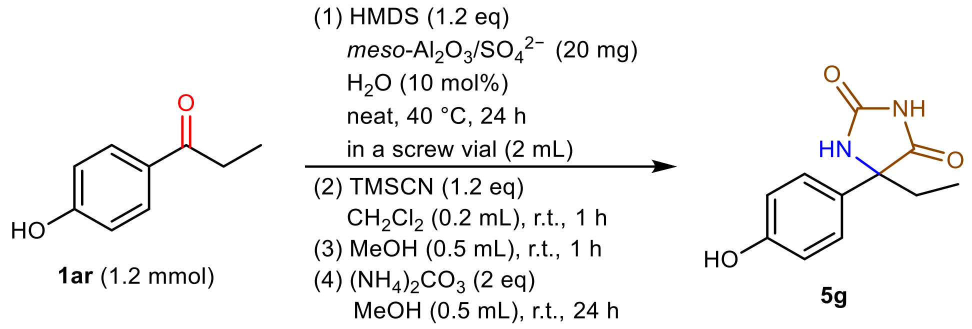

Isolated as a white solid (yield 96%).

^{1}H NMR (400 MHz, DMSO-d_6): δ = 10.62 (br, 1H), 9.46 (br, 1H), 8.48 (br, 1H), 7.26 (d, 2H, J_{HH} = 8.7 Hz), 6.75 (d, 2H, J_{HH} = 8.7 Hz) 2.07−1.76 (m, 2H), 0.78 (t, 3H, J_{HH} = 7.5 Hz) ppm.

^{13}C NMR (100 MHz, DMSO-d_6): δ = 176.69, 156.92, 156.67, 129.29, 126.57, 115.07, 67.57, 30.85, 7.99 ppm.

ESI-quadrupole-Orbitrap MS, positive (*m/z*): $[M+H]^+$ calcd for $C_{11}H_{13}N_2O_3^+$, 221.0921; found 221.0920.

5-Ethyl-5-(3-nitrophenyl)imidazolidine-2,4-dione (5h)

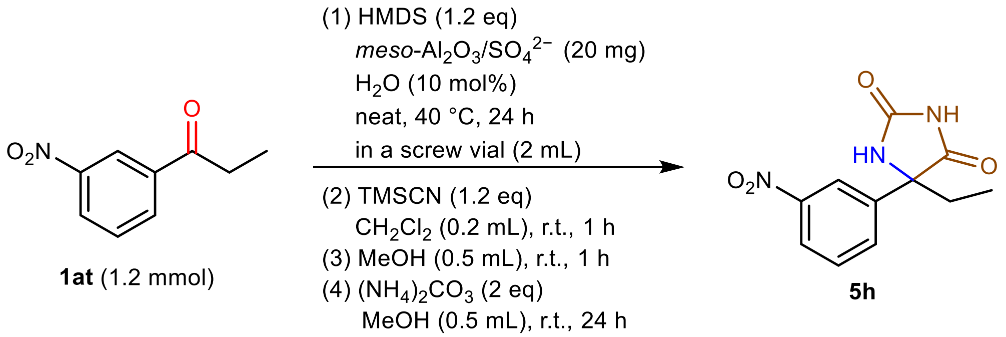

Isolated as a white solid (yield >99%).

^{1}H NMR (400 MHz, DMSO-d_6): δ = 10.98 (br, 1H), 8.89 (br, 1H), 8.34 (s, 1H), 8.01 (d, 1H, J_{HH} = 8.0 Hz), 8.00 (d, 1H, J_{HH} = 8.0 Hz), 7.73 (t, 1H, J_{HH} = 8.0 Hz), 2.17−1.86 (m, 2H), 0.82 (t, 3H, J_{HH} = 7.3 Hz) ppm.

^{13}C NMR (100 MHz, DMSO-d_6): δ = 175.54, 156.47, 147.84, 141.24, 132.30, 130.19, 122.88, 120.11, 67.72, 31.78, 7.93 ppm.

ESI-quadrupole-Orbitrap MS (*m/z*): $[M-H]^-$ calcd for $C_{11}H_{10}N_3O_4^-$, 248.0677; found 248.0675.

5-Isopropyl-5-phenylimidazolidine-2,4-dione (5i)

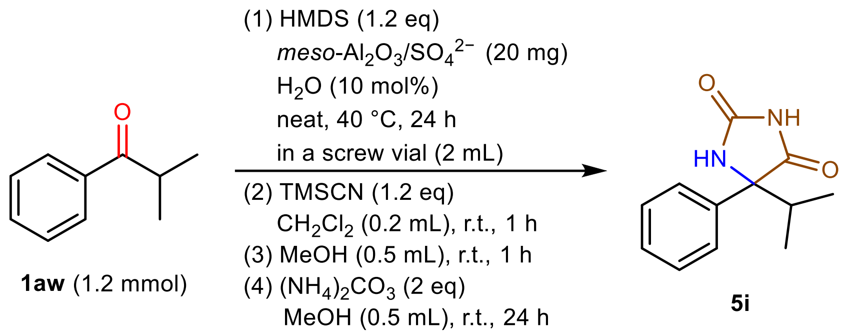

Isolated as a white solid (yield >99%).

^{1}H NMR (400 MHz, DMSO-d_6): δ = 10.72 (br, 1H), 8.69 (br, 1H), 7.55−7.49 (m, 2H), 7.43−7.35 (m, 2H), 7.35−7.28 (m, 1H), 2.46 (sep, 1H, J_{HH} = 6.8 Hz), 0.89 (d, 3H, J_{HH} = 6.8 Hz), 0.62 (d, 3H, J_{HH} = 6.8 Hz) ppm.

^{13}C NMR (100 MHz, DMSO-d_6): δ = 176.29, 156.99, 138.63, 128.32, 127.64, 125.43, 71.34, 35.04, 16.58, 16.22 ppm.

ESI-quadrupole-Orbitrap MS (*m/z*): $[M+H]^+$ calcd for $C_{12}H_{15}N_2O_2^+$, 219.1128; found 219.1129.

5,5-Diisopropylimidazolidine-2,4-dione (5j)

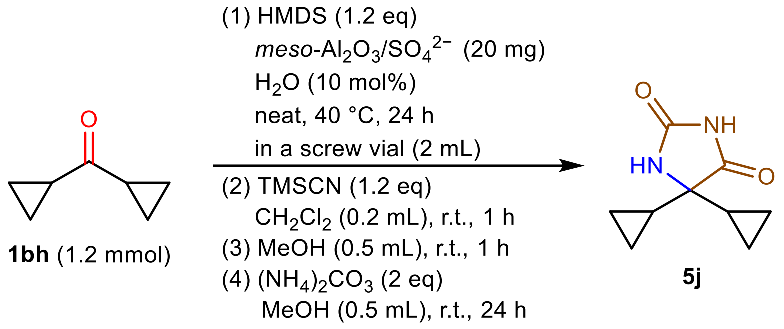

Isolated as a white solid (yield 99%).

^{1}H NMR (400 MHz, DMSO-d_6): δ = 10.45 (br, 1H), 7.52 (br, 1H), 1.21−1.12 (m, 2H), 0.49−0.10 (m, 8H) ppm.

^{13}C NMR (100 MHz, DMSO-d_6): δ = 177.42, 157.05, 63.66, 15.87, 0.32, −1.12 ppm.

ESI-quadrupole-Orbitrap MS (*m/z*): $[M+H]^+$ calcd for $C_9H_{13}N_2O_2^+$, 181.0972; found 181.0970.

5,5-Dicyclopropylimidazolidine-2,4-dione (5k)

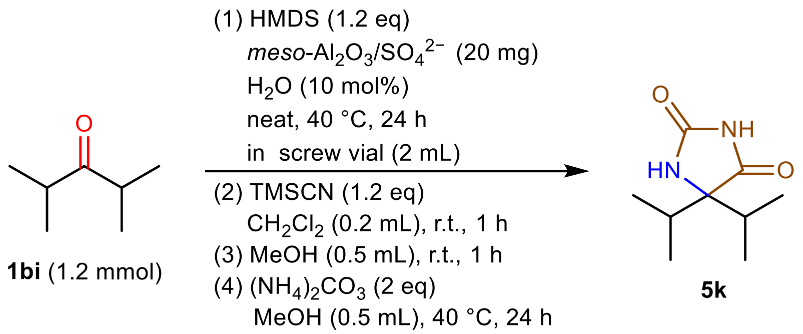

Isolated as a white solid (yield 98%).

^{1}H NMR (400 MHz, DMSO-d_6): δ = 10.48 (br, 1H), 7.74 (br, 1H), 2.00 (sep, 2H, J_{HH} = 6.8 Hz), 0.87 (d, 6H, J_{HH} = 6.8 Hz), 0.84 (d, 6H, J_{HH} = 6.8 Hz) ppm.

^{13}C NMR (100 MHz, DMSO-d_6): δ = 177.29, 157.92, 70.98, 30.61, 16.33, 16.26 ppm.

ESI-quadrupole-Orbitrap MS (*m/z*): [M+H]$^+$ calcd for $C_9H_{17}N_2O_2^+$, 185.1285; found 185.1283.

5-(*tert*-Butyl)-5-methylimidazolidine-2,4-dione (5l)

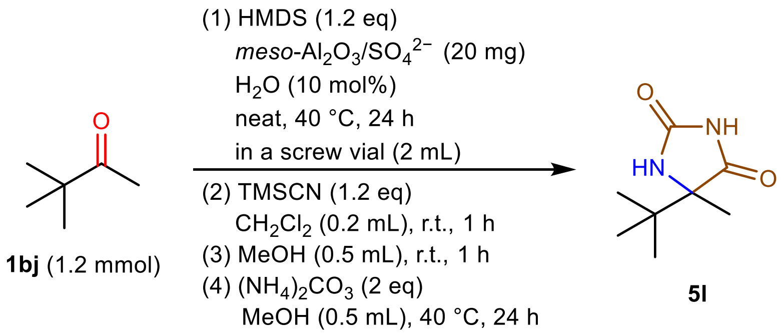

Isolated as a white solid (yield 92%).

^{1}H NMR (400 MHz, DMSO-d_6): δ = 10.49 (br, 1H), 7.95 (br, 1H), 1.24 (s, 3H), 0.92 (s, 9H) ppm.

^{13}C NMR (100 MHz, DMSO-d_6): δ = 178.18, 156.68, 66.75, 36.11, 24.53, 18.82 ppm.

ESI-quadrupole-Orbitrap MS (*m/z*): [M+H]$^+$ calcd for $C_8H_{15}N_2O_2^+$, 171.1128; found 171.1127.

5,5-Diethylimidazolidine-2,4-dione (5m)

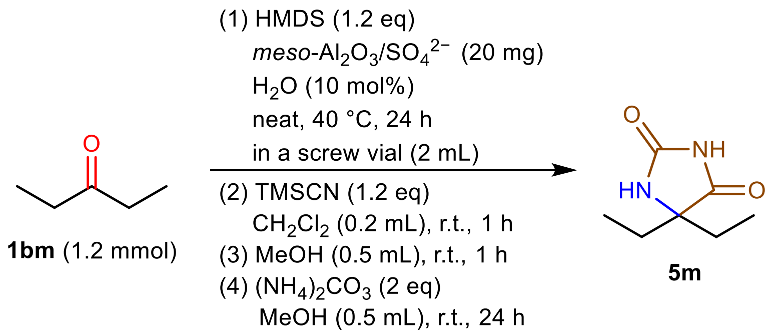

Isolated as a white solid (yield 94%).

^{1}H NMR (400 MHz, DMSO-d_6): δ = 10.55 (br, 1H), 7.76 (br, 1H), 1.66−1.45 (m, 4H), 0.77 (t, 6H, J_{HH} = 7.5 Hz) ppm.

^{13}C NMR (100 MHz, DMSO-d_6): δ = 177.87, 157.09, 66.52, 29.05, 7.55 ppm.

ESI-quadrupole-Orbitrap MS (*m/z*): [M+H]$^+$ calcd for $C_7H_{13}N_2O_2^+$, 157.0972; found 157.0970.

1,3-Diazaspiro[4.7]dodecane-2,4-dione (5n)

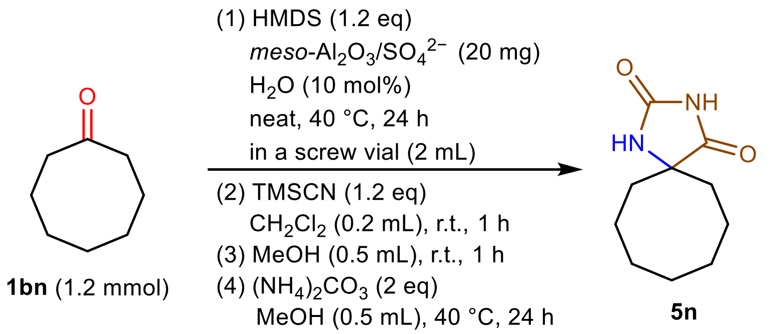

Isolated as a white solid (yield 97%).

^{1}H NMR (400 MHz, DMSO-d_6): δ = 10.41 (br, 1H), 8.16 (br, 1H), 2.90−1.74 (m, 2H), 1.74−1.36 (m, 12H) ppm.

^{13}C NMR (100 MHz, DMSO-d_6): δ = 179.18, 156.24, 63.85, 32.03, 27.50, 23.87, 21.21 ppm.

ESI-quadrupole-Orbitrap MS (*m/z*): [M+H]$^+$ calcd for $C_{10}H_{17}N_2O_2^+$, 197.1285; found 197.1289.

5,5'-(1,4-Phenylene)bis(5-phenylimidazolidine-2,4-dione) (5o)

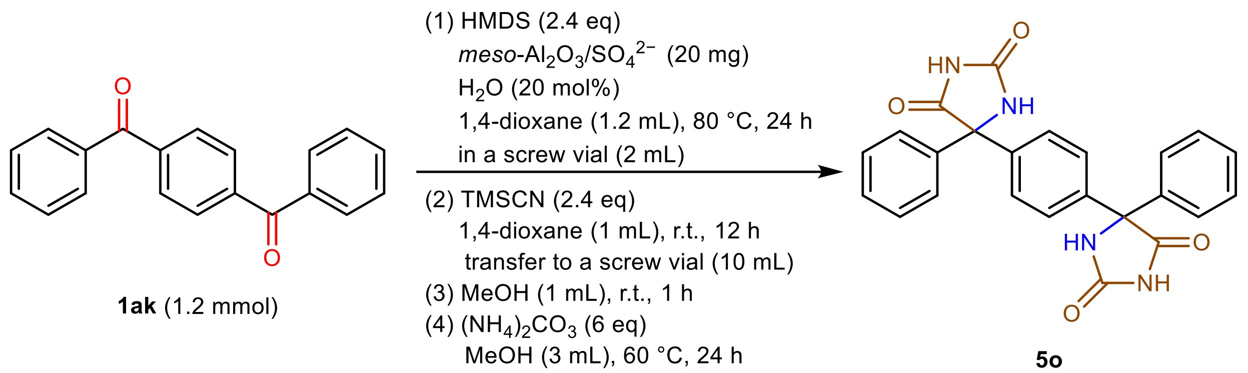

As workup, the catalyst was removed by filtration with DMF, and the low boiling point components were removed by concentration. The resulting solids were purified by reprecipitation in water: Isolated as a white solid (yield >99%).

^{1}H NMR (400 MHz, DMSO-*d*$_6$): δ = 11.12 (br, 2H), 9.31 (br, 2H), 7.43−7.31 (m, 14H) ppm.

^{13}C NMR (100 MHz, DMSO-*d*$_6$, Two types of diastereomer): δ = 174.61 (2C), 155.89 (2C), 139.69, 139.67, 139.64, 128.57 (2C), 128.10 (2C), 128.77 (2C), 126.51 (2C), 69.95 (2C) ppm.

ESI-quadrupole-Orbitrap MS (*m/z*): [M−H]$^-$ calcd for $C_{24}H_{17}N_4O_4^-$, 425.1255; found 425.1261.

5,5'-(1,3-Phenylene)bis(5-(4-bromophenyl)imidazolidine-2,4-dione) (5p)

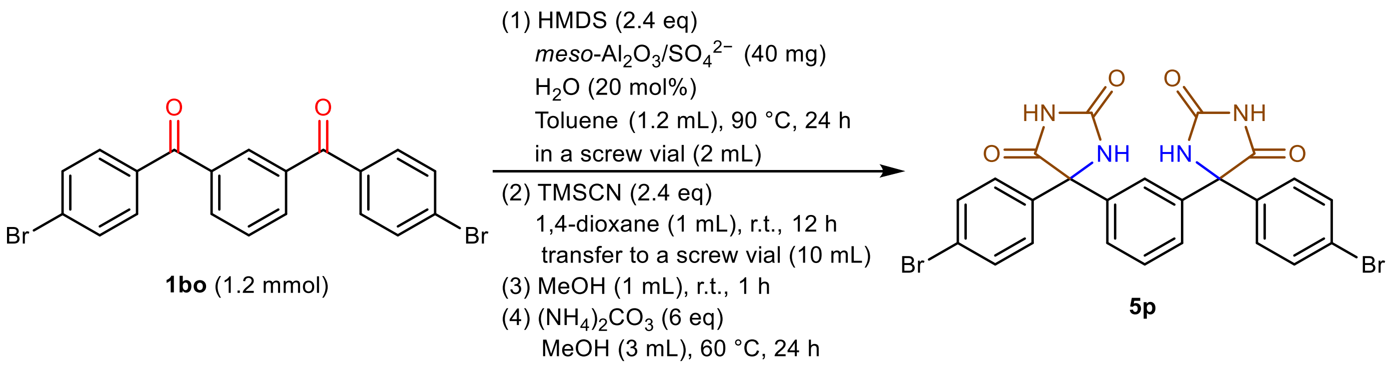

As workup, the catalyst was removed by filtration with DMF, and the low boiling point components were removed by concentration. The resulting solids were purified by reprecipitation in water: Isolated as a white solid (yield >99%).

^{1}H NMR (400 MHz, DMSO-*d*$_6$): δ = 11.18 (br, 2H), 9.34* (br, 1H), 9.38* (br, 1H), 7.60 (d, 4H, J_{HH} = 8.5 Hz), 7.51−7.39 (m, 2H), 7.34−7.16 (m, 6H) ppm. *Different peaks of diastereomer.

^{13}C NMR (100 MHz, DMSO-*d*$_6$, two type peaks of diastereomer): δ = 174.29, 174.27, 155.85, 155.85, 139.94, 139.76, 138.91, 138.82, 131.49, 131.48, 128.96 (2C), 128.82, 126.89, 126.81, 124.58, 124.31, 121.63, 121.61, 69.81, 69.77 ppm.

ESI-quadrupole-Orbitrap MS (*m/z*): [M−H]$^-$ calcd for $C_{24}H_{15}{}^{79}Br^{81}BrN_4O_4^-$, 582.9445; found 582.9451.

(3*S*,5*S*,8*R*,9*S*,10*S*,13*S*,14*S*,17*S*)-3-Hydroxy-10,13-dimethylhexadecahydrospiro[cyclopenta[*a*]phenanthrene-17,4'-imidazolidine]-2',5'-dione (5q)

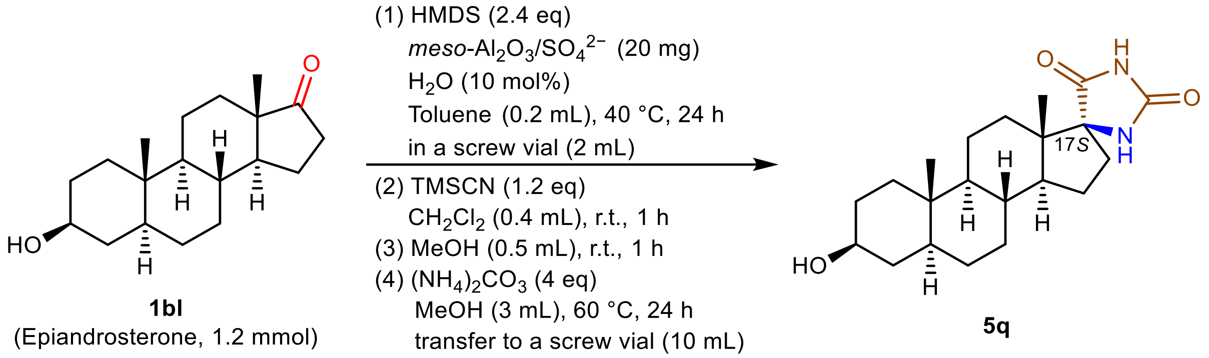

As workup, the catalyst was removed by filtration with DMF, and the low boiling point components were removed by concentration. The resulting solids were purified by reprecipitation in water: Isolated as a white solid (yield 95%, d.r. = 90:10). The diastereomers were unable to be separated by HPLC due to their low solubility in organic solvents. For example, **5q** could hardly be soluble in MeOH and DMF.

^{1}H NMR (400 MHz, DMSO-d_6, 17*S*+17*R* isomers): δ = 10.42 (br, 1H, 17*S*+17*R*), 8.02 (br, 0.1H, 17*R*), 7.83 (br, 0.9H, 17*S*), 4.41 (d, 1H, J_{HH} = 4.7 Hz, 17*S*+17*R*), 3.37−3.28 (m, 1H, 17*S*+17*R*), 2.03−0.49 (m, 28H, 17*S*+17*R*) ppm. Isomers were assigned by ROESY measurements.

^{13}C NMR (100 MHz, DMSO-d_6, 17*S* isomer): δ = 178.65, 156.47, 73.23, 69.23, 53.46, 48.27, 46.57, 44.31, 38.11, 36.63, 35.24, 35.12, 31.64, 31.51, 31.32, 31.19, 28.29, 23.43, 20.18, 15.08, 12.07 ppm.

ESI-quadrupole-Orbitrap MS (*m/z*): [M−H]$^-$ calcd for $C_{21}H_{31}N_2O_3^-$, 359.2340; found 359.2345.

(8*R*,9*S*,13*S*,14*S*,17*S*)-3-hydroxy-13-methyl-6,7,8,9,11,12,13,14,15,16-decahydrospiro[cyclopenta[*a*]phenanthrene-17,4'-imidazolidine]-2',5'-dione (5r)

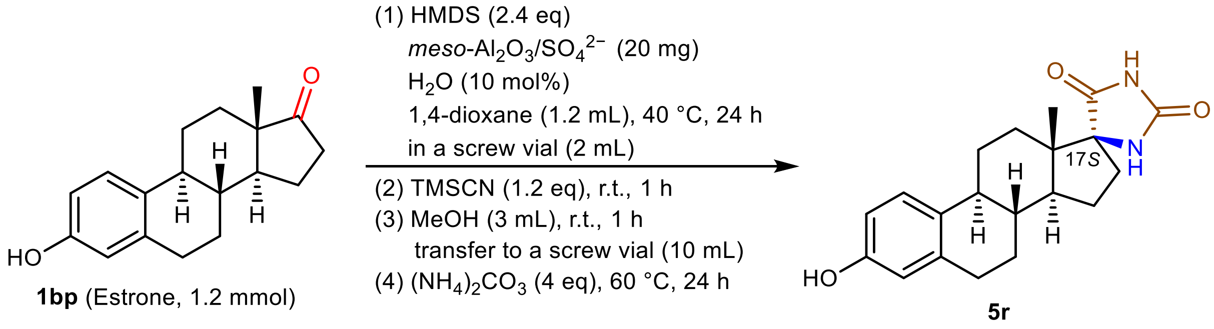

As workup, the catalyst was removed by filtration with DMF, and the low boiling point components were removed by concentration. The resulting solids were purified by reprecipitation in water: Isolated as a white solid (yield 93%, d.r. = 88:12). The diastereomers were unable to be separated by HPLC due to their low solubility in organic solvents. For example, **5r** could hardly be soluble in MeOH.

^{1}H NMR (400 MHz, DMSO-d_6, 17*S*+17*R* isomers): δ = 10.47 (br, 1H, 17*S*+17*R*), 8.96 (br, 1H, 17*S*+17*R*), 8.11 (br, 0.13H, 17*R*), 7.90 (br, 0.87H, 17*S*), 7.07−6.98 (m, 1H, 17*S*+17*R*), 6.54−6.36 (m, 2H, 17*S*+17*R*), 2.84−2.63 (m, 2H, 17*S*+17*R*), 2.37−1.08 (m, 13H, 17*S*+17*R*), 0.87 (s, 0.39H, 17*R*), 0.80 (s, 2.61H, 17*S*) ppm. The isomers were assigned by ROESY measurements.

^{13}C NMR (100 MHz, DMSO-d_6, 17*S* isomer): δ = 178.69, 156.51, 154.98, 154.97, 137.12, 130.08, 126.04, 114.91, 112.70, 73.28, 47.32, 46.80, 43.05, 31.74, 31.29, 29.13, 27.18, 25.65, 23.11, 15.06 ppm.

ESI-quadrupole-Orbitrap MS (*m/z*): $[M-H]^-$ calcd for $C_{20}H_{23}N_2O_3^-$, 339.1714; found 339.1716.

(8*R*,9*S*,13*S*,14*S*,17*S*)-3-(Benzyloxy)-13-methyl-4-nitro-6,7,8,9,11,12,13,14,15,16-decahydrospiro[cyclopenta[*a*]phenanthrene-17,4'-imidazolidine]-2',5'-dione (5s)

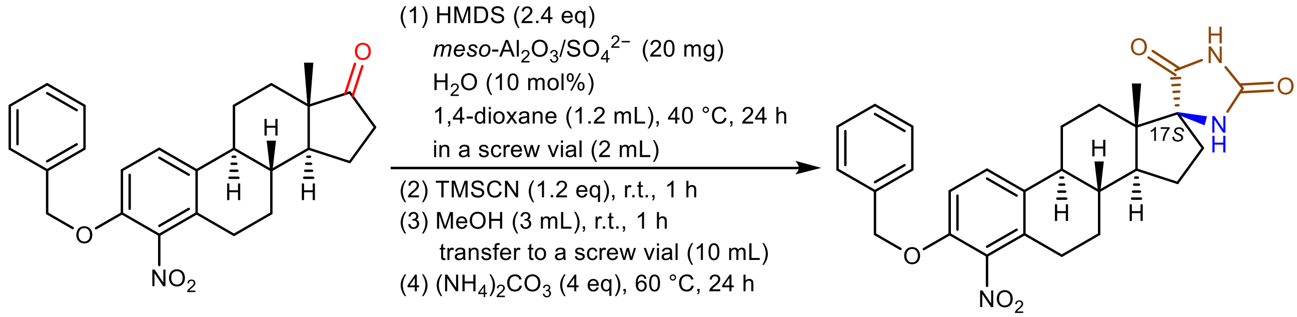

1bq (1.2 mmol)

5s

As workup, the catalyst was removed by filtration with DMF, and the low boiling point components were removed by concentration. The resulting solids were purified by reprecipitation in water: Isolated as a white solid (yield 93%, d.r. = 88:12). The diastereomers were unable to be separated by HPLC due to their low solubility in organic solvents. For example, **5s** could hardly be soluble in MeOH.

^{1}H NMR (400 MHz, DMSO-d_6, 17*S*+17*R* isomers): δ = 10.51 (br, 1H, 17*S*+17*R*), 8.12 (br, 0.12H, 17*R*), 7.91 (br, 0.88H, 17*S*), 7.49−7.25 (m, 6H, 17*S*+17*R*), 7.25−7.11 (m, 1H, 17*S*+17*R*), 5.23 (s, 2H, 17*S*+17*R*), 2.78−2.55 (m, 2H, 17*S*+17*R*), 2.44−1.25 (m, 13H, 17*S*+17*R*), 0.87 (s, 0.36H, 17*R*), 0.80 (s, 2.64H, 17*S*) ppm. The isomers were assigned by ROESY measurements.

^{13}C NMR (100 MHz, DMSO-d_6, 17*S* isomer): δ = 178.64, 156.50, 146.67, 141.33, 136.14, 133.60, 128.51, 128.16, 128.05, 127.34, 111.96, 73.20, 70.00, 47.13, 46.64, 42.80, 37.51, 31.72, 31.13, 25.75, 25.44, 23.47, 22.99, 14.98 ppm.

ESI-quadrupole-Orbitrap MS (*m/z*): $[M-H]^-$ calcd for $C_{27}H_{28}N_3O_5^-$, 474.2034; found 474.2039.

(8*R*,9*S*,13*S*,14*S*,17*S*)-2-Bromo-3-hydroxy-13-methyl-4-nitro-6,7,8,9,11,12,13,14,15,16-decahydrospiro[cyclopenta[*a*]phenanthrene-17,4'-imidazolidine]-2',5'-dione (5t)

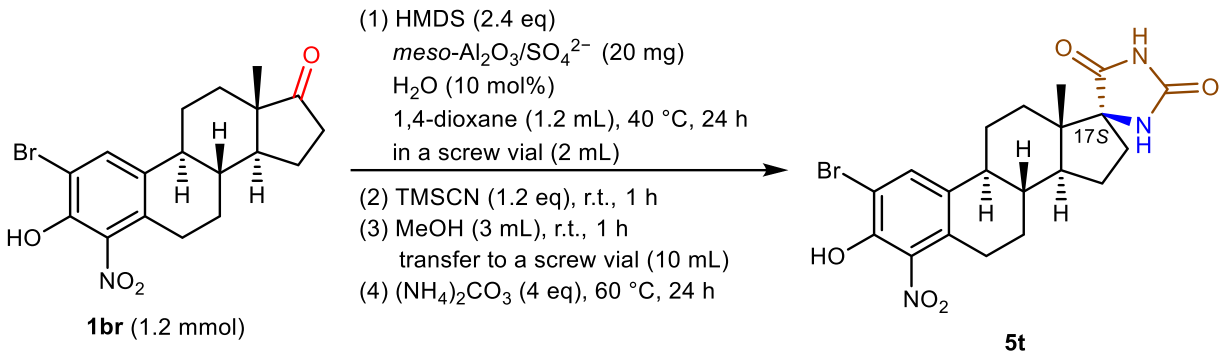

As workup, the catalyst was removed by filtration with DMF, and the low boiling point components were removed by concentration. The resulting solids were purified by reprecipitation in water: Isolated as a yellow solid (yield 98%, d.r. = 86:14). The diastereomers were unable to be separated by HPLC due to their low solubility in organic solvents. For example, **5t** could hardly be soluble in MeOH.

^{1}H NMR (400 MHz, DMSO-d_6, 17*S*+17*R* isomers): δ = 10.78 (br, 1H, 17*S*+17*R*), 10.51 (br, 1H, 17*S*+17*R*), 8.14 (br, 0.14H, 17*R*), 7.92 (br, 0.86H, 17*S*), 7.88 (s, 0.14H, 17*R*), 7.86 (s, 0.86H, 17*S*). 3.03−3.26 (m, 2H, 17*S*+17*R*), 2.42−1.13 (m, 13H, 17*S*+17*R*), 2.78−2.55 (m, 2H, 17*S*+17*R*), 2.44−1.25 (m, 13H, 17*S*+17*R*), 0.86 (s, 0.42H, 17*R*), 0.80 (s, 2.58H, 17*S*) ppm. The isomers were assigned by ROESY measurements.

^{13}C NMR (100 MHz, DMSO-d_6, 17*S* isomer): δ = 178.63, 156.51, 147.52, 145.76, 133.91, 133.58, 120.23, 116.16, 73.17, 47.21, 46.56, 42.59, 37.05, 31.71, 31.68, 30.96, 26.68, 25.42, 23.00, 14.90 ppm.

ESI-quadrupole-Orbitrap MS (*m/z*): $[M−H]^−$ calcd for $C_{20}H_{21}BrN_3O_5^−$, 462.0670; found 462.0678.

10. Spectral data for NMR, FT-IR, and HRMS

All spectral data were obtained after the experiments in Sections 3–6, without any additional purification procedures.

10.1 *N*–H Ketimine and *N*–H ketimine hydrochloride

^{1}H NMR (400 MHz, DMSO-d_6) and ^{13}C NMR (100 MHz, DMSO-d_6) spectra for **2a**

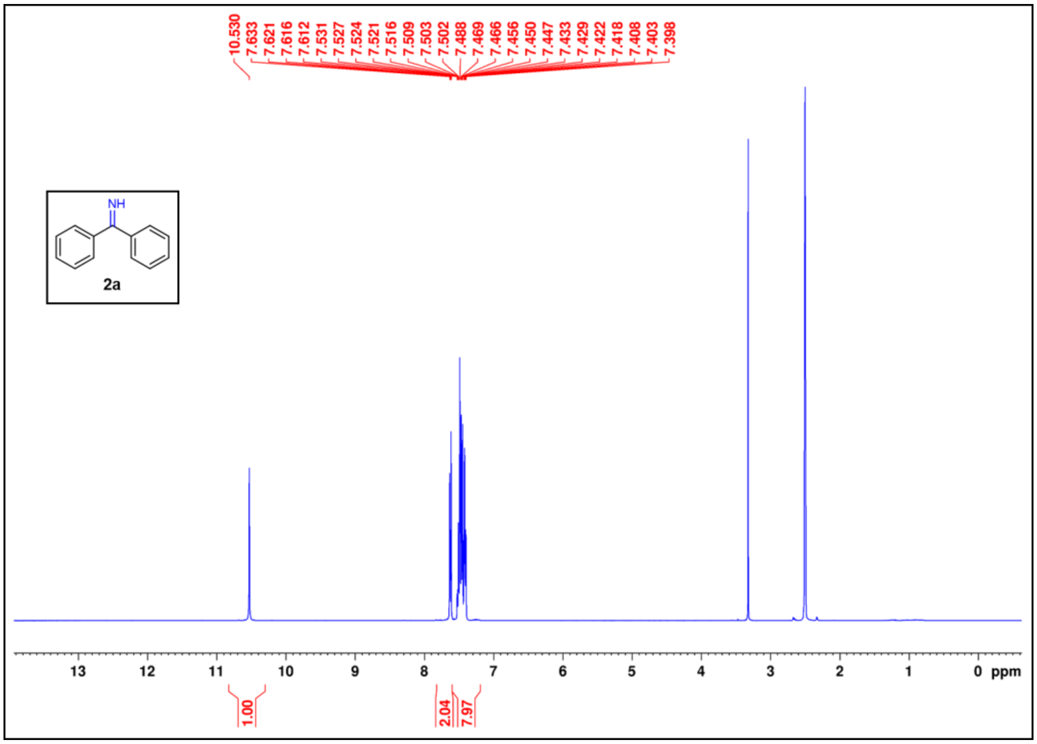

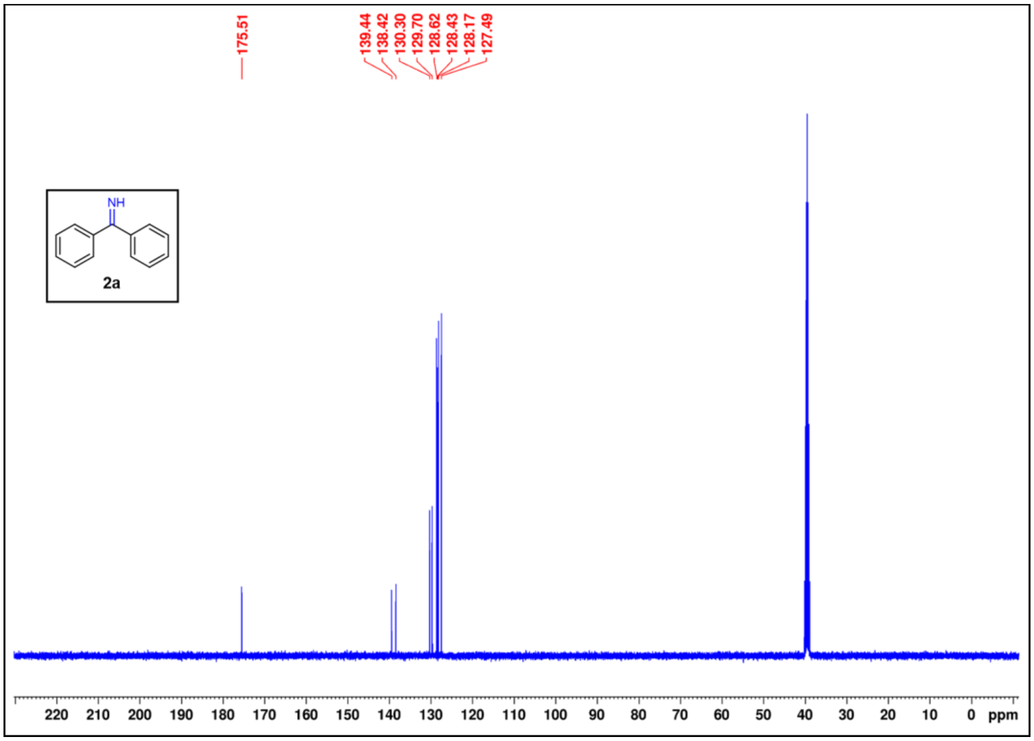

FT-IR (ATR, neat) and HRMS (ESI-positive) spectra for **2a**

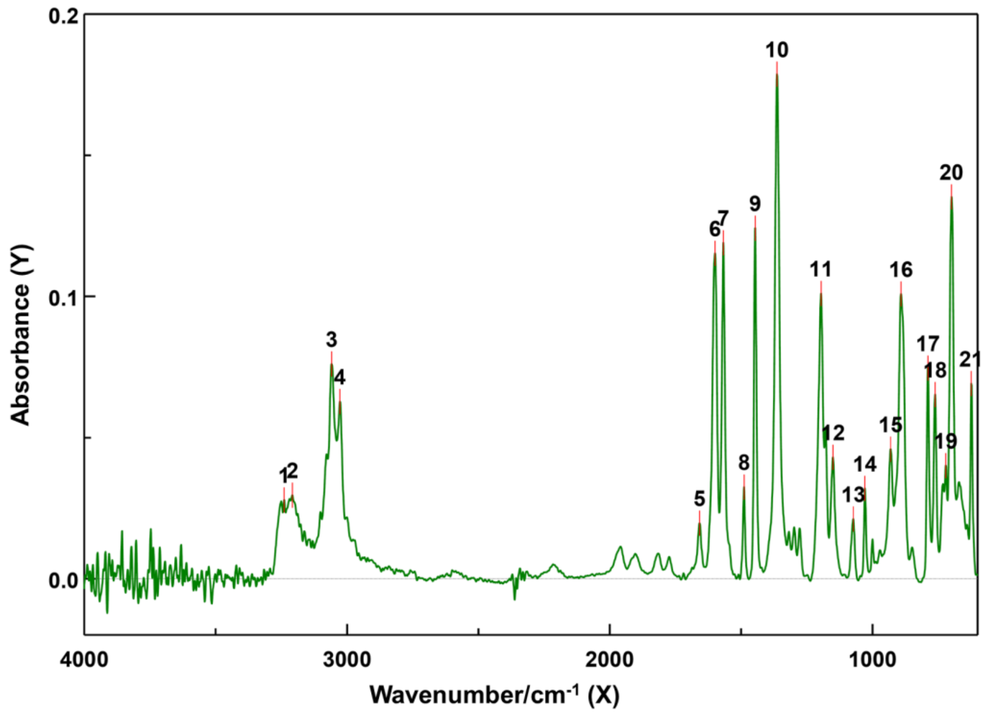

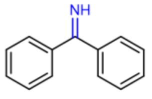

2a

No.	X	Y	No.	X	Y	No.	X	Y
1	3239.8	0.028	8	1488.8	0.032	15	931.5	0.046
2	3208.0	0.030	9	1446.4	0.124	16	891.9	0.101
3	3058.5	0.076	10	1363.4	0.179	17	788.7	0.075
4	3026.7	0.063	11	1195.7	0.101	18	761.7	0.065
5	1658.5	0.020	12	1150.3	0.043	19	721.2	0.040
6	1599.7	0.115	13	1073.2	0.021	20	699.1	0.135
7	1567.8	0.119	14	1028.8	0.032	21	623.9	0.069

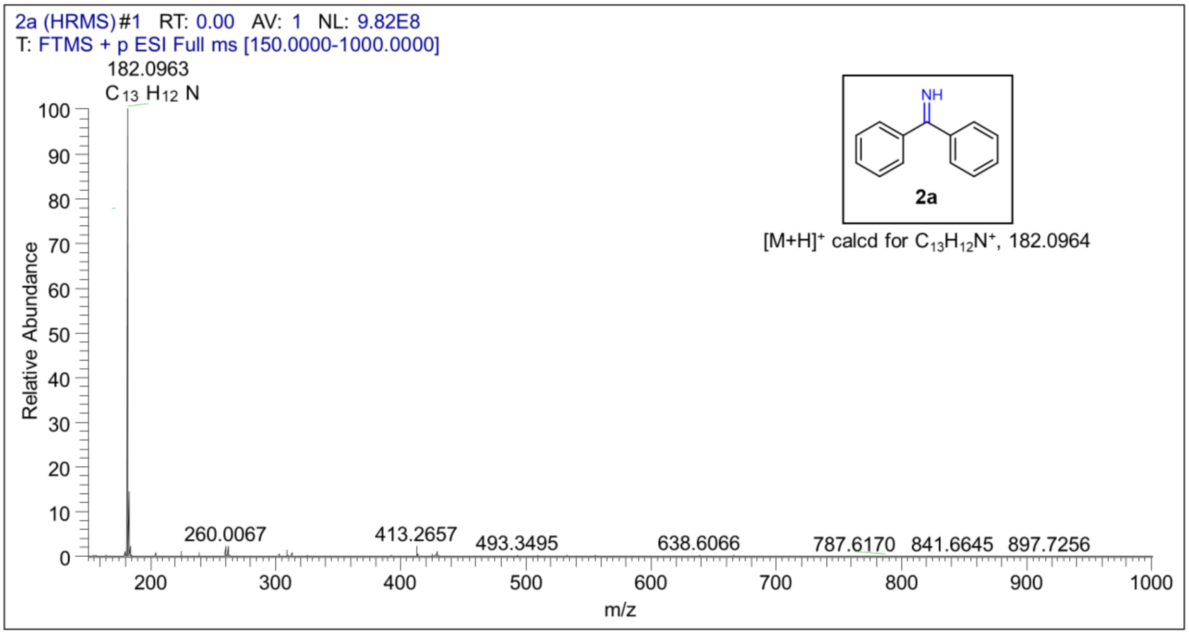

[M+H]+ calcd for $C_{13}H_{12}N^+$, 182.0964

^{1}H NMR (400 MHz, DMSO-d_6) and ^{13}C NMR (100 MHz, DMSO-d_6) spectra for **2a**·HCl

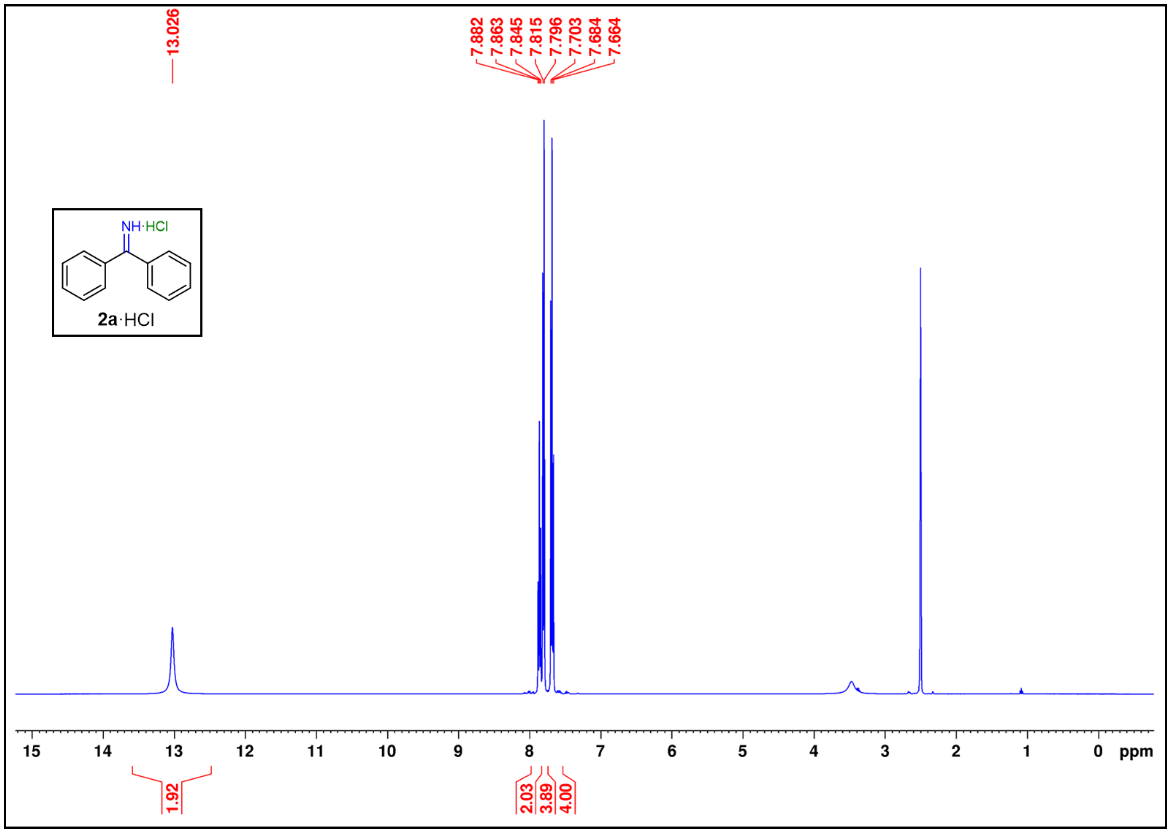

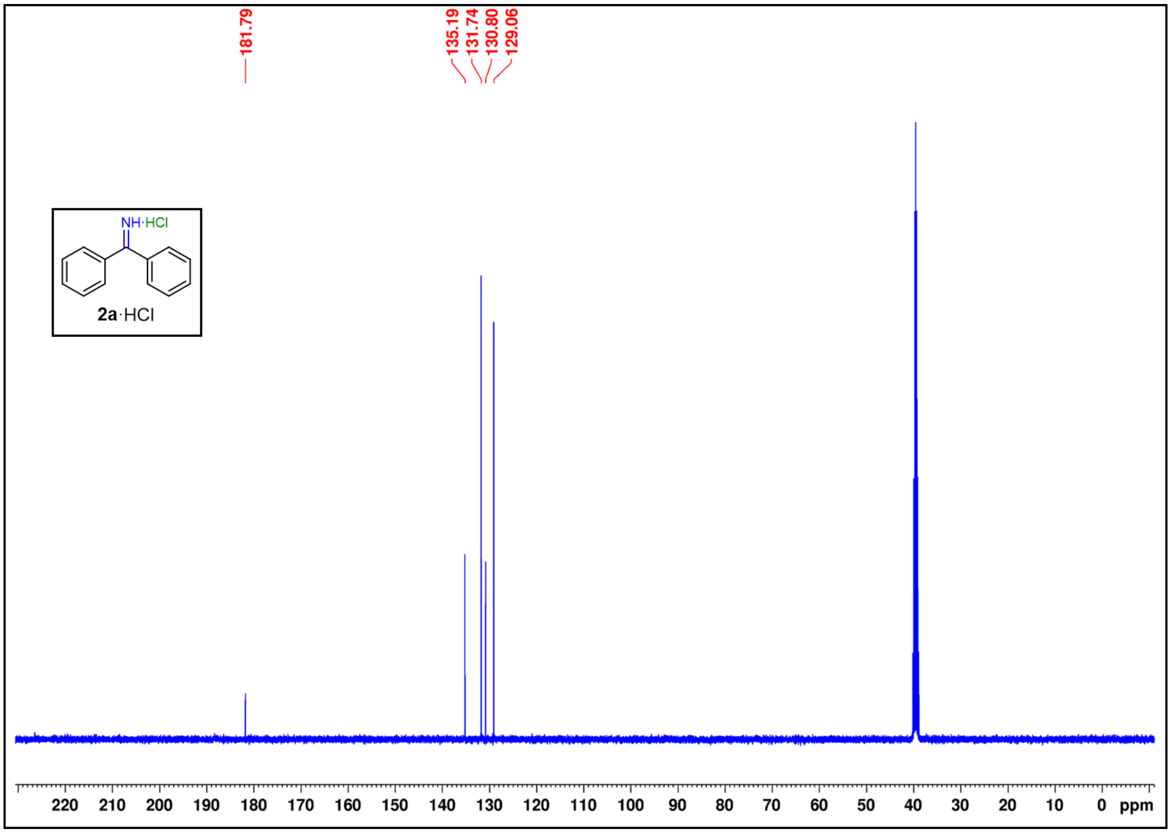

FT-IR (ATR, neat) and HRMS (ESI-positive) spectra for **2a**·HCl

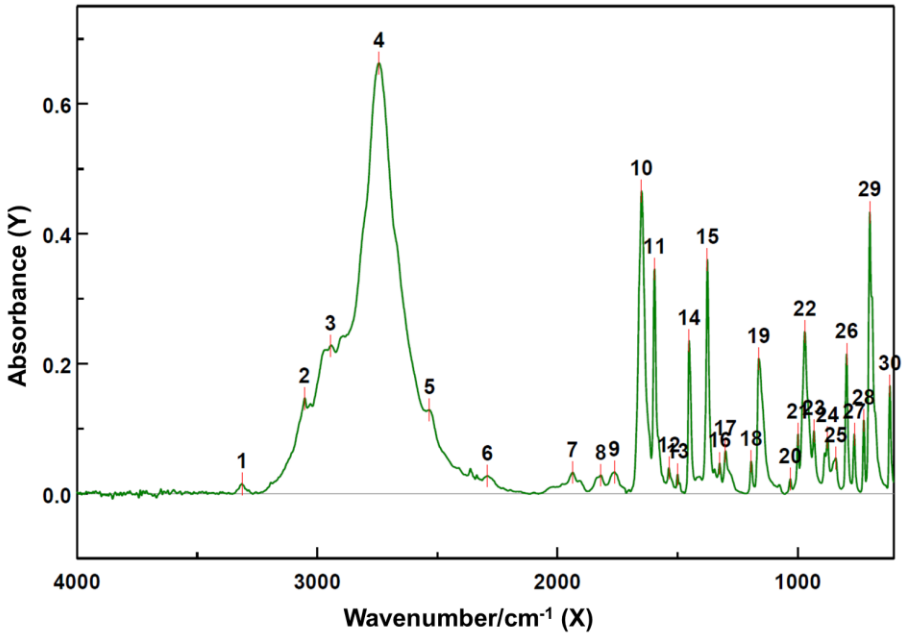

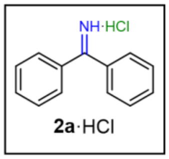

No.	X	Y	No.	X	Y	No.	X	Y
1	3313.1	0.015	2	3052.8	0.146	3	2945	0.227
4	2743.2	0.662	5	2533	0.129	6	2292	0.027
7	1937.1	0.032	8	1819.5	0.028	9	1764	0.033
10	1649.8	0.466	11	1595.8	0.345	12	1536	0.039
13	1500.3	0.029	14	1452.1	0.235	15	1376	0.36
16	1324.9	0.046	17	1300.8	0.066	18	1194	0.049
19	1161.9	0.207	20	1030.8	0.022	21	998.9	0.091
22	970	0.249	23	932.4	0.096	24	875.5	0.086
25	841.8	0.054	26	796.5	0.215	27	764.6	0.091
28	725.1	0.113	29	700	0.433	30	616.1	0.166

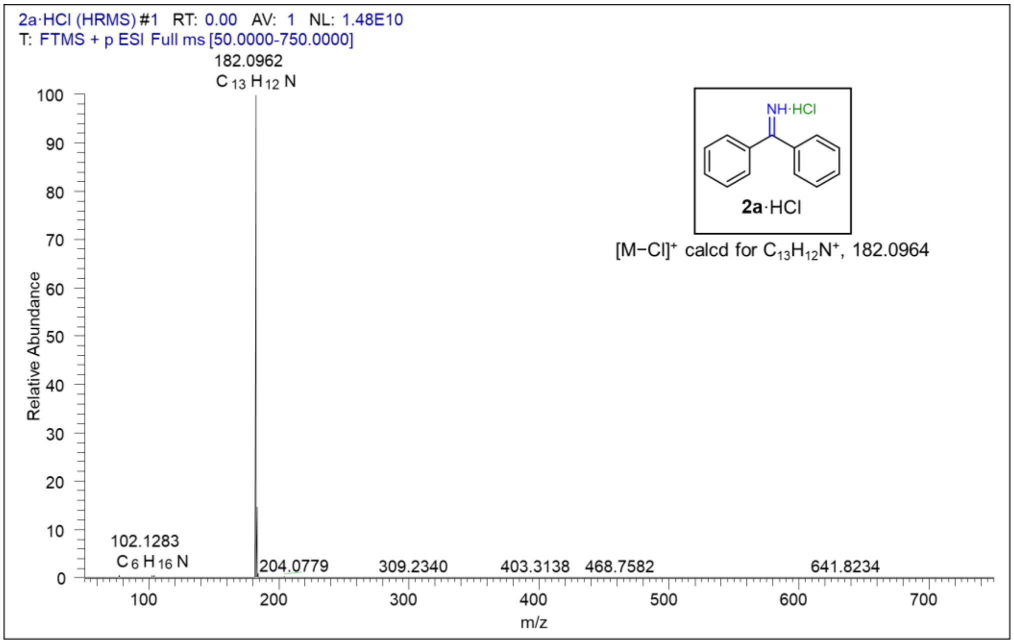

^{1}H NMR (400 MHz, DMSO-d_6) and ^{13}C NMR (100 MHz, DMSO-d_6) spectra for **2b**

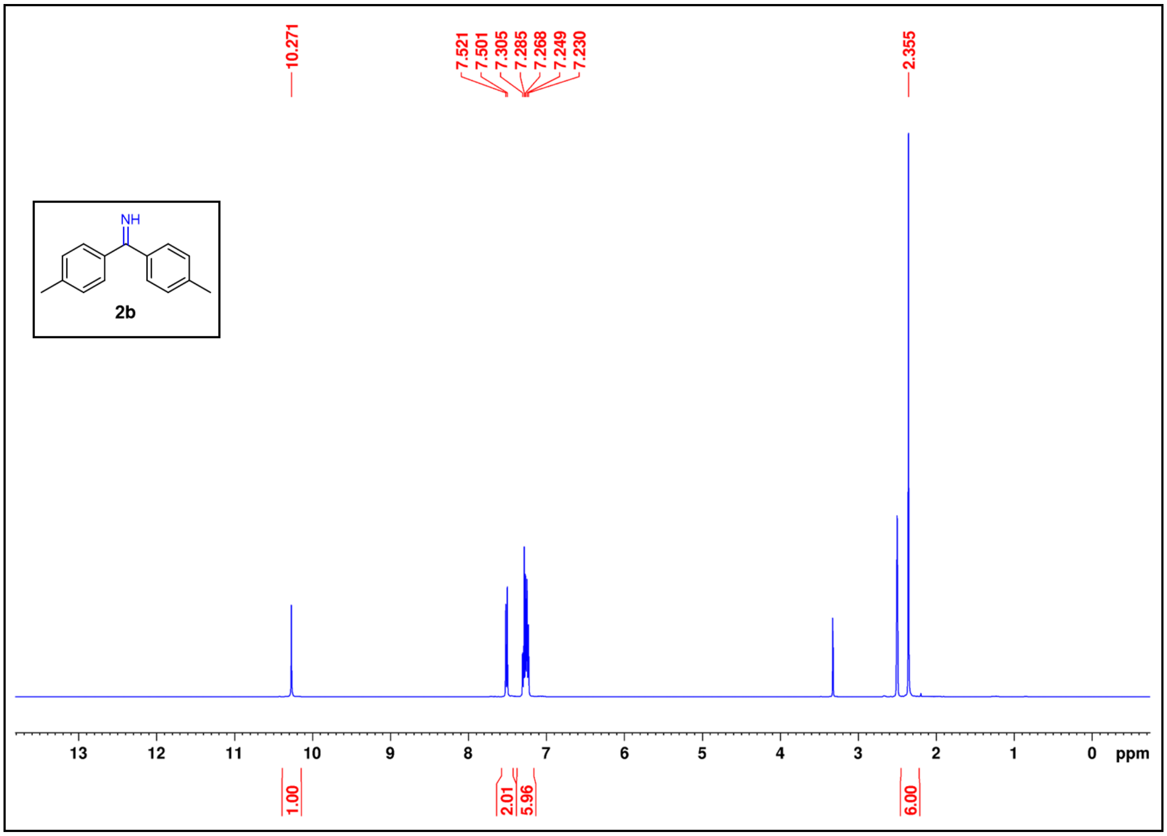

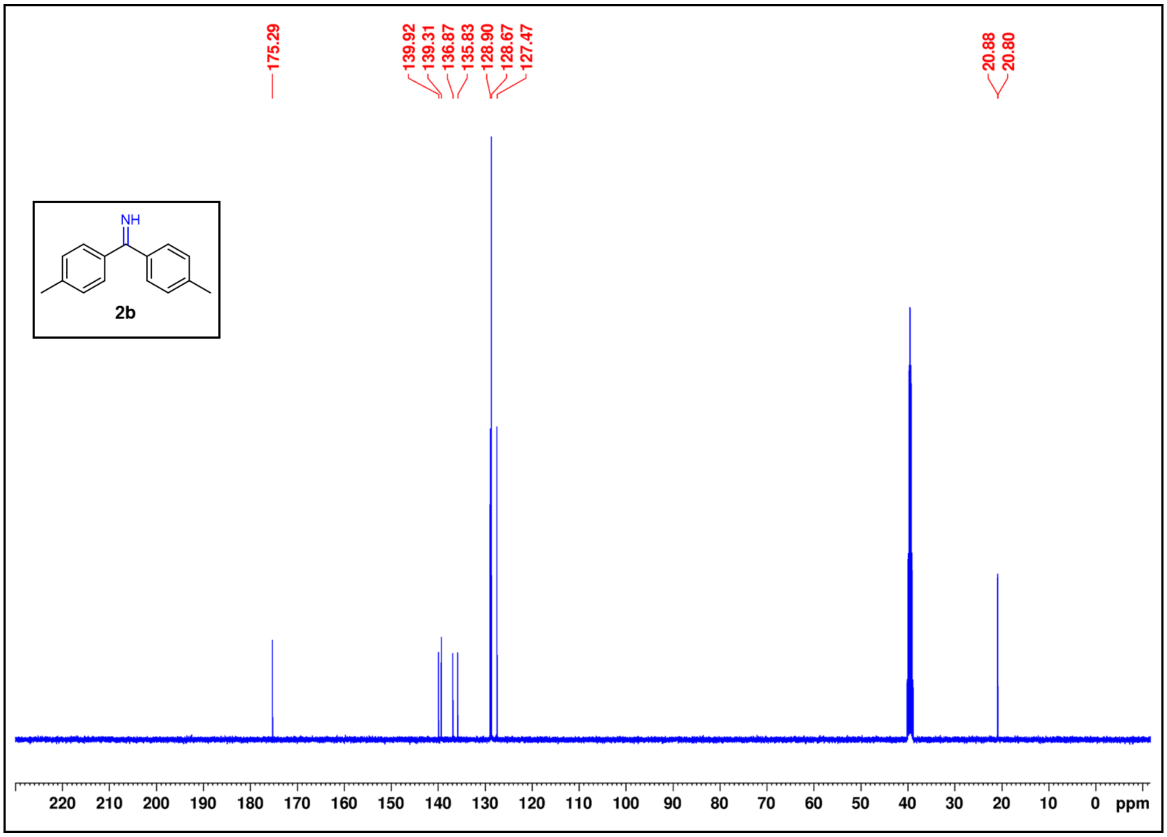

FT-IR (ATR, neat) and HRMS (ESI-positive) spectra for **2b**

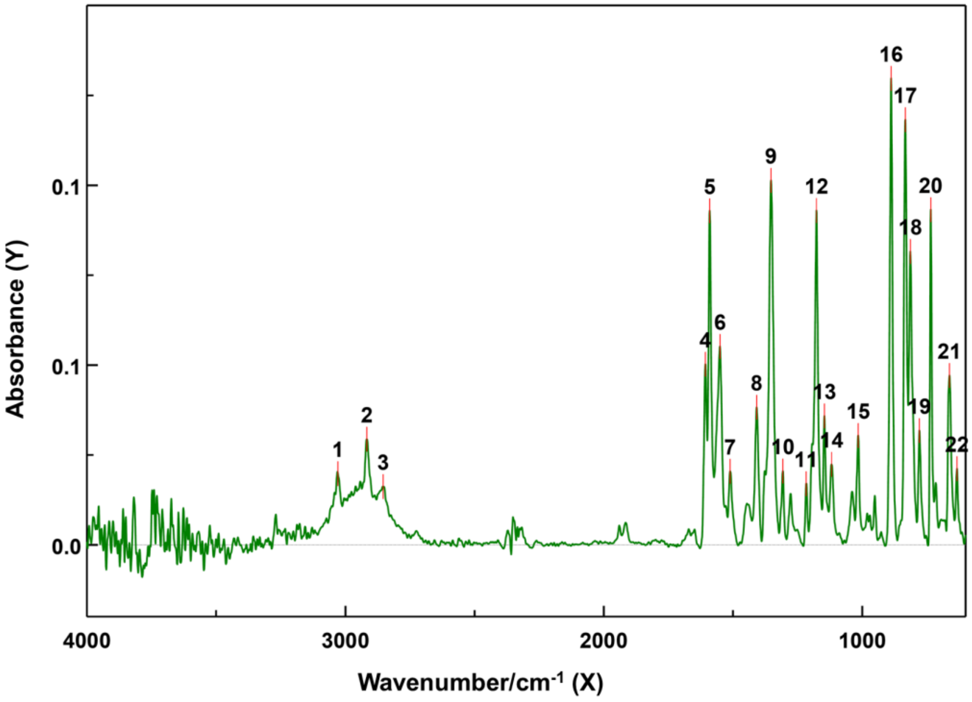

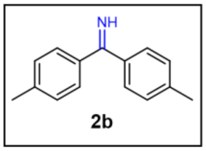

No.	X	Y	No.	X	Y	No.	X	Y
1	3028.7	0.02	9	1352.8	0.101	17	833.1	0.118
2	2916.8	0.029	10	1307.5	0.02	18	813.8	0.082
3	2854.1	0.016	11	1216.9	0.017	19	779.1	0.032
4	1607.4	0.05	12	1177.3	0.093	20	734.7	0.093
5	1590	0.093	13	1146.5	0.036	21	662.4	0.047
6	1550.5	0.055	14	1118.5	0.022	22	633.5	0.021
7	1511	0.02	15	1015.3	0.03			
8	1408.7	0.038	16	888.1	0.13			

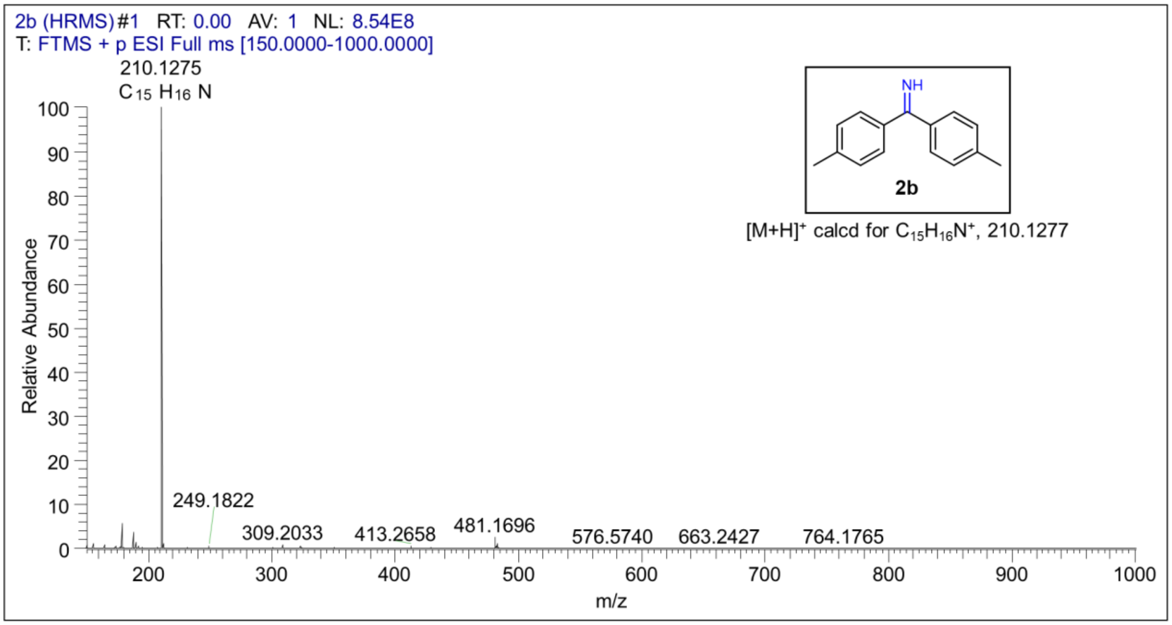

^{1}H NMR (400 MHz, DMSO-d_6) and ^{13}C NMR (100 MHz, DMSO-d_6) spectra for **2c**

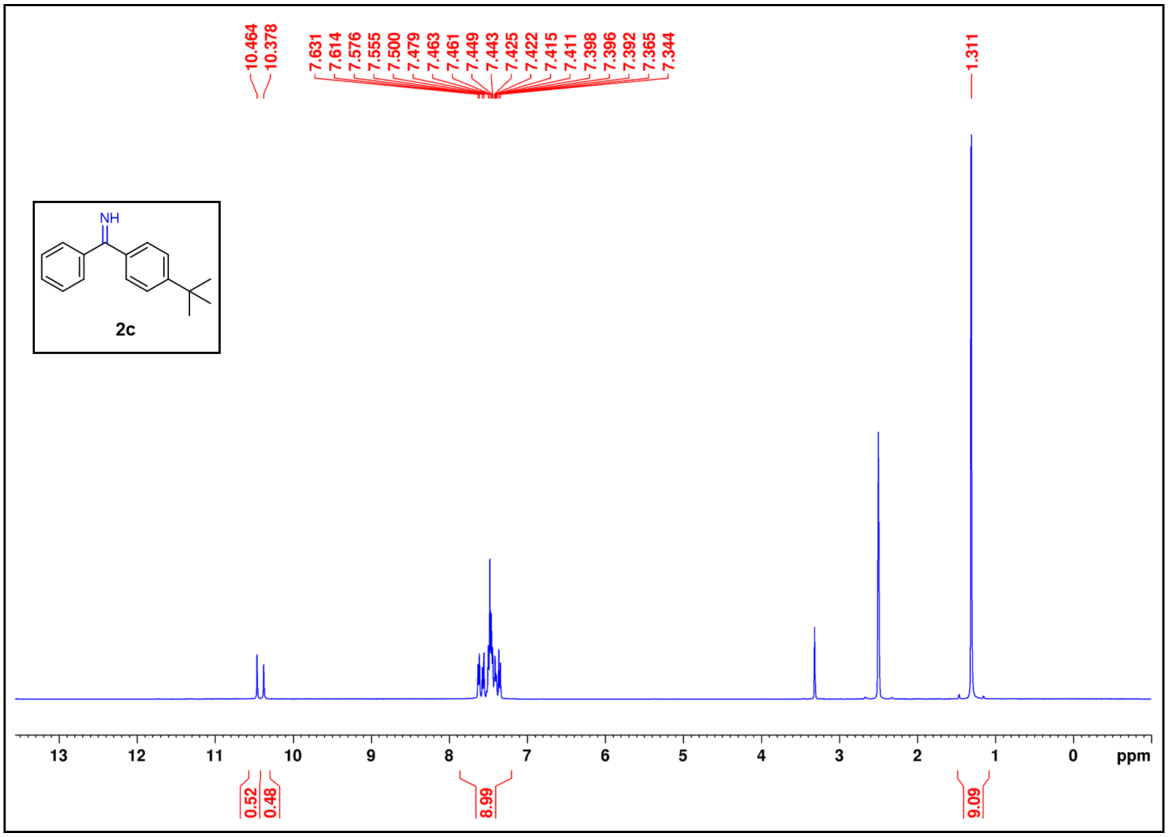

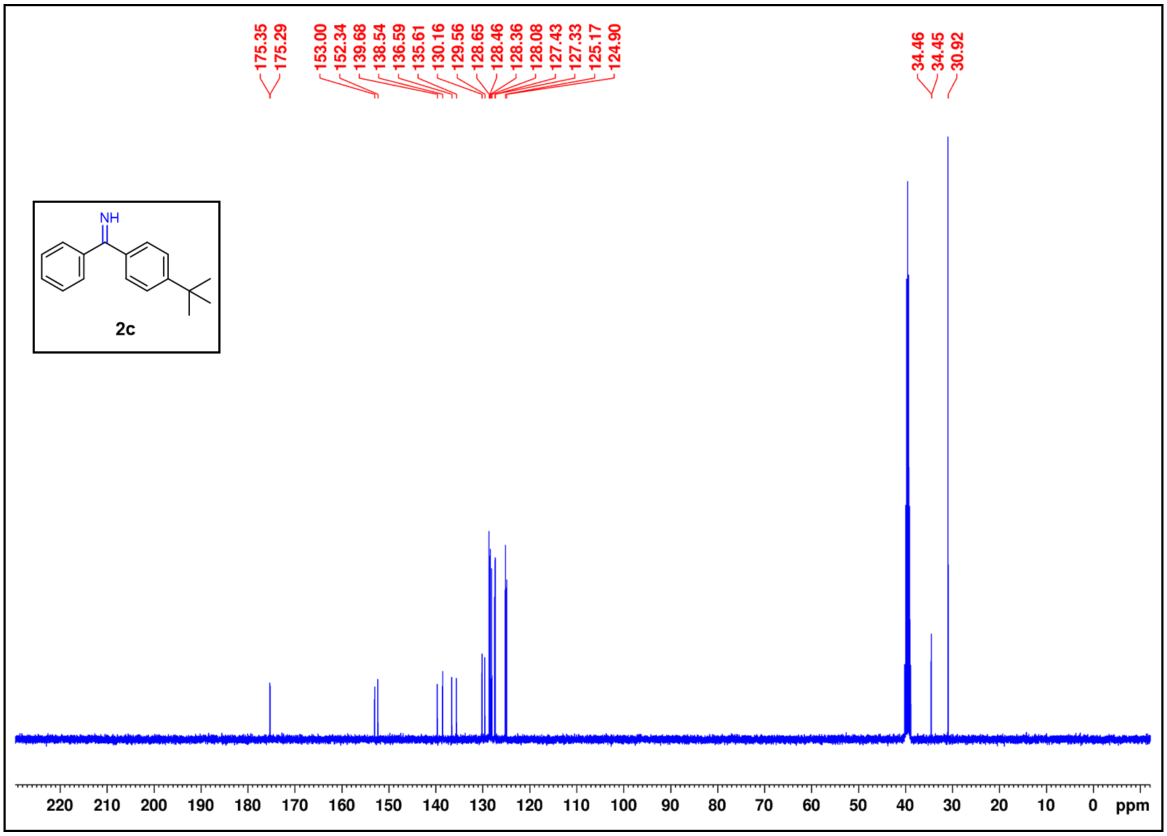

ROESY (400 MHz, DMSO-d_6) spectrum for **2c**

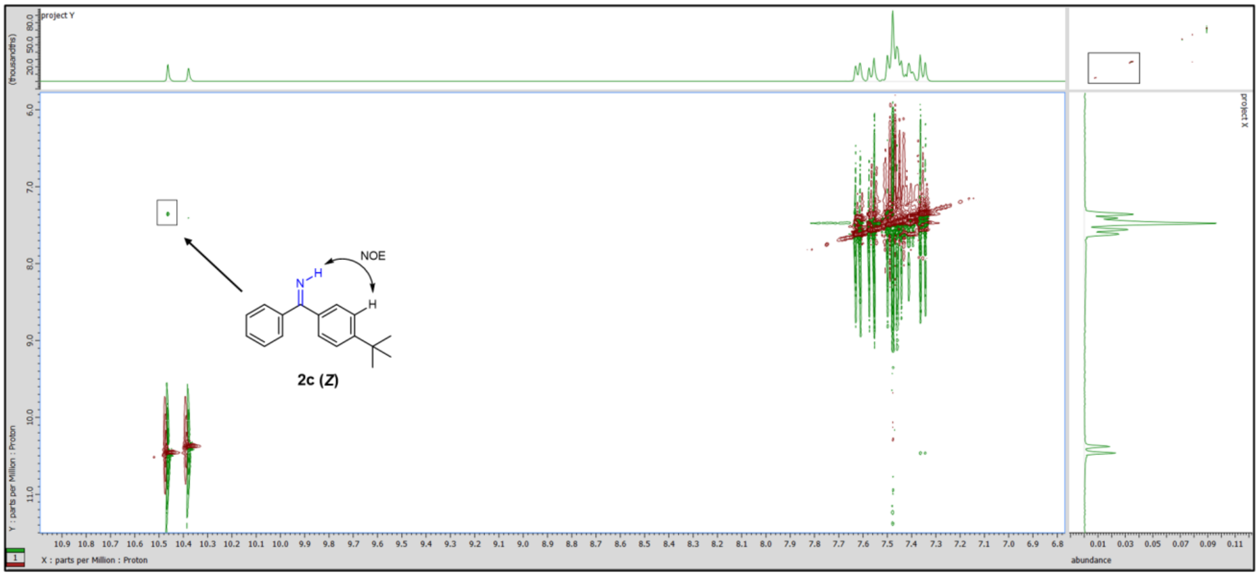

FT-IR (ATR, neat) and HRMS (ESI-positive) spectra for **2c**

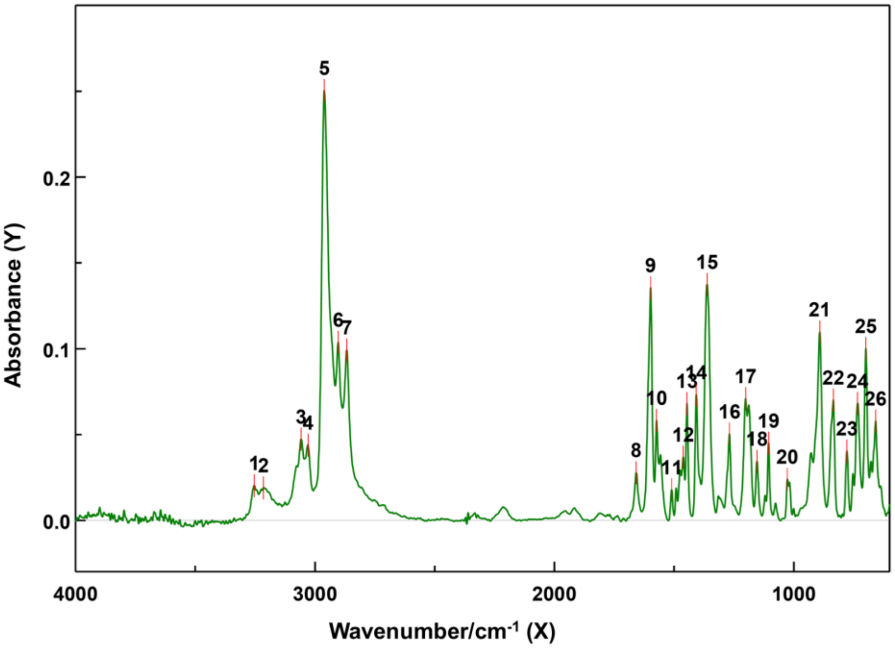

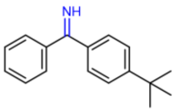

2c

No.	X	Y	No.	X	Y	No.	X	Y
1	3253.3	0.02	10	1573.6	0.058	19	1106	0.045
2	3215.7	0.019	11	1510	0.018	20	1027	0.024
3	3057.6	0.047	12	1461.8	0.037	21	891.9	0.11
4	3028.7	0.044	13	1446.4	0.068	22	835	0.07
5	2961.2	0.25	14	1407.8	0.073	23	778.1	0.04
6	2903.3	0.104	15	1362.5	0.137	24	733.8	0.068
7	2867.6	0.099	16	1268.9	0.05	25	699.1	0.1
8	1658.5	0.028	17	1201.4	0.071	26	658.6	0.058
9	1597.7	0.135	18	1154.2	0.034			

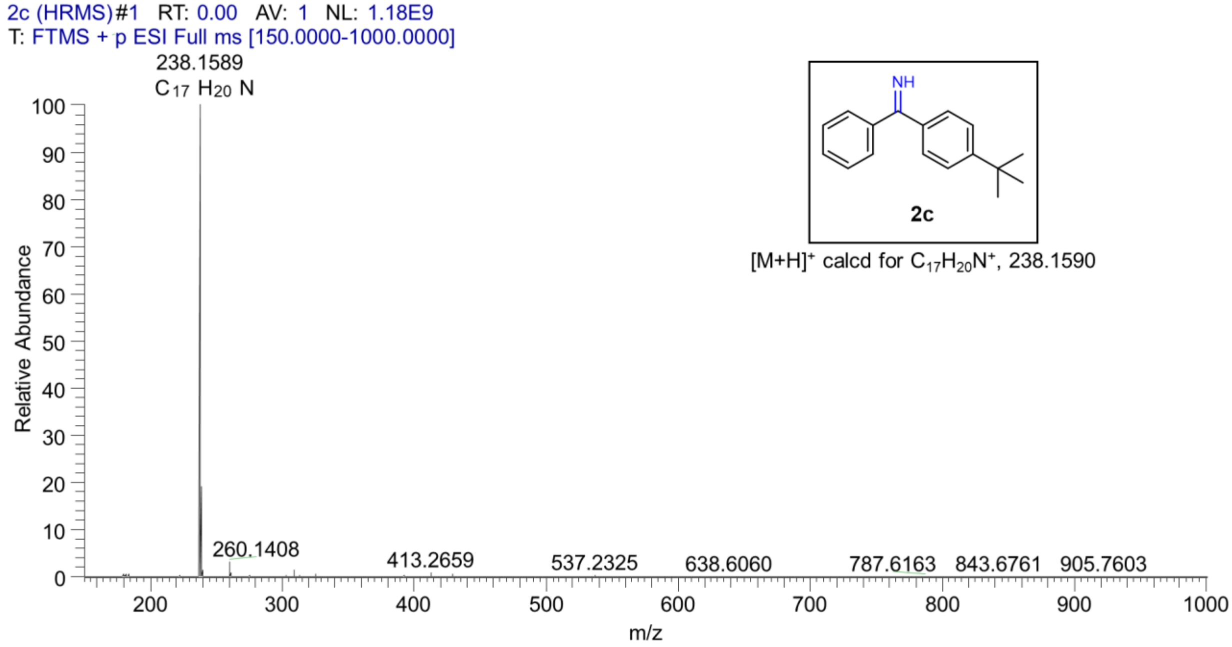

$[M+H]^+$ calcd for $C_{17}H_{20}N^+$, 238.1590

^{1}H NMR (400 MHz, DMSO-d_6) and ^{13}C NMR (100 MHz, DMSO-d_6) spectra for **2d**

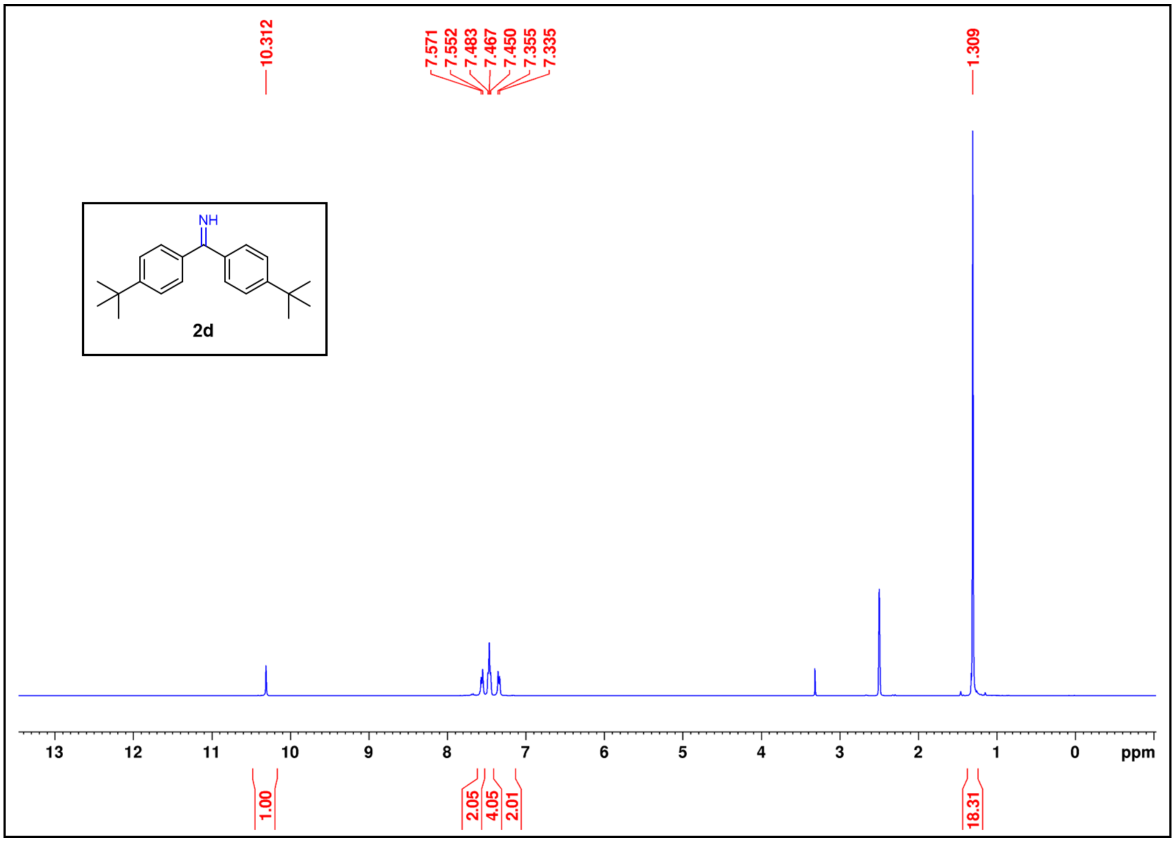

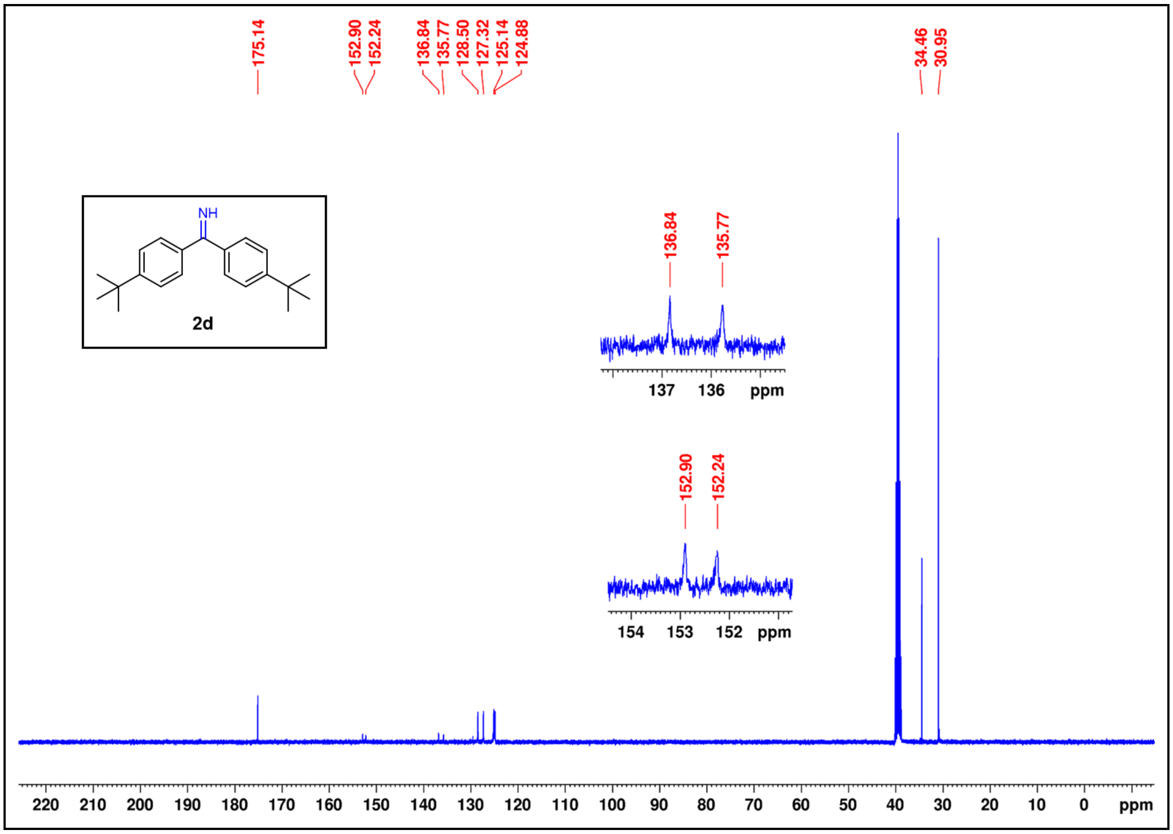

FT-IR (ATR, neat) and HRMS (ESI-positive) spectra for **2d**

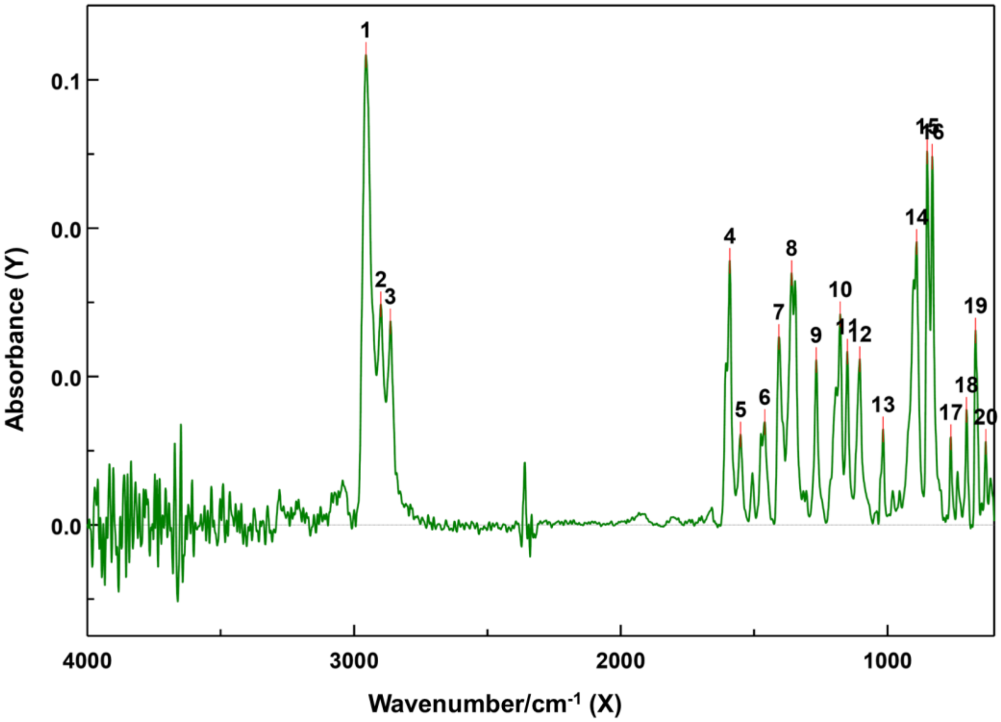

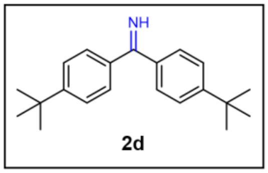

No.	X	Y	No.	X	Y	No.	X	Y
1	2955.4	0.063	8	1359.6	0.034	15	851.4	0.05
2	2899.5	0.03	9	1267	0.022	16	832.1	0.05
3	2863.8	0.027	10	1177.3	0.028	17	762.7	0.012
4	1591	0.036	11	1150.3	0.023	18	703.9	0.015
5	1551.5	0.012	12	1104	0.022	19	670.1	0.026
6	1460.8	0.014	13	1016.3	0.013	20	631.6	0.011
7	1406.8	0.025	14	891	0.038			

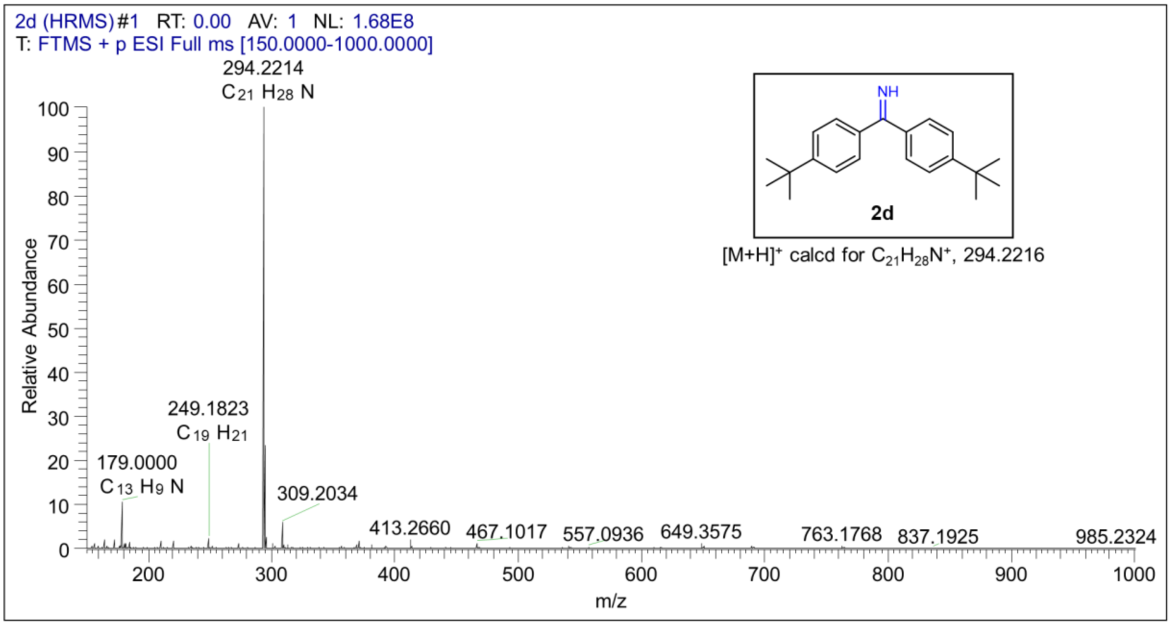

<u>^{1}H NMR (400 MHz, DMSO-d_6) and ^{13}C NMR (100 MHz, DMSO-d_6) spectra for **2e**</u>

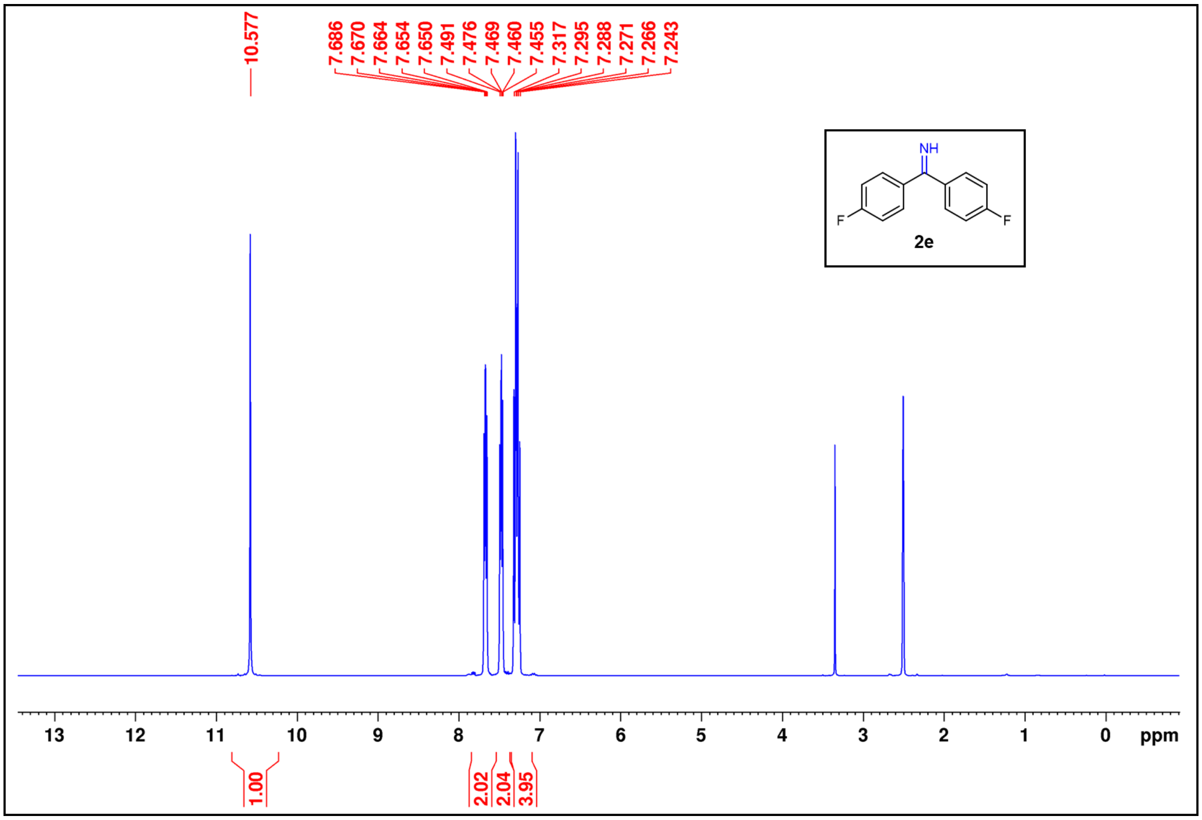

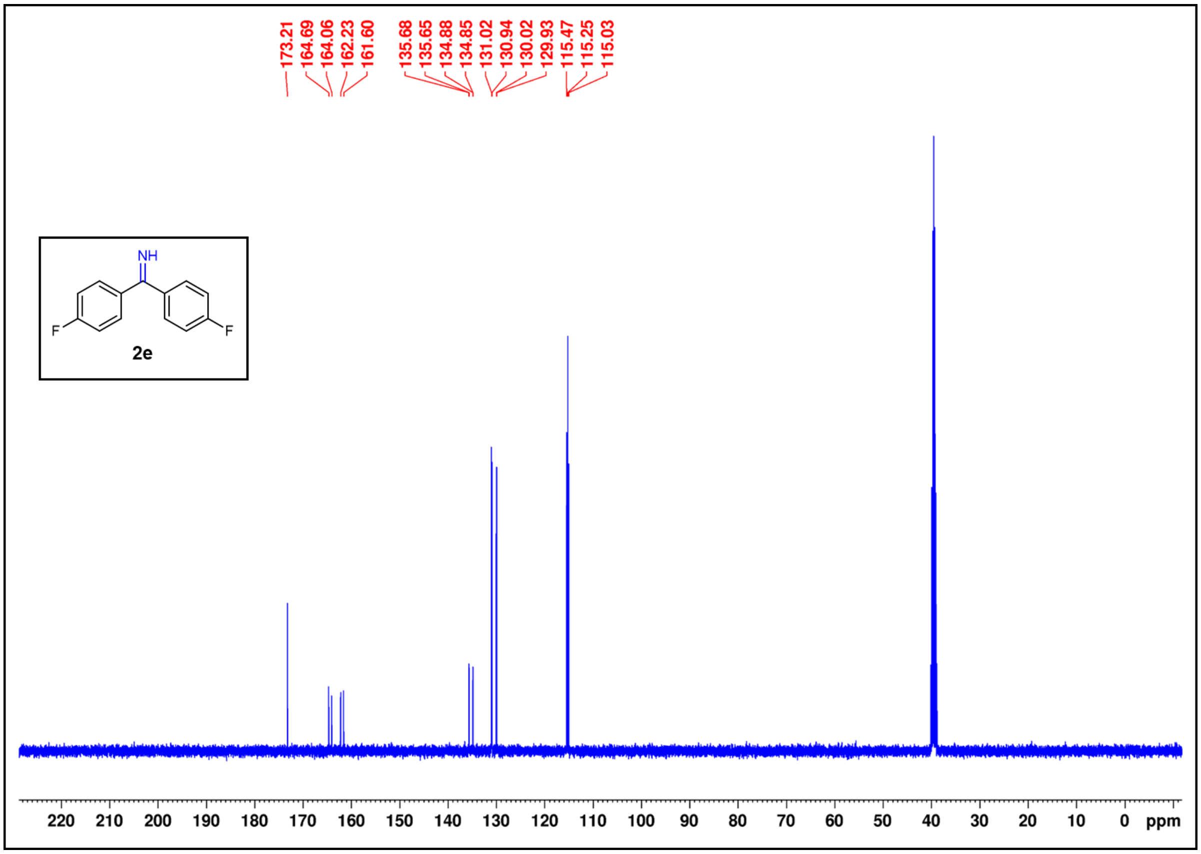

^{19}F NMR (376 MHz, DMSO-d_6) spectrum for **2e**

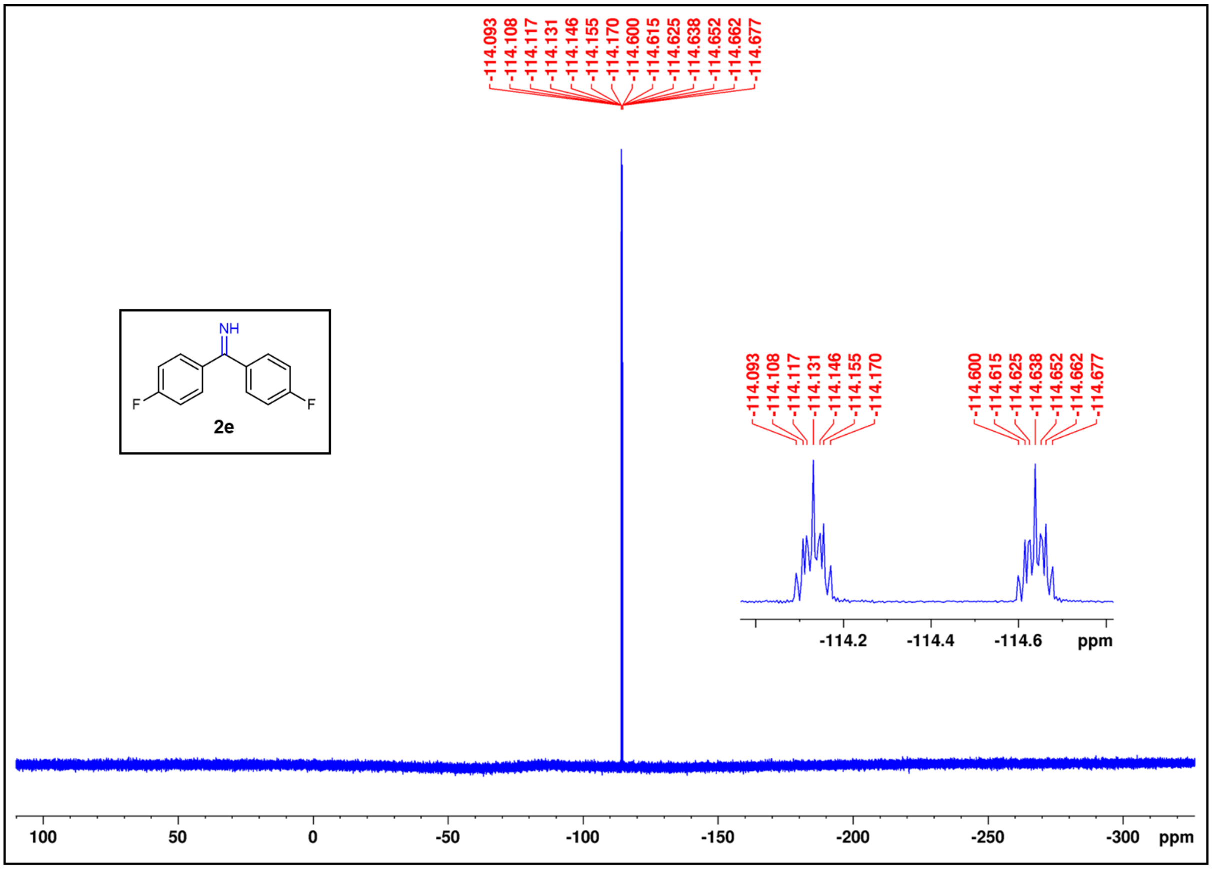

FT-IR (ATR, neat) and HRMS (ESI-positive) spectra for **2e**

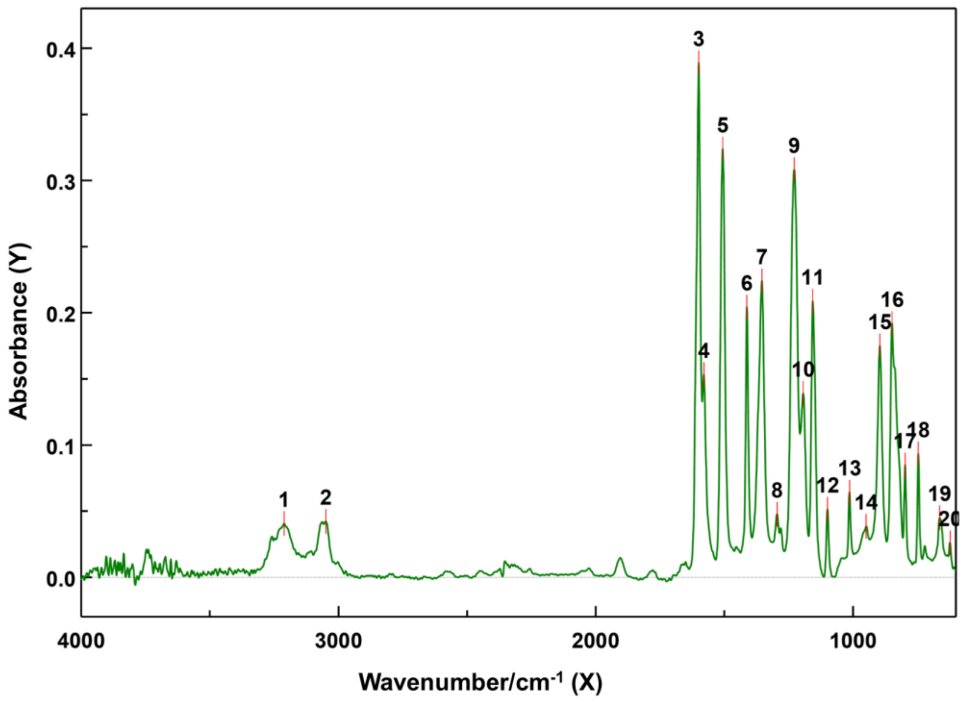

No.	X	Y	No.	X	Y	No.	X	Y
1	3210.9	0.04	8	1295	0.047	15	895.8	0.175
2	3048.9	0.042	9	1228.4	0.308	16	848.5	0.192
3	1599.7	0.389	10	1193.7	0.139	17	797.4	0.085
4	1579.4	0.153	11	1156.1	0.208	18	746.3	0.093
5	1506.1	0.323	12	1099.2	0.051	19	663.4	0.045
6	1412.6	0.204	13	1013.4	0.064	20	622.9	0.026
7	1353.8	0.224	14	948.8	0.038			

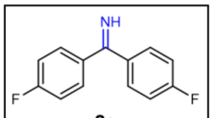

2e

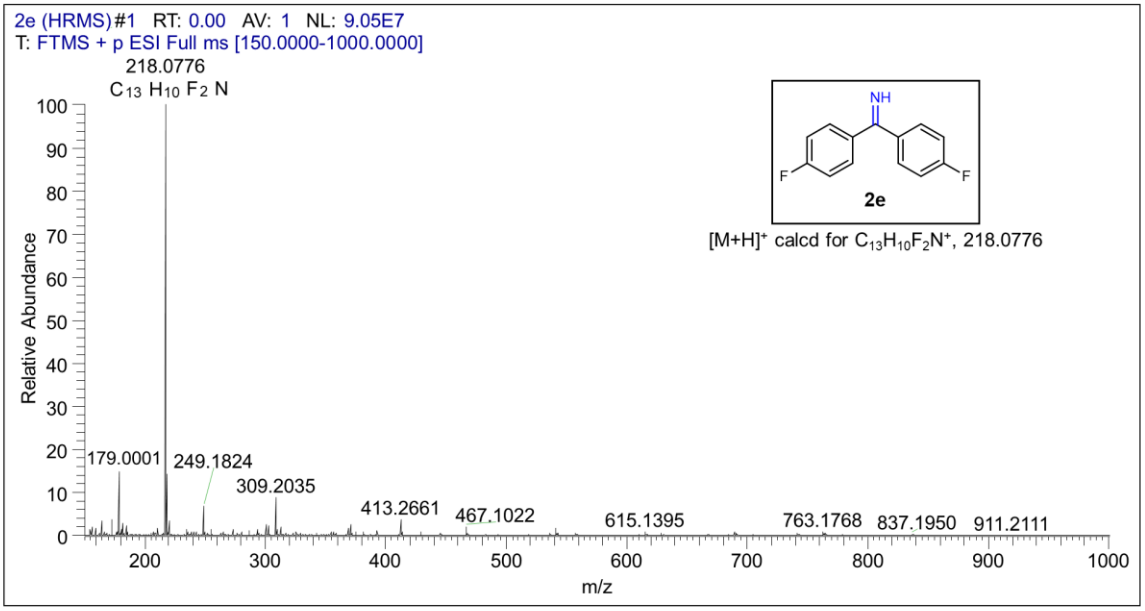

^{1}H NMR (400 MHz, DMSO-d_6) and ^{13}C NMR (100 MHz, DMSO-d_6) spectra for **2f**

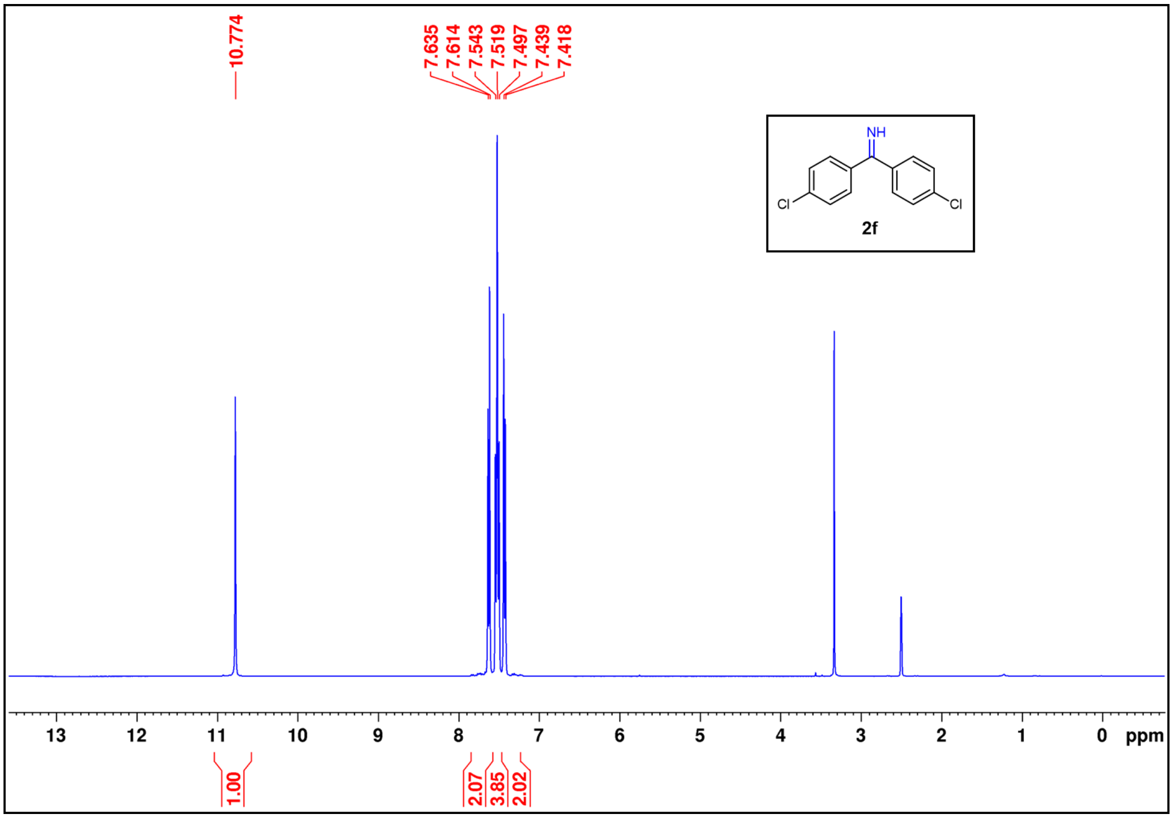

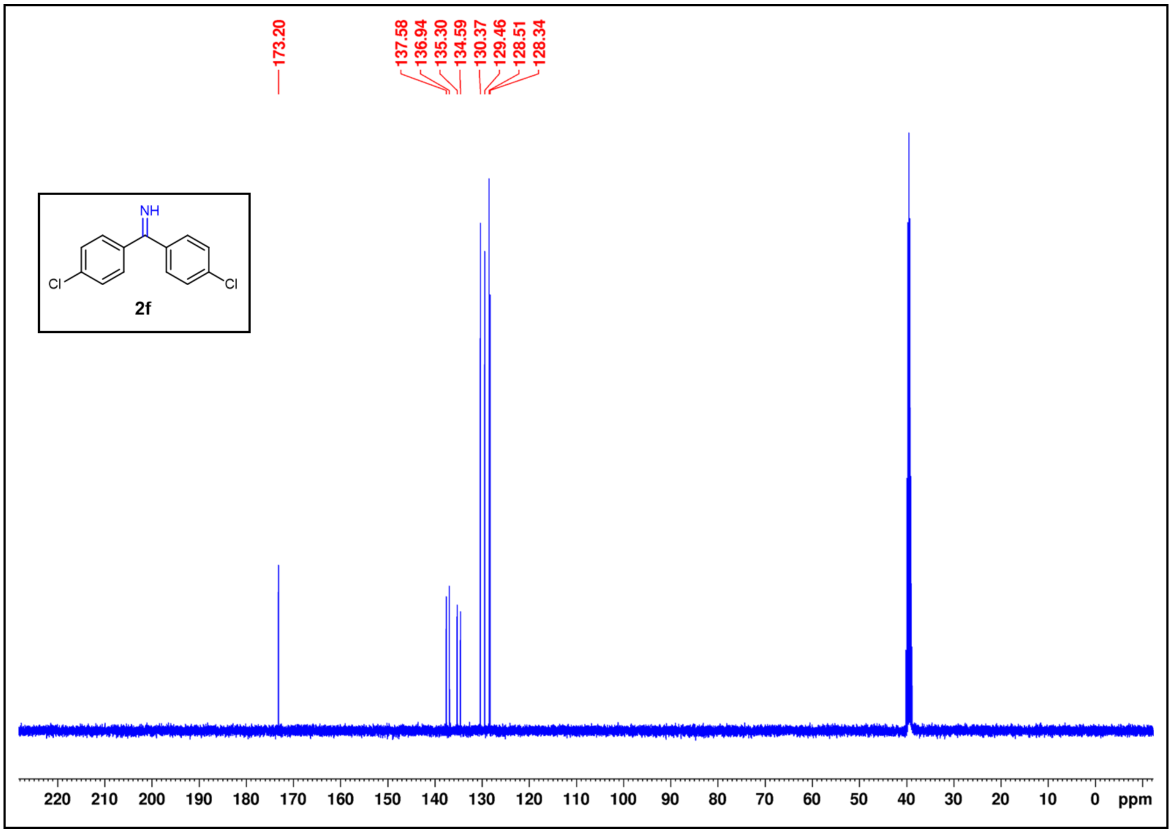

FT-IR (ATR, neat) and HRMS (ESI-positive) spectra for **2f**

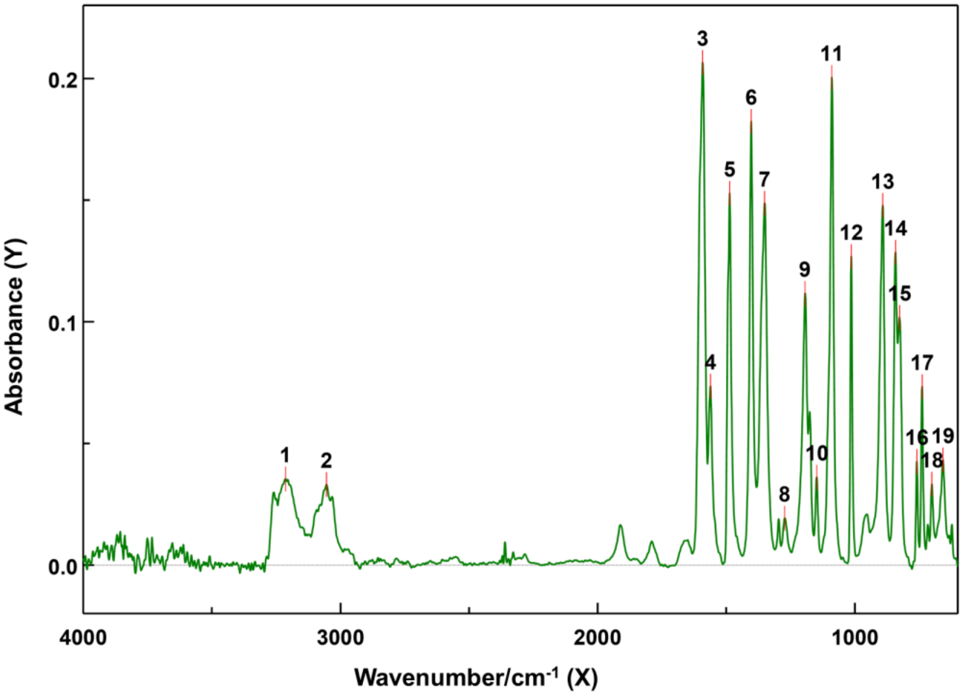

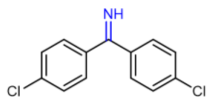

2f

No.	X	Y	No.	X	Y	No.	X	Y
1	3213.8	0.035	8	1272.8	0.019	15	826.3	0.102
2	3054.7	0.033	9	1193.7	0.112	16	758.9	0.043
3	1591.9	0.206	10	1148.4	0.036	17	738.6	0.073
4	1561.1	0.073	11	1089.6	0.2	18	701	0.033
5	1486.8	0.153	12	1014.4	0.127	19	657.6	0.043
6	1403	0.182	13	891.9	0.148			
7	1350.9	0.149	14	841.8	0.128			

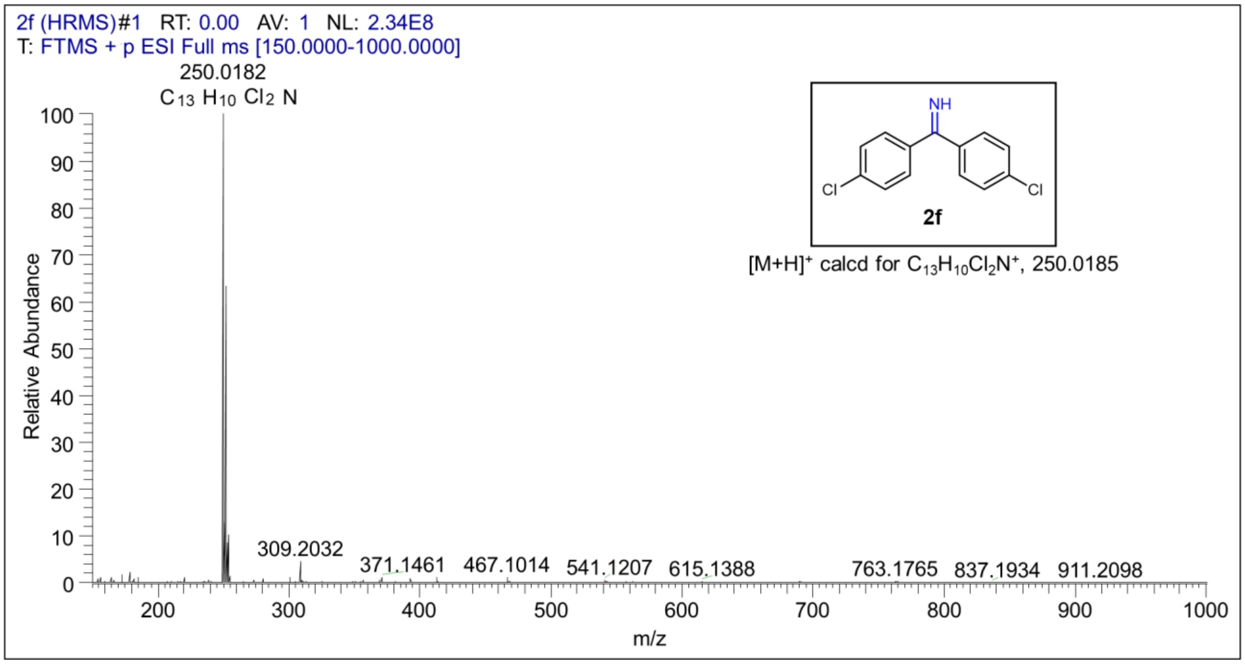

^{1}H NMR (400 MHz, DMSO-d_6) and ^{13}C NMR (100 MHz, DMSO-d_6) spectra for **2g**

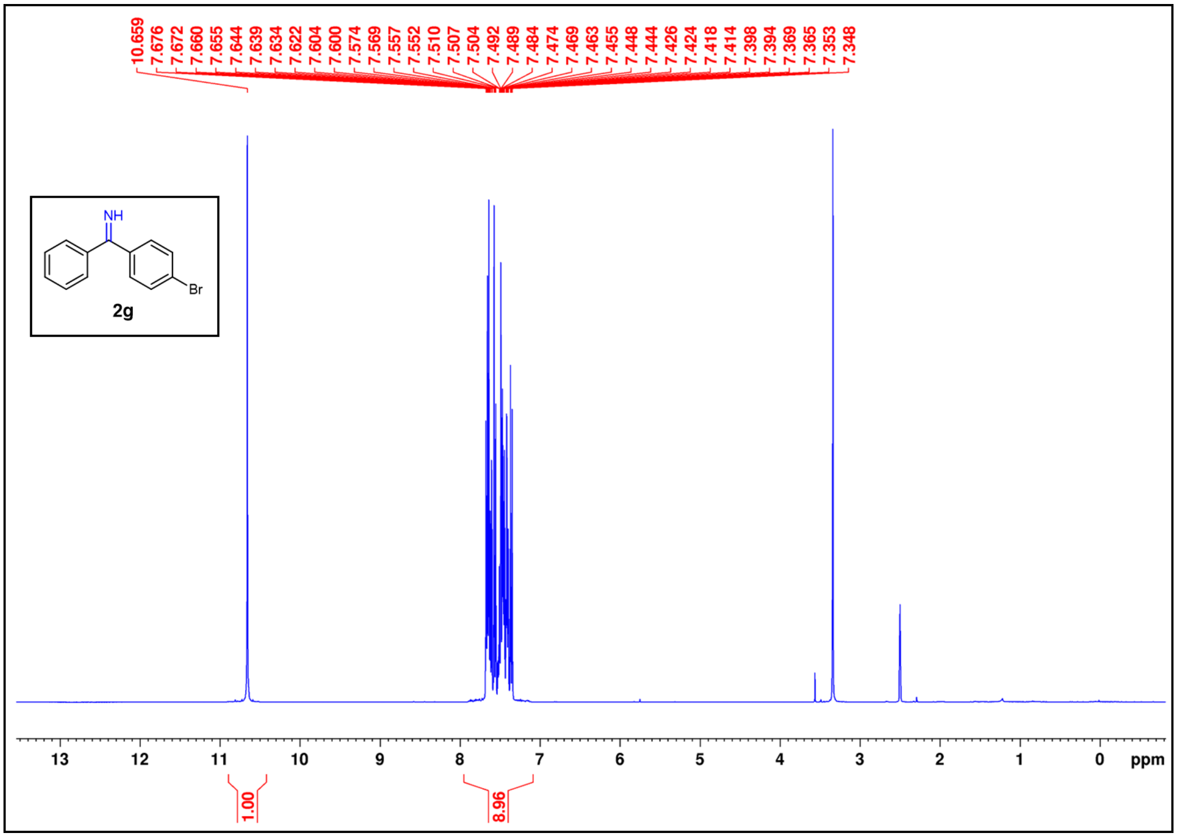

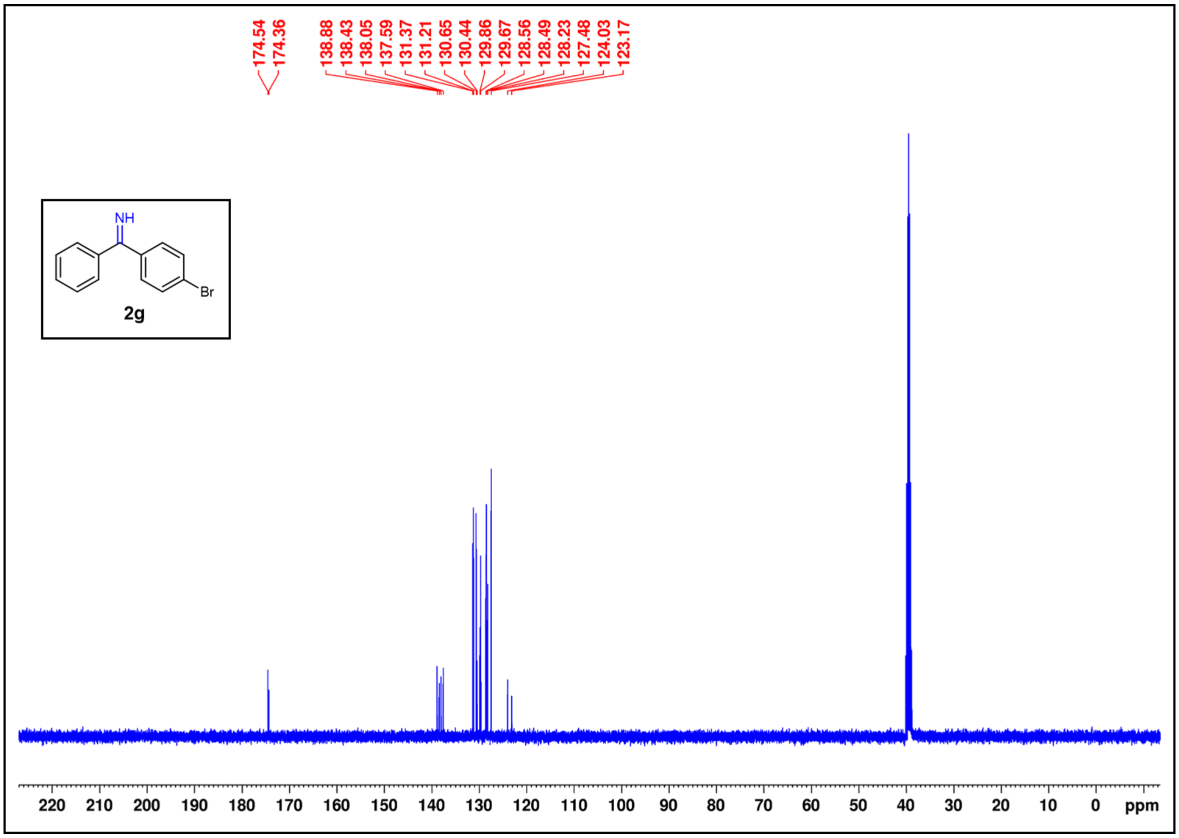

FT-IR (ATR, neat) and HRMS (ESI-positive) spectra for **2g**

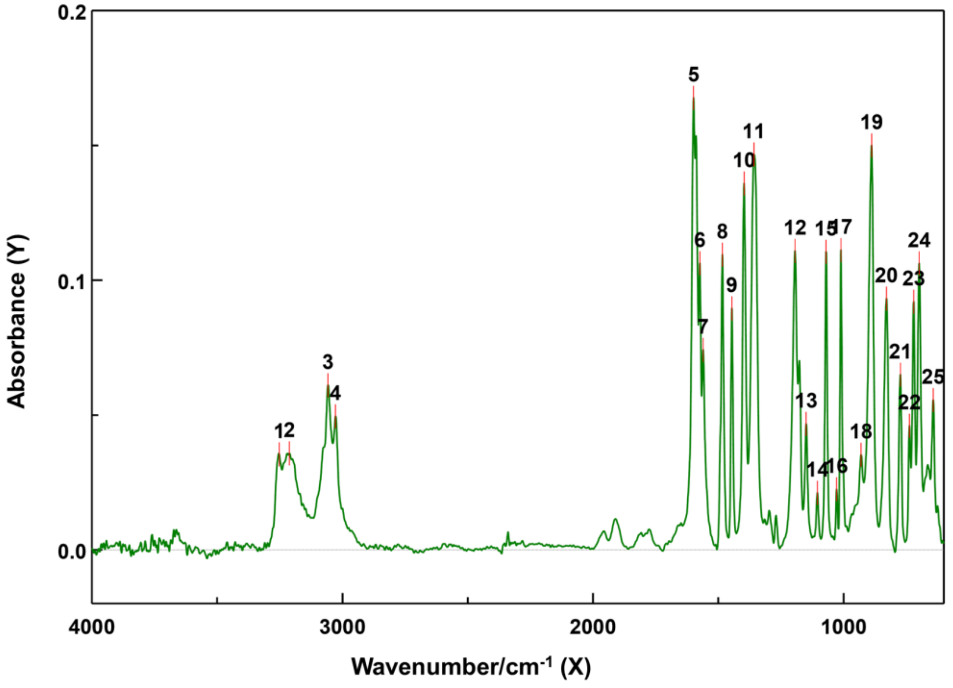

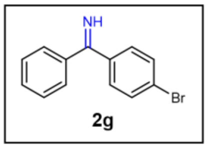

2g

No.	X	Y	No.	X	Y	No.	X	Y
1	3252.4	0.035	10	1397.2	0.136	19	888.1	0.15
2	3211.9	0.036	11	1357.6	0.147	20	829.2	0.093
3	3057.6	0.061	12	1193.7	0.111	21	773.3	0.065
4	3027.7	0.049	13	1149.4	0.047	22	737.6	0.046
5	1598.7	0.168	14	1105	0.021	23	720.3	0.092
6	1573.6	0.106	15	1070.3	0.11	24	698.1	0.106
7	1560.1	0.074	16	1027.9	0.022	25	642.2	0.055
8	1484	0.109	17	1010.5	0.111			
9	1446.4	0.089	18	930.5	0.035			

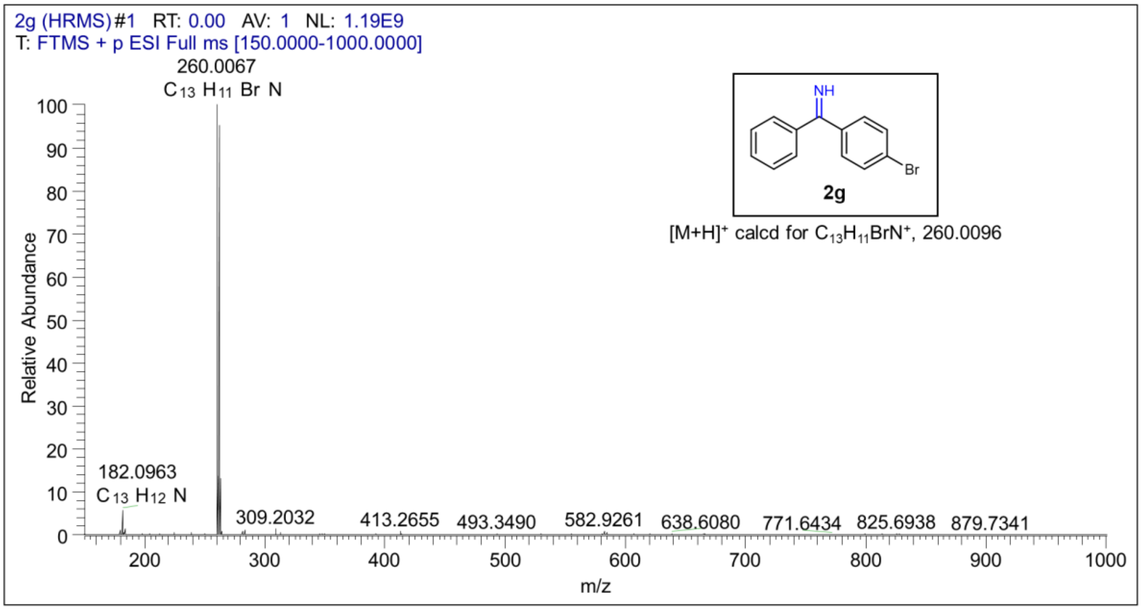

^{1}H NMR (400 MHz, DMSO-d_6) and ^{13}C NMR (100 MHz, DMSO-d_6) spectra for **2h**

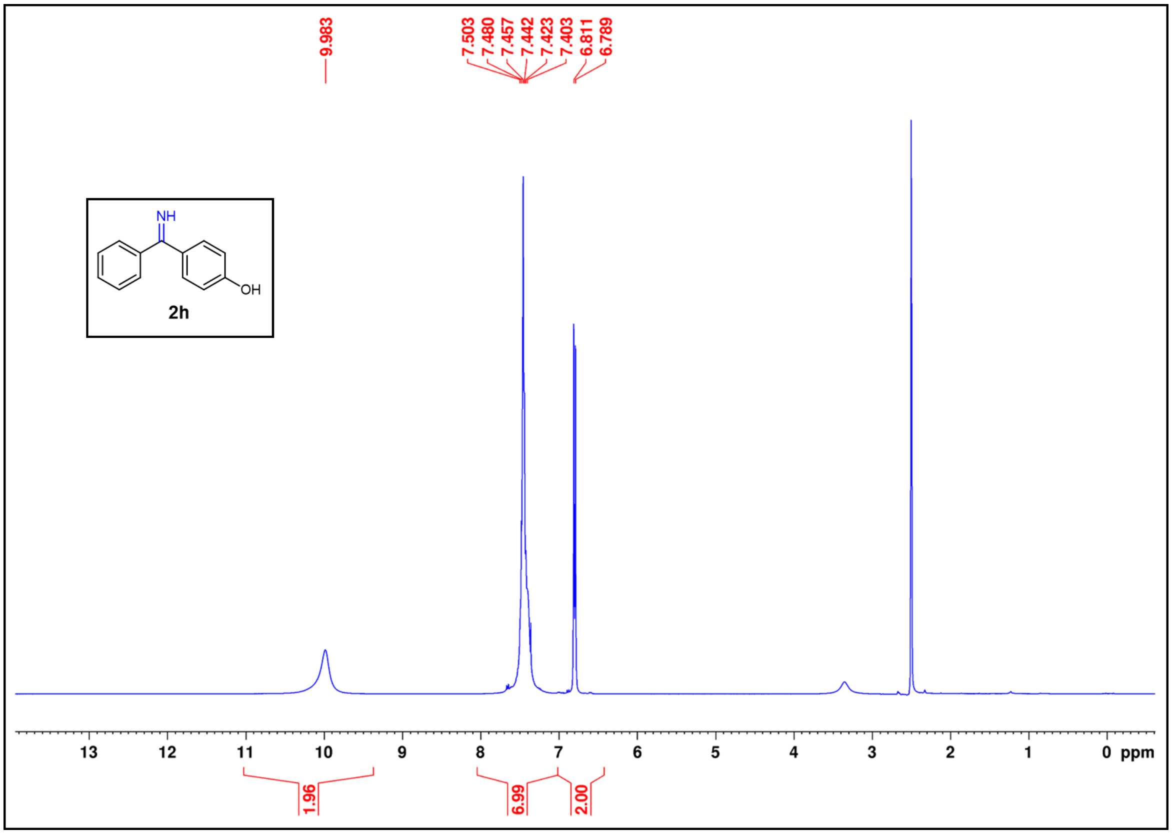

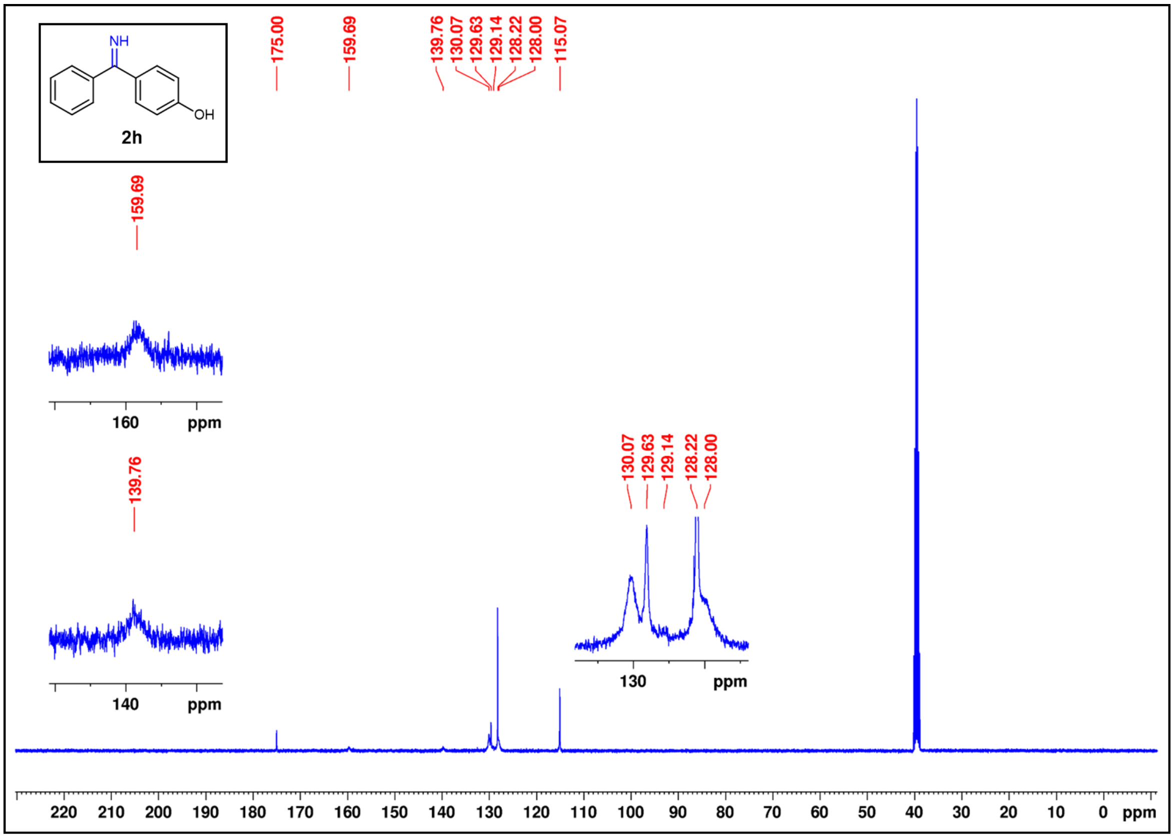

FT-IR (ATR, neat) and HRMS (ESI-positive) spectra for **2h**

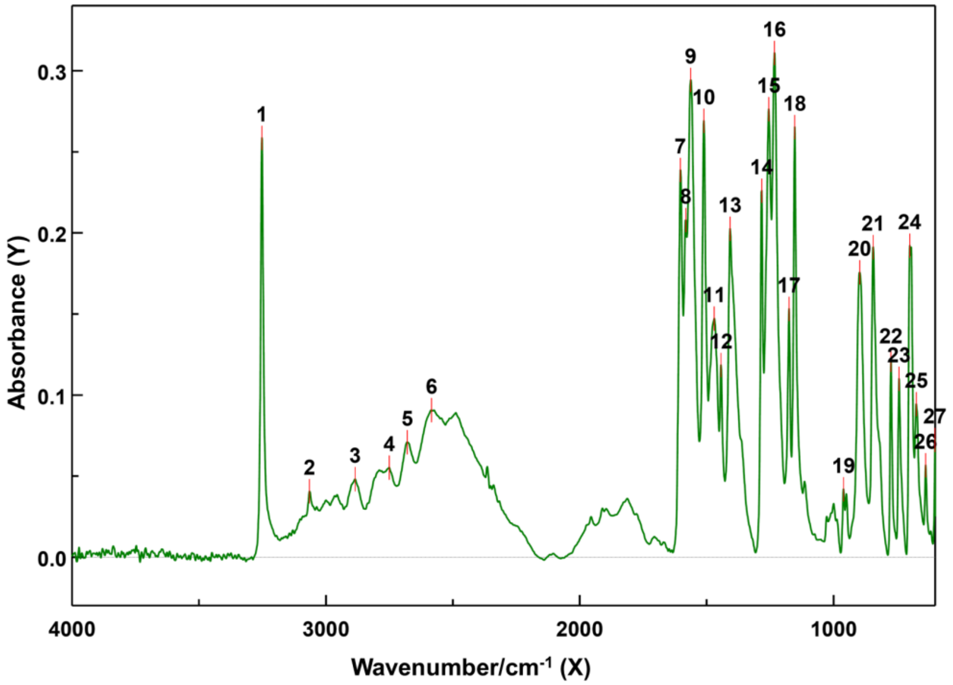

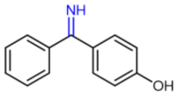

2h

No.	X	Y	No.	X	Y	No.	X	Y
1	3252.4	0.258	10	1511	0.269	19	960.4	0.042
2	3064.3	0.04	11	1470.5	0.147	20	897.7	0.176
3	2885	0.048	12	1443.5	0.118	21	843.7	0.191
4	2751	0.055	13	1407.8	0.202	22	774.3	0.122
5	2679.6	0.071	14	1283.4	0.226	23	742.5	0.11
6	2584.1	0.091	15	1255.4	0.276	24	700	0.192
7	1603.5	0.239	16	1233.3	0.311	25	674	0.094
8	1582.3	0.208	17	1175.4	0.153	26	637.4	0.057
9	1563	0.294	18	1153.2	0.265	27	600.7	0.072

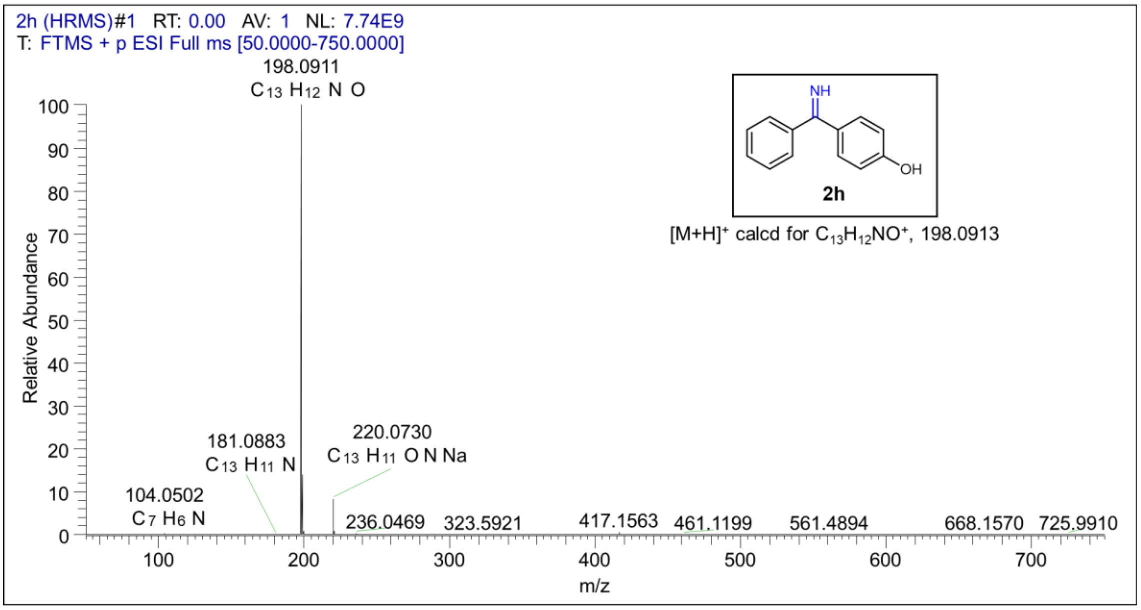

^{1}H NMR (400 MHz, DMSO-d_6) and ^{13}C NMR (100 MHz, DMSO-d_6) spectra for **2i**

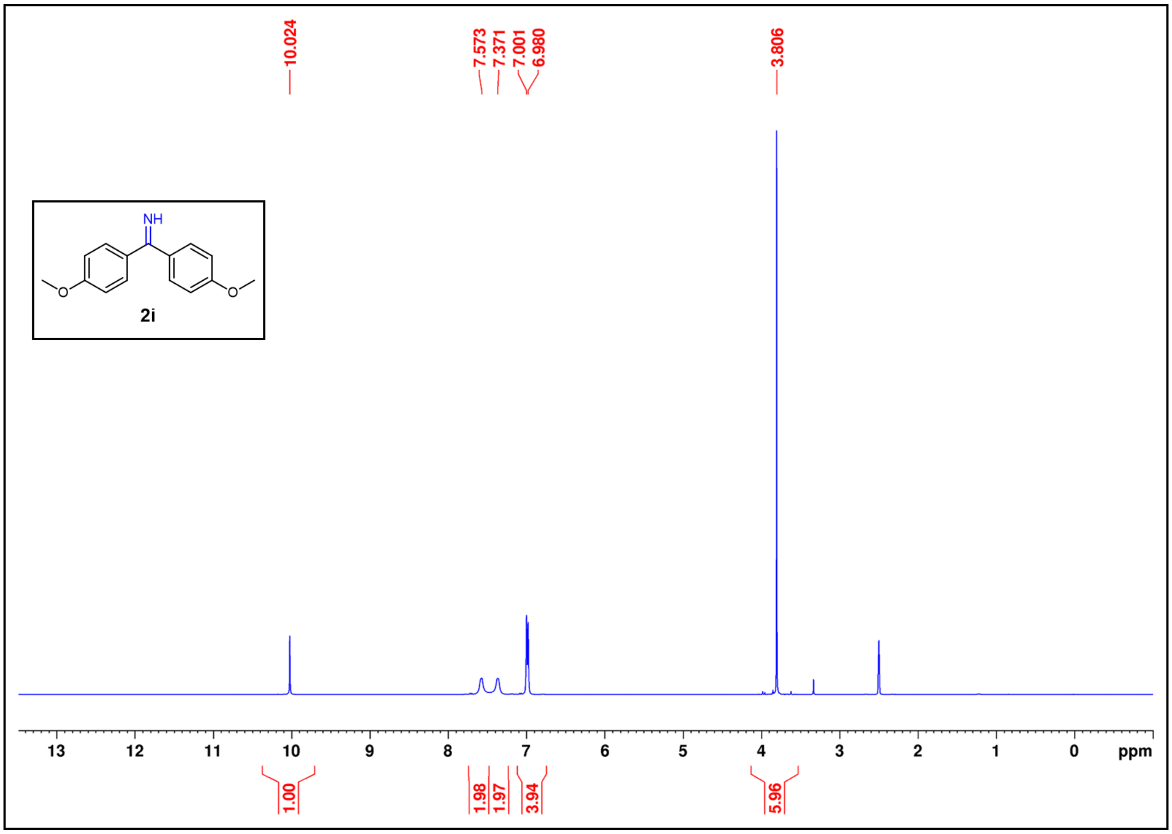

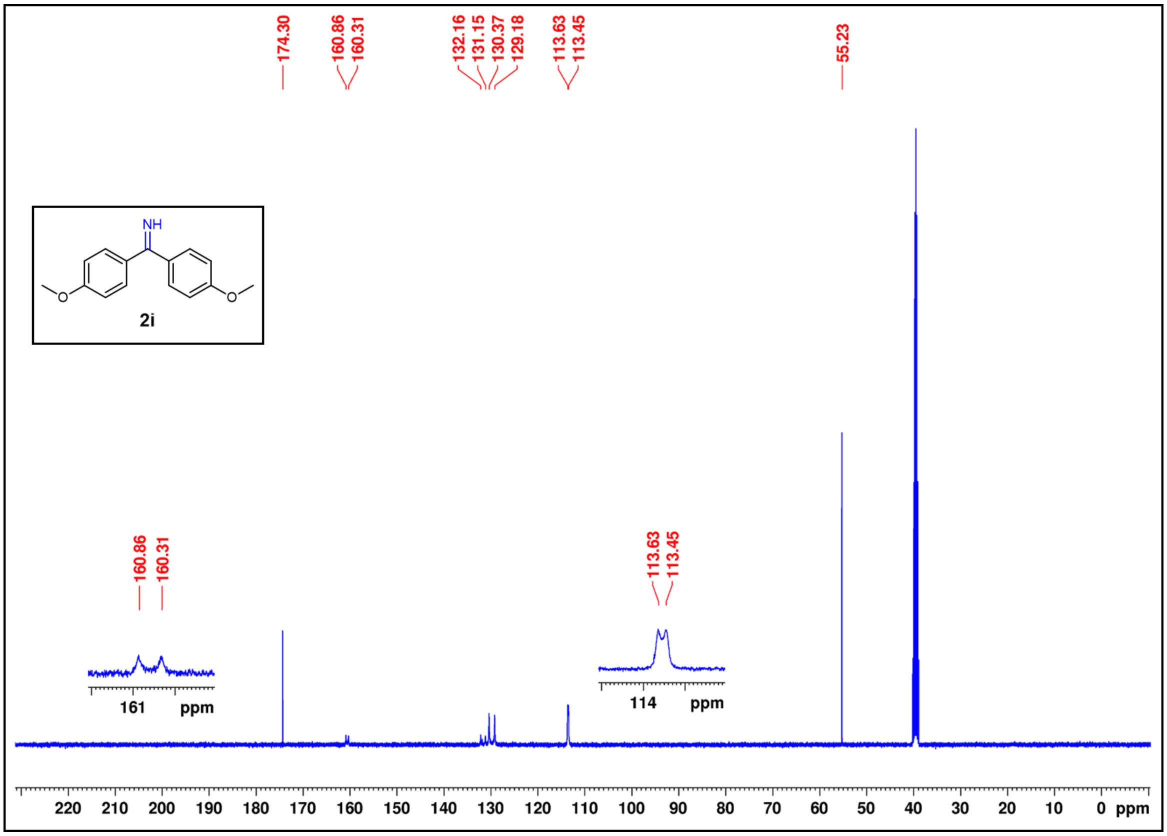

FT-IR (ATR, neat) and HRMS (ESI-positive) spectra for **2i**

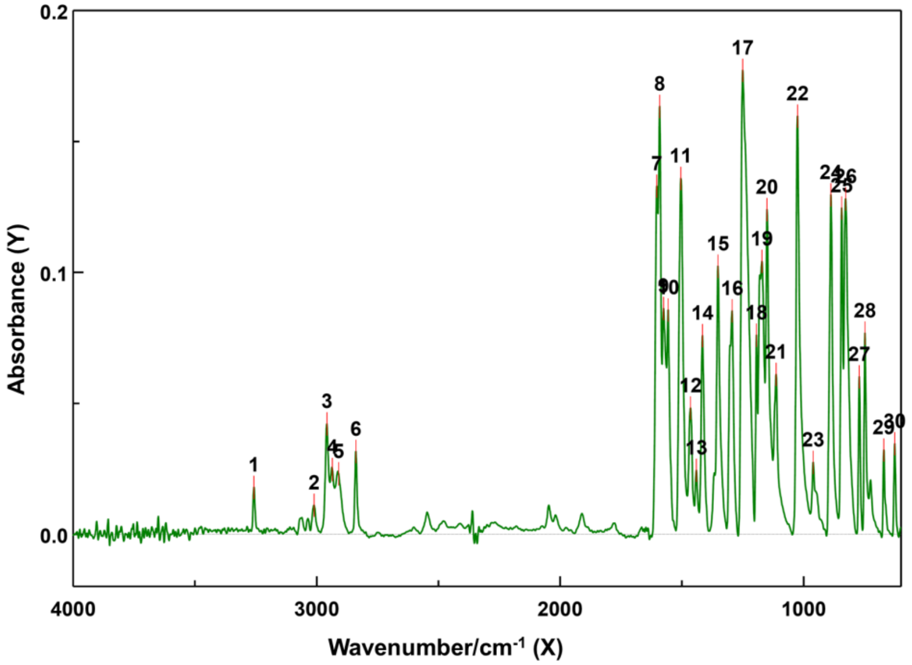

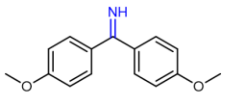

2i

No.	X	Y	No.	X	Y	No.	X	Y
1	3258.1	0.018	11	1504.2	0.136	21	1113	0.061
2	3010.3	0.011	12	1464.7	0.048	22	1025	0.16
3	2958.3	0.042	13	1440.6	0.024	23	960.4	0.027
4	2935.1	0.025	14	1415.5	0.076	24	888.1	0.13
5	2910.1	0.023	15	1351.9	0.102	25	843.7	0.125
6	2838.7	0.032	16	1294	0.085	26	827.3	0.128
7	1602.6	0.133	17	1250.6	0.177	27	771.4	0.06
8	1591	0.163	18	1193.7	0.076	28	748.2	0.077
9	1575.6	0.086	19	1171.5	0.104	29	671.1	0.032
10	1556.3	0.086	20	1150.3	0.124	30	625.8	0.034

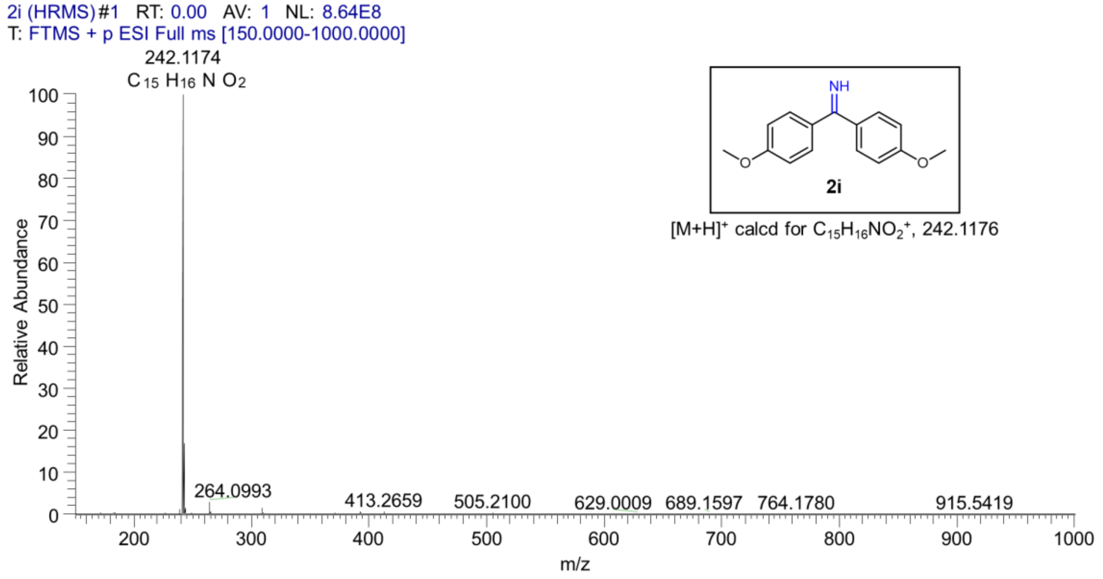

^1H NMR (400 MHz, DMSO-d_6) and ^{13}C NMR (100 MHz, DMSO-d_6) spectra for **2i**·HCl

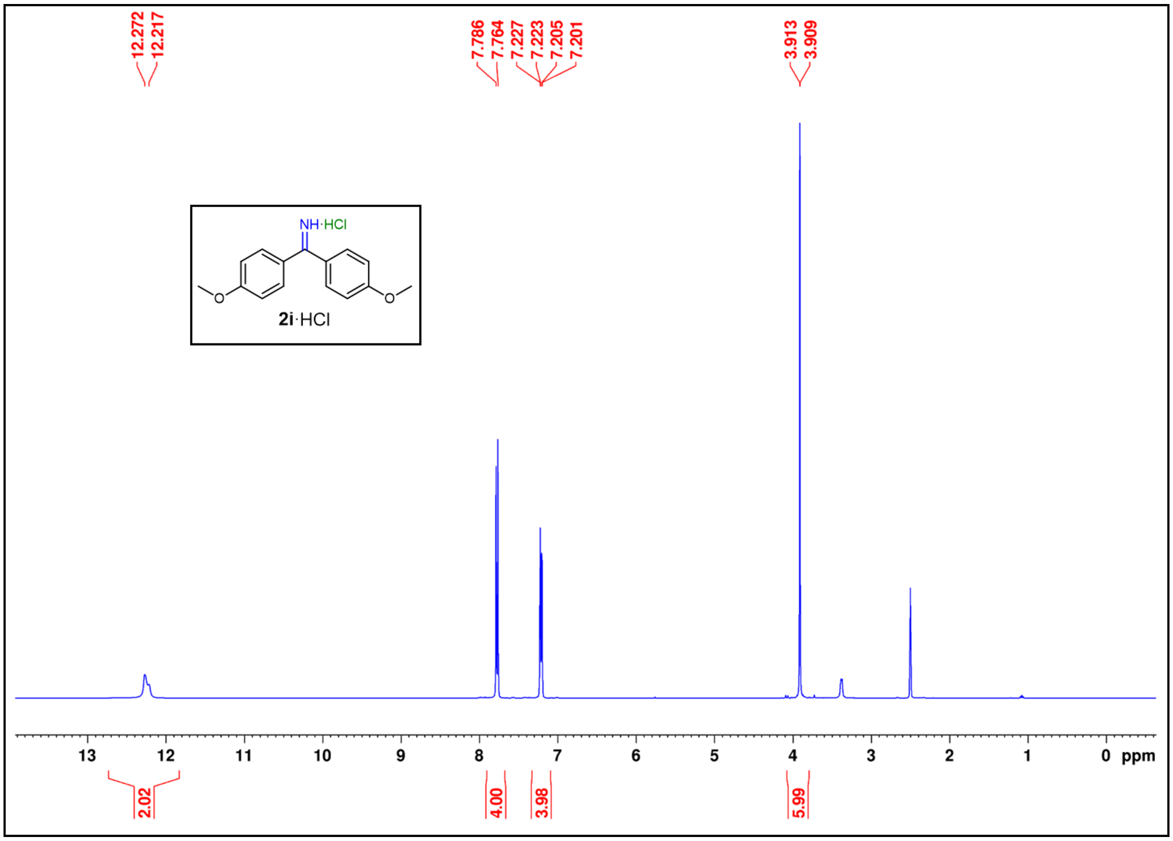

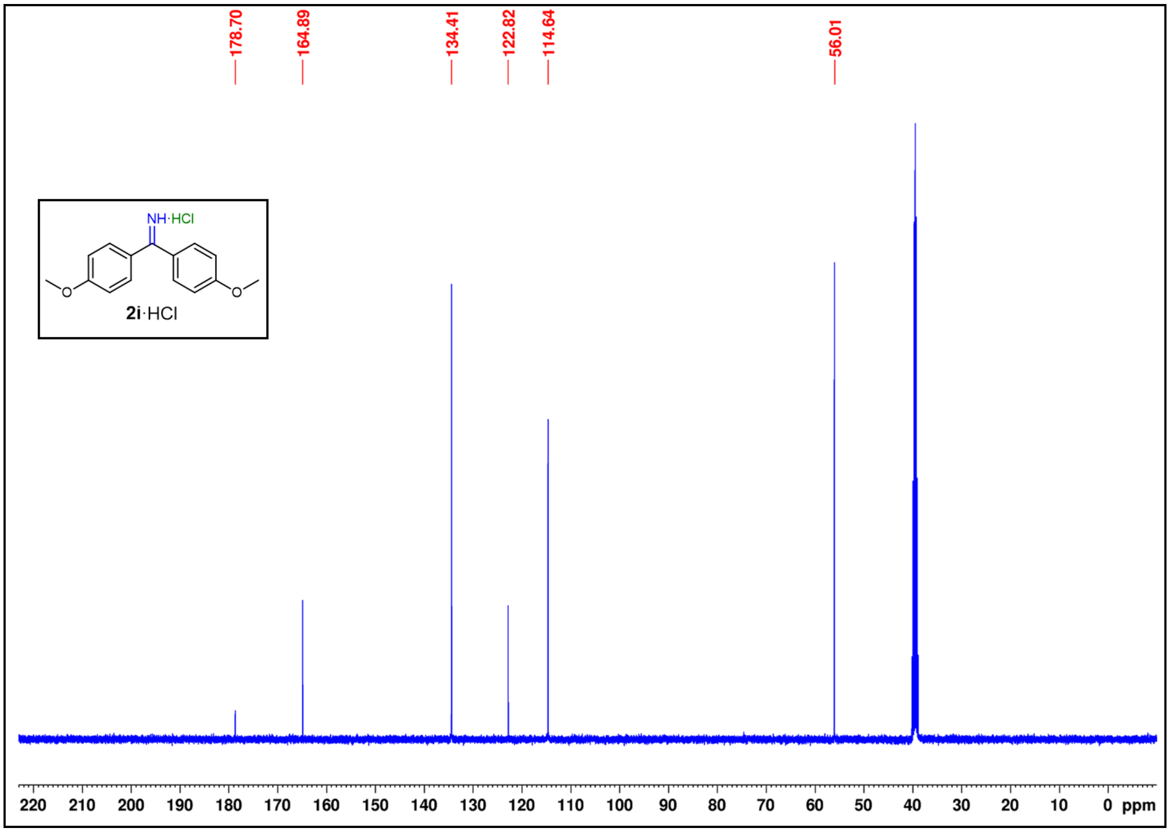

FT-IR (ATR, neat) and HRMS (ESI-positive) spectra for **2i**·HCl

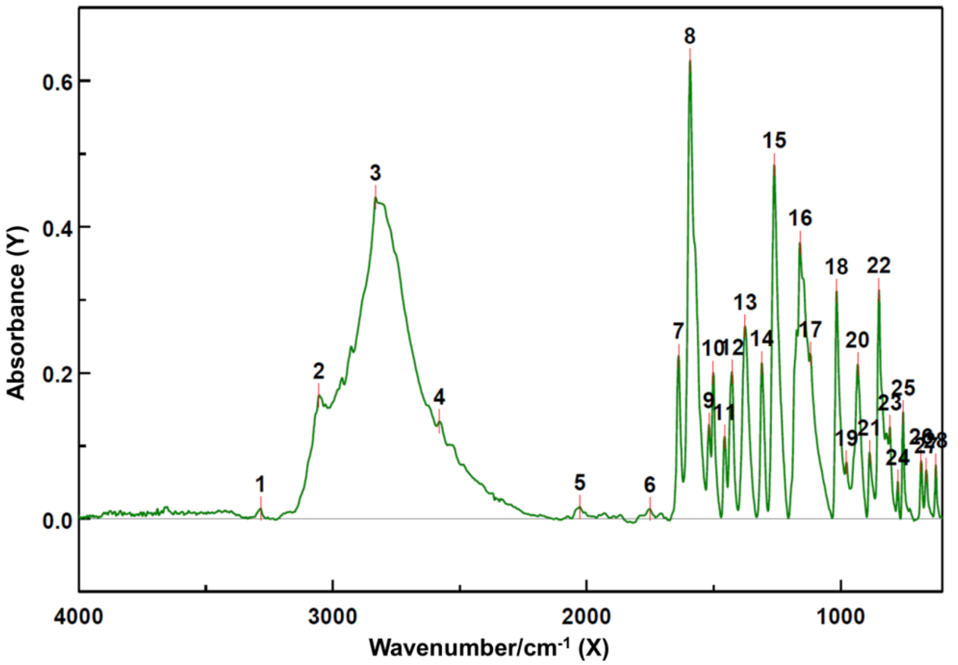

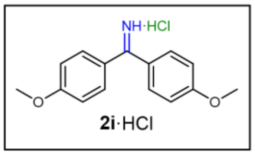

No.	X	Y	No.	X	Y	No.	X	Y
1	3284.2	0.014	2	3053.7	0.169	3	2831	0.44
4	2579.3	0.133	5	2026.8	0.016	6	1752	0.013
7	1638.2	0.223	8	1592.9	0.628	9	1519	0.129
10	1501.3	0.2	11	1457	0.112	12	1428	0.201
13	1376	0.263	14	1310.4	0.213	15	1261	0.485
16	1160	0.378	17	1119.5	0.226	18	1016	0.312
19	977.7	0.077	20	933.4	0.211	21	886.1	0.091
22	849.5	0.313	23	806.1	0.126	24	775.2	0.051
25	754	0.146	26	682.7	0.079	27	662.4	0.067
28	625.8	0.073						

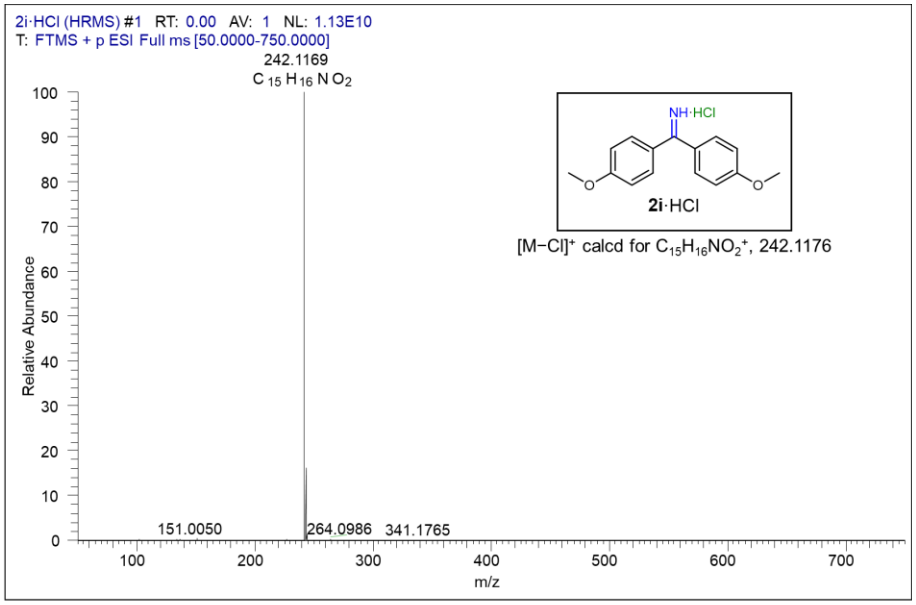

^{1}H NMR (400 MHz, DMSO-d_6) and ^{13}C NMR (100 MHz, DMSO-d_6) spectra for **2j**

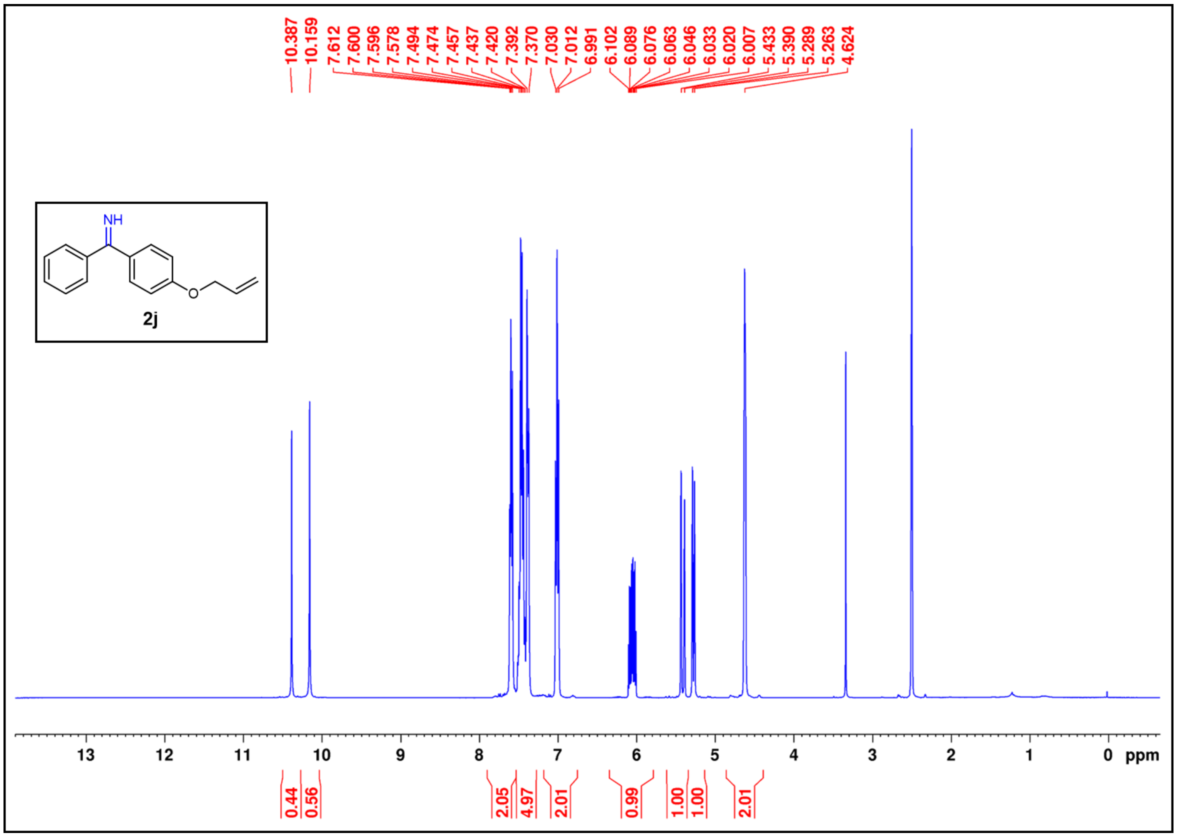

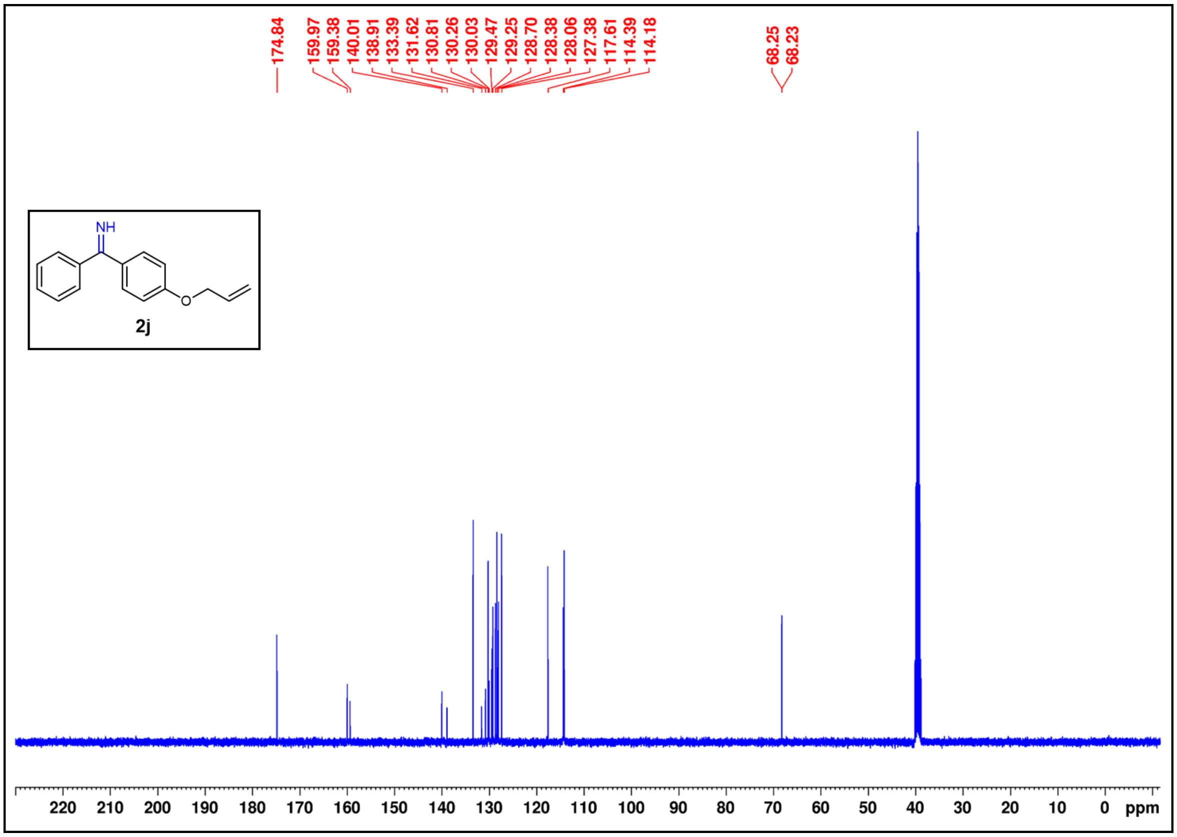

FT-IR (ATR, neat) and HRMS (ESI-positive) spectra for **2j**

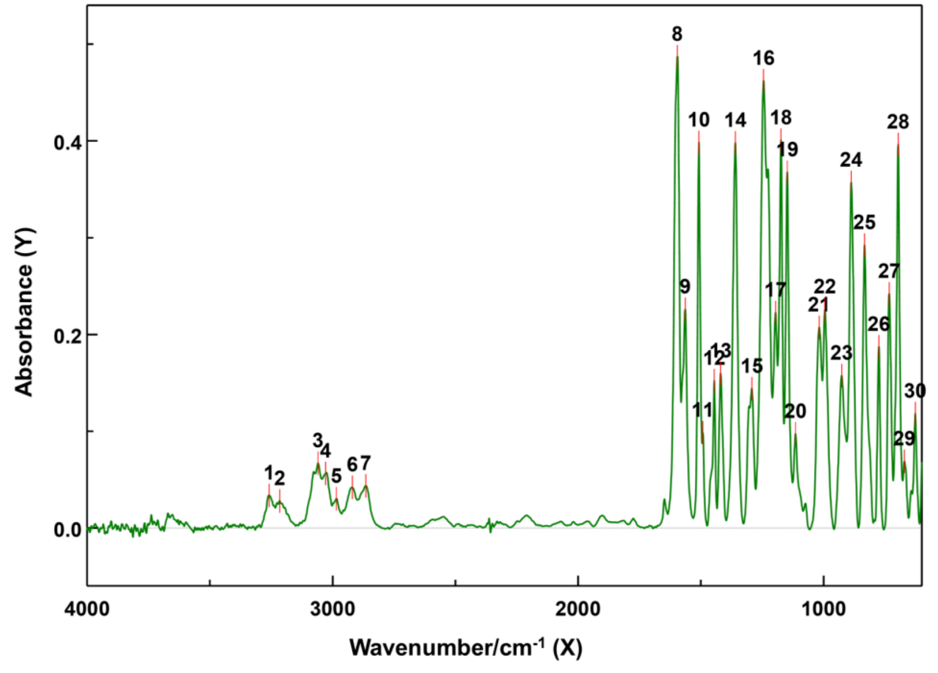

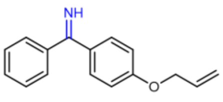

2j

No.	X	Y	No.	X	Y	No.	X	Y
1	3259.1	0.033	11	1491.7	0.098	21	1018	0.207
2	3215.7	0.028	12	1445.4	0.152	22	995.1	0.226
3	3059.5	0.067	13	1419.4	0.16	23	926.6	0.157
4	3028.7	0.056	14	1359.6	0.398	24	887.1	0.357
5	2984.3	0.03	15	1292.1	0.144	25	833.1	0.292
6	2919.7	0.042	16	1244.8	0.462	26	775.2	0.187
7	2864.7	0.043	17	1195.7	0.222	27	732.8	0.242
8	1595.8	0.487	18	1173.5	0.401	28	696.2	0.396
9	1564	0.226	19	1148.4	0.367	29	671.1	0.069
10	1508.1	0.398	20	1114.7	0.097	30	626.8	0.118

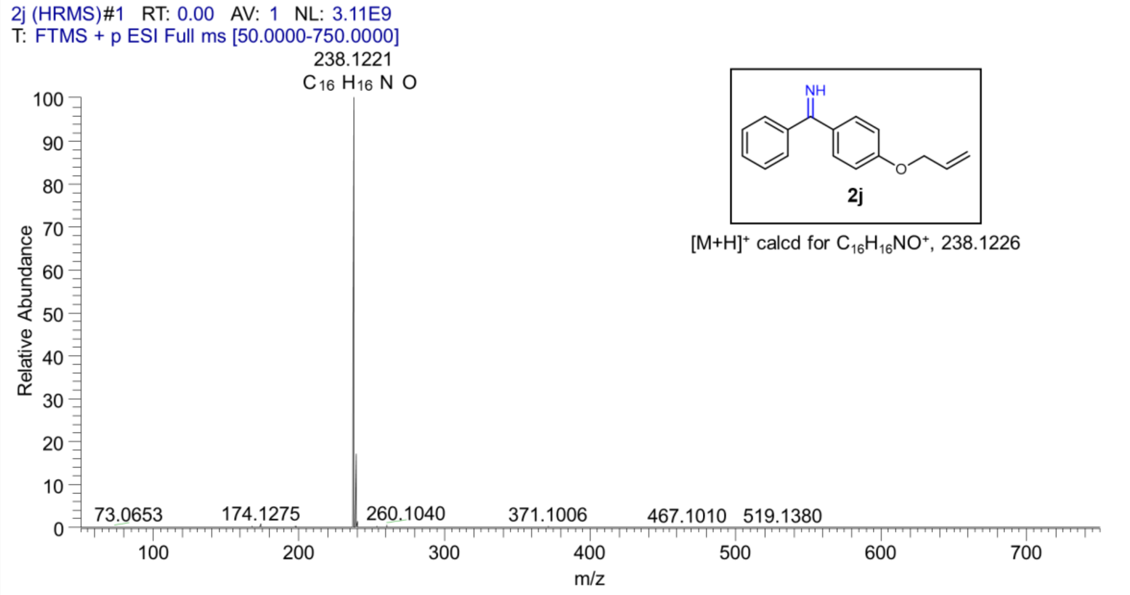

^{1}H NMR (400 MHz, DMSO-d_6) and ^{13}C NMR (100 MHz, DMSO-d_6) spectra for **2k**

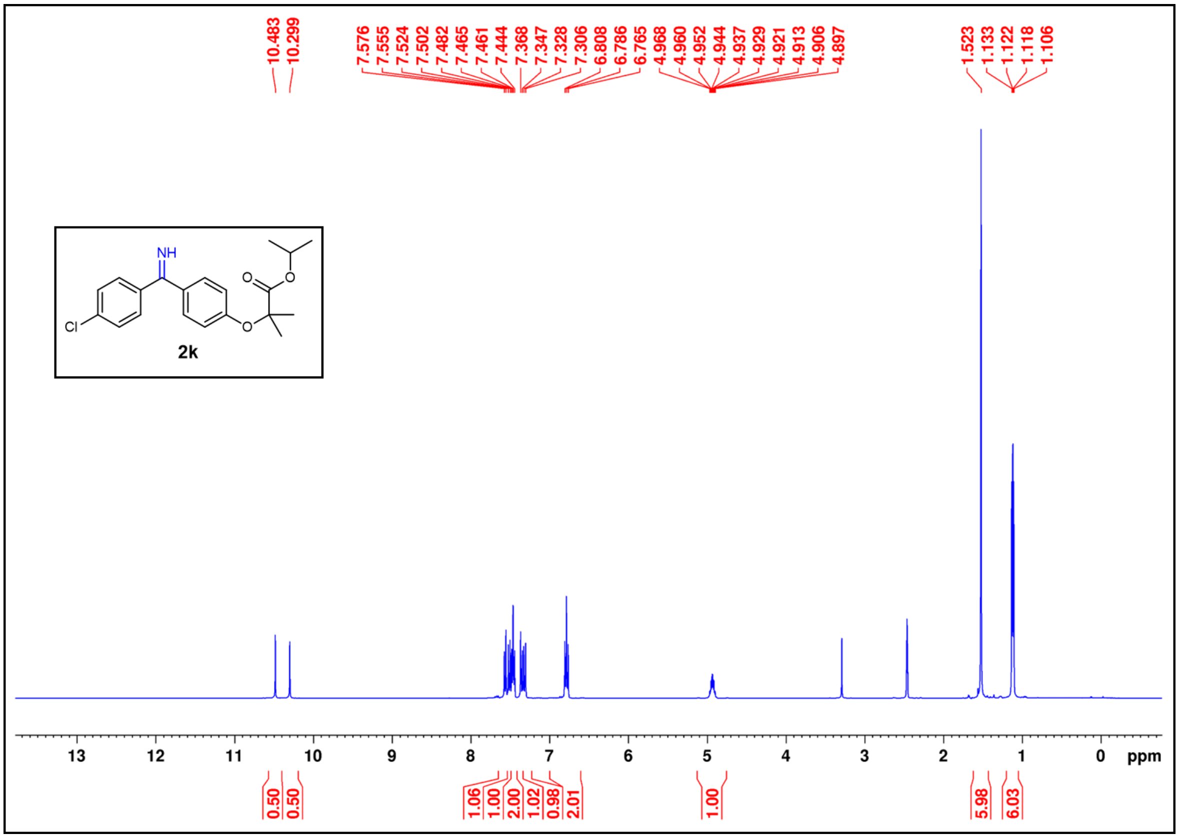

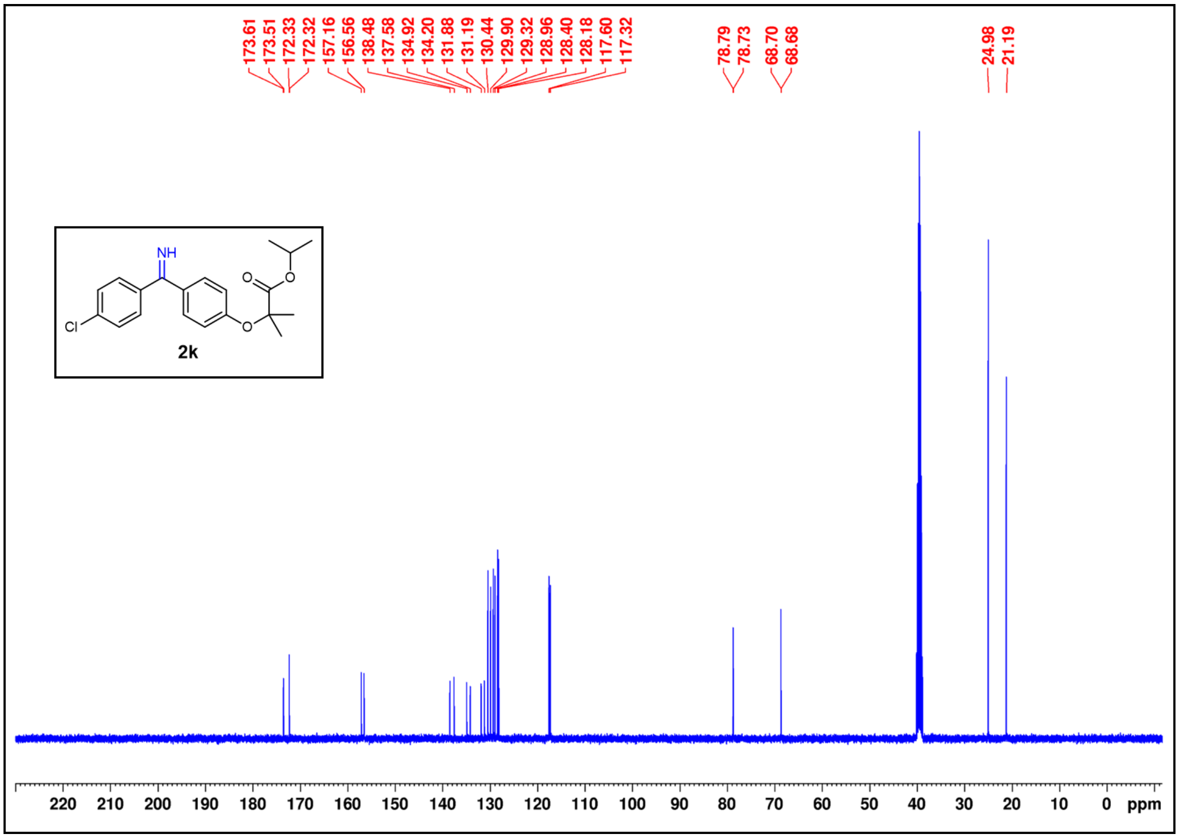

NOESY (400 MHz, DMSO-d_6) spectrum for **2k**

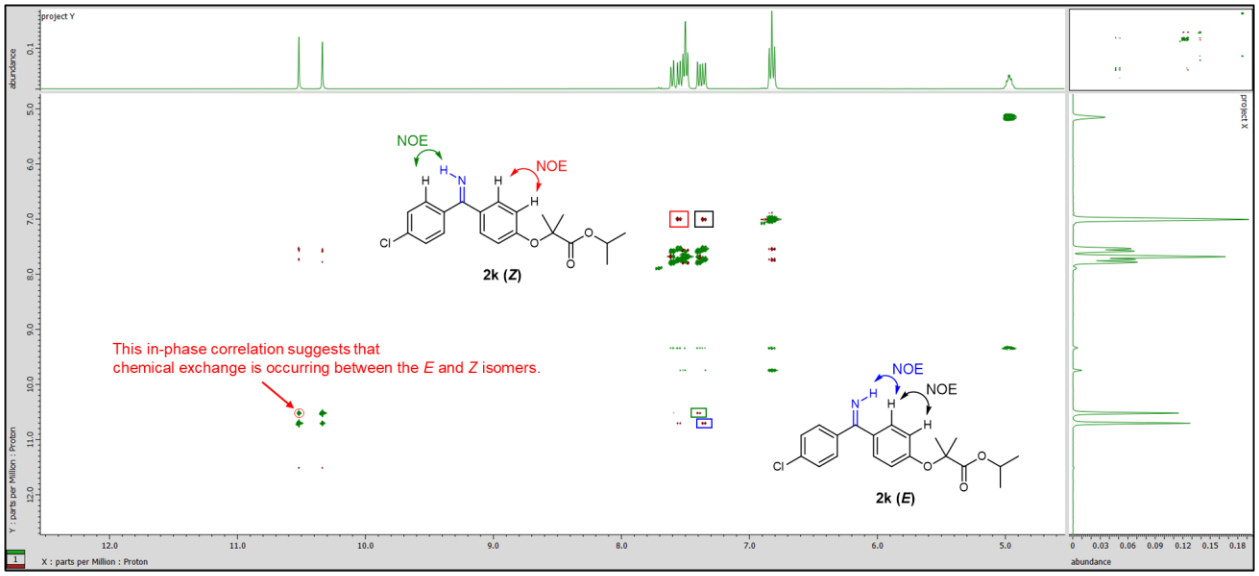

COSY (400 MHz, DMSO-d_6) spectrum for **2k**

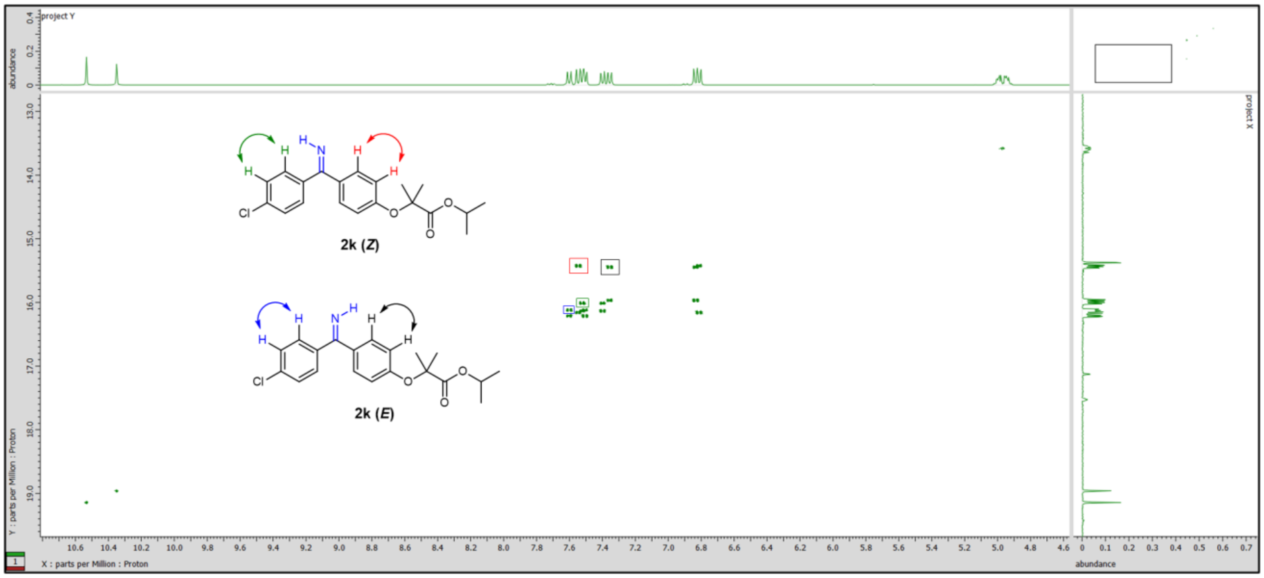

FT-IR (ATR, neat) and HRMS (ESI-positive) spectra for **2k**

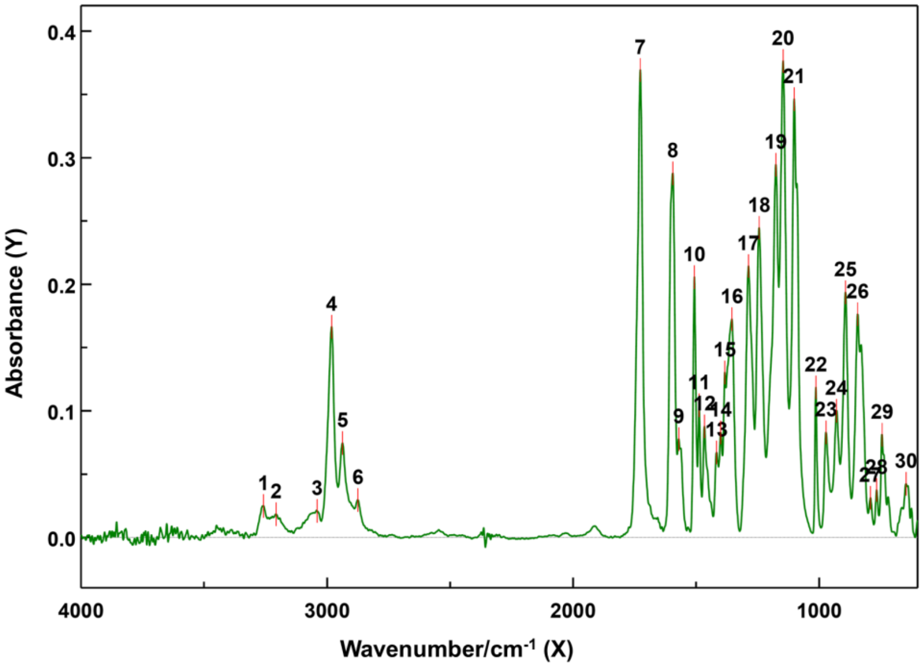

No.	X	Y	No.	X	Y	No.	X	Y
1	3259.1	0.025	11	1487.8	0.104	21	1101	0.346
2	3207	0.018	12	1466.6	0.087	22	1013	0.118
3	3040.2	0.021	13	1417.4	0.067	23	971.9	0.083
4	2981.4	0.166	14	1400.1	0.083	24	929.5	0.1
5	2937.1	0.074	15	1383.7	0.13	25	892.9	0.194
6	2874.4	0.029	16	1354.7	0.172	26	843.7	0.176
7	1726.9	0.369	17	1287.3	0.214	27	791.6	0.031
8	1594.8	0.288	18	1243.9	0.244	28	766.6	0.037
9	1570.7	0.077	19	1176.4	0.294	29	744.4	0.081
10	1507.1	0.206	20	1146.5	0.376	30	647	0.042

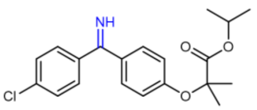

2k

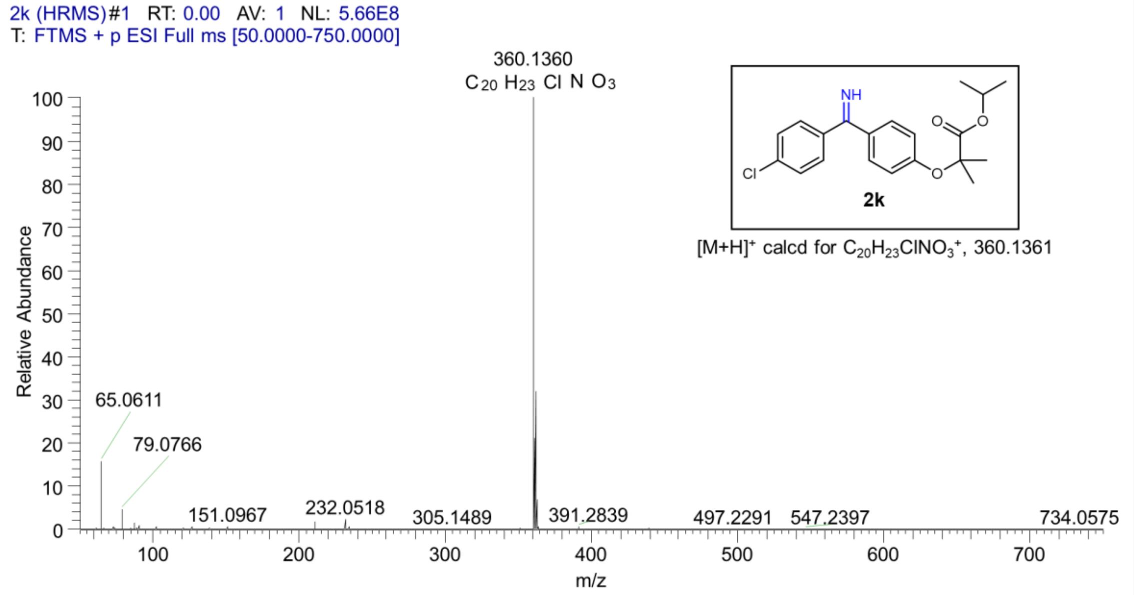

^{1}H NMR (400 MHz, DMSO-d_6) and ^{13}C NMR (100 MHz, DMSO-d_6) spectra for **2l**

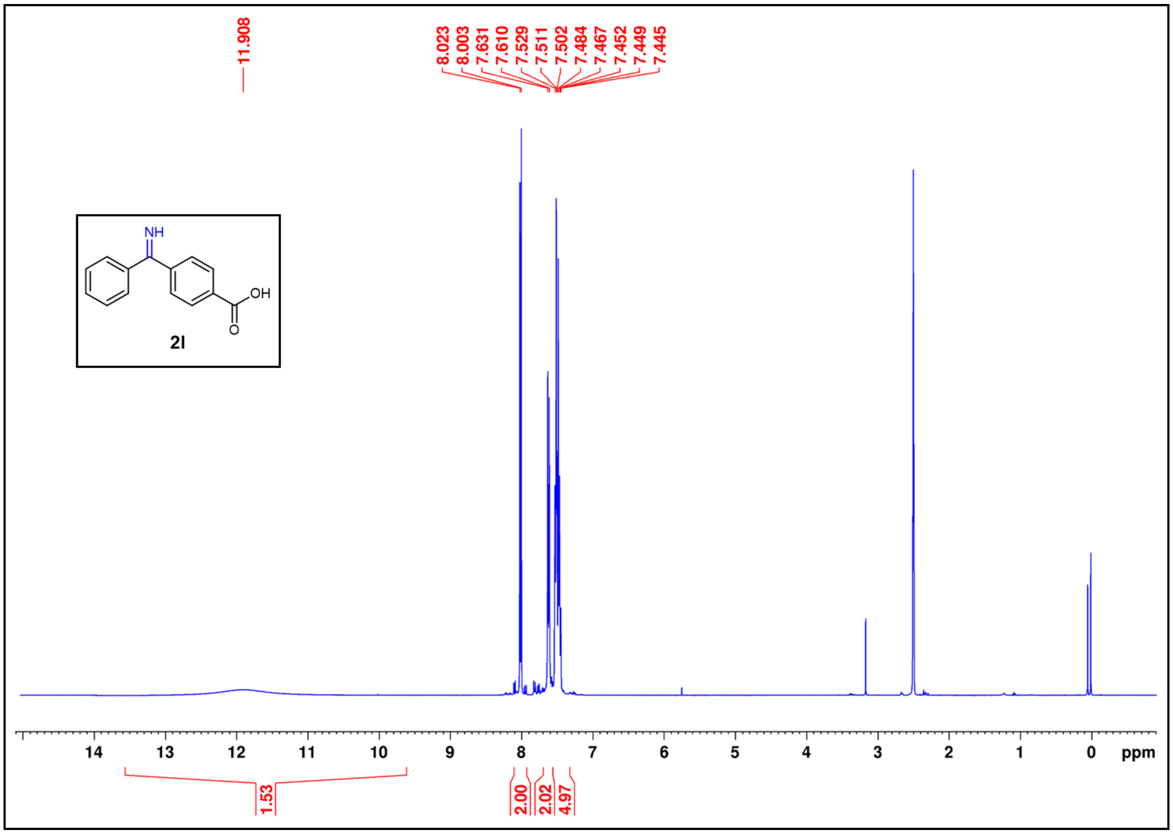

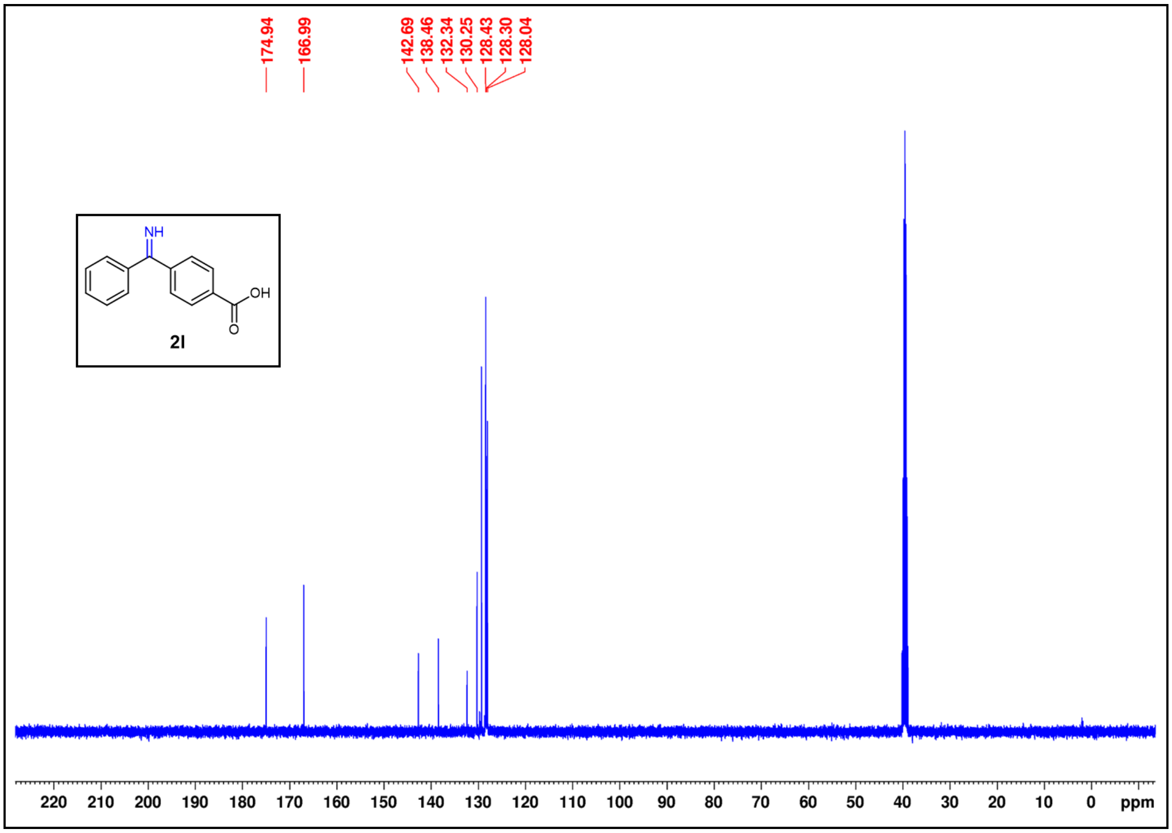

FT-IR (ATR, neat) and HRMS (ESI-positive) spectra for **2l**

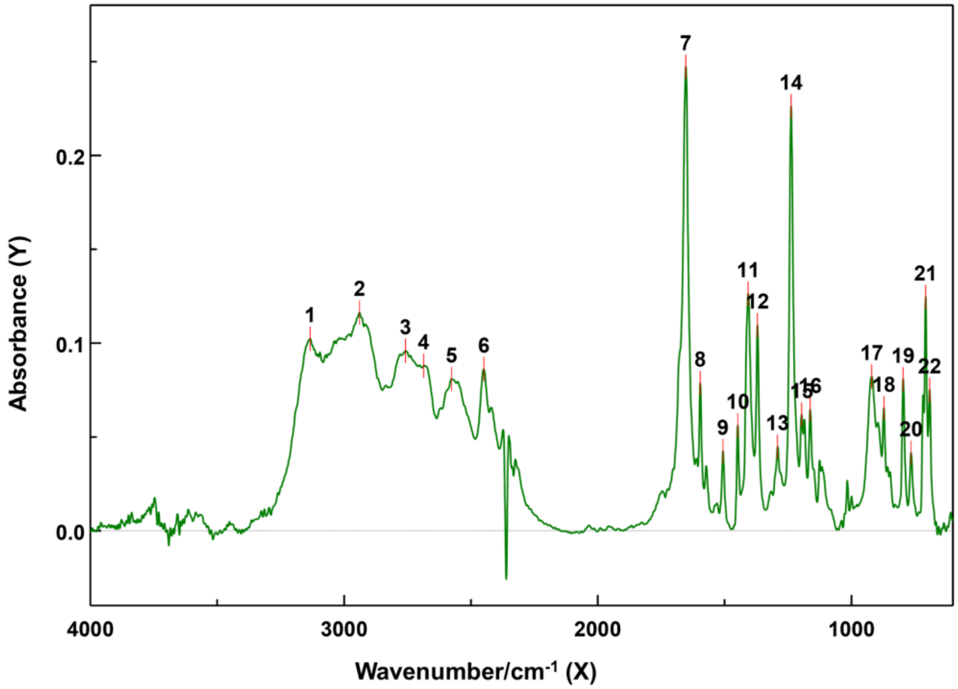

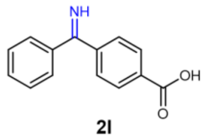

2l

No.	X	Y	No.	X	Y	No.	X	Y
1	3133.8	0.102	9	1506.1	0.042	17	919.9	0.082
2	2939	0.116	10	1448.3	0.056	18	872.6	0.065
3	2757.7	0.096	11	1406.8	0.126	19	796.5	0.081
4	2686.4	0.088	12	1370.2	0.109	20	764.6	0.041
5	2576.4	0.081	13	1291.1	0.045	21	707.7	0.124
6	2449.2	0.086	14	1238.1	0.226	22	691.4	0.075
7	1652.7	0.247	15	1196.6	0.062			
8	1595.8	0.079	16	1162.9	0.064			

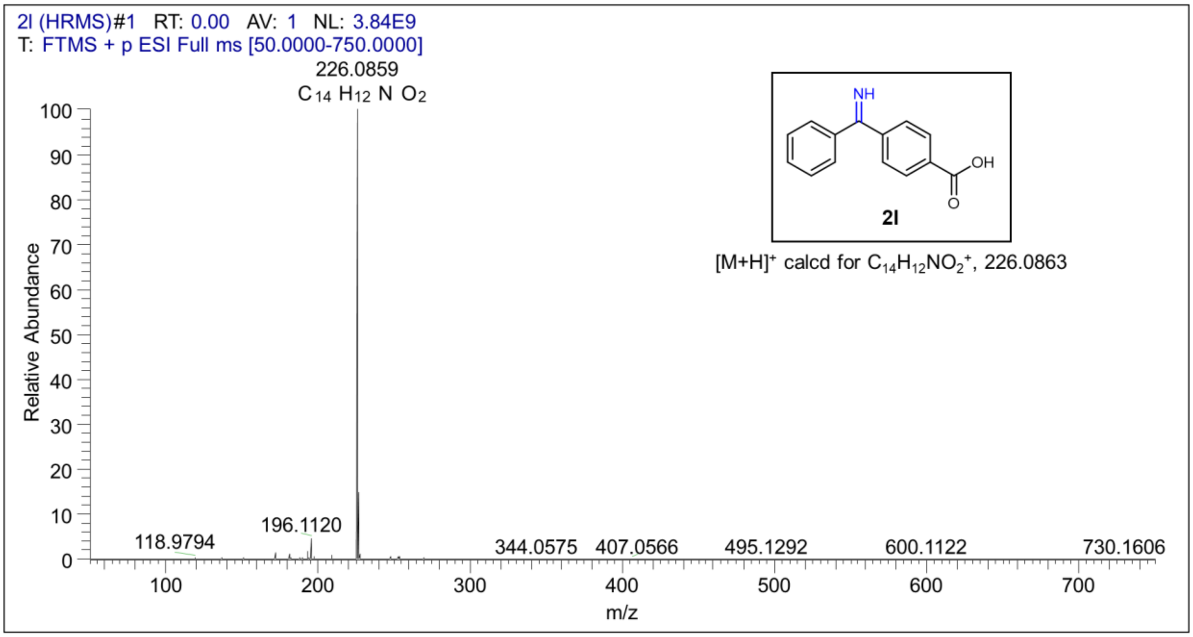

[M+H]+ calcd for $C_{14}H_{12}NO_2^+$, 226.0863

^{1}H NMR (400 MHz, DMSO-d_6) and ^{13}C NMR (100 MHz, DMSO-d_6) spectra for **2m**

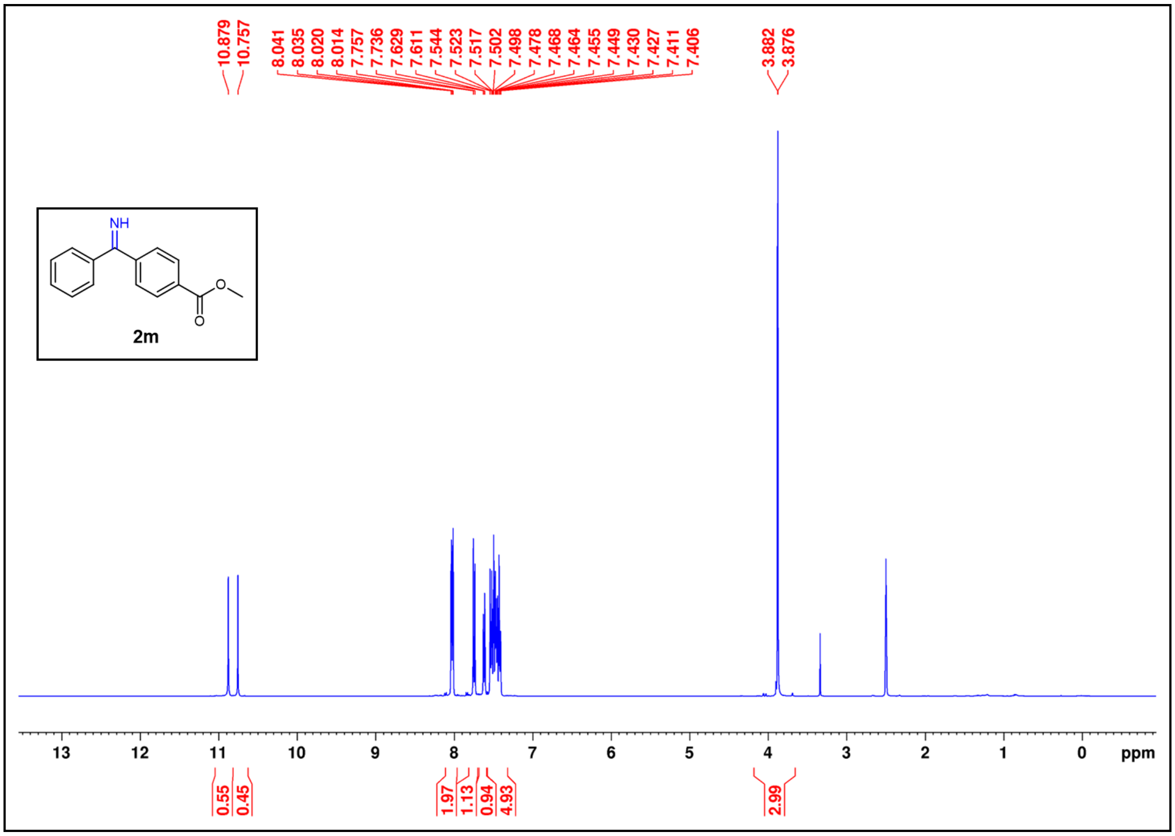

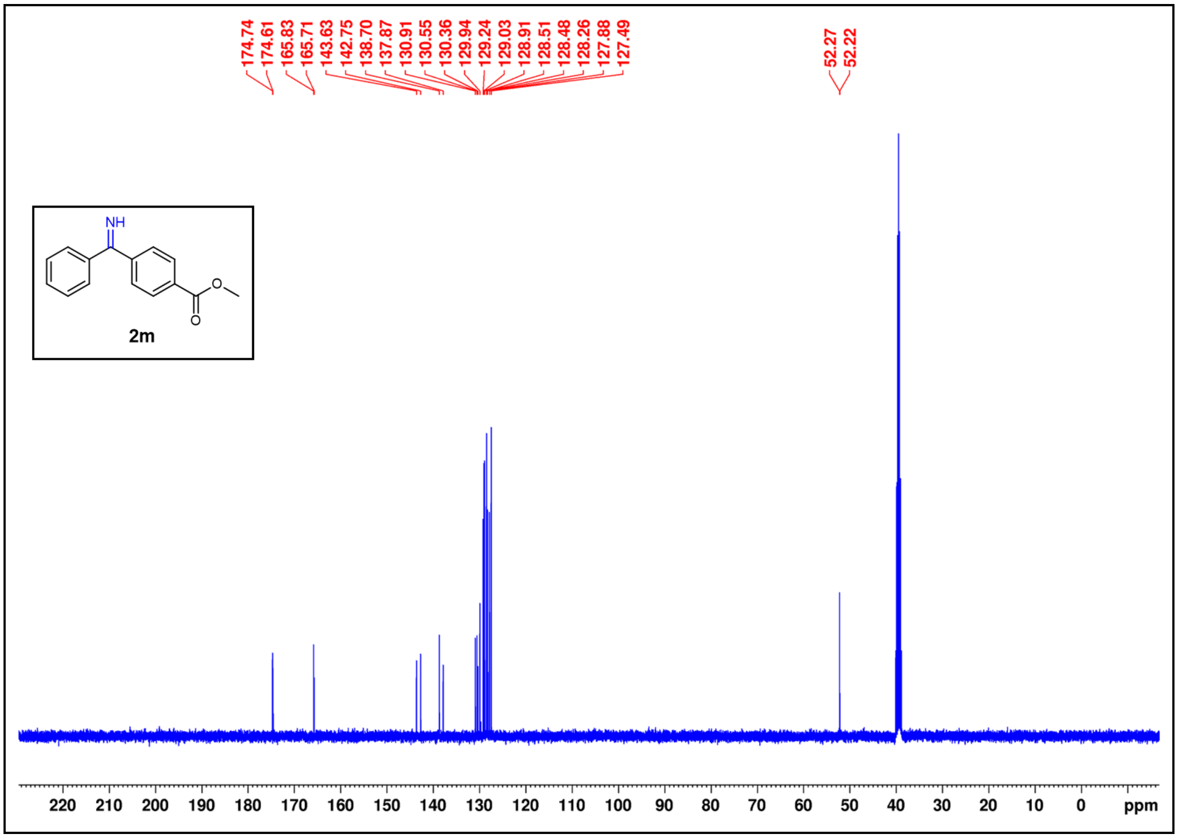

FT-IR (ATR, neat) and HRMS (ESI-positive) spectra for **2m**

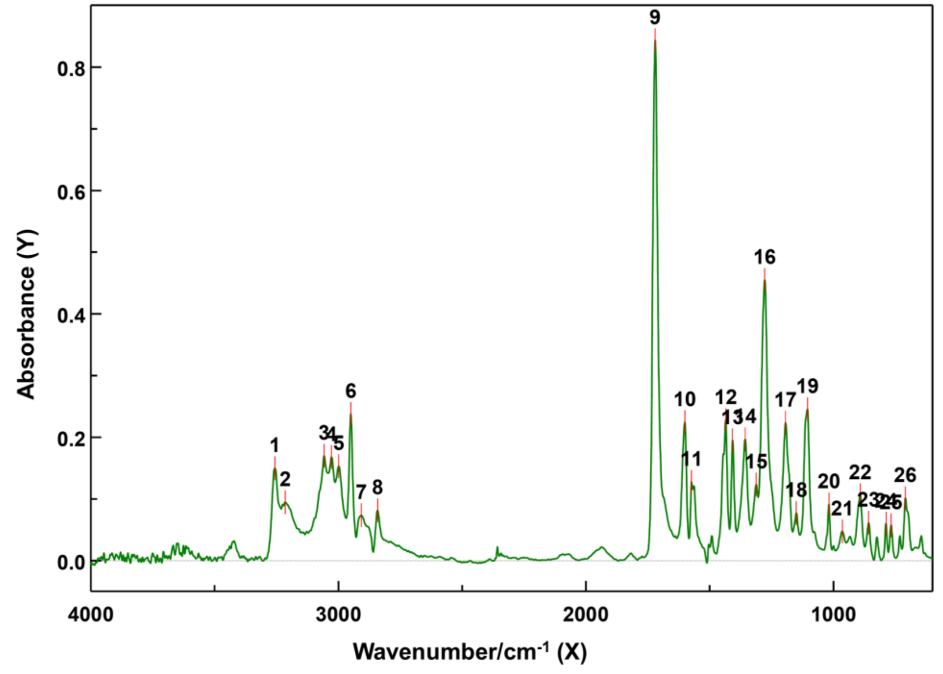

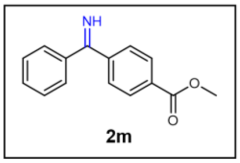

No.	X	Y	No.	X	Y	No.	X	Y
1	3256.2	0.149	10	1600.6	0.224	19	1105	0.245
2	3214.8	0.094	11	1573.6	0.127	20	1018	0.091
3	3057.6	0.169	12	1435.7	0.227	21	964.2	0.047
4	3027.7	0.167	13	1407.8	0.195	22	891.9	0.106
5	2998.8	0.153	14	1356.7	0.196	23	858.2	0.061
6	2949.6	0.238	15	1312.3	0.123	24	787.8	0.06
7	2907.2	0.073	16	1277.6	0.455	25	767.5	0.057
8	2841.6	0.081	17	1193.7	0.223	26	709.7	0.101
9	1720.2	0.843	18	1150.3	0.077			

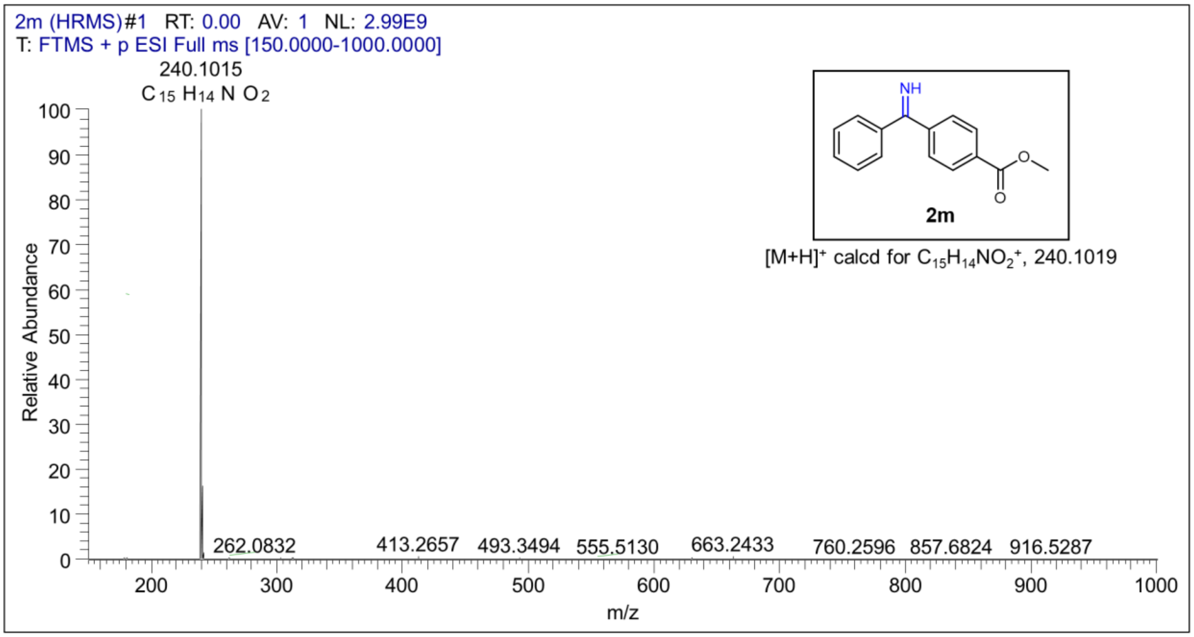

^{1}H NMR (400 MHz, DMSO-d_6) and ^{13}C NMR (100 MHz, DMSO-d_6) spectra for **2n**

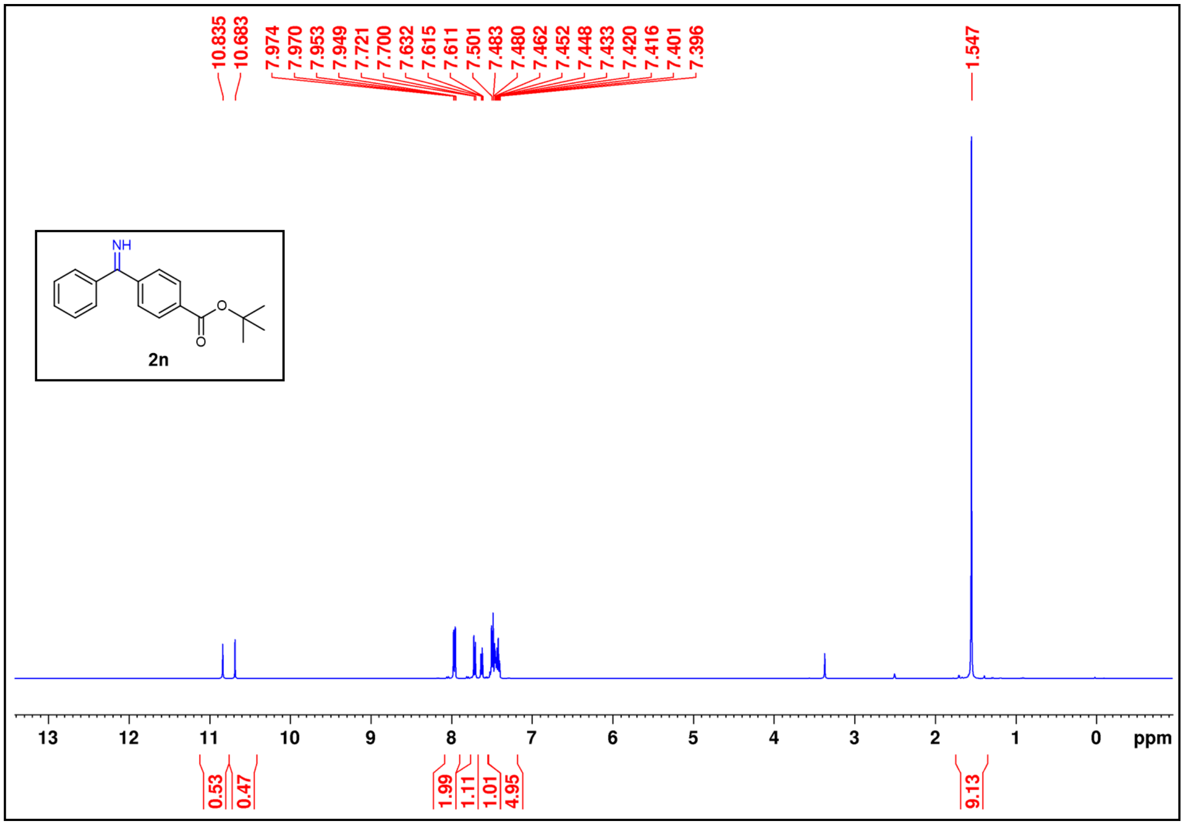

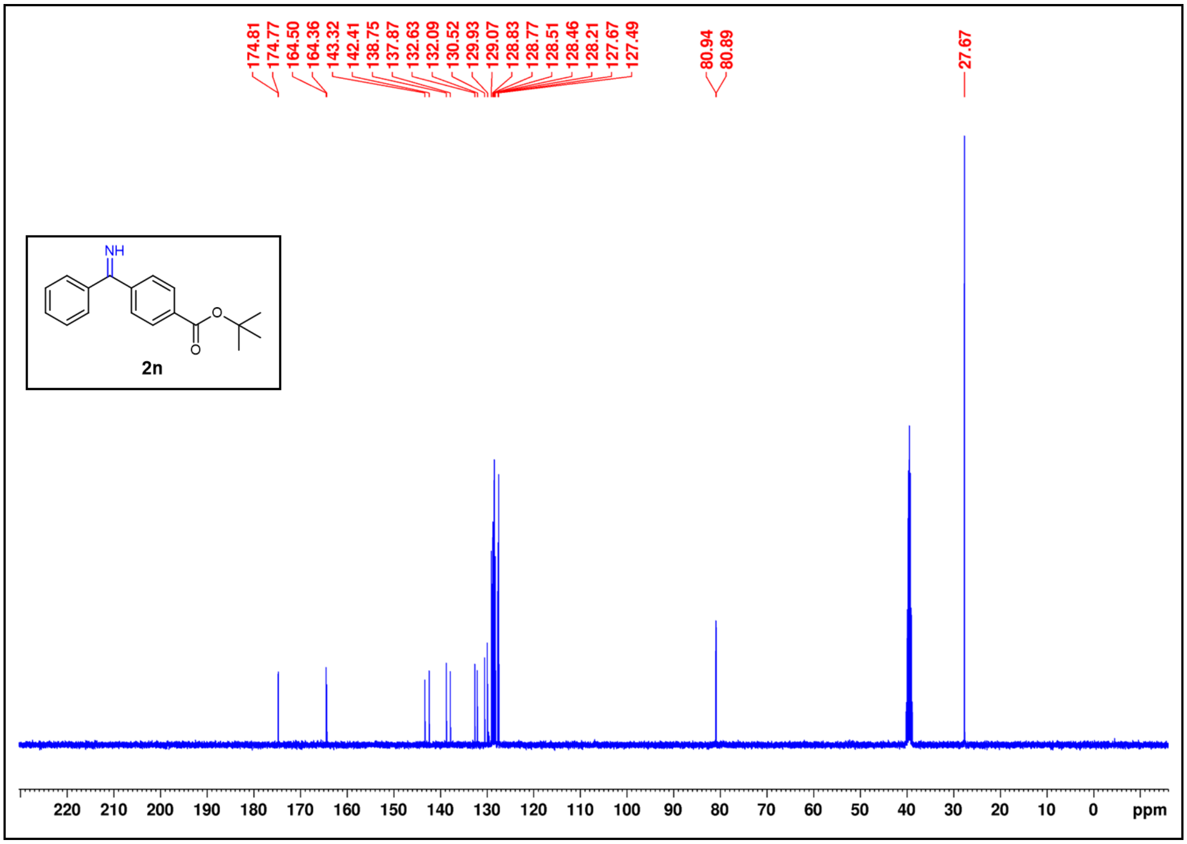

FT-IR (ATR, neat) and HRMS (ESI-positive) spectra for **2n**

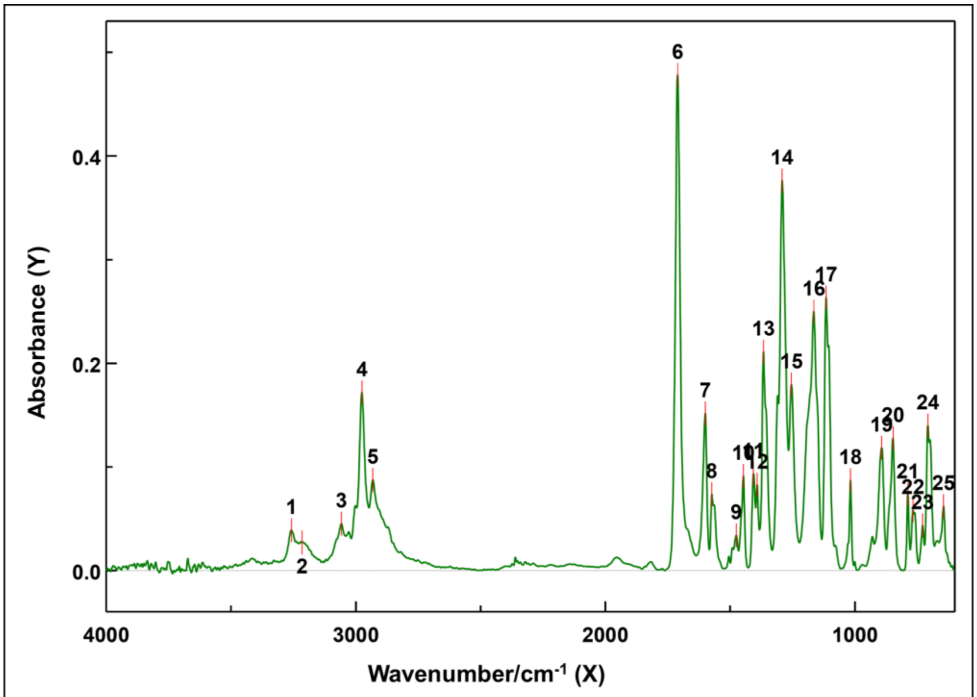

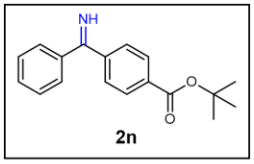

No.	X	Y	No.	X	Y	No.	X	Y
1	3258.1	0.039	10	1447.3	0.091	19	892.9	0.118
2	3215.7	0.027	11	1406.8	0.093	20	847.6	0.127
3	3057.6	0.045	12	1392.4	0.083	21	787.8	0.073
4	2975.6	0.172	13	1366.3	0.211	22	767.5	0.058
5	2932.2	0.087	14	1292.1	0.376	23	729	0.043
6	1710.6	0.478	15	1254.5	0.179	24	707.7	0.14
7	1599.7	0.151	16	1164.8	0.25	25	645.1	0.062
8	1573.6	0.073	17	1115.6	0.263			
9	1475.3	0.034	18	1018.2	0.087			

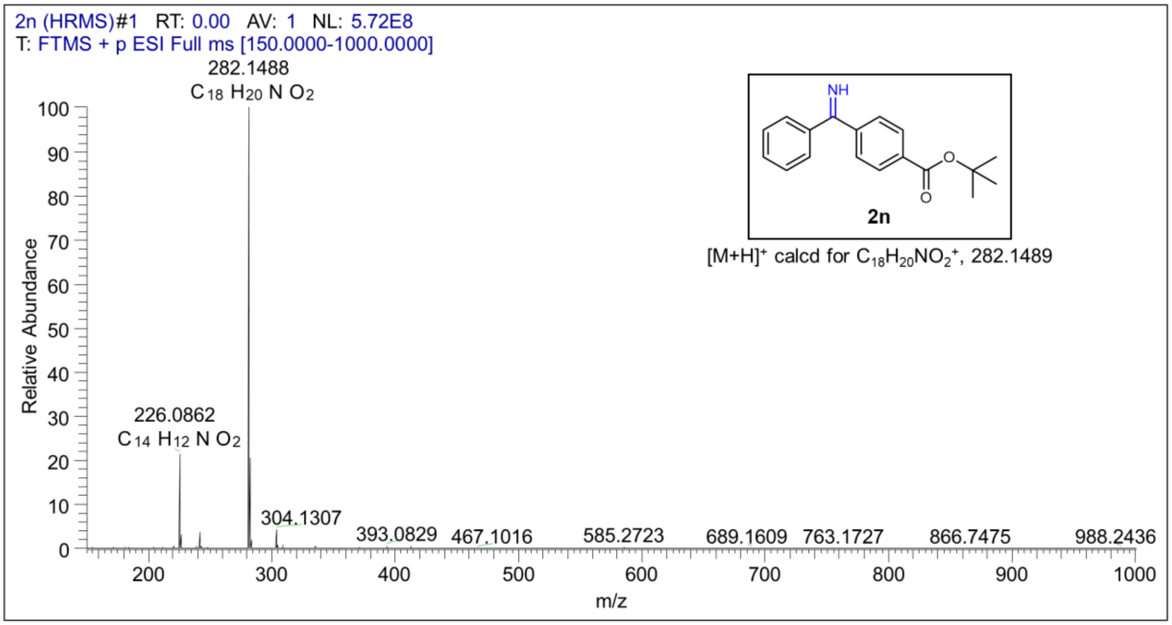

^{1}H NMR (400 MHz, $CDCl_3$) and ^{13}C NMR (100 MHz, $CDCl_3$) spectra for **2o**

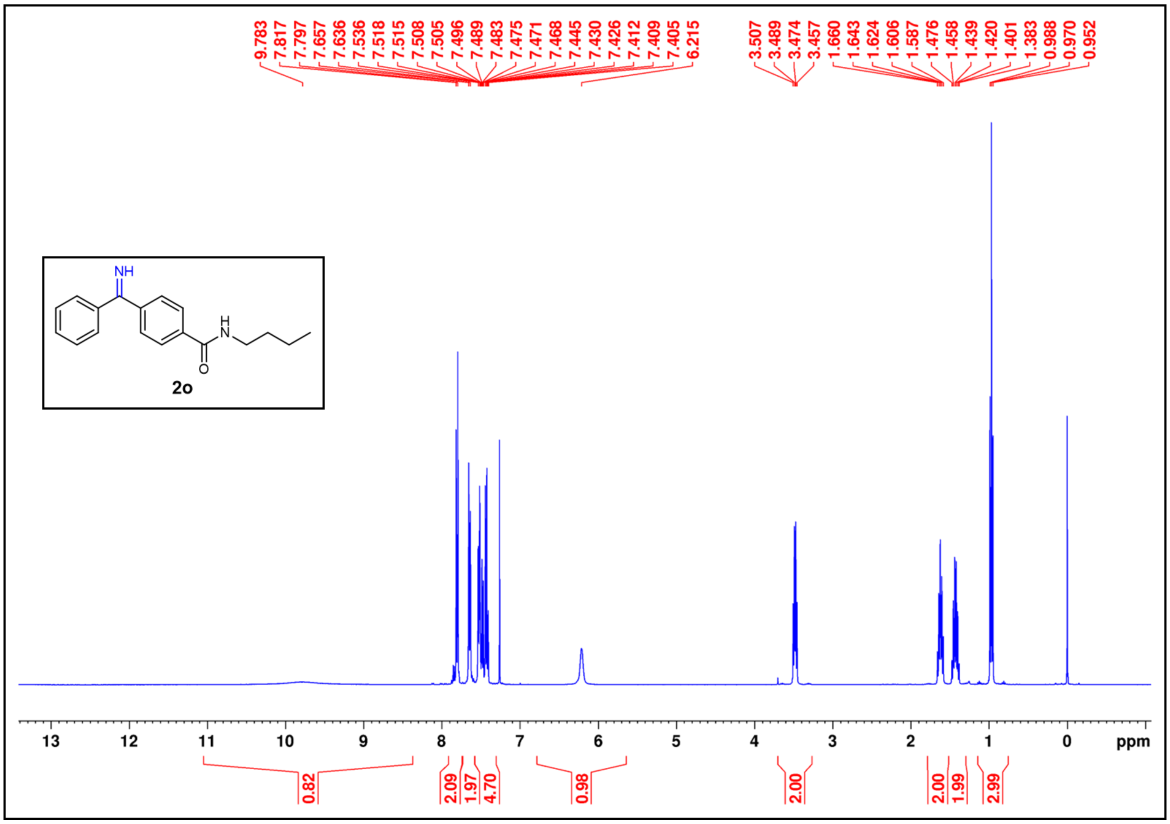

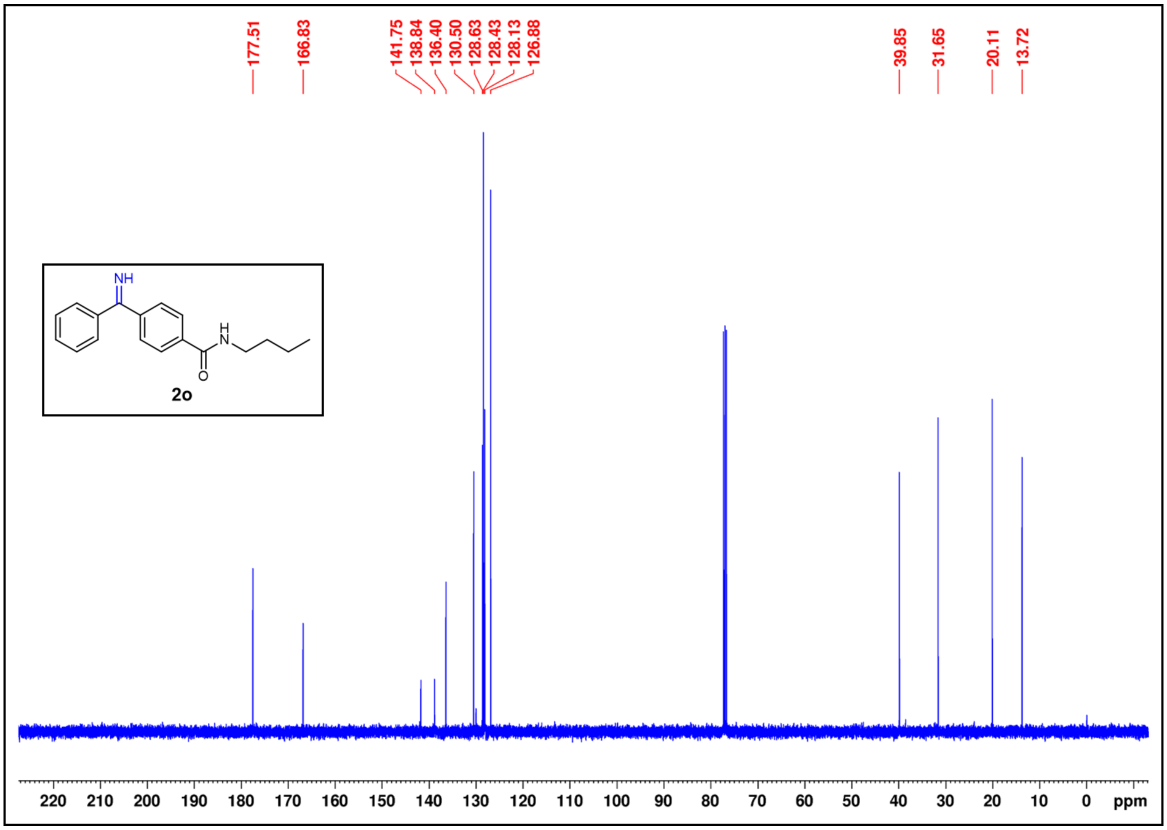

FT-IR (ATR, neat) and HRMS (ESI-positive) spectra for **2o**

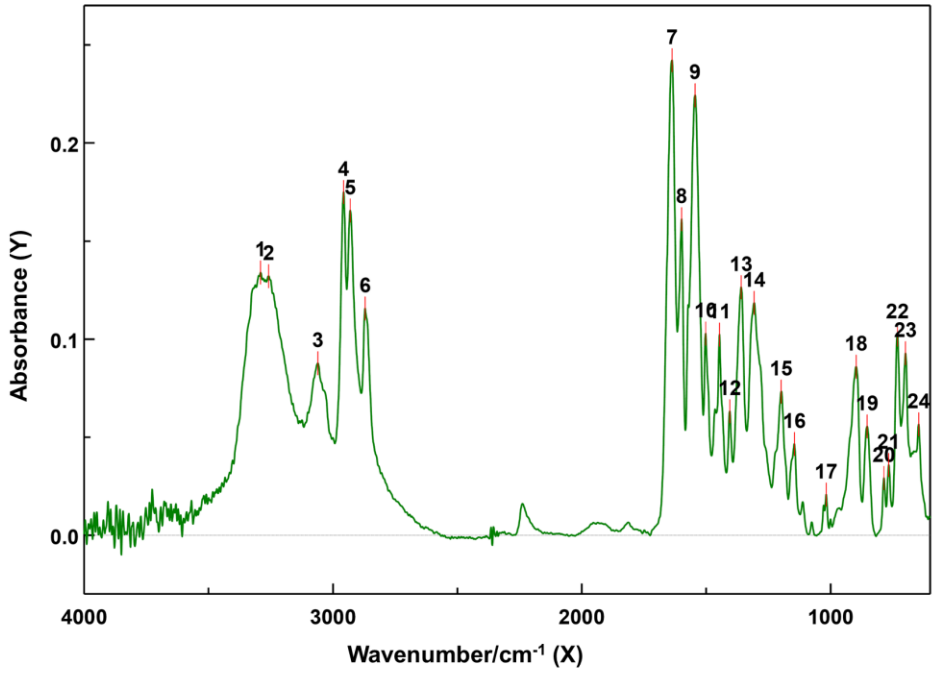

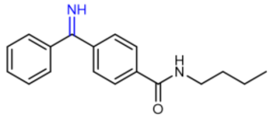

2o

No.	X	Y	No.	X	Y	No.	X	Y
1	3290.9	0.134	9	1544.7	0.224	17	1018	0.021
2	3259.1	0.132	10	1502.3	0.103	18	897.7	0.086
3	3060.5	0.088	11	1446.4	0.102	19	853.3	0.055
4	2957.3	0.175	12	1405.9	0.063	20	785.9	0.029
5	2930.3	0.166	13	1359.6	0.126	21	766.6	0.036
6	2870.5	0.115	14	1307.5	0.118	22	731.9	0.102
7	1637.3	0.242	15	1198.5	0.073	23	699.1	0.093
8	1598.7	0.161	16	1145.5	0.046	24	646	0.056

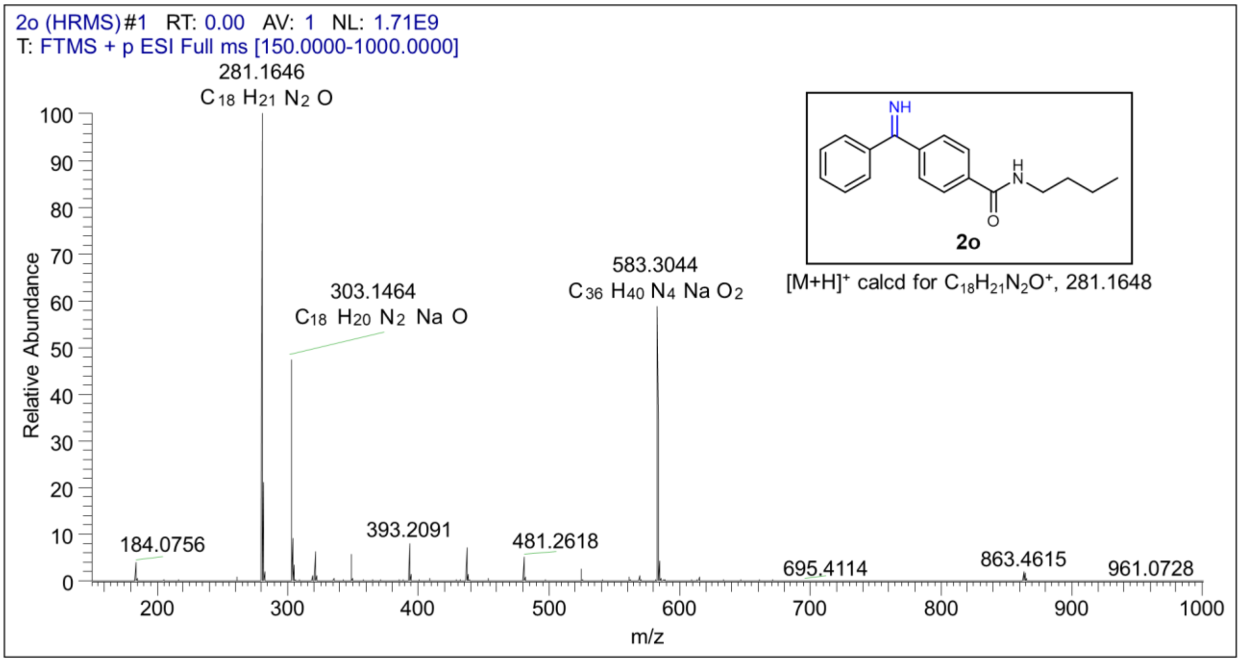

^{1}H NMR (400 MHz, $CDCl_3$) and ^{13}C NMR (100 MHz, $CDCl_3$) spectra for **2p**

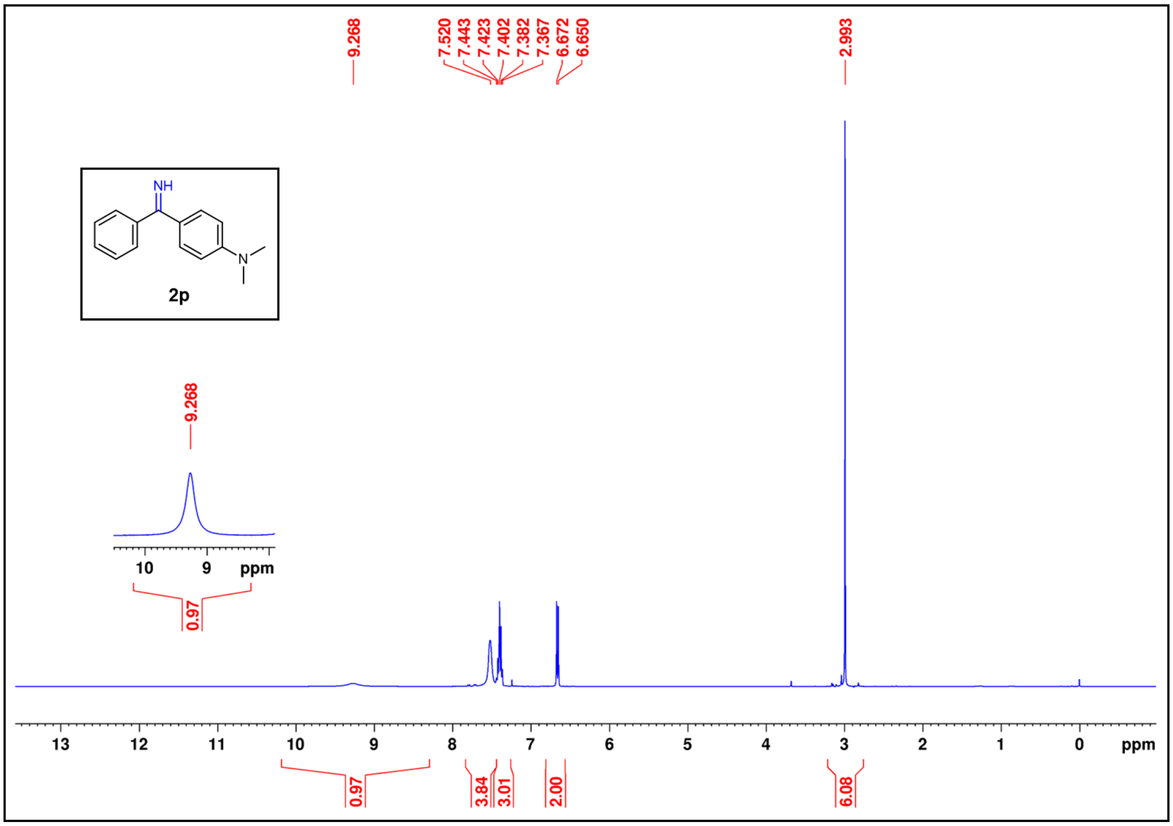

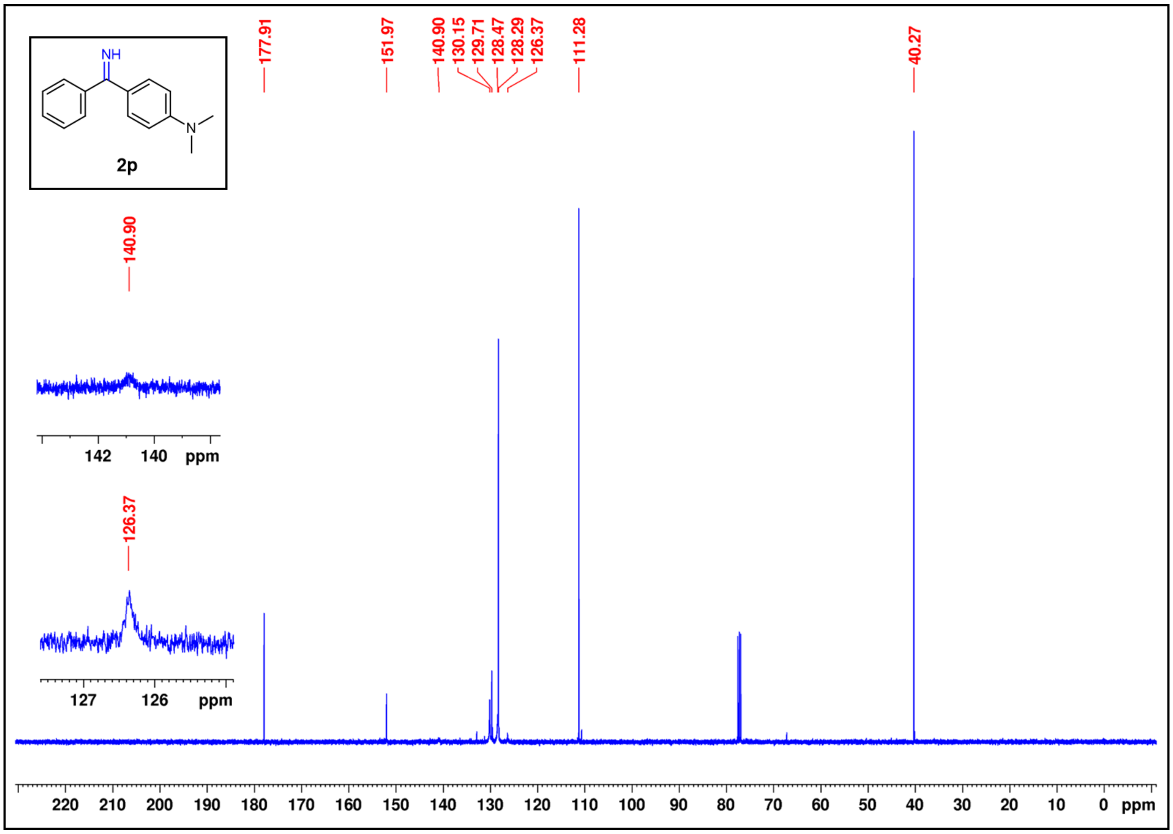

FT-IR (ATR, neat) and HRMS (ESI-positive) spectra for **2p**

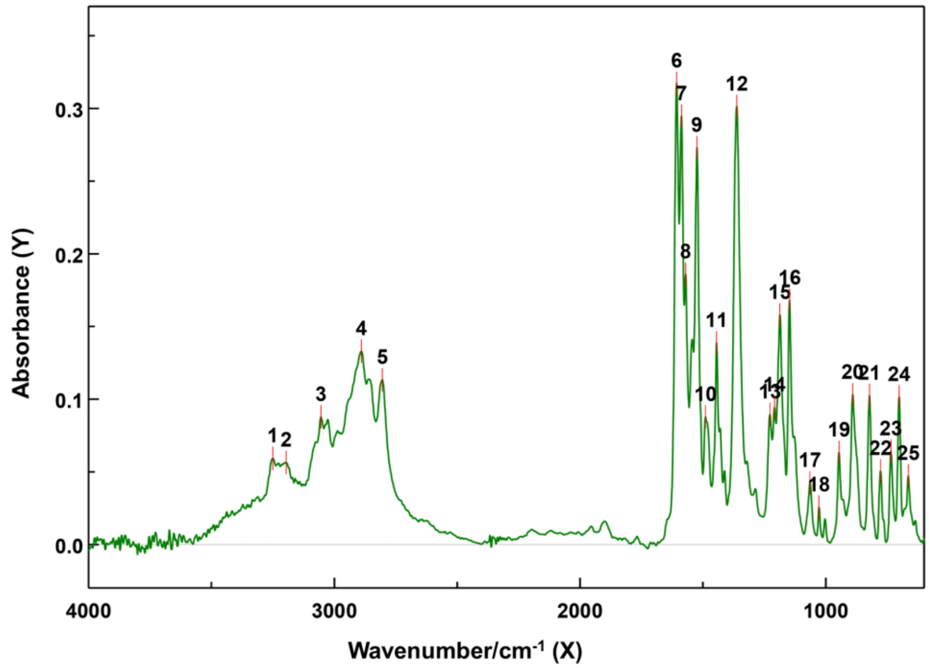

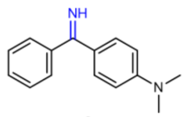

2p

No.	X	Y	No.	X	Y	No.	X	Y
1	3250.4	0.059	10	1489.7	0.088	19	945.9	0.063
2	3196.4	0.056	11	1444.4	0.138	20	891	0.103
3	3054.7	0.088	12	1362.5	0.301	21	822.5	0.102
4	2889.8	0.133	13	1227.5	0.089	22	777.2	0.051
5	2805	0.113	14	1209.1	0.094	23	734.7	0.064
6	1607.4	0.317	15	1187	0.158	24	702	0.101
7	1587.1	0.294	16	1147.4	0.168	25	664.4	0.047
8	1570.7	0.186	17	1064.5	0.042			
9	1524.5	0.273	18	1027.9	0.026			

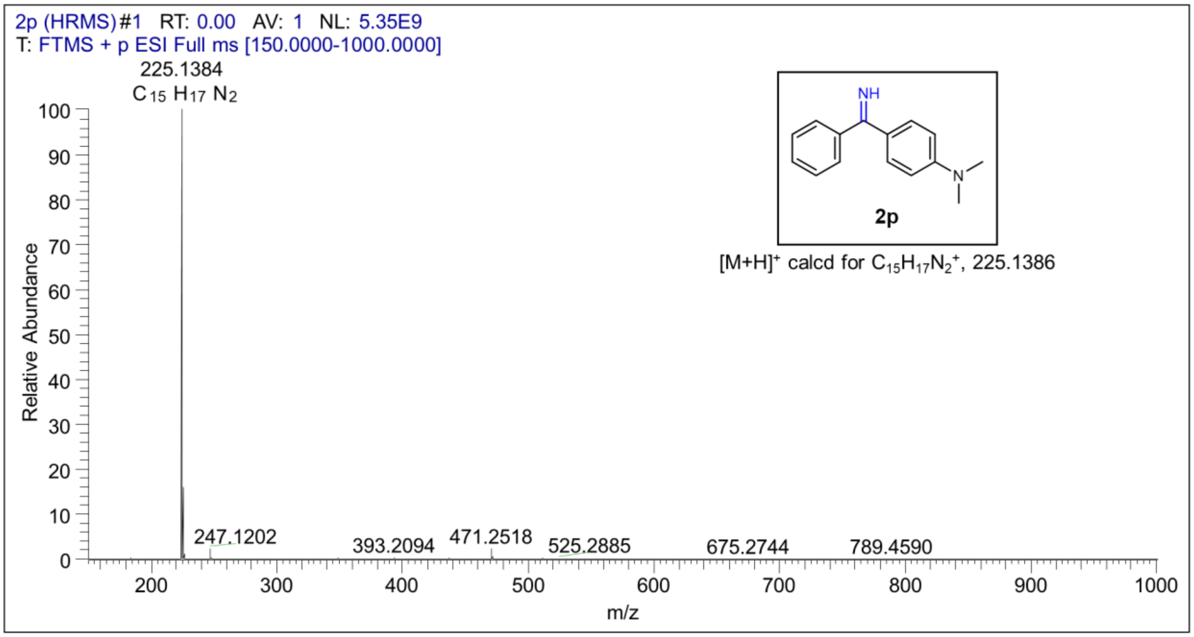

^{1}H NMR (400 MHz, DMSO-d_6) and ^{13}C NMR (100 MHz, DMSO-d_6) spectra for **2q**

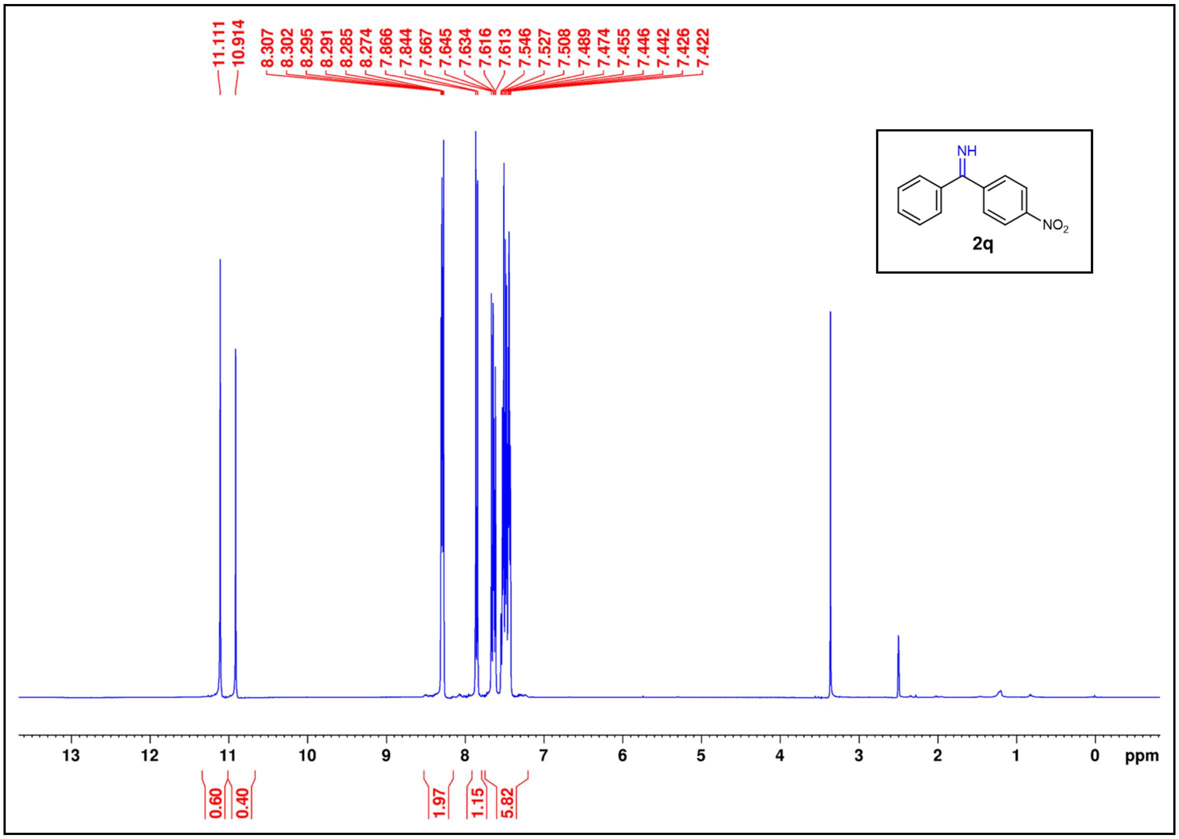

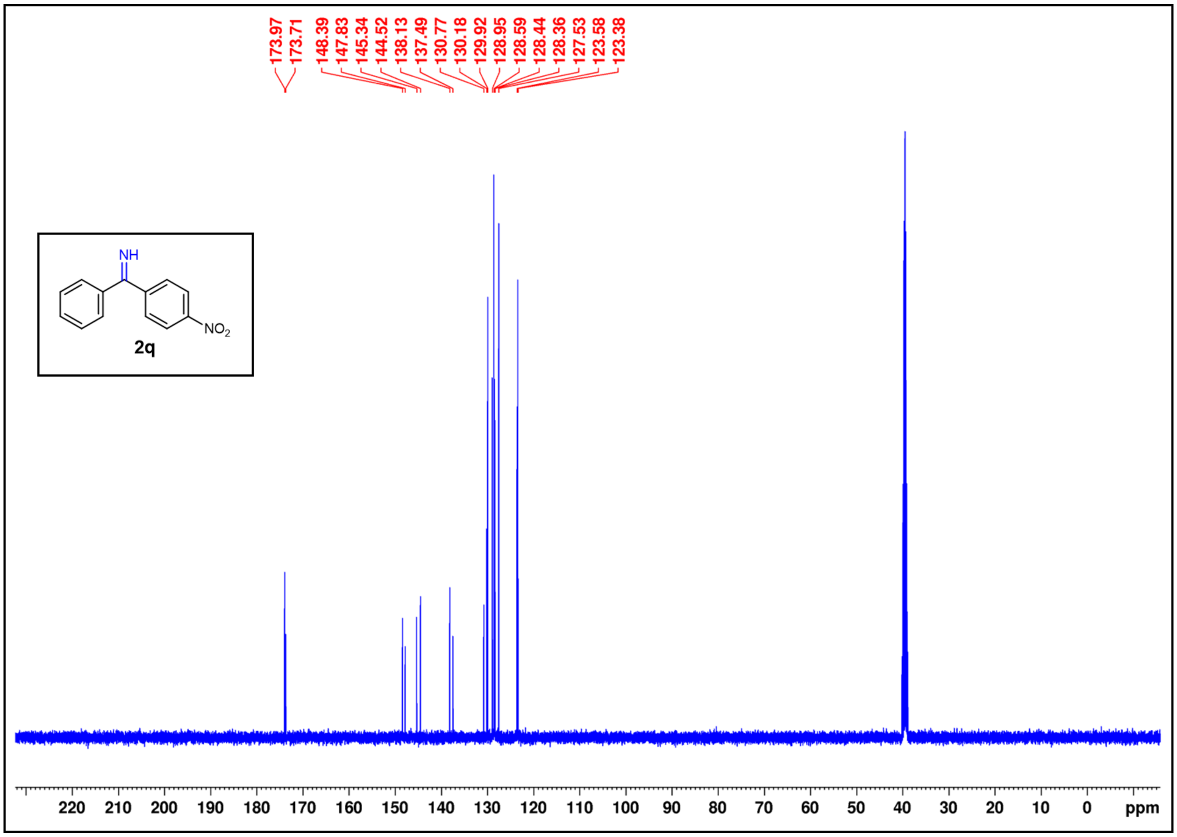

NOESY (400 MHz, DMSO-d_6) spectrum for **2q**

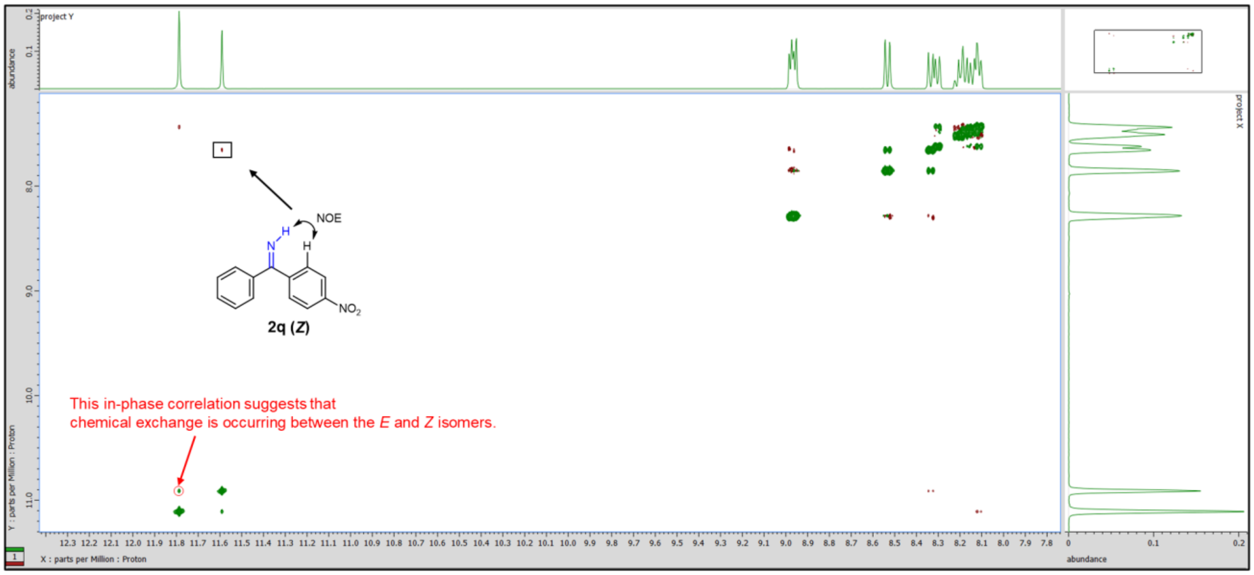

COSY (400 MHz, DMSO-d_6) spectrum for **2q**

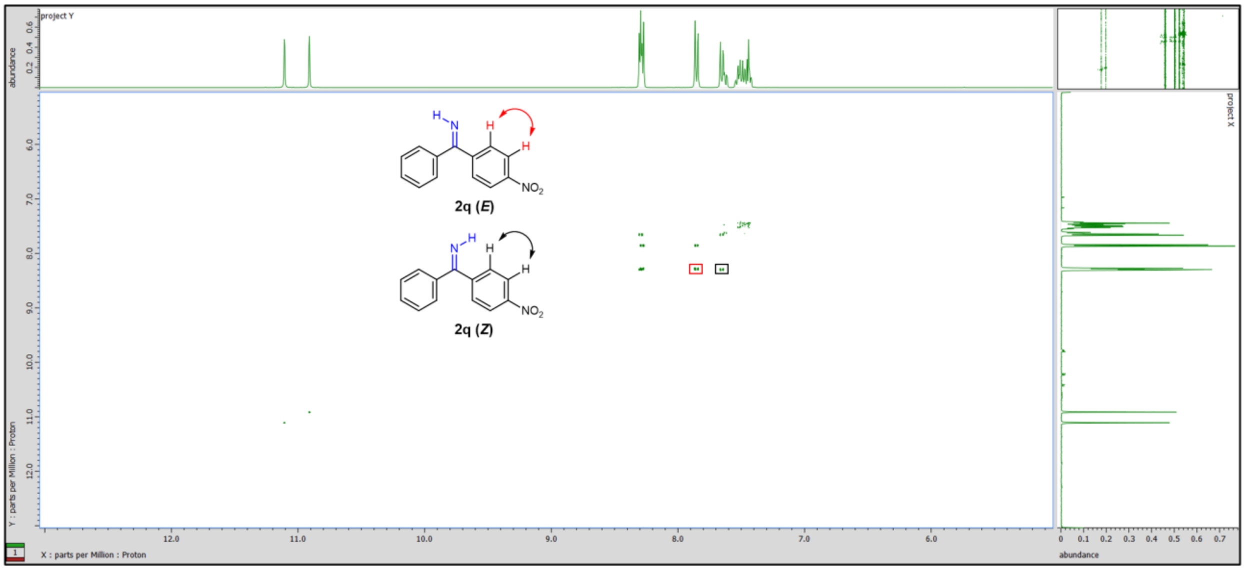

FT-IR (ATR, neat) and HRMS (ESI-positive) spectra for **2q**

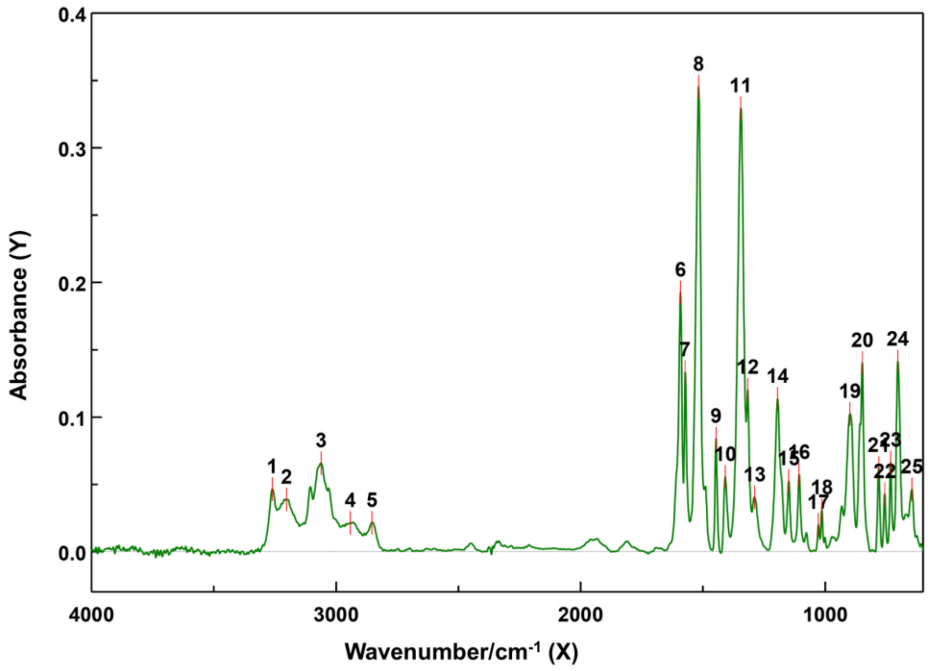

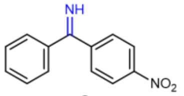

2q

No.	X	Y	No.	X	Y	No.	X	Y
1	3261	0.046	10	1408.7	0.055	19	898.7	0.102
2	3203.2	0.039	11	1345.1	0.329	20	848.5	0.14
3	3061.4	0.066	12	1317.1	0.12	21	781	0.062
4	2941.9	0.021	13	1289.2	0.04	22	756.9	0.043
5	2854.1	0.021	14	1194.7	0.113	23	732.8	0.066
6	1591.9	0.192	15	1149.4	0.052	24	702.9	0.141
7	1572.7	0.133	16	1106.9	0.057	25	646	0.046
8	1517.7	0.345	17	1027.9	0.02			
9	1446.4	0.084	18	1014.4	0.031			

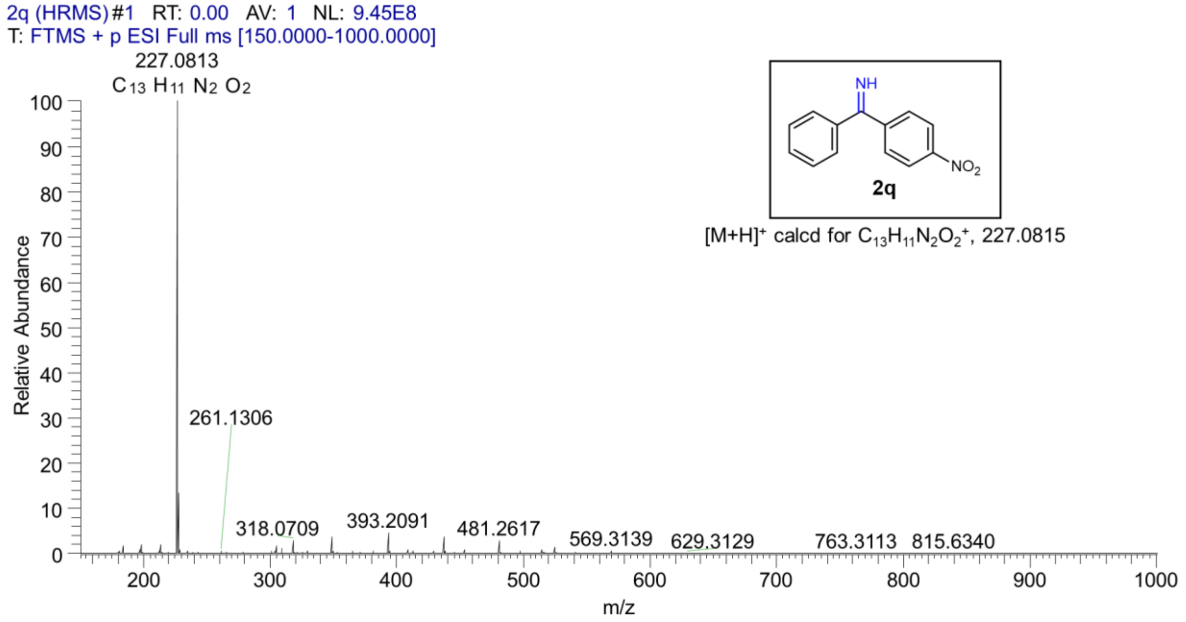

$[M+H]^+$ calcd for $C_{13}H_{11}N_2O_2^+$, 227.0815

^{1}H NMR (400 MHz, DMSO-d_6) and ^{13}C NMR (100 MHz, DMSO-d_6) spectra for **2q**·HCl

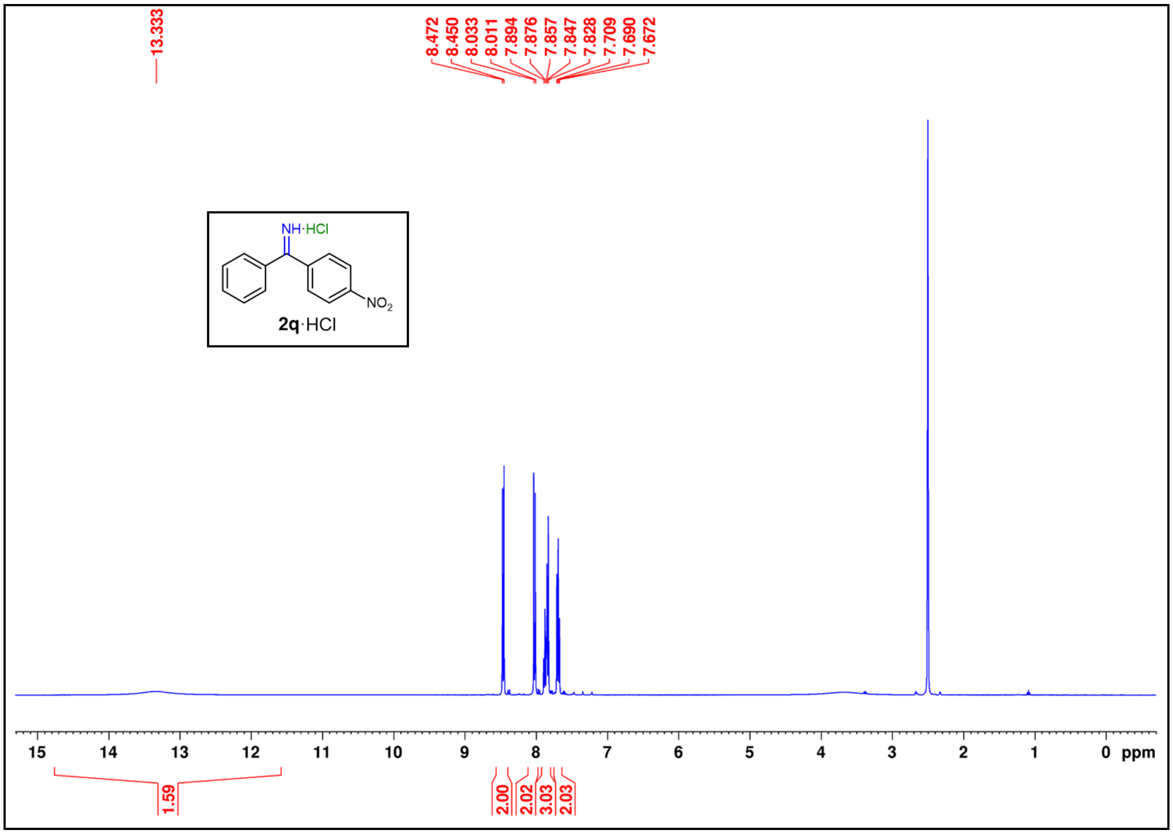

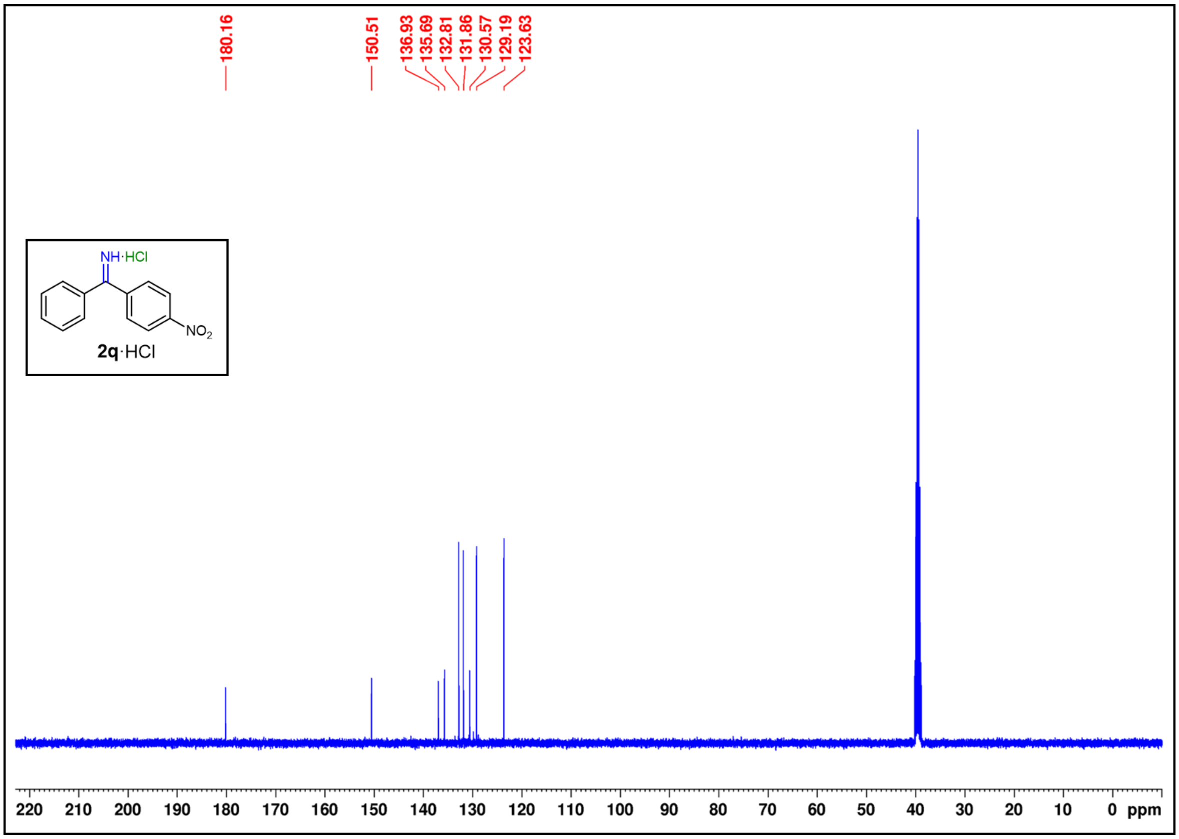

FT-IR (ATR, neat) and HRMS (ESI-positive) spectra for **2q**·HCl

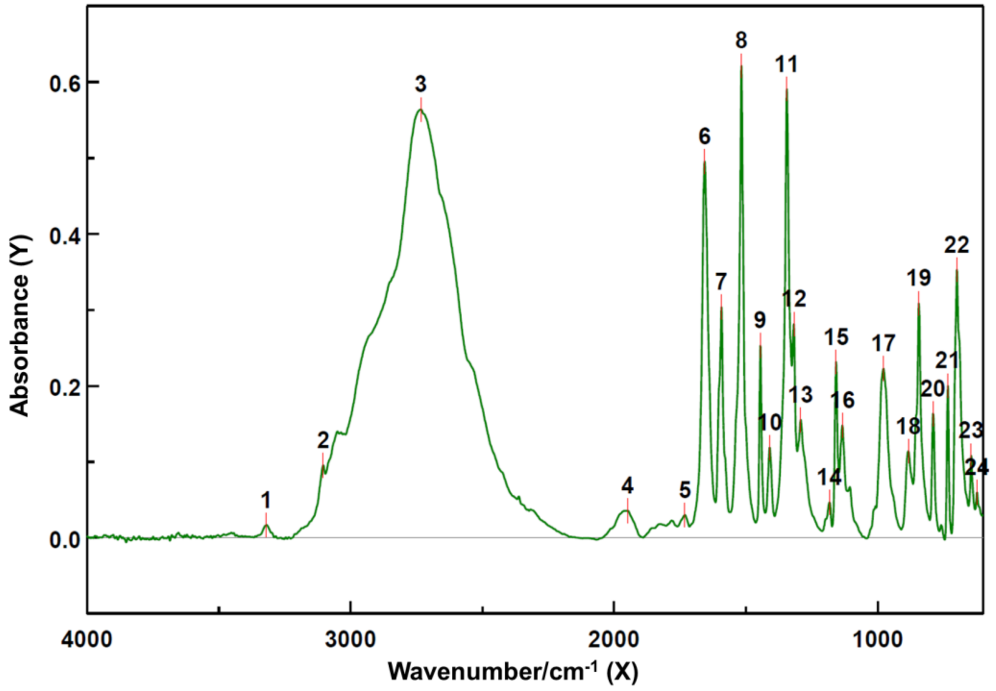

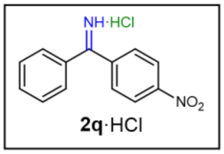

No.	X	Y	No.	X	Y	No.	X	Y
1	3318.9	0.016	2	3103.9	0.095	3	2734	0.563
4	1948.7	0.035	5	1731.8	0.03	6	1657	0.495
7	1592.9	0.304	8	1516.7	0.621	9	1445	0.253
10	1409.7	0.119	11	1344.1	0.591	12	1318	0.281
13	1292.1	0.155	14	1183.1	0.046	15	1158	0.231
16	1133.9	0.148	17	977.7	0.223	18	884.2	0.114
19	843.7	0.309	20	789.7	0.163	21	733.8	0.2
22	699.1	0.353	23	645.1	0.108	24	622.9	0.06

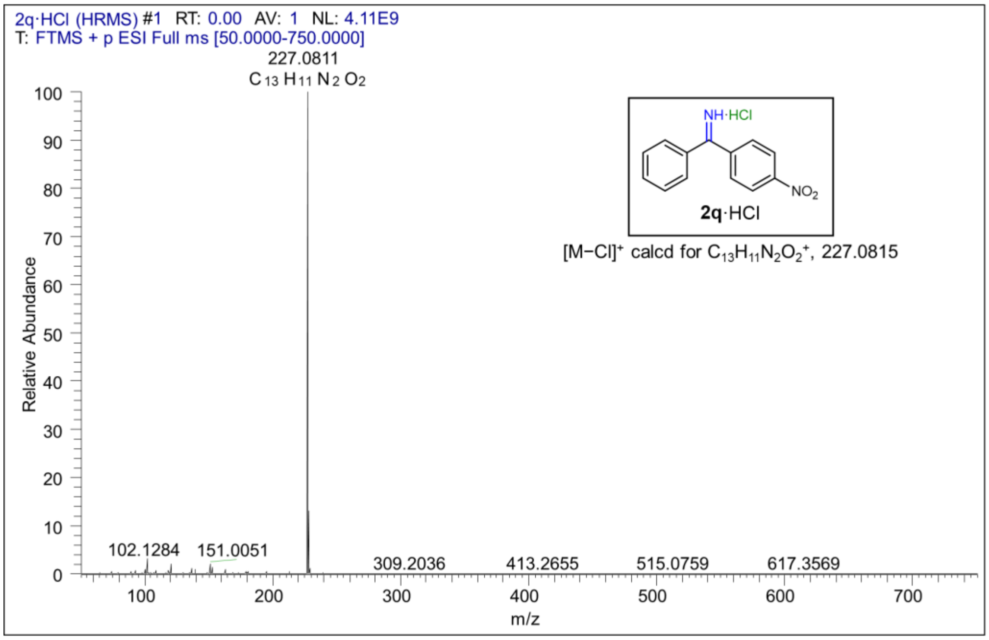

<u>^{1}H NMR (400 MHz, DMSO-d_6) and ^{13}C NMR (100 MHz, DMSO-d_6) spectra for **2r**·HCl</u>

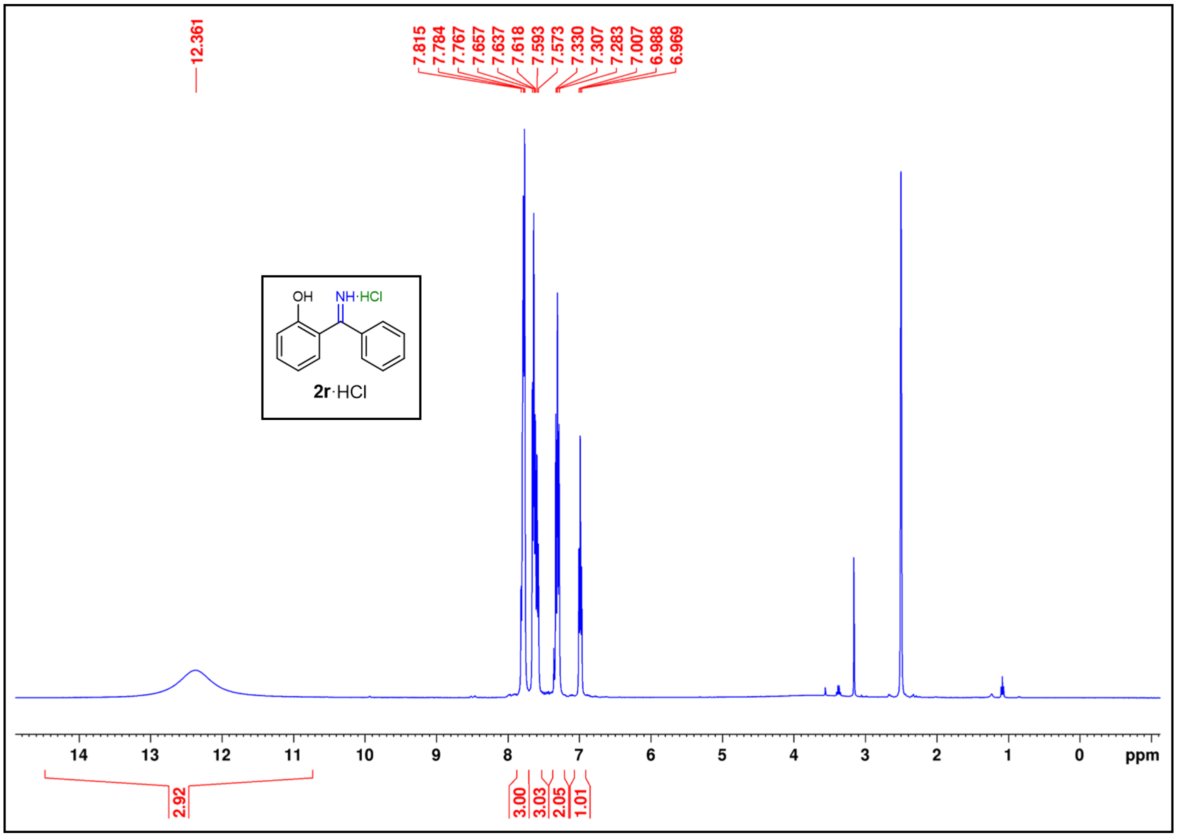

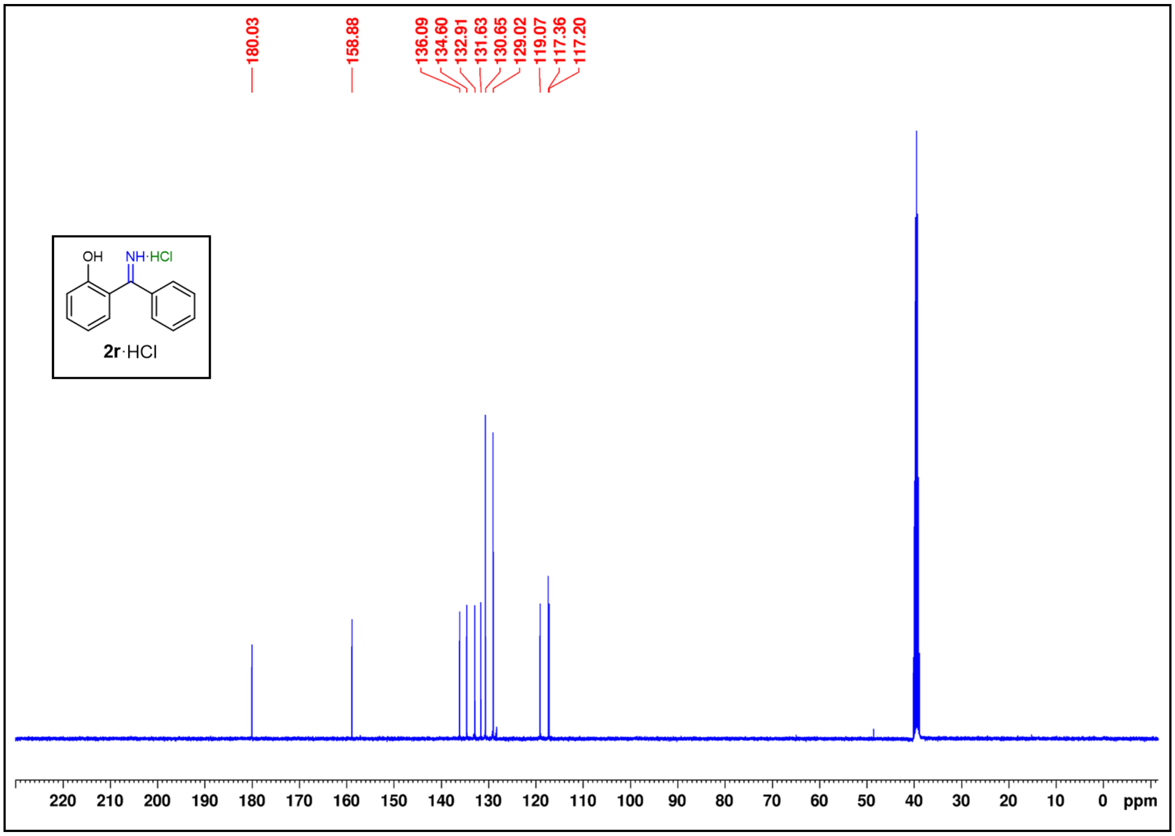

FT-IR (ATR, neat) and HRMS (ESI-positive) spectra for **2r**·HCl

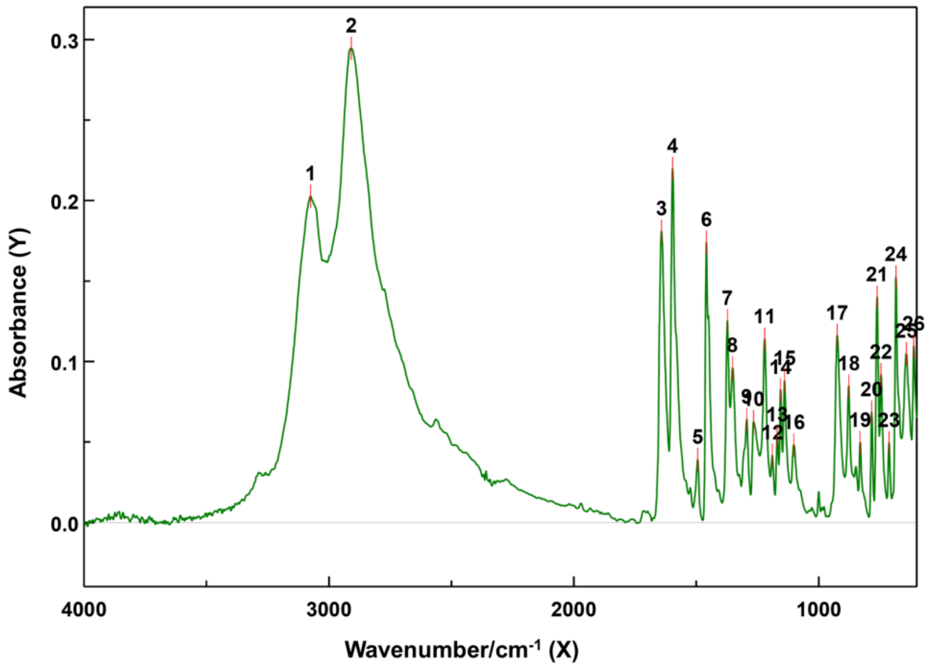

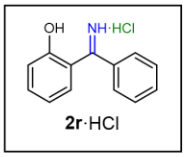

No.	X	Y	No.	X	Y	No.	X	Y
1	3074.9	0.203	10	1266	0.062	19	831.2	0.049
2	2909.1	0.294	11	1220.7	0.114	20	783.9	0.068
3	1642.1	0.181	12	1189.9	0.041	21	761.7	0.14
4	1596.8	0.22	13	1169.6	0.053	22	745.4	0.092
5	1494.6	0.039	14	1156.1	0.082	23	713.5	0.049
6	1458.9	0.174	15	1139.7	0.088	24	684.6	0.152
7	1373.1	0.125	16	1102.1	0.048	25	642.2	0.105
8	1351.9	0.096	17	924.7	0.116	26	612.3	0.109
9	1294	0.064	18	877.5	0.085			

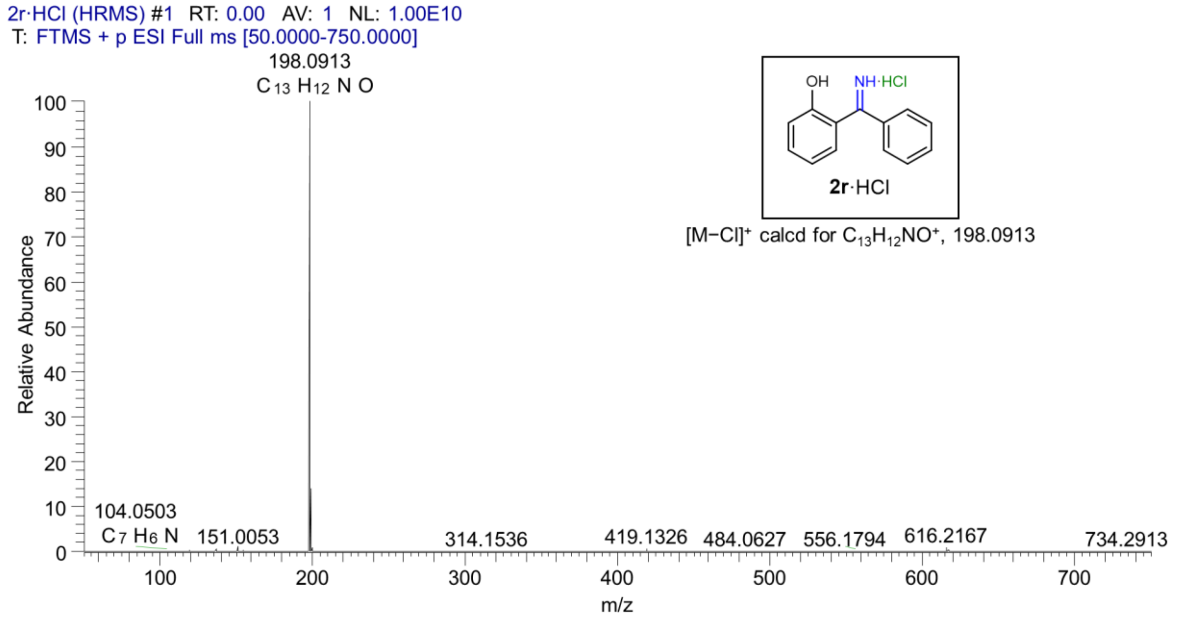

[M−Cl]$^+$ calcd for $C_{13}H_{12}NO^+$, 198.0913

^{1}H NMR (400 MHz, DMSO-d_6) and ^{13}C NMR (100 MHz, DMSO-d_6) spectra for **2s**

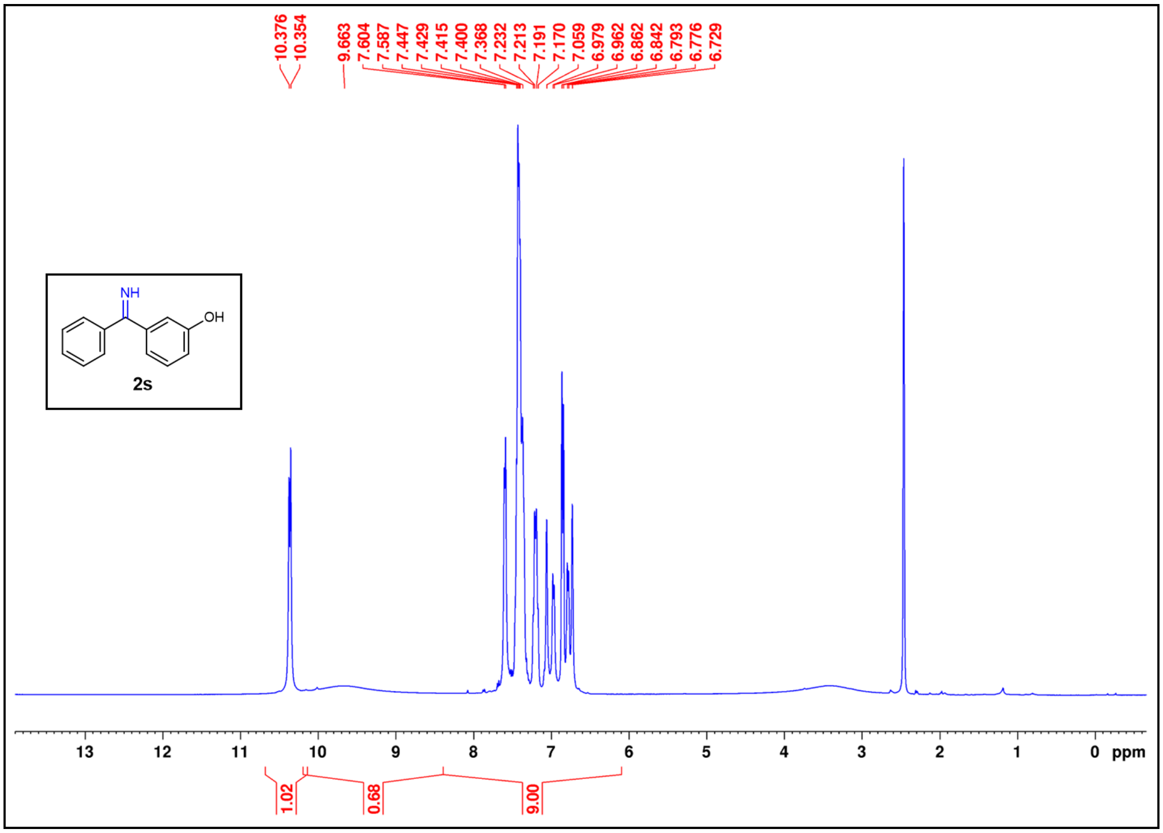

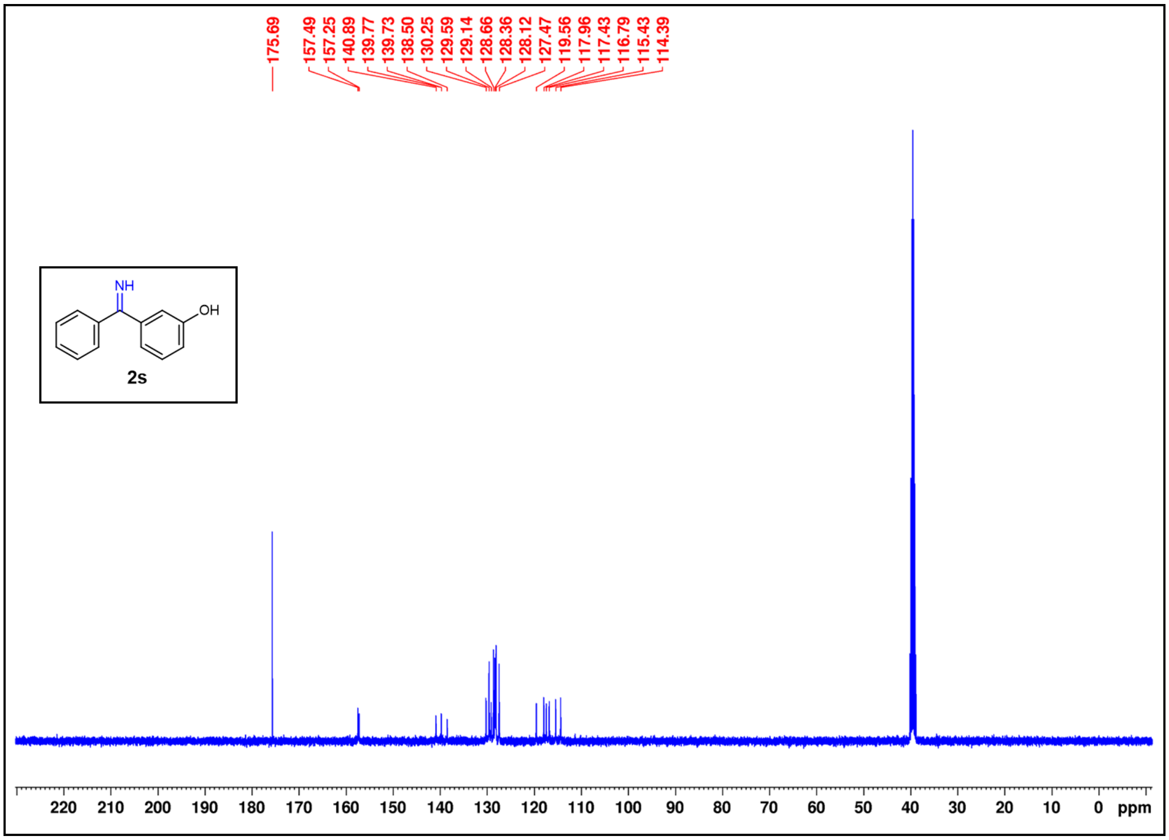

FT-IR (ATR, neat) and HRMS (ESI-positive) spectra for **2s**

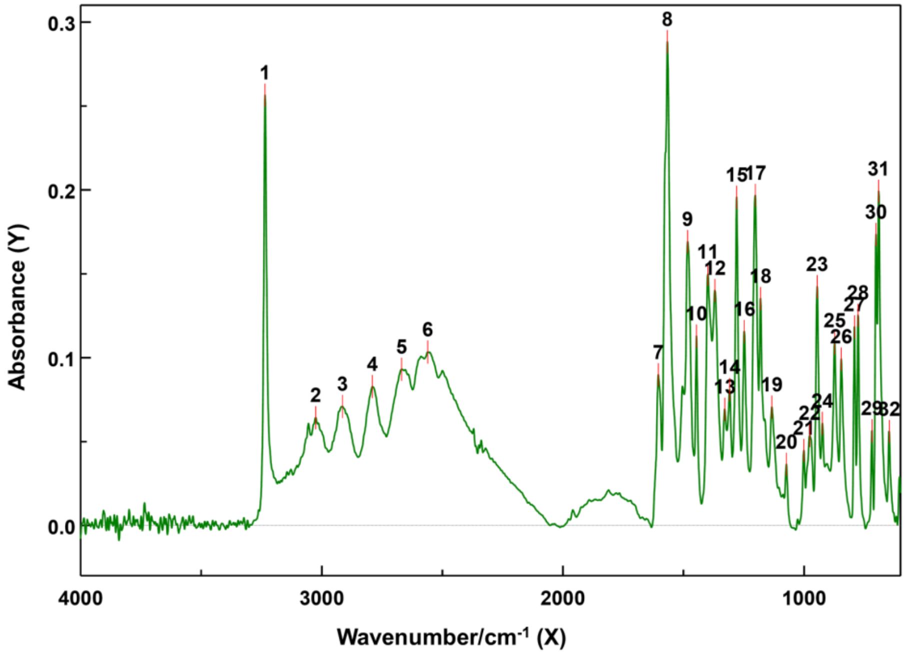

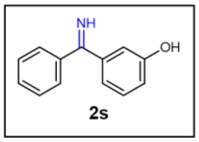

No.	X	Y	No.	X	Y	No.	X	Y
1	3235	0.256	12	1369.2	0.14	23	945.9	0.142
2	3025.8	0.064	13	1328.7	0.069	24	923.7	0.061
3	2913.9	0.071	14	1308.5	0.081	25	873.6	0.109
4	2789.5	0.083	15	1279.5	0.195	26	845.6	0.099
5	2669	0.093	16	1247.7	0.116	27	790.7	0.118
6	2561	0.103	17	1202.4	0.197	28	776.2	0.125
7	1604.5	0.09	18	1180.2	0.135	29	718.4	0.056
8	1566.9	0.288	19	1133.9	0.071	30	702	0.173
9	1483	0.169	20	1073.2	0.036	31	690.4	0.199
10	1446.4	0.113	21	1000.9	0.045	32	647	0.056
11	1399.1	0.15	22	976.8	0.054			

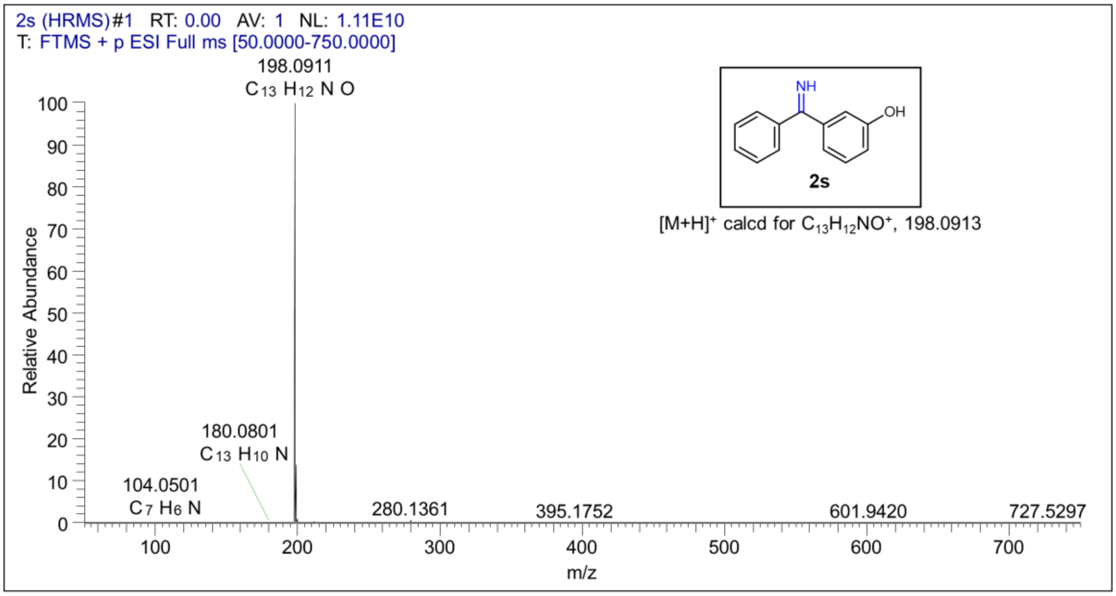

^{1}H NMR (400 MHz, DMSO-d_6) and ^{13}C NMR (100 MHz, DMSO-d_6) spectra for **2t**

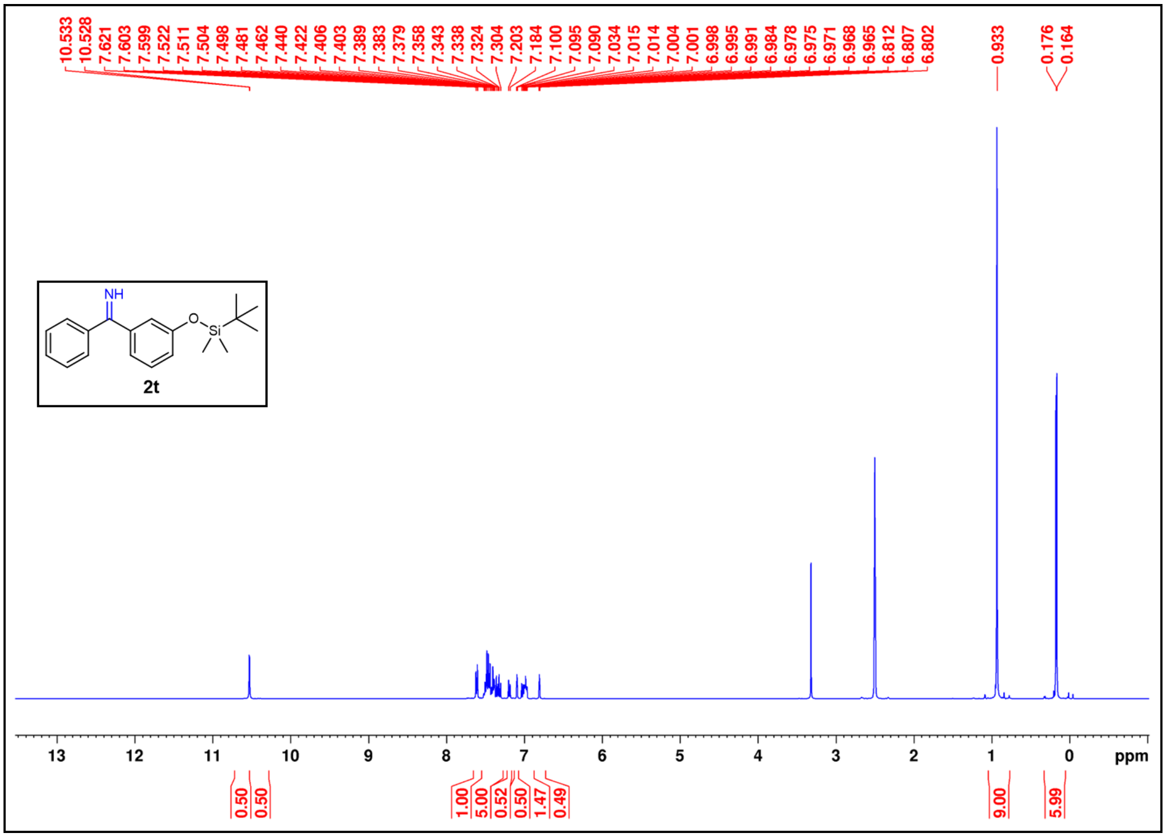

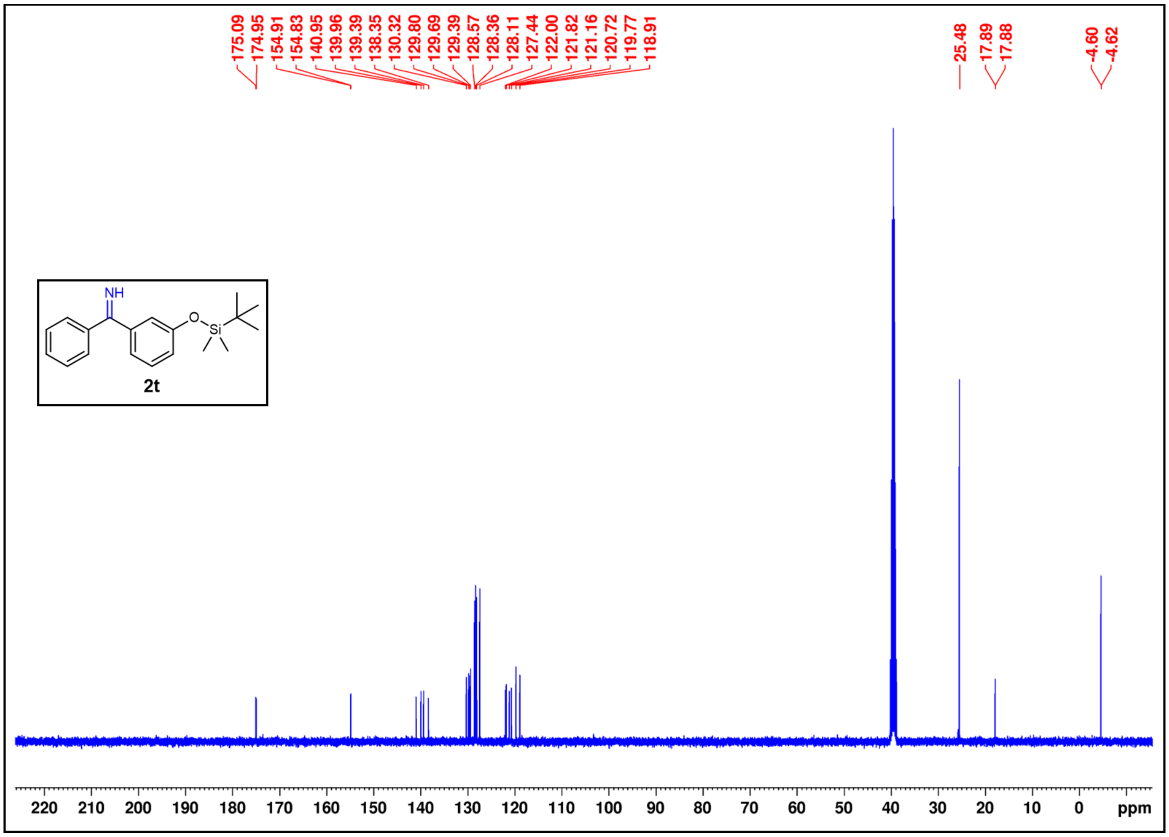

FT-IR (ATR, neat) and HRMS (ESI-positive) spectra for **2t**

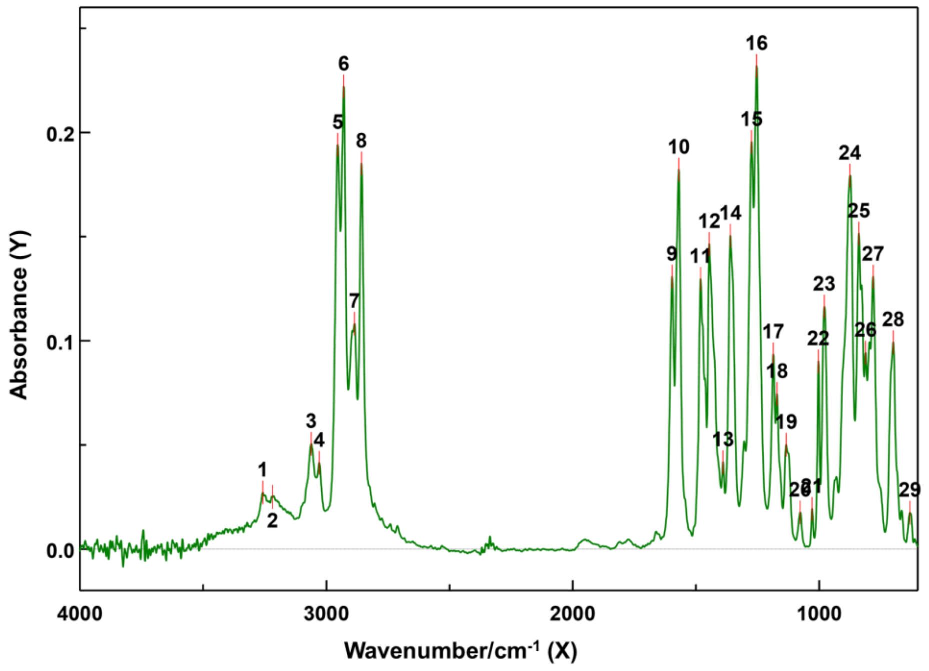

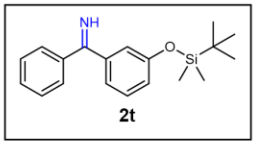

No.	X	Y	No.	X	Y	No.	X	Y
1	3258.1	0.027	11	1481.1	0.13	21	1029	0.019
2	3217.6	0.025	12	1446.4	0.146	22	1003	0.09
3	3061.4	0.05	13	1390.4	0.042	23	978.7	0.116
4	3028.7	0.041	14	1360.5	0.15	24	874.6	0.179
5	2953.4	0.194	15	1274.7	0.195	25	838.9	0.151
6	2929.3	0.222	16	1253.5	0.232	26	811.9	0.094
7	2886	0.108	17	1186	0.093	27	781	0.131
8	2857	0.185	18	1171.5	0.074	28	699.1	0.099
9	1596.8	0.131	19	1133.9	0.05	29	631.6	0.017
10	1569.8	0.182	20	1077	0.018			

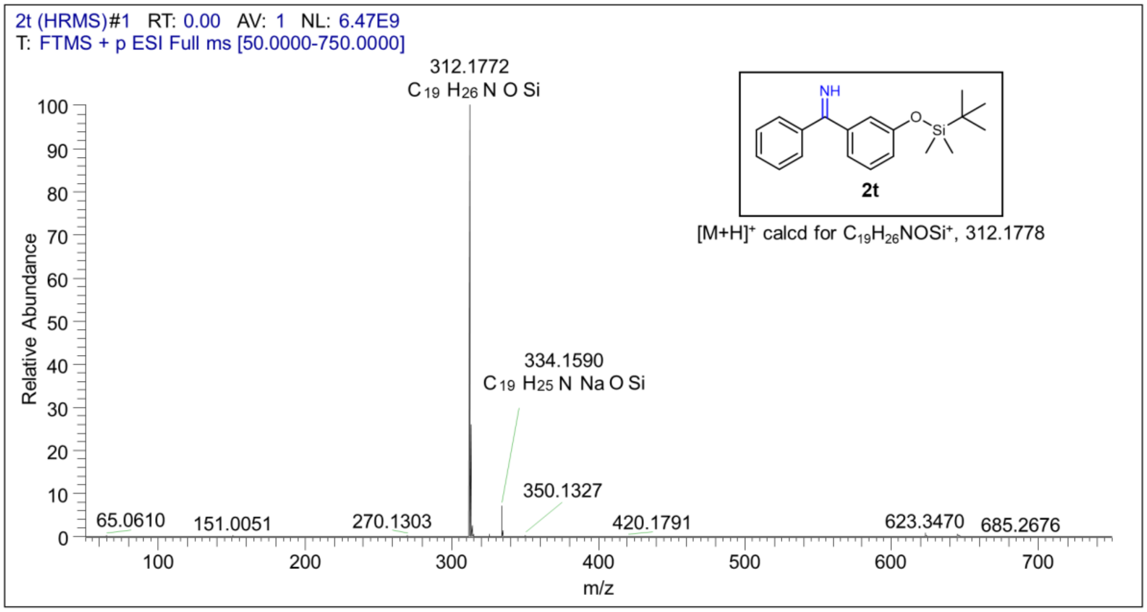

^{1}H NMR (400 MHz, DMSO-d_6) and ^{13}C NMR (100 MHz, DMSO-d_6) spectra for **2u**

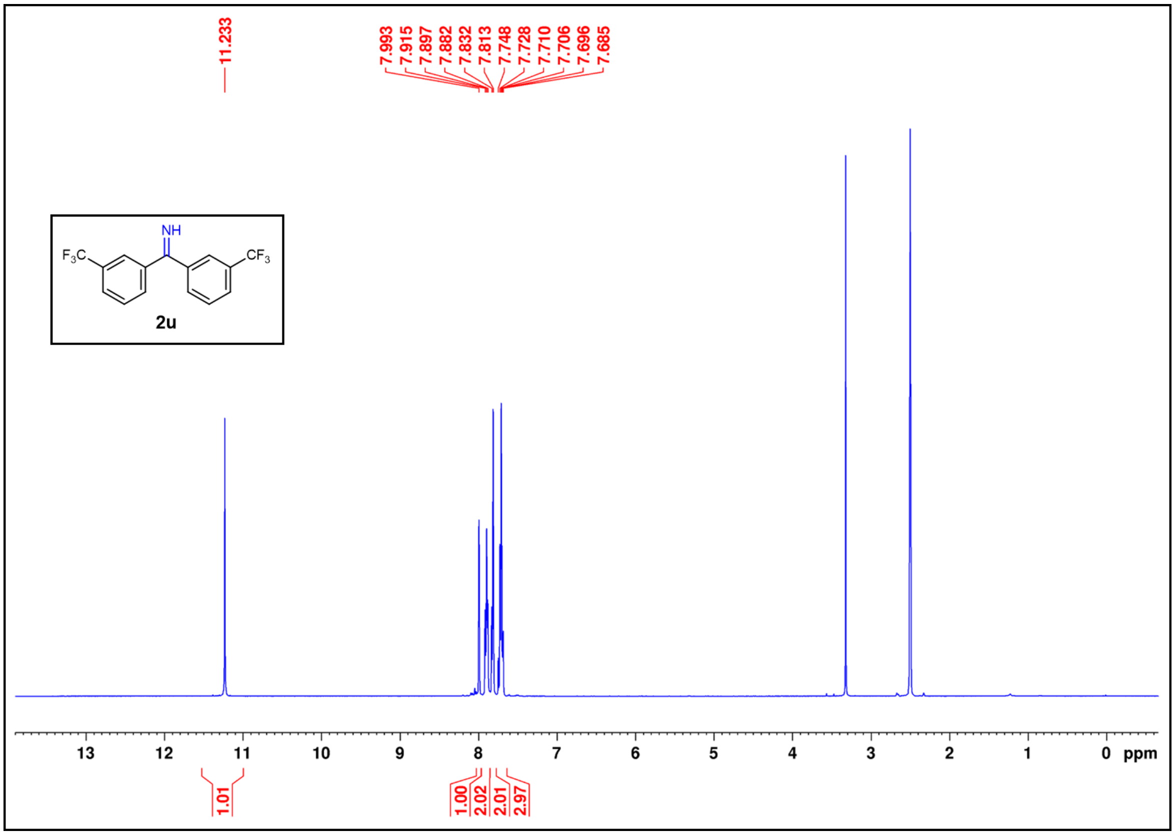

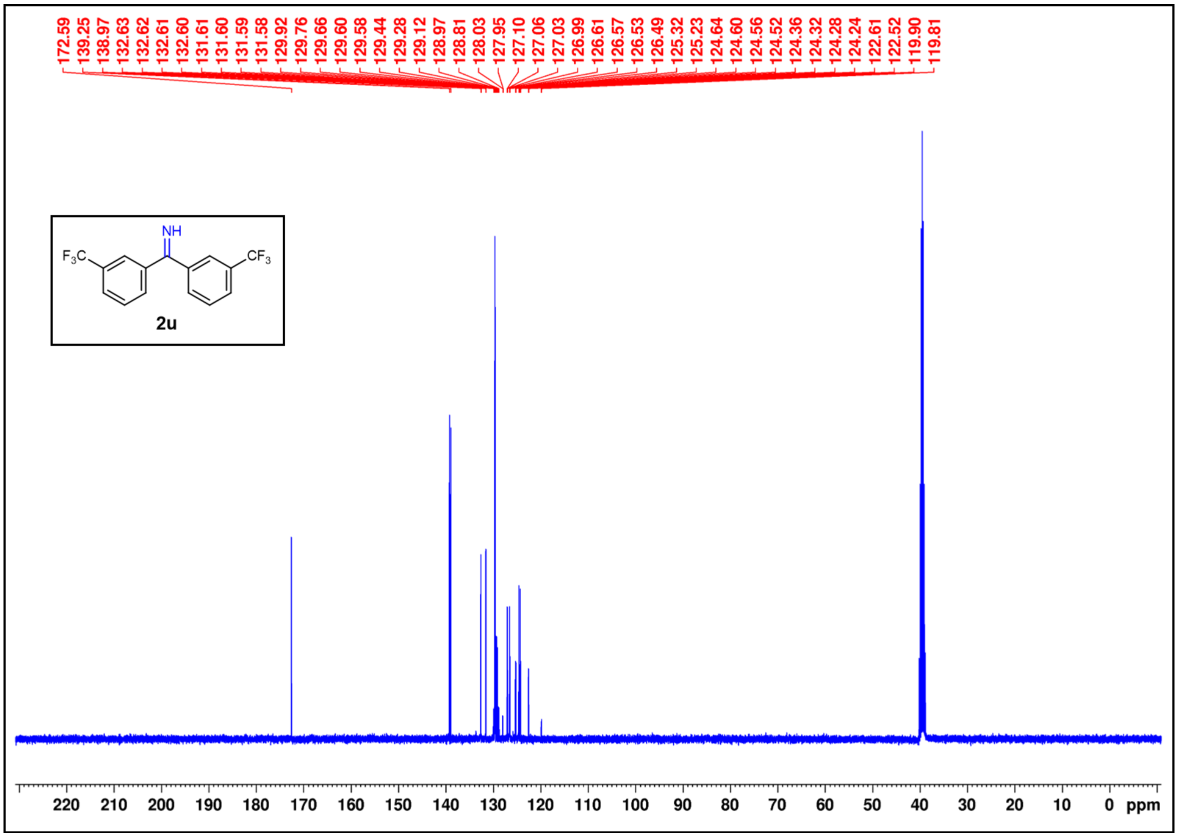

^{19}F NMR (376 MHz, DMSO-d_6) spectrum for **2u**

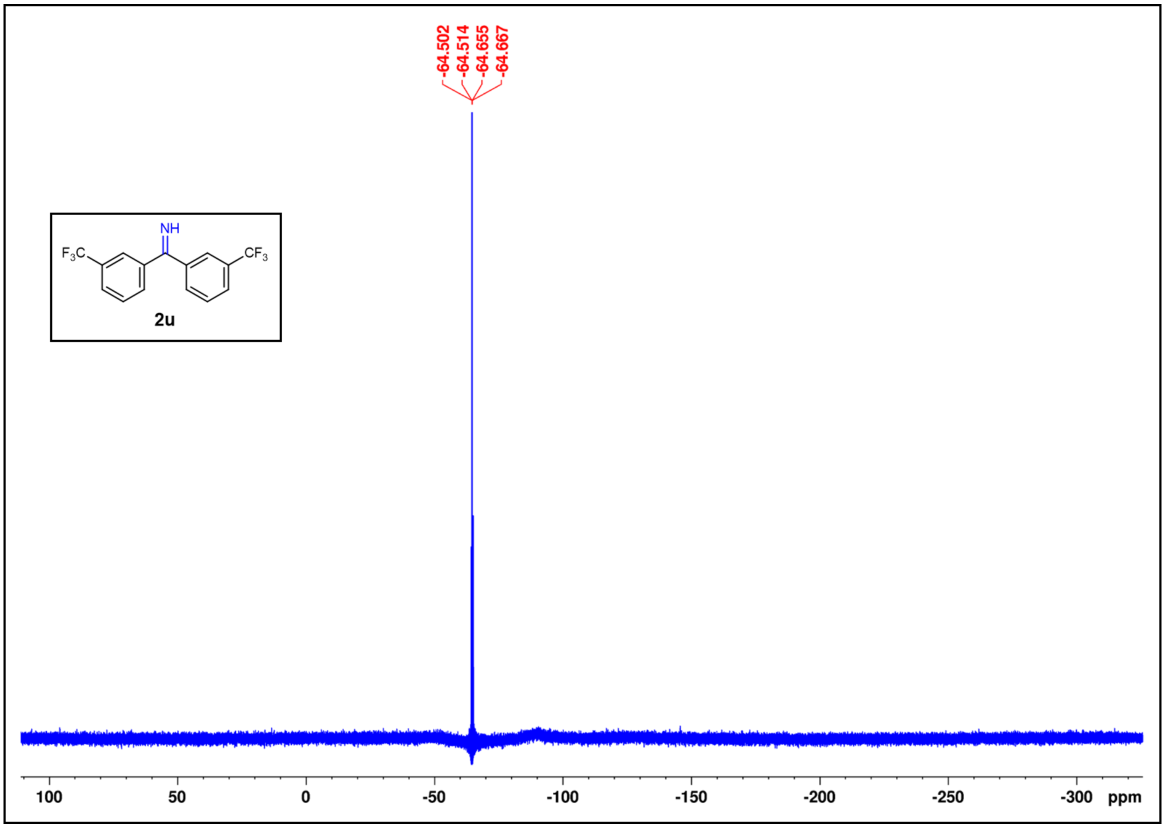

FT-IR (ATR, neat) and HRMS (ESI-positive) spectra for **2u**

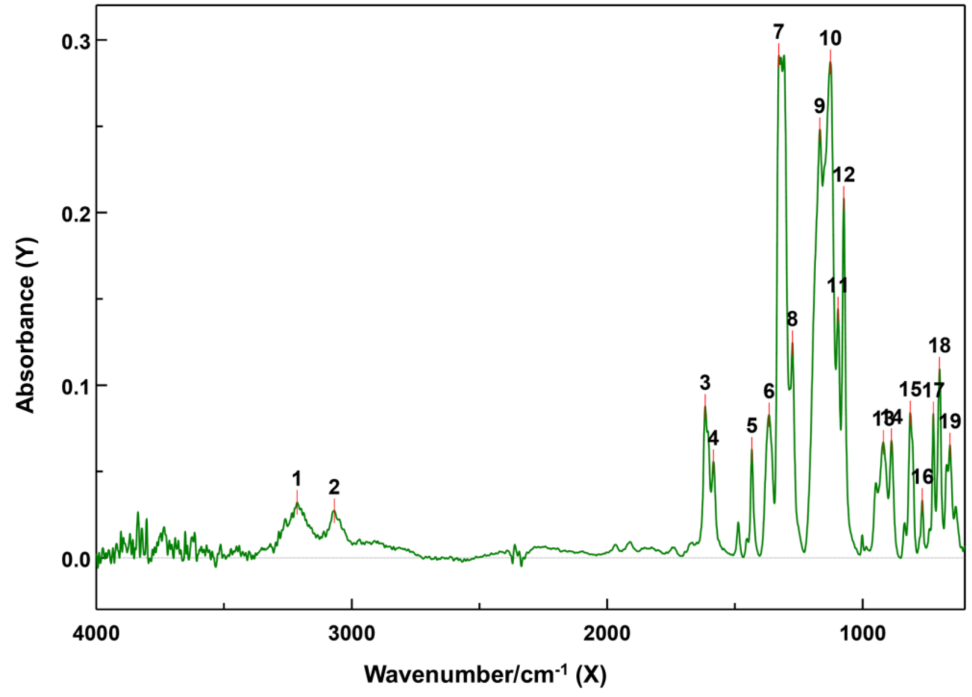

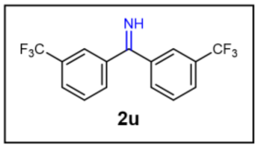

No.	X	Y	No.	X	Y	No.	X	Y
1	3213.8	0.032	8	1274.7	0.124	15	813.8	0.084
2	3068.2	0.027	9	1167.7	0.248	16	766.6	0.033
3	1616.1	0.088	10	1126.2	0.287	17	723.2	0.083
4	1584.2	0.056	11	1096.3	0.144	18	699.1	0.109
5	1433.8	0.063	12	1074.2	0.208	19	658.6	0.065
6	1366.3	0.083	13	918.9	0.067			
7	1327.7	0.291	14	887.1	0.068			

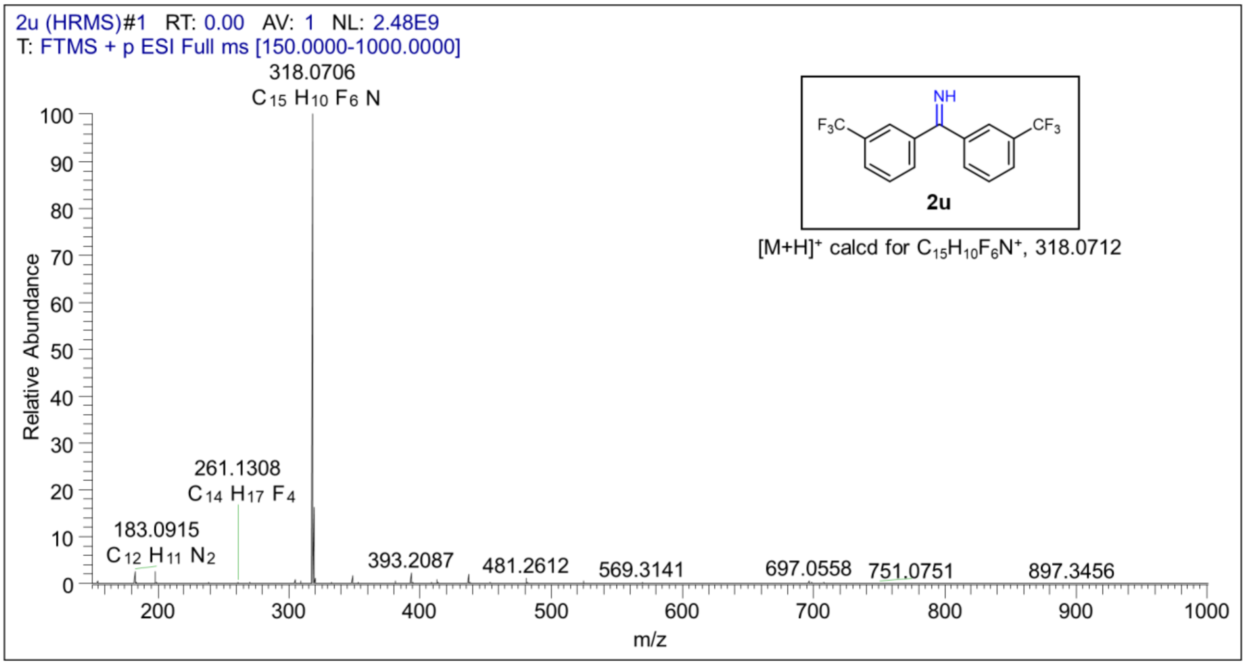

^{1}H NMR (400 MHz, $CDCl_3$) and ^{13}C NMR (100 MHz, $CDCl_3$) spectra for **2v**

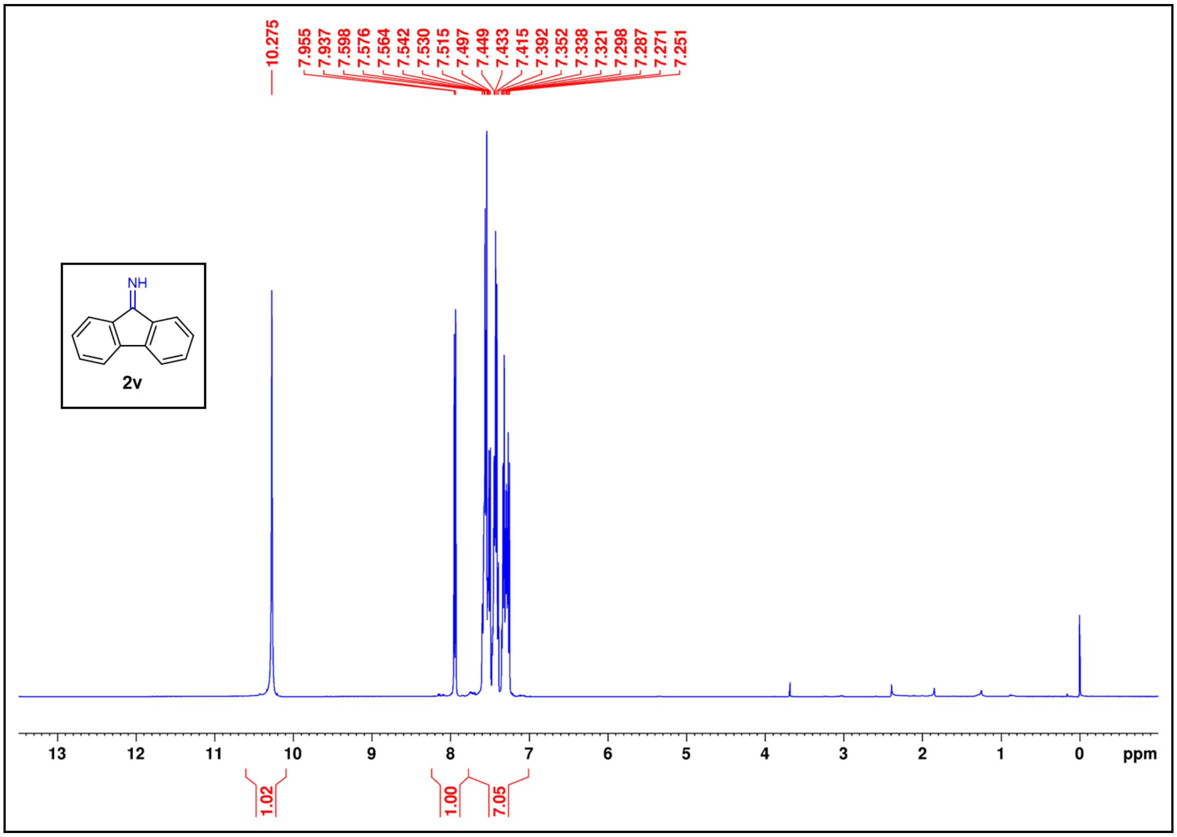

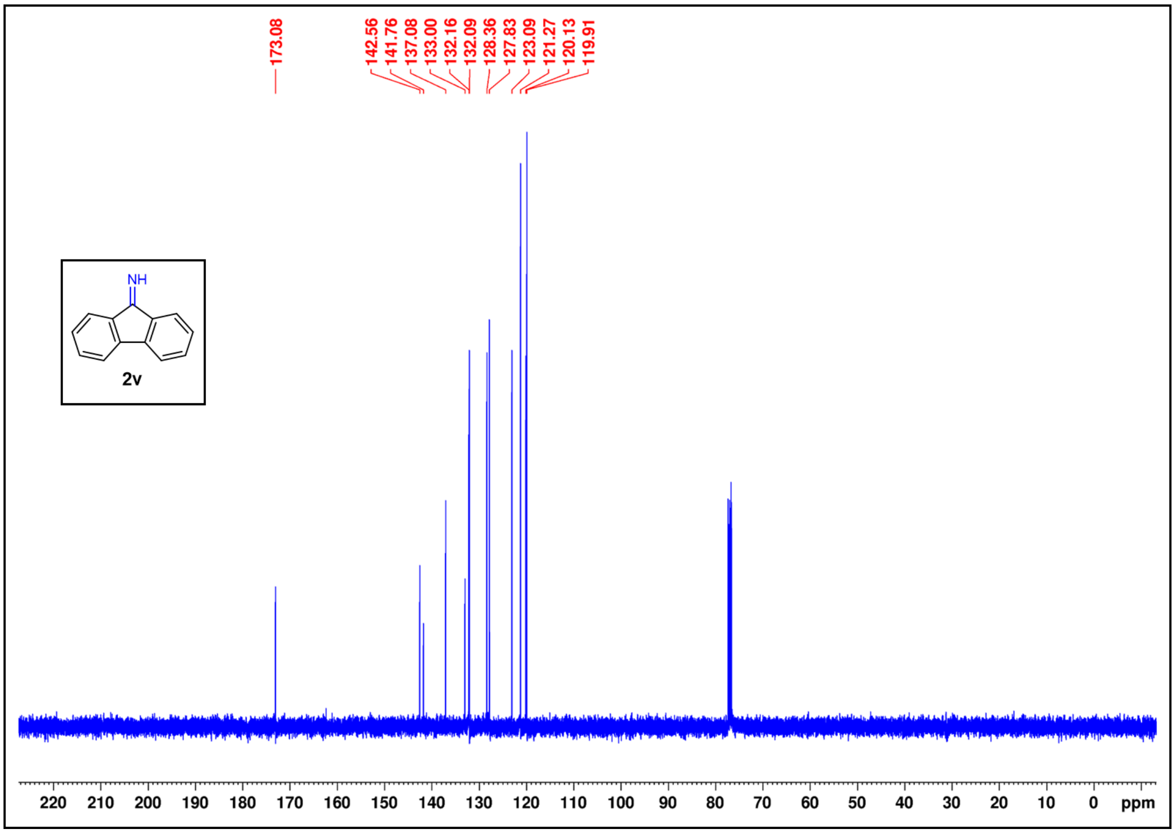

FT-IR (ATR, neat) and HRMS (ESI-positive) spectra for **2v**

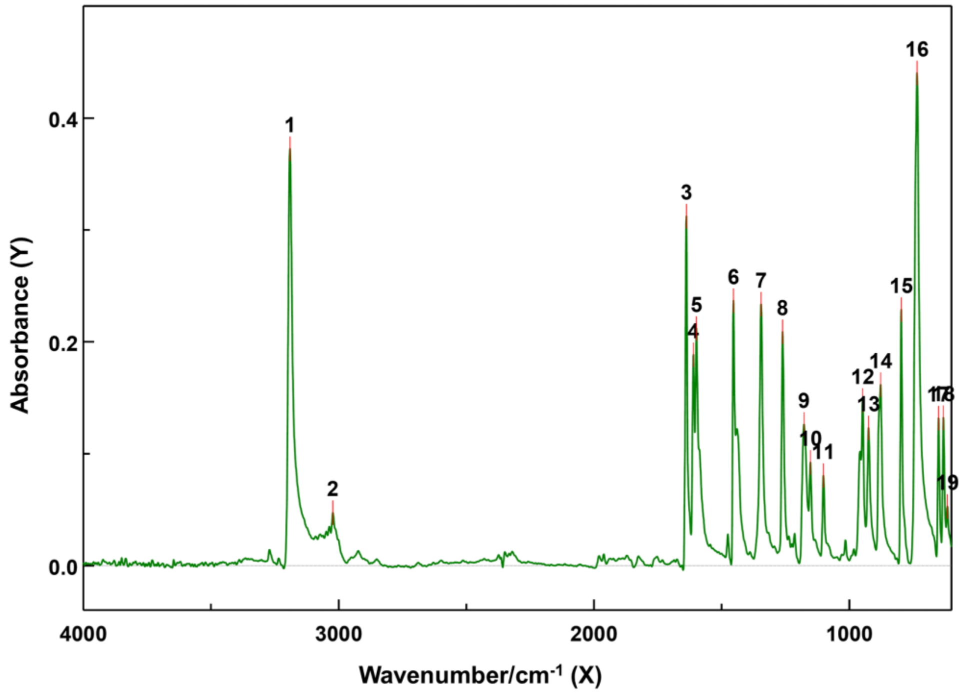

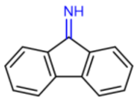

2v

No.	X	Y	No.	X	Y	No.	X	Y
1	3190.6	0.372	8	1261.2	0.209	15	796.5	0.229
2	3022.9	0.047	9	1177.3	0.126	16	734.7	0.44
3	1638.2	0.312	10	1152.3	0.092	17	650.9	0.132
4	1610.3	0.188	11	1101.2	0.08	18	631.6	0.132
5	1598.7	0.212	12	947.8	0.147	19	616.1	0.053
6	1454.1	0.237	13	924.7	0.123			
7	1345.1	0.233	14	877.5	0.162			

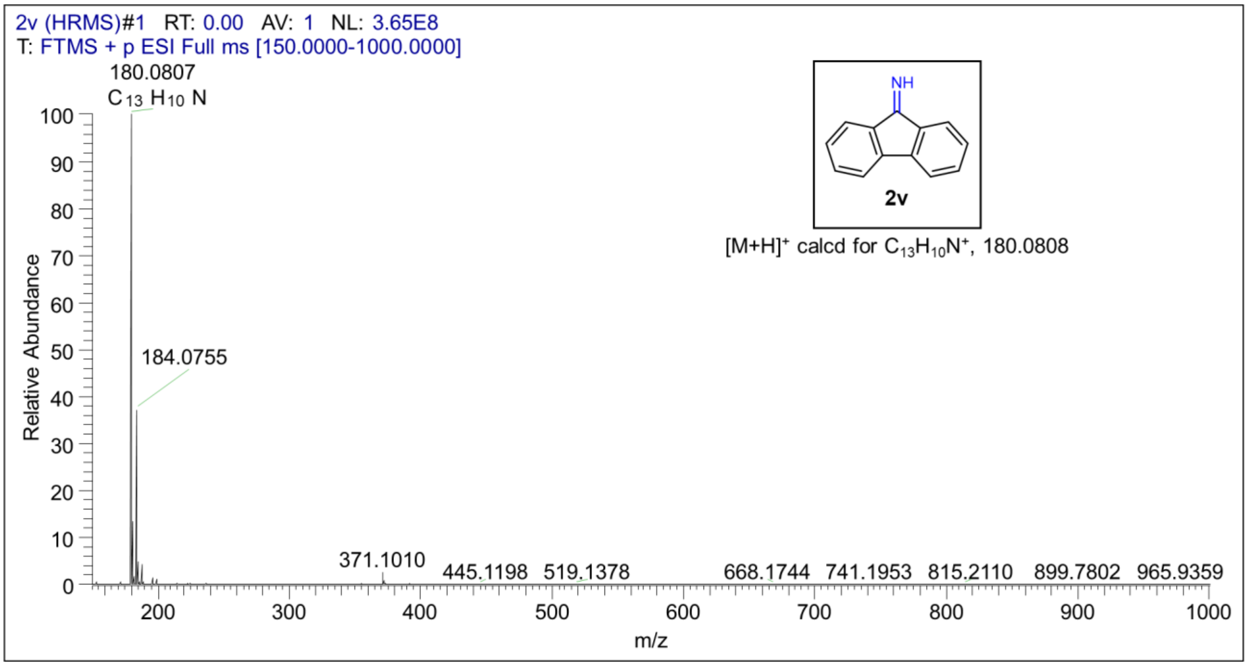

^{1}H NMR (400 MHz, DMSO-d_6) and ^{13}C NMR (100 MHz, DMSO-d_6) spectra for **2w**

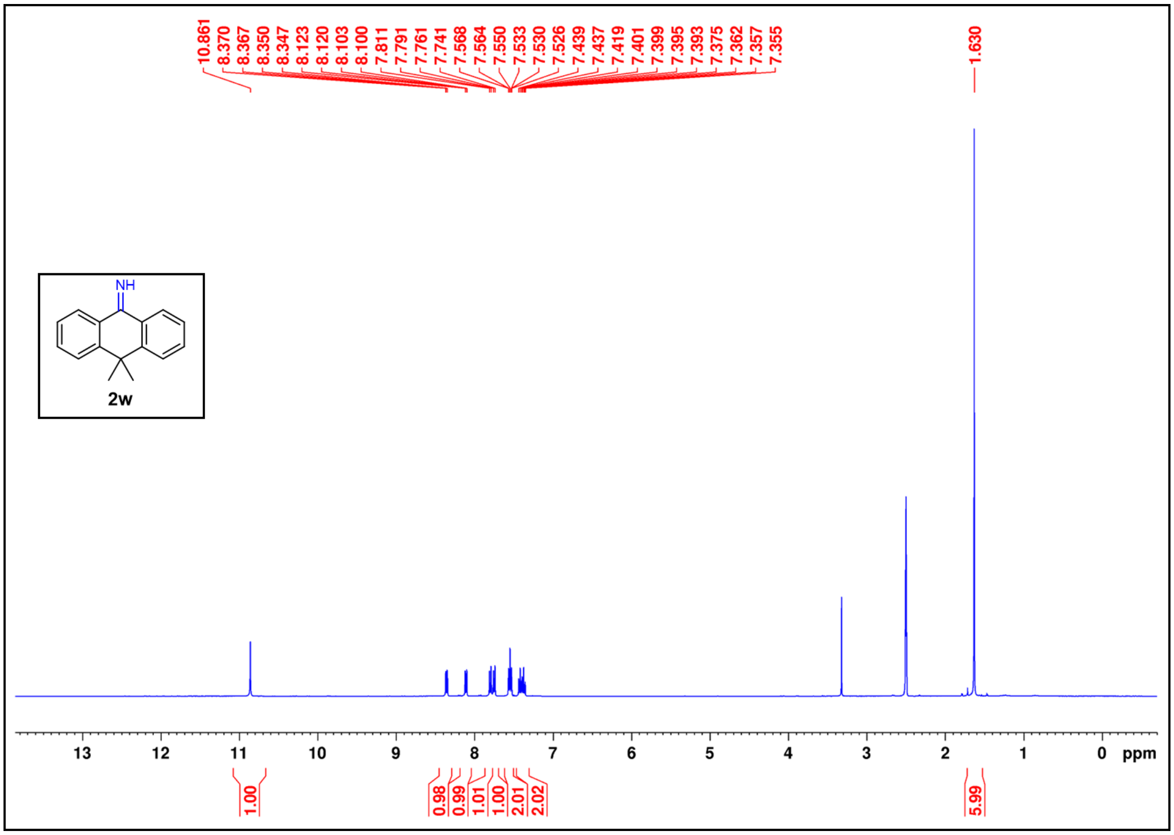

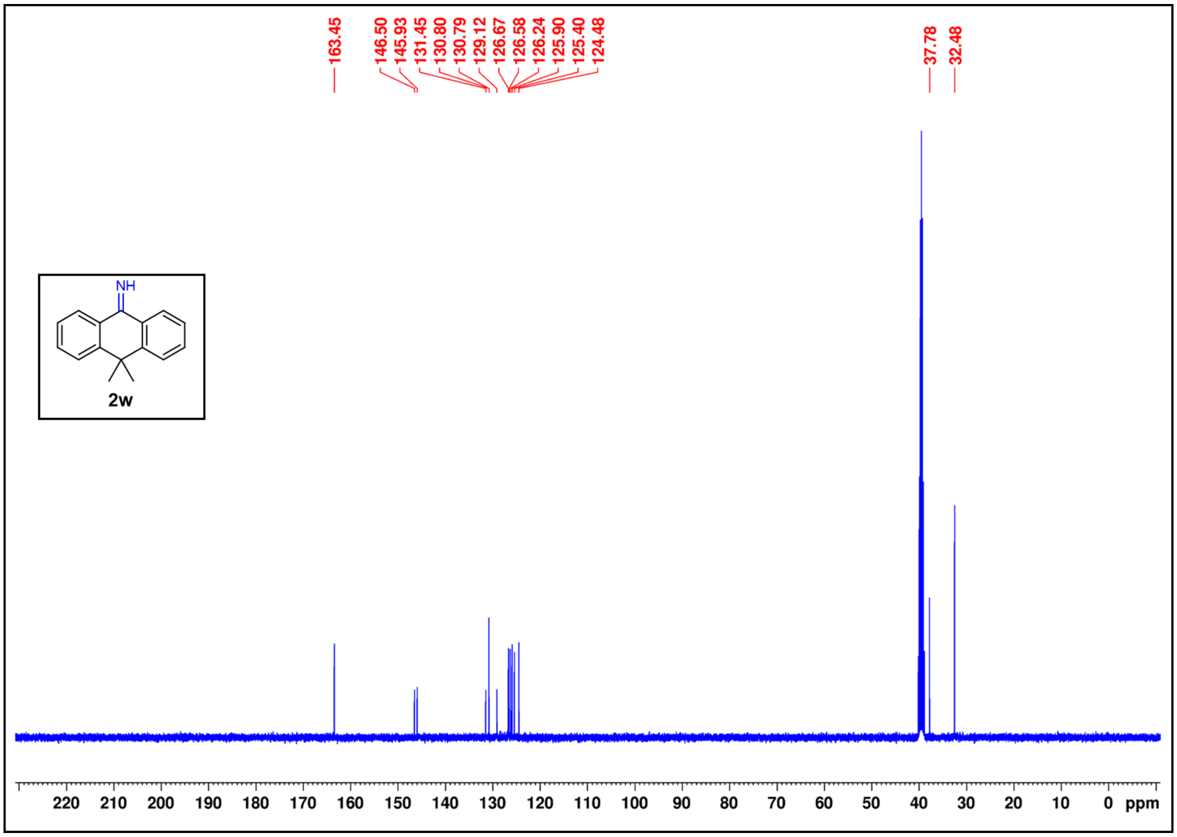

FT-IR (ATR, neat) and HRMS (ESI-positive) spectra for **2w**

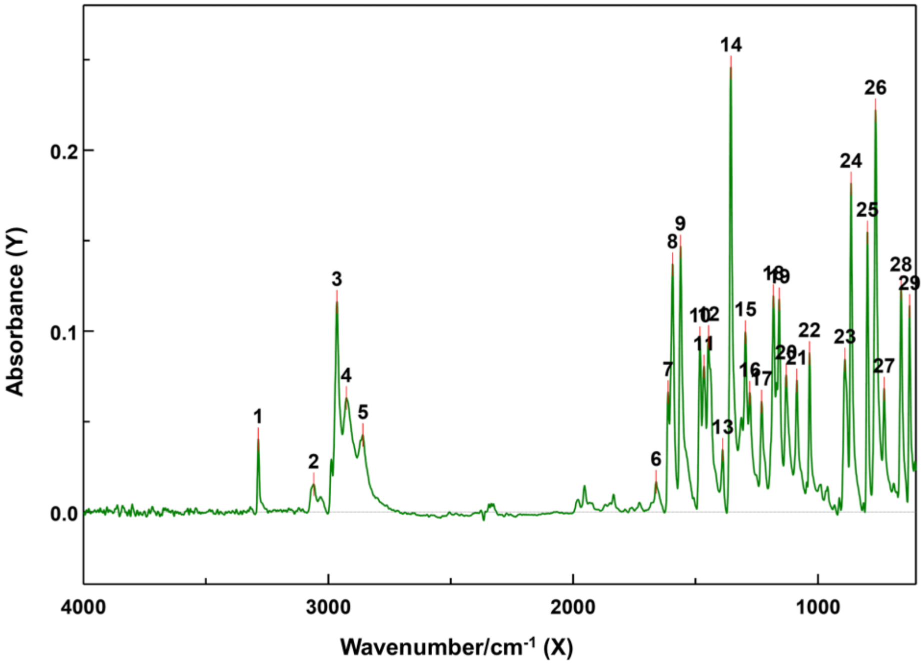

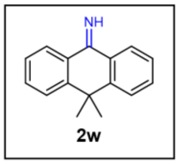

No.	X	Y	No.	X	Y	No.	X	Y
1	3286.1	0.04	11	1465.6	0.08	21	1087	0.073
2	3059.5	0.015	12	1447.3	0.097	22	1035	0.088
3	2965	0.116	13	1389.5	0.034	23	890	0.084
4	2926.4	0.063	14	1355.7	0.246	24	864.9	0.182
5	2859	0.043	15	1295.9	0.099	25	798.4	0.154
6	1661.4	0.017	16	1278.6	0.066	26	764.6	0.222
7	1612.2	0.066	17	1230.4	0.061	27	729.9	0.068
8	1593.9	0.137	18	1182.2	0.119	28	660.5	0.124
9	1561.1	0.147	19	1158	0.117	29	625.8	0.114
10	1482	0.096	20	1130.1	0.076			

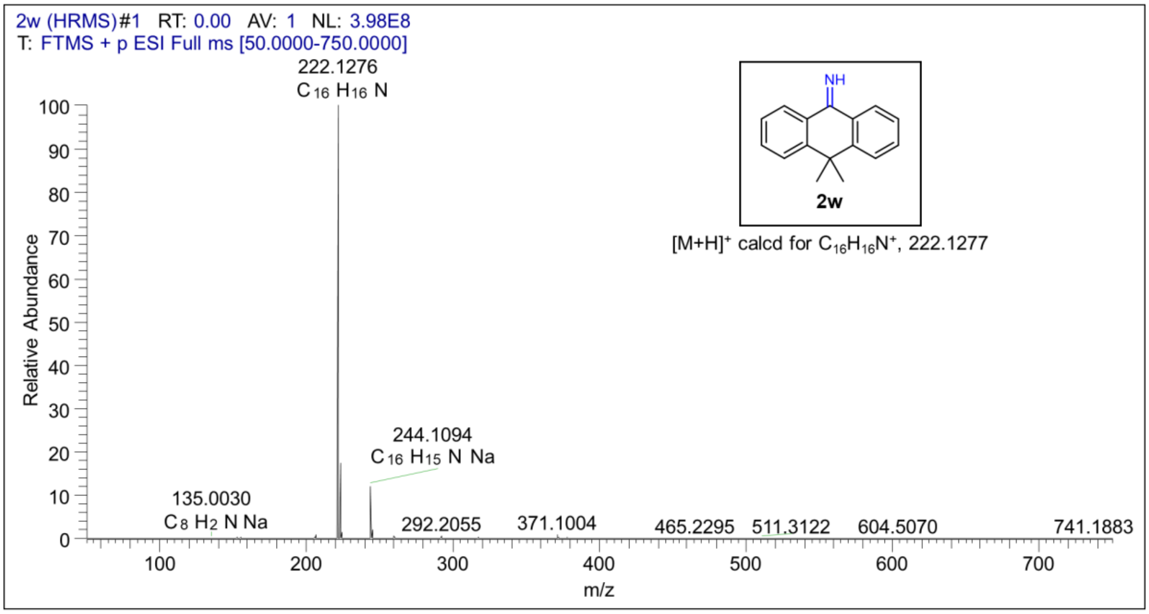

<u>^{1}H NMR (400 MHz, $CDCl_3$) and ^{13}C NMR (100 MHz, $CDCl_3$) spectra for **2x**</u>

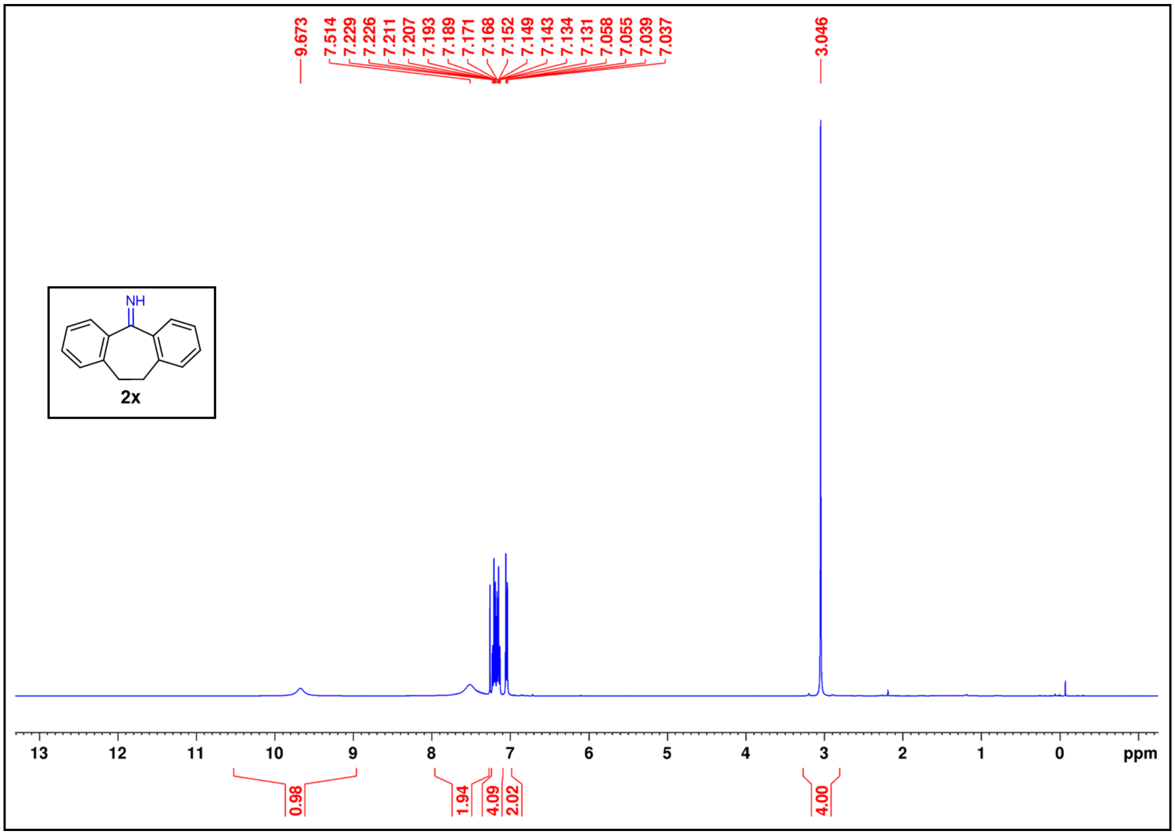

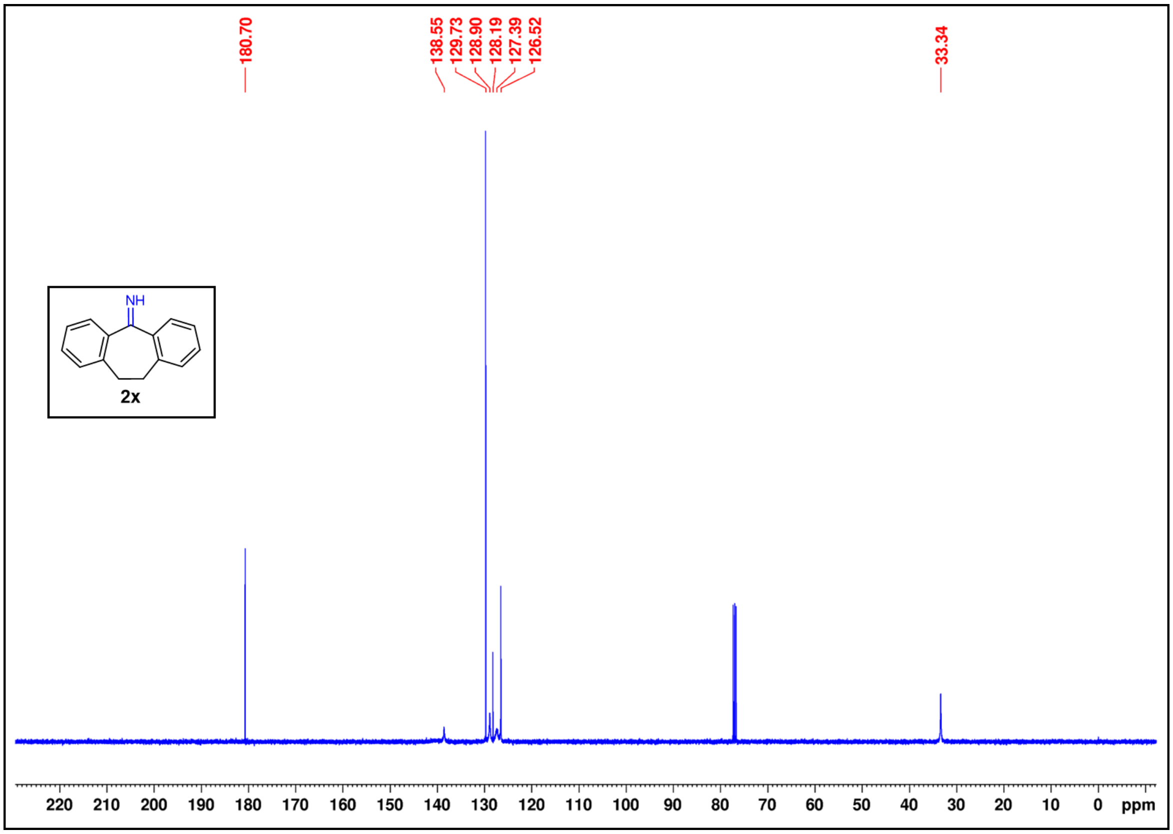

FT-IR (ATR, neat) and HRMS (ESI-positive) spectra for **2x**

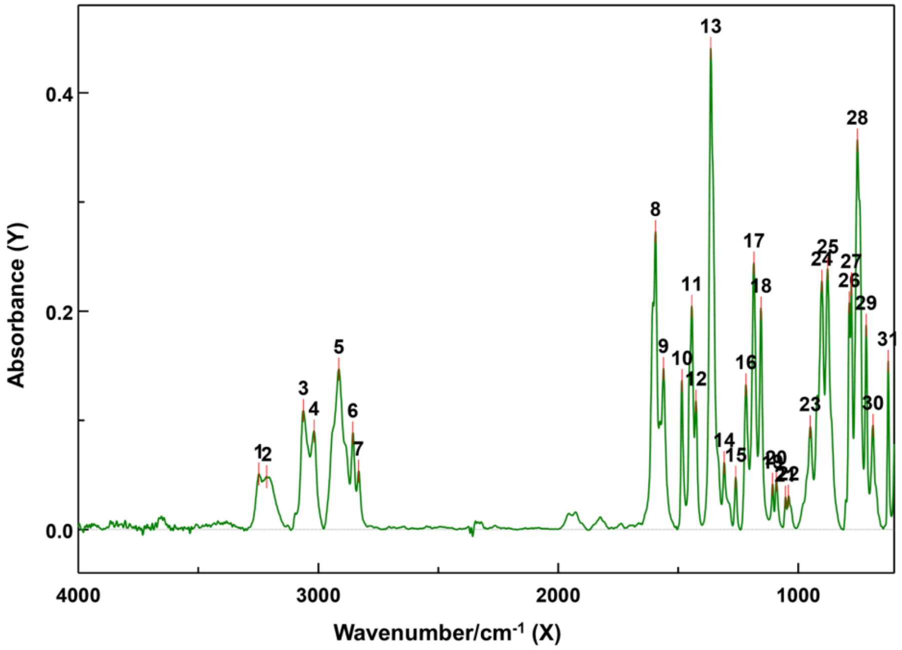

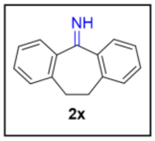

No.	X	Y	No.	X	Y	No.	X	Y
1	3247.5	0.051	12	1426.1	0.117	23	949.8	0.094
2	3215.7	0.048	13	1364.4	0.44	24	901.6	0.227
3	3062.4	0.109	14	1308.5	0.061	25	877.5	0.238
4	3018.1	0.09	15	1260.3	0.048	26	787.8	0.207
5	2914.9	0.147	16	1217.8	0.132	27	778.1	0.225
6	2856.1	0.088	17	1185	0.244	28	753.1	0.356
7	2832	0.053	18	1155.2	0.203	29	716.4	0.187
8	1594.8	0.273	19	1106.9	0.041	30	688.5	0.095
9	1561.1	0.147	20	1091.5	0.045	31	624.8	0.154
10	1484.9	0.136	21	1052.9	0.029			
11	1443.5	0.204	22	1040.4	0.031			

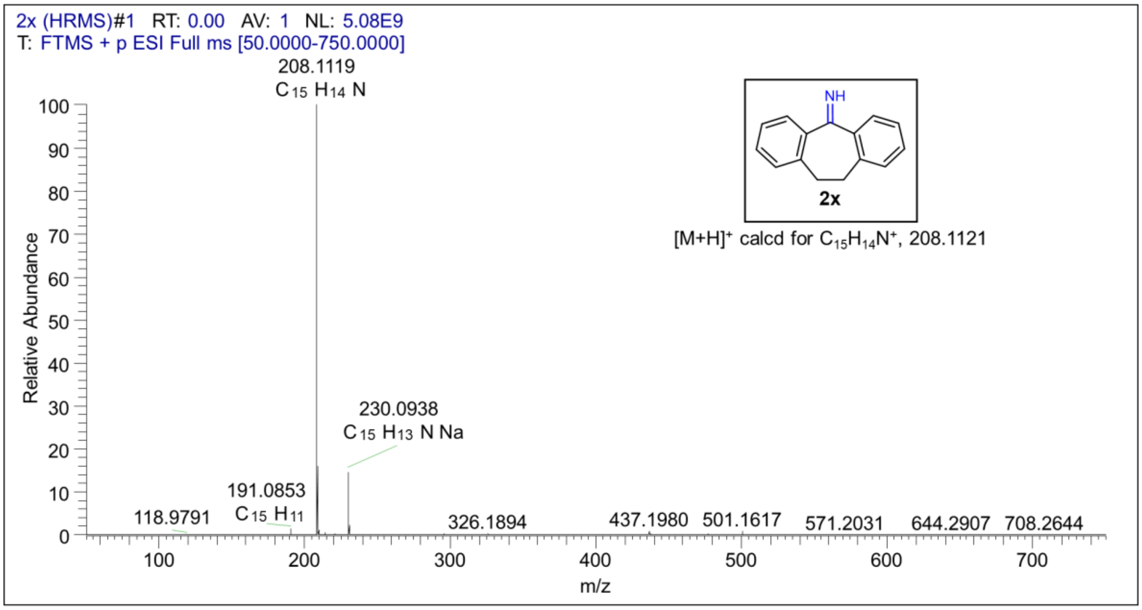

^{1}H NMR (400 MHz, $CDCl_3$) and ^{13}C NMR (100 MHz, $CDCl_3$) spectra for **2y**

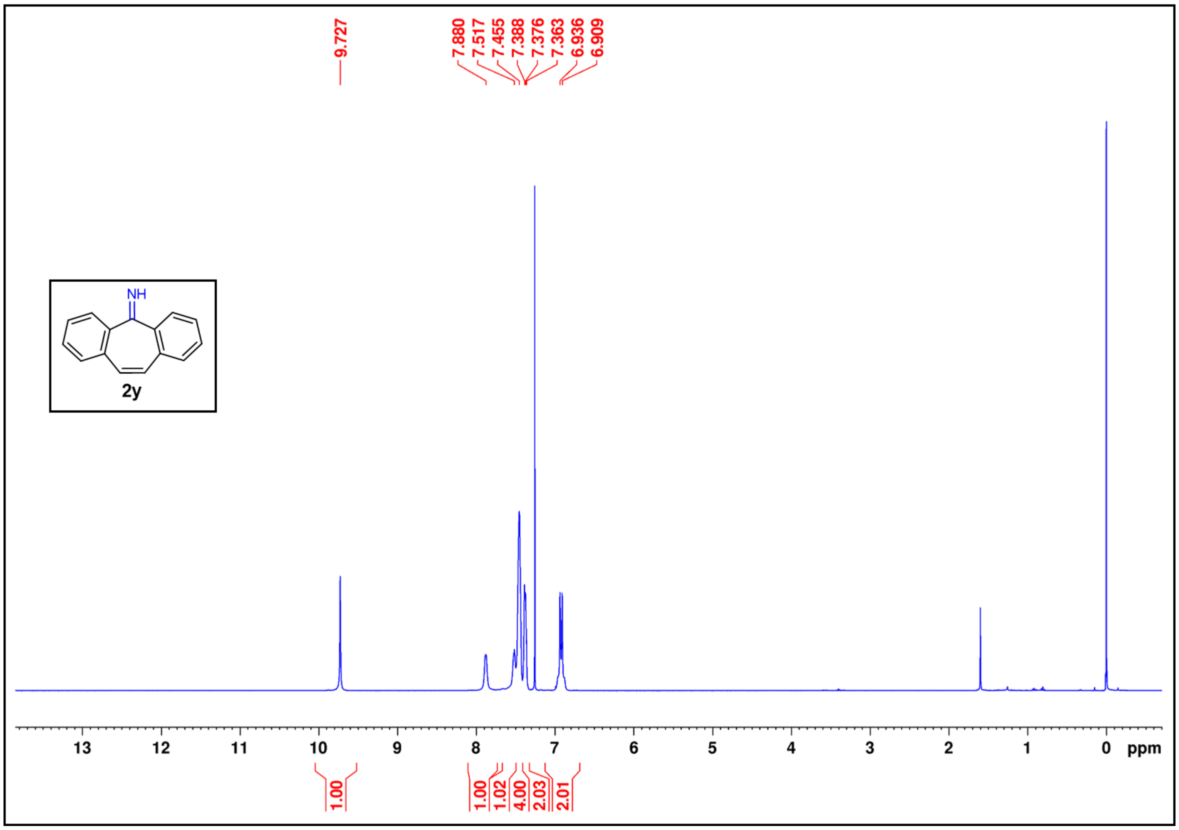

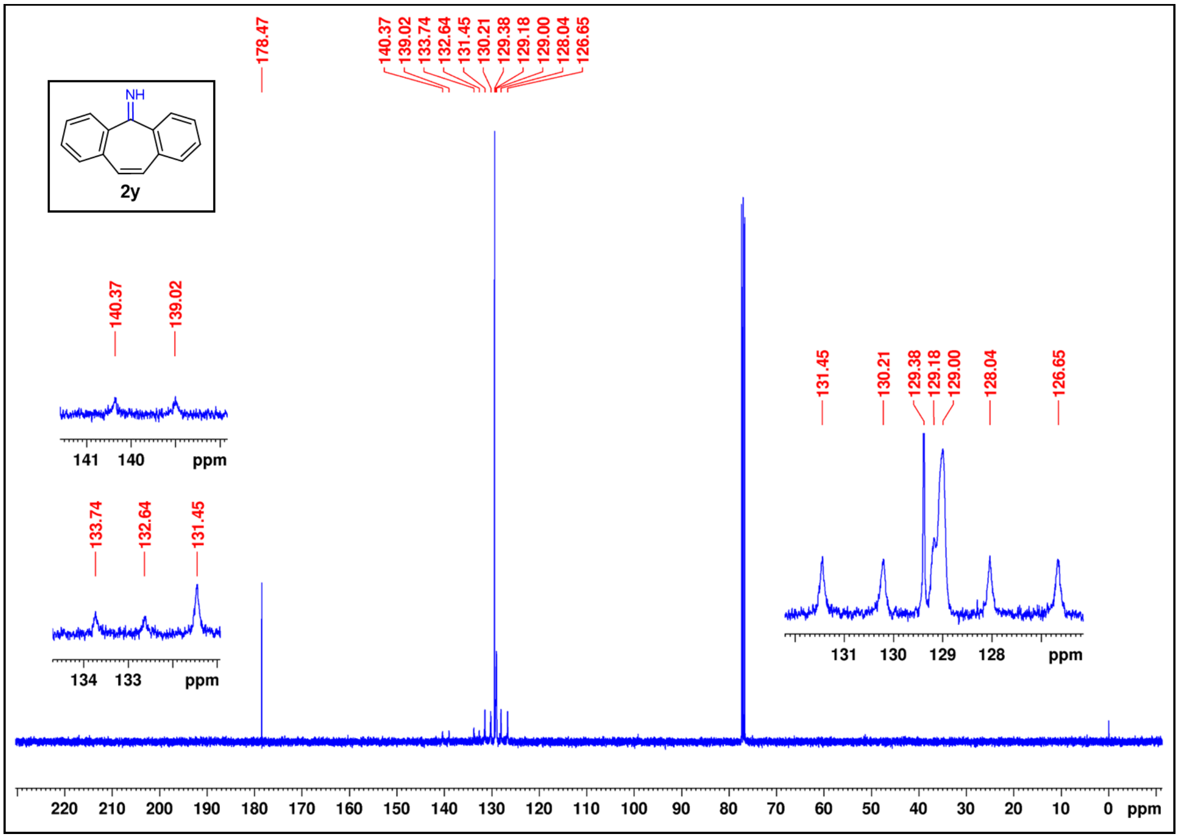

FT-IR (ATR, neat) and HRMS (ESI-positive) spectra for **2y**

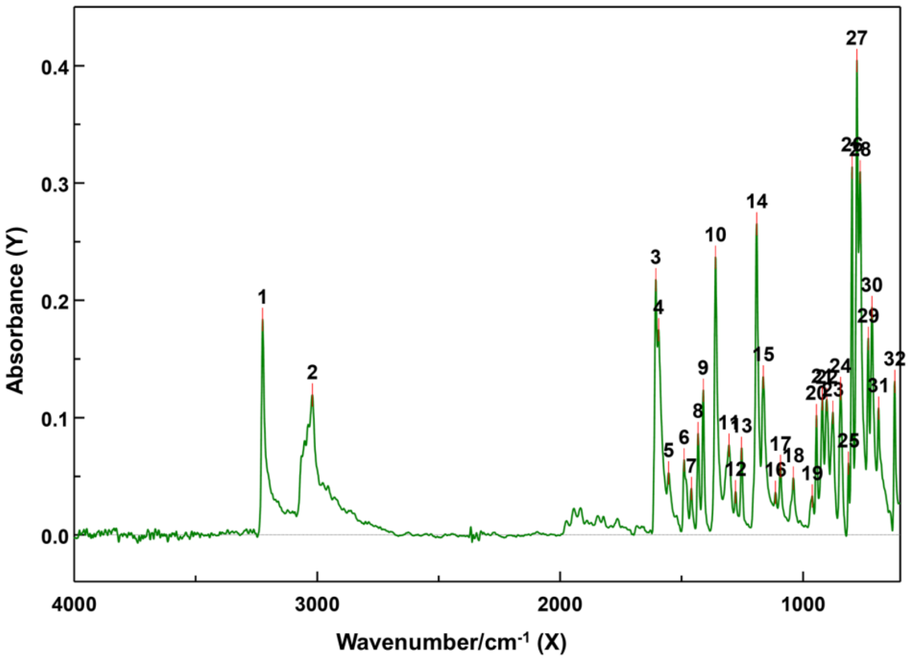

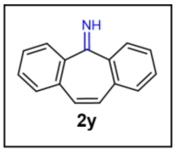

No.	X	Y	No.	X	Y	No.	X	Y
1	3224.4	0.183	12	1277.6	0.037	23	877.5	0.104
2	3020	0.119	13	1253.5	0.074	24	845.6	0.125
3	1606.4	0.217	14	1190.8	0.265	25	812.8	0.061
4	1594.8	0.175	15	1163.8	0.135	26	798.4	0.313
5	1553.4	0.053	16	1113.7	0.036	27	778.1	0.404
6	1489.7	0.064	17	1093.4	0.058	28	764.6	0.309
7	1459.8	0.039	18	1039.4	0.048	29	731.9	0.167
8	1431.9	0.086	19	962.3	0.033	30	716.4	0.194
9	1410.7	0.123	20	944.9	0.102	31	689.4	0.108
10	1360.5	0.236	21	920.8	0.116	32	622.9	0.131
11	1304.6	0.076	22	902.5	0.115			

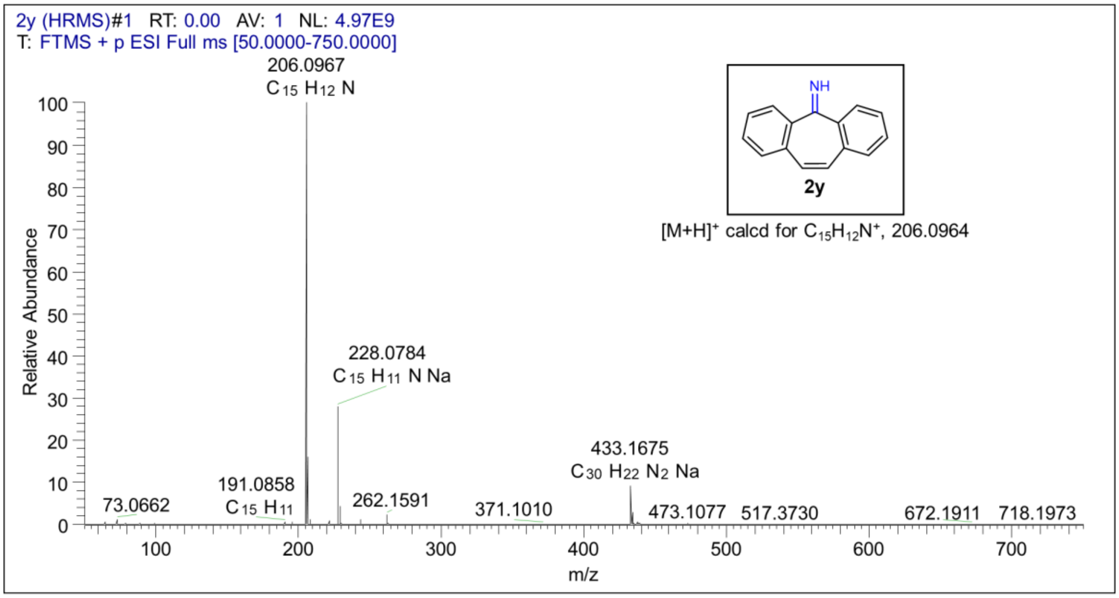

^{1}H NMR (400 MHz, $CDCl_3$) and ^{13}C NMR (100 MHz, $CDCl_3$) spectra for **2z**

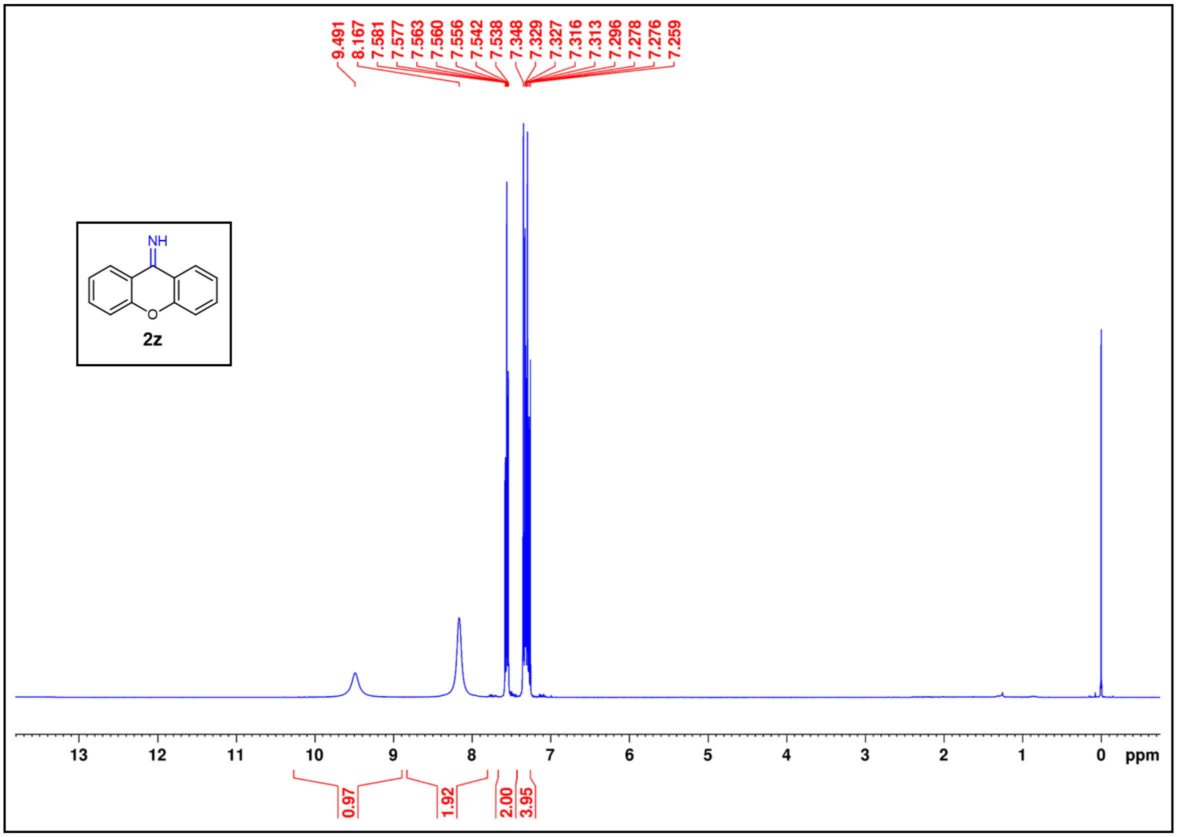

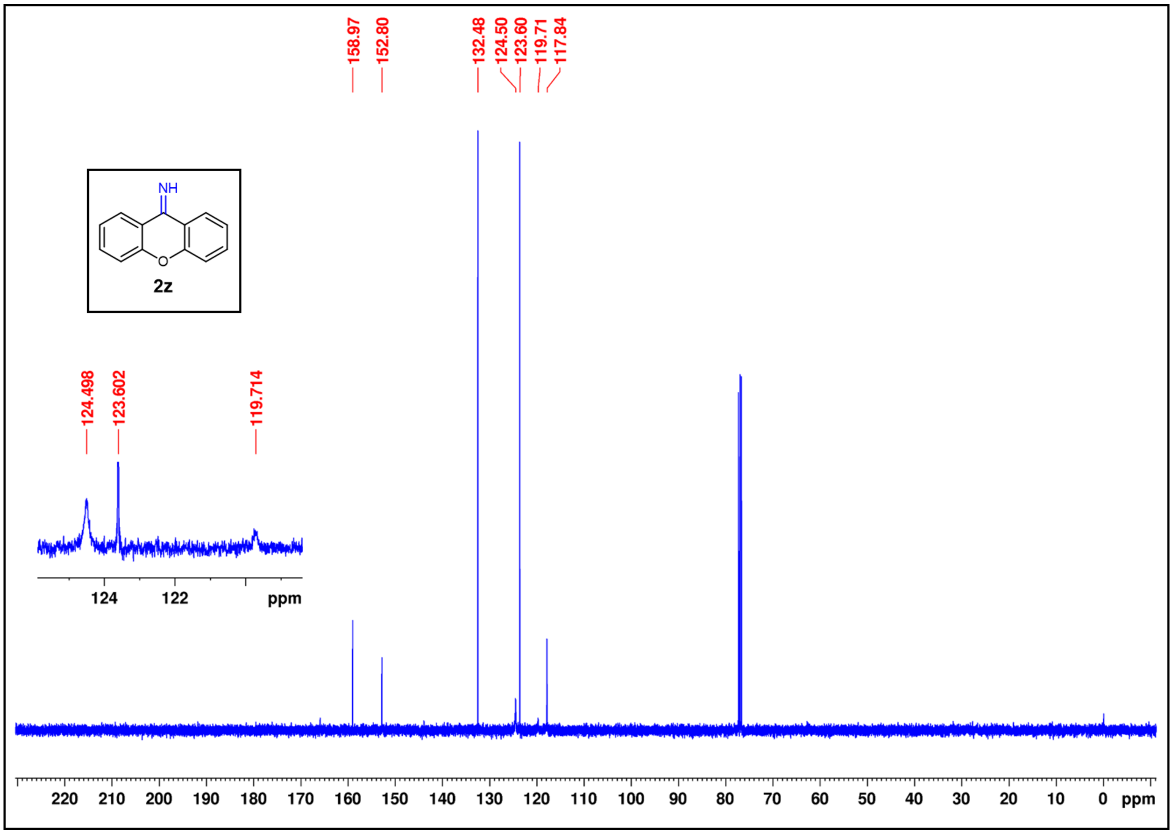

FT-IR (ATR, neat) and HRMS (ESI-positive) spectra for **2z**

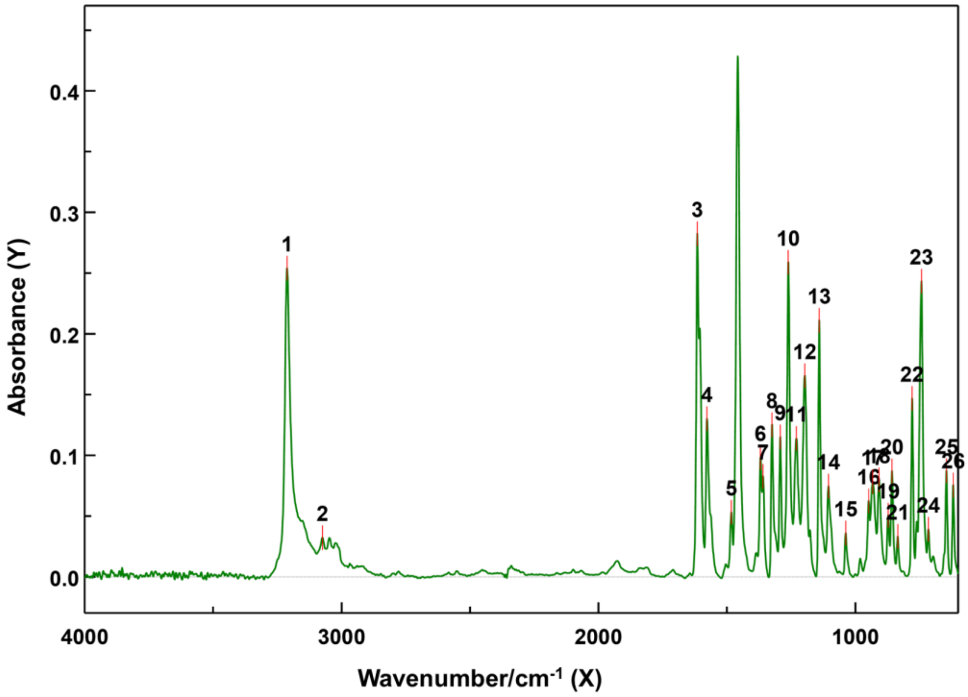

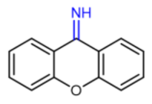

2z

No.	X	Y	No.	X	Y	No.	X	Y
1	3211.9	0.254	10	1261.2	0.259	19	872.6	0.051
2	3074.9	0.032	11	1229.4	0.114	20	857.2	0.087
3	1615.1	0.282	12	1197.6	0.165	21	835	0.033
4	1577.5	0.13	13	1140.7	0.211	22	779.1	0.147
5	1483	0.053	14	1105	0.074	23	742.5	0.243
6	1369.2	0.098	15	1037.5	0.036	24	715.5	0.039
7	1359.6	0.083	16	947.8	0.062	25	645.1	0.087
8	1324.9	0.125	17	932.4	0.078	26	619	0.075
9	1292.1	0.115	18	908.3	0.08			

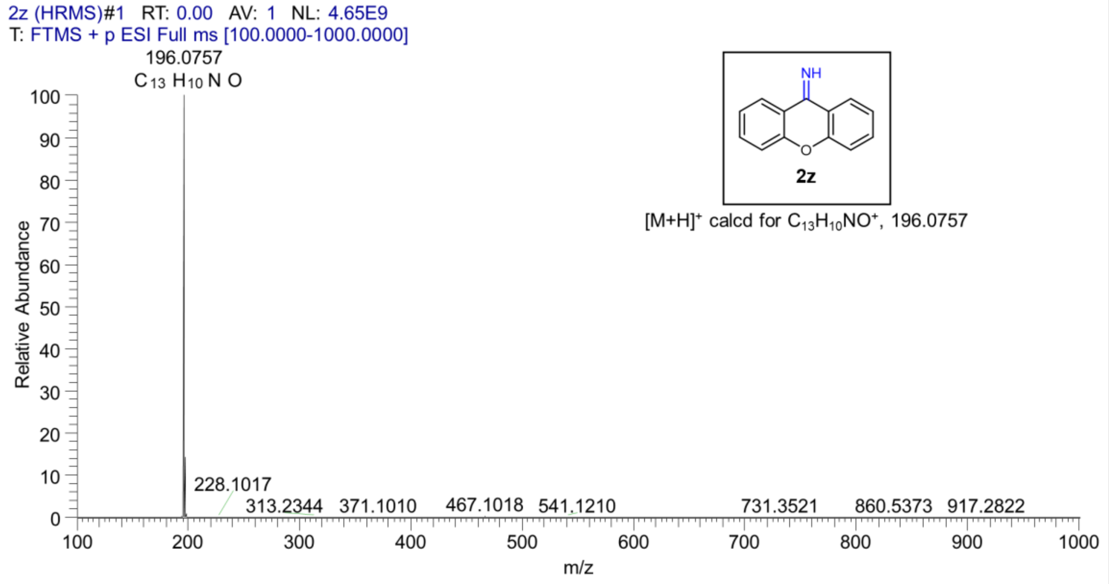

^1H NMR (400 MHz, DMSO-d_6) and ^{13}C NMR (100 MHz, DMSO-d_6) spectra for **2z**·HCl

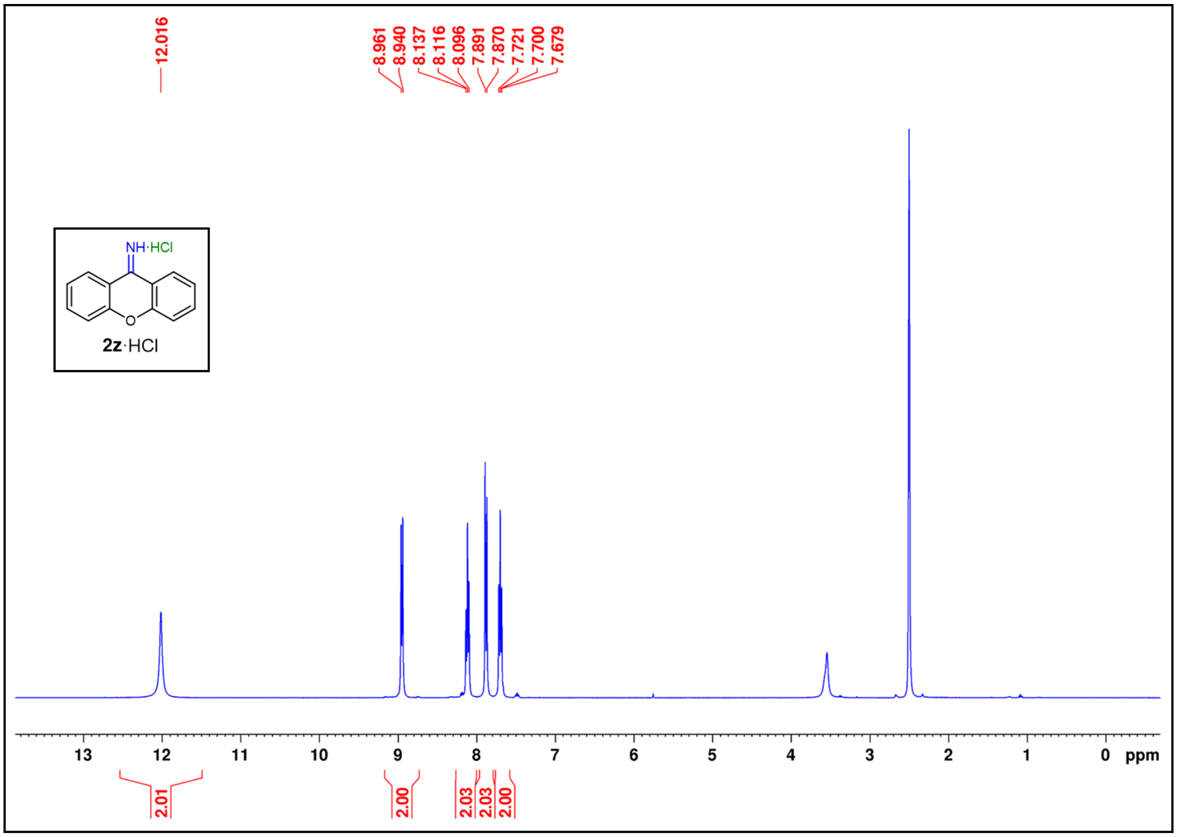

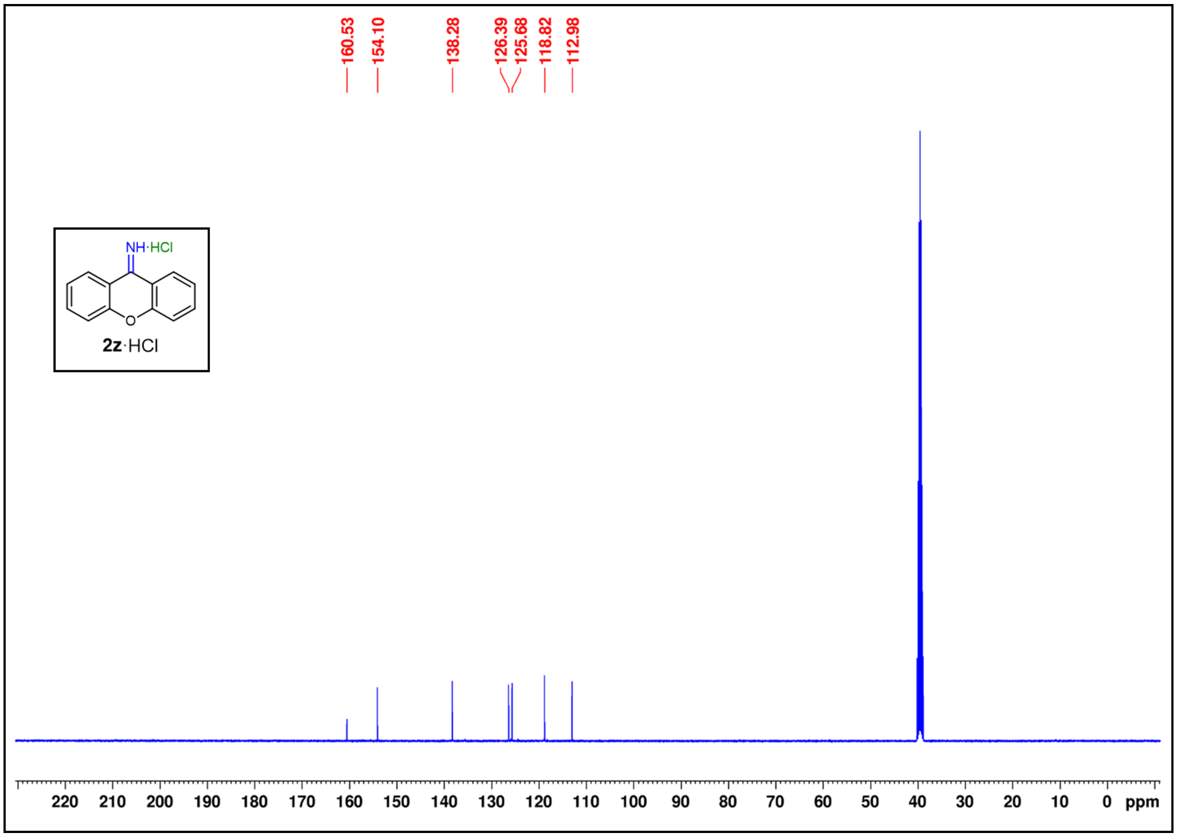

FT-IR (ATR, neat) and HRMS (ESI-positive) spectra for **2z**·HCl

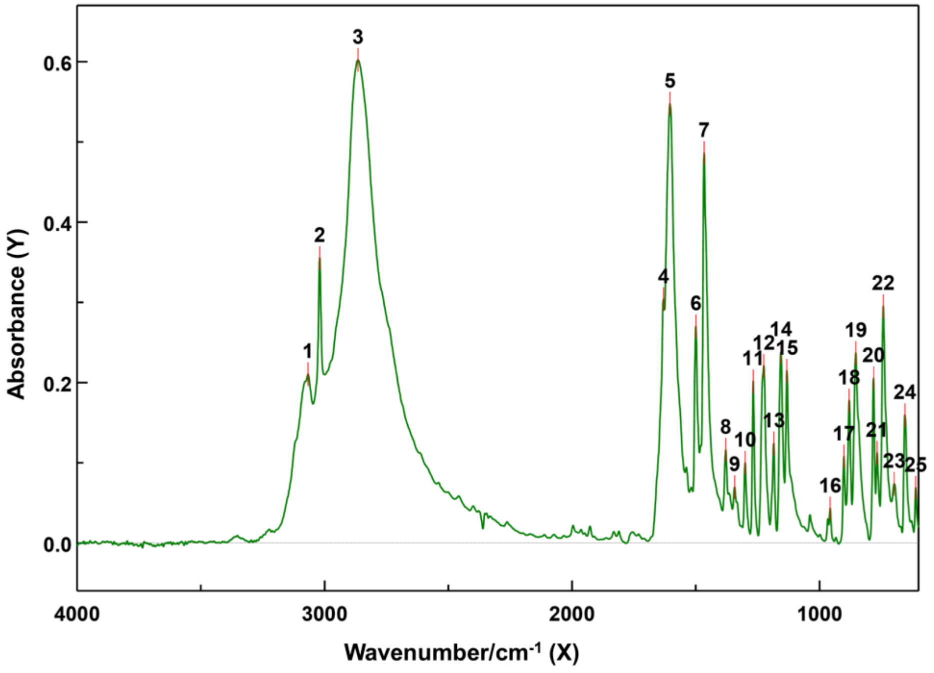

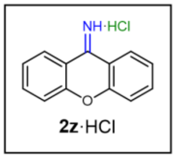

No.	X	Y	No.	X	Y	No.	X	Y
1	3067.2	0.21	10	1300.8	0.1	19	853.3	0.236
2	3020	0.355	11	1268	0.201	20	782	0.205
3	2864.7	0.602	12	1225.5	0.22	21	767.5	0.112
4	1630.5	0.304	13	1185	0.124	22	742.5	0.295
5	1604.5	0.547	14	1156.1	0.238	23	698.1	0.073
6	1499.4	0.27	15	1132	0.214	24	654.7	0.159
7	1466.6	0.486	16	957.5	0.043	25	611.3	0.069
8	1378.9	0.116	17	901.6	0.107			
9	1343.2	0.069	18	880.3	0.177			

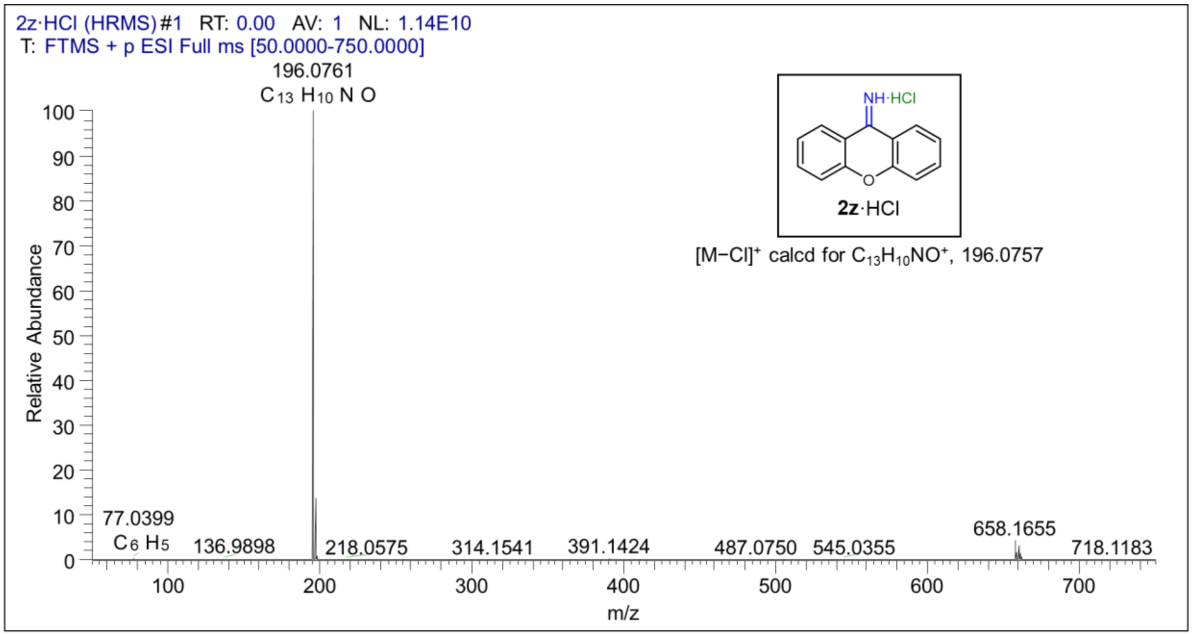

^{1}H NMR (400 MHz, DMSO-d_6) and ^{13}C NMR (100 MHz, DMSO-d_6) spectra for **2aa**

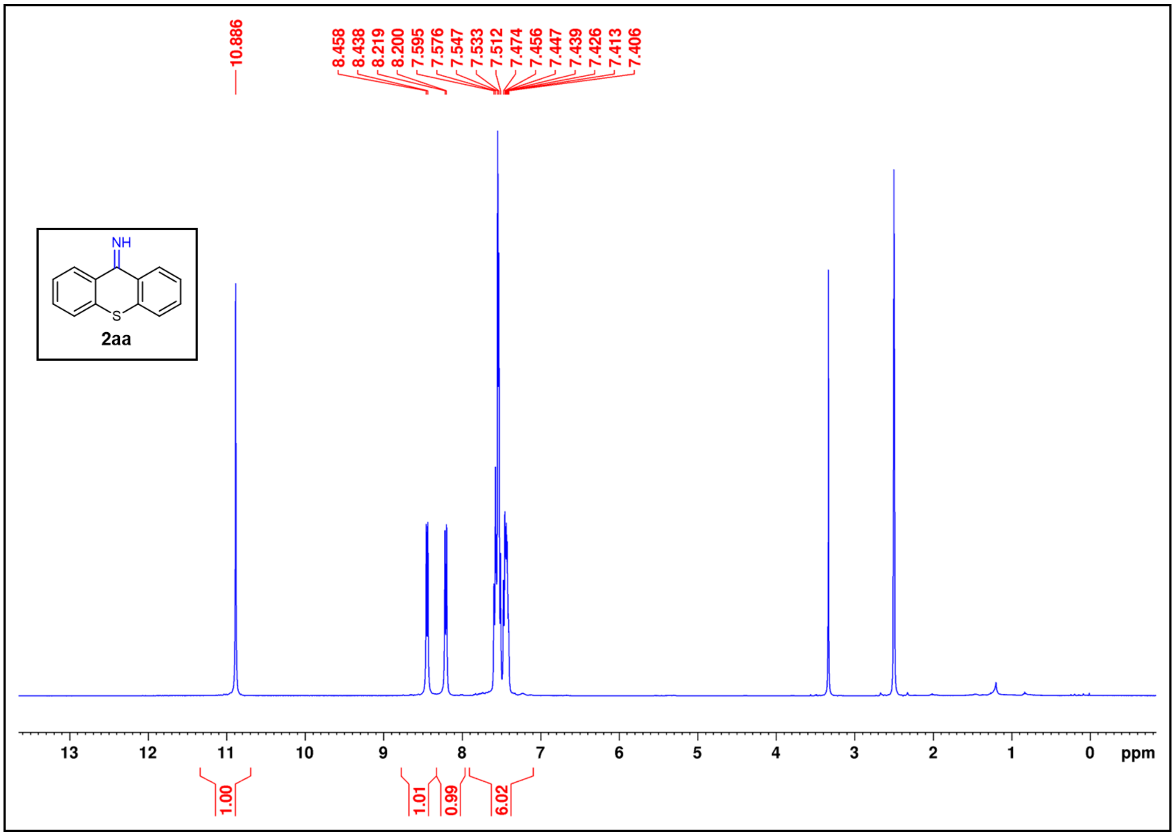

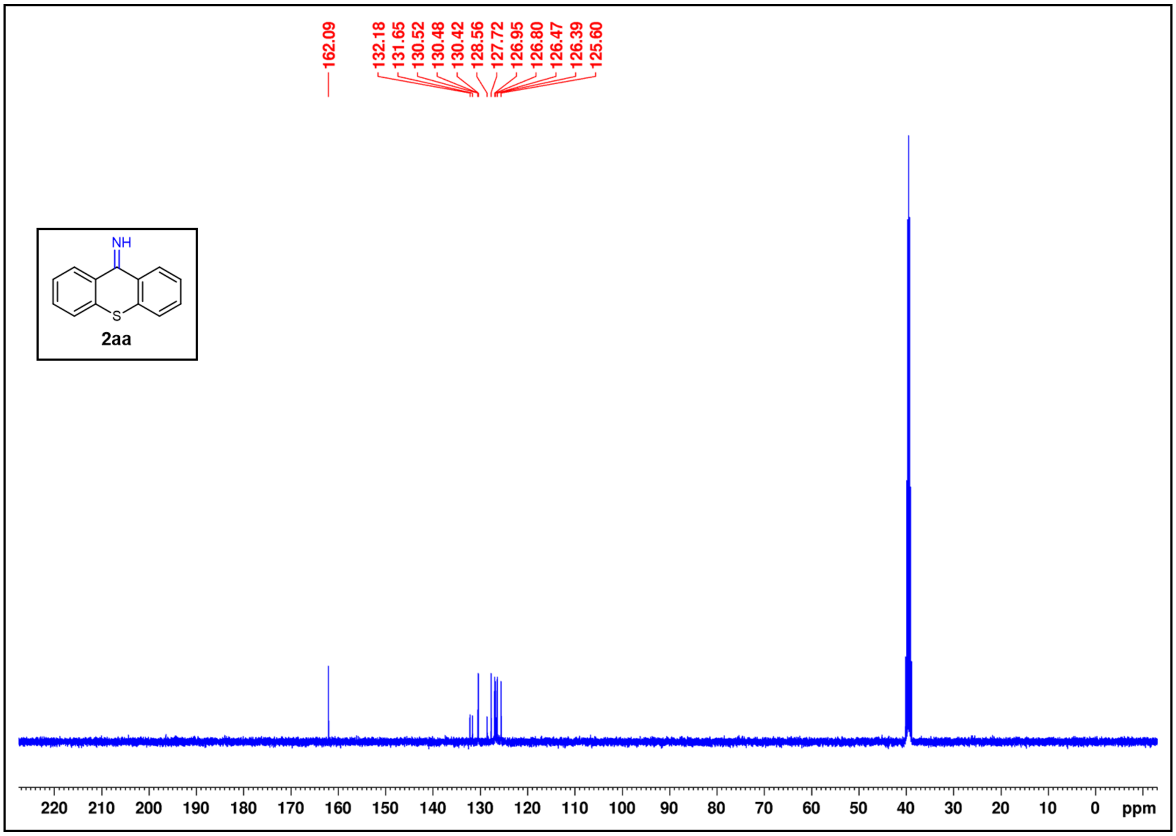

FT-IR (ATR, neat) and HRMS (ESI-positive) spectra for **2aa**

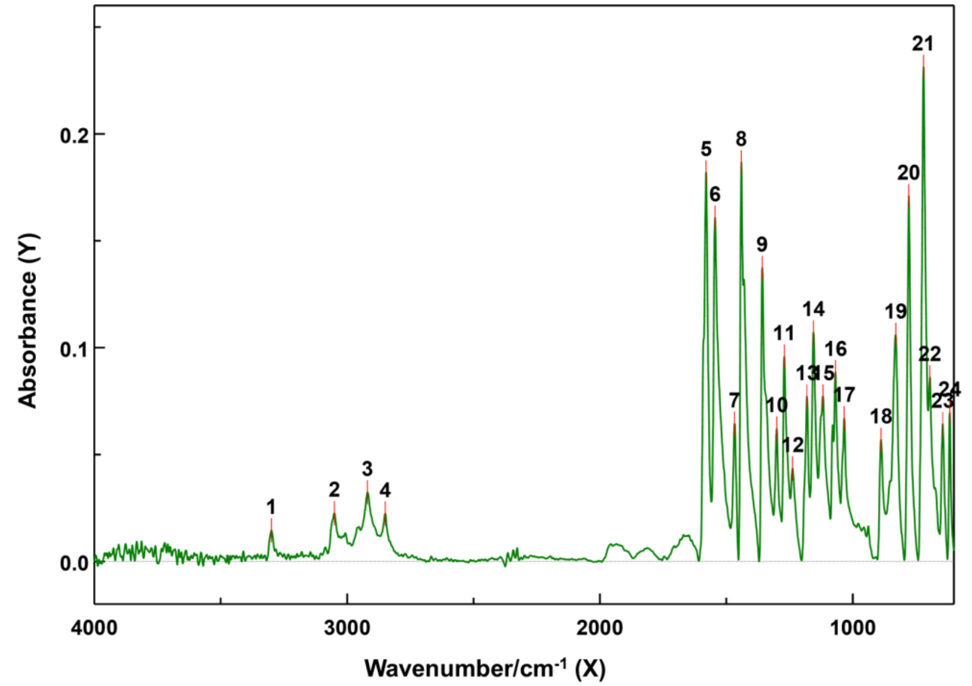

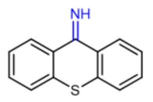

2aa

No.	X	Y	No.	X	Y	No.	X	Y
1	3299.6	0.015	9	1357.6	0.137	17	1034	0.067
2	3050.8	0.022	10	1300.8	0.062	18	888.1	0.057
3	2919.7	0.032	11	1270.9	0.096	19	830.2	0.106
4	2849.3	0.022	12	1238.1	0.043	20	778.1	0.171
5	1580.4	0.182	13	1181.2	0.077	21	720.3	0.231
6	1544.7	0.161	14	1155.2	0.107	22	695.2	0.086
7	1467.6	0.064	15	1117.5	0.077	23	644.1	0.064
8	1440.6	0.187	16	1068.4	0.088	24	616.1	0.069

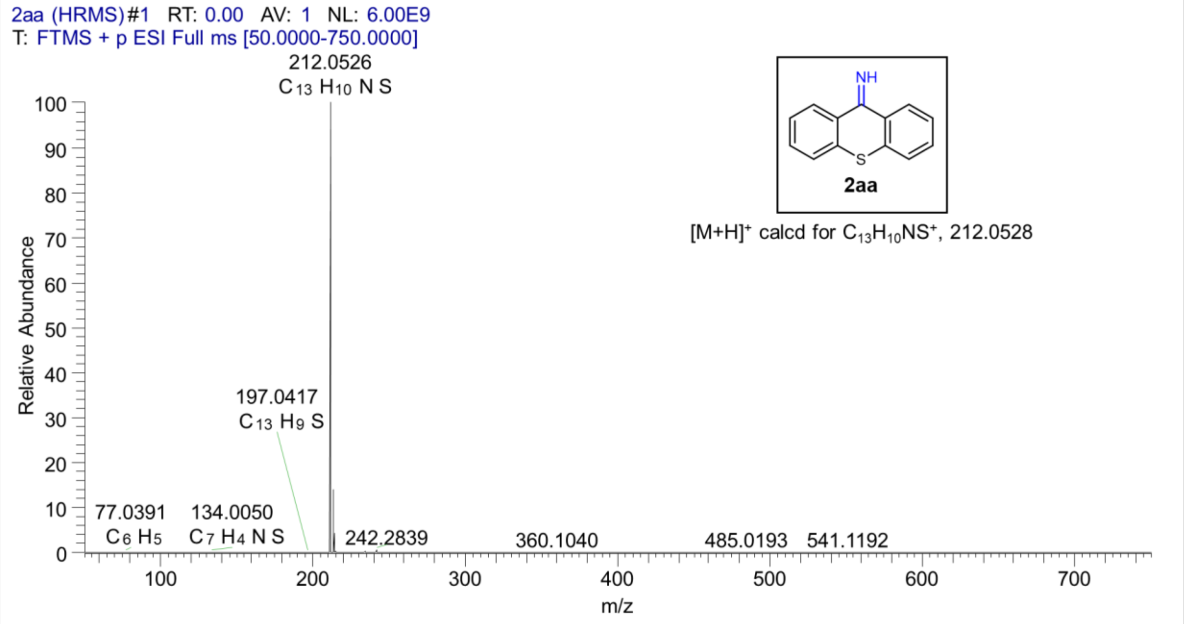

^{1}H NMR (400 MHz, DMSO-d_6) and ^{13}C NMR (100 MHz, DMSO-d_6) spectra for **2ab**

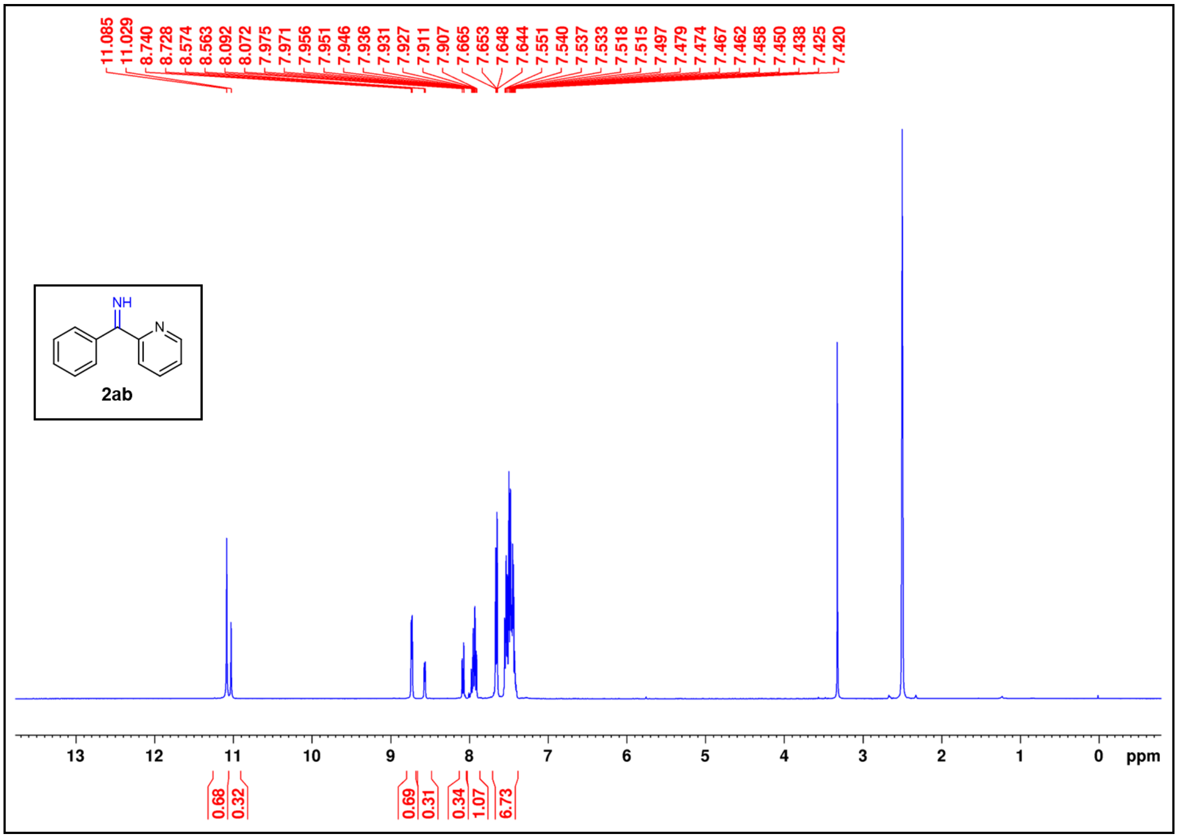

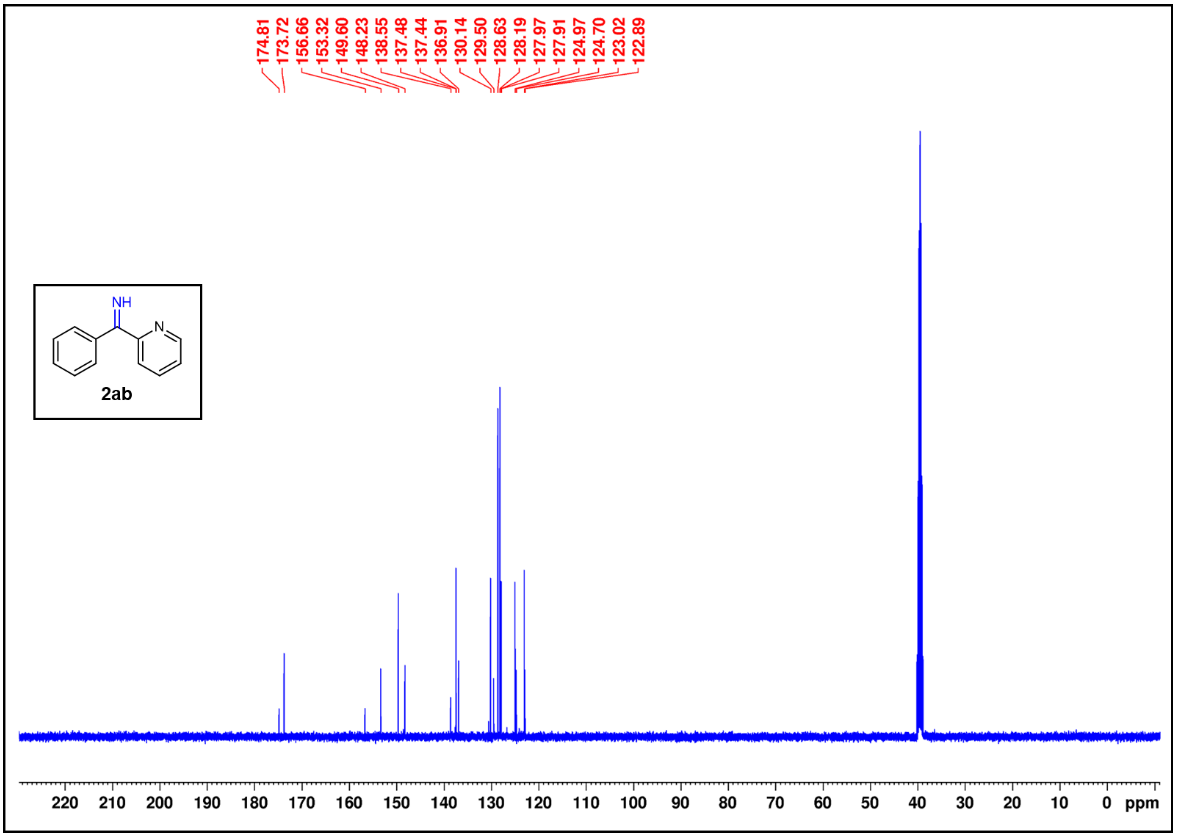

FT-IR (ATR, neat) and HRMS (ESI-positive) spectra for **2ab**

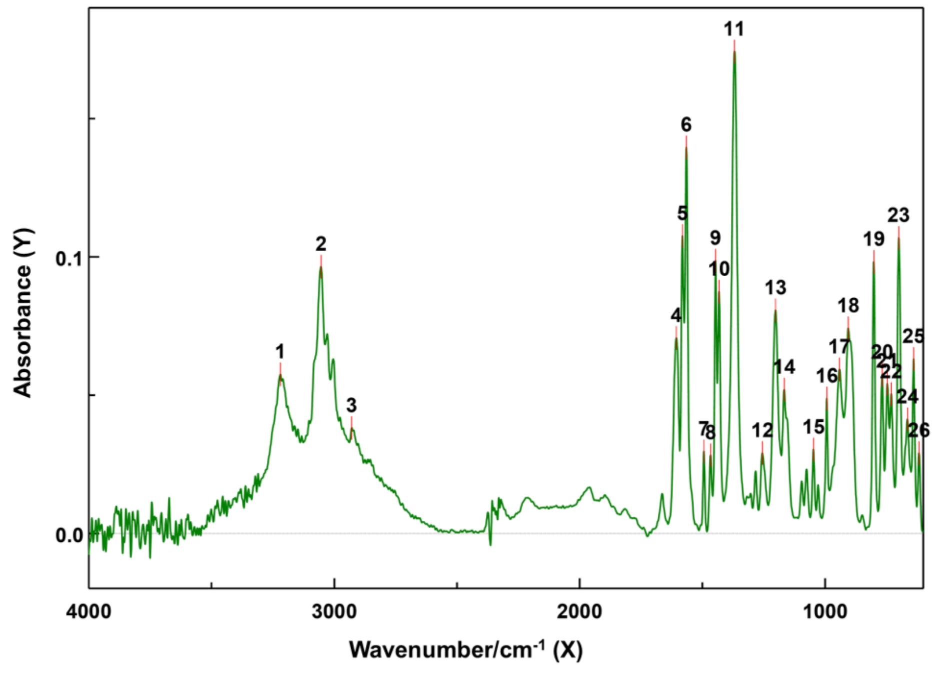

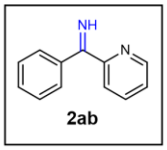

No.	X	Y	No.	X	Y	No.	X	Y
1	3219.6	0.057	10	1431.9	0.087	19	801.3	0.098
2	3053.7	0.096	11	1369.2	0.174	20	767.5	0.057
3	2929.3	0.038	12	1255.4	0.029	21	746.3	0.054
4	1606.4	0.071	13	1202.4	0.081	22	730.9	0.05
5	1581.3	0.107	14	1166.7	0.052	23	700	0.107
6	1565	0.139	15	1047.2	0.03	24	664.4	0.041
7	1493.6	0.03	16	993.2	0.049	25	639.3	0.063
8	1466.6	0.028	17	941.1	0.059	26	617.1	0.029
9	1446.4	0.099	18	905.4	0.074			

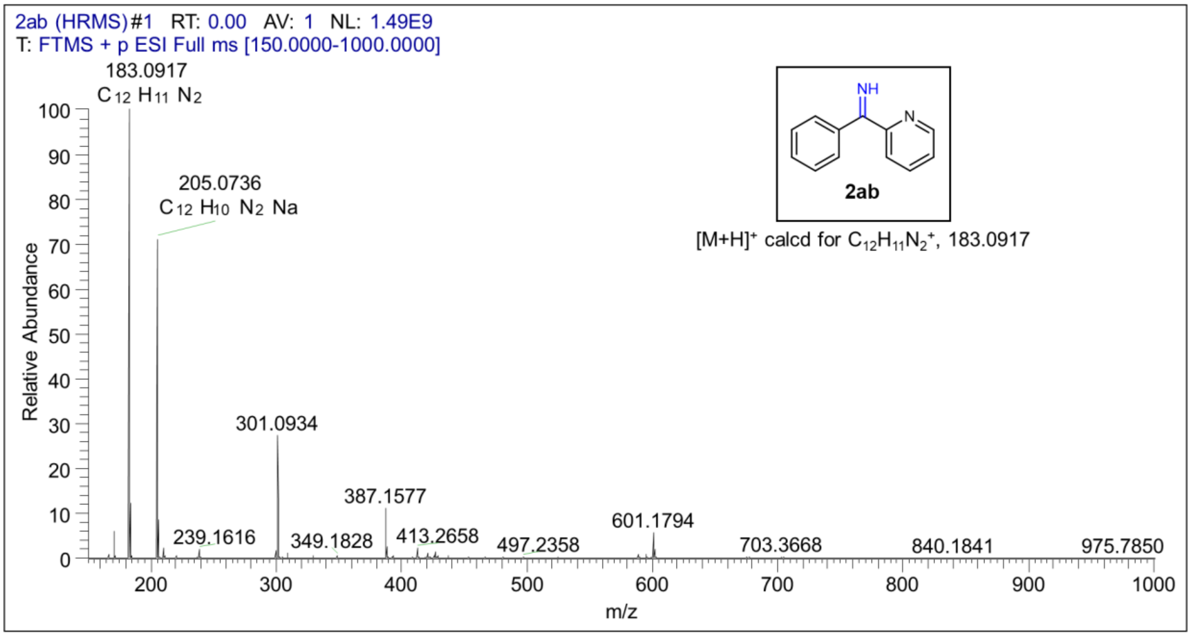

^1H NMR (400 MHz, $CDCl_3$) and ^{13}C NMR (100 MHz, $CDCl_3$) spectra for **2ac**

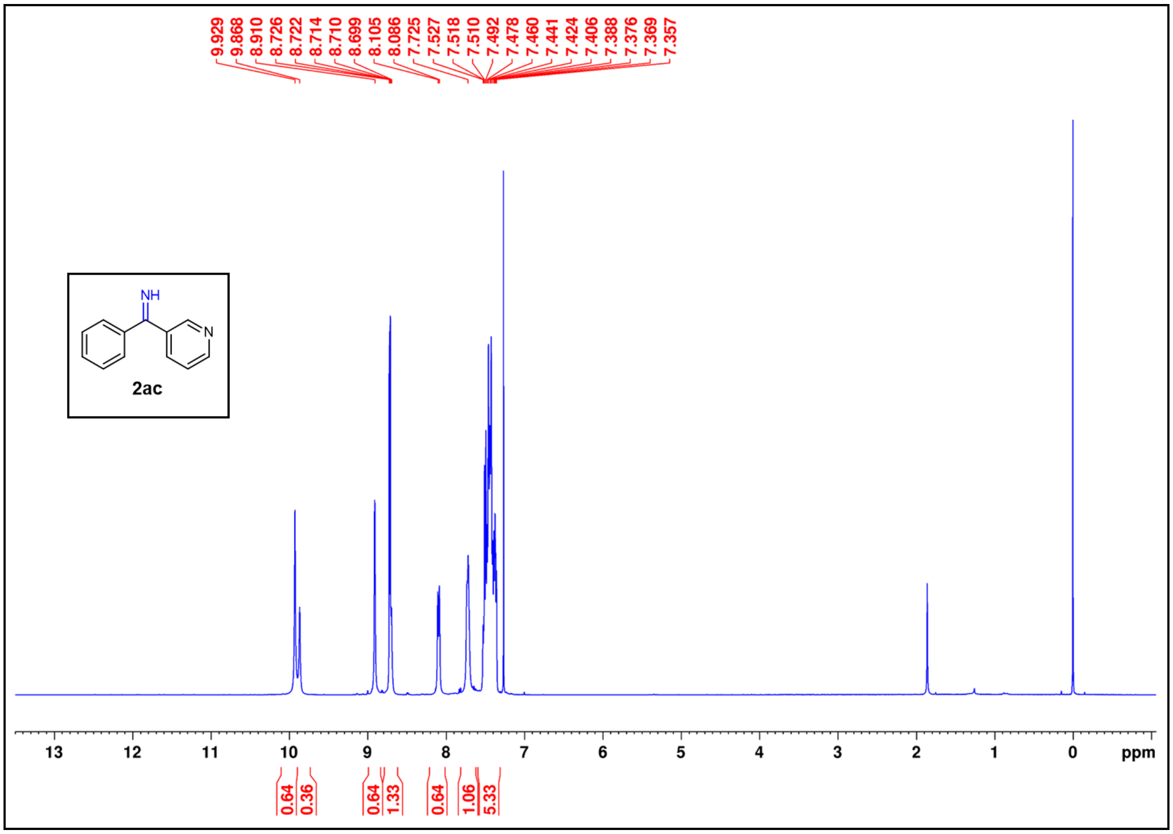

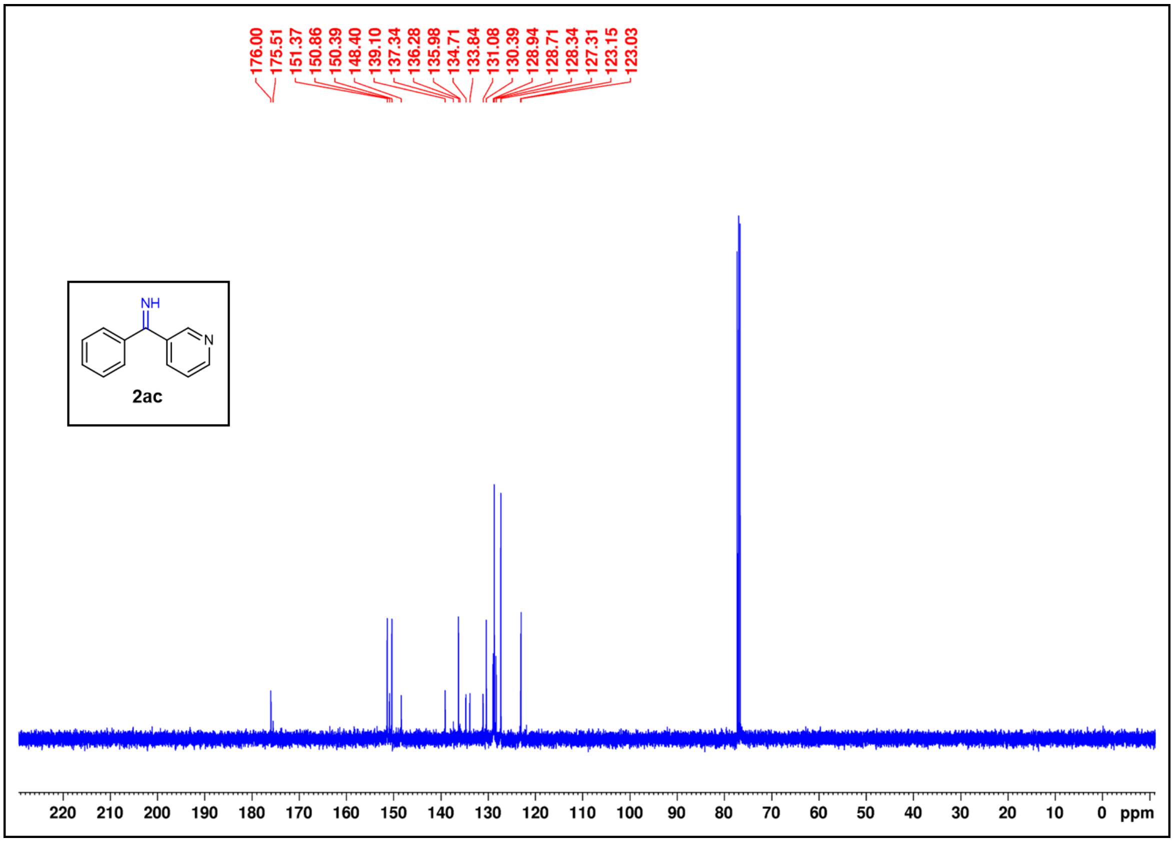

FT-IR (ATR, neat) and HRMS (ESI-positive) spectra for **2ac**

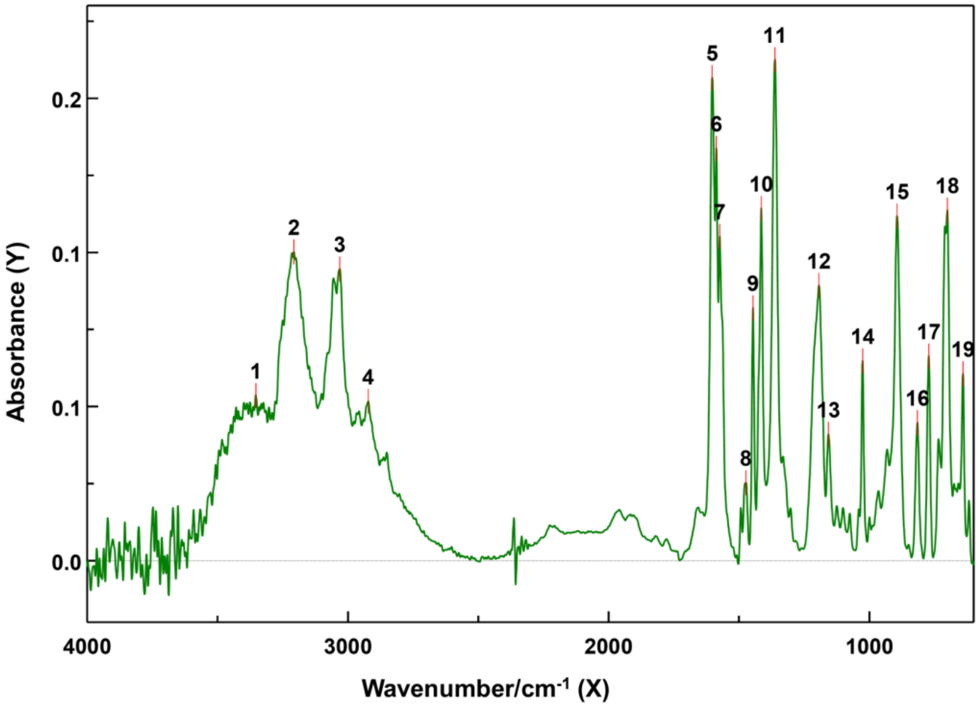

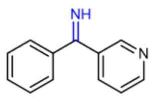

2ac

No.	X	Y	No.	X	Y	No.	X	Y
1	3352.6	0.053	8	1473.3	0.025	15	893.8	0.112
2	3207	0.1	9	1446.4	0.082	16	815.7	0.045
3	3031.6	0.095	10	1414.5	0.114	17	772.4	0.066
4	2922.6	0.052	11	1362.5	0.162	18	701	0.114
5	1602.6	0.157	12	1193.7	0.089	19	641.2	0.061
6	1587.1	0.134	13	1157.1	0.041			
7	1573.6	0.105	14	1025.9	0.065			

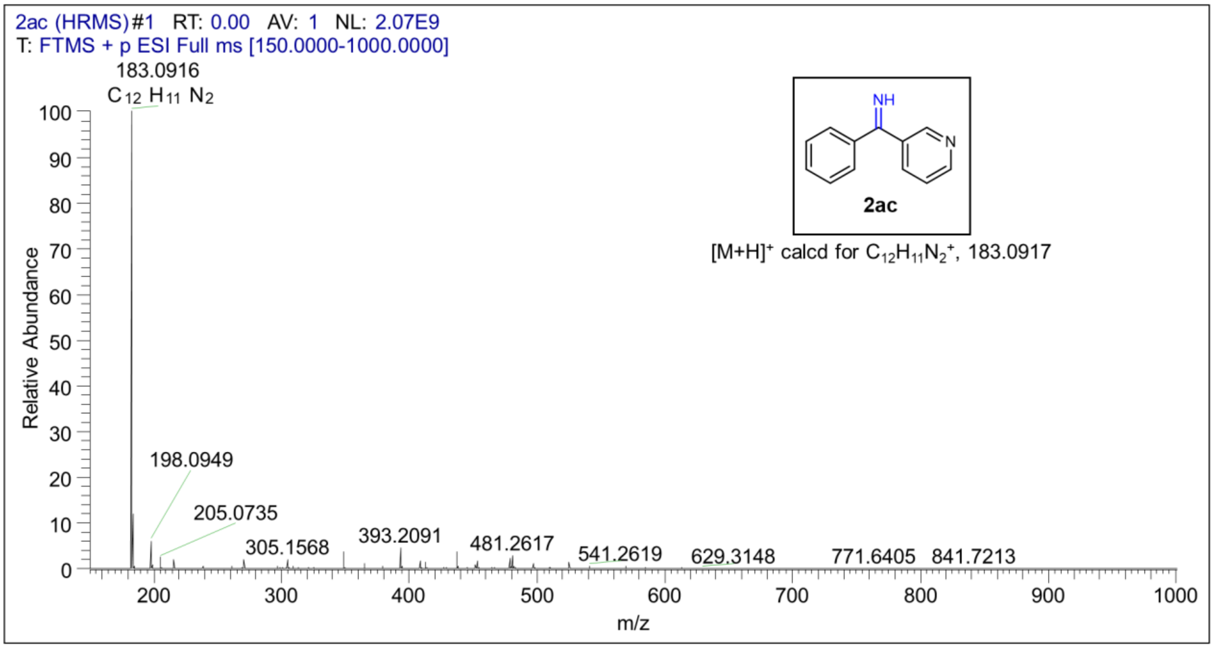

^{1}H NMR (400 MHz, $CDCl_3$) and ^{13}C NMR (100 MHz, $CDCl_3$) spectra for **2ad**

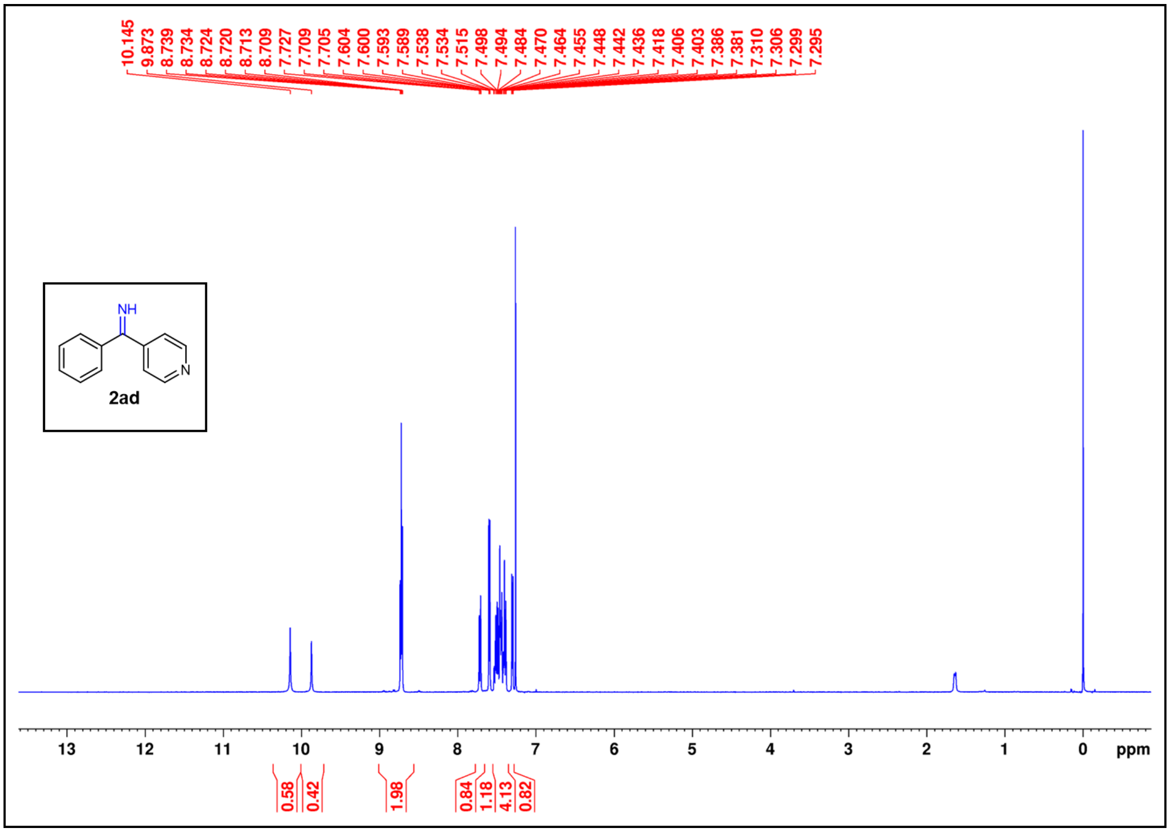

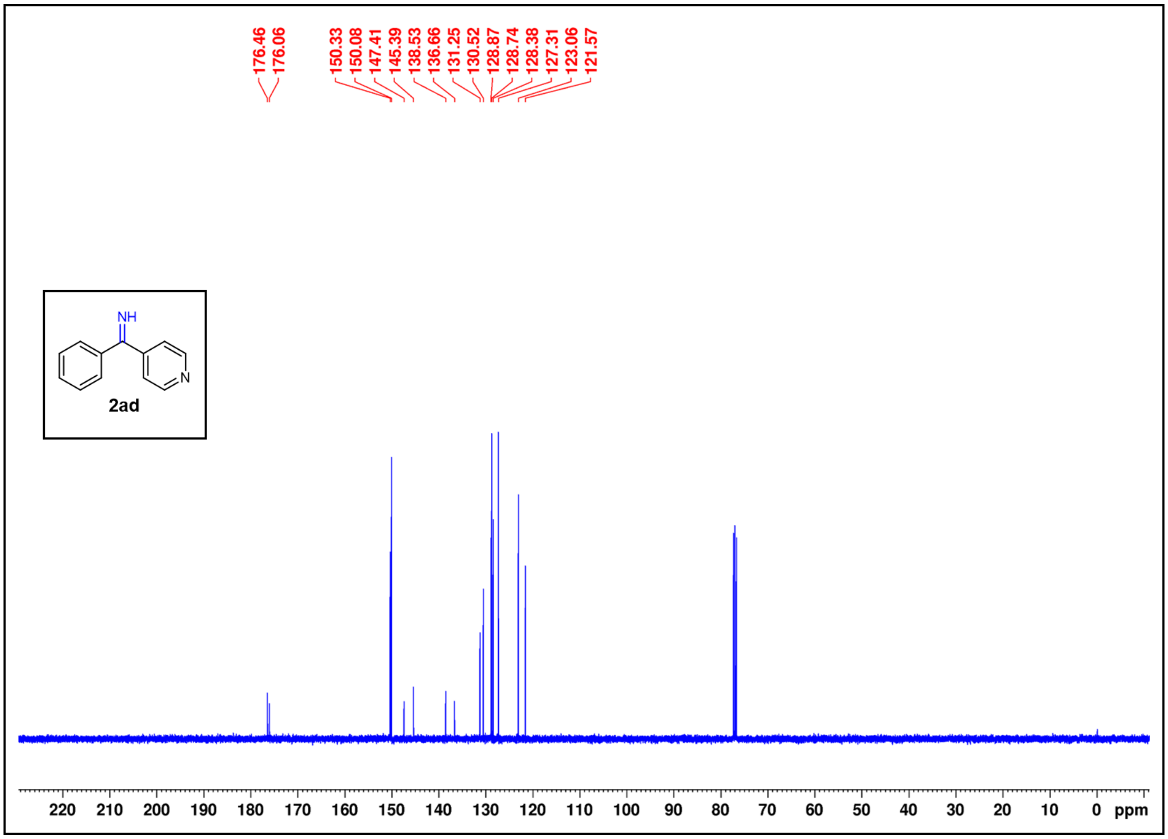

FT-IR (ATR, neat) and HRMS (ESI-positive) spectra for **2ad**

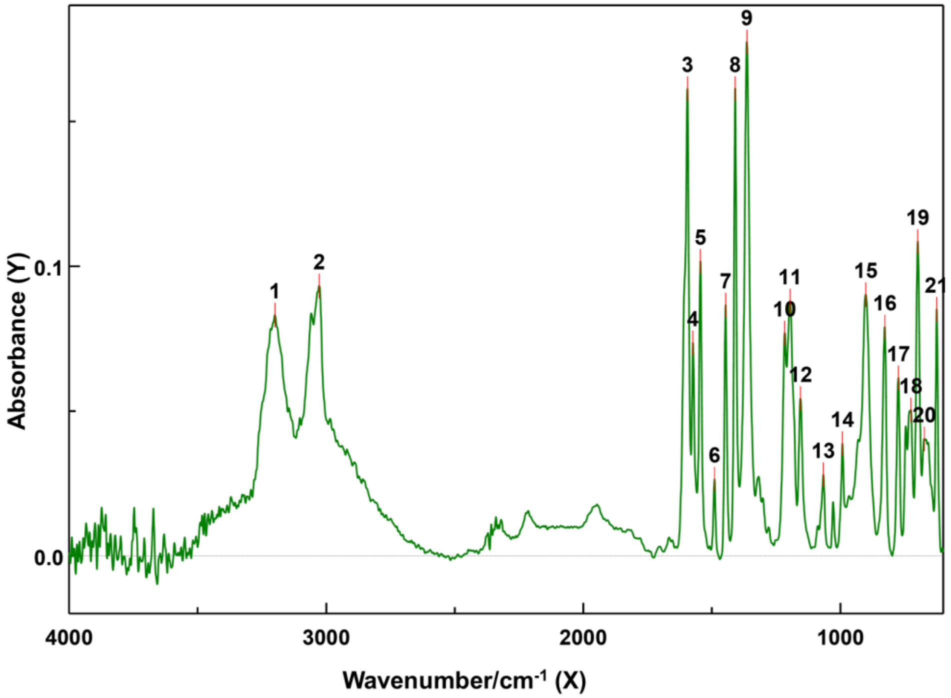

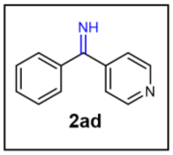

No.	X	Y	No.	X	Y	No.	X	Y
1	3199.3	0.083	8	1409.7	0.161	15	901.6	0.09
2	3026.7	0.093	9	1363.4	0.177	16	827.3	0.079
3	1594.8	0.161	10	1216.9	0.077	17	774.3	0.061
4	1573.6	0.073	11	1195.7	0.088	18	726.1	0.05
5	1544.7	0.102	12	1155.2	0.054	19	699.1	0.108
6	1489.7	0.026	13	1066.4	0.028	20	673	0.04
7	1446.4	0.086	14	992.2	0.039	21	625.8	0.085

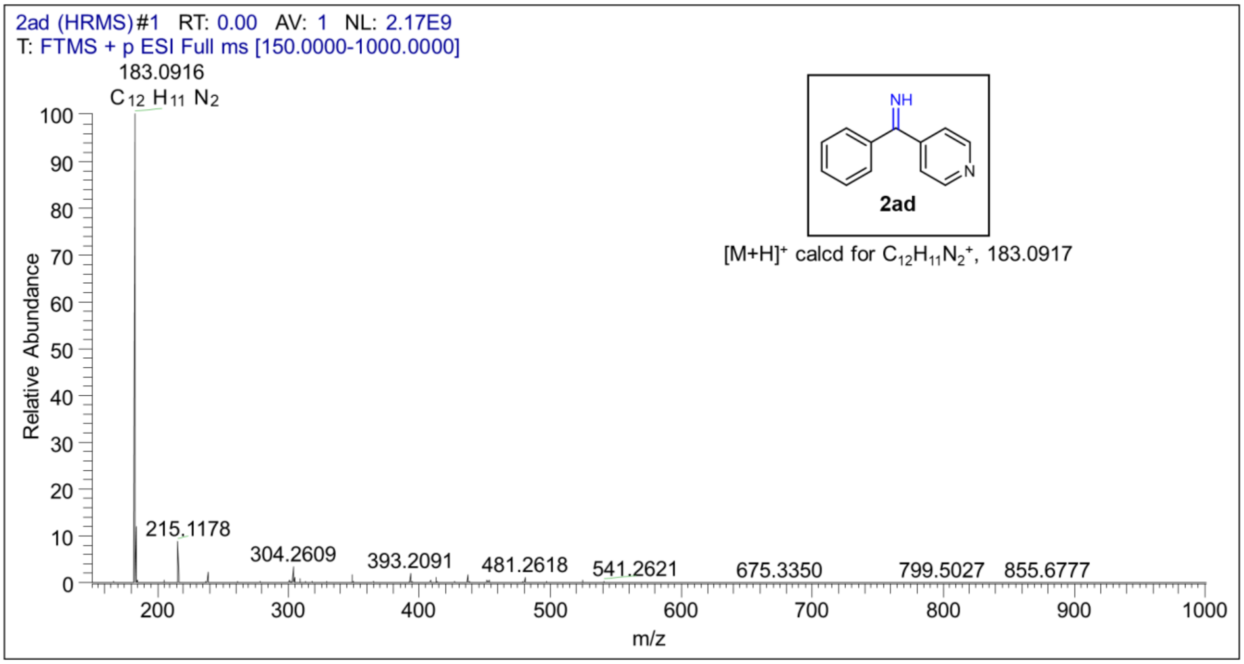

^{1}H NMR (400 MHz, $CDCl_3$) and ^{13}C NMR (100 MHz, $CDCl_3$) spectra for **2ae**

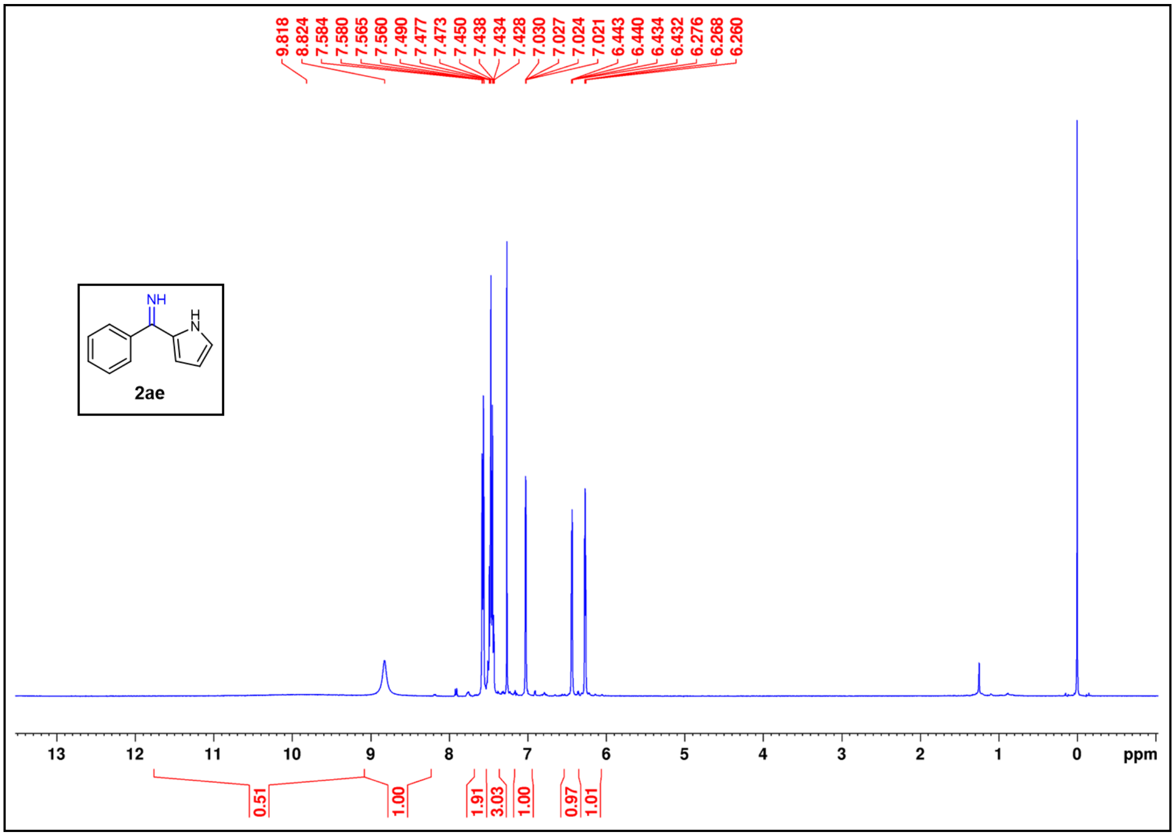

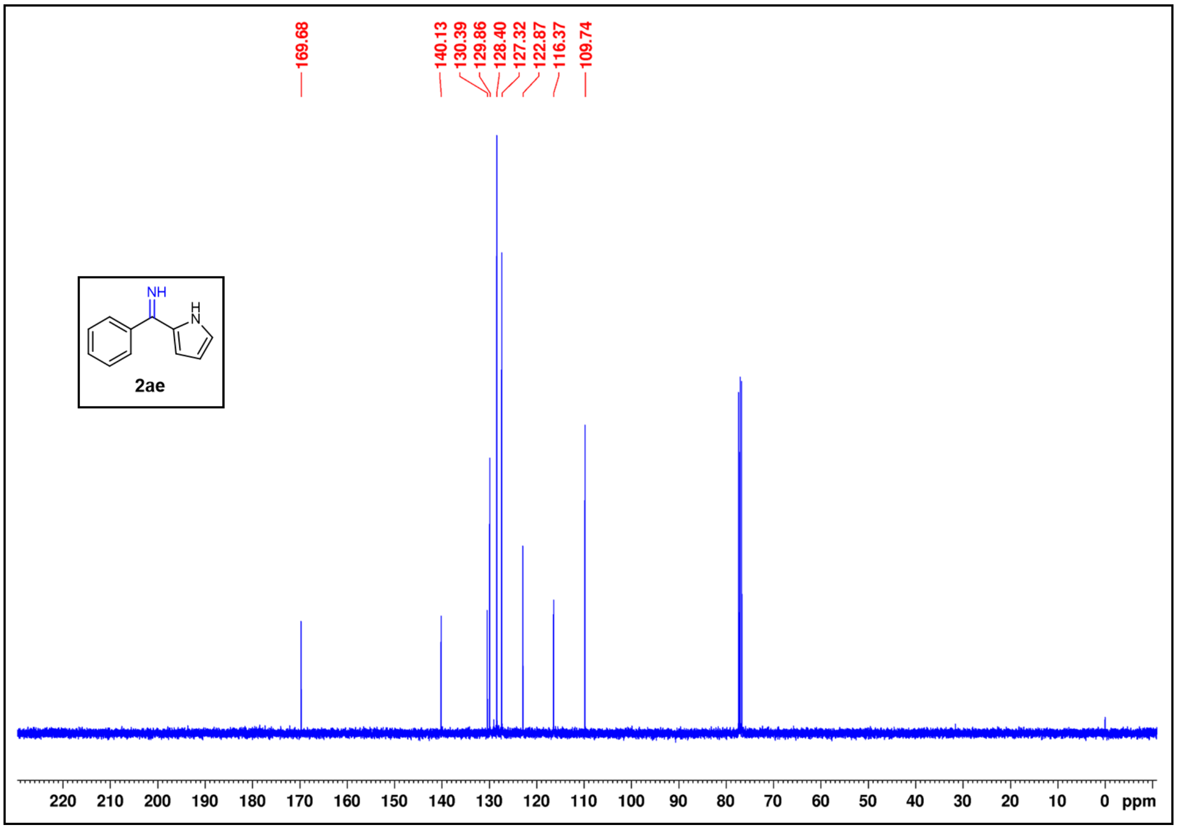

FT-IR (ATR, neat) and HRMS (ESI-positive) spectra for **2ae**

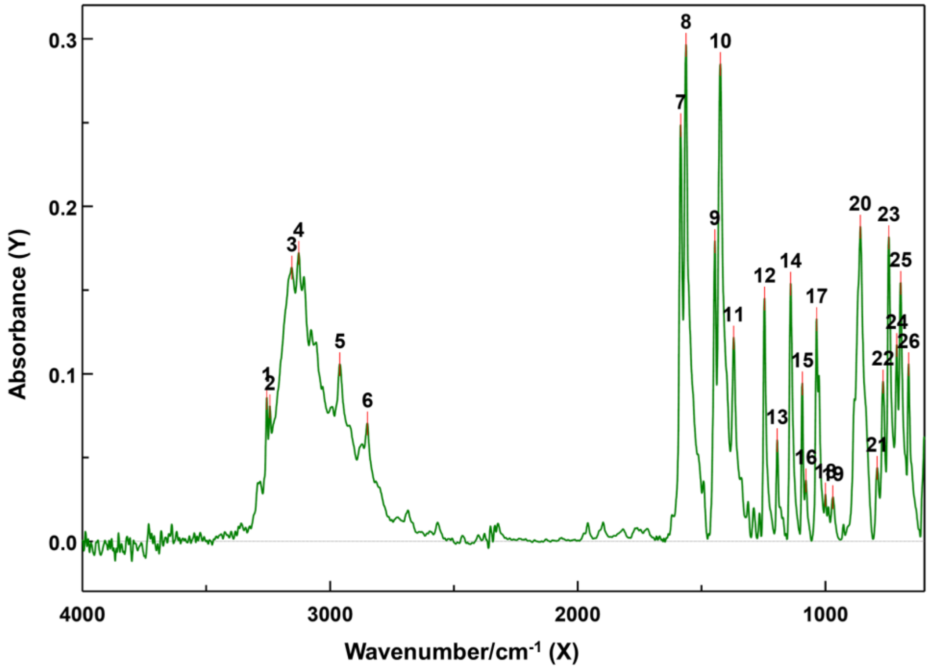

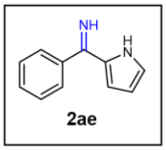

No.	X	Y	No.	X	Y	No.	X	Y
1	3255.3	0.086	10	1424.2	0.285	19	970	0.026
2	3242.7	0.081	11	1370.2	0.122	20	859.1	0.188
3	3155	0.163	12	1245.8	0.145	21	790.7	0.044
4	3126	0.172	13	1194.7	0.06	22	767.5	0.095
5	2960.2	0.106	14	1140.7	0.154	23	744.4	0.181
6	2849.3	0.07	15	1093.4	0.094	24	711.6	0.117
7	1584.2	0.248	16	1079	0.036	25	696.2	0.154
8	1563	0.296	17	1035.6	0.133	26	664.4	0.106
9	1446.4	0.179	18	999.9	0.028			

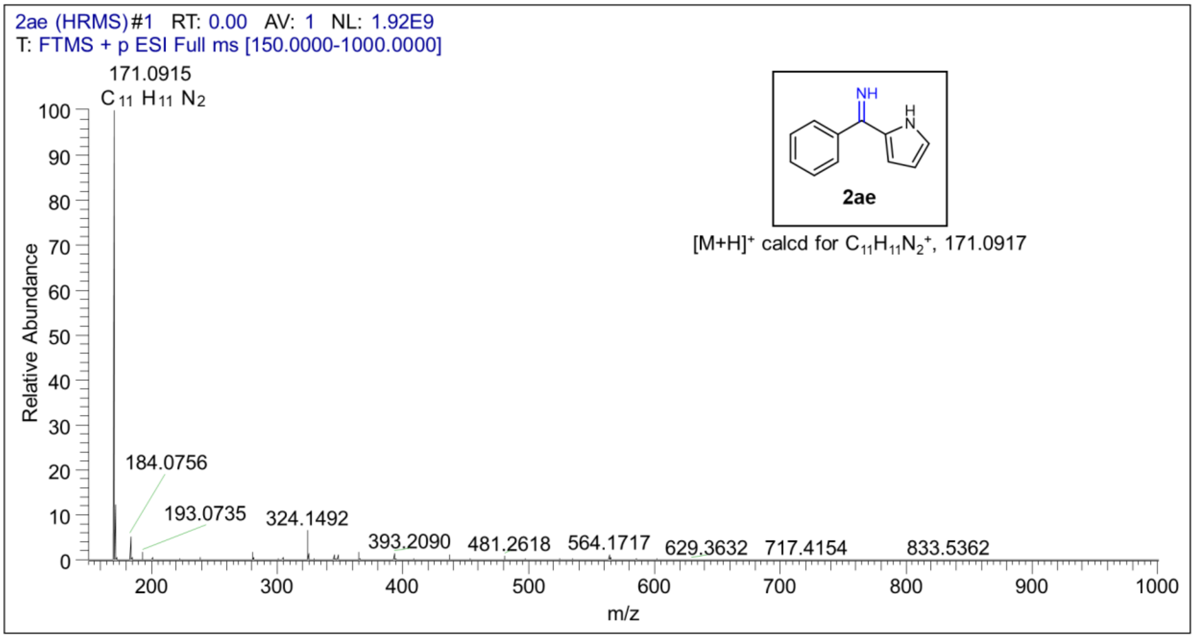

^{1}H NMR (400 MHz, DMSO-d_6) and ^{13}C NMR (100 MHz, DMSO-d_6) spectra for **2af**

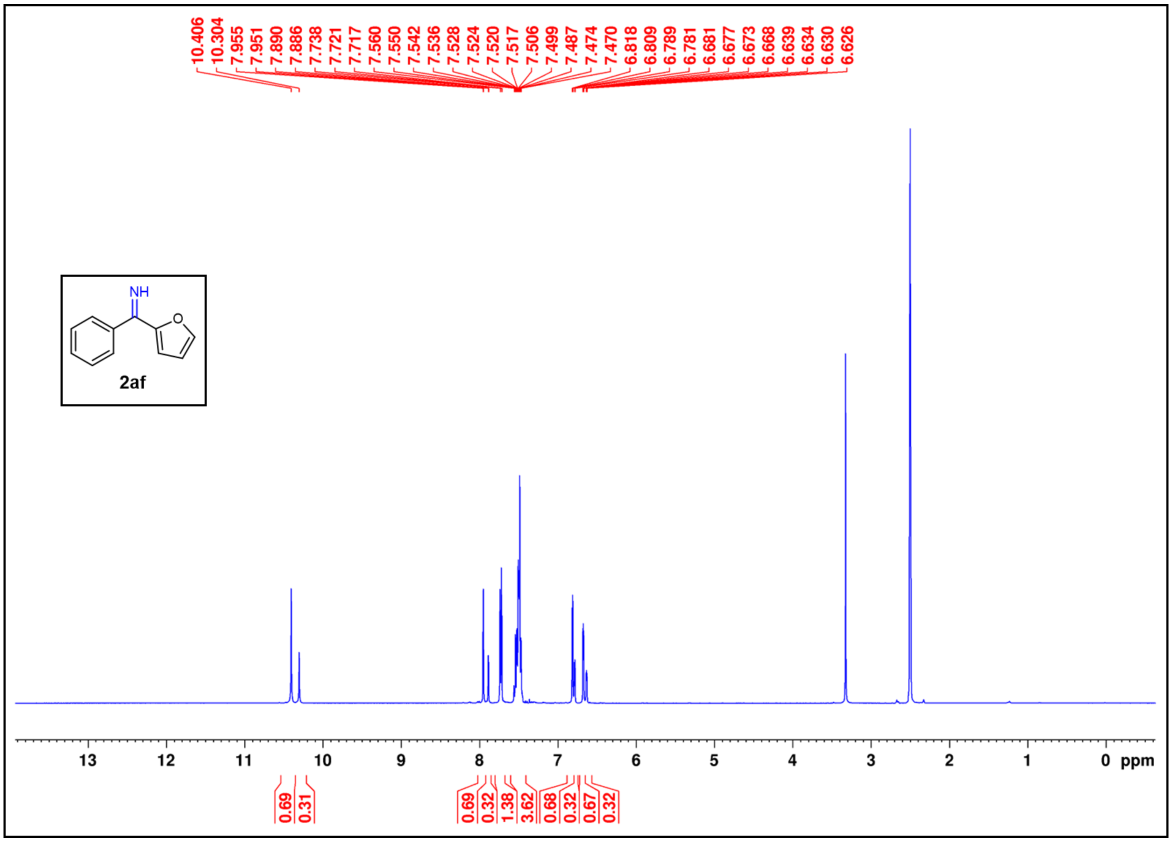

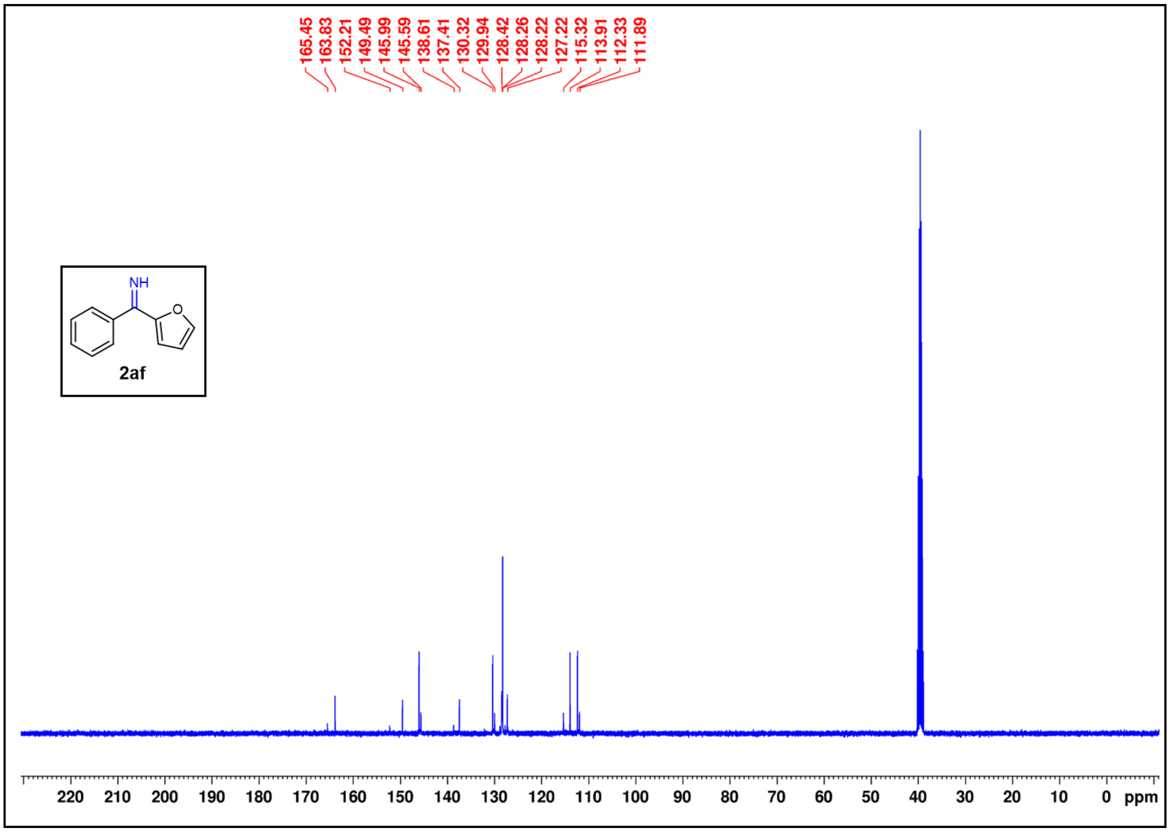

FT-IR (ATR, neat) and HRMS (ESI-positive) spectra for **2af**

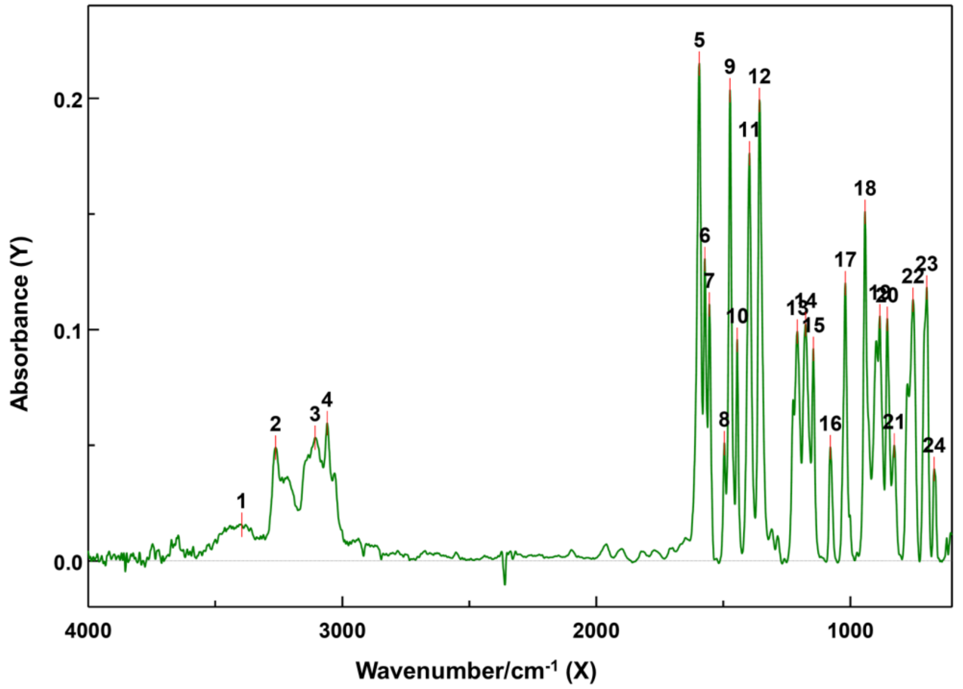

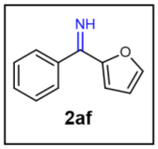

No.	X	Y	No.	X	Y	No.	X	Y
1	3396	0.016	9	1473.3	0.203	17	1020	0.12
2	3263	0.049	10	1445.4	0.095	18	942.1	0.151
3	3106.8	0.053	11	1397.2	0.176	19	884.2	0.106
4	3059.5	0.059	12	1357.6	0.199	20	855.3	0.105
5	1594.8	0.215	13	1209.1	0.099	21	827.3	0.05
6	1572.7	0.13	14	1176.4	0.102	22	753.1	0.113
7	1555.3	0.111	15	1145.5	0.092	23	699.1	0.118
8	1496.5	0.051	16	1079	0.049	24	670.1	0.04

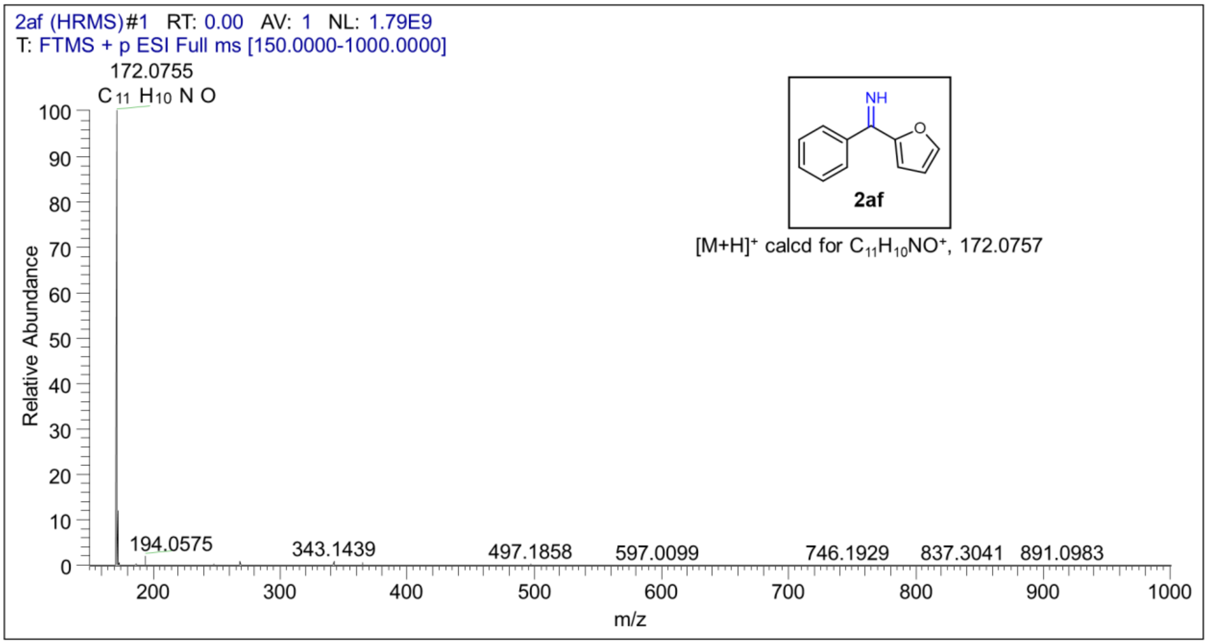

^{1}H NMR (400 MHz, DMSO-d_6) and ^{13}C NMR (100 MHz, DMSO-d_6) spectra for **2ag**

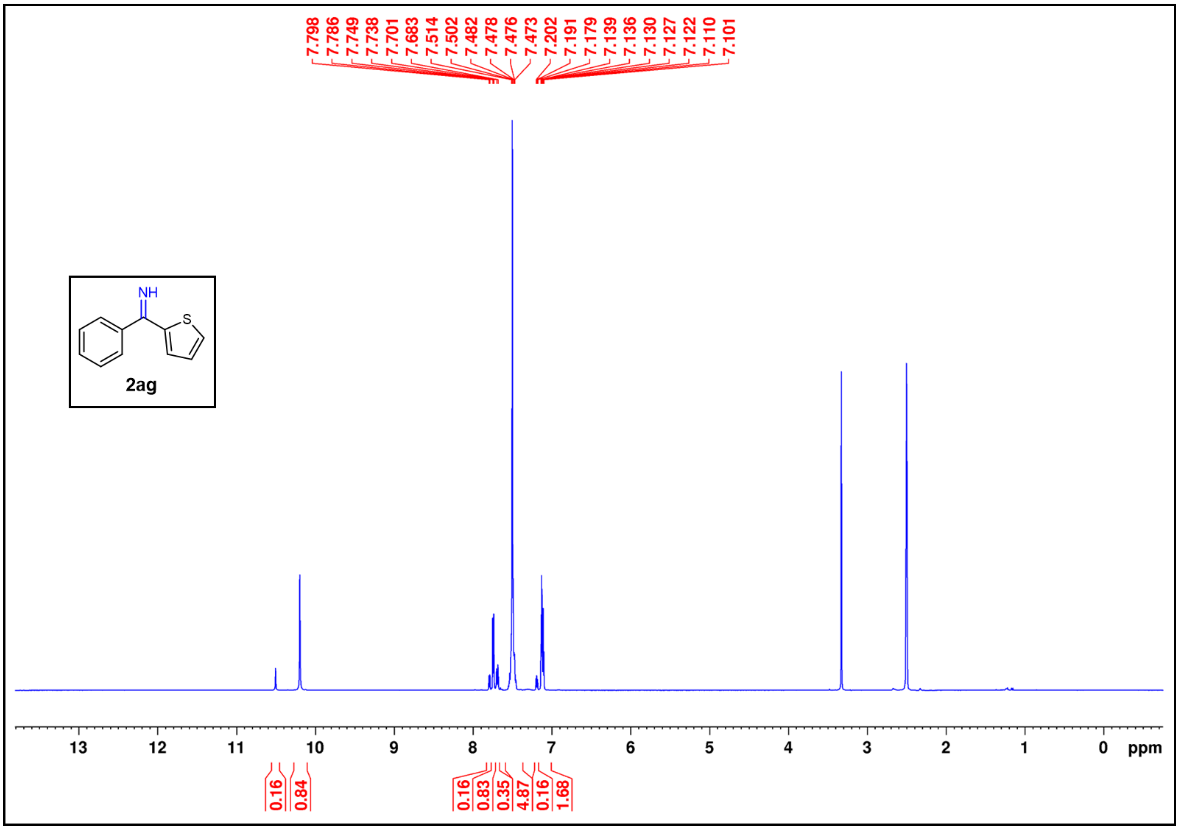

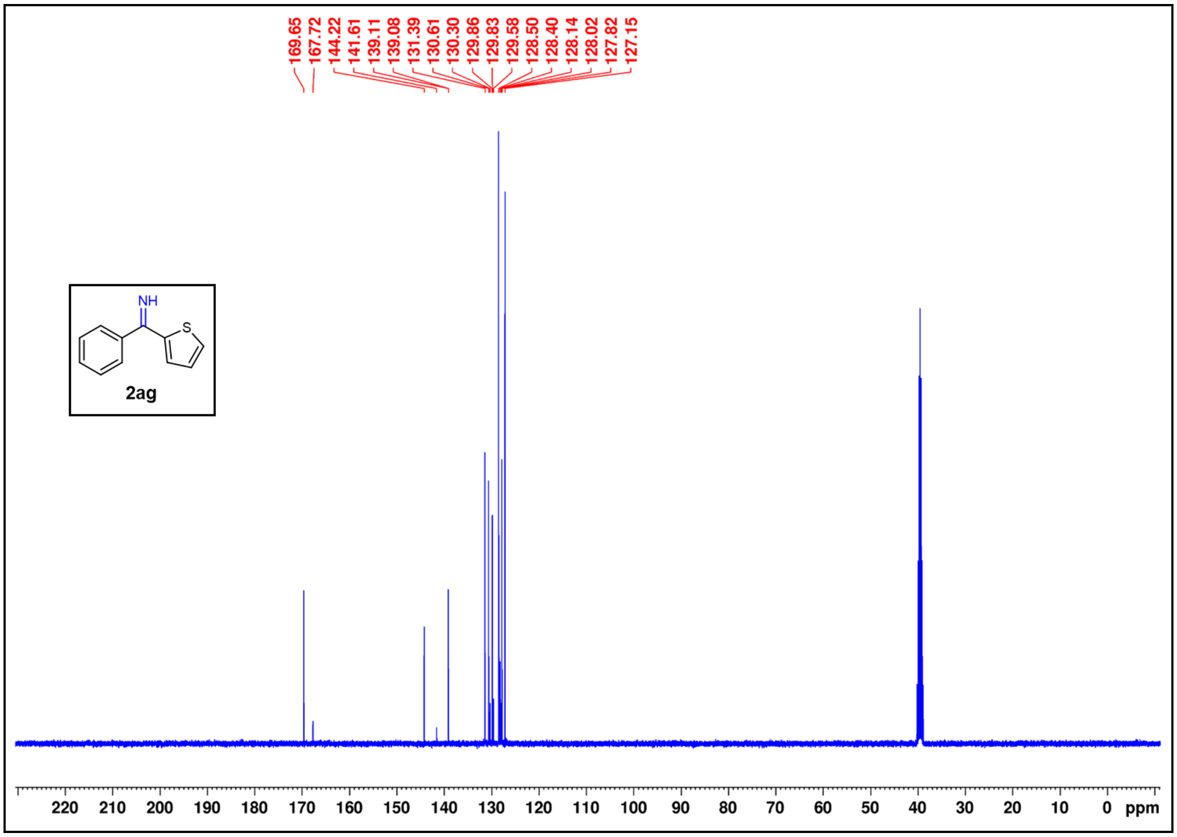

FT-IR (ATR, neat) and HRMS (ESI-positive) spectra for **2ag**

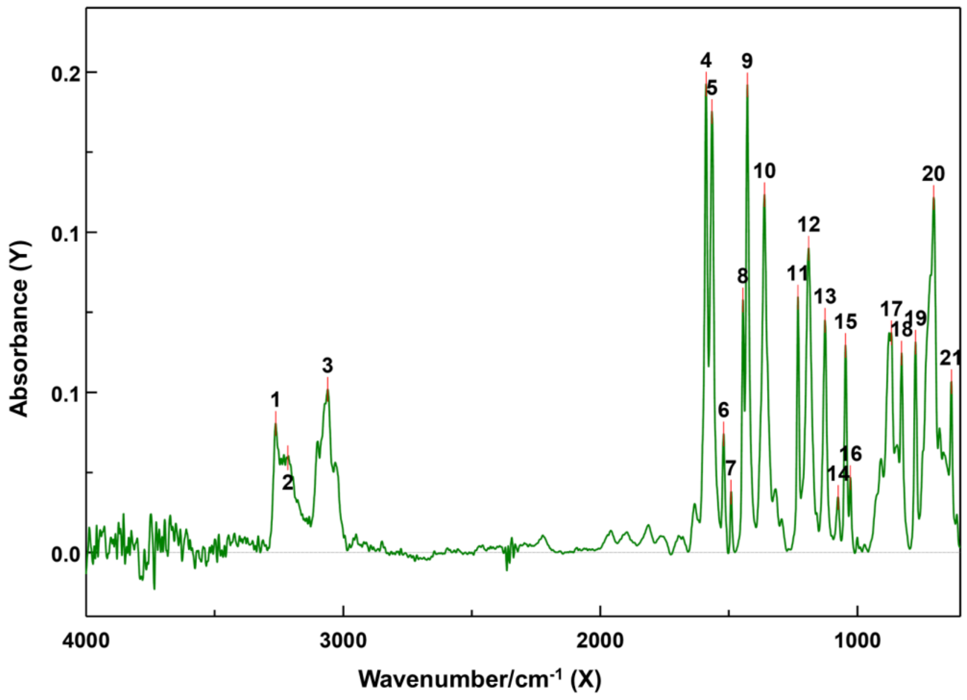

No.	X	Y		No.	X	Y		No.	X	Y
1	3263	0.04		8	1445.4	0.079		15	1046	0.065
2	3215.7	0.03		9	1428	0.146		16	1027	0.023
3	3060.5	0.051		10	1361.5	0.112		17	867.8	0.069
4	1589.1	0.146		11	1231.3	0.08		18	828.3	0.062
5	1565.9	0.138		12	1189.9	0.095		19	774.3	0.066
6	1520.6	0.037		13	1126.2	0.072		20	702.9	0.111
7	1491.7	0.019		14	1075.1	0.017		21	634.5	0.053

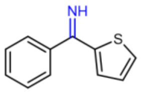

2ag

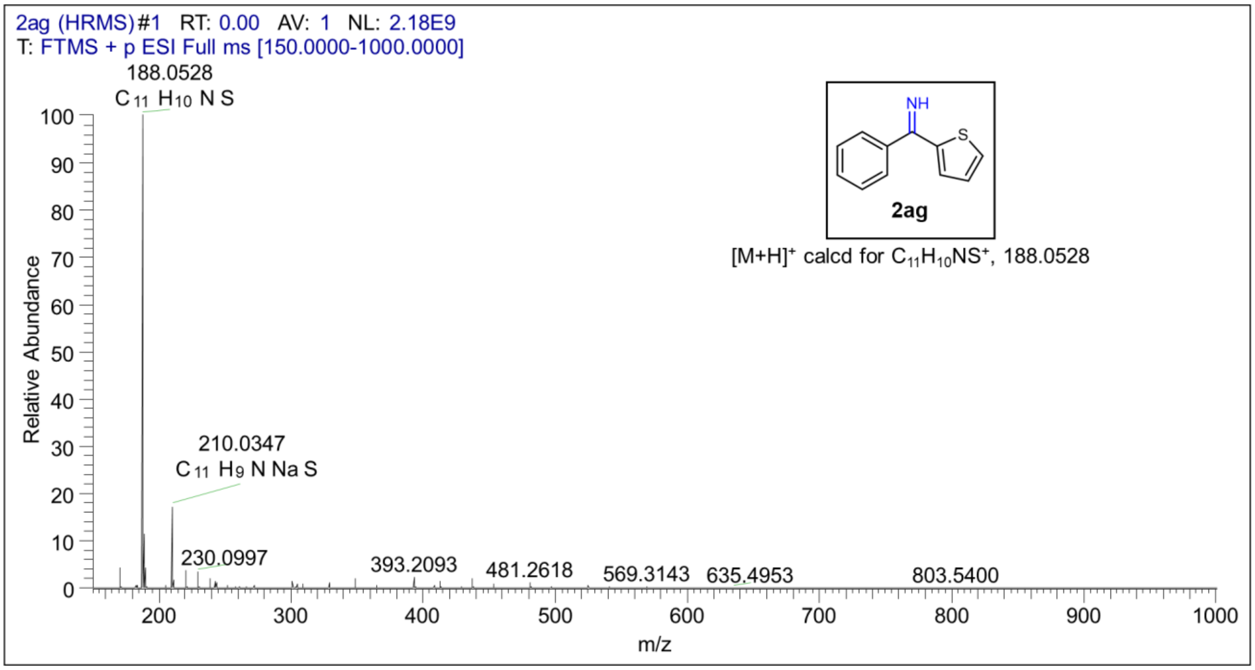

^{1}H NMR (400 MHz, DMSO-d_6) and ^{13}C NMR (100 MHz, DMSO-d_6) spectra for **2ag**·HCl

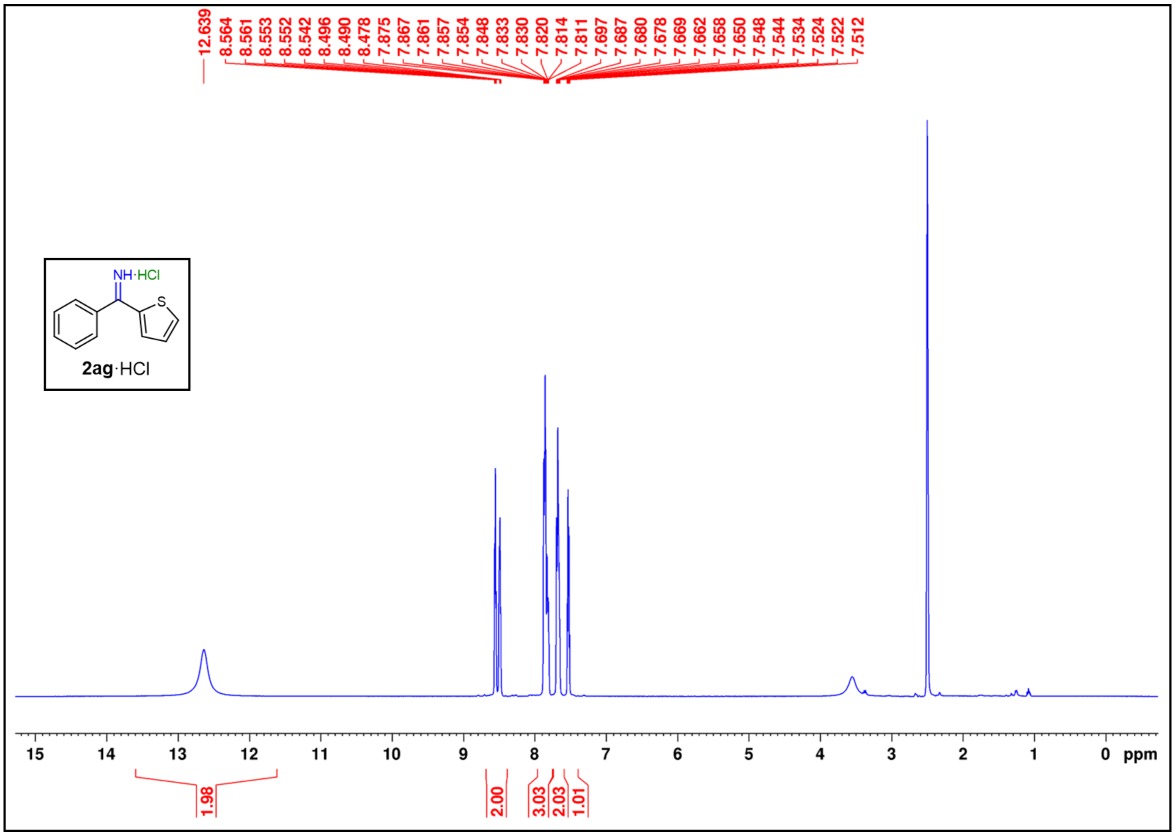

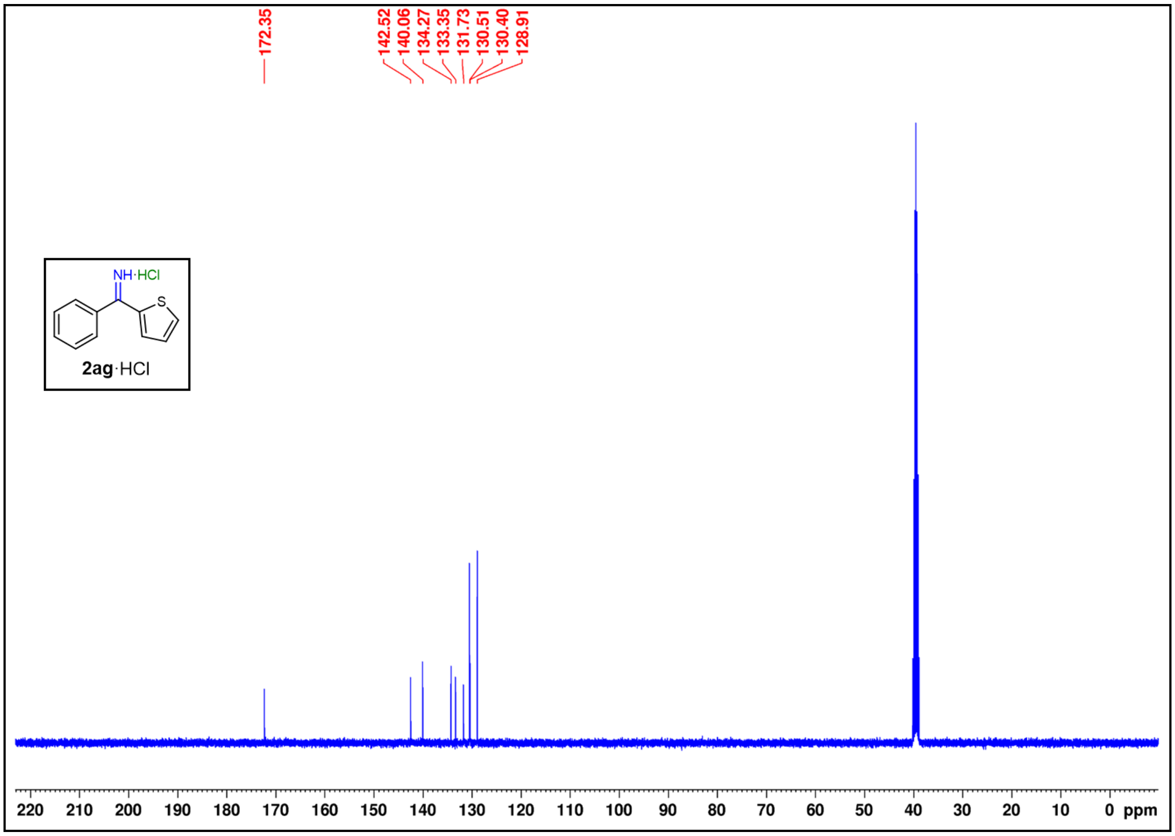

FT-IR (ATR, neat) and HRMS (ESI-positive) spectra for **2ag**·HCl

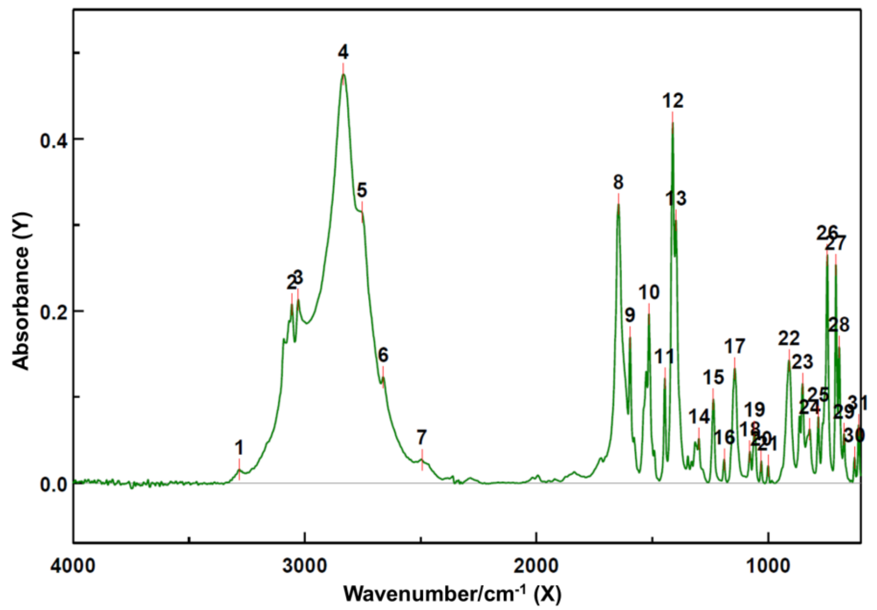

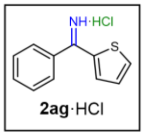

No.	X	Y	No.	X	Y	No.	X	Y
1	3282.3	0.015	2	3055.7	0.208	3	3029	0.213
4	2832.9	0.475	5	2753.9	0.315	6	2661	0.123
7	2494.5	0.027	8	1645.9	0.324	9	1596	0.169
10	1513.8	0.196	11	1446.4	0.121	12	1412	0.419
13	1398.1	0.305	14	1298.8	0.051	15	1236	0.097
16	1189.9	0.027	17	1143.6	0.133	18	1079	0.036
19	1058.7	0.06	20	1029.8	0.025	21	999.9	0.02
22	909.3	0.142	23	851.4	0.115	24	820.6	0.062
25	783.9	0.076	26	744.4	0.265	27	707.7	0.253
28	693.3	0.158	29	672.1	0.057	30	627.7	0.029
31	609.4	0.067						

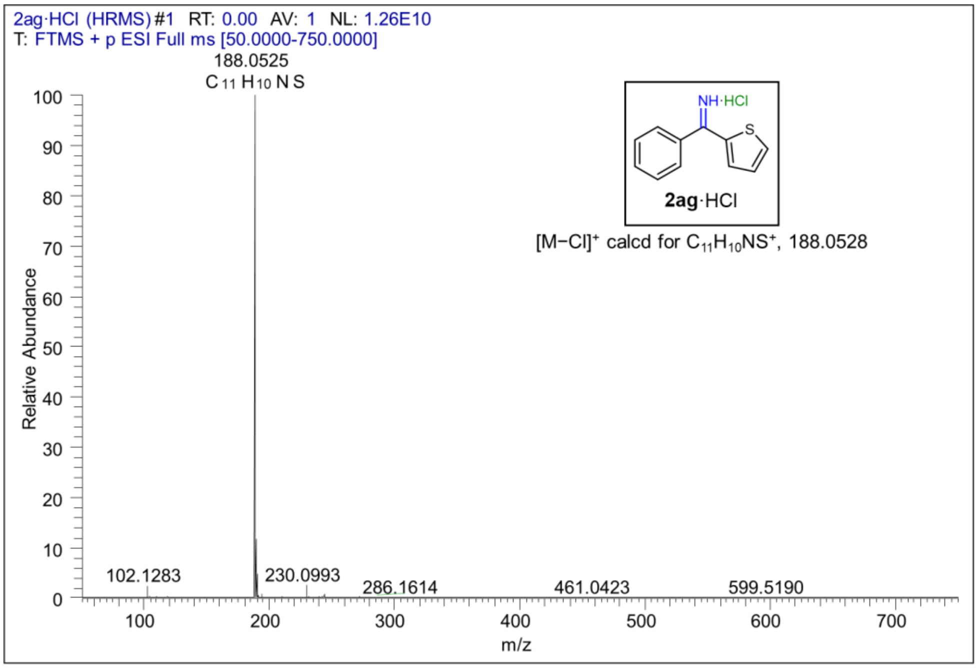

^1H NMR (400 MHz, DMSO-d_6) and ^{13}C NMR (100 MHz, DMSO-d_6) spectra for **2ah**

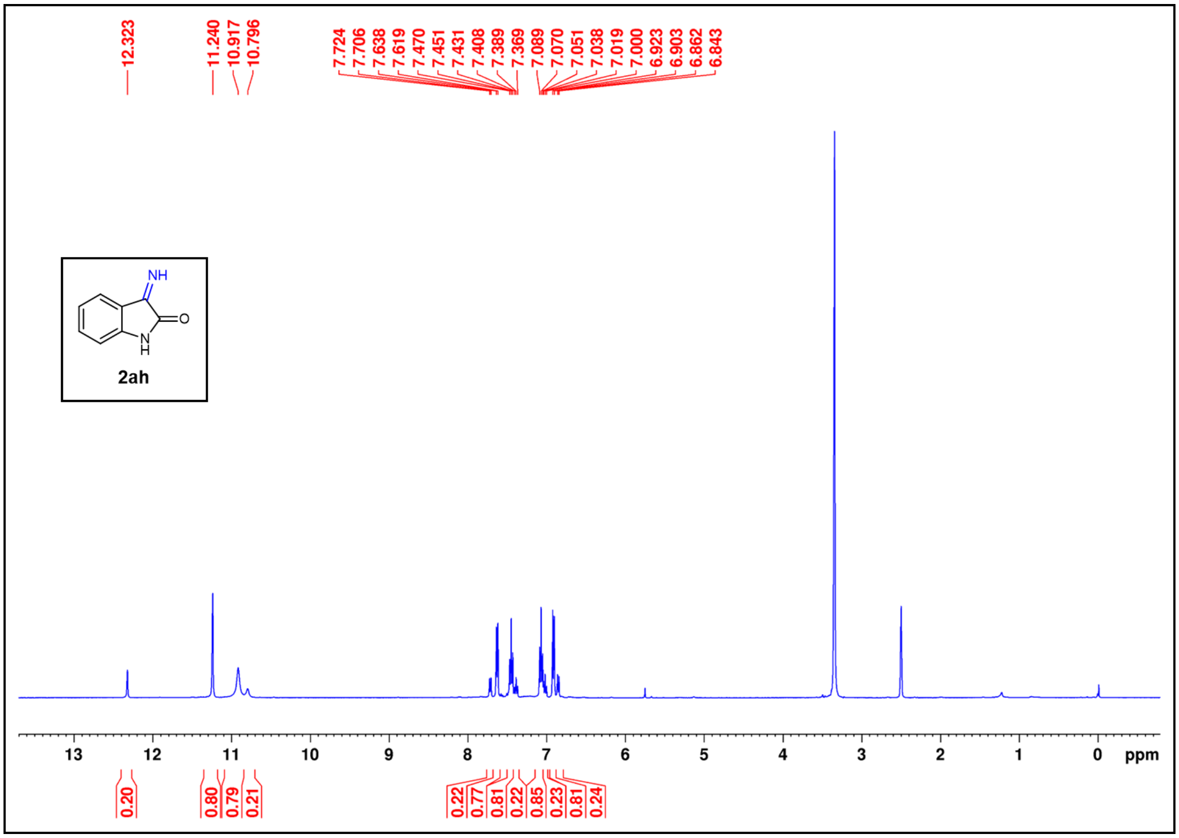

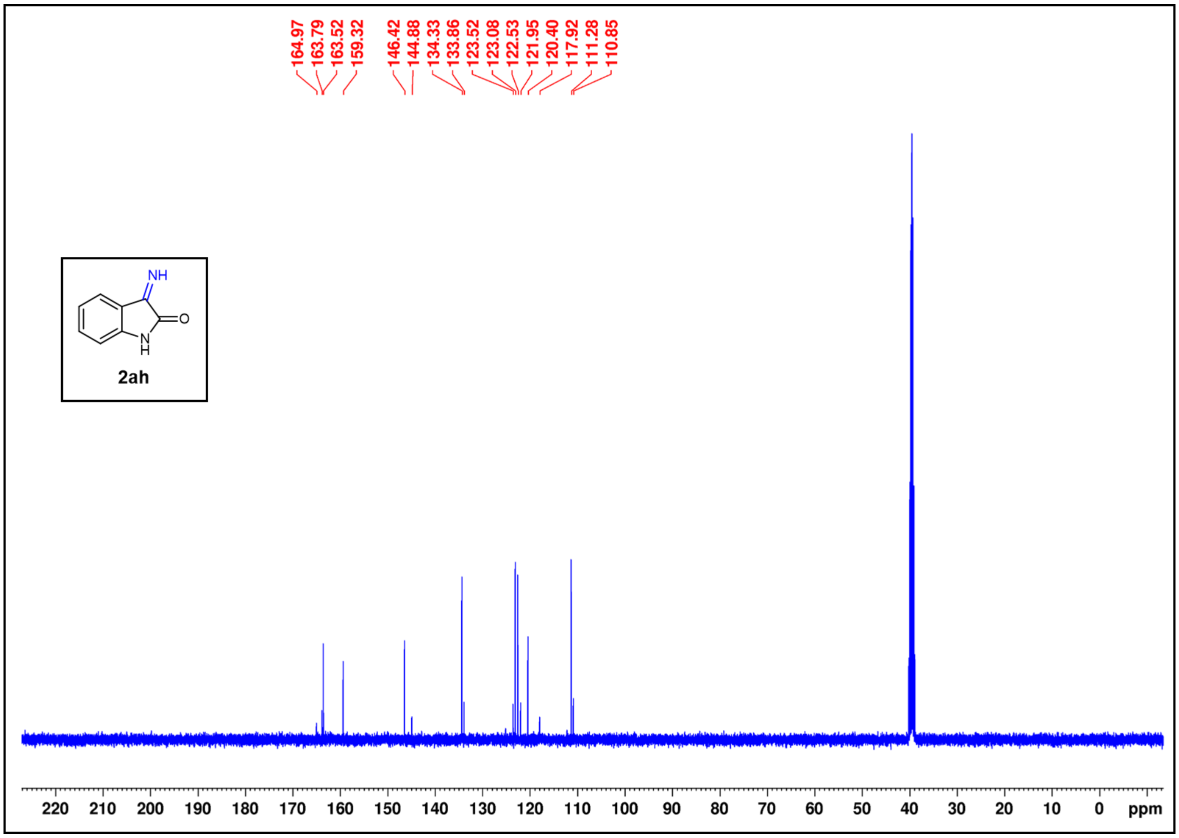

FT-IR (ATR, neat) and HRMS (ESI-positive) spectra for **2ah**

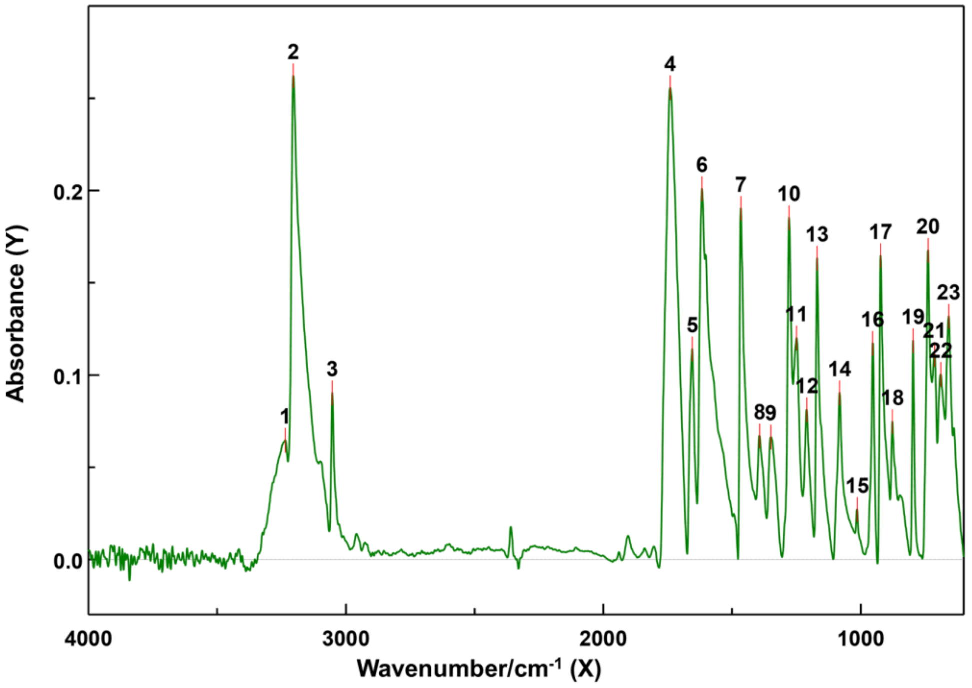

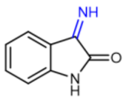

2ah

No.	X	Y	No.	X	Y	No.	X	Y
1	3235	0.064	9	1349	0.066	17	922.8	0.165
2	3204.1	0.262	10	1278.6	0.185	18	877.5	0.075
3	3052.8	0.09	11	1249.6	0.12	19	796.5	0.118
4	1740.4	0.256	12	1210.1	0.081	20	738.6	0.168
5	1654.6	0.114	13	1169.6	0.163	21	713.5	0.111
6	1617	0.201	14	1081.9	0.09	22	689.4	0.1
7	1465.6	0.19	15	1014.4	0.027	23	658.6	0.132
8	1393.3	0.067	16	953.6	0.117			

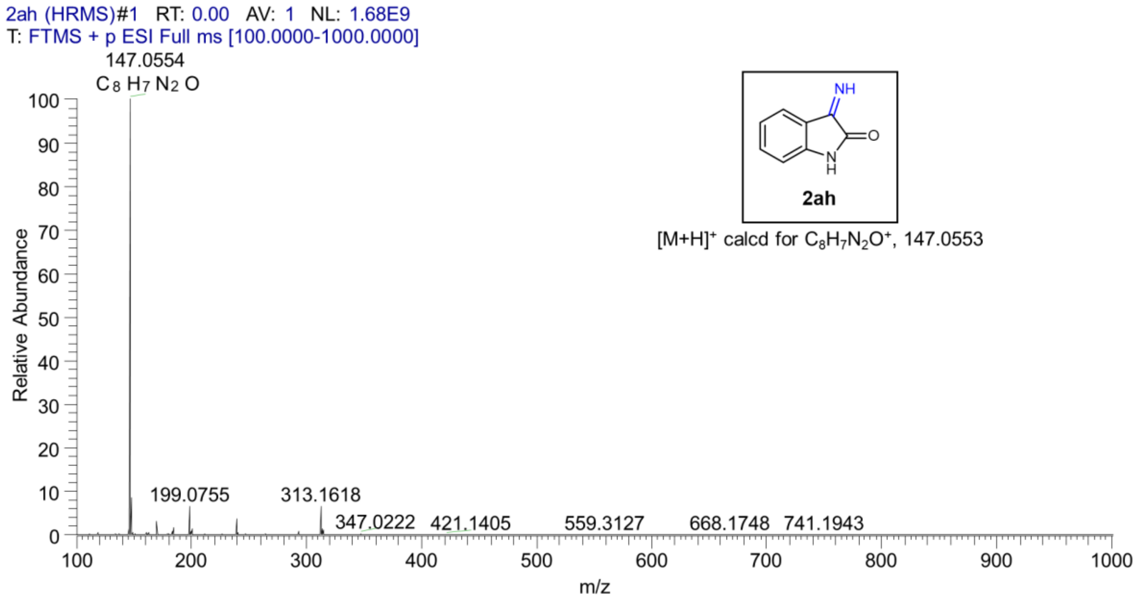

^{1}H NMR (400 MHz, $CDCl_3$) and ^{13}C NMR (100 MHz, $CDCl_3$) spectra for **2ai**

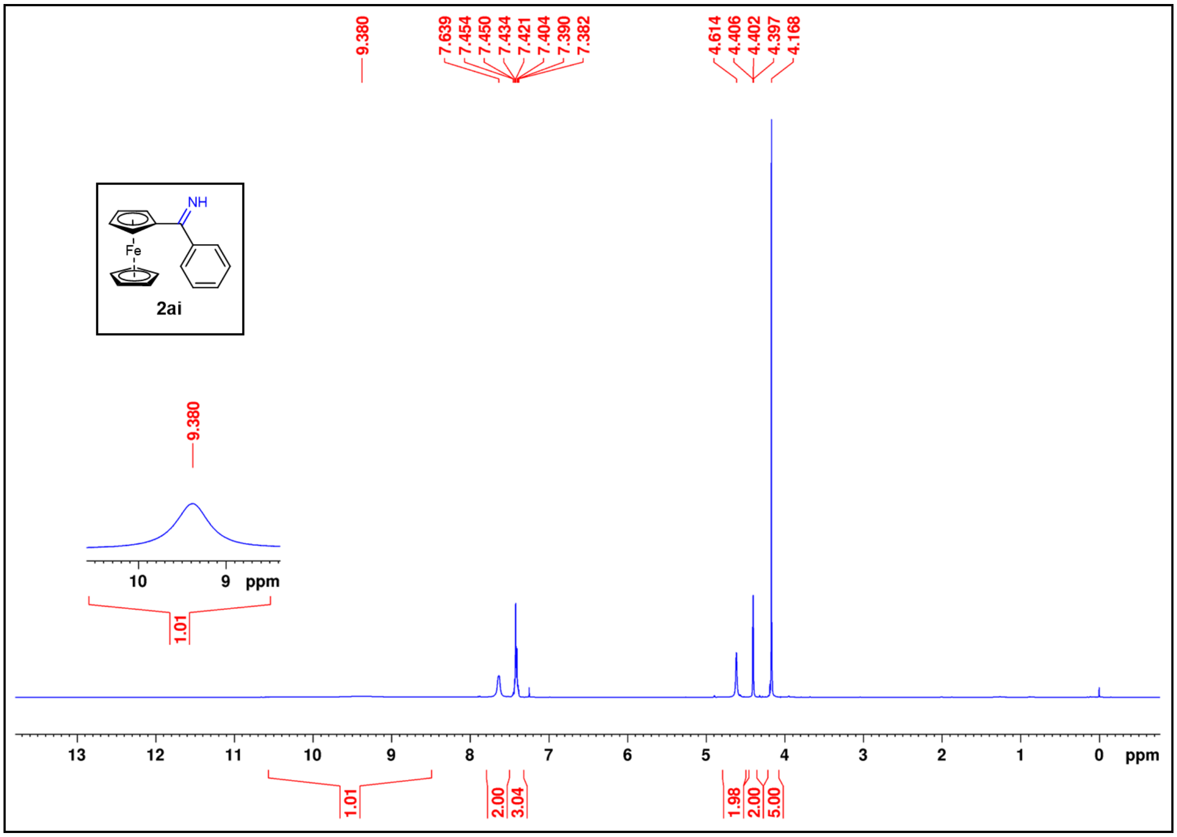

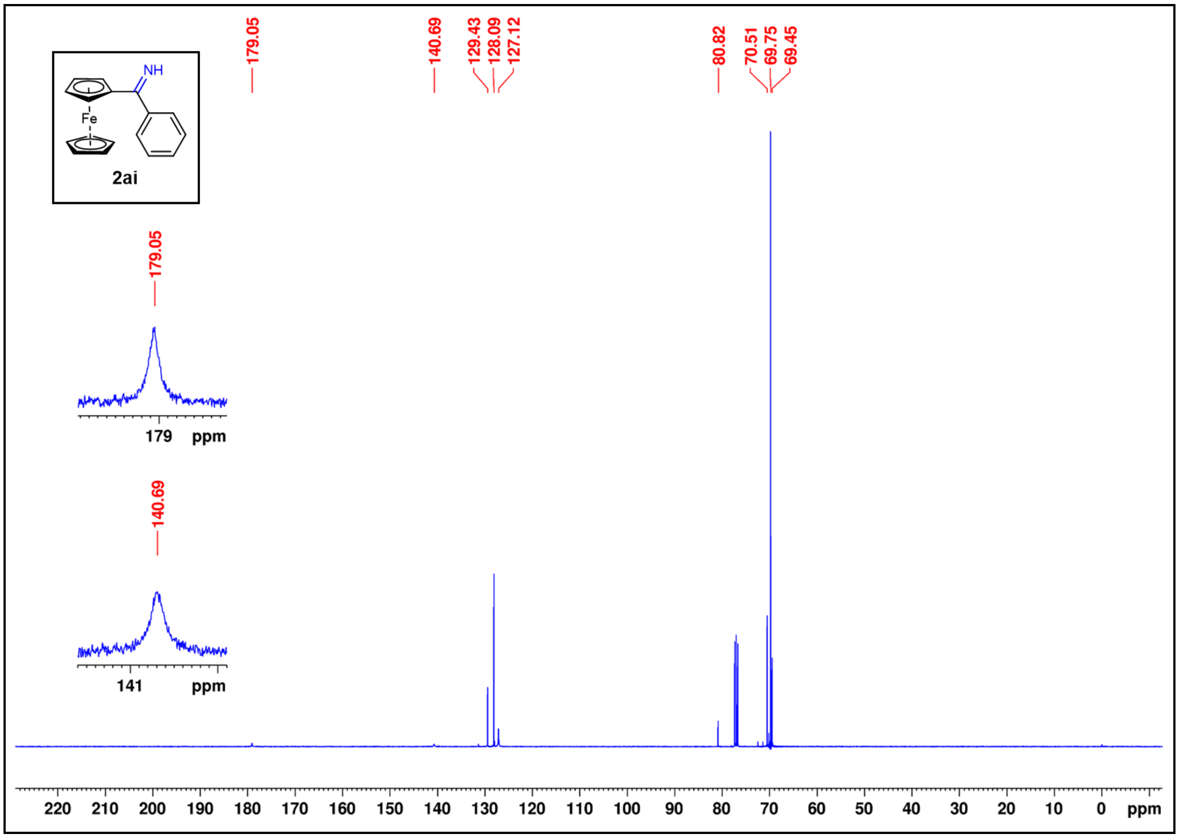

FT-IR (ATR, neat) and HRMS (ESI-positive) spectra for **2ai**

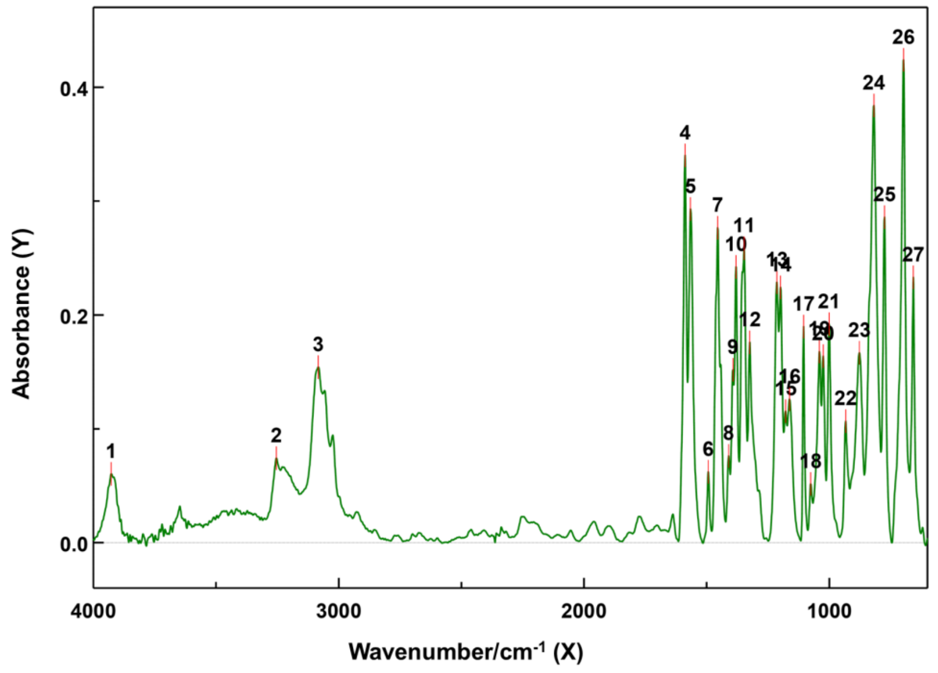

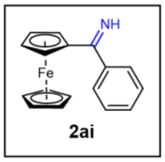

No.	X	Y	No.	X	Y	No.	X	Y
1	3927.3	0.06	10	1380.8	0.242	19	1040	0.168
2	3254.3	0.074	11	1347	0.258	20	1025	0.164
3	3083.6	0.154	12	1324.9	0.176	21	1001	0.192
4	1588.1	0.34	13	1214	0.229	22	933.4	0.107
5	1565.9	0.293	14	1198.5	0.224	23	877.5	0.167
6	1493.6	0.062	15	1178.3	0.115	24	818.6	0.384
7	1455	0.276	16	1161.9	0.126	25	775.2	0.286
8	1410.7	0.076	17	1105	0.19	26	697.1	0.424
9	1393.3	0.152	18	1076.1	0.051	27	657.6	0.233

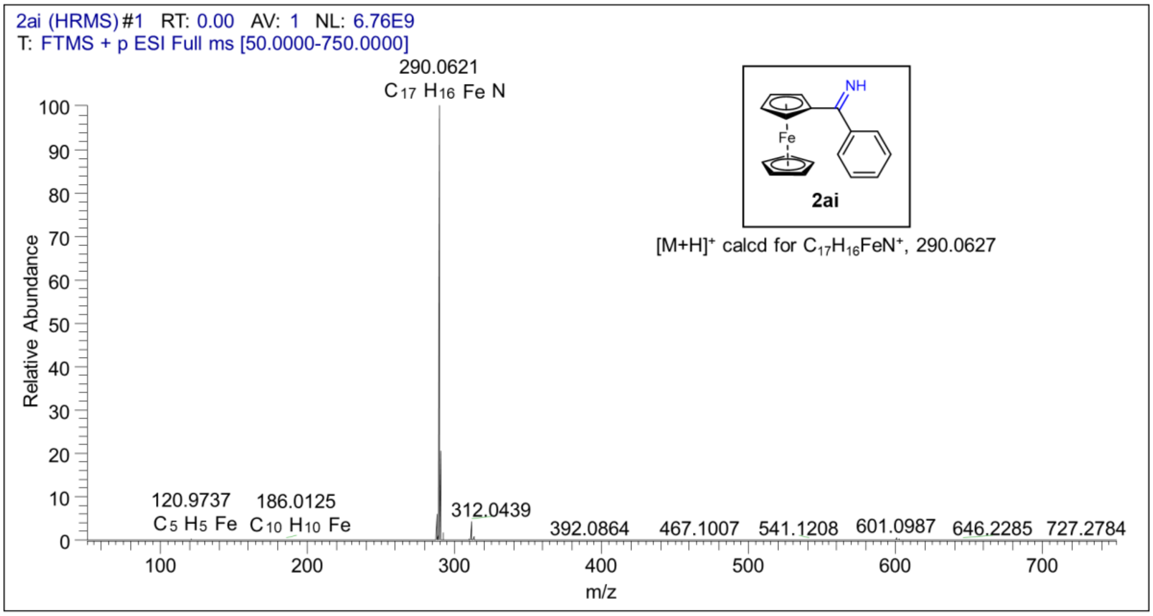

^{1}H NMR (400 MHz, $CDCl_3$) and ^{13}C NMR (100 MHz, $CDCl_3$) spectra for **2aj**

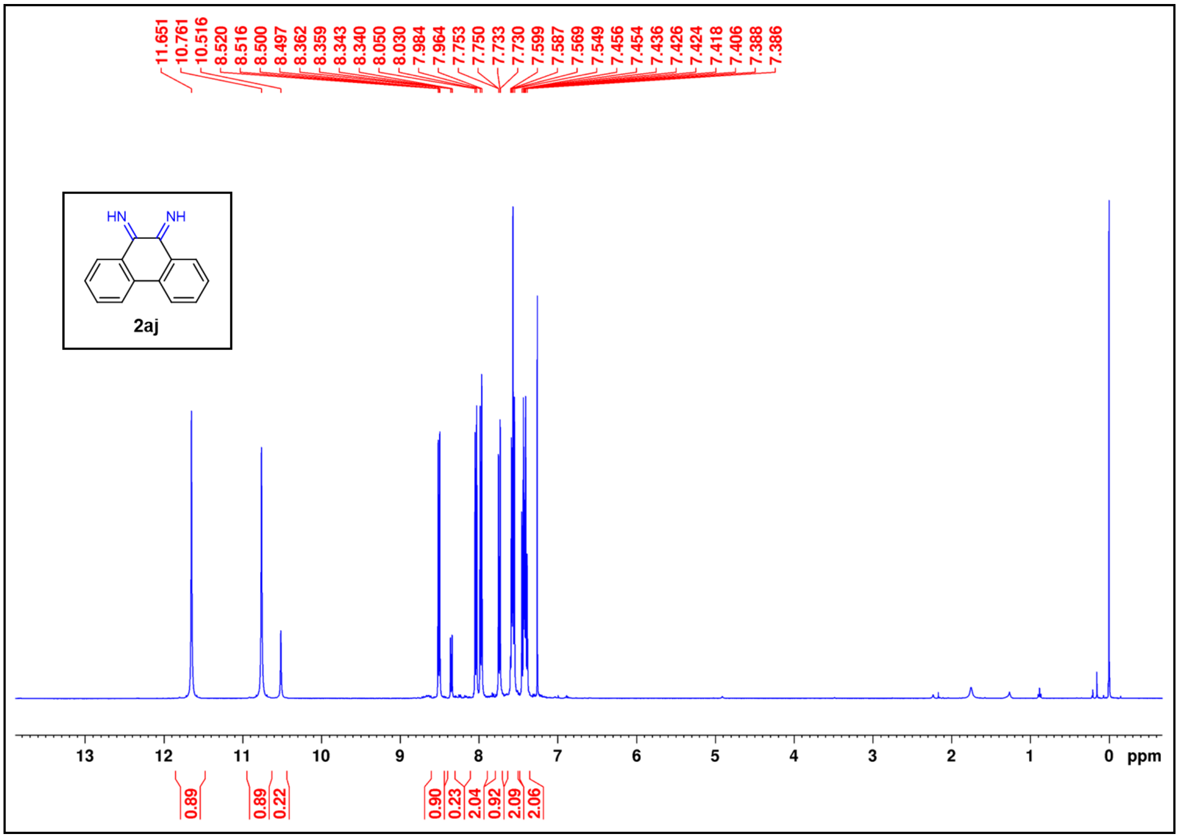

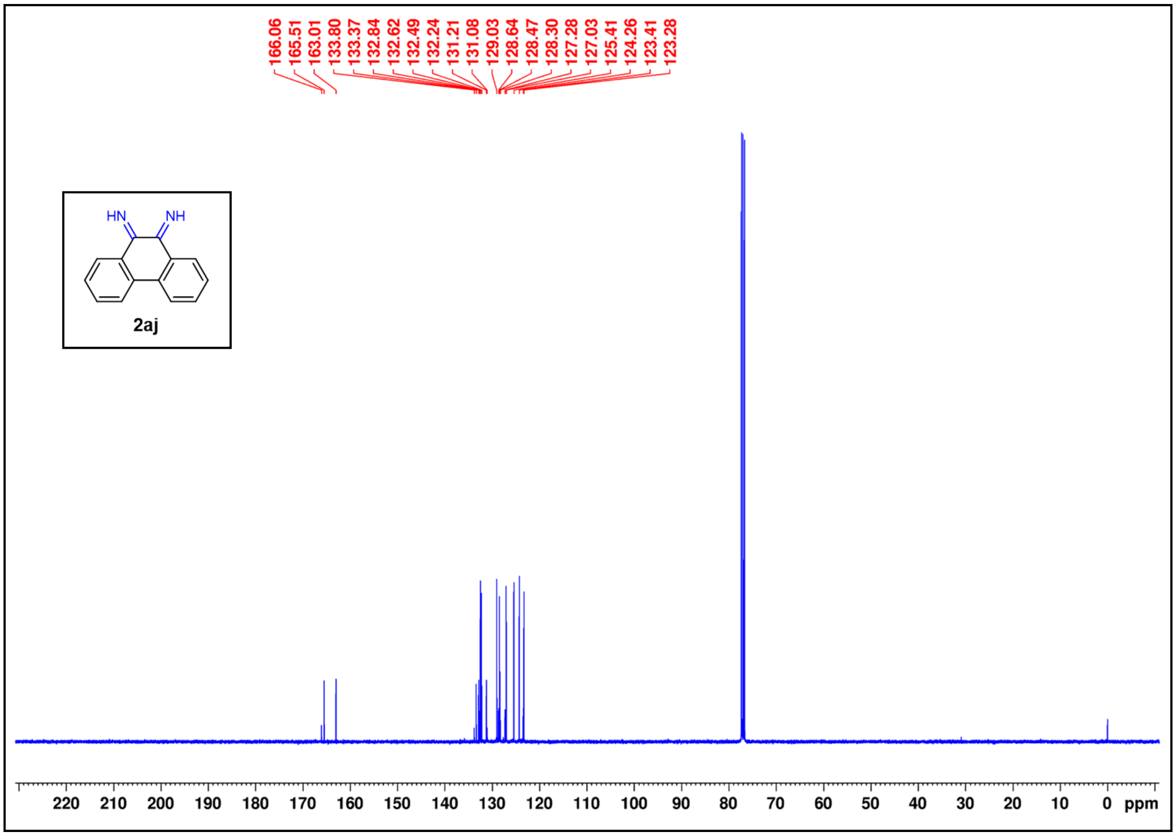

FT-IR (ATR, neat) and HRMS (ESI-positive) spectra for **2aj**

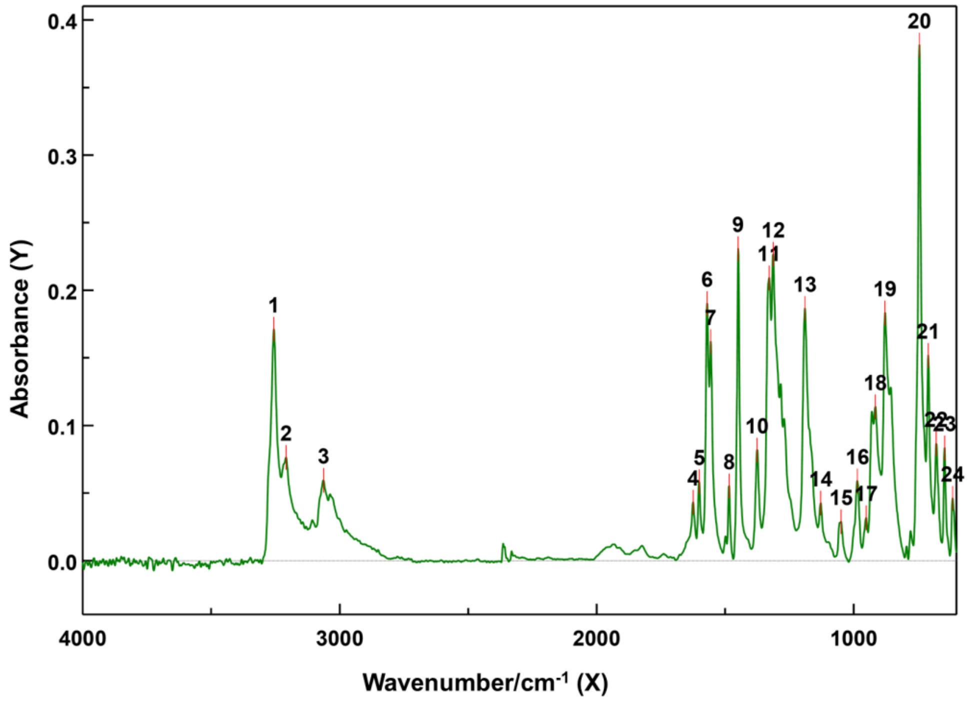

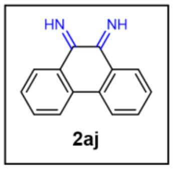

No.	X	Y	No.	X	Y	No.	X	Y
1	3256.2	0.171	9	1449.2	0.231	17	951.7	0.031
2	3209	0.076	10	1376	0.082	18	916	0.114
3	3063.4	0.059	11	1328.7	0.209	19	878.4	0.183
4	1624.7	0.043	12	1313.3	0.226	20	744.4	0.381
5	1601.6	0.058	13	1189.9	0.186	21	709.7	0.152
6	1570.7	0.19	14	1128.2	0.042	22	678.8	0.086
7	1556.3	0.162	15	1049.1	0.029	23	646	0.083
8	1484.9	0.055	16	985.4	0.059	24	615.2	0.046

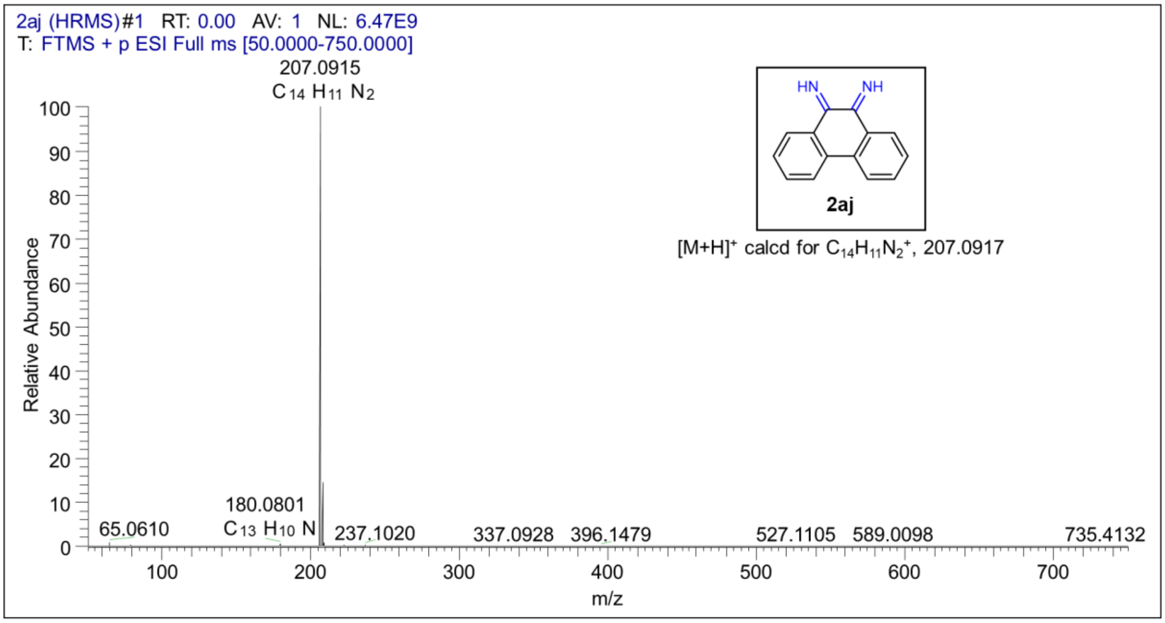

^{1}H NMR (400 MHz, $CDCl_3$) and ^{13}C NMR (100 MHz, $CDCl_3$) spectra for **2ak**

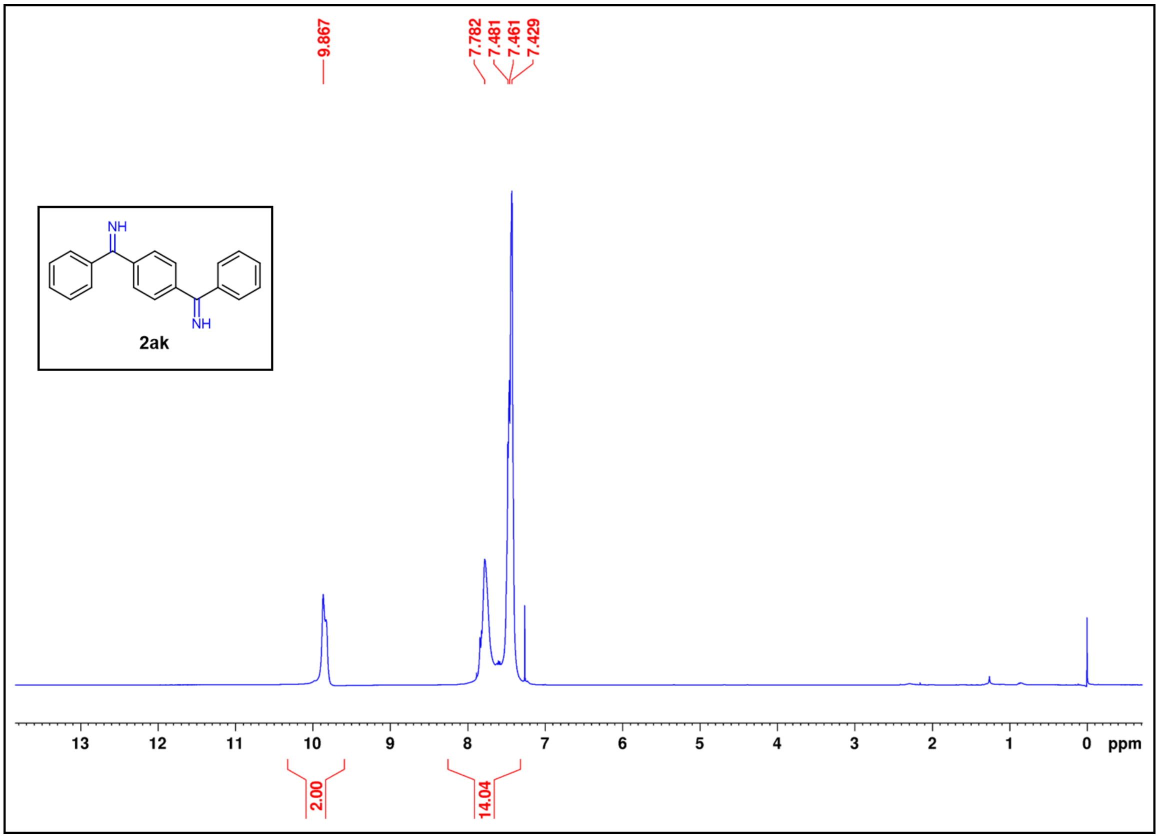

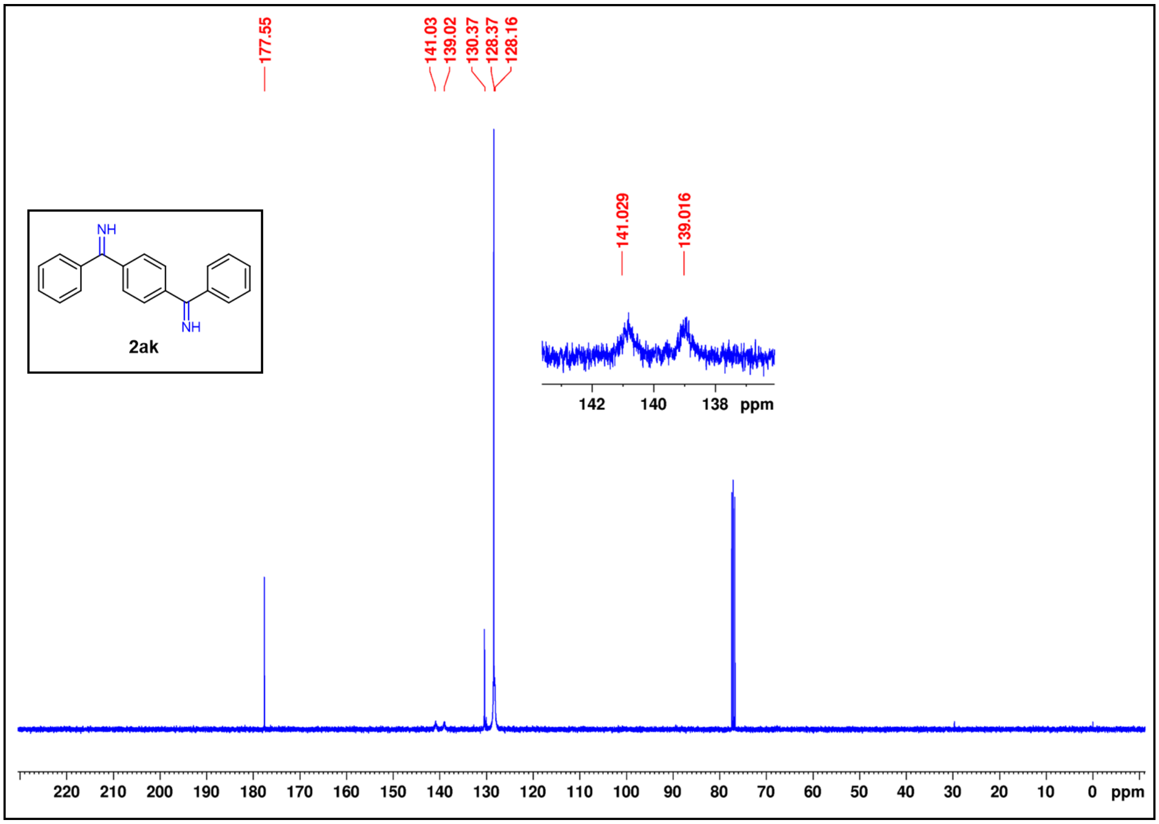

FT-IR (ATR, neat) and HRMS (ESI-positive) spectra for **2ak**

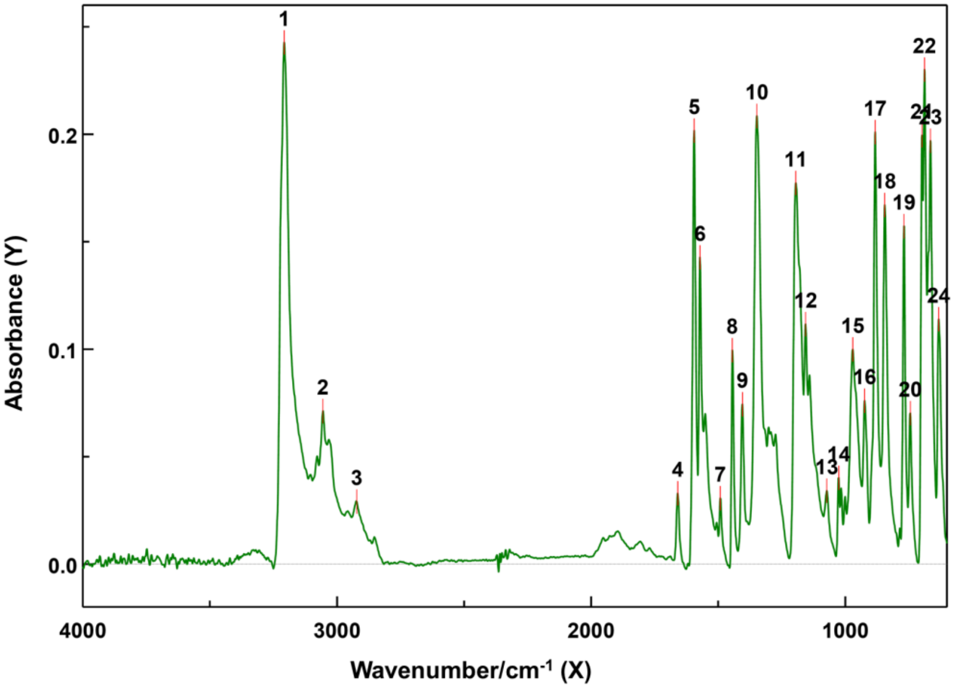

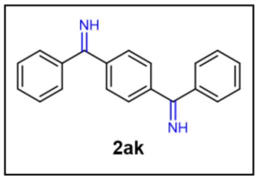

No.	X	Y	No.	X	Y	No.	X	Y
1	3208	0.243	9	1404.9	0.074	17	882.3	0.201
2	3055.7	0.071	10	1348	0.208	18	844.7	0.167
3	2922.6	0.029	11	1194.7	0.177	19	769.5	0.157
4	1659.4	0.033	12	1156.1	0.112	20	744.4	0.07
5	1594.8	0.202	13	1073.2	0.034	21	698.1	0.199
6	1571.7	0.143	14	1025.9	0.04	22	688.5	0.23
7	1491.7	0.031	15	971	0.1	23	665.3	0.197
8	1444.4	0.099	16	923.7	0.076	24	632.5	0.114

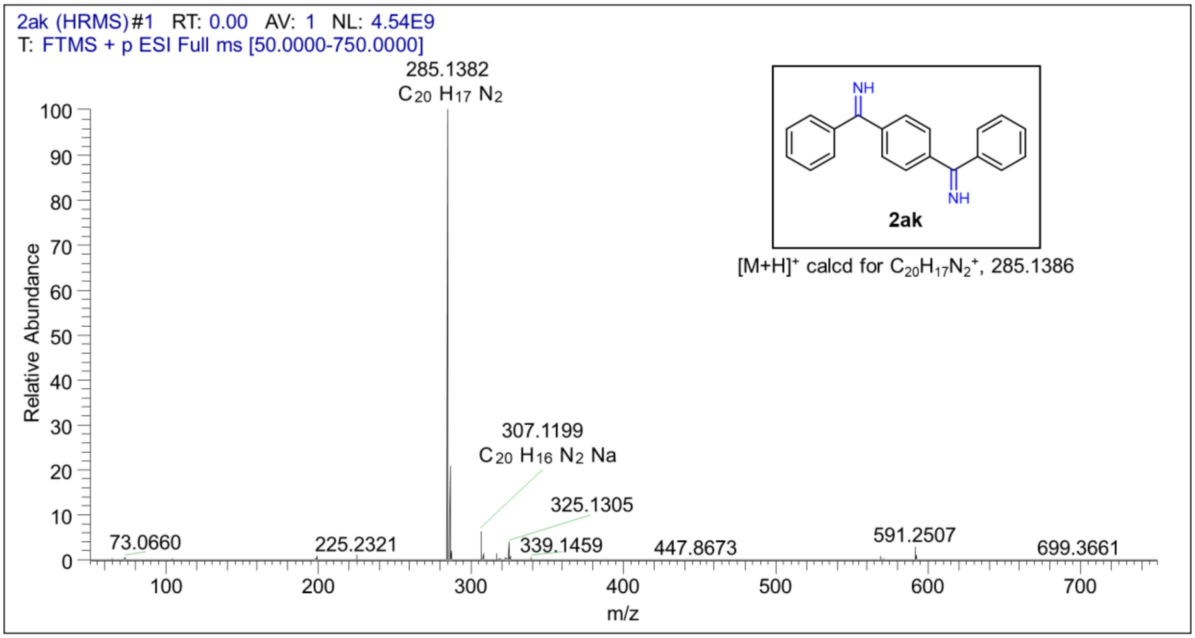

^{1}H NMR (400 MHz, DMSO-d_6) and ^{13}C NMR (100 MHz, DMSO-d_6) spectra for **2al**·HCl

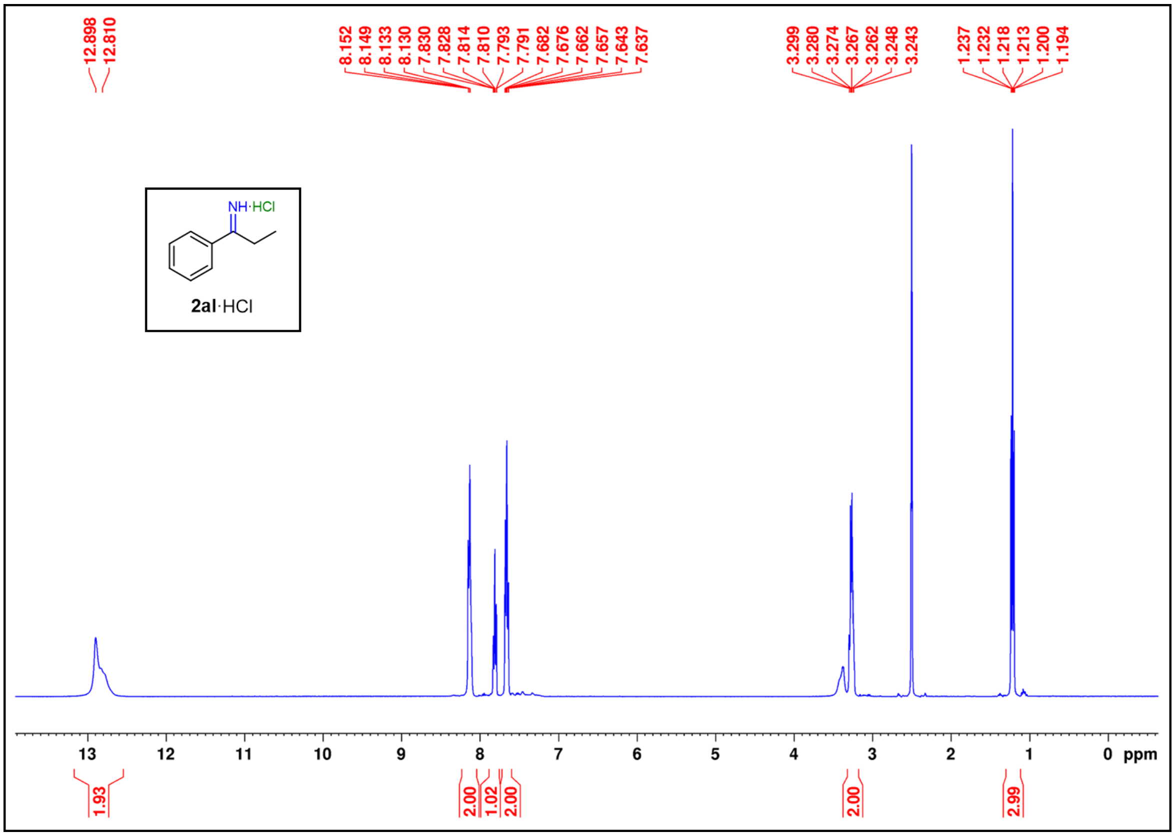

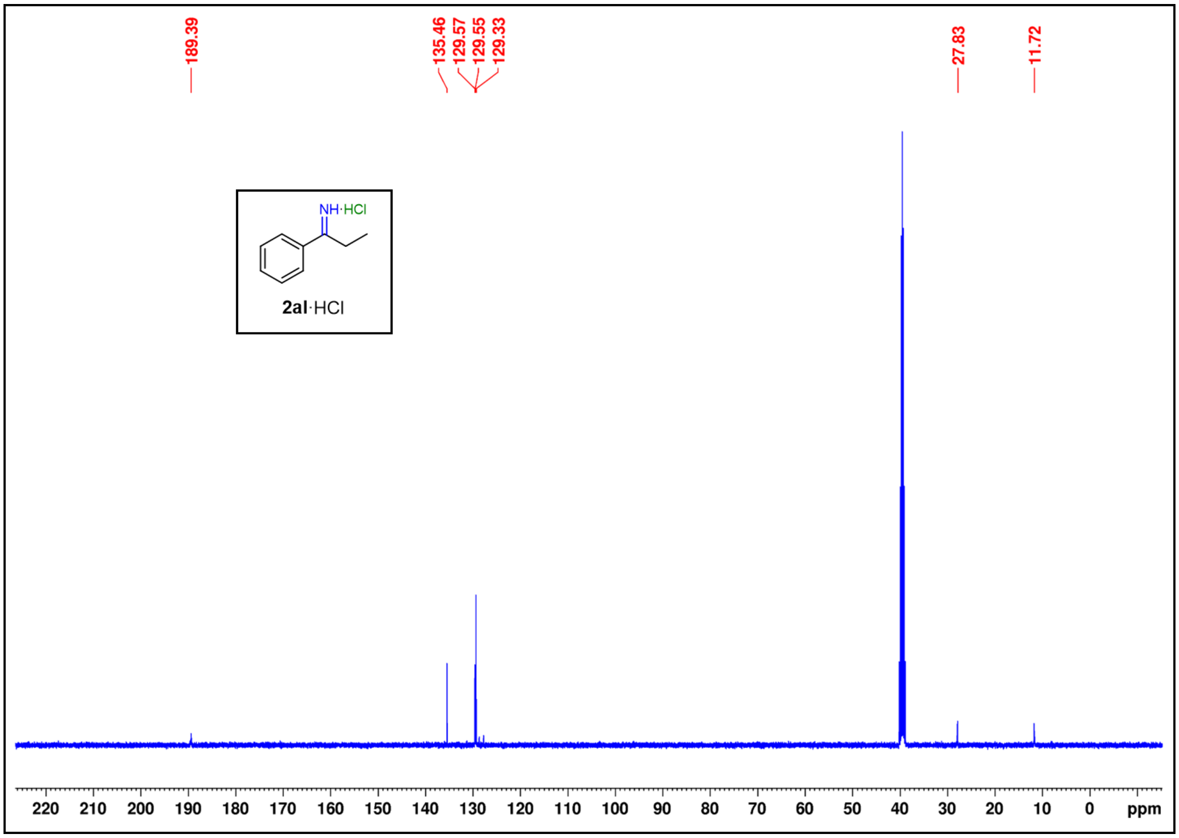

FT-IR (ATR, neat) and HRMS (ESI-positive) spectra for **2al**·HCl

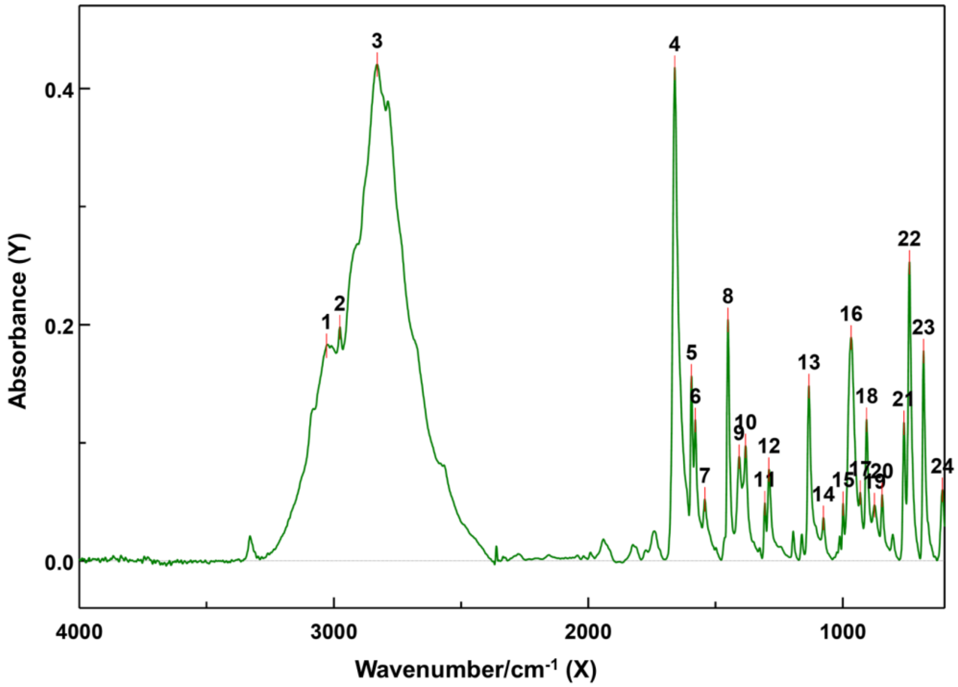

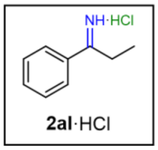

No.	X	Y	No.	X	Y	No.	X	Y
1	3028.7	0.182	9	1406.8	0.088	17	931.5	0.058
2	2976.6	0.198	10	1381.7	0.097	18	906.4	0.119
3	2830	0.42	11	1306.5	0.049	19	875.5	0.047
4	1660.4	0.417	12	1290.1	0.077	20	845.6	0.056
5	1594.8	0.156	13	1133	0.148	21	759.8	0.117
6	1579.4	0.119	14	1076.1	0.036	22	738.6	0.253
7	1541.8	0.052	15	998.9	0.048	23	682.7	0.177
8	1451.2	0.204	16	967.1	0.189	24	608.4	0.06

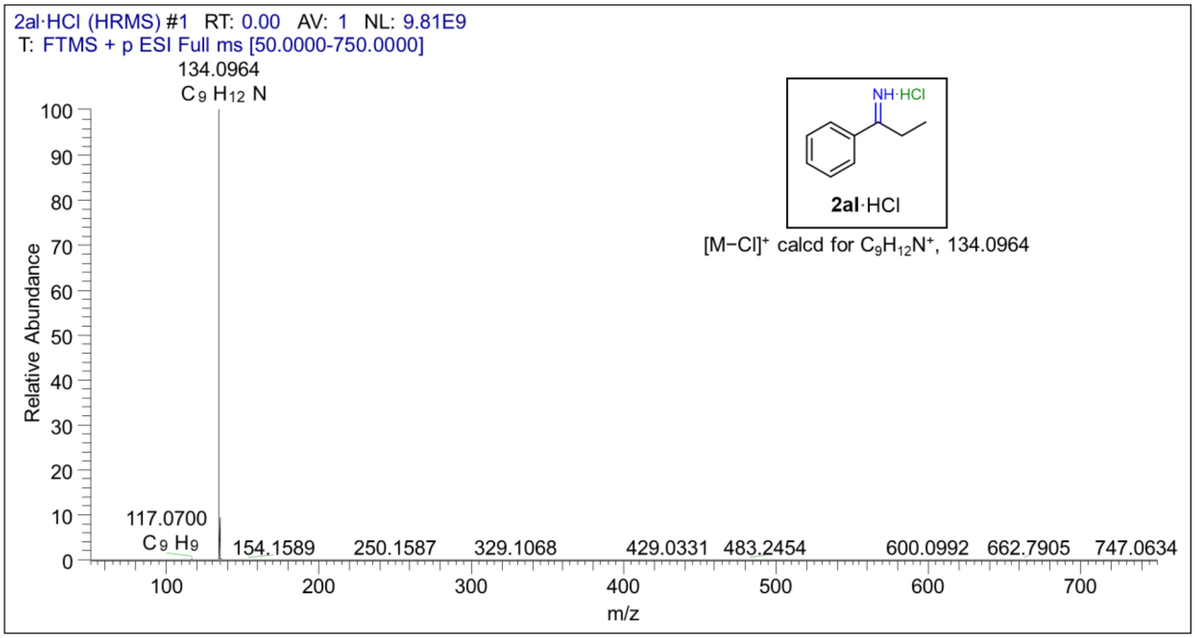

^{1}H NMR (400 MHz, DMSO-d_6) and ^{13}C NMR (100 MHz, DMSO-d_6) spectra for **2am**·HCl

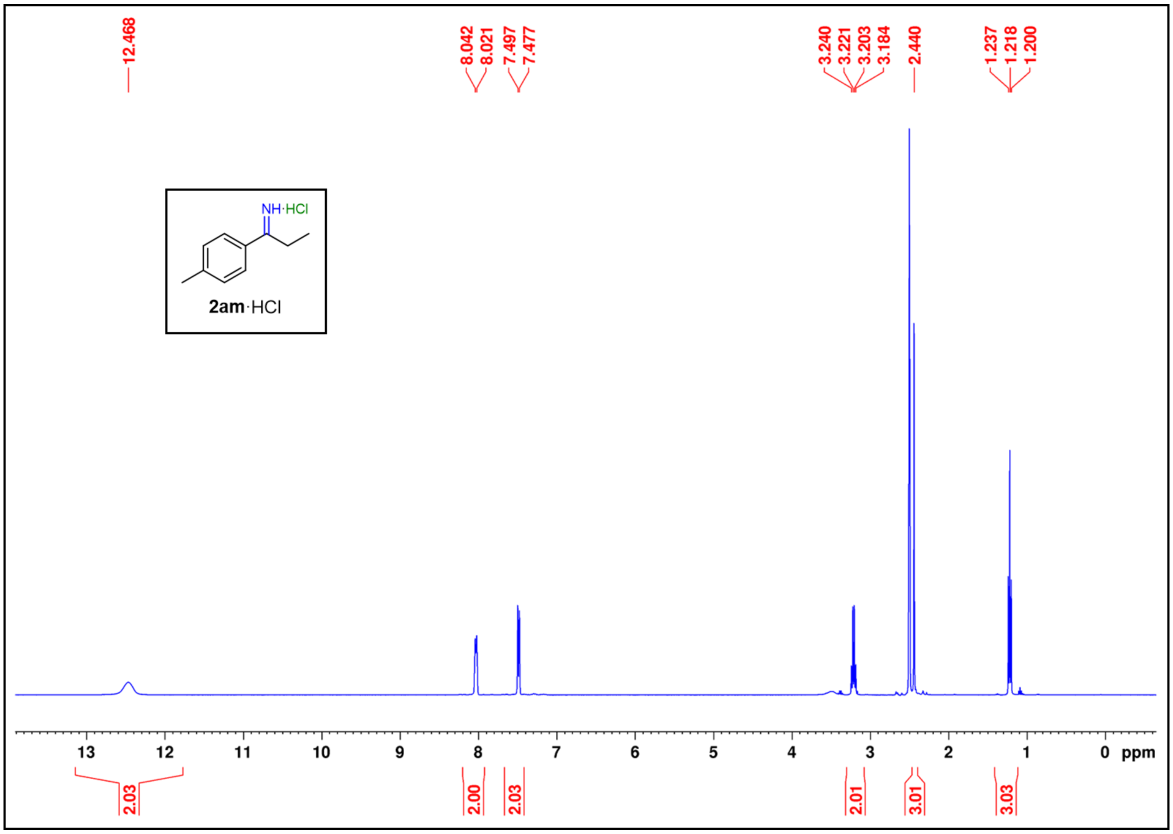

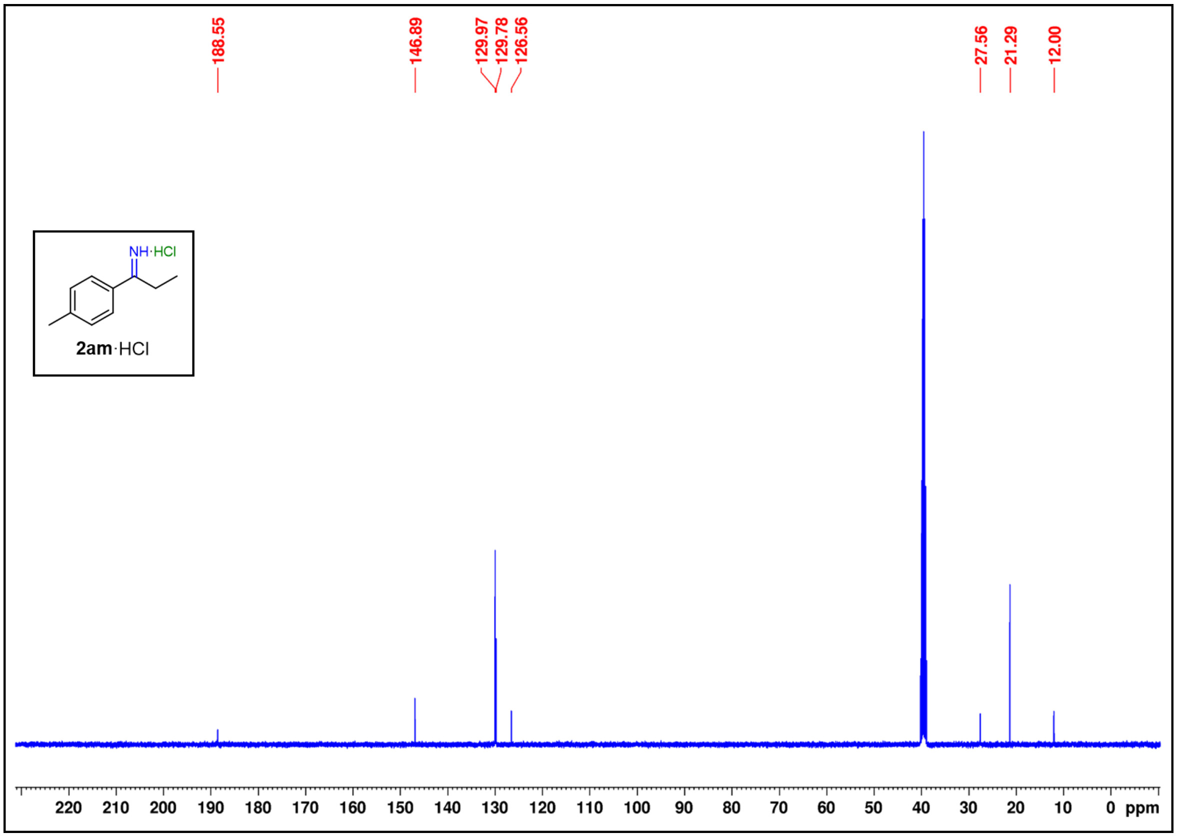

FT-IR (ATR, neat) and HRMS (ESI-positive) spectra for **2am**·HCl

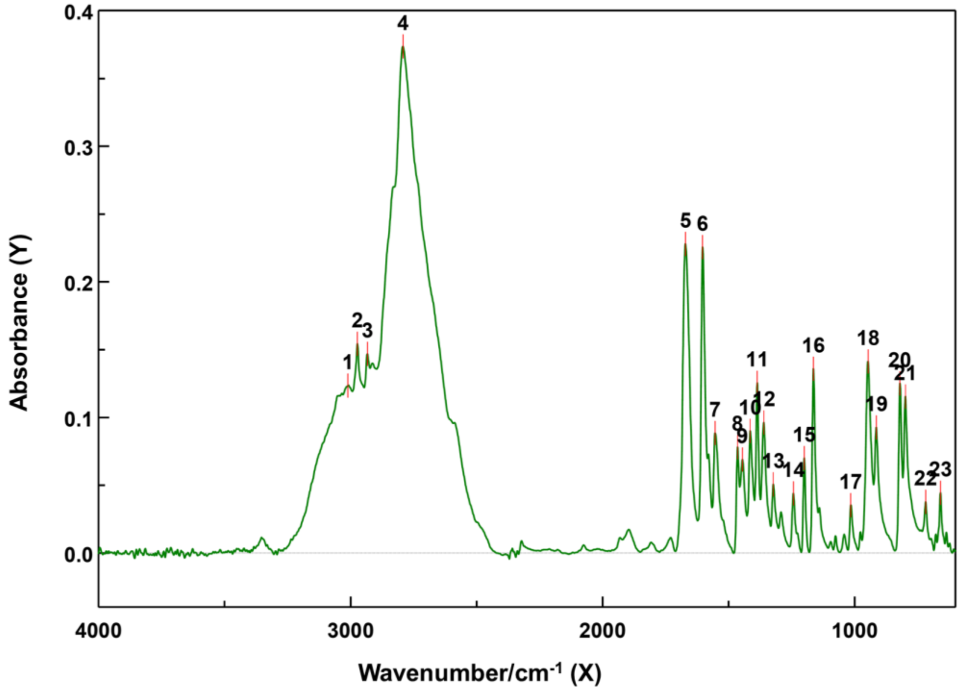

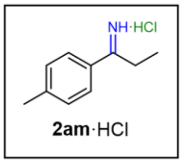

No.	X	Y	No.	X	Y	No.	X	Y
1	3010.3	0.123	9	1445.4	0.069	17	1015	0.035
2	2972.7	0.154	10	1414.5	0.09	18	946.9	0.141
3	2933.2	0.147	11	1386.6	0.125	19	914.1	0.093
4	2792.4	0.374	12	1360.5	0.096	20	820.6	0.125
5	1671	0.228	13	1322.9	0.05	21	798.4	0.115
6	1603.5	0.226	14	1242.9	0.044	22	718.4	0.038
7	1553.4	0.088	15	1199.5	0.07	23	659.5	0.044
8	1464.7	0.078	16	1163.8	0.136			

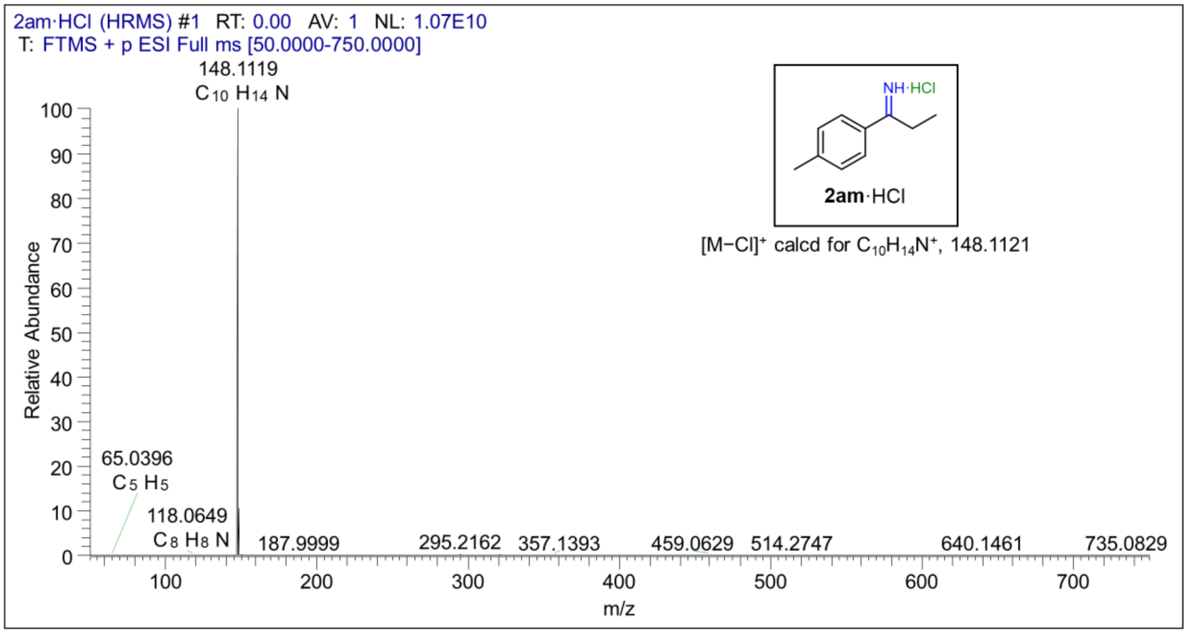

^{1}H NMR (400 MHz, DMSO-d_6) and ^{13}C NMR (100 MHz, DMSO-d_6) spectra for **2an**·HCl

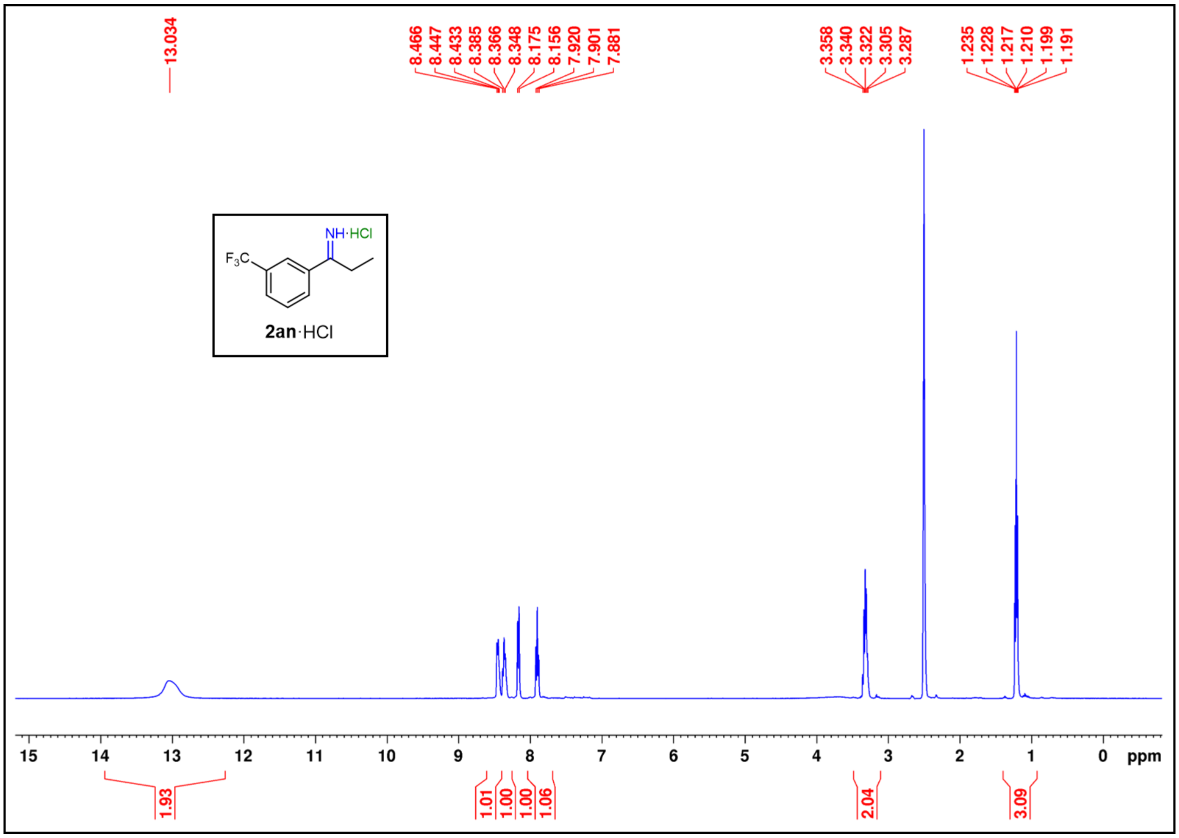

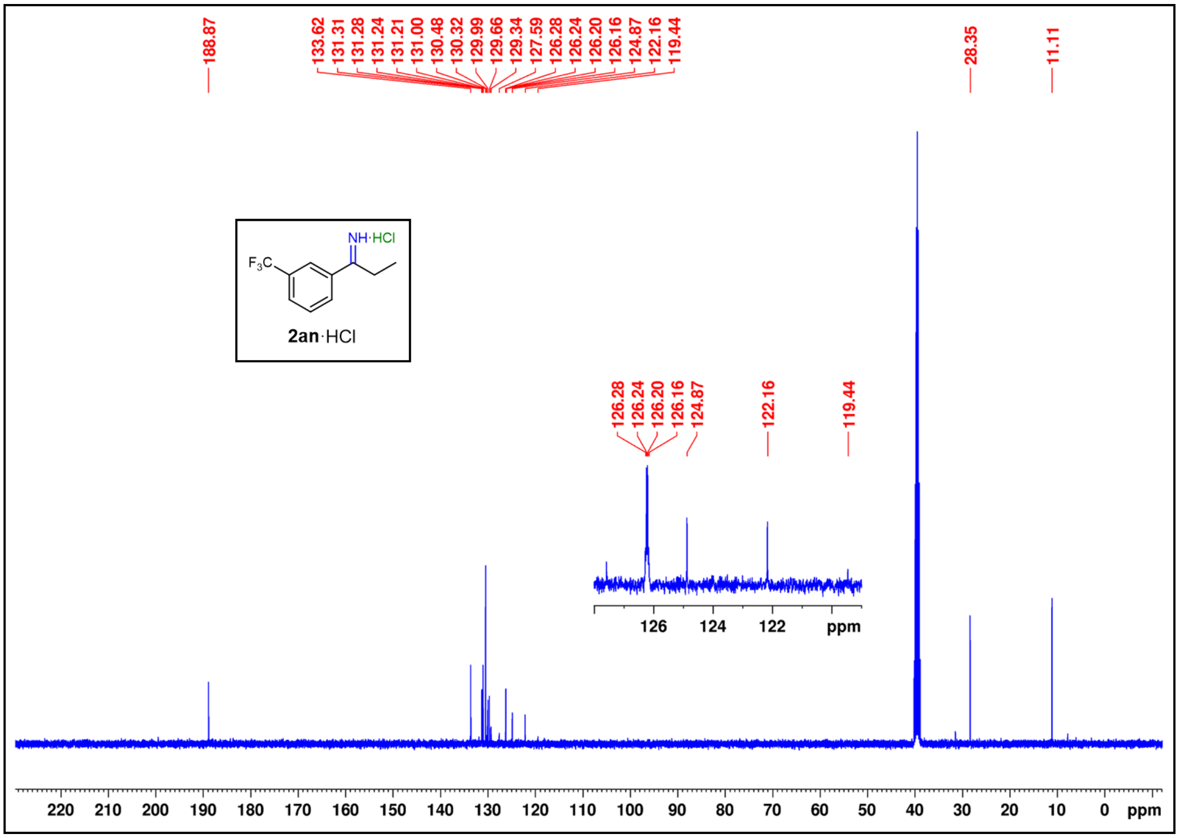

^{19}F NMR (376 MHz, DMSO-d_6) spectrum for **2an**·HCl

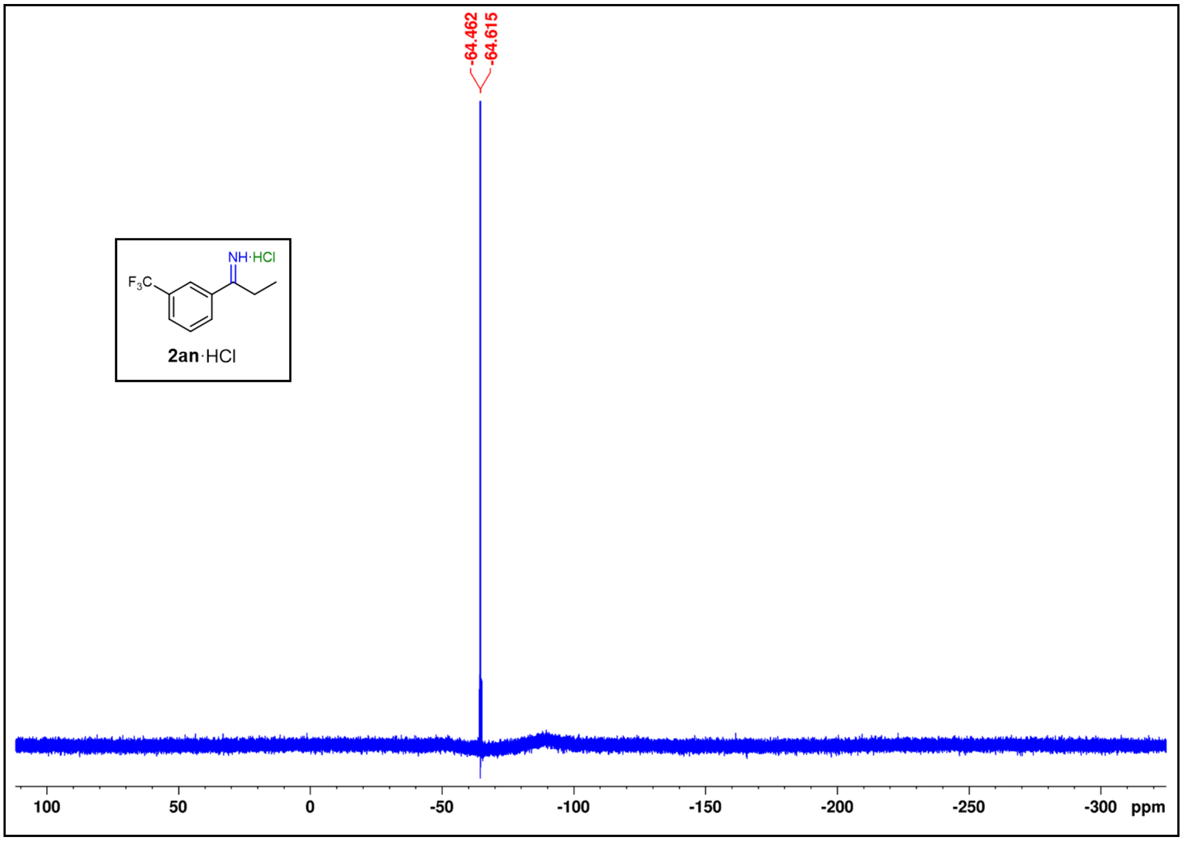

<u>FT-IR (ATR, neat) and HRMS (ESI-positive) spectra for **2an**·HCl</u>

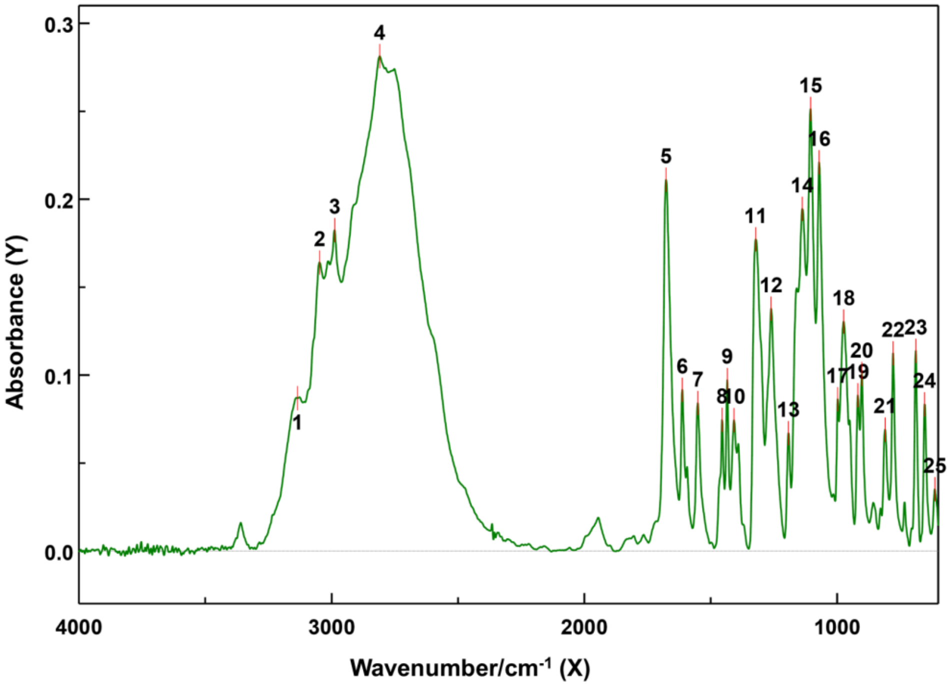

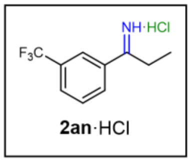

No.	X	Y	No.	X	Y	No.	X	Y
1	3134.7	0.087	10	1407.8	0.074	19	918	0.088
2	3047	0.164	11	1322	0.177	20	902.5	0.101
3	2988.2	0.182	12	1261.2	0.138	21	810	0.069
4	2809.8	0.281	13	1192.8	0.067	22	778.1	0.112
5	1676.8	0.211	14	1137.8	0.194	23	688.5	0.114
6	1613.2	0.092	15	1105	0.251	24	653.8	0.083
7	1551.5	0.084	16	1071.3	0.221	25	613.3	0.035
8	1455	0.074	17	998	0.086			
9	1434.8	0.097	18	973.9	0.13			

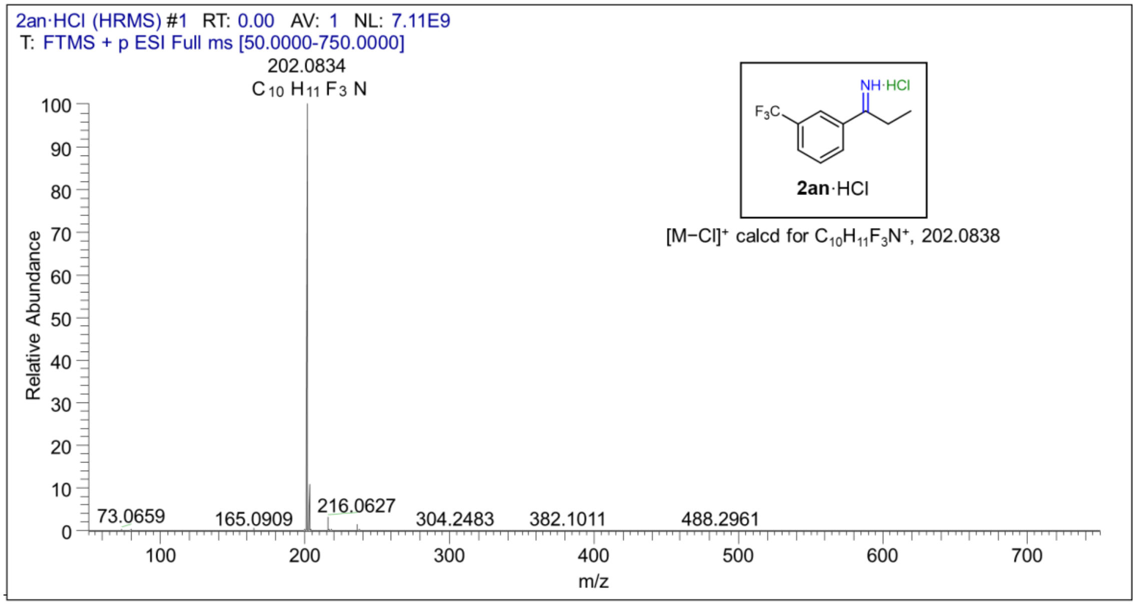

^{1}H NMR (400 MHz, DMSO-d_6) and ^{13}C NMR (100 MHz, DMSO-d_6) spectra for **2ao**·HCl

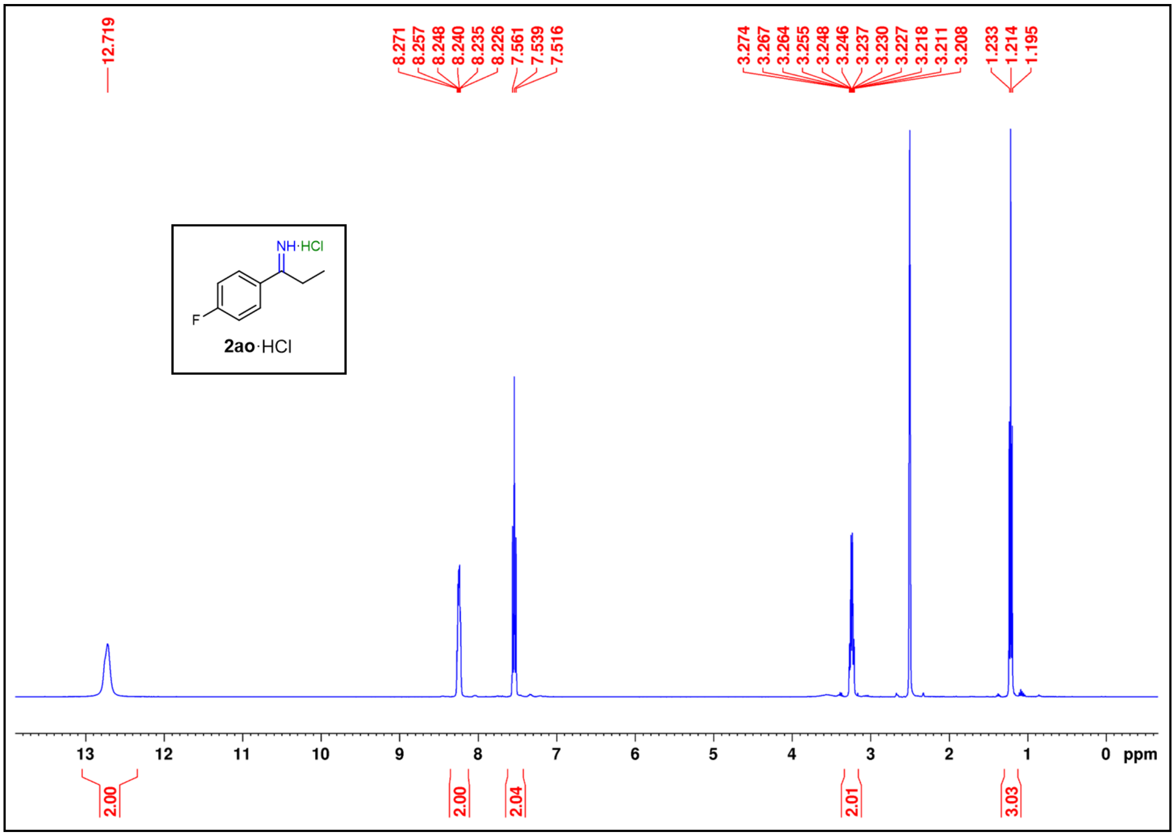

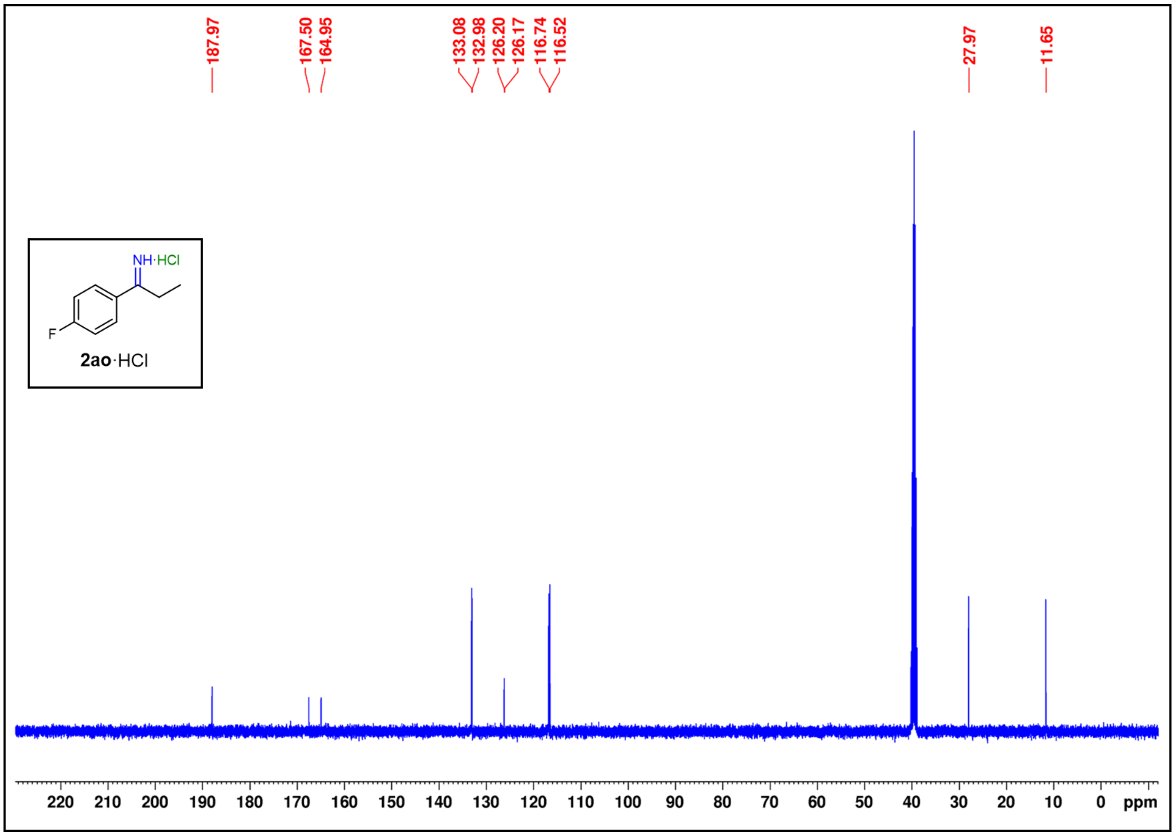

^{19}F NMR (376 MHz, DMSO-d_6) spectrum for **2ao**·HCl

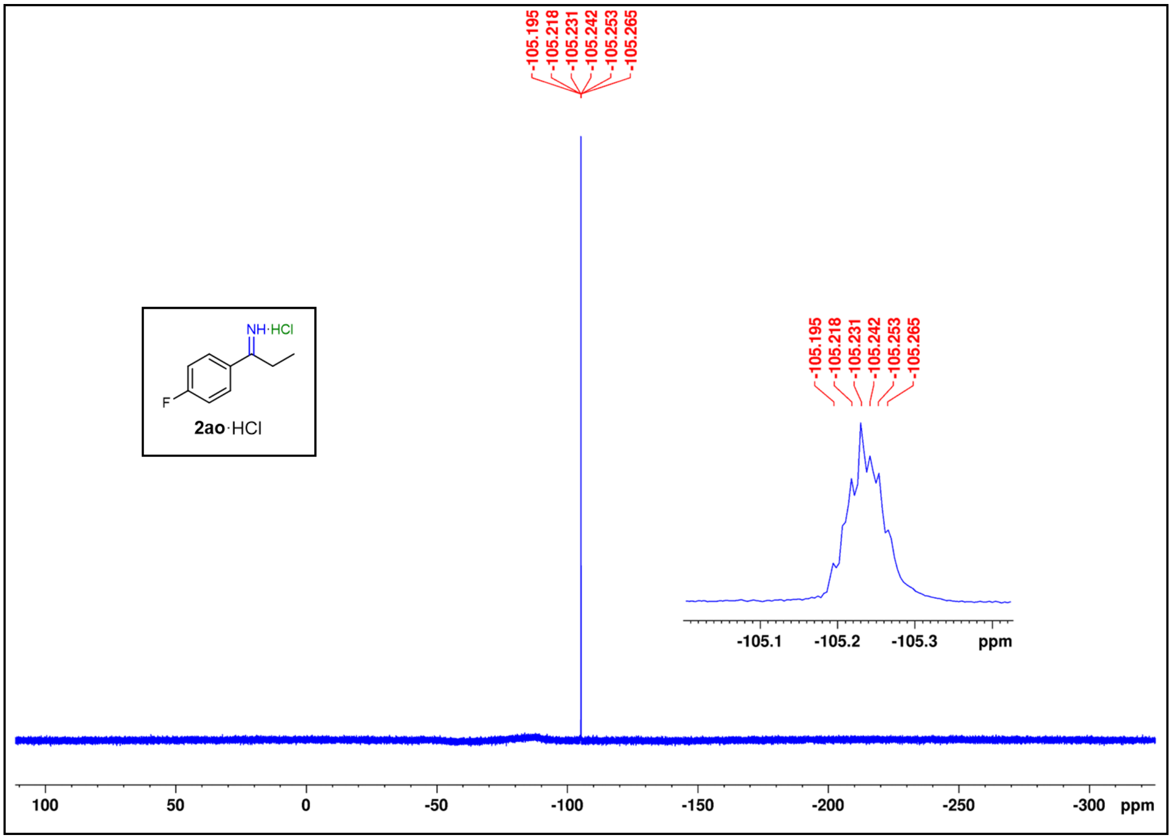

FT-IR (ATR, neat) and HRMS (ESI-positive) spectra for **2ao**·HCl

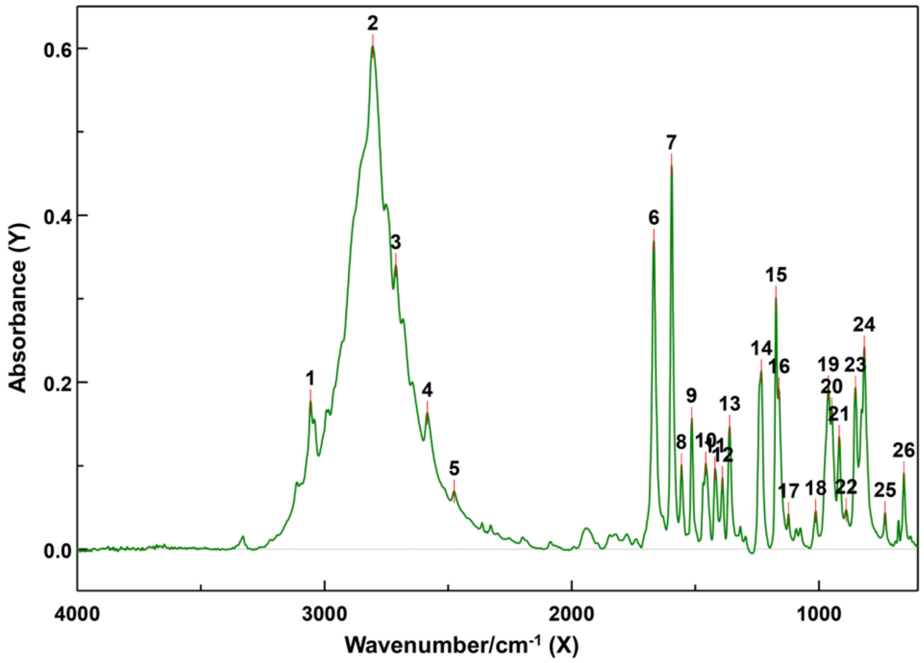

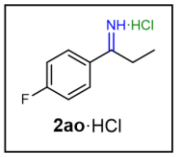

No.	X	Y	No.	X	Y	No.	X	Y
1	3056.6	0.177	10	1457.9	0.103	19	962.3	0.194
2	2805	0.602	11	1420.3	0.096	20	947.8	0.167
3	2711.4	0.34	12	1390.4	0.085	21	918	0.134
4	2584.1	0.163	13	1361.5	0.146	22	890	0.047
5	2476.2	0.069	14	1233.3	0.213	23	852.4	0.193
6	1668.1	0.369	15	1173.5	0.301	24	816.7	0.241
7	1595.8	0.459	16	1161.9	0.191	25	732.8	0.043
8	1556.3	0.101	17	1123.3	0.042	26	656.6	0.091
9	1514.8	0.156	18	1013.4	0.045			

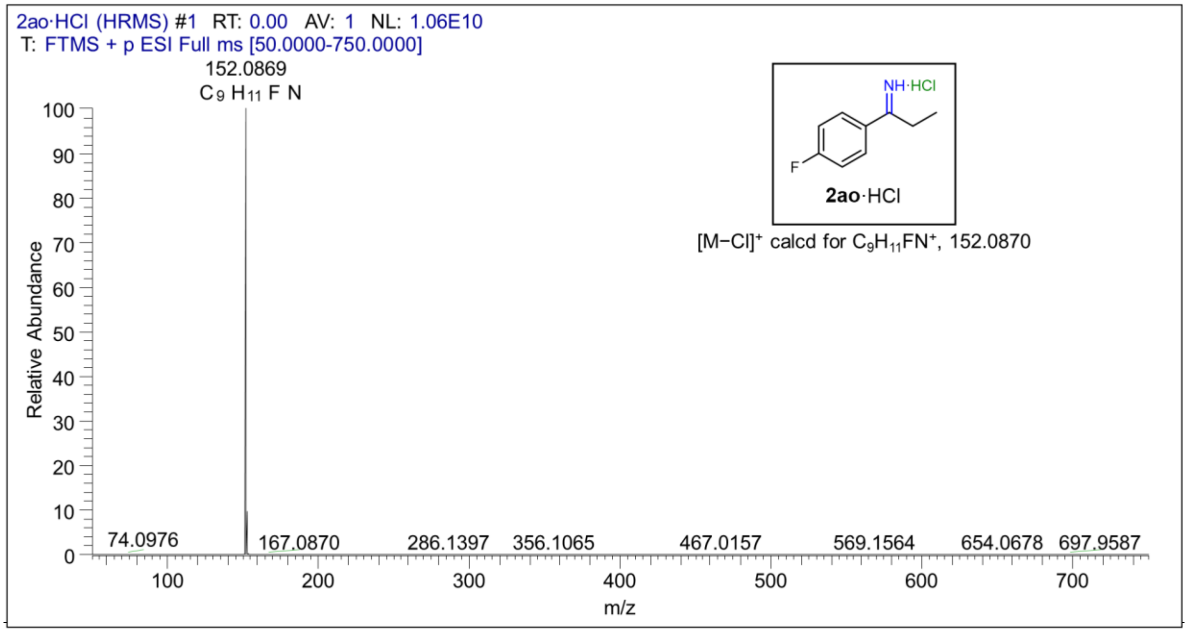

^{1}H NMR (400 MHz, DMSO-d_6) and ^{13}C NMR (100 MHz, DMSO-d_6) spectra for **2ap**·HCl

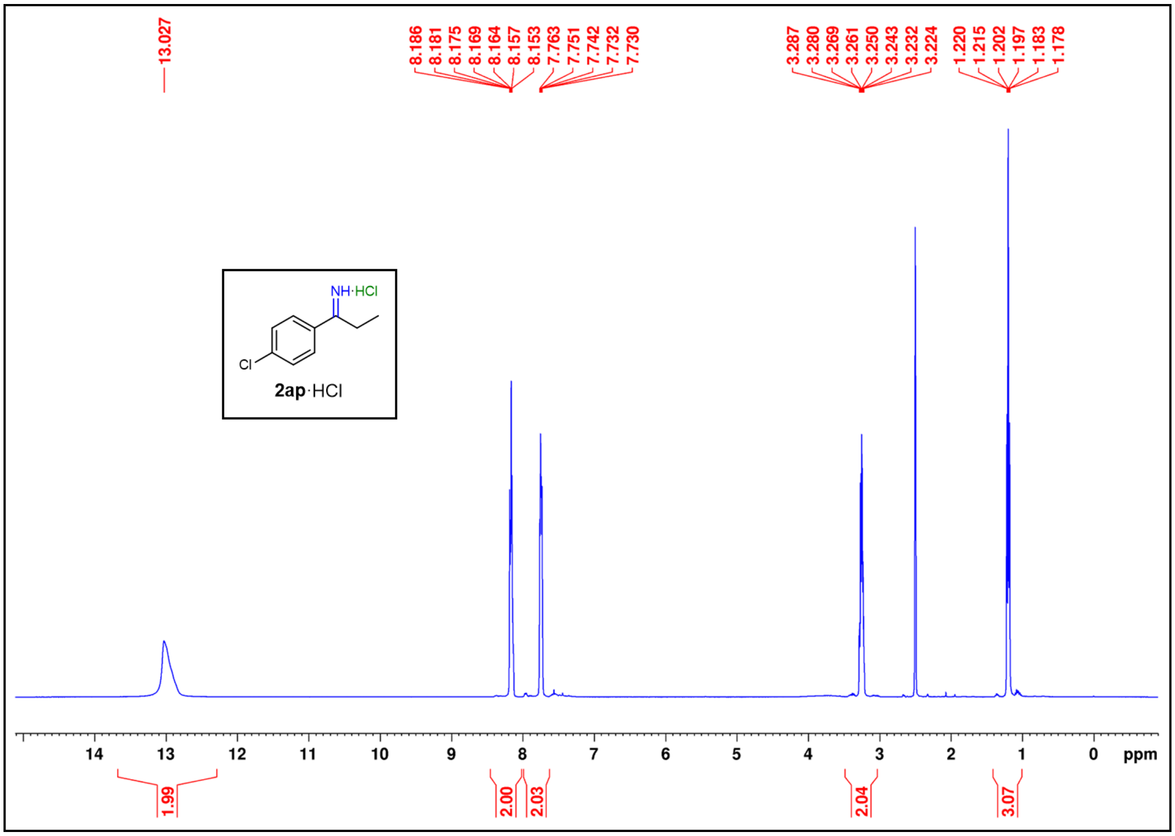

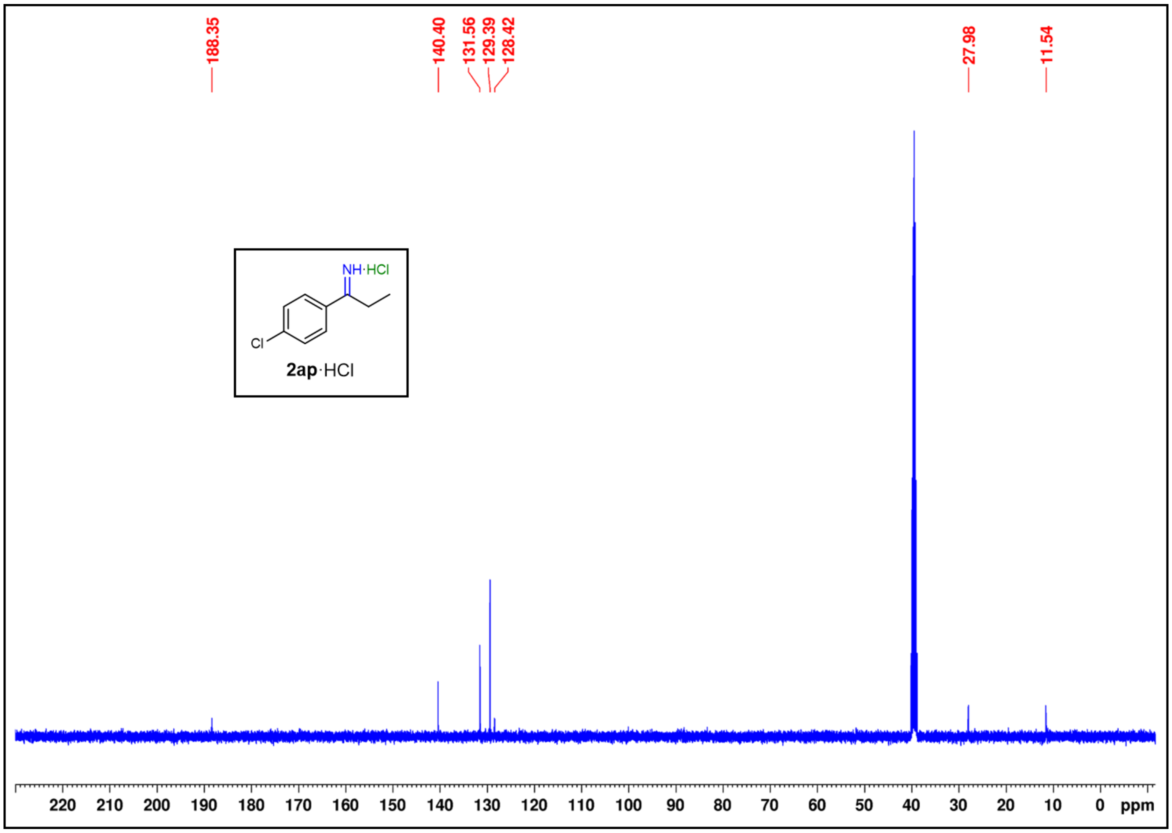

FT-IR (ATR, neat) and HRMS (ESI-positive) spectra for **2ap**·HCl

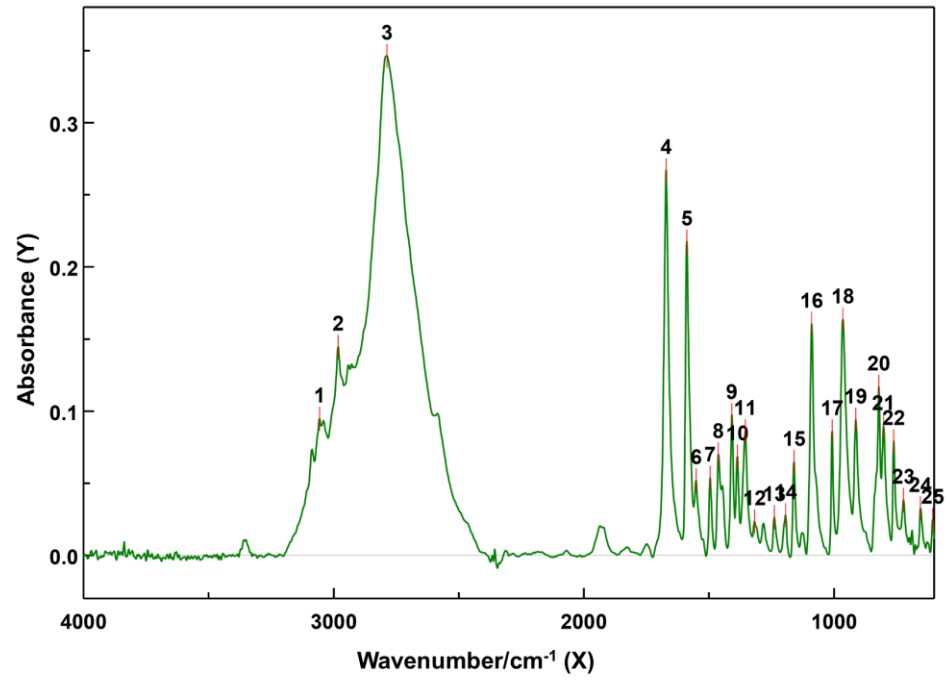

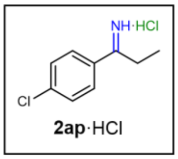

No.	X	Y	No.	X	Y	No.	X	Y
1	3056.6	0.095	10	1386.6	0.068	19	913.1	0.094
2	2982.4	0.145	11	1354.7	0.086	20	820.6	0.117
3	2787.6	0.346	12	1318.1	0.023	21	802.2	0.089
4	1671	0.267	13	1239	0.026	22	761.7	0.079
5	1589.1	0.217	14	1193.7	0.028	23	722.2	0.038
6	1551.5	0.052	15	1160	0.065	24	654.7	0.032
7	1495.5	0.054	16	1089.6	0.16	25	605.5	0.025
8	1462.7	0.07	17	1007.6	0.086			
9	1408.7	0.097	18	965.2	0.163			

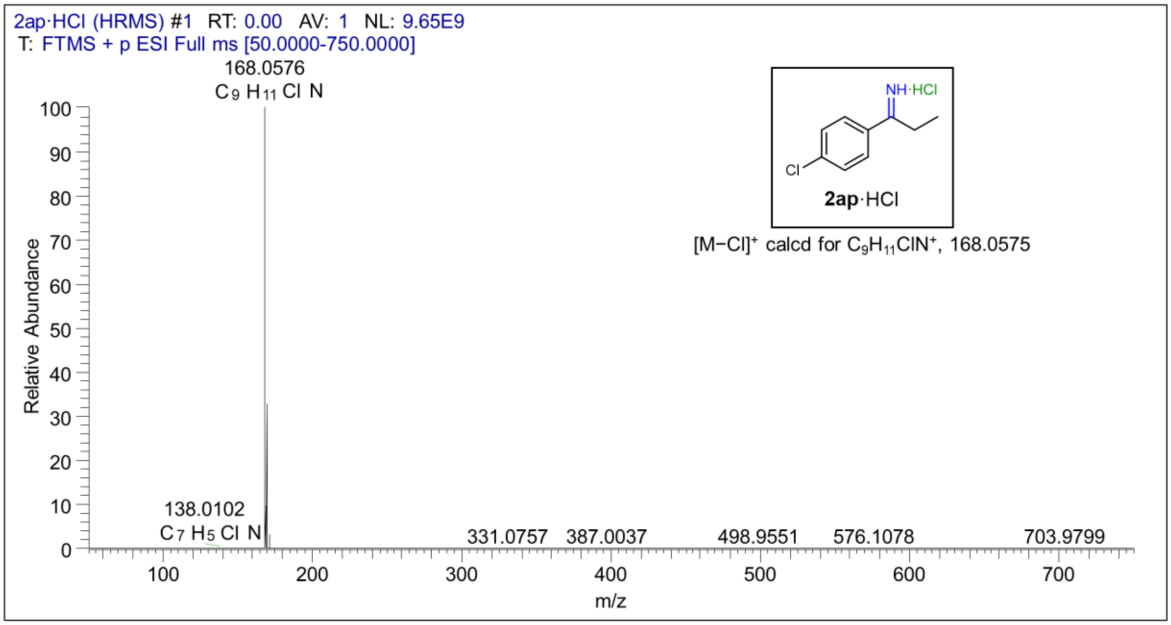

^{1}H NMR (400 MHz, DMSO-d_6) and ^{13}C NMR (100 MHz, DMSO-d_6) spectra for **2aq**·HCl

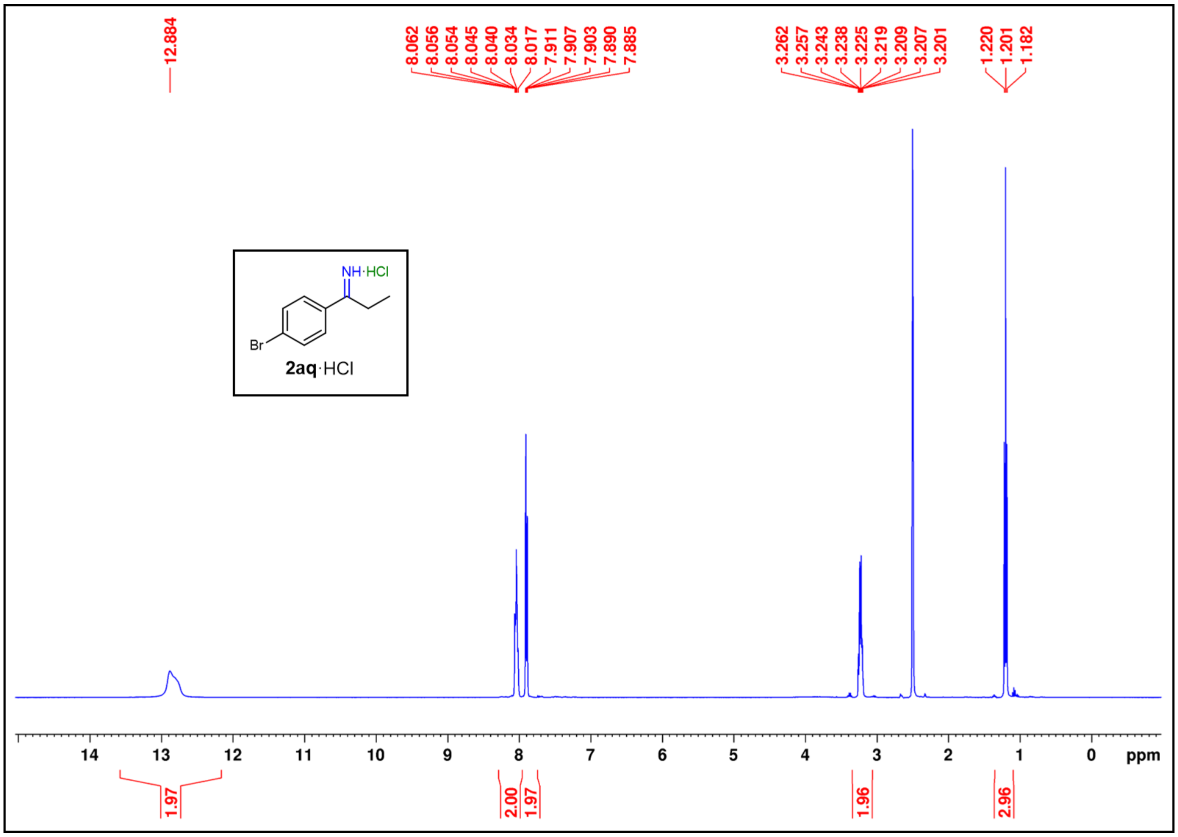

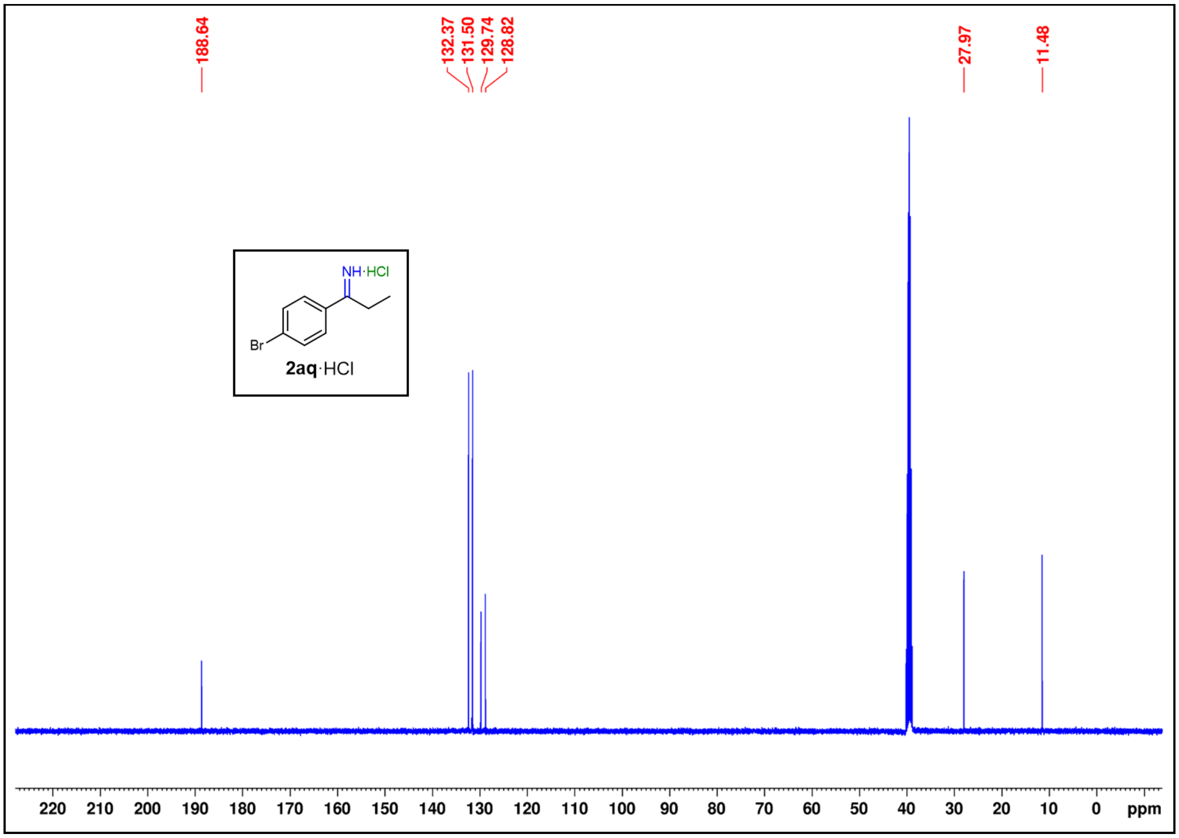

FT-IR (ATR, neat) and HRMS (ESI-positive) spectra for **2aq**·HCl

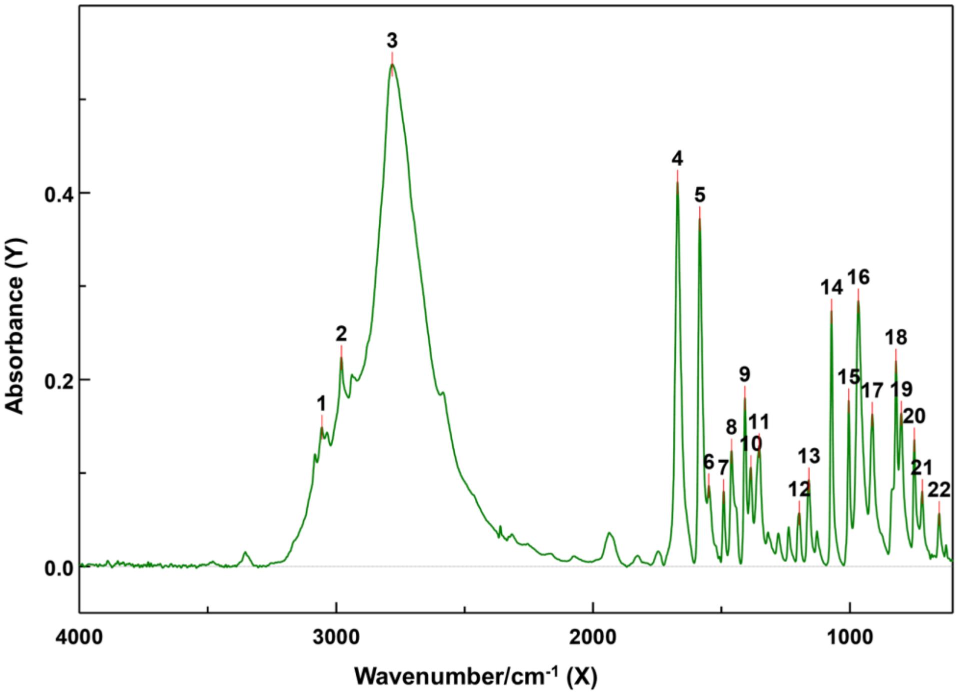

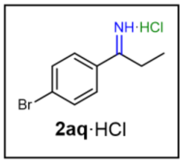

No.	X	Y	No.	X	Y	No.	X	Y
1	3055.7	0.149	9	1408.7	0.18	17	913.1	0.163
2	2979.5	0.223	10	1385.6	0.106	18	820.6	0.22
3	2781.8	0.537	11	1352.8	0.129	19	800.3	0.164
4	1671	0.411	12	1197.6	0.057	20	749.2	0.135
5	1584.2	0.372	13	1160	0.092	21	718.4	0.08
6	1549.5	0.086	14	1071.3	0.273	22	652.8	0.056
7	1491.7	0.08	15	1004.7	0.177			
8	1460.8	0.123	16	967.1	0.284			

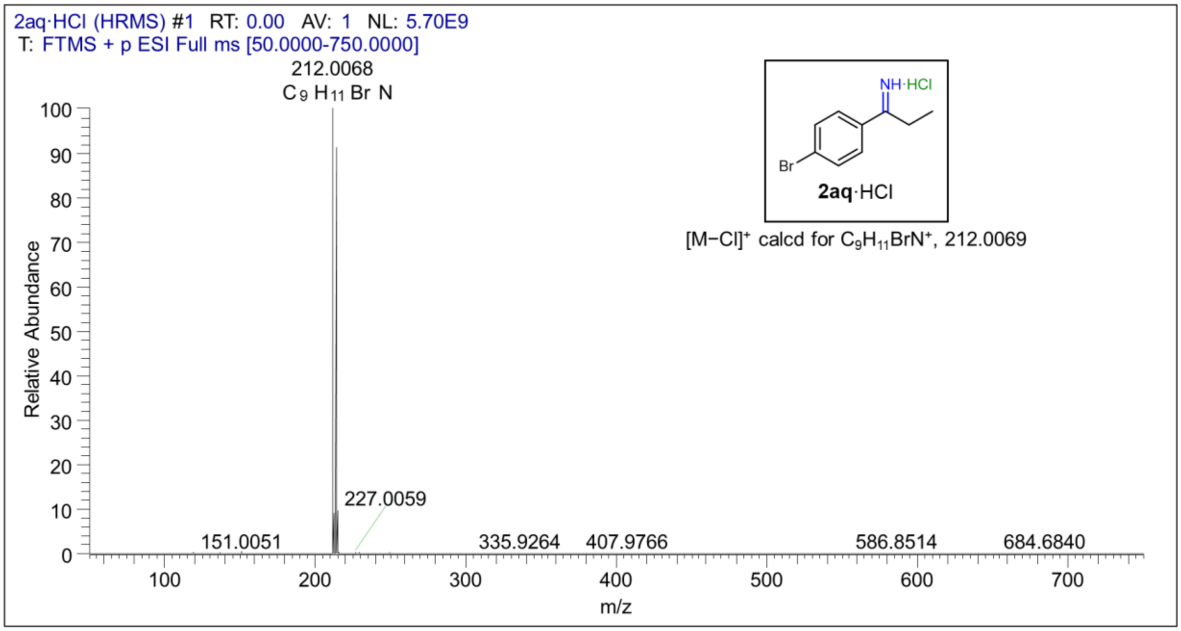

^{1}H NMR (400 MHz, DMSO-d_6) and ^{13}C NMR (100 MHz, DMSO-d_6) spectra for **2ar**·HCl

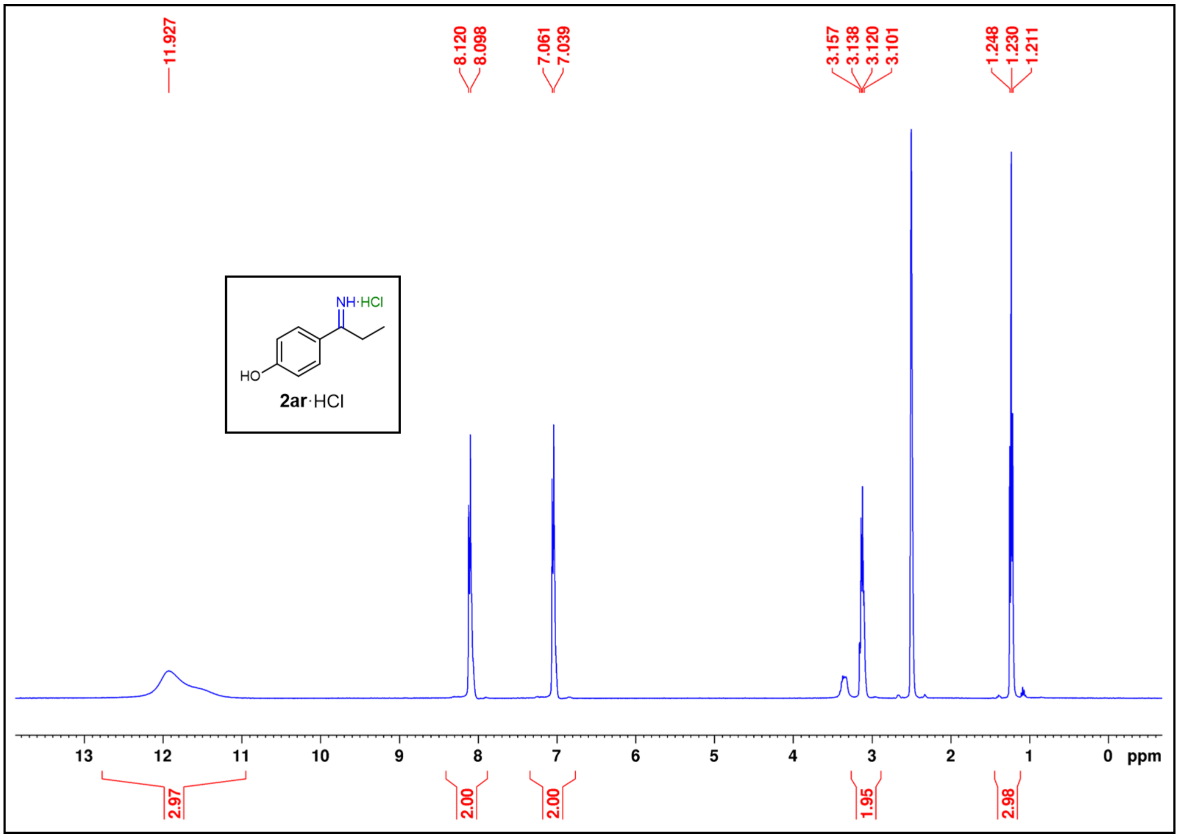

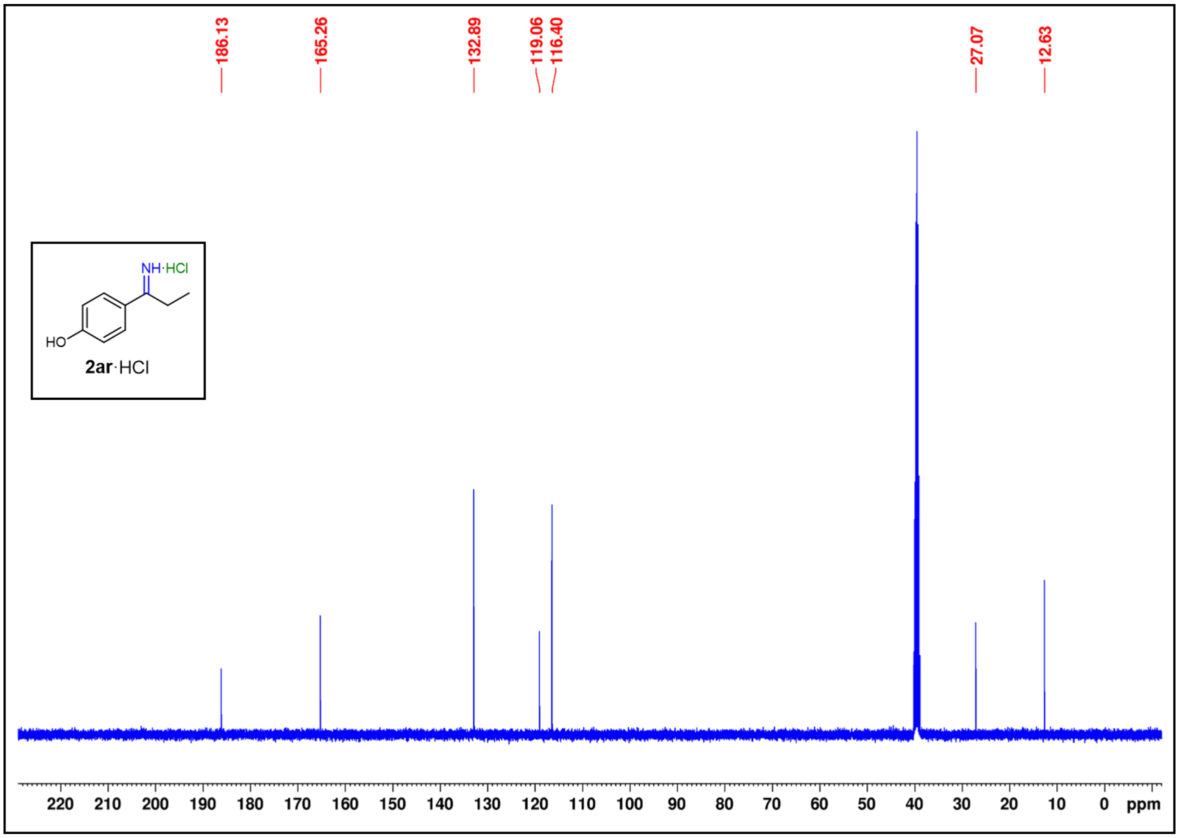

FT-IR (ATR, neat) and HRMS (ESI-positive) spectra for **2ar**·HCl

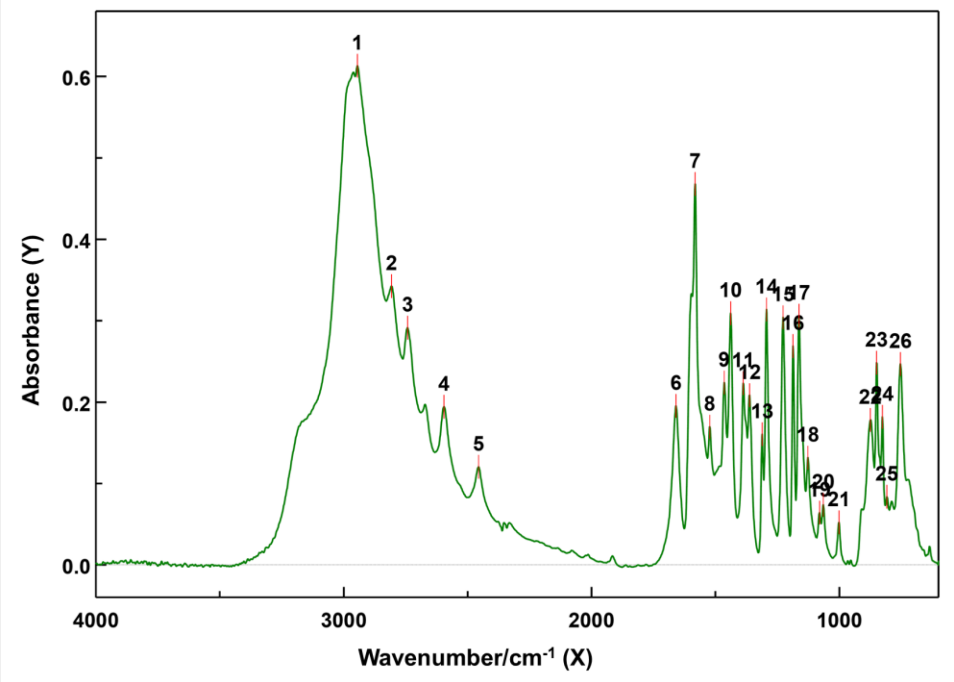

No.	X	Y	No.	X	Y	No.	X	Y
1	2944.8	0.613	10	1438.6	0.309	19	1080	0.064
2	2806.9	0.342	11	1387.5	0.223	20	1065	0.074
3	2742.3	0.291	12	1362.5	0.208	21	1002	0.052
4	2595.7	0.194	13	1311.4	0.16	22	874.6	0.178
5	2455.9	0.12	14	1294	0.313	23	849.5	0.248
6	1659.4	0.195	15	1227.5	0.304	24	826.3	0.182
7	1582.3	0.468	16	1187	0.269	25	808	0.083
8	1522.5	0.169	17	1162.9	0.306	26	753.1	0.247
9	1464.7	0.224	18	1127.2	0.131			

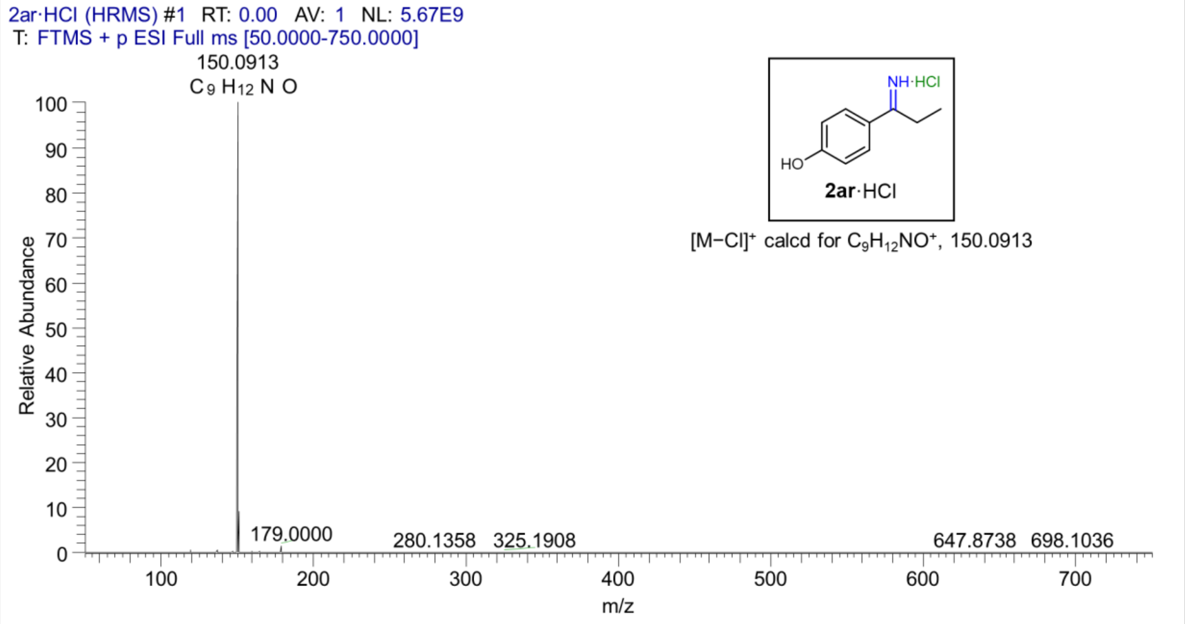

^{1}H NMR (400 MHz, DMSO-d_6) and ^{13}C NMR (100 MHz, DMSO-d_6) spectra for **2as**·HCl

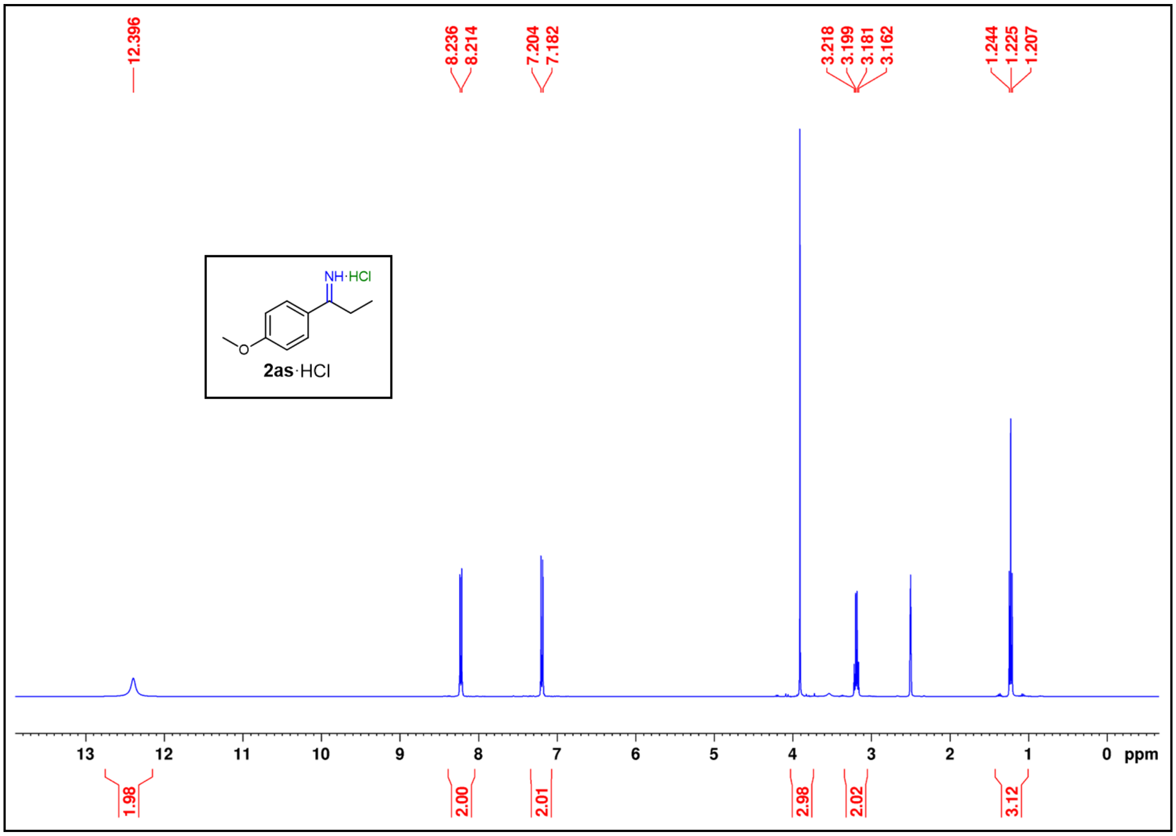

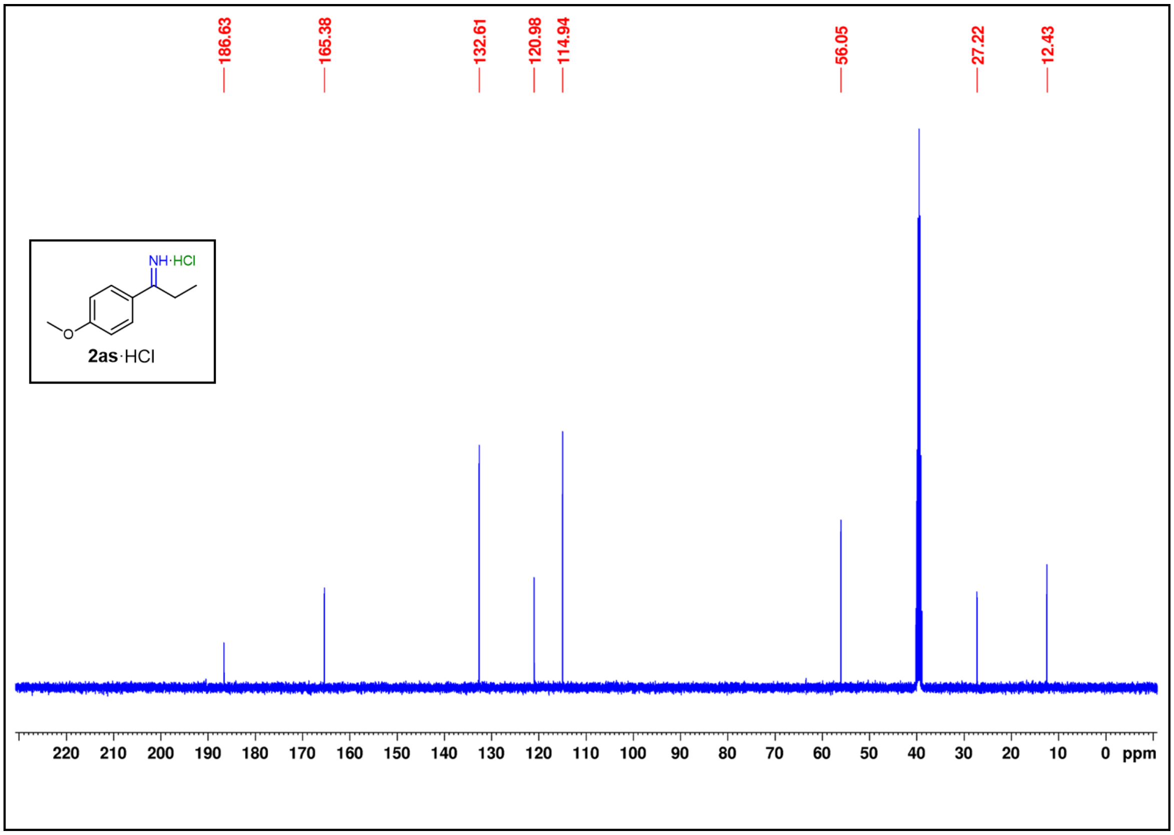

FT-IR (ATR, neat) and HRMS (ESI-positive) spectra for **2as**·HCl

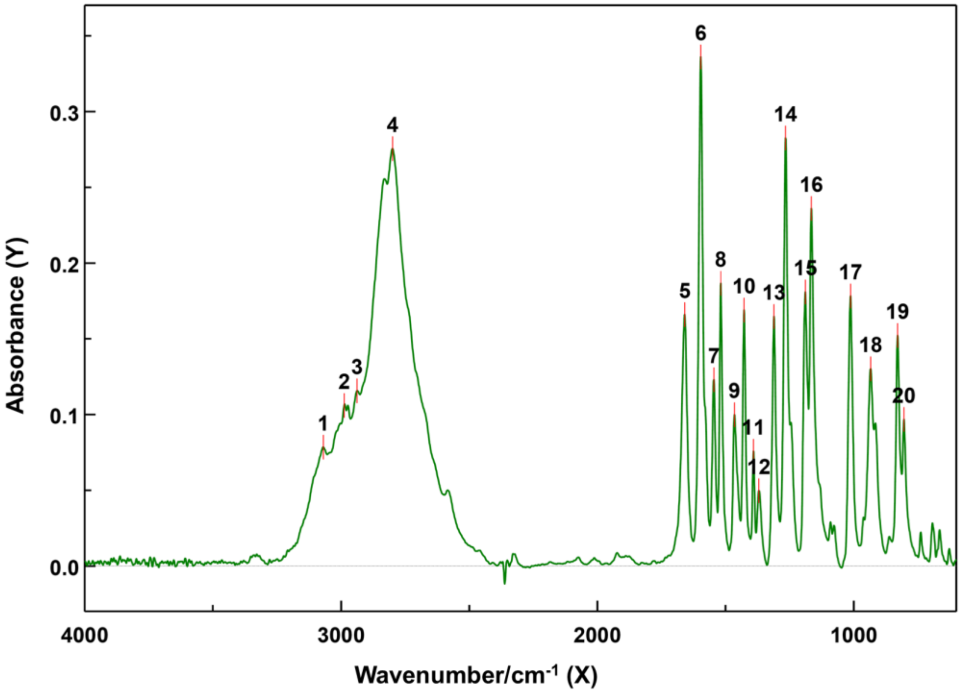

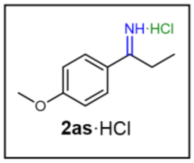

No.	X	Y		No.	X	Y		No.	X	Y
1	3069.2	0.078		8	1518.7	0.186		15	1189	0.181
2	2988.2	0.106		9	1465.6	0.1		16	1166	0.236
3	2938	0.116		10	1428	0.169		17	1012	0.178
4	2799.2	0.275		11	1391.4	0.076		18	934.3	0.13
5	1659.4	0.166		12	1370.2	0.05		19	829.2	0.152
6	1596.8	0.336		13	1311.4	0.165		20	804.2	0.097
7	1545.7	0.123		14	1266	0.282				

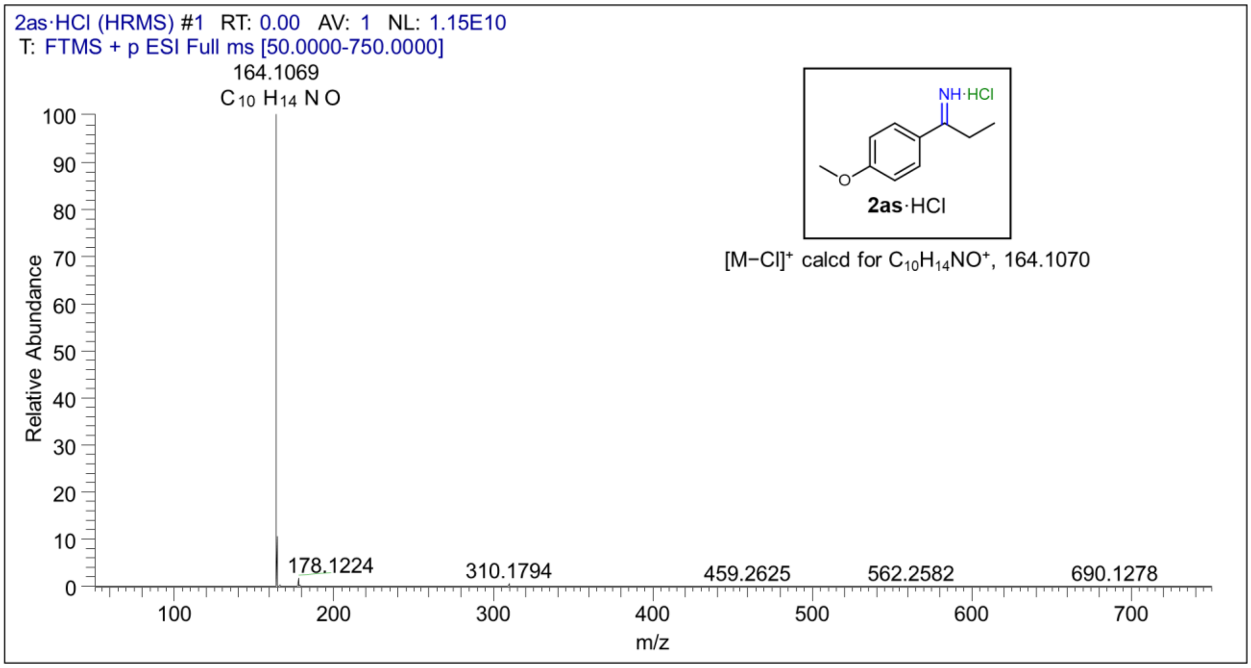

^{1}H NMR (400 MHz, DMSO-d_6) and ^{13}C NMR (100 MHz, DMSO-d_6) spectra for **2at**·HCl

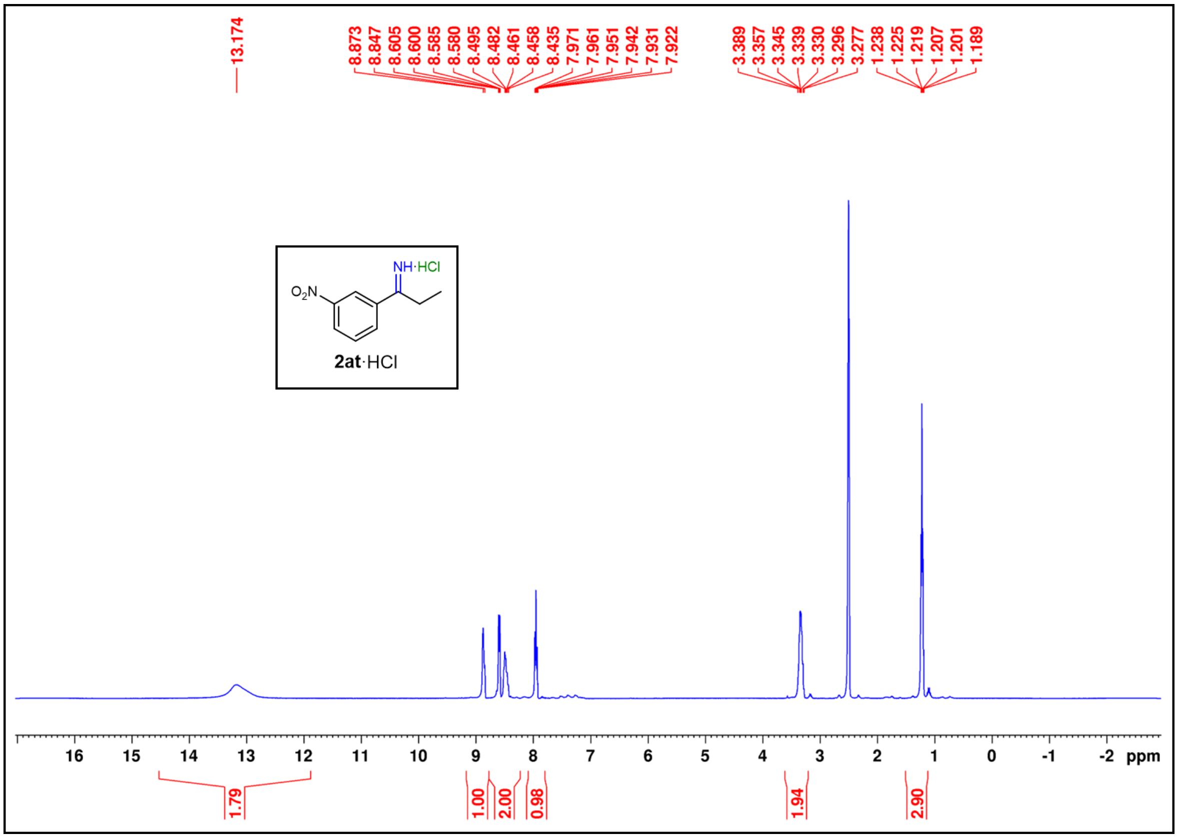

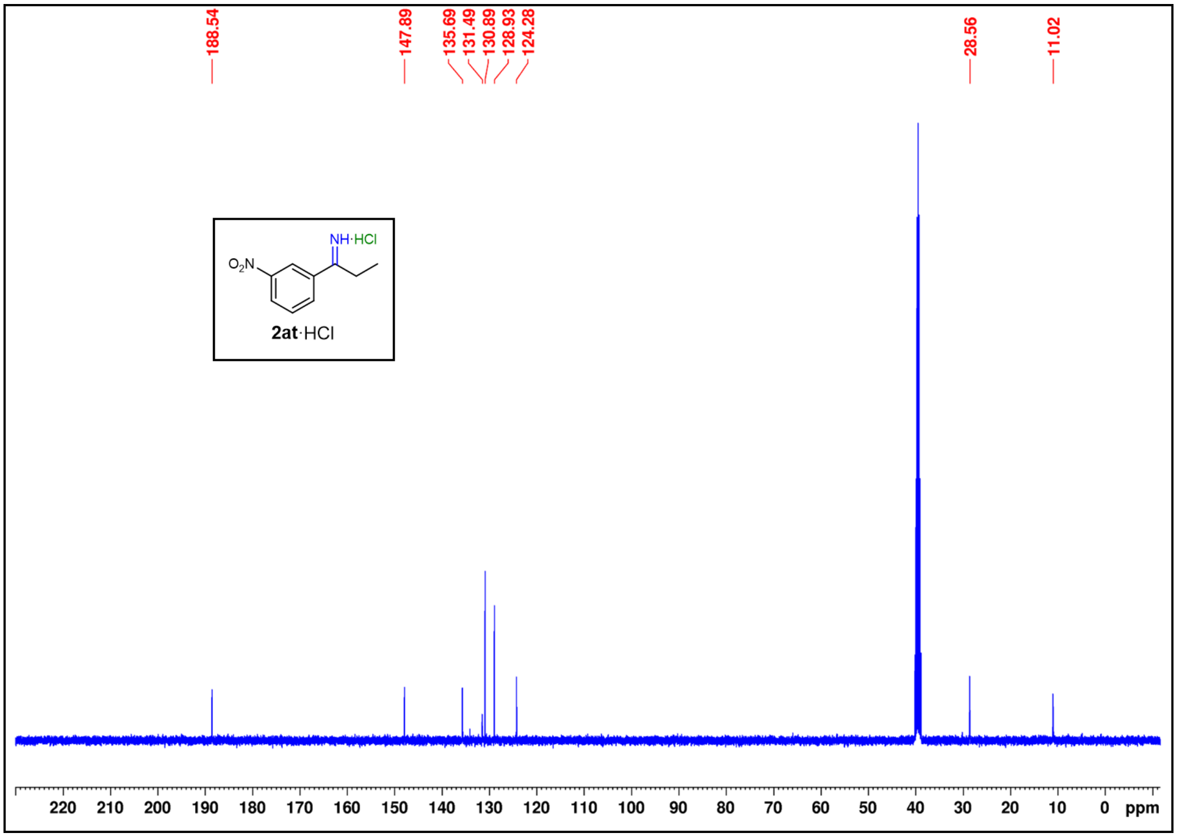

FT-IR (ATR, neat) and HRMS (ESI-positive) spectra for **2at**·HCl

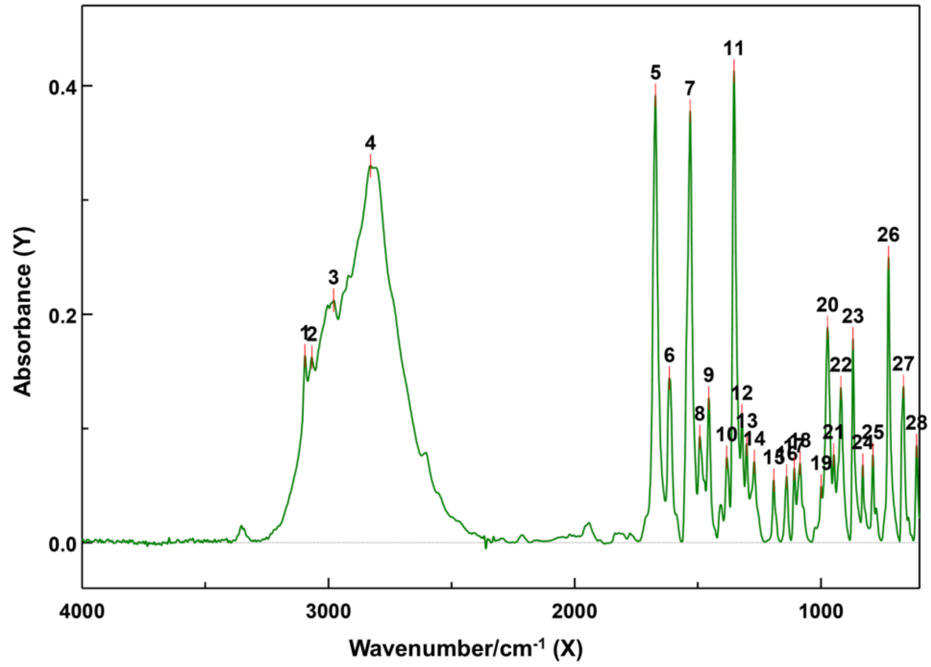

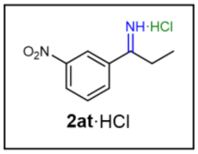

No.	X	Y	No.	X	Y	No.	X	Y
1	3095.2	0.164	11	1352.8	0.413	21	947.8	0.077
2	3067.2	0.162	12	1321	0.111	22	918.9	0.136
3	2978.5	0.212	13	1301.7	0.086	23	869.7	0.178
4	2829.1	0.33	14	1270.9	0.071	24	830.2	0.068
5	1672	0.391	15	1191.8	0.055	25	788.7	0.077
6	1615.1	0.144	16	1139.7	0.058	26	726.1	0.249
7	1531.2	0.378	17	1107.9	0.065	27	665.3	0.136
8	1491.7	0.093	18	1084.8	0.069	28	611.3	0.085
9	1455	0.126	19	998.9	0.049			
10	1382.7	0.075	20	972.9	0.188			

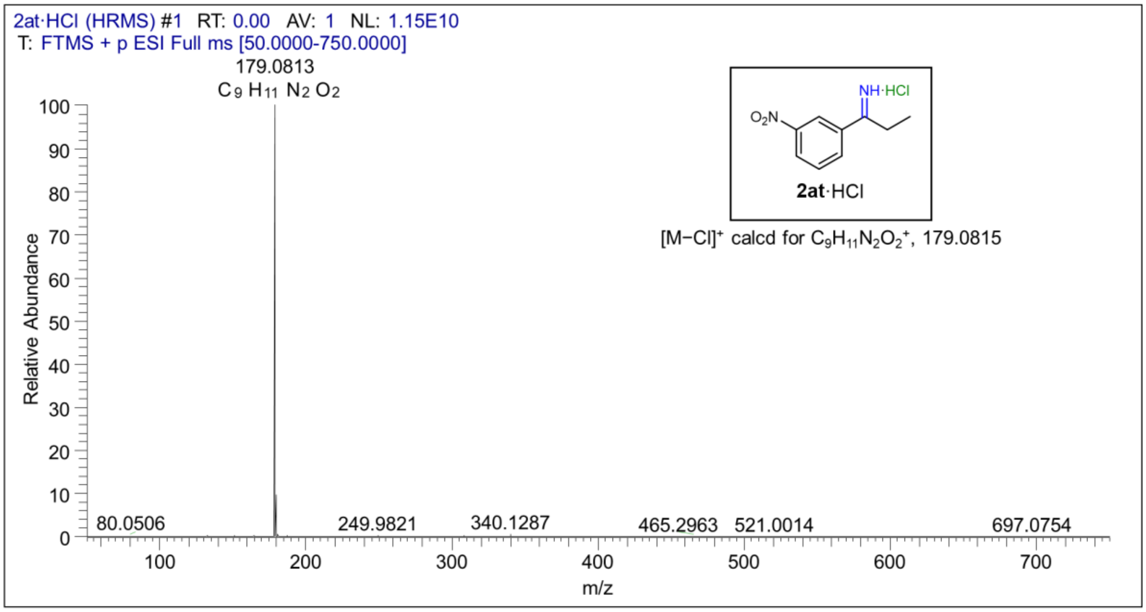

^{1}H NMR (400 MHz, DMSO-d_6) and ^{13}C NMR (100 MHz, DMSO-d_6) spectra for **2au**·HCl

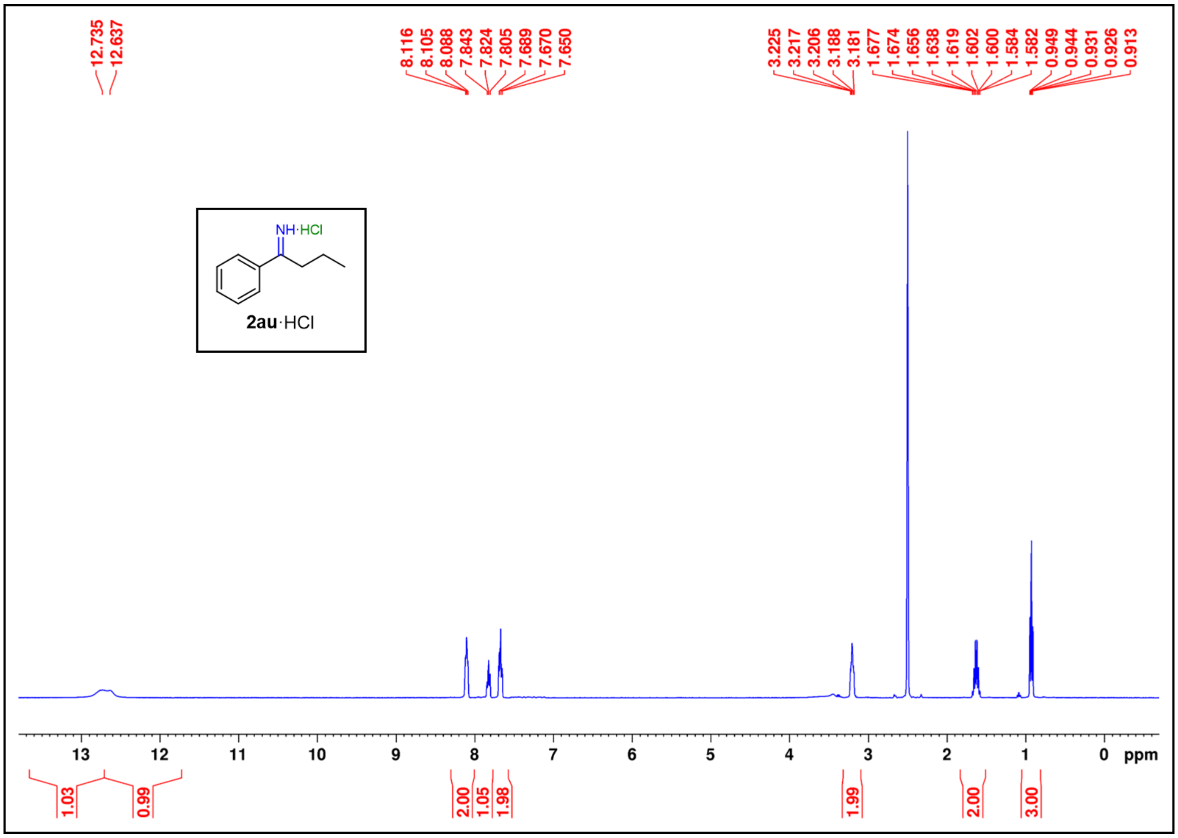

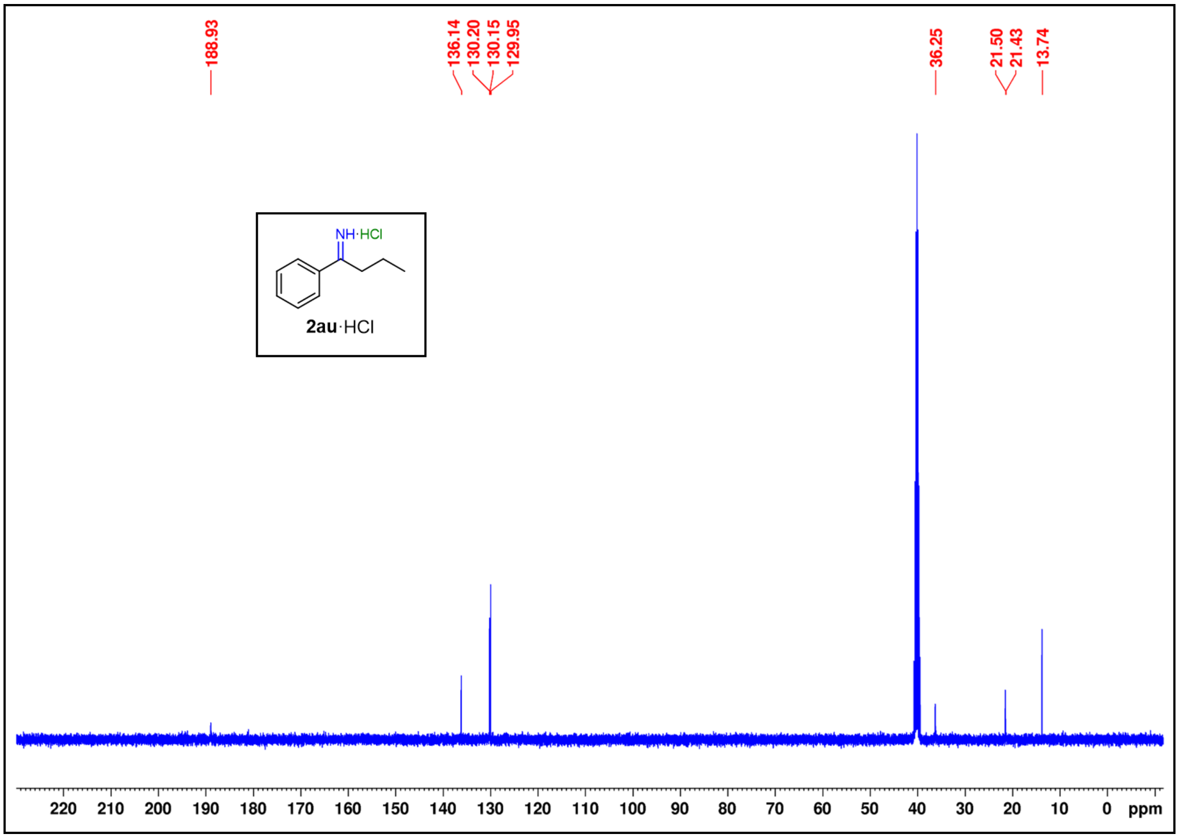

FT-IR (ATR, neat) and HRMS (ESI-positive) spectra for **2au**·HCl

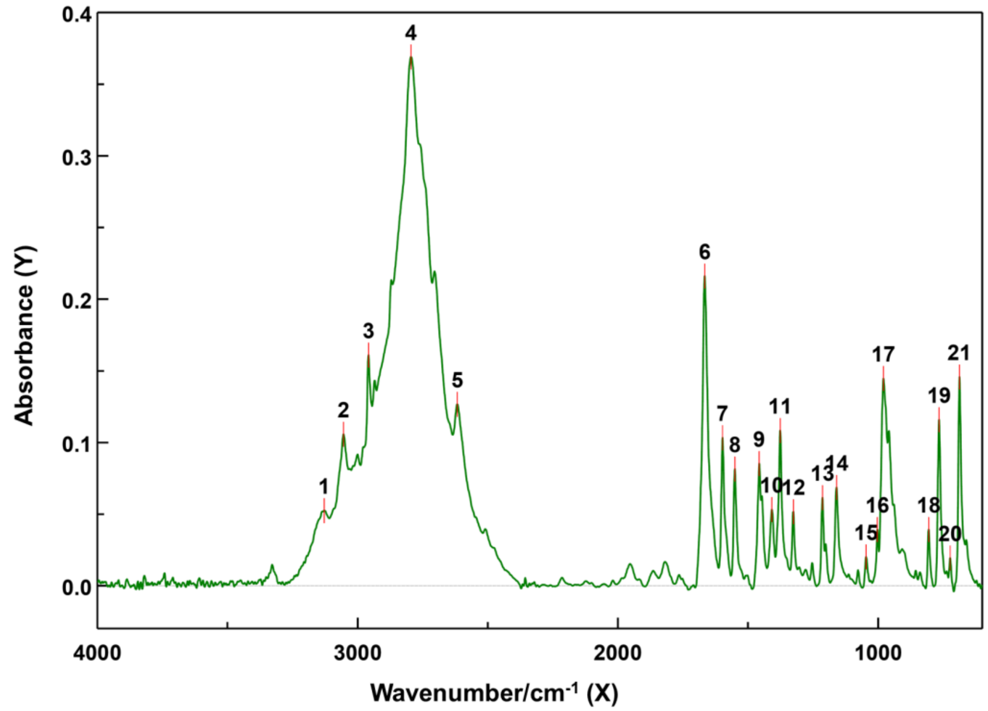

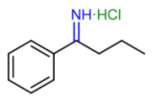

2au·HCl

No.	X	Y	No.	X	Y	No.	X	Y
1	3128.9	0.052	8	1550.5	0.081	15	1046	0.02
2	3054.7	0.106	9	1457	0.085	16	1003	0.039
3	2958.3	0.161	10	1408.7	0.053	17	979.7	0.145
4	2794.3	0.369	11	1376	0.108	18	806.1	0.039
5	2616.9	0.126	12	1325.8	0.051	19	765.6	0.116
6	1666.2	0.216	13	1214	0.061	20	723.2	0.019
7	1597.7	0.103	14	1160	0.069	21	687.5	0.145

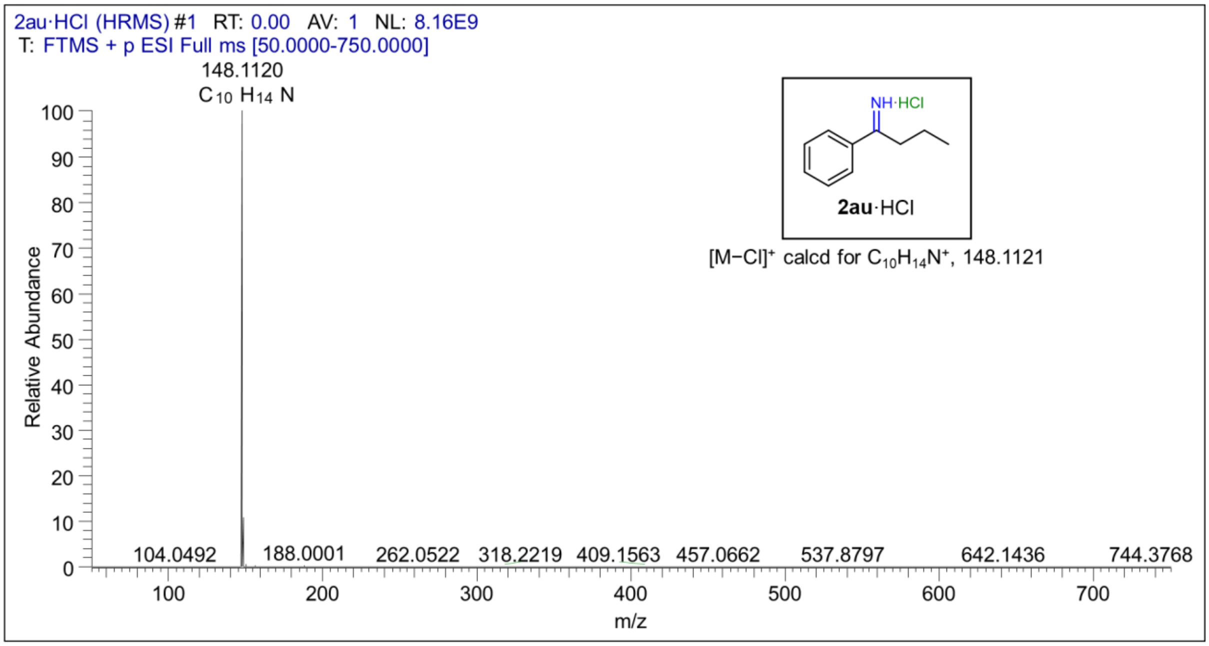

^{1}H NMR (400 MHz, DMSO-d_6) and ^{13}C NMR (100 MHz, DMSO-d_6) spectra for **2av**·HCl

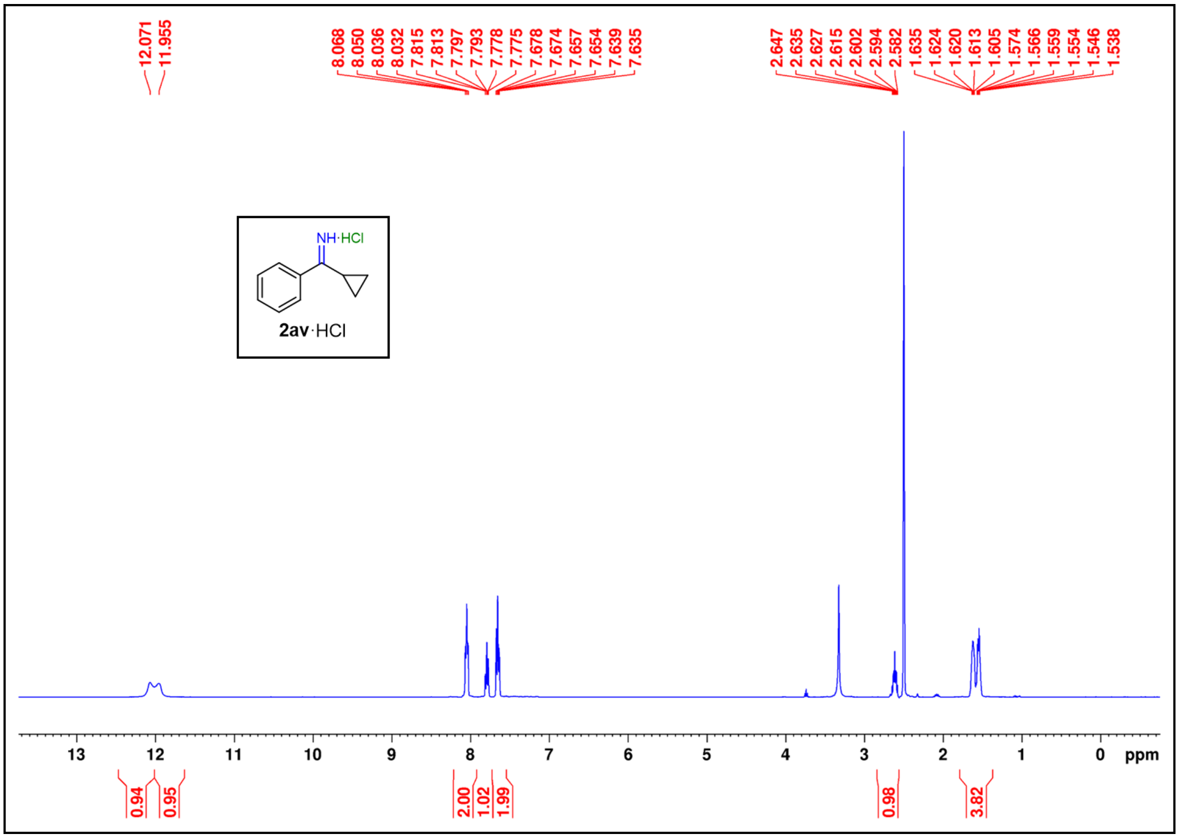

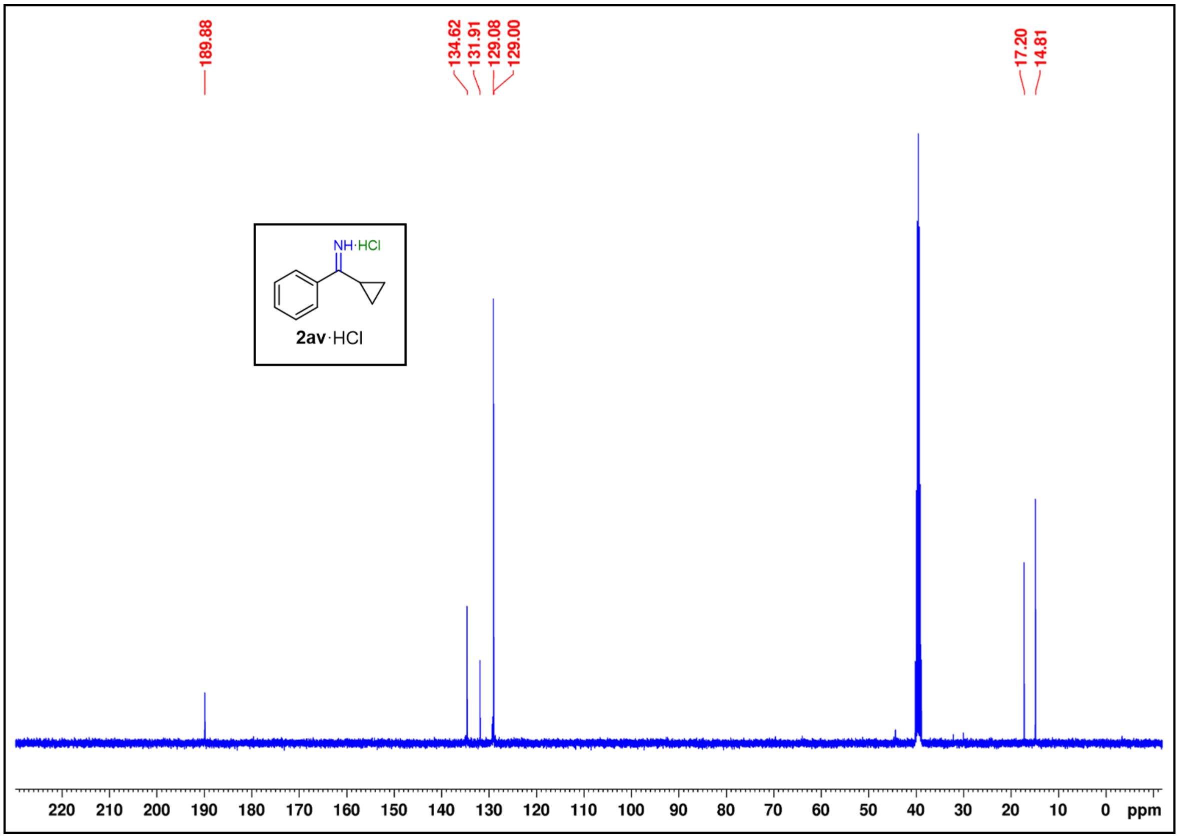

FT-IR (ATR, neat) and HRMS (ESI-positive) spectra for **2av**·HCl

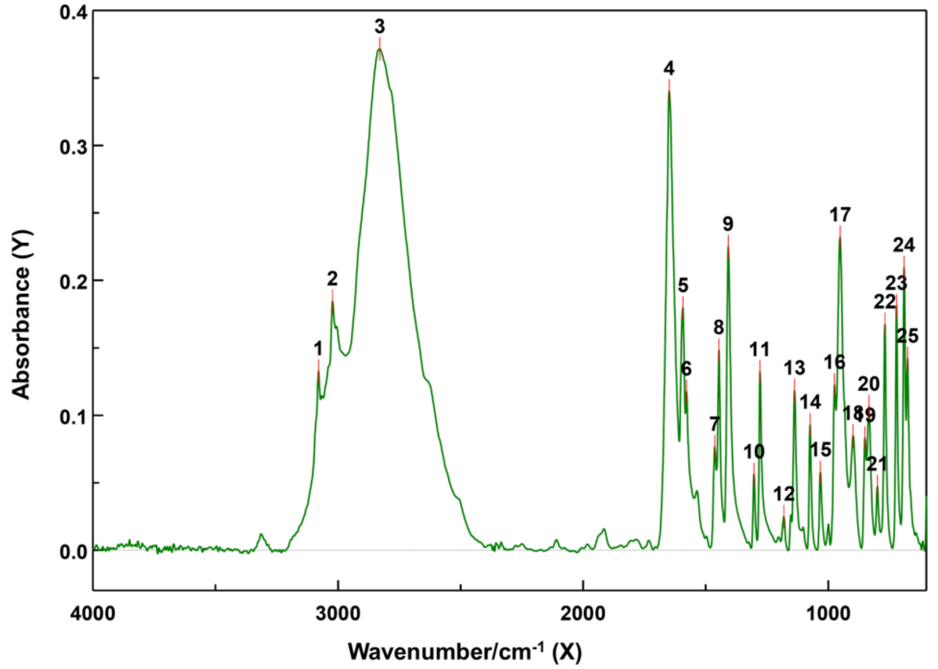

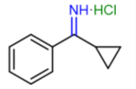

2av·HCl

No.	X	Y	No.	X	Y	No.	X	Y
1	3079.8	0.133	10	1302.7	0.056	19	850.5	0.083
2	3021.9	0.184	11	1278.6	0.132	20	834.1	0.106
3	2830	0.371	12	1181.2	0.025	21	799.4	0.047
4	1648.8	0.34	13	1137.8	0.118	22	769.5	0.167
5	1592.9	0.179	14	1074.2	0.093	23	721.2	0.181
6	1578.5	0.118	15	1032.7	0.058	24	690.4	0.209
7	1462.7	0.077	16	974.8	0.122	25	676.9	0.142
8	1446.4	0.148	17	951.7	0.232			
9	1407.8	0.225	18	898.7	0.085			

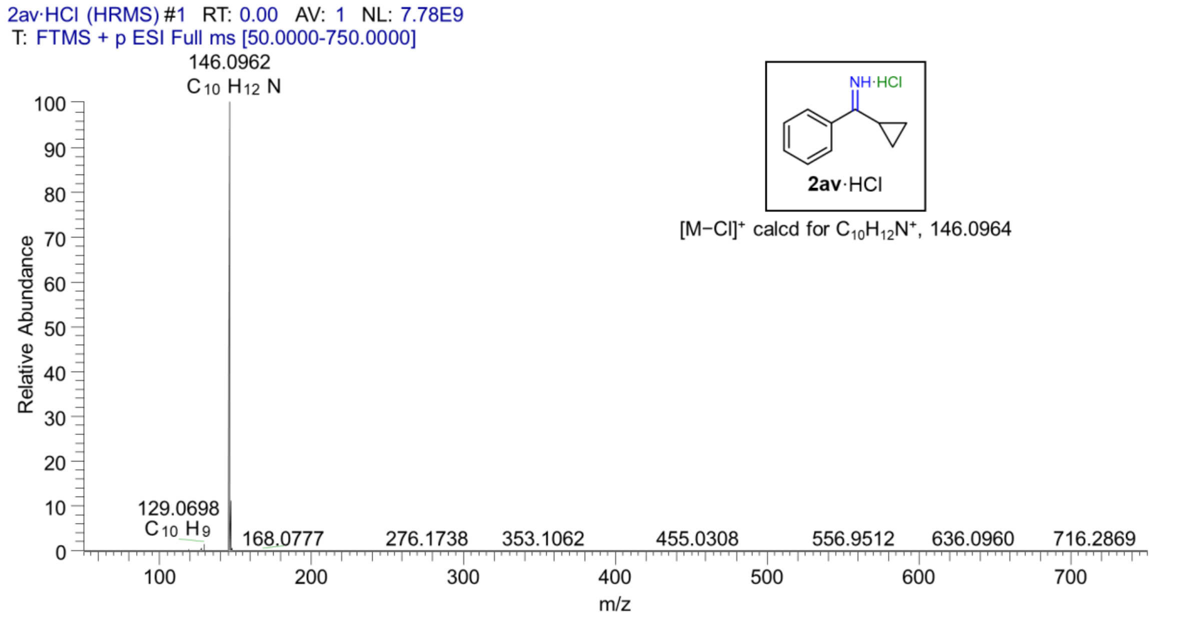

^{1}H NMR (400 MHz, DMSO-d_6) and ^{13}C NMR (100 MHz, DMSO-d_6) spectra for **2aw**·HCl

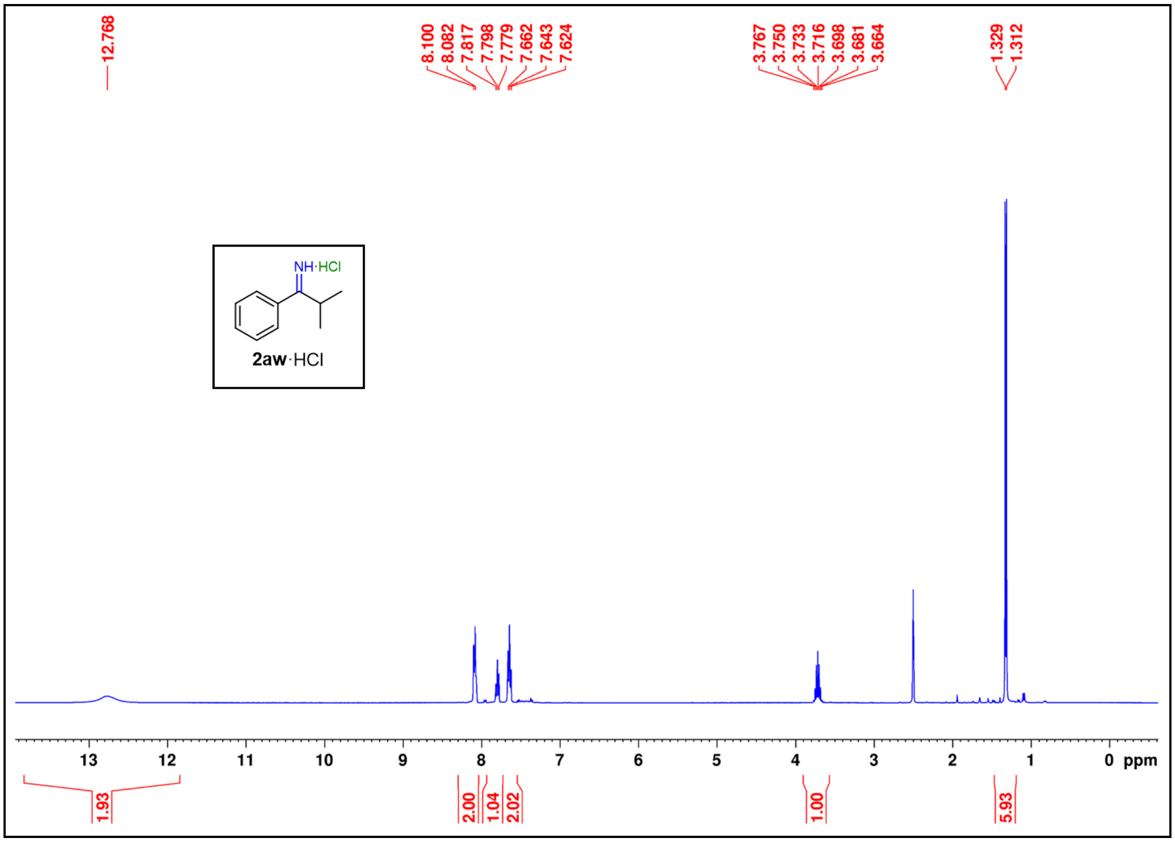

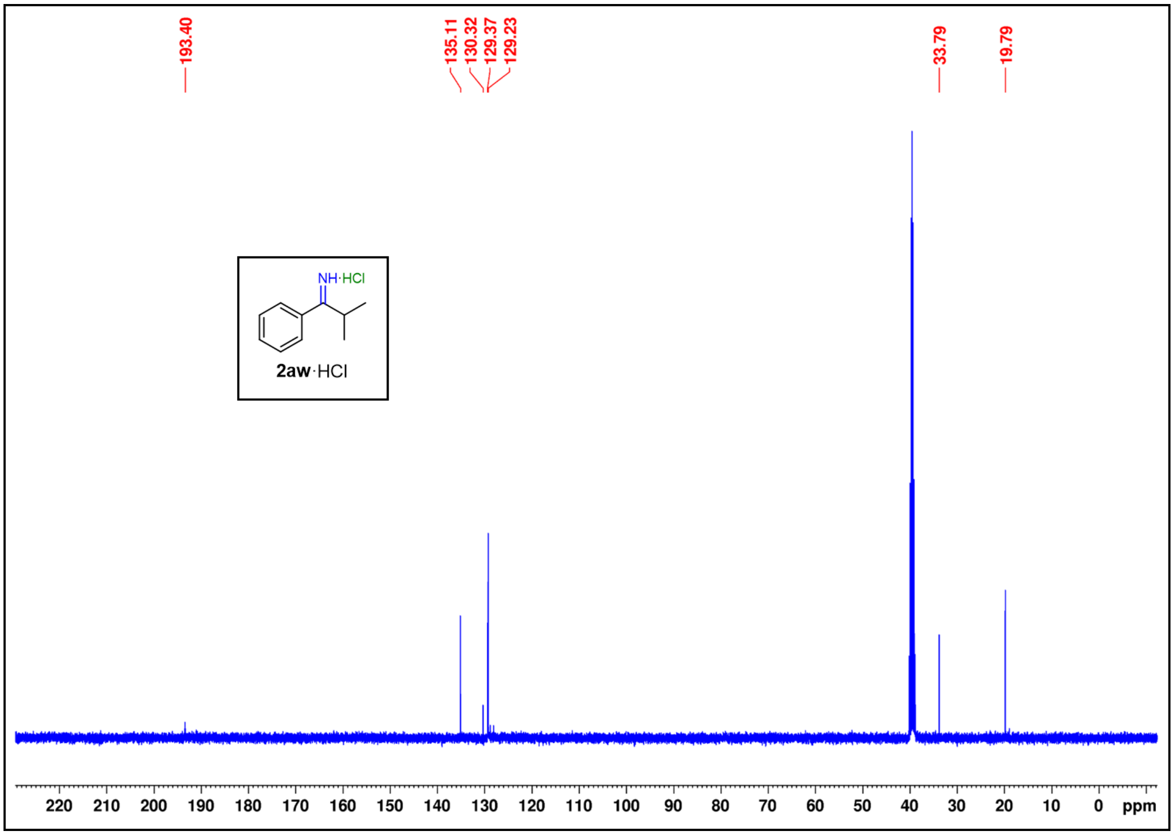

FT-IR (ATR, neat) and HRMS (ESI-positive) spectra for **2aw**·HCl

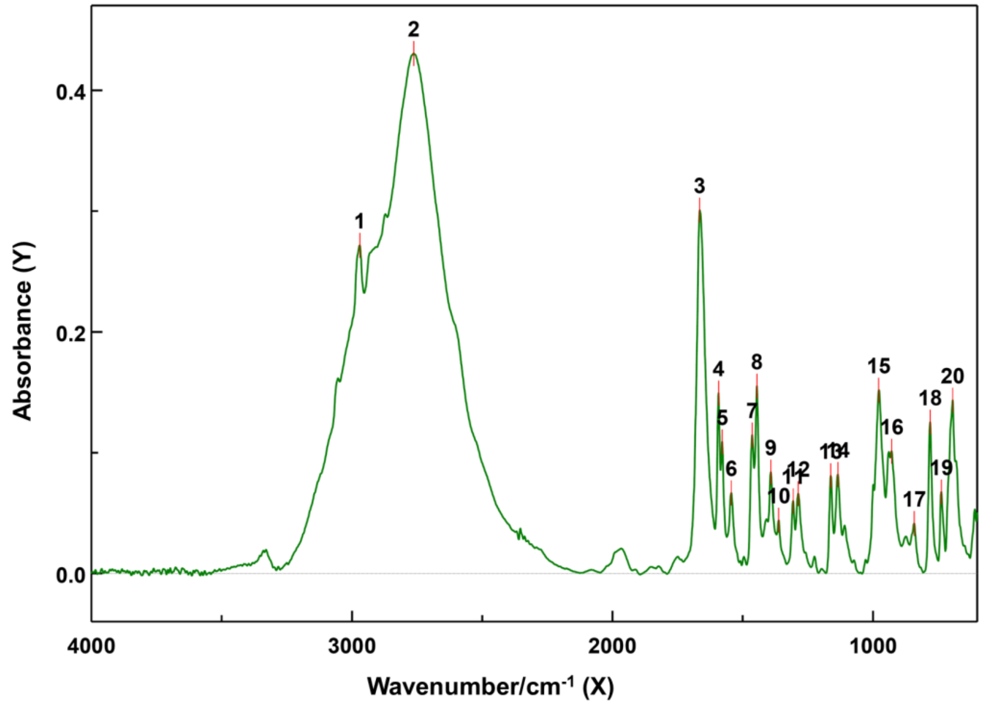

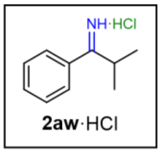

No.	X	Y	No.	X	Y	No.	X	Y
1	2969.8	0.271	8	1445.4	0.155	15	977.7	0.152
2	2763.5	0.43	9	1392.4	0.084	16	928.6	0.101
3	1665.2	0.301	10	1362.5	0.044	17	841.8	0.041
4	1592.9	0.149	11	1306.5	0.06	18	781	0.125
5	1578.5	0.109	12	1287.3	0.066	19	737.6	0.067
6	1543.7	0.067	13	1161.9	0.081	20	694.2	0.143
7	1463.7	0.114	14	1134.9	0.082			

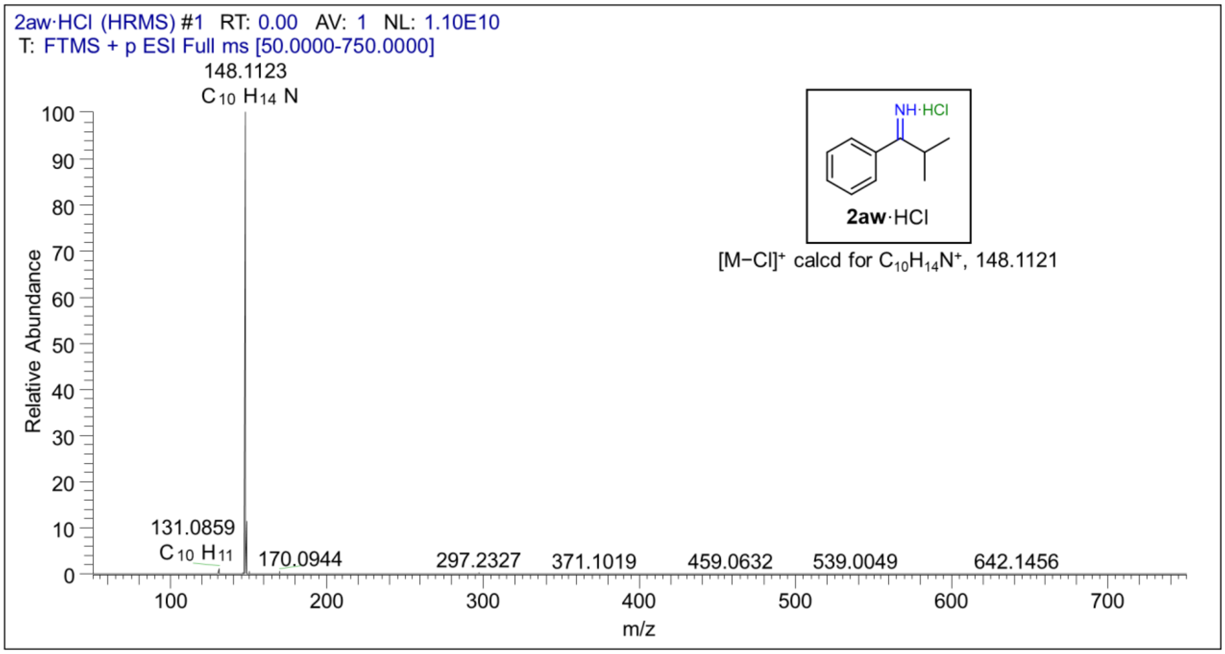

^{1}H NMR (400 MHz, DMSO-d_6) and ^{13}C NMR (100 MHz, DMSO-d_6) spectra for **2ax**·HCl

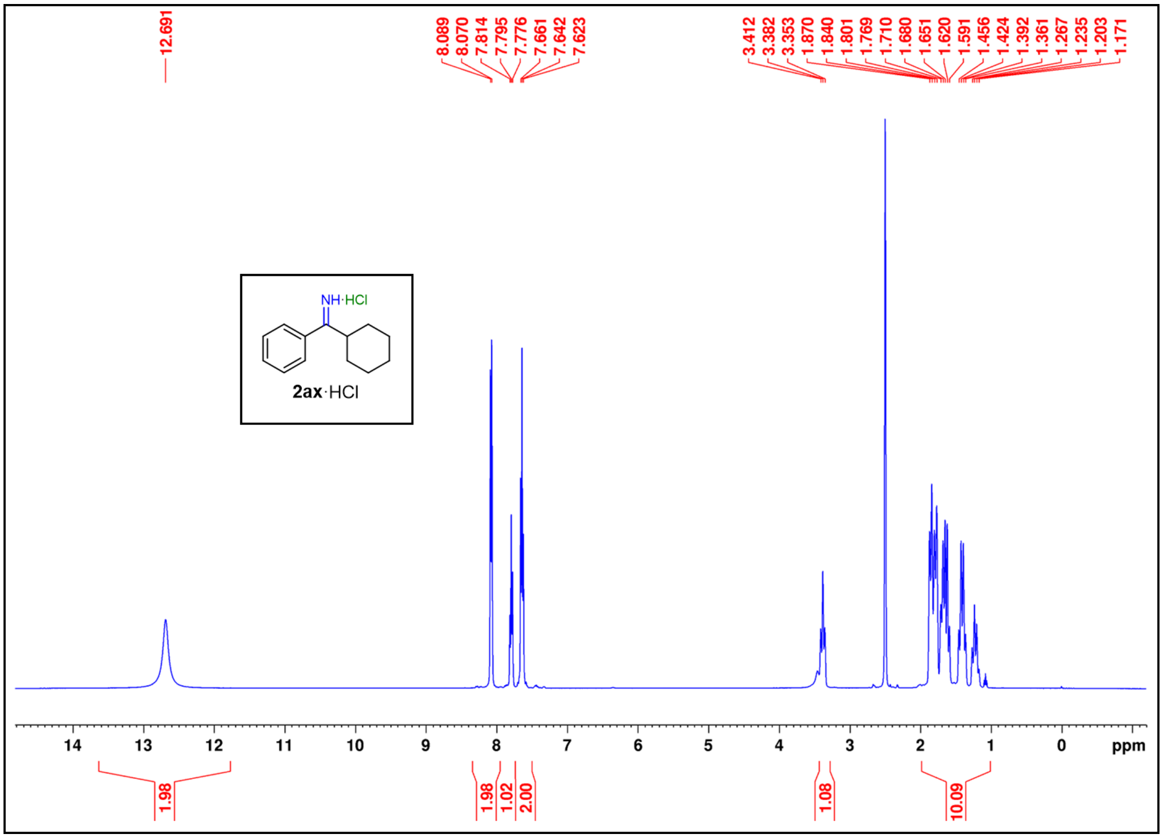

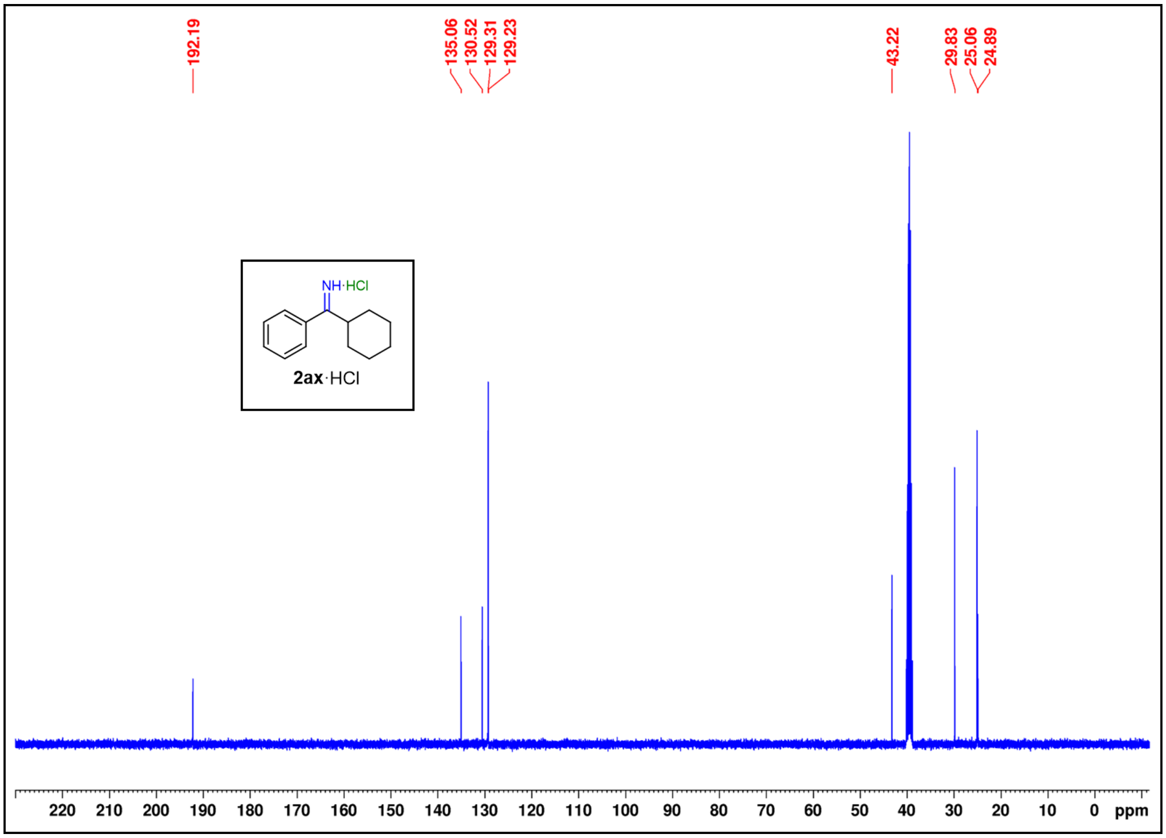

FT-IR (ATR, neat) and HRMS (ESI-positive) spectra for **2ax**·HCl

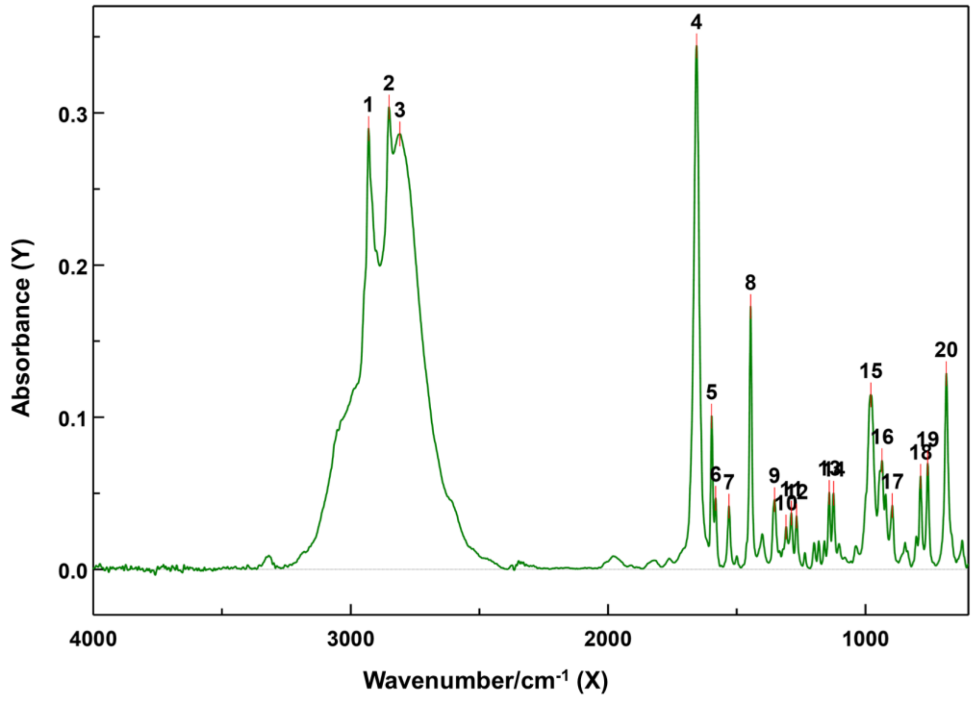

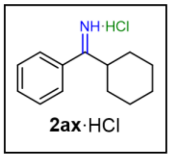

No.	X	Y	No.	X	Y	No.	X	Y
1	2930.3	0.289	8	1446.4	0.173	15	978.7	0.115
2	2851.2	0.304	9	1352.8	0.046	16	935.3	0.071
3	2808.8	0.286	10	1308.5	0.028	17	895.8	0.042
4	1656.6	0.344	11	1288.2	0.037	18	785.9	0.061
5	1597.7	0.101	12	1268	0.035	19	757.9	0.069
6	1582.3	0.047	13	1140.7	0.051	20	685.6	0.129
7	1530.2	0.042	14	1124.3	0.05			

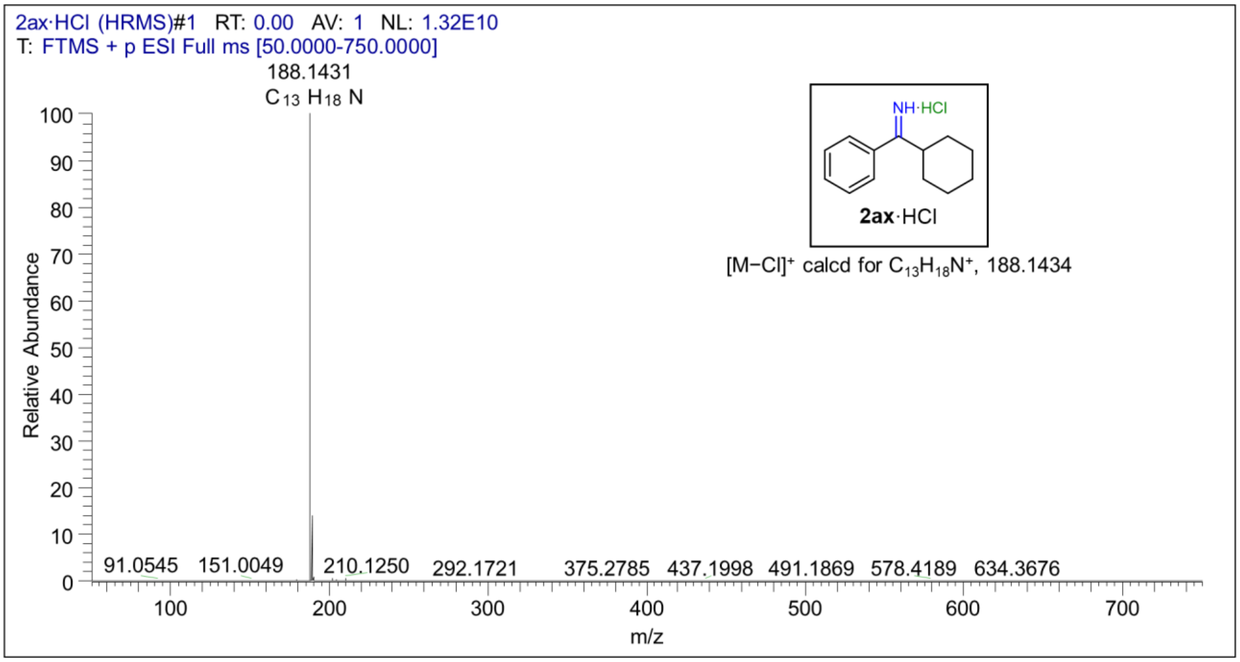

^{1}H NMR (400 MHz, DMSO-d_6) and ^{13}C NMR (100 MHz, DMSO-d_6) spectra for **2ay**·HCl

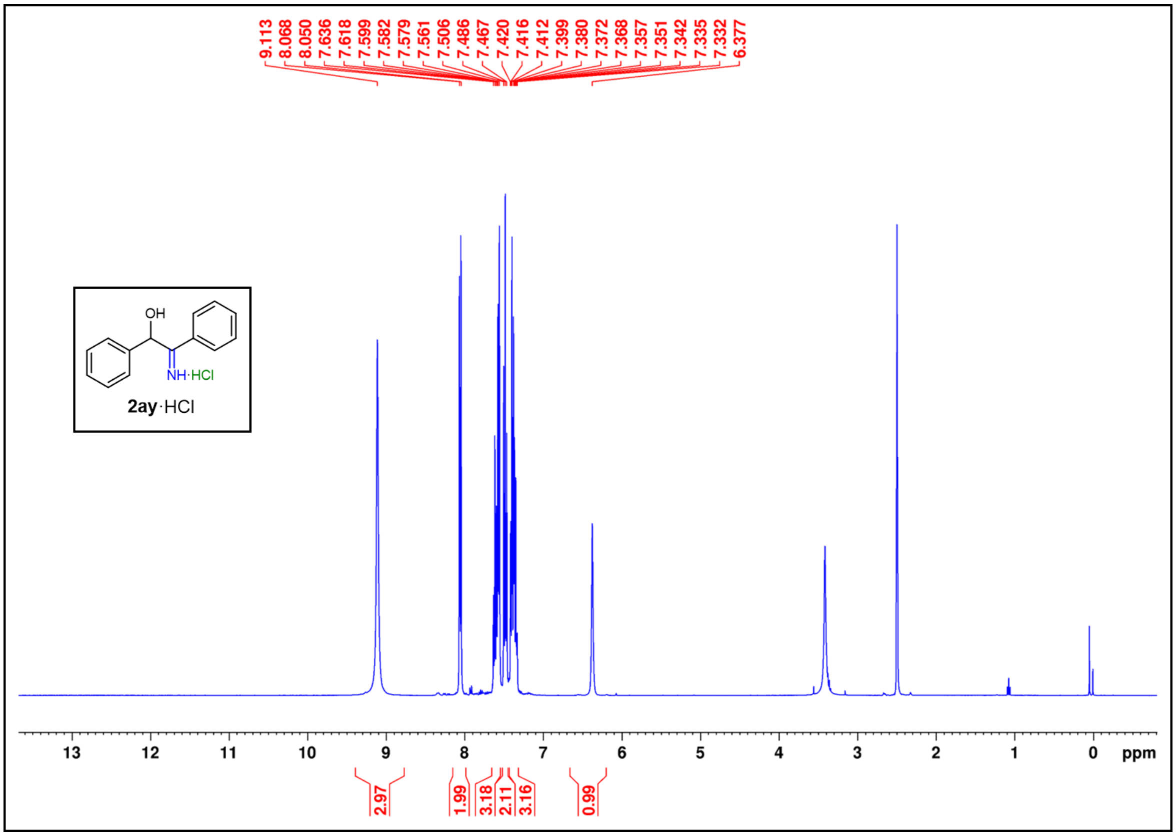

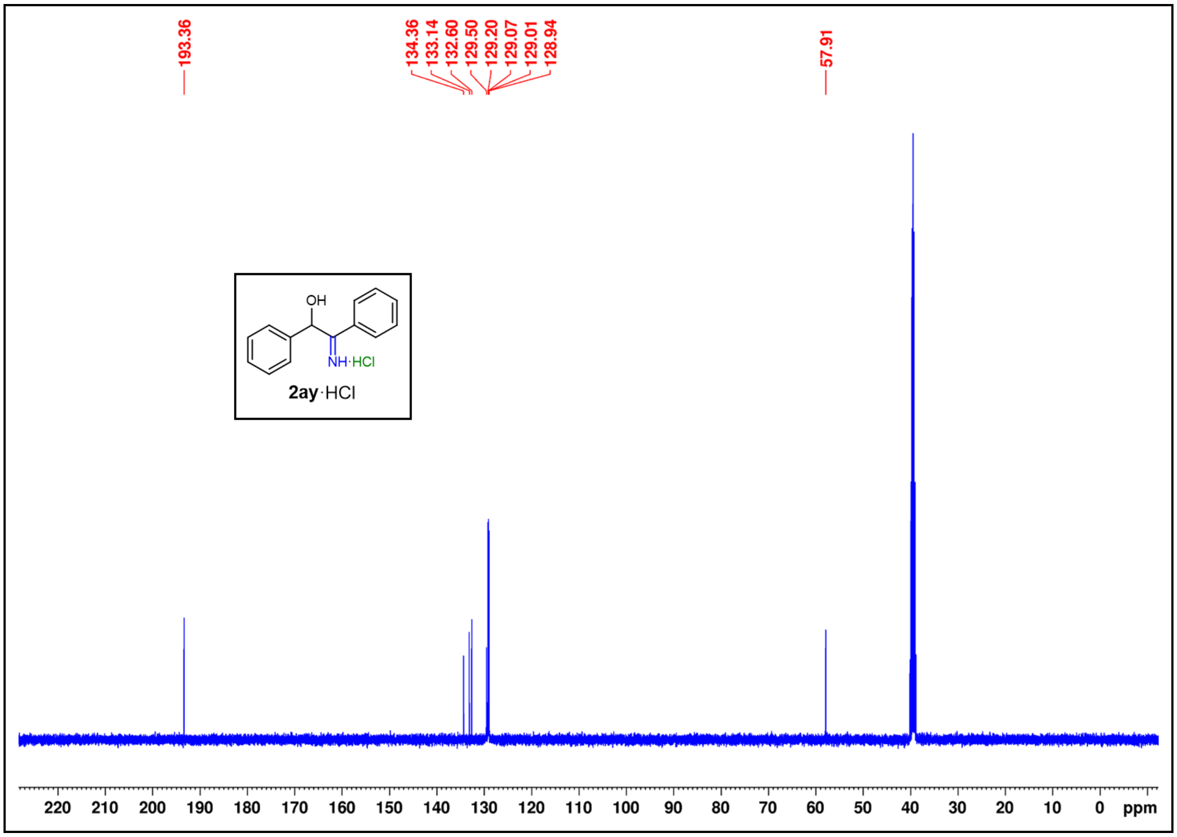

FT-IR (ATR, neat) and HRMS (ESI-positive) spectra for **2ay**·HCl

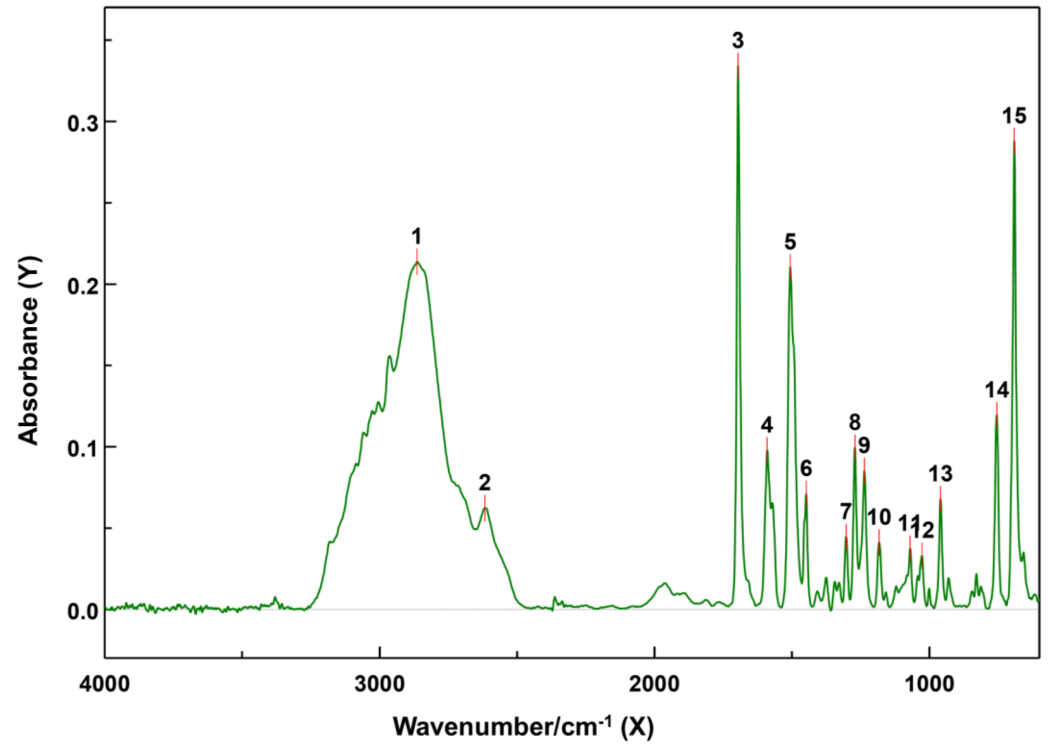

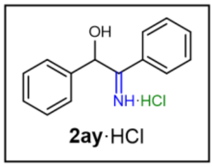

No.	X	Y	No.	X	Y	No.	X	Y
1	2863.8	0.214	6	1448.3	0.071	11	1070	0.037
2	2616.9	0.062	7	1302.7	0.044	12	1027	0.033
3	1696.1	0.334	8	1270.9	0.099	13	959.4	0.068
4	1590	0.098	9	1236.1	0.085	14	755	0.119
5	1506.1	0.21	10	1183.1	0.041	15	691.4	0.288

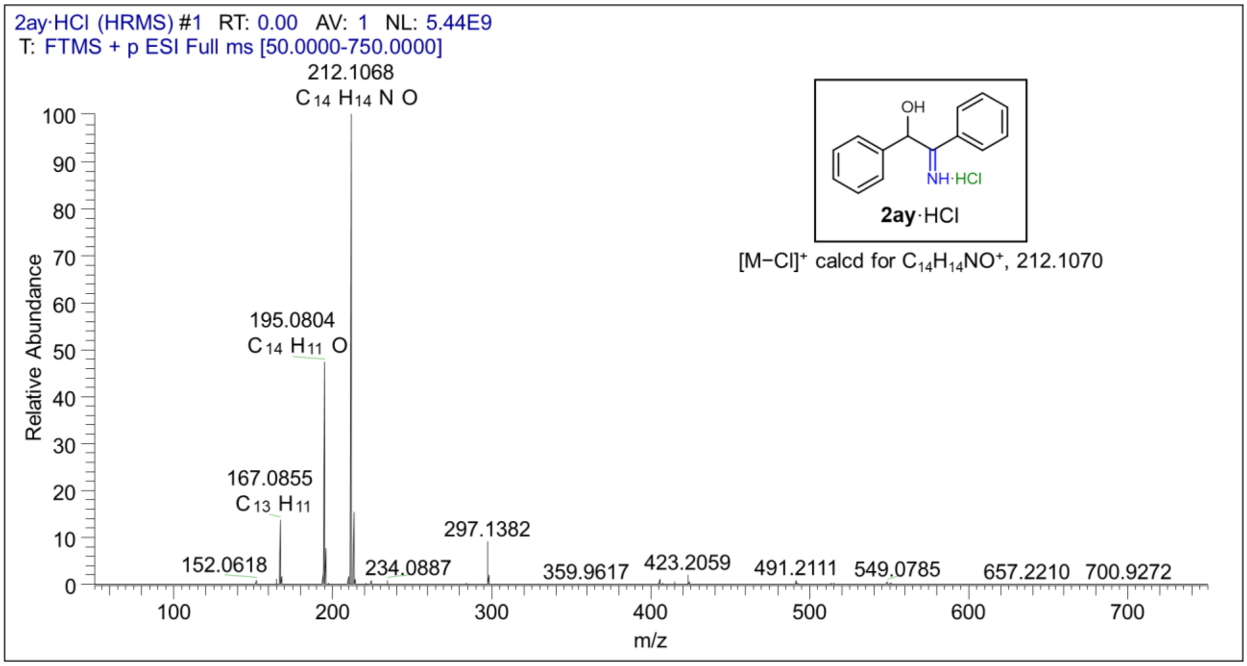

^{1}H NMR (400 MHz, DMSO-d_6) and ^{13}C NMR (100 MHz, DMSO-d_6) spectra for **2az**

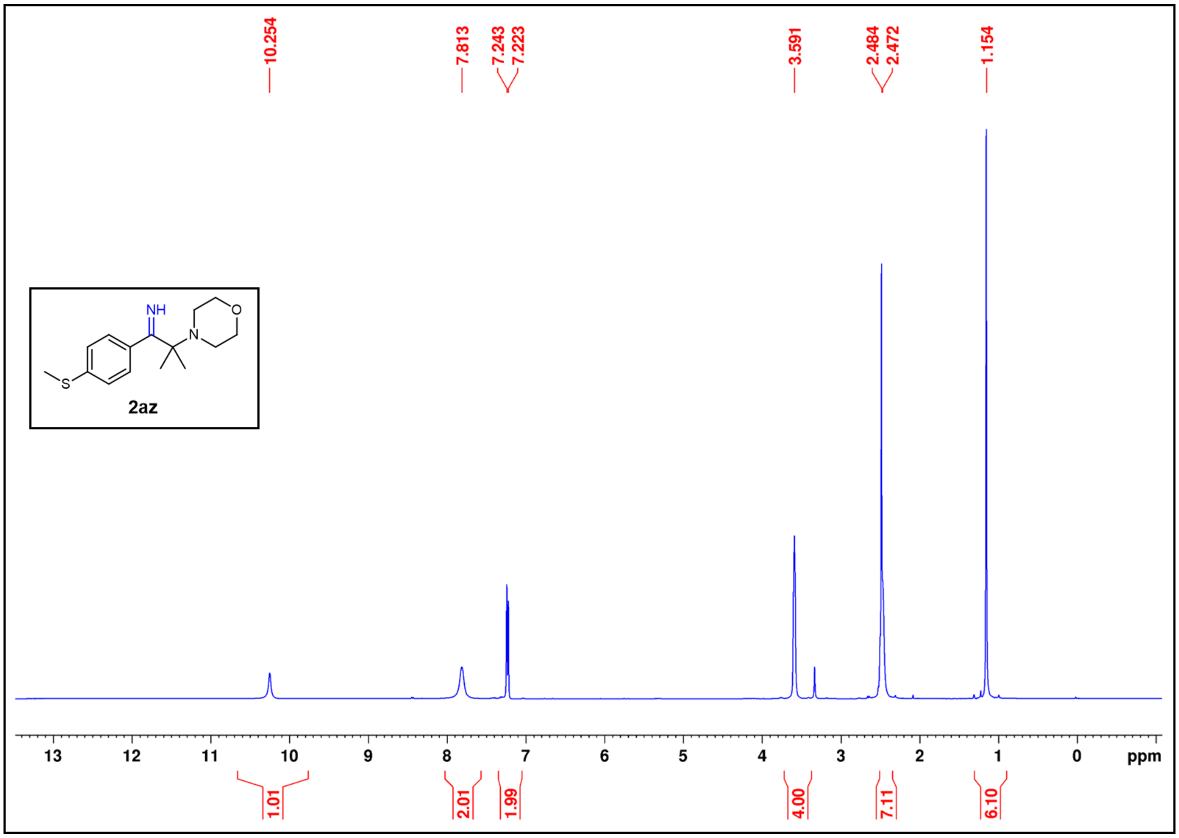

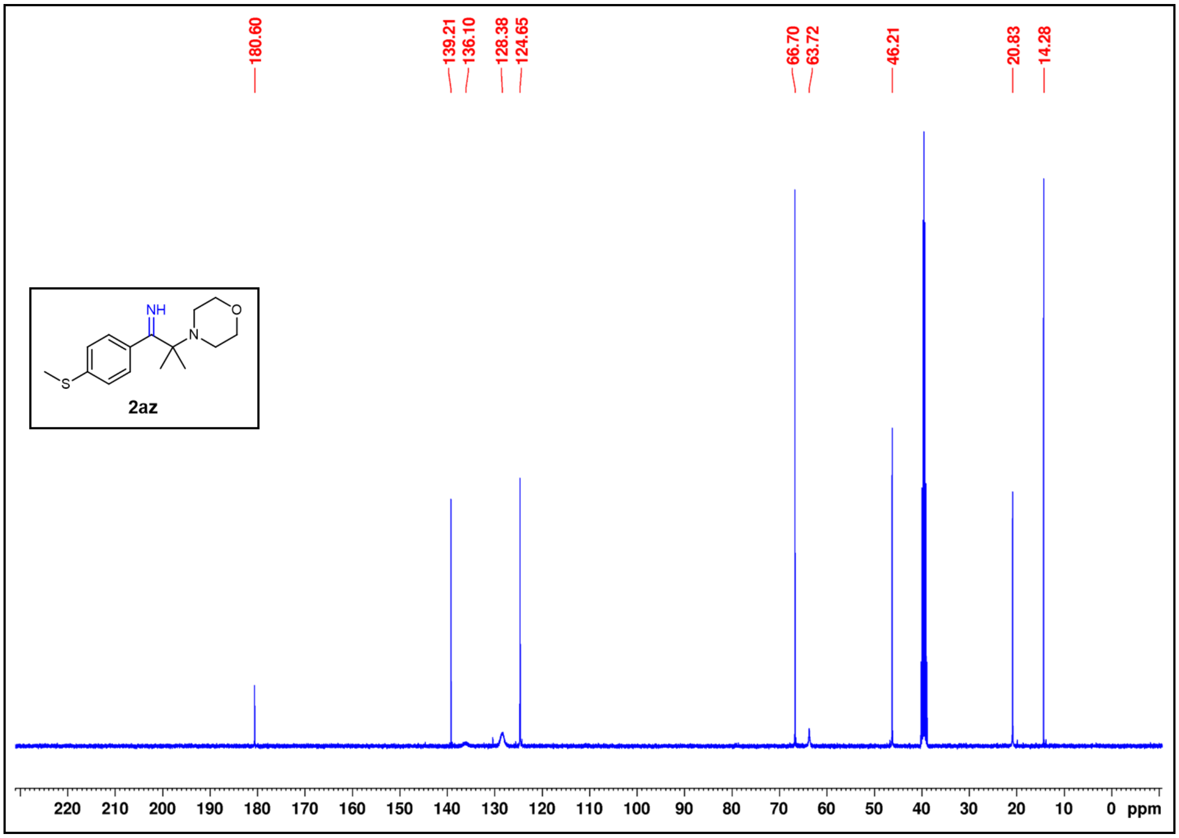

FT-IR (ATR, neat) and HRMS (ESI-positive) spectra for **2az**

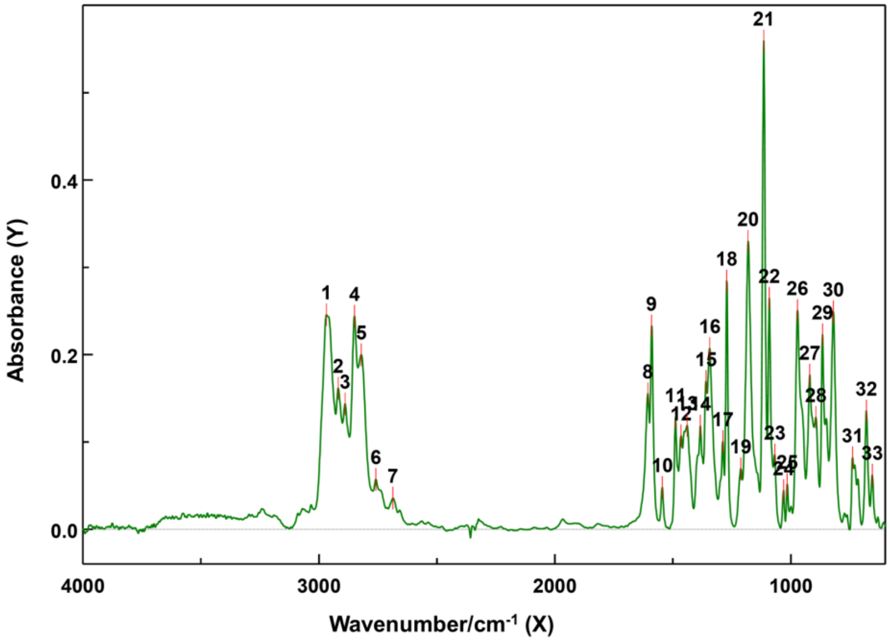

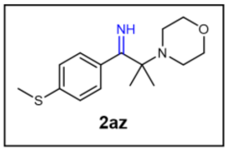

No.	X	Y	No.	X	Y	No.	X	Y
1	2967.9	0.245	12	1465.6	0.107	23	1068	0.085
2	2917.8	0.161	13	1439.6	0.119	24	1031	0.044
3	2888.8	0.143	14	1383.7	0.118	25	1015	0.051
4	2849.3	0.244	15	1359.6	0.169	26	971.9	0.25
5	2820.4	0.2	16	1344.1	0.207	27	919.9	0.176
6	2758.7	0.057	17	1288.2	0.1	28	893.8	0.128
7	2686.4	0.035	18	1271.8	0.284	29	865.9	0.223
8	1606.4	0.155	19	1212	0.069	30	819.6	0.249
9	1590	0.233	20	1181.2	0.329	31	738.6	0.082
10	1544.7	0.048	21	1114.7	0.559	32	679.8	0.135
11	1488.8	0.128	22	1091.5	0.264	33	654.7	0.062

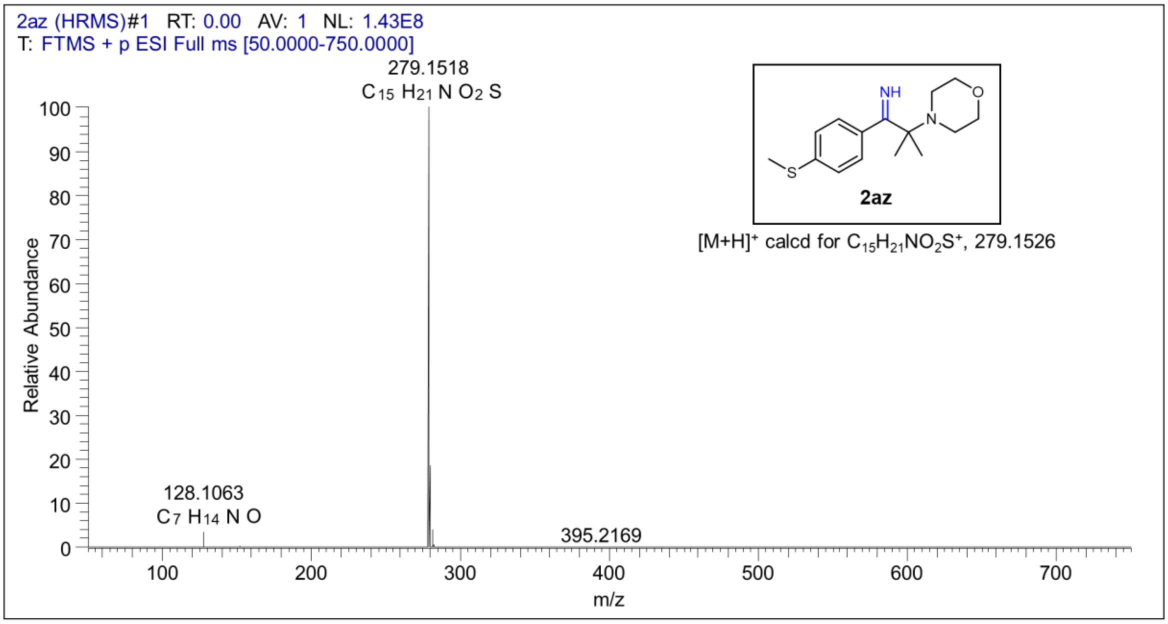

^{1}H NMR (400 MHz, DMSO-d_6) and ^{13}C NMR (100 MHz, DMSO-d_6) spectra for **2ba**·HCl

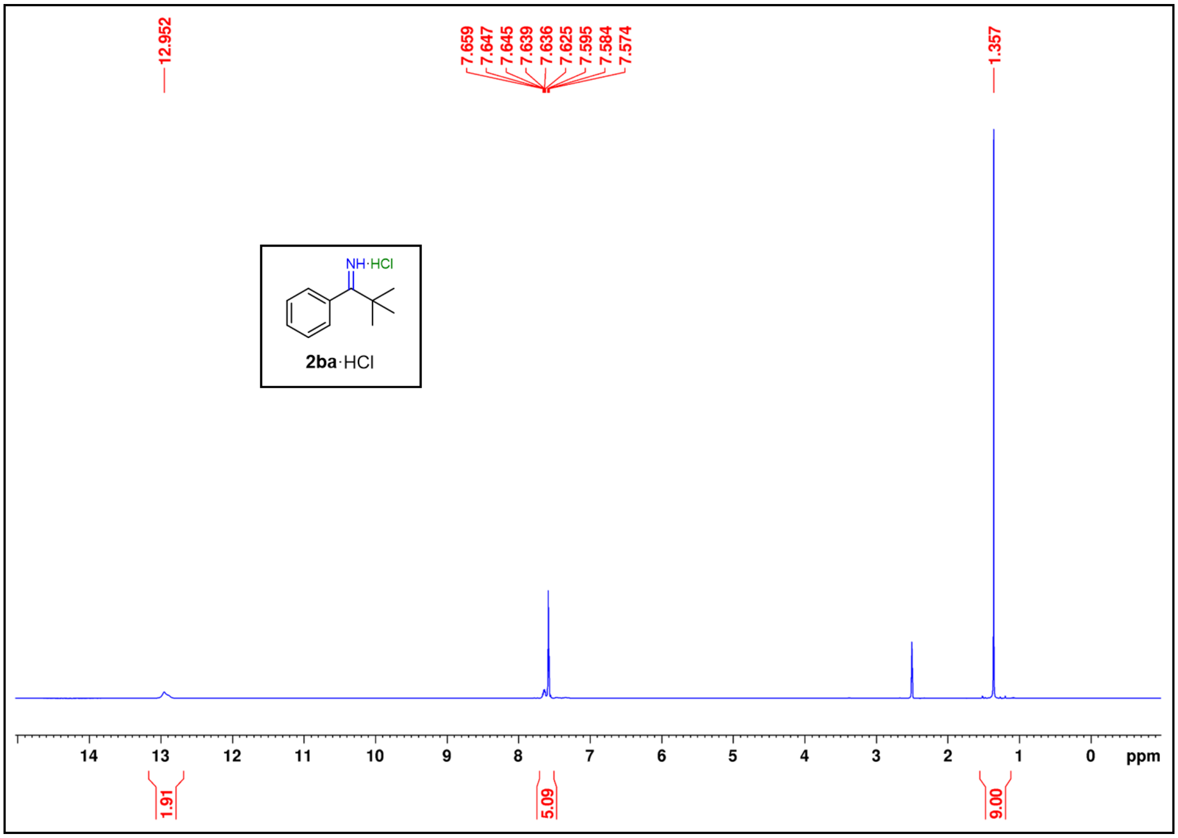

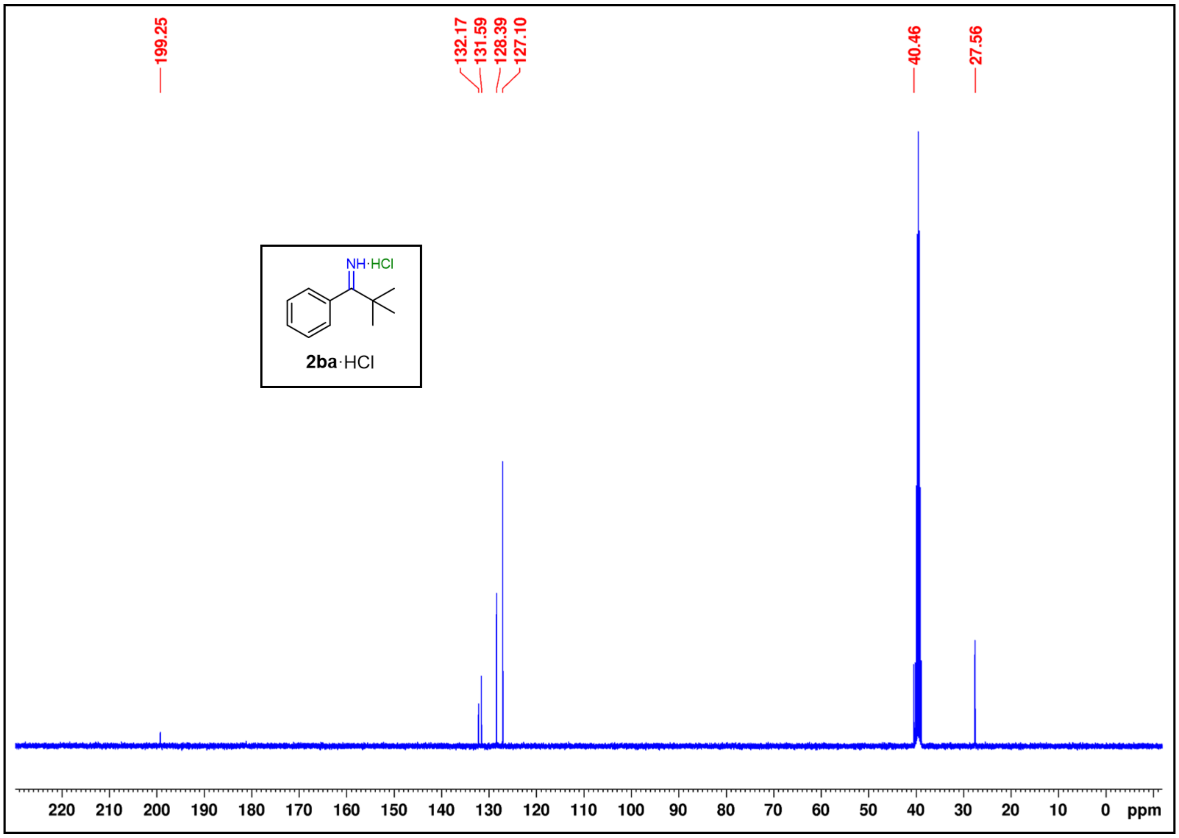

FT-IR (ATR, neat) and HRMS (ESI-positive) spectra for **2ba**·HCl

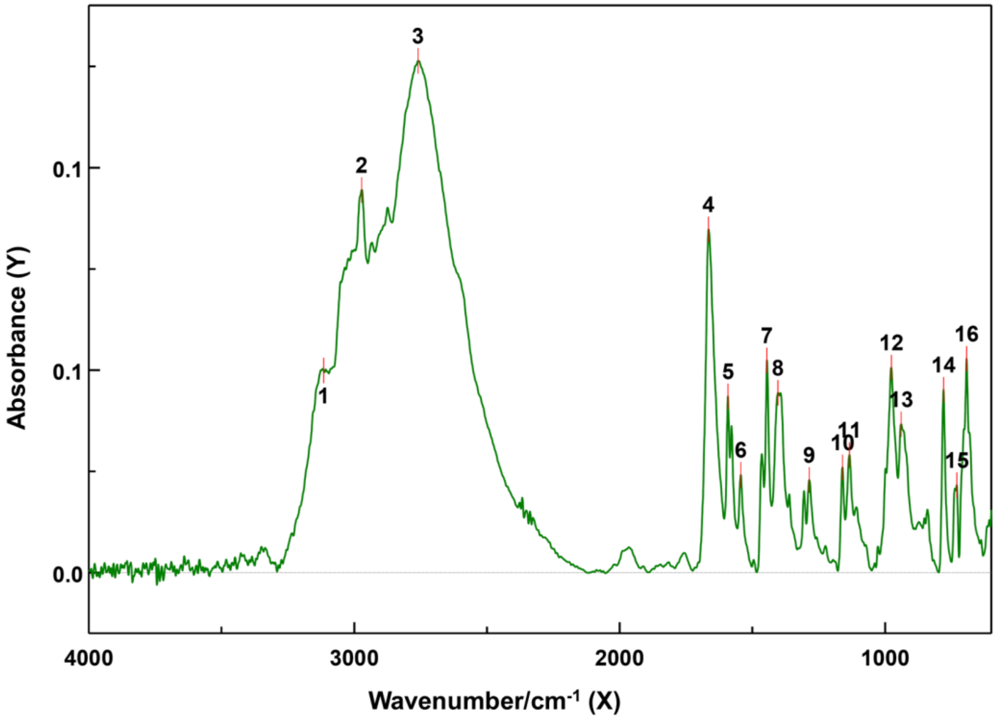

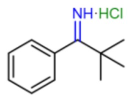

2ba·HCl

No.	X	Y	No.	X	Y	No.	X	Y
1	3115.4	0.05	7	1445.4	0.052	13	940.1	0.037
2	2971.8	0.094	8	1403.9	0.044	14	780.1	0.045
3	2759.6	0.126	9	1285.3	0.023	15	729.9	0.022
4	1665.2	0.085	10	1160.9	0.026	16	693.3	0.053
5	1591.9	0.043	11	1134.9	0.029			
6	1543.7	0.024	12	976.8	0.051			

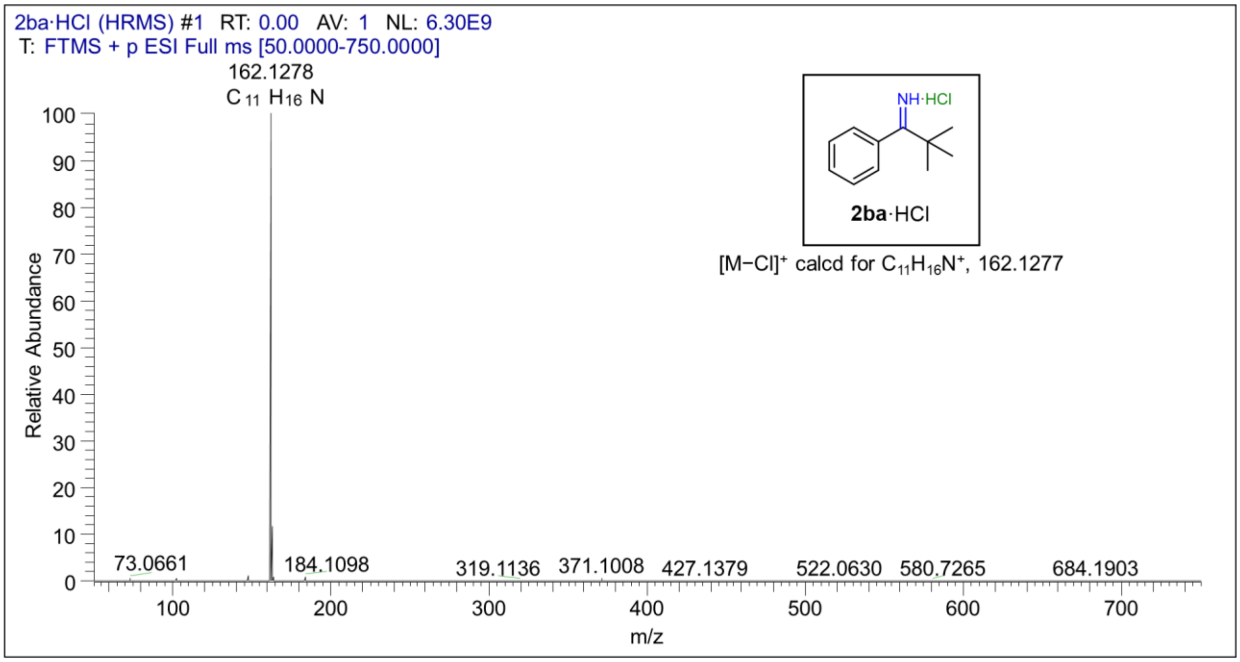

^1H NMR (400 MHz, $CDCl_3$) and ^{13}C NMR (100 MHz, $CDCl_3$) spectra for **2bb**

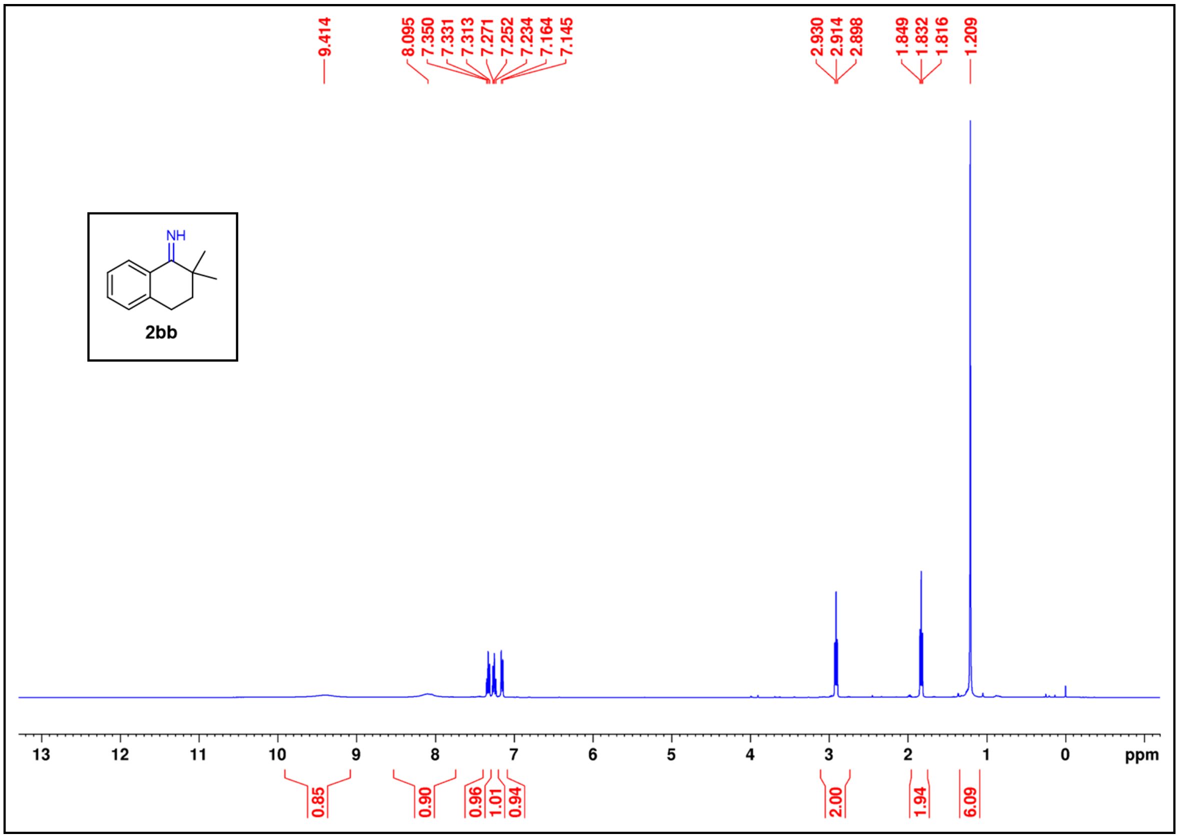

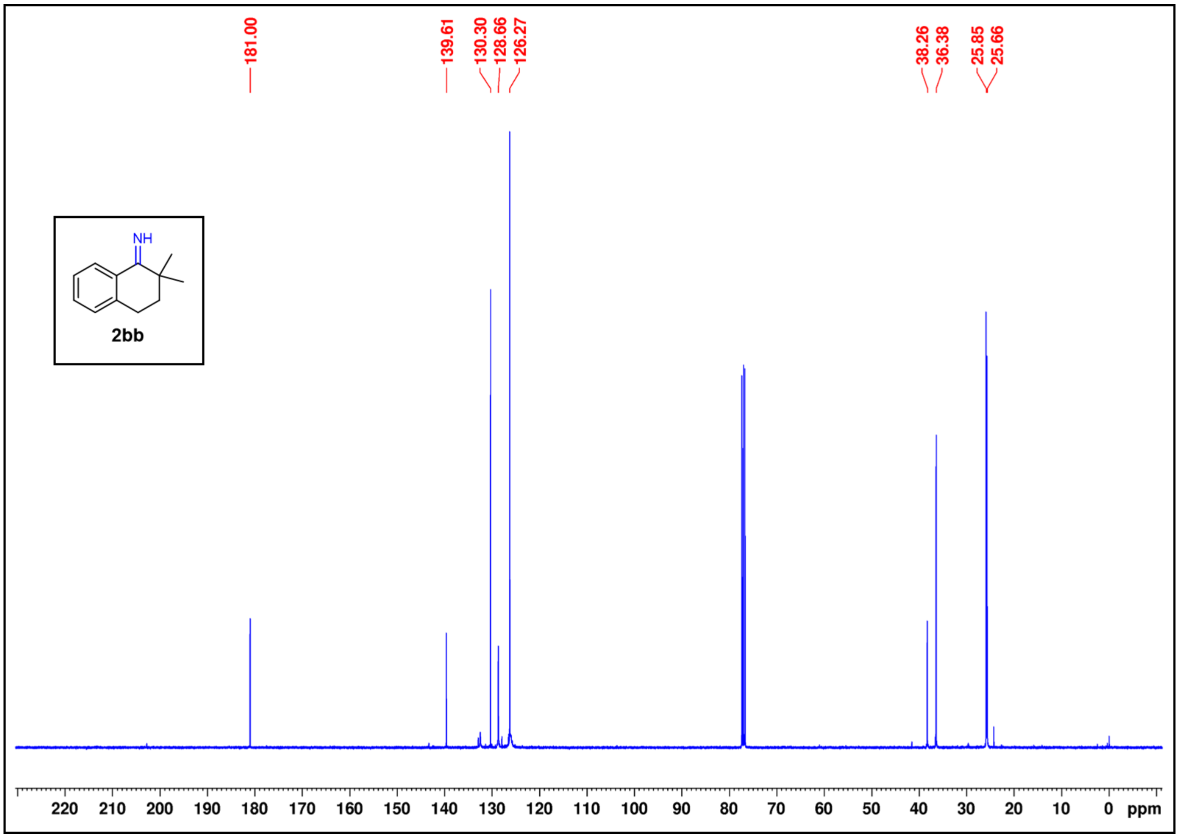

FT-IR (ATR, neat) and HRMS (ESI-positive) spectra for **2bb**

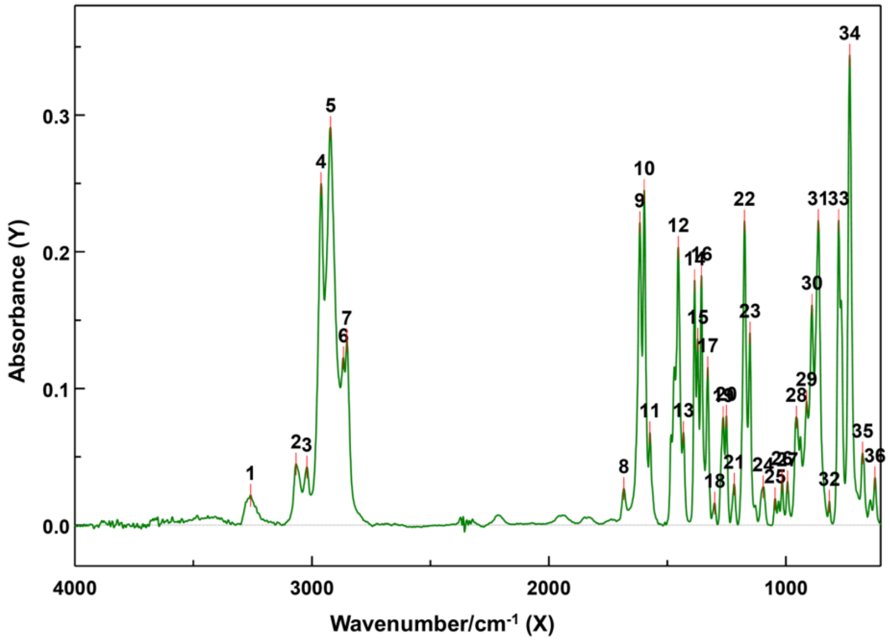

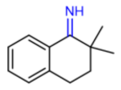

2bb

No.	X	Y	No.	X	Y	No.	X	Y
1	3259.1	0.022	13	1431.9	0.068	25	1045	0.019
2	3067.2	0.045	14	1385.6	0.179	26	1016	0.033
3	3020.9	0.042	15	1372.1	0.136	27	993.2	0.032
4	2961.2	0.25	16	1356.7	0.182	28	955.6	0.079
5	2921.6	0.291	17	1329.7	0.115	29	912.2	0.09
6	2867.6	0.122	18	1300.8	0.016	30	890	0.161
7	2852.2	0.135	19	1265.1	0.079	31	863	0.223
8	1683.6	0.027	20	1251.6	0.08	32	816.7	0.017
9	1616.1	0.221	21	1217.8	0.03	33	777.2	0.223
10	1597.7	0.245	22	1174.4	0.222	34	730.9	0.344
11	1573.6	0.068	23	1151.3	0.14	35	676.9	0.053
12	1454.1	0.203	24	1095.4	0.028	36	623.9	0.035

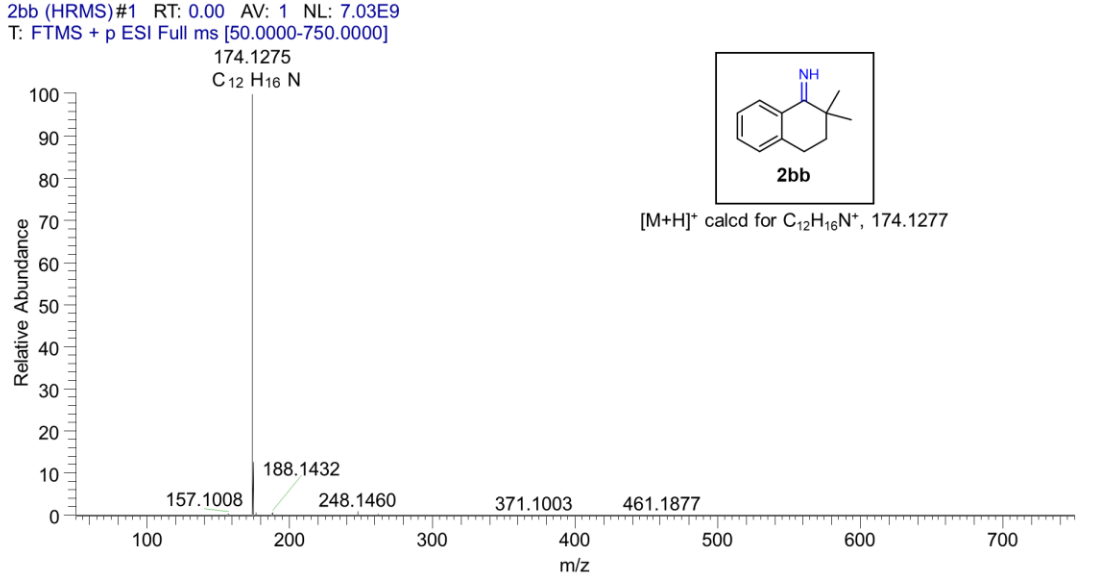

[M+H]$^+$ calcd for $C_{12}H_{16}N^+$, 174.1277

^{1}H NMR (400 MHz, DMSO-d_6) and ^{13}C NMR (100 MHz, DMSO-d_6) spectra for **2bc**·HCl

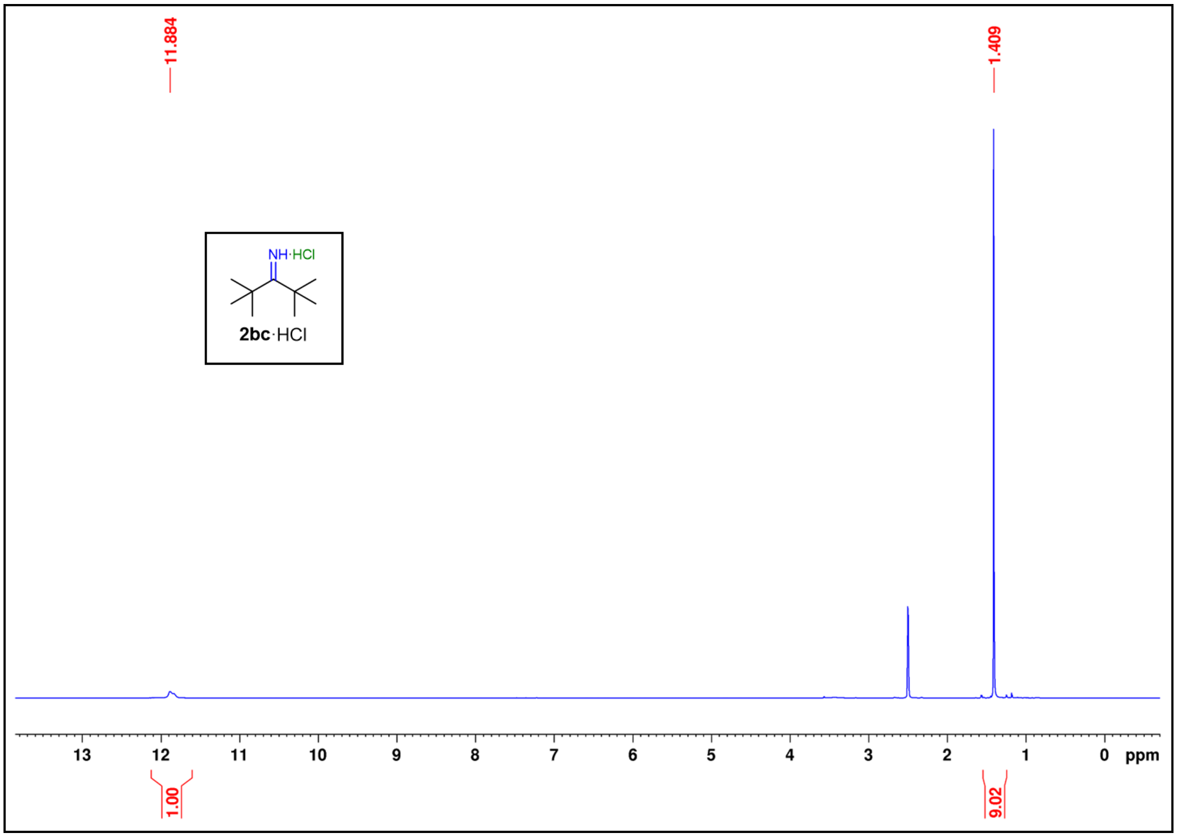

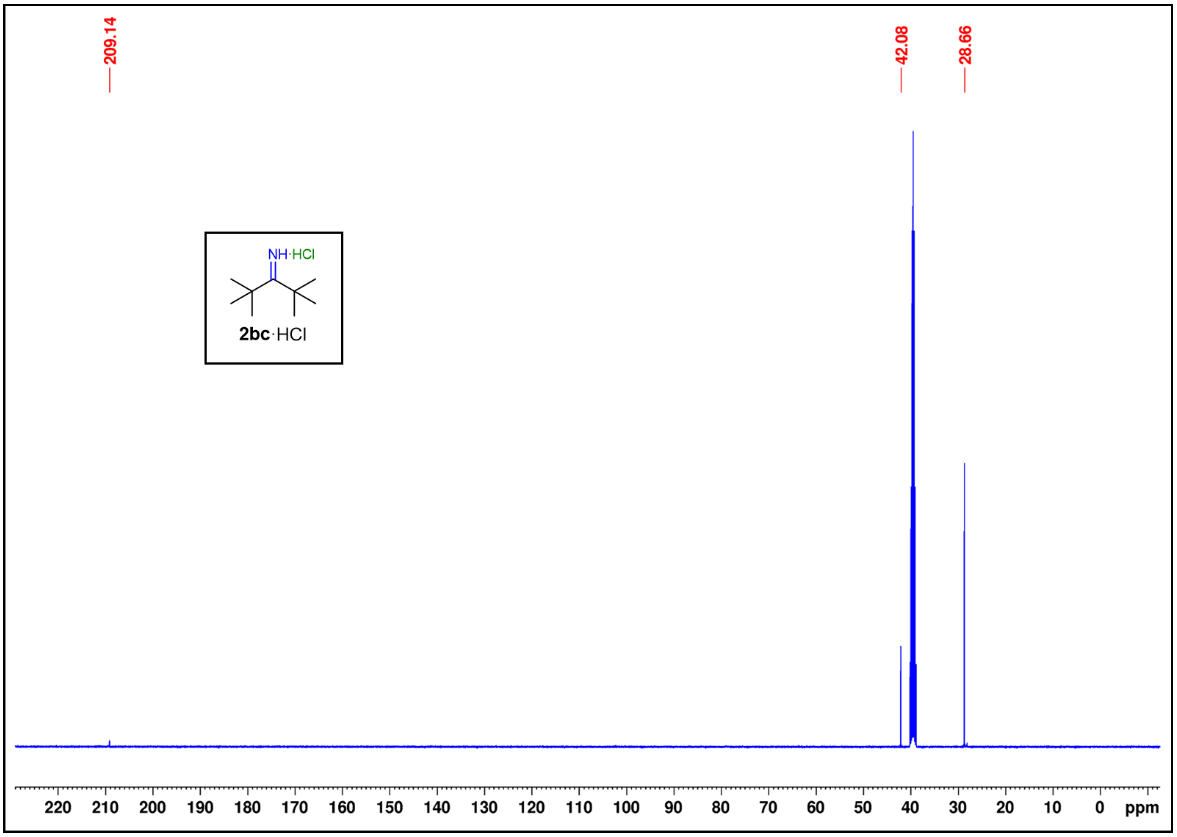

FT-IR (ATR, neat) and HRMS (ESI-positive) spectra for **2bc**·HCl

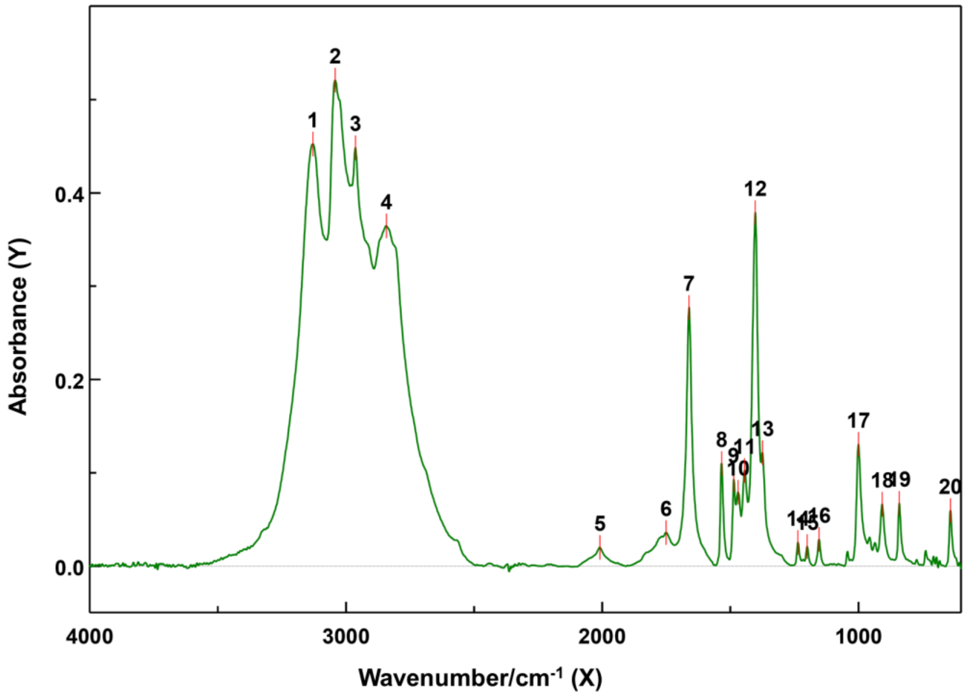

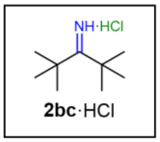

No.	X	Y	No.	X	Y	No.	X	Y
1	3128.9	0.452	8	1534.1	0.11	15	1201	0.021
2	3042.2	0.52	9	1486.8	0.092	16	1154	0.028
3	2963.1	0.448	10	1469.5	0.079	17	999.9	0.13
4	2842.6	0.364	11	1444.4	0.102	18	907.3	0.066
5	2009.5	0.02	12	1403	0.379	19	840.8	0.067
6	1751	0.036	13	1375	0.121	20	641.2	0.059
7	1661.4	0.277	14	1236.1	0.025			

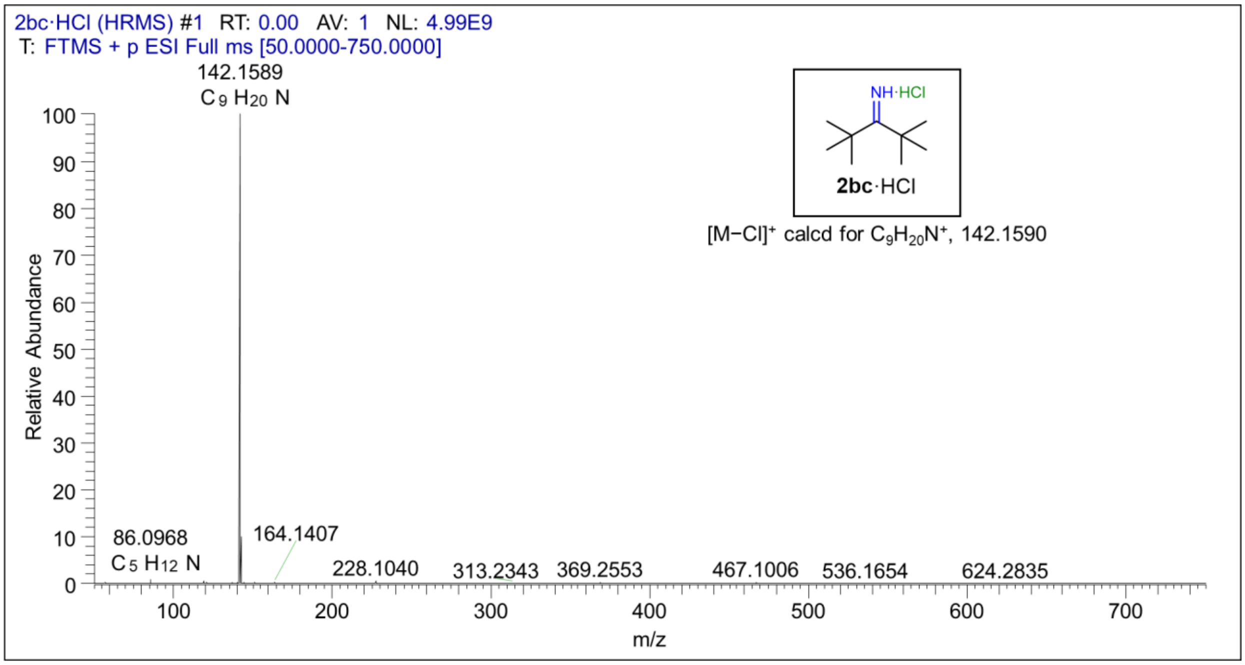

^{1}H NMR (400 MHz, DMSO-d_6) and ^{13}C NMR (100 MHz, DMSO-d_6) spectra for **2bd**·HCl

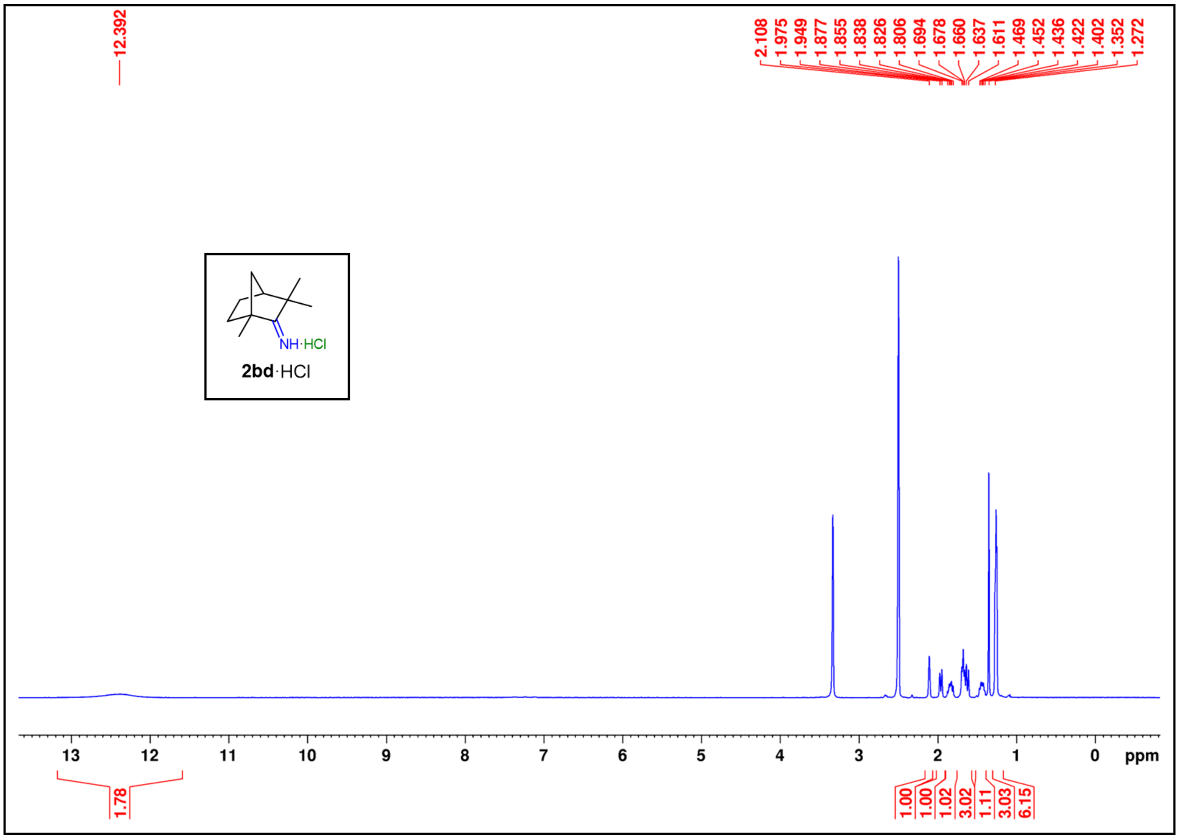

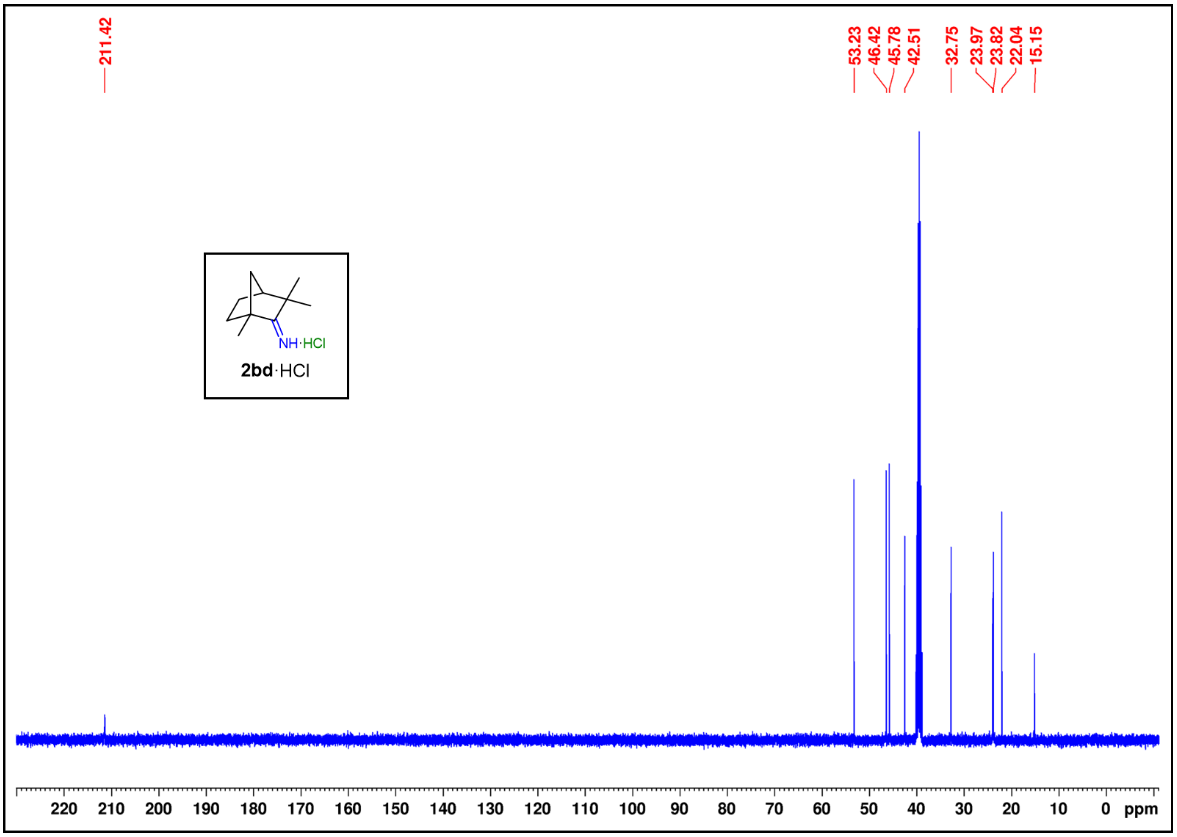

FT-IR (ATR, neat) and HRMS (ESI-positive) spectra for **2bd**·HCl

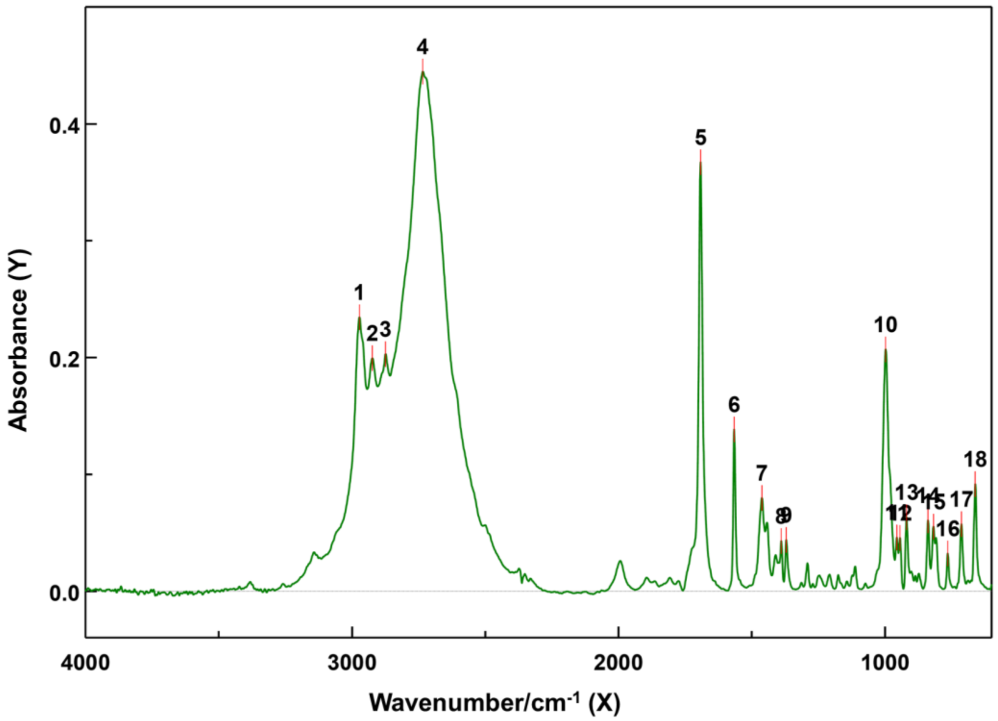

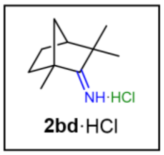

No.	X	Y	No.	X	Y	No.	X	Y
1	2971.8	0.234	7	1461.8	0.08	13	918.9	0.063
2	2923.6	0.199	8	1389.5	0.043	14	838.9	0.061
3	2874.4	0.203	9	1370.2	0.044	15	818.6	0.055
4	2734.6	0.445	10	998	0.207	16	764.6	0.032
5	1692.2	0.367	11	955.6	0.046	17	713.5	0.057
6	1565.9	0.138	12	944	0.045	18	661.5	0.092

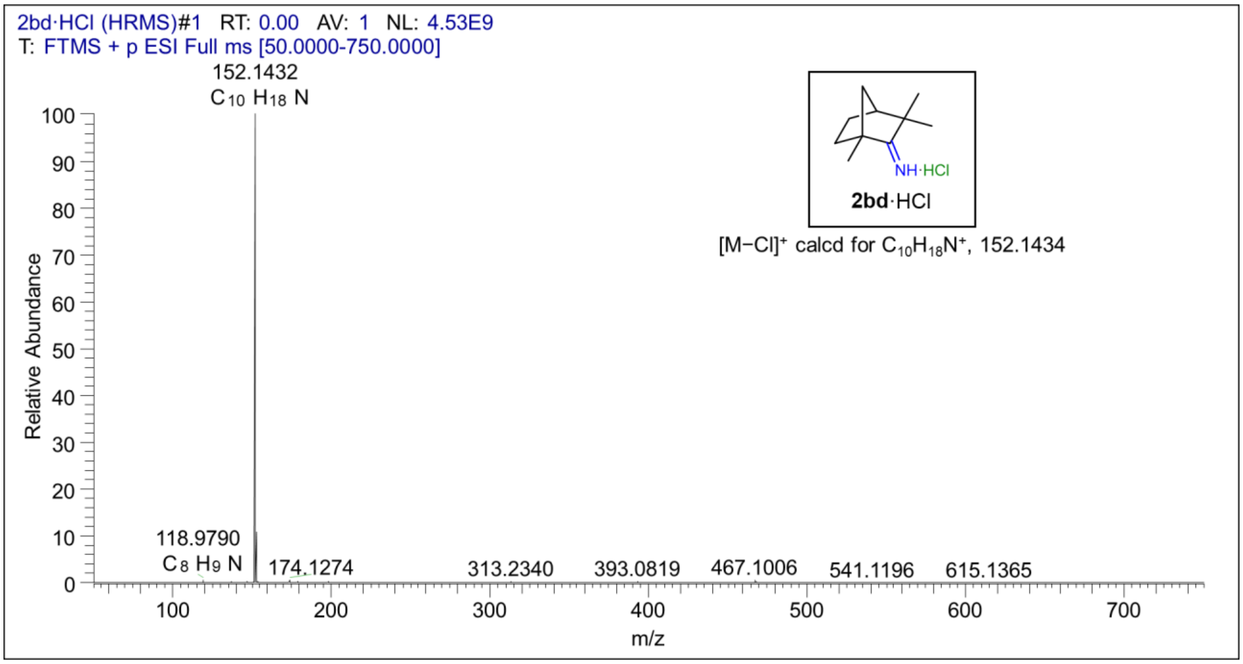

^{1}H NMR (400 MHz, DMSO-d_6) and ^{13}C NMR (100 MHz, DMSO-d_6) spectra for **2be**·HCl

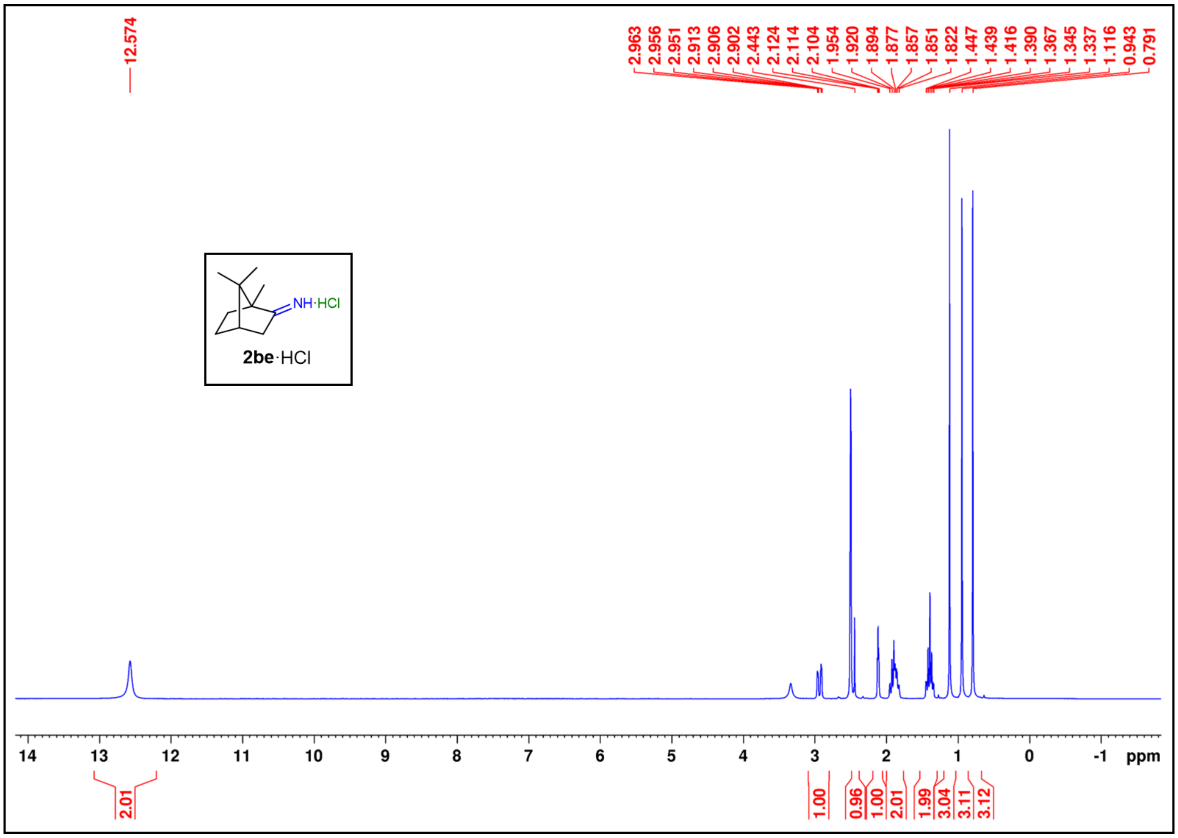

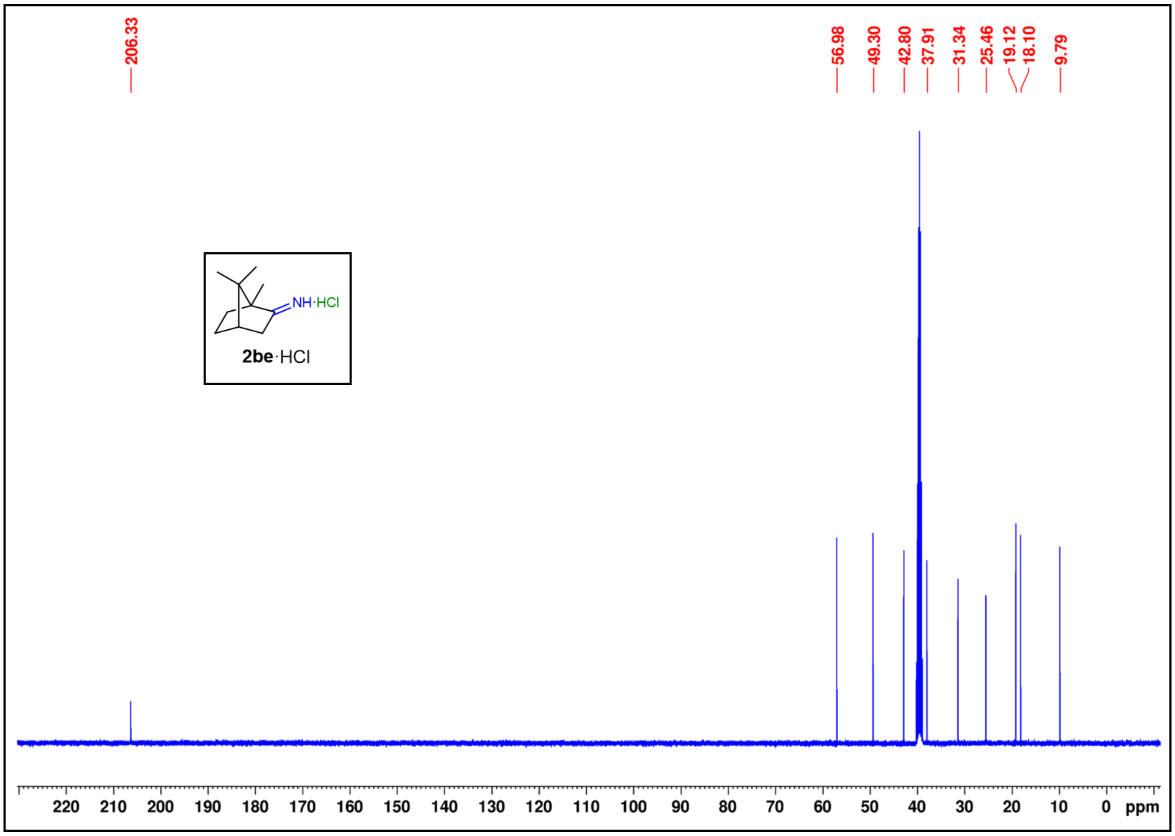

FT-IR (ATR, neat) and HRMS (ESI-positive) spectra for **2be**·HCl

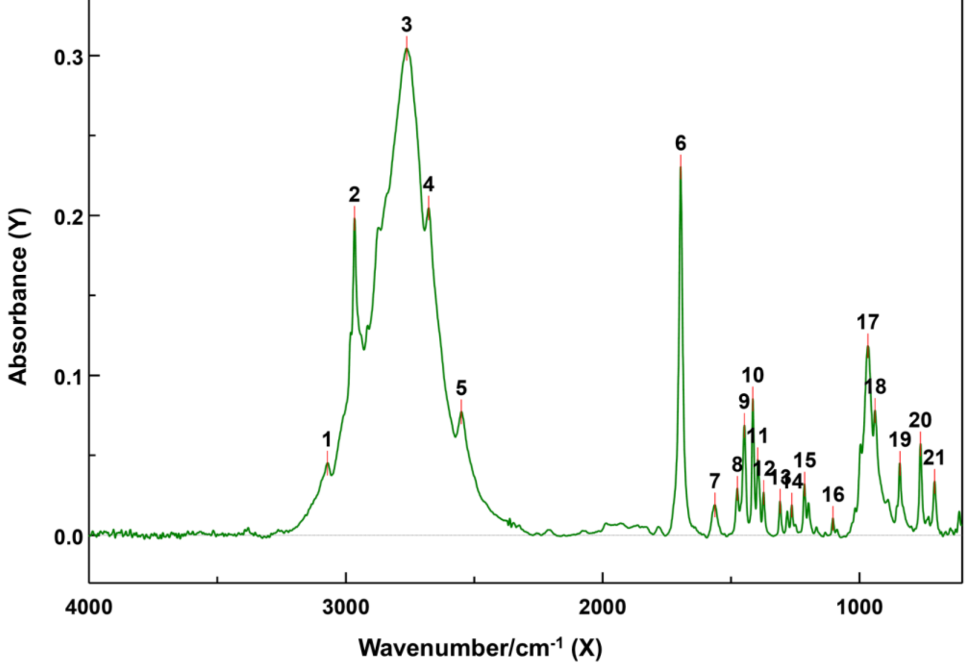

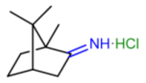

2be·HCl

No.	X	Y	No.	X	Y	No.	X	Y
1	3072	0.045	8	1475.3	0.029	15	1214	0.032
2	2966	0.198	9	1448.3	0.069	16	1103	0.01
3	2763.5	0.304	10	1415.5	0.085	17	967.1	0.118
4	2677.7	0.205	11	1396.2	0.047	18	940.1	0.078
5	2550.4	0.077	12	1373.1	0.027	19	842.7	0.045
6	1696.1	0.23	13	1309.4	0.021	20	762.7	0.057
7	1563	0.019	14	1264.1	0.019	21	707.7	0.034

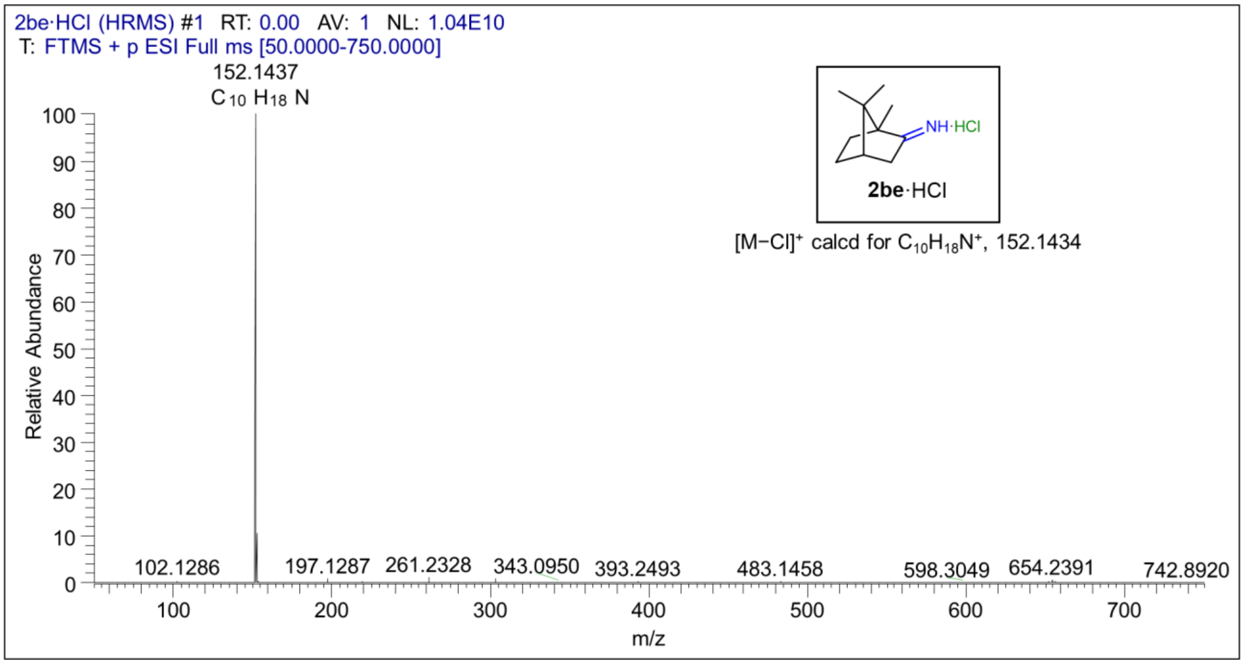

^{1}H NMR (400 MHz, $CDCl_3$) and ^{13}C NMR (100 MHz, $CDCl_3$) spectra for **2bf**

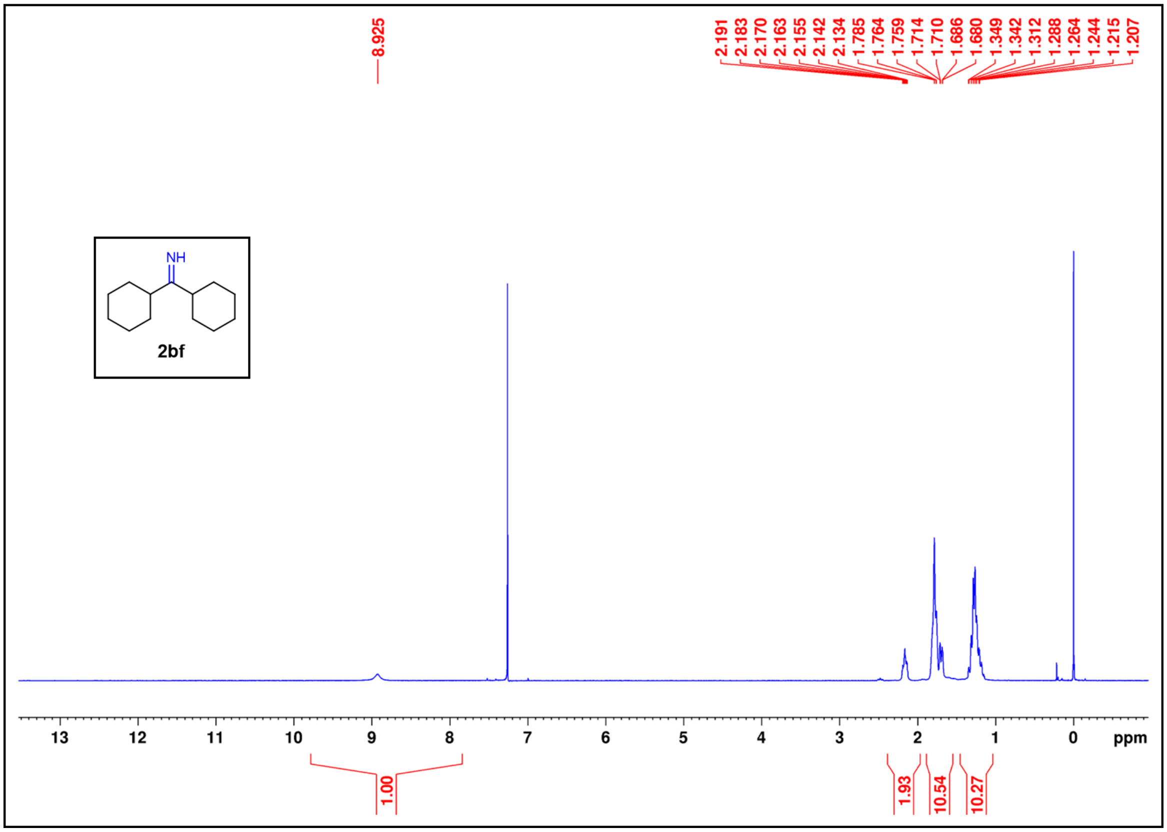

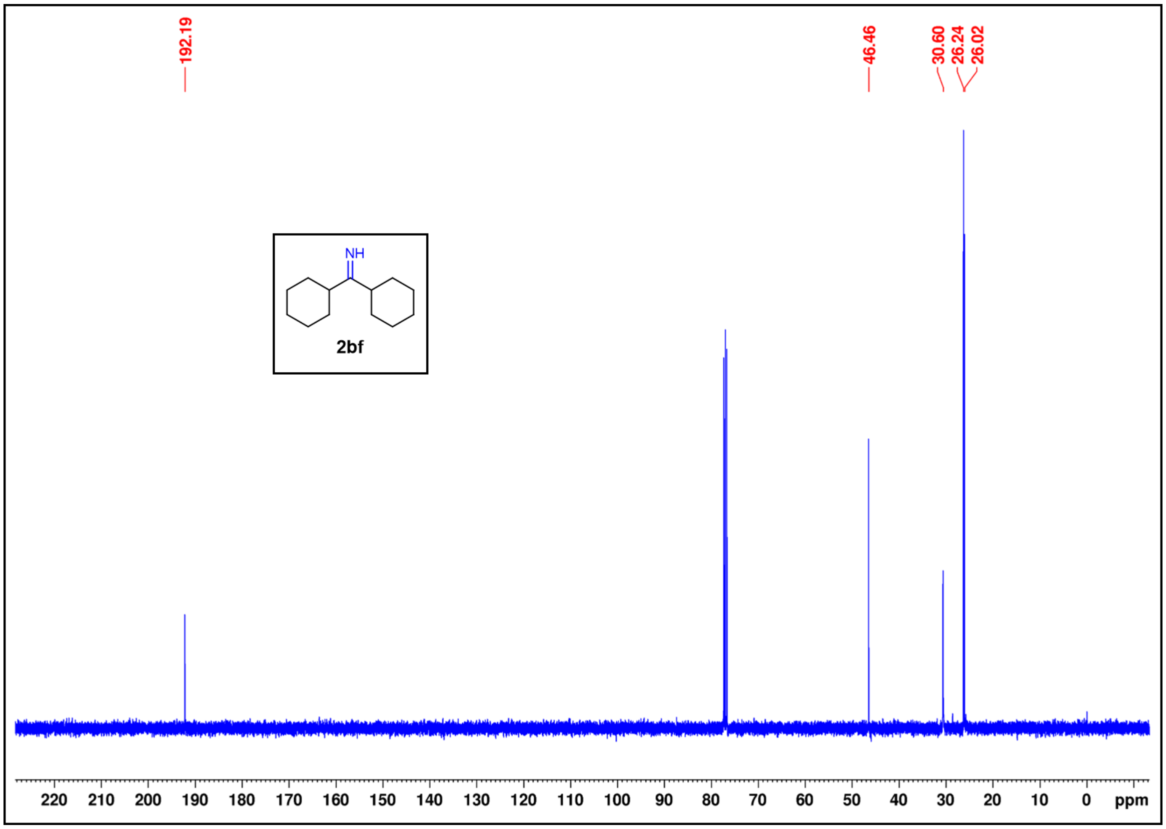

FT-IR (ATR, neat) and HRMS (ESI-positive) spectra for **2bf**

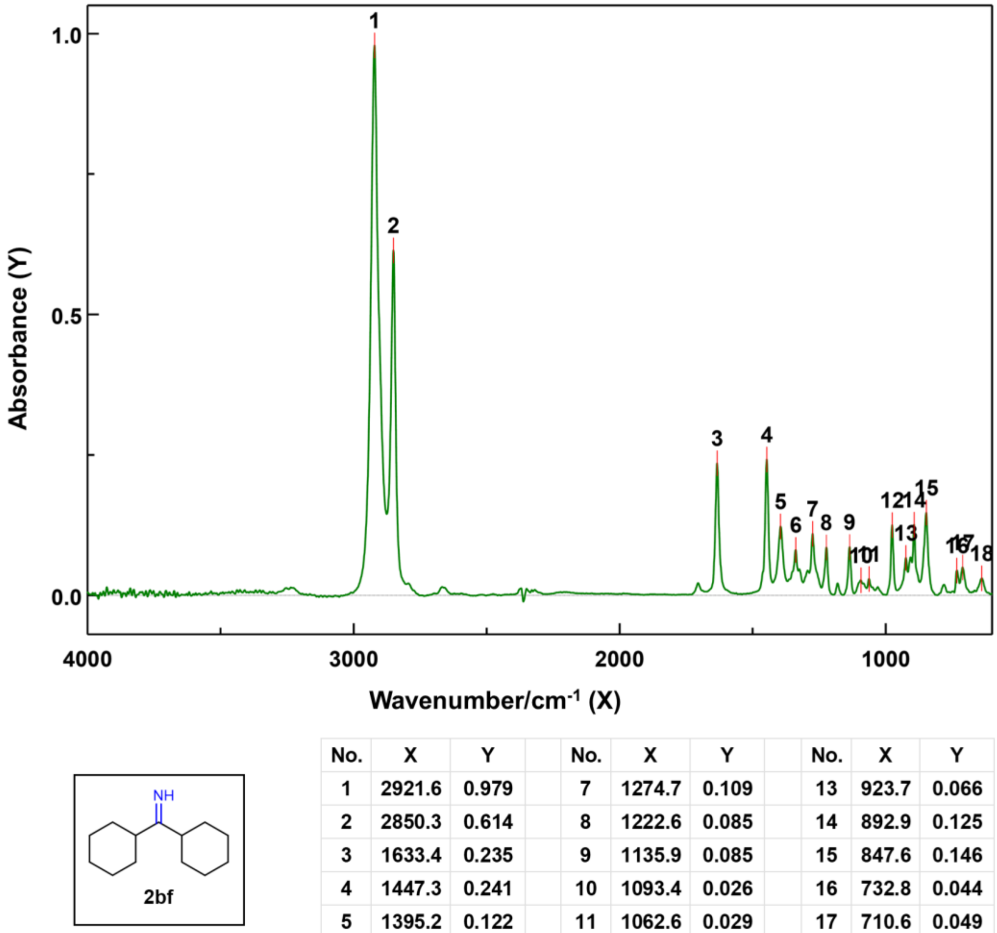

No.	X	Y	No.	X	Y	No.	X	Y
1	2921.6	0.979	7	1274.7	0.109	13	923.7	0.066
2	2850.3	0.614	8	1222.6	0.085	14	892.9	0.125
3	1633.4	0.235	9	1135.9	0.085	15	847.6	0.146
4	1447.3	0.241	10	1093.4	0.026	16	732.8	0.044
5	1395.2	0.122	11	1062.6	0.029	17	710.6	0.049
6	1338.4	0.08	12	975.8	0.124	18	639.3	0.03

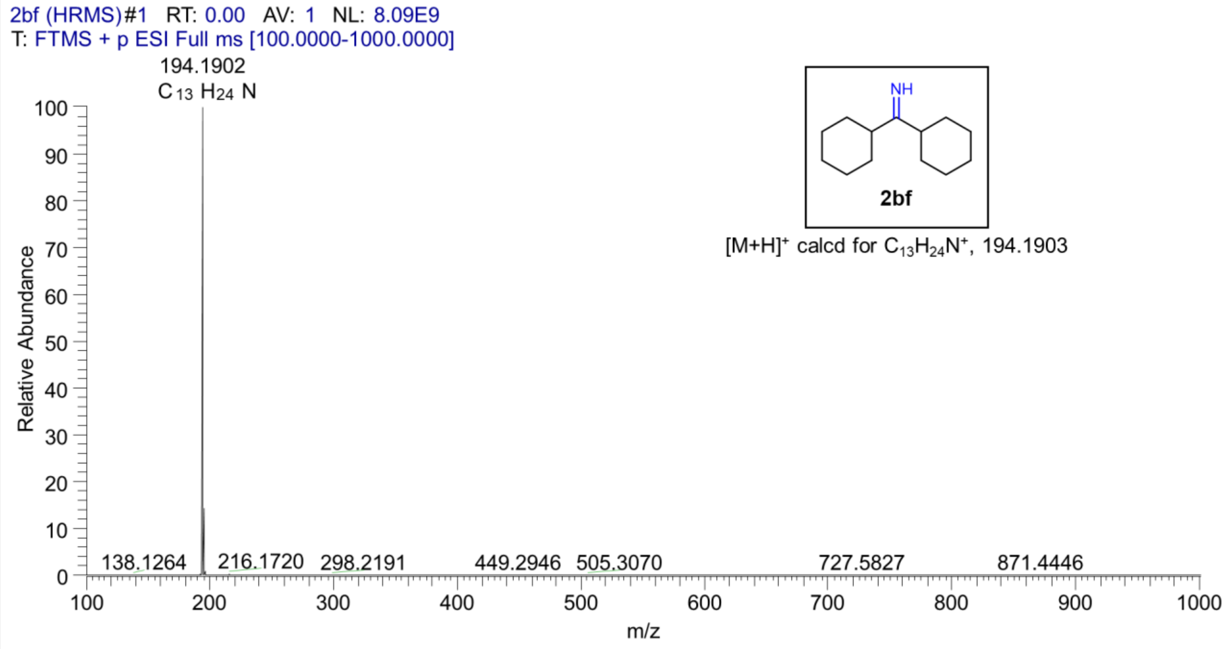

^{1}H NMR (400 MHz, DMSO-d_6) and ^{13}C NMR (100 MHz, DMSO-d_6) spectra for **2bf**·HCl

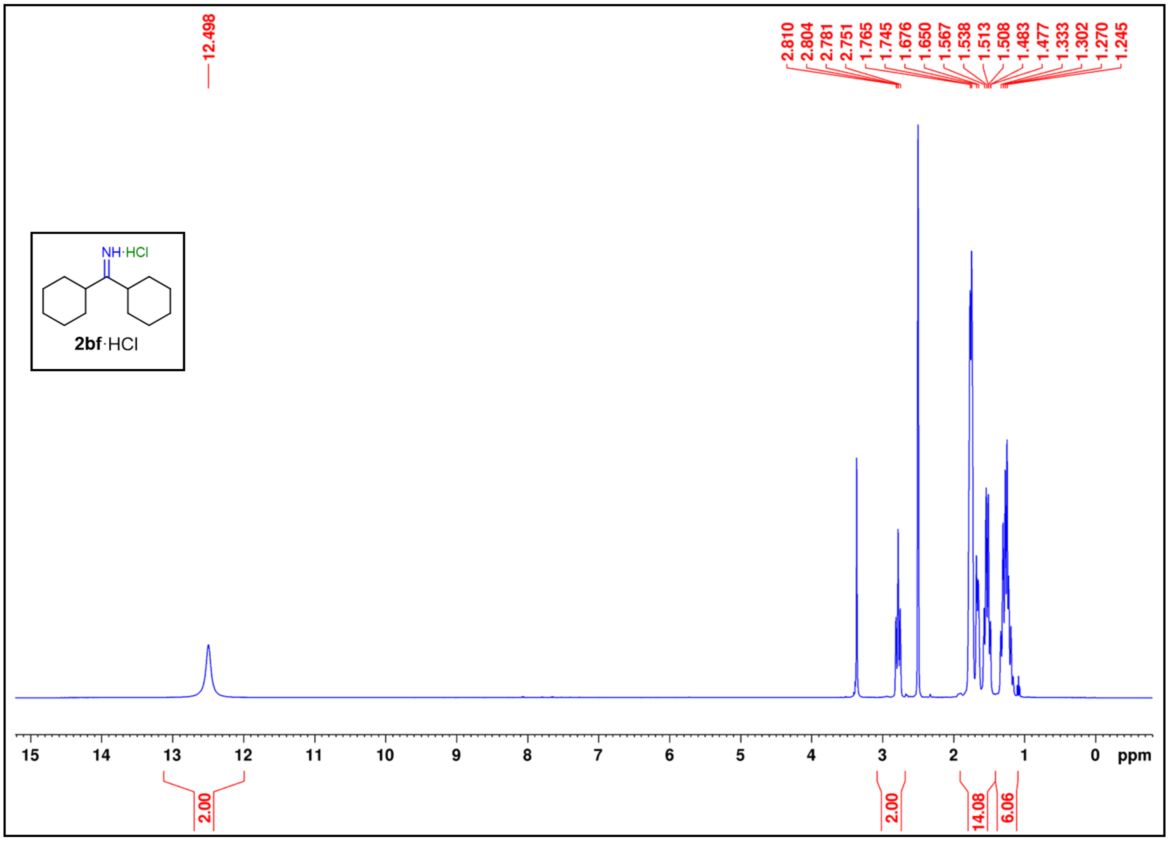

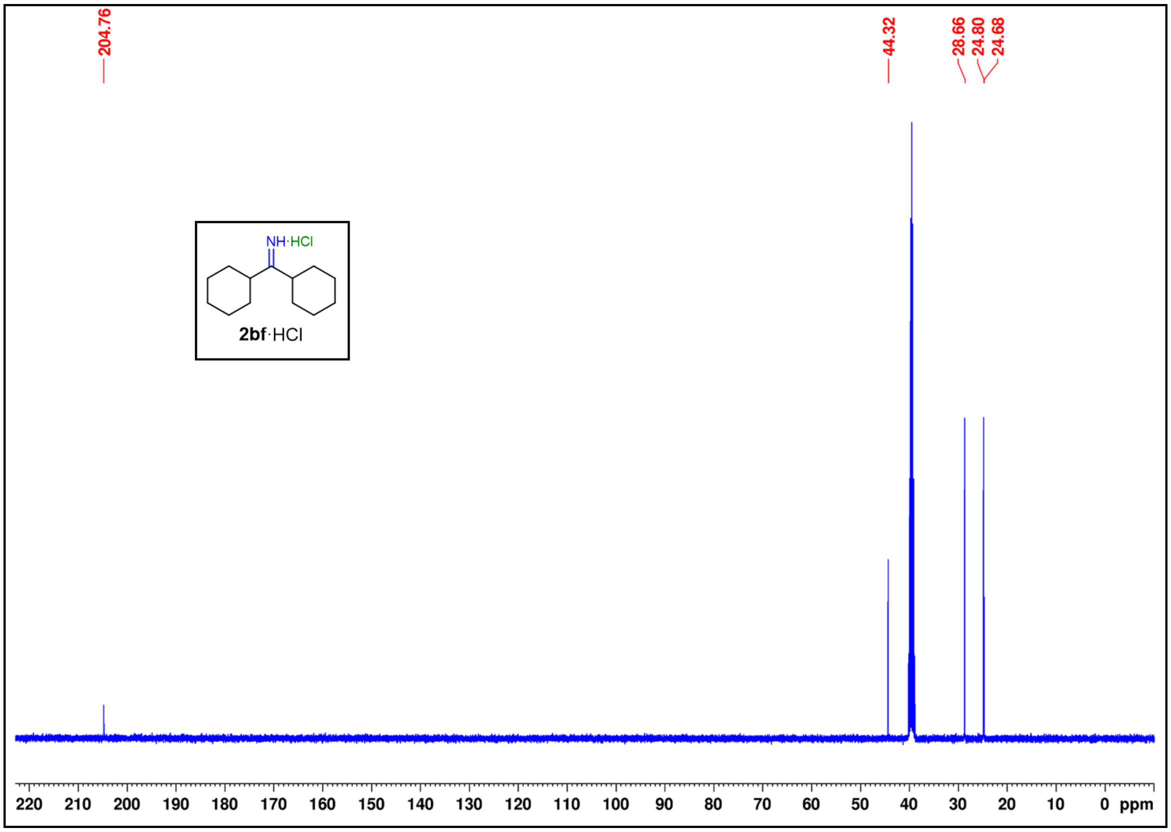

FT-IR (ATR, neat) and HRMS (ESI-positive) spectra for **2bf**·HCl

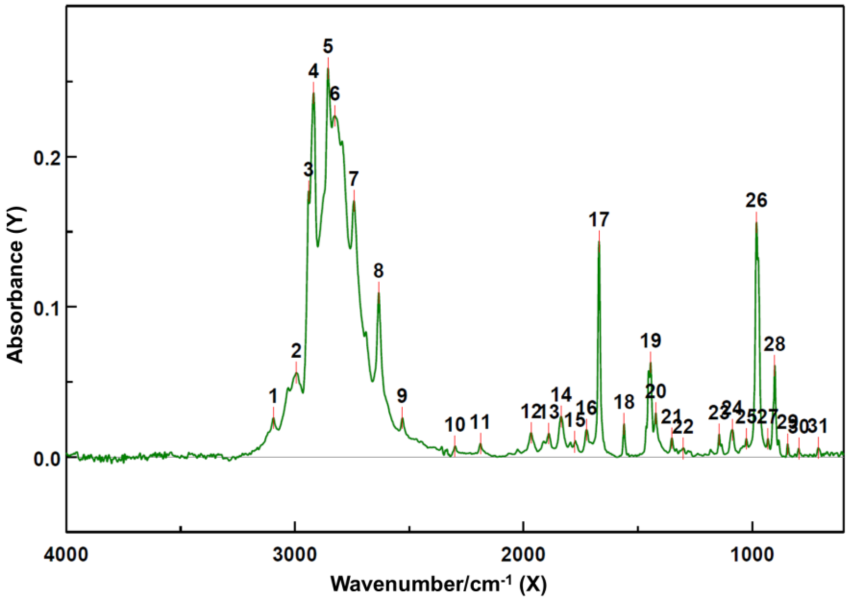

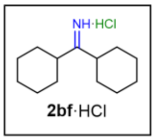

No.	X	Y	No.	X	Y	No.	X	Y
1	3094.2	0.026	2	2993	0.056	3	2940	0.177
4	2917.8	0.242	5	2855.1	0.259	6	2825	0.227
7	2742.3	0.17	8	2633.3	0.109	9	2530	0.026
10	2299.7	0.007	11	2188.8	0.009	12	1967	0.016
13	1889.9	0.016	14	1835.9	0.027	15	1776	0.01
16	1725	0.018	17	1670.1	0.143	18	1560	0.022
19	1444.4	0.063	20	1421.3	0.029	21	1351	0.013
22	1302.7	0.006	23	1144.5	0.015	24	1087	0.018
25	1025.9	0.012	26	980.6	0.156	27	931.5	0.012
28	901.6	0.061	29	844.7	0.009	30	796.5	0.006
31	711.6	0.006						

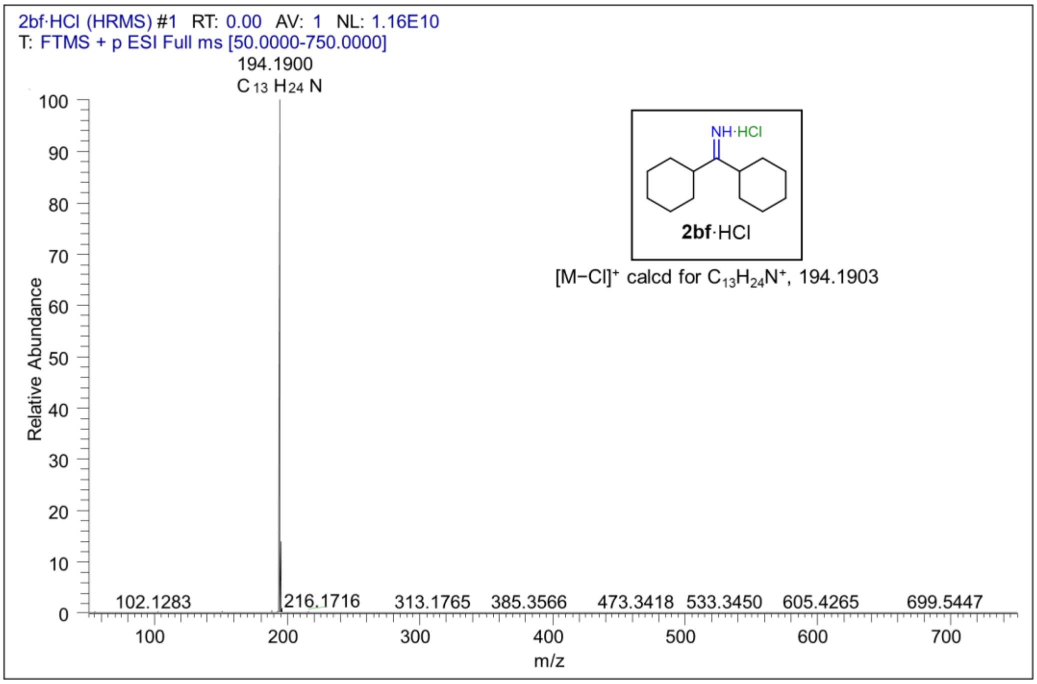

^{1}H NMR (400 MHz, DMSO-d_6) and ^{13}C NMR (100 MHz, DMSO-d_6) spectra for **2bg**·HCl

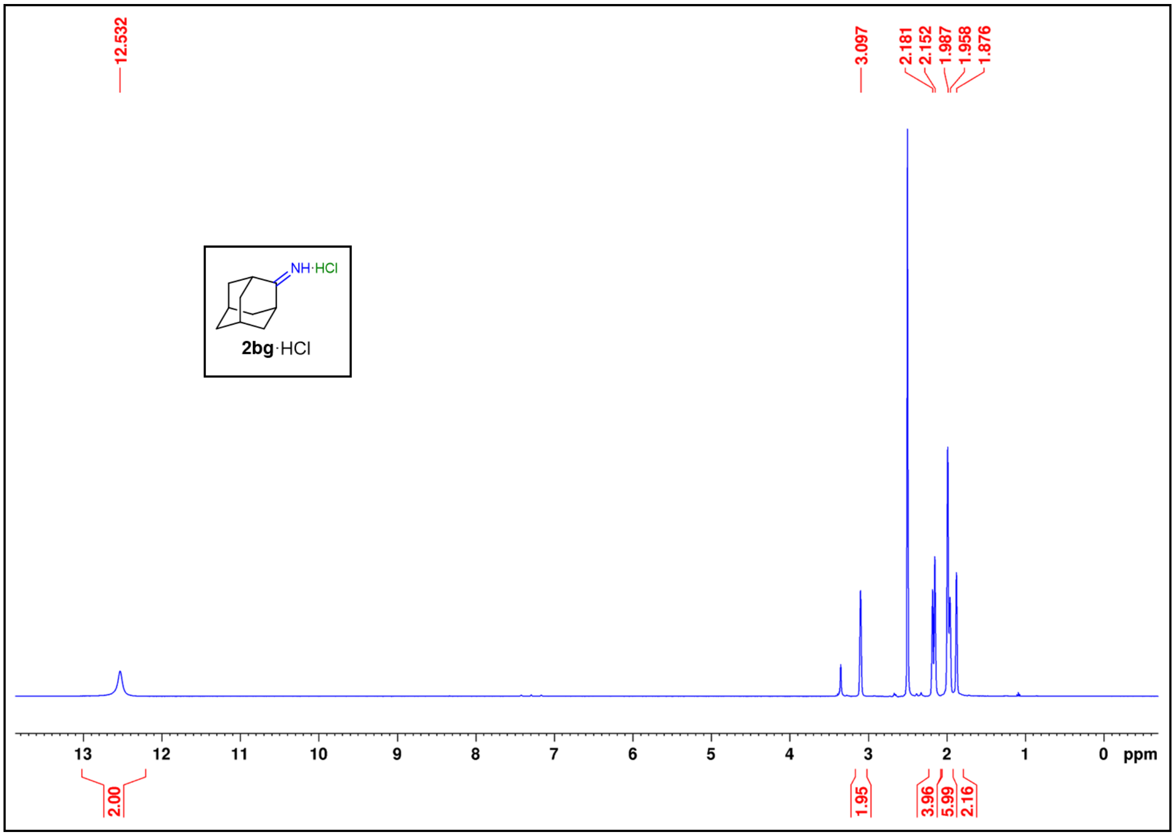

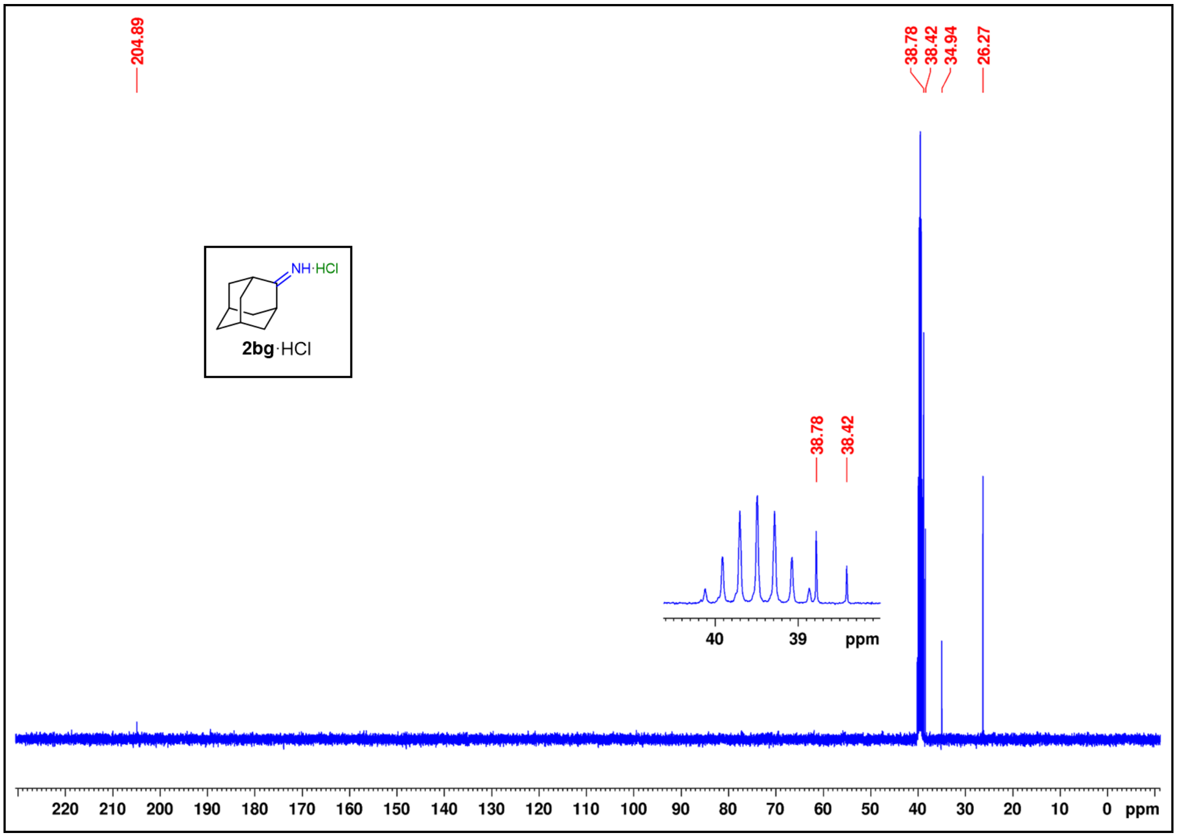

FT-IR (ATR, neat) and HRMS (ESI-positive) spectra for **2bg**·HCl

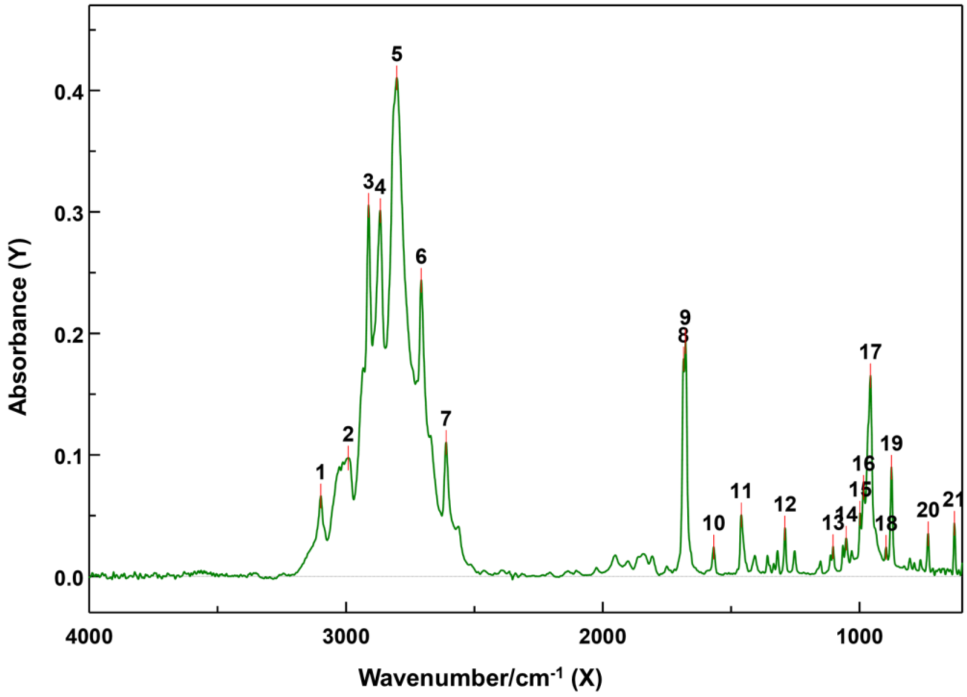

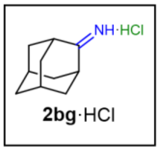

No.	X	Y	No.	X	Y	No.	X	Y
1	3098.1	0.066	8	1685.5	0.179	15	998	0.052
2	2991.1	0.097	9	1677.8	0.193	16	984.5	0.073
3	2912	0.305	10	1567.8	0.024	17	957.5	0.165
4	2866.7	0.301	11	1460.8	0.05	18	896.7	0.024
5	2802.1	0.41	12	1290.1	0.04	19	875.5	0.09
6	2707.6	0.244	13	1103.1	0.024	20	732.8	0.035
7	2610.2	0.11	14	1052	0.031	21	630.6	0.043

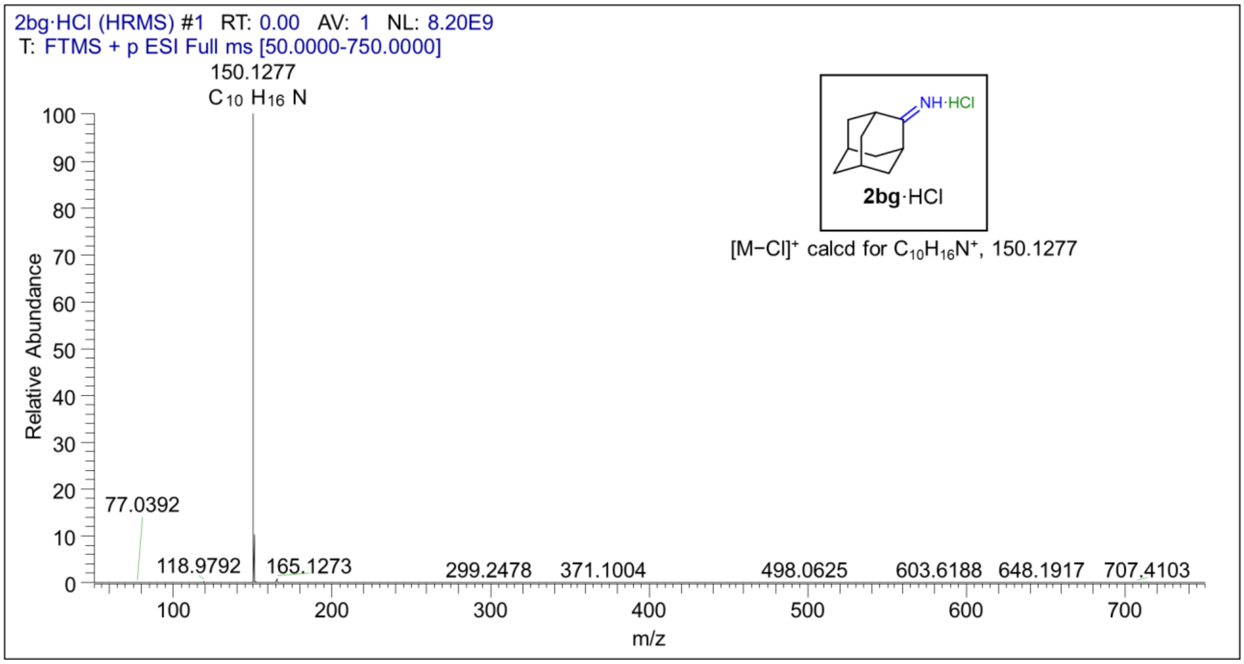

^{1}H NMR (400 MHz, DMSO-d_6) and ^{13}C NMR (100 MHz, DMSO-d_6) spectra for **2bh**·HCl

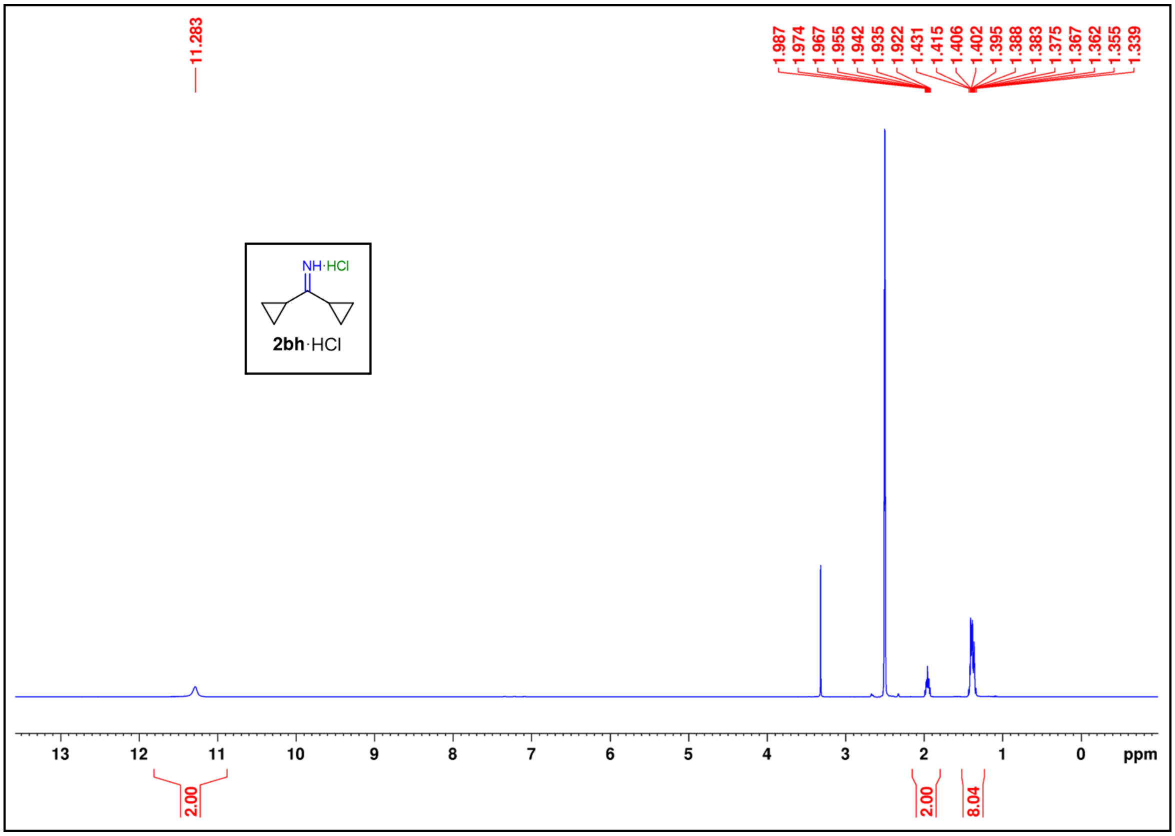

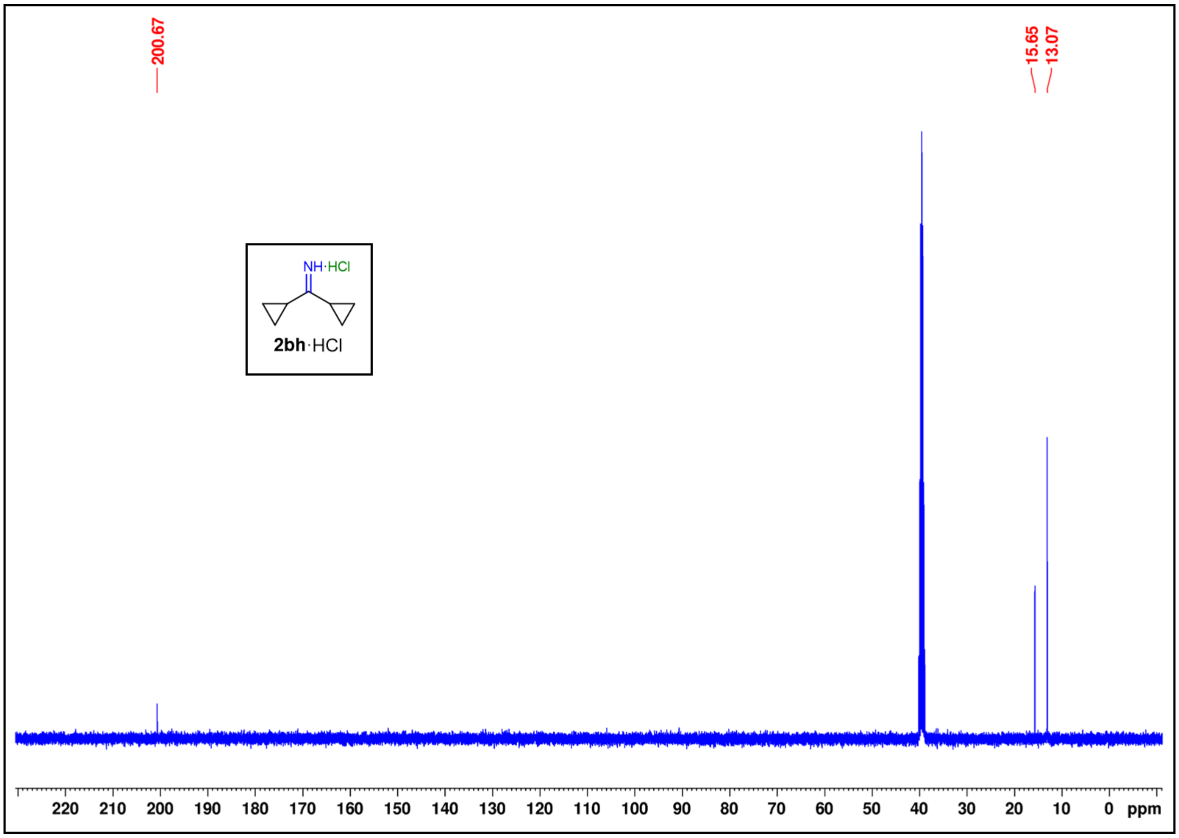

FT-IR (ATR, neat) and HRMS (ESI-positive) spectra for **2bh**·HCl

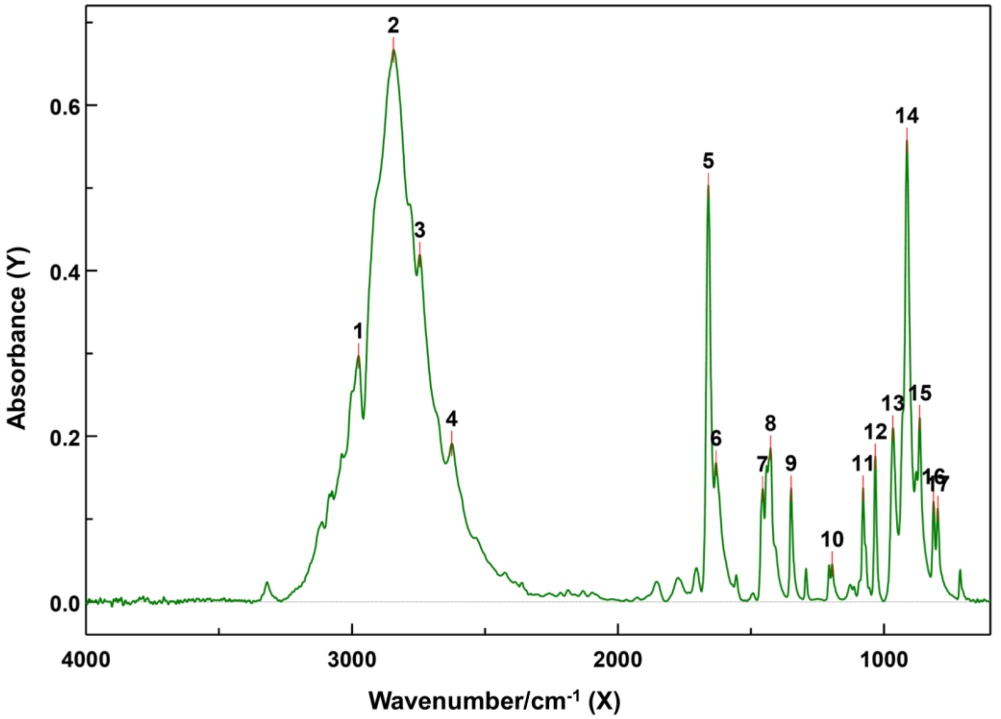

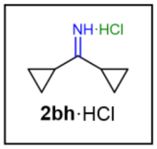

No.	X	Y	No.	X	Y	No.	X	Y
1	2975.6	0.297	7	1456	0.136	13	966.2	0.21
2	2843.5	0.667	8	1426.1	0.186	14	914.1	0.557
3	2745.2	0.419	9	1349	0.137	15	865.9	0.222
4	2624.6	0.191	10	1194.7	0.045	16	813.8	0.121
5	1660.4	0.503	11	1078	0.137	17	797.4	0.112
6	1631.5	0.167	12	1032.7	0.175			

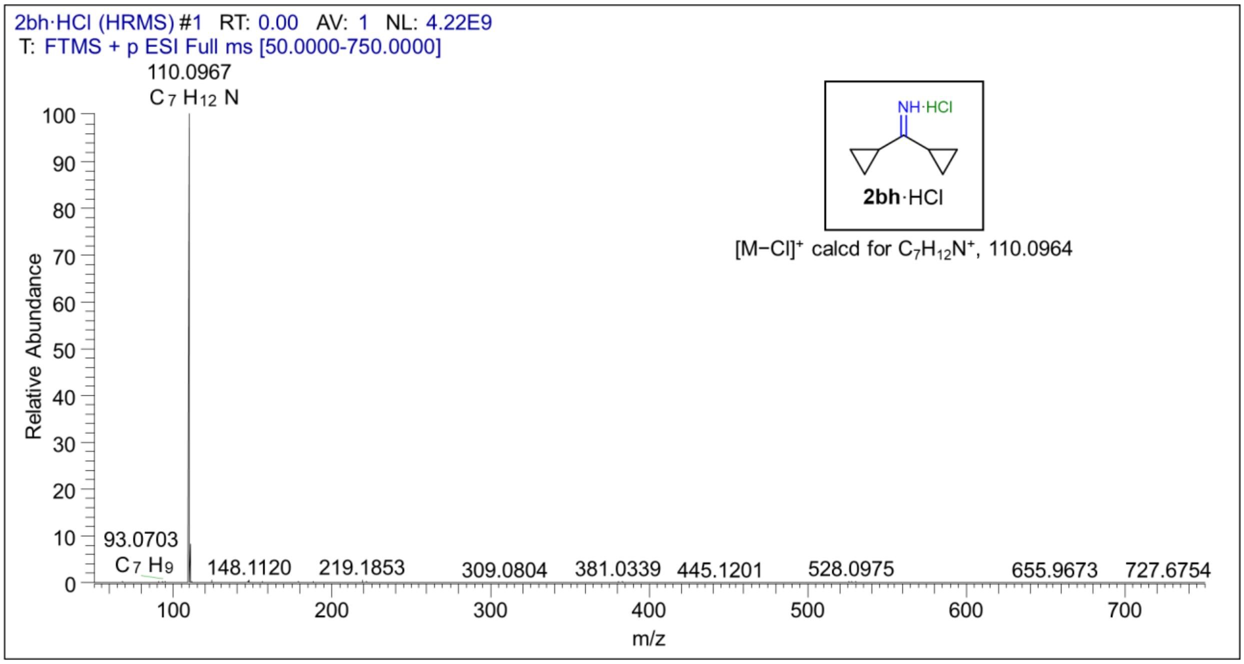

^{1}H NMR (400 MHz, DMSO-d_6) and ^{13}C NMR (100 MHz, DMSO-d_6) spectra for **2bi**·HCl

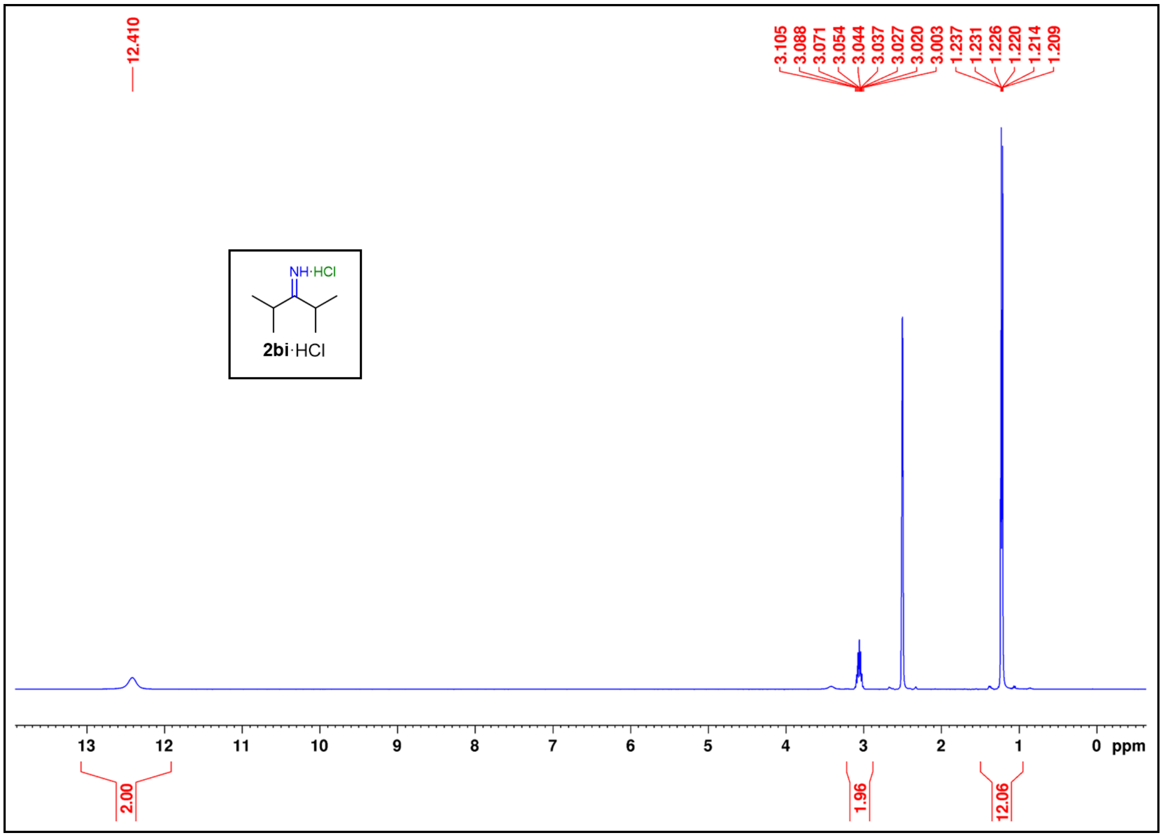

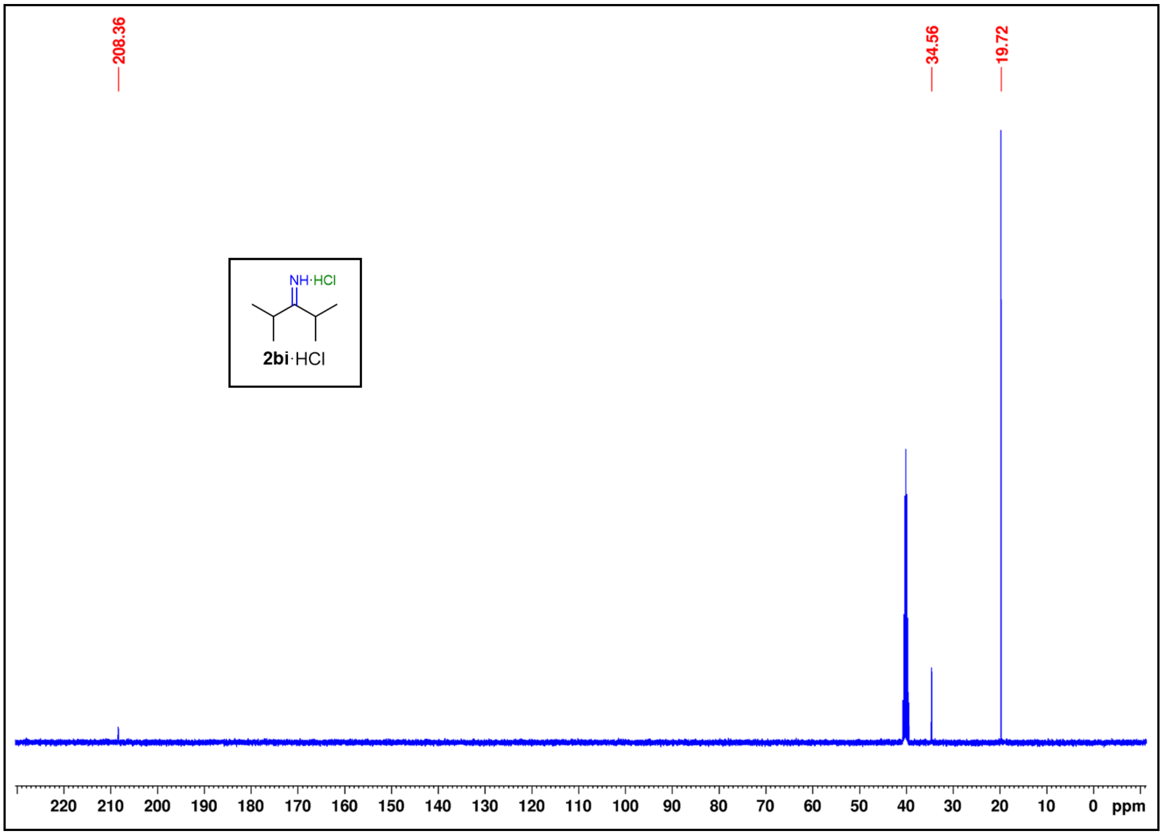

FT-IR (ATR, neat) and HRMS (ESI-positive) spectra for **2bi**·HCl

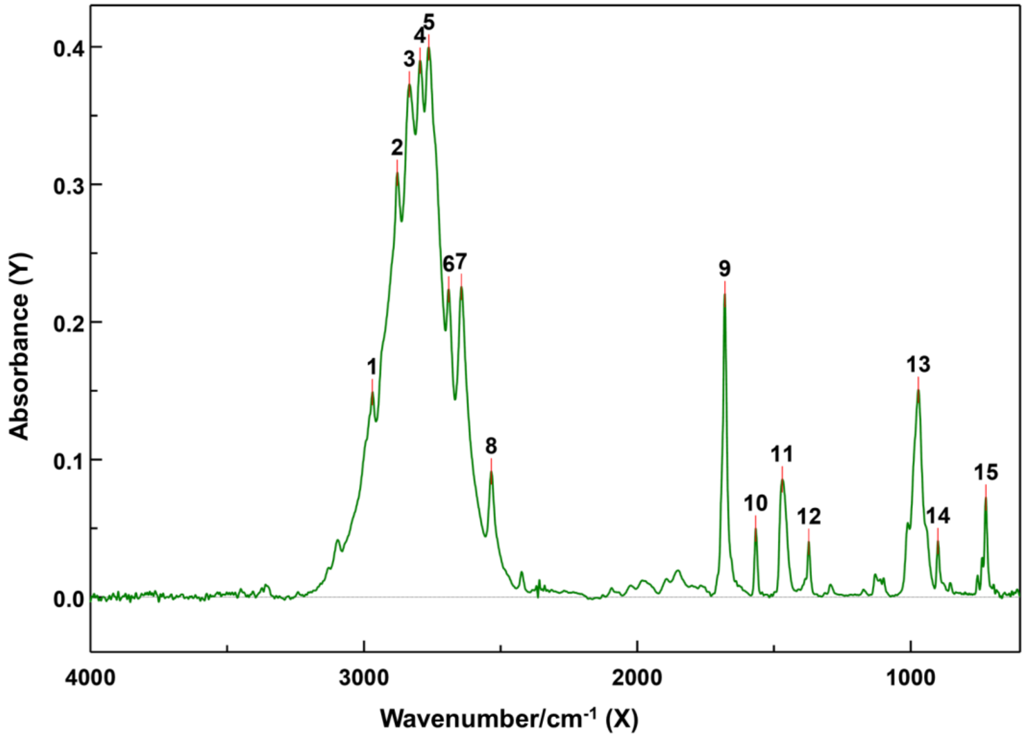

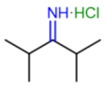

2bi·HCl

No.	X	Y	No.	X	Y	No.	X	Y
1	2968.9	0.149	6	2690.2	0.224	11	1470	0.085
2	2878.2	0.308	7	2643	0.225	12	1373	0.04
3	2833.9	0.373	8	2534	0.091	13	971.9	0.151
4	2794.3	0.39	9	1679.7	0.22	14	900.6	0.041
5	2763.5	0.399	10	1566.9	0.05	15	725.1	0.072

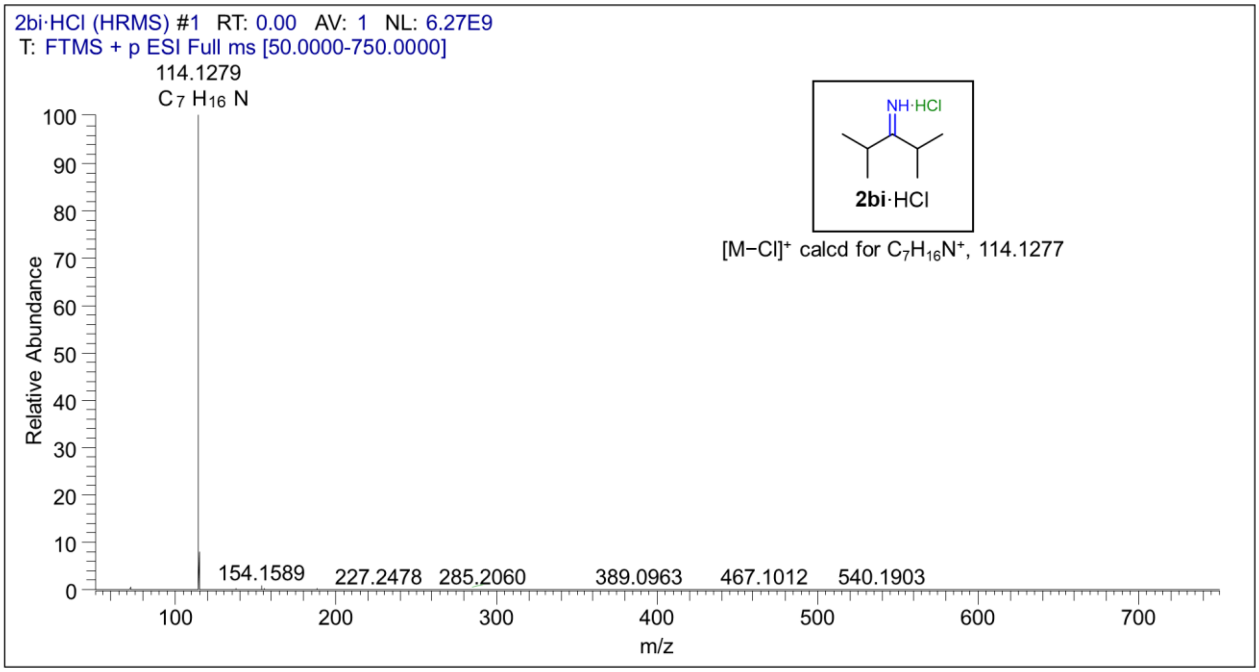

^{1}H NMR (400 MHz, CD_3CN) and ^{13}C NMR (100 MHz, CD_3CN) spectra for **2bj**·HCl

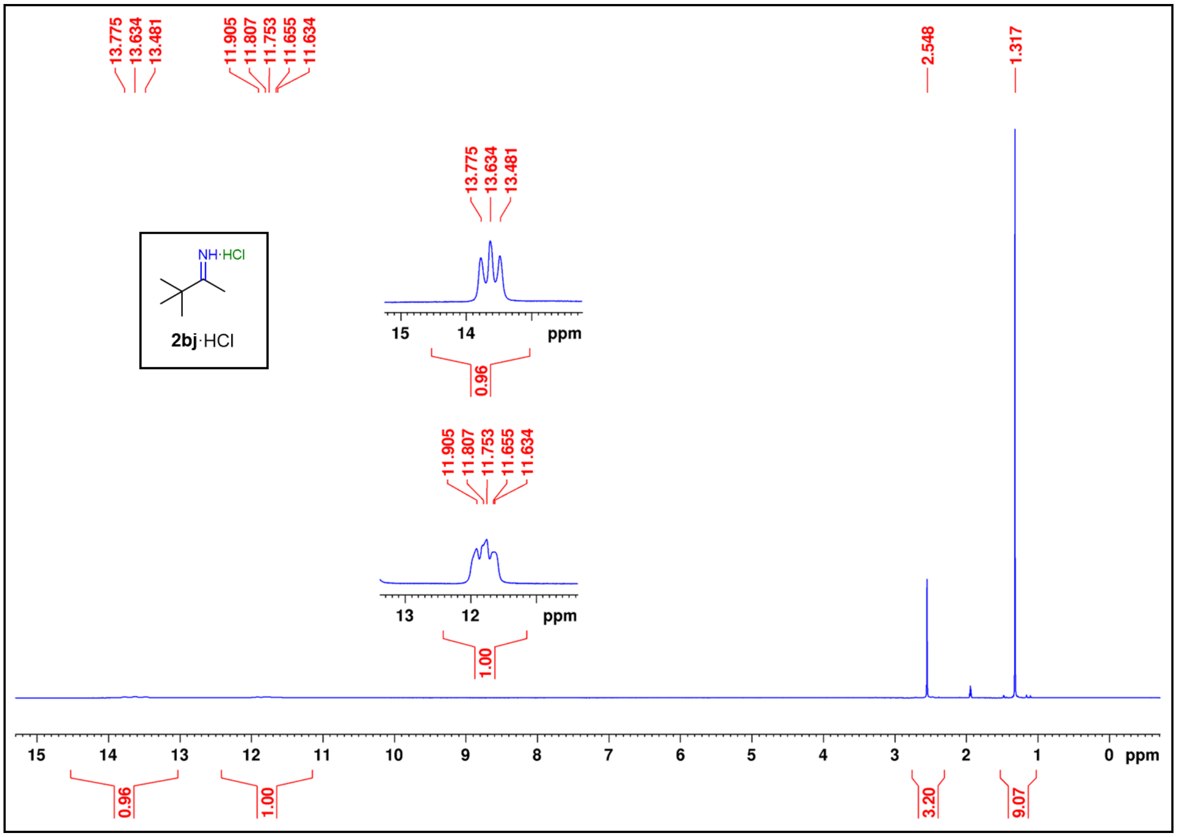

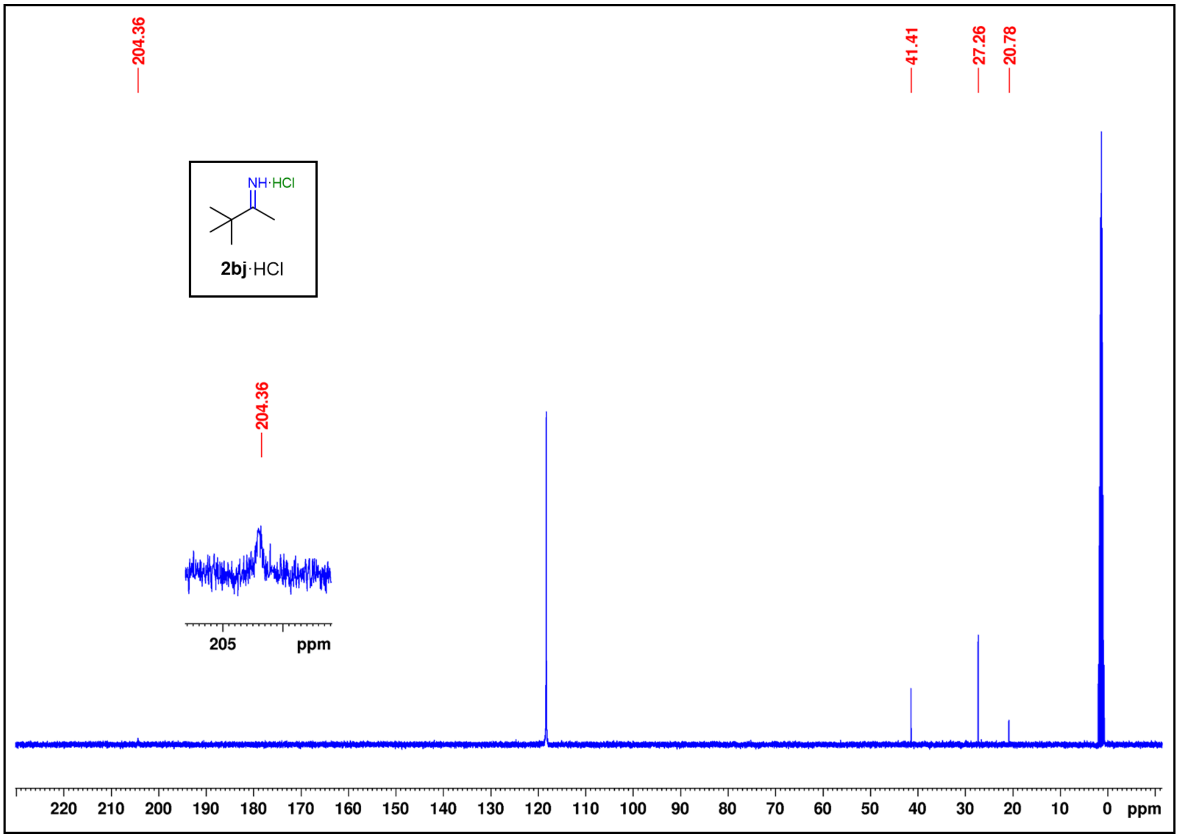

FT-IR (ATR, neat) and HRMS (ESI-positive) spectra for **2bj**·HCl

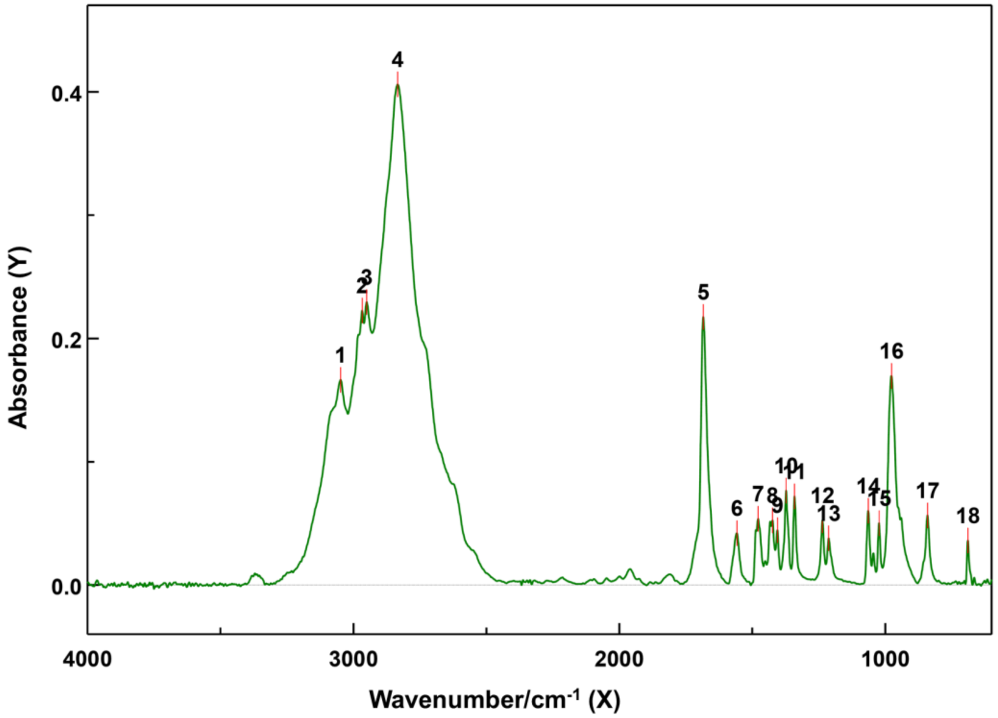

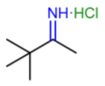

2bj·HCl

No.	X	Y	No.	X	Y	No.	X	Y
1	3047.9	0.166	7	1478.2	0.054	13	1213	0.038
2	2966.9	0.222	8	1424.2	0.052	14	1064	0.06
3	2950.6	0.229	9	1405.9	0.044	15	1023	0.05
4	2833.9	0.406	10	1373.1	0.076	16	976.8	0.17
5	1684.5	0.217	11	1341.2	0.072	17	840.8	0.056
6	1558.2	0.042	12	1236.1	0.052	18	689.4	0.036

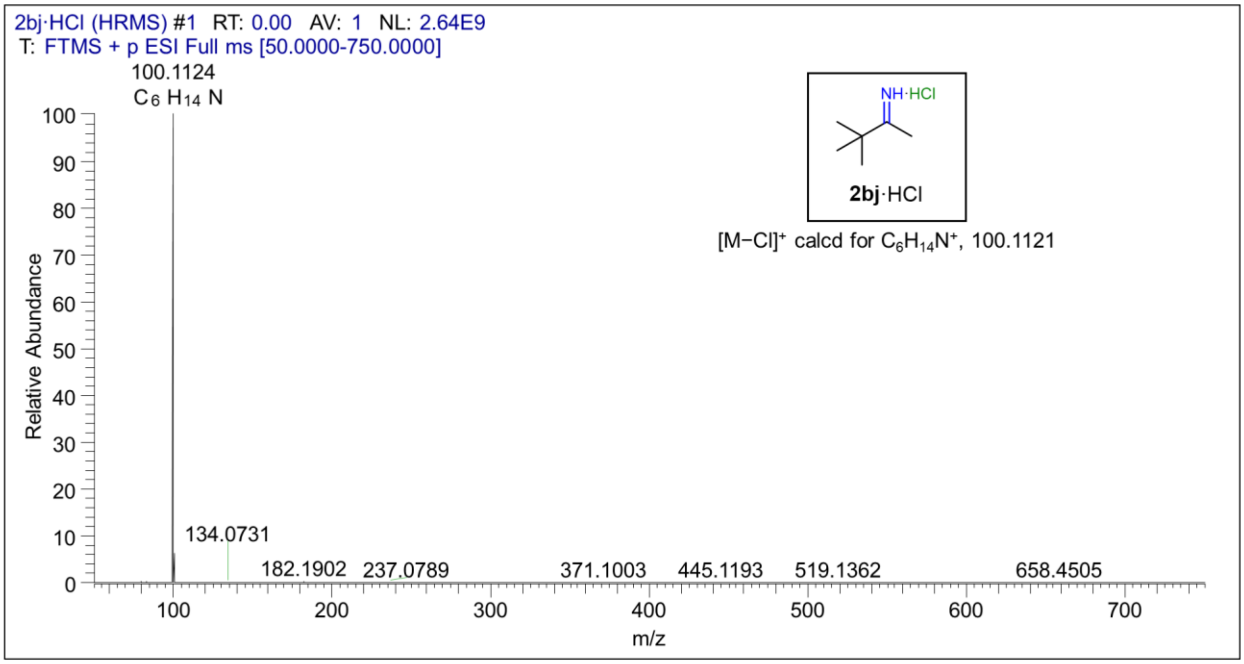

10.2 *N*–R Ketimine

<u>^{1}H NMR (400 MHz, $CDCl_3$) and ^{13}C NMR (100 MHz, $CDCl_3$) spectra for **3a**</u>

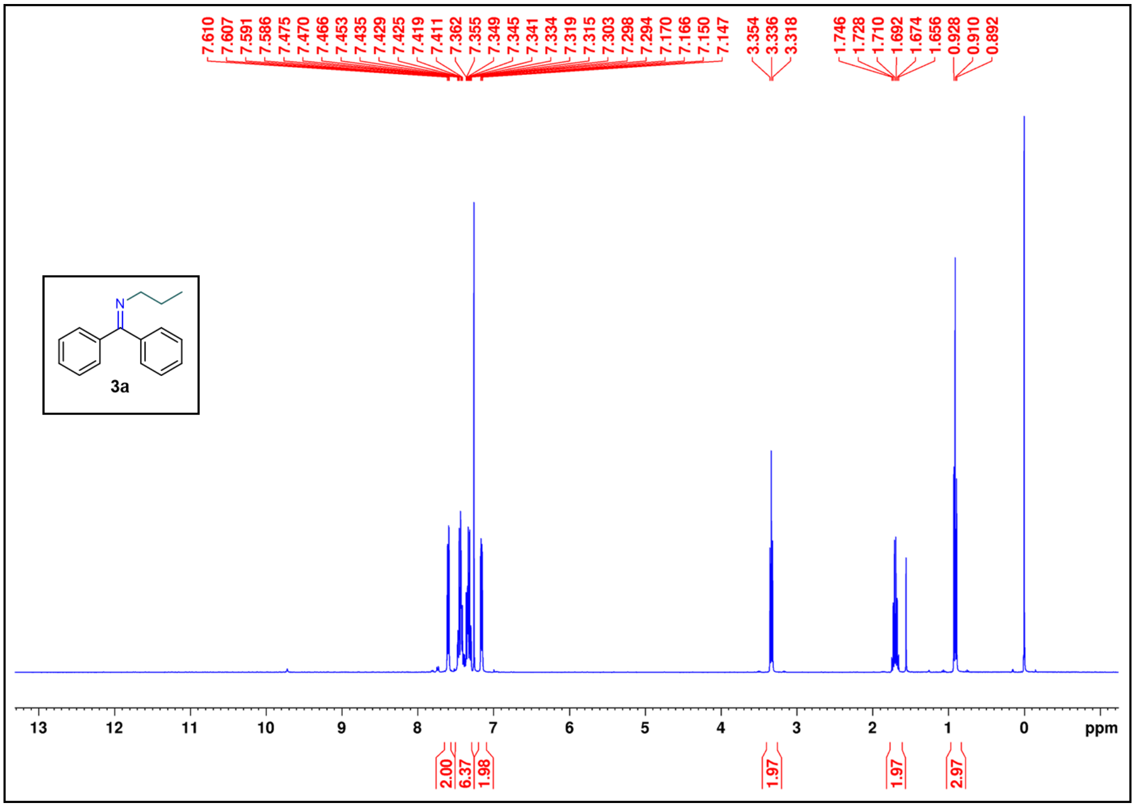

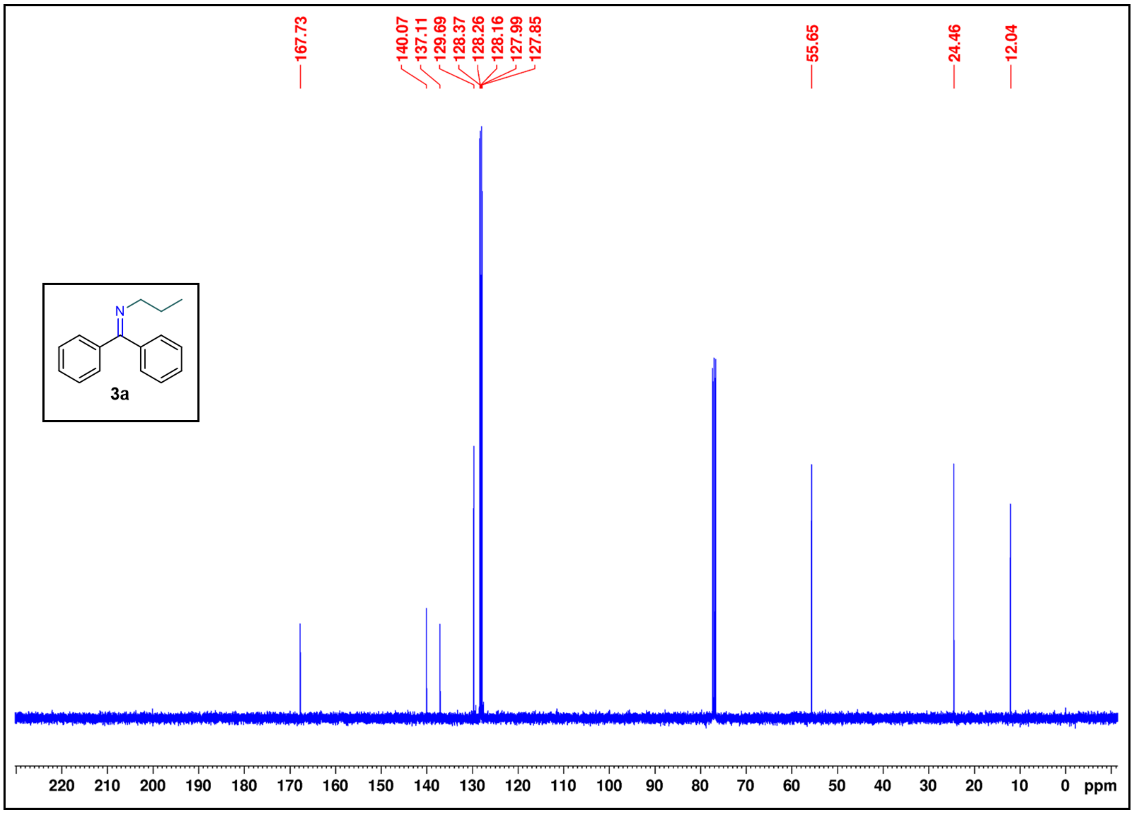

FT-IR (ATR, neat) and HRMS (ESI-positive) spectra for **3a**

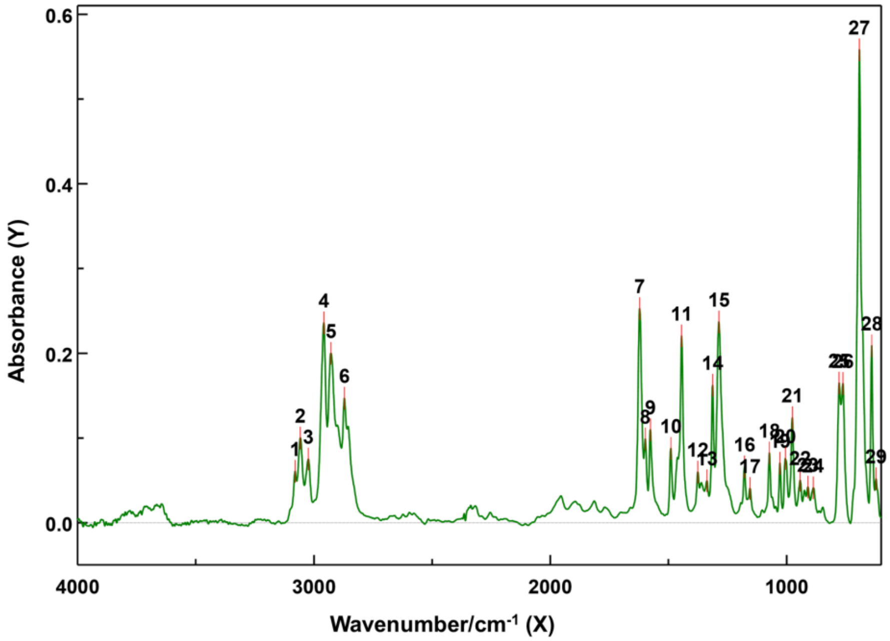

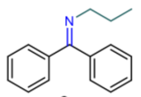

3a

No.	X	Y	No.	X	Y	No.	X	Y
1	3079.8	0.06	11	1444.4	0.22	21	975.8	0.124
2	3057.6	0.1	12	1376	0.06	22	943	0.05
3	3023.8	0.075	13	1337.4	0.049	23	910.2	0.042
4	2958.3	0.236	14	1313.3	0.162	24	887.1	0.041
5	2928.4	0.2	15	1286.3	0.237	25	778.1	0.164
6	2871.5	0.147	16	1178.3	0.066	26	761.7	0.164
7	1621.8	0.252	17	1155.2	0.04	27	692.3	0.558
8	1597.7	0.099	18	1073.2	0.082	28	640.3	0.208
9	1576.5	0.109	19	1027.9	0.07	29	621.9	0.052
10	1489.7	0.087	20	1005.7	0.075			

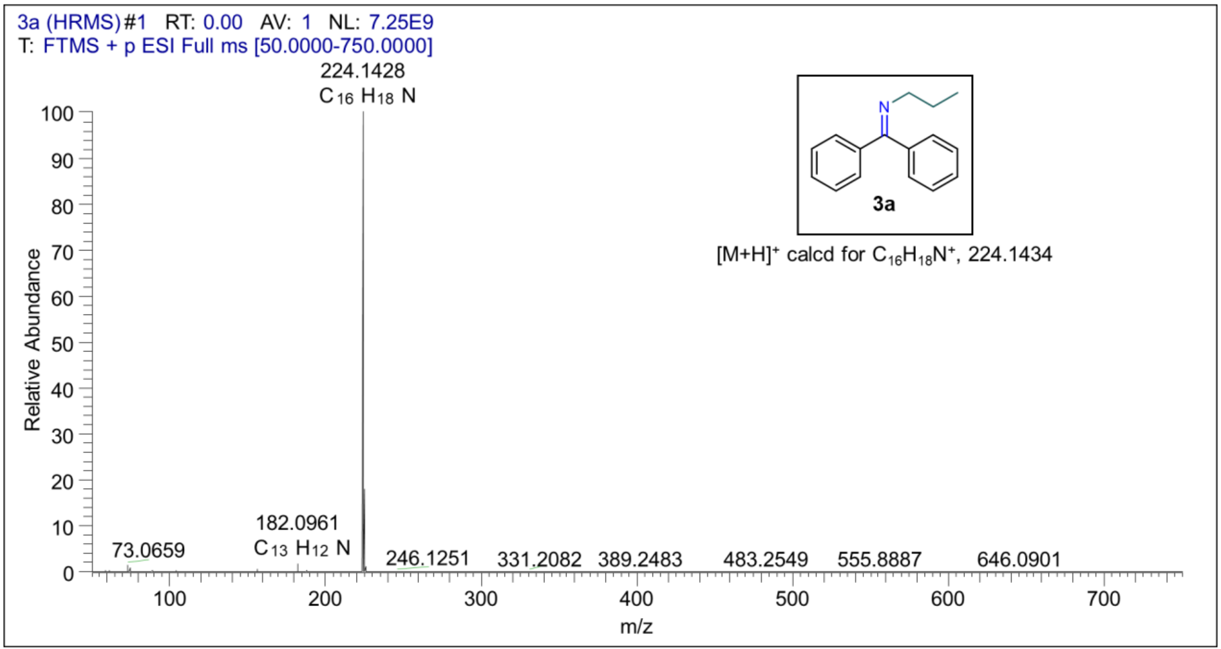

<u>^{1}H NMR (400 MHz, $CDCl_3$) and ^{13}C NMR (100 MHz, $CDCl_3$) spectra for **3b**</u>

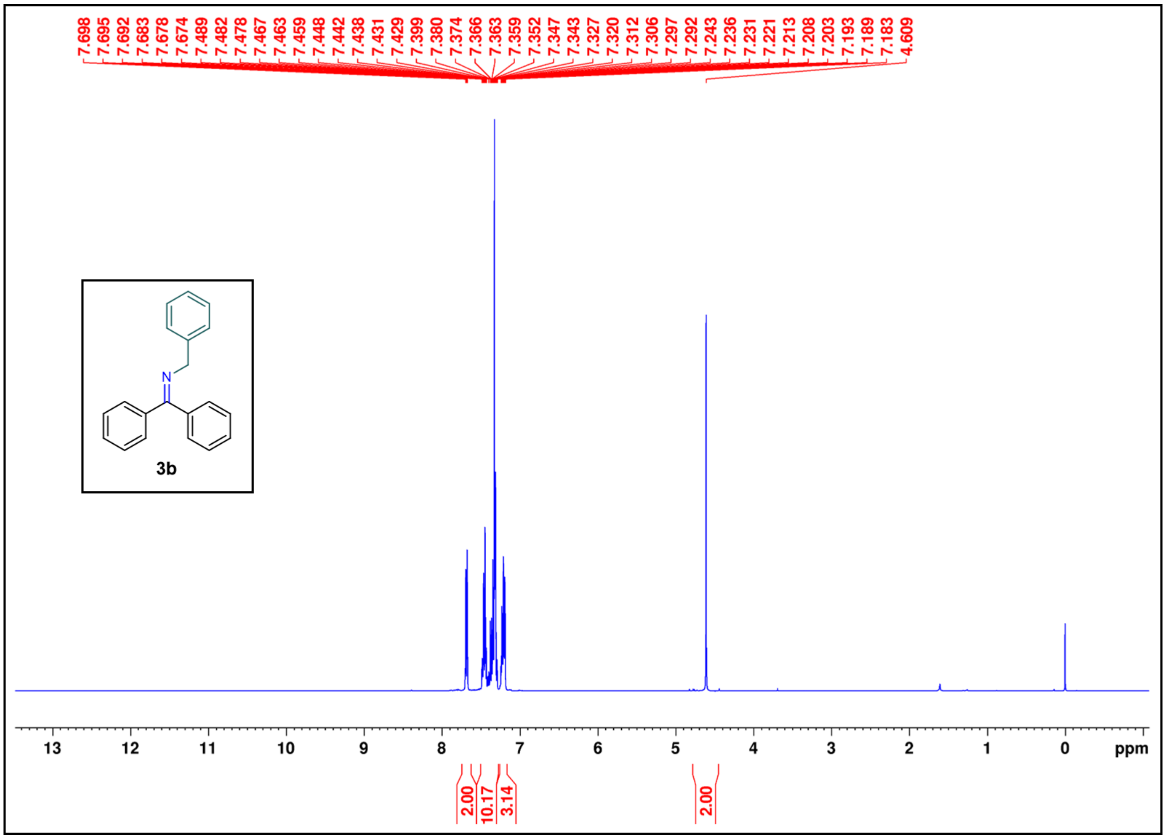

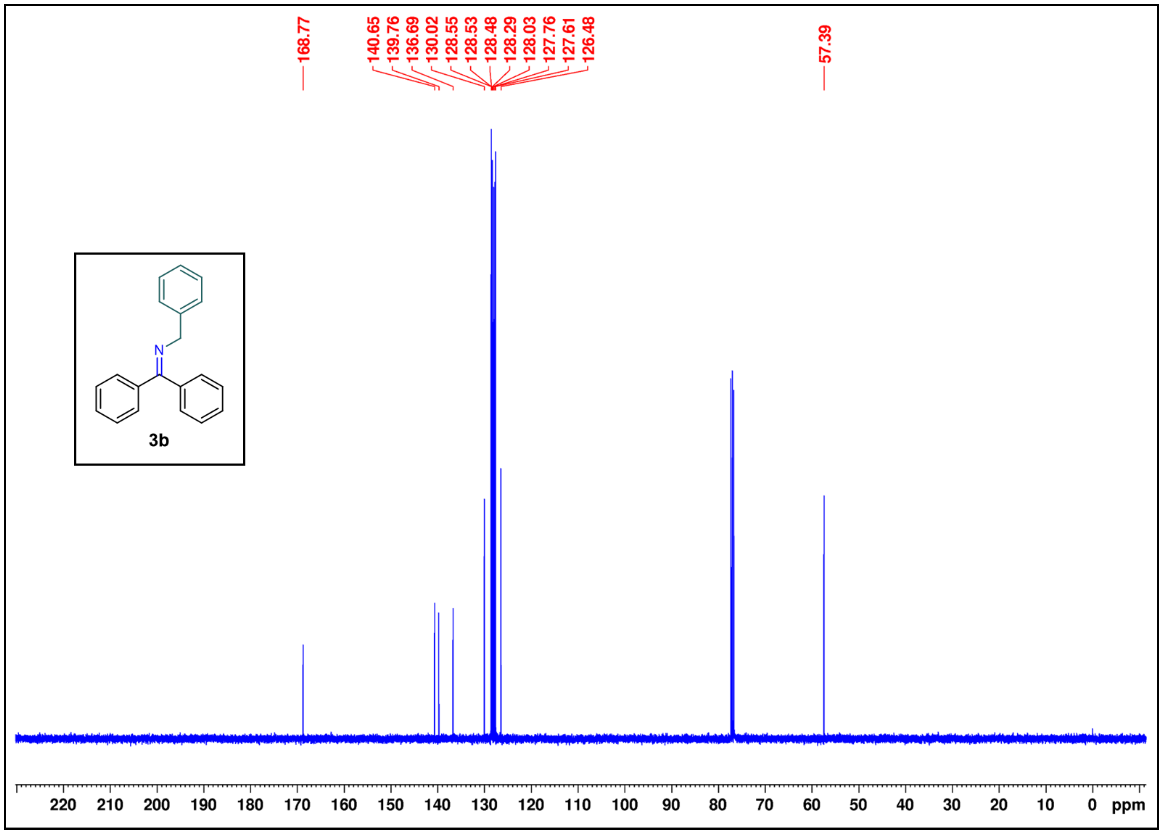

FT-IR (ATR, neat) and HRMS (ESI-positive) spectra for **3b**

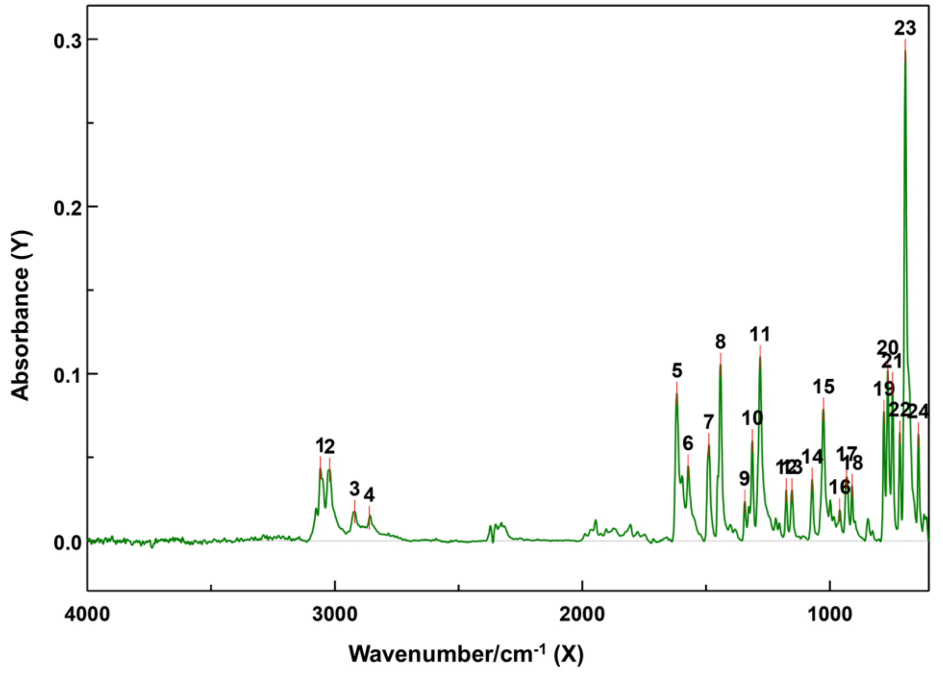

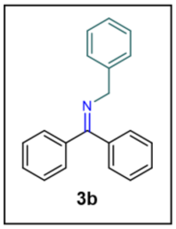

No.	X	Y	No.	X	Y	No.	X	Y
1	3058.5	0.043	9	1344.1	0.023	17	932.4	0.038
2	3020.9	0.042	10	1313.3	0.06	18	910.2	0.033
3	2919.7	0.017	11	1281.5	0.11	19	782	0.077
4	2860.9	0.014	12	1176.4	0.03	20	765.6	0.102
5	1618	0.088	13	1153.2	0.03	21	746.3	0.094
6	1572.7	0.045	14	1071.3	0.037	22	717.4	0.065
7	1488.8	0.057	15	1025.9	0.078	23	695.2	0.293
8	1441.5	0.105	16	960.4	0.018	24	642.2	0.064

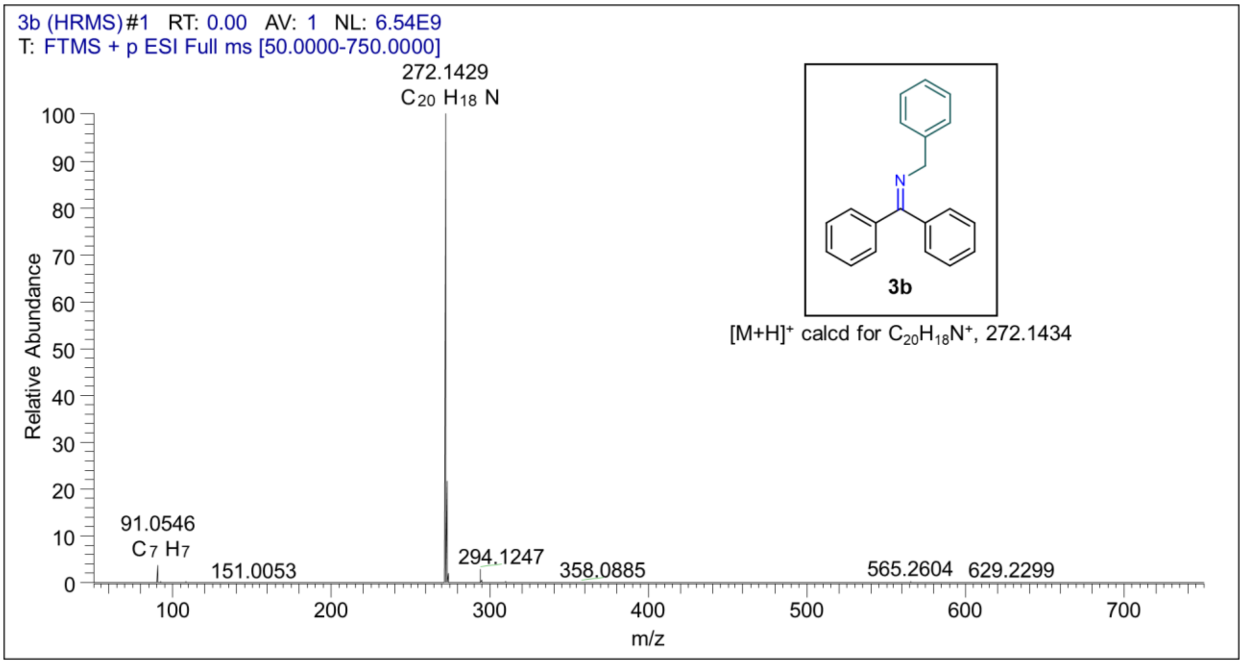

^{1}H NMR (400 MHz, $CDCl_3$) and ^{13}C NMR (100 MHz, $CDCl_3$) spectra for **3c**

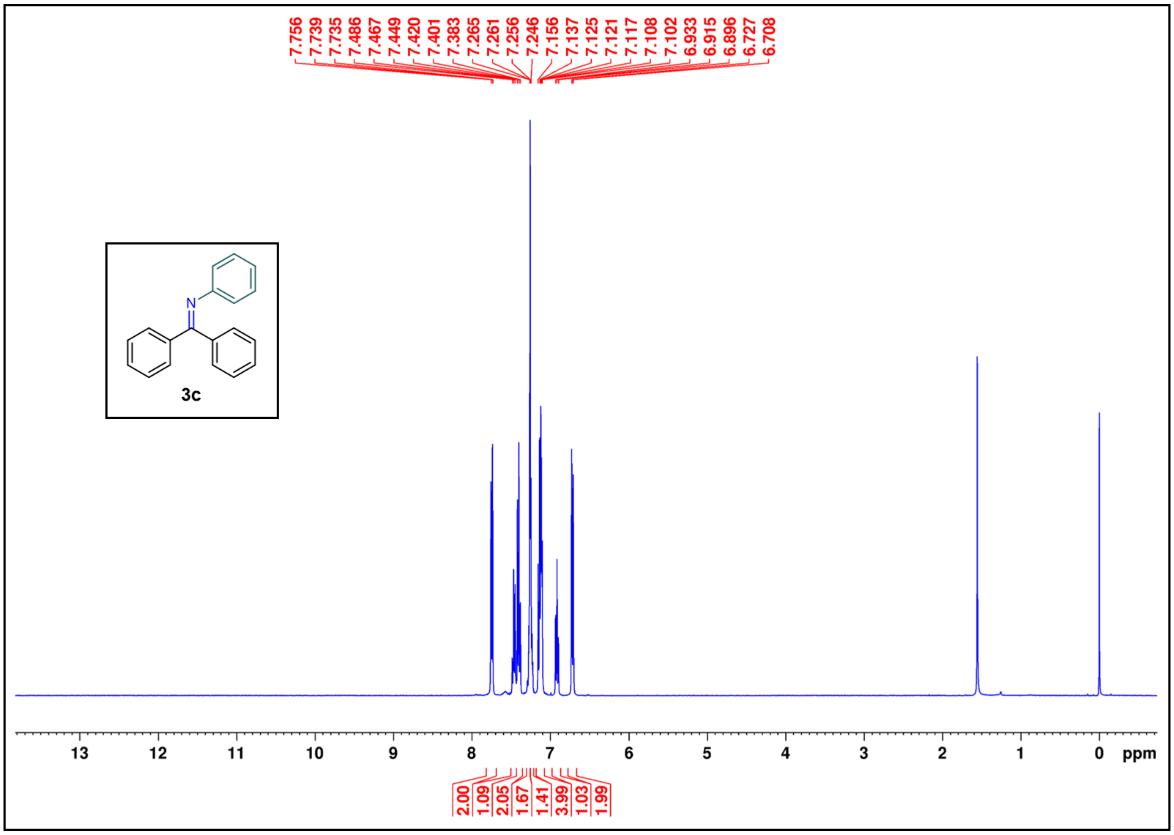

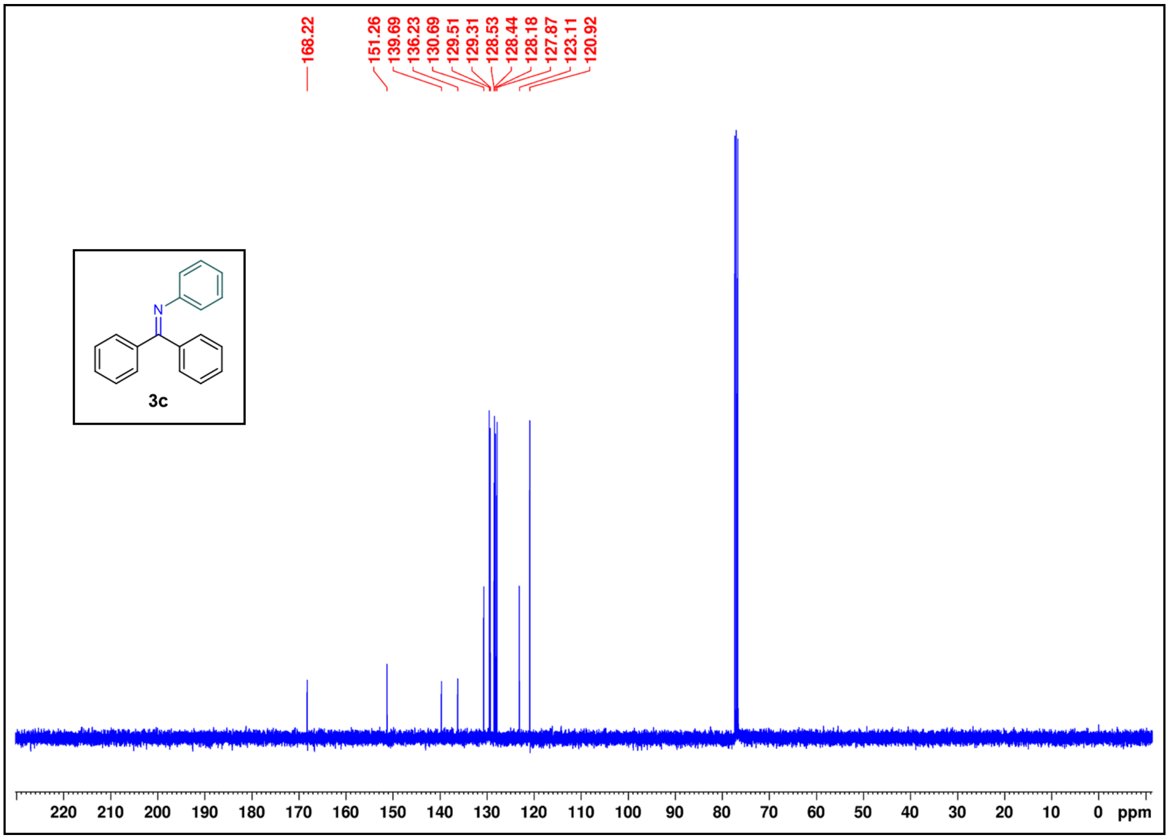

FT-IR (ATR, neat) and HRMS (ESI-positive) spectra for **3c**

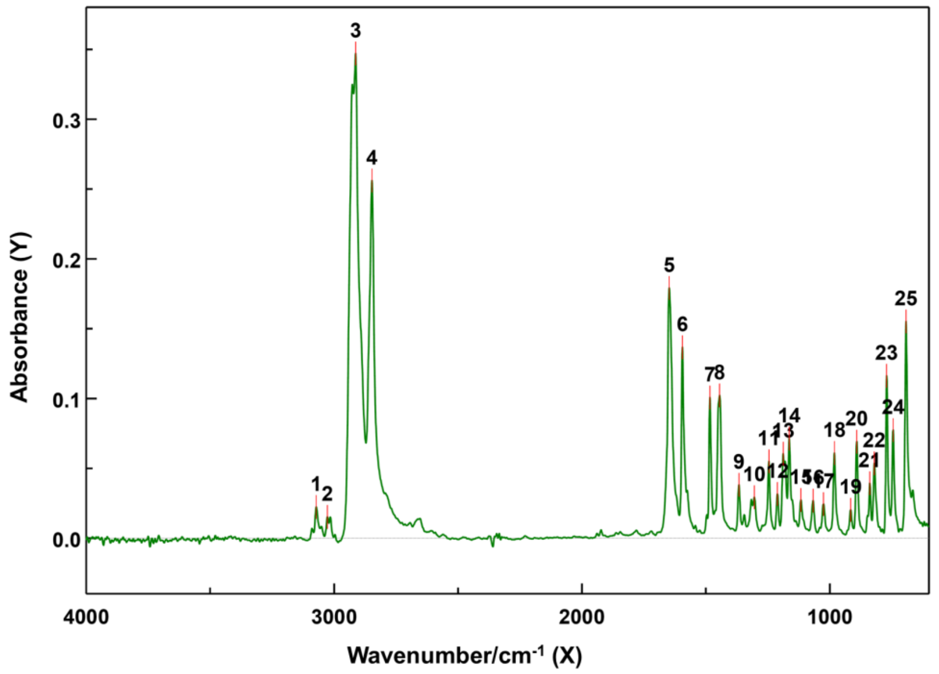

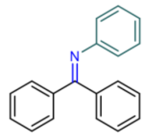

3c

No.	X	Y	No.	X	Y	No.	X	Y
1	3072	0.022	10	1304.6	0.029	19	916	0.02
2	3026.7	0.015	11	1245.8	0.055	20	891.9	0.069
3	2912.9	0.347	12	1211.1	0.031	21	838.9	0.039
4	2847.4	0.256	13	1187.9	0.06	22	820.6	0.053
5	1647.9	0.179	14	1163.8	0.071	23	770.4	0.116
6	1594.8	0.136	15	1116.6	0.027	24	744.4	0.077
7	1484	0.101	16	1067.4	0.027	25	692.3	0.155
8	1444.4	0.102	17	1025.9	0.024			
9	1366.3	0.038	18	981.6	0.061			

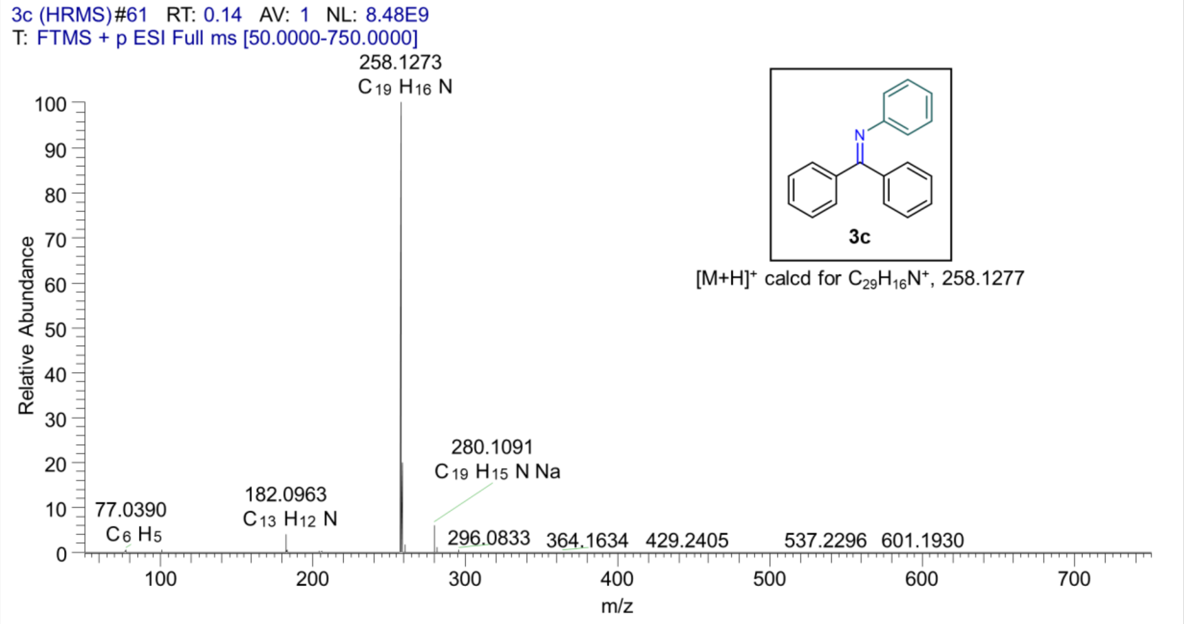

^{1}H NMR (400 MHz, $CDCl_3$) and ^{13}C NMR (100 MHz, $CDCl_3$) spectra for **3d**

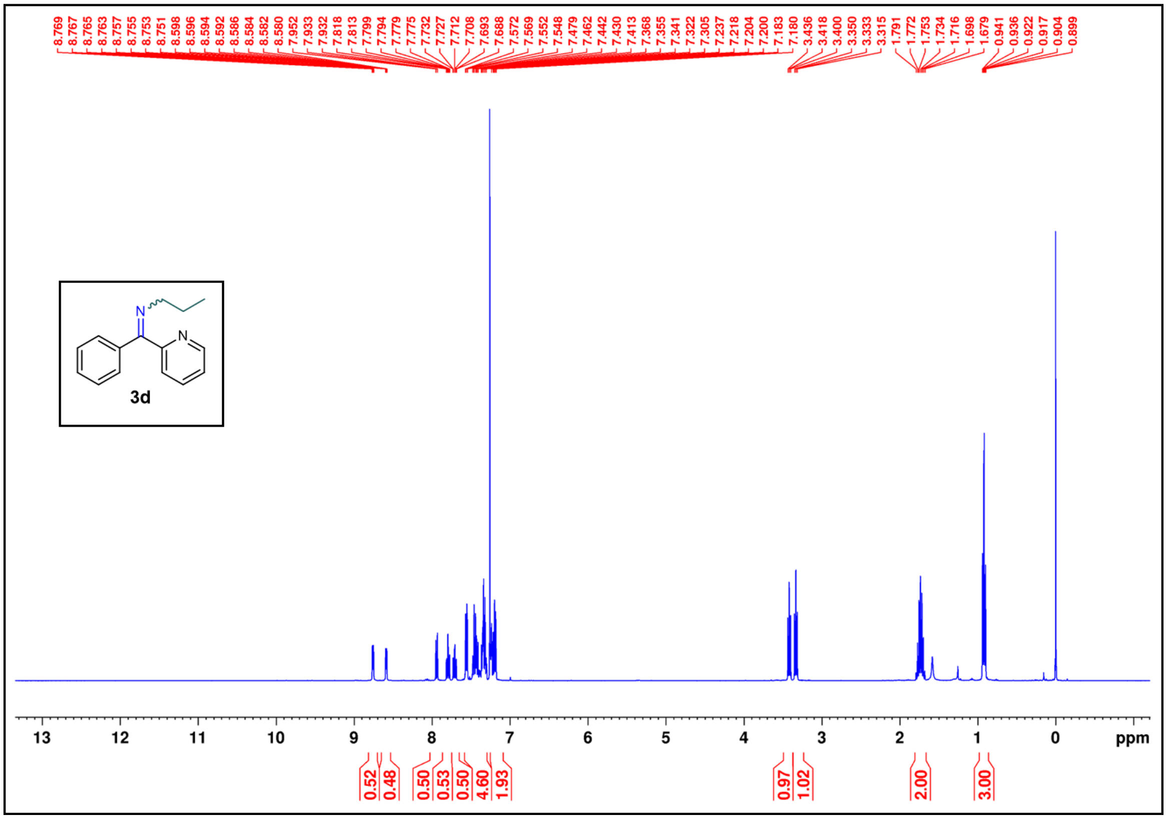

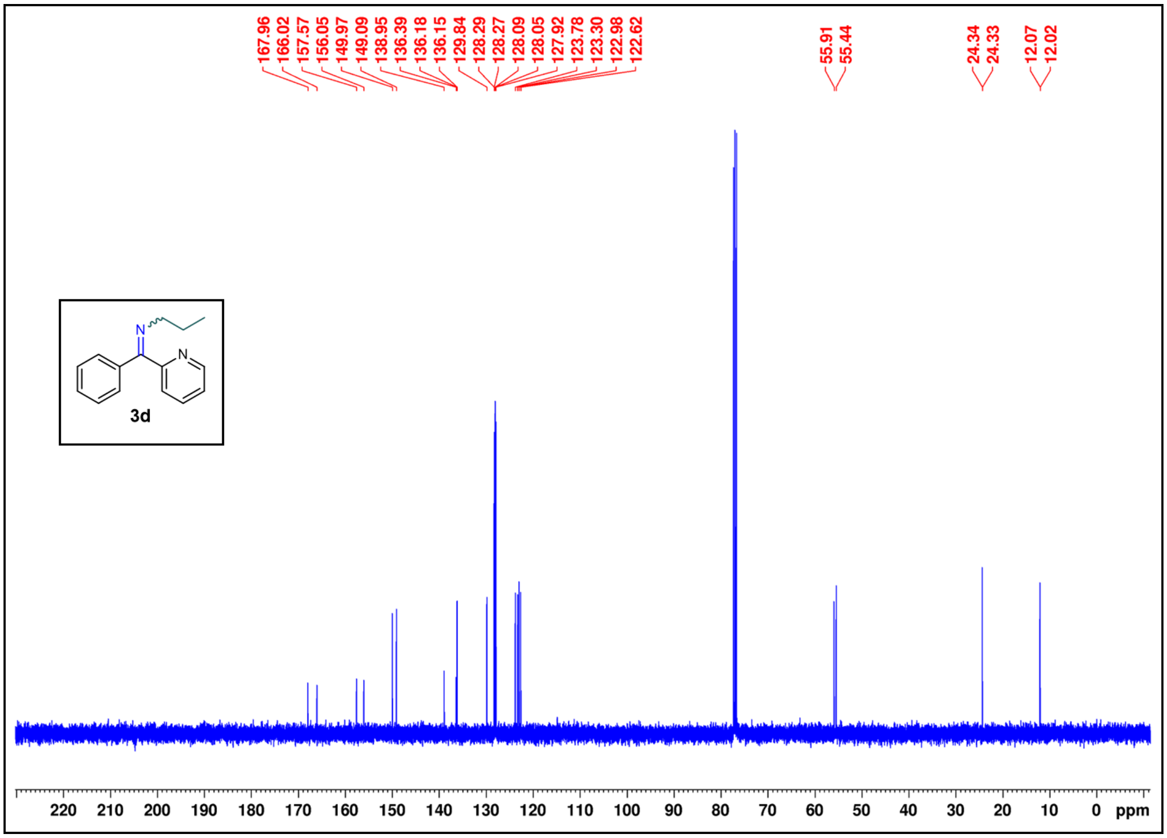

FT-IR (ATR, neat) and HRMS (ESI-positive) spectra for **3d**

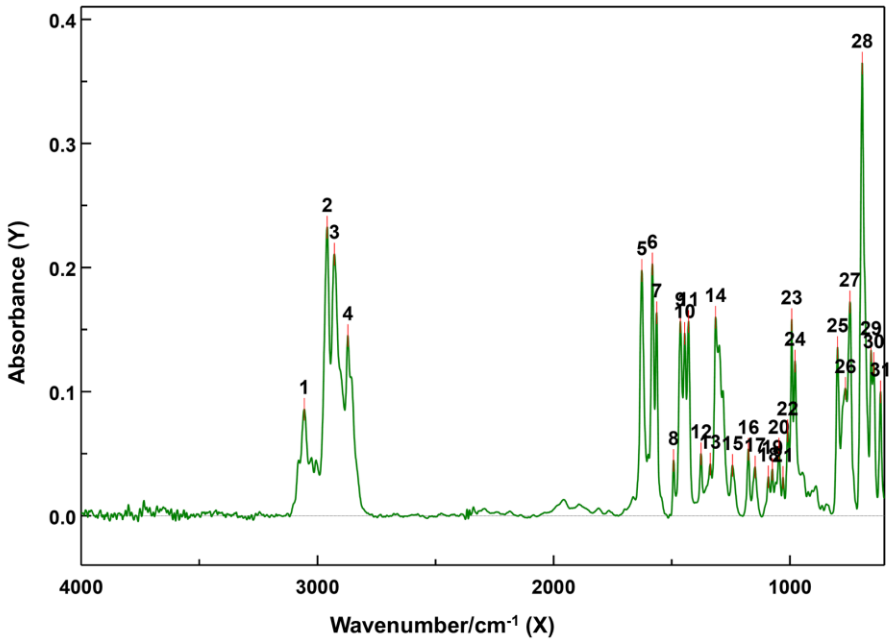

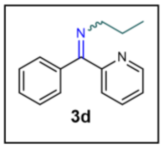

No.	X	Y	No.	X	Y	No.	X	Y
1	3055.7	0.086	12	1376	0.05	23	992.2	0.158
2	2959.2	0.232	13	1337.4	0.042	24	977.7	0.124
3	2928.4	0.211	14	1314.3	0.16	25	798.4	0.135
4	2871.5	0.145	15	1242.9	0.04	26	764.6	0.102
5	1626.7	0.197	16	1175.4	0.053	27	745.4	0.172
6	1582.3	0.203	17	1147.4	0.039	28	694.2	0.365
7	1564	0.163	18	1091.5	0.031	29	656.6	0.133
8	1492.6	0.044	19	1074.2	0.037	30	645.1	0.122
9	1463.7	0.156	20	1046.2	0.053	31	616.1	0.1
10	1445.4	0.147	21	1028.8	0.031			
11	1429	0.156	22	1009.6	0.068			

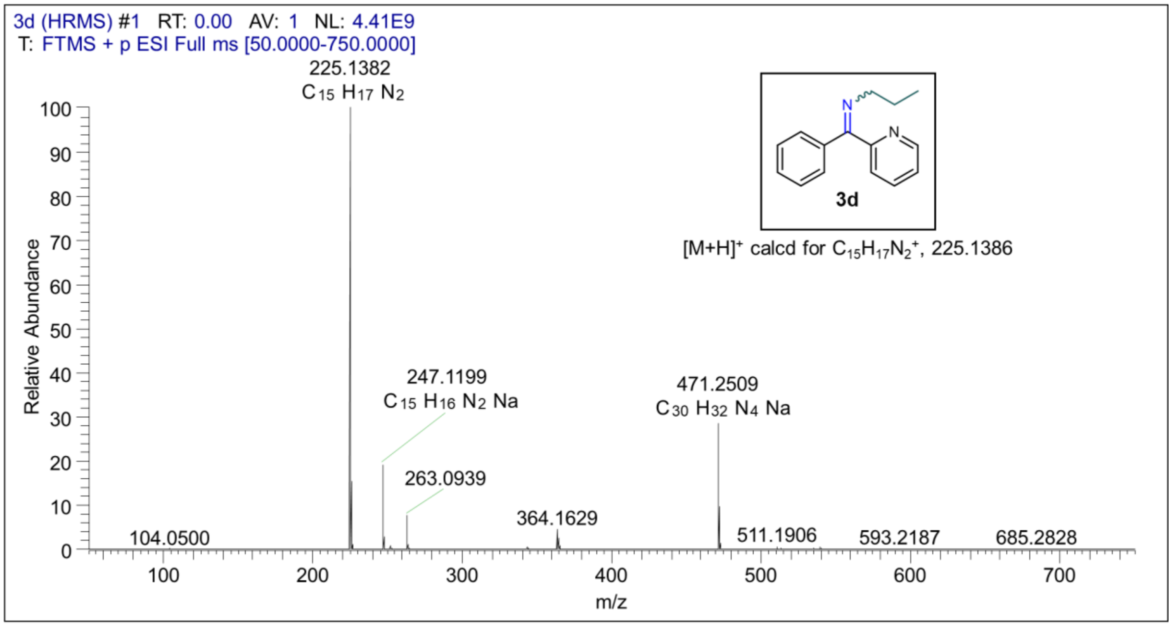

^{1}H NMR (400 MHz, $CDCl_3$) and ^{13}C NMR (100 MHz, $CDCl_3$) spectra for **3e**

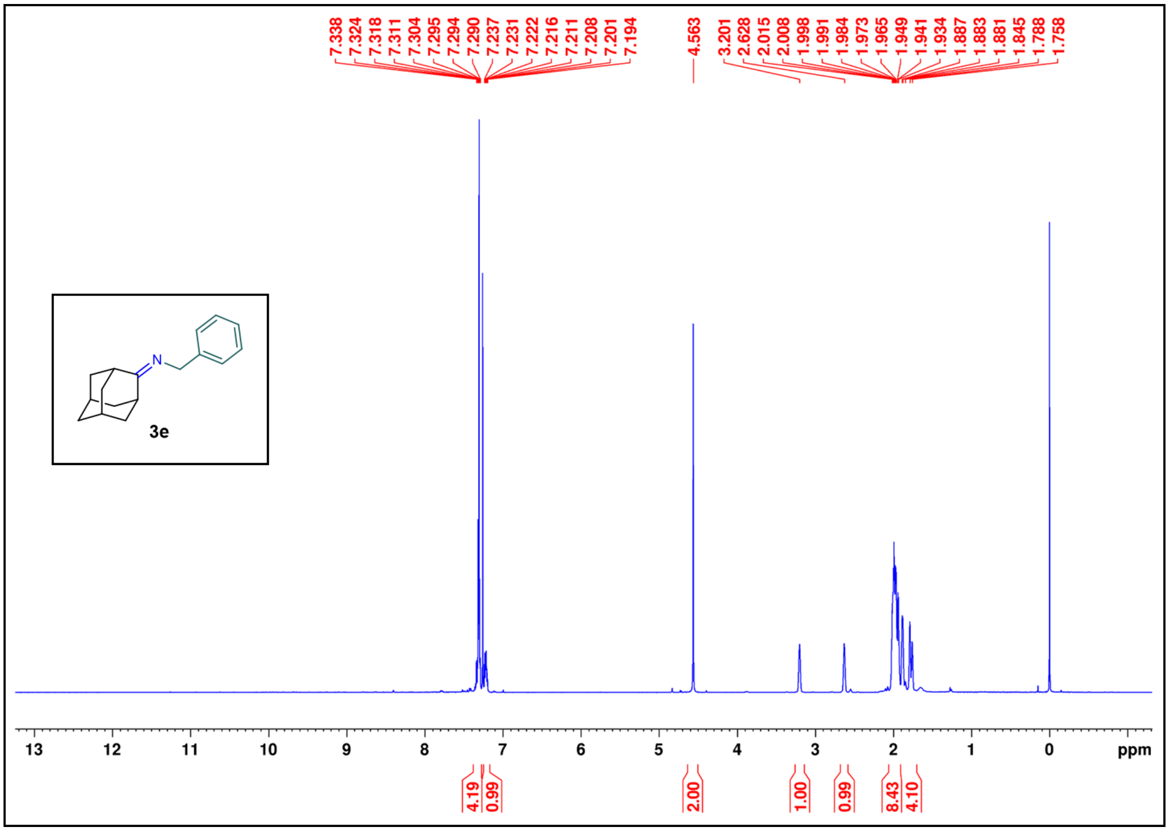

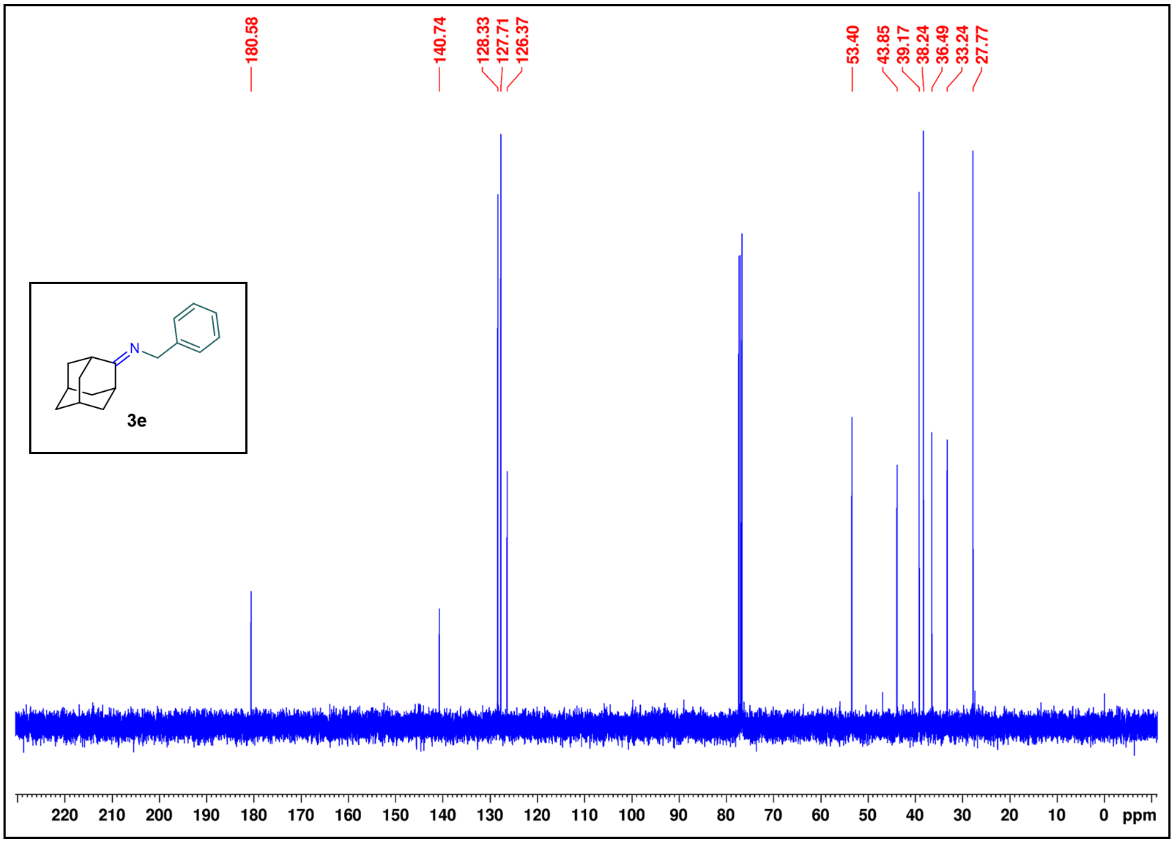

FT-IR (ATR, neat) and HRMS (ESI-positive) spectra for **3e**

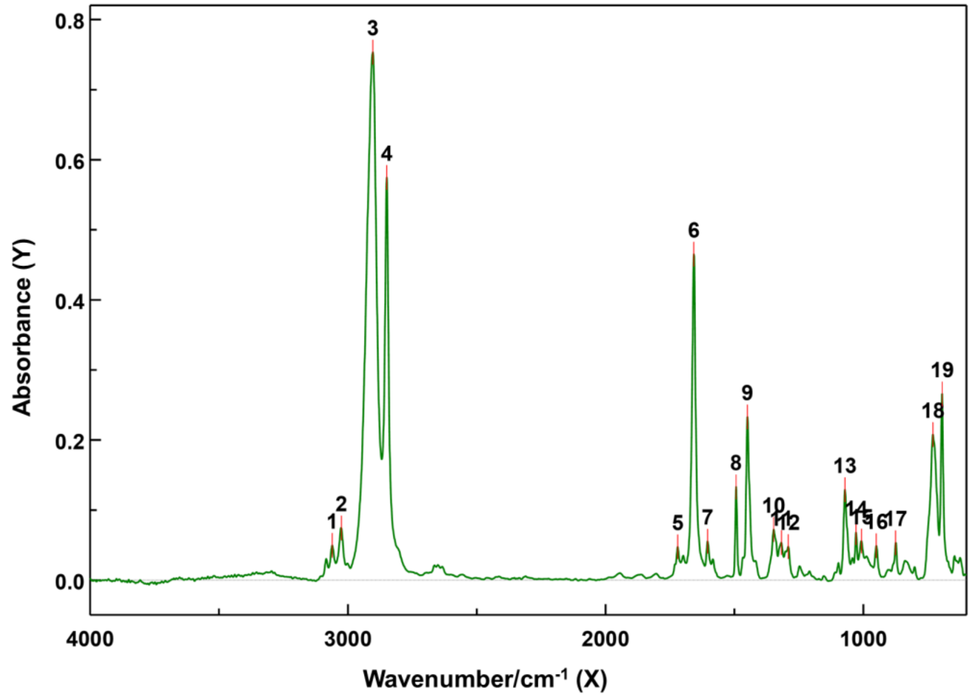

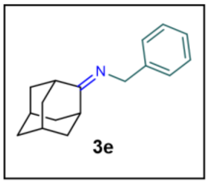

No.	X	Y		No.	X	Y		No.	X	Y
1	3060.5	0.049		8	1493.6	0.133		15	1008	0.056
2	3025.8	0.074		9	1449.2	0.233		16	949.8	0.049
3	2903.3	0.753		10	1348	0.072		17	874.6	0.053
4	2849.3	0.574		11	1318.1	0.053		18	729.9	0.207
5	1720.2	0.047		12	1291.1	0.047		19	694.2	0.265
6	1657.5	0.465		13	1071.3	0.129				
7	1604.5	0.055		14	1027.9	0.067				

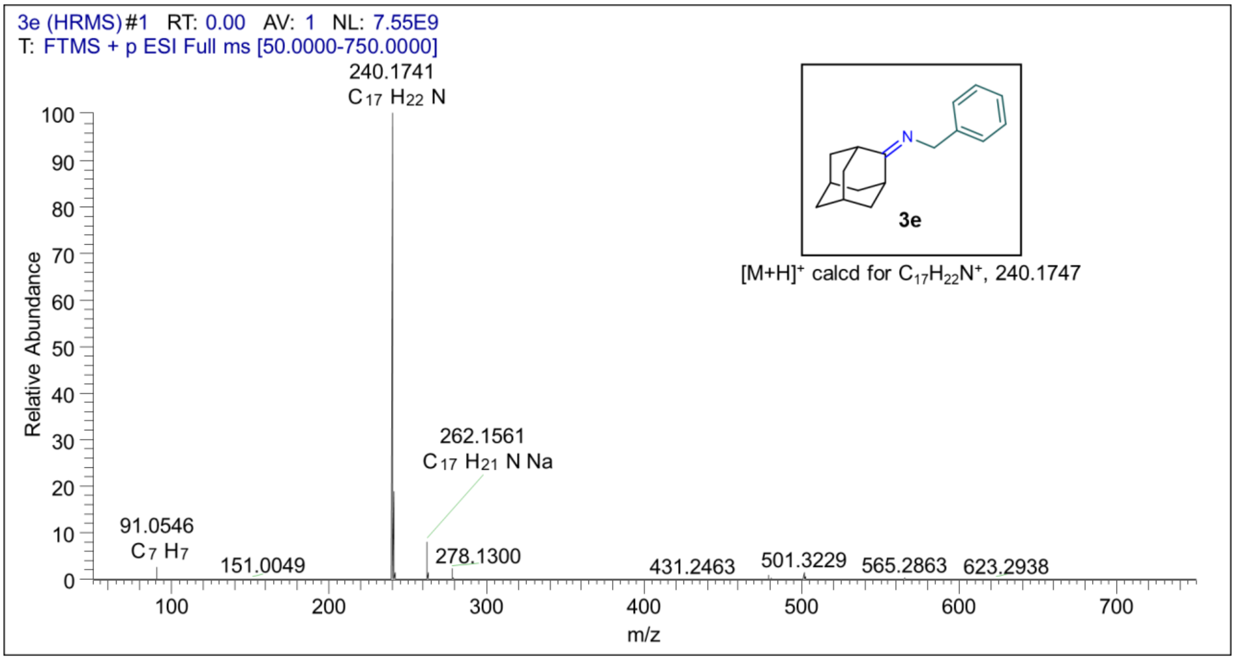

<u>^{1}H NMR (400 MHz, $CDCl_3$) and ^{13}C NMR (100 MHz, $CDCl_3$) spectra for **3f**</u>

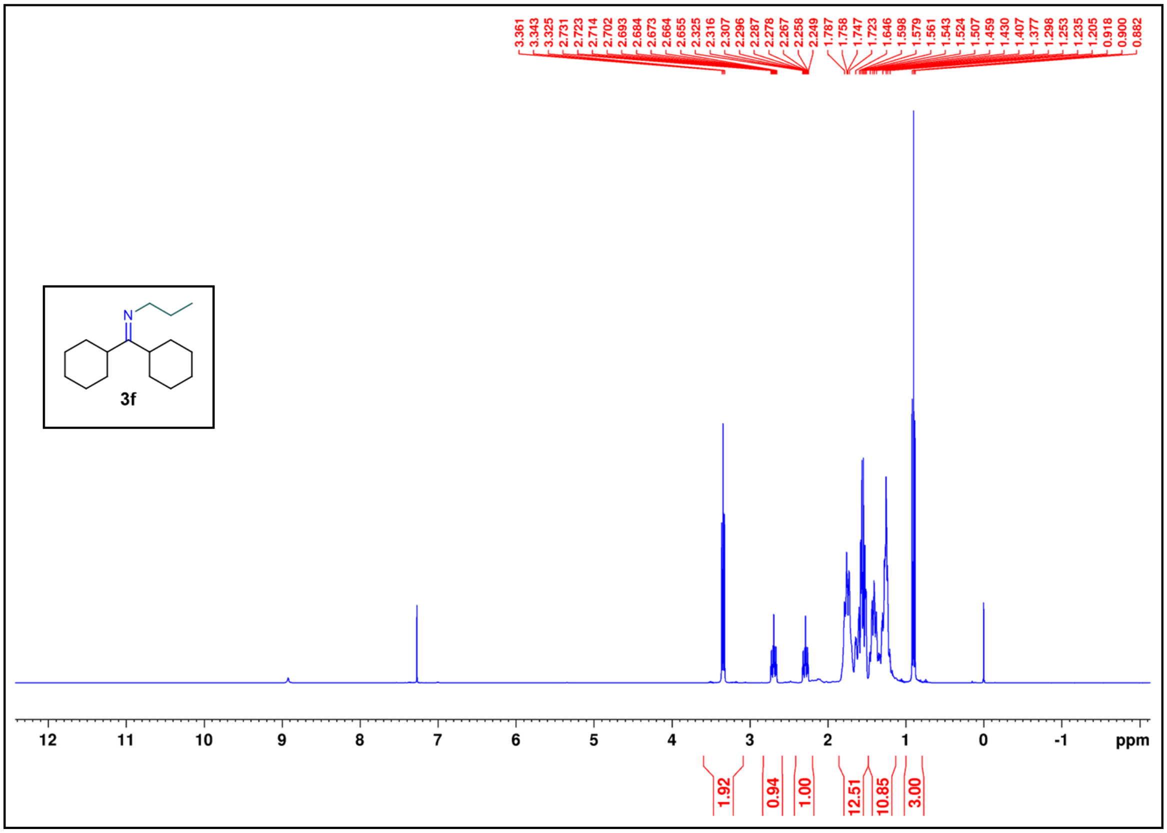

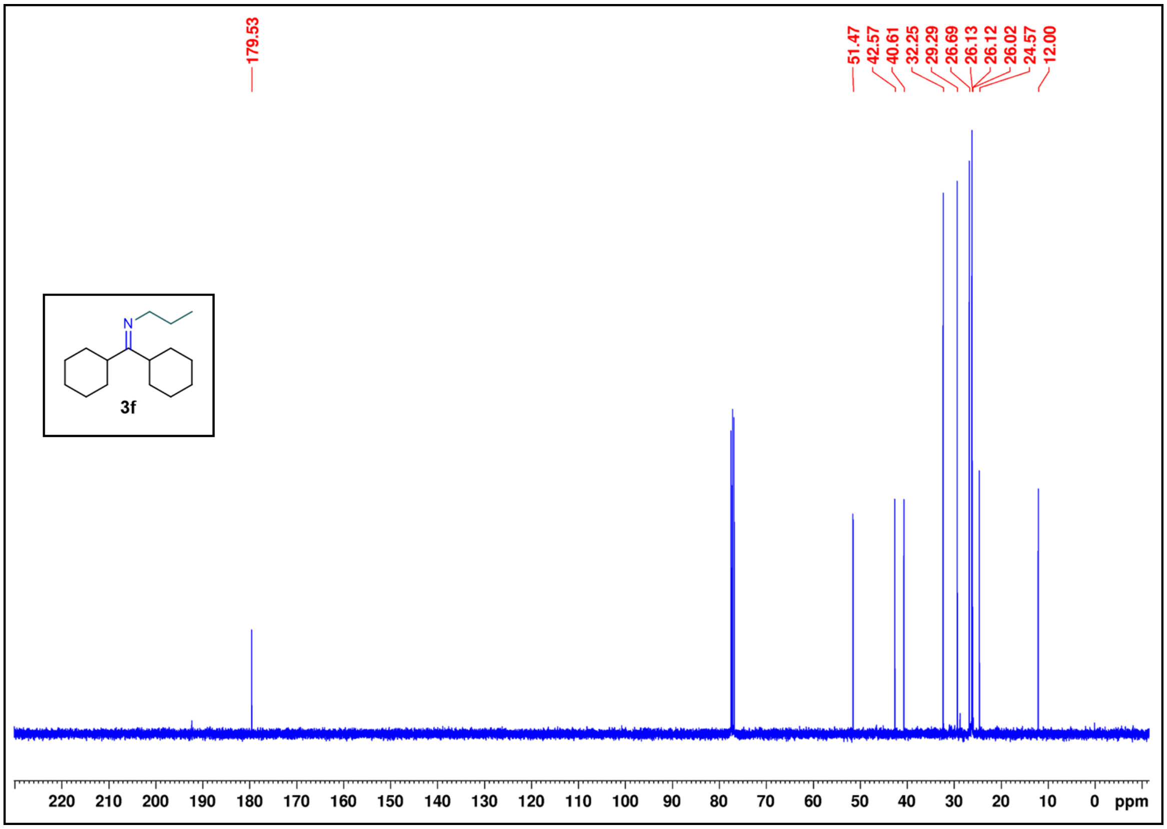

FT-IR (ATR, neat) and HRMS (ESI-positive) spectra for **3f**

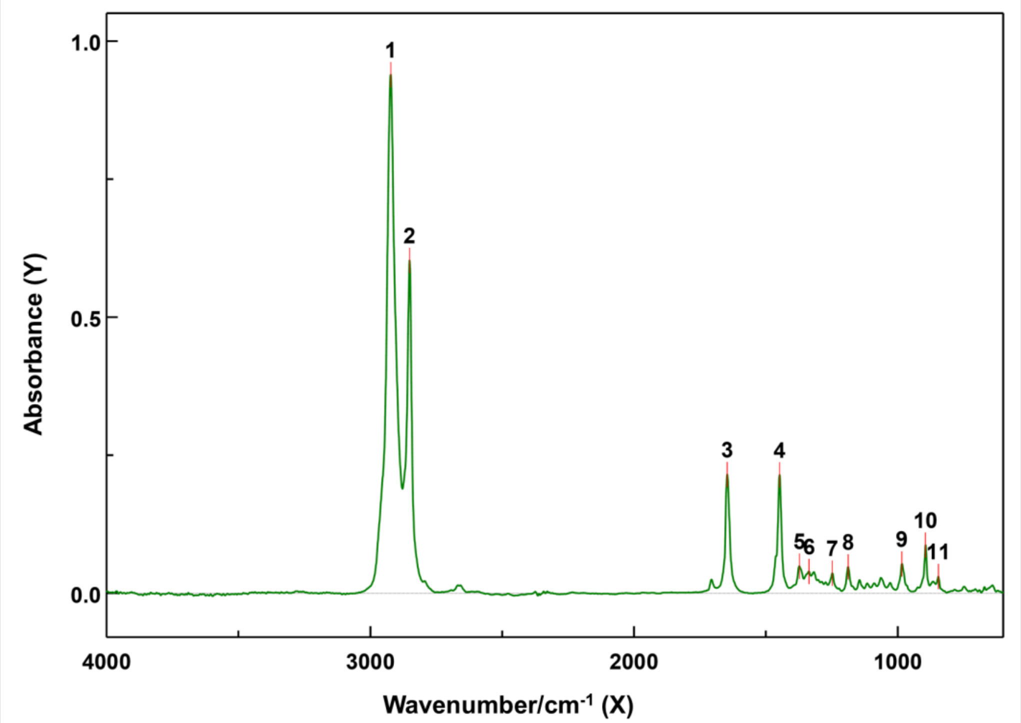

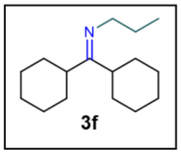

No.	X	Y	No.	X	Y	No.	X	Y
1	2922.6	0.939	5	1373.1	0.049	9	984.5	0.053
2	2851.2	0.602	6	1336.4	0.039	10	894.8	0.087
3	1646.9	0.214	7	1247.7	0.036	11	845.6	0.03
4	1448.3	0.214	8	1187.9	0.047			

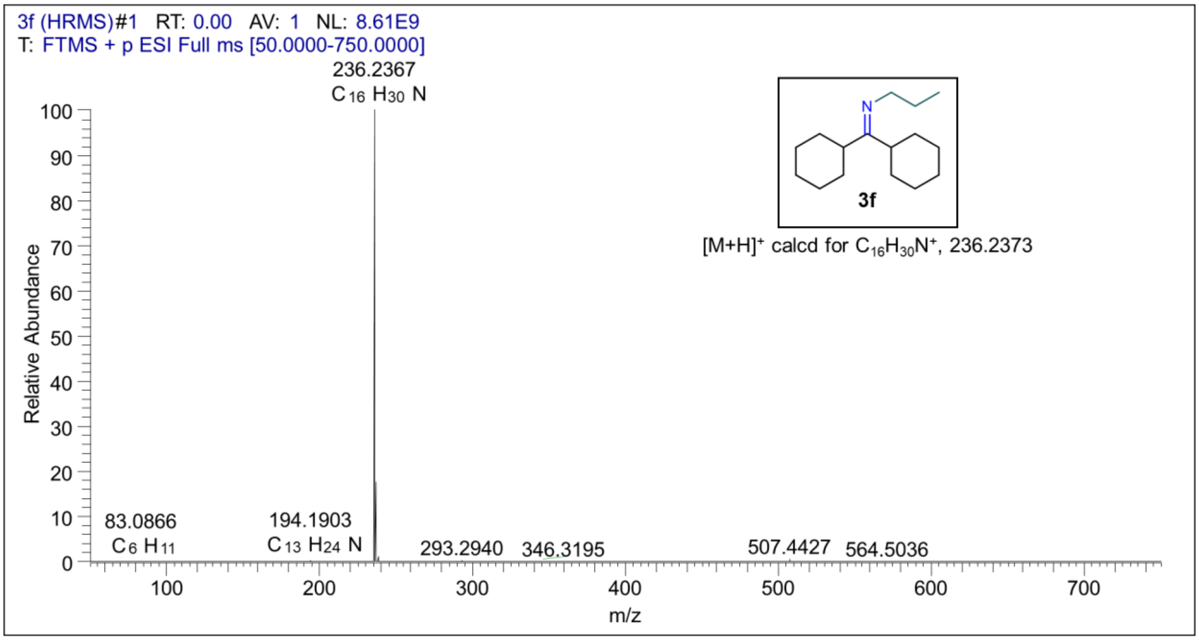

^{1}H NMR (400 MHz, $CDCl_3$) and ^{13}C NMR (100 MHz, $CDCl_3$) spectra for **3g**

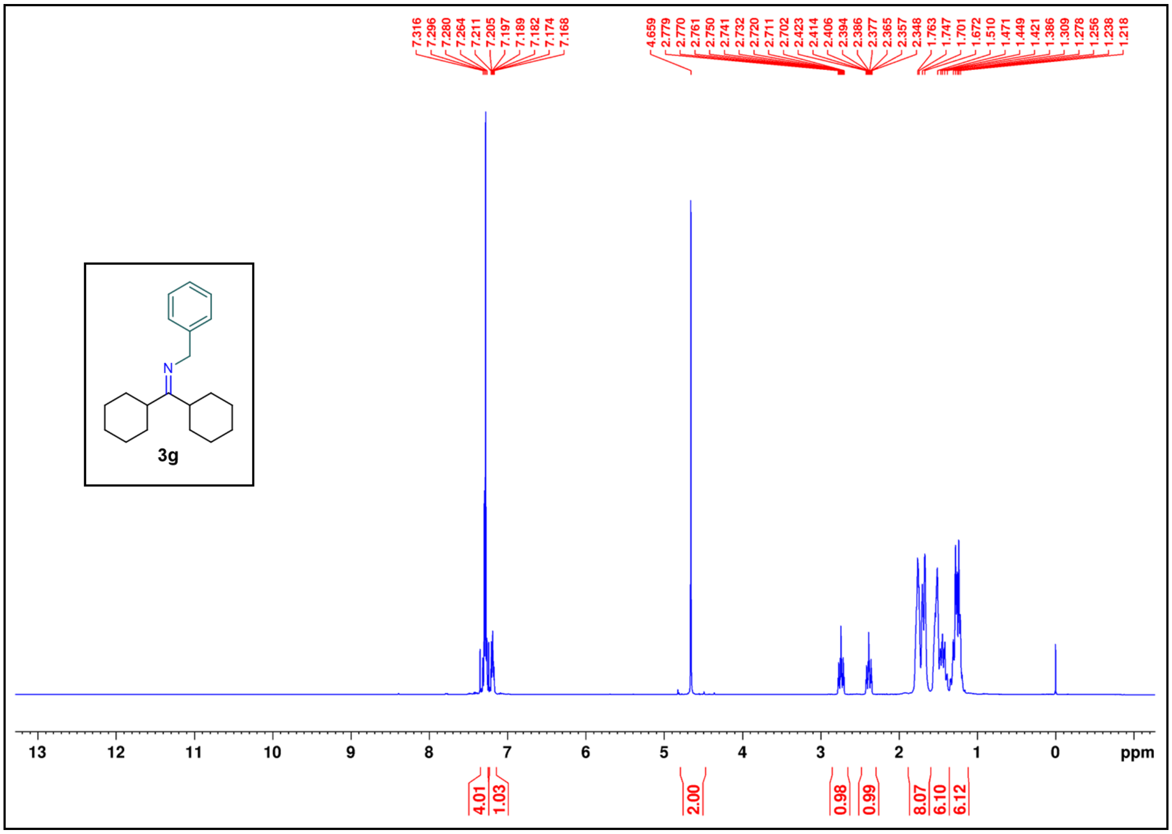

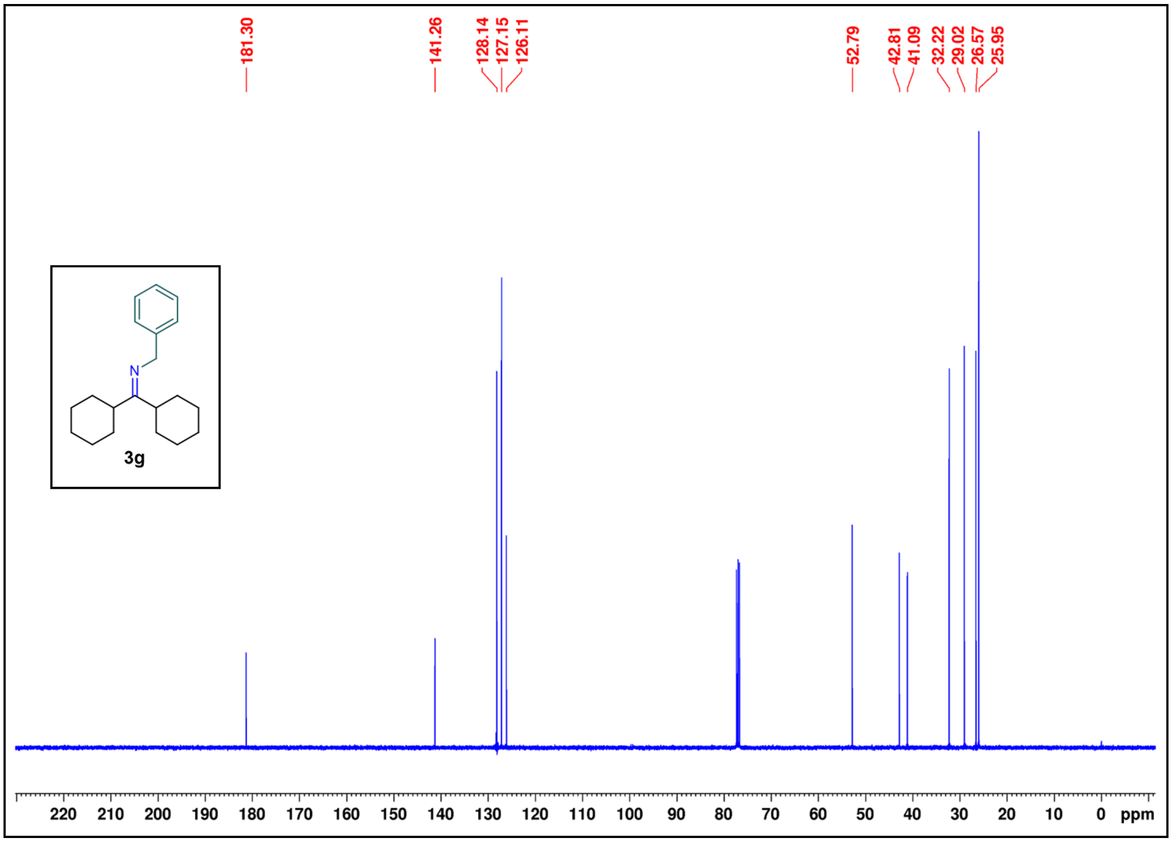

FT-IR (ATR, neat) and HRMS (ESI-positive) spectra for **3g**

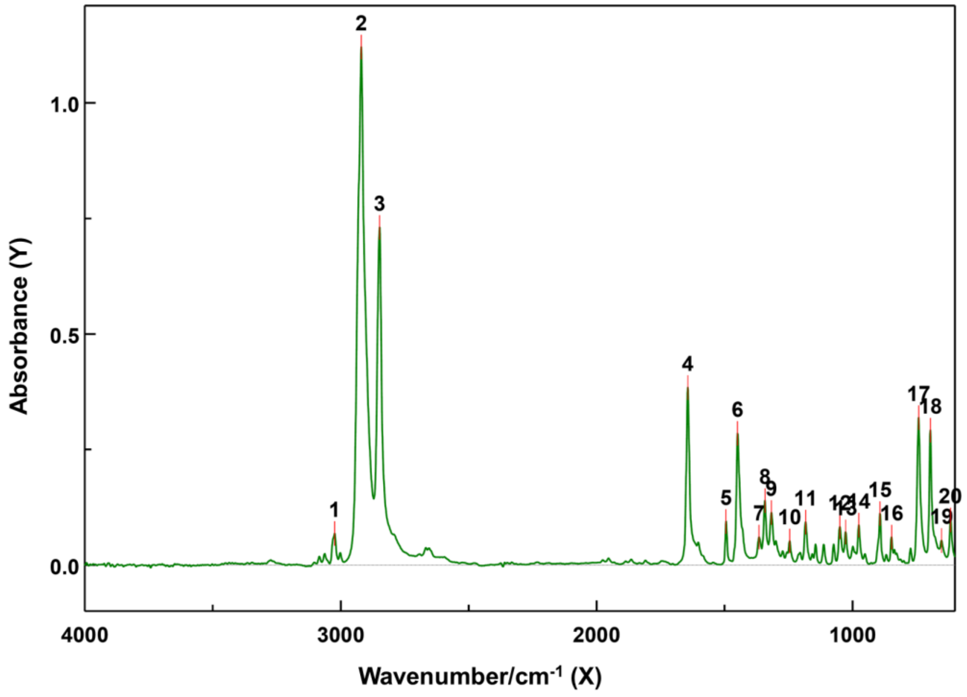

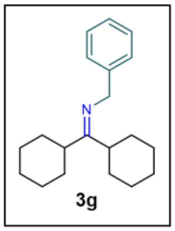

No.	X	Y	No.	X	Y	No.	X	Y
1	3023.8	0.068	8	1342.2	0.139	15	892.9	0.111
2	2919.7	1.12	9	1317.1	0.113	16	847.6	0.06
3	2848.3	0.73	10	1245.8	0.052	17	741.5	0.318
4	1644	0.384	11	1183.1	0.092	18	696.2	0.291
5	1494.6	0.094	12	1050.1	0.082	19	652.8	0.053
6	1449.2	0.284	13	1026.9	0.071	20	617.1	0.097
7	1365.4	0.06	14	975.8	0.086			

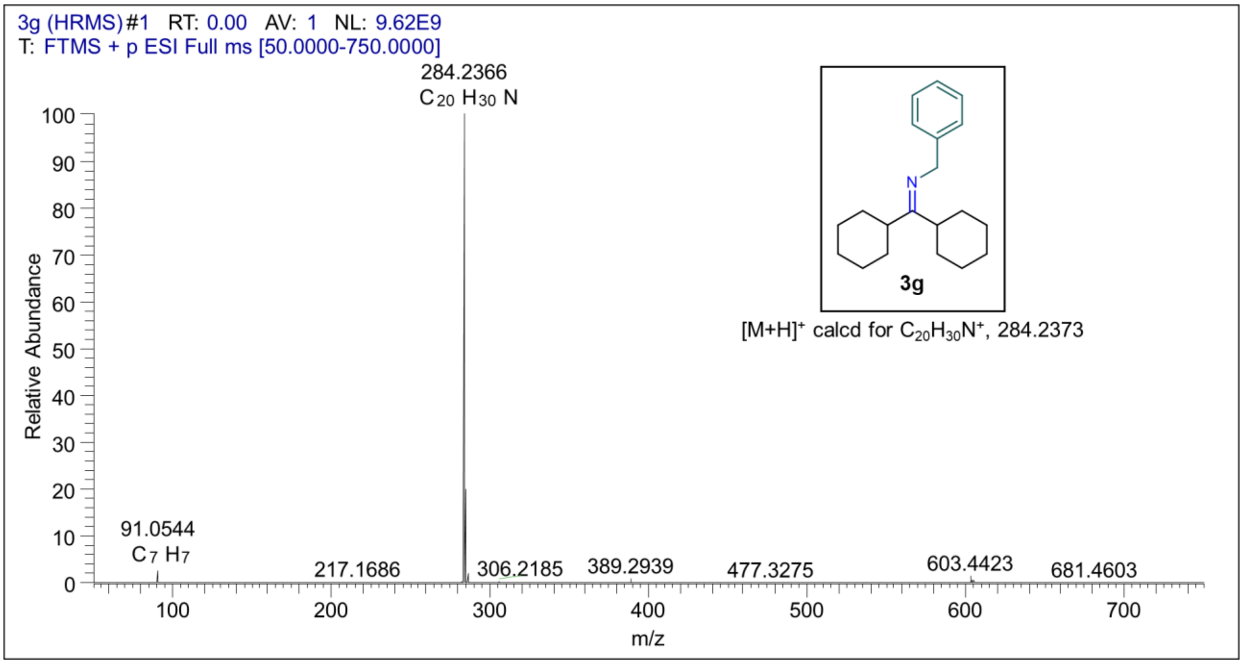

^{1}H NMR (400 MHz, $CDCl_3$) and ^{13}C NMR (100 MHz, $CDCl_3$) spectra for **3h**

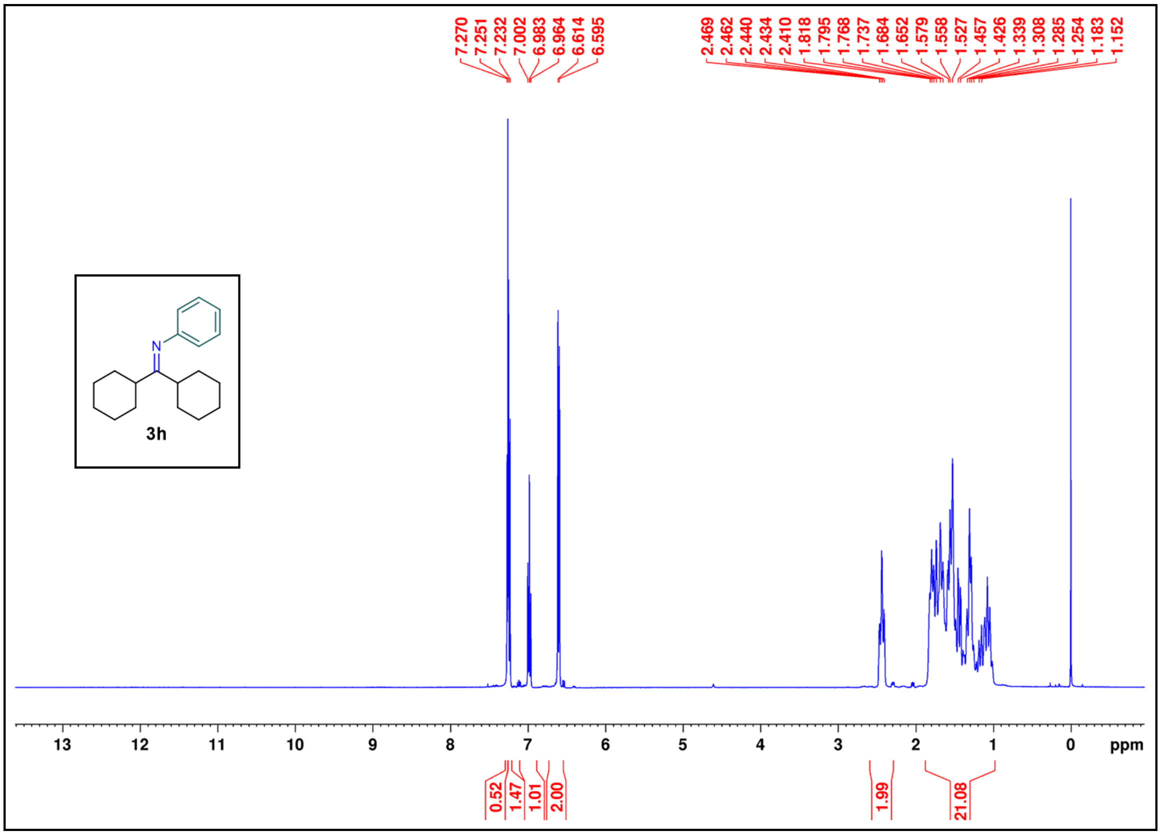

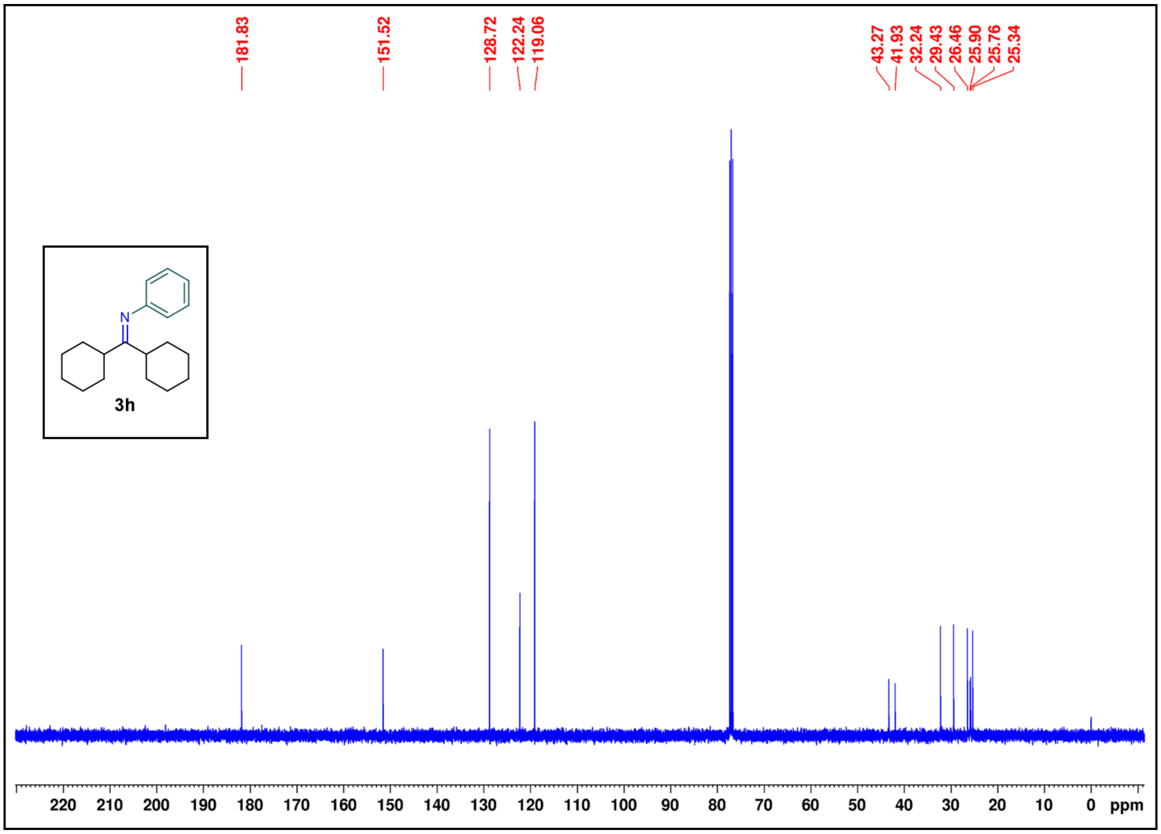

FT-IR (ATR, neat) and HRMS (ESI-positive) spectra for **3h**

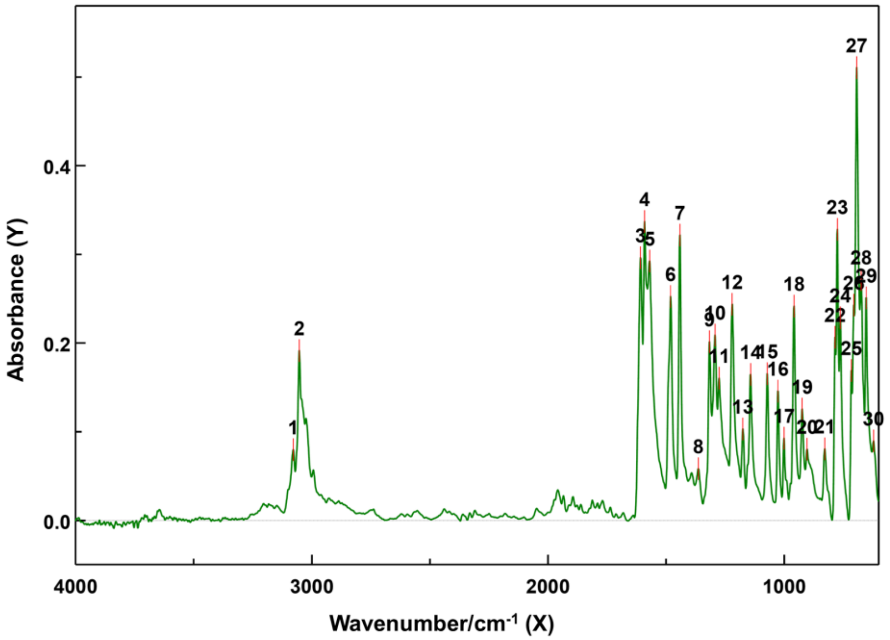

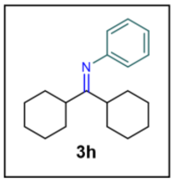

No.	X	Y	No.	X	Y	No.	X	Y
1	3078.8	0.08	11	1275.7	0.16	21	828.3	0.08
2	3052.8	0.191	12	1220.7	0.243	22	784.9	0.206
3	1608.3	0.296	13	1175.4	0.103	23	775.2	0.328
4	1591	0.337	14	1142.6	0.164	24	762.7	0.228
5	1569.8	0.292	15	1072.2	0.165	25	715.5	0.169
6	1481.1	0.252	16	1026.9	0.145	26	705.8	0.243
7	1441.5	0.321	17	1000.9	0.092	27	693.3	0.51
8	1363.4	0.058	18	958.4	0.241	28	674	0.271
9	1316.2	0.201	19	923.7	0.125	29	652.8	0.251
10	1293	0.209	20	903.5	0.08	30	621.9	0.089

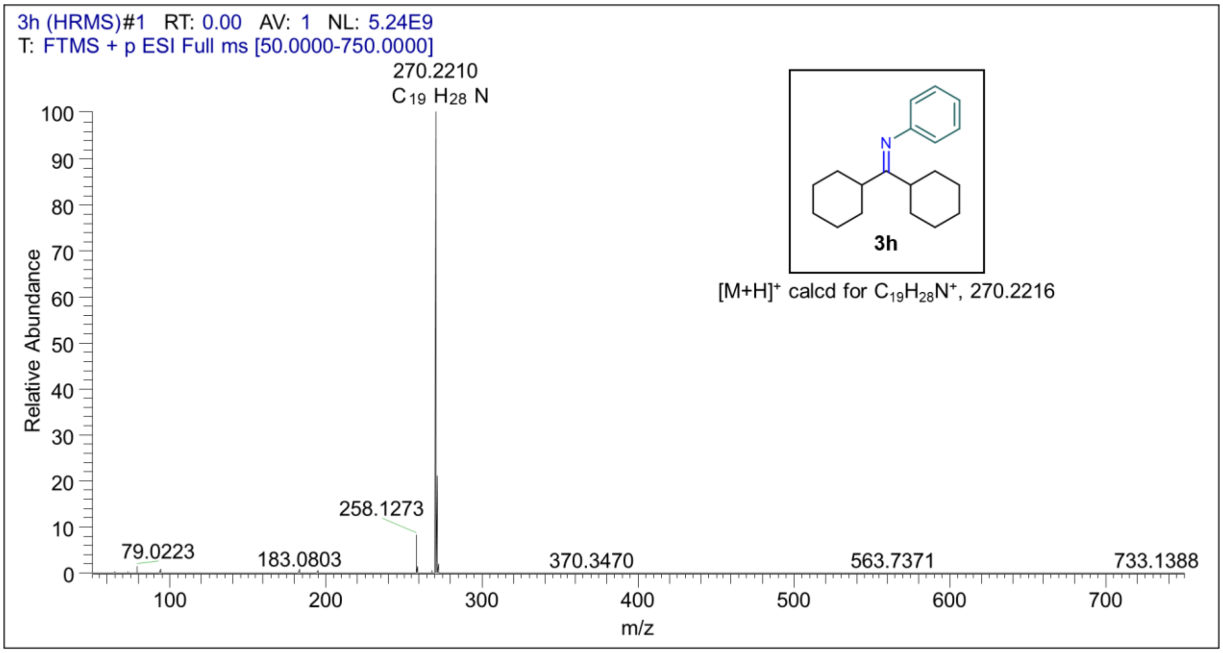

10.3 α-Aminonitrile

^{1}H NMR (400 MHz, $CDCl_3$) and ^{13}C NMR (100 MHz, $CDCl_3$) spectra for **4a**

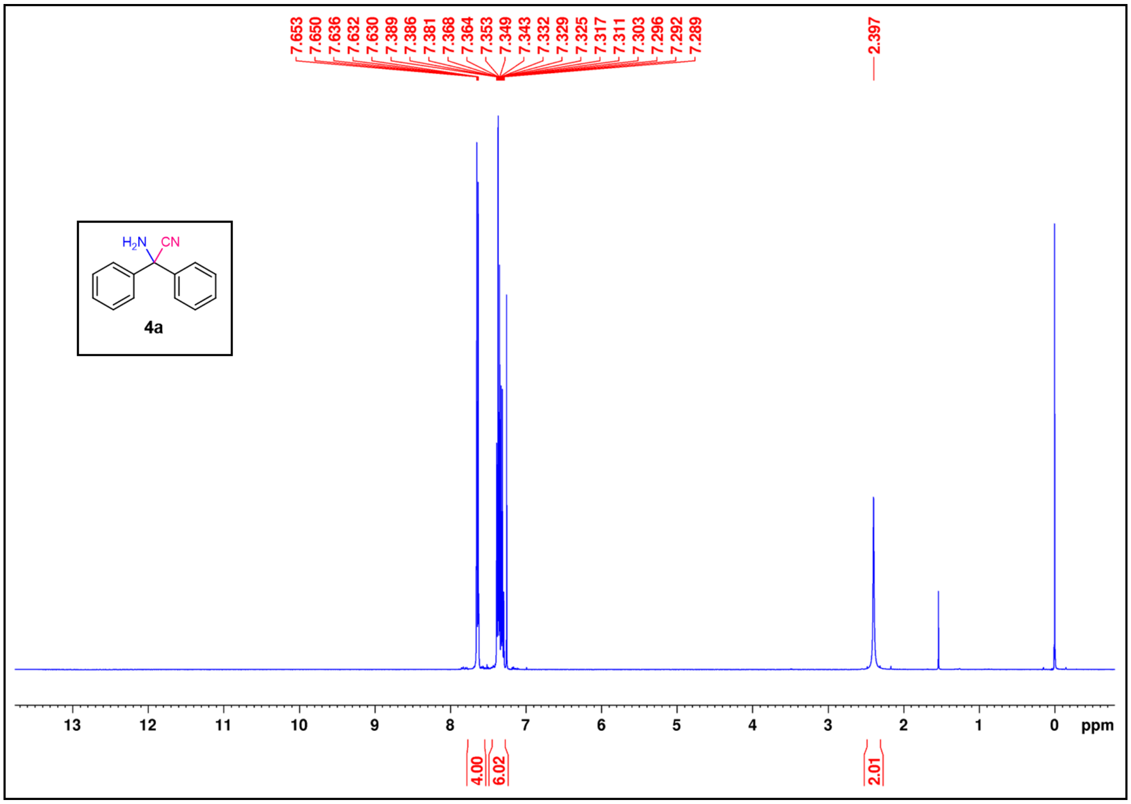

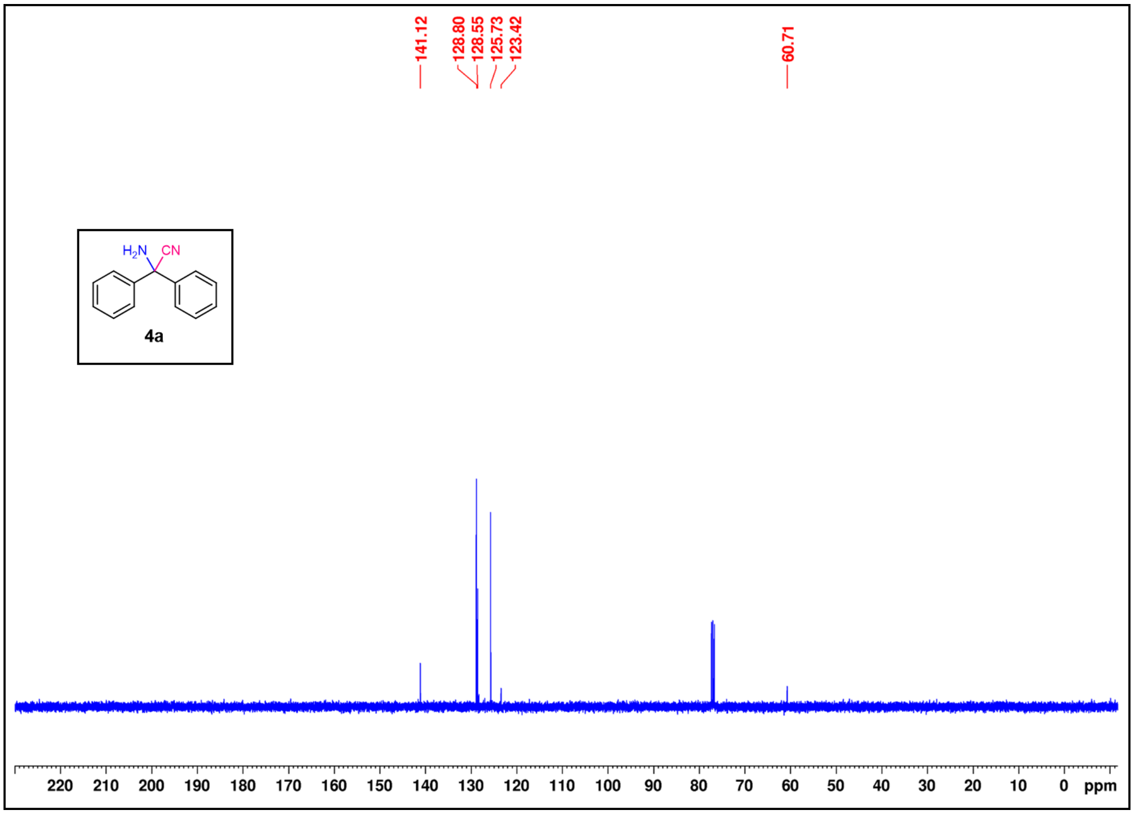

FT-IR (ATR, neat) and HRMS (ESI-positive) spectra for **4a**

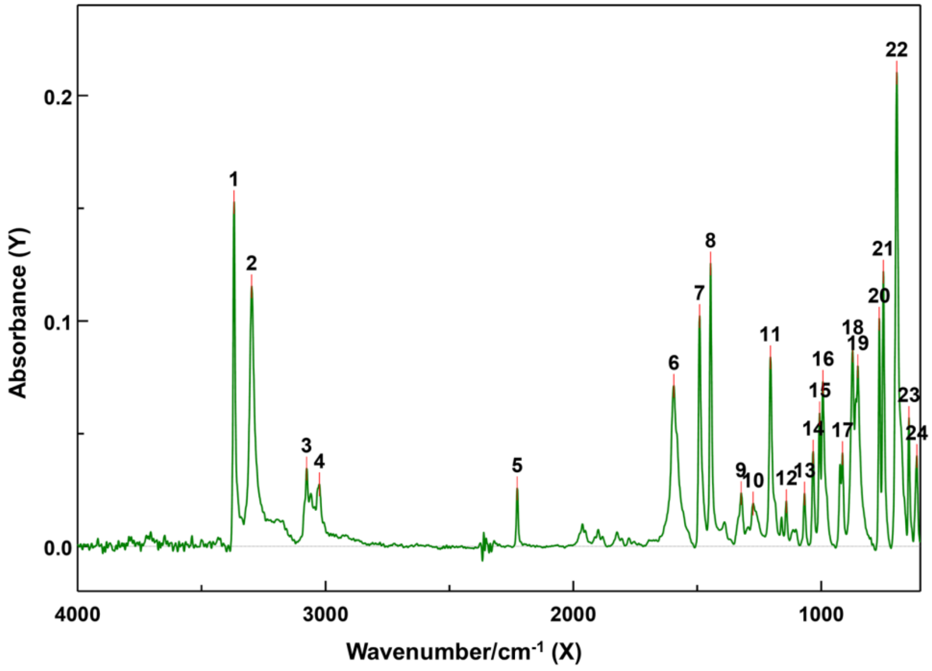

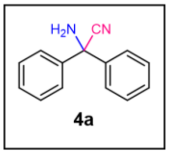

No.	X	Y	No.	X	Y	No.	X	Y
1	3369	0.153	9	1322.9	0.023	17	914.1	0.041
2	3297.7	0.115	10	1274.7	0.019	18	873.6	0.087
3	3075.9	0.034	11	1204.3	0.084	19	852.4	0.08
4	3024.8	0.027	12	1140.7	0.02	20	765.6	0.101
5	2226.4	0.026	13	1067.4	0.023	21	749.2	0.122
6	1594.8	0.071	14	1032.7	0.042	22	695.2	0.21
7	1490.7	0.102	15	1006.7	0.059	23	646	0.057
8	1446.4	0.125	16	993.2	0.073	24	614.2	0.04

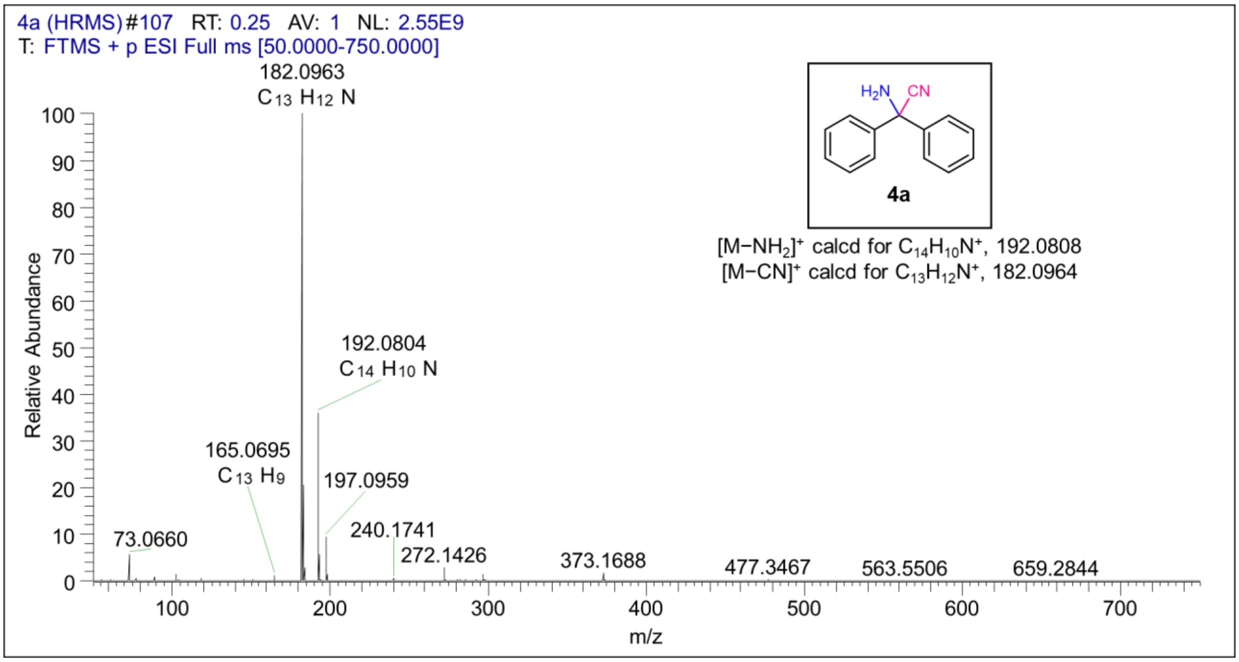

^{1}H NMR (400 MHz, $CDCl_3$) and ^{13}C NMR (100 MHz, $CDCl_3$) spectra for **4b**

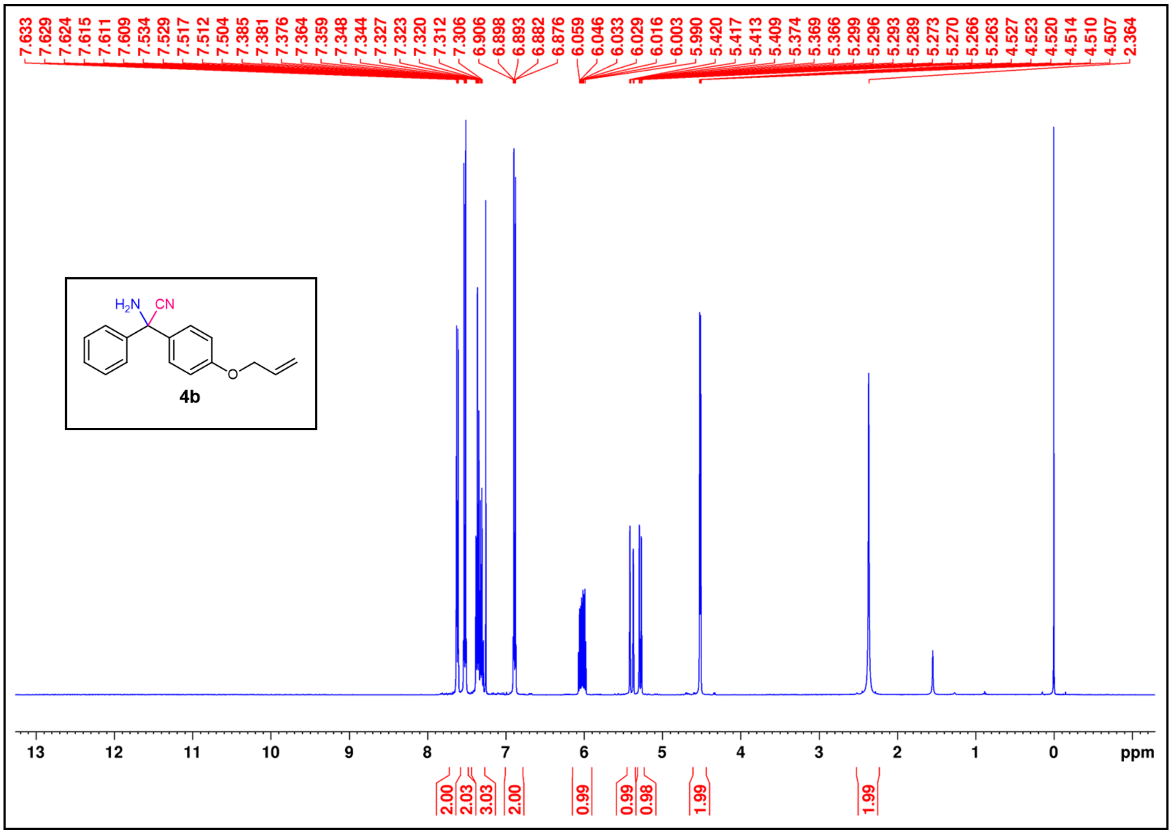

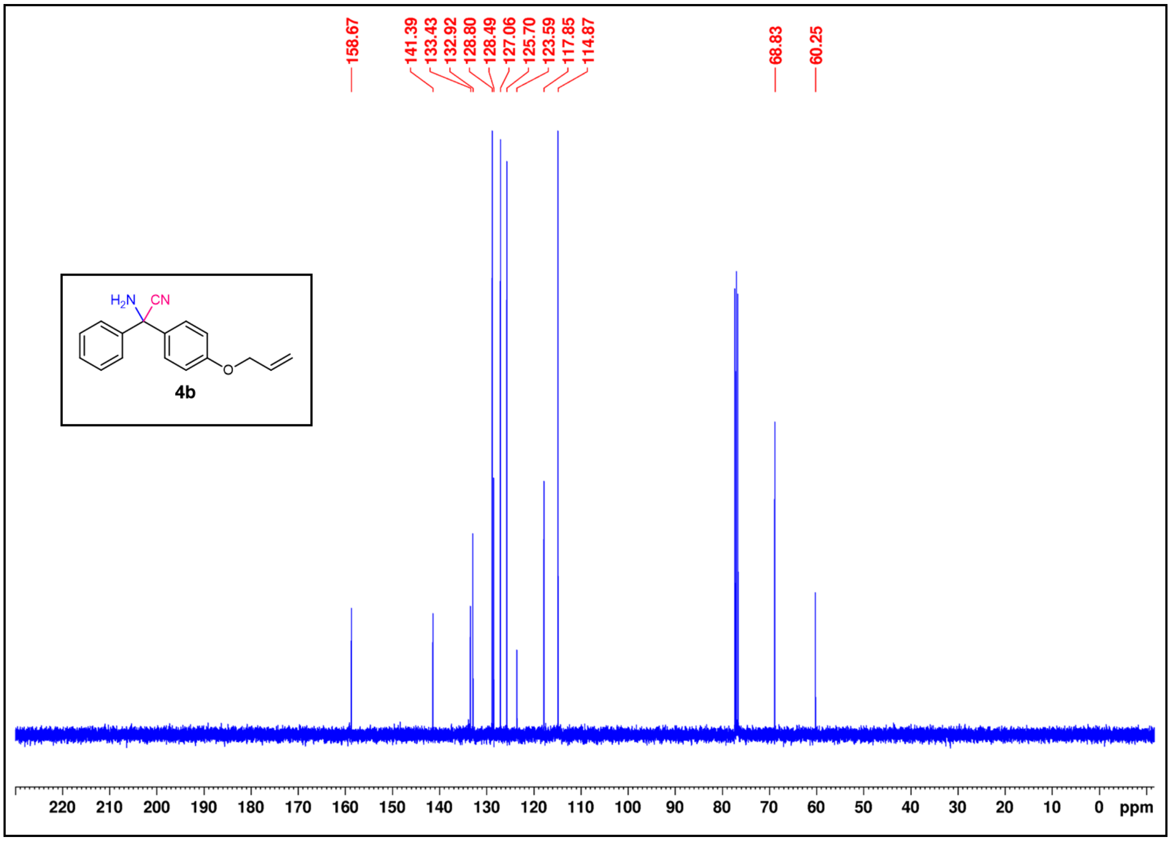

FT-IR (ATR, neat) and HRMS (ESI-positive) spectra for **4b**

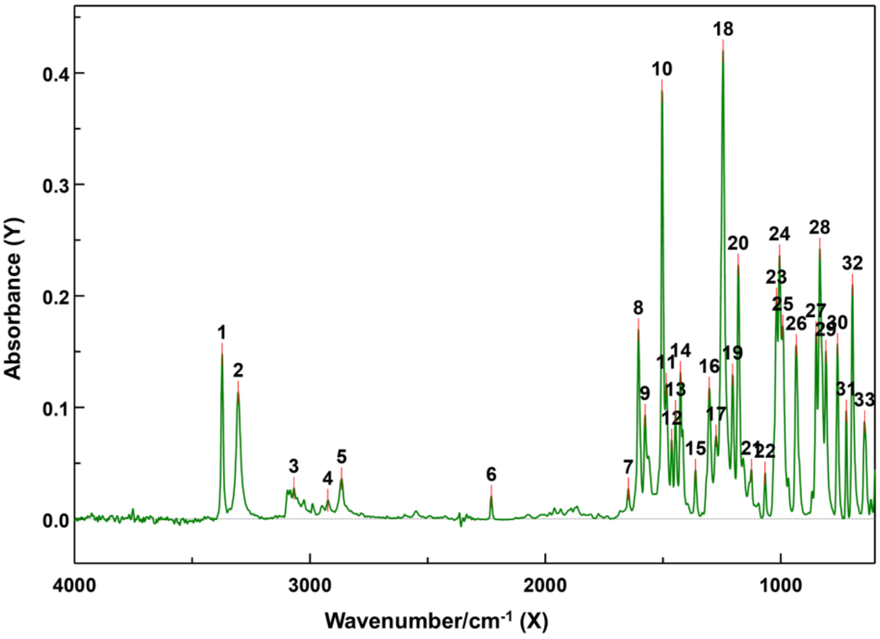

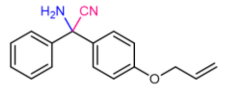

4b

No.	X	Y	No.	X	Y	No.	X	Y
1	3372.9	0.148	12	1463.7	0.071	23	1018	0.197
2	3304.4	0.113	13	1447.3	0.096	24	1005	0.236
3	3068.2	0.027	14	1426.1	0.131	25	991.2	0.173
4	2923.6	0.016	15	1362.5	0.043	26	934.3	0.155
5	2865.7	0.036	16	1303.6	0.117	27	849.5	0.167
6	2229.3	0.02	17	1274.7	0.074	28	834.1	0.242
7	1646.9	0.027	18	1244.8	0.42	29	808	0.15
8	1604.5	0.17	19	1204.3	0.129	30	758.9	0.156
9	1576.5	0.093	20	1180.2	0.228	31	721.2	0.097
10	1504.2	0.384	21	1124.3	0.044	32	695.2	0.21
11	1485.9	0.121	22	1066.4	0.041	33	644.1	0.087

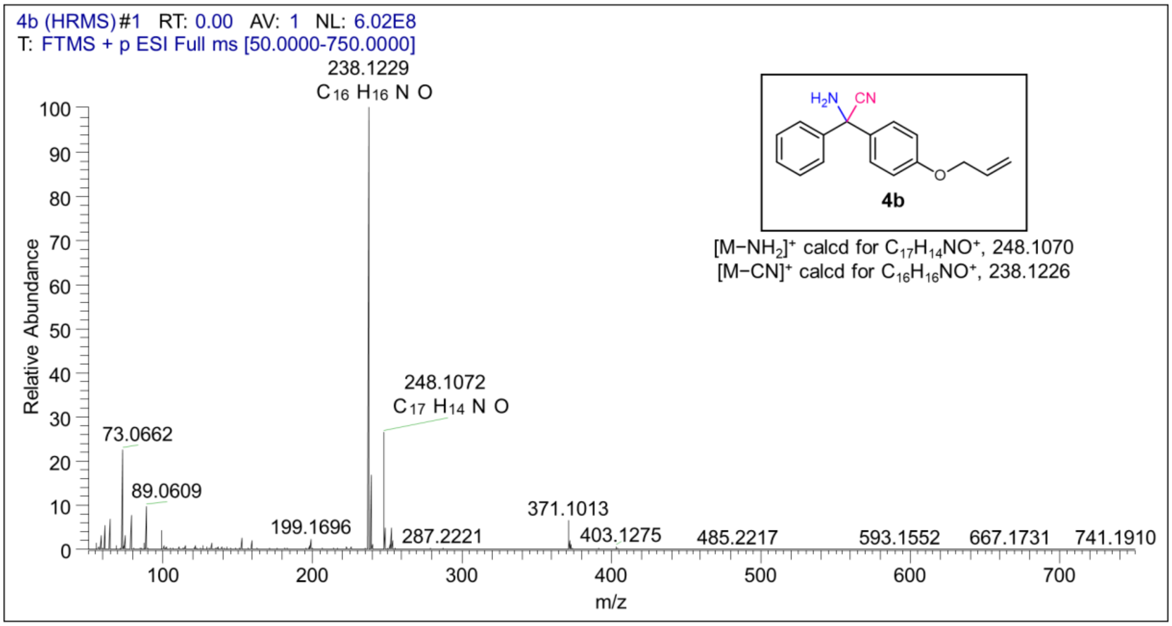

$[M-NH_2]^+$ calcd for $C_{17}H_{14}NO^+$, 248.1070
$[M-CN]^+$ calcd for $C_{16}H_{16}NO^+$, 238.1226

^1H NMR (400 MHz, $CDCl_3$) and ^{13}C NMR (100 MHz, $CDCl_3$) spectra for **4c**

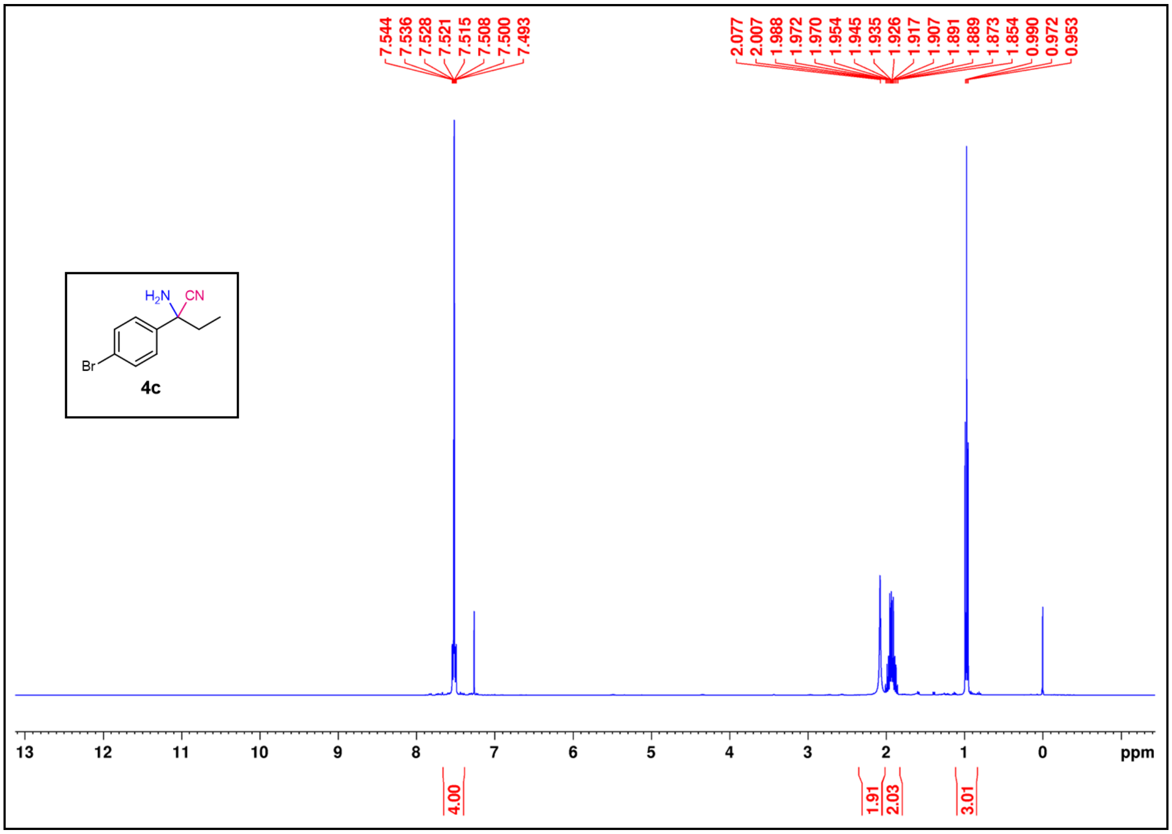

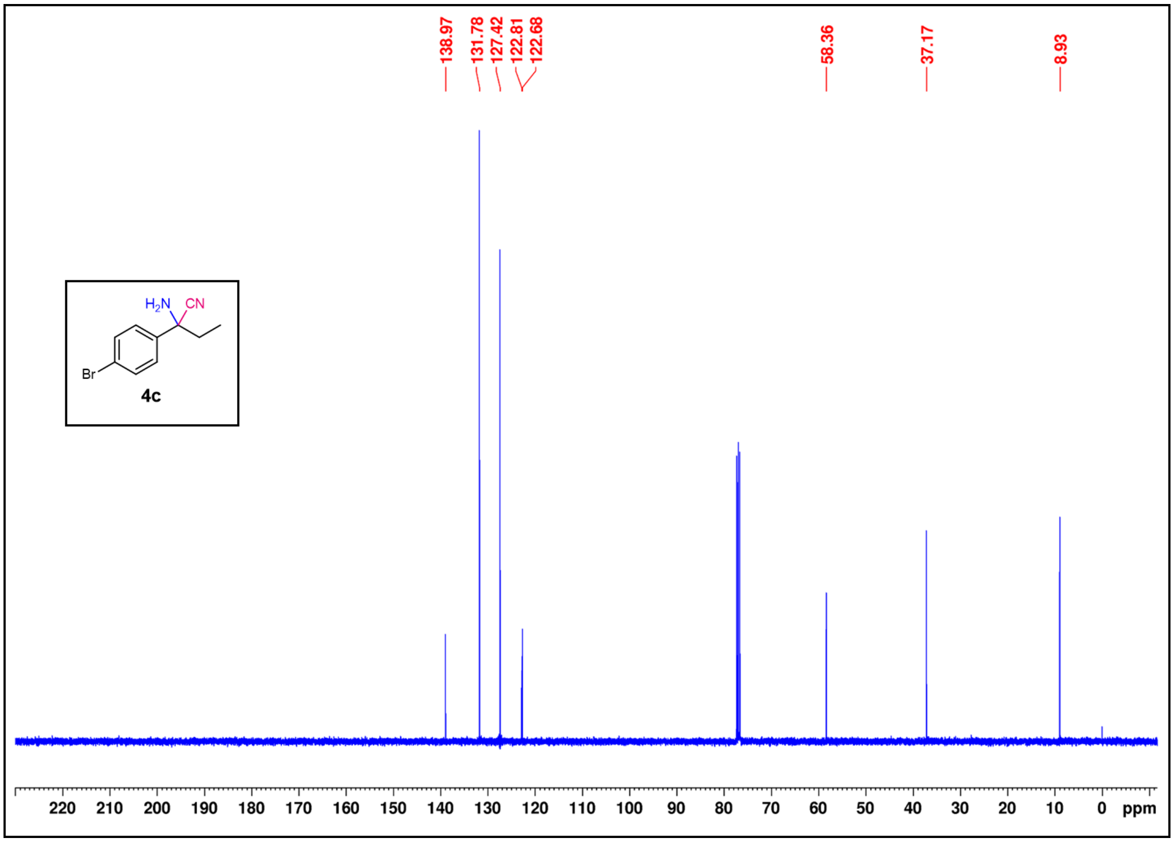

FT-IR (ATR, neat) and HRMS (ESI-positive) spectra for **4c**

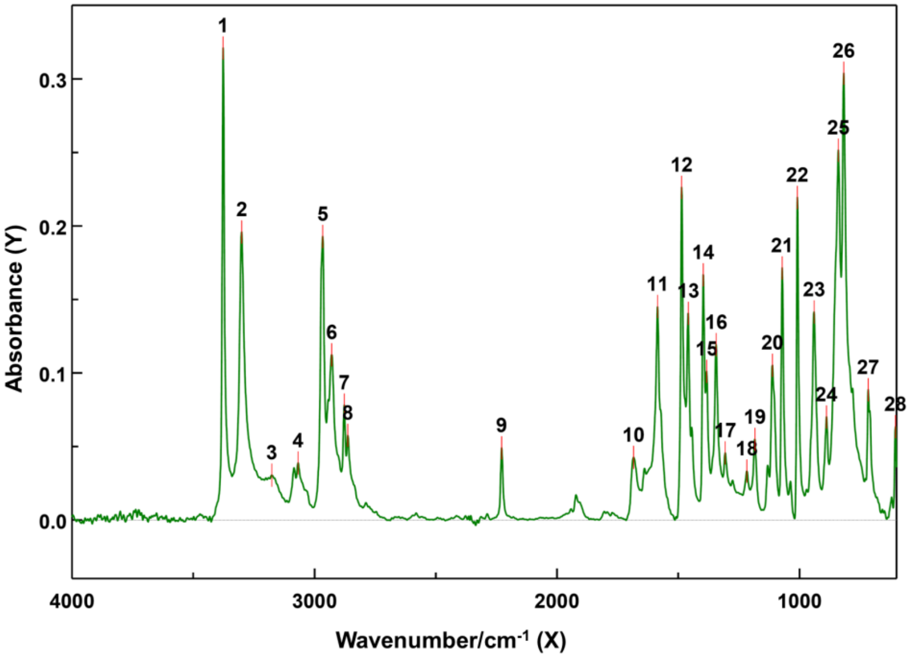

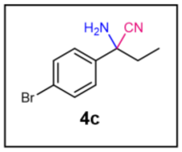

No.	X	Y	No.	X	Y	No.	X	Y
1	3376.7	0.321	11	1585.2	0.145	21	1071	0.171
2	3300.6	0.196	12	1485.9	0.226	22	1009	0.219
3	3176.2	0.03	13	1458.9	0.14	23	940.1	0.141
4	3068.2	0.039	14	1397.2	0.167	24	889	0.07
5	2966	0.193	15	1382.7	0.101	25	839.8	0.251
6	2929.3	0.112	16	1344.1	0.119	26	817.7	0.304
7	2877.3	0.078	17	1306.5	0.045	27	716.4	0.089
8	2862.8	0.057	18	1216.9	0.033	28	604.6	0.063
9	2228.3	0.049	19	1184.1	0.055			
10	1684.5	0.042	20	1111.8	0.105			

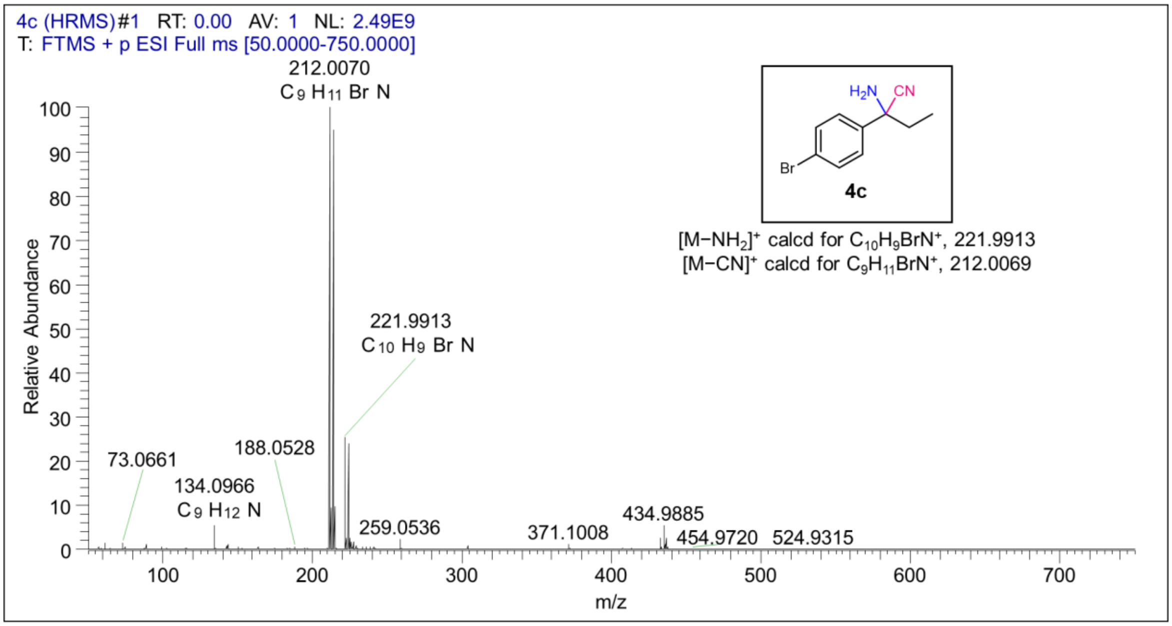

^{1}H NMR (400 MHz, $CDCl_3$) and ^{13}C NMR (100 MHz, $CDCl_3$) spectra for **4d**

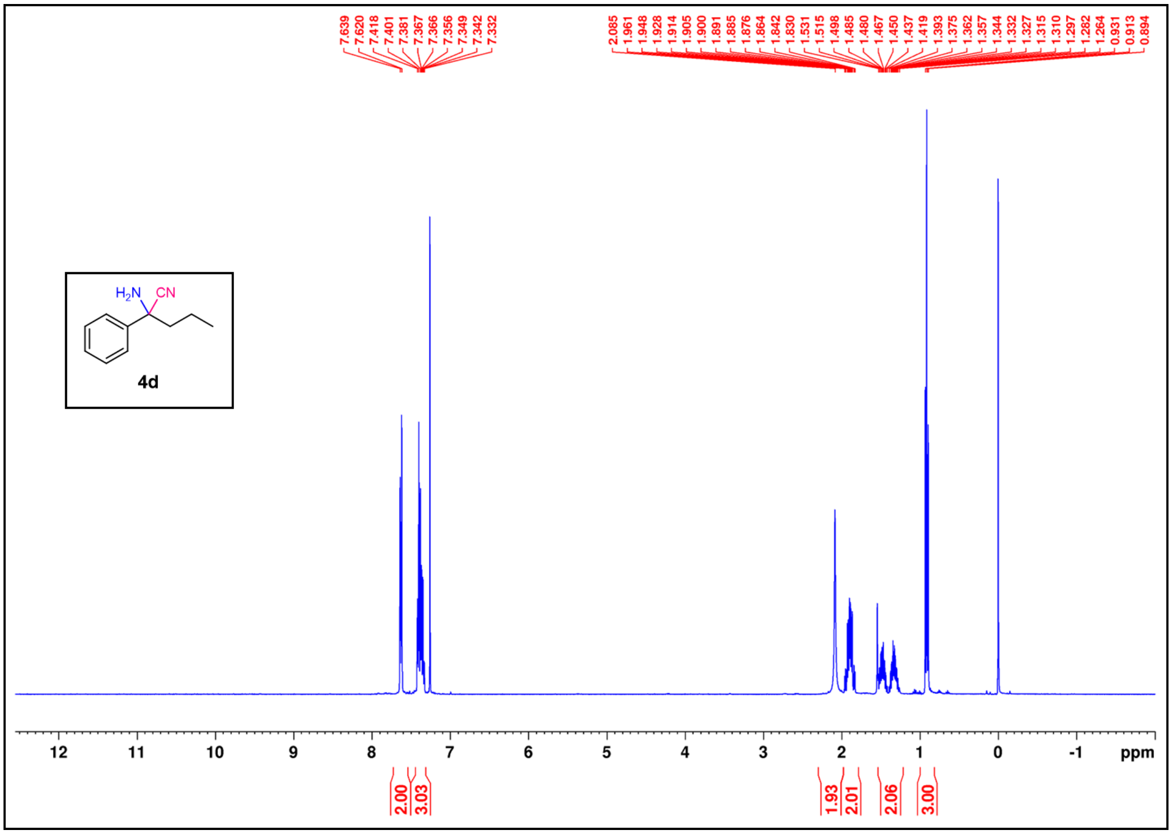

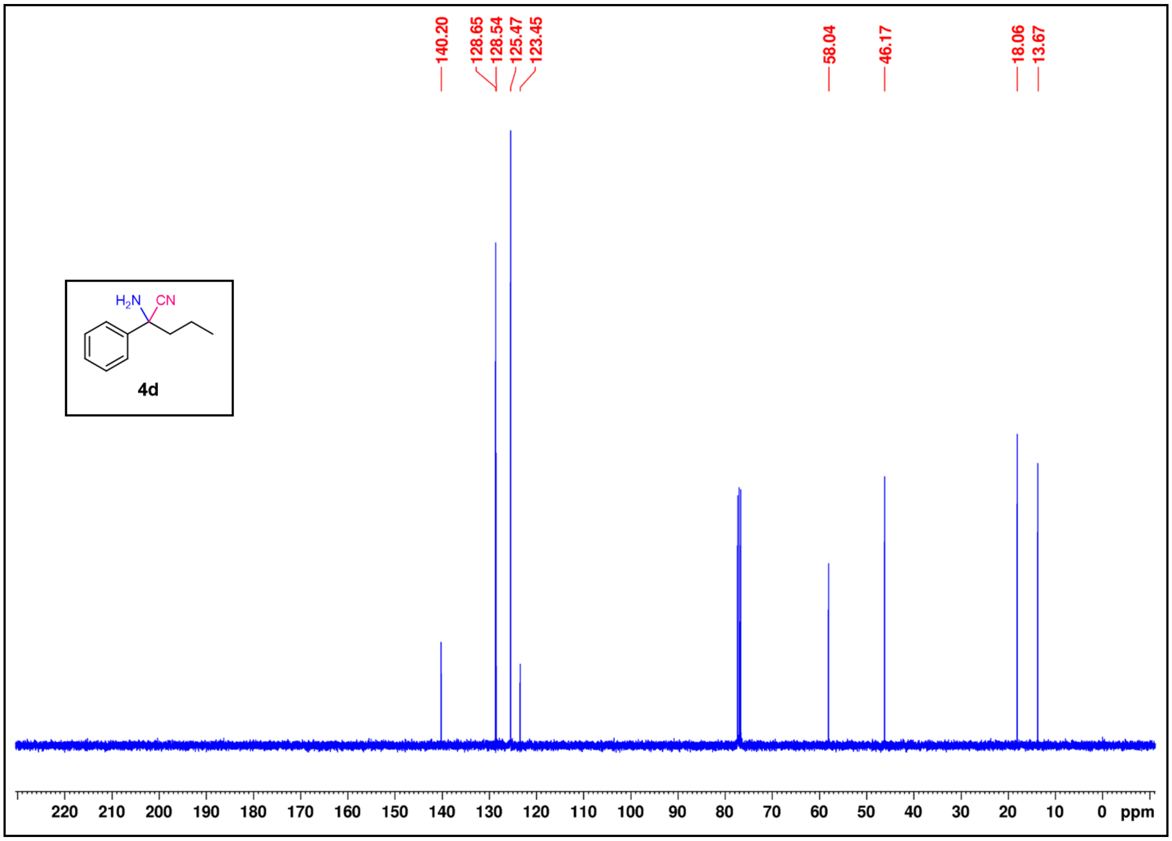

FT-IR (ATR, neat) and HRMS (ESI-positive) spectra for **4d**

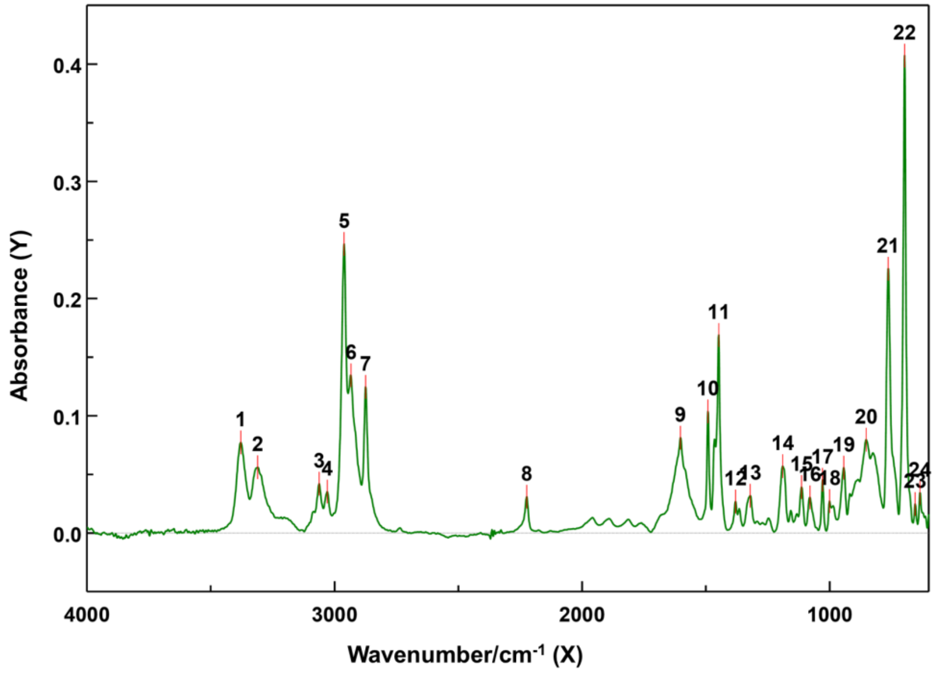

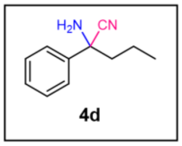

No.	X	Y	No.	X	Y	No.	X	Y
1	3378.7	0.077	9	1602.6	0.081	17	1029	0.045
2	3310.2	0.056	10	1491.7	0.104	18	1001	0.027
3	3062.4	0.042	11	1448.3	0.169	19	943	0.056
4	3029.6	0.035	12	1380.8	0.027	20	852.4	0.079
5	2961.2	0.246	13	1321	0.032	21	763.7	0.225
6	2934.2	0.134	14	1189.9	0.057	22	697.1	0.407
7	2874.4	0.125	15	1113.7	0.039	23	654.7	0.025
8	2223.5	0.031	16	1079.9	0.03	24	635.4	0.034

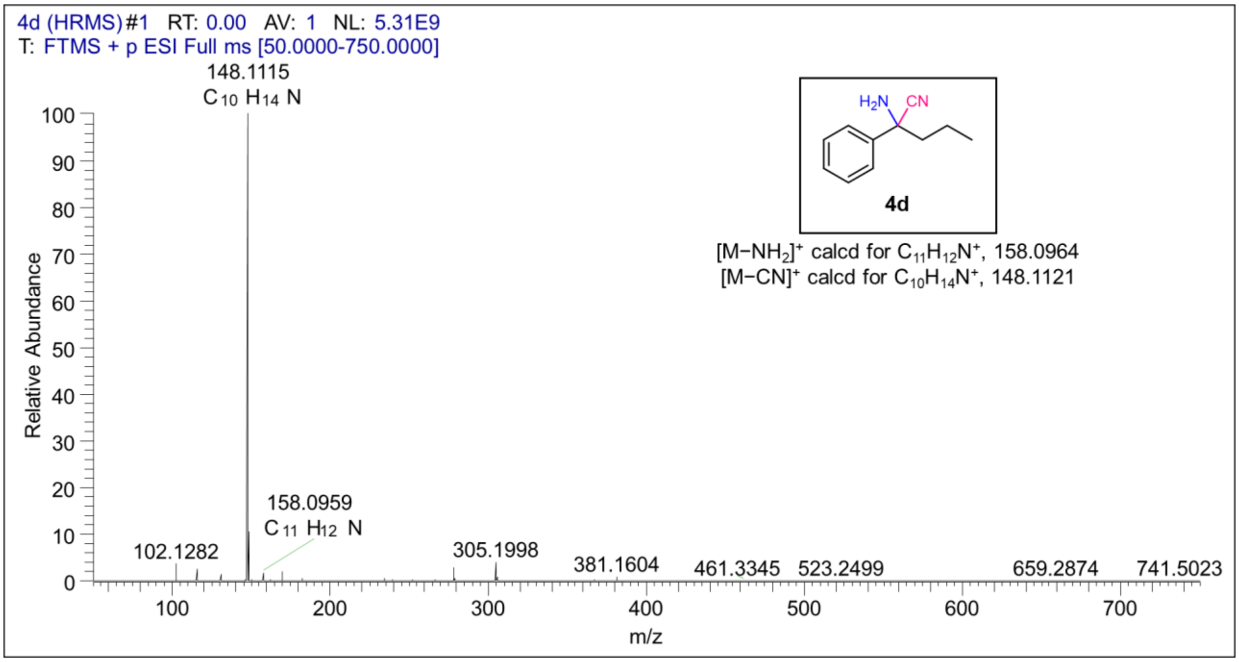

^{1}H NMR (400 MHz, $CDCl_3$) and ^{13}C NMR (100 MHz, $CDCl_3$) spectra for **4e**

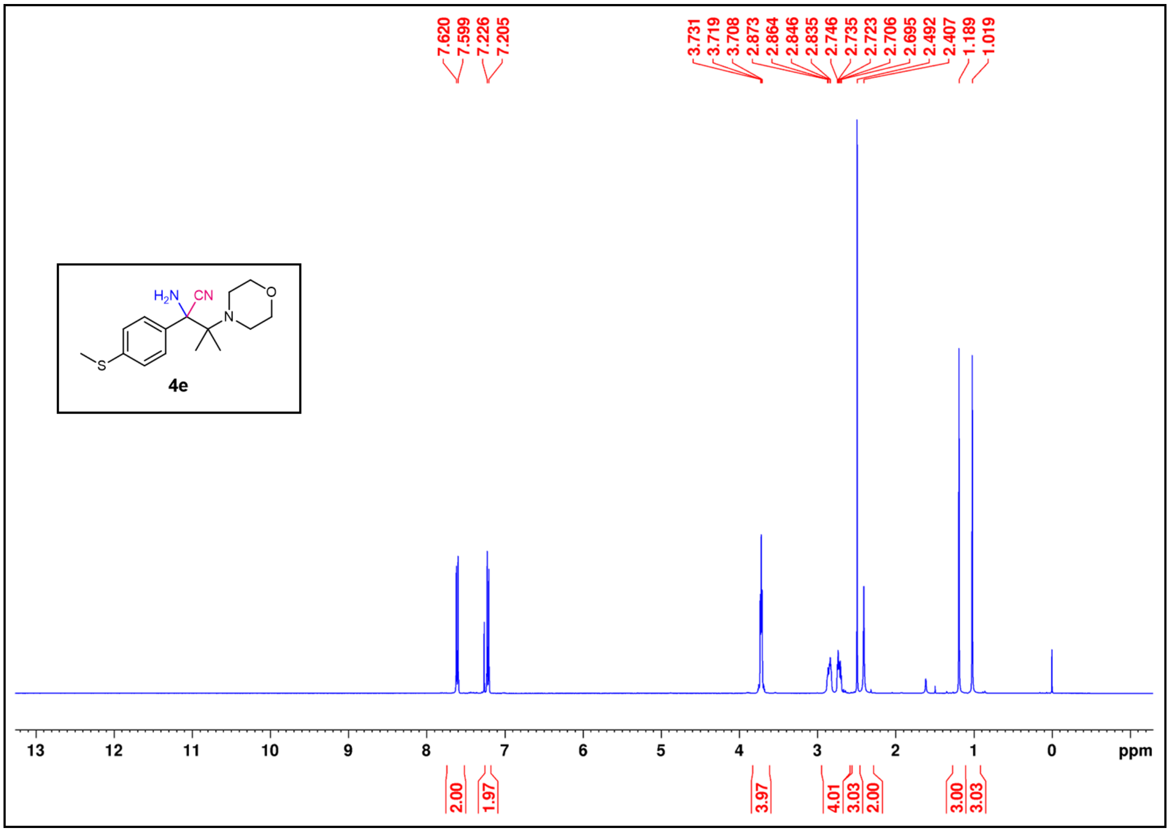

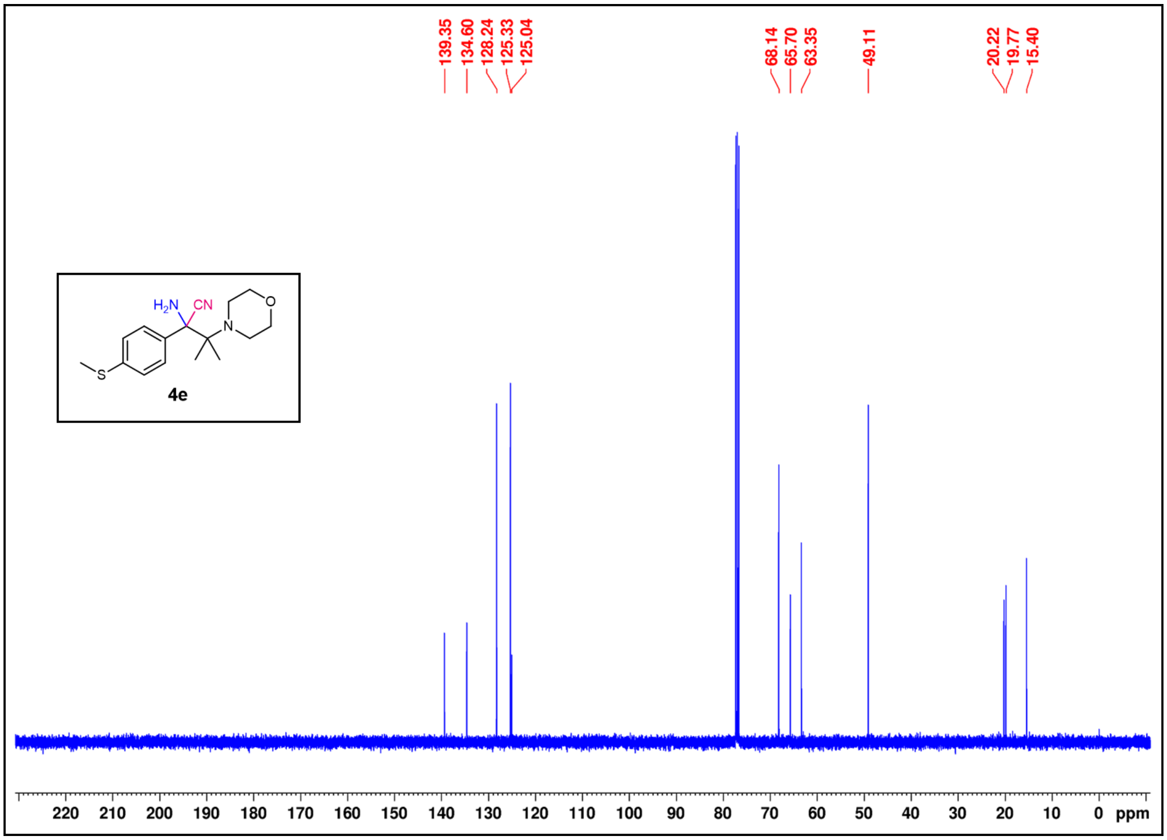

FT-IR (ATR, neat) and HRMS (ESI-positive) spectra for **4e**

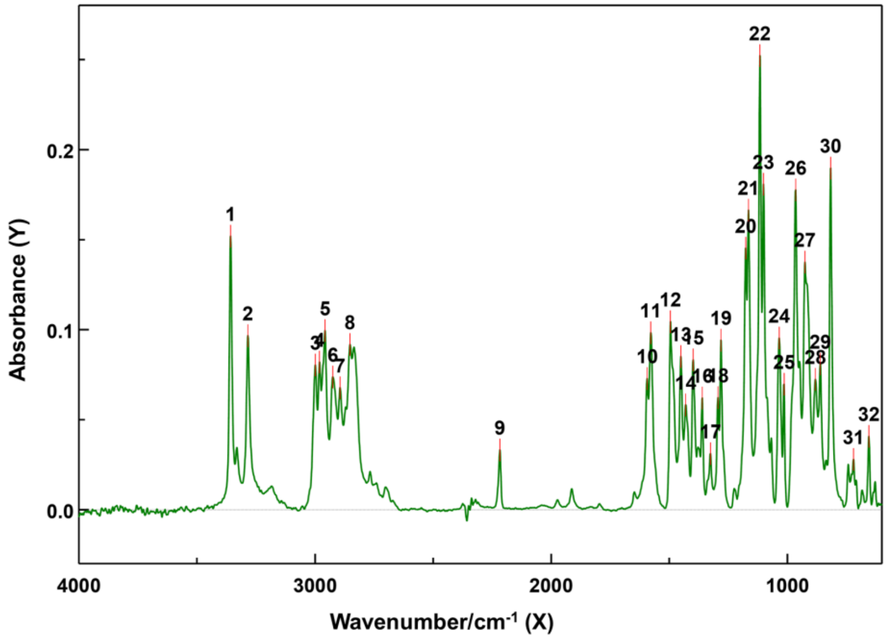

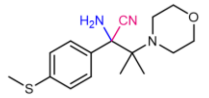

4e

No.	X	Y	No.	X	Y	No.	X	Y
1	3357.5	0.152	12	1495.5	0.104	23	1101	0.181
2	3284.2	0.097	13	1451.2	0.085	24	1035	0.095
3	2998.8	0.08	14	1430.9	0.058	25	1014	0.07
4	2981.4	0.082	15	1399.1	0.083	26	965.2	0.177
5	2958.3	0.099	16	1360.5	0.062	27	925.7	0.137
6	2924.5	0.074	17	1325.8	0.031	28	881.3	0.072
7	2893.7	0.068	18	1294	0.062	29	861.1	0.081
8	2852.2	0.092	19	1281.5	0.094	30	816.7	0.19
9	2217.7	0.033	20	1176.4	0.145	31	720.3	0.028
10	1593.9	0.073	21	1164.8	0.166	32	654.7	0.041
11	1578.5	0.098	22	1116.6	0.252			

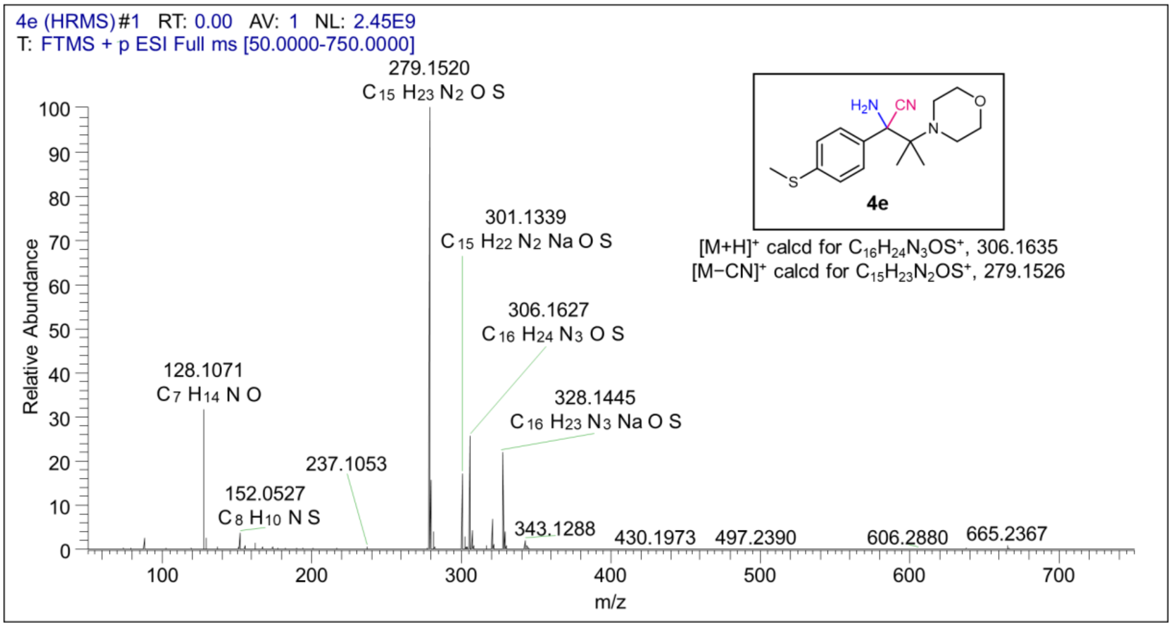

^{1}H NMR (400 MHz, $CDCl_3$) and ^{13}C NMR (100 MHz, $CDCl_3$) spectra for **4f**

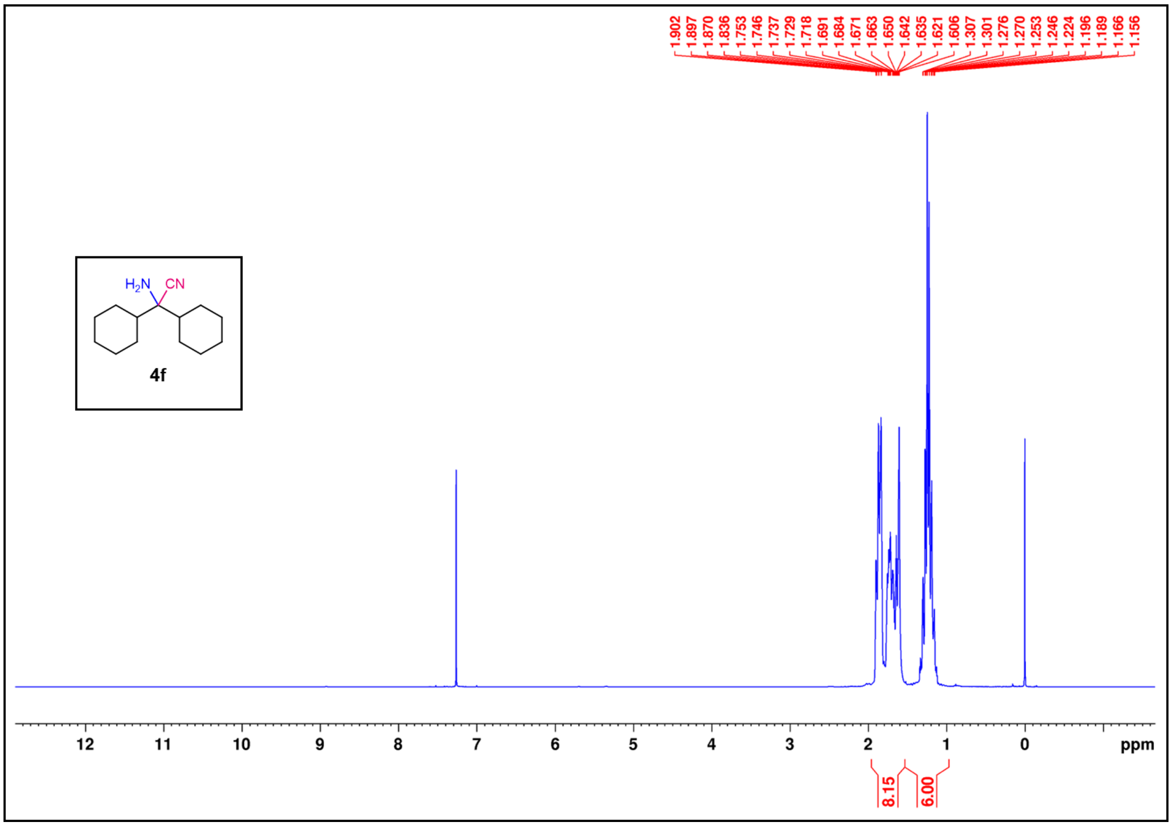

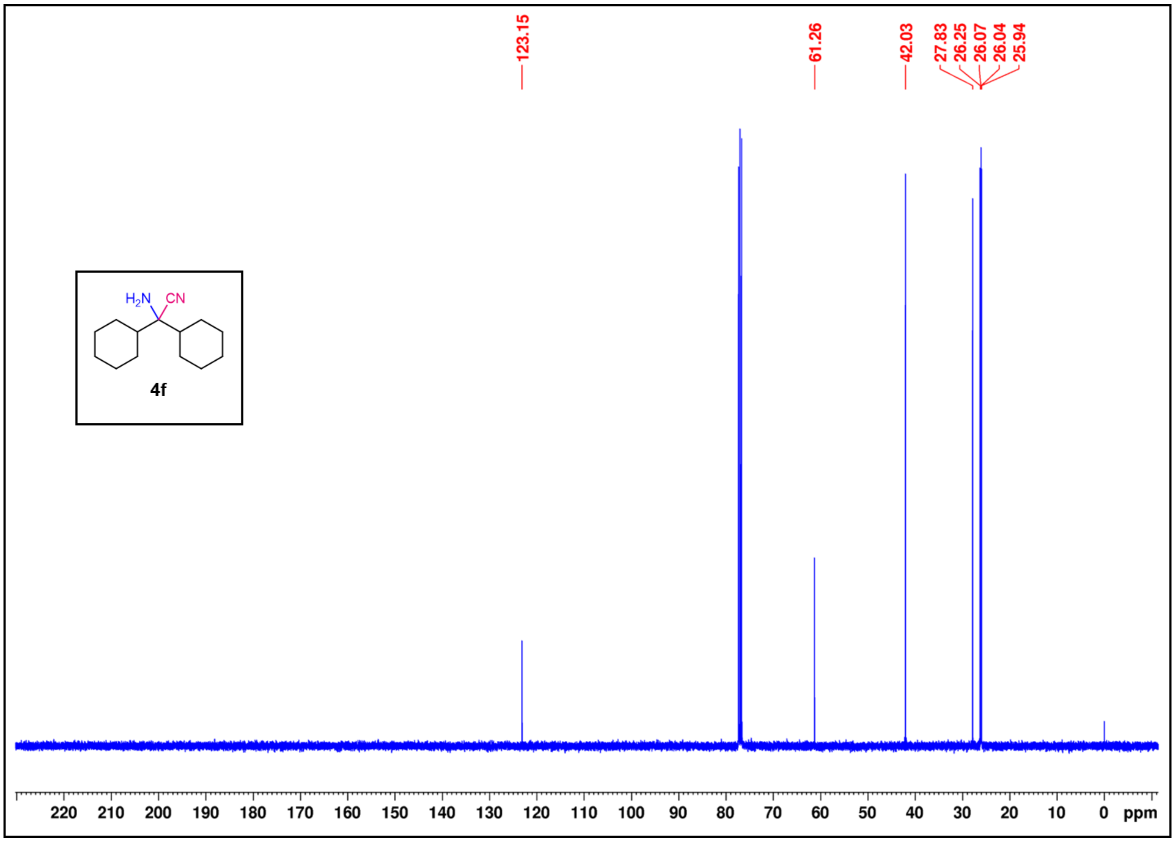

FT-IR (ATR, neat) and HRMS (ESI-positive) spectra for **4f**

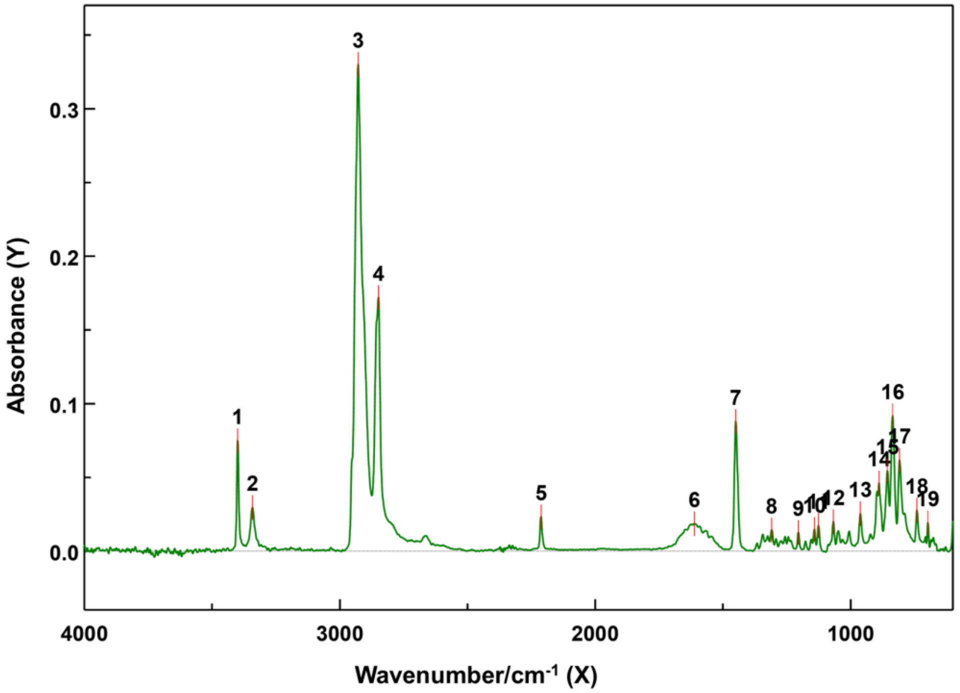

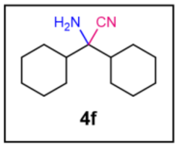

No.	X	Y	No.	X	Y	No.	X	Y
1	3399.9	0.075	8	1309.4	0.014	15	856.2	0.054
2	3342	0.029	9	1204.3	0.013	16	836	0.092
3	2928.4	0.33	10	1141.7	0.015	17	809	0.062
4	2848.3	0.172	11	1126.2	0.017	18	740.5	0.028
5	2212	0.023	12	1068.4	0.02	19	698.1	0.019
6	1612.2	0.018	13	962.3	0.025			
7	1450.2	0.088	14	889	0.046			

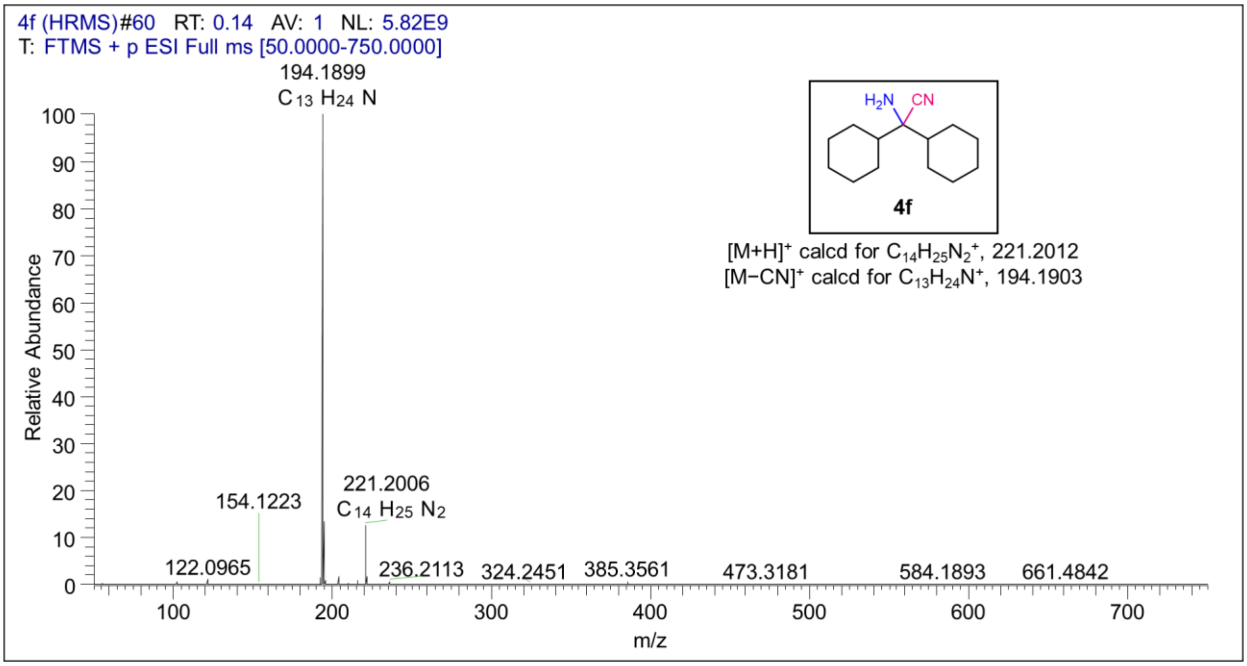

^{1}H NMR (400 MHz, $CDCl_3$) and ^{13}C NMR (100 MHz, $CDCl_3$) spectra for **4g**

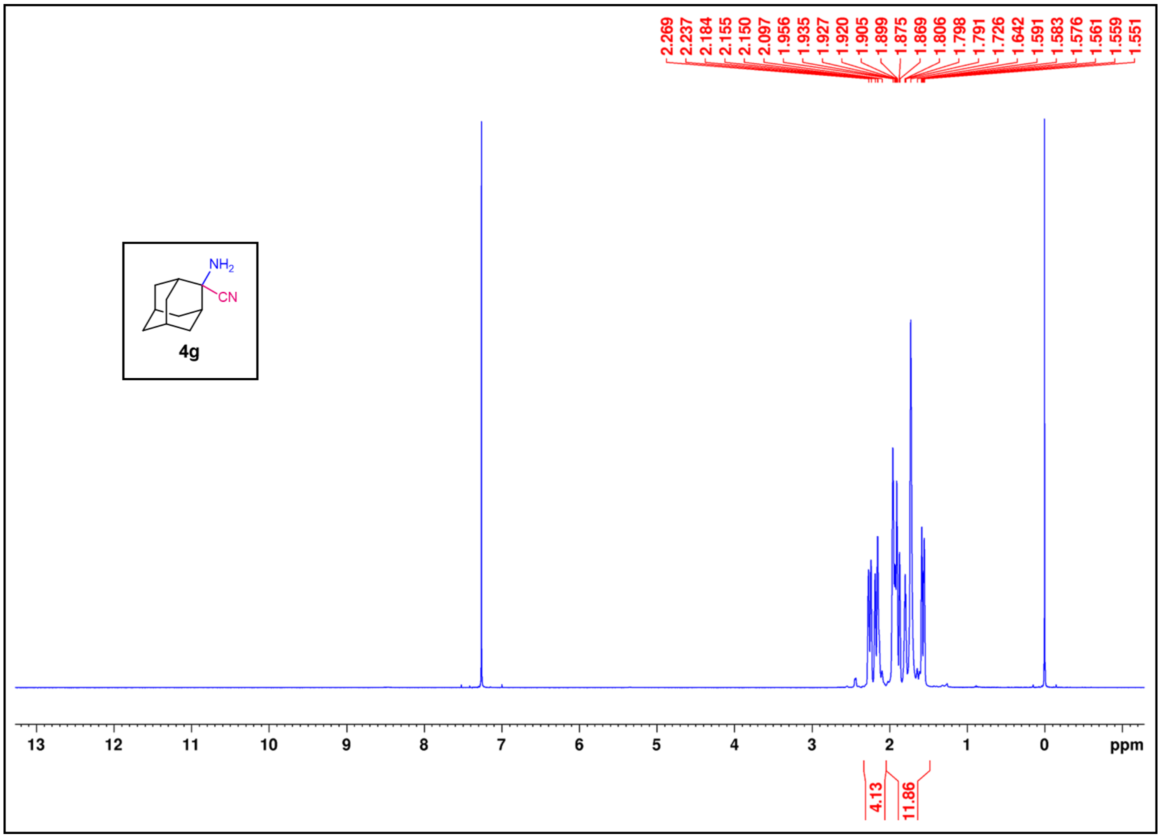

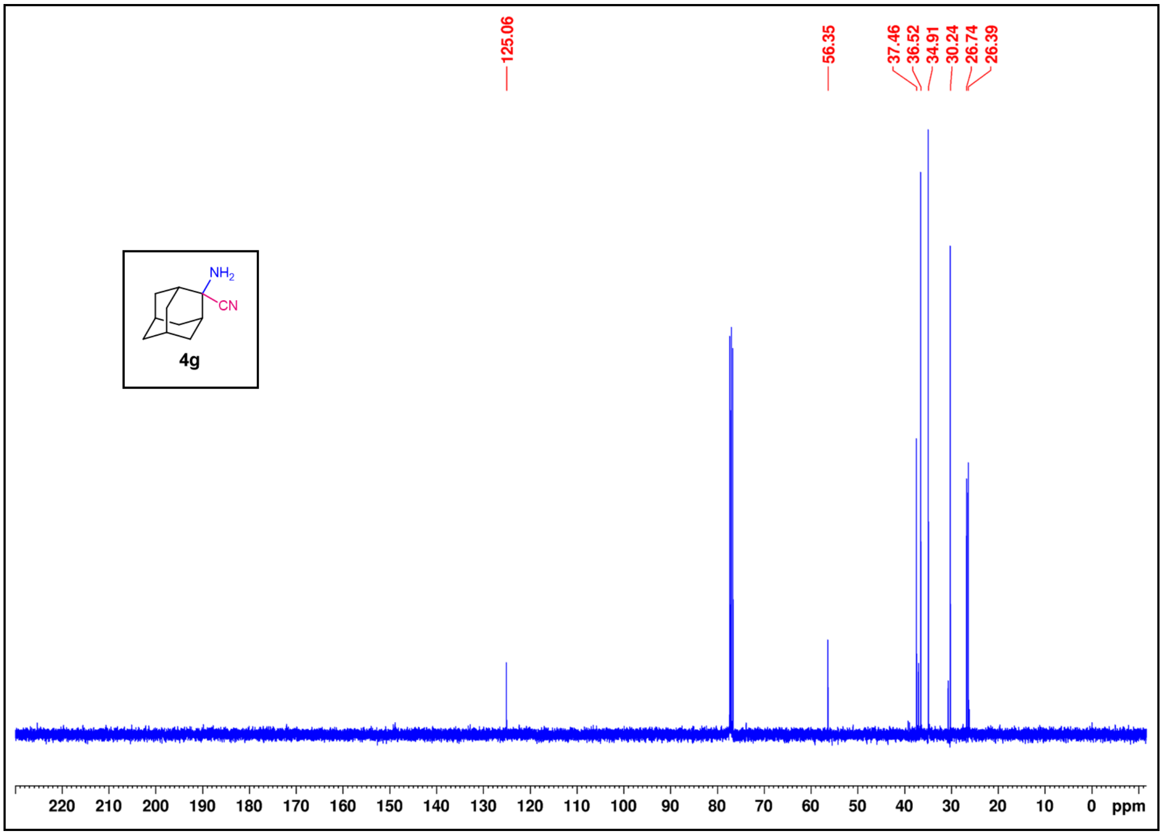

FT-IR (ATR, neat) and HRMS (ESI-positive) spectra for **4g**

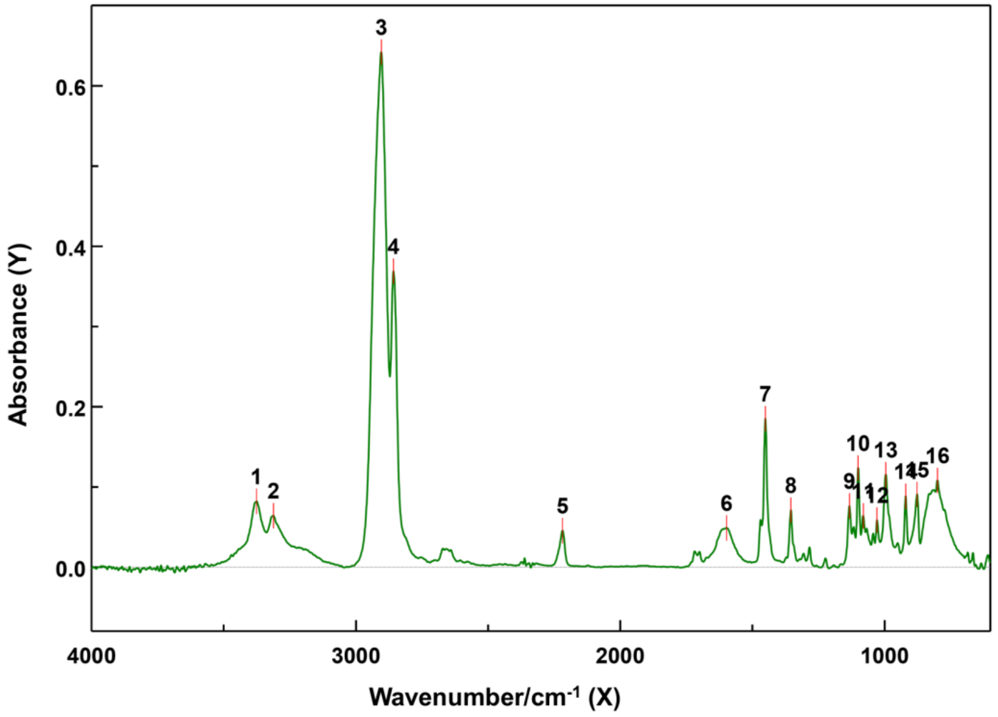

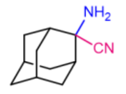

4g

No.	X	Y	No.	X	Y	No.	X	Y
1	3375.8	0.082	7	1451.2	0.185	13	996.1	0.115
2	3312.1	0.064	8	1354.7	0.071	14	919.9	0.088
3	2904.3	0.642	9	1133	0.076	15	877.5	0.09
4	2858	0.369	10	1100.2	0.123	16	800.3	0.108
5	2218.7	0.045	11	1080.9	0.064			
6	1597.7	0.049	12	1028.8	0.058			

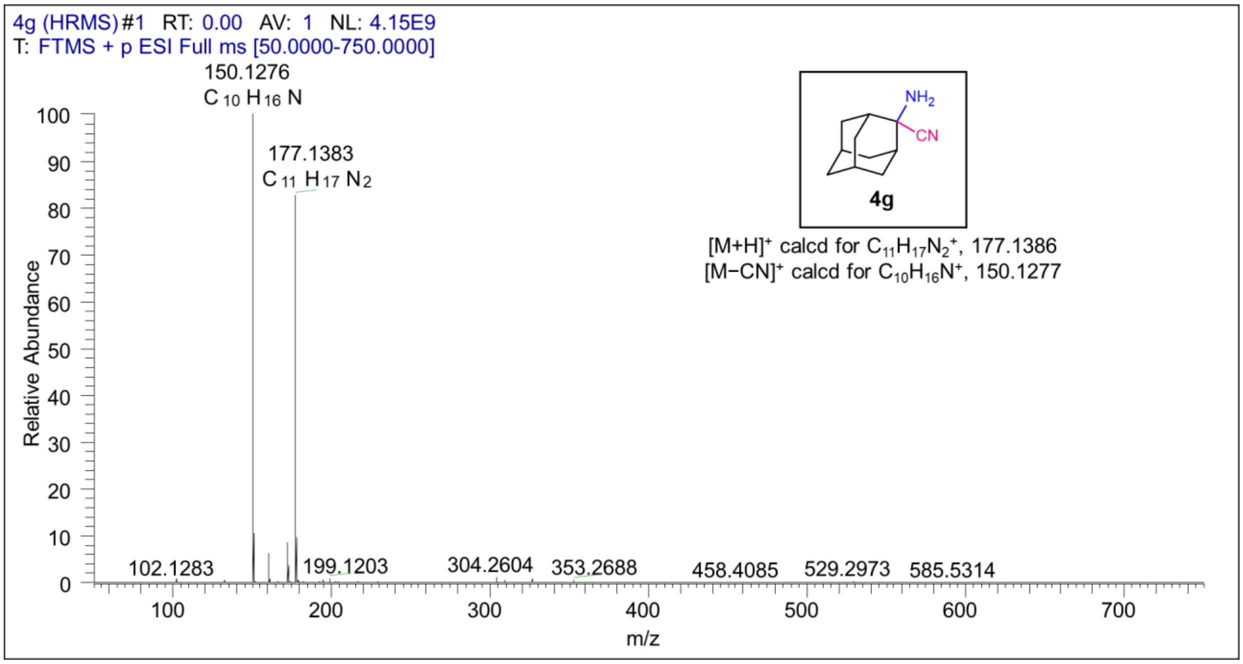

^{1}H NMR (400 MHz, $CDCl_3$) and ^{13}C NMR (100 MHz, $CDCl_3$) spectra for **4h**

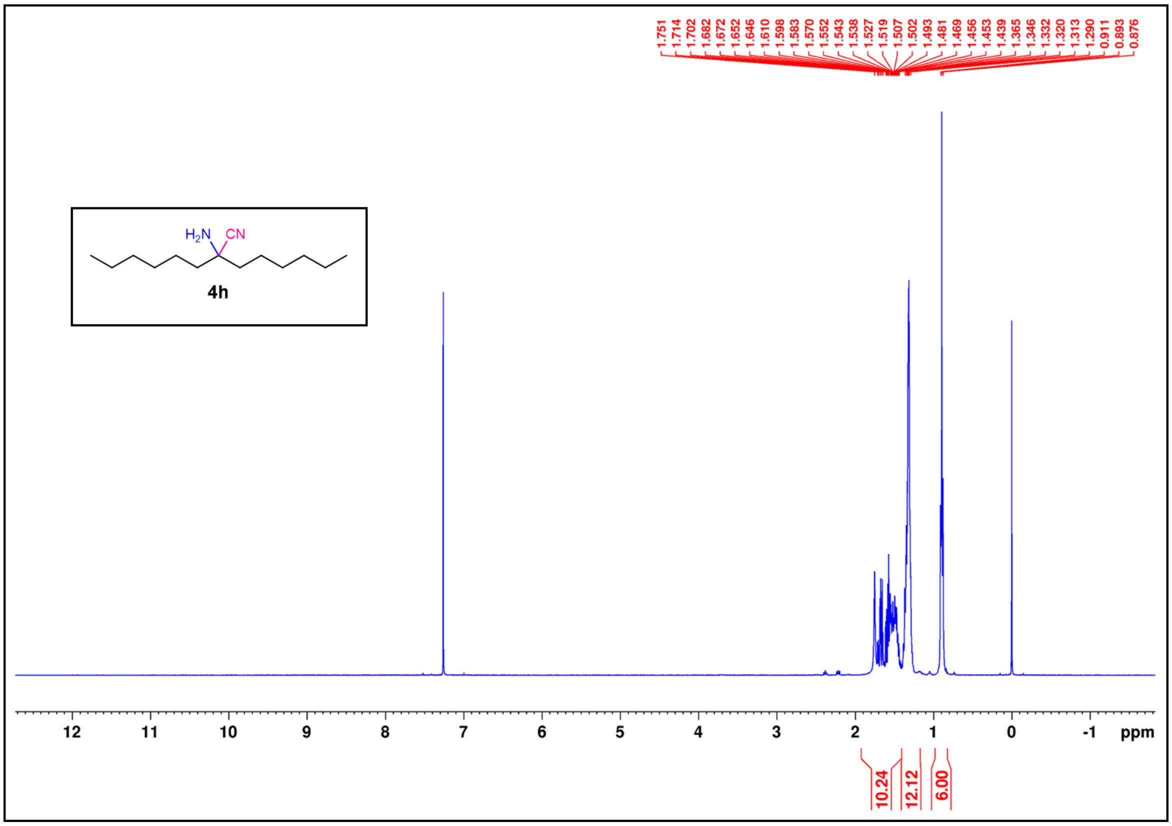

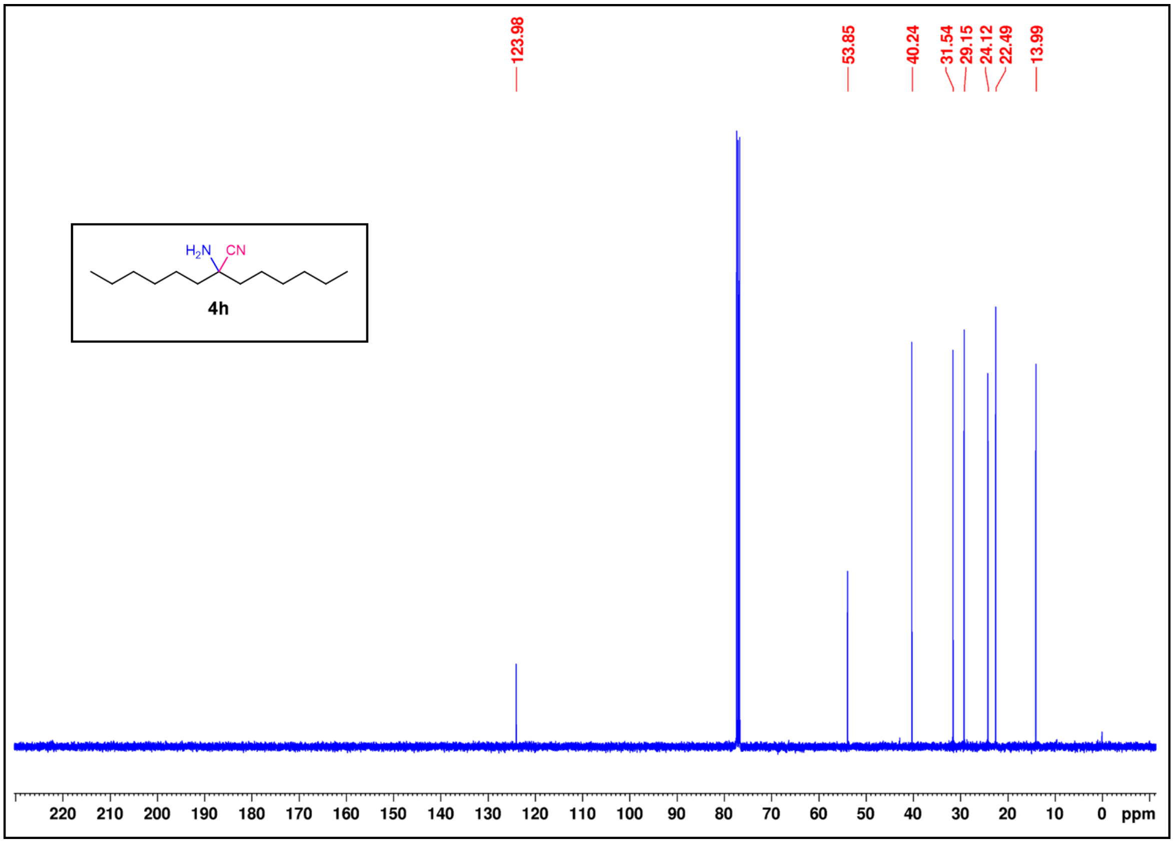

FT-IR (ATR, neat) and HRMS (ESI-positive) spectra for **4h**

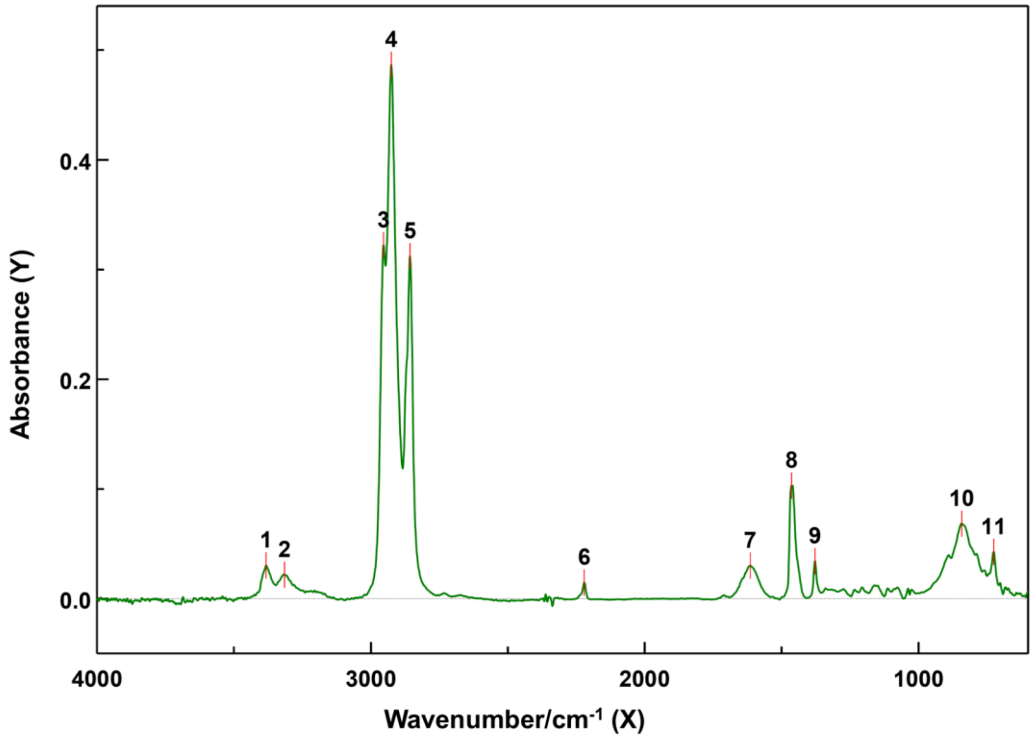

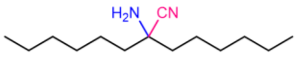

4h

No.	X	Y	No.	X	Y	No.	X	Y
1	3382.5	0.03	5	2857	0.312	9	1378	0.034
2	3315	0.022	6	2220.6	0.015	10	842.7	0.068
3	2953.4	0.322	7	1614.1	0.03	11	726.1	0.043
4	2925.5	0.486	8	1463.7	0.103			

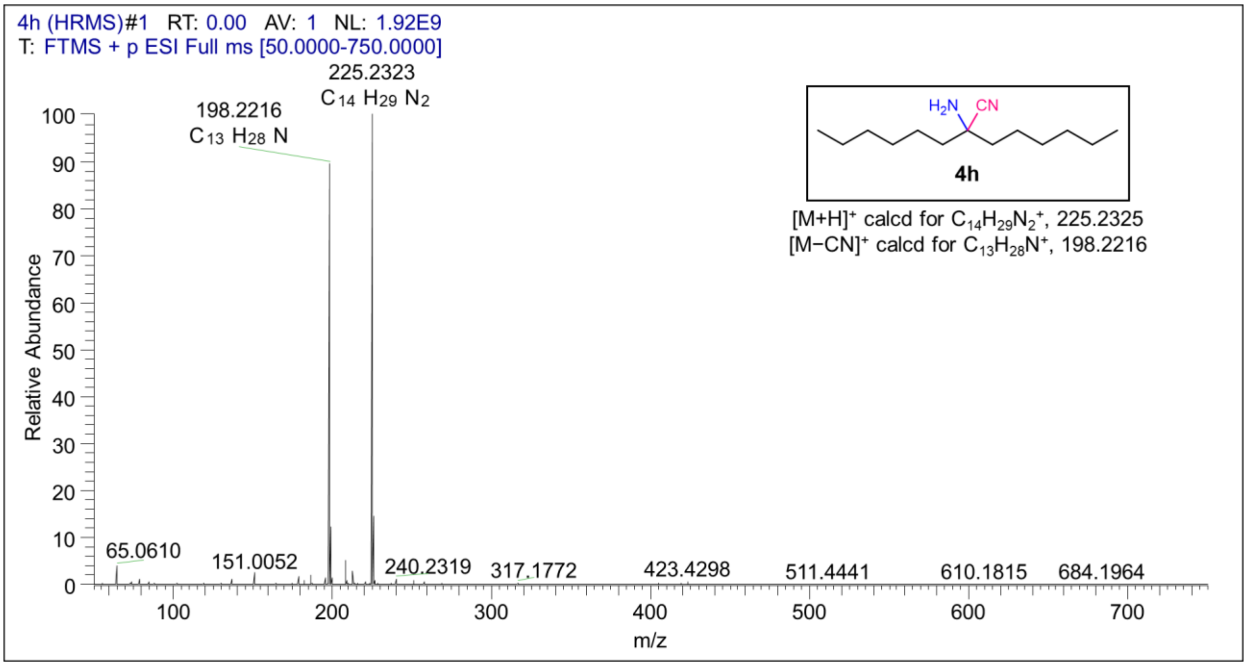

^{1}H NMR (400 MHz, CDCl$_3$) and ^{13}C NMR (100 MHz, CDCl$_3$) spectra for **4i**

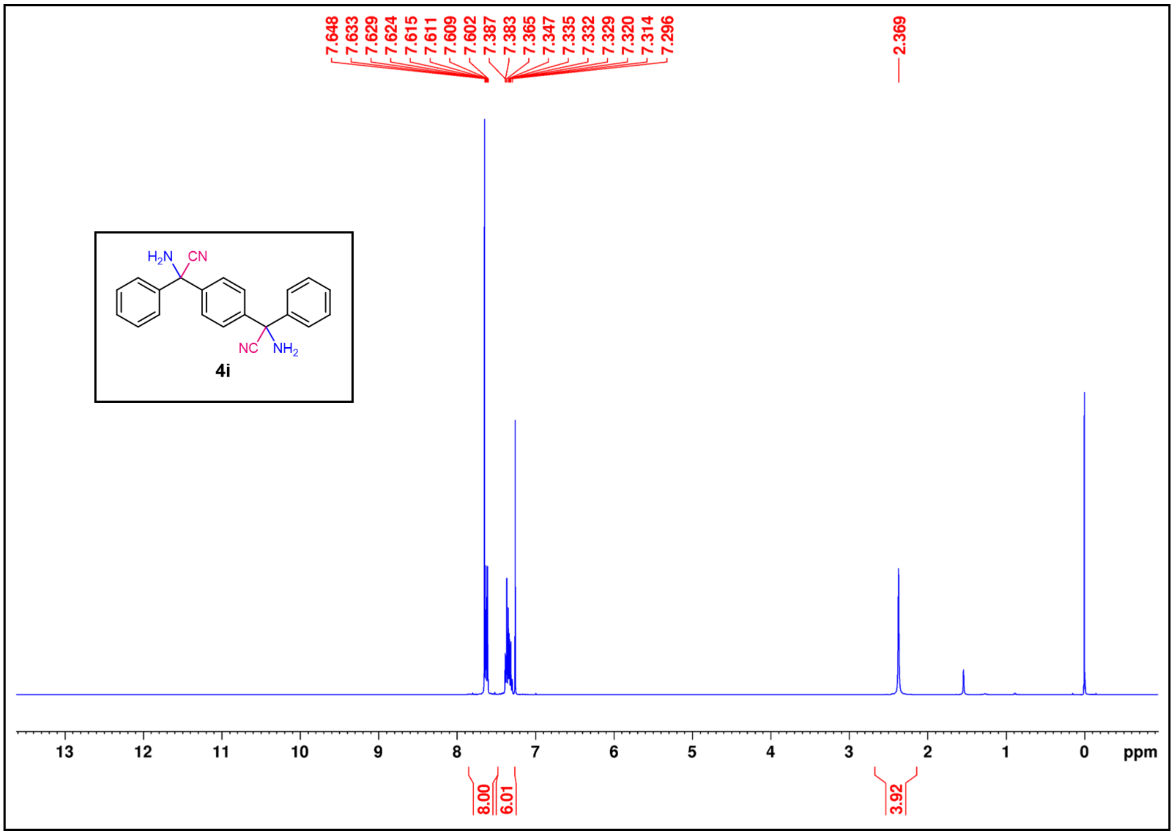

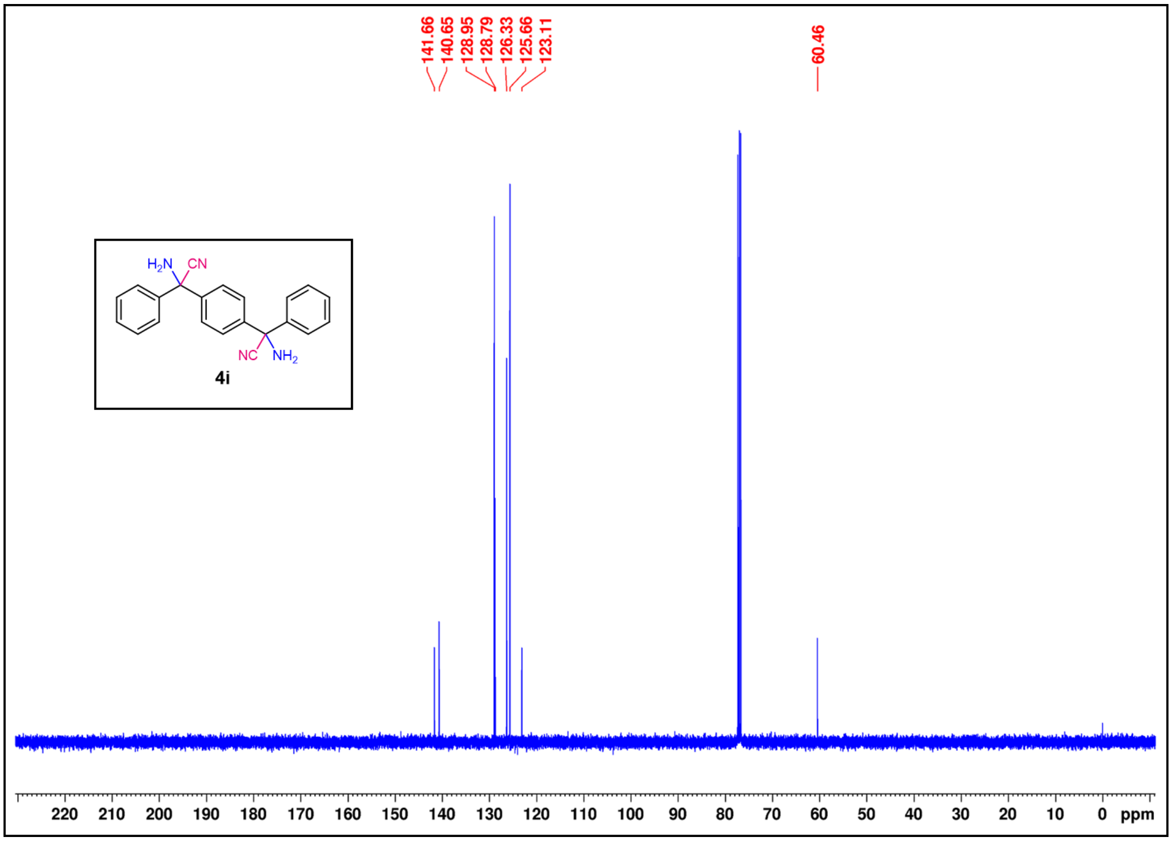

FT-IR (ATR, neat) and HRMS (ESI-positive) spectra for **4i**

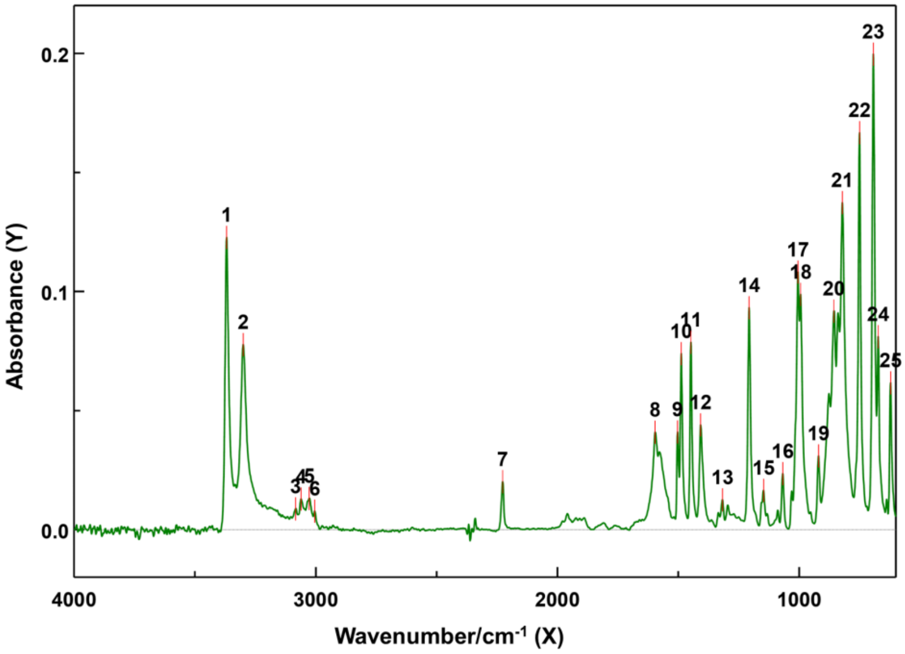

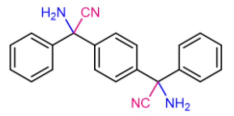

4i

No.	X	Y	No.	X	Y	No.	X	Y
1	3369	0.123	10	1487.8	0.074	19	919.9	0.031
2	3300.6	0.078	11	1448.3	0.079	20	856.2	0.092
3	3083.6	0.009	12	1407.8	0.044	21	821.5	0.137
4	3060.5	0.013	13	1317.1	0.012	22	750.2	0.167
5	3026.7	0.013	14	1207.2	0.093	23	693.3	0.2
6	3004.6	0.008	15	1147.4	0.016	24	673	0.081
7	2226.4	0.02	16	1068.4	0.023	25	621.9	0.062
8	1595.8	0.041	17	1004.7	0.108			
9	1503.2	0.041	18	994.1	0.099			

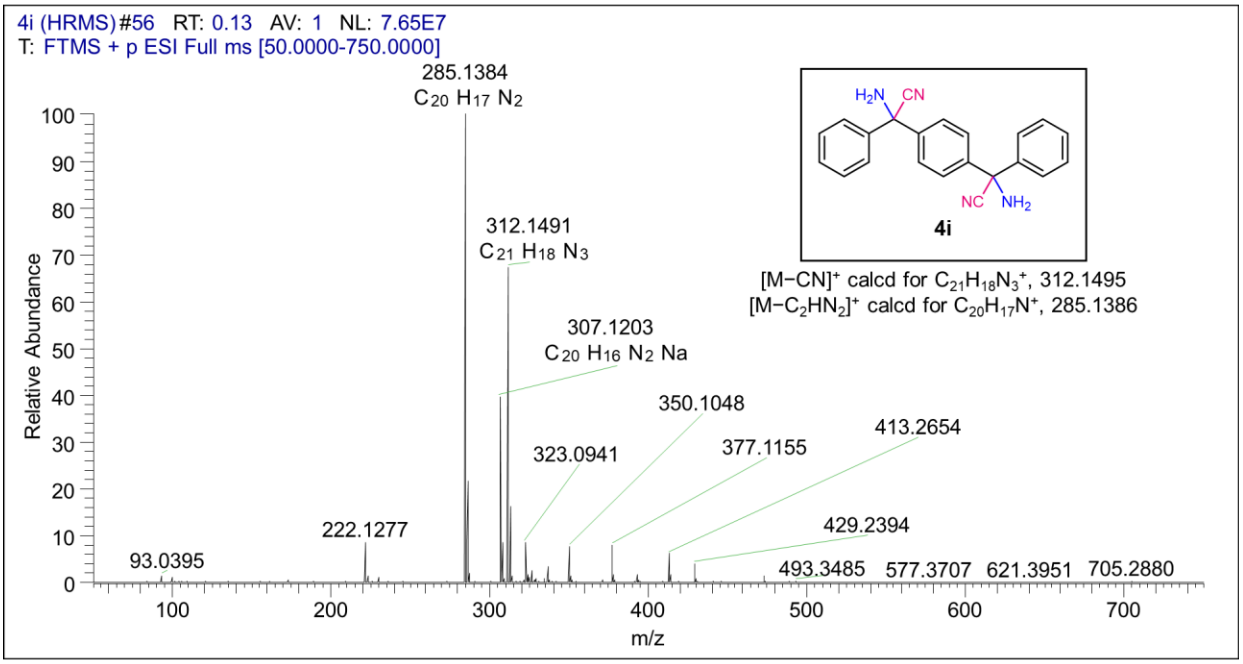

^{1}H NMR (400 MHz, $CDCl_3$) and ^{13}C NMR (100 MHz, $CDCl_3$) spectra for **4j**

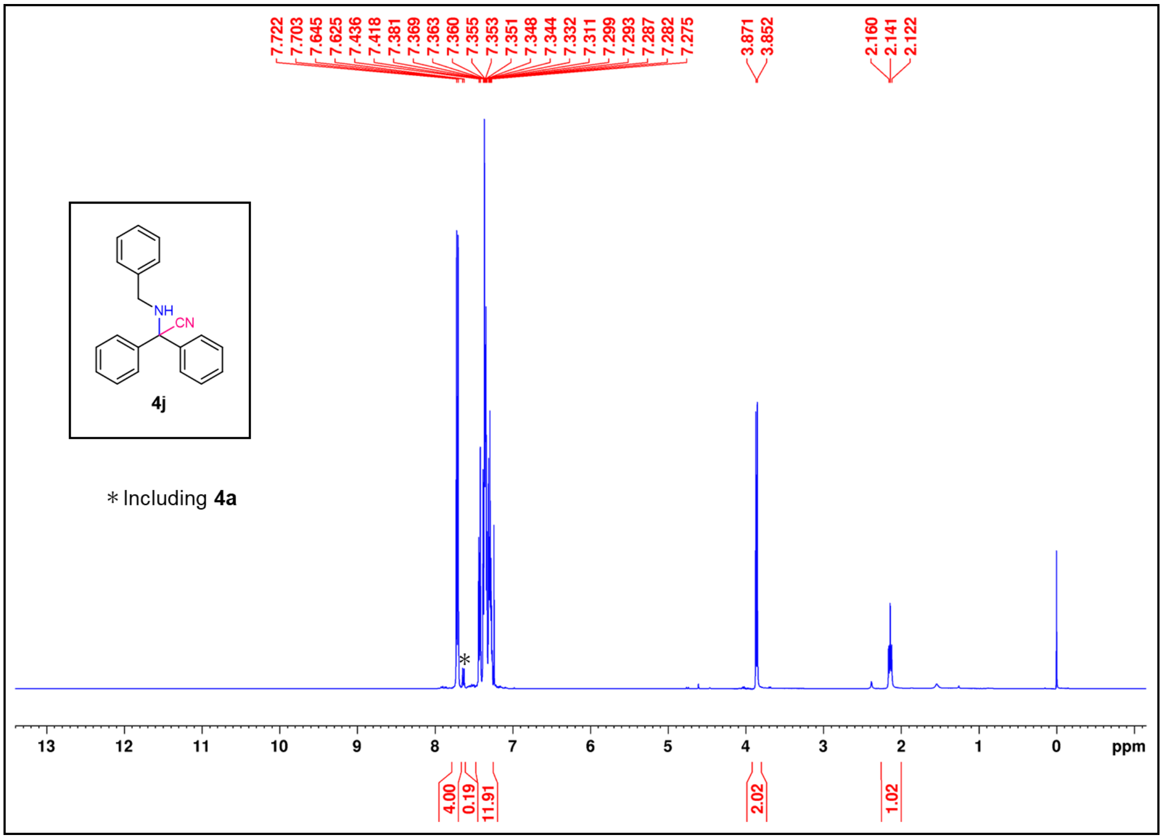

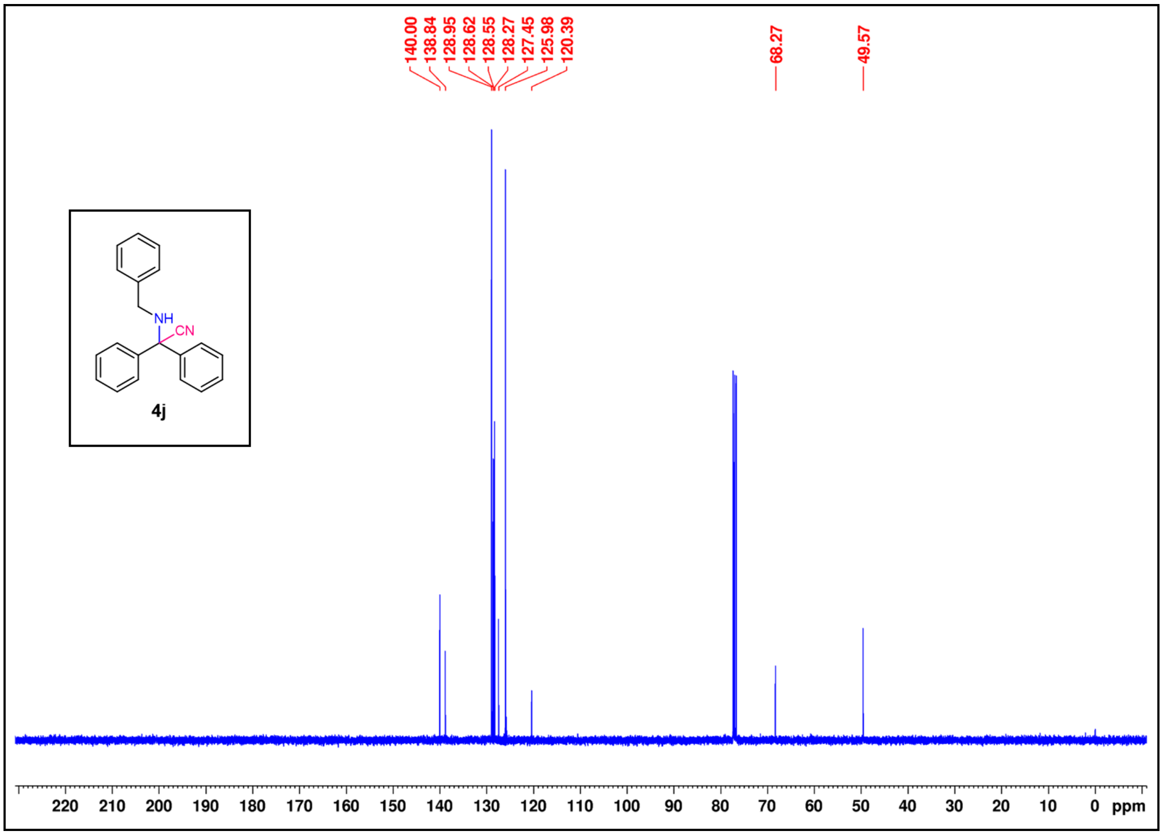

FT-IR (ATR, neat) and HRMS (ESI-positive) spectra for **4j**

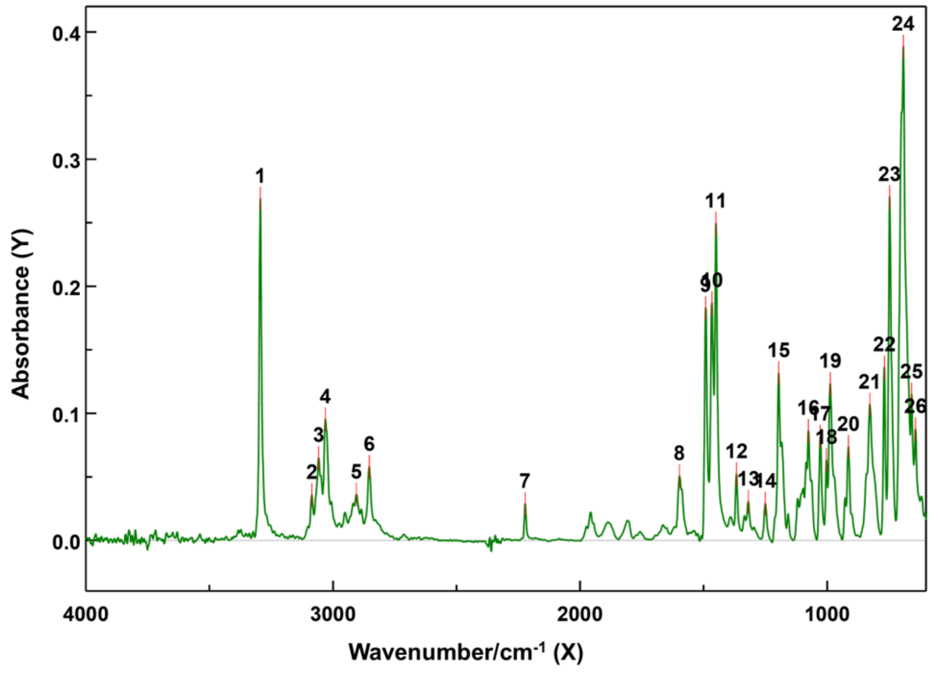

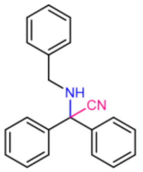

4j

No.	X	Y	No.	X	Y	No.	X	Y
1	3293.8	0.269	10	1466.6	0.186	19	987.4	0.123
2	3085.5	0.036	11	1450.2	0.249	20	914.1	0.073
3	3057.6	0.065	12	1367.3	0.052	21	827.3	0.107
4	3030.6	0.095	13	1319.1	0.03	22	768.5	0.136
5	2905.2	0.036	14	1250.6	0.029	23	747.3	0.27
6	2853.2	0.058	15	1196.6	0.131	24	691.4	0.388
7	2222.6	0.029	16	1076.1	0.086	25	658.6	0.115
8	1597.7	0.05	17	1027.9	0.082	26	642.2	0.087
9	1491.7	0.183	18	1002.8	0.063			

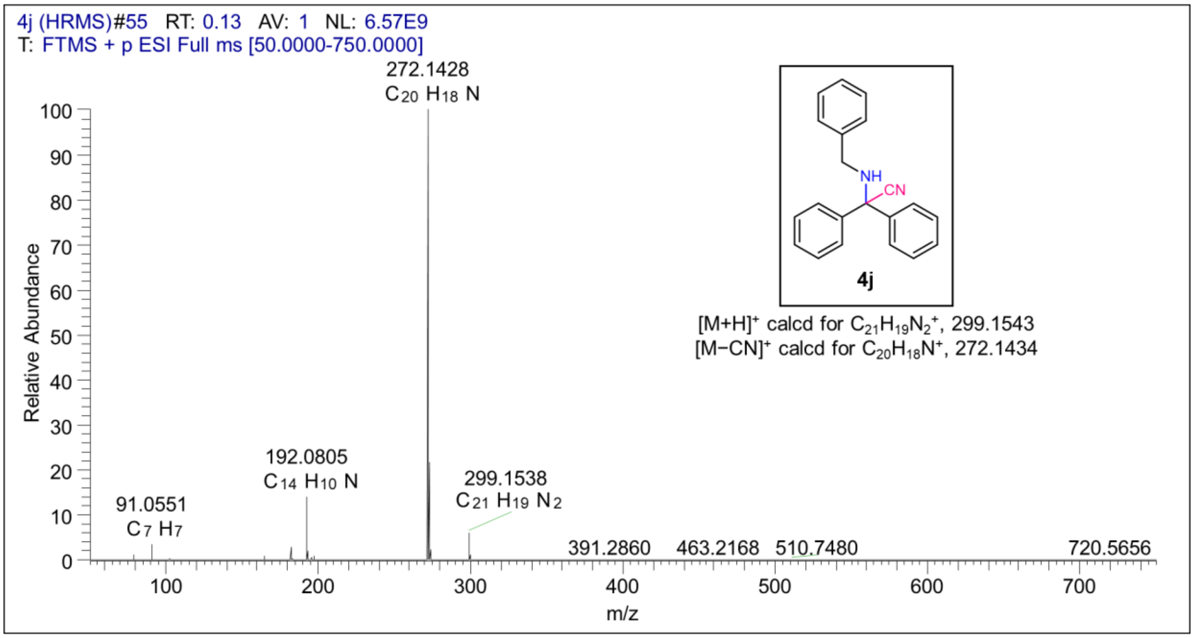

^{1}H NMR (400 MHz, $CDCl_3$) and ^{13}C NMR (100 MHz, $CDCl_3$) spectra for **4k**

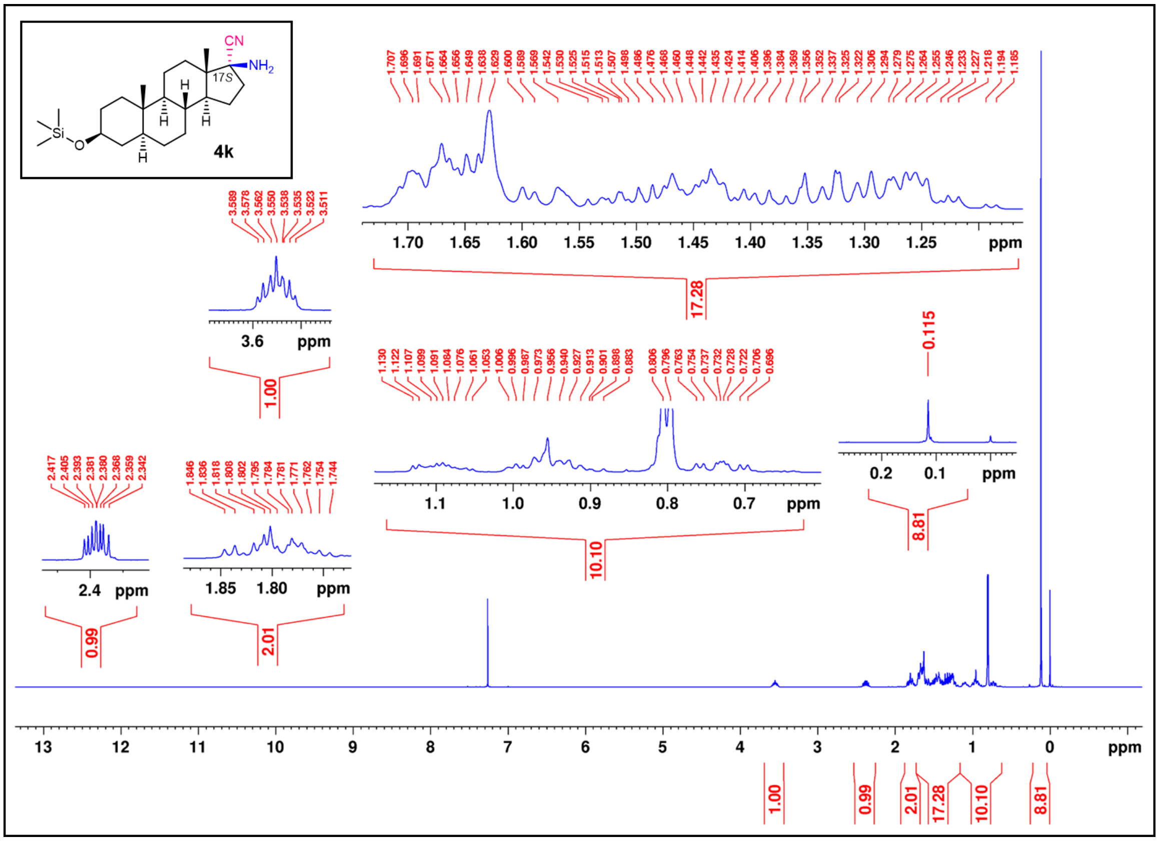

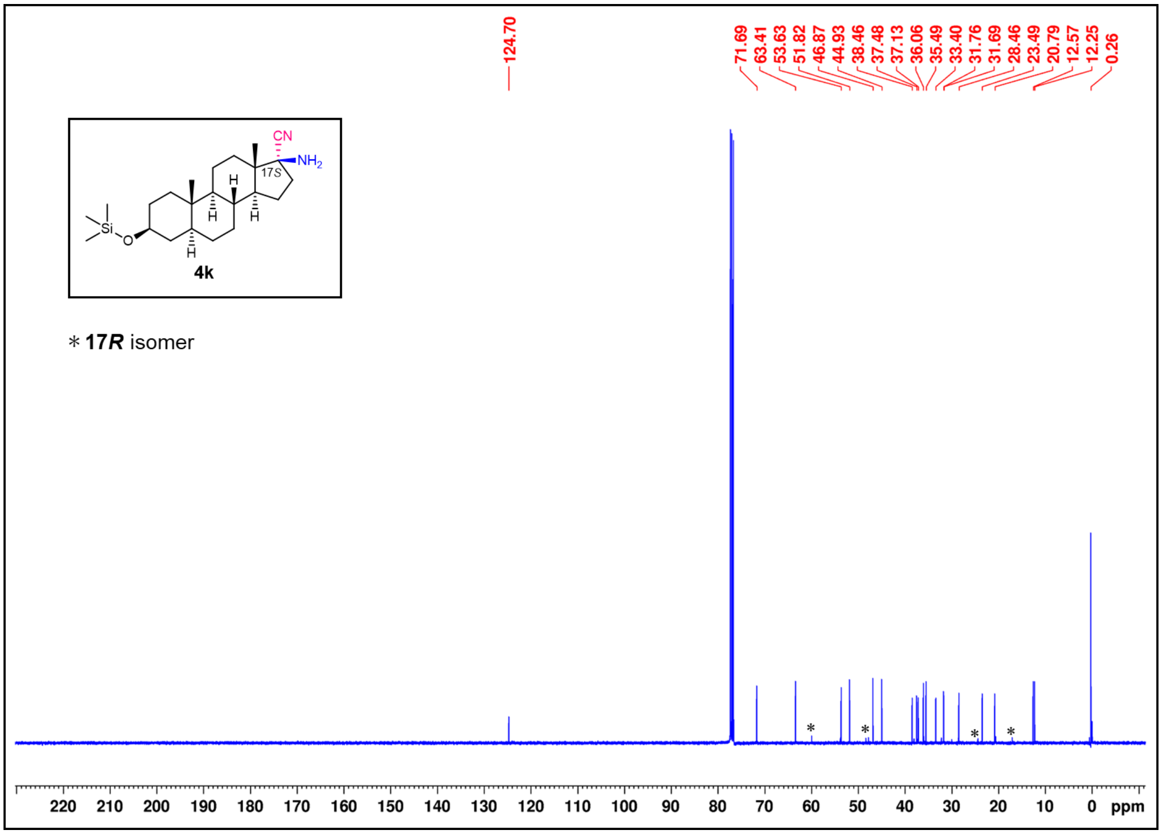

FT-IR (ATR, neat) and HRMS (ESI-positive) spectra for **4k**

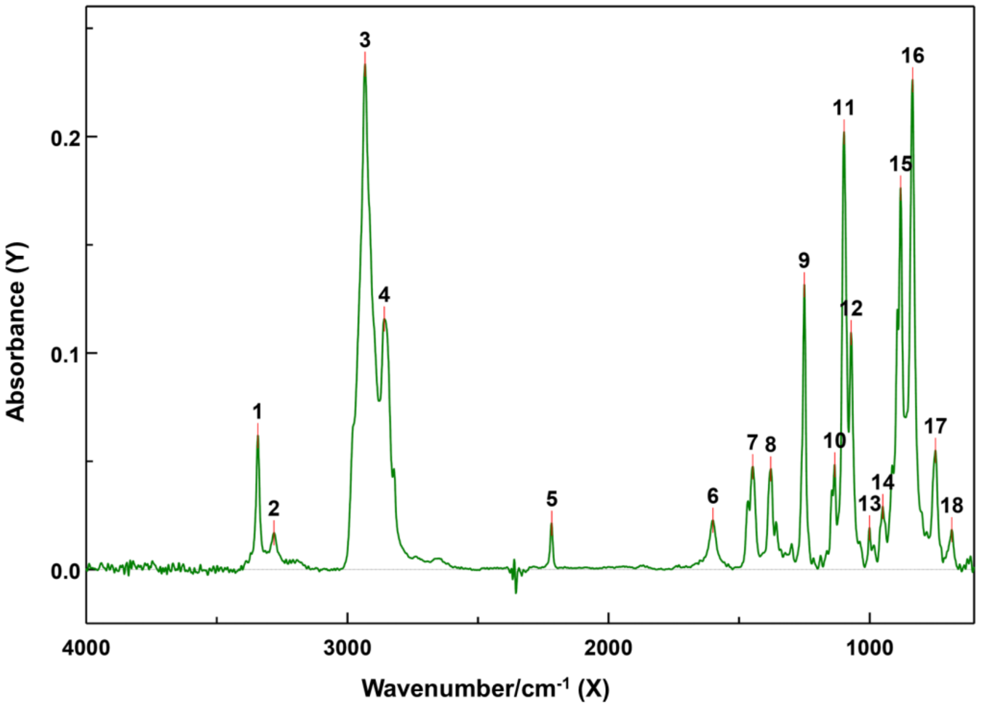

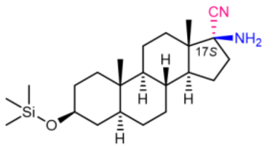

4k

No.	X	Y	No.	X	Y	No.	X	Y
1	3343	0.062	7	1448.3	0.047	13	1001	0.019
2	3281.3	0.017	8	1378.9	0.046	14	949.8	0.029
3	2932.2	0.233	9	1250.6	0.131	15	881.3	0.176
4	2859	0.116	10	1133.9	0.048	16	836	0.226
5	2217.7	0.021	11	1098.3	0.202	17	748.2	0.055
6	1600.6	0.023	12	1071.3	0.109	18	685.6	0.018

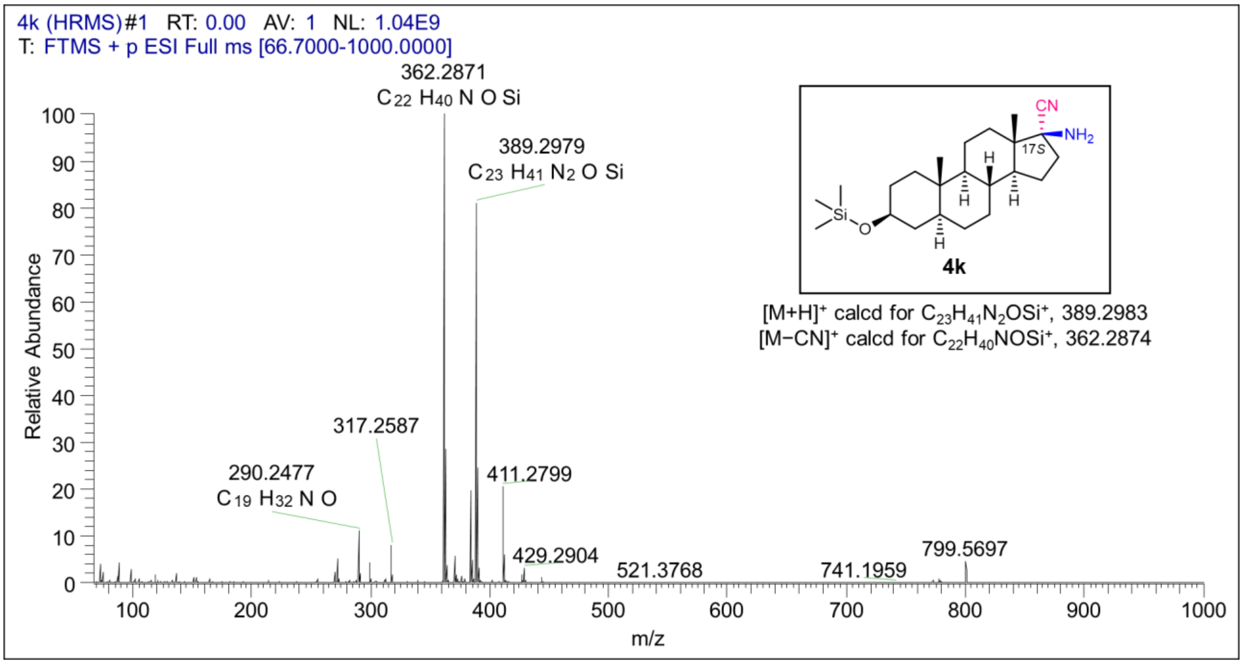

10.4 Hydantoin compound

^{1}H NMR (400 MHz, DMSO-d_6) and ^{13}C NMR (100 MHz, DMSO-d_6) spectra for **5a**

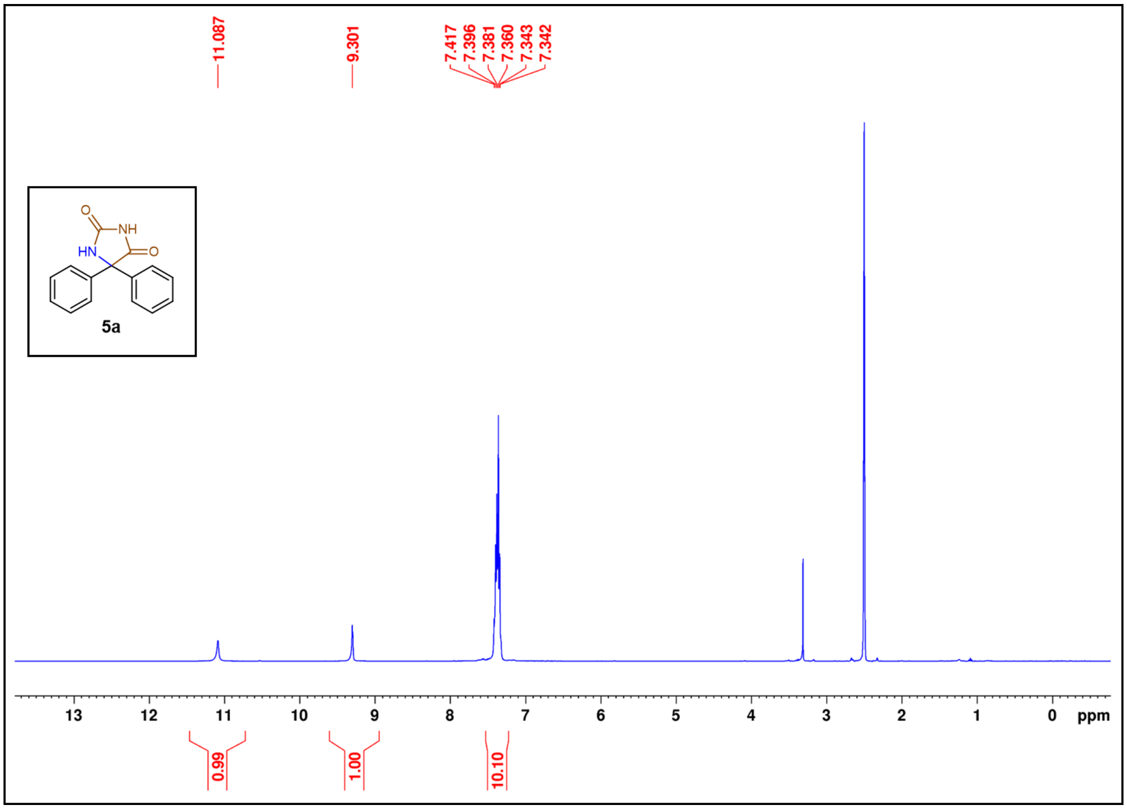

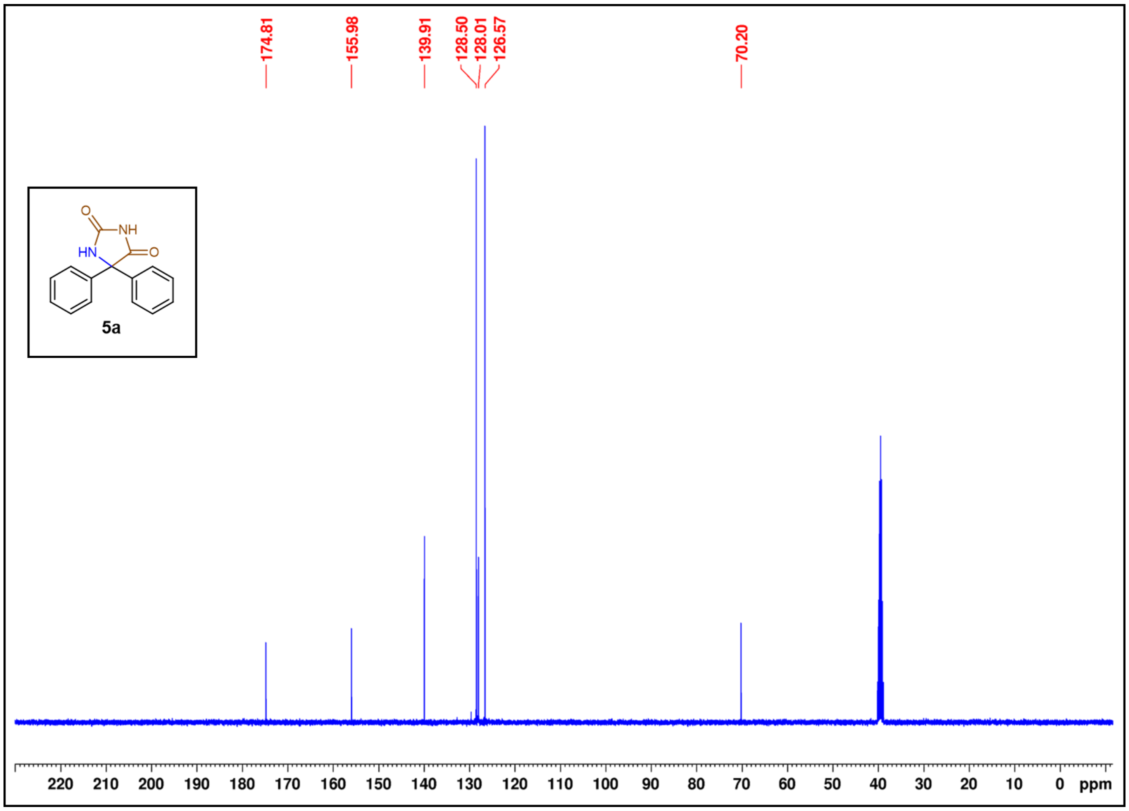

FT-IR (ATR, neat) and HRMS (ESI-positive) spectra for **5a**

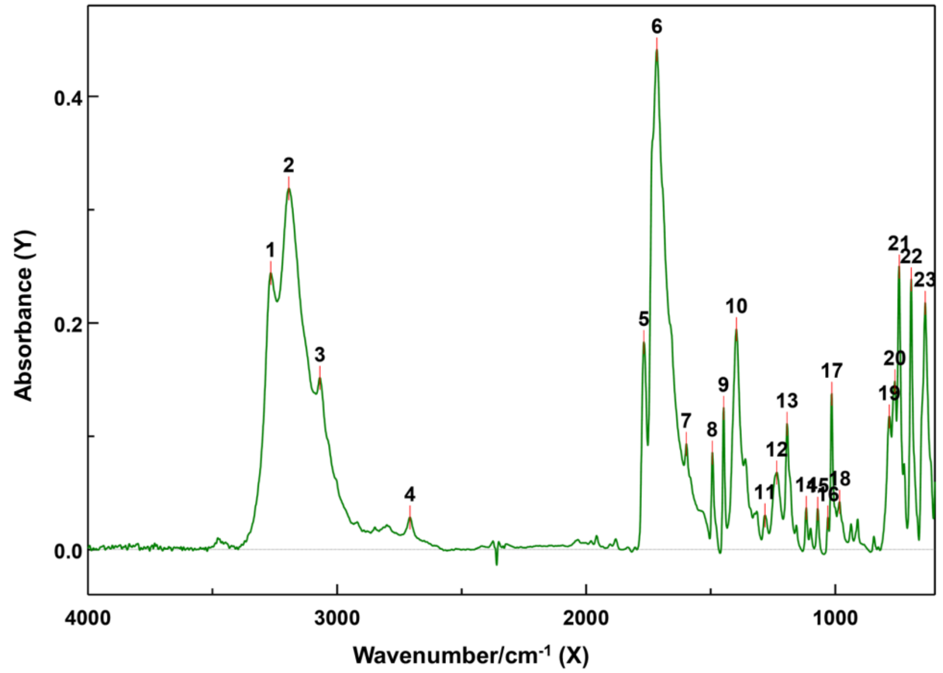

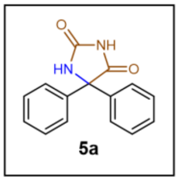

No.	X	Y	No.	X	Y	No.	X	Y
1	3265.9	0.244	9	1448.3	0.125	17	1014	0.137
2	3193.5	0.319	10	1397.2	0.194	18	982.6	0.042
3	3069.2	0.151	11	1282.4	0.03	19	783	0.117
4	2707.6	0.028	12	1235.2	0.068	20	760.8	0.148
5	1768.4	0.183	13	1193.7	0.111	21	743.4	0.25
6	1716.3	0.441	14	1116.6	0.037	22	695.2	0.238
7	1597.7	0.093	15	1070.3	0.036	23	639.3	0.217
8	1493.6	0.085	16	1029.8	0.028			

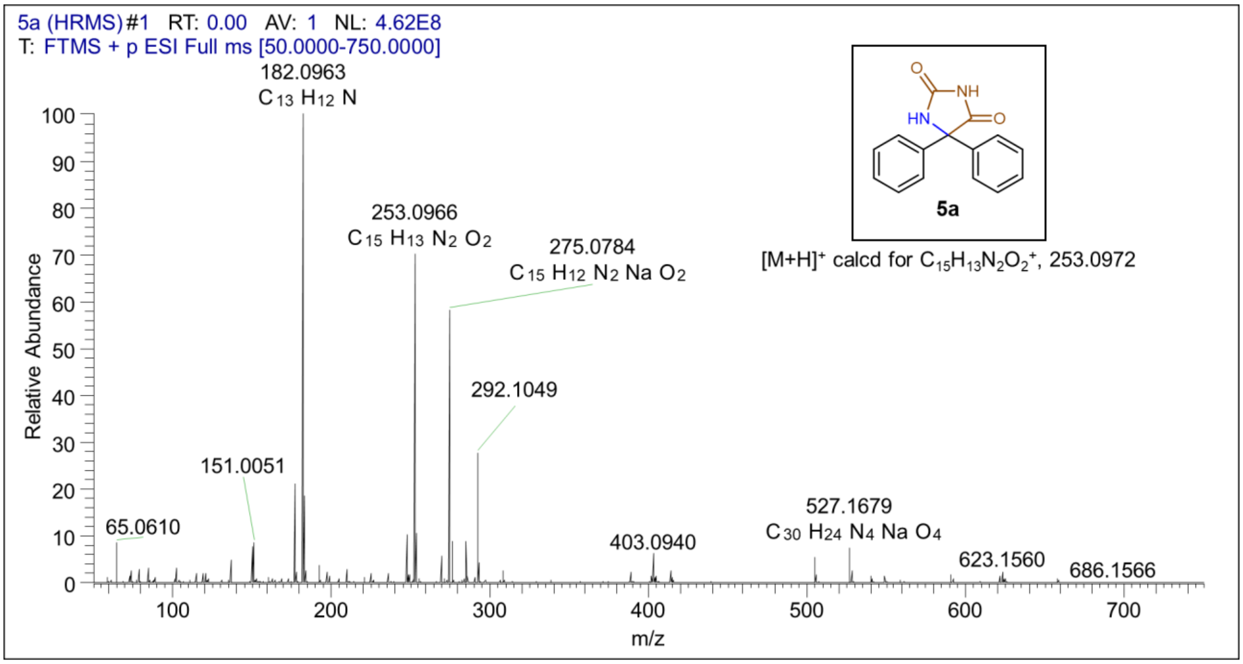

^{1}H NMR (400 MHz, DMSO-d_6) and ^{13}C NMR (100 MHz, DMSO-d_6) spectra for **5b**

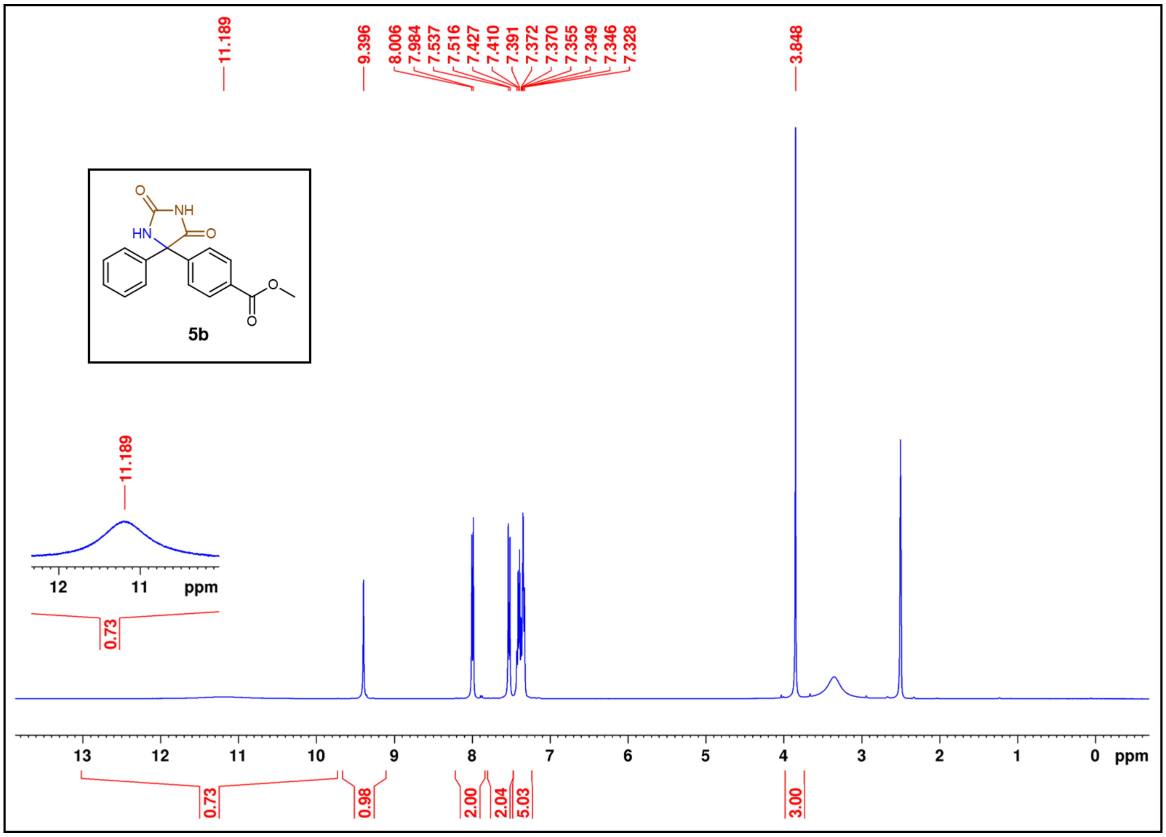

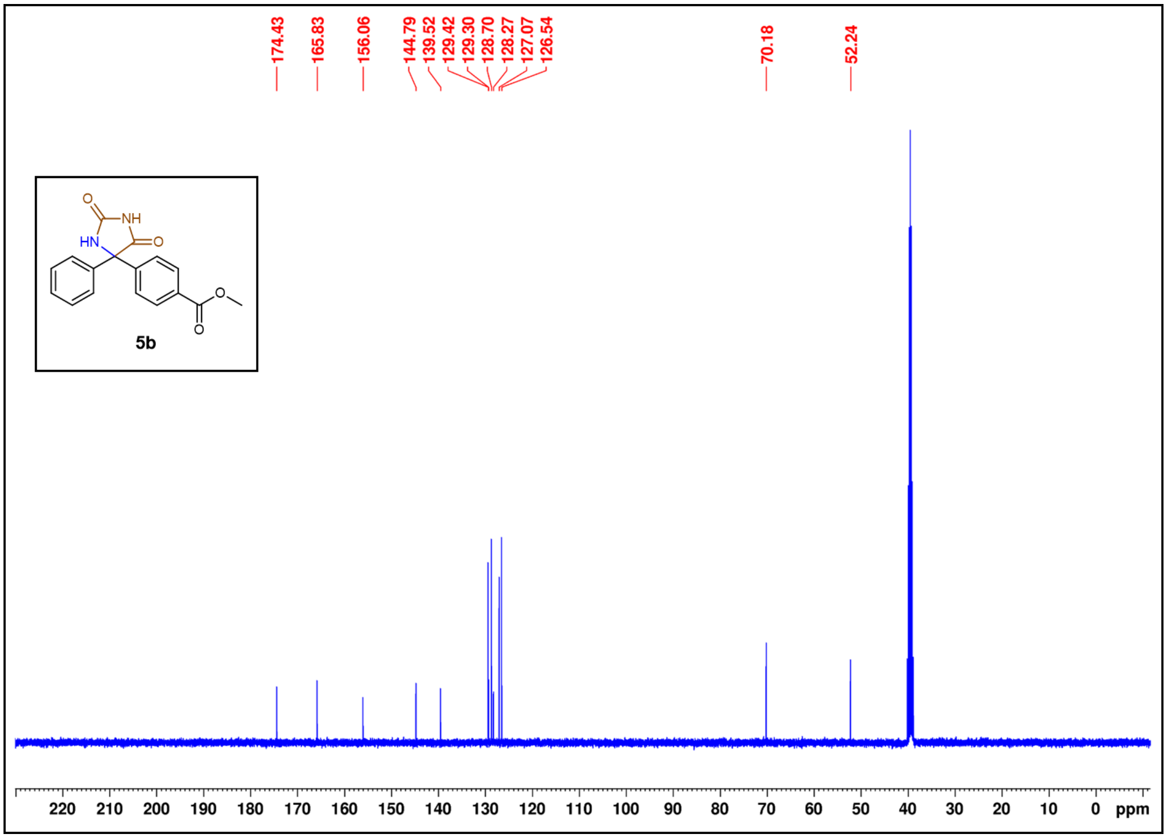

FT-IR (ATR, neat) and HRMS (ESI-positive) spectra for **5b**

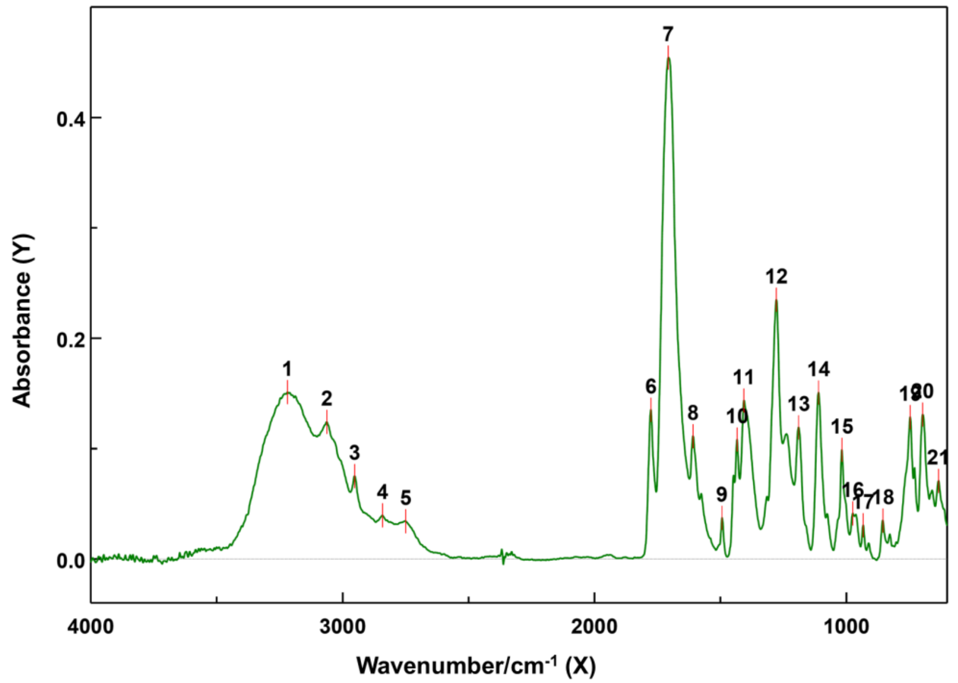

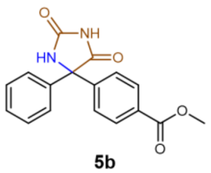

5b

No.	X	Y	No.	X	Y	No.	X	Y
1	3218.6	0.151	8	1608.3	0.111	15	1018	0.099
2	3063.4	0.124	9	1493.6	0.037	16	974.8	0.041
3	2952.5	0.075	10	1433.8	0.108	17	933.4	0.03
4	2841.6	0.039	11	1406.8	0.143	18	855.3	0.034
5	2750	0.034	12	1277.6	0.235	19	746.3	0.128
6	1776.1	0.135	13	1189.9	0.119	20	697.1	0.131
7	1706.7	0.454	14	1110.8	0.151	21	634.5	0.071

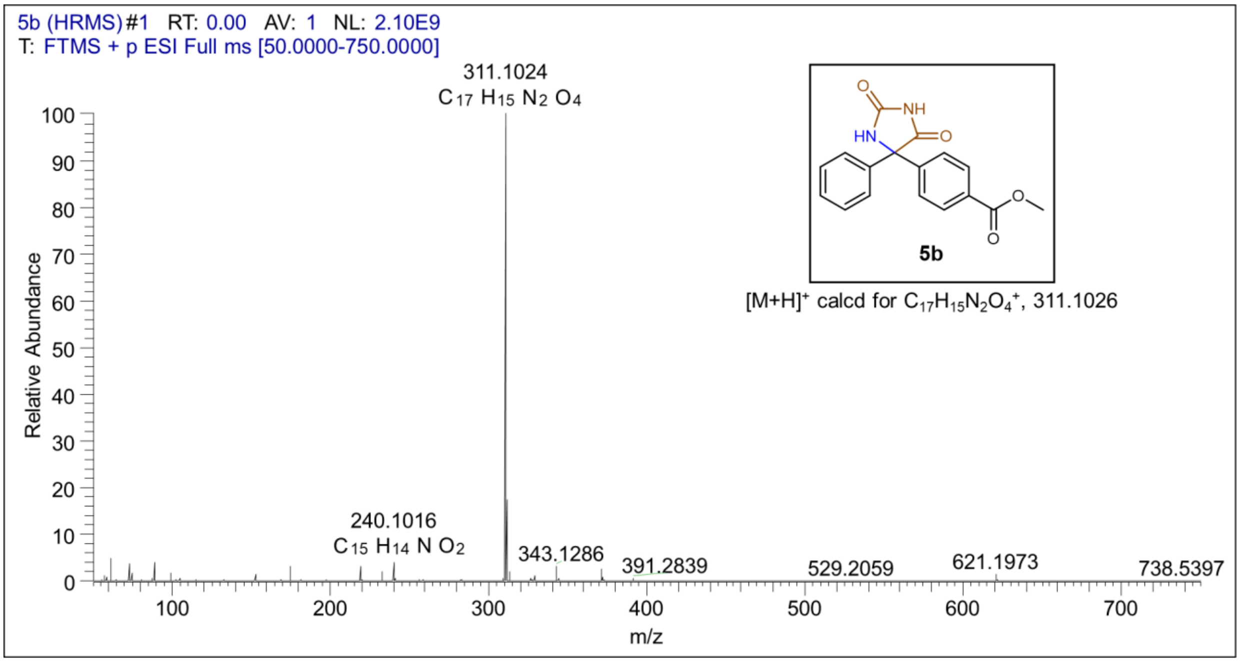

^1H NMR (400 MHz, DMSO-d_6) and ^{13}C NMR (100 MHz, DMSO-d_6) spectra for **5c**

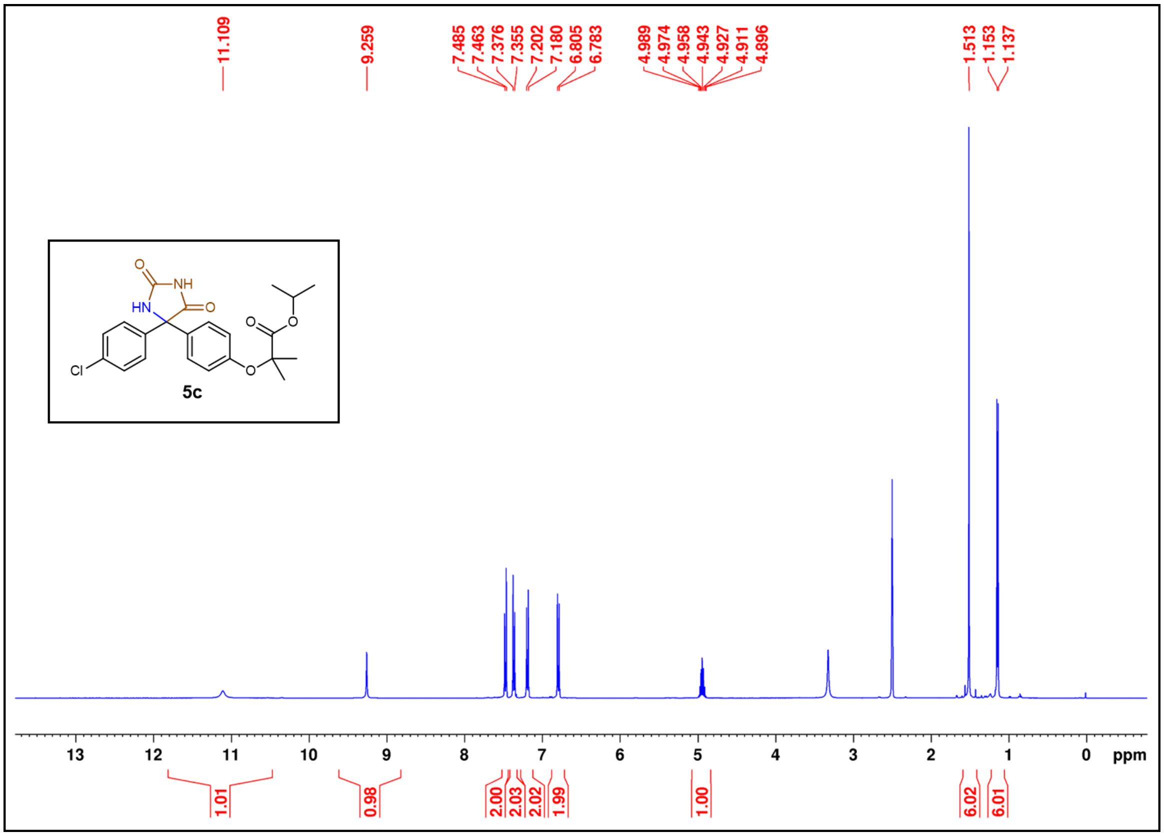

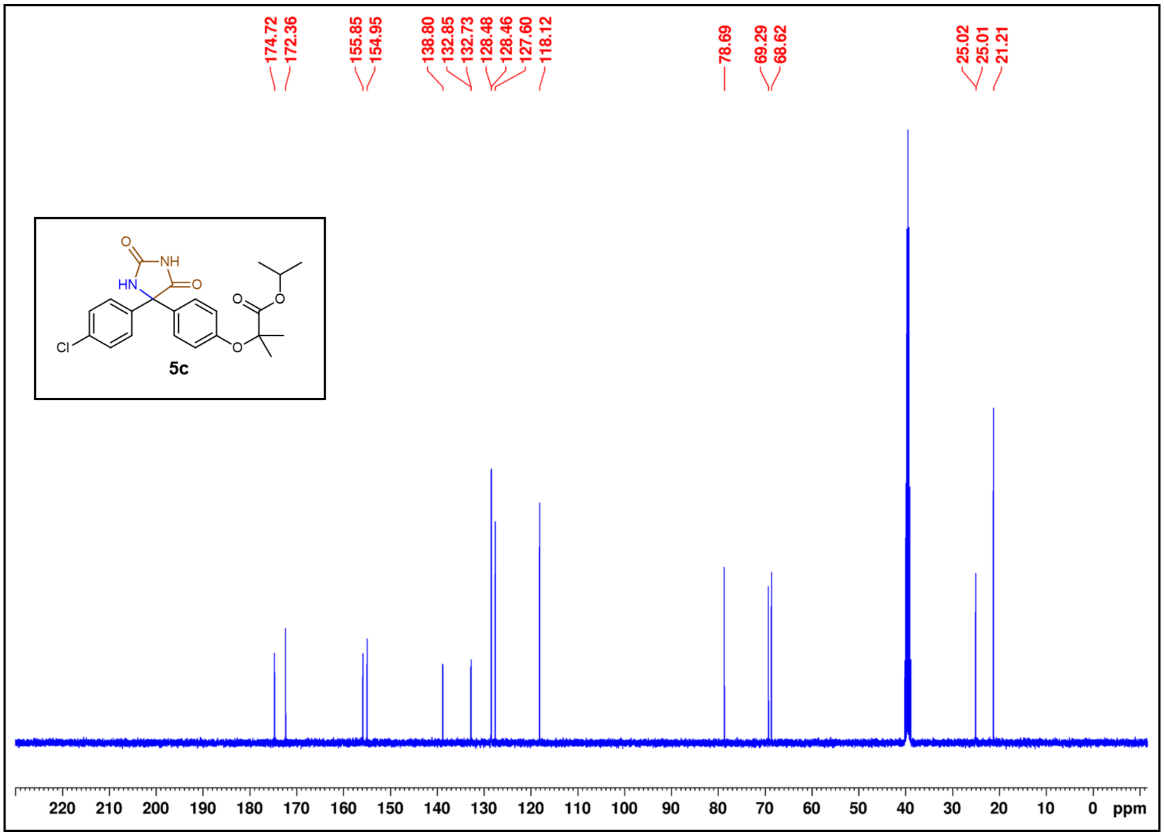

FT-IR (ATR, neat) and HRMS (ESI-positive) spectra for **5c**

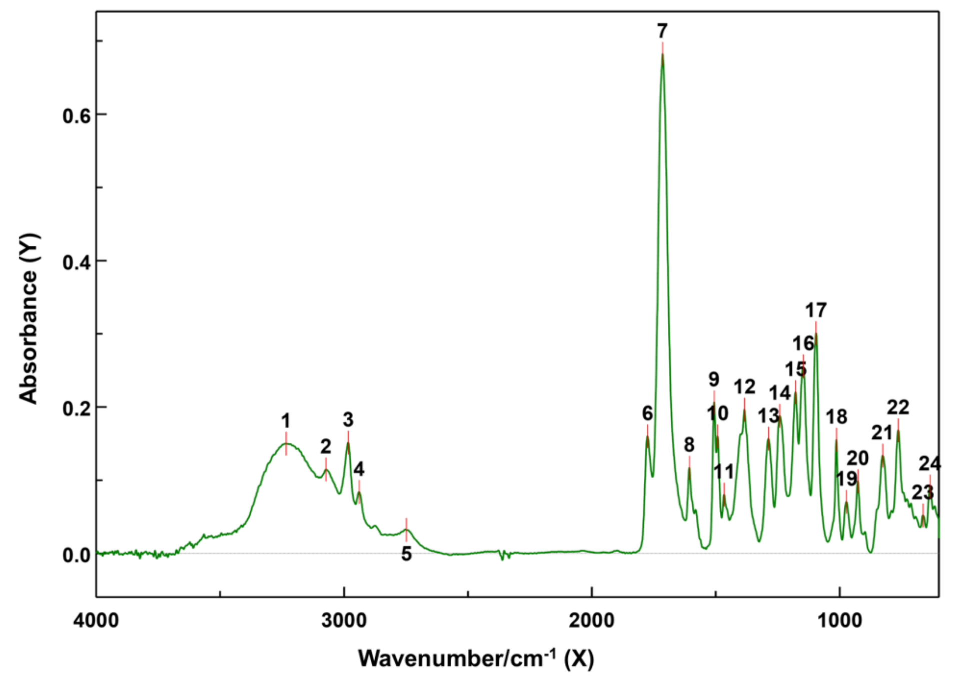

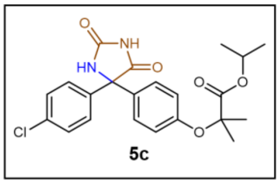

No.	X	Y	No.	X	Y	No.	X	Y
1	3234	0.15	9	1506.1	0.206	17	1095	0.301
2	3073	0.114	10	1492.6	0.16	18	1013	0.155
3	2983.3	0.151	11	1466.6	0.08	19	972.9	0.07
4	2939	0.084	12	1384.6	0.196	20	926.6	0.098
5	2748.1	0.032	13	1287.3	0.156	21	826.3	0.133
6	1775.2	0.16	14	1241.9	0.188	22	763.7	0.168
7	1714.4	0.682	15	1178.3	0.22	23	664.4	0.052
8	1606.4	0.117	16	1147.4	0.255	24	635.4	0.091

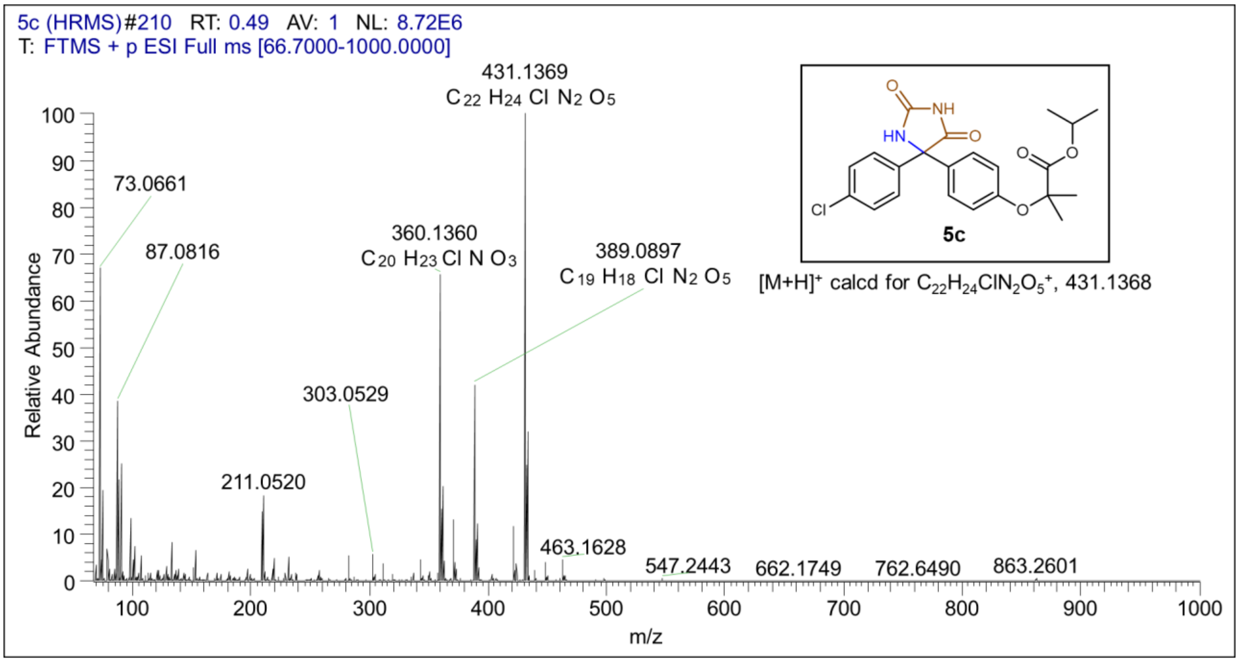

^{1}H NMR (400 MHz, DMSO-d_6) and ^{13}C NMR (100 MHz, DMSO-d_6) spectra for **5d**

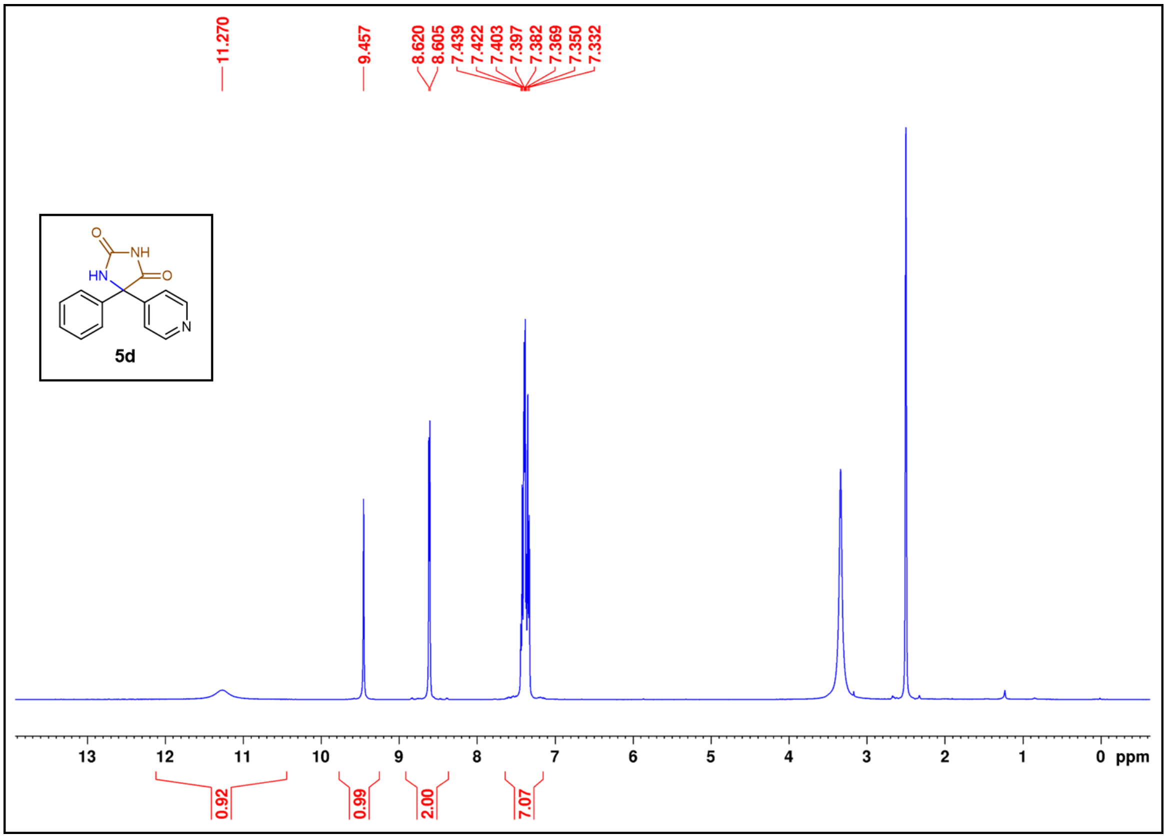

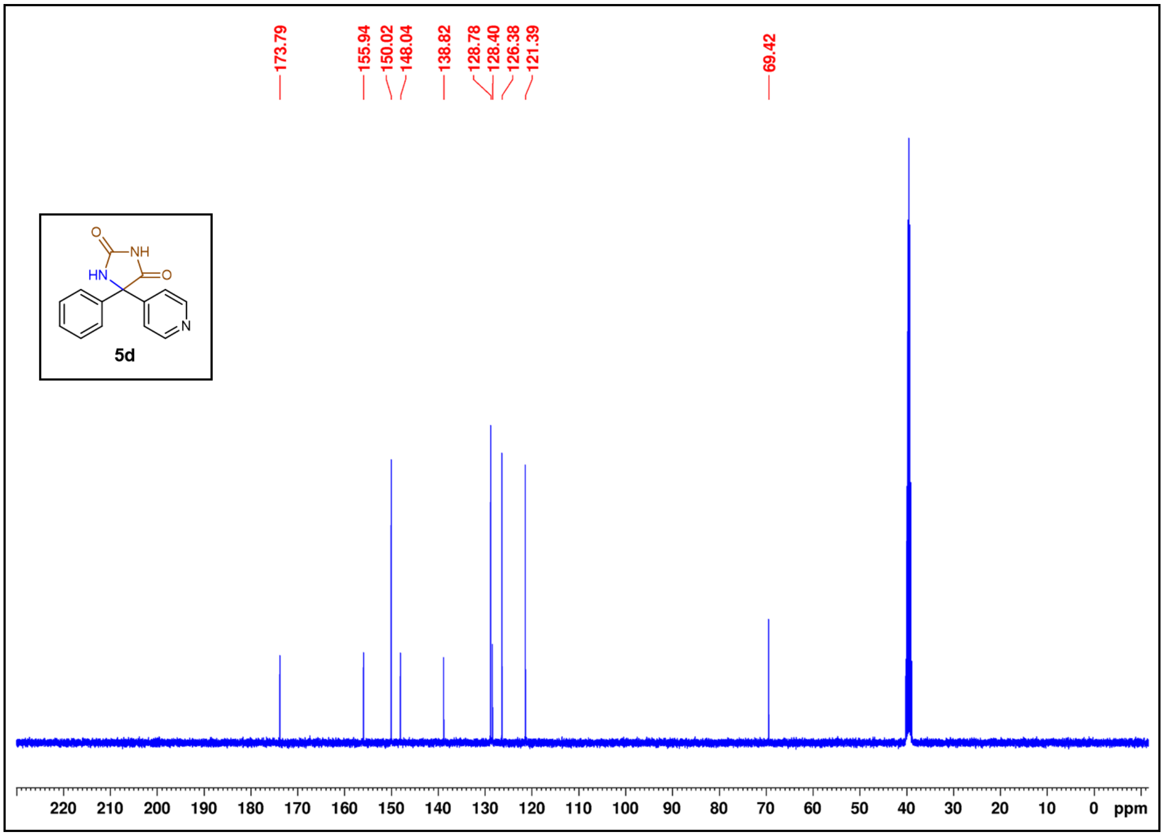

FT-IR (ATR, neat) and HRMS (ESI-positive) spectra for **5d**

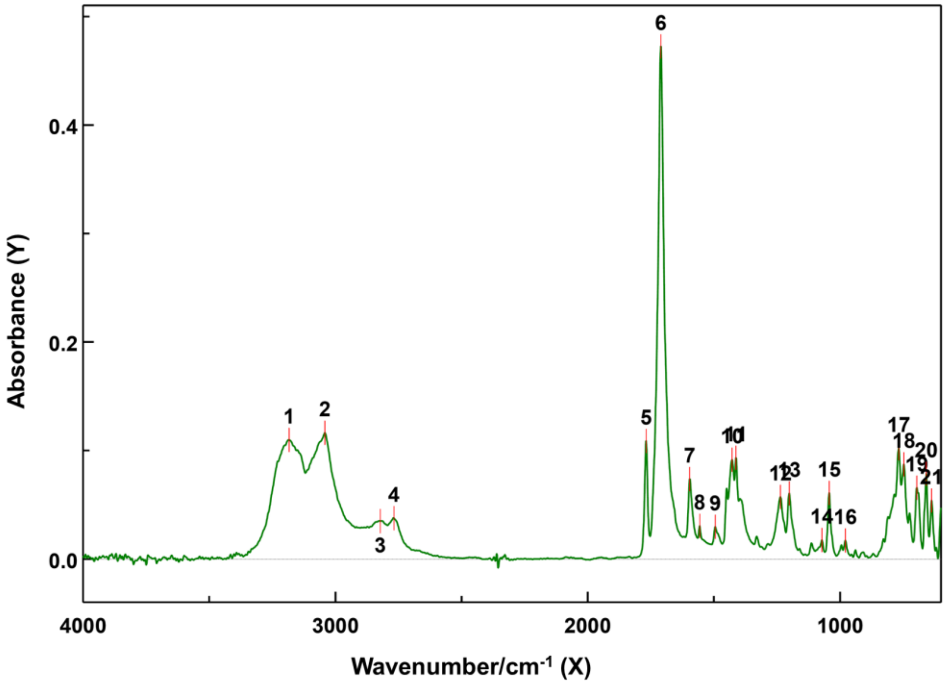

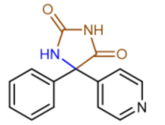

5d

No.	X	Y	No.	X	Y	No.	X	Y
1	3183.9	0.11	8	1556.3	0.03	15	1043	0.061
2	3042.2	0.116	9	1494.6	0.03	16	978.7	0.017
3	2822.3	0.035	10	1428	0.091	17	767.5	0.102
4	2768.3	0.038	11	1412.6	0.093	18	747.3	0.087
5	1768.4	0.109	12	1236.1	0.057	19	696.2	0.066
6	1710.6	0.472	13	1201.4	0.06	20	658.6	0.078
7	1595.8	0.074	14	1072.2	0.017	21	637.4	0.054

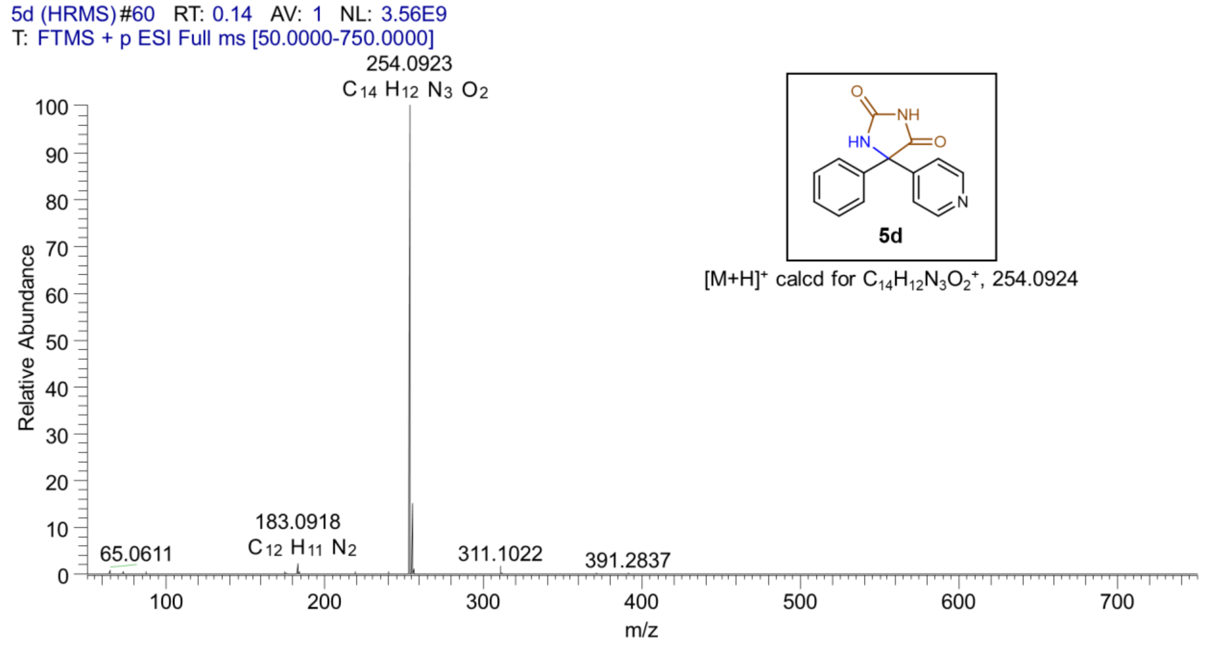

^{1}H NMR (400 MHz, DMSO-d_6) and ^{13}C NMR (100 MHz, DMSO-d_6) spectra for **5e**

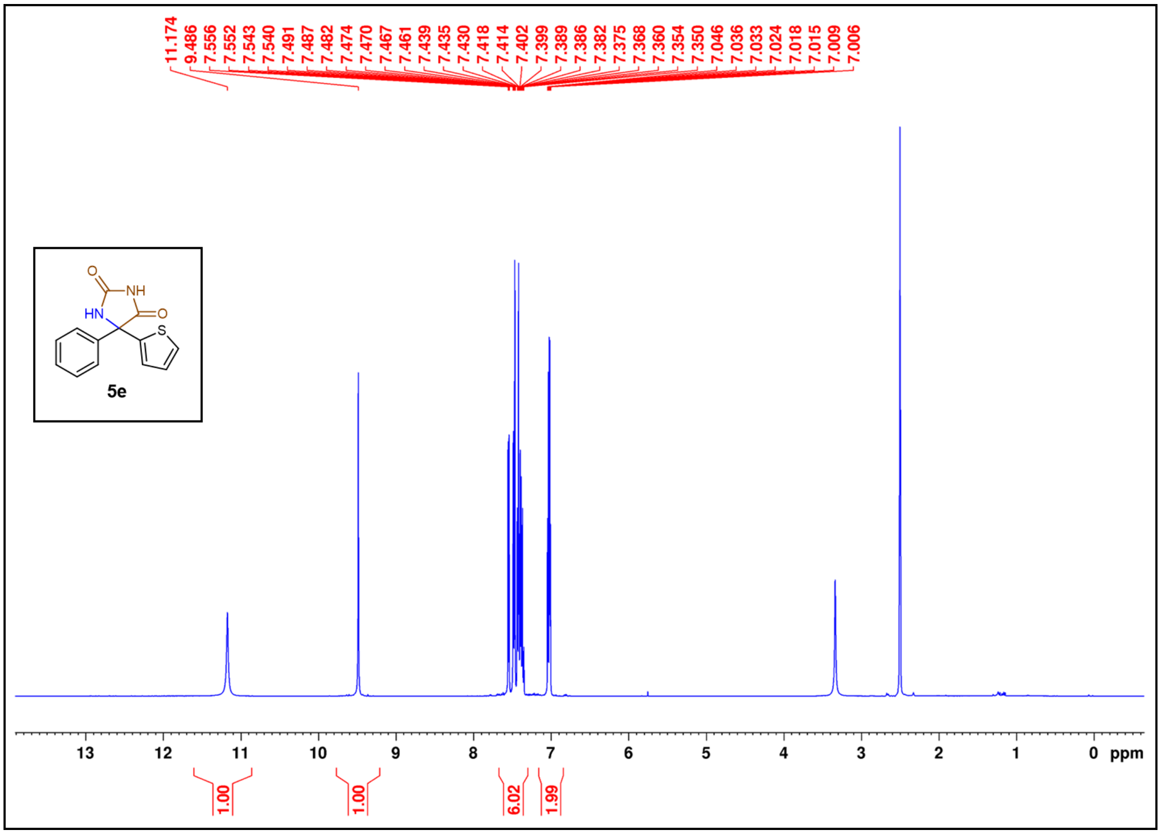

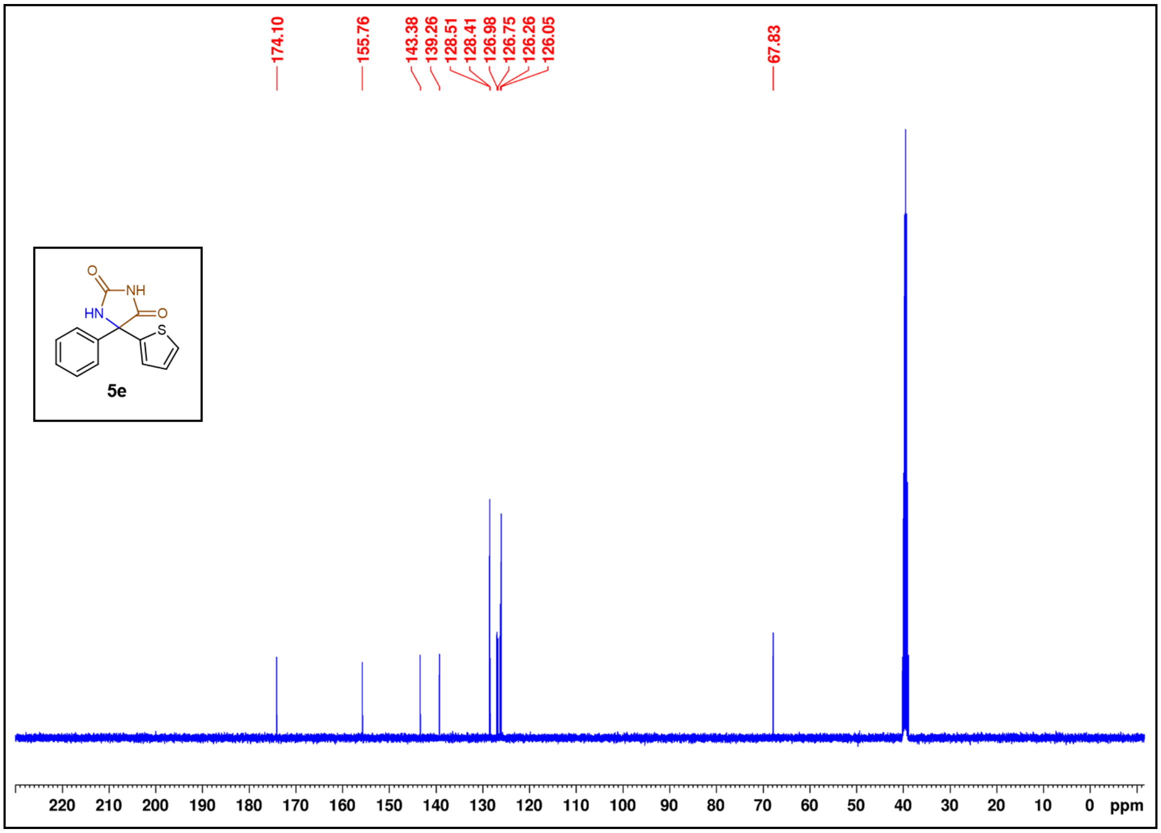

FT-IR (ATR, neat) and HRMS (ESI-positive) spectra for **5e**

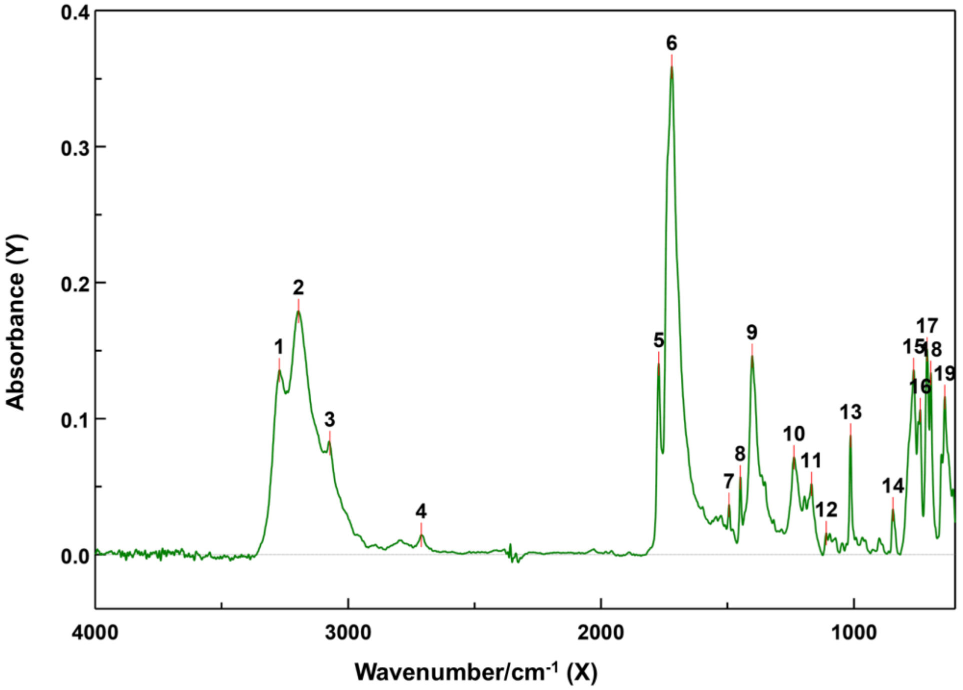

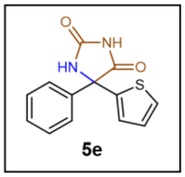

No.	X	Y	No.	X	Y	No.	X	Y
1	3271.6	0.135	8	1449.2	0.057	15	763.7	0.136
2	3196.4	0.179	9	1402	0.146	16	738.6	0.106
3	3072	0.082	10	1237.1	0.071	17	710.6	0.151
4	2710.5	0.014	11	1167.7	0.052	18	696.2	0.133
5	1772.3	0.14	12	1109.8	0.016	19	641.2	0.116
6	1720.2	0.359	13	1013.4	0.087			
7	1493.6	0.036	14	845.6	0.033			

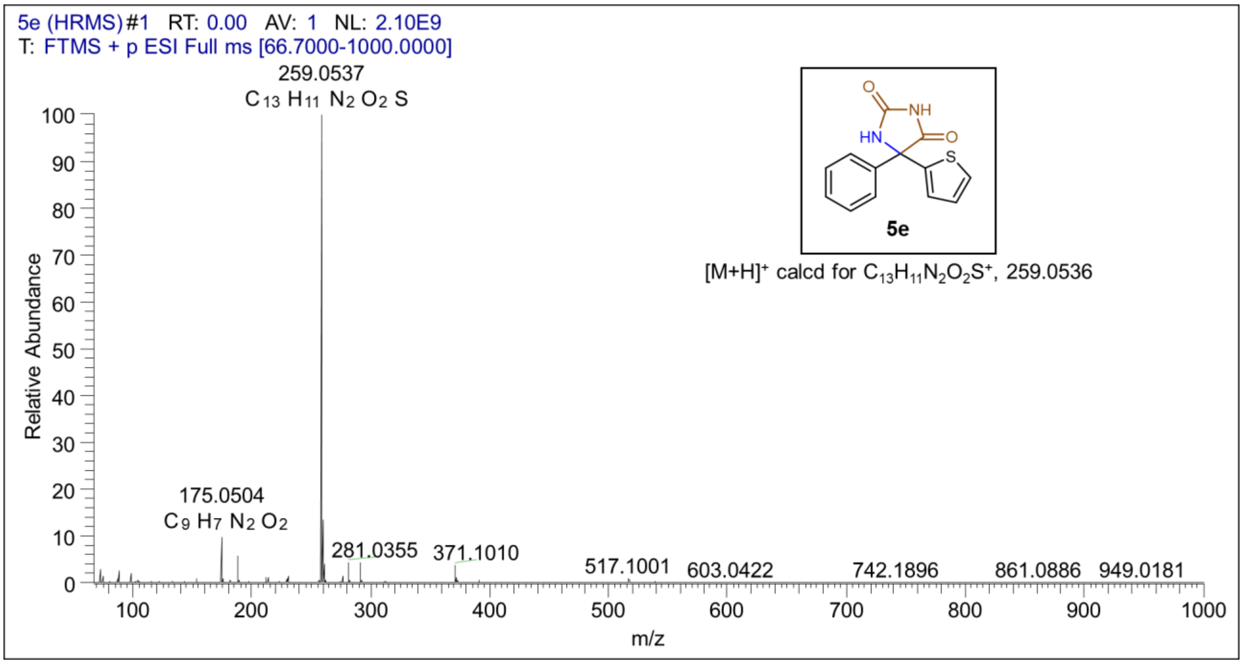

^{1}H NMR (400 MHz, DMSO-d_6) and ^{13}C NMR (100 MHz, DMSO-d_6) spectra for **5f**

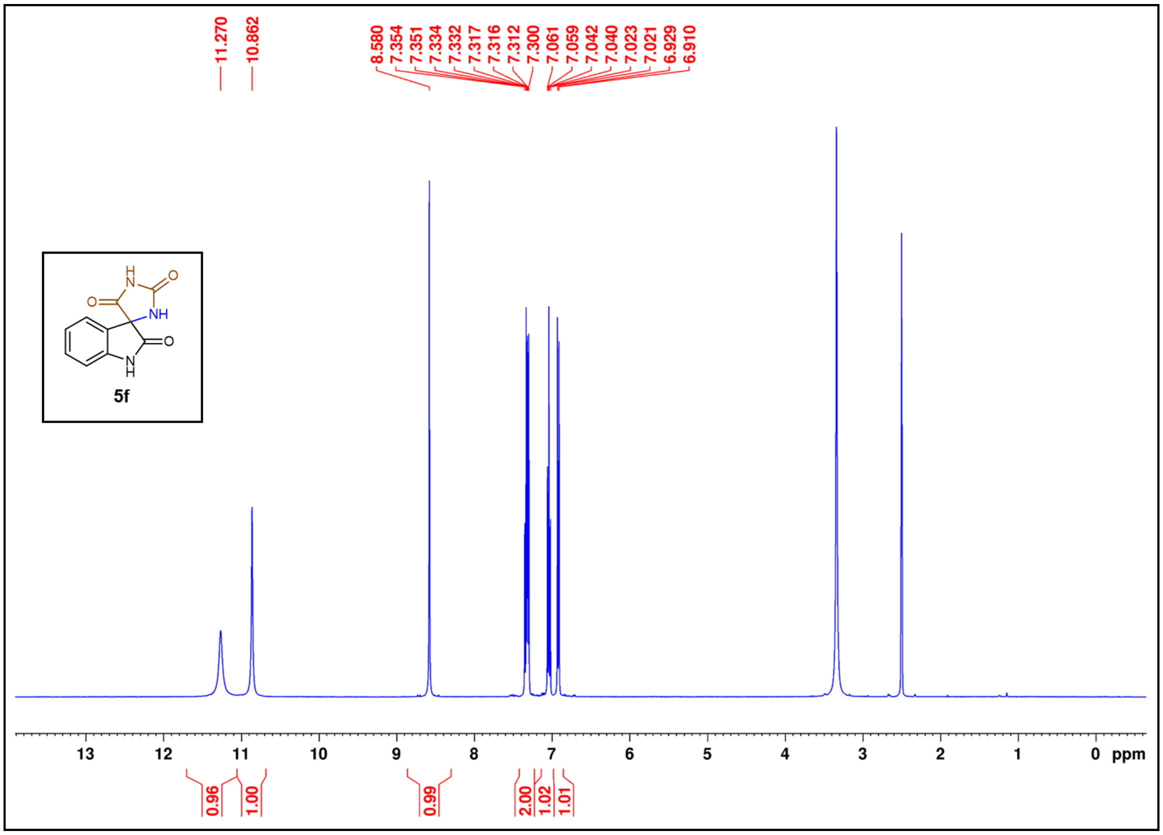

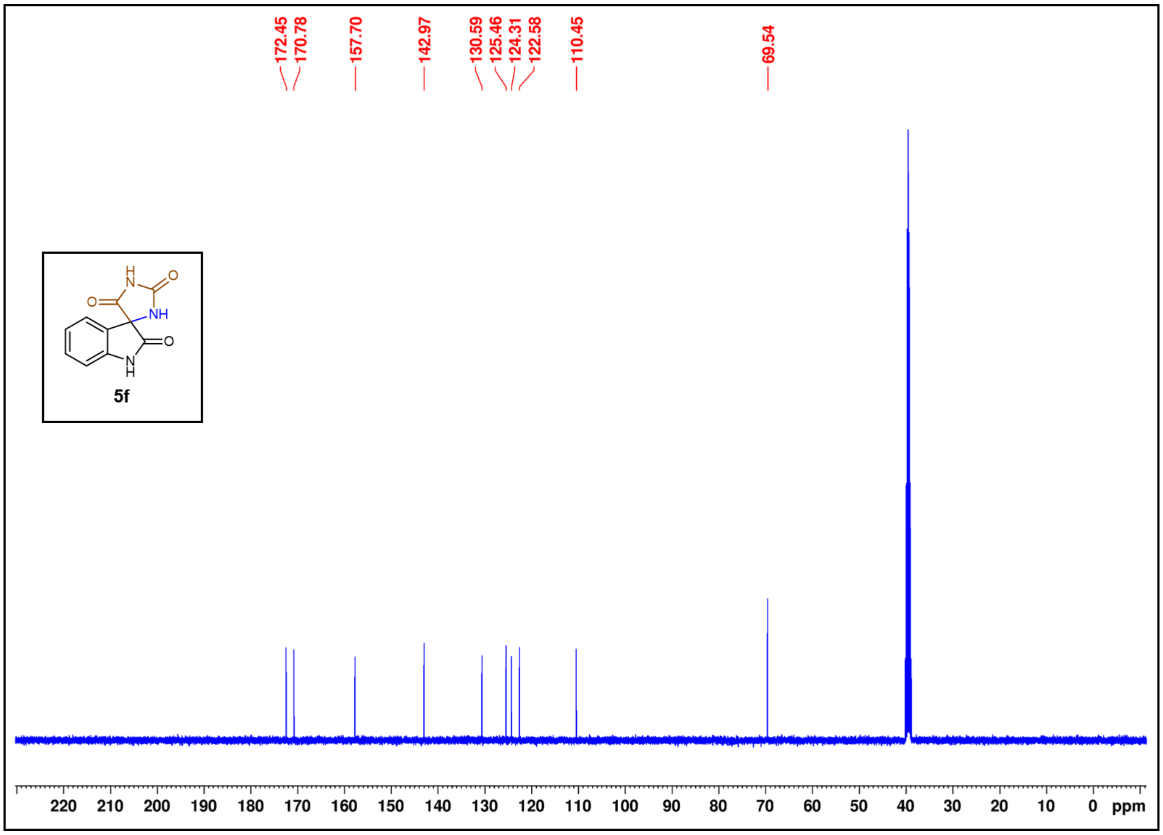

FT-IR (ATR, neat) and HRMS (ESI-positive) spectra for **5f**

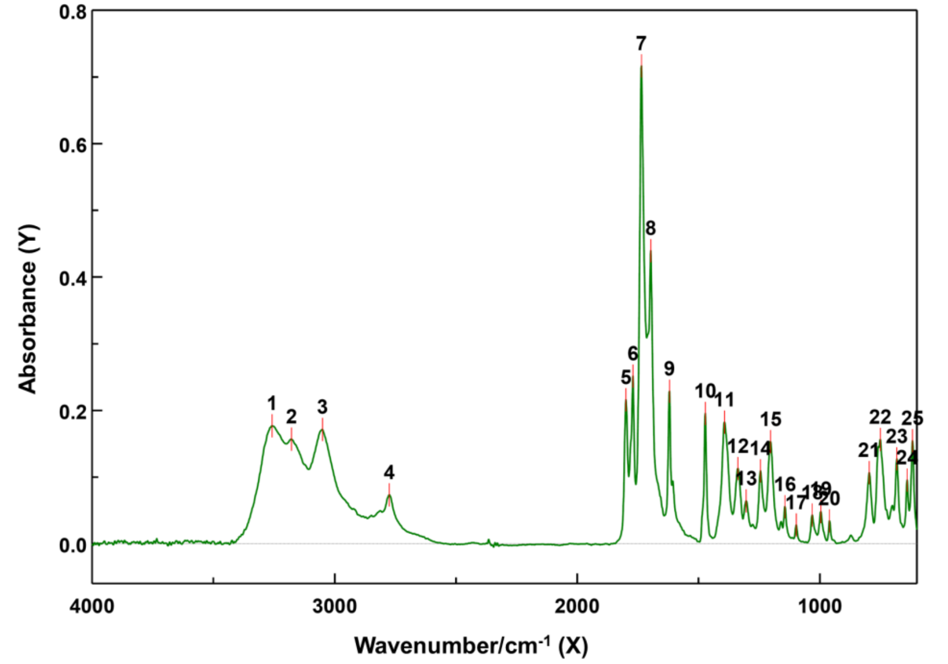

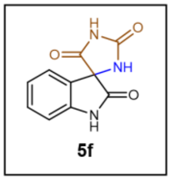

No.	X	Y	No.	X	Y	No.	X	Y
1	3258.1	0.177	10	1472.4	0.195	19	996.1	0.048
2	3178.1	0.156	11	1393.3	0.182	20	960.4	0.034
3	3050.8	0.171	12	1338.4	0.112	21	796.5	0.106
4	2775.1	0.073	13	1303.6	0.064	22	751.1	0.156
5	1799.3	0.216	14	1244.8	0.109	23	682.7	0.127
6	1770.3	0.251	15	1203.4	0.153	24	640.3	0.095
7	1735.6	0.717	16	1143.6	0.055	25	618.1	0.154
8	1697.1	0.439	17	1097.3	0.028			
9	1619.9	0.228	18	1031.7	0.043			

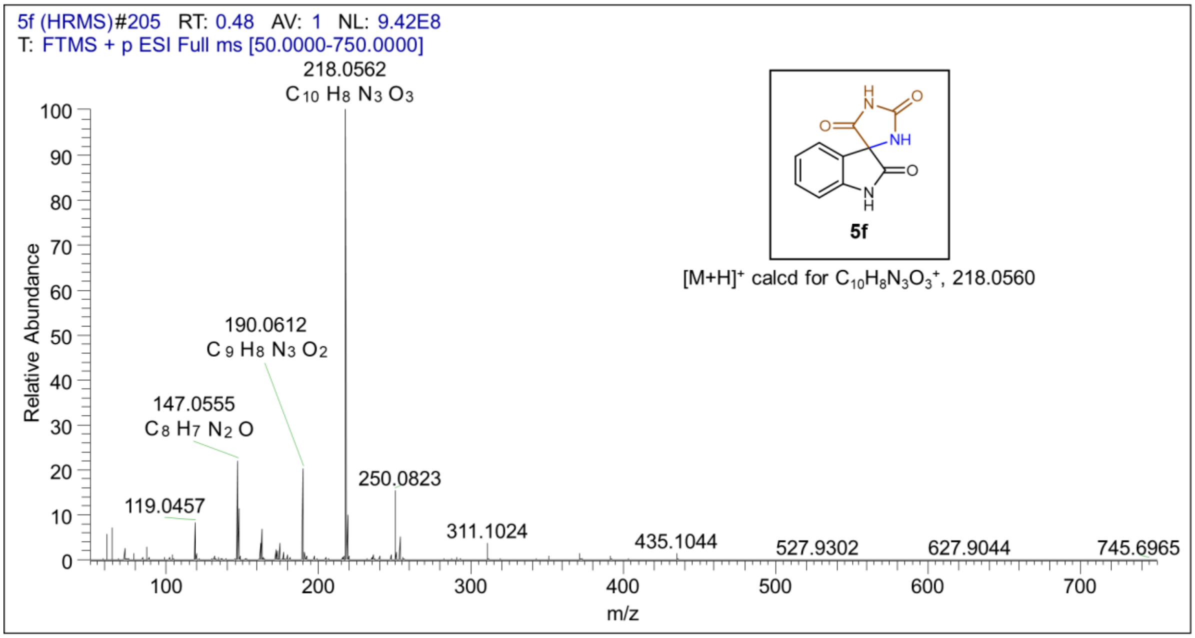

[M+H]+ calcd for $C_{10}H_8N_3O_3^+$, 218.0560

^{1}H NMR (400 MHz, DMSO-d_6) and ^{13}C NMR (100 MHz, DMSO-d_6) spectra for **5g**

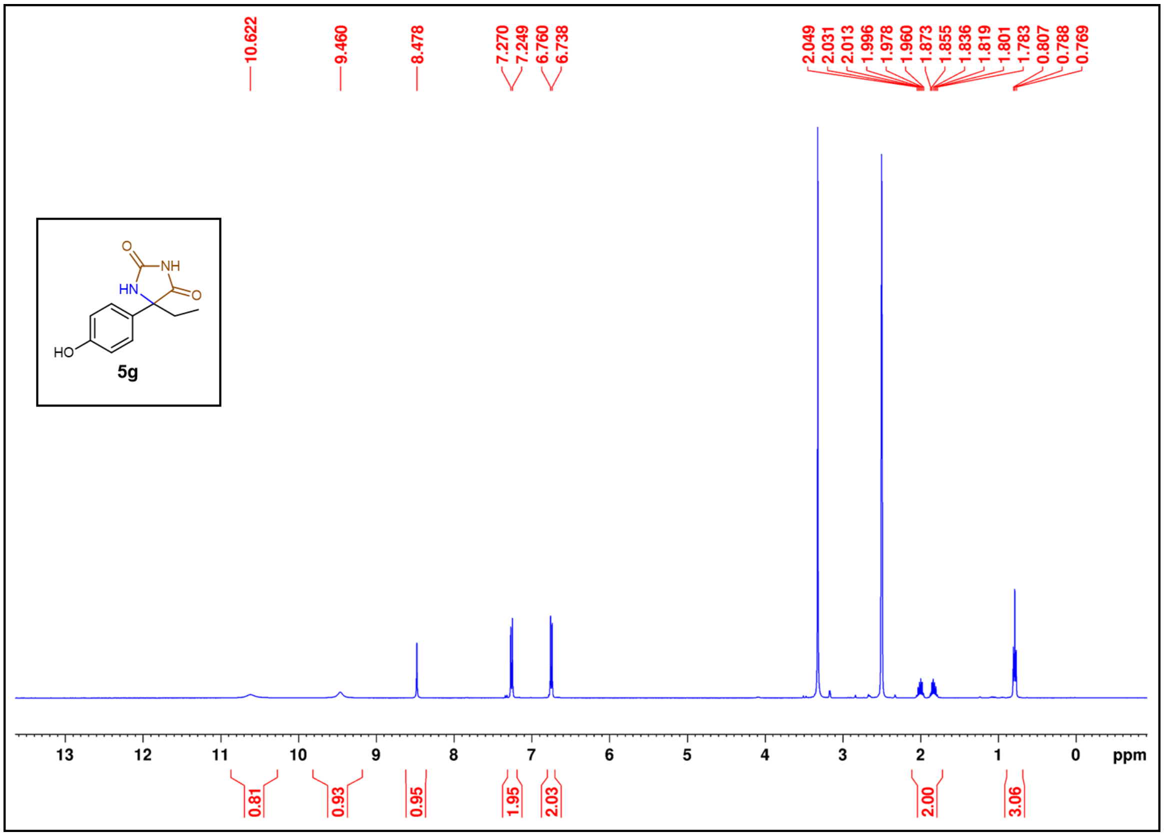

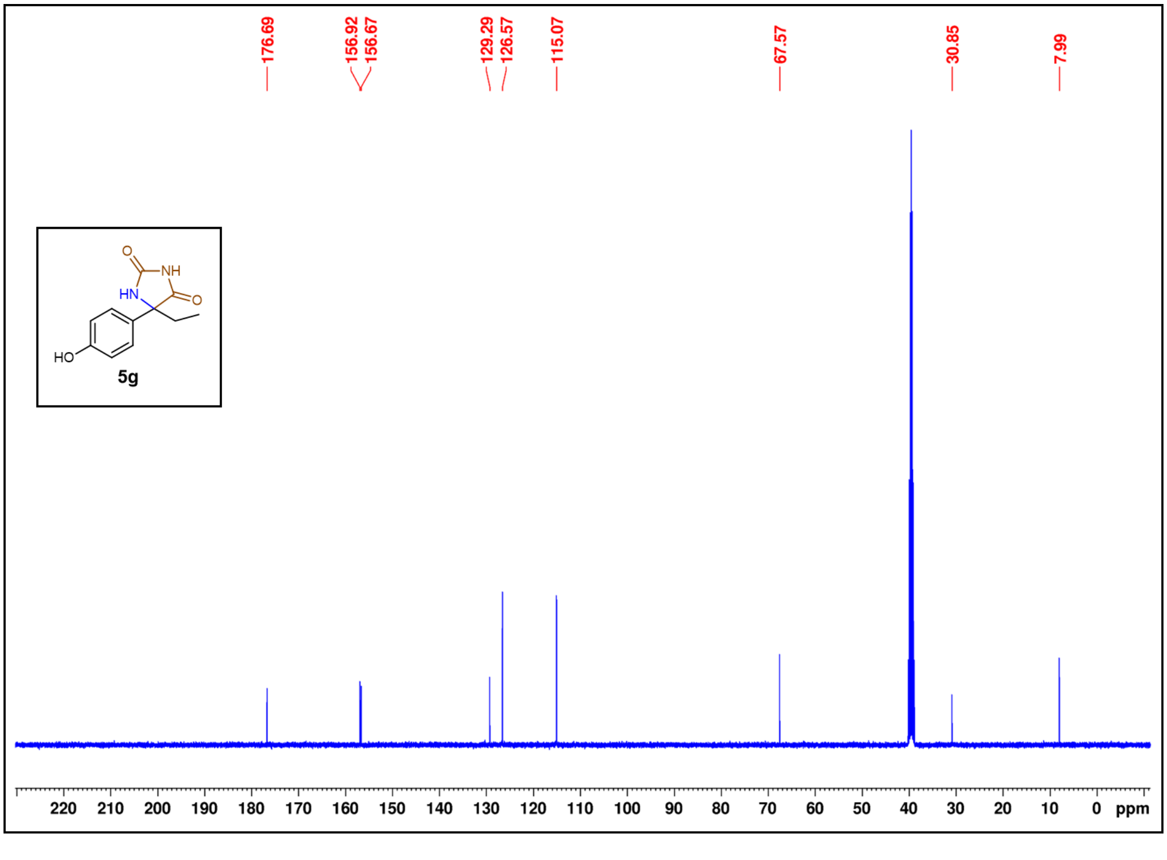

FT-IR (ATR, neat) and HRMS (ESI-positive) spectra for **5g**

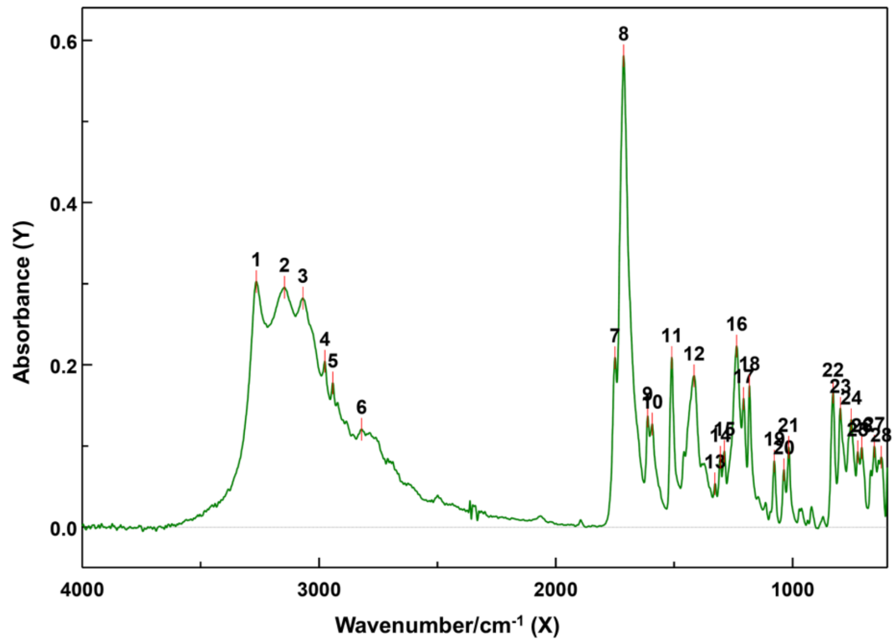

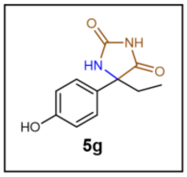

No.	X	Y	No.	X	Y	No.	X	Y
1	3264.9	0.302	11	1510	0.209	21	1016	0.098
2	3146.3	0.295	12	1416.5	0.186	22	829.2	0.166
3	3068.2	0.282	13	1327.7	0.053	23	798.4	0.146
4	2975.6	0.204	14	1305.6	0.086	24	752.1	0.132
5	2941.9	0.177	15	1288.2	0.093	25	725.1	0.093
6	2820.4	0.12	16	1236.1	0.223	26	707.7	0.098
7	1750.1	0.209	17	1207.2	0.158	27	654.7	0.099
8	1713.4	0.581	18	1182.2	0.174	28	625.8	0.086
9	1612.2	0.137	19	1077	0.081			
10	1592.9	0.127	20	1036.6	0.07			

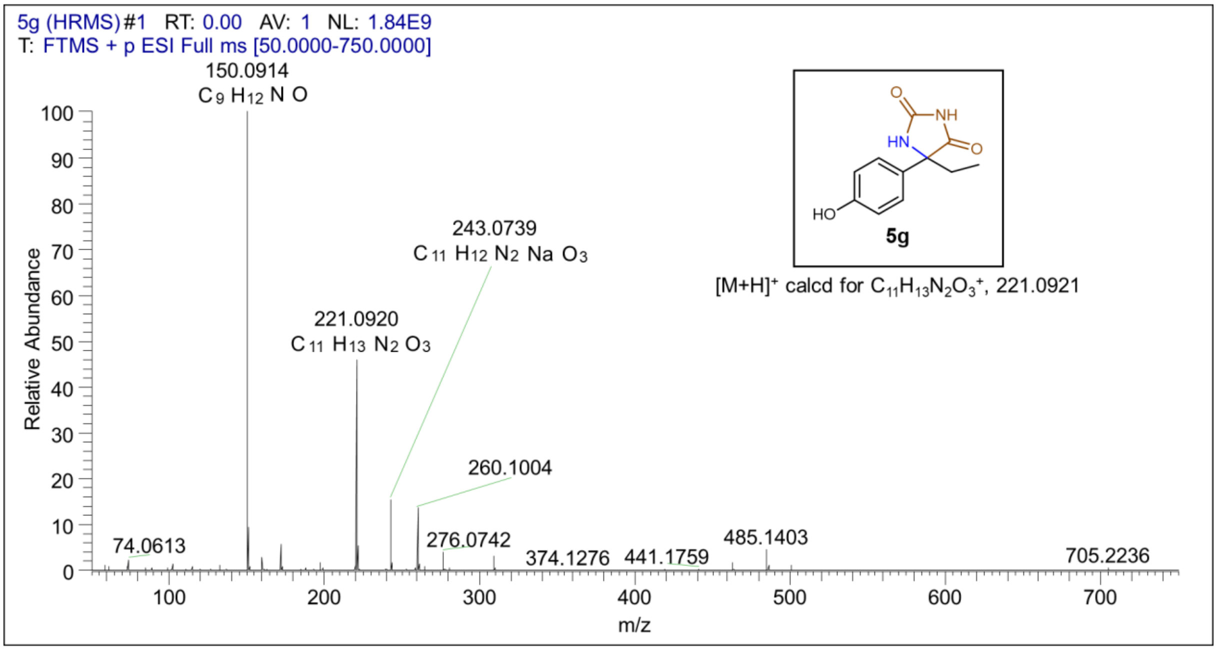

^1H NMR (400 MHz, DMSO-d_6) and ^{13}C NMR (100 MHz, DMSO-d_6) spectra for **5h**

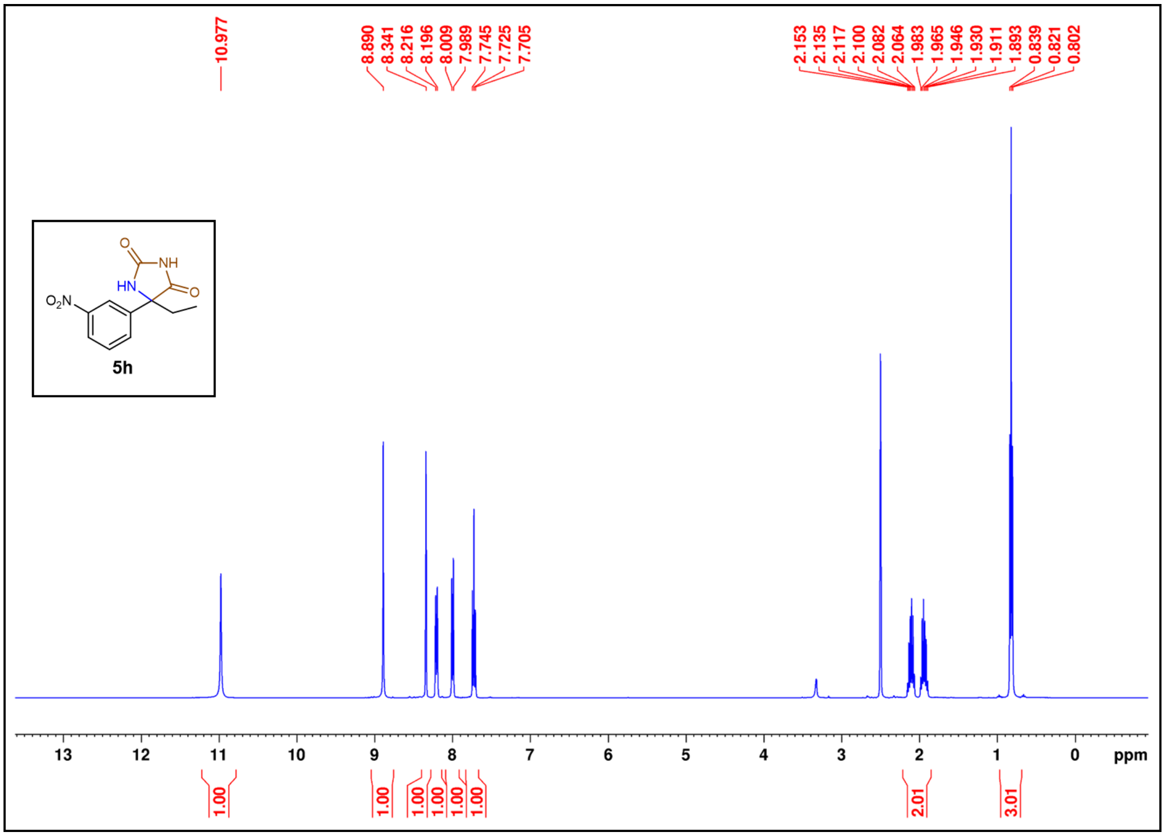

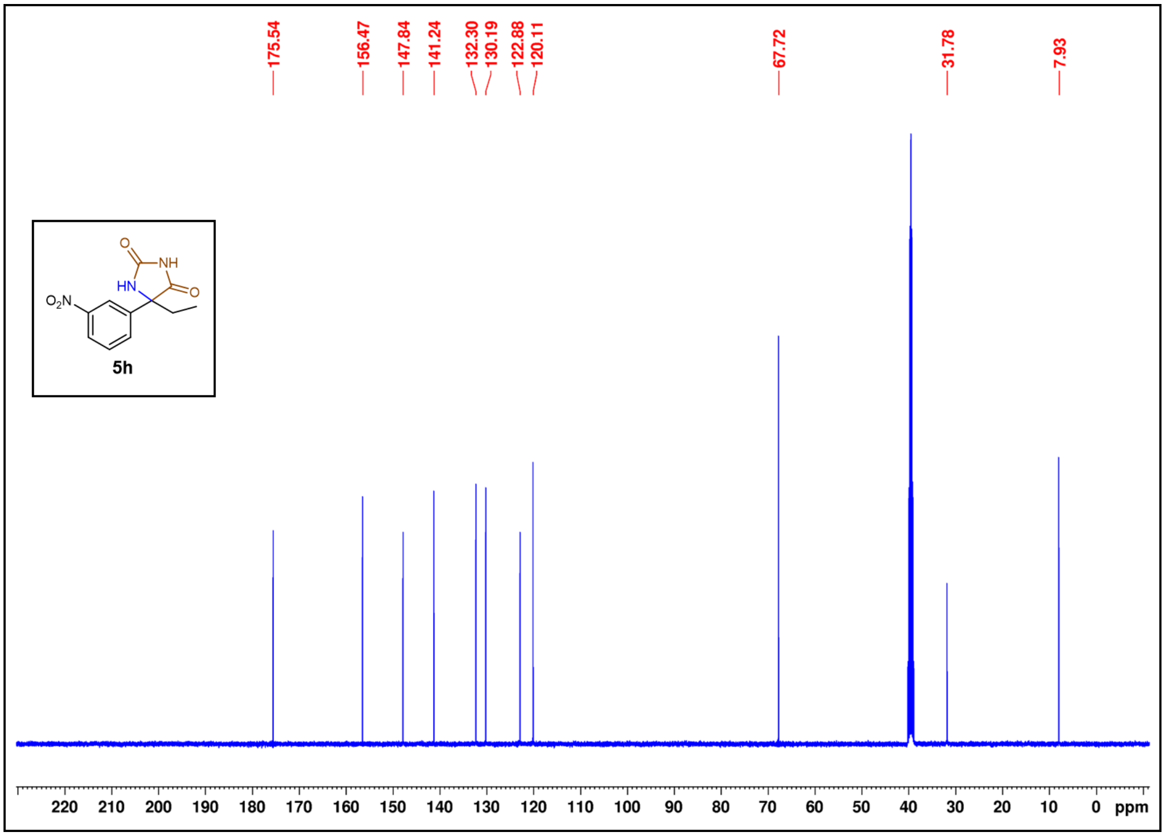

FT-IR (ATR, neat) and HRMS (ESI-negative) spectra for **5h**

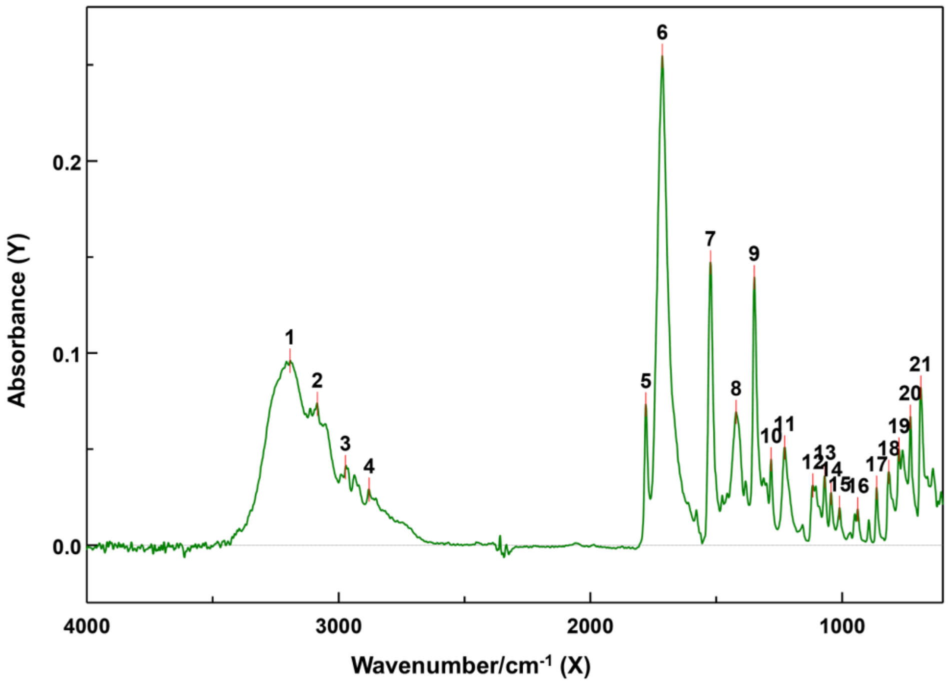

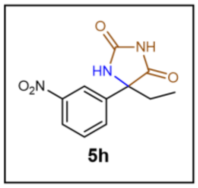

No.	X	Y	No.	X	Y	No.	X	Y
1	3192.6	0.096	8	1421.3	0.069	15	1011	0.019
2	3084.6	0.073	9	1349	0.139	16	938.2	0.019
3	2972.7	0.041	10	1282.4	0.045	17	863	0.03
4	2879.2	0.029	11	1228.4	0.051	18	814.8	0.038
5	1780	0.073	12	1116.6	0.031	19	774.3	0.05
6	1714.4	0.255	13	1069.3	0.036	20	729	0.067
7	1522.5	0.147	14	1044.3	0.027	21	687.5	0.082

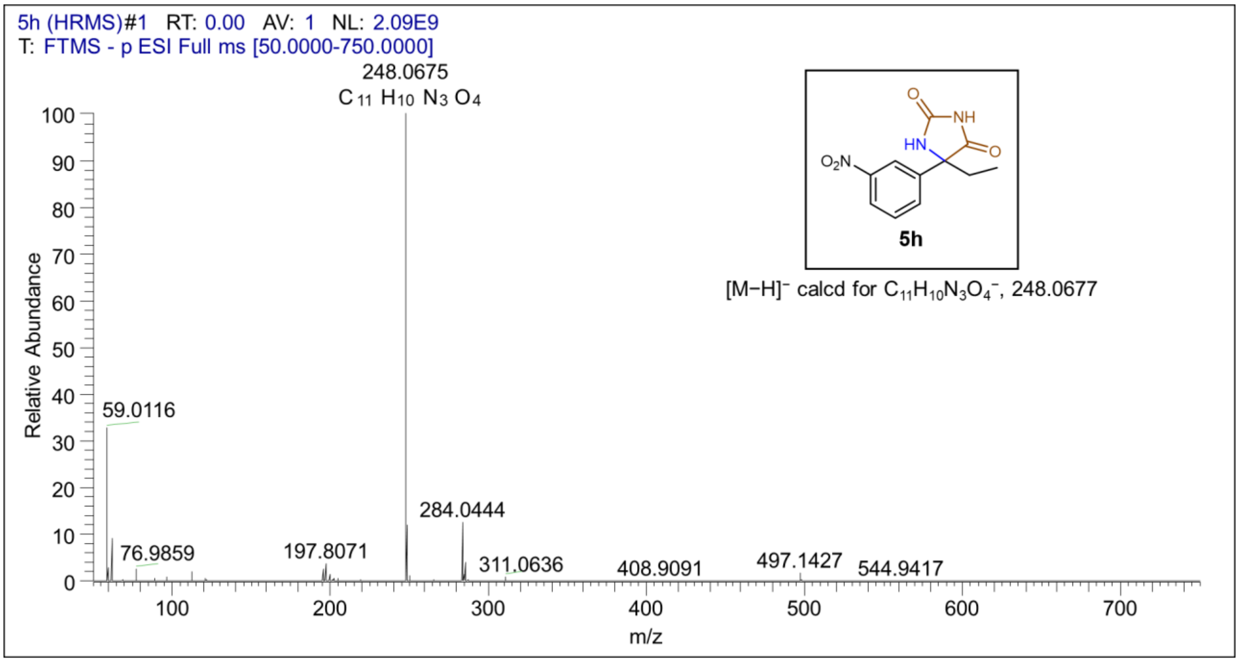

^{1}H NMR (400 MHz, DMSO-d_6) and ^{13}C NMR (100 MHz, DMSO-d_6) spectra for **5i**

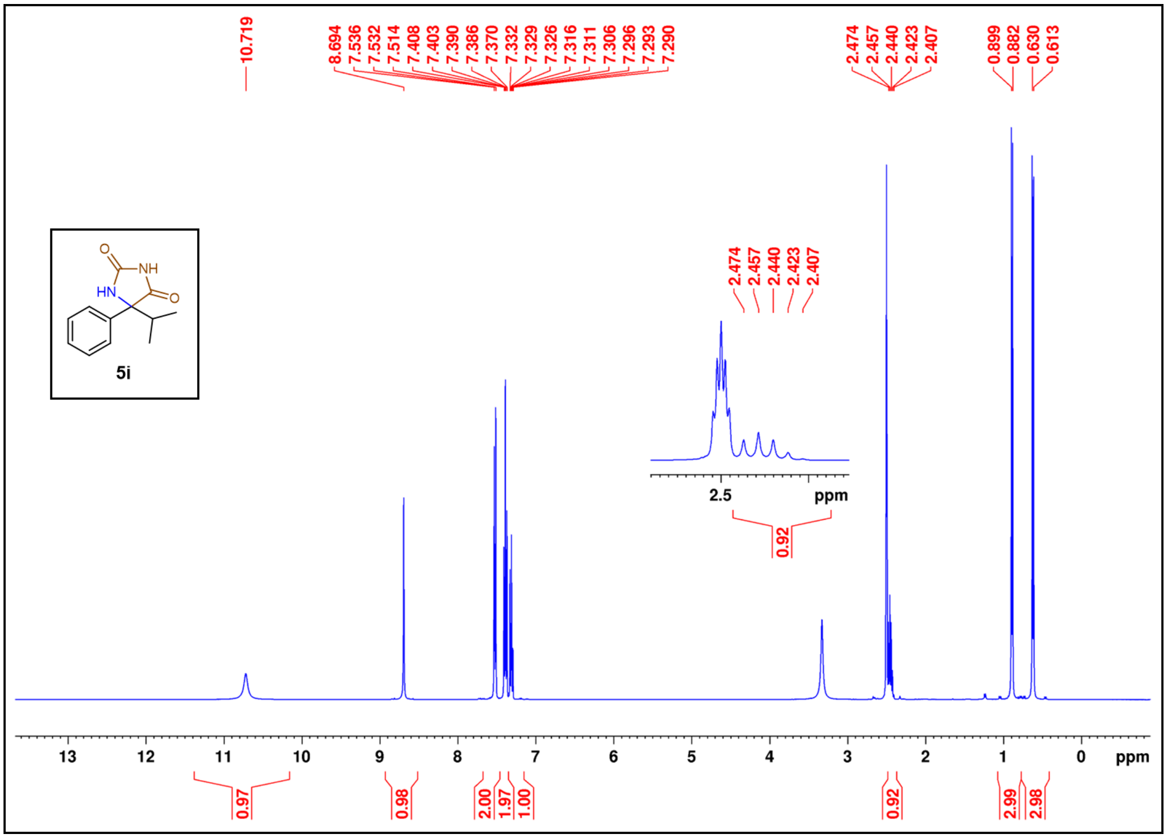

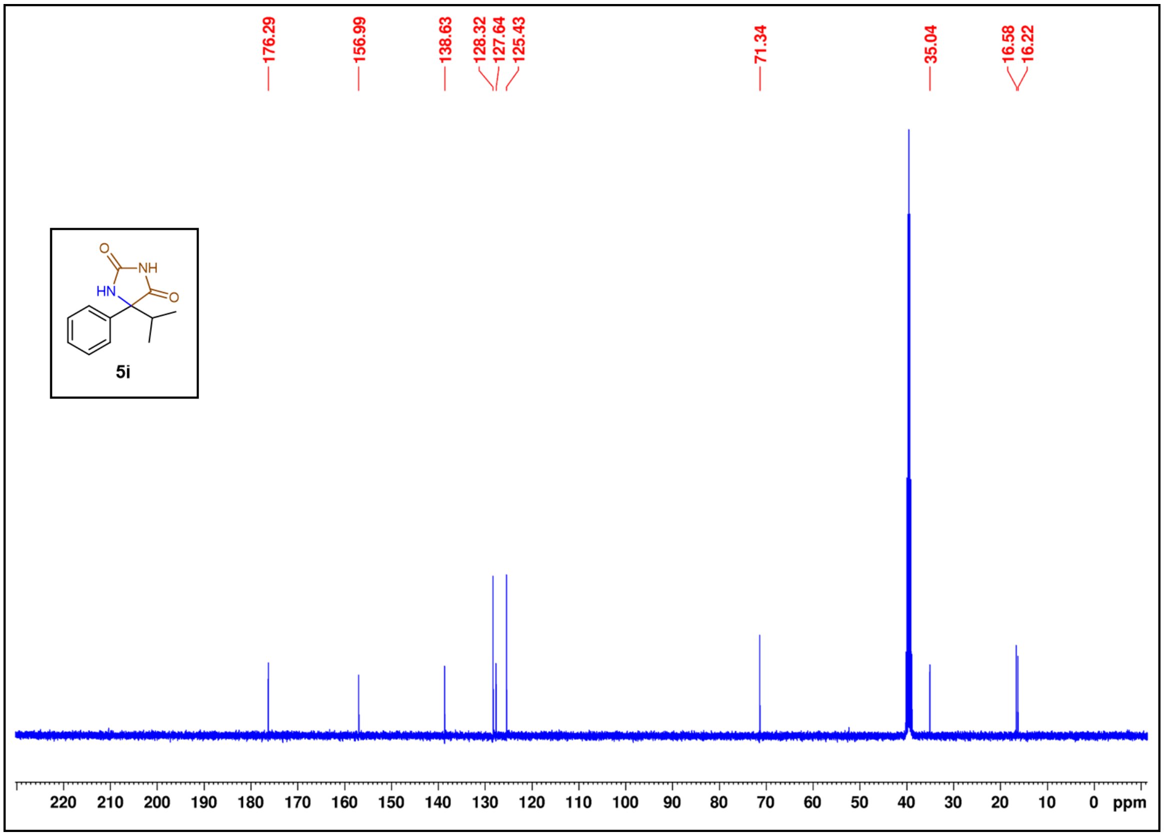

FT-IR (ATR, neat) and HRMS (ESI-positive) spectra for **5i**

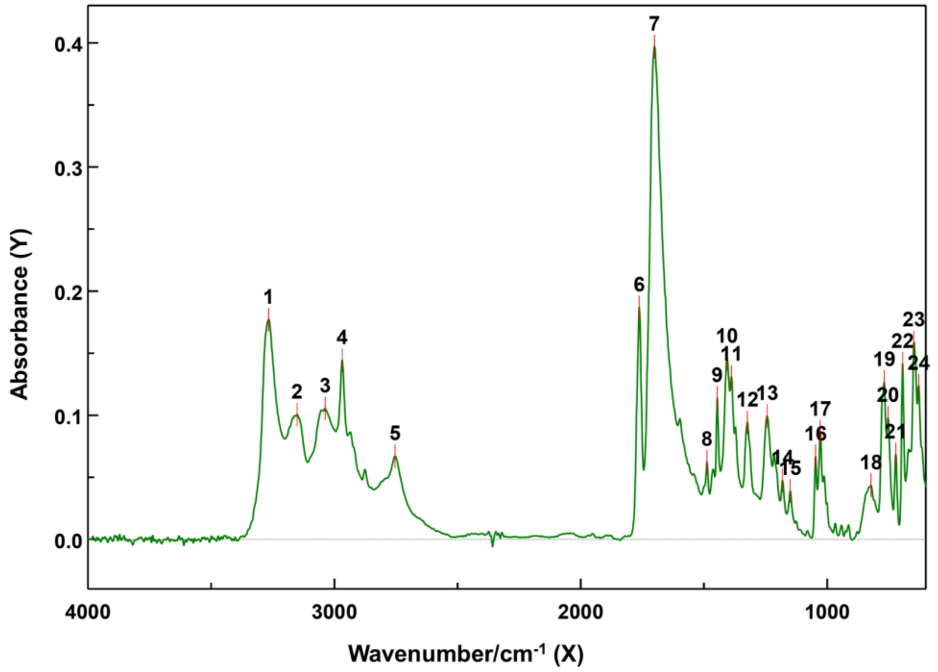

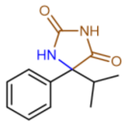

5i

No.	X	Y	No.	X	Y	No.	X	Y
1	3266.8	0.177	9	1446.4	0.114	17	1028	0.087
2	3151.1	0.1	10	1406.8	0.145	18	822.5	0.043
3	3037.3	0.105	11	1388.5	0.131	19	768.5	0.127
4	2967.9	0.144	12	1323.9	0.094	20	753.1	0.098
5	2753.9	0.067	13	1243.9	0.099	21	721.2	0.068
6	1762.6	0.187	14	1181.2	0.047	22	694.2	0.141
7	1700.9	0.397	15	1150.3	0.039	23	648	0.159
8	1487.8	0.062	16	1047.2	0.067	24	628.7	0.124

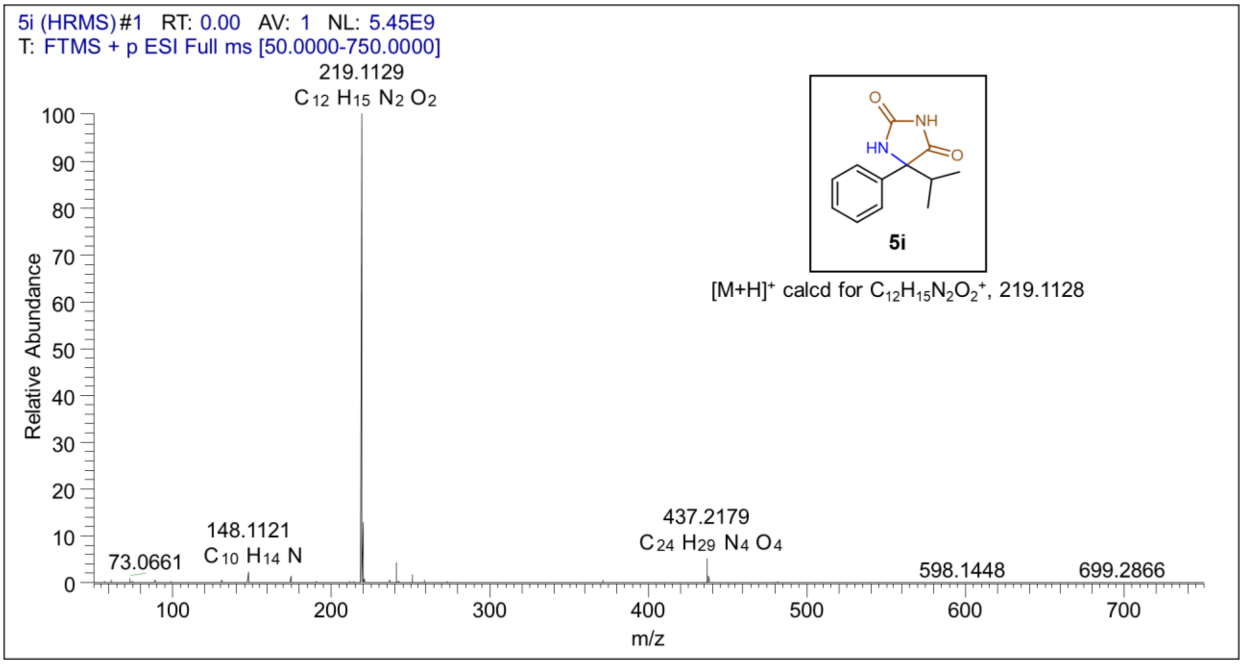

^{1}H NMR (400 MHz, DMSO-d_6) and ^{13}C NMR (100 MHz, DMSO-d_6) spectra for **5j**

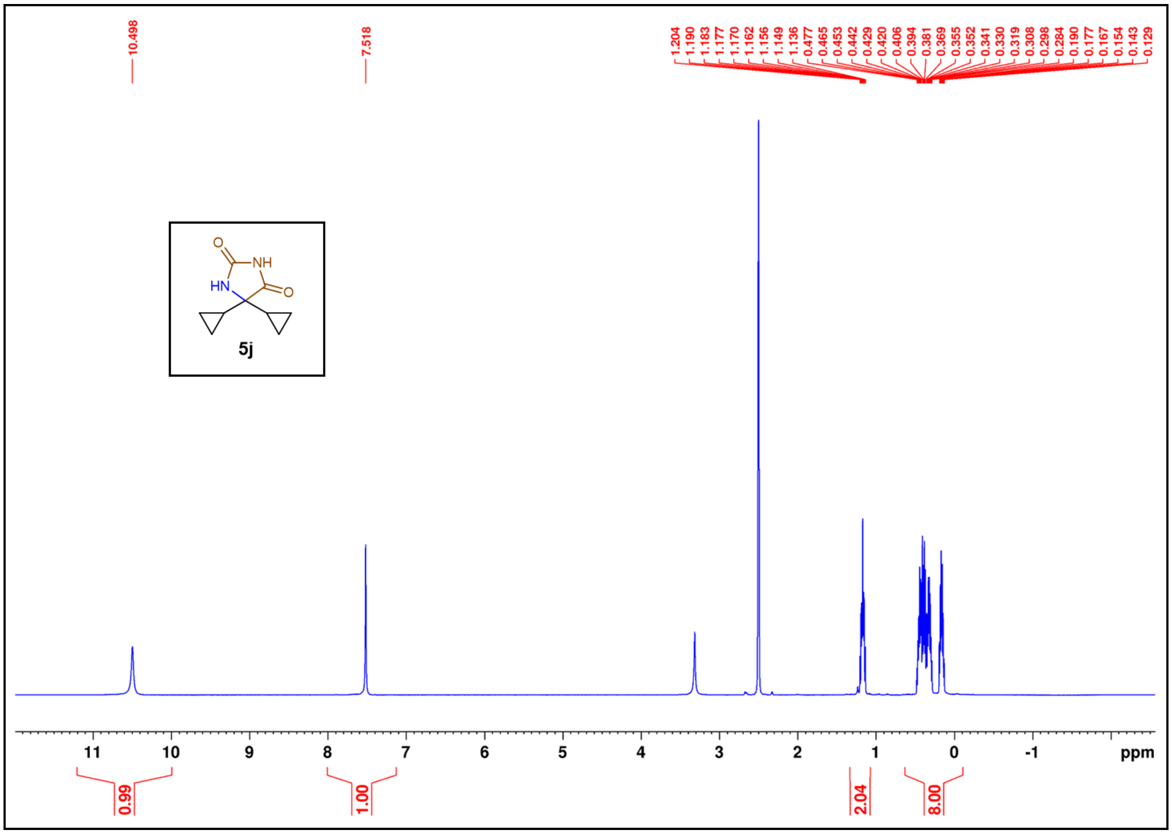

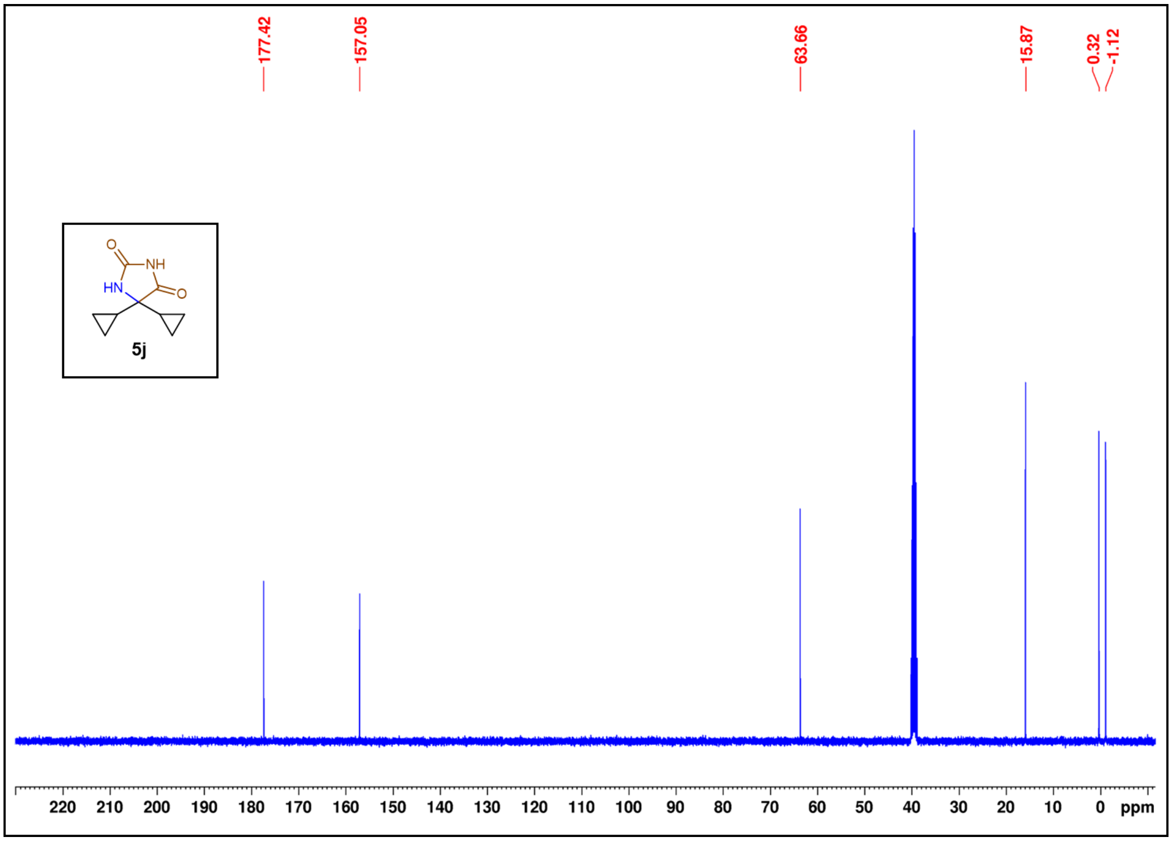

FT-IR (ATR, neat) and HRMS (ESI-positive) spectra for **5j**

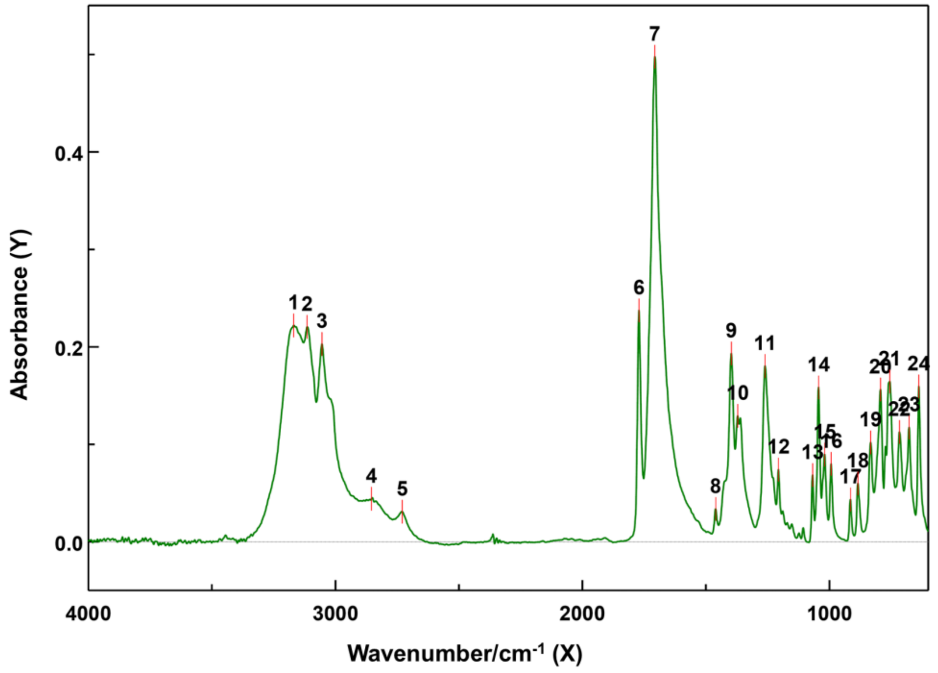

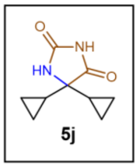

No.	X	Y	No.	X	Y	No.	X	Y
1	3168.5	0.222	9	1397.2	0.193	17	915.1	0.043
2	3114.5	0.22	10	1371.1	0.129	18	885.2	0.06
3	3054.7	0.203	11	1260.3	0.18	19	833.1	0.102
4	2854.1	0.044	12	1206.3	0.074	20	793.6	0.156
5	2729.7	0.031	13	1068.4	0.068	21	756	0.164
6	1771.3	0.237	14	1044.3	0.158	22	716.4	0.113
7	1706.7	0.497	15	1019.2	0.089	23	677.9	0.117
8	1460.8	0.034	16	993.2	0.08	24	638.3	0.159

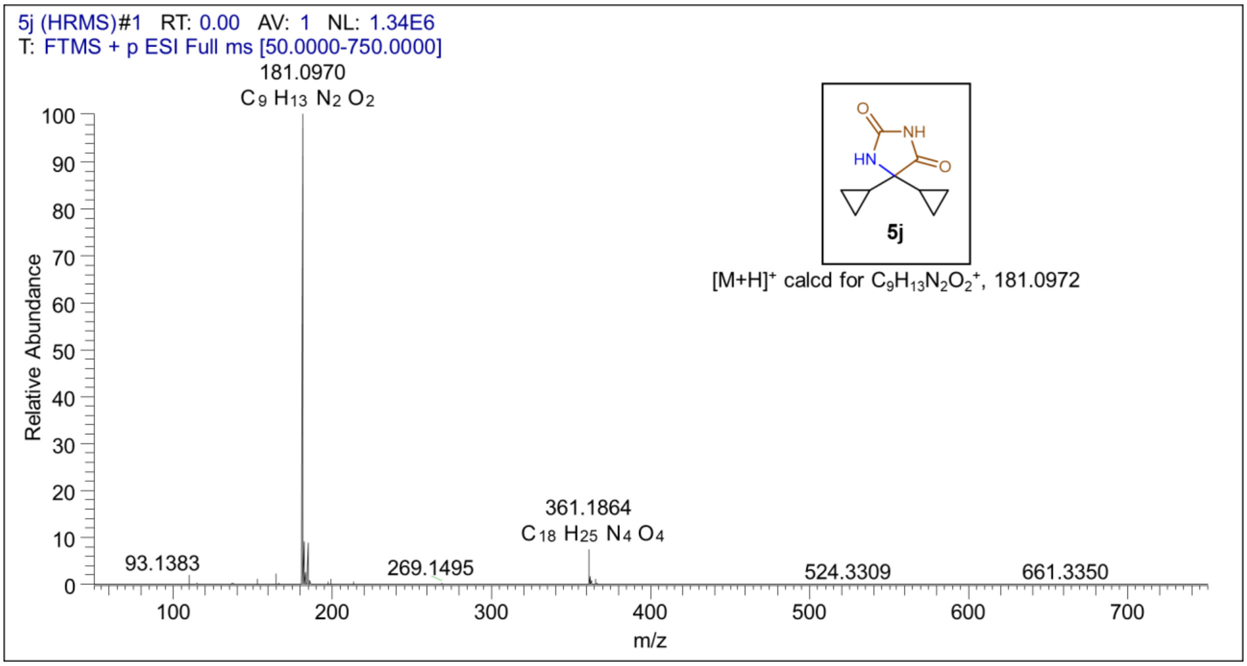

^{1}H NMR (400 MHz, DMSO-d_6) and ^{13}C NMR (100 MHz, DMSO-d_6) spectra for **5k**

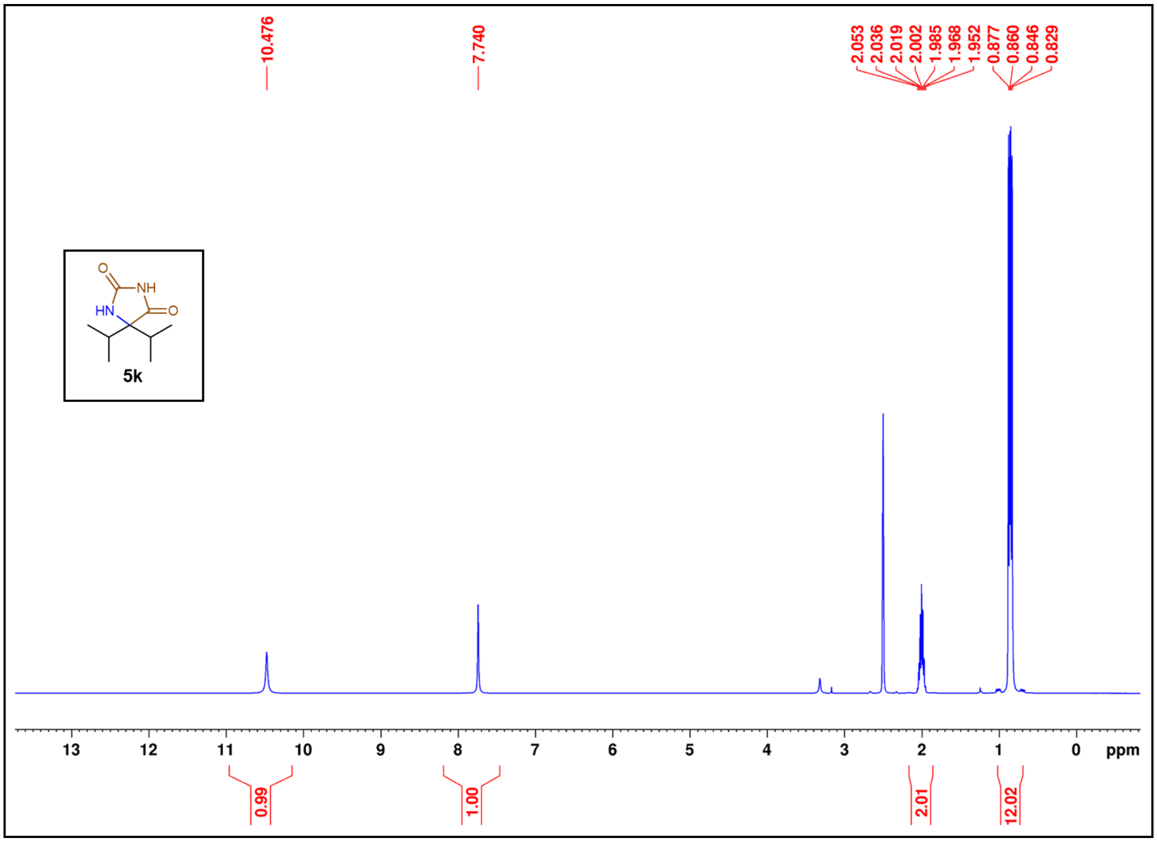

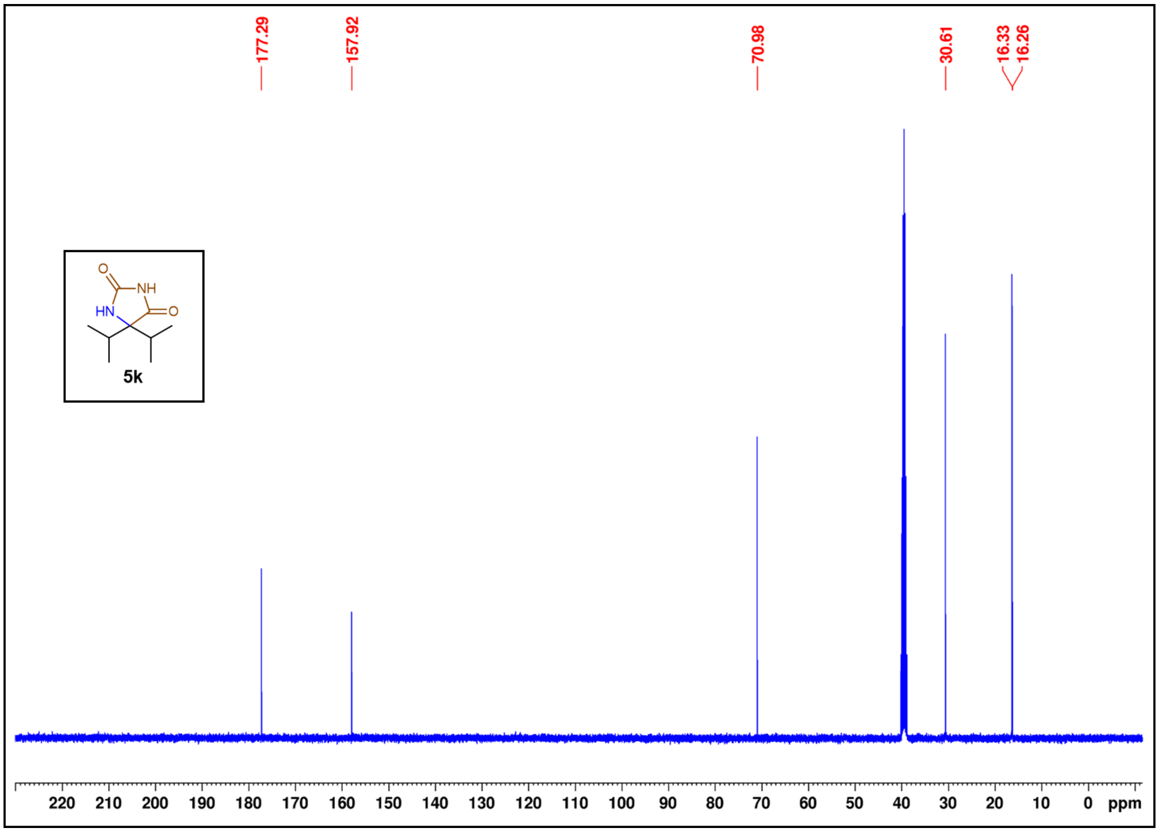

FT-IR (ATR, neat) and HRMS (ESI-positive) spectra for **5k**

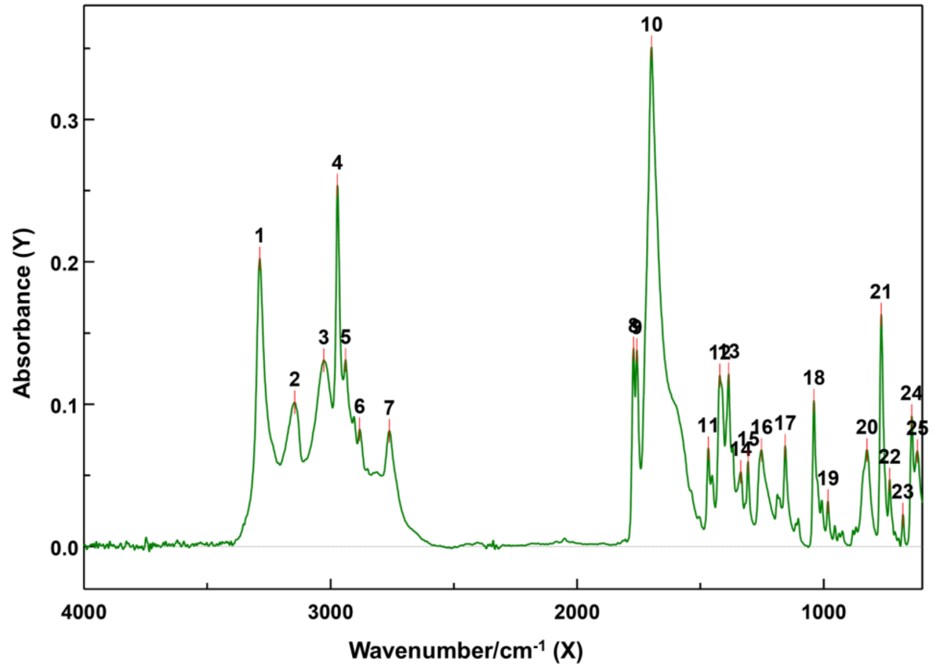

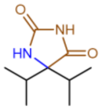

5k

No.	X	Y	No.	X	Y	No.	X	Y
1	3287.1	0.202	10	1698	0.351	19	982.6	0.032
2	3145.3	0.101	11	1467.6	0.069	20	824.4	0.068
3	3026.7	0.131	12	1421.3	0.12	21	766.6	0.163
4	2971.8	0.254	13	1385.6	0.121	22	732.8	0.047
5	2939	0.131	14	1336.4	0.052	23	678.8	0.022
6	2882.1	0.082	15	1306.5	0.059	24	643.1	0.091
7	2761.6	0.081	16	1252.5	0.068	25	620	0.067
8	1771.3	0.139	17	1156.1	0.07			
9	1757.8	0.138	18	1039.4	0.102			

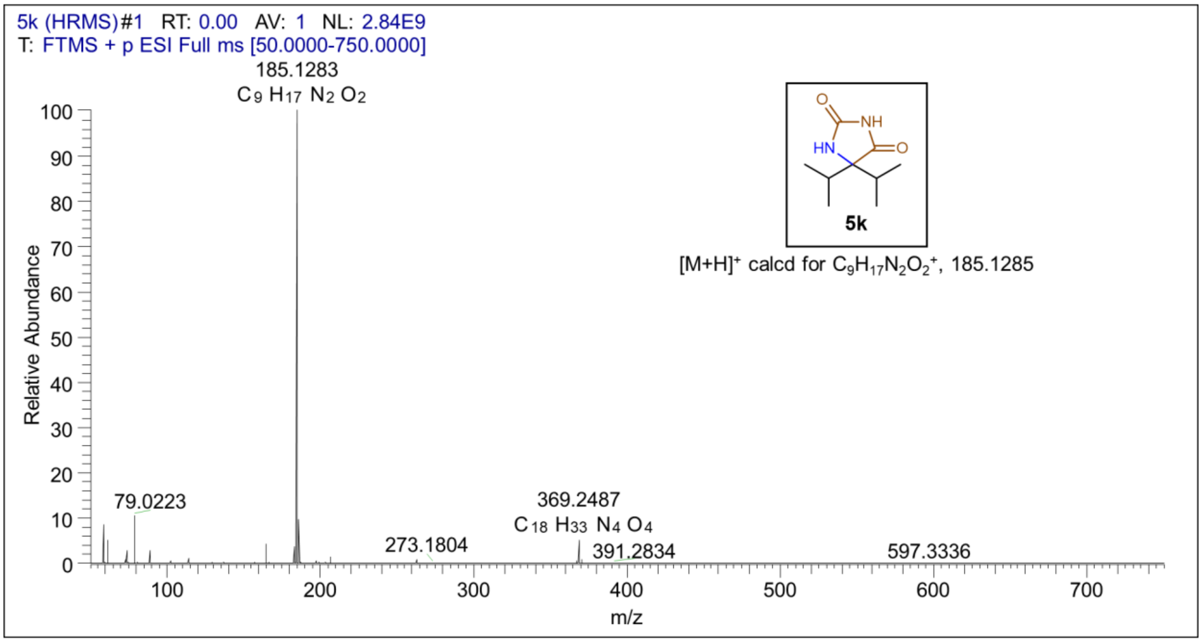

^{1}H NMR (400 MHz, DMSO-d_6) and ^{13}C NMR (100 MHz, DMSO-d_6) spectra for **5l**

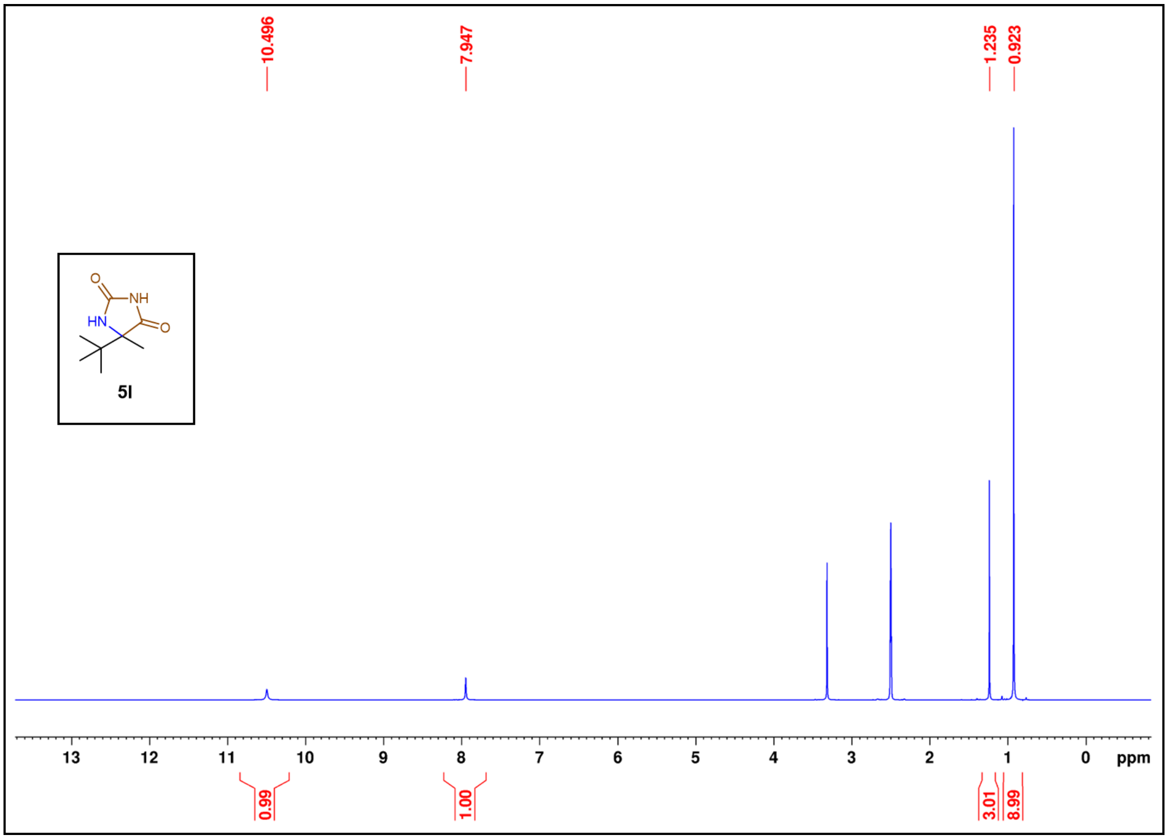

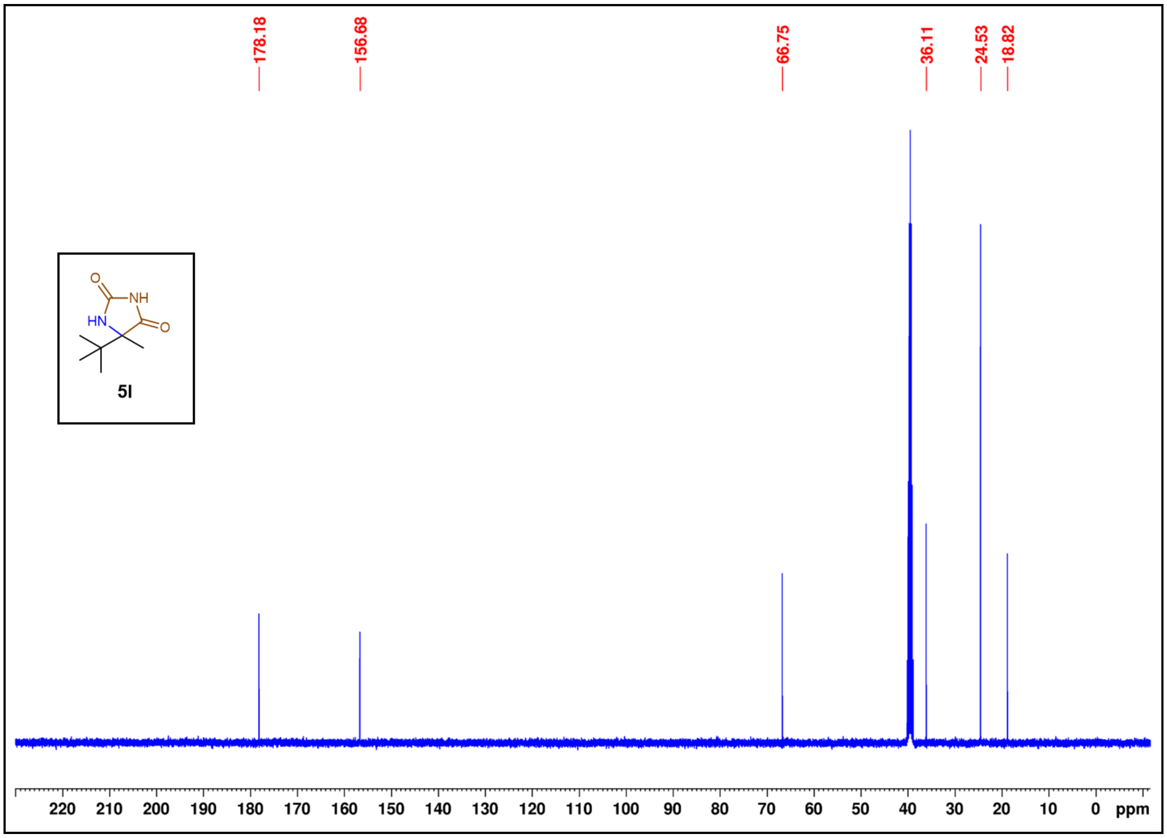

FT-IR (ATR, neat) and HRMS (ESI-positive) spectra for **5l**

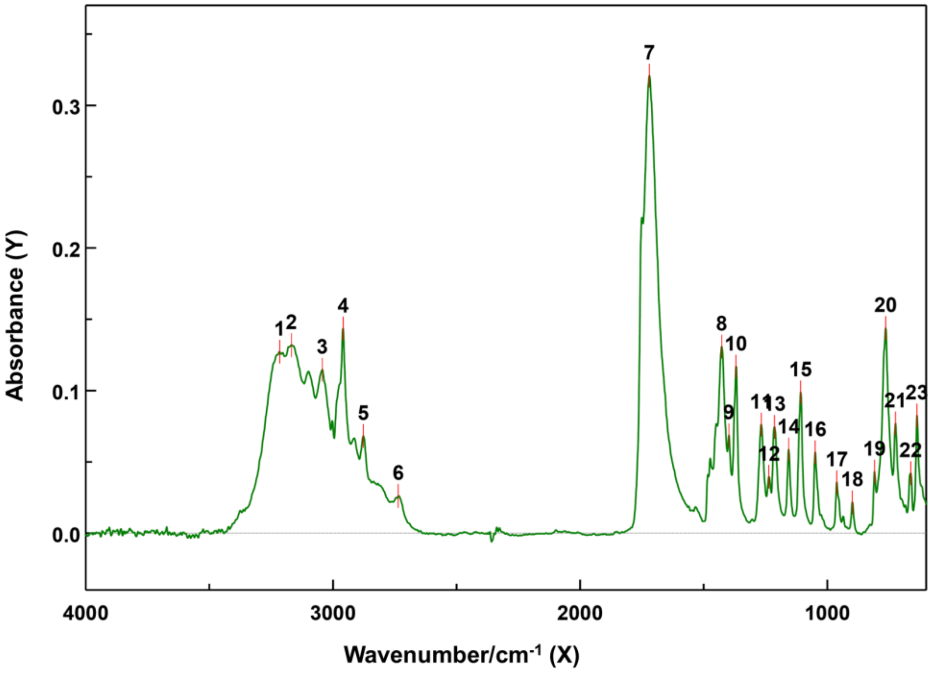

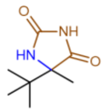

5l

No.	X	Y	No.	X	Y	No.	X	Y
1	3215.7	0.127	9	1398.1	0.068	17	961.3	0.036
2	3167.5	0.132	10	1369.2	0.117	18	898.7	0.021
3	3043.1	0.115	11	1267	0.076	19	809	0.043
4	2959.2	0.143	12	1236.1	0.039	20	763.7	0.144
5	2877.3	0.068	13	1214	0.074	21	724.1	0.077
6	2735.5	0.026	14	1156.1	0.058	22	662.4	0.042
7	1719.2	0.321	15	1107.9	0.099	23	637.4	0.082
8	1427.1	0.131	16	1049.1	0.057			

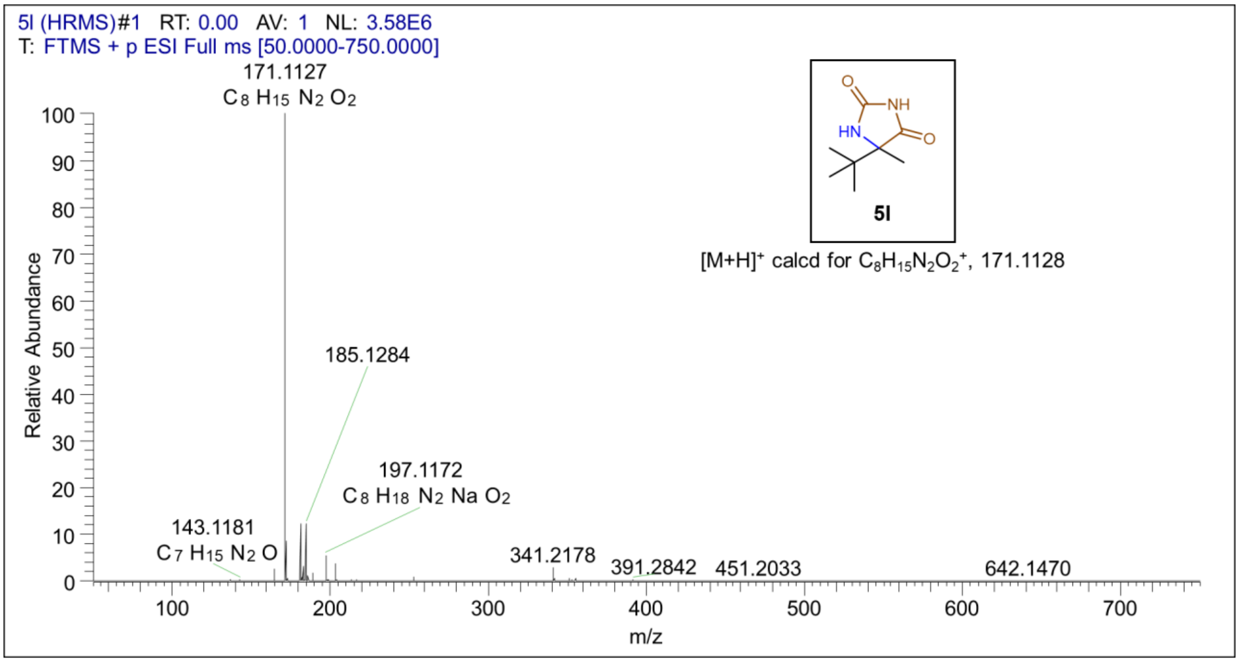

^{1}H NMR (400 MHz, DMSO-d_6) and ^{13}C NMR (100 MHz, DMSO-d_6) spectra for **5m**

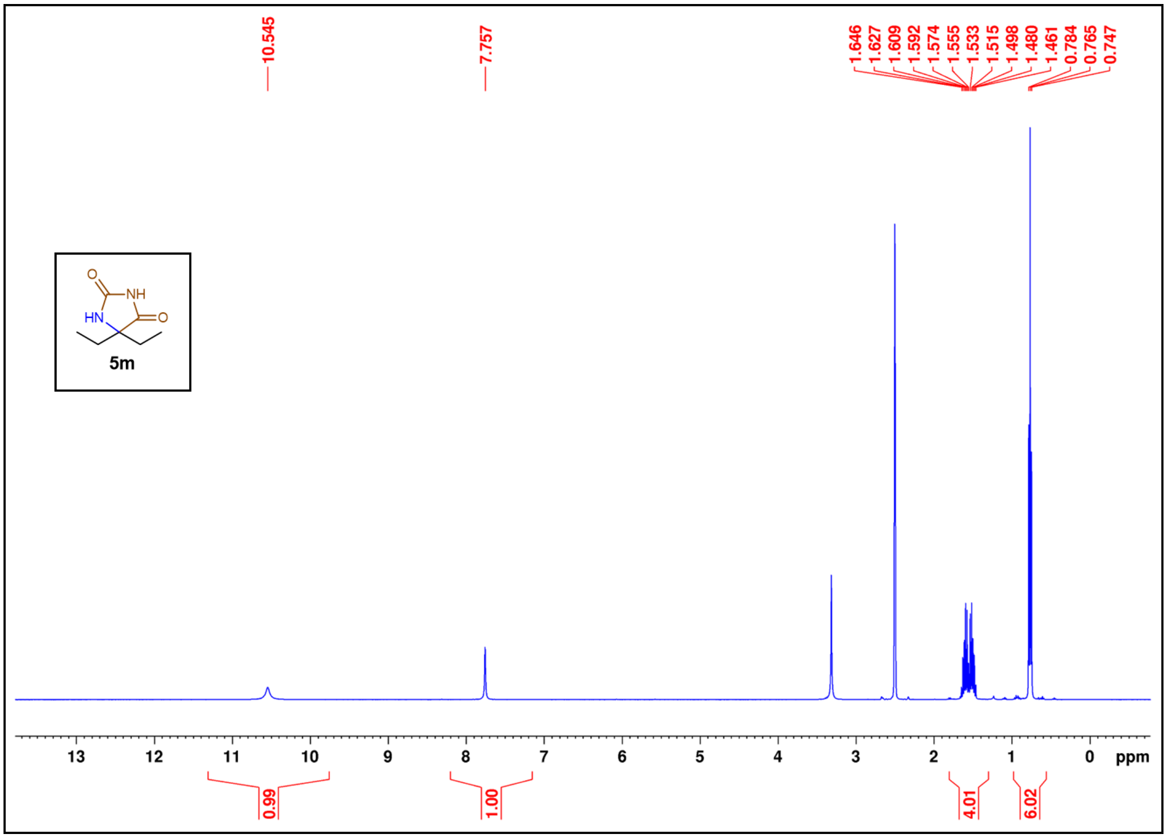

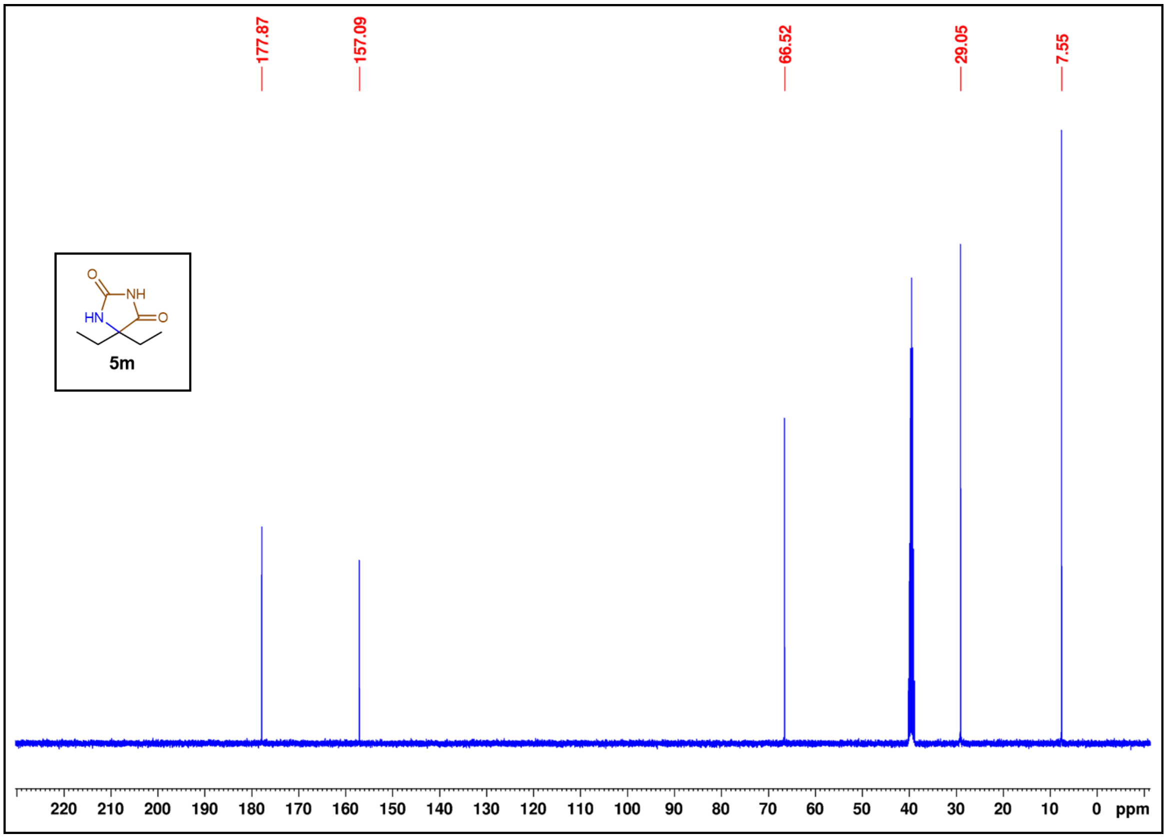

FT-IR (ATR, neat) and HRMS (ESI-positive) spectra for **5m**

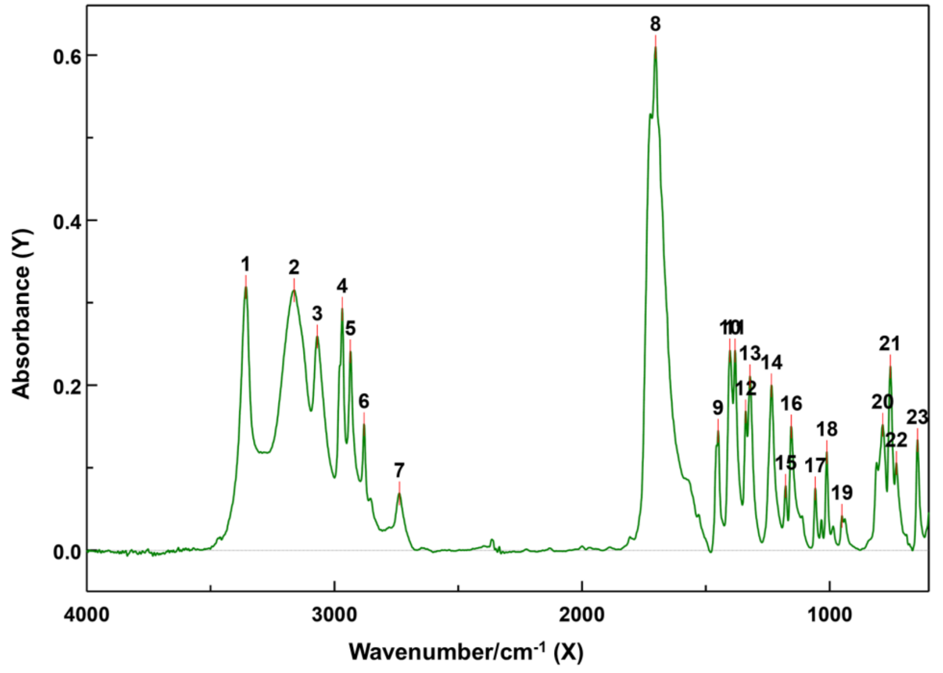

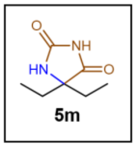

No.	X	Y	No.	X	Y	No.	X	Y
1	3357.5	0.319	9	1450.2	0.145	17	1058	0.075
2	3162.7	0.315	10	1403	0.242	18	1012	0.119
3	3069.2	0.259	11	1381.7	0.242	19	950.7	0.041
4	2967.9	0.292	12	1339.3	0.168	20	785.9	0.152
5	2935.1	0.241	13	1322	0.211	21	755	0.223
6	2880.2	0.153	14	1235.2	0.2	22	730.9	0.105
7	2737.5	0.069	15	1178.3	0.078	23	645.1	0.133
8	1702.8	0.61	16	1155.2	0.15			

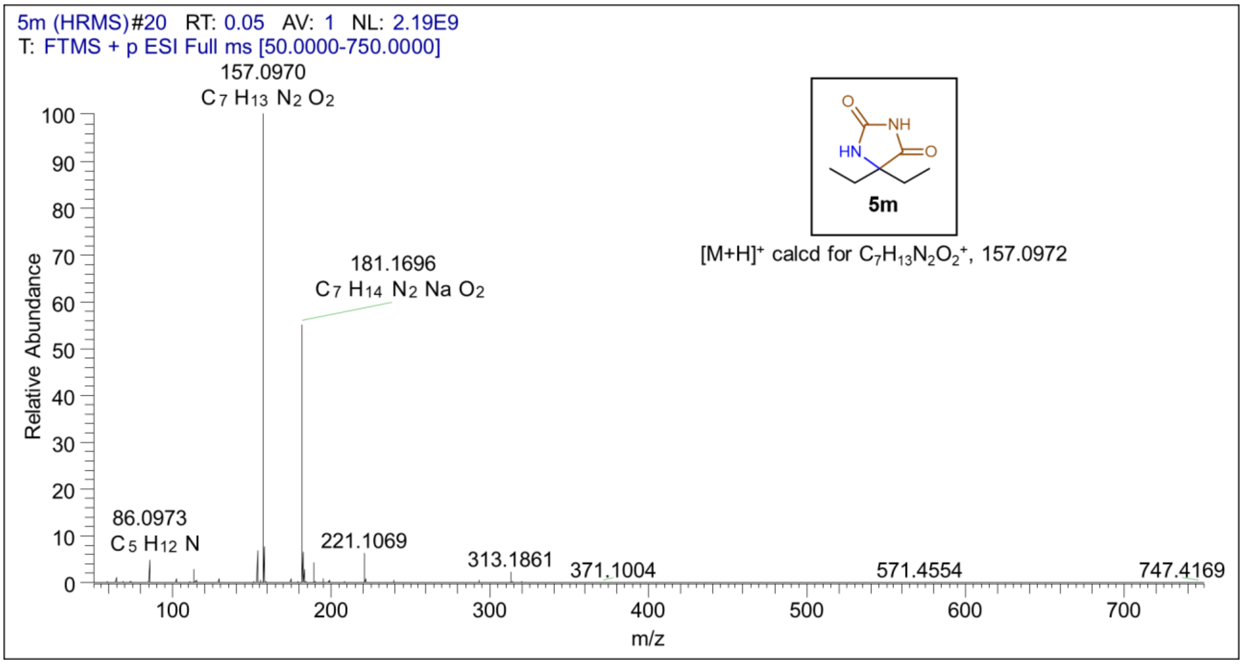

^{1}H NMR (400 MHz, DMSO-d_6) and ^{13}C NMR (100 MHz, DMSO-d_6) spectra for **5n**

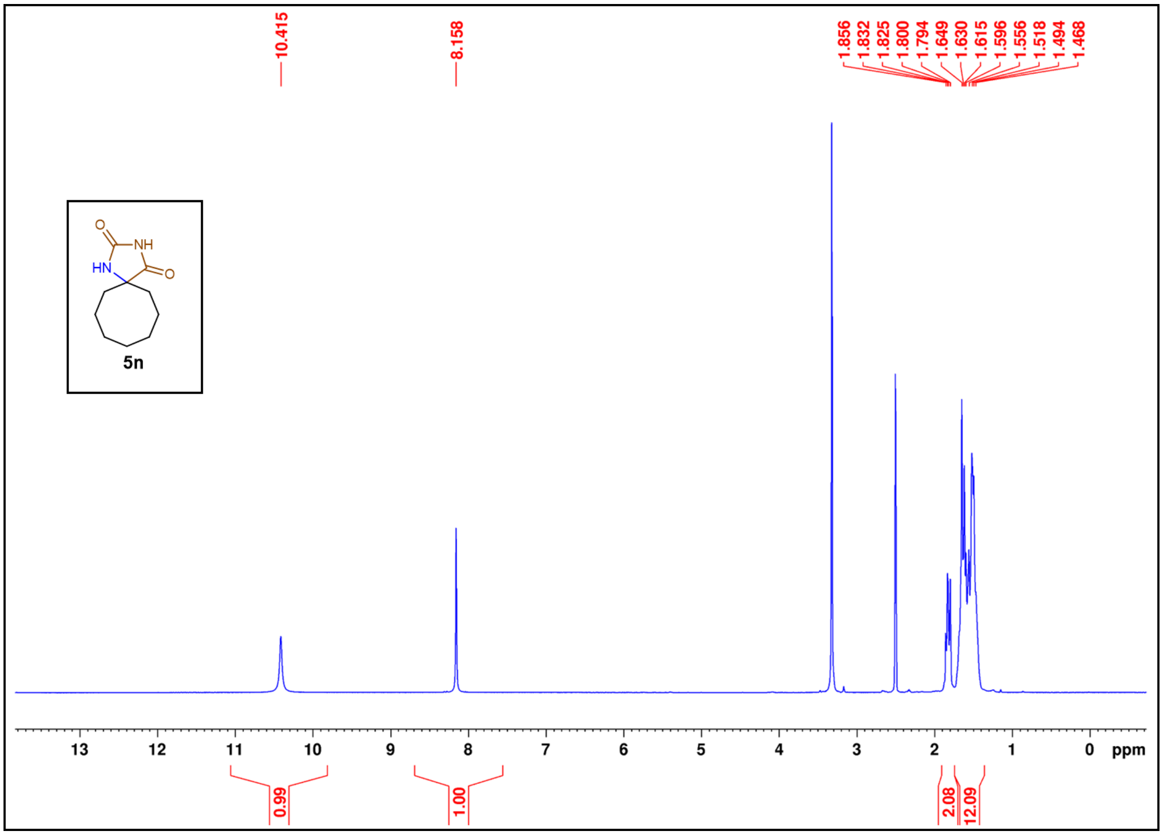

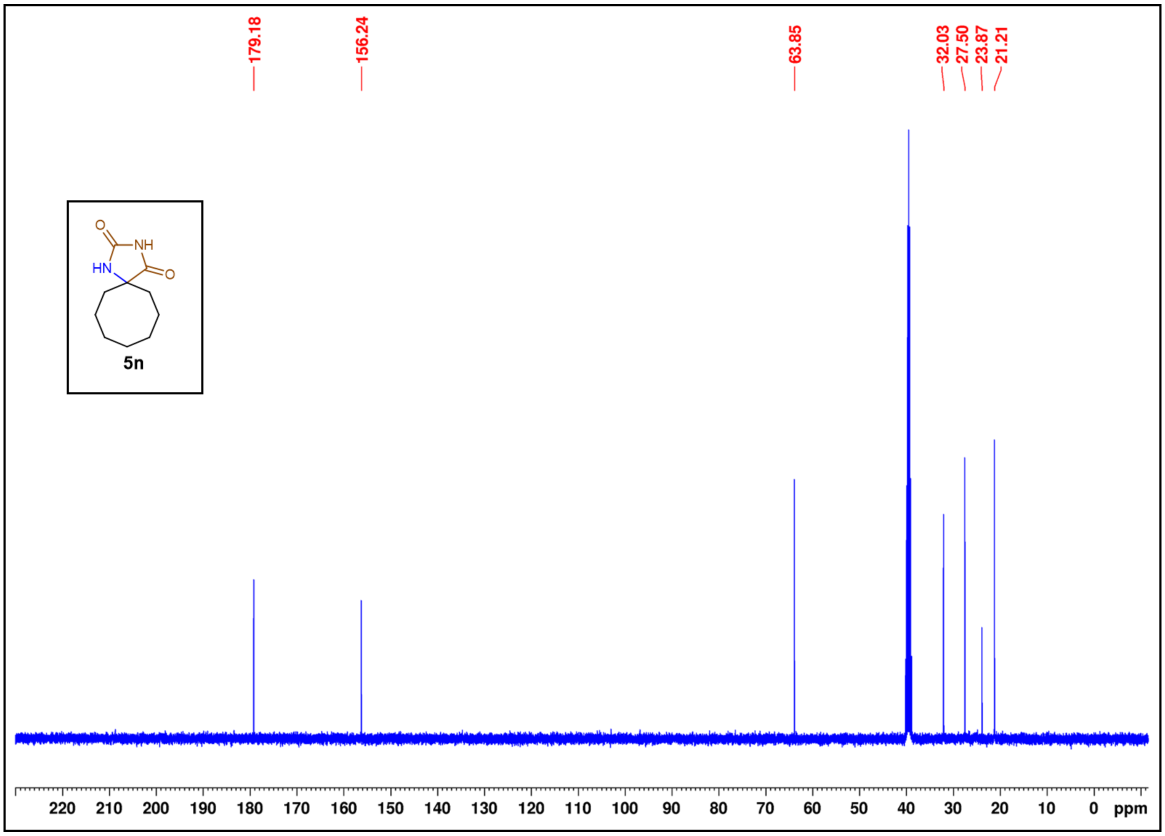

FT-IR (ATR, neat) and HRMS (ESI-positive) spectra for **5n**

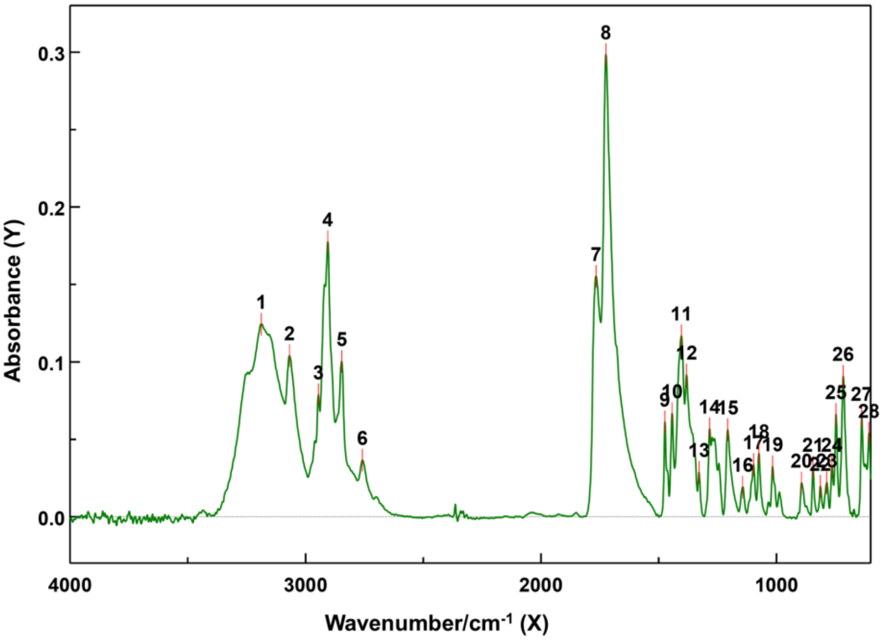

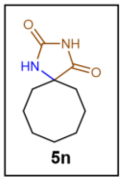

No.	X	Y	No.	X	Y	No.	X	Y
1	3187.8	0.124	11	1403.9	0.117	21	844.7	0.032
2	3068.2	0.104	12	1381.7	0.091	22	813.8	0.02
3	2945.7	0.079	13	1328.7	0.029	23	786.8	0.022
4	2905.2	0.177	14	1284.4	0.057	24	764.6	0.032
5	2846.4	0.1	15	1207.2	0.056	25	747.3	0.066
6	2758.7	0.036	16	1143.6	0.019	26	716.4	0.091
7	1766.5	0.155	17	1097.3	0.034	27	638.3	0.065
8	1724	0.298	18	1075.1	0.041	28	607.5	0.054
9	1473.3	0.061	19	1016.3	0.032			
10	1443.5	0.066	20	893.8	0.022			

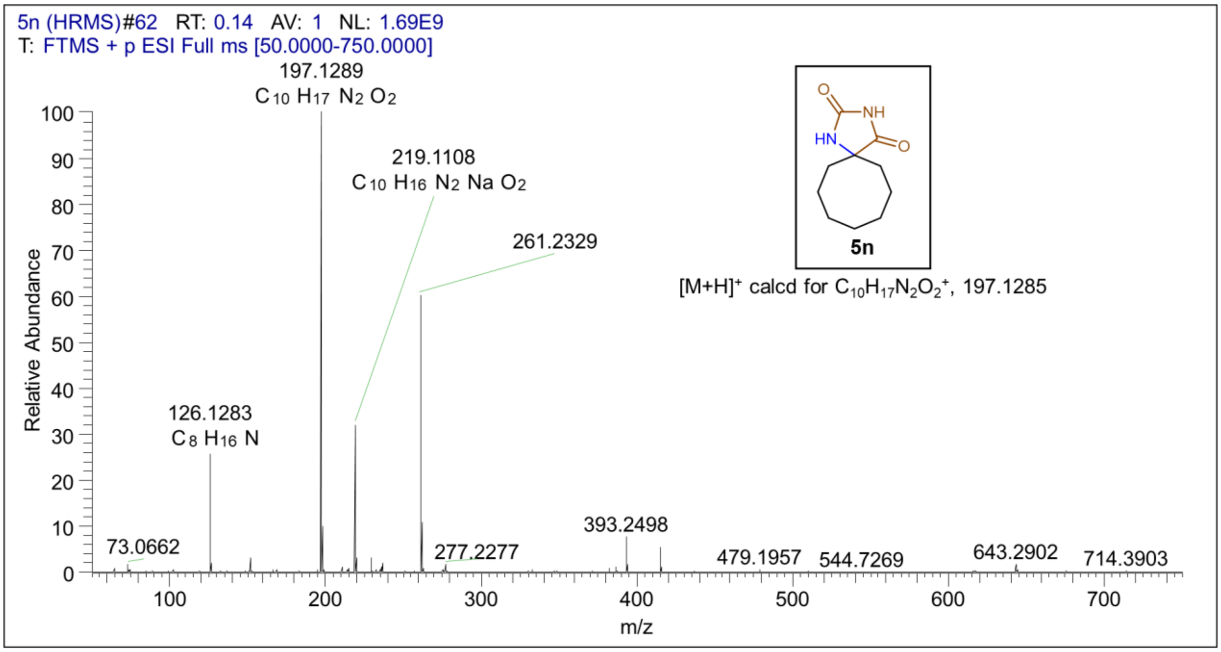

^{1}H NMR (400 MHz, DMSO-d_6) and ^{13}C NMR (100 MHz, DMSO-d_6) spectra for **5o**

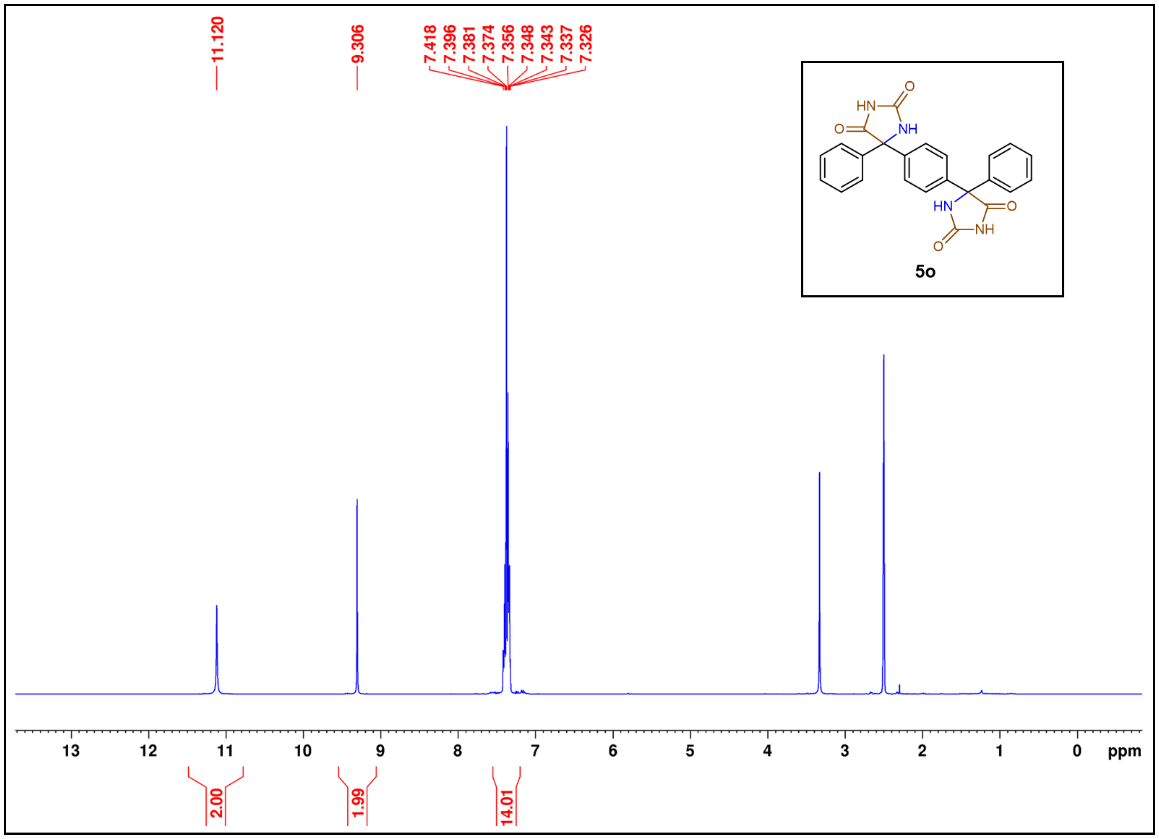

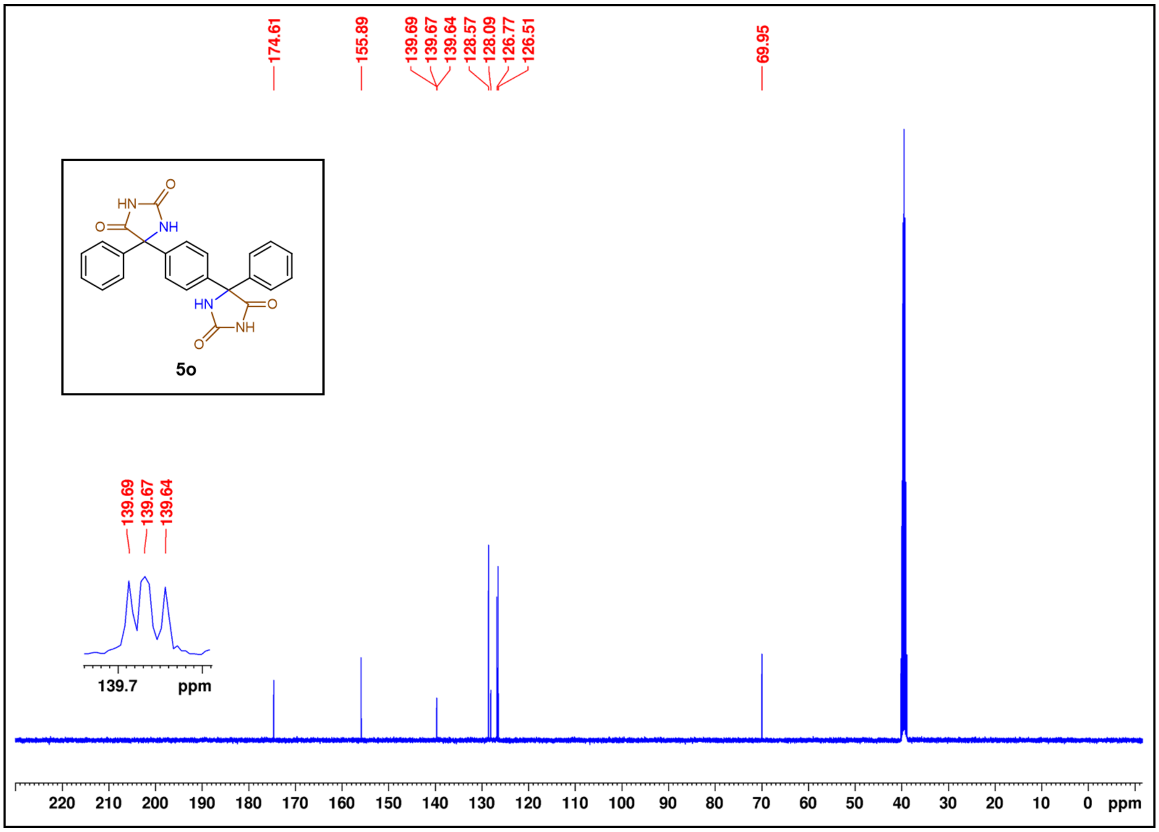

FT-IR (ATR, neat) and HRMS (ESI-negative) spectra for **5o**

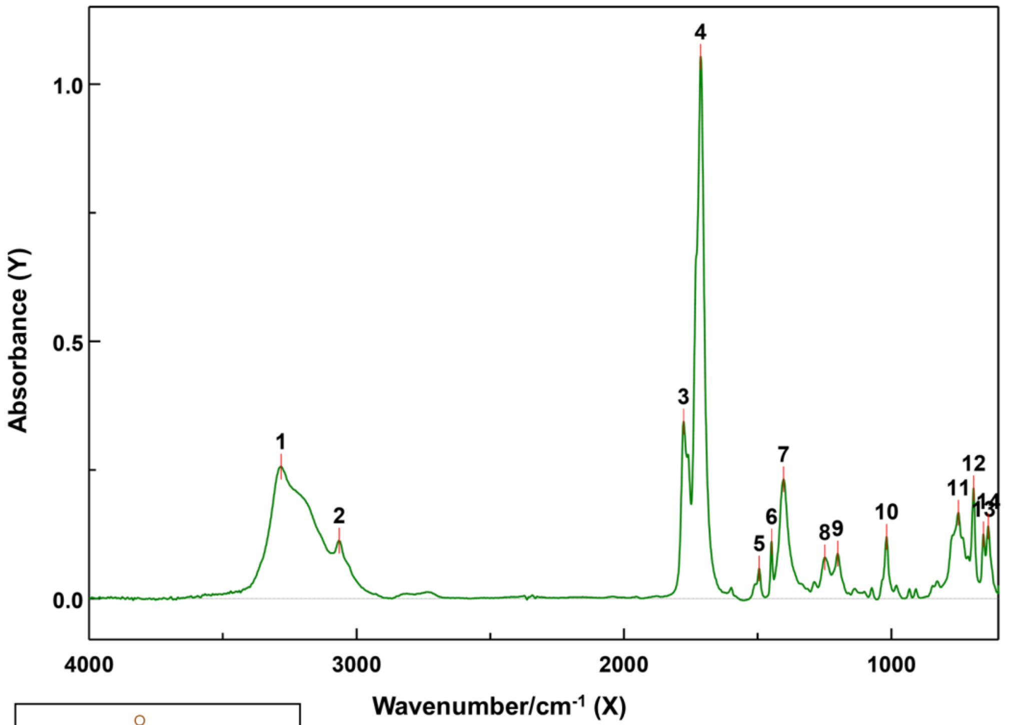

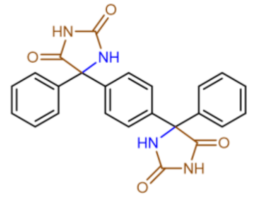

5o

No.	X	Y	No.	X	Y	No.	X	Y
1	3282.3	0.256	6	1448.3	0.111	11	749.2	0.167
2	3064.3	0.112	7	1403	0.232	12	692.3	0.214
3	1777.1	0.344	8	1248.7	0.08	13	655.7	0.125
4	1713.4	1.053	9	1200.5	0.087	14	637.4	0.14
5	1494.6	0.058	10	1018.2	0.12			

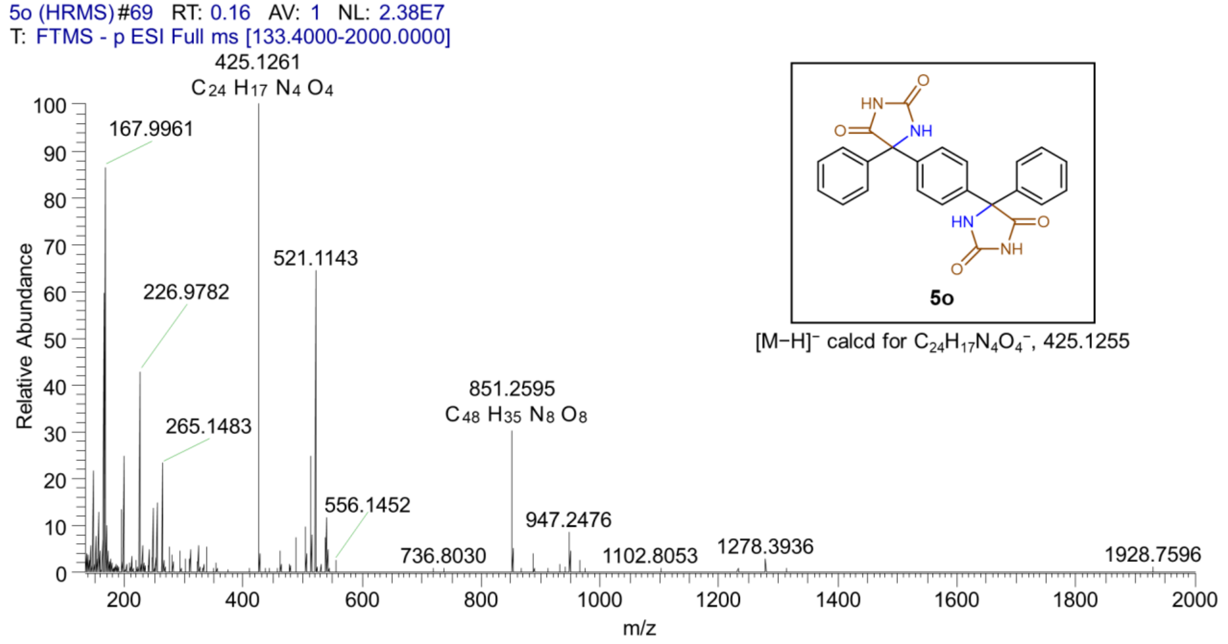

[M−H]⁻ calcd for $C_{24}H_{17}N_4O_4^-$, 425.1255

^{1}H NMR (400 MHz, DMSO-d_6) and ^{13}C NMR (100 MHz, DMSO-d_6) spectra for **5p**

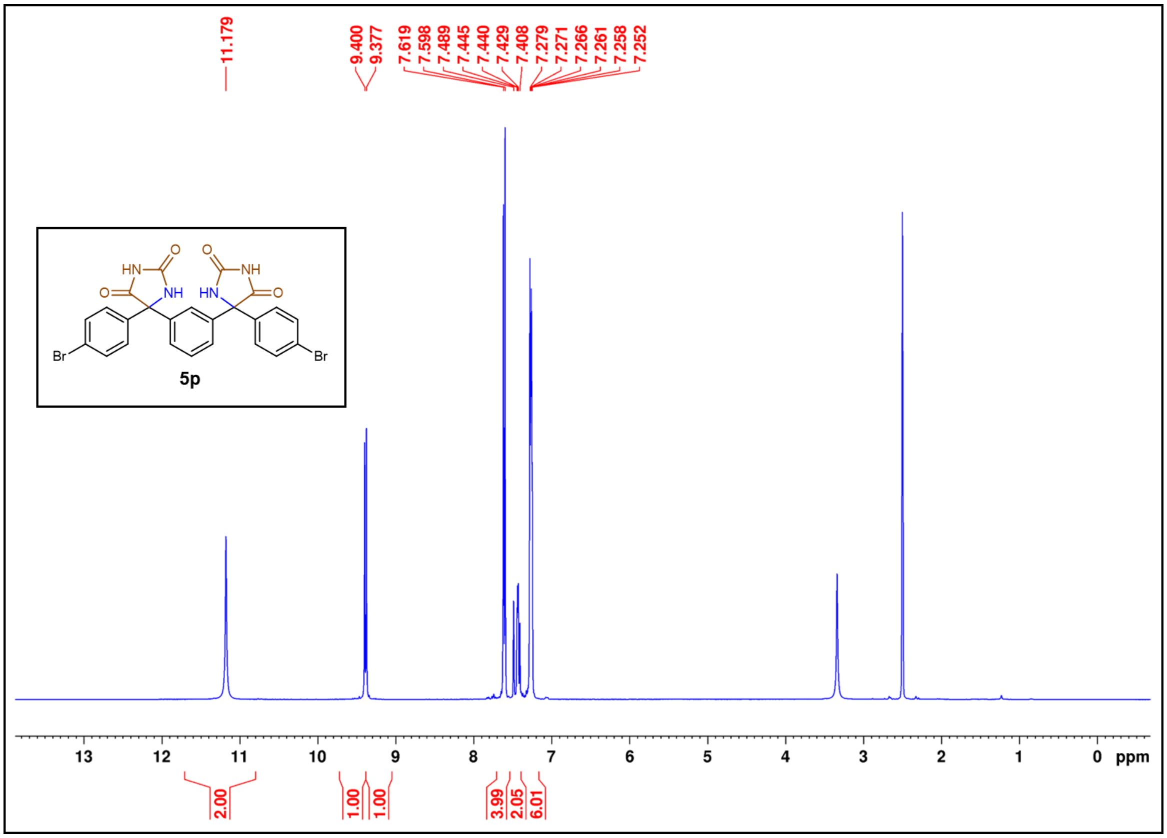

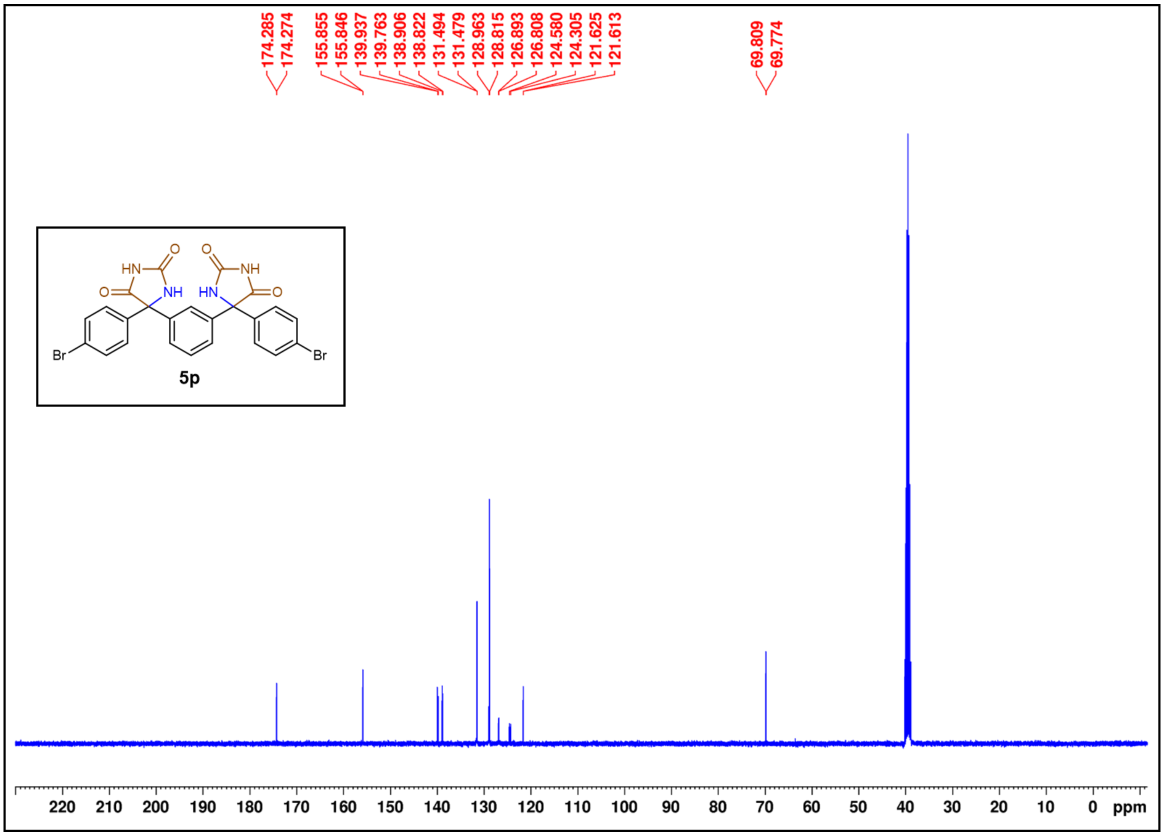

FT-IR (ATR, neat) and HRMS (ESI-negative) spectra for **5p**

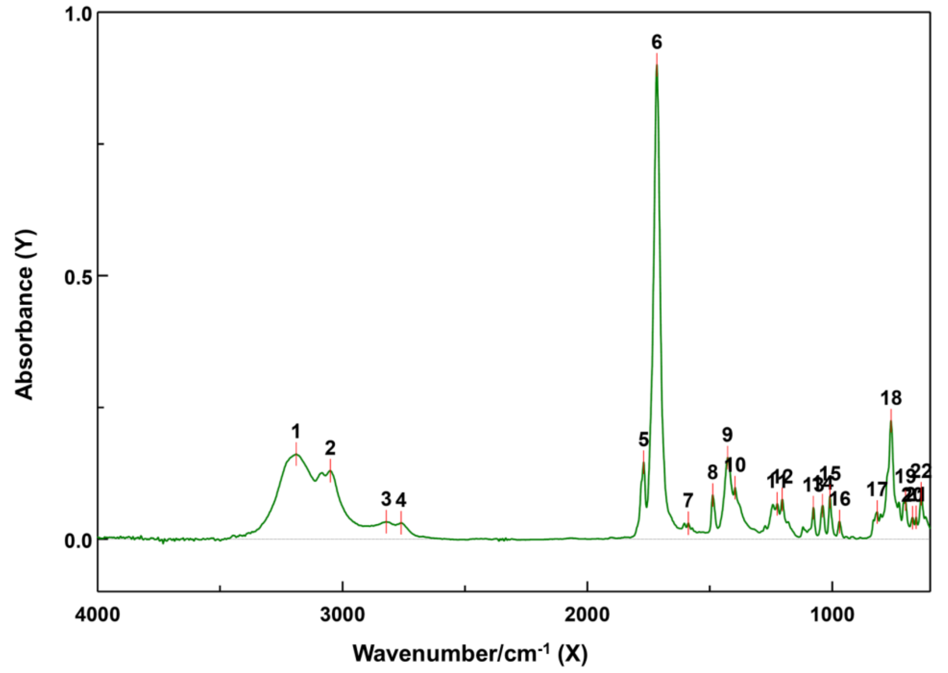

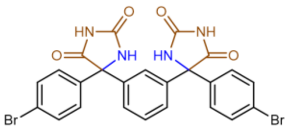

5p

No.	X	Y	No.	X	Y	No.	X	Y
1	3188.7	0.161	9	1427.1	0.154	17	816.7	0.051
2	3049.9	0.129	10	1397.2	0.097	18	759.8	0.224
3	2821.3	0.032	11	1224.6	0.066	19	701	0.075
4	2760.6	0.03	12	1204.3	0.075	20	672.1	0.04
5	1770.3	0.146	13	1077	0.059	21	657.6	0.041
6	1716.3	0.9	14	1039.4	0.063	22	636.4	0.085
7	1588.1	0.03	15	1009.6	0.08			
8	1487.8	0.083	16	970	0.033			

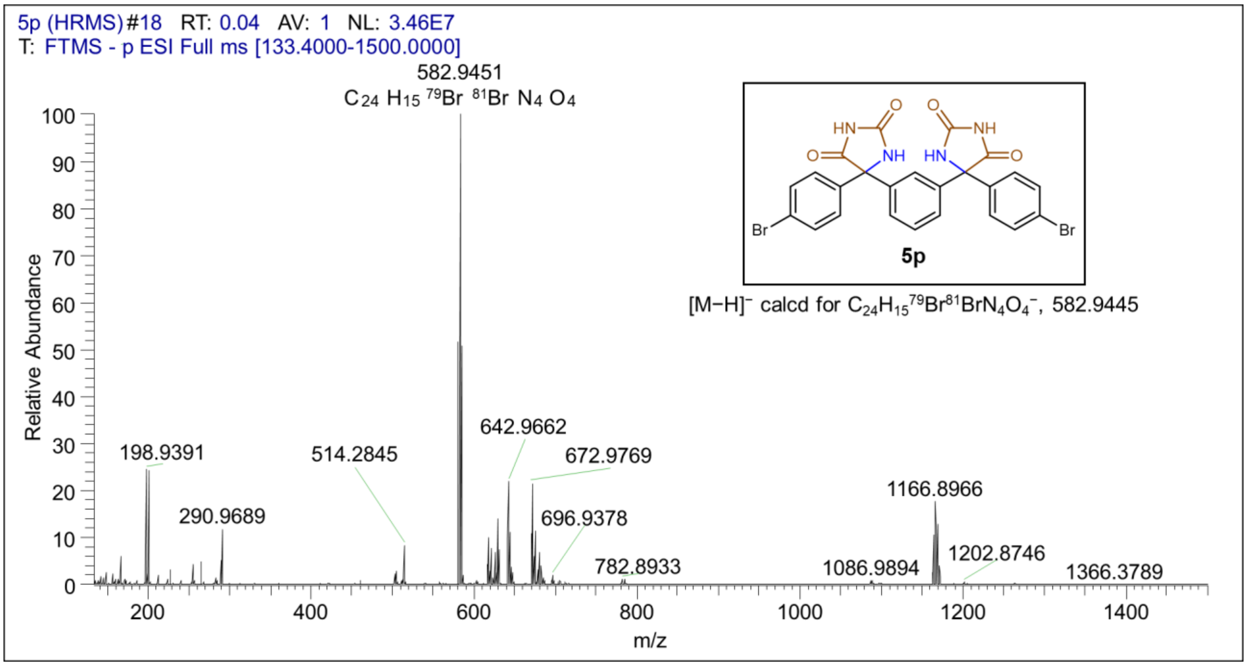

^{1}H NMR (400 MHz, DMSO-d_6) and ^{13}C NMR (100 MHz, DMSO-d_6) spectra for **5q**

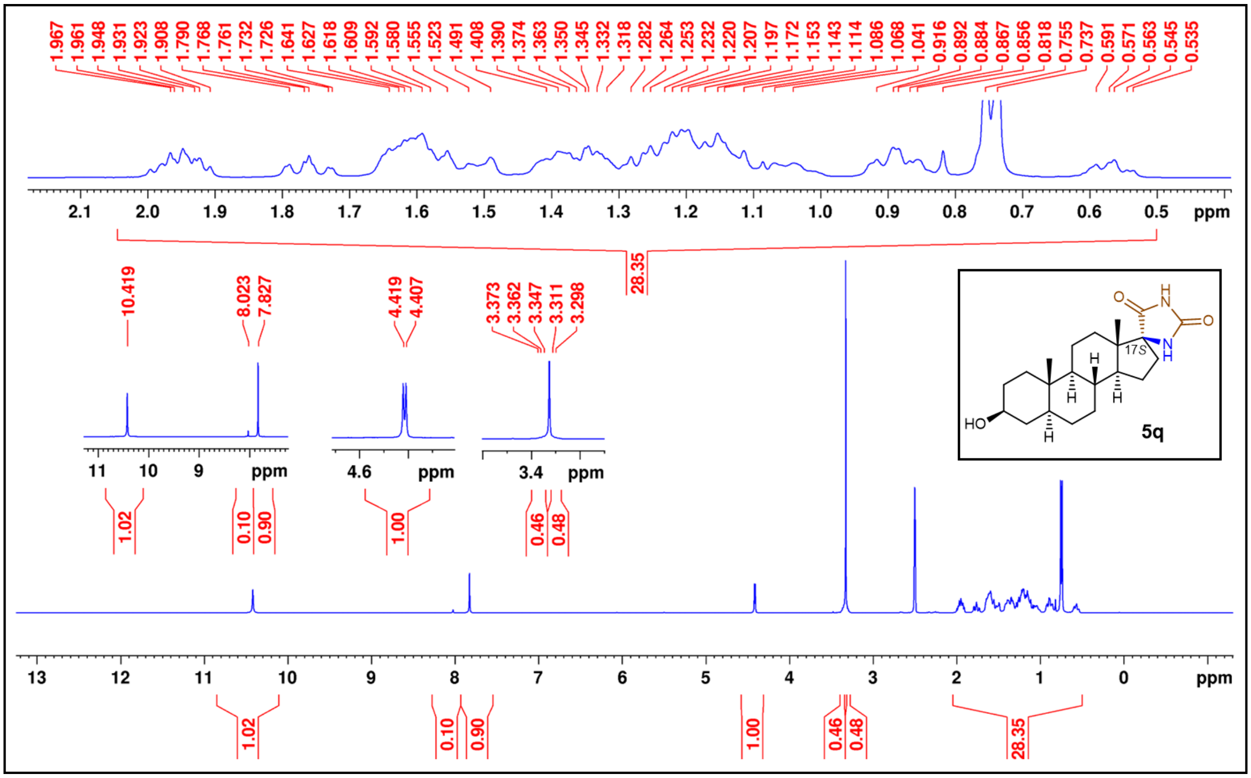

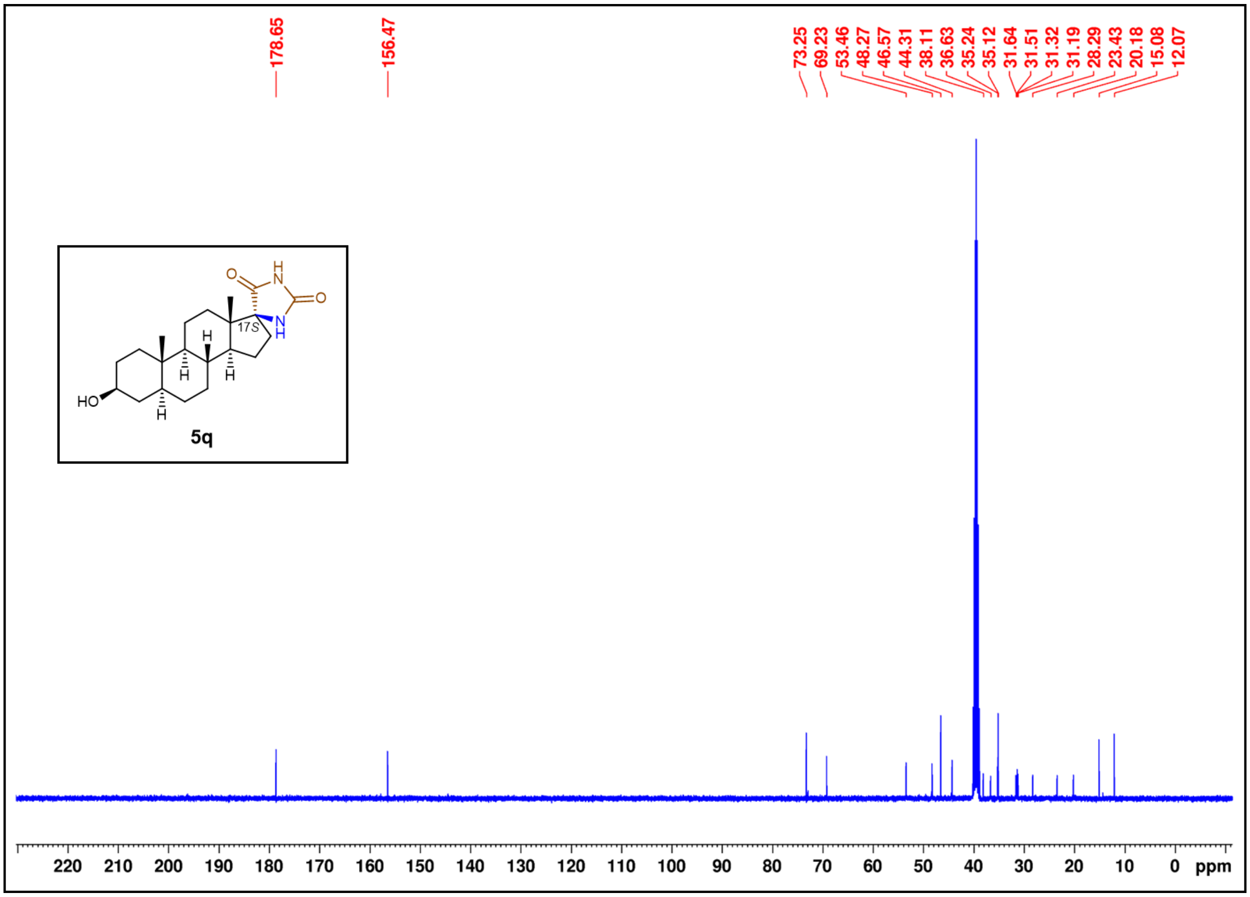

ROESY (400 MHz, DMSO-d_6) spectrum for **5q**

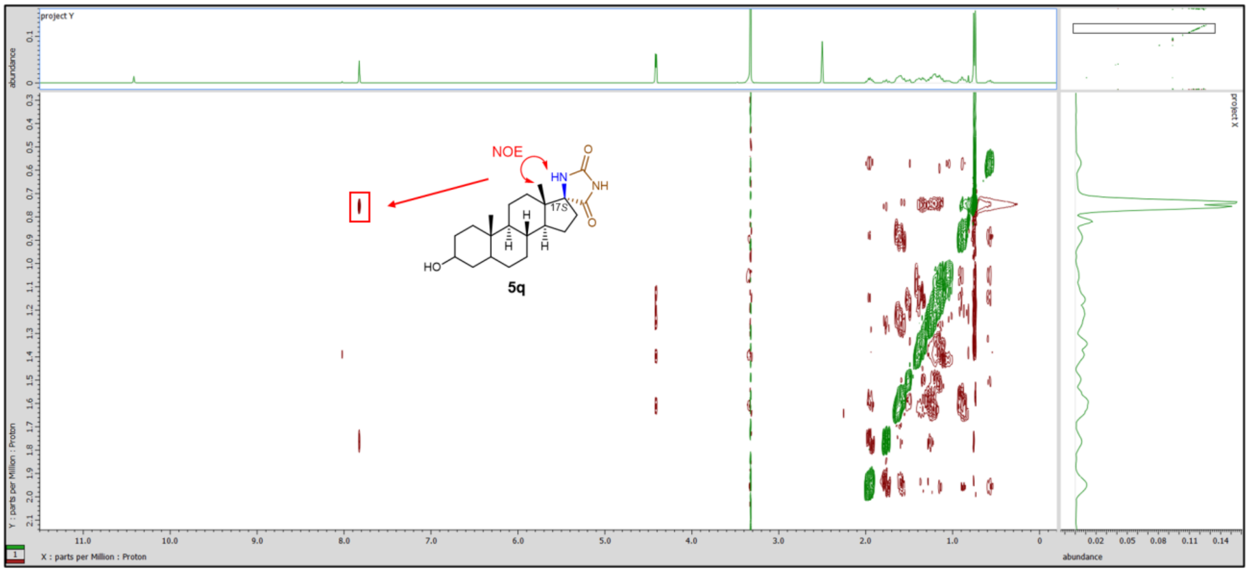

FT-IR (ATR, neat) and HRMS (ESI-negative) spectra for **5q**

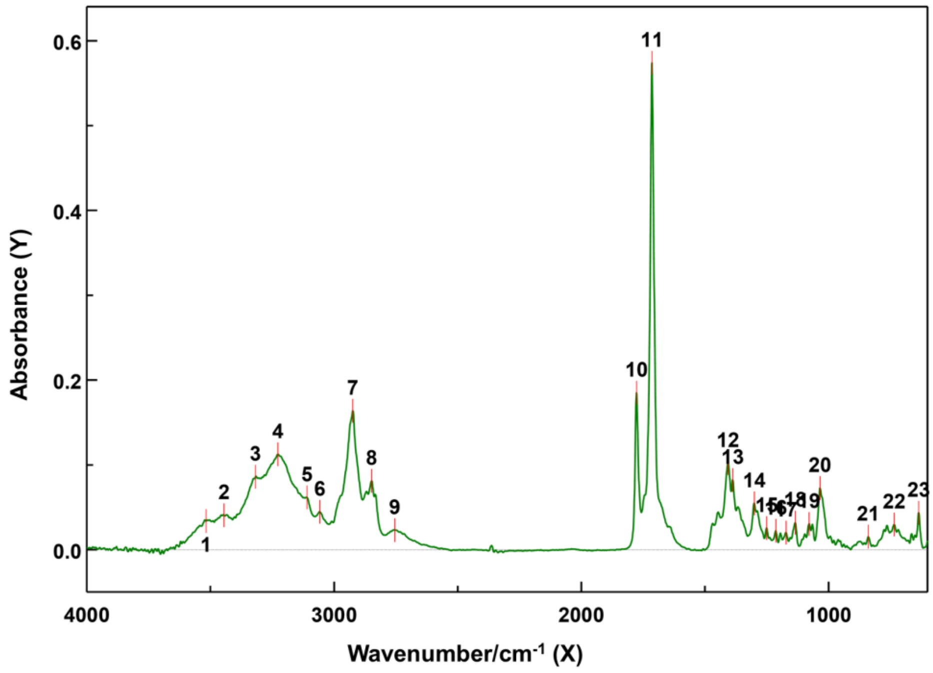

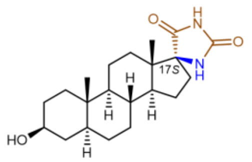

5q

No.	X	Y	No.	X	Y	No.	X	Y
1	3517.5	0.034	9	2754.8	0.023	17	1173	0.02
2	3445.2	0.04	10	1777.1	0.185	18	1135	0.032
3	3317.9	0.086	11	1714.4	0.574	19	1079	0.03
4	3227.3	0.112	12	1406.8	0.102	20	1035	0.072
5	3109.7	0.062	13	1387.5	0.082	21	839.8	0.015
6	3057.6	0.044	14	1300.8	0.054	22	734.7	0.029
7	2924.5	0.164	15	1250.6	0.025	23	635.4	0.043
8	2848.3	0.081	16	1214	0.022			

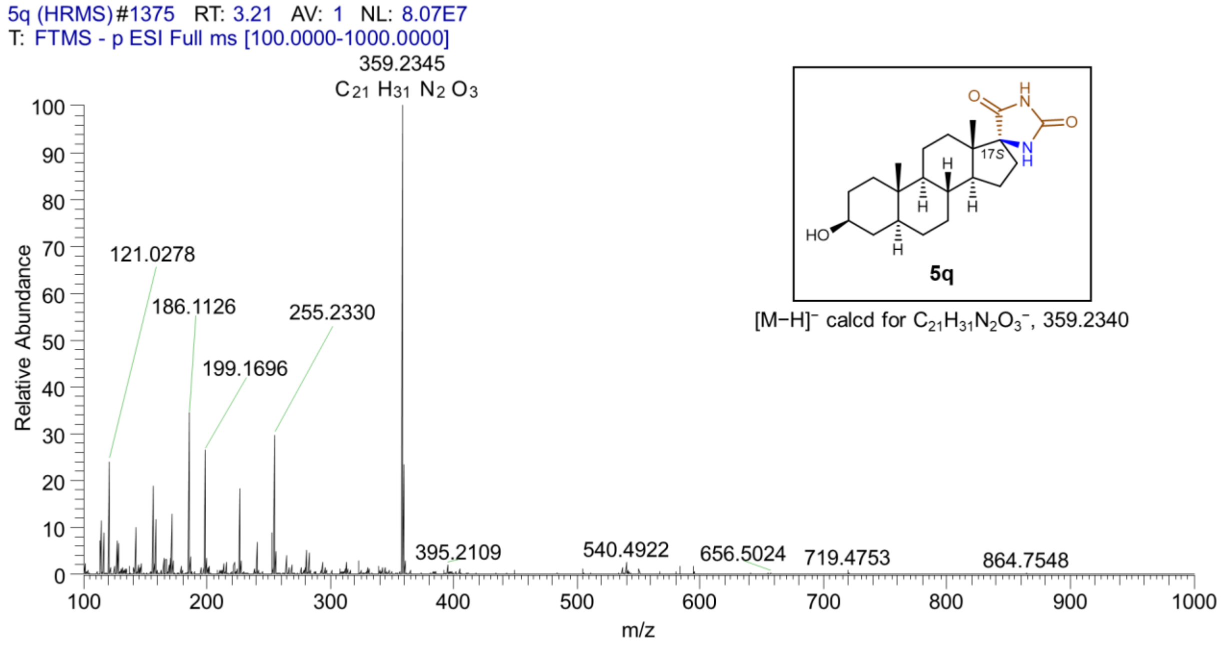

5q

[M−H]⁻ calcd for $C_{21}H_{31}N_2O_3^-$, 359.2340

^{1}H NMR (400 MHz, DMSO-d_6) and ^{13}C NMR (100 MHz, DMSO-d_6) spectra for **5r**

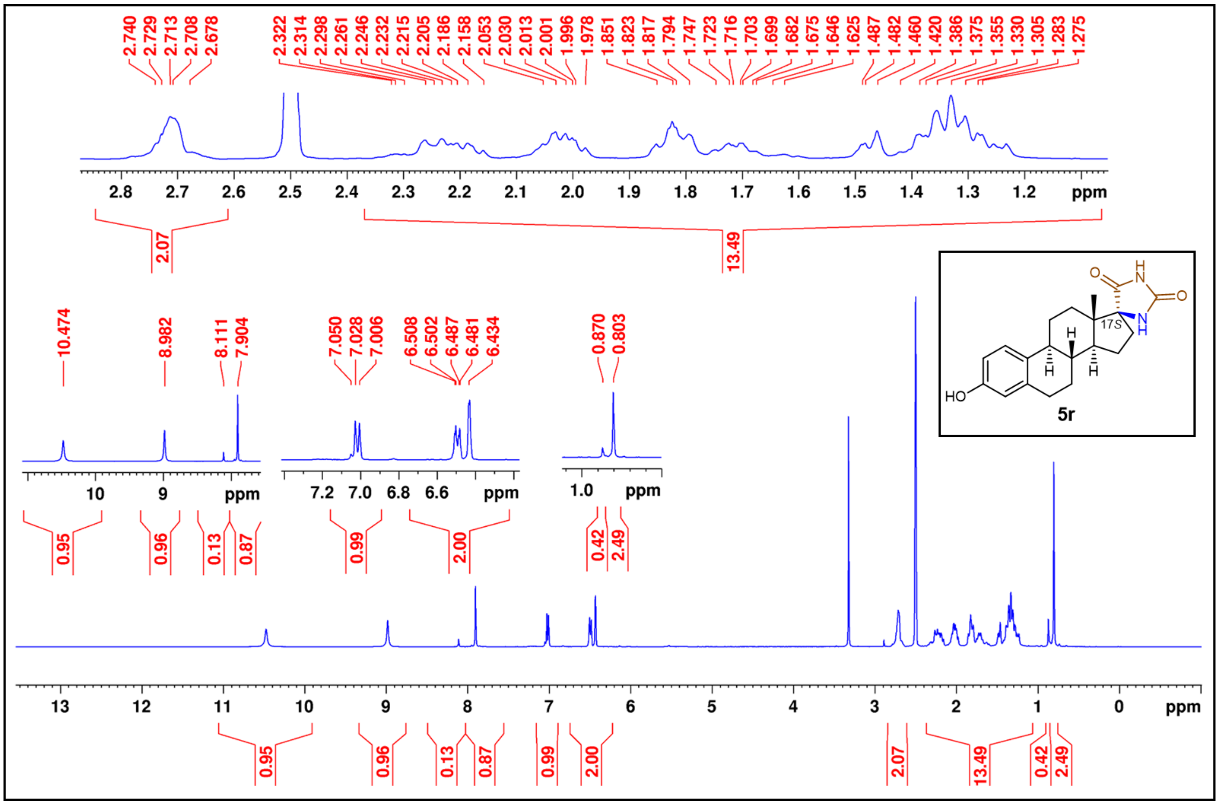

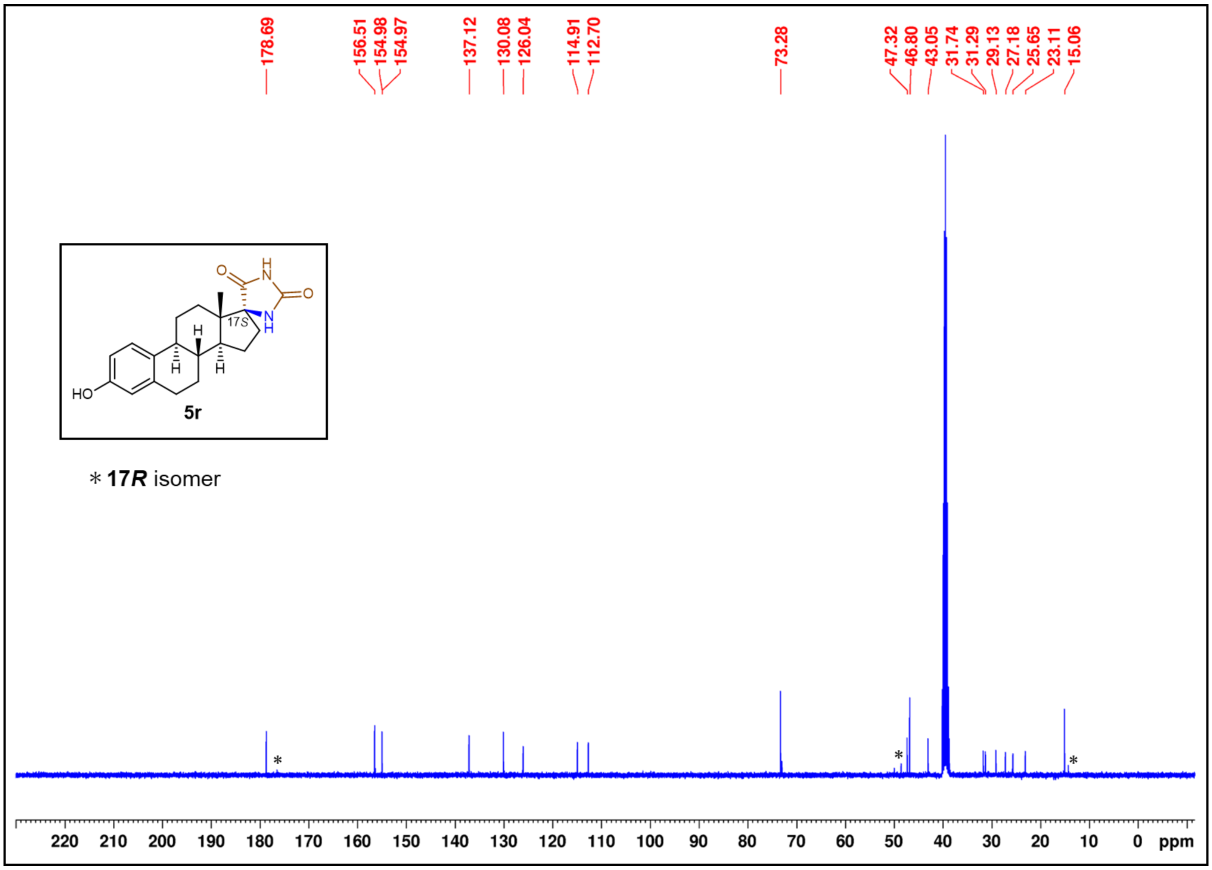

ROESY (400 MHz, DMSO-d_6) spectrum for **5r**

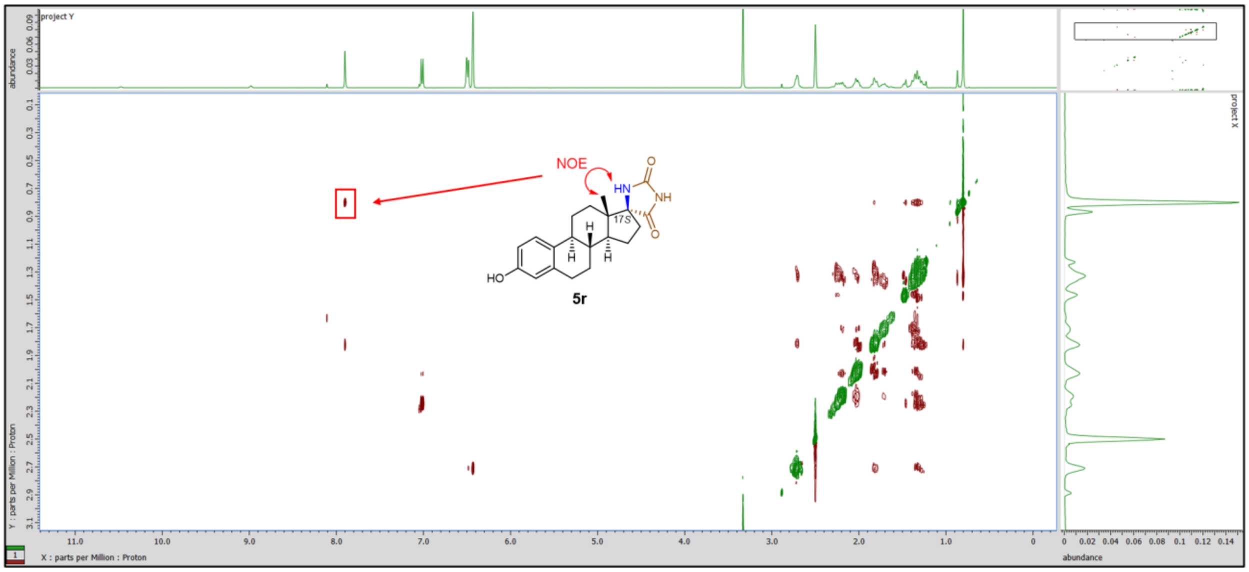

FT-IR (ATR, neat) and HRMS (ESI-negative) spectra for **5r**

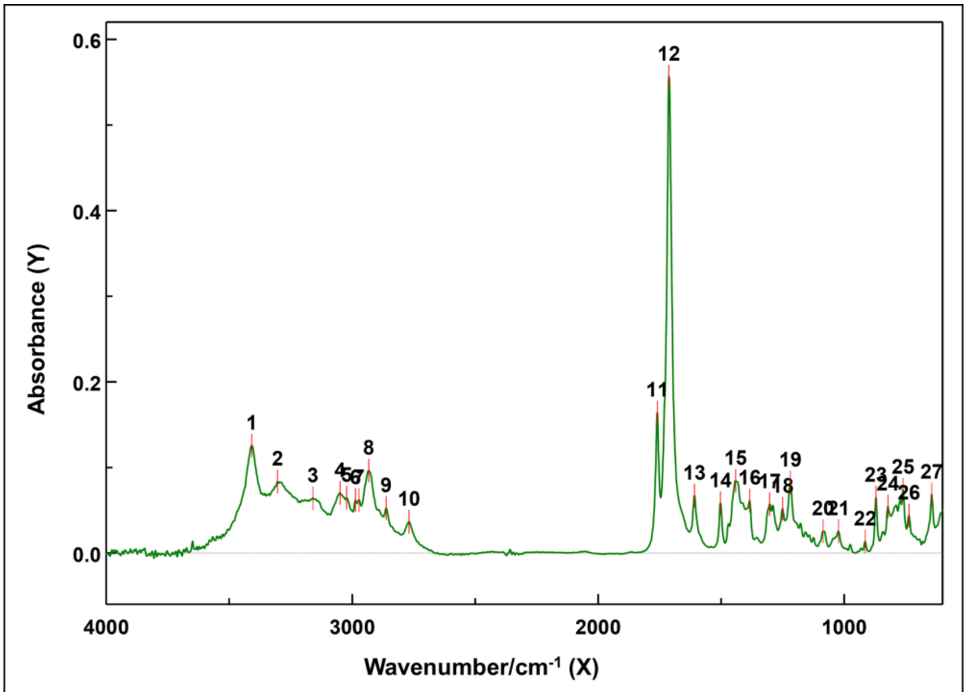

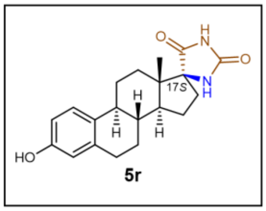

No.	X	Y	No.	X	Y	No.	X	Y
1	3408.6	0.125	10	2769.3	0.036	19	1219	0.082
2	3303.5	0.083	11	1759.7	0.164	20	1086	0.025
3	3159.8	0.064	12	1712.5	0.556	21	1023	0.025
4	3049.9	0.07	13	1608.3	0.067	22	915.1	0.013
5	3022.9	0.064	14	1503.2	0.058	23	870.7	0.065
6	2987.2	0.062	15	1441.5	0.084	24	822.5	0.054
7	2971.8	0.062	16	1384.6	0.061	25	760.8	0.074
8	2933.2	0.096	17	1302.7	0.057	26	736.7	0.044
9	2861.8	0.052	18	1250.6	0.052	27	644.1	0.068

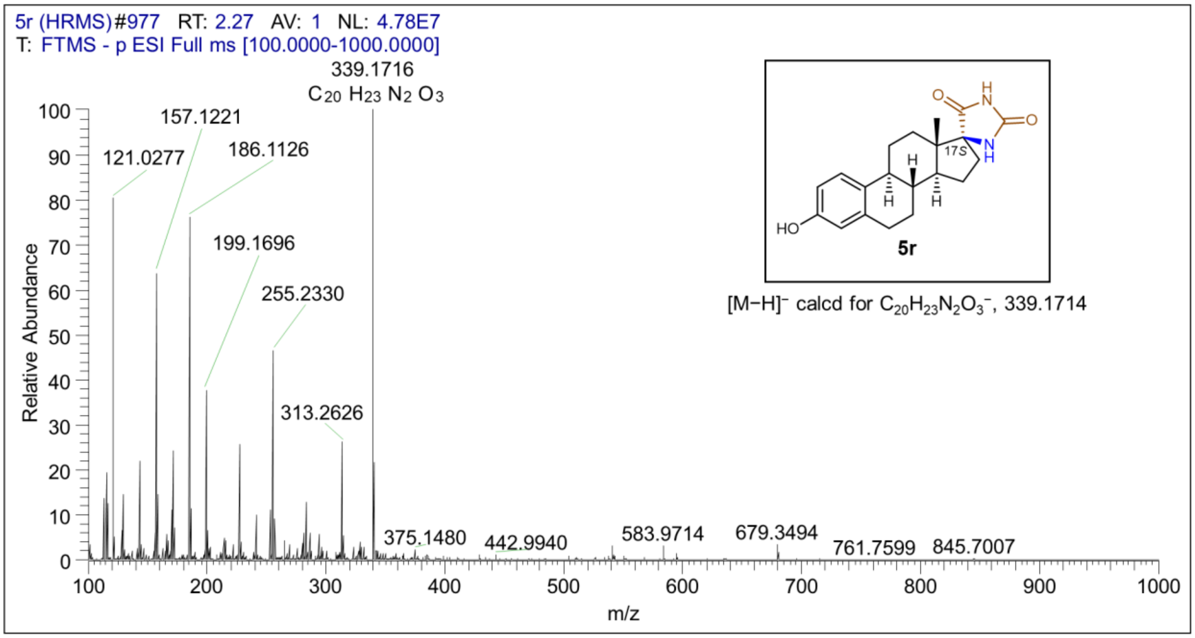

^{1}H NMR (400 MHz, DMSO-d_6) and ^{13}C NMR (100 MHz, DMSO-d_6) spectra for **5s**

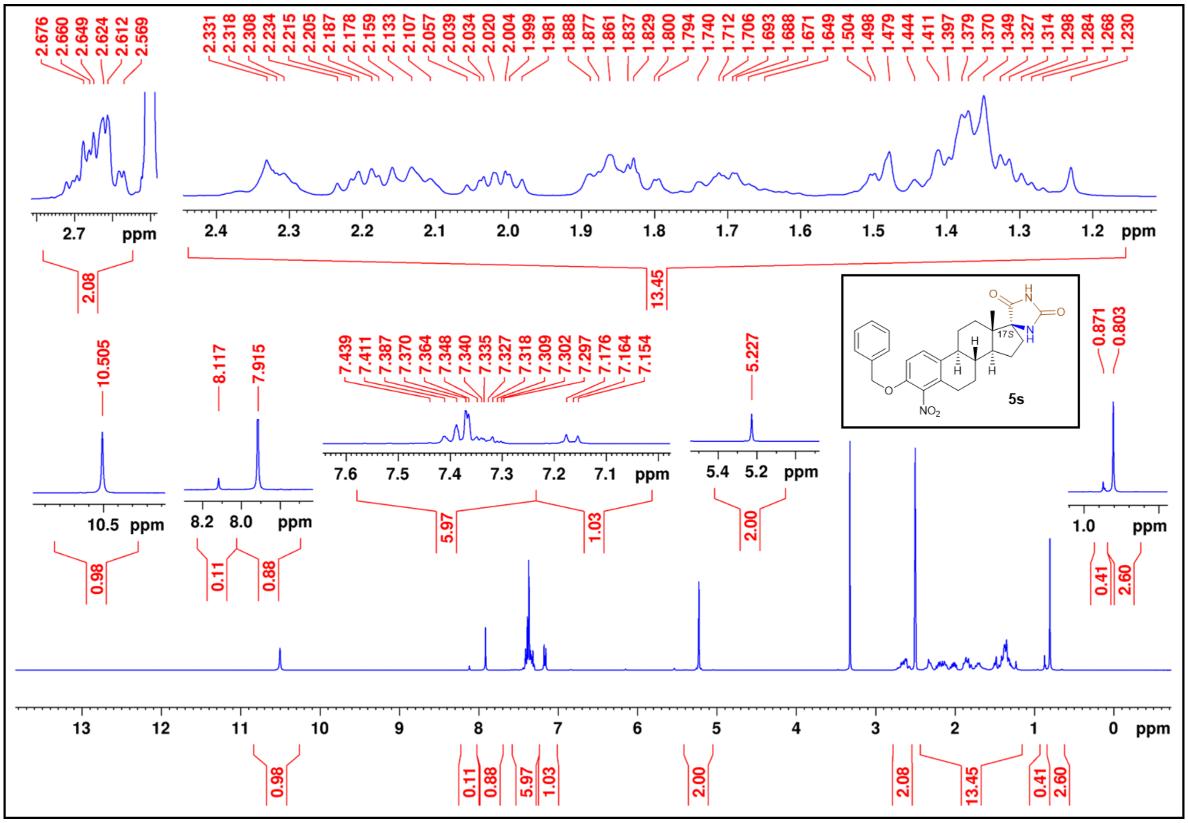

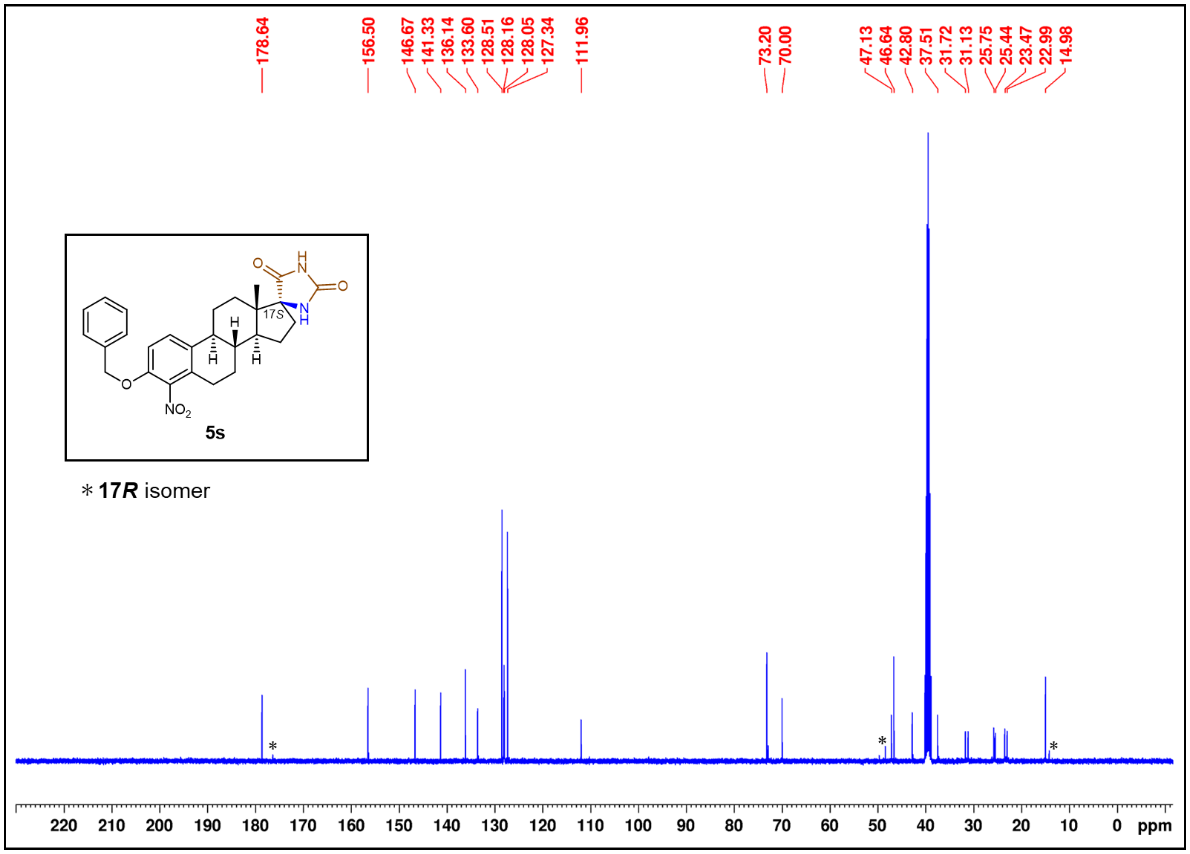

ROESY (400 MHz, DMSO-d_6) spectrum for **5s**

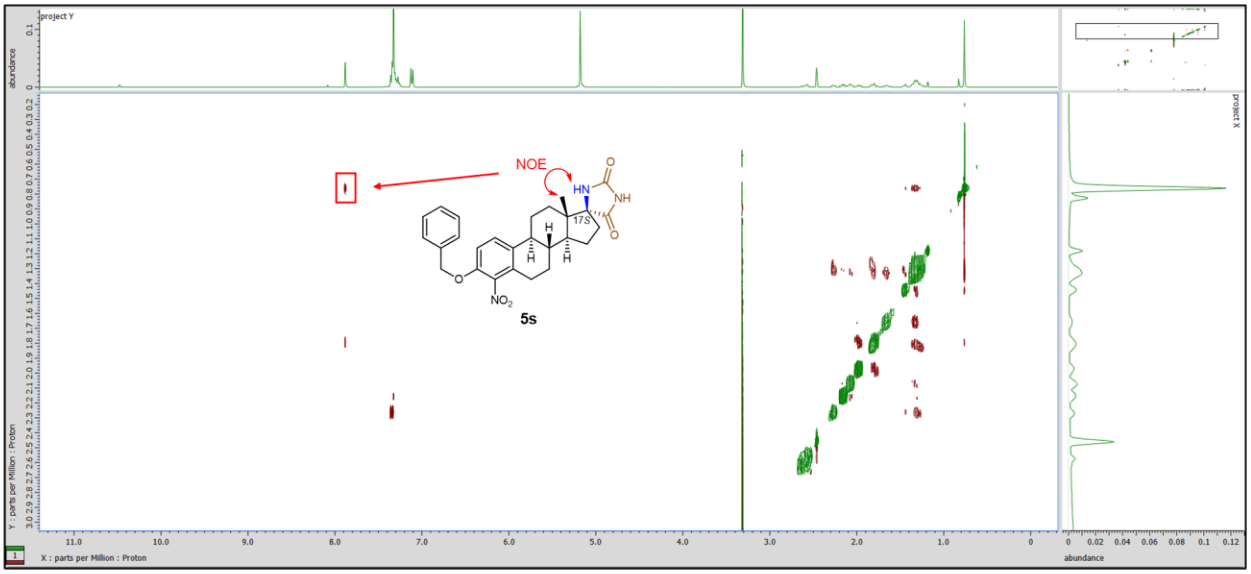

FT-IR (ATR, neat) and HRMS (ESI-negative) spectra for **5s**

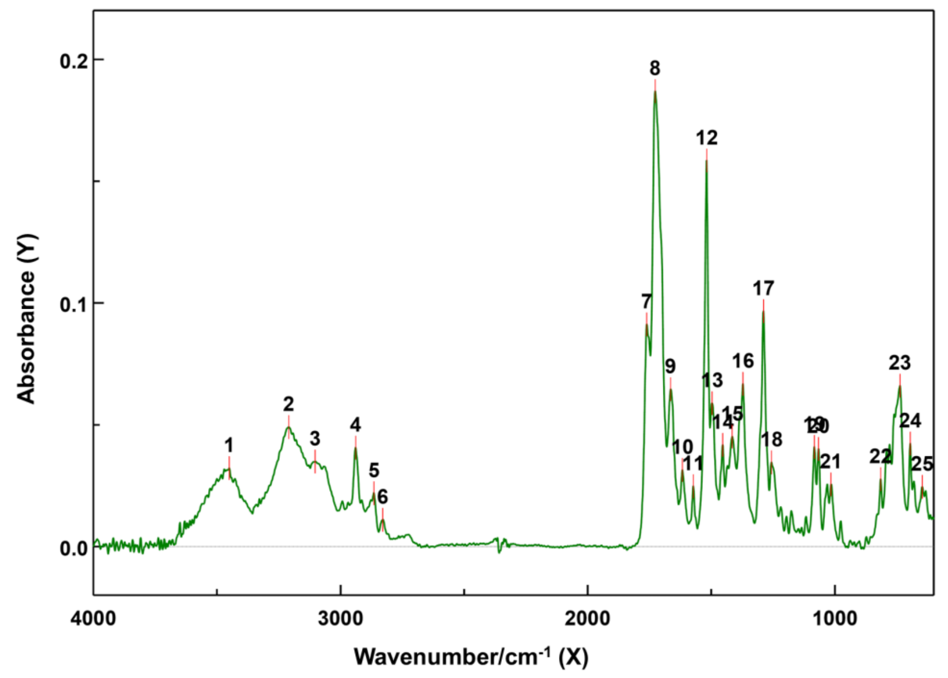

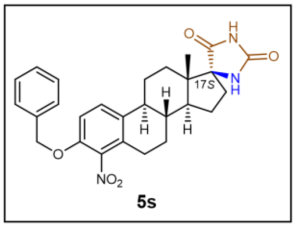

No.	X	Y	No.	X	Y	No.	X	Y
1	3450	0.032	10	1617	0.031	19	1083	0.041
2	3209.9	0.049	11	1572.7	0.025	20	1066	0.04
3	3102.9	0.035	12	1518.7	0.158	21	1015	0.025
4	2939	0.041	13	1497.5	0.059	22	814.8	0.028
5	2864.7	0.022	14	1454.1	0.042	23	736.7	0.066
6	2830	0.011	15	1415.5	0.045	24	695.2	0.042
7	1760.7	0.091	16	1372.1	0.067	25	647	0.024
8	1726.9	0.187	17	1289.2	0.097			
9	1664.3	0.065	18	1256.4	0.035			

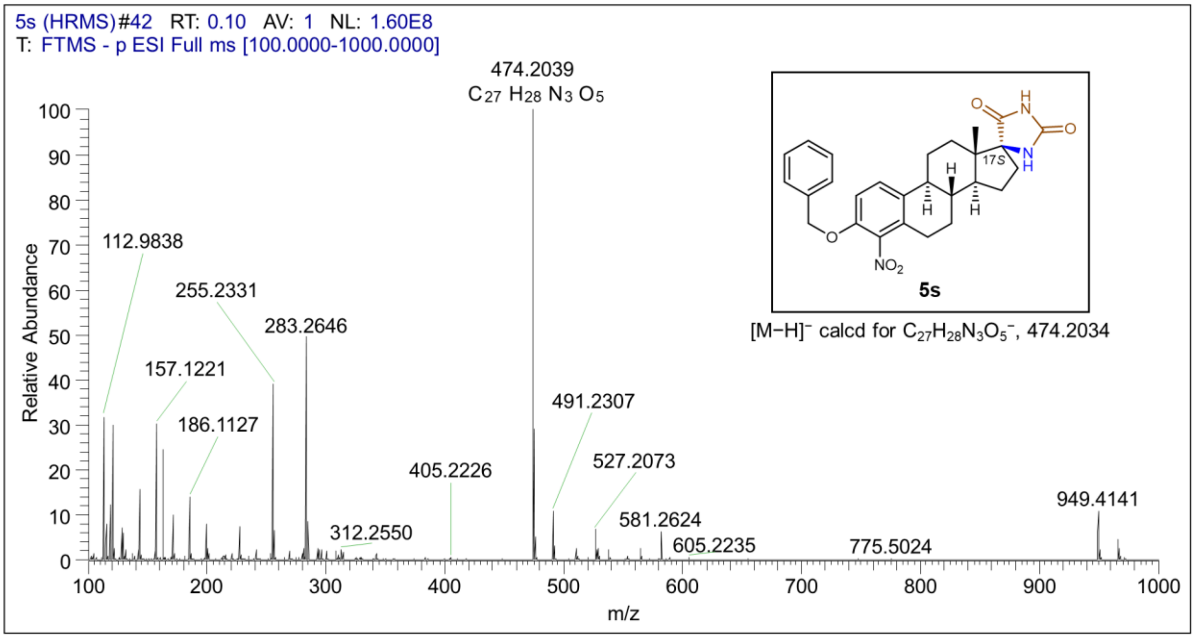

^{1}H NMR (400 MHz, DMSO-d_6) and ^{13}C NMR (100 MHz, DMSO-d_6) spectra for **5t**

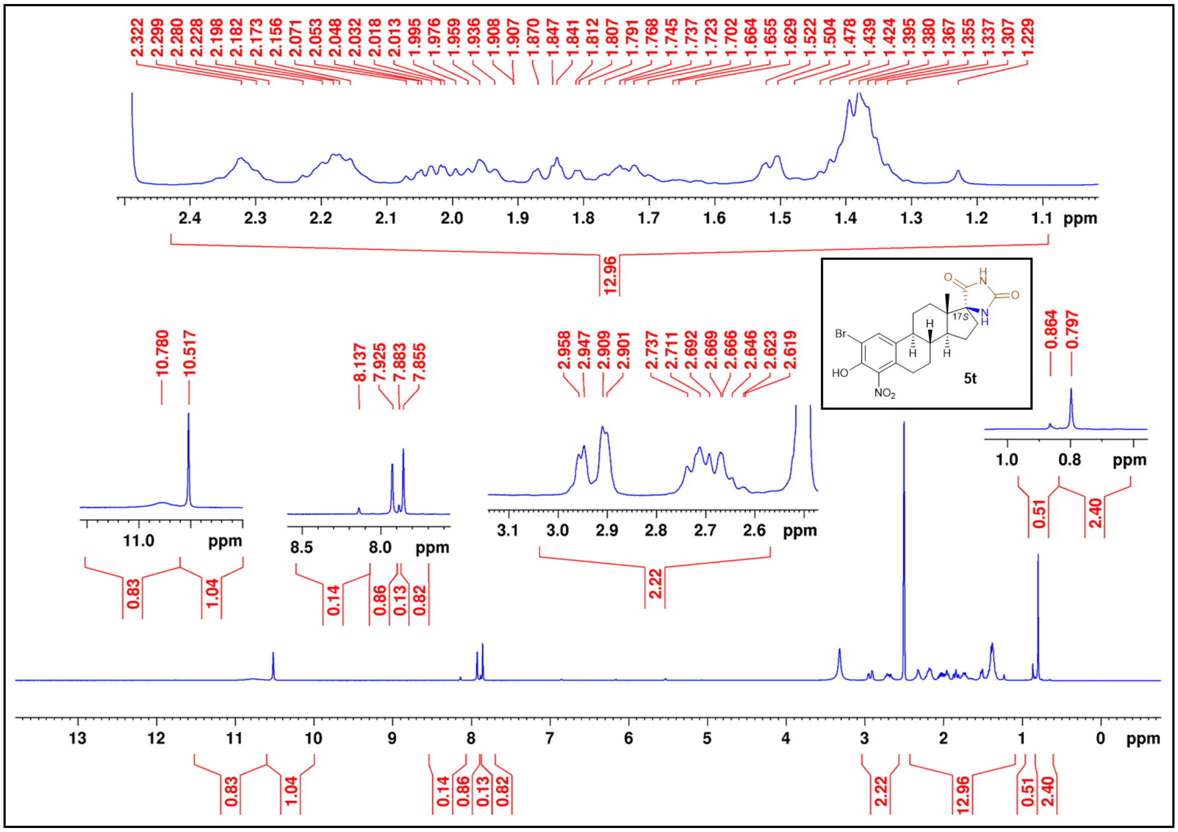

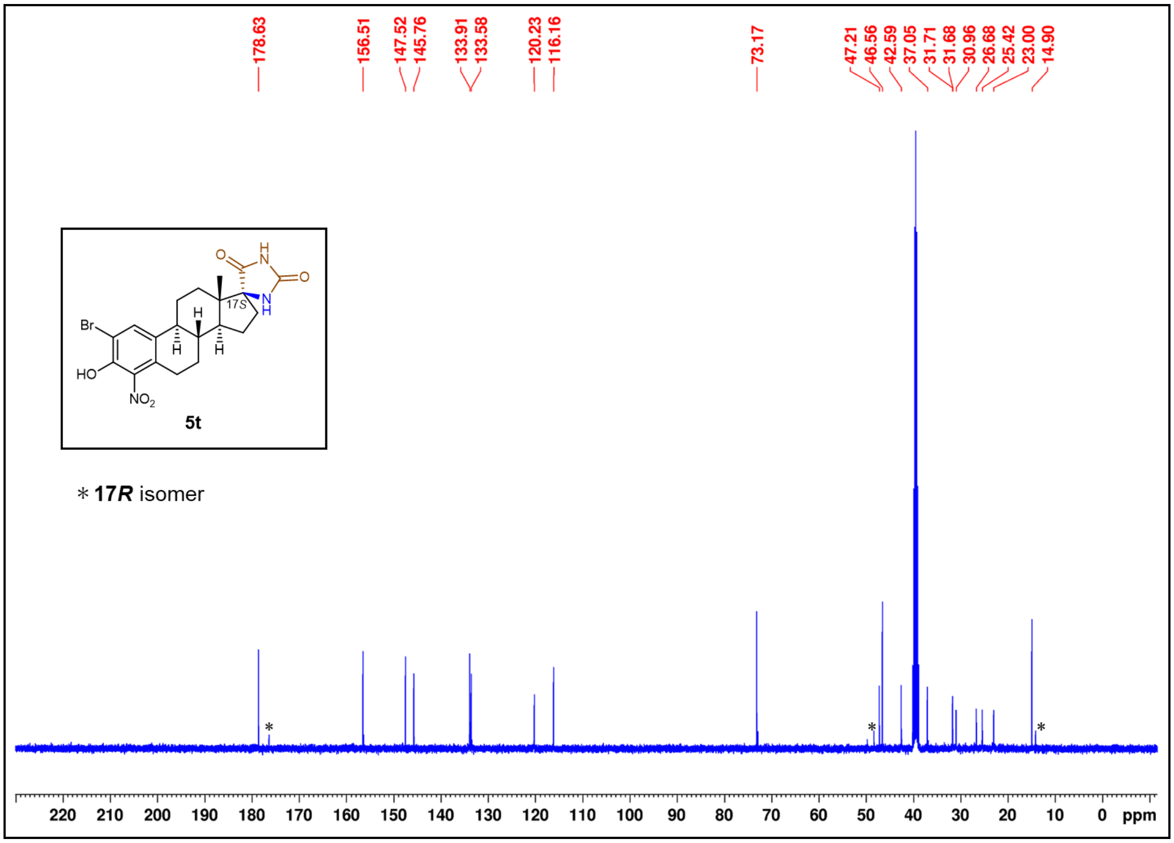

ROESY (400 MHz, DMSO-d_6) spectrum for **5t**

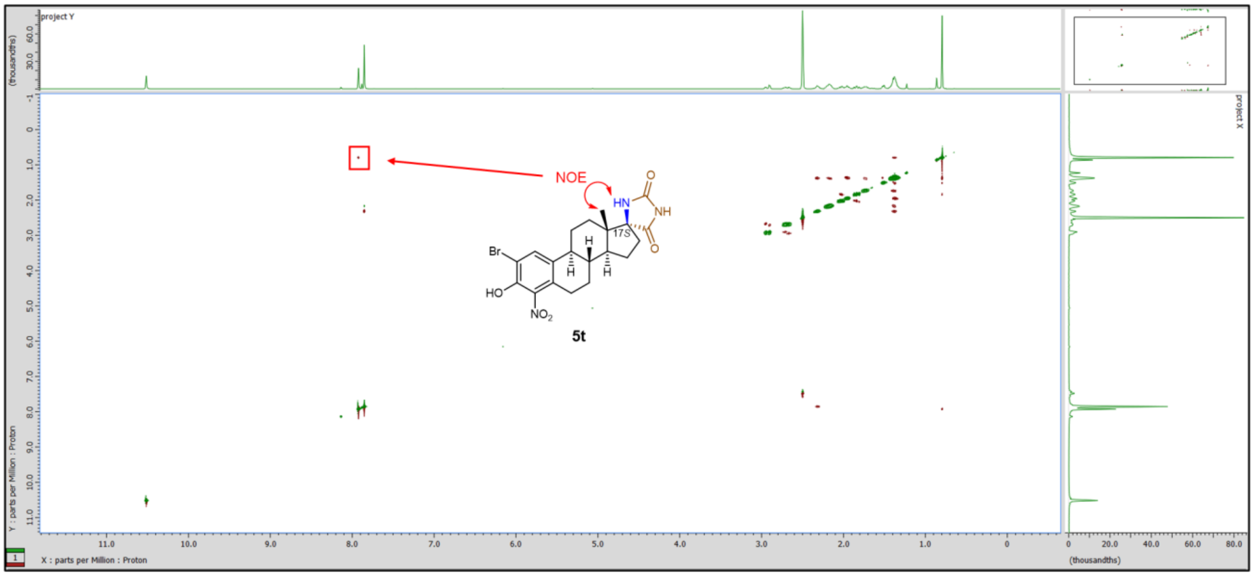

FT-IR (ATR, neat) and HRMS (ESI-negative) spectra for **5t**

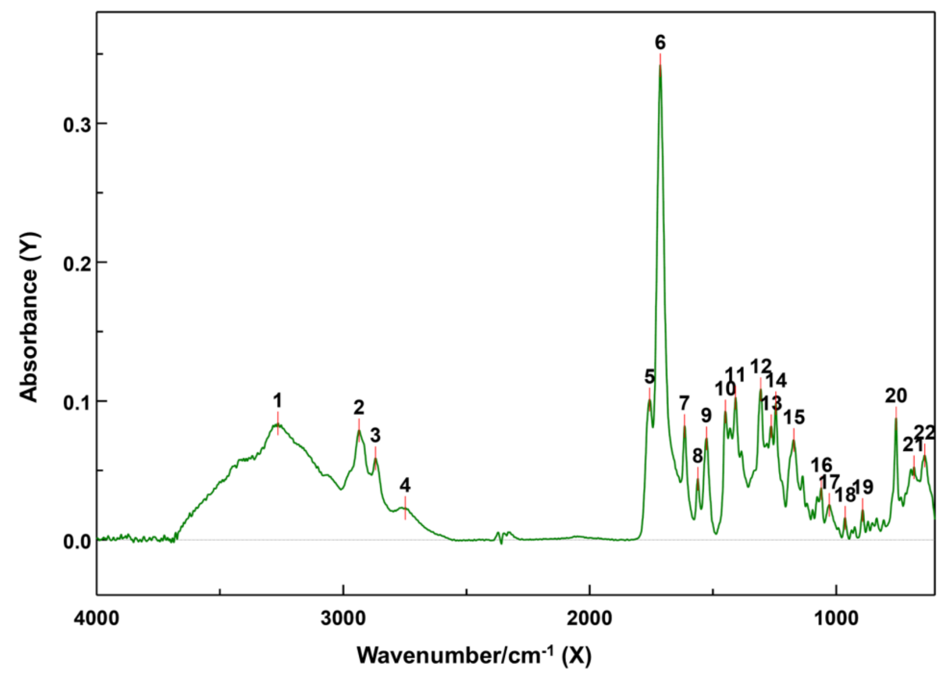

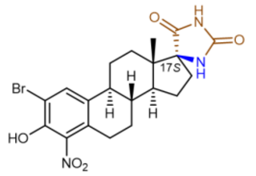

5t

No.	X	Y	No.	X	Y	No.	X	Y
1	3264.9	0.084	9	1526.4	0.073	17	1028	0.025
2	2935.1	0.079	10	1449.2	0.092	18	964.2	0.016
3	2868.6	0.059	11	1408.7	0.102	19	892.9	0.021
4	2748.1	0.023	12	1306.5	0.108	20	756.9	0.087
5	1756.8	0.101	13	1264.1	0.082	21	684.6	0.052
6	1713.4	0.342	14	1244.8	0.098	22	641.2	0.061
7	1615.1	0.082	15	1172.5	0.072			
8	1561.1	0.044	16	1060.7	0.037			

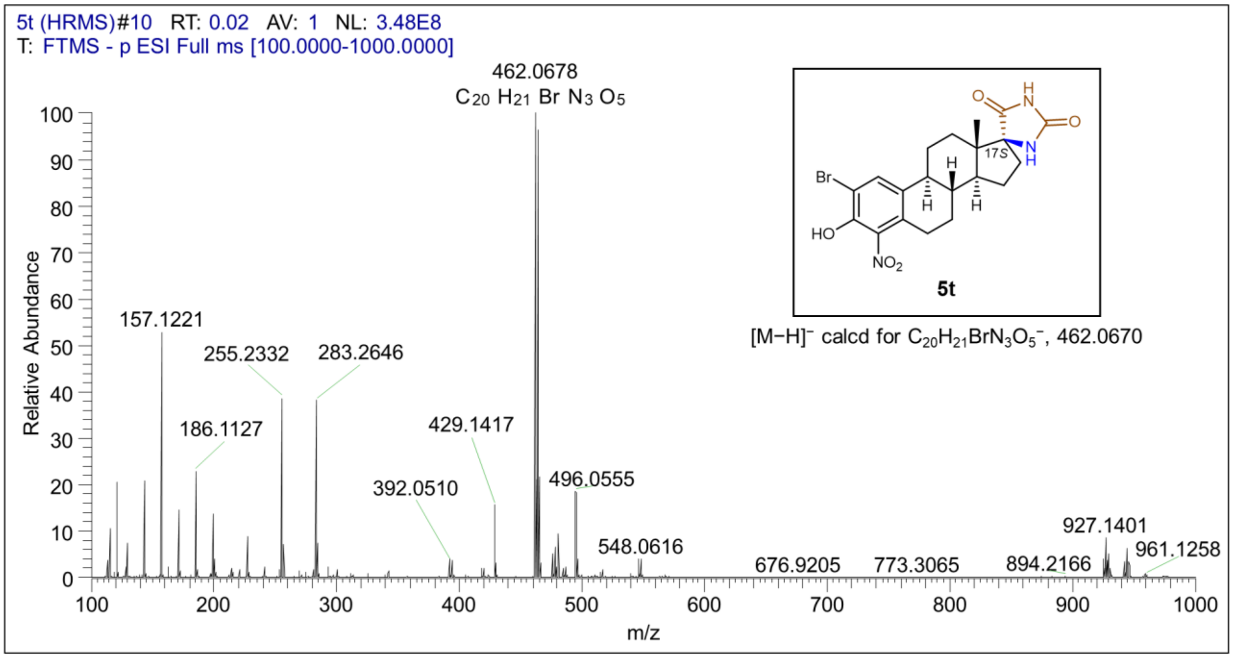

V **<u>Energetic and coordinate data from quantum chemical calculations</u>**

Section 7: Thermodynamic evaluations on the condensation of a ketone with ammonia or an amine to form a ketimine

Structure optimizations and frequency calculations were performed at the APFD/6-311+G(2d,p) level, as shown in Fig. S22.

Ammonia

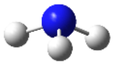

Electronic Energy: (*EE*) = −56.531655 Hartree
Thermal Correction to Free Energy = 0.016452 Hartree
Gibbs free energy: (*G*) = −56.515203 Hartree

1	N	0	0	0.11308	3	H	−0.815436	−0.470792	−0.263853
2	H	0	0.941585	−0.263853	4	H	0.815436	−0.470792	−0.263853

Methylamine

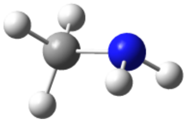

Electronic Energy: (*EE*) = −95.807837 Hartree
Thermal Correction to Free Energy = 0.04126 Hartree
Gibbs free energy: (*G*) = −95.766577 Hartree

1	N	0.049232	−0.754709	0	5	H	0.590095	1.063343	0.877764
2	H	−0.437557	−1.114871	0.812463	6	H	0.590095	1.063343	−0.877764
3	H	−0.437557	−1.114871	−0.812463	7	H	−0.94509	1.172058	0
4	C	0.049232	0.702327	0					

Aniline

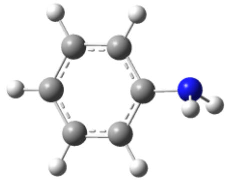

Electronic Energy: (*EE*) = −287.436749 Hartree
Thermal Correction to Free Energy = 0.088037 Hartree
Gibbs free energy: (*G*) = −287.348711 Hartree

1	N	2.355659	−0.116188	−0.000145	8	H	0.736013	−2.151652	−0.000033
2	H	2.743442	0.350032	−0.813369	9	C	−1.147638	1.217096	−0.000024

3	H	2.74346	0.348235	0.8141	10	H	0.807317	2.114303	−0.000105
4	C	0.923222	−0.029072	−0.000054	11	C	−1.870451	0.029831	0.000072
5	C	0.193075	−1.212531	0.000006	12	H	−1.753961	−2.115986	0.00015
6	C	0.240927	1.186891	−0.000064	13	H	−1.666802	2.170469	−0.000045
7	C	−1.196415	−1.184646	0.000097	14	H	−2.955412	0.052502	0.000117

Hexamethyldisilazane (HMDS)

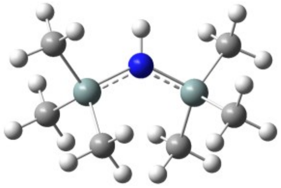

Electronic Energy: (*EE*) = −873.650884 Hartree
Thermal Correction to Free Energy = 0.194877 Hartree
Gibbs free energy: (*G*) = −873.456008 Hartree

1	Si	1.575329	0.008144	0.087216	15	C	−1.592686	−1.184625	−1.37316
2	Si	−1.575323	−0.008218	0.087218	16	H	−0.872579	−0.889169	−2.141052
3	C	2.064267	−1.69778	−0.520407	17	H	−1.351551	−2.202918	−1.058029
4	H	1.360591	−2.062103	−1.273346	18	H	−2.581946	−1.196584	−1.840027
5	H	3.0621	−1.686957	−0.969303	19	C	−2.064292	1.698314	−0.518653
6	H	2.071198	−2.415235	0.304805	20	H	−2.071446	2.41487	0.307337
7	C	2.797002	0.573629	1.390516	21	H	−1.360494	2.063512	−1.271054
8	H	2.7848	−0.09343	2.257734	22	H	−3.062031	1.68789	−0.967764
9	H	3.815532	0.576983	0.992518	23	C	−2.796912	−0.57509	1.389995
10	H	2.565389	1.584838	1.735316	24	H	−2.784571	0.090979	2.257971
11	C	1.592607	1.186037	−1.371956	25	H	−3.815481	−0.577917	0.992094
12	H	1.351539	2.204016	−1.055762	26	H	−2.56535	−1.586714	1.733619
13	H	2.581826	1.198434	−1.838898					
14	H	0.872423	0.891388	−2.140083					

Water

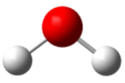

Electronic Energy: (*EE*) = −76.396231 Hartree
Thermal Correction to Free Energy = 0.003906 Hartree
Gibbs free energy: (*G*) = −76.392325 Hartree

1	O	0	0	0.116862	3	H	0	−0.761883	−0.467446
2	H	0	0.761883	−0.467446					

Hexamethylsiloxane

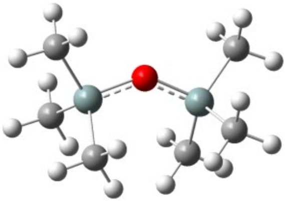

Electronic Energy: (*EE*) = −893.554236 Hartree
Thermal Correction to Free Energy = 0.184038 Hartree
Gibbs free energy: (*G*) = −893.370198 Hartree

1	O	0.000001	−0.000039	0.674925	15	H	1.014998	0.911285	−2.179094
2	Si	1.539783	0.000334	0.078436	16	C	−1.669058	−1.200865	−1.353009
3	Si	−1.539783	−0.000349	0.078442	17	H	−1.014898	−0.909854	−2.179662
4	C	1.976776	−1.722963	−0.50119	18	H	−1.394214	−2.212493	−1.042976
5	H	1.32828	−2.039436	−1.322126	19	H	−2.692974	−1.23297	−1.736889
6	H	3.011366	−1.765799	−0.854018	20	C	−1.977027	1.723237	−0.50013
7	H	1.867866	−2.444387	0.31303	21	H	−1.868331	2.444155	0.314567
8	C	2.660418	0.526127	1.473869	22	H	−1.328473	2.040341	−1.320779
9	H	2.568017	−0.158407	2.321072	23	H	−3.011578	1.766134	−0.853062
10	H	3.707201	0.535187	1.157144	24	C	−2.660329	−0.527192	1.47355
11	H	2.401753	1.529653	1.821484	25	H	−2.568017	0.156807	2.321195
12	C	1.669219	1.201706	−1.352281	26	H	−3.707114	−0.536197	1.15683
13	H	1.394504	2.213177	−1.041618	27	H	−2.40152	−1.530906	1.820514
14	H	2.693128	1.23392	−1.736167	15	H	1.014998	0.911285	−2.179094

3-Pentanone (1bm)

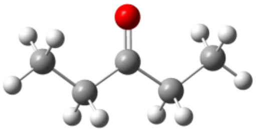

Electronic Energy: (*EE*) = −271.641174 Hartree
Thermal Correction to Free Energy = 0.105976 Hartree
Gibbs free energy: (*G*) = −271.535198 Hartree

1	C	0.000024	0.072914	−0.000162	9	C	−2.537989	0.127931	0.00072
2	C	1.285121	−0.729426	0.000691	10	H	−2.571507	0.768915	0.883834
3	H	1.257791	−1.397585	0.871361	11	H	−2.562815	0.784344	−0.871231
4	H	1.257967	−1.399414	−0.868578	12	H	−3.437144	−0.492708	−0.0089
5	C	2.537879	0.128012	−0.000034	13	C	−1.28542	−0.728872	−0.000988
6	H	2.566298	0.777475	0.876919	14	H	−1.257298	−1.400334	0.867322
7	H	3.436482	−0.493296	0.000742	15	H	−1.257864	−1.397358	−0.87163
8	H	2.566549	0.77563	−0.878345	16	O	0.000481	1.281373	−0.000358

Propiophenone (1al)

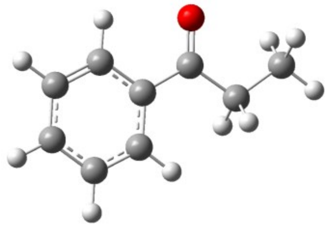

Electronic Energy: (EE) = −423.97625 Hartree
Thermal Correction to Free Energy = 0.131602 Hartree
Gibbs free energy: (G) = −423.844648 Hartree

1	C	−3.035407	−0.270347	0.000035	11	H	−3.17575	1.875834	−0.000001
2	C	−2.182941	−1.367124	0.000038	12	C	1.197471	0.377651	−0.00006
3	C	−0.807834	−1.177257	0.000003	13	C	2.148863	−0.800542	−0.000108
4	C	−0.273373	0.111571	−0.000038	14	H	1.917194	−1.424487	0.872279
5	C	−1.138688	1.206755	−0.000038	15	H	1.917371	−1.424305	−0.872669
6	C	−2.510535	1.018416	−0.000001	16	C	3.60863	−0.383272	0.000085
7	H	−4.11057	−0.419406	0.000064	17	H	3.842209	0.222063	0.877726
8	H	−2.590332	−2.372875	0.000069	18	H	4.261164	−1.259459	0.000036
9	H	−0.15466	−2.042594	0.000014	19	H	3.842374	0.222284	−0.87736
10	H	−0.705498	2.200957	−0.000067	20	O	1.614922	1.51586	0.000052

Benzophenone (1a)

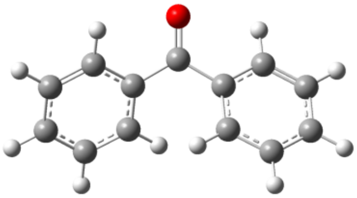

Electronic Energy: (EE) = −576.309995 Hartree
Thermal Correction to Free Energy = 0.154068 Hartree
Gibbs free energy: (G) = −576.155927 Hartree

1	C	1.288231	0.354552	0.030126	13	C	−2.652814	−1.509012	−0.725276
2	C	−0.000048	1.104143	0.000151	14	C	−1.416336	−0.880619	−0.664709
3	C	−1.288339	0.354619	−0.030019	15	H	−2.298218	1.926803	1.006519
4	O	0.000026	2.319746	0.000139	16	H	−4.507894	0.779923	0.956324
5	C	−2.411838	0.954914	0.538735	17	H	−4.726434	−1.41393	−0.173323
6	C	−3.640111	0.316104	0.498735	18	H	0.55148	−1.338081	1.13283
7	C	−3.762288	−0.916864	−0.135191	19	H	2.751054	−2.461667	1.235292
8	C	1.41649	−0.880548	0.664935	20	H	4.726582	−1.413665	0.173136
9	C	2.653061	−1.508828	0.725408	21	H	4.507553	0.779943	−0.95703

10	C	3.762362	−0.916745	0.135061	22	H	2.297889	1.92665	−1.006838
11	C	3.639953	0.316132	−0.499096	23	H	−2.750817	−2.461975	−1.234921
12	C	2.411655	0.954827	−0.538943	24	H	−0.551274	−1.338021	−1.132607

2-Adamantanone (1bg)

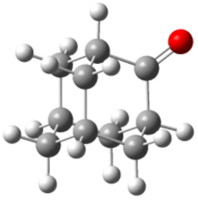

Electronic Energy: (*EE*) = −464.505522 Hartree
Thermal Correction to Free Energy = 0.192543 Hartree
Gibbs free energy: (*G*) = −464.312979 Hartree

1	C	−0.226133	−1.248903	1.25731	14	C	−1.114826	0.00003	−1.254083
2	H	−0.842214	−2.155771	1.272275	15	H	−1.747009	0.000052	−2.149398
3	H	0.398747	−1.270314	2.157038	16	C	−0.227087	1.248901	−1.257324
4	C	−1.113868	−0.00003	1.254741	17	H	0.397073	1.270319	−2.157554
5	H	−1.745368	−0.000052	2.15054	18	H	−0.843173	2.155775	−1.271803
6	C	0.66209	−1.262996	−0.0004	19	C	−0.226131	1.248841	1.257371
7	H	1.33382	−2.125287	−0.000647	20	H	0.398751	1.270206	2.157098
8	C	−0.227086	−1.24884	−1.257384	21	H	−0.842211	2.15571	1.272382
9	H	−0.843171	−2.155713	−1.27191	22	C	0.66209	1.262996	−0.000341
10	H	0.397076	−1.270211	−2.157614	23	H	1.33382	2.125287	−0.000545
11	C	−1.995332	0	0.000664	24	C	1.504397	0	−0.00051
12	H	−2.6478	0.881164	0.000934	25	O	2.713847	0	−0.000245
13	H	−2.647799	−0.881164	0.000892					

Pentan-3-imine

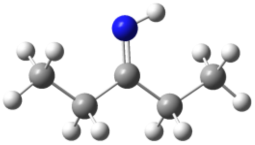

Electronic Energy: (*EE*) = −251.764398 Hartree
Thermal Correction to Free Energy = 0.122373 Hartree
Gibbs free energy: (*G*) = −251.642025 Hartree

1	C	−0.013251	0.088309	−0.00004	10	H	−2.616944	0.733532	0.879842
2	C	1.258259	−0.732104	0.00067	11	H	−2.613842	0.740556	−0.874002
3	H	1.22139	−1.400004	0.870899	12	H	−3.438446	−0.5669	−0.003644
4	H	1.221213	−1.401834	−0.868129	13	C	−1.282081	−0.725324	−0.000727

5	C	2.557403	0.05644	−0.00025	14	H	−1.249965	−1.396596	0.868186
6	H	2.643768	0.694328	0.884114	15	H	−1.250283	−1.394502	−0.871301
7	H	3.416876	−0.617753	0.000153	16	N	−0.08053	1.354835	−0.000135
8	H	2.643291	0.69288	−0.8857	17	H	0.850005	1.773218	0.000236
9	C	−2.564223	0.089217	0.000396					

(*E*)-phenylpropan-1-imine (2al)

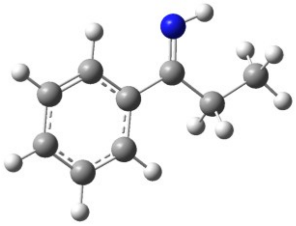

Electronic Energy: (*EE*) = −404.099088 Hartree
Thermal Correction to Free Energy = 0.144448 Hartree
Gibbs free energy: (*G*) = −403.954640 Hartree

1	C	−3.044021	−0.297307	−0.000071	12	C	1.188498	0.401129	0.000189
2	C	−2.170765	−1.37513	−0.000017	13	N	1.576385	1.613257	−0.000028
3	C	−0.797764	−1.160432	0.000056	14	H	2.592882	1.67605	−0.000182
4	C	−0.277311	0.134699	0.000086	15	C	2.114646	−0.796573	−0.000002
5	C	−1.168823	1.211803	0.000032	16	H	1.869434	−1.414985	0.871952
6	C	−2.536383	0.99879	−0.000047	17	H	1.86931	−1.414831	−0.872031
7	H	−4.11651	−0.464124	−0.000132	18	C	3.599555	−0.472602	−0.000083
8	H	−2.556791	−2.389442	−0.000034	19	H	3.891178	0.099256	0.885555
9	H	−0.137313	−2.01981	0.000098	20	H	4.18858	−1.392434	−0.000258
10	H	−0.758452	2.214904	0.000057	21	H	3.891025	0.099504	−0.885611
11	H	−3.213833	1.846852	−0.000086	12	C	1.188498	0.401129	0.000189

Diphenylmethanimine (2a)

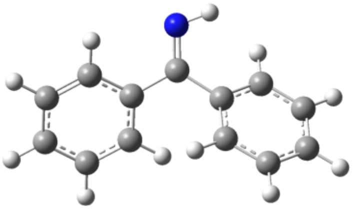

Electronic Energy: (*EE*) = −556.434573 Hartree
Thermal Correction to Free Energy = 0.166359 Hartree
Gibbs free energy: (*G*) = −556.268214 Hartree

1	C	1.289856	0.3734	0.020239	14	C	−1.461634	−0.695403	−0.900215
2	C	0.023266	1.141403	−0.056569	15	H	−0.857824	2.806925	−0.216285
3	C	−1.263142	0.392639	−0.049923	16	H	−2.148179	1.639312	1.456467
4	N	0.080356	2.414009	−0.133763	17	H	−4.329441	0.472759	1.408772
5	C	−2.306844	0.807845	0.776312	18	H	−4.681065	−1.433248	−0.135168
6	C	−3.528969	0.150526	0.750659	19	H	0.427283	−1.335875	0.997463
7	C	−3.724465	−0.921421	−0.111622	20	H	2.565033	−2.559193	1.167728
8	C	1.336159	−0.892522	0.604999	21	H	4.643569	−1.553872	0.269607
9	C	2.538933	−1.579638	0.701406	22	H	4.574681	0.69071	−0.779106
10	C	3.704492	−1.013778	0.202176	23	H	2.420555	1.925433	−0.917097
11	C	3.665561	0.247211	−0.385733	24	H	−2.839229	−2.174321	−1.617673
12	C	2.468321	0.938151	−0.471715	25	H	−0.649655	−1.026371	−1.539542
13	C	−2.689569	−1.340137	−0.939818					

Adamantan-2-imine (2bg)

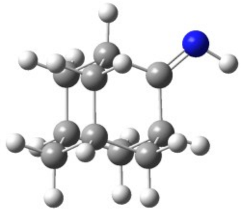

Electronic Energy: (*EE*) = −444.630531 Hartree
Thermal Correction to Free Energy = 0.205558 Hartree
Gibbs free energy: (*G*) = −444.424973 Hartree

1	C	0.261888	1.247551	1.255512	14	C	1.125393	−0.019452	−1.25396
2	H	0.89832	2.140072	1.26952	15	H	1.757535	−0.034405	−2.149216
3	H	−0.36365	1.282605	2.154252	16	C	0.211318	−1.249883	−1.255743
4	C	1.123699	−0.019711	1.255158	17	H	−0.412093	−1.258131	−2.156698
5	H	1.754624	−0.034795	2.151281	18	H	0.809258	−2.168922	−1.266938
6	C	−0.627563	1.274328	−0.00068	19	C	0.209776	−1.250254	1.255258
7	H	−1.276897	2.153219	−0.000802	20	H	−0.414412	−1.25908	2.155646
8	C	0.263747	1.247912	−1.255106	21	H	0.807988	−2.169144	1.266591
9	H	0.900394	2.140347	−1.267489	22	C	−0.680248	−1.236382	−0.000709
10	H	−0.359831	1.283499	−2.155189	23	H	−1.35477	−2.100249	−0.001375
11	C	2.005794	−0.039192	0.00119	24	C	−1.49131	0.039549	−0.000891
12	H	2.639589	−0.934099	0.001513	25	N	−2.756158	0.136643	−0.0003
13	H	2.676771	0.827915	0.00175	26	H	−3.184684	−0.792141	−0.000924

_N_-Methylpentan-3-imine

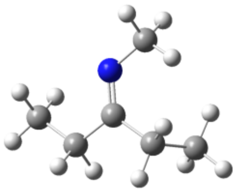

Electronic Energy: (*EE*) = −291.04513 Hartree
Thermal Correction to Free Energy = 0.14813 Hartree
Gibbs free energy: (*G*) = −290.897 Hartree

1	C	0.11151	−0.031502	−0.264807	11	H	2.42642	0.319649	1.321803
2	C	−1.102412	−0.856309	−0.639471	12	H	3.497239	−0.663319	0.304699
3	H	−1.770899	−0.283719	−1.288808	13	C	1.404268	−0.805178	−0.218185
4	H	−0.774284	−1.723888	−1.220351	14	H	1.599077	−1.181856	−1.232485
5	C	−1.858497	−1.32682	0.605009	15	H	1.231206	−1.70869	0.383665
6	H	−2.232035	−0.477921	1.182379	16	N	0.122268	1.199634	0.035563
7	H	−2.711512	−1.955316	0.336363	17	C	−1.080844	1.991581	0.027008
8	H	−1.208153	−1.909924	1.262755	18	H	−1.977515	1.501894	−0.372232
9	C	2.60294	−0.034432	0.304049	19	H	−1.296521	2.312315	1.051245
10	H	2.797136	0.847389	−0.308906	20	H	−0.897827	2.901908	−0.550695

(_E_)-_N_-Methyl-1-phenylpropan-1-imine

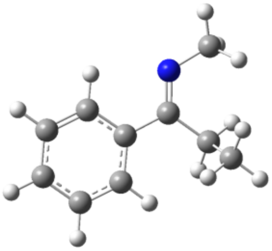

Electronic Energy: (*EE*) = −443.379706 Hartree
Thermal Correction to Free Energy = 0.169461 Hartree
Gibbs free energy: (*G*) = −443.210246 Hartree

1	C	3.250099	−0.037057	0.085098	13	N	−1.576143	−1.31855	−0.008327
2	C	2.538634	1.119636	−0.19614	14	C	−1.789079	1.086146	−0.513229
3	C	1.153217	1.0793	−0.299676	15	H	−1.239068	1.682989	−1.245343
4	C	0.454497	−0.11591	−0.120573	16	C	−3.010573	−1.467126	−0.065351
5	C	1.185631	−1.273518	0.166815	17	H	−3.559585	−0.682385	0.468317
6	C	2.56525	−1.235806	0.26577	18	H	−3.361458	−1.472469	−1.104987
7	H	4.332203	−0.007659	0.164014	19	H	−3.283491	−2.428004	0.373385
8	H	3.061025	2.060452	−0.337507	20	C	−2.029651	1.905662	0.758154
9	H	0.622267	1.998111	−0.520098	21	H	−2.618986	1.339196	1.483384
10	H	0.639282	−2.198666	0.30839	22	H	−1.08691	2.170041	1.242685

11	H	3.114225	−2.145982	0.486277	23	H	−2.567708	2.830302	0.534273
12	C	−1.030588	−0.187392	−0.215972	24	H	−2.7434	0.840311	−0.983885

N-Methyl-1,1-diphenylmethanimine

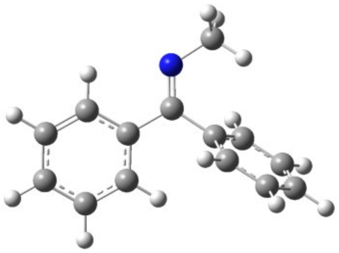

Electronic Energy: (*EE*) = −595.717205 Hartree
Thermal Correction to Free Energy = 0.19033 Hartree
Gibbs free energy: (*G*) = −595.526875 Hartree

1	C	−1.362404	0.198088	−0.026527	15	H	1.704382	0.602411	−1.997504
2	C	−0.074805	0.936618	0.009235	16	H	3.781006	−0.747019	−1.97303
3	C	1.17711	0.120423	0.024273	17	H	4.430549	−1.981701	0.074555
4	N	−0.10099	2.208319	0.024971	18	H	−0.475299	−1.707139	−0.476985
5	C	1.990679	0.056855	−1.103666	19	H	−2.626385	−2.912034	−0.557799
6	C	3.155407	−0.699608	−1.087407	20	H	−4.746085	−1.697592	−0.142365
7	C	3.5191	−1.392891	0.060341	21	H	−4.696106	0.73311	0.345216
8	C	−1.400199	−1.170416	−0.296005	22	H	−2.523508	1.93828	0.408417
9	C	−2.611968	−1.848566	−0.341638	23	H	2.991335	−1.871511	2.088695
10	C	−3.799059	−1.168086	−0.111455	24	H	0.901242	−0.545237	2.044832
11	C	−3.770063	0.197413	0.161375	25	C	1.108496	2.985494	0.09745
12	C	−2.563942	0.874878	0.201892	26	H	1.220223	3.552346	−0.832789
13	C	2.711206	−1.331816	1.189689	27	H	0.995144	3.724027	0.896413
14	C	1.541369	−0.584422	1.168869	28	H	2.024866	2.410032	0.268988

N-Methyladamantan-2-imine

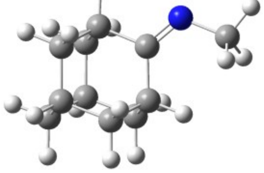

Electronic Energy: (*EE*) = −483.911574 Hartree
Thermal Correction to Free Energy = 0.230114 Hartree
Gibbs free energy: (*G*) = −483.681459 Hartree

1	C	−0.845749	1.209725	−1.254124	16	C	−0.310541	−1.22939	1.255892
2	H	−1.643133	1.962331	−1.266657	17	H	0.302749	−1.115321	2.156784
3	H	−0.239876	1.365936	−2.153665	18	H	−0.719369	−2.247022	1.270004
4	C	−1.445729	−0.199967	−1.254772	19	C	−0.310657	−1.229024	−1.256254
5	H	−2.063023	−0.337618	−2.150175	20	H	0.302519	−1.114723	−2.157194
6	C	0.02377	1.403102	0.000164	21	H	−0.719495	−2.246651	−1.270592
7	H	0.494199	2.389445	0.000283	22	C	0.56239	−1.050019	−0.000211
8	C	−0.845622	1.209362	1.254483	23	H	1.369659	−1.785612	−0.000343
9	H	−1.643017	1.961957	1.267287	24	C	1.113849	0.3607	−0.000019
10	H	−0.239687	1.365335	2.154021	25	N	2.326591	0.727964	0.000028
11	C	−2.306209	−0.39084	0.000029	26	C	3.381272	−0.267979	−0.000022
12	H	−2.754007	−1.391995	−0.000094	27	H	4.346216	0.240632	−0.000078
13	H	−3.132902	0.329396	0.000181	28	H	3.34442	−0.912782	0.886129
14	C	−1.445602	−0.200328	1.254802	29	H	3.3444	−0.912869	−0.886118
15	H	−2.062817	−0.338222	2.150223					

N-Phenylpentan-3-imine

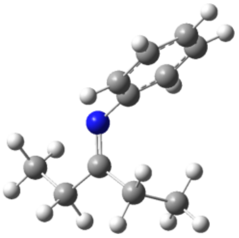

Electronic Energy: (*EE*) = −482.681664 Hartree
Thermal Correction to Free Energy = 0.195763 Hartree
Gibbs free energy: (*G*) = −482.485901 Hartree

1	C	−1.522146	0.029479	−0.283134	15	C	2.811313	−0.589057	−1.081622
2	C	−1.20694	1.374808	−0.891079	16	H	0.921415	−1.216835	−1.904597
3	H	−0.239115	1.332949	−1.396963	17	C	2.752593	0.20907	1.179289
4	C	−3.293012	−1.591763	0.57818	18	H	0.818917	0.195427	2.129917
5	H	−2.788369	−2.454315	0.139521	19	C	3.47486	−0.0758	0.026255
6	H	−2.94737	−1.511739	1.610963	20	H	3.364829	−0.823472	−1.985781
7	H	−4.368725	−1.784761	0.586866	21	H	3.260383	0.603049	2.054336
8	C	−2.980389	−0.325784	−0.198772	22	H	4.545915	0.092926	−0.005565
9	H	−3.354033	−0.400421	−1.22983	23	C	−1.176911	2.466751	0.182658
10	H	−3.512045	0.536593	0.225543	24	H	−1.006428	3.448047	−0.266758
11	N	−0.659244	−0.779718	0.177305	25	H	−2.11934	2.512035	0.735167
12	C	0.712101	−0.507565	0.108092	26	H	−0.374594	2.279375	0.898967
13	C	1.44134	−0.807541	−1.04425	27	H	−1.964296	1.615509	−1.644193
14	C	1.383452	−0.010322	1.226261					

(*E*)-*N*-Phenyl-1-phenylpropan-1-imine

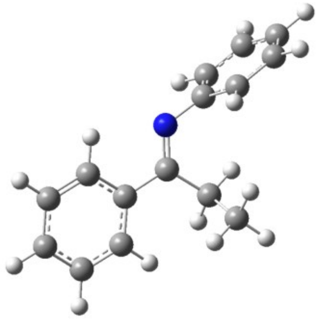

Electronic Energy: (*EE*) = −635.016806 Hartree
Thermal Correction to Free Energy = 0.216975 Hartree
Gibbs free energy: (*G*) = −634.799831 Hartree

1	C	4.568134	−0.701709	0.116853	17	C	−2.529238	−0.87489	−1.108245
2	C	4.169414	0.513095	−0.42154	18	C	−2.493936	−0.082663	1.165693
3	C	2.818339	0.81743	−0.53003	19	C	−3.909819	−0.737805	−1.129143
4	C	1.845476	−0.088438	−0.104926	20	H	−1.993837	−1.244063	−1.976826
5	C	2.26133	−1.308068	0.438163	21	C	−3.87517	0.048425	1.136488
6	C	3.60696	−1.61203	0.5473	22	H	−1.932966	0.156523	2.063182
7	H	5.623458	−0.94001	0.20329	23	C	−4.591285	−0.274235	−0.009916
8	H	4.911216	1.229337	−0.759774	24	H	−4.457982	−0.997813	−2.029495
9	H	2.529647	1.772243	−0.953699	25	H	−4.395912	0.405905	2.019543
10	H	1.502176	−2.007547	0.768988	26	H	−5.670945	−0.171447	−0.028986
11	H	3.911944	−2.563289	0.971692	27	H	−1.07414	1.559355	−0.955158
12	C	0.39328	0.210673	−0.212056	28	C	0.09412	2.591731	0.539729
13	N	−0.423362	−0.723183	0.07955	29	H	1.113701	2.624145	0.930694
14	C	−0.036085	1.599104	−0.618596	30	H	−0.178691	3.599699	0.217782
15	H	0.559664	1.934957	−1.47225	31	H	−0.56865	2.310527	1.360948
16	C	−1.807379	−0.534994	0.037427					

***N*-Phenyl -1,1-diphenylmethanimine (3a)**

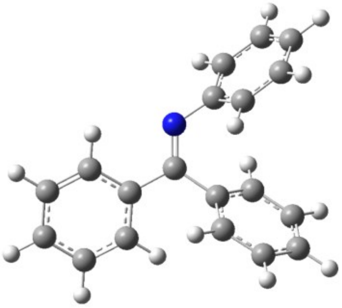

Electronic Energy: (*EE*) = −787.353029 Hartree
Thermal Correction to Free Energy = 0.239536 Hartree
Gibbs free energy: (*G*) = −787.113493 Hartree

1	C	2.020573	−0.561398	−0.03085	19	H	4.933989	0.971225	−0.871448
2	C	0.553897	−0.353651	−0.045376	20	H	5.849045	−1.173453	−0.03396
3	C	0.049447	1.048092	−0.07378	21	H	4.3097	−2.928961	0.79885
4	N	−0.195745	−1.388035	−0.048273	22	H	1.856866	−2.532661	0.780546
5	C	−0.852512	1.451054	−1.056444	23	H	0.274026	3.960502	1.655794
6	C	−1.337276	2.750182	−1.070671	24	H	1.17181	1.654558	1.652334
7	C	−0.937989	3.654422	−0.093511	25	C	−1.588734	−1.318492	0.042658
8	C	2.894597	0.424044	−0.492128	26	C	−2.235826	−0.686193	1.107701
9	C	4.265018	0.201538	−0.500331	27	C	−2.352212	−1.979956	−0.920705
10	C	4.777234	−1.002204	−0.035248	28	C	−3.619905	−0.696264	1.187829
11	C	3.912343	−1.988346	0.431019	29	H	−1.643446	−0.189831	1.868584
12	C	2.544893	−1.771914	0.429273	30	C	−3.736831	−1.965285	−0.846419
13	C	−0.040206	3.258255	0.890342	31	H	−1.841508	−2.494633	−1.727958
14	C	0.460892	1.964045	0.892644	32	C	−4.378894	−1.324589	0.207461
15	H	−1.178082	0.73772	−1.805513	33	H	−4.110107	−0.204473	2.022343
16	H	−2.034333	3.05675	−1.843696	34	H	−4.319231	−2.468755	−1.611833
17	H	−1.324704	4.6684	−0.100198	35	H	−5.461945	−1.324956	0.27029
18	H	2.497092	1.364787	−0.857008					

***N*-Phenyladamantan-2-imine**

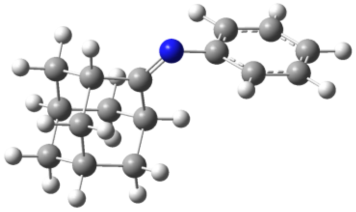

Electronic Energy: (*EE*) = −675.549519 Hartree
Thermal Correction to Free Energy = 0.277025 Hartree
Gibbs free energy: (*G*) = −675.272494 Hartree

1	C	−0.845749	1.209725	−1.254124	16	C	−0.310541	−1.22939	1.255892
2	H	−1.643133	1.962331	−1.266657	17	H	0.302749	−1.115321	2.156784
3	H	−0.239876	1.365936	−2.153665	18	H	−0.719369	−2.247022	1.270004
4	C	−1.445729	−0.199967	−1.254772	19	C	−0.310657	−1.229024	−1.256254
5	H	−2.063023	−0.337618	−2.150175	20	H	0.302519	−1.114723	−2.157194
6	C	0.02377	1.403102	0.000164	21	H	−0.719495	−2.246651	−1.270592
7	H	0.494199	2.389445	0.000283	22	C	0.56239	−1.050019	−0.000211
8	C	−0.845622	1.209362	1.254483	23	H	1.369659	−1.785612	−0.000343
9	H	−1.643017	1.961957	1.267287	24	C	1.113849	0.3607	−0.000019
10	H	−0.239687	1.365335	2.154021	25	N	2.326591	0.727964	0.000028
11	C	−2.306209	−0.39084	0.000029	26	C	3.381272	−0.267979	−0.000022
12	H	−2.754007	−1.391995	−0.000094	27	H	4.346216	0.240632	−0.000078

13	H	−3.132902	0.329396	0.000181	28	H	3.34442	−0.912782	0.886129
14	C	−1.445602	−0.200328	1.254802	29	H	3.3444	−0.912869	−0.886118
15	H	−2.062817	−0.338222	2.150223					

Section 8: Exploration into reaction pathways

<u>Chemical species in reaction pathway exploration (Fig. S23 and S24)</u>

Cluster A ([AlO3]*clus* + Benzophenone + NH3)

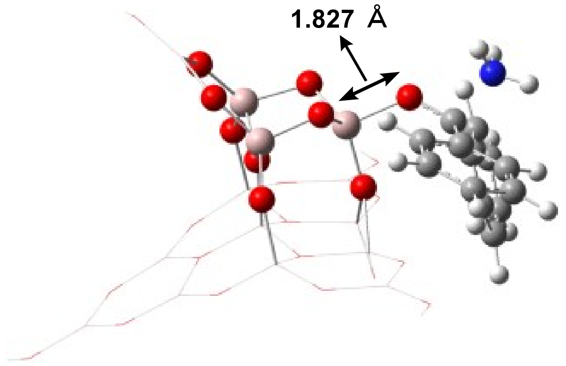

APFD/6-31G(d,p): Thermal Correction to Free Energy = 0.191845 Hartree

APFD/6-311+G(2d,p)//APFD/6-31G(d,p):
Electronic Energy (*EE*) = −5835.493815 Hartree
Gibbs free energy (*G*) = −5835.30197 Hartree

1	O	−1.395057	−2.11607	3.548977	36	Al	−6.548421	−0.882525	−1.826349
2	O	0.954321	0.869936	−2.693368	37	O	−8.145219	−1.21928	−2.257288
3	O	−0.432036	−1.182912	−1.273415	38	H	−8.856174	−0.577747	−2.292908
4	O	−1.070003	1.69951	−0.827514	39	O	1.103676	−1.257039	2.295031
5	O	−0.691473	−3.029028	0.791194	40	O	0.514257	1.455467	2.653355
6	O	−1.985325	2.547424	1.696067	41	O	1.351023	0.583111	−0.202942
7	O	1.154261	3.57386	−1.13923	42	Al	1.387925	0.349885	1.564608
8	O	2.338824	−1.824415	−1.934467	43	C	4.190938	0.321898	1.015292
9	O	−1.063911	−0.185593	1.309069	44	O	3.181074	0.520733	1.867404
10	Al	−1.271596	1.272029	2.601393	45	H	5.398532	0.067683	2.770425
11	Al	−0.54221	−1.901533	2.08127	46	N	5.478323	0.672234	1.95147
12	Al	−1.440282	0.015285	−0.447933	47	H	6.365622	0.506196	1.478098
13	Al	1.24423	−0.541971	−1.700348	48	H	5.414025	1.646386	2.248477
14	Al	0.685352	1.952593	−1.337786	49	C	4.485934	−1.124989	0.626211
15	O	−3.210933	−0.307429	−0.853488	50	C	5.564434	−1.438008	−0.211652
16	O	−3.703662	2.574691	−0.52327	51	C	3.704565	−2.155849	1.145338
17	O	−2.46786	−3.09423	−1.399891	52	C	5.810844	−2.755576	−0.580349
18	Al	−0.778755	−2.985299	−0.970511	53	H	6.186386	−0.645912	−0.625513
19	O	0.422385	−3.932129	−1.75556	54	C	3.949365	−3.477303	0.7687
20	H	3.060384	−5.508458	−2.748264	55	H	2.892894	−1.93229	1.836589
21	O	−1.420266	4.559701	−0.34483	56	C	4.993043	−3.779753	−0.101263
22	H	0.291974	7.260802	−0.524712	57	H	6.626247	−2.983096	−1.260751
23	Al	0.200238	4.923617	−0.743649	58	H	3.318356	−4.268655	1.165909

24	Al	2.027238	−3.487729	−2.126842	59	H	5.165651	−4.803347	−0.417125
25	O	0.828177	6.505429	−0.772526	60	C	4.260615	1.33787	−0.123051
26	O	3.226111	−4.568224	−2.661189	61	C	4.254794	2.699873	0.209035
27	Al	−2.093761	3.016747	−0.000793	62	C	4.196439	0.964032	−1.464801
28	O	−2.039166	0.620305	3.985374	63	C	4.239033	3.672895	−0.783052
29	Al	−4.361276	1.099814	−0.914261	64	H	4.189274	3.007756	1.252009
30	Al	−3.696481	−1.975321	−1.389387	65	C	4.152981	1.941457	−2.459324
31	O	−5.919224	0.642968	−1.376529	66	H	4.121597	−0.084663	−1.738819
32	O	−5.331192	−2.077099	−1.788021	67	C	4.192626	3.293871	−2.122449
33	Al	−2.15199	−0.973752	4.568149	68	H	4.212324	4.724094	−0.512871
34	O	−3.004175	−1.36185	5.983506	69	H	4.061527	1.638082	−3.497478
35	H	−3.053727	−2.252733	6.333803	70	H	4.143599	4.051129	−2.898869

TS I

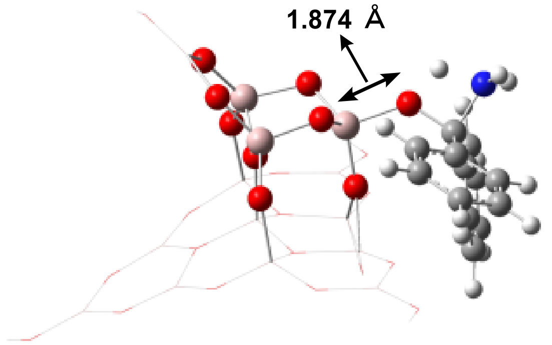

APFD/6-31G(d,p): Thermal Correction to Free Energy = 0.187349 Hartree

APFD/6-311+G(2d,p)//APFD/6-31G(d,p):
Electronic Energy (*EE*) = −5835.465692 Hartree
Gibbs free energy (*G*) = −5835.278343 Hartree

1	O	−1.40135	−2.129741	3.544006	36	Al	−6.528791	−0.904702	−1.857989
2	O	0.971118	0.882692	−2.676871	37	O	−8.121574	−1.247293	−2.299067
3	O	−0.414461	−1.179863	−1.270289	38	H	−8.835109	−0.608774	−2.337168
4	O	−1.067605	1.698582	−0.820686	39	O	1.109443	−1.278716	2.277731
5	O	−0.677794	−3.032453	0.788019	40	O	0.492255	1.464733	2.664651
6	O	−2.001251	2.535936	1.699706	41	O	1.357575	0.592525	−0.188442
7	O	1.150211	3.583413	−1.114643	42	Al	1.315674	0.341739	1.568073
8	O	2.362967	−1.807562	−1.916873	43	C	4.269643	0.356069	1.006783
9	O	−1.065662	−0.192024	1.311037	44	O	3.151599	0.560048	1.876641
10	Al	−1.287214	1.261315	2.60588	45	H	4.122027	0.851179	2.633479
11	Al	−0.54095	−1.907675	2.081838	46	N	5.253557	0.770219	2.101132

12	Al	−1.43271	0.011778	−0.4476	47	H	5.870147	0.013178	2.384937
13	Al	1.261434	−0.530514	−1.68582	48	H	5.784736	1.613091	1.897159
14	Al	0.689557	1.960639	−1.320093	49	C	4.535035	−1.090927	0.641919
15	O	−3.19955	−0.317595	−0.864269	50	C	5.643436	−1.404709	−0.153627
16	O	−3.706787	2.561481	−0.529503	51	C	3.727015	−2.118289	1.127629
17	O	−2.441138	−3.099701	−1.413515	52	C	5.891965	−2.722408	−0.522412
18	Al	−0.755045	−2.984526	−0.974045	53	H	6.293039	−0.611443	−0.516136
19	O	0.454759	−3.924067	−1.754534	54	C	3.97673	−3.439882	0.75041
20	H	3.105341	−5.486298	−2.735942	55	H	2.897022	−1.899521	1.797191
21	O	−1.433168	4.555955	−0.332685	56	C	5.048451	−3.744479	−0.082464
22	H	0.268256	7.264952	−0.495659	57	H	6.733782	−2.951844	−1.169208
23	Al	0.188015	4.927966	−0.721141	58	H	3.327052	−4.228649	1.122083
24	Al	2.05978	−3.471713	−2.115337	59	H	5.225458	−4.767367	−0.398111
25	O	0.809186	6.51257	−0.742296	60	C	4.279533	1.341549	−0.147708
26	O	3.266447	−4.54558	−2.645486	61	C	4.261366	2.713342	0.140594
27	Al	−2.101893	3.009191	0.003459	62	C	4.197444	0.92504	−1.474949
28	O	−2.059942	0.602656	3.983694	63	C	4.211167	3.653941	−0.885045
29	Al	−4.355665	1.084773	−0.928105	64	H	4.228009	3.057909	1.172357
30	Al	−3.674681	−1.986189	−1.407272	65	C	4.120387	1.870542	−2.501477
31	O	−5.908892	0.622344	−1.400595	66	H	4.14152	−0.132875	−1.715315
32	O	−5.306591	−2.094055	−1.815662	67	C	4.145497	3.233794	−2.209574
33	Al	−2.169172	−0.993387	4.561699	68	H	4.177091	4.712275	−0.645908
34	O	−3.027847	−1.388873	5.971075	69	H	4.019852	1.530954	−3.527364
35	H	−3.075534	−2.280871	6.318784	70	H	4.072387	3.964742	−3.008808

Cluster B ($[AlO_3]_{clus}$ + Hemiaminal−$OH_{adsorption}$)

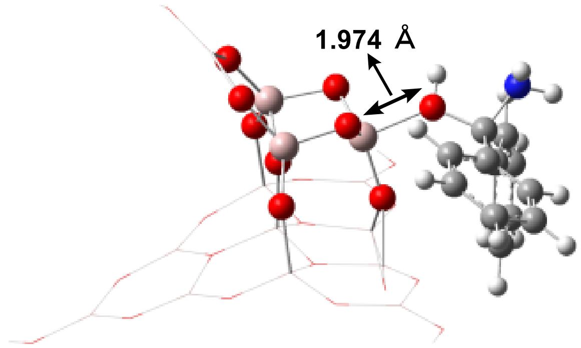

APFD/6-31G(d,p): Thermal Correction to Free Energy = 0.190780 Hartree

APFD/6-311+G(2d,p)//APFD/6-31G(d,p):
Electronic Energy (*EE*) = −5835.497548 Hartree
Gibbs free energy (*G*) = −5835.306768 Hartree

1	O	−1.423921	−2.037603	3.580883	36	Al	−6.598496	−0.654627	−1.737394
2	O	0.962358	0.735242	−2.745228	37	O	−8.215609	−0.924223	−2.138819
3	O	−0.494473	−1.228133	−1.270358	38	H	−8.89682	−0.251206	−2.174713
4	O	−0.992354	1.687516	−0.863801	39	O	1.101631	−1.359691	2.226592
5	O	−0.806151	−3.026621	0.828776	40	O	0.623584	1.455865	2.583564
6	O	−1.828257	2.617552	1.658407	41	O	1.392004	0.467767	−0.259943
7	O	1.310504	3.451864	−1.240142	42	Al	1.229152	0.232199	1.477088
8	O	2.233344	−2.007522	−1.961553	43	C	4.387211	0.296508	1.087903
9	O	−1.039481	−0.161007	1.304004	44	O	3.149426	0.463229	1.873156
10	Al	−1.159804	1.325389	2.574456	45	H	3.337998	1.129933	2.550717
11	Al	−0.585141	−1.886475	2.097257	46	N	5.437965	0.630659	2.029764
12	Al	−1.433617	0.028555	−0.450358	47	H	5.708396	−0.178985	2.57792
13	Al	1.202589	−0.672278	−1.732907	48	H	6.252268	1.009158	1.561628
14	Al	0.764591	1.850976	−1.404288	49	C	4.492394	−1.15653	0.649832
15	O	−3.223336	−0.218578	−0.824062	50	C	5.49061	−1.50357	−0.273083
16	O	−3.57797	2.688189	−0.53531	51	C	3.697436	−2.168895	1.207388
17	O	−2.617651	−3.045202	−1.334272	52	C	5.616475	−2.813306	−0.707796
18	Al	−0.918828	−3.007445	−0.931926	53	H	6.138963	−0.734311	−0.683357
19	O	0.225159	−4.021319	−1.718615	54	C	3.82718	−3.48594	0.764128
20	H	2.77216	−5.733652	−2.723609	55	H	2.963352	−1.938938	1.97406
21	O	−1.203315	4.568272	−0.424366	56	C	4.77351	−3.813048	−0.199332
22	H	0.628147	7.184159	−0.675273	57	H	6.362227	−3.064586	−1.456127
23	Al	0.425767	4.850464	−0.8533	58	H	3.188394	−4.253141	1.194196
24	Al	1.842735	−3.657572	−2.121147	59	H	4.863457	−4.830967	−0.563238
25	O	1.125159	6.400917	−0.918224	60	C	4.381676	1.307875	−0.050788
26	O	2.982232	−4.800786	−2.654905	61	C	4.59151	2.662823	0.258691
27	Al	−1.941505	3.063838	−0.044343	62	C	4.099802	0.94414	−1.369856
28	O	−1.934792	0.732303	3.980498	63	C	4.513505	3.632544	−0.731434
29	Al	−4.308647	1.239113	−0.891514	64	H	4.801963	2.954391	1.284573
30	Al	−3.793277	−1.87073	−1.324431	65	C	4.023262	1.914908	−2.362596
31	O	−5.892959	0.847269	−1.322809	66	H	3.919411	−0.096972	−1.623041
32	O	−5.436961	−1.903357	−1.696968	67	C	4.225474	3.262246	−2.044558
33	Al	−2.11159	−0.845196	4.591737	68	H	4.652431	4.679403	−0.478975
34	O	−2.958496	−1.170555	6.025949	69	H	3.769265	1.62201	−3.376043
35	H	−3.043434	−2.05241	6.391947	70	H	4.137316	4.021161	−2.81572

Cluster C ([AlO3]*clus* + Hemiaminal−NH2*adsorption*)

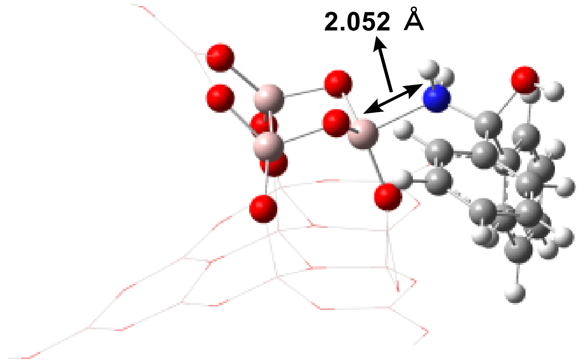

APFD/6-31G(d,p): Thermal Correction to Free Energy = 0.192117 Hartree

APFD/6-311+G(2d,p)//APFD/6-31G(d,p):
Electronic Energy (*EE*) = −5835.505765 Hartree
Gibbs free energy (*G*) = −5835.313648 Hartree

1	O	−1.397305	−2.07756	3.563921	36	Al	−6.577684	−0.787926	−1.77213
2	O	0.947961	0.816537	−2.723098	37	O	−8.184548	−1.097833	−2.185437
3	O	−0.463005	−1.199101	−1.27437	38	H	−8.883669	−0.443331	−2.219333
4	O	−1.042581	1.698092	−0.844775	39	O	1.119993	−1.318771	2.239694
5	O	−0.737281	−3.024376	0.806787	40	O	0.558139	1.47869	2.600655
6	O	−1.917751	2.582093	1.680665	41	O	1.362178	0.547778	−0.241095
7	O	1.213333	3.527876	−1.191842	42	Al	1.235905	0.280692	1.493165
8	O	2.288921	−1.897569	−1.956444	43	C	4.405058	0.33898	1.12127
9	O	−1.051432	−0.170612	1.306154	44	C	4.531618	−1.119613	0.696625
10	Al	−1.219439	1.300369	2.589073	45	C	5.544147	−1.446684	−0.211207
11	Al	−0.554626	−1.890183	2.086653	46	C	3.738834	−2.1455	1.213674
12	Al	−1.440709	0.024042	−0.448734	47	C	5.716839	−2.758596	−0.647
13	Al	1.220826	−0.593037	−1.721968	48	H	6.179814	−0.663668	−0.620346
14	Al	0.712261	1.91426	−1.373453	49	C	3.909025	−3.463444	0.770229
15	O	−3.220883	−0.268369	−0.835135	50	H	2.957142	−1.950276	1.94269
16	O	−3.656362	2.624917	−0.522578	51	C	4.891271	−3.774979	−0.161113
17	O	−2.535391	−3.072696	−1.366947	52	H	6.483356	−2.985238	−1.382206
18	Al	−0.840526	−2.992312	−0.954306	53	H	3.261525	−4.239944	1.170047
19	O	0.335126	−3.967462	−1.743266	54	H	5.00366	−4.788908	−0.529159
20	H	2.933545	−5.600569	−2.748499	55	C	4.358257	1.302545	−0.068587
21	O	−1.334581	4.567924	−0.380934	56	C	4.627581	2.650087	0.161891
22	H	0.426166	7.234915	−0.597752	57	C	4.027176	0.891979	−1.364346
23	Al	0.288577	4.898282	−0.797755	58	C	4.537507	3.584429	−0.878612
24	Al	1.944409	−3.556135	−2.133029	59	H	4.925904	2.973951	1.155847
25	O	0.945736	6.467743	−0.84475	60	C	3.950088	1.82621	−2.409576
26	O	3.117679	−4.662986	−2.67025	61	H	3.831699	−0.157019	−1.572515

27	Al	−2.033553	3.040551	−0.018675	62	C	4.196323	3.1737	−2.162954
28	O	−1.985847	0.673659	3.985197	63	H	4.731501	4.633502	−0.675209
29	Al	−4.345188	1.159759	−0.895969	64	H	3.675503	1.484677	−3.402168
30	Al	−3.742681	−1.930832	−1.353548	65	H	4.111017	3.901186	−2.9637
31	O	−5.915759	0.728818	−1.340039	66	N	3.208625	0.579469	1.970674
32	O	−5.382737	−2.004854	−1.73601	67	H	3.350093	0.066506	2.843791
33	Al	−2.122953	−0.91355	4.581304	68	H	3.240213	1.563699	2.242991
34	O	−2.96872	−1.274859	6.007559	69	O	5.482288	0.685581	1.993827
35	H	−3.031612	−2.161983	6.365178	70	H	6.304004	0.418509	1.565792

TS II

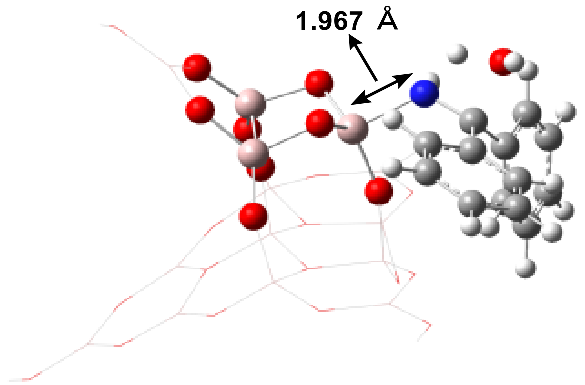

APFD/6-31G(d,p): Thermal Correction to Free Energy = 0.186105 Hartree

APFD/6-311+G(2d,p)//APFD/6-31G(d,p):
Electronic Energy (*EE*) = −5835.432207 Hartree
Gibbs free energy (*G*) = −5835.246102 Hartree

1	O	−1.557729	−2.052885	3.556951	36	Al	−6.605272	−0.480174	−1.830238
2	O	1.004254	0.72738	−2.696771	37	O	−8.221698	−0.704023	−2.261446
3	O	−0.526463	−1.214252	−1.268695	38	H	−8.884952	−0.013499	−2.300955
4	O	−0.957151	1.708515	−0.837268	39	O	1.007858	−1.401542	2.2824
5	O	−0.918905	−3.027108	0.804374	40	O	0.590512	1.409469	2.620973
6	O	−1.811627	2.631863	1.68117	41	O	1.375779	0.419772	−0.209111
7	O	1.395652	3.417577	−1.155289	42	Al	1.327337	0.187369	1.556168
8	O	2.192009	−2.055332	−1.923428	43	C	4.259003	0.212353	0.933916
9	O	−1.087589	−0.161857	1.308268	44	C	4.364274	−1.235222	0.560015
10	Al	−1.191635	1.313107	2.593395	45	C	5.299743	−1.635409	−0.403726
11	Al	−0.69051	−1.906943	2.089241	46	C	3.611665	−2.210592	1.225263
12	Al	−1.447176	0.056914	−0.45011	47	C	5.413332	−2.97426	−0.759729
13	Al	1.191681	−0.696822	−1.696704	48	H	5.922868	−0.892855	−0.892829
14	Al	0.812228	1.833028	−1.346675	49	C	3.724606	−3.552374	0.859244

15	O	−3.236027	−0.140383	−0.856205	50	H	2.9182	−1.940073	2.016003
16	O	−3.521774	2.771143	−0.540289	51	C	4.615421	−3.937069	−0.138409
17	O	−2.693584	−2.975772	−1.388463	52	H	6.113859	−3.268034	−1.535429
18	Al	−1.001373	−2.985766	−0.957621	53	H	3.11129	−4.29212	1.366942
19	O	0.129675	−4.019802	−1.736742	54	H	4.685344	−4.975426	−0.444736
20	H	2.649085	−5.785414	−2.718832	55	C	4.541073	1.231817	−0.126076
21	O	−1.102488	4.588723	−0.368616	56	C	4.99903	2.50699	0.225997
22	H	0.79858	7.159644	−0.559341	57	C	4.157849	0.977871	−1.446287
23	Al	0.540212	4.833962	−0.767258	58	C	5.057611	3.518418	−0.729486
24	Al	1.762496	−3.693035	−2.10825	59	H	5.319703	2.701682	1.245818
25	O	1.279628	6.366693	−0.802927	60	C	4.202531	1.998232	−2.395246
26	O	2.881518	−4.859018	−2.636038	61	H	3.811963	−0.010703	−1.733266
27	Al	−1.88483	3.09953	−0.01803	62	C	4.646044	3.270734	−2.03612
28	O	−2.004973	0.724602	3.97956	63	H	5.412426	4.5051	−0.446607
29	Al	−4.282795	1.345165	−0.925028	64	H	3.864726	1.793575	−3.406232
30	Al	−3.839099	−1.771878	−1.384729	65	H	4.662651	4.068734	−2.772157
31	O	−5.869026	0.998606	−1.386947	66	N	3.231718	0.571337	1.862218
32	O	−5.47657	−1.758488	−1.784794	67	H	4.390688	0.390564	2.655433
33	Al	−2.231928	−0.854481	4.569784	68	H	3.18508	1.593747	1.860834
34	O	−3.110848	−1.173837	5.98597	69	O	5.417761	0.381633	2.157751
35	H	−3.224242	−2.057197	6.340445	70	H	5.890679	−0.459131	2.266884

Cluster D ($[AlO_3]_{clus}$ + Diphenylmethanimine + H_2O)

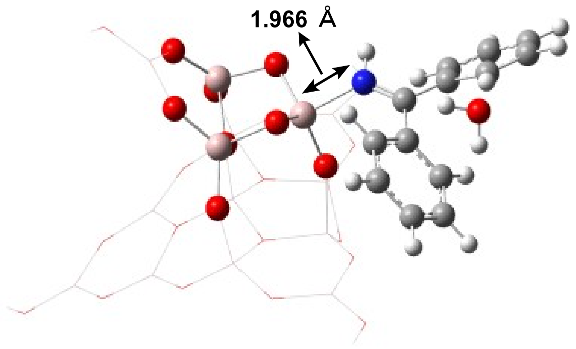

APFD/6-31G(d,p): Thermal Correction to Free Energy = 0.184045 Hartree

APFD/6-311+G(2d,p)//APFD/6-31G(d,p):
Electronic Energy (*EE*) = −5835.502290 Hartree
Gibbs free energy (*G*) = −5835.318245 Hartree

1	O	−1.46612	−1.530619	3.777713	36	Al	−6.968961	−0.413181	−1.26639
2	O	0.55284	0.325021	−2.995991	37	O	−8.626884	−0.61455	−1.510567
3	O	−0.892587	−1.350291	−1.191413	38	H	−9.267179	0.095405	−1.575964
4	O	−1.179668	1.620183	−1.101669	39	O	0.989541	−1.181488	2.155164
5	O	−1.137518	−2.86972	1.125787	40	O	0.683607	1.690344	2.21802
6	O	−1.747739	2.889514	1.346049	41	O	1.169819	0.319563	−0.540592
7	O	1.185945	3.165953	−1.858099	42	Al	1.242952	0.315745	1.237066
8	O	1.717404	−2.390858	−1.985989	43	C	4.156223	0.397313	0.649053
9	O	−1.158767	0.040753	1.269742	44	C	4.197162	−1.008564	0.222272
10	Al	−1.084745	1.668038	2.358025	45	C	4.770645	−1.3577	−1.00721
11	Al	−0.744852	−1.609404	2.228164	46	C	3.683242	−2.011039	1.062608
12	Al	−1.683995	0.054269	−0.46029	47	C	4.812169	−2.690624	−1.400973
13	Al	0.790858	−0.97007	−1.842864	48	H	5.160216	−0.57997	−1.656577
14	Al	0.533415	1.598456	−1.787655	49	C	3.748573	−3.346524	0.670257
15	O	−3.509998	−0.109709	−0.666819	50	H	3.252091	−1.744254	2.021815
16	O	−3.664813	2.828511	−0.703339	51	C	4.313555	−3.689148	−0.557886
17	O	−3.118876	−3.011336	−0.877915	52	H	5.217513	−2.956643	−2.372235
18	Al	−1.393486	−3.045258	−0.611107	53	H	3.361426	−4.117764	1.330633
19	O	−0.381005	−4.219549	−1.353705	54	H	4.337199	−4.724781	−0.881625
20	H	1.967355	−6.208174	−2.333443	55	C	5.253683	1.303863	0.282626
21	O	−1.180703	4.539999	−0.996636	56	C	6.588727	0.881477	0.366115
22	H	0.777325	6.976652	−1.695864	57	C	4.943873	2.600129	−0.159355
23	Al	0.421484	4.6576	−1.576883	58	C	7.605503	1.773843	0.042751
24	Al	1.2168	−4.017299	−1.916744	59	H	6.821247	−0.127953	0.704397
25	O	1.204805	6.138174	−1.879514	60	C	5.969112	3.472971	−0.504131
26	O	2.237896	−5.290383	−2.393093	61	H	3.905726	2.897842	−0.292789
27	Al	−1.973908	3.143926	−0.384695	62	C	7.298424	3.0636	−0.392283
28	O	−1.775428	1.295531	3.879046	63	H	8.641838	1.459154	0.126907
29	Al	−4.507866	1.402169	−0.827436	64	H	5.729748	4.466411	−0.872111
30	Al	−4.217195	−1.764983	−0.921272	65	H	8.098293	3.749959	−0.657323
31	O	−6.142943	1.073954	−1.088961	66	N	3.139535	0.825975	1.330135
32	O	−5.88497	−1.726366	−1.163305	67	H	6.285938	−2.479905	1.771312
33	Al	−1.995494	−0.185231	4.686692	68	H	3.253734	1.780766	1.665407
34	O	−2.739357	−0.284216	6.208842	69	O	7.031536	−2.315967	1.184951
35	H	−2.846554	−1.11016	6.683353	70	H	6.698743	−2.607665	0.328498

Cluster E ([AlO3]$_{clus}$ + Benzophenone + TMSNH2)

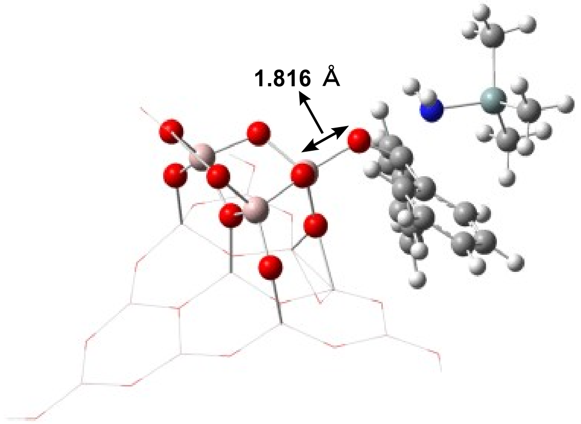

APFD/6-31G(d,p): Thermal Correction to Free Energy = 0.284883 Hartree

APFD/6-311+G(2d,p)//APFD/6-31G(d,p):
Electronic Energy (*EE*) = −6244.056693 Hartree
Gibbs free energy (*G*) = −6243.77181 Hartree

1	O	−1.803331	−1.987032	3.630762	42	Al	0.964076	0.250176	1.347583
2	O	0.17692	0.706423	−2.867435	43	C	3.718051	0.053622	0.592515
3	O	−1.212114	−1.217621	−1.279647	44	O	2.770376	0.338992	1.508844
4	O	−1.613621	1.71014	−0.853977	45	H	4.967206	−0.381942	2.196331
5	O	−1.410132	−3.000449	0.846474	46	N	5.058689	0.30872	1.446105
6	O	−2.2424	2.668662	1.717229	47	H	4.853395	1.20021	1.904816
7	O	0.694604	3.420213	−1.406658	48	C	3.810617	−1.423492	0.200043
8	O	1.439921	−2.061484	−2.163274	49	C	4.622629	−1.877092	−0.845002
9	O	−1.543973	−0.128459	1.321646	50	C	3.098865	−2.360903	0.952271
10	Al	−1.538048	1.365178	2.589286	51	C	4.670776	−3.22809	−1.172979
11	Al	−1.071764	−1.860738	2.089207	52	H	5.19825	−1.173667	−1.436384
12	Al	−2.060607	0.063244	−0.400388	53	C	3.142867	−3.716691	0.620448
13	Al	0.458732	−0.70228	−1.867554	54	H	2.482917	−2.034854	1.786453
14	Al	0.102466	1.83153	−1.521817	55	C	3.92282	−4.154604	−0.444831
15	O	−3.87789	−0.144579	−0.641457	56	H	5.269219	−3.557212	−2.017293
16	O	−4.145354	2.770592	−0.343656	57	H	2.55963	−4.42461	1.203563
17	O	−3.374456	−2.986186	−1.178835	58	H	3.939664	−5.202625	−0.724847
18	Al	−1.650404	−2.985509	−0.901435	59	C	3.73884	1.066566	−0.54782
19	O	−0.589801	−4.028154	−1.763719	60	C	3.906523	2.419626	−0.220528
20	H	1.838179	−5.801811	−2.942011	61	C	3.476499	0.724087	−1.873846
21	O	−1.727429	4.596621	−0.416216	62	C	3.883733	3.400884	−1.204299
22	H	0.138984	7.169206	−0.814168	63	H	4.00562	2.722906	0.82005
23	Al	−0.128041	4.840045	−0.964145	64	C	3.433863	1.709206	−2.859816
24	Al	1.001866	−3.702828	−2.284928	65	H	3.25619	−0.305233	−2.141432
25	O	0.599299	6.373939	−1.088339	66	C	3.656885	3.045343	−2.532157

26	O	2.073538	−4.873704	−2.893917	67	H	3.996452	4.446038	−0.931578
27	Al	−2.469452	3.110835	0.02481	68	H	3.19299	1.427355	−3.880032
28	O	−2.22156	0.795294	4.051254	69	H	3.606849	3.812031	−3.299224
29	Al	−4.932319	1.337138	−0.637749	70	Si	6.846976	0.332126	0.859795
30	Al	−4.519642	−1.785265	−1.089909	71	C	7.622621	1.475717	2.123309
31	O	−6.552233	0.979751	−0.950408	72	H	8.701156	1.545408	1.946585
32	O	−6.186428	−1.781954	−1.341625	73	H	7.215868	2.489941	2.052318
33	Al	−2.388608	−0.775409	4.682414	74	H	7.482362	1.118067	3.148343
34	O	−3.135781	−1.075897	6.17624	75	C	7.448968	−1.423293	1.042065
35	H	−3.213535	−1.954173	6.552298	76	H	6.889248	−2.115281	0.408405
36	Al	−7.319567	−0.507306	−1.304261	77	H	8.506185	−1.478347	0.761343
37	O	−8.967271	−0.741617	−1.585419	78	H	7.367364	−1.765606	2.078974
38	H	−9.634044	−0.053431	−1.575333	79	C	6.977835	1.047946	−0.853107
39	O	0.625036	−1.310937	2.155161	80	H	8.041975	1.112624	−1.108771
40	O	0.252474	1.425445	2.48328	81	H	6.481319	0.451087	−1.619239
41	O	0.772645	0.440974	−0.414407	82	H	6.552454	2.053778	−0.896285

TS III

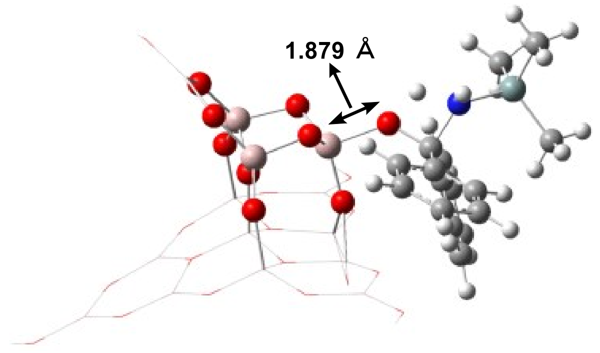

APFD/6-31G(d,p): Thermal Correction to Free Energy = 0.280096 Hartree

APFD/6-311+G(2d,p)//APFD/6-31G(d,p):
Electronic Energy (*EE*) = −6244.024391 Hartree
Gibbs free energy (*G*) = −6243.744295 Hartree

1	O	−1.819387	−1.736047	3.744583	42	Al	0.947375	0.225429	1.317091
2	O	0.220752	0.482353	−2.912683	43	C	3.791318	−0.271226	0.464082
3	O	−1.239118	−1.280869	−1.206149	44	O	2.822984	0.193264	1.418324
4	O	−1.504084	1.681933	−0.949664	45	H	5.206628	−0.799857	1.865837
5	O	−1.497921	−2.926326	1.021758	46	N	4.958597	0.071089	1.394498
6	O	−2.065315	2.815838	1.565193	47	H	3.889143	0.464786	1.987522
7	O	0.873838	3.249951	−1.616381	48	C	3.789477	−1.770694	0.236791

8	O	1.363884	−2.294715	−2.056258	49	C	4.757899	−2.34041	−0.598877
9	O	−1.497569	−0.028477	1.329171	50	C	2.891088	−2.60499	0.901412
10	Al	−1.412569	1.534428	2.507306	51	C	4.766662	−3.710509	−0.834205
11	Al	−1.097041	−1.732991	2.193538	52	H	5.48568	−1.705167	−1.095391
12	Al	−2.020801	0.086264	−0.397712	53	C	2.895301	−3.980708	0.656767
13	Al	0.447829	−0.877292	−1.834061	54	H	2.181707	−2.193443	1.619368
14	Al	0.209532	1.685929	−1.634647	55	C	3.823588	−4.536084	−0.216964
15	O	−3.847751	−0.052096	−0.614354	56	H	5.498305	−4.135827	−1.515048
16	O	−3.980558	2.88461	−0.485656	57	H	2.172388	−4.611672	1.168151
17	O	−3.478101	−2.940116	−0.988054	58	H	3.816723	−5.601139	−0.423987
18	Al	−1.753304	−3.001974	−0.722431	59	C	3.765113	0.58657	−0.786394
19	O	−0.748823	−4.140227	−1.529227	60	C	3.800631	1.978315	−0.619032
20	H	1.585672	−6.08822	−2.617699	61	C	3.629812	0.060667	−2.068909
21	O	−1.48341	4.591146	−0.680575	62	C	3.794046	2.828887	−1.720114
22	H	0.493538	7.048461	−1.240343	63	H	3.786925	2.396158	0.382541
23	Al	0.120243	4.72913	−1.252223	64	C	3.586455	0.91776	−3.171571
24	Al	0.851061	−3.918661	−2.078936	65	H	3.49261	−1.005268	−2.2166
25	O	0.914936	6.218446	−1.470578	66	C	3.699362	2.29845	−3.003155
26	O	1.863132	−5.17059	−2.625455	67	H	3.806249	3.90416	−1.573301
27	Al	−2.287691	3.168876	−0.148652	68	H	3.445292	0.498608	−4.162979
28	O	−2.107672	1.082179	4.004506	69	H	3.660061	2.958693	−3.864081
29	Al	−4.834116	1.473888	−0.690345	70	Si	6.52484	0.977382	1.06948
30	Al	−4.567025	−1.685072	−0.961974	71	C	6.300928	2.80392	1.409413
31	O	−6.471317	1.173212	−0.971003	72	H	7.269736	3.231033	1.691243
32	O	−6.234201	−1.620358	−1.202564	73	H	5.93824	3.347952	0.534905
33	Al	−2.339678	−0.439962	4.727442	74	H	5.610377	2.979774	2.240009
34	O	−3.085917	−0.618795	6.24112	75	C	7.65533	0.21221	2.356432
35	H	−3.199798	−1.469288	6.668354	76	H	7.791592	−0.862087	2.193206
36	Al	−7.308234	−0.295333	−1.232344	77	H	8.644966	0.678335	2.31072
37	O	−8.967349	−0.470172	−1.488565	78	H	7.270717	0.35959	3.370897
38	H	−9.602245	0.247135	−1.514354	79	C	7.142028	0.642965	−0.663289
39	O	0.622234	−1.286594	2.197687	80	H	8.052979	1.228283	−0.831861
40	O	0.377126	1.526634	2.373553	81	H	7.399514	−0.410795	−0.803583
41	O	0.829264	0.3347	−0.453311	82	H	6.410999	0.932456	−1.422055

Cluster F ($[AlO_3]_{clus}$ + *N*−TMS hemiaminal−$OH_{adsorption}$)

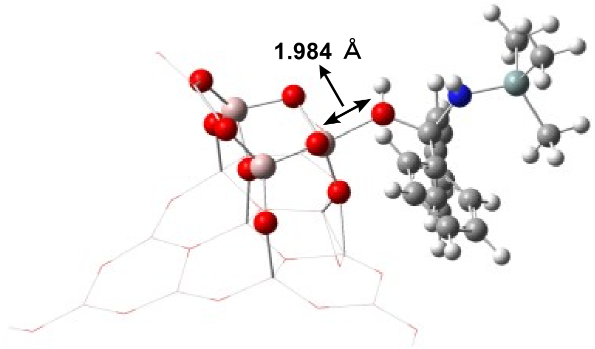

APFD/6-31G(d,p): Thermal Correction to Free Energy = 0.283995 Hartree

APFD/6-311+G(2d,p)//APFD/6-31G(d,p):
Electronic Energy (*EE*) = −6244.058048 Hartree
Gibbs free energy (*G*) = −6243.774053 Hartree

1	O	−1.786462	−1.871551	3.67215	42	Al	0.789088	0.118599	1.235732
2	O	0.142241	0.541604	−2.950496	43	C	3.879056	−0.085258	0.560171
3	O	−1.307971	−1.256309	−1.272157	44	O	2.755422	0.244681	1.471143
4	O	−1.519436	1.69971	−0.909635	45	H	4.999302	−0.666967	2.134337
5	O	−1.544196	−2.972821	0.904047	46	N	5.064069	0.002155	1.371996
6	O	−2.006861	2.75544	1.654167	47	H	2.984033	1.051711	1.956123
7	O	0.868213	3.252895	−1.57631	48	C	3.714429	−1.551093	0.168506
8	O	1.25902	−2.28036	−2.21453	49	C	4.389984	−2.070237	−0.944808
9	O	−1.490368	−0.087527	1.309278	50	C	3.011237	−2.439316	1.010976
10	Al	−1.35429	1.432686	2.537422	51	C	4.264015	−3.408986	−1.273971
11	Al	−1.098453	−1.826554	2.106215	52	H	4.979556	−1.417371	−1.576329
12	Al	−2.049542	0.094225	−0.400503	53	C	2.879299	−3.785879	0.667848
13	Al	0.371042	−0.857459	−1.923901	54	H	2.552045	−2.083106	1.925499
14	Al	0.17854	1.700802	−1.632528	55	C	3.498075	−4.276207	−0.473007
15	O	−3.882808	−0.008563	−0.580483	56	H	4.739375	−3.791113	−2.172016
16	O	−3.965395	2.923718	−0.349503	57	H	2.304188	−4.441927	1.314699
17	O	−3.568071	−2.887452	−1.059951	58	H	3.401145	−5.318784	−0.756493
18	Al	−1.83907	−2.984847	−0.835506	59	C	3.886979	0.933702	−0.565804
19	O	−0.871058	−4.110141	−1.702793	60	C	4.242226	2.24898	−0.243875
20	H	1.407224	−6.055406	−2.908899	61	C	3.464991	0.646616	−1.865902
21	O	−1.445953	4.597358	−0.542769	62	C	4.226041	3.247534	−1.211199
22	H	0.557559	7.041717	−1.063449	63	H	4.527365	2.495785	0.775389
23	Al	0.146753	4.730158	−1.145408	64	C	3.454621	1.643911	−2.836712
24	Al	0.719711	−3.894511	−2.280598	65	H	3.12341	−0.349711	−2.127416
25	O	0.960355	6.213685	−1.331082	66	C	3.842506	2.943455	−2.514905
26	O	1.699202	−5.142418	−2.891842	67	H	4.489957	4.26635	−0.942724

27	Al	−2.261162	3.170253	−0.041301	68	H	3.100297	1.40658	−3.834833
28	O	−2.023492	0.940234	4.033739	69	H	3.809629	3.724505	−3.268202
29	Al	−4.845879	1.5341	−0.582537	70	Si	6.763954	0.39107	0.97257
30	Al	−4.635785	−1.617454	−0.966878	71	C	7.179149	2.168204	1.424538
31	O	−6.493491	1.268384	−0.83625	72	H	8.267051	2.26295	1.517058
32	O	−6.30659	−1.518946	−1.167513	73	H	6.846736	2.875572	0.66105
33	Al	−2.264046	−0.601995	4.709787	74	H	6.737108	2.455233	2.383383
34	O	−2.979595	−0.821001	6.232975	75	C	7.715822	−0.774582	2.096117
35	H	−3.097746	−1.683757	6.633636	76	H	7.502663	−1.821345	1.85707
36	Al	−7.359519	−0.177335	−1.128221	77	H	8.793163	−0.6188	1.980199
37	O	−9.026471	−0.317801	−1.352764	78	H	7.475569	−0.608044	3.15173
38	H	−9.650135	0.409637	−1.339948	79	C	7.183595	0.085731	−0.828014
39	O	0.634639	−1.435778	2.056115	80	H	8.195171	0.461118	−1.018948
40	O	0.42253	1.411608	2.365182	81	H	7.173654	−0.980153	−1.070506
41	O	0.81131	0.293271	−0.513377	82	H	6.501913	0.611754	−1.501934

Cluster G ($[AlO_3]_{clus}$ + *N*−TMS hemiaminal−NHTMS$_{adsorption}$)

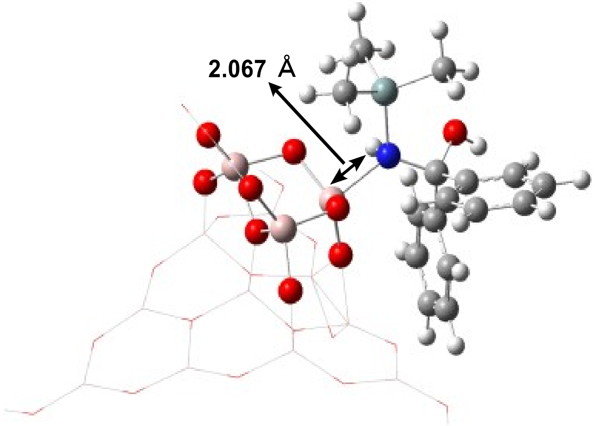

APFD/6-31G(d,p): Thermal Correction to Free Energy = 0.286368 Hartree

APFD/6-311+G(2d,p)//APFD/6-31G(d,p):
Electronic Energy (*EE*) = −6244.060147 Hartree
Gibbs free energy (*G*) = −6243.773779 Hartree

1	O	−0.897386	−1.354603	3.812832	42	Al	1.215933	0.362269	0.724119
2	O	−0.169005	0.035438	−3.32439	43	C	4.263974	0.591215	−0.176027
3	O	−1.198174	−1.54393	−1.179899	44	O	5.219177	1.651684	−0.086044
4	O	−1.610433	1.41225	−1.252155	45	H	5.693949	1.706387	−0.922894
5	O	−0.965555	−2.884839	1.246193	46	N	3.186632	0.983165	0.768096
6	O	−1.809635	2.848394	1.160944	47	C	3.744373	0.575047	−1.614597

7	O	0.510979	2.977664	−2.51975	48	C	3.570791	−0.61878	−2.334784
8	O	1.283589	−2.549475	−2.344338	49	C	3.463543	1.805993	−2.237709
9	O	−1.104005	0.025331	1.184776	50	C	3.09097	−0.584145	−3.634376
10	Al	−0.923248	1.734523	2.124932	51	H	3.769673	−1.572651	−1.862177
11	Al	−0.450929	−1.52874	2.170306	52	C	2.97362	1.840667	−3.531618
12	Al	−1.919502	−0.115615	−0.422757	53	H	3.602606	2.734724	−1.691932
13	Al	0.327199	−1.155922	−2.141993	54	C	2.768151	0.64053	−4.232089
14	Al	−0.042194	1.398158	−2.224841	55	H	2.920761	−1.514772	−4.163565
15	O	−3.743539	−0.360545	−0.293451	56	H	2.712318	2.789456	−3.985099
16	O	−4.04585	2.55834	−0.51061	57	H	2.35764	0.663045	−5.236482
17	O	−3.253202	−3.25392	−0.363501	58	C	4.887779	−0.748258	0.19261
18	Al	−1.50808	−3.205035	−0.40192	59	C	4.143803	−1.76941	0.789754
19	O	−0.582496	−4.396921	−1.225464	60	C	6.225984	−0.981136	−0.14138
20	H	1.656332	−6.370877	−2.458531	61	C	4.741415	−2.999396	1.057994
21	O	−1.73616	4.329402	−1.356001	62	H	3.104961	−1.618662	1.072122
22	H	−0.049457	6.772277	−2.559107	63	C	6.817671	−2.21086	0.123472
23	Al	−0.265617	4.458438	−2.215288	64	H	6.819668	−0.191774	−0.591567
24	Al	0.882561	−4.182359	−2.073091	65	C	6.07587	−3.226787	0.725046
25	O	0.380475	5.937824	−2.754968	66	H	4.155684	−3.780666	1.534795
26	O	1.867332	−5.451491	−2.629704	67	H	7.860177	−2.374734	−0.13499
27	Al	−2.342814	2.958094	−0.516863	68	H	6.53659	−4.187935	0.935844
28	O	−1.322459	1.457901	3.766097	69	H	2.93594	1.929015	0.461029
29	Al	−4.82689	1.097394	−0.383304	70	Si	3.755963	1.352077	2.53878
30	Al	−4.402186	−2.054924	−0.301746	71	C	5.502225	0.741108	2.837497
31	O	−6.464537	0.691365	−0.329856	72	H	6.205042	1.095453	2.083028
32	O	−6.086582	−2.095132	−0.249444	73	H	5.808864	1.138451	3.813046
33	Al	−1.327378	0.038082	4.702822	74	H	5.556676	−0.348767	2.887905
34	O	−1.792091	0.032151	6.335024	75	C	2.649576	0.532935	3.803652
35	H	−1.775394	−0.757364	6.878369	76	H	1.684821	1.038709	3.878918
36	Al	−7.235035	−0.833716	−0.253595	77	H	2.46761	−0.516505	3.553901
37	O	−8.898369	−1.11283	−0.188101	78	H	3.158109	0.582298	4.773507
38	H	−9.5743	−0.433566	−0.190315	79	C	3.664602	3.218639	2.616816
39	O	1.207094	−1.131405	1.662644	80	H	2.641165	3.54807	2.414606
40	O	0.78407	1.813958	1.641668	81	H	3.945451	3.573024	3.613883
41	O	0.88942	0.257087	−1.028439	82	H	4.344197	3.673971	1.889891

TS IV

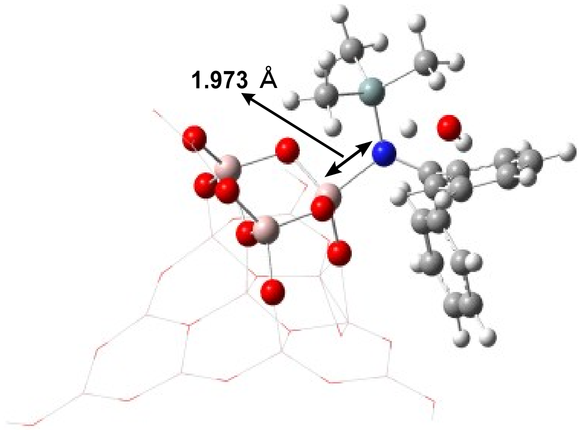

APFD/6-31G(d,p): Thermal Correction to Free Energy = 0.280465 Hartree

APFD/6-311+G(2d,p)//APFD/6-31G(d,p):
Electronic Energy (*EE*) = −6243.984470 Hartree
Gibbs free energy (*G*) = −6243.704005 Hartree

1	O	−0.808208	−1.073775	3.876867	42	Al	1.280158	0.570917	0.610992
2	O	−0.268371	−0.129274	−3.349417	43	C	4.147206	0.461024	−0.232698
3	O	−1.188577	−1.605974	−1.085568	44	O	5.012283	1.868874	−0.316169
4	O	−1.732422	1.318481	−1.342419	45	H	5.114557	2.142029	−1.243891
5	O	−0.853996	−2.772087	1.417691	46	N	3.193664	1.048112	0.65604
6	O	−1.952254	2.901338	0.975225	47	C	3.706639	0.277901	−1.669658
7	O	0.294912	2.886267	−2.752976	48	C	3.905272	−0.939108	−2.329568
8	O	1.314258	−2.57881	−2.232562	49	C	3.130797	1.355201	−2.370743
9	O	−1.122116	0.118988	1.169033	50	C	3.527496	−1.083456	−3.661329
10	Al	−1.00055	1.892857	1.991536	51	H	4.325968	−1.781484	−1.793078
11	Al	−0.383603	−1.336922	2.240406	52	C	2.775516	1.215836	−3.702401
12	Al	−1.958882	−0.162992	−0.408743	53	H	2.892864	2.289244	−1.867979
13	Al	0.300884	−1.217312	−2.102124	54	C	2.967547	−0.011478	−4.35098
14	Al	−0.182543	1.307438	−2.344138	55	H	3.645732	−2.04653	−4.146209
15	O	−3.767725	−0.476991	−0.22623	56	H	2.305038	2.047491	−4.216283
16	O	−4.20266	2.405418	−0.627465	57	H	2.65635	−0.12989	−5.384312
17	O	−3.151186	−3.344903	−0.117062	58	C	5.031663	−0.630389	0.283808
18	Al	−1.410887	−3.223711	−0.194464	59	C	4.442986	−1.631085	1.061306
19	O	−0.448192	−4.42662	−0.95719	60	C	6.381096	−0.706003	−0.05975
20	H	1.853611	−6.379829	−2.104352	61	C	5.220537	−2.698373	1.506264
21	O	−1.988893	4.214254	−1.634124	62	H	3.386581	−1.572225	1.317023
22	H	−0.433452	6.642349	−3.028765	63	C	7.15493	−1.76596	0.402217
23	Al	−0.540925	4.349201	−2.530059	64	H	6.828435	0.077075	−0.664652
24	Al	0.990719	−4.205877	−1.84697	65	C	6.575231	−2.763964	1.185308
25	O	0.029443	5.816011	−3.178458	66	H	4.763852	−3.47639	2.111595

26	O	2.020672	−5.465575	−2.339559	67	H	8.210969	−1.813156	0.151528
27	Al	−2.519353	2.876705	−0.694847	68	H	7.179945	−3.592601	1.543393
28	O	−1.358045	1.708591	3.655133	69	H	3.970825	2.110461	0.119627
29	Al	−4.915932	0.92389	−0.388902	70	Si	3.694256	1.52298	2.378329
30	Al	−4.350814	−2.19494	−0.110206	71	C	5.50168	1.141226	2.736658
31	O	−6.532812	0.452283	−0.27537	72	H	6.176386	1.499062	1.956173
32	O	−6.030594	−2.303953	−0.02077	73	H	5.750847	1.671922	3.66382
33	Al	−1.283582	0.355005	4.682568	74	H	5.690484	0.077151	2.89445
34	O	−1.718639	0.437177	6.320875	75	C	2.659022	0.707342	3.710135
35	H	−1.657425	−0.313186	6.914209	76	H	1.657757	1.140767	3.752876
36	Al	−7.233619	−1.096106	−0.083756	77	H	2.560091	−0.367514	3.531476
37	O	−8.881559	−1.441501	0.034025	78	H	3.156309	0.866499	4.673725
38	H	−9.586811	−0.793553	0.001311	79	C	3.467456	3.386019	2.413141
39	O	1.241987	−0.911771	1.624332	80	H	2.447987	3.635723	2.109904
40	O	0.706897	1.985924	1.521146	81	H	3.628201	3.759515	3.430247
41	O	0.806562	0.287998	−1.086042	82	H	4.179167	3.901239	1.758621

Cluster H ($[AlO_3]_{clus}$ + *N*−TMS diphenylmethanimine + H_2O)

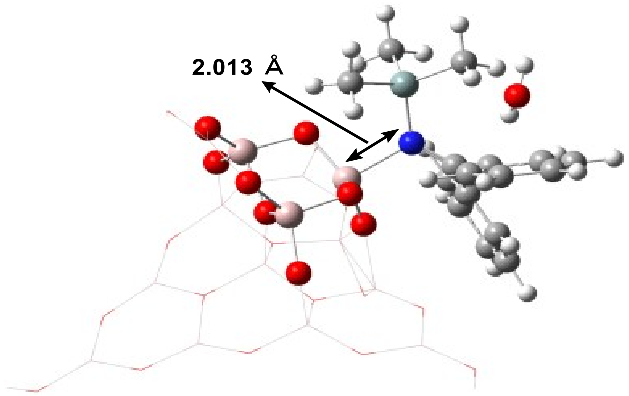

APFD/6-31G(d,p): Thermal Correction to Free Energy = 0.277816 Hartree

APFD/6-311+G(2d,p)//APFD/6-31G(d,p):
Electronic Energy (*EE*) = −6244.057447 Hartree
Gibbs free energy (*G*) = −6243.779631 Hartree

1	O	−0.860343	−1.894411	3.544938	42	Al	1.103005	0.396325	0.764474
2	O	−0.248347	0.637178	−3.282888	43	C	3.83668	0.050799	−0.04984
3	O	−1.177936	−1.313372	−1.416434	44	O	5.965944	2.193223	−0.151453
4	O	−1.751284	1.590399	−1.024562	45	H	5.452723	2.561581	−0.87886
5	O	−0.859091	−3.003925	0.769531	46	N	3.105623	0.526089	0.929924
6	O	−2.015218	2.616607	1.583592	47	C	3.649729	0.54046	−1.414396

7	O	0.274511	3.449266	−2.023801	48	C	3.873633	−0.294604	−2.524615
8	O	1.348527	−1.986118	−2.716659	49	C	3.223115	1.872141	−1.614522
9	O	−1.156472	−0.132231	1.165733	50	C	3.62504	0.18001	−3.804748
10	Al	−1.064058	1.415378	2.363341	51	H	4.162463	−1.327482	−2.370479
11	Al	−0.414132	−1.783687	1.896855	52	C	3.019095	2.353122	−2.895141
12	Al	−1.971933	−0.063377	−0.446517	53	H	3.032086	2.498476	−0.747712
13	Al	0.318652	−0.69613	−2.30084	54	C	3.201006	1.496952	−3.991343
14	Al	−0.190011	1.815153	−1.982478	55	H	3.728002	−0.486954	−4.654438
15	O	−3.779145	−0.425084	−0.362999	56	H	2.652218	3.363169	−3.040286
16	O	−4.241476	2.470692	−0.119746	57	H	2.992044	1.859411	−4.993724
17	O	−3.13205	−3.240298	−0.885296	58	C	4.804005	−1.036154	0.175662
18	Al	−1.392434	−3.090501	−0.910108	59	C	4.385254	−2.1226	0.958118
19	O	−0.407756	−4.085747	−1.907813	60	C	6.083647	−1.020847	−0.397211
20	H	1.928601	−5.715935	−3.428742	61	C	5.254681	−3.187015	1.170033
21	O	−2.036576	4.476338	−0.669125	62	H	3.370767	−2.125928	1.352819
22	H	−0.492361	7.166508	−1.468155	63	C	6.95358	−2.076444	−0.152605
23	Al	−0.580036	4.819065	−1.492867	64	H	6.398491	−0.1538	−0.968644
24	Al	1.038704	−3.660816	−2.706617	65	C	6.539105	−3.159728	0.624224
25	O	−0.018625	6.397986	−1.791361	66	H	4.930387	−4.039581	1.760007
26	O	2.08817	−4.770918	−3.452662	67	H	7.957037	−2.057321	−0.568431
27	Al	−2.562852	2.959114	−0.05779	68	H	7.219312	−3.988754	0.800824
28	O	−1.438505	0.862975	3.939397	69	H	6.46496	2.932944	0.208058
29	Al	−4.940917	0.967336	−0.226026	70	Si	3.730256	0.922866	2.59173
30	Al	−4.344388	−2.13062	−0.639492	71	C	5.515165	0.398718	2.821823
31	O	−6.553648	0.468895	−0.242156	72	H	6.130572	0.723905	1.979589
32	O	−6.023766	−2.270716	−0.599417	73	H	5.887255	0.890964	3.72841
33	Al	−1.360765	−0.684462	4.641363	74	H	5.635577	−0.679734	2.945591
34	O	−1.815436	−0.972124	6.250898	75	C	2.632041	0.151727	3.899056
35	H	−1.75268	−1.835067	6.663483	76	H	1.665101	0.656046	3.950548
36	Al	−7.239345	−1.089108	−0.40889	77	H	2.46274	−0.903233	3.662033
37	O	−8.884584	−1.4657	−0.393262	78	H	3.128708	0.231612	4.872145
38	H	−9.596575	−0.832564	−0.290823	79	C	3.627844	2.797356	2.57532
39	O	1.211202	−1.274456	1.383265	80	H	2.590987	3.098243	2.408056
40	O	0.636193	1.651342	1.907497	81	H	3.971557	3.200882	3.533662
41	O	0.790359	0.550905	−0.971954	82	H	4.264829	3.19312	1.77906

I (Benzophenone + NH_3)

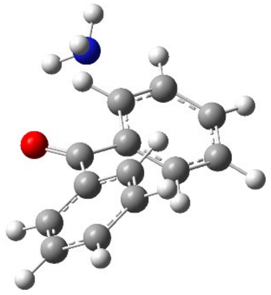

APFD/6-31G(d,p): Thermal Correction to Free Energy = 0.187004 Hartree

APFD/6-311+G(2d,p)//APFD/6-31G(d,p):
Electronic Energy (*EE*) = −632.847394 Hartree
Gibbs free energy (*G*) = −632.66039 Hartree

1	C	0.008767	0.747047	−0.69669	15	H	−2.85707	−1.62184	2.138532
2	O	0.015647	1.828546	−1.2709	16	H	−4.44484	−0.16093	−1.5812
3	H	−0.08836	2.671387	1.066989	17	H	−4.75313	−1.39031	0.553977
4	N	0.052341	2.172205	1.94014	18	C	1.28967	0.053194	−0.38196
5	H	−0.76288	2.363974	2.513415	19	C	2.455745	0.825485	−0.32669
6	H	0.842703	2.611156	2.401307	20	C	1.373547	−1.33213	−0.20851
7	C	−1.2901	0.108173	−0.33518	21	C	3.68215	0.227411	−0.0713
8	C	−1.46876	−0.57432	0.873257	22	H	2.370116	1.89551	−0.49015
9	C	−2.3714	0.272497	−1.20642	23	C	2.606463	−1.93412	0.032973
10	C	−2.7156	−1.10446	1.193166	24	H	0.476134	−1.93929	−0.2808
11	H	−0.63943	−0.63783	1.570583	25	C	3.759042	−1.15551	0.110354
12	C	−3.61067	−0.27587	−0.89404	26	H	4.582653	0.833645	−0.01705
13	H	−2.21875	0.833529	−2.12395	27	H	2.667039	−3.01194	0.15818
14	C	−3.78311	−0.96669	0.306067	28	H	4.719718	−1.62509	0.306206

TS V

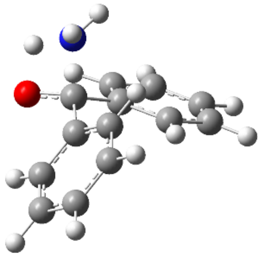

APFD/6-31G(d,p): Thermal Correction to Free Energy = 0.188052 Hartree

APFD/6-311+G(2d,p)//APFD/6-31G(d,p):
Electronic Energy (*EE*) = −632.788758 Hartree
Gibbs free energy (*G*) = −632.600706 Hartree

1	C	0.034342	1.031481	0.158535	15	H	−3.375977	−1.920495	1.58112
2	O	0.087524	2.212582	−0.479727	16	H	−3.821395	0.036967	−2.21959
3	H	0.095074	2.553355	0.878919	17	H	−4.68177	−1.531283	−0.495567
4	N	0.095451	1.66471	1.637716	18	C	1.277005	0.146949	−0.01179
5	H	−0.724043	1.520495	2.224885	19	C	2.450888	0.79514	−0.404887
6	H	0.956393	1.47907	2.147771	20	C	1.301252	−1.231909	0.209465
7	C	−1.288062	0.282278	0.034298	21	C	3.638052	0.083309	−0.547583
8	C	−1.779776	−0.59459	1.005514	22	H	2.387004	1.8583	−0.619043
9	C	−2.034467	0.510973	−1.121018	23	C	2.489288	−1.946193	0.061629
10	C	−2.998842	−1.244665	0.817691	24	H	0.389092	−1.75768	0.473971
11	H	−1.211214	−0.778432	1.916369	25	C	3.661497	−1.290866	−0.310167
12	C	−3.246729	−0.145143	−1.314947	26	H	4.544384	0.598088	−0.857094
13	H	−1.64361	1.226426	−1.83823	27	H	2.495666	−3.02047	0.228782
14	C	−3.731745	−1.024613	−0.346887	28	H	4.585826	−1.850872	−0.427618

J (Hemiaminal: type 1)

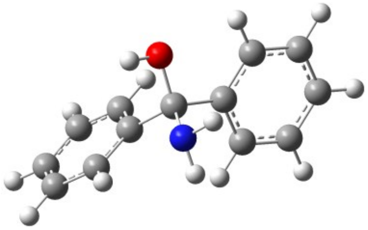

APFD/6-31G(d,p): Thermal Correction to Free Energy = 0.193317 Hartree

APFD/6-311+G(2d,p)//APFD/6-31G(d,p):
Electronic Energy (*EE*) = −632.846982 Hartree
Gibbs free energy (*G*) = −632.653665 Hartree

1	C	−0.004625	1.128001	−0.027069	15	H	−4.415975	0.09824	1.252723
2	O	0.053892	1.893699	−1.215319	16	H	−2.52429	−2.459701	−1.637056
3	H	−0.718145	2.472	−1.185156	17	H	−4.506119	−1.874612	−0.253877
4	N	−0.046579	2.067782	1.09532	18	C	1.257205	0.271267	0.012783
5	H	−0.044397	1.554091	1.972831	19	C	2.331722	0.514727	−0.842576
6	H	0.818165	2.602299	1.072011	20	C	1.370535	−0.73402	0.979136
7	C	−1.269038	0.266573	−0.030128	21	C	3.503891	−0.233019	−0.728646
8	C	−2.390455	0.602589	0.728507	22	H	2.239199	1.287479	−1.598083
9	C	−1.332032	−0.83702	−0.887425	23	C	2.539382	−1.478437	1.093865
10	C	−3.55055	−0.169286	0.65156	24	H	0.525776	−0.944613	1.632053
11	H	−2.354618	1.478205	1.369697	25	C	3.613231	−1.228573	0.238539
12	C	−2.489161	−1.602638	−0.969374	26	H	4.333499	−0.036864	−1.403431
13	H	−0.461246	−1.090676	−1.485941	27	H	2.611253	−2.259561	1.846439
14	C	−3.602557	−1.27329	−0.194302	28	H	4.526525	−1.811601	0.323884

K (Hemiaminal: type 2)

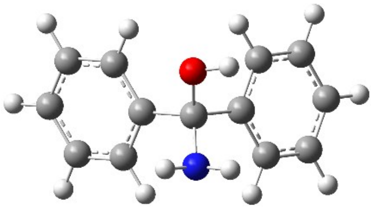

APFD/6-31G(d,p): Thermal Correction to Free Energy = 0.194138 Hartree

APFD/6-311+G(2d,p)//APFD/6-31G(d,p):
Electronic Energy (*EE*) = −632.849457 Hartree
Gibbs free energy (*G*) = −632.655319 Hartree

1	C	0.005215	1.21557	−0.089337	15	C	1.686556	−0.309896	−1.200938
2	C	−1.239514	0.327511	−0.017975	16	C	3.045871	−0.71469	1.191581
3	C	−1.711961	−0.094363	1.22838	17	H	1.601036	0.637085	2.052847
4	C	−1.889587	−0.107763	−1.175088	18	C	2.79487	−1.151105	−1.163729
5	C	−2.826131	−0.92399	1.317268	19	H	1.149803	−0.140934	−2.12939
6	H	−1.193189	0.226927	2.127059	20	C	3.478651	−1.357036	0.033606
7	C	−3.006223	−0.936265	−1.084932	21	H	3.575441	−0.866667	2.128835
8	H	−1.511207	0.20788	−2.142436	22	H	3.125343	−1.647442	−2.072739
9	C	−3.479436	−1.34545	0.160069	23	H	4.344052	−2.014153	0.063272
10	H	−3.18359	−1.24445	2.292587	24	N	−0.00372	1.951003	−1.334701
11	H	−3.506946	−1.264443	−1.992364	25	H	−0.835306	2.532009	−1.389972
12	H	−4.349534	−1.993146	0.228719	26	H	0.808603	2.559332	−1.360919
13	C	1.251821	0.33735	−0.041649	27	O	0.0673	2.064321	1.059366
14	C	1.936334	0.127439	1.156271	28	H	−0.815665	2.43255	1.181315

TS VI

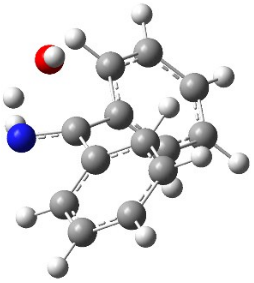

APFD/6-31G(d,p): Thermal Correction to Free Energy = 0.186689 Hartree

APFD/6-311+G(2d,p)//APFD/6-31G(d,p):
Electronic Energy (*EE*) = −632.771540 Hartree
Gibbs free energy (*G*) = −632.584851 Hartree

1	C	0.018578	0.913399	−0.304754	15	C	1.368954	−1.178755	−0.604099
2	C	−1.267314	0.197446	−0.109803	16	C	3.633958	0.110387	0.388602

3	C	−1.412549	−0.757202	0.906021	17	H	2.321126	1.798234	0.718714
4	C	−2.342081	0.47052	−0.959193	18	C	2.589455	−1.849315	−0.560918
5	C	−2.61937	−1.424876	1.069267	19	H	0.482632	−1.673832	−0.990096
6	H	−0.575667	−0.953947	1.570589	20	C	3.72159	−1.20755	−0.064253
7	C	−3.551473	−0.201249	−0.793415	21	H	4.511803	0.605804	0.794793
8	H	−2.202476	1.208161	−1.743435	22	H	2.654018	−2.874967	−0.914171
9	C	−3.692249	−1.148711	0.217951	23	H	4.671116	−1.734765	−0.022391
10	H	−2.728757	−2.159605	1.862567	24	N	−0.011483	2.065616	−1.017123
11	H	−4.383499	0.01255	−1.459155	25	H	−0.27323	2.578487	0.054968
12	H	−4.635216	−1.67421	0.345013	26	H	0.931842	2.328811	−1.290795
13	C	1.282357	0.146641	−0.166553	27	O	−0.091432	2.115056	1.281327
14	C	2.422437	0.78833	0.332316	28	H	−0.875616	2.00514	1.83562

L (Diphenylmethanimine + H_2O)

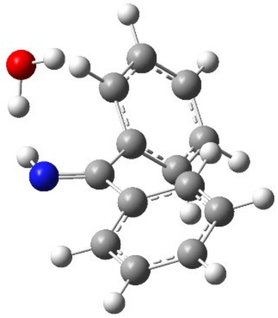

APFD/6-31G(d,p): Thermal Correction to Free Energy = 0.187883 Hartree

APFD/6-311+G(2d,p)//APFD/6-31G(d,p):
Electronic Energy (*EE*) = −632.840685 Hartree
Gibbs free energy (*G*) = −632.652802 Hartree

1	C	−0.04002	0.623284	−0.663992	15	C	1.449563	−1.412274	−0.678019
2	C	−1.322031	0.00145	−0.253718	16	C	3.481778	0.058941	0.543041
3	C	−1.37378	−0.97199	0.752033	17	H	2.063501	1.69752	0.559862
4	C	−2.514568	0.421623	−0.859764	18	C	2.680532	−2.007682	−0.418671
5	C	−2.594817	−1.514584	1.144738	19	H	0.65168	−1.979634	−1.149873
6	H	−0.455343	−1.293995	1.23403	20	C	3.696729	−1.273766	0.193538
7	C	−3.730582	−0.124821	−0.470488	21	H	4.267289	0.626992	1.034358
8	H	−2.458728	1.178692	−1.636023	22	H	2.847683	−3.045917	−0.692964
9	C	−3.773972	−1.095035	0.532986	23	H	4.654294	−1.743363	0.403006
10	H	−2.624547	−2.26439	1.930772	24	N	−0.070438	1.768781	−1.246071
11	H	−4.64899	0.201714	−0.95119	25	H	−0.069787	3.160879	0.247317
12	H	−4.726263	−1.523045	0.83537	26	H	0.880221	2.045549	−1.499888
13	C	1.237704	−0.068774	−0.347787	27	O	0.421028	3.469775	1.024709
14	C	2.258876	0.665646	0.269583	28	H	0.011358	2.987216	1.749168

M (Benzophenone + $TMSNH_2$)

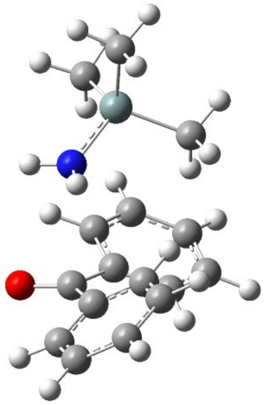

APFD/6-31G(d,p): Thermal Correction to Free Energy = 0.279594 Hartree

APFD/6-311+G(2d,p)//APFD/6-31G(d,p):
Electronic Energy (*EE*) = −1041.409195 Hartree
Gibbs free energy (*G*) = −1041.129601 Hartree

1	C	1.073798	0.950994	−1.095966	21	H	−1.345909	1.485398	−1.970858
2	O	1.081034	0.960845	−2.31787	22	C	−0.770127	2.960941	1.561833
3	H	0.287933	−1.971384	−0.640724	23	H	1.209484	2.130307	1.402521
4	N	−0.583477	−1.547844	−0.924953	24	C	−2.006455	3.124371	0.941161
5	H	−0.627062	−1.473328	−1.93066	25	H	−3.183532	2.731823	−0.823019
6	C	2.13791	0.209815	−0.351756	26	H	−0.59259	3.388449	2.544972
7	C	1.916317	−0.372572	0.902205	27	H	−2.800281	3.669184	1.445519
8	C	3.373608	0.034048	−0.983049	28	Si	−2.042008	−1.81727	−0.014537
9	C	2.923302	−1.110533	1.51848	29	C	−3.451351	−1.08495	−1.021132
10	H	0.943687	−0.270987	1.37311	30	H	−4.411003	−1.262591	−0.524779
11	C	4.385074	−0.687276	−0.358439	31	H	−3.326392	−0.004789	−1.134325
12	H	3.515291	0.469871	−1.967701	32	H	−3.508619	−1.531688	−2.019398
13	C	4.161117	−1.260988	0.893753	33	C	−2.385163	−3.64479	0.311346
14	H	2.741432	−1.571319	2.485934	34	H	−1.556096	−4.114309	0.852295
15	H	5.347399	−0.809209	−0.848568	35	H	−3.289917	−3.778947	0.914962
16	H	4.949114	−1.830754	1.379579	36	H	−2.522047	−4.192517	−0.626874
17	C	0.023979	1.699314	−0.344991	37	C	−1.895127	−0.932527	1.640101
18	C	−1.208	1.899228	−0.976959	38	H	−2.812112	−1.04633	2.228565
19	C	0.242518	2.247894	0.922339	39	H	−1.07379	−1.337898	2.241773
20	C	−2.222363	2.596153	−0.333981	40	H	−1.715055	0.13621	1.489163

TS VII

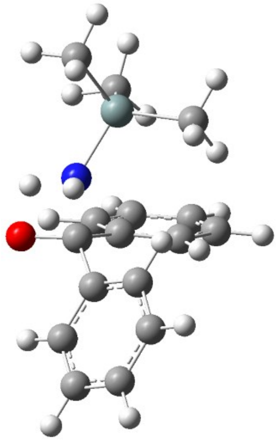

APFD/6-31G(d,p): Thermal Correction to Free Energy = 0.282136 Hartree

APFD/6-311+G(2d,p)//APFD/6-31G(d,p):
Electronic Energy (*EE*) = −1041.350392 Hartree
Gibbs free energy (*G*) = −1041.068256 Hartree

1	C	−0.474831	0.06573	0.922214	21	H	0.969961	1.861636	2.149429
2	O	−0.429775	0.080875	2.267435	22	C	1.030851	2.497376	−1.665056
3	H	−0.326551	−2.055384	0.587429	23	H	−0.348618	0.855074	−1.781325
4	N	0.350759	−1.308836	0.752393	24	C	1.787289	3.318503	−0.828533
5	H	0.294917	−1.044633	1.89991	25	H	2.334436	3.756698	1.208992
6	C	−1.863021	−0.142788	0.323946	26	H	1.022163	2.673035	−2.738009
7	C	−2.101473	−0.807633	−0.881816	27	H	2.376906	4.129314	−1.248926
8	C	−2.941018	0.37135	1.045043	28	Si	1.921118	−1.648855	−0.098579
9	C	−3.401267	−0.94301	−1.368776	29	C	3.184493	−0.353272	0.358161
10	H	−1.272283	−1.231584	−1.445635	30	H	4.186062	−0.720644	0.108178
11	C	−4.237635	0.244745	0.556065	31	H	3.01432	0.584526	−0.175797
12	H	−2.72937	0.847899	1.997475	32	H	3.159472	−0.137626	1.430003
13	C	−4.47152	−0.411181	−0.653089	33	C	2.408004	−3.336433	0.56687
14	H	−3.576918	−1.467603	−2.304735	34	H	1.653691	−4.097673	0.342241
15	H	−5.071569	0.649773	1.12394	35	H	3.352348	−3.666144	0.120885
16	H	−5.48517	−0.515367	−1.03137	36	H	2.543512	−3.304435	1.65243
17	C	0.2793	1.219762	0.249297	37	C	1.6284	−1.732246	−1.948817
18	C	1.013305	2.062972	1.083225	38	H	2.550483	−2.035663	−2.456773
19	C	0.269477	1.462156	−1.126799	39	H	0.855402	−2.463263	−2.20739
20	C	1.764388	3.106939	0.549393	40	H	1.335599	−0.756938	−2.345034

N (*N*−TMS hemiaminal: type 1)

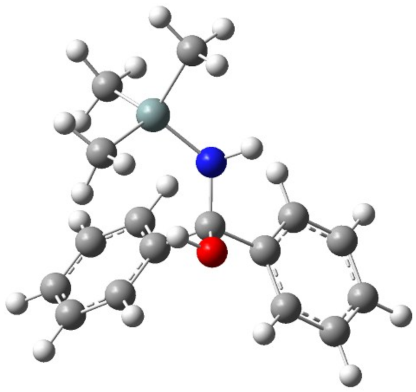

APFD/6-31G(d,p): Thermal Correction to Free Energy = 0.287227 Hartree

APFD/6-311+G(2d,p)//APFD/6-31G(d,p):
Electronic Energy (*EE*) = −1041.409195 Hartree
Gibbs free energy (*G*) = −1041.121968 Hartree

1	C	0.309422	−0.340023	−0.590829	21	H	0.06236	1.887241	−2.160503
2	O	0.275924	−0.476523	−2.011942	22	C	−0.573522	2.692105	1.568338
3	H	−0.19648	−2.221264	−0.050756	23	H	−0.184945	0.600499	1.912385
4	N	−0.563954	−1.282682	0.065726	24	C	−0.672865	3.707446	0.616913
5	H	−0.63916	−0.326353	−2.276323	25	H	−0.51154	4.201103	−1.47332
6	C	1.765377	−0.586186	−0.197641	26	H	−0.736014	2.912772	2.620322
7	C	2.086355	−1.138978	1.043053	27	H	−0.916129	4.721556	0.922847
8	C	2.795753	−0.201298	−1.059129	28	Si	−2.325669	−1.243538	0.085465
9	C	3.417964	−1.313021	1.415687	29	C	−3.032876	−0.35109	−1.420008
10	H	1.284966	−1.435311	1.71363	30	H	−4.123863	−0.292433	−1.336905
11	C	4.125115	−0.376229	−0.686916	31	H	−2.660524	0.676462	−1.489898
12	H	2.544279	0.226006	−2.023916	32	H	−2.811181	−0.87778	−2.355414
13	C	4.44194	−0.932458	0.552044	33	C	−2.833077	−3.05503	0.058604
14	H	3.653794	−1.748406	2.383559	34	H	−2.405393	−3.60006	0.906916
15	H	4.918161	−0.078269	−1.368249	35	H	−3.9216	−3.153743	0.121034
16	H	5.480869	−1.068066	0.841391	36	H	−2.504352	−3.548424	−0.861815
17	C	−0.058295	1.089402	−0.172693	37	C	−3.014383	−0.429137	1.632181
18	C	−0.134856	2.113415	−1.117279	38	H	−4.096277	−0.593247	1.691528
19	C	−0.264779	1.393668	1.175573	39	H	−2.559368	−0.853233	2.532911
20	C	−0.448093	3.414553	−0.725605	40	H	−2.833031	0.64861	1.635761

O (*N*−TMS hemiaminal: type 2)

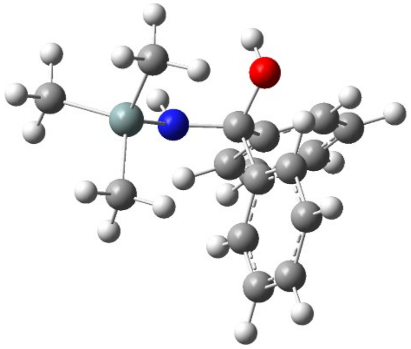

APFD/6-31G(d,p): Thermal Correction to Free Energy = 0.286177 Hartree

APFD/6-311+G(2d,p)//APFD/6-31G(d,p):
Electronic Energy (*EE*) = −1041.409283 Hartree
Gibbs free energy (*G*) = −1041.123106 Hartree

1	C	−0.343194	−0.308258	0.550936	21	C	1.209841	3.188285	0.745343
2	O	−0.186671	−0.414322	1.970907	22	H	0.791703	1.61031	2.158646
3	H	−0.487309	−1.297801	2.210974	23	C	1.112734	3.606007	−0.57883
4	N	0.438359	−1.281127	−0.16937	24	H	0.43296	3.087528	−2.558883
5	C	−1.828318	−0.452383	0.196975	25	H	1.667821	3.835835	1.488849
6	C	−2.788116	0.032447	1.090573	26	H	1.49464	4.579509	−0.875286
7	C	−2.249024	−1.017056	−1.009343	27	H	−0.018358	−2.186979	−0.185529
8	C	−4.143271	−0.056366	0.788641	28	Si	2.202122	−1.376997	−0.136374
9	H	−2.454954	0.476754	2.023532	29	C	2.897643	−1.266575	1.607813
10	C	−3.607271	−1.107096	−1.311498	30	H	2.502997	−2.063242	2.246155
11	H	−1.507175	−1.381347	−1.714381	31	H	3.990366	−1.347426	1.597748
12	C	−4.558242	−0.628847	−0.413786	32	H	2.62638	−0.311586	2.065219
13	H	−4.878954	0.32282	1.493493	33	C	2.984797	−0.038607	−1.194338
14	H	−3.920852	−1.551961	−2.252583	34	H	2.549448	−0.024659	−2.197617
15	H	−5.616947	−0.698883	−0.649532	35	H	2.848417	0.954245	−0.757251
16	C	0.141053	1.093794	0.182642	36	H	4.060674	−0.225027	−1.289615
17	C	0.036098	1.521664	−1.143364	37	C	2.563146	−3.073604	−0.87061
18	C	0.720276	1.938692	1.127979	38	H	2.160758	−3.161579	−1.884919
19	C	0.516948	2.768611	−1.523164	39	H	3.643278	−3.243897	−0.925456
20	H	−0.415662	0.862343	−1.878323	40	H	2.135318	−3.878769	−0.263489

TS VIII

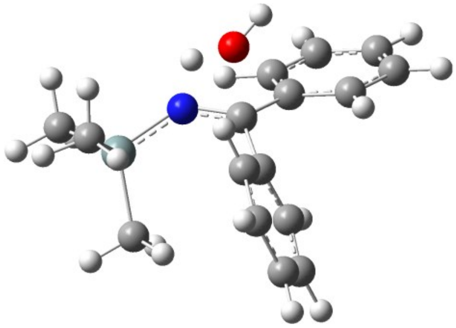

APFD/6-31G(d,p): Thermal Correction to Free Energy = 0.278236 Hartree

APFD/6-311+G(2d,p)//APFD/6-31G(d,p):
Electronic Energy (*EE*) = −1041.329222 Hartree
Gibbs free energy (*G*) = −1041.050986 Hartree

1	C	−0.38145	−0.181192	0.363634	21	C	1.866057	2.838141	0.773153
2	O	−0.389588	−0.517275	2.243782	22	H	1.336965	1.126841	1.991735
3	H	−1.290723	−0.648463	2.570875	23	C	1.62853	3.514498	−0.424296
4	N	0.34026	−1.322733	0.241406	24	H	0.492844	3.547845	−2.255789
5	C	−1.856562	−0.25717	0.11909	25	H	2.583749	3.232596	1.487751
6	C	−2.69736	0.814038	0.448031	26	H	2.169333	4.430042	−0.64908
7	C	−2.399613	−1.411482	−0.447962	27	H	0.022228	−1.42249	1.459748
8	C	−4.064849	0.725612	0.216078	28	Si	2.01371	−1.543829	−0.232434
9	H	−2.268894	1.709667	0.890158	29	C	3.215157	−1.211812	1.183546
10	C	−3.769969	−1.497491	−0.682173	30	H	2.87168	−1.680369	2.111563
11	H	−1.721473	−2.221886	−0.698931	31	H	4.207168	−1.615028	0.951133
12	C	−4.603925	−0.431169	−0.351035	32	H	3.322916	−0.139101	1.367041
13	H	−4.713531	1.557876	0.476418	33	C	2.474278	−0.472093	−1.710172
14	H	−4.187041	−2.396278	−1.128972	34	H	1.732718	−0.55894	−2.510591
15	H	−5.672904	−0.497285	−0.536748	35	H	2.556196	0.583888	−1.438568
16	C	0.250953	1.155926	0.150025	36	H	3.440548	−0.795092	−2.113716
17	C	−0.010322	1.851168	−1.034297	37	C	2.135018	−3.35998	−0.707139
18	C	1.183121	1.660782	1.060084	38	H	1.475752	−3.584174	−1.551574
19	C	0.686781	3.021232	−1.325175	39	H	3.157603	−3.630219	−0.991404
20	H	−0.744458	1.460137	−1.733185	40	H	1.837185	−4.002681	0.127756

P (*N*−TMS diphenylmethanimine + H_2O)

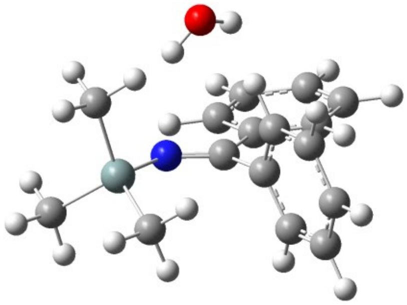

APFD/6-31G(d,p): Thermal Correction to Free Energy = 0.279283 Hartree

APFD/6-311+G(2d,p)//APFD/6-31G(d,p):
Electronic Energy (*EE*) = −1041.399218 Hartree
Gibbs free energy (*G*) = −1041.119935 Hartree

1	C	−0.339705	−0.164262	−0.289781	21	C	1.406899	2.79953	1.259119
2	O	−0.698576	−1.059545	2.616593	22	H	0.52265	0.949123	1.97005
3	H	−1.555605	−0.73556	2.320083	23	C	1.654873	3.601678	0.145221
4	N	0.318026	−1.256639	−0.326104	24	H	1.399874	3.825999	−1.982953
5	C	−1.82849	−0.194073	−0.30655	25	H	1.739409	3.11743	2.243968
6	C	−2.58274	0.971214	−0.118788	26	H	2.186544	4.542672	0.259464
7	C	−2.49266	−1.417119	−0.48093	27	H	−0.378696	−1.547926	1.84794
8	C	−3.974648	0.914212	−0.103922	28	Si	2.052881	−1.566991	−0.254007
9	H	−2.073528	1.920156	0.021406	29	C	2.407898	−2.019104	1.537202
10	C	−3.879798	−1.471954	−0.466798	30	H	1.817334	−2.883805	1.856064
11	H	−1.888754	−2.308402	−0.625627	31	H	3.465908	−2.272266	1.663999
12	C	−4.625182	−0.305291	−0.277679	32	H	2.173671	−1.18828	2.209146
13	H	−4.550869	1.823317	0.045884	33	C	3.17825	−0.162122	−0.806005
14	H	−4.385942	−2.423948	−0.60412	34	H	2.922483	0.184858	−1.811633
15	H	−5.711175	−0.349543	−0.265732	35	H	3.124571	0.698012	−0.133743
16	C	0.310839	1.177144	−0.152974	36	H	4.214996	−0.517734	−0.828979
17	C	0.532639	1.993148	−1.263581	37	C	2.327766	−3.048701	−1.375824
18	C	0.734106	1.588406	1.11505	38	H	2.083152	−2.805485	−2.414805
19	C	1.213007	3.199522	−1.114621	39	H	3.372766	−3.374931	−1.345038
20	H	0.187331	1.673022	−2.243491	40	H	1.69767	−3.889379	−1.070642

Chemical species in the calculation of adsorption energy (Table S7)

γ-Al_2O_3 cluster: [AlO_3]$_{clus}$

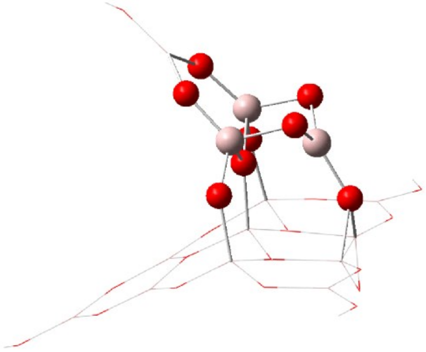

APFD/6-31G(d,p):

Electronic Energy (*EE*) = −5201.601247 Hartree
Thermal Correction to Free Energy = −0.025782 Hartree
Gibbs free energy (*G*) = −5201.627028

APFD/6-31+G(2d,p)//APFD/6-31G(d,p):
Electronic Energy (*EE*) = −5201.878777 Hartree
Gibbs free energy (*G*) = −5201.904559 Hartree

APFD/6-311+G(2d,p)//APFD/6-31G(d,p):
Electronic Energy (*EE*) = −5202.585423 Hartree
Gibbs free energy (*G*) = −5202.611205 Hartree

1	O	−0.278278	−1.537123	3.834101	22	H	1.559193	7.139115	−1.524729
2	O	2.33529	0.502043	−2.678674	23	Al	1.484045	4.794749	−1.394091
3	O	0.893627	−1.327947	−1.027768	24	Al	3.394898	−3.71113	−1.418471
4	O	0.228176	1.582584	−1.047682	25	O	2.108028	6.360273	−1.632889
5	O	0.54982	−2.846515	1.27774	26	O	4.619362	−4.848042	−1.732157
6	O	−0.799651	2.790802	1.278205	27	Al	−0.835053	2.998915	−0.473106
7	O	2.458554	3.410086	−1.540925	28	O	−0.949352	1.22792	3.826343
8	O	3.692839	−2.034874	−1.464758	29	Al	−3.054294	0.94533	−1.185563
9	O	0.144914	0.038743	1.346243	30	Al	−2.360186	−2.159815	−1.164425
10	Al	−0.123792	1.670659	2.394283	31	O	−4.588973	0.409562	−1.640886
11	Al	0.63709	−1.534863	2.387509	32	O	−3.975397	−2.335736	−1.613523
12	Al	−0.153363	−0.029276	−0.435875	33	Al	−1.083852	−0.262293	4.635927
13	Al	2.584992	−0.742274	−1.472862	34	O	−1.995441	−0.441668	6.056024
14	Al	2.003901	1.772488	−1.513387	35	H	−2.058236	−1.270517	6.533349
15	O	−1.90366	−0.425809	−0.86458	36	Al	−5.193234	−1.172033	−1.883333
16	O	−2.418898	2.468336	−0.992394	37	O	−6.768459	−1.584749	−2.32742
17	O	−1.129033	−3.255916	−0.953717	38	H	−7.479018	−0.962593	−2.489645
18	Al	0.53862	−3.067779	−0.473058	39	O	2.314823	−0.945754	2.418187
19	O	1.776536	−4.110115	−1.054305	40	O	1.642342	1.89067	2.433709

20	H	4.460382	−5.792242	−1.684173	41	O	2.660403	0.610756	−0.163715
21	O	−0.151416	4.479308	−1.015496	42	Al	2.143952	0.518208	1.489367

Benzophenone (1a)

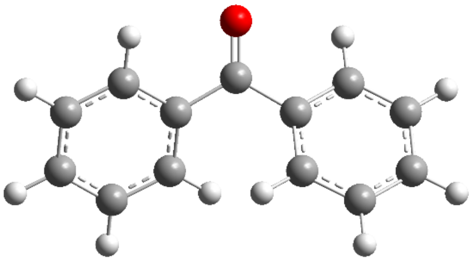

APFD/6-31G(d,p):
Electronic Energy (*EE*) = −576.173615 Hartree
Thermal Correction to Free Energy = 0.155415 Hartree
Gibbs free energy (*G*) = −576.018200 Hartree

APFD/6-31+G(2d,p)//APFD/6-31G(d,p):
Electronic Energy (*EE*) = −576.204710 Hartree
Gibbs free energy (*G*) = −576.049295 Hartree

APFD/6-311+G(2d,p)//APFD/6-31G(d,p):
Electronic Energy (*EE*) = −576.309594 Hartree
Gibbs free energy (*G*) = −576.154179 Hartree

1	C	−3.660178	0.348887	−0.471961	13	C	2.422203	0.977896	0.50692
2	C	−3.787267	−0.907541	0.12364	14	O	0.000041	2.314593	0.000201
3	C	−2.673554	−1.530602	0.683718	15	H	−4.530303	0.837562	−0.902171
4	C	−1.42786	−0.90995	0.631263	16	H	−4.757136	−1.397229	0.156949
5	C	−1.292967	0.34601	0.027824	17	H	−2.775339	−2.499668	1.164834
6	C	−2.422063	0.977838	−0.507173	18	H	−0.562458	−1.387767	1.081225
7	C	0.00003	1.092454	0.000096	19	H	−2.301172	1.96477	−0.943889
8	C	1.292977	0.345974	−0.027761	20	H	0.562252	−1.387976	−1.080726
9	C	1.427731	−0.91008	−0.631	21	H	2.775089	−2.499938	−1.164513
10	C	2.673415	−1.530783	−0.683555	22	H	4.757083	−1.397343	−0.157177
11	C	3.787224	−0.907639	−0.123802	23	H	4.530515	0.837624	0.901547
12	C	3.660276	0.348923	0.471597	24	H	2.301339	1.964904	0.94347

Diphenylmethanimine (2a)

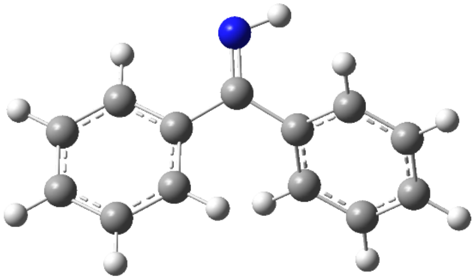

APFD/6-31G(d,p):
Electronic Energy (*EE*) = −556.306997 Hartree
Thermal Correction to Free Energy = 0.167847 Hartree
Gibbs free energy (*G*) = −556.139150 Hartree

APFD/6-31+G(2d,p)//APFD/6-31G(d,p):
Electronic Energy (*EE*) = −556.334805 Hartree
Gibbs free energy (*G*) = −556.166958 Hartree

APFD/6-311+G(2d,p)//APFD/6-31G(d,p):
Electronic Energy (*EE*) = −556.434205 Hartree
Gibbs free energy (*G*) = −556.266356 Hartree

1	C	0.02211	1.134159	0.067071	14	C	−1.267779	0.386231	0.051223
2	N	0.085327	2.411189	0.154808	15	C	−2.324813	0.841685	−0.745243
3	C	1.293149	0.368393	−0.015802	16	C	−1.46368	−0.735778	0.865931
4	C	1.344018	−0.912409	−0.579177	17	C	−3.554883	0.190663	−0.726994
5	C	2.477953	0.95583	0.447827	18	H	−2.168046	1.698043	−1.39697
6	C	2.555122	−1.593775	−0.674925	19	C	−2.698747	−1.376957	0.895556
7	H	0.43341	−1.369856	−0.955124	20	H	−0.644684	−1.093631	1.483834
8	C	3.683851	0.272195	0.358671	21	C	−3.745663	−0.917494	0.097247
9	H	2.424124	1.95335	0.873012	22	H	−4.363947	0.545157	−1.360129
10	C	3.726065	−1.005634	−0.202527	23	H	−2.84453	−2.237939	1.542577
11	H	2.58329	−2.5843	−1.121309	24	H	−4.706758	−1.424523	0.114376
12	H	4.595676	0.733855	0.728659	25	H	−0.856291	2.798785	0.245223
13	H	4.670247	−1.539924	−0.27096					

***N*−TMS diphenylmethanimine**

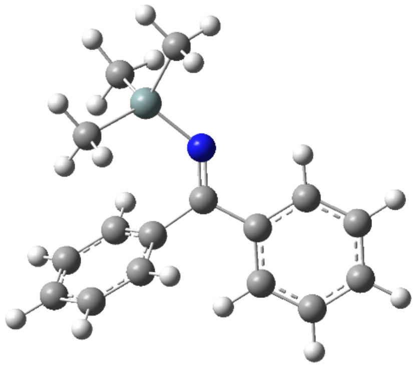

APFD/6-31G(d,p):
Electronic Energy (*EE*) = −964.813349 Hartree
Thermal Correction to Free Energy = 0.258609 Hartree
Gibbs free energy (*G*) = −964.554740 Hartree

APFD/6-31+G(2d,p)//APFD/6-31G(d,p):
Electronic Energy (*EE*) = −964.848224 Hartree
Gibbs free energy (*G*) = −964.589615 Hartree

APFD/6-311+G(2d,p)//APFD/6-31G(d,p):
Electronic Energy (*EE*) = −964.990940 Hartree
Gibbs free energy (*G*) = −964.732331 Hartree

1	C	0.399082	−0.255833	0.046862	20	H	0.389059	1.545942	−1.987969
2	N	−0.288527	−1.329013	0.062331	21	C	−1.547211	3.568584	−0.05446
3	C	1.886139	−0.321028	0.051798	22	H	−2.120547	3.148713	1.980506
4	C	2.671049	0.82656	0.217926	23	H	−0.807418	3.720705	−2.072768
5	C	2.51774	−1.5628	−0.10256	24	H	−2.067526	4.522045	−0.088855
6	C	4.061056	0.734818	0.227154	25	Si	−2.023087	−1.601422	−0.043554
7	H	2.189577	1.791627	0.346205	26	C	−2.971935	−0.307315	−1.033077
8	C	3.902817	−1.65294	−0.096097	27	H	−2.518509	−0.154097	−2.017517
9	H	1.88903	−2.43997	−0.224946	28	H	−4.001638	−0.648235	−1.191077
10	C	4.67938	−0.50307	0.068264	29	H	−3.004186	0.660896	−0.526947
11	H	4.66099	1.631255	0.360402	30	C	−2.702041	−1.712084	1.709169
12	H	4.383713	−2.619851	−0.220587	31	H	−2.082039	−2.368141	2.327597
13	H	5.764123	−0.574598	0.0727	32	H	−2.738739	−0.727444	2.184579
14	C	−0.225198	1.106925	0.025809	33	H	−3.719458	−2.11779	1.700874
15	C	−0.92416	1.568099	1.144509	34	C	−2.197906	−3.265654	−0.898626
16	C	−0.168327	1.899219	−1.124107	35	H	−1.674881	−4.045429	−0.336807
17	C	−1.580494	2.795869	1.105736	36	H	−3.249948	−3.555838	−0.99088
18	H	−0.949915	0.957237	2.042946	37	H	−1.768359	−3.234223	−1.905105
19	C	−0.838393	3.119015	−1.16812					

Water

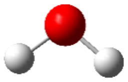

APFD/6-31G(d,p):
Electronic Energy (*EE*) = −76.359795 Hartree
Thermal Correction to Free Energy = 0.004024 Hartree
Gibbs free energy (*G*) = −76.355771 Hartree

APFD/6-31+G(2d,p)//APFD/6-31G(d,p):
Electronic Energy (*EE*) = −76.377403 Hartree
Gibbs free energy (*G*) = −76.373379 Hartree

APFD/6-311+G(2d,p)//APFD/6-31G(d,p):
Electronic Energy (*EE*) = −76.396202 Hartree
Gibbs free energy (*G*) = −76.392178 Hartree

1	O	0	0	0.11849	3	H	0	−0.757256	−0.47396
2	H	0	0.757256	−0.47396					

Ammonia

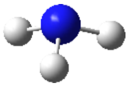

APFD/6-31G(d,p):
Electronic Energy (*EE*) = −56.507929 Hartree
Thermal Correction to Free Energy = 0.015684 Hartree
Gibbs free energy (*G*) = −56.492245 Hartree

APFD/6-31+G(2d,p)//APFD/6-31G(d,p):
Electronic Energy (*EE*) = −56.517588 Hartree
Gibbs free energy (*G*) = −56.501904 Hartree

APFD/6-311+G(2d,p)//APFD/6-31G(d,p):
Electronic Energy (*EE*) = −56.531599 Hartree
Gibbs free energy (*G*) = −56.515915 Hartree

1	N	0	−0.000006	−0.117946	3	H	0.811697	−0.466509	0.275213
2	H	−0.809916	−0.469594	0.275212	4	H	−0.001782	0.936144	0.275195

$TMSNH_2$

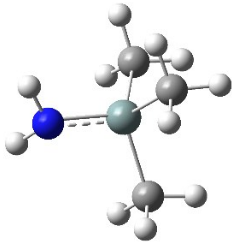

APFD/6-31G(d,p):
Electronic Energy (*EE*) = −465.015306 Hartree
Thermal Correction to Free Energy = 0.105199 Hartree
Gibbs free energy (*G*) = −464.910107 Hartree

APFD/6-31+G(2d,p)//APFD/6-31G(d,p):
Electronic Energy (*EE*) = −465.030269 Hartree
Gibbs free energy (*G*) = −464.92507 Hartree

APFD/6-311+G(2d,p)//APFD/6-31G(d,p):
Electronic Energy (*EE*) = −465.088044 Hartree
Gibbs free energy (*G*) = −464.982845 Hartree

1	N	−0.002467	−0.762224	1.599865	9	H	−1.614195	−0.152579	−1.864709
2	Si	−0.000024	−0.006155	0.028211	10	H	−2.458071	−0.295653	−0.321081
3	C	−1.551103	−0.587418	−0.861763	11	H	0.004188	2.30198	−0.939417
4	C	0.004472	1.882367	0.073065	12	H	0.890533	2.26585	0.590393
5	C	1.548945	−0.594578	−0.860691	13	H	−0.878313	2.270135	0.592764
6	H	0.824561	−0.672933	2.174089	14	H	2.456776	−0.308391	−0.31846
7	H	−0.829653	−0.669322	2.173279	15	H	1.546945	−1.683992	−0.961051
8	H	−1.554697	−1.676973	−0.960576	16	H	1.615645	−0.158609	−1.862912

Benzophenone (1a) @[AlO_3]$_{clus}$

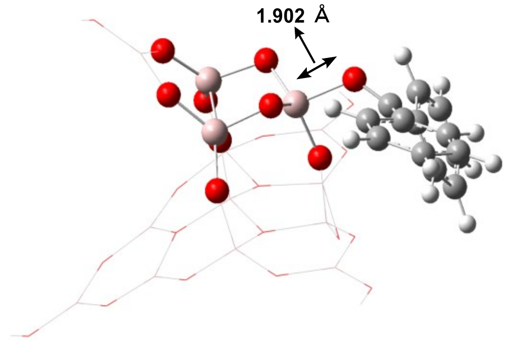

APFD/6-31G(d,p):
Electronic Energy (*EE*) = −5777.841732 Hartree
Thermal Correction to Free Energy = 0.153751 Hartree
Gibbs free energy (*G*) = −5777.687982 Hartree

APFD/6-31+G(2d,p)//APFD/6-31G(d,p):
Electronic Energy (*EE*) = −5778.142169 Hartree
Gibbs free energy (*G*) = −5777.988418 Hartree

APFD/6-311+G(2d,p)//APFD/6-31G(d,p):
Electronic Energy (*EE*) = −5778.956184 Hartree
Gibbs free energy (*G*) = −5778.802433 Hartree

1	O	−1.316237	−2.322722	3.437671	34	O	−3.008246	−1.752411	5.866022
2	O	1.053779	1.05727	−2.592367	35	H	−3.030559	−2.660836	6.171148
3	O	−0.282306	−1.115059	−1.308506	36	Al	−6.391765	−1.021567	−1.995462
4	O	−1.043164	1.715165	−0.738319	37	O	−7.963949	−1.397284	−2.481207
5	O	−0.515296	−3.070013	0.656674	38	H	−8.698632	−0.782416	−2.502871
6	O	−2.047787	2.40108	1.800478	39	O	1.205714	−1.375737	2.189107
7	O	1.112026	3.686325	−0.903816	40	O	0.468209	1.360824	2.757326
8	O	2.525855	−1.616564	−1.93213	41	O	1.391616	0.664472	−0.113467
9	O	−1.010985	−0.272126	1.303318	42	Al	1.318744	0.304827	1.624697
10	Al	−1.304968	1.110502	2.659714	43	C	4.043051	0.321897	0.931568
11	Al	−0.439649	−2.003109	2.003453	44	O	3.200275	0.451812	1.864543
12	Al	−1.355294	0.001244	−0.450473	45	C	4.74951	−0.942991	0.769442
13	Al	1.376614	−0.390105	−1.662583	46	C	5.961515	−1.024358	0.062018
14	Al	0.711914	2.060186	−1.192588	47	C	4.164223	−2.109336	1.296428
15	O	−3.102277	−0.368365	−0.914437	48	C	6.559289	−2.259385	−0.149458
16	O	−3.715428	2.472854	−0.456225	49	H	6.434936	−0.117942	−0.302009
17	O	−2.237922	−3.094147	−1.577745	50	C	4.759376	−3.341184	1.061577
18	Al	−0.56452	−2.9422	−1.102409	51	H	3.232325	−2.034379	1.852321
19	O	0.690354	−3.802267	−1.903099	52	C	5.949999	−3.417764	0.334744
20	H	3.410073	−5.225364	−2.906709	53	H	7.495625	−2.322957	−0.695522
21	O	−1.516567	4.53237	−0.125455	54	H	4.295278	−4.245865	1.444016
22	H	0.091601	7.302538	−0.131518	55	H	6.408359	−4.385247	0.148906
23	Al	0.096923	4.977316	−0.466329	56	C	4.242628	1.435092	0.001421
24	Al	2.28442	−3.278961	−2.212935	57	C	4.092045	2.747096	0.47497
25	O	0.662579	6.581453	−0.402689	58	C	4.420924	1.19886	−1.371752
26	O	3.536644	−4.28497	−2.769869	59	C	4.164312	3.816114	−0.410067
27	Al	−2.136363	2.94962	0.126377	60	H	3.917285	2.909648	1.534298
28	O	−2.077265	0.361979	3.991097	61	C	4.449387	2.27616	−2.255725
29	Al	−4.30548	0.995164	−0.93462	62	H	4.447205	0.179142	−1.746538
30	Al	−3.509575	−2.024917	−1.542729	63	C	4.336668	3.579796	−1.774337
31	O	−5.83342	0.502632	−1.456553	64	H	4.048864	4.831713	−0.045056
32	O	−5.129614	−2.169174	−1.985687	65	H	4.536284	2.091518	−3.321855
33	Al	−2.140333	−1.262203	4.492523	66	H	4.352392	4.415905	−2.467158

Diphenylmetaneimine (2a) @[AlO_3]$_{clus}$

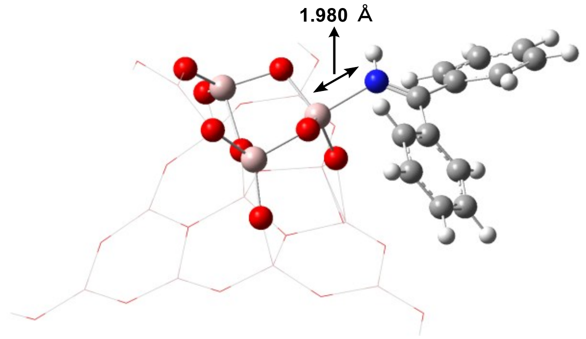

APFD/6-31G(d,p):
Electronic Energy (*EE*) = −5757.991114 Hartree
Thermal Correction to Free Energy = 0.164105 Hartree
Gibbs free energy (*G*) = −5757.827009 Hartree

APFD/6-31+G(2d,p)//APFD/6-31G(d,p):
Electronic Energy (*EE*) = −5758.288022 Hartree
Gibbs free energy (*G*) = −5758.123917 Hartree

APFD/6-311+G(2d,p)//APFD/6-31G(d,p):
Electronic Energy (*EE*) = −5759.095867 Hartree
Gibbs free energy (*G*) = −5758.931762 Hartree

1	O	−1.454035	−1.698366	3.674895	35	H	−2.939411	−1.406059	6.544276
2	O	0.85317	0.472184	−2.910557	36	Al	−6.740163	−0.226824	−1.508185
3	O	−0.685994	−1.269815	−1.252591	37	O	−8.39023	−0.386993	−1.825207
4	O	−0.931711	1.696435	−1.017703	38	H	−9.016786	0.336192	−1.877376
5	O	−1.043193	−2.903231	0.970567	39	O	1.067611	−1.285288	2.200077
6	O	−1.57496	2.846321	1.470112	40	O	0.801616	1.576878	2.363808
7	O	1.48434	3.238955	−1.601696	41	O	1.377151	0.330173	−0.433179
8	O	1.936877	−2.311937	−2.001401	42	Al	1.359387	0.240254	1.344888
9	O	−1.026306	−0.004233	1.266462	43	C	4.261525	0.134299	0.827941
10	Al	−0.970035	1.562798	2.440831	44	C	4.212353	−1.311149	0.564122
11	Al	−0.674607	−1.708773	2.151767	45	C	4.690917	−1.835772	−0.638079
12	Al	−1.483929	0.108112	−0.479109	46	C	3.635545	−2.171126	1.519951
13	Al	1.026918	−0.884949	−1.818944	47	C	4.562987	−3.201185	−0.901512
14	Al	0.806116	1.681245	−1.638532	48	H	5.117562	−1.174058	−1.385068
15	O	−3.302828	−0.013819	−0.762959	49	C	3.519265	−3.53778	1.252311
16	O	−3.41199	2.924536	−0.650758	50	H	3.311517	−1.774912	2.474151
17	O	−2.94743	−2.906788	−1.111386	51	C	3.98301	−4.051769	0.045216
18	Al	−1.234361	−2.983882	−0.781599	52	H	4.884265	−3.60207	−1.857204
19	O	−0.211643	−4.135049	−1.546054	53	H	3.071853	−4.189837	1.996255
20	H	2.142756	−6.109736	−2.539395	54	H	3.885717	−5.109029	−0.18109
21	O	−0.89298	4.606441	−0.759391	55	C	5.365284	0.950406	0.301443

22	H	1.126996	7.042473	−1.254887	56	C	6.688258	0.48903	0.336832
23	Al	0.732046	4.726762	−1.271576	57	C	5.072003	2.20122	−0.265167
24	Al	1.40961	−3.931025	−2.036781	58	C	7.713119	1.289562	−0.153494
25	O	1.548628	6.207521	−1.466058	59	H	6.904756	−0.485048	0.766306
26	O	2.42915	−5.194821	−2.540415	60	C	6.1011	2.985921	−0.773894
27	Al	−1.730139	3.194149	−0.252179	61	H	4.039616	2.531565	−0.364155
28	O	−1.724575	1.123551	3.912918	62	C	7.419761	2.536131	−0.708528
29	Al	−4.270925	1.521122	−0.881516	63	H	8.740999	0.941424	−0.107525
30	Al	−4.024415	−1.641359	−1.130695	64	H	5.86922	3.942232	−1.233402
31	O	−5.899399	1.23481	−1.221655	65	H	8.221793	3.153828	−1.103389
32	O	−5.680807	−1.561845	−1.433331	66	N	3.285731	0.674868	1.487199
33	Al	−1.997933	−0.393226	4.632669	67	H	3.438539	1.658157	1.703736
34	O	−2.801546	−0.558514	6.118253					

_N_-TMS diphenylmetaneimine@[AlO3]_clus_

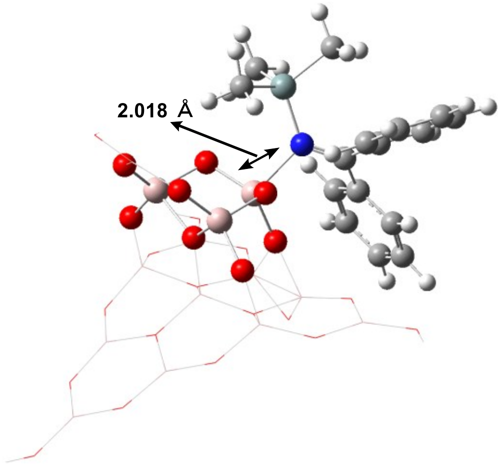

APFD/6-31G(d,p):
Electronic Energy (*EE*) = −6166.495458 Hartree
Thermal Correction to Free Energy = 0.258175 Hartree
Gibbs free energy (*G*) = −6166.237283 Hartree

APFD/6-31+G(2d,p)//APFD/6-31G(d,p):
Electronic Energy (*EE*) = −6166.798927 Hartree
Gibbs free energy (*G*) = −6166.540752 Hartree

APFD/6-311+G(2d,p)//APFD/6-31G(d,p):
Electronic Energy (*EE*) = −6167.650003 Hartree
Gibbs free energy (*G*) = −6167.391828 Hartree

1	O	−0.3924735	−1.6433171	3.9437938	41	O	1.1266025	0.8018209	−0.6212652
2	O	0.0677585	0.8085939	−2.9249042	42	Al	1.4572035	0.6924089	1.1168918

3	O	−0.7652885	−1.1516971	−1.0233992
4	O	−1.4610435	1.7302839	−0.6708552
5	O	−0.3603535	−2.7936501	1.1852418
6	O	−1.7530255	2.7835509	1.9234868
7	O	0.4764465	3.6593379	−1.7113332
8	O	1.7798365	−1.7346091	−2.3305872
9	O	−0.7789925	0.0682399	1.5407198
10	Al	−0.7461285	1.6363519	2.7145358
11	Al	0.0384215	−1.5384471	2.2912578
12	Al	−1.6065425	0.0774829	−0.0668072
13	Al	0.6978035	−0.4840291	−1.9270132
14	Al	0.0831645	2.0075469	−1.6425742
15	O	−3.3959075	−0.3604251	0.0343348
16	O	−3.9813175	2.5161009	0.2376458
17	O	−2.6310995	−3.1527401	−0.4504332
18	Al	−0.8997575	−2.9285441	−0.4892682
19	O	0.1208275	−3.8954481	−1.4787252
20	H	2.5160065	−5.4463341	−2.9910652
21	O	−1.8684695	4.6063079	−0.3564292
22	H	−0.4467605	7.3480869	−1.2058162
23	Al	−0.4331645	4.9989409	−1.1950362
24	Al	1.5426665	−3.4207531	−2.2935502
25	O	0.0577185	6.5958769	−1.5207682
26	O	2.6344805	−4.4957911	−3.0301122
27	Al	−2.3249955	3.0772559	0.2809468
28	O	−1.0867025	1.0922639	4.3011328
29	Al	−4.6158455	0.9825759	0.1584718
30	Al	−3.8886815	−2.0927161	−0.2129342
31	O	−6.2056235	0.4149449	0.1606918
32	O	−5.5601685	−2.3043951	−0.1593912
33	Al	−0.9379265	−0.4395801	5.0254808
34	O	−1.3698715	−0.7221521	6.6421608
35	H	−1.2673895	−1.5752441	7.0670928
36	Al	−6.8244325	−1.1734931	0.0217908
37	O	−8.4517535	−1.6203211	0.0541738
38	H	−9.1897715	−1.0169621	0.1520168
39	O	1.6374085	−0.9633391	1.7601648
40	O	0.9411025	1.9331469	2.2528098
43	C	4.1913765	0.4557769	0.2948598
44	N	3.4560185	0.9172769	1.2788628
45	C	3.9699825	0.9221819	−1.0717802
46	C	4.1733035	0.0686839	−2.1748062
47	C	3.5222115	2.2456619	−1.2806972
48	C	3.8648955	0.5124269	−3.4506342
49	H	4.4830385	−0.9567871	−2.0121852
50	C	3.2589285	2.6966559	−2.5604472
51	H	3.3720095	2.8925419	−0.4217842
52	C	3.4054415	1.8194659	−3.6439432
53	H	3.9430475	−0.1694281	−4.2909412
54	H	2.8804825	3.7008209	−2.7131722
55	H	3.1481295	2.1564419	−4.6439792
56	C	5.2091145	−0.5852611	0.5218988
57	C	4.8662555	−1.6608491	1.3550378
58	C	6.4654985	−0.5422671	−0.0983102
59	C	5.7893435	−2.6769011	1.5759598
60	H	3.8640555	−1.6965821	1.7783958
61	C	7.3922505	−1.5466011	0.1540398
62	H	6.7145755	0.2912839	−0.7490202
63	C	7.0537745	−2.6133701	0.9882828
64	H	5.5222115	−3.5213031	2.2049638
65	H	8.3766195	−1.5031231	−0.3031482
66	H	7.7762165	−3.4040061	1.1723418
67	Si	4.0491305	1.3278909	2.9496968
68	C	5.8702805	0.9214499	3.1638898
69	H	6.4791335	1.2959009	2.3360288
70	H	6.2099855	1.4248109	4.0767998
71	H	6.0654165	−0.1475611	3.2716938
72	C	3.0159655	0.4700009	4.2536138
73	H	2.0194555	0.9134019	4.3085608
74	H	2.9108095	−0.5922201	4.0143968
75	H	3.5064405	0.5771169	5.2271618
76	C	3.8280615	3.1902809	2.9859288
77	H	2.7758045	3.4252579	2.8091708
78	H	4.1216305	3.5762389	3.9682048
79	H	4.4457485	3.6831329	2.2288268

Water@[AlO3]*clus*

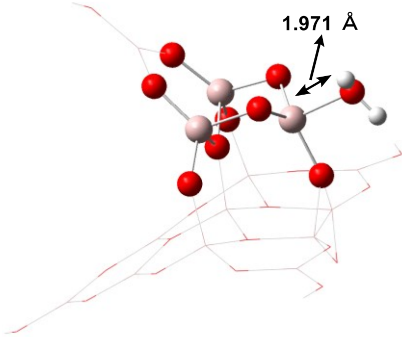

APFD/6-31G(d,p):
Electronic Energy (*EE*) = −5278.017399 Hartree
Thermal Correction to Free Energy = −0.003055 Hartree
Gibbs free energy (*G*) = −5278.017399 Hartree

APFD/6-31+G(2d,p)//APFD/6-31G(d,p):
Electronic Energy (*EE*) = −5278.297048 Hartree
Gibbs free energy (*G*) = −5278.300103 Hartree

APFD/6-311+G(2d,p)//APFD/6-31G(d,p):
Electronic Energy (*EE*) = −5279.024464 Hartree
Gibbs free energy (*G*) = −5279.027519 Hartree

1	O	−0.270583	−1.467524	3.826457	24	Al	2.445078	−4.228152	−1.724454
2	O	1.978907	0.101917	−2.946965	25	O	2.833797	5.91658	−1.986142
3	O	0.426333	−1.460478	−1.130143	26	O	3.425955	−5.553785	−2.137815
4	O	0.254908	1.520255	−1.134891	27	Al	−0.4951	3.094742	−0.482861
5	O	0.067086	−2.898236	1.223965	28	O	−0.470206	1.370262	3.836974
6	O	−0.319924	2.884385	1.259672	29	Al	−3.084838	1.440035	−0.936622
7	O	2.696927	2.949351	−1.877822	30	Al	−2.917507	−1.7374	−0.930093
8	O	3.011095	−2.625223	−1.829113	31	O	−4.725014	1.167759	−1.228673
9	O	0.157561	0.013906	1.281714	32	O	−4.576297	−1.641431	−1.214257
10	Al	0.270267	1.669345	2.323165	33	Al	−0.767575	−0.075637	4.682132
11	Al	0.483263	−1.621227	2.298178	34	O	−1.55036	−0.099428	6.18771
12	Al	−0.325888	−0.004711	−0.460398	35	H	−1.701259	−0.905808	6.683509
13	Al	2.13834	−1.165837	−1.750727	36	Al	−5.604791	−0.290884	−1.382638
14	Al	1.981431	1.411214	−1.777433	37	O	−7.263177	−0.435053	−1.661645
15	O	−2.151587	−0.103872	−0.707039	38	H	−7.873181	0.29691	−1.763675
16	O	−2.189193	2.835681	−0.833741	39	O	2.228962	−1.317614	2.211243
17	O	−1.870658	−3.023561	−0.82301	40	O	2.04146	1.596569	2.223685
18	Al	−0.154709	−3.11633	−0.512806	41	O	2.568393	0.157092	−0.481194
19	O	0.827729	−4.351037	−1.195329	42	Al	2.450213	0.167311	1.282061
20	H	3.118005	−6.458148	−2.057898	43	O	4.41062	0.107045	1.472854
21	O	0.366859	4.439752	−1.115812	44	H	4.661394	−0.650724	2.019258

22	H	2.435608	6.776209	−1.837831	45	H	4.832899	0.016772	0.607626
23	Al	1.985645	4.477399	−1.659537					

Ammonia@[AlO3]*clus*

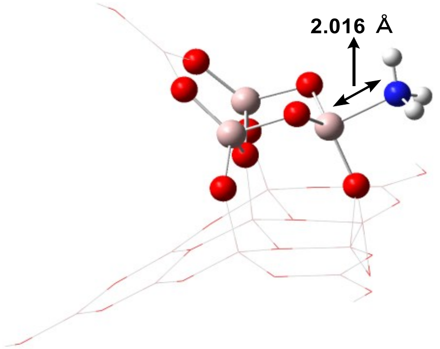

APFD/6-31G(d,p):
Electronic Energy (*EE*) = −5258.175716 Hartree
Thermal Correction to Free Energy = 0.006913 Hartree
Gibbs free energy (*G*) = −5258.168804 Hartree

APFD/6-31+G(2d,p)//APFD/6-31G(d,p):
Electronic Energy (*EE*) = −5258.453010 Hartree
Gibbs free energy (*G*) = −5258.446097 Hartree

APFD/6-311+G(2d,p)//APFD/6-31G(d,p):
Electronic Energy (*EE*) = −5259.175083 Hartree
Gibbs free energy (*G*) = −5259.168170 Hartree

1	O	0.325343	1.430864	3.836138	24	Al	−2.176305	4.381298	−1.717708
2	O	−1.949905	0.039893	−2.967621	25	O	−3.147676	−5.722009	−2.056482
3	O	−0.32614	1.497626	−1.1262	26	O	−3.075251	5.76417	−2.129837
4	O	−0.327048	−1.487932	−1.151148	27	Al	0.324907	−3.107505	−0.504149
5	O	0.094293	2.896266	1.241312	28	O	0.360686	−1.413732	3.827745
6	O	0.146485	−2.89916	1.238294	29	Al	3.009834	−1.602205	−0.923573
7	O	−2.840708	−2.768452	−1.925416	30	Al	3.026169	1.579472	−0.895381
8	O	−2.83295	2.814506	−1.83888	31	O	4.665557	−1.423209	−1.199517
9	O	−0.164672	−0.006093	1.27711	32	O	4.679145	1.38972	−1.165955
10	Al	−0.382133	−1.65928	2.305498	33	Al	0.73342	0.006822	4.685916
11	Al	−0.404559	1.638221	2.302584	34	O	1.502682	−0.024844	6.198302
12	Al	0.334722	−0.003677	−0.460609	35	H	1.695413	0.768095	6.70123
13	Al	−2.046651	1.306631	−1.763616	36	Al	5.629428	−0.016841	−1.335299
14	Al	−2.038556	−1.274962	−1.80771	37	O	7.295818	0.033127	−1.598976
15	O	2.165244	−0.008531	−0.690804	38	H	7.863441	−0.732161	−1.701075
16	O	2.034221	−2.944421	−0.838551	39	O	−2.165903	1.436873	2.193832

17	O	2.054397	2.923231	−0.787957	40	O	−2.147196	−1.484643	2.200678
18	Al	0.34394	3.112917	−0.491857	41	O	−2.553256	0.003374	−0.507665
19	O	−0.559388	4.406925	−1.173812	42	Al	−2.50793	−0.028678	1.261739
20	H	−2.716334	6.648662	−2.040703	43	N	−4.510209	−0.047416	1.495778
21	O	−0.607531	−4.396143	−1.154256	44	H	−4.930308	0.786556	1.092159
22	H	−2.80113	−6.604198	−1.91101	45	H	−4.757656	−0.082571	2.481479
23	Al	−2.220827	−4.336493	−1.712143	46	H	−4.922915	−0.856099	1.036806

$TMSNH_2$@[AlO_3]*clus*

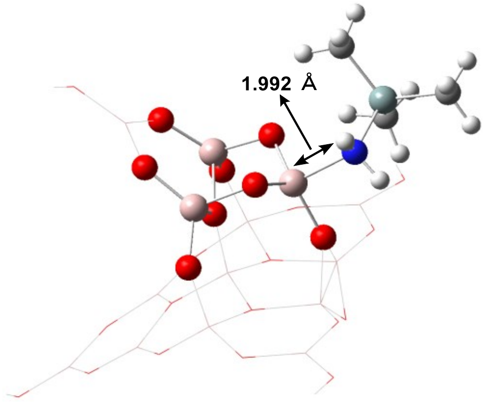

APFD/6-31G(d,p):
Electronic Energy (*EE*) = −5666.691257 Hartree
Thermal Correction to Free Energy = 0.103556 Hartree
Gibbs free energy (*G*) = −5666.587702 Hartree

APFD/6-31+G(2d,p)//APFD/6-31G(d,p):
Electronic Energy (*EE*) = −5666.974252 Hartree
Gibbs free energy (*G*) = −5666.870696 Hartree

1	O	0.471623	1.217146	3.855471	30	Al	4.026268	0.777936	−0.252492
2	O	−0.647787	0.678261	−3.345857	31	O	4.872317	−2.542664	−0.484901
3	O	0.883626	1.597729	−1.118359	32	O	5.595489	0.163065	−0.220448
4	O	0.126674	−1.277956	−1.386048	33	Al	0.321205	−0.288013	4.648204
5	O	1.155372	2.765285	1.391821	34	O	0.732186	−0.566791	6.271003
6	O	−0.272311	−2.832579	0.926863	35	H	1.014087	0.131456	6.864151
7	O	−2.418127	−1.820611	−2.722923	36	Al	6.169636	−1.440163	−0.321166
8	O	−1.005036	3.554115	−2.182182	37	O	7.811249	−1.827824	−0.260315
9	O	0.16334	0.036638	1.141556	38	H	8.173481	−2.713292	−0.315308
10	Al	−0.67318	−1.528053	1.972217	39	O	−1.552307	1.929009	1.783988
11	Al	0.146888	1.655385	2.23376	40	O	−2.274826	−0.870219	1.57334
12	Al	0.989925	−0.043807	−0.464425	41	O	−1.731523	0.730068	−1.055105
13	Al	−0.661522	1.889842	−2.082715	42	Al	−2.116998	0.655397	0.679626

14	Al	−1.303032	−0.598404	−2.334541	43	H	−4.094578	1.630428	−0.419498
15	O	2.765935	−0.528951	−0.344251	44	N	−4.025859	1.195356	0.500764
16	O	1.923667	−3.318615	−0.752643	45	H	−4.169335	1.943394	1.179848
17	O	3.428405	2.328153	−0.221536	46	Si	−5.319084	−0.100102	0.701794
18	Al	1.79964	2.957519	−0.239473	47	C	−6.895984	0.645856	0.012047
19	O	1.414587	4.464324	−0.972136	48	H	−7.727666	−0.05749	0.126186
20	H	0.123842	7.223182	−2.045446	49	H	−7.171732	1.569611	0.531063
21	O	−0.879271	−4.005352	−1.678893	50	H	−6.802048	0.868302	−1.05609
22	H	−3.361584	−5.527371	−3.013675	51	C	−4.777129	−1.595271	−0.271813
23	Al	−2.275398	−3.502533	−2.524779	52	H	−3.963312	−2.097717	0.259742
24	Al	−0.009561	4.885537	−1.811964	53	H	−5.61318	−2.295216	−0.375465
25	O	−3.434606	−4.580967	−3.149676	54	H	−4.425855	−1.342309	−1.277552
26	O	−0.422773	6.469028	−2.2725	55	C	−5.418041	−0.400523	2.54094
27	Al	0.198227	−3.030558	−0.761604	56	H	−6.166538	−1.168584	2.760873
28	O	−0.21846	−1.532835	3.622294	57	H	−4.448502	−0.752115	2.904288
29	Al	3.205393	−2.282515	−0.542242	58	H	−5.698834	0.503219	3.091574

Section 9.1: ^{1}H NMR assignment for *N*−H ketimine

Structure optimization, frequency, and NMR (GIAO method) calculations were performed at the APFD/6-311+G(2d,p) level in $CDCl_3$ or DMSO (SMD method).

***E* isomer (2j)**

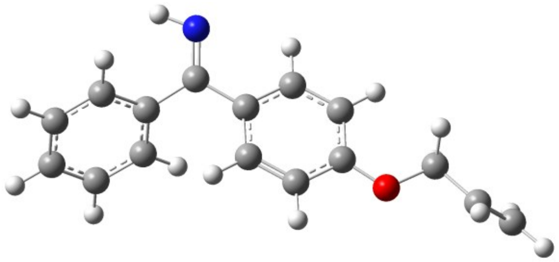

Solvent: DMSO
Electronic Energy: (*EE*) = −748.27335 Hartree
Thermal Correction to Free Energy = 0.224517 Hartree
Gibbs free energy: (*G*) = −748.048833 Hartree
Chemical shift of imino proton: δ = 9.3697 ppm

1	C	3.912488	−1.794336	0.914792	18	N	2.066153	2.515884	0.152332
2	C	5.065088	−1.559162	0.172757	19	H	3.837834	−2.679345	1.539045
3	C	5.157059	−0.420511	−0.620931	20	H	5.88942	−2.264704	0.209507
4	C	4.105277	0.484635	−0.663751	21	H	6.049644	−0.238696	−1.21149
5	C	2.944183	0.253896	0.07601	22	H	4.176206	1.369867	−1.288988
6	C	2.852226	−0.899661	0.857054	23	H	1.951969	−1.087655	1.433288
7	C	1.845472	1.257107	0.066958	24	H	0.927606	−1.014611	−1.140042
8	C	0.452227	0.775628	−0.036518	25	H	−1.416169	−1.745523	−1.363554
9	C	0.136784	−0.414322	−0.702654	26	H	−2.693446	1.71272	0.847558
10	C	−1.173477	−0.829785	−0.834304	27	H	−0.362538	2.442286	1.034915
11	C	−2.210395	−0.071823	−0.283229	28	H	−4.539204	1.187803	−0.383463
12	C	−1.912006	1.11332	0.396923	29	H	−4.4708	0.283199	1.148454
13	C	−0.592178	1.523643	0.505286	30	H	−5.948693	−0.719598	−1.377396
14	O	−3.453064	−0.569882	−0.452136	31	H	−7.650785	−1.377005	0.255419
15	C	−4.555025	0.184276	0.060994	32	H	−6.601319	−0.709728	1.629761
16	C	−5.806909	−0.528147	−0.314946	33	H	3.065911	2.694472	0.257634
17	C	−6.729845	−0.892361	0.565579					

***Z* isomer (2j)**

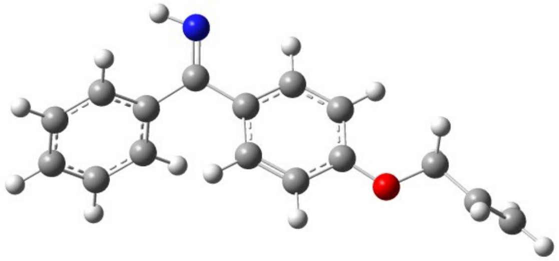

Solvent: DMSO
Electronic Energy: (*EE*) = −748.273154 Hartree
Thermal Correction to Free Energy = 0.224295 Hartree
Gibbs free energy: (*G*) = −748.048859 Hartree
Chemical shift of imino proton: δ = 9.6446 ppm

1	C	−3.829052	−1.879768	−0.819898	18	N	−2.189803	2.496869	−0.09534
2	C	−5.045006	−1.598365	−0.207264	19	H	−3.690701	−2.81562	−1.352387
3	C	−5.21741	−0.393714	0.468353	20	H	−5.856976	−2.317717	−0.252996
4	C	−4.180769	0.526669	0.524451	21	H	−6.16224	−0.173922	0.955969
5	C	−2.954651	0.24966	−0.083287	22	H	−4.308511	1.467274	1.049856
6	C	−2.785666	−0.96563	−0.749286	23	H	−1.838062	−1.190205	−1.227907
7	C	−1.865498	1.259279	−0.044447	24	H	−0.907739	−0.86515	1.369292
8	C	−0.46631	0.782233	0.051555	25	H	1.444425	−1.569069	1.608892
9	C	−0.129685	−0.323469	0.841468	26	H	2.646779	1.587357	−1.049752
10	C	1.184125	−0.723219	0.980865	27	H	0.321413	2.29774	−1.253754
11	C	2.202776	−0.040624	0.309111	28	H	4.530531	1.21551	0.185628
12	C	1.882045	1.052437	−0.500353	29	H	4.41781	0.133633	−1.223696
13	C	0.558353	1.453641	−0.612552	30	H	5.969593	−0.567669	1.35865
14	O	3.450525	−0.519028	0.497932	31	H	7.633867	−1.397886	−0.23409
15	C	4.535018	0.165795	−0.135281	32	H	6.545024	−0.89357	−1.646792
16	C	5.799503	−0.49762	0.285459	33	H	−1.360105	3.087673	−0.020709
17	C	6.702114	−0.954678	−0.572608					

***E* isomer (2m)**

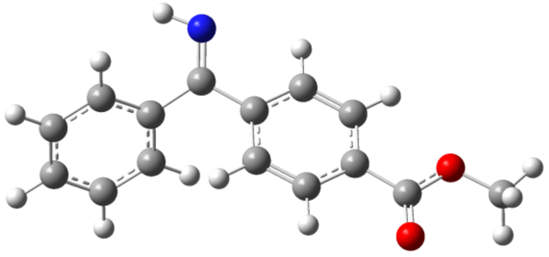

Solvent: DMSO
Electronic Energy: (*EE*) = −784.227192 Hartree
Thermal Correction to Free Energy = 0.201755 Hartree
Gibbs free energy: (*G*) = −784.025437 Hartree
Chemical shift of imino proton: δ = 10.0903 ppm

1	C	−1.332157	−0.847245	−0.727512	17	H	0.761508	−1.023736	−1.138889
2	C	−2.017501	1.137586	0.457252	18	C	3.980734	0.473088	−0.711619
3	C	0.308983	0.802045	−0.091705	19	H	4.06203	1.299004	−1.412201
4	C	1.709251	1.298162	−0.084268	20	C	2.703842	−0.776324	0.90471
5	N	1.903986	2.557517	−0.173598	21	H	1.795652	−0.914699	1.482487
6	H	−1.590262	−1.787027	−1.202458	22	C	4.924486	−1.492225	0.310165
7	H	2.902011	2.770717	−0.129498	23	H	5.746621	−2.193009	0.417708
8	C	2.809198	0.306209	0.029108	24	C	−2.339003	−0.077016	−0.150221
9	C	3.761388	−1.66338	1.053042	25	C	−3.731291	−0.591846	−0.204544
10	C	5.029882	−0.425343	−0.576352	26	O	−4.0451	−1.635885	−0.732191
11	H	3.676075	−2.492464	1.748528	27	O	−4.599806	0.226963	0.394188
12	H	5.930654	−0.295641	−1.168078	28	C	−5.967178	−0.198044	0.383579
13	H	−2.794528	1.73815	0.914852	29	H	−6.0771	−1.149202	0.906876
14	C	−0.70418	1.573874	0.479177	30	H	−6.520431	0.582327	0.902147
15	H	−0.449536	2.516288	0.951393	31	H	−6.32904	−0.299398	−0.640724
16	C	−0.016839	−0.416634	−0.689635					

***Z* isomer (2m)**

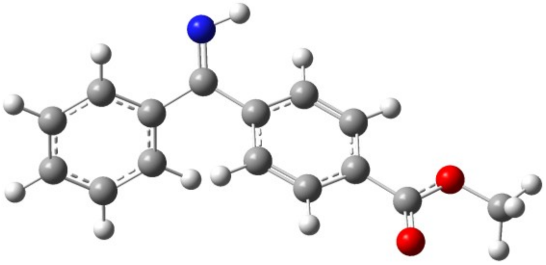

Solvent: DMSO
Electronic Energy: (*EE*) = −784.227134 Hartree
Thermal Correction to Free Energy = 0.20159 Hartree
Gibbs free energy: (*G*) = −784.025544 Hartree
Chemical shift of imino proton: δ = 9.9208 ppm

1	C	−3.691453	−1.747721	−0.9735	17	H	−1.689792	−1.039568	−1.280982
2	C	−5.097863	−0.370698	0.413617	18	C	0.658529	1.491289	−0.593151
3	C	−2.821322	0.301076	−0.03304	19	H	0.384312	2.364558	−1.176771
4	C	−1.725533	1.287629	0.122282	20	C	0.036548	−0.344252	0.838041
5	N	−2.01783	2.522679	0.268945	21	H	−0.720148	−0.895791	1.385748
6	H	−3.547358	−2.633998	−1.583444	22	C	2.332719	−0.061443	0.168384

7	H	−1.170798	3.079765	0.393514	23	C	−4.917646	−1.51243	−0.362442
8	C	−0.322432	0.792649	0.111352	24	H	−5.732082	−2.21948	−0.486972
9	C	1.356212	−0.759479	0.876425	25	C	3.731264	−0.557962	0.231002
10	C	1.976211	1.065023	−0.57229	26	O	4.56652	0.180662	−0.503682
11	H	1.642125	−1.63218	1.452686	27	O	4.076461	−1.52335	0.875653
12	H	2.727388	1.60616	−1.134941	28	C	5.937009	−0.234463	−0.495787
13	H	−6.051365	−0.188345	0.899626	29	H	6.032991	−1.24558	−0.894402
14	C	−4.058305	0.533272	0.572496	30	H	6.461298	0.473019	−1.134983
15	H	−4.191157	1.423362	1.1782	31	H	6.341952	−0.199662	0.51669
16	C	−2.645007	−0.850743	−0.802116					

***E* isomer (2n)**

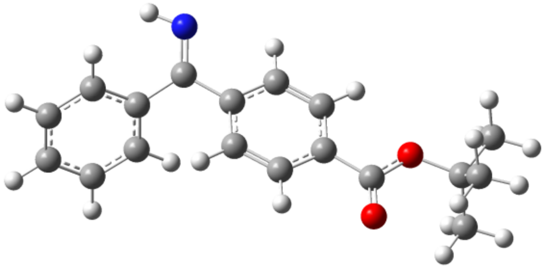

Solvent: DMSO
Electronic Energy: (*EE*) = −902.116483 Hartree
Thermal Correction to Free Energy = 0.280186 Hartree
Gibbs free energy: (*G*) = −901.836297 Hartree
Chemical shift of imino proton: δ = 10.0513 ppm

1	C	−0.258241	−0.828845	−0.834967	21	H	2.798111	−0.915864	1.475552
2	C	−0.974393	1.144857	0.346819	22	C	5.959255	−1.500289	0.397083
3	C	1.36702	0.811151	−0.136853	23	H	6.774931	−2.204944	0.526281
4	C	2.767163	1.30382	−0.083269	24	C	−1.281052	−0.063335	−0.280333
5	N	2.968619	2.563114	−0.159785	25	C	−2.676067	−0.579915	−0.378465
6	H	−0.50458	−1.763662	−1.325712	26	O	−2.95032	−1.630549	−0.916764
7	H	3.965456	2.77243	−0.08262	27	O	−3.545136	0.24984	0.19046
8	C	3.86028	0.307869	0.059821	28	C	−4.988072	−0.042923	0.224658
9	C	4.773345	−1.67006	1.103317	29	C	−5.54634	−0.117399	−1.187982
10	C	6.095732	−0.429852	−0.480847	30	H	−5.180314	−0.993261	−1.72267
11	H	4.663644	−2.501864	1.792096	31	H	−6.636502	−0.174682	−1.133161
12	H	7.014604	−0.301023	−1.044253	32	H	−5.282186	0.780539	−1.753194
13	H	−1.764536	1.741008	0.787277	33	C	−5.55175	1.162669	0.954693
14	C	0.338497	1.57912	0.411246	34	H	−6.635562	1.063413	1.047309
15	H	0.580791	2.516487	0.899948	35	H	−5.126322	1.243785	1.958309
16	C	1.056412	−0.401183	−0.755383	36	H	−5.335646	2.084289	0.408126

17	H	1.846545	−1.005617	−1.187562	37	C	−5.249278	−1.314384	1.017037
18	C	5.054686	0.473442	−0.643765	38	H	−4.8805	−2.198413	0.49782
19	H	5.160557	1.301926	−1.337941	39	H	−4.77663	−1.25694	2.001324
20	C	3.72419	−0.778189	0.926786	40	H	−6.326857	−1.423058	1.164428

Z **isomer (2n)**

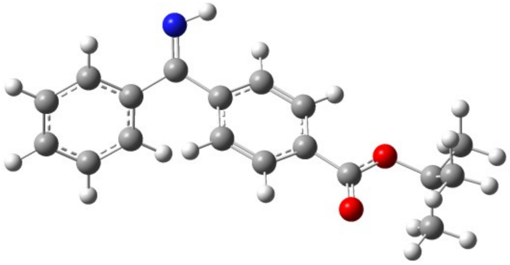

Solvent: DMSO
Electronic Energy: (*EE*) = −902.116476 Hartree
Thermal Correction to Free Energy = 0.280663 Hartree
Gibbs free energy: (*G*) = −901.835813 Hartree
Chemical shift of imino proton: δ = 9.8988 ppm

1	C	4.688736	−1.7691	1.021968	21	H	1.816254	−0.874868	−1.43926
2	C	6.159574	−0.378799	−0.282423	22	C	−1.278432	−0.032754	−0.335929
3	C	3.86799	0.296587	0.073649	23	C	5.941397	−1.530586	0.468654
4	C	2.784106	1.29074	−0.115048	24	H	6.746969	−2.24286	0.618413
5	N	3.089328	2.525703	−0.235089	25	C	−2.68089	−0.523097	−0.459396
6	H	4.514529	−2.663303	1.612171	26	O	−3.521774	0.217621	0.255818
7	H	2.251119	3.089001	−0.388063	27	O	−2.983216	−1.481466	−1.136689
8	C	1.379212	0.80447	−0.166003	28	C	−4.966307	−0.067413	0.293594
9	C	−0.278157	−0.729283	−1.011047	29	C	−5.56894	0.077485	−1.095095
10	C	−0.94361	1.084624	0.42796	30	H	−5.229078	−0.709008	−1.768147
11	H	−0.54705	−1.59449	−1.606506	31	H	−6.657458	0.019195	−1.014859
12	H	−1.714477	1.624379	0.964515	32	H	−5.31228	1.049392	−1.525264
13	H	7.134165	−0.193677	−0.723459	33	C	−5.218062	−1.442493	0.892288
14	C	5.131267	0.531732	−0.473731	34	H	−6.291671	−1.563304	1.058115
15	H	5.29399	1.429913	−1.059883	35	H	−4.878115	−2.240282	0.232734
16	C	3.654235	−0.865071	0.818096	36	H	−4.713181	−1.539408	1.857288
17	H	2.678457	−1.056328	1.252383	37	C	−5.491957	1.017241	1.216281
18	C	0.37478	1.502867	0.504973	38	H	−6.573514	0.913327	1.328082
19	H	0.631063	2.369384	1.10669	39	H	−5.034533	0.940888	2.206096
20	C	1.041879	−0.323289	−0.916782	40	H	−5.281486	2.009726	0.809498

***E* isomer (2s)**

K = exp(−Δ*G*/RT) = E_2/E_1 = 0.979040415 (R: 1.9872×10^{-3} kcal·mol^{-1}·K^{-1}, T: 298.15 K)

In DMSO	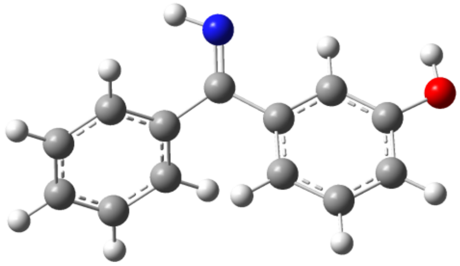 ***E*1**	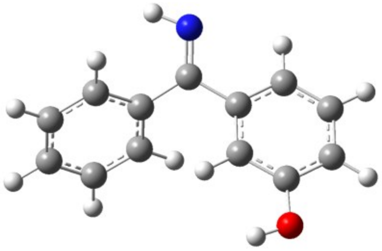 ***E*2**
Ratio	0.505295391	0.494704609
$G_{average}$	−631.4796549 Hartree	
$\delta_{average}$	9.7798 ppm	

E_1 isomer
Solvent: DMSO
Electronic Energy: (*EE*) = −631.648753 Hartree
Thermal Correction to Free Energy = 0.169088 Hartree
Gibbs free energy: (*G*) = −631.479665 Hartree
Chemical shift of imino proton: δ = 9.7705 ppm

1	C	−2.085598	−1.71616	−0.896981	14	C	1.61901	0.399645	0.019522
2	C	−3.274682	−1.217379	−0.387937	15	C	1.770687	−0.683141	0.887756
3	C	−3.287979	0.033569	0.226806	16	C	2.725167	0.858252	−0.698174
4	C	−2.116869	0.776817	0.322257	17	C	3.01254	−1.281773	1.051955
5	C	−0.921911	0.267753	−0.185767	18	H	0.915065	−1.04738	1.446994
6	C	−0.906381	−0.988132	−0.79232	19	C	3.961791	0.245639	−0.548501
7	C	0.310262	1.092879	−0.106907	20	H	2.611153	1.68604	−1.391974
8	N	0.199784	2.365781	−0.147069	21	C	4.109205	−0.822299	0.33057
9	H	−2.078622	−2.688234	−1.379959	22	H	3.123596	−2.112058	1.742411
10	H	−4.195856	−1.787133	−0.455419	23	H	4.812066	0.599471	−1.1232
11	H	−2.122365	1.752769	0.799516	24	H	5.077327	−1.298807	0.45006
12	H	0.017807	−1.387764	−1.193871	25	O	−4.476349	0.472092	0.7179
13	H	1.116693	2.80611	−0.054571	26	H	−4.356016	1.345752	1.112623

E_2 isomer
Solvent: DMSO
Electronic Energy: (*EE*) = −631.648512 Hartree
Thermal Correction to Free Energy = 0.168867 Hartree
Gibbs free energy: (*G*) = −631.479645 Hartree
Chemical shift of imino proton: δ = 9.789 ppm

1	C	−2.526642	−1.114436	−0.342562	14	C	1.585246	−0.518042	1.005236
2	C	−3.568256	−0.361168	0.190962	15	C	2.617269	0.595394	−0.865124
3	C	−3.323366	0.935527	0.622292	16	C	2.723387	−1.296179	1.168434
4	C	−2.055216	1.490527	0.52237	17	H	0.741168	−0.644519	1.675463
5	C	−1.009634	0.733908	−0.006885	18	C	3.746238	−0.197989	−0.714156
6	C	−1.247084	−0.572831	−0.431848	19	H	2.571029	1.325685	−1.667722
7	C	0.343356	1.33089	−0.143288	20	C	3.803819	−1.141476	0.306366
8	N	0.438142	2.585123	−0.370009	21	H	2.76569	−2.028291	1.968901
9	H	−4.558535	−0.798833	0.265776	22	H	4.58179	−0.082847	−1.397556
10	H	−1.863582	2.50226	0.860902	23	H	4.688533	−1.759033	0.426727
11	H	−0.438736	−1.16779	−0.847736	24	O	−2.815581	−2.374981	−0.758426
12	H	1.419037	2.86734	−0.408275	25	H	−2.013658	−2.790973	−1.100988
13	C	1.527747	0.443182	−0.005795	26	H	−4.136258	1.517152	1.045964

***Z* isomer (2s)**

K = exp(−Δ*G*/RT) = Z_2/Z_1 = 0.891914752 (R: 1.9872×10^{-3} kcal・mol^{-1}・K^{-1}, T: 298.15 K)

In DMSO	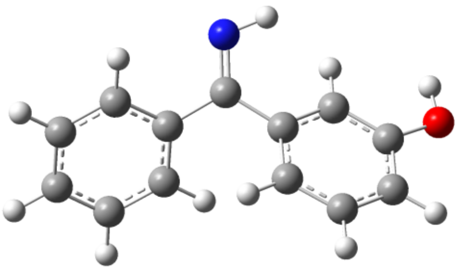 $\boldsymbol{Z_1}$	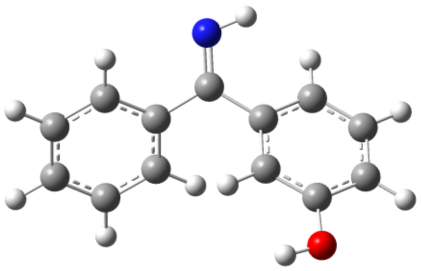 $\boldsymbol{Z_2}$
Ratio	0.528565042	0.471434958
$G_{average}$	−631.4798079 Hartree	
$\delta_{average}$	9.7389 ppm	

<u>Z_1 isomer</u>
Solvent: DMSO
Electronic Energy: (*EE*) = −631.648561 Hartree
Thermal Correction to Free Energy = 0.168696 Hartree
Gibbs free energy: (*G*) = −631.479865 Hartree
Chemical shift of imino proton: δ = 9.7152 ppm

1	C	2.939608	−1.389415	0.98013	14	C	−0.947081	−0.904672	−0.95751
2	C	4.090954	−0.863395	0.405403	15	C	−2.06055	0.745294	0.407517
3	C	4.013542	0.291016	−0.368765	16	C	−2.139518	−1.610443	−1.058047
4	C	2.791938	0.919264	−0.560177	17	H	−0.053709	−1.266221	−1.454166
5	C	1.629797	0.394678	0.009675	18	C	−3.244905	0.019618	0.316652
6	C	1.713516	−0.769739	0.775342	19	H	−2.030677	1.660188	0.994586
7	C	0.335826	1.097555	−0.173235	20	C	−3.287064	−1.15982	−0.42316
8	N	0.341577	2.371217	−0.28137	21	H	−2.176191	−2.527761	−1.637276
9	H	5.047649	−1.353962	0.55661	22	H	−4.217645	−1.714341	−0.487313
10	H	2.724587	1.818697	−1.16302	23	H	4.908881	0.699652	−0.827058
11	H	0.817764	−1.184846	1.225282	24	H	2.994871	−2.286885	1.588287
12	H	−0.605395	2.722078	−0.433698	25	O	−4.39078	0.408636	0.93272
13	C	−0.909252	0.284962	−0.230661	26	H	−4.235804	1.236376	1.406612

<u>Z_2 isomer</u>
Solvent: DMSO
Electronic Energy: (*EE*) = −631.648672 Hartree
Thermal Correction to Free Energy = 0.168915 Hartree
Gibbs free energy: (*G*) = −631.479757 Hartree
Chemical shift of imino proton: δ = 9.7601 ppm

1	C	2.67306	−1.397478	1.1006	14	C	−1.288744	−0.540688	−0.523123
2	C	3.822142	−1.156872	0.356232	15	C	−1.965447	1.445082	0.669845
3	C	3.838627	−0.125568	−0.578791	16	C	−2.573728	−1.058702	−0.388404
4	C	2.713446	0.663959	−0.762974	17	H	−0.526921	−1.112009	−1.046341
5	C	1.553261	0.426273	−0.022484	18	C	−3.239063	0.910075	0.815821
6	C	1.541092	−0.616489	0.905961	19	H	−1.726416	2.414319	1.095256
7	C	0.368418	1.300456	−0.205983	20	C	−3.551887	−0.334056	0.288023
8	N	0.551371	2.522338	−0.53351	21	H	−4.546416	−0.754388	0.39716
9	H	4.703549	−1.77406	0.501043	22	H	4.731283	0.059912	−1.168221
10	H	2.719113	1.468137	−1.491102	23	H	2.656311	−2.197602	1.834083
11	H	0.646609	−0.808917	1.489082	24	H	−4.000171	1.467133	1.353233
12	H	−0.344978	2.996213	−0.656105	25	O	−2.929916	−2.2679	−0.893508
13	C	−0.985273	0.71708	−0.003947	26	H	−2.167657	−2.670338	−1.3301

E **isomer (2t)**

K = exp(−Δ*G*/RT) = E_2/E_1 = 0.842335338 (R: 1.9872×10^{-3} kcal·mol^{-1}·K^{-1}, T: 298.15 K)

In DMSO	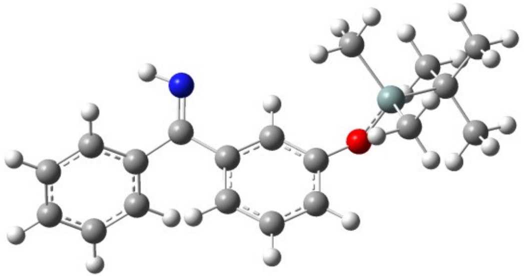 ***E*₁**	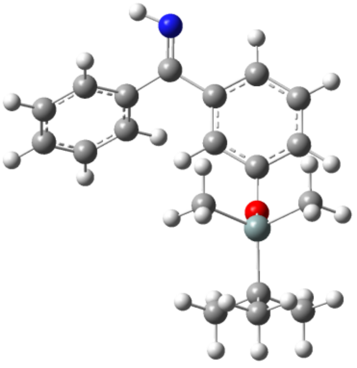 ***E*₂**
Ratio	0.54278935	0.45721065
$G_{average}$	−1157.757078 Hartree	
$\delta_{average}$	9.7471 ppm	

E_1 isomer
Solvent: DMSO
Electronic Energy: (*EE*) = −1158.094931 Hartree
Thermal Correction to Free Energy = 0.337765 Hartree
Gibbs free energy: (*G*) = −1157.757166 Hartree
Chemical shift of imino proton: δ = 9.7577 ppm

1	C	−0.859559	2.421435	−0.650741	25	O	1.979977	0.496913	0.593145
2	C	0.432337	2.103761	−0.255043	26	Si	3.160931	−0.110086	−0.450186
3	C	0.727735	0.806429	0.165093	27	C	2.540506	−1.696063	−1.207636
4	C	−0.271248	−0.159293	0.184242	28	H	2.325632	−2.454	−0.449924
5	C	−1.569838	0.161513	−0.209029	29	H	1.625423	−1.516639	−1.780378
6	C	−1.86353	1.46192	−0.622309	30	H	3.286341	−2.103626	−1.897338
7	C	−2.609228	−0.898191	−0.220131	31	C	3.4838	1.150697	−1.784485
8	N	−2.253104	−2.104086	−0.451965	32	H	3.805082	2.109376	−1.369114
9	H	−1.086032	3.430064	−0.982346	33	H	4.263348	0.794193	−2.465086
10	H	1.219994	2.850213	−0.263602	34	H	2.580475	1.321045	−2.378386
11	H	−0.037363	−1.16519	0.514276	35	C	4.650521	−0.37737	0.67489
12	H	−2.869651	1.719405	−0.933934	36	C	5.79738	−0.967511	−0.150759
13	H	−3.058946	−2.72951	−0.40036	37	H	6.099701	−0.304622	−0.968277
14	C	−4.020213	−0.511384	0.042535	38	H	6.678469	−1.123581	0.48534
15	C	−4.334993	0.390586	1.060688	39	H	5.533647	−1.937414	−0.585061
16	C	−5.051979	−1.087934	−0.700516	40	C	4.279828	−1.347315	1.799073
17	C	−5.659997	0.692514	1.345104	41	H	5.143023	−1.509043	2.458251
18	H	−3.538638	0.844757	1.641262	42	H	3.461897	−0.962488	2.415518

19	C	−6.375431	−0.769946	−0.428356	43	H	3.977067	−2.325984	1.413197
20	H	−4.814635	−1.773907	−1.508437	44	C	5.090517	0.9587	1.27771
21	C	−6.682447	0.116777	0.598565	45	H	5.949352	0.807293	1.944978
22	H	−5.894934	1.381684	2.150241	46	H	5.398308	1.673816	0.508189
23	H	−7.169508	−1.211942	−1.022076	47	H	4.293697	1.422437	1.867234
24	H	−7.717847	0.36222	0.81366					

E_2 isomer
Solvent: DMSO
Electronic Energy: (*EE*) = −1158.095216 Hartree
Thermal Correction to Free Energy = 0.338212 Hartree
Gibbs free energy: (*G*) = −1157.757004 Hartree
Chemical shift of imino proton: δ = 9.7382 ppm

1	C	0.54336	1.227842	−0.599386	25	O	1.587638	0.371082	−0.747646
2	C	0.699858	2.579366	−0.897697	26	Si	2.59805	−0.045073	0.539844
3	C	−0.382621	3.442268	−0.771313	27	C	1.586707	−0.886073	1.86082
4	C	−1.614924	2.971696	−0.342487	28	H	0.822556	−0.207633	2.252031
5	C	−1.777625	1.616685	−0.04835	29	H	1.087919	−1.782904	1.484375
6	C	−0.696771	0.747863	−0.188661	30	H	2.227844	−1.177683	2.698727
7	C	−3.085117	1.113621	0.442082	31	C	3.364158	1.512403	1.218864
8	N	−3.826872	1.898111	1.126864	32	H	4.037775	1.276334	2.048585
9	H	1.668114	2.940565	−1.228845	33	H	3.934825	2.050996	0.457899
10	H	−2.460381	3.643283	−0.244332	34	H	2.58985	2.183535	1.603579
11	H	−0.801752	−0.307933	0.034282	35	C	3.863474	−1.200398	−0.247451
12	H	−4.703576	1.435651	1.372932	36	C	3.148607	−2.407815	−0.858405
13	C	−3.47294	−0.283942	0.114897	37	H	2.437705	−2.110102	−1.635084
14	C	−3.274558	−0.797526	−1.168195	38	H	3.878982	−3.08506	−1.320606
15	C	−4.078808	−1.085468	1.083877	39	H	2.601387	−2.984104	−0.105517
16	C	−3.695966	−2.082571	−1.481865	40	C	4.840399	−1.677611	0.831085
17	H	−2.800466	−0.182124	−1.92591	41	H	5.590166	−2.347898	0.390779
18	C	−4.481508	−2.377456	0.774626	42	H	5.380494	−0.845292	1.294407
19	H	−4.216354	−0.700899	2.090208	43	H	4.334186	−2.23377	1.626872
20	C	−4.295621	−2.876801	−0.510247	44	C	4.633997	−0.455751	−1.339994
21	H	−3.552335	−2.466688	−2.486979	45	H	5.185539	0.4012	−0.940314
22	H	−4.937194	−2.998204	1.539783	46	H	5.366717	−1.125762	−1.808975
23	H	−4.61322	−3.886208	−0.752756	47	H	3.970642	−0.089154	−2.12926
24	H	−0.259722	4.493456	−1.013777					

***Z* isomer (2t)**

K = exp(−Δ*G*/RT) = Z_2/Z_1 = 0.941416415 (R: 1.9872×10^{-3} kcal·mol^{-1}·K^{-1}, T: 298.15 K)

In DMSO	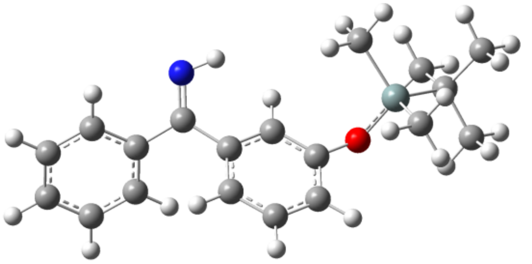 ***Z*1**	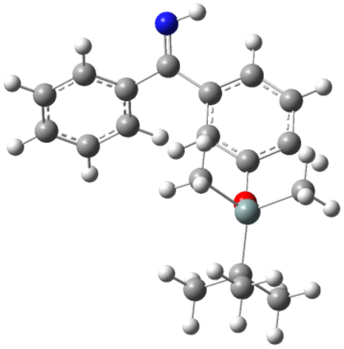 ***Z*2**
Ratio	0.515087846	0.484912154
$G_{average}$	−1157.757104 Hartree	
$\delta_{average}$	9.7495 ppm	

Z_1 isomer

Solvent: DMSO

Electronic Energy: (*EE*) = −1158.095098 Hartree

Thermal Correction to Free Energy = 0.337965 Hartree

Gibbs free energy: (*G*) = −1157.757133 Hartree

Chemical shift of imino proton: δ = 9.7231 ppm

1	C	−5.534956	0.674889	1.455724	25	O	1.944988	0.513492	0.582684
2	C	−6.611638	0.054208	0.832673	26	C	2.517392	−1.703941	−1.185471
3	C	−6.386441	−0.849566	−0.201926	27	H	2.259107	−2.448395	−0.427953
4	C	−5.09067	−1.135348	−0.605719	28	H	1.628535	−1.512129	−1.794439
5	C	−4.00372	−0.512246	0.01124	29	H	3.279654	−2.134809	−1.842299
6	C	−4.238005	0.40112	1.040847	30	C	3.521656	1.122795	−1.757375
7	C	−2.621532	−0.852443	−0.407708	31	H	3.86267	2.074366	−1.341274
8	N	−2.381046	−2.038422	−0.820569	32	H	4.300209	0.742631	−2.426097
9	H	−7.626009	0.276589	1.149468	33	H	2.631108	1.314864	−2.363932
10	H	−4.908434	−1.837591	−1.412263	34	C	4.59668	−0.405349	0.745539
11	H	−3.400944	0.888896	1.529241	35	C	5.765721	−0.995989	−0.047956
12	H	−1.404155	−2.126006	−1.106001	36	H	6.100421	−0.32638	−0.847166
13	C	−1.578071	0.204504	−0.334932	37	H	6.62365	−1.166325	0.615529
14	C	−1.853098	1.509884	−0.744997	38	H	5.509271	−1.958873	−0.501897
15	C	−0.295717	−0.1267	0.098674	39	C	4.18572	−1.380791	1.850892
16	C	−0.842515	2.462177	−0.738008	40	H	5.025403	−1.547932	2.538449
17	H	−2.849591	1.773793	−1.081898	41	H	3.347814	−0.997948	2.441334
18	C	0.710055	0.833964	0.116615	42	H	3.894843	−2.356617	1.449058

19	H	−0.07081	−1.131124	0.442746	43	C	5.024355	0.924353	1.370817
20	C	0.435679	2.133962	−0.306013	44	H	5.863827	0.764445	2.060339
21	H	−1.052141	3.474031	−1.070971	45	H	5.355933	1.644202	0.615741
22	H	1.228162	2.875072	−0.287719	46	H	4.21301	1.386332	1.941478
23	H	−7.225039	−1.329513	−0.696945	47	Si	3.14431	−0.122464	−0.423107
24	H	−5.704793	1.377033	2.265988					

Z_2 isomer

Solvent: DMSO

Electronic Energy: (*EE*) = −1158.095265 Hartree

Thermal Correction to Free Energy = 0.338189 Hartree

Gibbs free energy: (*G*) = −1157.757076 Hartree

Chemical shift of imino proton: δ = 9.7744 ppm

1	C	−3.799644	−2.013325	−1.481134	25	O	1.594697	0.294794	−0.750603
2	C	−4.513993	−2.769337	−0.558604	26	Si	2.684283	0.012613	0.508743
3	C	−4.734902	−2.271487	0.722403	27	C	1.742568	−0.597654	1.997079
4	C	−4.249364	−1.021088	1.075602	28	H	2.431959	−0.797367	2.823564
5	C	−3.526032	−0.257829	0.156856	29	H	1.029454	0.158914	2.338621
6	C	−3.298968	−0.768459	−1.122585	30	H	1.190287	−1.516405	1.783696
7	C	−3.03783	1.090312	0.539672	31	C	3.551354	1.611539	0.915141
8	N	−3.735013	1.784492	1.356	32	H	4.085672	2.015681	0.05139
9	H	−4.89527	−3.747466	−0.835488	33	H	2.830277	2.362388	1.253119
10	H	−4.416602	−0.627826	2.072746	34	H	4.272853	1.460034	1.724134
11	H	−2.739904	−0.183139	−1.845124	35	C	3.848205	−1.290856	−0.19921
12	H	−3.269095	2.67061	1.557928	36	C	3.054694	−2.548826	−0.559681
13	C	−1.75322	1.56885	−0.036106	37	H	2.280541	−2.342772	−1.305044
14	C	−0.672085	0.697363	−0.153109	38	H	3.724711	−3.310087	−0.980734
15	C	−1.609802	2.902716	−0.421514	39	H	2.568558	−2.991322	0.315632
16	C	0.552273	1.156617	−0.628015	40	C	4.536404	−0.744123	−1.451965
17	H	−0.766031	−0.342919	0.13838	41	H	5.213225	−1.500296	−1.871335
18	C	−0.392476	3.351072	−0.91556	42	H	3.814954	−0.479868	−2.231023
19	H	−2.455234	3.580509	−0.35759	43	H	5.136595	0.145148	−1.234419
20	C	0.691299	2.488063	−1.016176	44	C	4.905326	−1.638156	0.853069
21	H	1.647034	2.832146	−1.397862	45	H	4.458526	−2.045651	1.76595
22	H	−5.284347	−2.86267	1.448559	46	H	5.593454	−2.397741	0.459623
23	H	−3.628812	−2.395002	−2.482902	47	H	5.507675	−0.767768	1.133504
24	H	−0.285765	4.384133	−1.231896					

E isomer (2ab)

K = exp(−Δ*G*/RT) = E_2/E_1 = 5.614221118 (R: 1.9872×10^{-3} kcal·mol^{-1}·K^{-1}, T: 298.15 K)

In DMSO	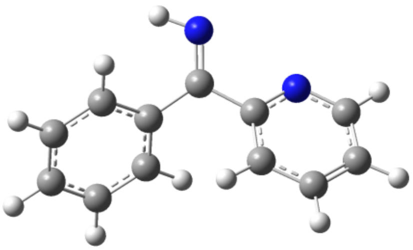 ***E*1**	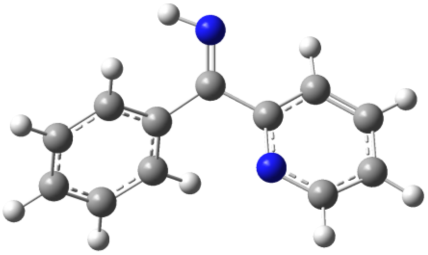 ***E*2**
Ratio	0.151189381	0.848810619
$G_{average}$	−572.3252653 Hartree	
$\delta_{average}$	10.2302 ppm	

<u>E_1 isomer</u>
Solvent: DMSO
Electronic Energy: (*EE*) = −572.479159 Hartree
Thermal Correction to Free Energy = 0.15414 Hartree
Gibbs free energy: (*G*) = −572.325019 Hartree
Chemical shift of imino proton: δ = 10.227 ppm

1	C	2.598285	−1.454959	0.85179	13	C	−1.383081	−0.766887	−0.817943
2	C	3.686441	−0.935965	0.169163	14	C	−2.348547	0.857599	0.676434
3	C	3.50683	0.232068	−0.563523	15	C	−2.597995	−1.436371	−0.873559
4	C	1.302087	0.378112	0.019137	16	H	−0.535381	−1.13708	−1.385659
5	C	1.381836	−0.793677	0.767537	17	C	−3.556986	0.17649	0.634987
6	C	0.025439	1.151212	−0.040181	18	H	−2.248801	1.745054	1.294454
7	N	0.091861	2.424467	−0.065124	19	C	−3.685608	−0.969135	−0.143726
8	H	2.690571	−2.362525	1.439576	20	H	−2.694704	−2.327604	−1.485621
9	H	4.656131	−1.420909	0.195251	21	H	−4.399486	0.5371	1.216794
10	H	0.508043	−1.171979	1.285436	22	H	−4.631761	−1.500309	−0.177374
11	H	−0.845053	2.82889	−0.118373	23	H	4.3357	0.662356	−1.120109
12	C	−1.251272	0.392604	−0.051021	24	N	2.35102	0.88417	−0.637583

<u>E_2 isomer</u>
Solvent: DMSO
Electronic Energy: (*EE*) = −572.480799 Hartree
Thermal Correction to Free Energy = 0.154151 Hartree
Gibbs free energy: (*G*) = −572.326648 Hartree
Chemical shift of imino proton: δ = 10.2483 ppm

1	C	−2.433907	−1.441103	−0.82275	13	C	2.593546	−1.447859	0.851459

2	C	−3.622186	−1.03279	−0.230606	14	H	0.52184	−1.170217	1.33553
3	C	−3.62399	0.148391	0.497318	15	C	3.565717	0.193144	−0.617461
4	C	−1.305109	0.381656	−0.031762	16	H	2.258278	1.765698	−1.269203
5	C	−0.025919	1.144984	0.030198	17	C	3.689651	−0.963414	0.144898
6	N	−0.095722	2.418584	0.075653	18	H	2.686445	−2.348631	1.450112
7	H	−2.395657	−2.360187	−1.401801	19	H	4.41453	0.568445	−1.180532
8	H	−4.519105	−1.63341	−0.335816	20	H	4.638262	−1.489829	0.184618
9	H	0.837844	2.828339	0.139922	21	H	−4.528398	0.498964	0.984266
10	C	1.248782	0.385608	0.038246	22	C	−2.446962	0.872919	0.595417
11	C	1.375643	−0.785431	0.788048	23	H	−2.398232	1.800832	1.152783
12	C	2.353517	0.868215	−0.664985	24	N	−1.294066	−0.76229	−0.722511

***Z* isomer (2ab)**

K = exp(−ΔG/RT) = Z_2/Z_1 = 0.219676879 (R: 1.9872×10^{-3} kcal·mol^{-1}·K^{-1}, T: 298.15 K)

In DMSO	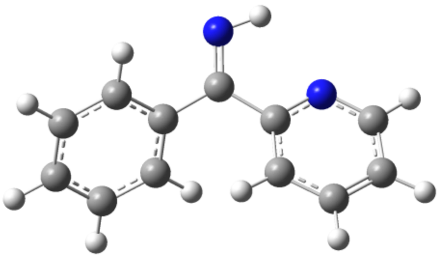 ***Z*1**	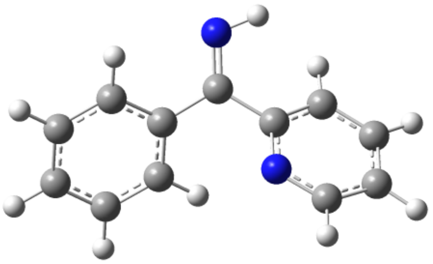 ***Z*2**
Ratio	0.819889281	0.180110719
$G_{average}$	−572.3263977 Hartree	
$\delta_{average}$	10.2453 ppm	

<u>Z_1 isomer</u>
Solvent: DMSO
Electronic Energy: (*EE*) = −572.481816 Hartree
Thermal Correction to Free Energy = 0.154245 Hartree
Gibbs free energy: (*G*) = −572.327571 Hartree
Chemical shift of imino proton: δ = 11.057 ppm

1	C	2.686774	−1.41627	0.814995	13	H	−0.468197	−1.368923	−1.034209
2	C	3.749465	−0.89154	0.087837	14	C	−3.54673	0.307903	0.422333
3	C	3.574799	0.274371	−0.651569	15	C	−3.70771	−0.934961	−0.181703
4	C	1.270361	0.388947	0.061054	16	H	−2.670382	−2.517254	−1.209305
5	C	−0.024866	1.108988	0.075803	17	H	−4.400219	0.823436	0.855037
6	N	−0.025616	2.382997	0.132551	18	H	−4.687871	−1.396585	−0.227611
7	H	2.82044	−2.321394	1.399125	19	H	4.40036	0.683821	−1.225468
8	H	4.713015	−1.391685	0.095433	20	C	2.344063	0.915061	−0.658825
9	H	−0.993063	2.718332	0.133287	21	H	2.201071	1.825271	−1.23187
10	C	−1.297445	0.326284	0.003571	22	C	1.449847	−0.785271	0.794413
11	C	−1.360776	−0.91621	−0.619428	23	H	0.623917	−1.197537	1.365112
12	C	−2.590411	−1.552813	−0.718314	24	N	−2.377713	0.930659	0.510229

<u>Z_2 isomer</u>
Solvent: DMSO
Electronic Energy: (*EE*) = −572.480463 Hartree
Thermal Correction to Free Energy = 0.154323 Hartree
Gibbs free energy: (*G*) = −572.32614 Hartree
Chemical shift of imino proton: δ = 10.0671 ppm

1	C	−2.548282	−1.526529	−0.75877	13	C	3.620189	−1.007884	0.208765
2	C	−3.688808	−0.988407	−0.172898	14	H	2.495877	−2.18887	1.616246

3	C	−3.618119	0.236379	0.484508	15	H	4.418176	0.367393	−1.244897
4	C	−1.263561	0.386191	−0.033251	16	H	4.525347	−1.596547	0.310092
5	C	0.005611	1.148588	0.006556	17	H	−4.504278	0.655648	0.950916
6	N	−0.029523	2.424523	0.024243	18	C	−2.414089	0.92211	0.548619
7	H	−2.599418	−2.479682	−1.275694	19	H	−2.351709	1.876258	1.06107
8	H	−4.631475	−1.524532	−0.224612	20	C	−1.339045	−0.848573	−0.680575
9	H	0.914494	2.810928	0.078089	21	H	−0.452569	−1.275051	−1.137609
10	C	1.288777	0.385088	0.035391	22	N	1.336052	−0.675068	0.847873
11	C	2.484181	−1.341387	0.935723	23	C	2.374011	0.796718	−0.733118
12	C	3.558443	0.080841	−0.647774	24	H	2.28401	1.649459	−1.397472

***E* isomer (2ac)**

K = exp(−Δ*G*/RT) = E_2/E_1 = 1.303143529 (R: 1.9872×10^{-3} kcal·mol^{-1}·K^{-1}, T: 298.15 K)

In $CHCl_3$	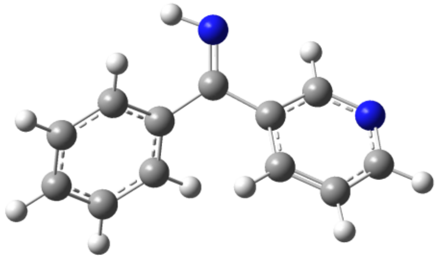 ***E*₁**	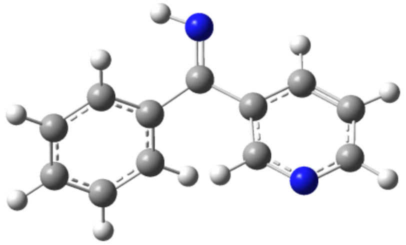 ***E*₂**
Ratio	0.43418918	0.56581082
$G_{average}$	−572.3289865 Hartree	
$\delta_{average}$	9.9384 ppm	

<u>E_1 isomer</u>
Solvent: $CHCl_3$
Electronic Energy: (*EE*) = −572.483362 Hartree
Thermal Correction to Free Energy = 0.154484 Hartree
Gibbs free energy: (*G*) = −572.328878 Hartree
Chemical shift of imino proton: δ = 9.9557 ppm

1	C	2.61928	−1.473177	0.797123	13	C	−2.354995	0.858149	0.68432
2	C	3.697721	−0.898177	0.140629	14	C	−2.624306	−1.431262	−0.867088
3	C	1.286182	0.365549	0.043155	15	H	−0.558737	−1.148056	−1.378774
4	C	1.390682	−0.834603	0.740332	16	C	−3.569569	0.188303	0.641606
5	C	0.021405	1.135147	−0.028625	17	H	−2.245916	1.743642	1.303653
6	N	0.09417	2.409092	−0.078866	18	C	−3.708138	−0.954972	−0.138067
7	H	2.742849	−2.402815	1.34189	19	H	−2.728921	−2.320586	−1.480563
8	H	4.674831	−1.373533	0.163347	20	H	−4.409212	0.556072	1.222965
9	H	0.525715	−1.255132	1.242876	21	H	−4.659126	−1.477149	−0.173457
10	H	−0.837782	2.818267	−0.162863	22	C	2.434304	0.85267	−0.58434
11	C	−1.261563	0.385057	−0.043297	23	H	2.378267	1.778145	−1.149893
12	C	−1.403696	−0.772608	−0.81056	24	N	3.613572	0.242434	−0.546891

<u>E_2 isomer</u>
Solvent: $CHCl_3$
Electronic Energy: (*EE*) = −572.483698 Hartree
Thermal Correction to Free Energy = 0.15457 Hartree
Gibbs free energy: (*G*) = −572.329128 Hartree
Chemical shift of imino proton: δ = 9.9159 ppm

1	C	−3.610793	−1.037943	−0.245216	13	C	3.551464	0.187991	−0.667815

2	C	−3.636417	0.169631	0.44222	14	H	2.215755	1.735562	−1.324948
3	C	−1.297579	0.382952	−0.015191	15	C	3.703122	−0.946676	0.121902
4	C	−0.02664	1.143361	0.05338	16	H	2.746035	−2.298887	1.49365
5	N	−0.091716	2.417403	0.115183	17	H	4.381128	0.549244	−1.2673
6	H	−4.51507	−1.631954	−0.349546	18	H	4.654525	−1.468686	0.146873
7	H	0.843436	2.820526	0.190084	19	H	−4.561013	0.528636	0.881193
8	C	1.254885	0.390951	0.052095	20	C	−2.462693	0.892496	0.554474
9	C	1.409826	−0.758012	0.829638	21	H	−2.430432	1.841092	1.079651
10	C	2.336115	0.857076	−0.697809	22	N	−2.510409	−1.537988	−0.805137
11	C	2.631539	−1.415485	0.873479	23	C	−1.384516	−0.838964	−0.681691
12	H	0.573756	−1.128681	1.413951	24	H	−0.503132	−1.267046	−1.150727

***Z* isomer (2ac)**

K = exp(−Δ*G*/RT) = Z_2/Z_1 = 0.733988152 (R: 1.9872×10^{-3} kcal·mol^{-1}·K^{-1}, T: 298.15 K)

In $CHCl_3$	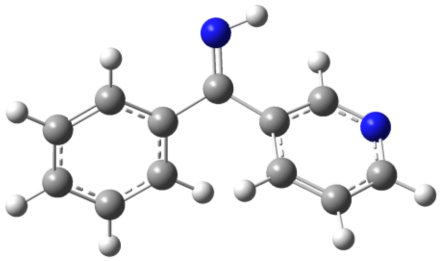 ***Z*1**	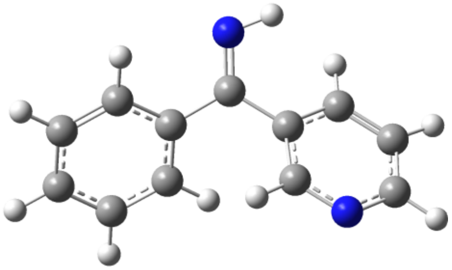 ***Z*2**
Ratio	0.576705209	0.423294791
$G_{average}$	−572.3287766 Hartree	
$\delta_{average}$	9.8544 ppm	

Z_1 isomer
Solvent: $CHCl_3$
Electronic Energy: (*EE*) = −572.483235 Hartree
Thermal Correction to Free Energy = 0.15429 Hartree
Gibbs free energy: (*G*) = −572.328945 Hartree
Chemical shift of imino proton: δ = 9.8532 ppm

1	C	2.552208	−1.56223	0.7067	13	H	−0.660058	−1.062265	−1.540772
2	C	3.705031	−0.996942	0.175688	14	C	−3.69747	−0.861056	−0.0731
3	C	3.647583	0.255751	−0.429134	15	H	−2.89875	−2.177678	−1.573125
4	C	1.279508	0.377041	0.028297	16	H	−4.679314	−1.326764	−0.060639
5	C	0.006486	1.13465	−0.030697	17	H	4.545192	0.697695	−0.850344
6	N	0.043885	2.410909	−0.06443	18	C	2.444559	0.940293	−0.497941
7	H	2.59279	−2.535583	1.185319	19	H	2.389705	1.917843	−0.964429
8	H	4.648158	−1.531853	0.230319	20	C	1.343525	−0.882755	0.626423
9	H	−0.898101	2.797421	−0.138777	21	H	0.447626	−1.327983	1.046282
10	C	−1.269037	0.37311	−0.054598	22	C	−2.33421	0.763039	0.756814
11	C	−1.464414	−0.716652	−0.89933	23	H	−2.208642	1.589648	1.45246
12	C	−2.702562	−1.336731	−0.916914	24	N	−3.521864	0.165434	0.75878

Z_2 isomer
Solvent: $CHCl_3$
Electronic Energy: (*EE*) = −572.483035 Hartree
Thermal Correction to Free Energy = 0.154382 Hartree
Gibbs free energy: (*G*) = −572.328653 Hartree
Chemical shift of imino proton: δ = 9.8553 ppm

1	C	−2.54813	−1.553552	−0.719069	13	H	4.409151	0.370504	−1.265142
2	C	−3.696795	−0.996007	−0.171273	14	H	4.539967	−1.559425	0.297524
3	C	−3.636506	0.251563	0.443789	15	H	−4.530653	0.686831	0.879074
4	C	−1.273798	0.383429	−0.037527	16	C	−2.434824	0.938972	0.505628
5	C	−0.003388	1.145969	0.011604	17	H	−2.377635	1.912152	0.9808
6	N	−0.047864	2.422174	0.04551	18	C	−1.340572	−0.871396	−0.645673
7	H	−2.590636	−2.523525	−1.204255	19	H	−0.447707	−1.311521	−1.077231
8	H	−4.638774	−1.533522	−0.219821	20	C	2.359603	0.796947	−0.750564
9	H	0.892664	2.81398	0.110998	21	H	2.261369	1.641585	−1.426393
10	C	1.277592	0.392111	0.026514	22	N	2.599452	−1.384788	0.962471
11	C	3.549379	0.093585	−0.664777	23	C	1.456676	−0.712449	0.859969
12	C	3.621429	−0.985909	0.205466	24	H	0.637151	−1.05834	1.484071

***E* isomer (2ad)**

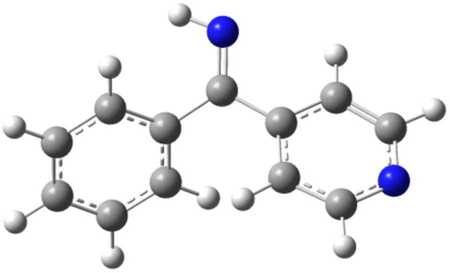

Solvent: $CDCl_3$
Electronic Energy: (*EE*) = −572.48304 Hartree
Thermal Correction to Free Energy = 0.154496 Hartree
Gibbs free energy: (*G*) = −572.328544 Hartree
Chemical shift of imino proton: δ = 10.2199 ppm

1	C	−2.613591	−1.471833	−0.774696	13	H	−4.528679	0.512145	0.936184
2	C	−3.618743	0.148974	0.465647	14	C	−2.443027	0.873995	0.571336
3	C	−1.28986	0.37763	−0.029927	15	H	−2.414834	1.810375	1.116571
4	C	−0.016424	1.143573	0.038134	16	C	−1.384561	−0.830105	−0.714745
5	N	−0.090561	2.416123	0.084378	17	H	−0.52135	−1.264124	−1.206745
6	H	−2.712583	−2.409966	−1.314565	18	C	2.353275	0.857774	−0.690897
7	H	0.841664	2.828184	0.150834	19	H	2.241474	1.738462	−1.316554
8	C	1.264044	0.391195	0.047068	20	C	1.40893	−0.760522	0.822603
9	C	2.629042	−1.420115	0.876647	21	H	0.566765	−1.131191	1.398004
10	C	3.566989	0.186357	−0.650846	22	C	3.708746	−0.950914	0.136984
11	H	2.736221	−2.304995	1.495995	23	H	4.659123	−1.474304	0.170225
12	H	4.403322	0.548139	−1.240615	24	N	−3.718015	−1.005948	−0.194832

***Z* isomer (2ad)**

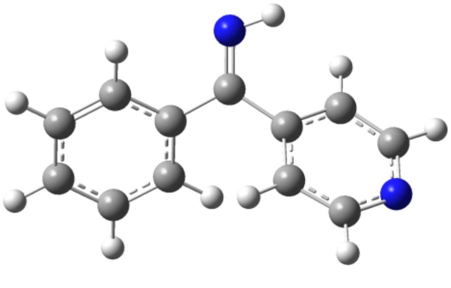

Solvent: $CHCl_3$
Electronic Energy: (*EE*) = −572.482897 Hartree
Thermal Correction to Free Energy = 0.154186 Hartree
Gibbs free energy: (*G*) = −572.328711 Hartree
Chemical shift of imino proton: δ = 9.911 ppm

1	C	−2.548219	−1.563832	−0.706779	13	H	2.892752	−2.146342	1.589313
2	C	−3.704204	−0.999614	−0.181626	14	H	4.360877	0.346696	−1.3318

3	C	−3.652353	0.255277	0.419282	15	H	−4.552542	0.696019	0.836147
4	C	−1.283366	0.380634	−0.029974	16	C	−2.451598	0.943271	0.490065
5	C	−0.013688	1.141591	0.028288	17	H	−2.400938	1.922393	0.953651
6	N	−0.048099	2.416222	0.079361	18	C	−1.341601	−0.881135	−0.624518
7	H	−2.584459	−2.539208	−1.181519	19	H	−0.442941	−1.326576	−1.038101
8	H	−4.645651	−1.537334	−0.237408	20	C	2.329419	0.771109	−0.773359
9	H	0.895722	2.800699	0.142546	21	H	2.221904	1.593723	−1.473038
10	C	1.269596	0.384842	0.041322	22	C	1.468504	−0.693397	0.89869
11	C	2.708779	−1.313705	0.91543	23	H	0.675227	−1.038749	1.552543
12	C	3.52441	0.071541	−0.694672	24	N	3.726096	−0.952052	0.134178

***E* isomer (2af)**

K = exp(−Δ*G*/RT) = E_2/E_1 = 0.571658103 (R: 1.9872×10^{-3} kcal·mol^{-1}·K^{-1}, T: 298.15 K)

In DMSO	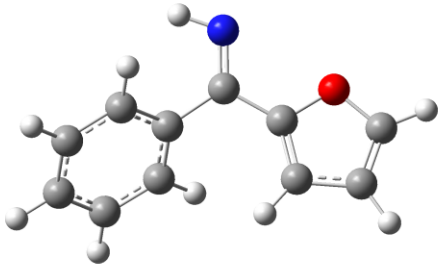 ***E*1**	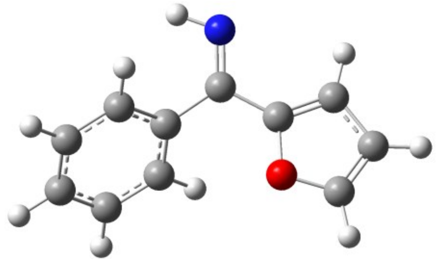 ***E*2**
Ratio	0.636270699	0.363729301
$G_{average}$	−554.1039 Hartree	
$\delta_{average}$	9.3046 ppm	

E_1 isomer
Solvent: DMSO
Electronic Energy: (*EE*) = −554.241655 Hartree
Thermal Correction to Free Energy = 0.137419 Hartree
Gibbs free energy: (*G*) = −554.104236 Hartree
Chemical shift of imino proton: δ = 9.2015 ppm

1	C	−2.549538	−1.38176	−0.70922	12	C	3.641998	−0.296188	0.028344
2	C	−3.536415	−0.769444	0.055442	13	O	2.740383	0.650315	−0.295427
3	C	−3.250129	0.404216	0.745118	14	H	−2.771245	−2.292921	−1.256139
4	C	−1.983518	0.966367	0.665674	15	H	−4.528316	−1.206648	0.114745
5	C	−0.986933	0.352067	−0.094418	16	H	−4.01504	0.881585	1.34962
6	C	−1.276899	−0.829874	−0.777407	17	H	−1.75776	1.879752	1.207993
7	C	0.346487	0.999205	−0.206431	18	H	−0.509756	−1.306287	−1.378816
8	N	0.506221	2.245783	−0.455809	19	H	−0.402536	2.691328	−0.589625
9	C	1.509104	0.150525	0.001441	20	H	0.834107	−1.744959	0.850692
10	C	1.639193	−1.109246	0.514804	21	H	3.511723	−2.301734	0.874887
11	C	3.02926	−1.397964	0.533294	22	H	4.676556	−0.05055	−0.159126

E_2 isomer
Solvent: DMSO
Electronic Energy: (*EE*) = −554.24028 Hartree
Thermal Correction to Free Energy = 0.136572 Hartree
Gibbs free energy: (*G*) = −554.103708 Hartree
Chemical shift of imino proton: δ = 9.3636 ppm

1	C	2.409991	−1.429191	0.719288	12	C	−3.635051	−0.530355	−0.030825
2	C	3.450387	−0.85446	−0.003154	13	C	−2.866878	0.597801	0.358669

3	C	3.244467	0.340943	−0.683691	14	H	2.56883	−2.358816	1.256795
4	C	2.004753	0.963542	−0.635662	15	H	4.420789	−1.339792	−0.038195
5	C	0.955709	0.38876	0.083415	16	H	4.050818	0.788051	−1.256519
6	C	1.164306	−0.817647	0.753612	17	H	1.841667	1.893355	−1.172508
7	C	−0.347197	1.095874	0.170902	18	H	0.355734	−1.268162	1.319231
8	N	−0.459049	2.355815	0.373817	19	H	0.462813	2.773565	0.505592
9	C	−1.572505	0.317242	0.031256	20	H	−2.867473	−2.398175	−0.999694
10	O	−1.502557	−0.9198	−0.538191	21	H	−4.700192	−0.672839	0.077473
11	C	−2.754996	−1.414578	−0.569623	22	H	−3.217098	1.50292	0.831501

***Z* isomer (2af)**

K = exp(−Δ*G*/RT) = Z_2/Z_1 = 0.145189921 (R: 1.9872×10^{-3} kcal·mol^{-1}·K^{-1}, T: 298.15 K)

In DMSO	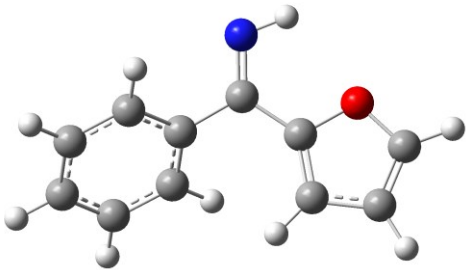<br>***Z*₁**	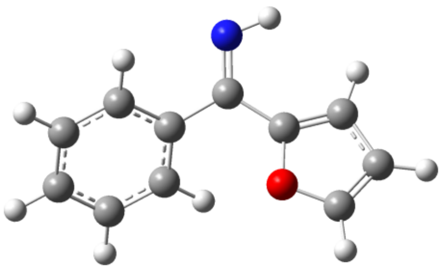<br>***Z*₂**
Ratio	0.819889281	0.180110719
$G_{average}$	−554.103433 Hartree	
$\delta_{average}$	9.7347 ppm	

Z_1 isomer
Solvent: DMSO
Electronic Energy: (*EE*) = −554.242178 Hartree
Thermal Correction to Free Energy = 0.137154 Hartree
Gibbs free energy: (*G*) = −554.105024 Hartree
Chemical shift of imino proton: δ = 9.7757 ppm

1	C	−2.560947	−1.406369	−0.650324	12	C	3.637208	−0.289066	0.018879
2	C	−3.562378	−0.747483	0.05385	13	O	2.717767	0.643166	−0.302508
3	C	−3.287307	0.465205	0.678559	14	H	−2.773725	−2.347071	−1.148574
4	C	−2.017011	1.017257	0.596024	15	H	−4.557181	−1.177806	0.115927
5	C	−1.004887	0.355266	−0.100192	16	H	−4.065335	0.980795	1.232971
6	C	−1.28475	−0.862428	−0.721612	17	H	−1.795071	1.962718	1.080092
7	C	0.335034	0.984263	−0.198176	18	H	−0.508808	−1.374224	−1.280973
8	N	0.413486	2.243209	−0.425059	19	H	1.387655	2.549069	−0.437281
9	C	1.493584	0.123811	−0.001154	20	H	0.853643	−1.779753	0.855051
10	C	1.64622	−1.131986	0.512809	21	H	3.538659	−2.294597	0.866025
11	C	3.041388	−1.39841	0.52565	22	H	4.666701	−0.027286	−0.173636

Z_2 isomer
Solvent: DMSO
Electronic Energy: (*EE*) = −554.24011 Hartree
Thermal Correction to Free Energy = 0.136908 Hartree
Gibbs free energy: (*G*) = −554.103202 Hartree
Chemical shift of imino proton: δ = 9.7288 ppm

1	C	−2.394013	−1.463889	−0.669002	12	C	3.616491	−0.554739	−0.027849

2	C	−3.462634	−0.853742	−0.021122	13	C	2.838973	0.56182	−0.433168
3	C	−3.285815	0.380528	0.596705	14	H	−2.529769	−2.423167	−1.158616
4	C	−2.04682	1.00403	0.560918	15	H	−4.432641	−1.340696	0.0043
5	C	−0.968742	0.393686	−0.081533	16	H	−4.11589	0.856927	1.109066
6	C	−1.149409	−0.848754	−0.691153	17	H	−1.900658	1.966807	1.039432
7	C	0.334198	1.098866	−0.144768	18	H	−0.32059	−1.326995	−1.20199
8	N	0.342469	2.369129	−0.310094	19	H	1.29778	2.729577	−0.292572
9	C	1.56	0.31211	−0.028405	20	H	2.884174	−2.363347	1.070797
10	O	1.508754	−0.896477	0.601971	21	H	4.67334	−0.713314	−0.184092
11	C	2.75769	−1.401998	0.596274	22	H	3.177425	1.434619	−0.972813

***E* isomer (2ag)**

K = exp(−Δ*G*/RT) = E_2/E_1 = 0.093650327 (R: 1.9872×10^{-3} kcal·mol^{-1}·K^{-1}, T: 298.15 K)

In DMSO	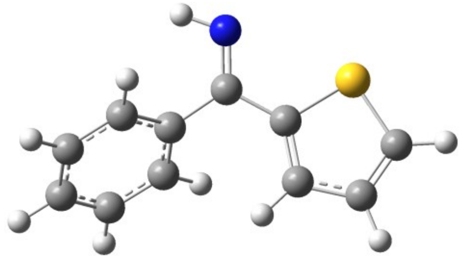 ***E*1**	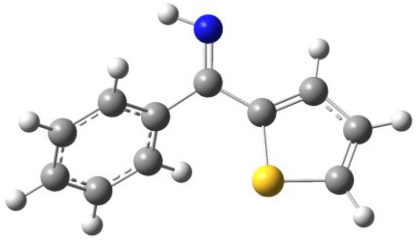 ***E*2**
Ratio	0.91436904	0.08563096
$G_{average}$	−877.0354555 Hartree	
$\delta_{average}$	9.0923 ppm	

<u>E_1 isomer</u>
Solvent: DMSO
Electronic Energy: (*EE*) = −877.170495 Hartree
Thermal Correction to Free Energy = 0.132995 Hartree
Gibbs free energy: (*G*) = −877.0375 Hartree
Chemical shift of imino proton: δ = 8.9062 ppm

1	C	2.854295	−1.301361	0.822007	12	C	−3.542258	−0.782113	−0.166403
2	C	3.835655	−0.732901	0.017324	13	S	−2.827944	0.701135	0.306907
3	C	3.533691	0.376948	−0.764404	14	H	3.087953	−2.162795	1.439016
4	C	2.256734	0.92065	−0.736189	15	H	4.834851	−1.155593	−0.001968
5	C	1.266367	0.350644	0.064324	16	H	4.293755	0.818235	−1.400714
6	C	1.571955	−0.7694	0.838315	17	H	2.017891	1.782706	−1.35126
7	C	−0.080662	0.97728	0.119574	18	H	0.806853	−1.21231	1.467031
8	N	−0.261159	2.2321	0.304798	19	H	0.633826	2.703121	0.436928
9	C	−1.24168	0.111643	−0.051064	20	H	−0.413201	−1.731657	−0.815211
10	C	−1.294936	−1.177727	−0.519098	21	H	−2.859538	−2.684856	−0.932758
11	C	−2.611317	−1.688981	−0.587414	22	H	−4.614233	−0.907804	−0.106991

<u>E_2 isomer</u>
Solvent: DMSO
Electronic Energy: (*EE*) = −877.167996 Hartree
Thermal Correction to Free Energy = 0.132732 Hartree
Gibbs free energy: (*G*) = −877.035264 Hartree
Chemical shift of imino proton: δ = 9.1097 ppm

1	C	2.563062	−1.380864	0.868685	12	C	−3.594464	−0.076725	0.38563
2	C	3.631411	−0.9021	0.119487	13	C	−2.547935	0.841822	0.624276
3	C	3.48355	0.255483	−0.639267	14	H	2.676951	−2.277396	1.46913

4	C	2.273227	0.932414	−0.644684	15	H	4.579156	−1.430772	0.125198
5	C	1.192533	0.451738	0.096367	16	H	4.314146	0.628352	−1.229748
6	C	1.345541	−0.712763	0.850463	17	H	2.146508	1.836516	−1.229877
7	C	−0.08549	1.205609	0.094996	18	H	0.515703	−1.085194	1.44142
8	N	−0.038997	2.48482	0.09602	19	H	−0.979075	2.876873	0.03533
9	C	−1.348064	0.450714	0.08516	20	H	−3.770949	−2.004248	−0.671646
10	S	−1.519237	−1.074078	−0.721792	21	H	−4.6089	0.052449	0.741981
11	C	−3.184474	−1.158619	−0.341406	22	H	−2.666151	1.754668	1.196505

Z isomer (2ag)

K = exp(−Δ*G*/RT) = Z_2/Z_1 = 0.772267151 (R: 1.9872×10^{-3} kcal·mol^{-1}·K^{-1}, T: 298.15 K)

In DMSO	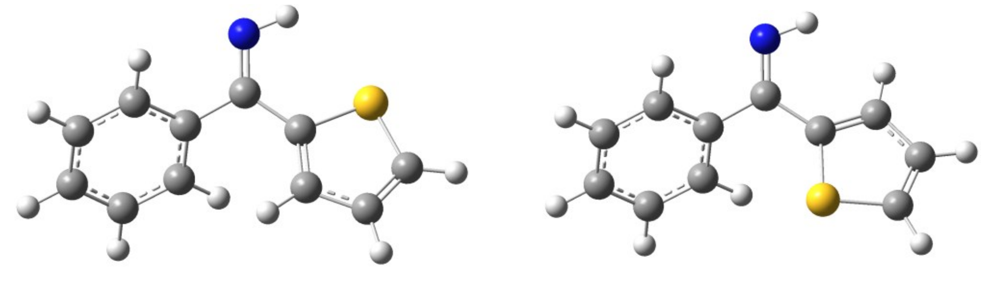 ***Z*1**	***Z*2**
Ratio	0.564249018	0.435750982
$G_{average}$	−877.0353193 Hartree	
$\delta_{average}$	9.4694 ppm	

Z_1 isomer
Solvent: DMSO
Electronic Energy: (*EE*) = −877.168134 Hartree
Thermal Correction to Free Energy = 0.132677 Hartree
Gibbs free energy: (*G*) = −877.035457 Hartree
Chemical shift of imino proton: δ = 9.3032 ppm

1	C	−2.758898	−1.394398	−0.778254	12	C	3.521311	−0.796382	0.098101
2	C	−3.815705	−0.78853	−0.108591	13	S	2.784057	0.631524	−0.491646
3	C	−3.604685	0.395254	0.592214	14	H	−2.920352	−2.313051	−1.332672
4	C	−2.343578	0.972306	0.617901	15	H	−4.802945	−1.23863	−0.13079
5	C	−1.275973	0.364601	−0.044211	16	H	−4.425837	0.867456	1.121705
6	C	−1.492285	−0.826178	−0.738753	17	H	−2.168361	1.896271	1.157815
7	C	0.057849	1.017898	−0.026986	18	H	−0.669217	−1.299408	−1.263134
8	N	0.106686	2.295536	−0.107734	19	H	1.068089	2.631177	−0.035272
9	C	1.230216	0.145131	0.098028	20	H	0.455226	−1.56745	1.159107
10	C	1.31208	−1.079601	0.711908	21	H	2.886661	−2.565676	1.16194
11	C	2.62091	−1.613917	0.719007	22	H	4.577575	−0.960943	−0.060412

Z_2 isomer
Solvent: DMSO
Electronic Energy: (*EE*) = −877.167815 Hartree
Thermal Correction to Free Energy = 0.132602 Hartree
Gibbs free energy: (*G*) = −877.035213 Hartree
Chemical shift of imino proton: δ = 9.5977 ppm

1	C	2.617354	−1.277857	0.93849	12	C	−3.636098	−0.043872	0.202125
2	C	3.615595	−0.874726	0.058667	13	C	−2.617961	0.904421	0.436968

3	C	3.391973	0.195594	−0.80104	14	H	2.791291	−2.1067	1.616736
4	C	2.176336	0.864807	−0.776236	15	H	4.567385	−1.395533	0.041525
5	C	1.167375	0.45986	0.098598	16	H	4.165275	0.507095	−1.495461
6	C	1.393918	−0.621407	0.951687	17	H	1.997363	1.695482	−1.451833
7	C	−0.114183	1.21283	0.141981	18	H	0.616926	−0.934953	1.640788
8	N	−0.169682	2.48938	0.224514	19	H	0.772763	2.872882	0.303412
9	C	−1.369572	0.458982	0.080497	20	H	−3.706905	−2.091258	−0.618479
10	S	−1.460642	−1.151477	−0.553043	21	H	−4.683035	0.118865	0.426738
11	C	−3.155515	−1.204636	−0.339358	22	H	−2.778352	1.888102	0.859372

***E* isomer (2ah)**

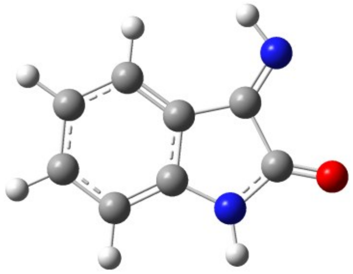

Solvent: DMSO
Electronic Energy: (*EE*) = −492.953359 Hartree
Thermal Correction to Free Energy = 0.095273 Hartree
Gibbs free energy: (*G*) = −492.858086 Hartree
Chemical shift of imino proton: δ = 11.298 ppm

1	C	−0.389476	−0.821933	0.000069	10	H	−3.739268	−1.024	−0.000092
2	C	−0.245847	0.571116	0.000081	11	H	−1.741826	−2.499915	0.000053
3	C	−1.358773	1.394885	−0.000045	12	C	1.877777	−0.515082	0.000009
4	C	−2.62112	0.80794	−0.000118	13	O	3.067254	−0.743694	−0.000294
5	C	−2.748347	−0.580567	−0.000063	14	N	0.864316	−1.431097	0.00021
6	C	−1.63424	−1.420608	0.000031	15	H	1.014387	−2.431024	0.000057
7	C	1.186523	0.856657	0.000099	16	N	1.857012	1.931268	0.00009
8	H	−1.244539	2.474883	−0.000087	17	H	1.234264	2.74354	0.000141
9	H	−3.509328	1.430423	−0.000199					

***Z* isomer (2ah)**

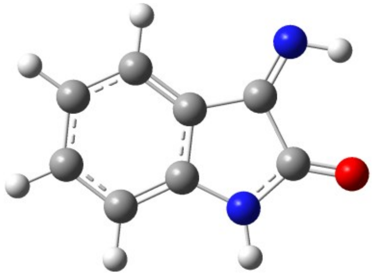

Solvent: DMSO
Electronic Energy: (*EE*) = −492.955204 Hartree
Thermal Correction to Free Energy = 0.095271 Hartree
Gibbs free energy: (*G*) = −492.859933 Hartree
Chemical shift of imino proton: δ = 10.8357 ppm

1	C	−0.41123	−0.810466	0.000021	10	H	−3.762102	−0.981132	−0.000014
2	C	−0.254027	0.581921	0.000052	11	H	−1.77838	−2.475756	−0.00009
3	C	−1.359259	1.41607	0.000035	12	C	1.853503	−0.528906	−0.000008
4	C	−2.626789	0.840154	0.000031	13	O	3.048032	−0.73857	0.000108

5	C	−2.767154	−0.54687	0.000004	14	N	0.838576	−1.436079	−0.00015
6	C	−1.660665	−1.397568	−0.000029	15	H	0.976068	−2.438197	0.00034
7	C	1.177241	0.848873	−0.000043	16	N	1.791508	1.955709	−0.000062
8	H	−1.232897	2.494021	0.000047	17	H	2.802109	1.782271	−0.00009
9	H	−3.509356	1.4707	0.000037					

***EE* isomer (2aj)**

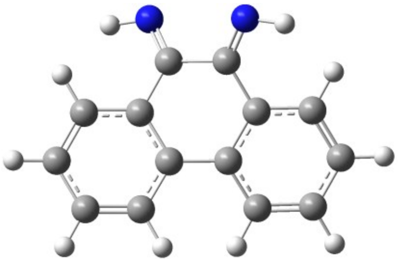

Solvent: $CDCl_3$
Electronic Energy: (EE) = −648.661325 Hartree
Thermal Correction to Free Energy = 0.16891 Hartree
Gibbs free energy: (G) = −648.492415 Hartree
Chemical shift of imino proton: δ = 10.7695 ppm

1	C	−0.111044	3.544326	−0.725681	14	C	−0.068164	−2.833732	0.44902
2	C	−0.250258	2.855764	−1.926375	15	N	0.887309	1.100291	2.740667
3	C	−0.20996	1.470754	−1.947066	16	N	−0.887309	−1.100291	2.740667
4	C	−0.029487	0.736642	−0.772162	17	H	−0.159403	4.628065	−0.705098
5	C	0.111044	1.440684	0.437467	18	H	−0.403776	3.40143	−2.852008
6	C	0.068164	2.833732	0.44902	19	H	−0.338226	0.958219	−2.893234
7	C	0.029487	−0.736642	−0.772162	20	H	0.148666	3.364672	1.393012
8	C	−0.111044	−1.440684	0.437467	21	H	0.338226	−0.958219	−2.893234
9	C	−0.287775	−0.690199	1.696895	22	H	0.403776	−3.40143	−2.852008
10	C	0.287775	0.690199	1.696895	23	H	0.159403	−4.628065	−0.705098
11	C	0.20996	−1.470754	−1.947066	24	H	−0.148666	−3.364672	1.393012
12	C	0.250258	−2.855764	−1.926375	25	H	1.246915	2.046731	2.600065
13	C	0.111044	−3.544326	−0.725681	26	H	−1.246915	−2.046731	2.600065

***EZ* isomer (2aj)**

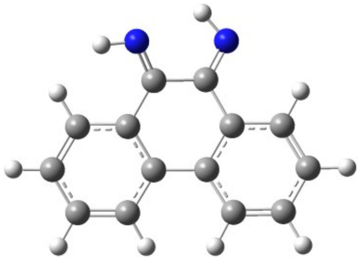

Solvent: $CDCl_3$
Electronic Energy: (*EE*) = −648.666617 Hartree
Thermal Correction to Free Energy = 0.167606 Hartree
Gibbs free energy: (*G*) = −648.499011 Hartree
Chemical shift of imino proton: δ = 11.4845, 10.8872 ppm

1	C	−3.534354	−0.787457	−0.139095	14	C	2.845016	0.44715	−0.08759
2	C	−2.826636	−1.983813	−0.193935	15	N	−1.216137	2.797538	0.622523
3	C	−1.443211	−1.980084	−0.13119	16	N	1.299253	2.772829	−0.581454
4	C	−0.72422	−0.787599	−0.002715	17	H	−4.616947	−0.783892	−0.209822
5	C	−1.448189	0.417269	0.066853	18	H	−3.355635	−2.925404	−0.301716
6	C	−2.841204	0.402492	−0.011775	19	H	−0.919608	−2.925575	−0.200788
7	C	0.74968	−0.774803	0.048326	20	H	−3.389769	1.339117	0.000793
8	C	1.450035	0.436554	−0.084911	21	H	0.986812	−2.901211	0.326352
9	C	0.719164	1.709381	−0.191397	22	H	3.424619	−2.858782	0.3272
10	C	−0.730273	1.697386	0.204662	23	H	4.645517	−0.709636	0.057104
11	C	1.491105	−1.951169	0.198807	24	H	3.35347	1.399375	−0.18934
12	C	2.875794	−1.930703	0.201589	25	H	−2.200058	2.706859	0.877535
13	C	3.560527	−0.728039	0.051742	26	H	0.630379	3.547193	−0.55102

***ZZ* isomer (2aj)**

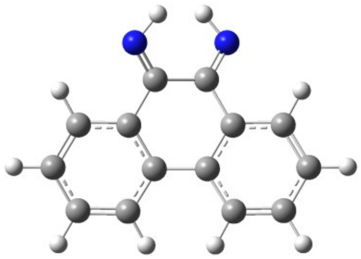

Solvent: $CDCl_3$
Electronic Energy: (*EE*) = −648.665785 Hartree
Thermal Correction to Free Energy = 0.168092 Hartree
Gibbs free energy: (*G*) = −648.497693 Hartree
Chemical shift of imino proton: δ = 10.5156 ppm

1	C	−3.546645	−0.745578	−0.103962	14	C	2.838971	0.431951	−0.05888
2	C	−2.854291	−1.945771	−0.237876	15	N	−1.279047	2.712696	0.745587
3	C	−1.469472	−1.963296	−0.201021	16	N	1.279049	2.712706	−0.745548
4	C	−0.736413	−0.784972	−0.03305	17	H	−4.6311	−0.73072	−0.138879
5	C	−1.444866	0.423034	0.088732	18	H	−3.397621	−2.874752	−0.379743
6	C	−2.838971	0.431951	0.058886	19	H	−0.956746	−2.910311	−0.319936
7	C	0.736412	−0.784973	0.033038	20	H	−3.353261	1.381958	0.153804
8	C	1.444865	0.423035	−0.088726	21	H	0.956745	−2.910315	0.319895
9	C	0.712751	1.691048	−0.237965	22	H	3.397621	−2.874758	0.379704
10	C	−0.71275	1.691045	0.237987	23	H	4.6311	−0.730723	0.13887
11	C	1.469471	−1.963299	0.200994	24	H	3.353261	1.381959	−0.153785
12	C	2.85429	−1.945775	0.23785	25	H	−0.636213	3.506457	0.791907
13	C	3.546645	−0.74558	0.103952	26	H	0.636216	3.506468	−0.791857

VI References

1. Gaussian 16, Revision C.01, M. J. Frisch, G. W. Trucks, H. B. Schlegel, G. E. Scuseria, M. A. Robb, J. R. Cheeseman, G. Scalmani, V. Barone, G. A. Petersson, H. Nakatsuji, X. Li, M. Caricato, A. V. Marenich, J. Bloino, B. G. Janesko, R. Gomperts, B. Mennucci, H. P. Hratchian, J. V. Ortiz, A. F. Izmaylov, J. L. Sonnenberg, D. Williams-Young, F. Ding, F. Lipparini, F. Egidi, J. Goings, B. Peng, A. Petrone, T. Henderson, D. Ranasinghe, V. G. Zakrzewski, J. Gao, N. Rega, G. Zheng, W. Liang, M. Hada, M. Ehara, K. Toyota, R. Fukuda, J. Hasegawa, M. Ishida, T. Nakajima, Y. Honda, O. Kitao, H. Nakai, T. Vreven, K. Throssell, J. A. Montgomery, Jr., J. E. Peralta, F. Ogliaro, M. J. Bearpark, J. J. Heyd, E. N. Brothers, K. N. Kudin, V. N. Staroverov, T. A. Keith, R. Kobayashi, J. Normand, K. Raghavachari, A. P. Rendell, J. C. Burant, S. S. Iyengar, J. Tomasi, M. Cossi, J. M. Millam, M. Klene, C. Adamo, R. Cammi, J. W. Ochterski, R. L. Martin, K. Morokuma, O. Farkas, J. B. Foresman, D. J. Fox, Gaussian, Inc., Wallingford CT, **2016**.
2. Shibata, S., Masui, Y. & Onaka, M. Coordination behaviors of diphenylketene adsorbed in the nanocages of zeolite NaY and AgY. *Bull. Chem. Soc. Jpn.* **93***,* 663–670 (2020). 10.1246/bcsj.20200039
3. Motokura, K., Fujita, N., Mori, K., Mizugaki, T., Ebitani, K. & Kaneda, K. Brønsted acid mediated heterogeneous addition reaction of 1,3-dicarbonyl compounds to alkenes and alcohols. *Angew. Chem., Int. Ed.* **45**, 2605–2609 (2006). 10.1002/anie.200504609
4. Tandiary, M. A., Asano, M., Hattori, T., Takehira, S., Masui, Y. & Onaka, M. Unprecedented alkylation of silicon enolates with alcohols via carbenium ion formations catalyzed by tin hydroxide-embedded montmorillonite. *Tetrahedron Lett.* **58**, 1925–1928 (2017). 10.1016/j.tetlet.2017.03.073
5. Motokura, K., Nakagiri, N., Mizugaki, T., Ebitani, K. & Kaneda, K. Nucleophilic substitution reactions of alcohols with use of montmorillonite catalysts as solid Brønsted acids. *J. Org. Chem.* **72**, 6006–6015 (2007). 10.1021/jo070416w
6. Kawabata, T., Mizugaki, T., Ebitani, K. & Kaneda, K. Highly efficient esterification of carboxylic acids with alcohols by montmorillonite-enwrapped titanium as a heterogeneous acid catalyst. *Tetrahedron Lett.* **44**, 9205–9208 (2003). 10.1016/j.tetlet.2003.10.024
7. Ebitani, K., Ide, M., Mitsudome, T., Mizugaki, T. & Kaneda, K. Creation of a chain-like cationic iron species in montmorillonite as a highly active heterogeneous catalyst for alkane oxygenations using hydrogen peroxide. *Chem. Commun.* **2002**, 690–691 (2002). 10.1039/b200255h
8. Kawabata, T., Kato, M., Mizugaki, T., Ebitani, K. & Kaneda, K. Monomeric metal aqua complexes in the interlayer space of montmorillonites as strong Lewis acid catalysts for heterogeneous carbon–carbon bond-forming reactions. *Chem. Eur. J.* **11**, 288–297 (2005). 10.1002/chem.200400672
9. Masui, Y. *et al.* Unique structural characteristics of tin hydroxide nanoparticles-embedded montmorillonite (Sn-Mont) demonstrating efficient acid catalysis for various organic reactions. *Microporous Mesoporous Mater.* **198**, 129–138 (2014). doi.org/10.1016/j.micromeso.2014.07.024

10. Seki, T. & Onaka, M. Sulfated mesoporous alumina: A highly effective solid strong base catalyst for the Tishchenko reaction in supercritical carbon dioxide. *J. Phys. Chem. B* **110**, 1240–1248 (2006). doi.org/10.1021/jp055895m

11. Suzuki, E., Iwasaki, R., Goto, J., Matsuki, Y. & Nambara, T. Synthesis of N-acetylcysteine conjugates of catechol estrogens. *Steroids* **61**, 296–301 (1996). 10.1016/0039-128X(95)00232-F

12. Numazawa, M., Komatsu, S., Tominaga, T. & Yamashita, K. Structure–activity relationships of estrogen derivatives as aromatase inhibitors. Effects of heterocyclic substituents. *Chem. Pharm. Bull.* **56**, 1304–1309 (2008). 10.1248/cpb.56.1304

13. Shibata, S. *et al.* Synthesis of N-unprotected diaryl ketimines and alkyl ketimines from ketones and ammonia using porous solid acids with analysis of their adsorption behavior. *Bull. Chem. Soc. Jpn.* **96**, 555–567 (2023). 10.1246/bcsj.20230055

14. Matori, K., Wah, L., Hashim, M., Ismail, I. & Zaid, M. Phase transformations of α-Alumina made from waste aluminum via a precipitation technique. *Int. J. Mol. Sci.* **13**, 16812–16821 (2012). 10.3390/ijms131216812

15. XPS International, LLC. The International XPS Database of Monochromatic Reference Spectra. Available at: https://xpsdatabase.net (accessed September 24, 2025).

16. Konnert, L., Lamaty, F., Martinez, J. & Colacino, E. Recent advances in the synthesis of hydantoins: The state of the art of a valuable scaffold. *Chem. Rev.* **117**, 13757–13809 (2017). 10.1021/acs.chemrev.7b00067

17. Austin, A. *et al.* A density functional with spherical atom dispersion terms. *J. Chem. Theory Comput.* **8**, 4989–5007 (2012). 10.1021/ct300778e

18. Digne, M., Sautet, P., Raybaud, P., Euzen, P. & Toulhoat, H. Hydroxyl groups on γ-alumina surfaces: A DFT study. *J. Catal.* **211**, 1–5 (2002). 10.1006/jcat.2002.3741

19. Digne, M., Sautet, P., Raybaud, P., Euzen, P. & Toulhoat, H. Use of DFT to achieve a rational understanding of acid-basic properties of γ-alumina surfaces. *J. Catal.* **226**, 54–68 (2004). 10.1016/j.jcat.2004.04.020

20. Batista, A. T. F. *et al.* Beyond γ-Al_2O_3 crystallite surfaces: The hidden features of edges revealed by solid-state ^1H NMR and DFT calculations. *J. Catal.* **378**, 140–143 (2019). 10.1016/j.jcat.2019.08.009

21. Lee, J. H., Gupta, S., Jeong, W., Rhee, Y. H. & Park, J. Characterization and utility of N-unsubstituted imines synthesized from alkyl azides by ruthenium catalysis. *Angew. Chem. Int. Ed.* **51**, 10851–10855 (2012). 10.1002/anie.201204483
